# In silico analysis of alternative splicing on drug-target gene interactions

**DOI:** 10.1038/s41598-019-56894-x

**Published:** 2020-01-10

**Authors:** Yanrong Ji, Rama K. Mishra, Ramana V. Davuluri

**Affiliations:** 1 Division of Health and Biomedical Informatics, Department of Preventive Medicine, Northwestern University Feinberg School of Medicine, Chicago, IL USA; 2 The Center for Molecular Innovation and Drug Discovery, Northwestern University, Evanston, IL USA; 3 Department of Biochemistry and Molecular Genetics, Feinberg School of Medicine, Northwestern University, Chicago, IL USA; 4 Department of Pharmacology, Feinberg School of Medicine, Northwestern University, Chicago, IL USA

**Keywords:** Data integration, Computational science

## Abstract

Identifying and evaluating the right target are the most important factors in early drug discovery phase. Most studies focus on one protein ignoring the multiple splice-variant or protein-isoforms, which might contribute to unexpected therapeutic activity or adverse side effects. Here, we present computational analysis of cancer drug-target interactions affected by alternative splicing. By integrating information from publicly available databases, we curated 883 FDA approved or investigational stage small molecule cancer drugs that target 1,434 different genes, with an average of 5.22 protein isoforms per gene. Of these, 618 genes have ≥5 annotated protein-isoforms. By analyzing the interactions with binding pocket information, we found that 76% of drugs either miss a potential target isoform or target other isoforms with varied expression in multiple normal tissues. We present sequence and structure level alignments at isoform-level and make this information publicly available for all the curated drugs. Structure-level analysis showed ligand binding pocket architectures differences in size, shape and electrostatic parameters between isoforms. Our results emphasize how potentially important isoform-level interactions could be missed by solely focusing on the canonical isoform, and suggest that on- and off-target effects at isoform-level should be investigated to enhance the productivity of drug-discovery research.

## Introduction

Discovering a right drug candidate and bringing it to the market is a highly complex process. In recent years, the cost of identifying a new compound and converting it into an FDA approved drug has increased enormously, with an estimated median cost of developing a single cancer drug at $648.0 million^1^. Another study has estimated the cost to drug-makers at $2.6 billion, which includes the cost of the compounds that failed to make it to the market^2^. A promising drug candidate can fail at any of the different stages of the drug discovery process due to various reasons, including lack of clinical efficacy of the potential drug (approximately 57%) and unexpected toxicities or safety concerns (17%)^3,4^. This low productivity is quite troubling, despite great advances in multi-omics technologies and medicinal chemistry assays, together with screening and secondary assays are generating enormous amount of data and knowledge. While the ever increasing datasets should have aided *in silico* experimentation to expedite the overall search for new drugs, the rate of new drugs entering into the market is falling and many drugs approved by regulators are being withdrawn due to toxicity and safety concerns. Most of the chemical biology and genomic approaches are primarily gene-centric (one target-one gene/protein-one disease model) not leading to the desired results. Almost all the experimental and/or computational studies assume “one gene – one protein” paradigm ignoring the true dynamic complexity of the proteome, which include alternative protein isoforms generated from the same gene by mechanisms known as alternative transcription and alternative splicing^5^.

It is now well known that alternative splicing events (exon skipping, intron retention, alternative 5′ or 3′ splice sites, and mutually exclusive exons) and alternative transcriptional events (alternative promoters and alternative 3′ polyadenylation) contribute to the transcriptome and proteome diversity^5^. At least 40% of the human genes produce two or more protein isoforms according to recent annotations of the protein-coding regions that were identically annotated on the human and mouse reference genome assembly in genome annotations produced independently by NCBI and the Ensembl group at EMBL-EBI^6^. The latest annotations of the human genome (GRCh38.p12, GENCODE Release, version 30) contains annotations for 19,986 protein-coding genes and 57,687 full-length protein-coding transcripts, highlighting the complexity of the total proteome that can be expressed by different cells and tissues in the human body. Numerous studies have noted the functional importance of maintaining a coordinated regulation of alternative events in various biological processes, such as tissue development and aging^7–9^. Isoforms of a gene often appear to have different, sometimes even opposite functions, and are tightly regulated to express in a context-specific manner. Conversely, a disruption of such coordinated regulation is often linked to diseases, such as cancer. A recent large transcriptome-wide study revealed that ~19% genes consistently undergo isoform switching (context-dependent differential usage of isoforms) that potentially have functional consequences across 12 solid cancer types^10^. Such genes with isoform switching were previously found to relate to all hallmarks of cancer, in particular apoptosis and metastasis^11,12^.

One best example of such aberrant isoform switching in angiogenesis is *VEGFA* gene, where a switching from anti-angiogenic isoform VEGFA_165_b to pro-angiogenic splice variant VEGFA_165_ is observed in multiple types of cancers^13–17^. Another apoptosis-related example is the *BCL2L1* gene, where a switching from pro-apoptotic short isoform Bcl-xs to anti-apoptotic long splice variant Bcl-xl enable cancer to bypass programmed cell death^18–20^. Examples for metastasis-enabling isoform switching include cell surface adhesion receptors, such as *CD44*^21^ and *CDH1* (E-cadherin)^22^; growth factor receptors, such as *FGFR2*^23^ and *TGFBR2*^24^; as well as other proteins that induce EMT and confer enhanced invasiveness/motility of cells, such as the Rac1b isoform of *RAC1* gene^25–27^ and a short-form, constitutively active isoform of *RON* gene (RonΔ165)^28^. Importantly, the *RON* gene simultaneously produces other tumor-promoting or tumor-opposing isoforms involved in different pathways under different conditions^5^. Meanwhile, the aberrant splicing of *RAC1* gene has also been linked with multiple other cancer hallmarks, including proliferation, genome instability and inflammation^12^. Another aberrant splicing example responsible for sustained proliferative signaling is *BRAF* gene, as alternative isoforms of wild-type and V600E mutant affect its kinase domain and may confer resistance to treatment^29,30^. For isoforms related to evading growth suppressors, human *TP53* itself serves as a perfect example, as many splice variants exists for this well-studied tumor suppressor, some of which are dominant-negative hampering anti-tumor function of wild-type p53^31,32^. In addition, alternatively spliced, dominant negative isoforms of human telomerase gene *TERT* were identified in multiple cancers^33,34^, while splice variants for HLA-G protein were found on surface of tumor cells that enhance immune evasion^35^.

The above examples suggest that disease-causing splice variants, or aberrant isoforms, not only can function as important biomarkers but also have the potential of becoming successful drug targets. Several recent studies have started to explore on the first aspect by performing large-scale pharmacogenomic association studies using transcriptomic expression data^36,37^. However, no study thus far has evaluated the impact of alternative splicing on drug target interactions to the best of our knowledge, perhaps due to lack of availability of sufficient data. In this study, we curated drug interaction data for isoforms from multiple databases, and investigated the binding profile of different isoforms of drug target genes with small molecule compounds, in particular kinase inhibitors. We evaluated the expression patterns of the drug-target genes transcript-variants (or isoforms) in normal and cancer tissues by mining the publicly available databases, such as The Cancer Genome Atlas (TCGA) and Genotype-Tissue Expression (GTEx). Finally, based on our findings, we suggest that the search for the drug targets should also include alternatively spliced protein-isoforms rather than solely focusing on canonical isoform for more efficient and cost-effective drug discovery processes.

## Methods

### Curation of cancer drug-target interaction data with sequence-level binding pockets

We obtained all downloadable entries of drug-target interaction pairs from the Drug Gene Interaction Database (DGIdb), converted from Entrez to Ensembl annotation using MyGene.Info API, and annotated with transcript- and protein-level isoform information from latest Ensembl GRCh37.p13 and GRCh38.p12 assembly^38–40^. The duplicate protein isoform entries having distinct Ensembl protein IDs with the same sequence were removed. We downloaded non-redundant set of receptor data, proteins with sequence and binding site residues identity ≤90% from BioLiP protein function database^41^. We then annotated all the entries in PDB with Ensembl ID in order to filter human drug binding data and achieve a uniformity with our previous anti-tumor drug-target interactions list^42^. PDB uses 3-letter ligand ID code to label the ligands and not all ligands are small molecule drugs, and generic names and ChEMBL IDs are used to annotate drugs in DGIdb^43,44^. Therefore, to integrate small molecule drug information from PDB, we queried DrugBank database and converted ligand ID to drug generic names and ChEMBL ID. Finally, we merged the anti-tumor drug-target list from DGIdb with ligand-protein binding site information from BioLiP to obtain final drug interaction data with binding pockets.

### Extraction of binding residues from canonical sequence and multiple sequence alignments with protein isoforms and Pfam domains

We extracted sequence-level ligand binding sites for all the curated drug targets, and replaced the residues that do not directly interact with the ligand by “−” in each sequence, by our custom R script (http://www.R-project.org/, version 3.4.3). Since multiple PDB entries could be representing the same ligand-protein interaction with slight differences in the binding site residues, we eliminated duplicated versions by combining all the residues. Meanwhile, we treated any two binding sites as independent if their residues have completely different numbering and/or amino acid composition. We generated multiple alignments of sequences by using Bioconductor package *msa*, which allows choice of several alignment algorithms and output alignment plots in a LaTeX format^45^. We generated alignment of binding site sequence with all protein isoforms of a same gene (both GRCh37.p13 and GRCh38.p7 assembly) by Cluster Omega algorithm available in the *msa* package^46^. We obtained Pfam domains from EMBL-EBI Pfam database (https://pfam.xfam.org/) and aligned to the sequences^47^. Sequences of fusion proteins were obtained from FusionGDB (https://ccsm.uth.edu/FusionGDB/)^48^.

### Expression analysis of drug target isoforms

We downloaded harmonized RNA-Seq data (19,131 samples) from TCGA, GTEx and TARGET cohorts from Toil RNA-Seq Recompute Compendium on UCSC Xena browser^49,50^. We used log-transformed RSEM Transcript per Million (TPM) data from only TCGA and GTEx in this analysis. We mapped 30 TCGA cancer types to same/adjacent GTEx tissue, and filtered out all normal controls from TCGA (not pooled with GTEx) for consistency. We computed the Fold change (FC) values for each transcript and upregulation/downregulation declared with logFC > 0 and <0 respectively.

### Structure modeling of protein isoforms and ligand docking

In order to have a better understating on the interactions between the protein isoforms and ligands(drugs), we constructed 3D structures of different protein isoforms by considering their primary sequences and using homology building tools implemented in Schrodinger suite^51^. The MolProbity software^52^ was utilized to assess the suitability of the homology models for the in-silico studies of protein-drug interactions. Then the validated 3D structures of the different isoforms were subjected to protein preparation panel implemented in Prime module of Schrodinger platform^51^. After refining the 3D structures of various isoforms, we used the SiteMap^53^ to identify the druggable pocket in different isoforms. Then we considered a set of known drugs already identified for that disease protein and carried out a ligand preparation suitable to study ligand-protein docking simulations at pH = 7.4 ± 1. The Glide docking tool^54^ in the extra precision mode implemented in Schrodinger platform was used to find the ligand-protein binding mode as well as the binding energy of various ligands (drugs).

## Results

### Majority of cancer drug-target genes have more than five isoforms

**Figure 1:**
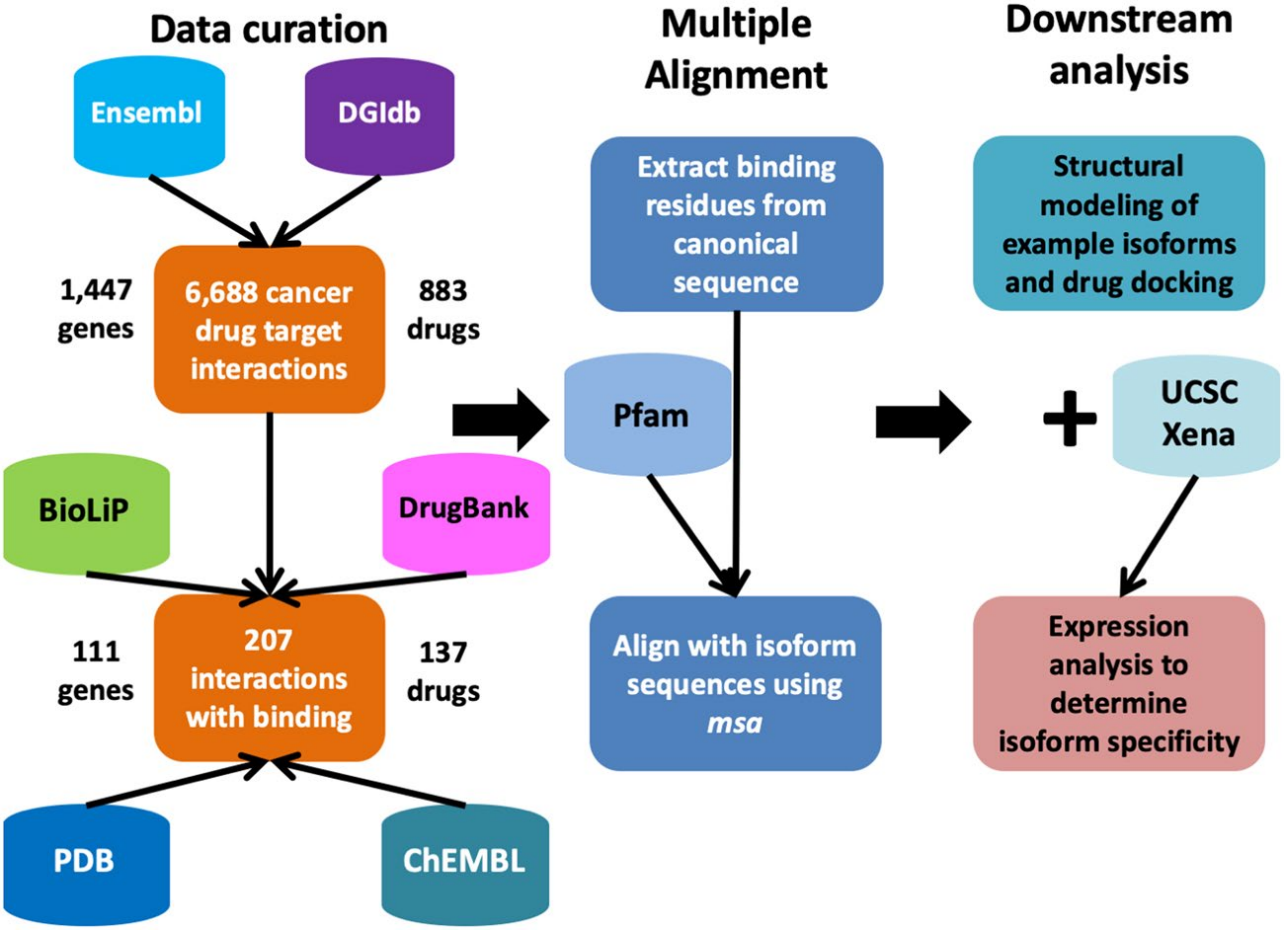
Workflow of the analysis pipeline.

We developed an informatics pipeline (Fig. 1) for evaluating the differences in interaction profiles between a drug and its target protein isoforms, and curated FDA-approved and investigational anti-tumor drug-target interactions with known ligand binding residues. In this study, we focused our analysis on small molecule inhibitors, one of the two major classes of cancer drugs successfully used for targeted therapies. We started with the Drug Gene Interaction Database (DGIdb), a recently developed comprehensive resource containing to-date 29,783 established drug-target interactions, with an emphasis on interactions in cancer context. We obtained all drug interactions available for download with total 42,727 entries corresponding to 2,994 unique genes (based on Entrez gene symbol annotation) and 6,538 drugs with annotated drug names. By setting the antineoplastic preset filter, we extracted 6,688 cancer drug-target interactions, corresponding to 1,447 genes and 883 drugs, among which 3,477 pairs (1,122 genes and 280 drugs) were FDA-approved. To ensure consistency on gene annotation across multiple databases and throughout the analysis, all Entrez gene symbols were queried using MyGene.info API to retrieve Ensembl annotation. Out of the 1,447 genes, 1,434 unique ones were successfully annotated, of which 1,110 were targets of FDA-approved drugs. The resulting 1,434 gene-level drug interaction summary was further annotated with transcript and protein-level isoform information. A summary table was included in Supplementary Files (Supplementary Dataset 1) with a partial list shown here as an example (Table 1). We found that the majority of the target genes in our list contained two or more transcript splice variants and protein isoforms, based on the most recent GENCODE release (version 30) annotations (Fig. S1). Out of 1,434 drug-target genes, 1,036 and 618 have more than five splice variants and protein isoforms respectively. On average, each drug target was found to have 9.23 splice variants and 5.22 protein isoforms. The results indicate that majority of the cancer drug target genes undergo alternative splicing and produce multiple protein isoforms that could be functionally distinct and interact with drugs differently, which emphasizes the importance of taking isoforms and alternative splicing into consideration in drug discovery.

**Table 1:** Example summary of 10 FDA-approved/investigational anti-cancer drug-target interactions.

Gene symbol	Targeting drugs	No. of Transcript variants	No. of Protein isoforms
FGR	ILORASERTIB, XL-228, DASATINIB, ENMD-981693, APITOLISIB, NINTEDANIB	8	3
GCLC	CARBOPLATIN	12	8
CFTR	GENISTEIN, RETINOL	11	6
BAD	ISOSORBIDE, NAVITOCLAX	7	5
CFLAR	BICALUTAMIDE, FINASTERIDE, NINTEDANIB, IDRONOXIL, CABOZANTINIB, DOVITINIB	26	17
TFPI	FULVESTRANT, LOVASTATIN, DACTINOMYCIN	13	8
CD38	SAR-650984, HUMAX-CD38, DARATUMUMAB	5	3
FKBP4	SIROLIMUS	7	5
KDM1A	DIPHENHYDRAMINE HYDROCHLORIDE	7	5
NDUFAB1	METFORMIN HYDROCHLORIDE	5	4

All genes listed have more than one transcript variants as well as protein isoforms due to alternative splicing. Complete list of drug-target interactions for anti-cancer therapy was included in Supplementary Files (Supplementary Dataset 1).

### Drug-target protein isoforms show binding pocket differences on sequence level

Our goal is to evaluate drug-target interactions at splice-variant isoform-level, however, such isoform-level drug binding data with annotations for residues is not currently available to the best of our knowledge. We, therefore, identified the specific interacting residues within the drug binding pocket of each isoform through multiple-sequence alignments. Frist, we obtained the ligand binding information by accessing the BioLiP protein function database, a comprehensive ligand-protein interactions database with ligand binding sites and affinity information. BioLiP has documented specific binding residues either previously validated or predicted using the COACH algorithm, which allows us to retrieve sequence-level drug binding information^55^. We initially obtained a total 180,750 non-redundant entries (as of Apr 2018). Multiple types of ligands are available in BioLiP including DNA/RNAs, peptides, metals and regular ligands. For this study, we confined our analysis to small molecule drugs, the most abundant class of cancer drugs currently in use. We obtained a total of 10,714 binding entries corresponding to 1,790 human genes that interact with 1,633 small molecule drugs, after filtering out multiple ID entries. We eventually merged these entries with our interaction list from DGIdb and obtained a total 207 unique drug-gene interaction pairs that correspond to 111 genes and 137 drugs, among which 51 drugs targeting 67 genes are FDA-approved, with predicted binding pocket information (Supplementary Dataset 2). For each of these drug-gene interaction pairs, we performed multiple sequence alignment between drug-target binding sequence, sequences of protein isoforms, and the PFam functional domain that the drug is known to interact with. Multiple sequence alignment plots for few representative gene-drug pairs (*ABL1* with Imatinib, *EGFR* with Erlotinib and *MAP2K1* with PD-0325901) are discussed here, while all 207 interaction pairs are included as a Supplementary Data (Supplementary Dataset 3). We summarized the isoforms with different binding pockets from the canonical ones in each of the 207 cases, as well as number of aligned PFam functional domains for each gene (Supplementary Dataset 4). Based on the multiple sequence alignments, we found that 86 out of 111 genes have at least one isoform having different binding pocket (either completely or partially missing the binding pocket residues) from the canonical isoform (77.5%). Of these, 55 (64%) genes have more than one functional domain. The sequence alignments of these examples suggest that many approved and investigational drugs can have multi-target effects, and that this database can be a good resource for discovery of isoform-level drug targets. In the following we present three case studies for three different drugs.

**Figure 2:**
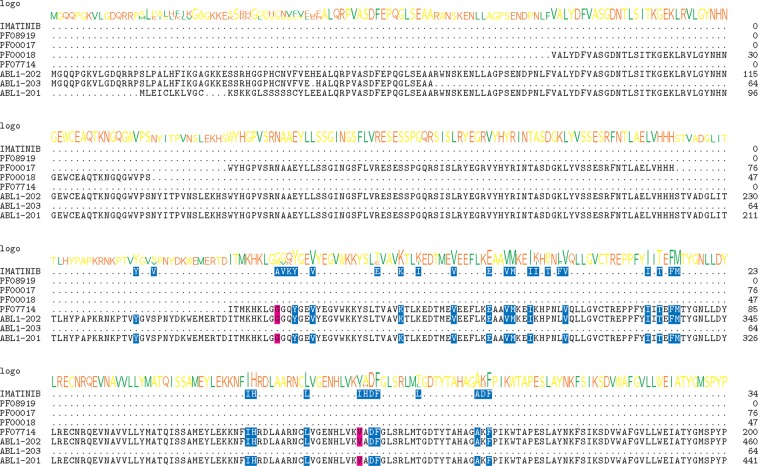
Multiple sequence alignments of predicted interacting residues of Imatinib on different isoforms of ABL1 protein. Cluster Omega was applied to align the binding residues with isoform sequences using Bioconductor package msa. From top to down: predicted Imatinib interacting residues; aligned Pfam domains; ABL1-202; ABL1-203 and ABL1-201. Sequence logo of the consensus sequences were shown on top of each line. Blue shading indicates overlapping residues of a sequence with the predicted binding residues. Purple shading indicates ≥50% of all sequences are conserved with this residue. Pink shading indicates similar amino acids. Only regions near the predicted binding pockets were shown.

Imatinib (Gleevec), a well-known FDA-approved BCR-ABL fusion protein inhibitor for treatment of Philadelphia chromosome positive chronic myelogenous leukemia (CML), targets the tyrosine kinase domain on the ABL side^56^. *ABL1* gene has 3 protein isoforms (ABL1-201 - ENST00000318560, ABL1-202 - ENST00000372348 and ABL1-203 - ENST00000393293), and only two of them contain residues documented within Imatinib binding site (ABL1-201 and ABL1-202). In contrast, ABL1-203 is a shorter isoform (64 residues only) and completely lacks the predicted binding pocket, which will be ignored. The sequence alignment (Fig. 2) shows that ABL1-201 and ABL1-202 have identical sequence in the Imatinib-binding region (the tyrosine kinase domain, as specified by Pfam PF07714), while significant differences occurs in the N-terminal region of these two protein isoforms. Previous studies discovered that the N-terminal region functions as a cap that is responsible for regulation and autoinhibition of its kinase activity^57–59^. In BCR-ABL, however, the whole N-terminal region (corresponding to the first exon on transcript level) is substituted with the BCR protein, resulting in loss of autoinhibition and constitutively active mutant^60^. Since the entire downstream regions of ABL1-201 and 202 are same, the resulting active site of fusion proteins from the two isoforms should ideally have same structure. To confirm this, we also aligned 9 unique BCR-ABL protein sequences in FusionGDB database, and all sequences show overlapping interacting residues with Imatinib (Fig. S2). This sequence level information shows that Imatinib would potentially target all the splice-variant isoforms of BCR-ABL fusion protein, therefore, splice-variation within ABL gene does not affect the Imatinib binding to its targets. We used Imatinib as an example of successful drug not affected by the alternative splicing of the target fusion gene.

**Figure 3:**
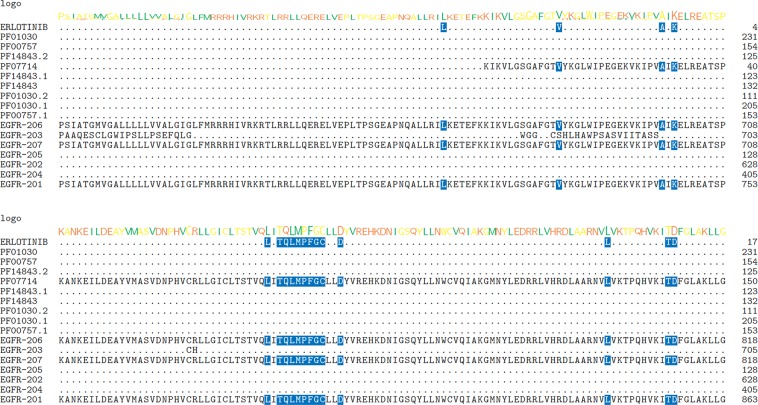
Multiple sequence alignments of predicted interacting residues of Erlotinib on different isoforms of EGFR protein. Cluster Omega was applied to align the binding residues with isoform sequences using Bioconductor package msa. From top to down: predicted Imatinib interacting residues; aligned Pfam domains (different versions represent different sequences with same Pfam ID); EGFR-206; EGFR-203; EGFR-207; EGFR-205; EGFR-202; EGFR-204 and EGFR-201. Sequence logo of the consensus sequences were shown on top of each line. Blue shading indicates overlapping residues of a sequence with the predicted binding residues. Purple shading indicates ≥50% of all sequences are conserved with this residue. Pink shading indicates similar amino acids. Only regions near the predicted binding pockets were shown.

**Figure 4:**
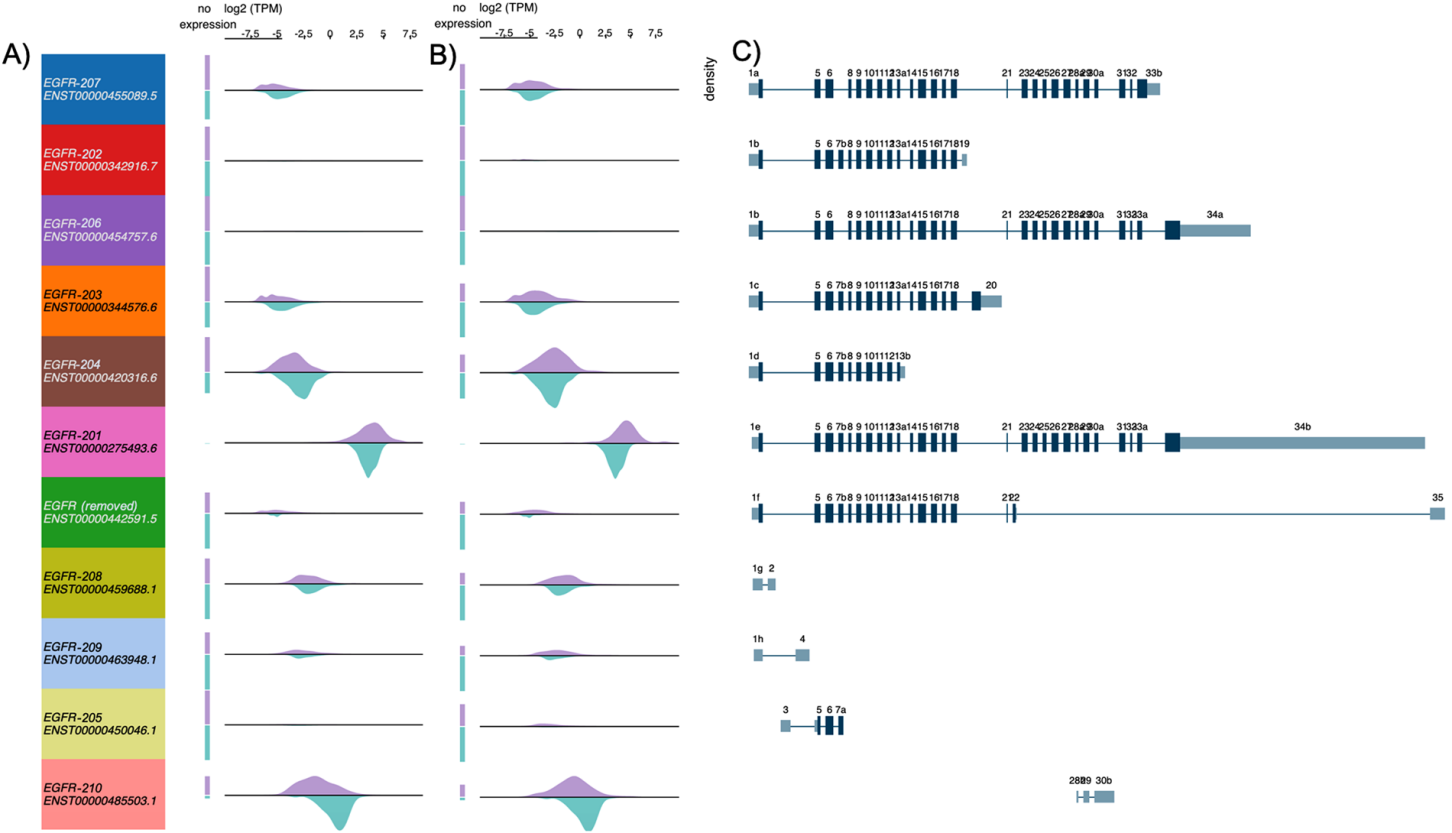
Expression and exon structure of EGFR isoforms. 11 isoforms correspond to (top to down) EGFR-207, 202, 206, 203, 204, 201, 202 (removed in latest version), 208, 209, 205 and 210. Purple density indicates log2(TPM) from (**A**) TCGA Lung Adenocarcinoma (LUAD) samples or (**B**) TCGA Lung Squamous Cell Carcinoma (LUSC) samples; green density indicates those from GTEx normal lung samples. Exon plot (**C**) follows the same order as density plots. All plots are generated using UCSC Xena browser^50^.

Erlotinib selectively targets the ATP binding site within the cytoplasmic tyrosine kinase domain of the EGFR protein and disrupt the kinase activity, and is approved for treatment of Non-small cell lung cancer (NSCLC) as well as investigational in other types of cancers^61,62^. Human *EGFR* gene is well known for producing multiple isoforms via alternative splicing, while its shorter-form splice variants that contain only exons encoding for the extracellular domain have been extensively studied in multiple cancers^63–65^. These shorter protein isoforms were found to be soluble and commonly serve as potential biomarkers in different cancers^66^. These soluble EGFR isoforms (EGFR-202, 203, 204 and 205) lack the ATP binding pockets in the canonical form, as shown in the multiple sequence alignment of EGFR isoforms (Fig. 3), therefore, not expected to serve as targets of Erlotinib. However, other than the full-length isoform (EGFR-201/ENST00000275493), two previously unreported isoforms (EGFR-206/ENST00000454757 and EGFR-207/ENST00000455089) also share the same binding residues and are therefore likely to be targeted by Erlotinib. We checked the expression of these two isoforms in TCGA samples, and found much lower expression than the canonical isoform, EGFR-201, while EGFR-206 is expressed only in a few samples as the density is low (Fig. 4). Importantly, both alternative isoforms, although lowly expressed in cancer samples, showed varied expression in normal GTEx samples, in contrast to their canonical counterpart, which is elevated in tumors, especially in lung squamous cell carcinoma samples. Therefore, these isoforms should be included in further studies for evaluating on- and off-target effects of Erlotinib due to the presence of its binding residues in the target pocket.

**Figure 5:**
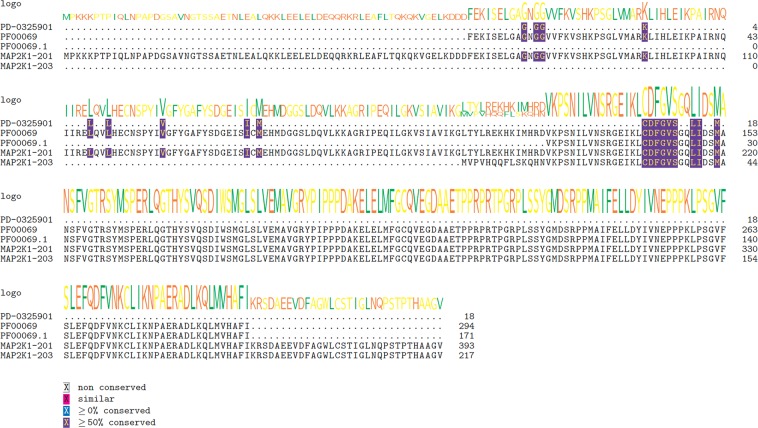
Multiple sequence alignments of predicted interacting residues of PD-0325901 on different isoforms of MAP2K1 protein. Cluster Omega was applied to align the binding residues with isoform sequences using Bioconductor package msa. From top to down: predicted Imatinib interacting residues; aligned Pfam domains (different versions represent different sequences with same Pfam ID); MAP2K1-201 and MAP2K1-203. Sequence logo of the consensus sequences were shown on top of each line. Blue shading indicates overlapping residues of a sequence with the predicted binding residues. Purple shading indicates ≥50% of all sequences are conserved with this residue. Pink shading indicates similar amino acids.

**Figure 6:**
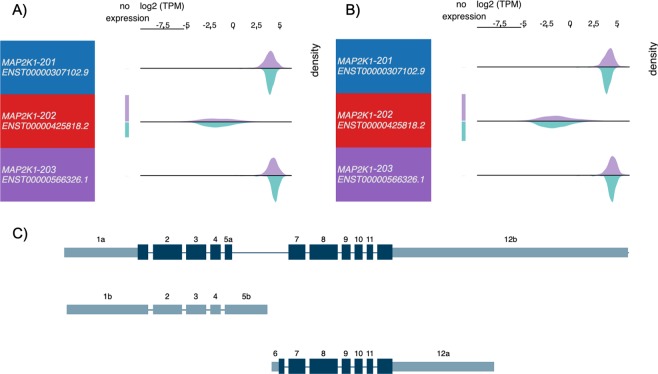
Expression and exon structure of MAP2K1 isoforms. Three isoforms correspond to (top to down) MAP2K1-201, MAP2K1-202 and MAP2K1-203. Purple density indicates log2(TPM) from (**A**) TCGA Lung Adenocarcinoma (LUAD) samples or (**B**) TCGA Lung Squamous Cell Carcinoma (LUSC) samples; green density indicates those from GTEx normal lung samples. Exon plot (**C**) follows the same order as density plots. All plots are generated using UCSC Xena browser^50^.

As third example, we choose PD-0325901, an oral MEK inhibitor that was discontinued for phase II clinical trial in treatment of advanced NSCLC as the efficacy was not met and the cause of lacking objective responses is not fully understood^67^. We investigated the target binding sites of PD-0325901 in both the isoforms of MAP2K1, MAP2K1-201/ENST00000307102 (canonical isoform), and MAP2K1-203/ENST00000566326 (alternative isoform). Based on the multiple sequence alignment (Fig. 5), we found that the alternative isoform lacks the entire upstream domain, which is expected to greatly hamper the interaction with PD-0325901. We investigated the expression of these isoforms and found that the expression of alternative isoform is higher than the canonical form in both lung adenocarcinoma (TCGA-LUAD) and lung squamous cell carcinoma (TCGA-LUSC) (Fig. 6). These results suggest that the therapeutic effects of PD-0325901 might have been significantly impacted due to the lack of partial binding site in the target region of the highly expressed alternative isoform (MAP2K1-203).

### Drugs target genes showed varied expression patterns at isoform-level

**Figure 7:**
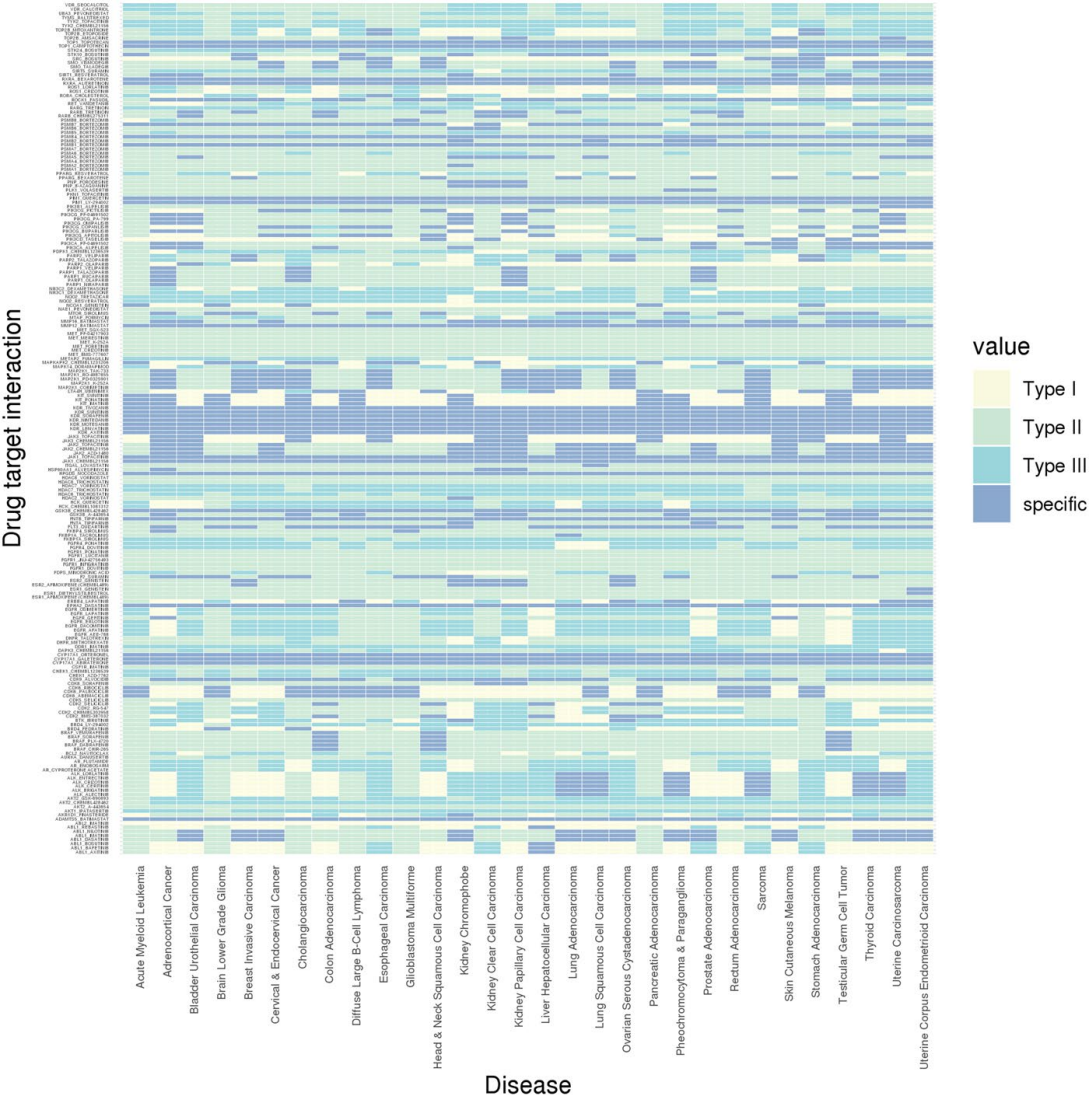
Heatmap of isoform specificity profile for 207 drug target interactions in 33 types of cancers. Type I: targets ≥2 isoforms when at least one is downregulated; Type II: ignores at least one upregulated isoform in cancer; Type III: both Type I and II.

**Figure 8:**
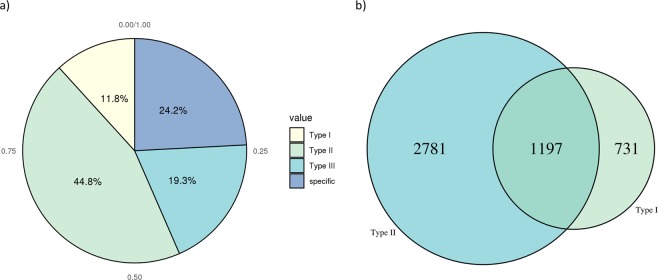
Summary of isoform specificity of different drugs. (**a**) Pie chart showing percentages of the 4 types. Type I: targets ≥2 isoforms when at least one is downregulated; Type II: ignores at least one upregulated isoform in cancer; Type III: both Type I and II. (**b**) Venn diagram showing counts of Type I, Type II and Type III (overlap of Type I and II). Type I: targets ≥2 isoforms when at least one is downregulated; Type II: ignores at least one upregulated isoform in cancer; Type III: both Type I and II.

An ideal small molecule drug should deplete the expression of the target protein, leading to complete suppression of downstream signaling pathways in cancer cells. In order to evaluate which protein isoforms should also be included (and excluded) as targets of the drug, we investigated the transcript-level expression profiles of all protein-coding isoforms of the 111 drug target genes on transcript level across 30 types of human cancers, as compared to their respective normal tissues. By assuming that the isoforms that are beneficial to tumor growth (“onco-isoforms”) should have higher expression in cancer, and vice versa, we defined three types of nonspecific drug-interactions on isoform-level (Fig. 7). If a drug could potentially interact with two or more isoforms of a gene, while at least one of them is downregulated in cancer, we classify such drug-target gene pair as Type I. In contrast, if the drug ignored at least one isoform, which is overexpressed in cancer, we classify it as Type II. Meanwhile, Type I and II are not mutually exclusive, so we defined Type III as being both Type I and II to avoid confusion. Drugs that do not fall into any of these three nonspecific categories will be considered “isoform specific”. Based on this definition, we found that 4,916 (75.8%) out of 6,417 drug-target gene pairs (207 in 30 cancers) as nonspecific, falling into one of the three defined categories (Fig. 8). Only 1,501 pairs are considered specific on isoform-level, meaning that the drug either targets one isoform only or targets the correct isoform(s) among several isoforms of its target (Supplementary Dataset 5).

### Drugs interact with different isoforms of target on structural level

Although we have observed differences in binding pockets between isoforms on sequence level, more convincing evidence that the drugs bind to isoforms of their targets differently can only be obtained by structural-level analysis. In order to understand how a particular drug molecule interacts with different isoforms of a protein, we have analyzed *EGFR* gene with three different isoforms (Isoform-201, Isoform-206 and Isoform-207) along with reported drugs targeting them as a specific case study.

#### EGFR

Searching the isoform database, we found this gene has 7 isoforms of which 3 of them (EGFR-201, EGFR-206 and EGFR-207) contain the ligand binding domains (LBD). Then we searched the protein database and found only one isoform has been solved (EGFR-201). Considering the crystal structure of EGFR-201 as the template, we build the other 3D structures of EGFR-206 and EGFR-207. The structural homology of EGFR-206 and 207 are found to be 96% and 92% with respect to EGFR-201 (crystal structure). Then we superposed the 3 structures to identify the ligand binding pockets for EGFR-206 and 207. After identifying the LBD for all the isoforms, we did a structural homology of the pockets and found that EGFR-206 and 207 have 98% and 97% homology with EGFR-201 respectively.

**Figure 9:**
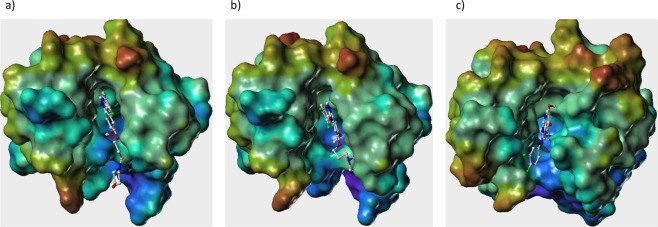
Ligand-binding pocket of EGFR isoforms (**a**) EGFR-201 (**b**) EGFR-206, and (**c**) EGFR-207, with binding of Gefinitib.

A careful analysis of the ligand binding pockets reveals that shape, size and electrostatic map of the LBDs are very different in all the three isoforms (see Fig. 9). Then we considered a set of 7 reported drugs for this disease target and carried out the docking simulations using Glide-XP module of the Schrodinger suite^54^. After analysis of the docked poses we observed that some of the drugs are binding similarly and few others are binding in very different way. For example, in case Lapatinib it has the lowest binding score in EGFR-206 but for EGFR-201 and 207 it has better scores (see Table 2). On the other hand, Erlotinib has similar binding scores in EGFR-201 and EGFR-206 but in the case of EGFR-207 it has much lower score. For Osimertinib drug produces a similar score for EGFR-201 and 206 but for EGFR-207 it has a much better score. Again, for AEE we found the binding scores to be similar in EGFR-201 and 207 but in case of EGFR-206 isoform it showed a much lower score. The rest of the drugs Gefitinib, Afatinib and Dacomitinib have all similar scores across the three isoforms. In order to show that even if the scores are identical in some cases the binding mode seems to be different as the pocket size, shape and electrostatic potential surfaces are different. Here, we analyzed the binding mode of Gefitinib in all the three isoforms and found that it has similar binding score but the binding modes are very different (Fig. 9). Based on these results, we believe that though the binding site residues are almost similar, their ligand binding pocket architectures differ in size, shape and electrostatic parameters as result of which the same drug binds differently in different isoforms with different binding scores.

**Table 2:** Glide -XP scores of the Drug Compounds binding with EGFR-201, EGFR-206 and EGFR-207.

Drug	Glide-XP Score in EGFR-201	Glide-XP Score in EGFR-206	Glide-XP Score in EGFR-207
Lapatinib	−8.11	−3.30	−7.44
Gefitinib	−6.38	−6.56	−6.0
Afatinib	−7.46	−6.32	−6.69
Erlotinib	−7.62	−7.73	−3.73
Dacomitinib	−6.13	−6.11	−6.92
Osimertinib	−4.64	−4.21	−8.41
AEE	−7.40	−4.40	−7.68

## Discussion

Although recent target prediction methods have demonstrated that genomic, chemical and pharmacological data can provide reliable information for drug target interaction prediction; those methods often focus solely on the canonical isoforms (“one gene-one protein” model), thereby carrying the risk of ignoring the on- or off-target isoform-level interactions that are related to the compound’s activity^68^. Several studies have previously linked cancer specific aberrant splicing with drug resistance mechanisms, for example, BCR-ABL35INS protein with a truncated inactive kinase domain that Imatinib is unable to interact^69–72^. However, the therapeutic effect of the drug in the target tissue and unwanted effects in other tissues are poorly understood. Alternative splicing driven protein isoforms can express at varying levels and display different, sometime opposing, functions in multiple tissues and/or organs^73,74^. Further, it was found that a subset of alternative splicing changes could affect protein domain families that were frequently mutated in tumors and potentially disrupt protein-protein interactions in cancer related pathways^75^. In this study, we hypothesized that different protein isoforms, resulting from alternative transcription and/or alternative splicing, could become non-target or off-target drug interacting candidates due to the presence (or absence) of target binding sequence in different isoforms. We developed an informatics pipeline for mining multiple public databases, and curated sequence-level drug target interaction data with actual interacting residues. Our results demonstrate that the majority of small molecule drug targets have multiple protein isoforms, similar to the earlier results we published on a much smaller list of drug candidates^5^. Thus, it is conceivable that the protein isoforms of majority of drug target genes could be functionally distinct and exhibit isoform-level differences in their interactions with the compound.

Indeed, multiple sequence analysis coupled with the data mining of the gene expression profiles in TCGA and GTEx datasets revealed important details, such as (i) drugs that miss alternative isoforms, which are also expressed in cancer but remain non-targets, (ii) drugs that could potentially target alternative isoforms that are variably expressed in several normal tissues, and (iii) drugs that remain specific despite presence of alternative protein isoforms. Further, the structural analysis and drug docking analysis of an example confirmed that the binding of same drug to multiple structurally similar isoforms with different affinities. These results suggest potentially two direct mechanisms that could both contribute to missed- and off-target effects, leading to poor efficacy and drug resistance. We hereby define the concept of isoform-level specificity as being able to only target the correct isoform(s) in a specific context. Based on our analysis, we conclude that majority of drugs currently do not possess such isoform-level specificity, leading to the risk of unwanted target-interactions that are not related to the compound’s activity.

We presented our analysis on three kinase inhibitors as case studies. Imatinib family of Tyrosine Kinase Inhibitors (TKIs) were reported to inhibit not only mutant BCR-ABL fusion protein but also normal ABL protein from noncancer cells, which agrees with our prediction since the binding pocket sequences in ABL isoforms are not affected by alternative splicing^56,76^. However, the binding affinity could be different due to different conformations between the two normal isoforms and the BCR-ABL protein, but the extent of such difference is currently unknown. Meanwhile, normal ABL1 protein was found to act as a tumor suppressor when co-expressed with BCR-ABL, while loss of expression of normal ABL1 results in higher aggressiveness of the disease and reduced sensitivity to Imatinib-like TKIs, although we are not sure which isoform is primarily responsible for this effect either^77,78^. These results reinforce the fact that potentially important splicing changes in ABL isoforms can influence the therapeutic effect of Imatinib-like TKIs, which require further investigation in future studies.

In our analysis of EGFR isoforms, we found almost no or relatively low expression in TCGA samples for two previously uncharacterized and structurally different isoforms (EGFR-206 and EGFR-207), when compared with the canonical isoform. From structural docking analysis, we found that different drugs can interact with all three isoforms differently. Currently, whether the two alternative isoforms function in similar or opposite manner as the dysregulated primary isoform (which is oncogenic and often overexpressed) is still unclear. However, the alternative isoforms, which displayed varied and higher expression in normal tissues than in cancer samples, may function as regulators or tumor suppressors antagonizing the activity of the oncogenic isoform. In such cases, direct inhibition of these isoforms may not be desired. Although the exact functions of these isoforms remain uncertain at this point, it is conceivable that differentiating targets and non-targets at isoform-level is a critical step in early drug-target identification studies.

We also identified a new isoform of *MAP2K1* gene (MAP2K1-203), which is annotated across different publicly available genome databases but not reported in the literature. This isoform lacks exon 1-5 including part of the kinase domain, indicating that it may have disrupted kinase activity (Figs. 5 and 6). Most importantly, this isoform, instead of the canonical long isoform, is the major one expressed in both lung adenocarcinoma and lung squamous cell carcinoma samples. PD-0325901, small molecule drug which targets MAP2K1failed the phase II clinical trial due to lack of objective responses and severe side effects. It is possible that this highly expressed alternative isoform, which remains as non-target due to lack of target sequence and kinase domain, could be an important contributing factor for the drug failure. These results demonstrate that designing more effective drug requires not only gene-level but isoform-level understanding of the target.

We have to admit that our current study has a few limitations, due to restrictions on data availability. The initial problem is that the mapping of isoforms between public online database and previous literatures is poor. For instance, the numbering of exons between these two sources often does not agree with each other. Many isoforms that were previously documented in literature were not found in public databases such as Ensembl. This creates huge difficulty for us in doing structural and functional annotations of these isoforms. As such, our analysis is based primarily on two assumptions: (1) isoforms that are more beneficial for cancer progression are commonly overexpressed and (2) isoforms that are more expressed in cancer should be the primary targets to be inhibited, but not the other way around. This is clearly a limitation as these two assumptions can be wrong, but currently we lack better measures to comprehensively evaluate functions of these unknown isoforms. Moreover, it would have been more convincing if actual protein-level expression of these isoforms (e.g. from Mass Spectrometry data) are included. To the best of our knowledge, so far there is no comprehensive database that contains expression of all protein isoforms at a whole proteome scale either. We believe that the significance of understanding drug targets on isoform level should be even better highlighted, provided that such data is available. Nevertheless, our findings complement those of a recent study that discovered mean mRNA expression across tissues and standard deviation of expression across tissues as the two dominant features that discriminate successful drugs from failed ones^79^.

That being said, we hope that our study can inspire more future research that further explores the potential of isoform-level drug design. To achieve this goal, sufficient structural and functional understanding of these isoforms is crucial. An essential next step would be to robustly identify more isoform-level cancer biomarkers and associate them with sensitivity of drugs via computational approaches. Accurate structural modeling and prediction of these isoforms are also very important if isoform-level drug design is desired, given that currently no database contains such structural information in a well-annotated manner. Different databases should also further integrate isoform-level data and annotation with previous literatures and make sure that they agree each other, especially the functional annotations of rare isoforms.

## Conclusions

Given the limited clinical success with the small molecule inhibitors and inconsistencies in gene-level drug–target interaction predictions, integrating isoform expression data along-with genomic, chemical and pharmacological data across different databases might be a prudent strategy for improving the confidence of drug target interaction predictions. This study demonstrates how alternative splicing effects target binding residues in the target genes, both at sequence and structure level, and how varied expression of target gene isoforms in different normal tissues and cancers might lead to missed- and/or off-target effects of the drug molecule. Therefore, with a better understanding of the isoform-level expression patterns from transcriptome and proteomics studies, future drug target identification studies can increase their success by incorporating isoform-level sequence and structure data.

## Supplementary information

Supplementary Information.

.Supplementary Information 2.

Supplementary Information 3.

Supplementary Information 4.

Supplementary Information 5.

Supplementary Information 6
