# Supplementary material for: In silico analysis of alternative splicing on drug-target gene interactions
Source: Sci Rep. 2020 Jan 10;10:134. doi: 10.1038/s41598-019-56894-x (PMC6954184; doi:10.1038/s41598-019-56894-x)
Supplement: Supplementary file 1 — Supplementary Information. [file 41598_2019_56894_MOESM1_ESM.pdf]

In silico analysis of alternative splicing on drug-target gene interactions

Yanrong Ji[1], Rama K Mishra[2,3,4] and Ramana V Davuluri[1*]

[1]Division of Health and Biomedical Informatics, Department of Preventive Medicine, Northwestern University Feinberg School of Medicine, Chicago, IL, USA.

[2]The Center for Molecular Innovation and Drug Discovery, Northwestern University, Evanston, IL, USA.

[3]Department of Biochemistry and Molecular Genetics, Feinberg School of Medicine, Northwestern University, Chicago, IL, USA

[4]Department of Pharmacology, Feinberg School of Medicine, Northwestern University, Chicago, IL, USA

*Corresponding Author: Ramana V Davuluri (ramana.davuluri@northwestern.edu)

Supplementary figures

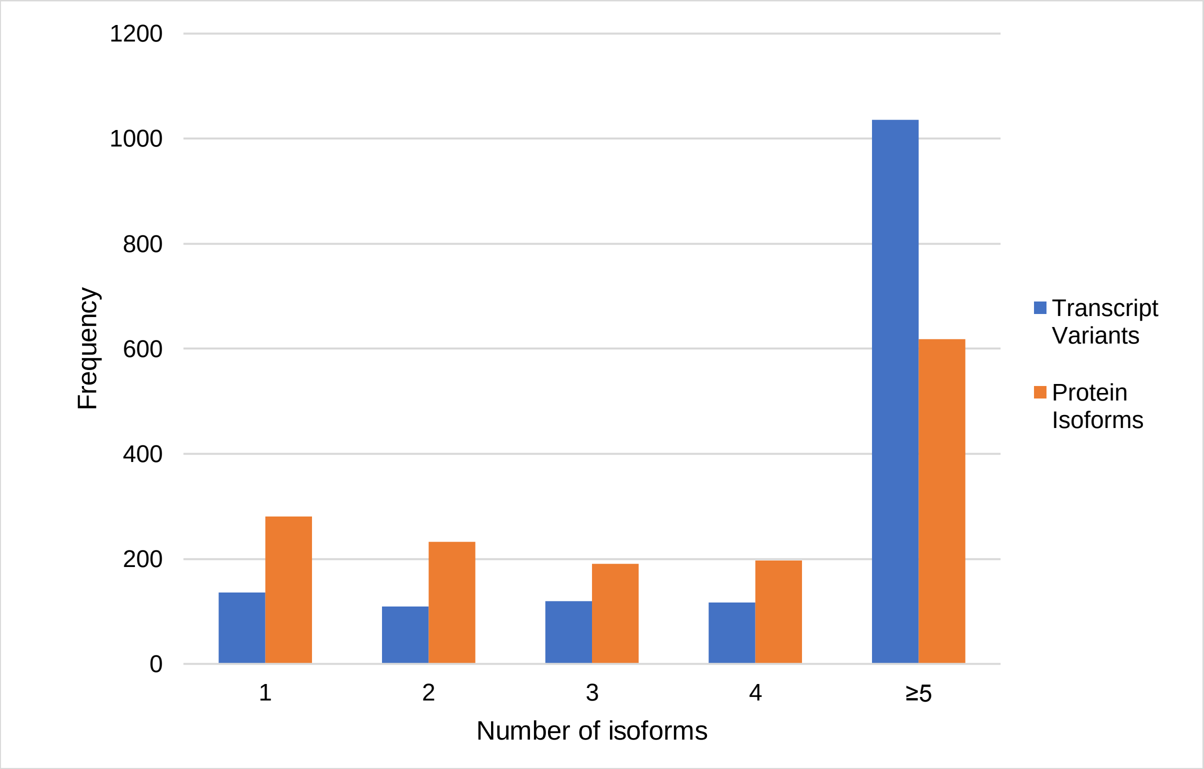

Figure S1. Frequency of drug-target genes with x transcript/protein isoforms. Most drug targets have ≥5 transcript and protein isoforms.

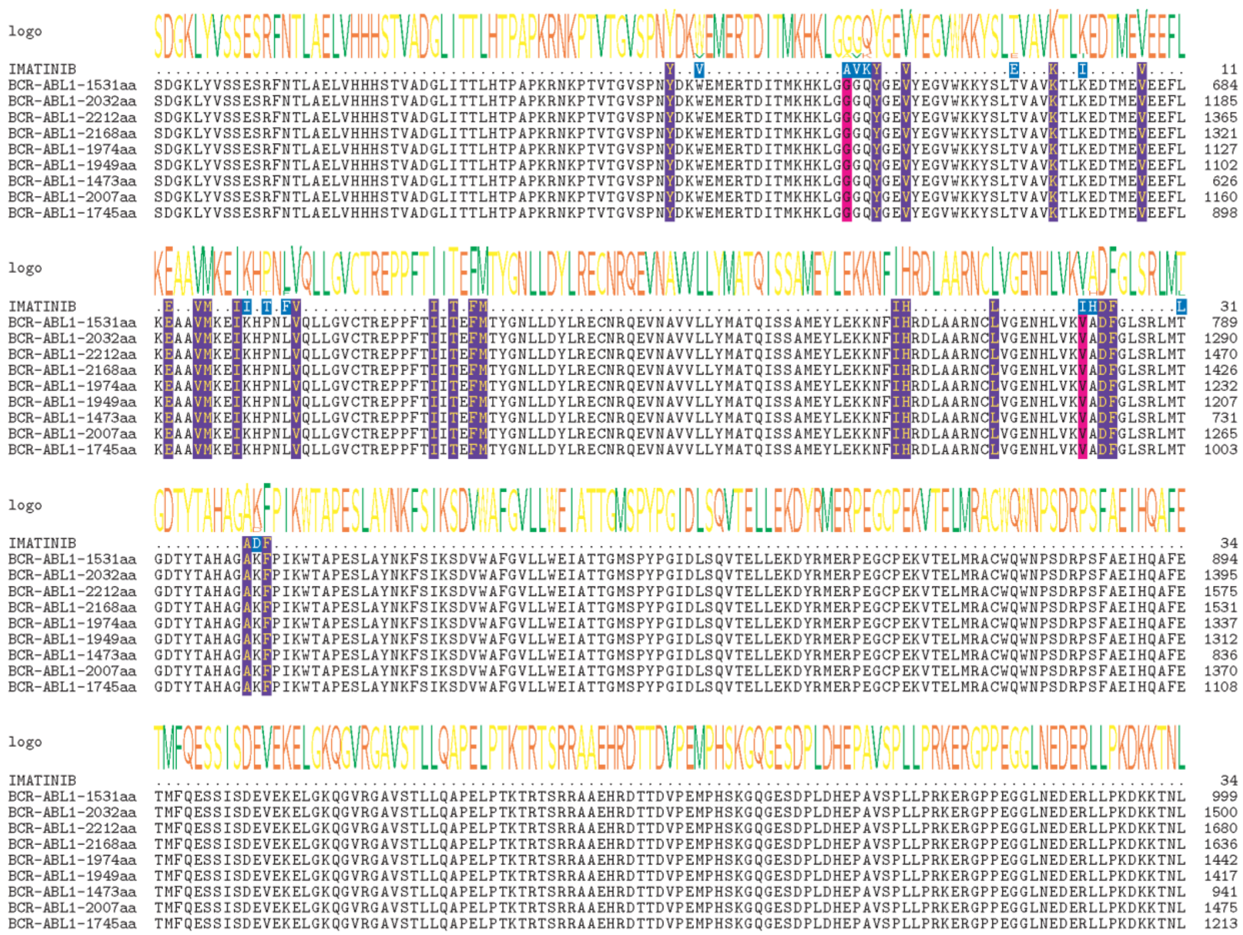

Figure S2. Multiple sequence alignments of predicted interacting residues of Imatinib on different BCR-ABL1 fusion protein sequences. Cluster Omega was applied to align the binding residues with fusion protein sequences using Bioconductor package *msa*. Sequence logo of the consensus sequences were shown on top of each line. Blue shading indicates overlapping residues of a sequence with the predicted binding residues. Purple shading indicates ≥ 50% of all sequences are conserved with this residue. Pink shading indicates similar amino acids.
