# Supplementary material for: In silico analysis of alternative splicing on drug-target gene interactions
Source: Sci Rep. 2020 Jan 10;10:134. doi: 10.1038/s41598-019-56894-x (PMC6954184; doi:10.1038/s41598-019-56894-x)
Supplement: Supplementary file 4 — Supplementary Information 4. [file 41598_2019_56894_MOESM4_ESM.pdf]

In silico analysis of alternative splicing on drug-target gene interactions

Yanrong Ji[1], Rama K Mishra[2,3,4] and Ramana V Davuluri[1*]

[1]Division of Health and Biomedical Informatics, Department of Preventive Medicine, Northwestern University Feinberg School of Medicine, Chicago, IL, USA.

[2]The Center for Molecular Innovation and Drug Discovery, Northwestern University, Evanston, IL, USA.

[3]Department of Biochemistry and Molecular Genetics, Feinberg School of Medicine, Northwestern University, Chicago, IL, USA

[4]Department of Pharmacology, Feinberg School of Medicine, Northwestern University, Chicago, IL, USA

*Corresponding Author: Ramana V Davuluri (ramana.davuluri@northwestern.edu)

```
logo
AXITINIB   ...................................................................................................................   0
PF08919    ...................................................................................................................   0
PF00017    ...................................................................................................................   0
PF00018    .....................................................................................VALYDFVASGDNTLSITKGEKLRVLGYNHN  30
PF07714    ...................................................................................................................   0
ABL1-202   MGQQPGKVLGDQRRPSLPALHFIKGAGKKESSRHGGPHCNVFVEHEALQRPVASDFEPQGLSEAARWNSKENLLAGPSENDPNLFVALYDFVASGDNTLSITKGEKLRVLGYNHN 115
ABL1-203   MGQQPGKVLGDQRRPSLPALHFIKGAGKKESSRHGGPHCNVFVE.HALQRPVASDFEPQGLSEAA..................................................  64
ABL1-201   ...............MLEICLKLVGC....KSKKGLSSSSSCYLEEALQRPVASDFEPQGLSEAARWNSKENLLAGPSENDPNLFVALYDFVASGDNTLSITKGEKLRVLGYNHN  96

logo
AXITINIB   ...................................................................................................................   0
PF08919    ...................................................................................................................   0
PF00017    ..............................WYHGPVSRNAAEYLLSSGINGSFLVRESESSPGQRSISLRYEGRVYHYRINTASDGKLYVSSESRFNTLAELVHHH.........  76
PF00018    GEWCEAQTKNGQGWVPS..................................................................................................  47
PF07714    ...................................................................................................................   0
ABL1-202   GEWCEAQTKNGQGWVPSNYITPVNSLEKHSWYHGPVSRNAAEYLLSSGINGSFLVRESESSPGQRSISLRYEGRVYHYRINTASDGKLYVSSESRFNTLAELVHHHSTVADGLIT 230
ABL1-203   ...................................................................................................................  64
ABL1-201   GEWCEAQTKNGQGWVPSNYITPVNSLEKHSWYHGPVSRNAAEYLLSSGINGSFLVRESESSPGQRSISLRYEGRVYHYRINTASDGKLYVSSESRFNTLAELVHHHSTVADGLIT 211

logo
AXITINIB   ....................................LG...Y..V............A.K...........................................TEFMT.G.....  12
PF08919    ...................................................................................................................   0
PF00017    ...................................................................................................................  76
PF00018    ...................................................................................................................  47
PF07714    ..............................ITMKHKLGGGQYGEVYEGVWKKYSLTVAVKTLKEDTMEVEEFLKEAAVMKEIKHPNLVQLLGVCTREPPFYIITEFMTYGNLLDY  85
ABL1-202   TLHYPAPKRNKPTVYGVSPNYDKWEMERTDITMKHKLGGGQYGEVYEGVWKKYSLTVAVKTLKEDTMEVEEFLKEAAVMKEIKHPNLVQLLGVCTREPPFYIITEFMTYGNLLDY 345
ABL1-203   ...................................................................................................................  64
ABL1-201   TLHYPAPKRNKPTVYGVSPNYDKWEMERTDITMKHKLGGGQYGEVYEGVWKKYSLTVAVKTLKEDTMEVEEFLKEAAVMKEIKHPNLVQLLGVCTREPPFYIITEFMTYGNLLDY 326

logo
AXITINIB   .........................................N.L.........ADF...........................................................  17
PF08919    ...................................................................................................................   0
PF00017    ...................................................................................................................  76
PF00018    ...................................................................................................................  47
PF07714    LRECNRQEVNAVVLLYMATQISSAMEYLEKKNFIHRDLAARNCLVGENHLVKVADFGLSRLMTGDTYTAHAGAKFPIKWTAPESLAYNKFSIKSDVWAFGVLLWEIATYGMSPYP 200
ABL1-202   LRECNRQEVNAVVLLYMATQISSAMEYLEKKNFIHRDLAARNCLVGENHLVKVADFGLSRLMTGDTYTAHAGAKFPIKWTAPESLAYNKFSIKSDVWAFGVLLWEIATYGMSPYP 460
ABL1-203   ...................................................................................................................  64
ABL1-201   LRECNRQEVNAVVLLYMATQISSAMEYLEKKNFIHRDLAARNCLVGENHLVKVADFGLSRLMTGDTYTAHAGAKFPIKWTAPESLAYNKFSIKSDVWAFGVLLWEIATYGMSPYP 441
```

```
logo
            GIDLSQVYELLEKDYRMERPEGCPEKVYELMRACWQWNPSDRPSFAEIHQAFETMFQESSISDEVEKELGKQGVRGAVSTLLQAPELPTKTRTSRRAAEHRDTTDVPEMPHSKGQ
AXITINIB    ...................................................................................................................   17
PF08919     ...................................................................................................................    0
PF00017     ...................................................................................................................   76
PF00018     ...................................................................................................................   47
PF07714     GIDLSQVYELLEKDYRMERPEGCPEKVYELMRACWQWNPSDRPSFAEIHQA................................................................  251
ABL1-202    GIDLSQVYELLEKDYRMERPEGCPEKVYELMRACWQWNPSDRPSFAEIHQAFETMFQESSISDEVEKELGKQGVRGAVSTLLQAPELPTKTRTSRRAAEHRDTTDVPEMPHSKGQ  575
ABL1-203    ...................................................................................................................   64
ABL1-201    GIDLSQVYELLEKDYRMERPEGCPEKVYELMRACWQWNPSDRPSFAEIHQAFETMFQESSISDEVEKELGKQGVRGAVSTLLQAPELPTKTRTSRRAAEHRDTTDVPEMPHSKGQ  556

logo
            GESDPLDHEPAVSPLLPRKERGPPEGGLNEDERLLPKDKKTNLFSALIKKKKKTAPTPPKRSSSFREMDGQPERRGAGEEEGRDISNGALAFTPLDTADPAKSPKPSNGAGVPNG
AXITINIB    ...................................................................................................................   17
PF08919     ...................................................................................................................    0
PF00017     ...................................................................................................................   76
PF00018     ...................................................................................................................   47
PF07714     ...................................................................................................................  251
ABL1-202    GESDPLDHEPAVSPLLPRKERGPPEGGLNEDERLLPKDKKTNLFSALIKKKKKTAPTPPKRSSSFREMDGQPERRGAGEEEGRDISNGALAFTPLDTADPAKSPKPSNGAGVPNG  690
ABL1-203    ...................................................................................................................   64
ABL1-201    GESDPLDHEPAVSPLLPRKERGPPEGGLNEDERLLPKDKKTNLFSALIKKKKKTAPTPPKRSSSFREMDGQPERRGAGEEEGRDISNGALAFTPLDTADPAKSPKPSNGAGVPNG  671

logo
            ALRESGGSGFRSPHLWKKSSTLTSSRLATGEEEGGGSSSKRFLRSCSASCVPHGAKDTEWRSVTLPRDLQSTGRQFDSSTFGGHKSEKPALPRKRAGENRSDQVTRGTVTPPPRL
AXITINIB    ...................................................................................................................   17
PF08919     ...................................................................................................................    0
PF00017     ...................................................................................................................   76
PF00018     ...................................................................................................................   47
PF07714     ...................................................................................................................  251
ABL1-202    ALRESGGSGFRSPHLWKKSSTLTSSRLATGEEEGGGSSSKRFLRSCSASCVPHGAKDTEWRSVTLPRDLQSTGRQFDSSTFGGHKSEKPALPRKRAGENRSDQVTRGTVTPPPRL  805
ABL1-203    ...................................................................................................................   64
ABL1-201    ALRESGGSGFRSPHLWKKSSTLTSSRLATGEEEGGGSSSKRFLRSCSASCVPHGAKDTEWRSVTLPRDLQSTGRQFDSSTFGGHKSEKPALPRKRAGENRSDQVTRGTVTPPPRL  786

logo
            VKKNEEAADEVFKDIMESSPGSSPPNLTPKPLRRQVTVAPASGLPHKEEAGKGSALGTPAAAEPVTPTSKAGSGAPGGTSKGPAEESRVRRHKHSSESPGRDKGKLSRLKPAPPP
AXITINIB    ...................................................................................................................   17
PF08919     ...................................................................................................................    0
PF00017     ...................................................................................................................   76
PF00018     ...................................................................................................................   47
PF07714     ...................................................................................................................  251
ABL1-202    VKKNEEAADEVFKDIMESSPGSSPPNLTPKPLRRQVTVAPASGLPHKEEAGKGSALGTPAAAEPVTPTSKAGSGAPGGTSKGPAEESRVRRHKHSSESPGRDKGKLSRLKPAPPP  920
ABL1-203    ...................................................................................................................   64
ABL1-201    VKKNEEAADEVFKDIMESSPGSSPPNLTPKPLRRQVTVAPASGLPHKEEAGKGSALGTPAAAEPVTPTSKAGSGAPGGTSKGPAEESRVRRHKHSSESPGRDKGKLSRLKPAPPP  901
```

```
logo
           PPAASAGKAGGKPSQSPSQEAAGEAVLGAKTKATSLVDAVNSDAAKPSQPGEGLKKPVLPATPKPQSAKPSGTPISPAPVPSTLPSASSALAGDQPSSTAFIPLISTRVSLRKTR
AXITINIB   ...................................................................................................................   17
PF08919    ...................................................................................................................    0
PF00017    ...................................................................................................................   76
PF00018    ...................................................................................................................   47
PF07714    ...................................................................................................................  251
ABL1-202   PPAASAGKAGGKPSQSPSQEAAGEAVLGAKTKATSLVDAVNSDAAKPSQPGEGLKKPVLPATPKPQSAKPSGTPISPAPVPSTLPSASSALAGDQPSSTAFIPLISTRVSLRKTR 1035
ABL1-203   ...................................................................................................................   64
ABL1-201   PPAASAGKAGGKPSQSPSQEAAGEAVLGAKTKATSLVDAVNSDAAKPSQPGEGLKKPVLPATPKPQSAKPSGTPISPAPVPSTLPSASSALAGDQPSSTAFIPLISTRVSLRKTR 1016

logo
           QPPERIASGAITKGVVLDSTEALCLAISRNSEQMASHSAVLEAGKNLYTFCVSYVDSIQQMRNKFAFREAINKLENNLRELQICPATAGSGPAATQDFSKLLSSVKEISDIVQR
AXITINIB   ..................................................................................................................   17
PF08919    ..........ITKGVVLDSTEALCLAISRNSEQMASHSAVLEAGKNLYTFCVSYVDSIQQMRNKFAFREAINKLENNLRELQICPATAGSGPAATQDFSKLLSSVKEISDIVQR  104
PF00017    ..................................................................................................................   76
PF00018    ..................................................................................................................   47
PF07714    ..................................................................................................................  251
ABL1-202   QPPERIASGAITKGVVLDSTEALCLAISRNSEQMASHSAVLEAGKNLYTFCVSYVDSIQQMRNKFAFREAINKLENNLRELQICPATAGSGPAATQDFSKLLSSVKEISDIVQR 1149
ABL1-203   ..................................................................................................................   64
ABL1-201   QPPERIASGAITKGVVLDSTEALCLAISRNSEQMASHSAVLEAGKNLYTFCVSYVDSIQQMRNKFAFREAINKLENNLRELQICPATAGSGPAATQDFSKLLSSVKEISDIVQR 1130
```

X non conserved
X similar
X ≥ 0% conserved
X ≥ 50% conserved

logo

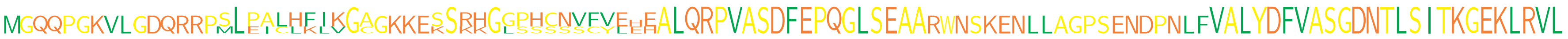

```
BAFETINIB   ..............................................................................................................   0
PF08919     ..............................................................................................................   0
PF00017     ..............................................................................................................   0
PF00018     .....................................................................................VALYDFVASGDNTLSITKGEKLRVL   25
PF07714     ..............................................................................................................   0
ABL1-202    MGQQPGKVLGDQRRPSLPALHFIKGAGKKESSRHGGPHCNVFVEHEALQRPVASDFEPQGLSEAARWNSKENLLAGPSENDPNLFVALYDFVASGDNTLSITKGEKLRVL   110
ABL1-203    MGQQPGKVLGDQRRPSLPALHFIKGAGKKESSRHGGPHCNVFVE.HALQRPVASDFEPQGLSEAA.............................................   64
ABL1-201    ...............MLEICLKLVGC....KSKKGLSSSSSCYLEEALQRPVASDFEPQGLSEAARWNSKENLLAGPSENDPNLFVALYDFVASGDNTLSITKGEKLRVL   91
```

logo

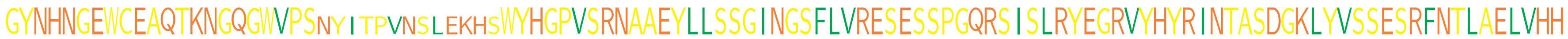

```
BAFETINIB   ..............................................................................................................   0
PF08919     ..............................................................................................................   0
PF00017     ...................................WYHGPVSRNAAEYLLSSGINGSFLVRESESSPGQRSISLRYEGRVYHYRINTASDGKLYVSSESRFNTLAELVHH   75
PF00018     GYNHNGEWCEAQTKNGQGWVPS........................................................................................   47
PF07714     ..............................................................................................................   0
ABL1-202    GYNHNGEWCEAQTKNGQGWVPSNYITPVNSLEKHSWYHGPVSRNAAEYLLSSGINGSFLVRESESSPGQRSISLRYEGRVYHYRINTASDGKLYVSSESRFNTLAELVHH   220
ABL1-203    ..............................................................................................................   64
ABL1-201    GYNHNGEWCEAQTKNGQGWVPSNYITPVNSLEKHSWYHGPVSRNAAEYLLSSGINGSFLVRESESSPGQRSISLRYEGRVYHYRINTASDGKLYVSSESRFNTLAELVHH   201
```

logo

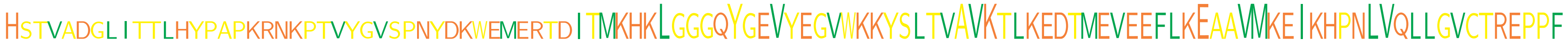

```
BAFETINIB   ..............................................L....Y..V............AVK..............E..VM..I....LV............   12
PF08919     ..............................................................................................................   0
PF00017     H.............................................................................................................   76
PF00018     ..............................................................................................................   47
PF07714     ........................................ITMKHKLGGGQYGEVYEGVWKKYSLTVAVKTLKEDTMEVEEFLKEAAVMKEIKHPNLVQLLGVCTREPPF   70
ABL1-202    HSTVADGLITTLHYPAPKRNKPTVYGVSPNYDKWEMERTDITMKHKLGGGQYGEVYEGVWKKYSLTVAVKTLKEDTMEVEEFLKEAAVMKEIKHPNLVQLLGVCTREPPF   330
ABL1-203    ..............................................................................................................   64
ABL1-201    HSTVADGLITTLHYPAPKRNKPTVYGVSPNYDKWEMERTDITMKHKLGGGQYGEVYEGVWKKYSLTVAVKTLKEDTMEVEEFLKEAAVMKEIKHPNLVQLLGVCTREPPF   311
```

logo

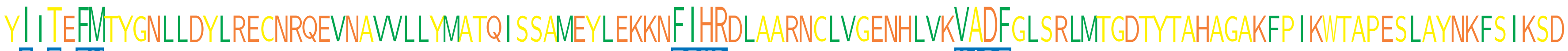

```
BAFETINIB   .I.T.FM........................................FIHR................VADF.......................................   24
PF08919     ..............................................................................................................   0
PF00017     ..............................................................................................................   76
PF00018     ..............................................................................................................   47
PF07714     YIITEFMTYGNLLDYLRECNRQEVNAVVLLYMATQISSAMEYLEKKNFIHRDLAARNCLVGENHLVKVADFGLSRLMTGDTYTAHAGAKFPIKWTAPESLAYNKFSIKSD   180
ABL1-202    YIITEFMTYGNLLDYLRECNRQEVNAVVLLYMATQISSAMEYLEKKNFIHRDLAARNCLVGENHLVKVADFGLSRLMTGDTYTAHAGAKFPIKWTAPESLAYNKFSIKSD   440
ABL1-203    ..............................................................................................................   64
ABL1-201    YIITEFMTYGNLLDYLRECNRQEVNAVVLLYMATQISSAMEYLEKKNFIHRDLAARNCLVGENHLVKVADFGLSRLMTGDTYTAHAGAKFPIKWTAPESLAYNKFSIKSD   421
```

```
logo
             VWAFGVLLWEIATYGMSPYPGIDLSQVYELLEKDYRMERPEGCPEKVYELMRACWQWNPSDRPSFAEIHQAFETMFQESSISDEVEKELGKQGVRGAVSTLLQAPELPTK
BAFETINIB    ..............................................................................................................   24
PF08919      ..............................................................................................................    0
PF00017      ..............................................................................................................   76
PF00018      ..............................................................................................................   47
PF07714      VWAFGVLLWEIATYGMSPYPGIDLSQVYELLEKDYRMERPEGCPEKVYELMRACWQWNPSDRPSFAEIHQA.......................................  251
ABL1-202     VWAFGVLLWEIATYGMSPYPGIDLSQVYELLEKDYRMERPEGCPEKVYELMRACWQWNPSDRPSFAEIHQAFETMFQESSISDEVEKELGKQGVRGAVSTLLQAPELPTK  550
ABL1-203     ..............................................................................................................   64
ABL1-201     VWAFGVLLWEIATYGMSPYPGIDLSQVYELLEKDYRMERPEGCPEKVYELMRACWQWNPSDRPSFAEIHQAFETMFQESSISDEVEKELGKQGVRGAVSTLLQAPELPTK  531

logo
             TRTSRRAAEHRDTTDVPEMPHSKGQGESDPLDHEPAVSPLLPRKERGPPEGGLNEDERLLPKDKKTNLFSALIKKKKKTAPTPPKRSSSFREMDGQPERRGAGEEEGRDI
BAFETINIB    ..............................................................................................................   24
PF08919      ..............................................................................................................    0
PF00017      ..............................................................................................................   76
PF00018      ..............................................................................................................   47
PF07714      ..............................................................................................................  251
ABL1-202     TRTSRRAAEHRDTTDVPEMPHSKGQGESDPLDHEPAVSPLLPRKERGPPEGGLNEDERLLPKDKKTNLFSALIKKKKKTAPTPPKRSSSFREMDGQPERRGAGEEEGRDI  660
ABL1-203     ..............................................................................................................   64
ABL1-201     TRTSRRAAEHRDTTDVPEMPHSKGQGESDPLDHEPAVSPLLPRKERGPPEGGLNEDERLLPKDKKTNLFSALIKKKKKTAPTPPKRSSSFREMDGQPERRGAGEEEGRDI  641

logo
             SNGALAFTPLDTADPAKSPKPSNGAGVPNGALRESGGSGFRSPHLWKKSSTLTSSRLATGEEEGGGSSSKRFLRSCSASCVPHGAKDTEWRSVTLPRDLQSTGRQFDSST
BAFETINIB    ..............................................................................................................   24
PF08919      ..............................................................................................................    0
PF00017      ..............................................................................................................   76
PF00018      ..............................................................................................................   47
PF07714      ..............................................................................................................  251
ABL1-202     SNGALAFTPLDTADPAKSPKPSNGAGVPNGALRESGGSGFRSPHLWKKSSTLTSSRLATGEEEGGGSSSKRFLRSCSASCVPHGAKDTEWRSVTLPRDLQSTGRQFDSST  770
ABL1-203     ..............................................................................................................   64
ABL1-201     SNGALAFTPLDTADPAKSPKPSNGAGVPNGALRESGGSGFRSPHLWKKSSTLTSSRLATGEEEGGGSSSKRFLRSCSASCVPHGAKDTEWRSVTLPRDLQSTGRQFDSST  751

logo
             FGGHKSEKPALPRKRAGENRSDQVTRGTVTPPPRLVKKNEEAADEVFKDIMESSPGSSPPNLTPKPLRRQVTVAPASGLPHKEEAGKGSALGTPAAAEPVTPTSKAGSGA
BAFETINIB    ..............................................................................................................   24
PF08919      ..............................................................................................................    0
PF00017      ..............................................................................................................   76
PF00018      ..............................................................................................................   47
PF07714      ..............................................................................................................  251
ABL1-202     FGGHKSEKPALPRKRAGENRSDQVTRGTVTPPPRLVKKNEEAADEVFKDIMESSPGSSPPNLTPKPLRRQVTVAPASGLPHKEEAGKGSALGTPAAAEPVTPTSKAGSGA  880
ABL1-203     ..............................................................................................................   64
ABL1-201     FGGHKSEKPALPRKRAGENRSDQVTRGTVTPPPRLVKKNEEAADEVFKDIMESSPGSSPPNLTPKPLRRQVTVAPASGLPHKEEAGKGSALGTPAAAEPVTPTSKAGSGA  861
```

```
logo
            PGGTSKGPAEESRVRRHKHSSESPGRDKGKLSRLKPAPPPPPAASAGKAGGKPSQSPSQEAAGEAVLGAKTKATSLVDAVNSDAAKPSQPGEGLKKPVLPATPKPQSAKP
BAFETINIB   ..............................................................................................................    24
PF08919     ..............................................................................................................     0
PF00017     ..............................................................................................................    76
PF00018     ..............................................................................................................    47
PF07714     ..............................................................................................................   251
ABL1-202    PGGTSKGPAEESRVRRHKHSSESPGRDKGKLSRLKPAPPPPPAASAGKAGGKPSQSPSQEAAGEAVLGAKTKATSLVDAVNSDAAKPSQPGEGLKKPVLPATPKPQSAKP   990
ABL1-203    ..............................................................................................................    64
ABL1-201    PGGTSKGPAEESRVRRHKHSSESPGRDKGKLSRLKPAPPPPPAASAGKAGGKPSQSPSQEAAGEAVLGAKTKATSLVDAVNSDAAKPSQPGEGLKKPVLPATPKPQSAKP   971

logo
            SGTPISPAPVPSTLPSASSALAGDQPSSTAFIPLISTRVSLRKTRQPPERIASGAITKGVVLDSTEALCLAISRNSEQMASHSAVLEAGKNLYTFCVSYVDSIQQMRNKF
BAFETINIB   ..............................................................................................................    24
PF08919     .......................................................ITKGVVLDSTEALCLAISRNSEQMASHSAVLEAGKNLYTFCVSYVDSIQQMRNKF    55
PF00017     ..............................................................................................................    76
PF00018     ..............................................................................................................    47
PF07714     ..............................................................................................................   251
ABL1-202    SGTPISPAPVPSTLPSASSALAGDQPSSTAFIPLISTRVSLRKTRQPPERIASGAITKGVVLDSTEALCLAISRNSEQMASHSAVLEAGKNLYTFCVSYVDSIQQMRNKF  1100
ABL1-203    ..............................................................................................................    64
ABL1-201    SGTPISPAPVPSTLPSASSALAGDQPSSTAFIPLISTRVSLRKTRQPPERIASGAITKGVVLDSTEALCLAISRNSEQMASHSAVLEAGKNLYTFCVSYVDSIQQMRNKF  1081

logo
            AFREAINKLENNLRELQICPATAGSGPAATQDFSKLLSSVKEISDIVQR
BAFETINIB   .................................................    24
PF08919     AFREAINKLENNLRELQICPATAGSGPAATQDFSKLLSSVKEISDIVQR   104
PF00017     .................................................    76
PF00018     .................................................    47
PF07714     .................................................   251
ABL1-202    AFREAINKLENNLRELQICPATAGSGPAATQDFSKLLSSVKEISDIVQR  1149
ABL1-203    .................................................    64
ABL1-201    AFREAINKLENNLRELQICPATAGSGPAATQDFSKLLSSVKEISDIVQR  1130
```

X non conserved

X similar

X ≥ 0% conserved

X ≥ 50% conserved

```
logo        MGQQPGKVLGDQRRPSLPALHFIKGAGKKESSRHGGPHCNVFVEHEALQRPVASDFEPQGLSEAARWNSKENLLAGPSENDPNLFVALYDFVASGDNTLSITKGEKLRVL
BOSUTINIB   ..............................................................................................................     0
PF08919     ..............................................................................................................     0
PF00017     ..............................................................................................................     0
PF00018     .....................................................................................VALYDFVASGDNTLSITKGEKLRVL    25
PF07714     ..............................................................................................................     0
ABL1-202    MGQQPGKVLGDQRRPSLPALHFIKGAGKKESSRHGGPHCNVFVEHEALQRPVASDFEPQGLSEAARWNSKENLLAGPSENDPNLFVALYDFVASGDNTLSITKGEKLRVL   110
ABL1-203    MGQQPGKVLGDQRRPSLPALHFIKGAGKKESSRHGGPHCNVFVE.HALQRPVASDFEPQGLSEAA.............................................    64
ABL1-201    ...............MLEICLKLVGC....KSKKGLSSSSSCYLEEALQRPVASDFEPQGLSEAARWNSKENLLAGPSENDPNLFVALYDFVASGDNTLSITKGEKLRVL    91
```

```
logo        GYNHNGEWCEAQTKNGQGWVPSNYITPVNSLEKHSWYHGPVSRNAAEYLLSSGINGSFLVRESESSPGQRSISLRYEGRVYHYRINTASDGKLYVSSESRFNTLAELVHH
BOSUTINIB   ..............................................................................................................     0
PF08919     ..............................................................................................................     0
PF00017     ...................................WYHGPVSRNAAEYLLSSGINGSFLVRESESSPGQRSISLRYEGRVYHYRINTASDGKLYVSSESRFNTLAELVHH    75
PF00018     GYNHNGEWCEAQTKNGQGWVPS........................................................................................    47
PF07714     ..............................................................................................................     0
ABL1-202    GYNHNGEWCEAQTKNGQGWVPSNYITPVNSLEKHSWYHGPVSRNAAEYLLSSGINGSFLVRESESSPGQRSISLRYEGRVYHYRINTASDGKLYVSSESRFNTLAELVHH   220
ABL1-203    ..............................................................................................................    64
ABL1-201    GYNHNGEWCEAQTKNGQGWVPSNYITPVNSLEKHSWYHGPVSRNAAEYLLSSGINGSFLVRESESSPGQRSISLRYEGRVYHYRINTASDGKLYVSSESRFNTLAELVHH   201
```

```
logo        HSTVADGLITTLHYPAPKRNKPTVYGVSPNYDKWEMERTDITMKHKLGGGQYGEVYEGVWKKYSLTVAVKTLKEDTMEVEEFLKEAAVMKEIKHPNLVQLLGVCTREPPF
BOSUTINIB   ..............................................L.......V............A.K..............E...M........V............     7
PF08919     ..............................................................................................................     0
PF00017     H.............................................................................................................    76
PF00018     ..............................................................................................................    47
PF07714     ........................................ITMKHKLGGGQYGEVYEGVWKKYSLTVAVKTLKEDTMEVEEFLKEAAVMKEIKHPNLVQLLGVCTREPPF    70
ABL1-202    HSTVADGLITTLHYPAPKRNKPTVYGVSPNYDKWEMERTDITMKHKLGGGQYGEVYEGVWKKYSLTVAVKTLKEDTMEVEEFLKEAAVMKEIKHPNLVQLLGVCTREPPF   330
ABL1-203    ..............................................................................................................    64
ABL1-201    HSTVADGLITTLHYPAPKRNKPTVYGVSPNYDKWEMERTDITMKHKLGGGQYGEVYEGVWKKYSLTVAVKTLKEDTMEVEEFLKEAAVMKEIKHPNLVQLLGVCTREPPF   311
```

```
logo        YIITEFMTYGNLLDYLRECNRQEVNAVVLLYMATQISSAMEYLEKKNFIHRDLAARNCLVGENHLVKVADFGLSRLMTGDTYTAHAGAKFPIKWTAPESLAYNKFSIKSD
BOSUTINIB   .I.TEFMTYG................................................L...........F.......................................    17
PF08919     ..............................................................................................................     0
PF00017     ..............................................................................................................    76
PF00018     ..............................................................................................................    47
PF07714     YIITEFMTYGNLLDYLRECNRQEVNAVVLLYMATQISSAMEYLEKKNFIHRDLAARNCLVGENHLVKVADFGLSRLMTGDTYTAHAGAKFPIKWTAPESLAYNKFSIKSD   180
ABL1-202    YIITEFMTYGNLLDYLRECNRQEVNAVVLLYMATQISSAMEYLEKKNFIHRDLAARNCLVGENHLVKVADFGLSRLMTGDTYTAHAGAKFPIKWTAPESLAYNKFSIKSD   440
ABL1-203    ..............................................................................................................    64
ABL1-201    YIITEFMTYGNLLDYLRECNRQEVNAVVLLYMATQISSAMEYLEKKNFIHRDLAARNCLVGENHLVKVADFGLSRLMTGDTYTAHAGAKFPIKWTAPESLAYNKFSIKSD   421
```

```
logo
            VWAFGVLLWEIATYGMSPYPGIDLSQVYELLEKDYRMERPEGCPEKVYELMRACWQWNPSDRPSFAEIHQAFETMFQESSISDEVEKELGKQGVRGAVSTLLQAPELPTK
BOSUTINIB   ..............................................................................................................  17
PF08919     ..............................................................................................................   0
PF00017     ..............................................................................................................  76
PF00018     ..............................................................................................................  47
PF07714     VWAFGVLLWEIATYGMSPYPGIDLSQVYELLEKDYRMERPEGCPEKVYELMRACWQWNPSDRPSFAEIHQA....................................... 251
ABL1-202    VWAFGVLLWEIATYGMSPYPGIDLSQVYELLEKDYRMERPEGCPEKVYELMRACWQWNPSDRPSFAEIHQAFETMFQESSISDEVEKELGKQGVRGAVSTLLQAPELPTK 550
ABL1-203    ..............................................................................................................  64
ABL1-201    VWAFGVLLWEIATYGMSPYPGIDLSQVYELLEKDYRMERPEGCPEKVYELMRACWQWNPSDRPSFAEIHQAFETMFQESSISDEVEKELGKQGVRGAVSTLLQAPELPTK 531

logo
            TRTSRRAAEHRDTTDVPEMPHSKGQGESDPLDHEPAVSPLLPRKERGPPEGGLNEDERLLPKDKKTNLFSALIKKKKKTAPTPPKRSSSFREMDGQPERRGAGEEEGRDI
BOSUTINIB   ..............................................................................................................  17
PF08919     ..............................................................................................................   0
PF00017     ..............................................................................................................  76
PF00018     ..............................................................................................................  47
PF07714     .............................................................................................................. 251
ABL1-202    TRTSRRAAEHRDTTDVPEMPHSKGQGESDPLDHEPAVSPLLPRKERGPPEGGLNEDERLLPKDKKTNLFSALIKKKKKTAPTPPKRSSSFREMDGQPERRGAGEEEGRDI 660
ABL1-203    ..............................................................................................................  64
ABL1-201    TRTSRRAAEHRDTTDVPEMPHSKGQGESDPLDHEPAVSPLLPRKERGPPEGGLNEDERLLPKDKKTNLFSALIKKKKKTAPTPPKRSSSFREMDGQPERRGAGEEEGRDI 641

logo
            SNGALAFTPLDTADPAKSPKPSNGAGVPNGALRESGGSGFRSPHLWKKSSTLTSSRLATGEEEGGGSSSKRFLRSCSASCVPHGAKDTEWRSVTLPRDLQSTGRQFDSST
BOSUTINIB   ..............................................................................................................  17
PF08919     ..............................................................................................................   0
PF00017     ..............................................................................................................  76
PF00018     ..............................................................................................................  47
PF07714     .............................................................................................................. 251
ABL1-202    SNGALAFTPLDTADPAKSPKPSNGAGVPNGALRESGGSGFRSPHLWKKSSTLTSSRLATGEEEGGGSSSKRFLRSCSASCVPHGAKDTEWRSVTLPRDLQSTGRQFDSST 770
ABL1-203    ..............................................................................................................  64
ABL1-201    SNGALAFTPLDTADPAKSPKPSNGAGVPNGALRESGGSGFRSPHLWKKSSTLTSSRLATGEEEGGGSSSKRFLRSCSASCVPHGAKDTEWRSVTLPRDLQSTGRQFDSST 751

logo
            FGGHKSEKPALPRKRAGENRSDQVTRGTVTPPPRLVKKNEEAADEVFKDIMESSPGSSPPNLTPKPLRRQVTVAPASGLPHKEEAGKGSALGTPAAAEPVTPTSKAGSGA
BOSUTINIB   ..............................................................................................................  17
PF08919     ..............................................................................................................   0
PF00017     ..............................................................................................................  76
PF00018     ..............................................................................................................  47
PF07714     .............................................................................................................. 251
ABL1-202    FGGHKSEKPALPRKRAGENRSDQVTRGTVTPPPRLVKKNEEAADEVFKDIMESSPGSSPPNLTPKPLRRQVTVAPASGLPHKEEAGKGSALGTPAAAEPVTPTSKAGSGA 880
ABL1-203    ..............................................................................................................  64
ABL1-201    FGGHKSEKPALPRKRAGENRSDQVTRGTVTPPPRLVKKNEEAADEVFKDIMESSPGSSPPNLTPKPLRRQVTVAPASGLPHKEEAGKGSALGTPAAAEPVTPTSKAGSGA 861
```

```
logo
           PGGTSKGPAEESRVRRHKHSSESPGRDKGKLSRLKPAPPPPPAASAGKAGGKPSQSPSQEAAGEAVLGAKTKATSLVDAVNSDAAKPSQPGEGLKKPVLPATPKPQSAKP
BOSUTINIB  ..............................................................................................................    17
PF08919    ..............................................................................................................     0
PF00017    ..............................................................................................................    76
PF00018    ..............................................................................................................    47
PF07714    ..............................................................................................................   251
ABL1-202   PGGTSKGPAEESRVRRHKHSSESPGRDKGKLSRLKPAPPPPPAASAGKAGGKPSQSPSQEAAGEAVLGAKTKATSLVDAVNSDAAKPSQPGEGLKKPVLPATPKPQSAKP   990
ABL1-203   ..............................................................................................................    64
ABL1-201   PGGTSKGPAEESRVRRHKHSSESPGRDKGKLSRLKPAPPPPPAASAGKAGGKPSQSPSQEAAGEAVLGAKTKATSLVDAVNSDAAKPSQPGEGLKKPVLPATPKPQSAKP   971

logo
           SGTPISPAPVPSTLPSASSALAGDQPSSTAFIPLISTRVSLRKTRQPPERIASGAITKGVVLDSTEALCLAISRNSEQMASHSAVLEAGKNLYTFCVSYVDSIQQMRNKF
BOSUTINIB  ..............................................................................................................    17
PF08919    .......................................................ITKGVVLDSTEALCLAISRNSEQMASHSAVLEAGKNLYTFCVSYVDSIQQMRNKF    55
PF00017    ..............................................................................................................    76
PF00018    ..............................................................................................................    47
PF07714    ..............................................................................................................   251
ABL1-202   SGTPISPAPVPSTLPSASSALAGDQPSSTAFIPLISTRVSLRKTRQPPERIASGAITKGVVLDSTEALCLAISRNSEQMASHSAVLEAGKNLYTFCVSYVDSIQQMRNKF  1100
ABL1-203   ..............................................................................................................    64
ABL1-201   SGTPISPAPVPSTLPSASSALAGDQPSSTAFIPLISTRVSLRKTRQPPERIASGAITKGVVLDSTEALCLAISRNSEQMASHSAVLEAGKNLYTFCVSYVDSIQQMRNKF  1081

logo
           AFREAINKLENNLRELQICPATAGSGPAATQDFSKLLSSVKEISDIVQR
BOSUTINIB  .................................................    17
PF08919    AFREAINKLENNLRELQICPATAGSGPAATQDFSKLLSSVKEISDIVQR   104
PF00017    .................................................    76
PF00018    .................................................    47
PF07714    .................................................   251
ABL1-202   AFREAINKLENNLRELQICPATAGSGPAATQDFSKLLSSVKEISDIVQR  1149
ABL1-203   .................................................    64
ABL1-201   AFREAINKLENNLRELQICPATAGSGPAATQDFSKLLSSVKEISDIVQR  1130
```

- X non conserved
- X similar
- X $\geq$0% conserved
- X $\geq$50% conserved

```
logo
DASATINIB  ..............................................................................................................   0
PF08919    ..............................................................................................................   0
PF00017    ..............................................................................................................   0
PF00018    .....................................................................................VALYDFVASGDNTLSITKGEKLRVL  25
PF07714    ..............................................................................................................   0
ABL1-202   MGQQPGKVLGDQRRPSLPALHFIKGAGKKESSRHGGPHCNVFVEHEALQRPVASDFEPQGLSEAARWNSKENLLAGPSENDPNLFVALYDFVASGDNTLSITKGEKLRVL 110
ABL1-203   MGQQPGKVLGDQRRPSLPALHFIKGAGKKESSRHGGPHCNVFVE.HALQRPVASDFEPQGLSEAA.............................................  64
ABL1-201   ...............MLEICLKLVGC....KSKKGLSSSSSCYLEEALQRPVASDFEPQGLSEAARWNSKENLLAGPSENDPNLFVALYDFVASGDNTLSITKGEKLRVL  91

logo
DASATINIB  ..............................................................................................................   0
PF08919    ..............................................................................................................   0
PF00017    ...................................WYHGPVSRNAAEYLLSSGINGSFLVRESESSPGQRSISLRYEGRVYHYRINTASDGKLYVSSESRFNTLAELVHH  75
PF00018    GYNHNGEWCEAQTKNGQGWVPS........................................................................................  47
PF07714    ..............................................................................................................   0
ABL1-202   GYNHNGEWCEAQTKNGQGWVPSNYITPVNSLEKHSWYHGPVSRNAAEYLLSSGINGSFLVRESESSPGQRSISLRYEGRVYHYRINTASDGKLYVSSESRFNTLAELVHH 220
ABL1-203   ..............................................................................................................  64
ABL1-201   GYNHNGEWCEAQTKNGQGWVPSNYITPVNSLEKHSWYHGPVSRNAAEYLLSSGINGSFLVRESESSPGQRSISLRYEGRVYHYRINTASDGKLYVSSESRFNTLAELVHH 201

logo
DASATINIB  ...........L..................................L.AVK..............E.AVK........V.........M...I.TEFVTYG.........  19
PF08919    ..............................................................................................................   0
PF00017    H.............................................................................................................  76
PF00018    ..............................................................................................................  47
PF07714    ........................................ITMKHKLGGGQYGEVYEGVWKKYSLTVAVKTLKEDTMEVEEFLKEAAVMKEIKHPNLVQLLGVCTREPPF  70
ABL1-202   HSTVADGLITTLHYPAPKRNKPTVYGVSPNYDKWEMERTDITMKHKLGGGQYGEVYEGVWKKYSLTVAVKTLKEDTMEVEEFLKEAAVMKEIKHPNLVQLLGVCTREPPF 330
ABL1-203   ..............................................................................................................  64
ABL1-201   HSTVADGLITTLHYPAPKRNKPTVYGVSPNYDKWEMERTDITMKHKLGGGQYGEVYEGVWKKYSLTVAVKTLKEDTMEVEEFLKEAAVMKEIKHPNLVQLLGVCTREPPF 311

logo
DASATINIB  .I.TEFMTYG.............................L.........A........L.........ADF.......................................  33
PF08919    ..............................................................................................................   0
PF00017    ..............................................................................................................  76
PF00018    ..............................................................................................................  47
PF07714    YIITEFMTYGNLLDYLRECNRQEVNAVVLLYMATQISSAMEYLEKKNFIHRDLAARNCLVGENHLVKVADFGLSRLMTGDTYTAHAGAKFPIKWTAPESLAYNKFSIKSD 180
ABL1-202   YIITEFMTYGNLLDYLRECNRQEVNAVVLLYMATQISSAMEYLEKKNFIHRDLAARNCLVGENHLVKVADFGLSRLMTGDTYTAHAGAKFPIKWTAPESLAYNKFSIKSD 440
ABL1-203   ..............................................................................................................  64
ABL1-201   YIITEFMTYGNLLDYLRECNRQEVNAVVLLYMATQISSAMEYLEKKNFIHRDLAARNCLVGENHLVKVADFGLSRLMTGDTYTAHAGAKFPIKWTAPESLAYNKFSIKSD 421
```

```
logo
            VWAFGVLLWEIATYGMSPYPGIDLSQVYELLEKDYRMERPEGCPEKVYELMRACWQWNPSDRPSFAEIHQAFETMFQESSISDEVEKELGKQGVRGAVSTLLQAPELPTK
DASATINIB   ..............................................................................................................  33
PF08919     ..............................................................................................................   0
PF00017     ..............................................................................................................  76
PF00018     ..............................................................................................................  47
PF07714     VWAFGVLLWEIATYGMSPYPGIDLSQVYELLEKDYRMERPEGCPEKVYELMRACWQWNPSDRPSFAEIHQA........................................ 251
ABL1-202    VWAFGVLLWEIATYGMSPYPGIDLSQVYELLEKDYRMERPEGCPEKVYELMRACWQWNPSDRPSFAEIHQAFETMFQESSISDEVEKELGKQGVRGAVSTLLQAPELPTK 550
ABL1-203    ..............................................................................................................  64
ABL1-201    VWAFGVLLWEIATYGMSPYPGIDLSQVYELLEKDYRMERPEGCPEKVYELMRACWQWNPSDRPSFAEIHQAFETMFQESSISDEVEKELGKQGVRGAVSTLLQAPELPTK 531

logo
            TRTSRRAAEHRDTTDVPEMPHSKGQGESDPLDHEPAVSPLLPRKERGPPEGGLNEDERLLPKDKKTNLFSALIKKKKKTAPTPPKRSSSFREMDGQPERRGAGEEEGRDI
DASATINIB   ..............................................................................................................  33
PF08919     ..............................................................................................................   0
PF00017     ..............................................................................................................  76
PF00018     ..............................................................................................................  47
PF07714     .............................................................................................................. 251
ABL1-202    TRTSRRAAEHRDTTDVPEMPHSKGQGESDPLDHEPAVSPLLPRKERGPPEGGLNEDERLLPKDKKTNLFSALIKKKKKTAPTPPKRSSSFREMDGQPERRGAGEEEGRDI 660
ABL1-203    ..............................................................................................................  64
ABL1-201    TRTSRRAAEHRDTTDVPEMPHSKGQGESDPLDHEPAVSPLLPRKERGPPEGGLNEDERLLPKDKKTNLFSALIKKKKKTAPTPPKRSSSFREMDGQPERRGAGEEEGRDI 641

logo
            SNGALAFTPLDTADPAKSPKPSNGAGVPNGALRESGGSGFRSPHLWKKSSTLTSSRLATGEEEGGGSSSKRFLRSCSASCVPHGAKDTEWRSVTLPRDLQSTGRQFDSST
DASATINIB   ..............................................................................................................  33
PF08919     ..............................................................................................................   0
PF00017     ..............................................................................................................  76
PF00018     ..............................................................................................................  47
PF07714     .............................................................................................................. 251
ABL1-202    SNGALAFTPLDTADPAKSPKPSNGAGVPNGALRESGGSGFRSPHLWKKSSTLTSSRLATGEEEGGGSSSKRFLRSCSASCVPHGAKDTEWRSVTLPRDLQSTGRQFDSST 770
ABL1-203    ..............................................................................................................  64
ABL1-201    SNGALAFTPLDTADPAKSPKPSNGAGVPNGALRESGGSGFRSPHLWKKSSTLTSSRLATGEEEGGGSSSKRFLRSCSASCVPHGAKDTEWRSVTLPRDLQSTGRQFDSST 751

logo
            FGGHKSEKPALPRKRAGENRSDQVTRGTVTPPPRLVKKNEEAADEVFKDIMESSPGSSPPNLTPKPLRRQVTVAPASGLPHKEEAGKGSALGTPAAAEPVTPTSKAGSGA
DASATINIB   ..............................................................................................................  33
PF08919     ..............................................................................................................   0
PF00017     ..............................................................................................................  76
PF00018     ..............................................................................................................  47
PF07714     .............................................................................................................. 251
ABL1-202    FGGHKSEKPALPRKRAGENRSDQVTRGTVTPPPRLVKKNEEAADEVFKDIMESSPGSSPPNLTPKPLRRQVTVAPASGLPHKEEAGKGSALGTPAAAEPVTPTSKAGSGA 880
ABL1-203    ..............................................................................................................  64
ABL1-201    FGGHKSEKPALPRKRAGENRSDQVTRGTVTPPPRLVKKNEEAADEVFKDIMESSPGSSPPNLTPKPLRRQVTVAPASGLPHKEEAGKGSALGTPAAAEPVTPTSKAGSGA 861
```

```
logo
            PGGTSKGPAEESRVRRHKHSSESPGRDKGKLSRLKPAPPPPPAASAGKAGGKPSQSPSQEAAGEAVLGAKTKATSLVDAVNSDAAKPSQPGEGLKKPVLPATPKPQSAKP
DASATINIB   ..............................................................................................................    33
PF08919     ..............................................................................................................     0
PF00017     ..............................................................................................................    76
PF00018     ..............................................................................................................    47
PF07714     ..............................................................................................................   251
ABL1-202    PGGTSKGPAEESRVRRHKHSSESPGRDKGKLSRLKPAPPPPPAASAGKAGGKPSQSPSQEAAGEAVLGAKTKATSLVDAVNSDAAKPSQPGEGLKKPVLPATPKPQSAKP   990
ABL1-203    ..............................................................................................................    64
ABL1-201    PGGTSKGPAEESRVRRHKHSSESPGRDKGKLSRLKPAPPPPPAASAGKAGGKPSQSPSQEAAGEAVLGAKTKATSLVDAVNSDAAKPSQPGEGLKKPVLPATPKPQSAKP   971

logo
            SGTPISPAPVPSTLPSASSALAGDQPSSTAFIPLISTRVSLRKTRQPPERIASGAITKGVVLDSTEALCLAISRNSEQMASHSAVLEAGKNLYTFCVSYVDSIQQMRNKF
DASATINIB   ..............................................................................................................    33
PF08919     .......................................................ITKGVVLDSTEALCLAISRNSEQMASHSAVLEAGKNLYTFCVSYVDSIQQMRNKF    55
PF00017     ..............................................................................................................    76
PF00018     ..............................................................................................................    47
PF07714     ..............................................................................................................   251
ABL1-202    SGTPISPAPVPSTLPSASSALAGDQPSSTAFIPLISTRVSLRKTRQPPERIASGAITKGVVLDSTEALCLAISRNSEQMASHSAVLEAGKNLYTFCVSYVDSIQQMRNKF  1100
ABL1-203    ..............................................................................................................    64
ABL1-201    SGTPISPAPVPSTLPSASSALAGDQPSSTAFIPLISTRVSLRKTRQPPERIASGAITKGVVLDSTEALCLAISRNSEQMASHSAVLEAGKNLYTFCVSYVDSIQQMRNKF  1081

logo
            AFREAINKLENNLRELQICPATAGSGPAATQDFSKLLSSVKEISDIVQR
DASATINIB   .................................................    33
PF08919     AFREAINKLENNLRELQICPATAGSGPAATQDFSKLLSSVKEISDIVQR   104
PF00017     .................................................    76
PF00018     .................................................    47
PF07714     .................................................   251
ABL1-202    AFREAINKLENNLRELQICPATAGSGPAATQDFSKLLSSVKEISDIVQR  1149
ABL1-203    .................................................    64
ABL1-201    AFREAINKLENNLRELQICPATAGSGPAATQDFSKLLSSVKEISDIVQR  1130
```

X non conserved

X similar

X $\geq$ 0% conserved

X $\geq$ 50% conserved

```
logo
IMATINIB  ...................................................................................................................    0
PF08919   ...................................................................................................................    0
PF00017   ...................................................................................................................    0
PF00018   .....................................................................................VALYDFVASGDNTLSITKGEKLRVLGYNHN   30
PF07714   ...................................................................................................................    0
ABL1-202  MGQQPGKVLGDQRRPSLPALHFIKGAGKKESSRHGGPHCNVFVEHEALQRPVASDFEPQGLSEAARWNSKENLLAGPSENDPNLFVALYDFVASGDNTLSITKGEKLRVLGYNHN  115
ABL1-203  MGQQPGKVLGDQRRPSLPALHFIKGAGKKESSRHGGPHCNVFVE.HALQRPVASDFEPQGLSEAA..................................................   64
ABL1-201  ...............MLEICLKLVGC....KSKKGLSSSSSCYLEEALQRPVASDFEPQGLSEAARWNSKENLLAGPSENDPNLFVALYDFVASGDNTLSITKGEKLRVLGYNHN   96

logo
IMATINIB  ...................................................................................................................    0
PF08919   ...................................................................................................................    0
PF00017   ..............................WYHGPVSRNAAEYLLSSGINGSFLVRESESSPGQRSISLRYEGRVYHYRINTASDGKLYVSSESRFNTLAELVHHH.........   76
PF00018   GEWCEAQTKNGQGWVPS..................................................................................................   47
PF07714   ...................................................................................................................    0
ABL1-202  GEWCEAQTKNGQGWVPSNYITPVNSLEKHSWYHGPVSRNAAEYLLSSGINGSFLVRESESSPGQRSISLRYEGRVYHYRINTASDGKLYVSSESRFNTLAELVHHHSTVADGLIT  230
ABL1-203  ...................................................................................................................   64
ABL1-201  GEWCEAQTKNGQGWVPSNYITPVNSLEKHSWYHGPVSRNAAEYLLSSGINGSFLVRESESSPGQRSISLRYEGRVYHYRINTASDGKLYVSSESRFNTLAELVHHHSTVADGLIT  211

logo
IMATINIB  ..............Y..V....................AVKY..V..........E...K..I.....V.....E..VM..II.T.FV.............I.T.FM........   23
PF08919   ...................................................................................................................    0
PF00017   ...................................................................................................................   76
PF00018   ...................................................................................................................   47
PF07714   ..............................ITMKHKLGGGQYGEVYEGVWKKYSLTVAVKTLKEDTMEVEEFLKEAAVMKEIKHPNLVQLLGVCTREPPFYIITEFMTYGNLLDY   85
ABL1-202  TLHYPAPKRNKPTVYGVSPNYDKWEMERTDITMKHKLGGGQYGEVYEGVWKKYSLTVAVKTLKEDTMEVEEFLKEAAVMKEIKHPNLVQLLGVCTREPPFYIITEFMTYGNLLDY  345
ABL1-203  ...................................................................................................................   64
ABL1-201  TLHYPAPKRNKPTVYGVSPNYDKWEMERTDITMKHKLGGGQYGEVYEGVWKKYSLTVAVKTLKEDTMEVEEFLKEAAVMKEIKHPNLVQLLGVCTREPPFYIITEFMTYGNLLDY  326

logo
IMATINIB  .................................IH........L........IHDF......L.........ADF........................................   34
PF08919   ...................................................................................................................    0
PF00017   ...................................................................................................................   76
PF00018   ...................................................................................................................   47
PF07714   LRECNRQEVNAVVLLYMATQISSAMEYLEKKNFIHRDLAARNCLVGENHLVKVADFGLSRLMTGDTYTAHAGAKFPIKWTAPESLAYNKFSIKSDVWAFGVLLWEIATYGMSPYP  200
ABL1-202  LRECNRQEVNAVVLLYMATQISSAMEYLEKKNFIHRDLAARNCLVGENHLVKVADFGLSRLMTGDTYTAHAGAKFPIKWTAPESLAYNKFSIKSDVWAFGVLLWEIATYGMSPYP  460
ABL1-203  ...................................................................................................................   64
ABL1-201  LRECNRQEVNAVVLLYMATQISSAMEYLEKKNFIHRDLAARNCLVGENHLVKVADFGLSRLMTGDTYTAHAGAKFPIKWTAPESLAYNKFSIKSDVWAFGVLLWEIATYGMSPYP  441
```

```
logo
          GIDLSQVYELLEKDYRMERPEGCPEKVYELMRACWQWNPSDRPSFAEIHQAFETMFQESSISDEVEKELGKQGVRGAVSTLLQAPELPTKTRTSRRAAEHRDTTDVPEMPHSKGQ
IMATINIB  ...................................................................................................................  34
PF08919   ...................................................................................................................  0
PF00017   ...................................................................................................................  76
PF00018   ...................................................................................................................  47
PF07714   GIDLSQVYELLEKDYRMERPEGCPEKVYELMRACWQWNPSDRPSFAEIHQA................................................................  251
ABL1-202  GIDLSQVYELLEKDYRMERPEGCPEKVYELMRACWQWNPSDRPSFAEIHQAFETMFQESSISDEVEKELGKQGVRGAVSTLLQAPELPTKTRTSRRAAEHRDTTDVPEMPHSKGQ  575
ABL1-203  ...................................................................................................................  64
ABL1-201  GIDLSQVYELLEKDYRMERPEGCPEKVYELMRACWQWNPSDRPSFAEIHQAFETMFQESSISDEVEKELGKQGVRGAVSTLLQAPELPTKTRTSRRAAEHRDTTDVPEMPHSKGQ  556

logo
          GESDPLDHEPAVSPLLPRKERGPPEGGLNEDERLLPKDKKTNLFSALIKKKKKTAPTPPKRSSSFREMDGQPERRGAGEEEGRDISNGALAFTPLDTADPAKSPKPSNGAGVPNG
IMATINIB  ...................................................................................................................  34
PF08919   ...................................................................................................................  0
PF00017   ...................................................................................................................  76
PF00018   ...................................................................................................................  47
PF07714   ...................................................................................................................  251
ABL1-202  GESDPLDHEPAVSPLLPRKERGPPEGGLNEDERLLPKDKKTNLFSALIKKKKKTAPTPPKRSSSFREMDGQPERRGAGEEEGRDISNGALAFTPLDTADPAKSPKPSNGAGVPNG  690
ABL1-203  ...................................................................................................................  64
ABL1-201  GESDPLDHEPAVSPLLPRKERGPPEGGLNEDERLLPKDKKTNLFSALIKKKKKTAPTPPKRSSSFREMDGQPERRGAGEEEGRDISNGALAFTPLDTADPAKSPKPSNGAGVPNG  671

logo
          ALRESGGSGFRSPHLWKKSSTLTSSRLATGEEEGGGSSSKRFLRSCSASCVPHGAKDTEWRSVTLPRDLQSTGRQFDSSTFGGHKSEKPALPRKRAGENRSDQVTRGTVTPPPRL
IMATINIB  ...................................................................................................................  34
PF08919   ...................................................................................................................  0
PF00017   ...................................................................................................................  76
PF00018   ...................................................................................................................  47
PF07714   ...................................................................................................................  251
ABL1-202  ALRESGGSGFRSPHLWKKSSTLTSSRLATGEEEGGGSSSKRFLRSCSASCVPHGAKDTEWRSVTLPRDLQSTGRQFDSSTFGGHKSEKPALPRKRAGENRSDQVTRGTVTPPPRL  805
ABL1-203  ...................................................................................................................  64
ABL1-201  ALRESGGSGFRSPHLWKKSSTLTSSRLATGEEEGGGSSSKRFLRSCSASCVPHGAKDTEWRSVTLPRDLQSTGRQFDSSTFGGHKSEKPALPRKRAGENRSDQVTRGTVTPPPRL  786

logo
          VKKNEEAADEVFKDIMESSPGSSPPNLTPKPLRRQVTVAPASGLPHKEEAGKGSALGTPAAAEPVTPTSKAGSGAPGGTSKGPAEESRVRRHKHSSESPGRDKGKLSRLKPAPPP
IMATINIB  ...................................................................................................................  34
PF08919   ...................................................................................................................  0
PF00017   ...................................................................................................................  76
PF00018   ...................................................................................................................  47
PF07714   ...................................................................................................................  251
ABL1-202  VKKNEEAADEVFKDIMESSPGSSPPNLTPKPLRRQVTVAPASGLPHKEEAGKGSALGTPAAAEPVTPTSKAGSGAPGGTSKGPAEESRVRRHKHSSESPGRDKGKLSRLKPAPPP  920
ABL1-203  ...................................................................................................................  64
ABL1-201  VKKNEEAADEVFKDIMESSPGSSPPNLTPKPLRRQVTVAPASGLPHKEEAGKGSALGTPAAAEPVTPTSKAGSGAPGGTSKGPAEESRVRRHKHSSESPGRDKGKLSRLKPAPPP  901
```

```
logo
             PPAASAGKAGGKPSQSPSQEAAGEAVLGAKTKATSLVDAVNSDAAKPSQPGEGLKKPVLPATPKPQSAKPSGTPISPAPVPSTLPSASSALAGDQPSSTAFIPLISTRVSLRKTR
IMATINIB     ...................................................................................................................    34
PF08919      ...................................................................................................................     0
PF00017      ...................................................................................................................    76
PF00018      ...................................................................................................................    47
PF07714      ...................................................................................................................   251
ABL1-202     PPAASAGKAGGKPSQSPSQEAAGEAVLGAKTKATSLVDAVNSDAAKPSQPGEGLKKPVLPATPKPQSAKPSGTPISPAPVPSTLPSASSALAGDQPSSTAFIPLISTRVSLRKTR  1035
ABL1-203     ...................................................................................................................    64
ABL1-201     PPAASAGKAGGKPSQSPSQEAAGEAVLGAKTKATSLVDAVNSDAAKPSQPGEGLKKPVLPATPKPQSAKPSGTPISPAPVPSTLPSASSALAGDQPSSTAFIPLISTRVSLRKTR  1016

logo
             QPPERIASGAITKGVVLDSTEALCLAISRNSEQMASHSAVLEAGKNLYTFCVSYVDSIQQMRNKFAFREAINKLENNLRELQICPATAGSGPAATQDFSKLLSSVKEISDIVQR
IMATINIB     ..................................................................................................................    34
PF08919      ..........ITKGVVLDSTEALCLAISRNSEQMASHSAVLEAGKNLYTFCVSYVDSIQQMRNKFAFREAINKLENNLRELQICPATAGSGPAATQDFSKLLSSVKEISDIVQR   104
PF00017      ..................................................................................................................    76
PF00018      ..................................................................................................................    47
PF07714      ..................................................................................................................   251
ABL1-202     QPPERIASGAITKGVVLDSTEALCLAISRNSEQMASHSAVLEAGKNLYTFCVSYVDSIQQMRNKFAFREAINKLENNLRELQICPATAGSGPAATQDFSKLLSSVKEISDIVQR  1149
ABL1-203     ..................................................................................................................    64
ABL1-201     QPPERIASGAITKGVVLDSTEALCLAISRNSEQMASHSAVLEAGKNLYTFCVSYVDSIQQMRNKFAFREAINKLENNLRELQICPATAGSGPAATQDFSKLLSSVKEISDIVQR  1130
```

X non conserved
X similar
X ≥ 0% conserved
X ≥ 50% conserved

```
logo
NILOTINIB   ..............................................................................................................   0
PF08919     ..............................................................................................................   0
PF00017     ..............................................................................................................   0
PF00018     .....................................................................................VALYDFVASGDNTLSITKGEKLRVL   25
PF07714     ..............................................................................................................   0
ABL1-202    MGQQPGKVLGDQRRPSLPALHFIKGAGKKESSRHGGPHCNVFVEHEALQRPVASDFEPQGLSEAARWNSKENLLAGPSENDPNLFVALYDFVASGDNTLSITKGEKLRVL   110
ABL1-203    MGQQPGKVLGDQRRPSLPALHFIKGAGKKESSRHGGPHCNVFVE.HALQRPVASDFEPQGLSEAA.............................................   64
ABL1-201    ...............MLEICLKLVGC....KSKKGLSSSSSCYLEEALQRPVASDFEPQGLSEAARWNSKENLLAGPSENDPNLFVALYDFVASGDNTLSITKGEKLRVL   91

logo
NILOTINIB   ..............................................................................................................   0
PF08919     ..............................................................................................................   0
PF00017     ...................................WYHGPVSRNAAEYLLSSGINGSFLVRESESSPGQRSISLRYEGRVYHYRINTASDGKLYVSSESRFNTLAELVHH   75
PF00018     GYNHNGEWCEAQTKNGQGWVPS........................................................................................   47
PF07714     ..............................................................................................................   0
ABL1-202    GYNHNGEWCEAQTKNGQGWVPSNYITPVNSLEKHSWYHGPVSRNAAEYLLSSGINGSFLVRESESSPGQRSISLRYEGRVYHYRINTASDGKLYVSSESRFNTLAELVHH   220
ABL1-203    ..............................................................................................................   64
ABL1-201    GYNHNGEWCEAQTKNGQGWVPSNYITPVNSLEKHSWYHGPVSRNAAEYLLSSGINGSFLVRESESSPGQRSISLRYEGRVYHYRINTASDGKLYVSSESRFNTLAELVHH   201

logo
NILOTINIB   ........L....Y..V.............................L.A.KY..V.........KE.AVK..I....LV.....E..VM..II.T.LV..G.........   25
PF08919     ..............................................................................................................   0
PF00017     H.............................................................................................................   76
PF00018     ..............................................................................................................   47
PF07714     ........................................ITMKHKLGGGQYGEVYEGVWKKYSLTVAVKTLKEDTMEVEEFLKEAAVMKEIKHPNLVQLLGVCTREPPF   70
ABL1-202    HSTVADGLITTLHYPAPKRNKPTVYGVSPNYDKWEMERTDITMKHKLGGGQYGEVYEGVWKKYSLTVAVKTLKEDTMEVEEFLKEAAVMKEIKHPNLVQLLGVCTREPPF   330
ABL1-203    ..............................................................................................................   64
ABL1-201    HSTVADGLITTLHYPAPKRNKPTVYGVSPNYDKWEMERTDITMKHKLGGGQYGEVYEGVWKKYSLTVAVKTLKEDTMEVEEFLKEAAVMKEIKHPNLVQLLGVCTREPPF   311

logo
NILOTINIB   .I.I.FM..G..................F.H........L.......F.HDF......L.........ADF.......................................   41
PF08919     ..............................................................................................................   0
PF00017     ..............................................................................................................   76
PF00018     ..............................................................................................................   47
PF07714     YIITEFMTYGNLLDYLRECNRQEVNAVVLLYMATQISSAMEYLEKKNFIHRDLAARNCLVGENHLVKVADFGLSRLMTGDTYTAHAGAKFPIKWTAPESLAYNKFSIKSD   180
ABL1-202    YIITEFMTYGNLLDYLRECNRQEVNAVVLLYMATQISSAMEYLEKKNFIHRDLAARNCLVGENHLVKVADFGLSRLMTGDTYTAHAGAKFPIKWTAPESLAYNKFSIKSD   440
ABL1-203    ..............................................................................................................   64
ABL1-201    YIITEFMTYGNLLDYLRECNRQEVNAVVLLYMATQISSAMEYLEKKNFIHRDLAARNCLVGENHLVKVADFGLSRLMTGDTYTAHAGAKFPIKWTAPESLAYNKFSIKSD   421
```

```
logo
            VWAFGVLLWEIATYGMSPYPGIDLSQVYELLEKDYRMERPEGCPEKVYELMRACWQWNPSDRPSFAEIHQAFETMFQESSISDEVEKELGKQGVRGAVSTLLQAPELPTK
NILOTINIB   ..............................................................................................................    41
PF08919     ..............................................................................................................     0
PF00017     ..............................................................................................................    76
PF00018     ..............................................................................................................    47
PF07714     VWAFGVLLWEIATYGMSPYPGIDLSQVYELLEKDYRMERPEGCPEKVYELMRACWQWNPSDRPSFAEIHQA.......................................   251
ABL1-202    VWAFGVLLWEIATYGMSPYPGIDLSQVYELLEKDYRMERPEGCPEKVYELMRACWQWNPSDRPSFAEIHQAFETMFQESSISDEVEKELGKQGVRGAVSTLLQAPELPTK   550
ABL1-203    ..............................................................................................................    64
ABL1-201    VWAFGVLLWEIATYGMSPYPGIDLSQVYELLEKDYRMERPEGCPEKVYELMRACWQWNPSDRPSFAEIHQAFETMFQESSISDEVEKELGKQGVRGAVSTLLQAPELPTK   531

logo
            TRTSRRAAEHRDTTDVPEMPHSKGQGESDPLDHEPAVSPLLPRKERGPPEGGLNEDERLLPKDKKTNLFSALIKKKKKTAPTPPKRSSSFREMDGQPERRGAGEEEGRDI
NILOTINIB   ..............................................................................................................    41
PF08919     ..............................................................................................................     0
PF00017     ..............................................................................................................    76
PF00018     ..............................................................................................................    47
PF07714     ..............................................................................................................   251
ABL1-202    TRTSRRAAEHRDTTDVPEMPHSKGQGESDPLDHEPAVSPLLPRKERGPPEGGLNEDERLLPKDKKTNLFSALIKKKKKTAPTPPKRSSSFREMDGQPERRGAGEEEGRDI   660
ABL1-203    ..............................................................................................................    64
ABL1-201    TRTSRRAAEHRDTTDVPEMPHSKGQGESDPLDHEPAVSPLLPRKERGPPEGGLNEDERLLPKDKKTNLFSALIKKKKKTAPTPPKRSSSFREMDGQPERRGAGEEEGRDI   641

logo
            SNGALAFTPLDTADPAKSPKPSNGAGVPNGALRESGGSGFRSPHLWKKSSTLTSSRLATGEEEGGGSSSKRFLRSCSASCVPHGAKDTEWRSVTLPRDLQSTGRQFDSST
NILOTINIB   ..............................................................................................................    41
PF08919     ..............................................................................................................     0
PF00017     ..............................................................................................................    76
PF00018     ..............................................................................................................    47
PF07714     ..............................................................................................................   251
ABL1-202    SNGALAFTPLDTADPAKSPKPSNGAGVPNGALRESGGSGFRSPHLWKKSSTLTSSRLATGEEEGGGSSSKRFLRSCSASCVPHGAKDTEWRSVTLPRDLQSTGRQFDSST   770
ABL1-203    ..............................................................................................................    64
ABL1-201    SNGALAFTPLDTADPAKSPKPSNGAGVPNGALRESGGSGFRSPHLWKKSSTLTSSRLATGEEEGGGSSSKRFLRSCSASCVPHGAKDTEWRSVTLPRDLQSTGRQFDSST   751

logo
            FGGHKSEKPALPRKRAGENRSDQVTRGTVTPPPRLVKKNEEAADEVFKDIMESSPGSSPPNLTPKPLRRQVTVAPASGLPHKEEAGKGSALGTPAAAEPVTPTSKAGSGA
NILOTINIB   ..............................................................................................................    41
PF08919     ..............................................................................................................     0
PF00017     ..............................................................................................................    76
PF00018     ..............................................................................................................    47
PF07714     ..............................................................................................................   251
ABL1-202    FGGHKSEKPALPRKRAGENRSDQVTRGTVTPPPRLVKKNEEAADEVFKDIMESSPGSSPPNLTPKPLRRQVTVAPASGLPHKEEAGKGSALGTPAAAEPVTPTSKAGSGA   880
ABL1-203    ..............................................................................................................    64
ABL1-201    FGGHKSEKPALPRKRAGENRSDQVTRGTVTPPPRLVKKNEEAADEVFKDIMESSPGSSPPNLTPKPLRRQVTVAPASGLPHKEEAGKGSALGTPAAAEPVTPTSKAGSGA   861
```

```
logo
            PGGTSKGPAEESRVRRHKHSSESPGRDKGKLSRLKPAPPPPPAASAGKAGGKPSQSPSQEAAGEAVLGAKTKATSLVDAVNSDAAKPSQPGEGLKKPVLPATPKPQSAKP
NILOTINIB   ..............................................................................................................    41
PF08919     ..............................................................................................................     0
PF00017     ..............................................................................................................    76
PF00018     ..............................................................................................................    47
PF07714     ..............................................................................................................   251
ABL1-202    PGGTSKGPAEESRVRRHKHSSESPGRDKGKLSRLKPAPPPPPAASAGKAGGKPSQSPSQEAAGEAVLGAKTKATSLVDAVNSDAAKPSQPGEGLKKPVLPATPKPQSAKP   990
ABL1-203    ..............................................................................................................    64
ABL1-201    PGGTSKGPAEESRVRRHKHSSESPGRDKGKLSRLKPAPPPPPAASAGKAGGKPSQSPSQEAAGEAVLGAKTKATSLVDAVNSDAAKPSQPGEGLKKPVLPATPKPQSAKP   971

logo
            SGTPISPAPVPSTLPSASSALAGDQPSSTAFIPLISTRVSLRKTRQPPERIASGAITKGVVLDSTEALCLAISRNSEQMASHSAVLEAGKNLYTFCVSYVDSIQQMRNKF
NILOTINIB   ..............................................................................................................    41
PF08919     .......................................................ITKGVVLDSTEALCLAISRNSEQMASHSAVLEAGKNLYTFCVSYVDSIQQMRNKF    55
PF00017     ..............................................................................................................    76
PF00018     ..............................................................................................................    47
PF07714     ..............................................................................................................   251
ABL1-202    SGTPISPAPVPSTLPSASSALAGDQPSSTAFIPLISTRVSLRKTRQPPERIASGAITKGVVLDSTEALCLAISRNSEQMASHSAVLEAGKNLYTFCVSYVDSIQQMRNKF  1100
ABL1-203    ..............................................................................................................    64
ABL1-201    SGTPISPAPVPSTLPSASSALAGDQPSSTAFIPLISTRVSLRKTRQPPERIASGAITKGVVLDSTEALCLAISRNSEQMASHSAVLEAGKNLYTFCVSYVDSIQQMRNKF  1081

logo
            AFREAINKLENNLRELQICPATAGSGPAATQDFSKLLSSVKEISDIVQR
NILOTINIB   .................................................    41
PF08919     AFREAINKLENNLRELQICPATAGSGPAATQDFSKLLSSVKEISDIVQR   104
PF00017     .................................................    76
PF00018     .................................................    47
PF07714     .................................................   251
ABL1-202    AFREAINKLENNLRELQICPATAGSGPAATQDFSKLLSSVKEISDIVQR  1149
ABL1-203    .................................................    64
ABL1-201    AFREAINKLENNLRELQICPATAGSGPAATQDFSKLLSSVKEISDIVQR  1130
```

- X non conserved
- X similar
- X $\geq 0\%$ conserved
- X $\geq 50\%$ conserved

logo

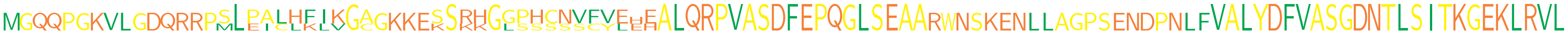

```
REBASTINIB  ..............................................................................................................   0
PF08919     ..............................................................................................................   0
PF00017     ..............................................................................................................   0
PF00018     .....................................................................................VALYDFVASGDNTLSITKGEKLRVL  25
PF07714     ..............................................................................................................   0
ABL1-202    MGQQPGKVLGDQRRPSLPALHFIKGAGKKESSRHGGPHCNVFVEHEALQRPVASDFEPQGLSEAARWNSKENLLAGPSENDPNLFVALYDFVASGDNTLSITKGEKLRVL 110
ABL1-203    MGQQPGKVLGDQRRPSLPALHFIKGAGKKESSRHGGPHCNVFVE.HALQRPVASDFEPQGLSEAA.............................................  64
ABL1-201    ...............MLEICLKLVGC....KSKKGLSSSSSCYLEEALQRPVASDFEPQGLSEAARWNSKENLLAGPSENDPNLFVALYDFVASGDNTLSITKGEKLRVL  91
```

logo

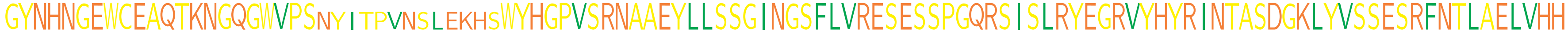

```
REBASTINIB  ..............................................................................................................   0
PF08919     ..............................................................................................................   0
PF00017     ...................................WYHGPVSRNAAEYLLSSGINGSFLVRESESSPGQRSISLRYEGRVYHYRINTASDGKLYVSSESRFNTLAELVHH  75
PF00018     GYNHNGEWCEAQTKNGQGWVPS........................................................................................  47
PF07714     ..............................................................................................................   0
ABL1-202    GYNHNGEWCEAQTKNGQGWVPSNYITPVNSLEKHSWYHGPVSRNAAEYLLSSGINGSFLVRESESSPGQRSISLRYEGRVYHYRINTASDGKLYVSSESRFNTLAELVHH 220
ABL1-203    ..............................................................................................................  64
ABL1-201    GYNHNGEWCEAQTKNGQGWVPSNYITPVNSLEKHSWYHGPVSRNAAEYLLSSGINGSFLVRESESSPGQRSISLRYEGRVYHYRINTASDGKLYVSSESRFNTLAELVHH 201
```

logo

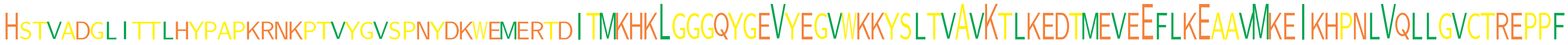

```
REBASTINIB  ..............................................L.......V............A.K..........E...E...M..I.....V............   9
PF08919     ..............................................................................................................   0
PF00017     H.............................................................................................................  76
PF00018     ..............................................................................................................  47
PF07714     ........................................ITMKHKLGGGQYGEVYEGVWKKYSLTVAVKTLKEDTMEVEEFLKEAAVMKEIKHPNLVQLLGVCTREPPF  70
ABL1-202    HSTVADGLITTLHYPAPKRNKPTVYGVSPNYDKWEMERTDITMKHKLGGGQYGEVYEGVWKKYSLTVAVKTLKEDTMEVEEFLKEAAVMKEIKHPNLVQLLGVCTREPPF 330
ABL1-203    ..............................................................................................................  64
ABL1-201    HSTVADGLITTLHYPAPKRNKPTVYGVSPNYDKWEMERTDITMKHKLGGGQYGEVYEGVWKKYSLTVAVKTLKEDTMEVEEFLKEAAVMKEIKHPNLVQLLGVCTREPPF 311
```

logo

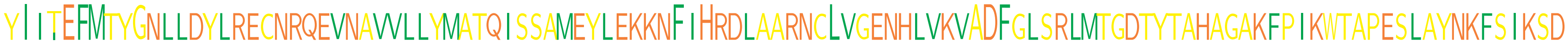

```
REBASTINIB  .I.IEFM..G.....................................F.H........L.........ADF.......................................  21
PF08919     ..............................................................................................................   0
PF00017     ..............................................................................................................  76
PF00018     ..............................................................................................................  47
PF07714     YIITEFMTYGNLLDYLRECNRQEVNAVVLLYMATQISSAMEYLEKKNFIHRDLAARNCLVGENHLVKVADFGLSRLMTGDTYTAHAGAKFPIKWTAPESLAYNKFSIKSD 180
ABL1-202    YIITEFMTYGNLLDYLRECNRQEVNAVVLLYMATQISSAMEYLEKKNFIHRDLAARNCLVGENHLVKVADFGLSRLMTGDTYTAHAGAKFPIKWTAPESLAYNKFSIKSD 440
ABL1-203    ..............................................................................................................  64
ABL1-201    YIITEFMTYGNLLDYLRECNRQEVNAVVLLYMATQISSAMEYLEKKNFIHRDLAARNCLVGENHLVKVADFGLSRLMTGDTYTAHAGAKFPIKWTAPESLAYNKFSIKSD 421
```

```
logo
             VWAFGVLLWEIATYGMSPYPGIDLSQVYELLEKDYRMERPEGCPEKVYELMRACWQWNPSDRPSFAEIHQAFETMFQESSISDEVEKELGKQGVRGAVSTLLQAPELPTK
REBASTINIB   ..............................................................................................................   21
PF08919      ..............................................................................................................    0
PF00017      ..............................................................................................................   76
PF00018      ..............................................................................................................   47
PF07714      VWAFGVLLWEIATYGMSPYPGIDLSQVYELLEKDYRMERPEGCPEKVYELMRACWQWNPSDRPSFAEIHQA.......................................  251
ABL1-202     VWAFGVLLWEIATYGMSPYPGIDLSQVYELLEKDYRMERPEGCPEKVYELMRACWQWNPSDRPSFAEIHQAFETMFQESSISDEVEKELGKQGVRGAVSTLLQAPELPTK  550
ABL1-203     ..............................................................................................................   64
ABL1-201     VWAFGVLLWEIATYGMSPYPGIDLSQVYELLEKDYRMERPEGCPEKVYELMRACWQWNPSDRPSFAEIHQAFETMFQESSISDEVEKELGKQGVRGAVSTLLQAPELPTK  531

logo
             TRTSRRAAEHRDTTDVPEMPHSKGQGESDPLDHEPAVSPLLPRKERGPPEGGLNEDERLLPKDKKTNLFSALIKKKKKTAPTPPKRSSSFREMDGQPERRGAGEEEGRDI
REBASTINIB   ..............................................................................................................   21
PF08919      ..............................................................................................................    0
PF00017      ..............................................................................................................   76
PF00018      ..............................................................................................................   47
PF07714      ..............................................................................................................  251
ABL1-202     TRTSRRAAEHRDTTDVPEMPHSKGQGESDPLDHEPAVSPLLPRKERGPPEGGLNEDERLLPKDKKTNLFSALIKKKKKTAPTPPKRSSSFREMDGQPERRGAGEEEGRDI  660
ABL1-203     ..............................................................................................................   64
ABL1-201     TRTSRRAAEHRDTTDVPEMPHSKGQGESDPLDHEPAVSPLLPRKERGPPEGGLNEDERLLPKDKKTNLFSALIKKKKKTAPTPPKRSSSFREMDGQPERRGAGEEEGRDI  641

logo
             SNGALAFTPLDTADPAKSPKPSNGAGVPNGALRESGGSGFRSPHLWKKSSTLTSSRLATGEEEGGGSSSKRFLRSCSASCVPHGAKDTEWRSVTLPRDLQSTGRQFDSST
REBASTINIB   ..............................................................................................................   21
PF08919      ..............................................................................................................    0
PF00017      ..............................................................................................................   76
PF00018      ..............................................................................................................   47
PF07714      ..............................................................................................................  251
ABL1-202     SNGALAFTPLDTADPAKSPKPSNGAGVPNGALRESGGSGFRSPHLWKKSSTLTSSRLATGEEEGGGSSSKRFLRSCSASCVPHGAKDTEWRSVTLPRDLQSTGRQFDSST  770
ABL1-203     ..............................................................................................................   64
ABL1-201     SNGALAFTPLDTADPAKSPKPSNGAGVPNGALRESGGSGFRSPHLWKKSSTLTSSRLATGEEEGGGSSSKRFLRSCSASCVPHGAKDTEWRSVTLPRDLQSTGRQFDSST  751

logo
             FGGHKSEKPALPRKRAGENRSDQVTRGTVTPPPRLVKKNEEAADEVFKDIMESSPGSSPPNLTPKPLRRQVTVAPASGLPHKEEAGKGSALGTPAAAEPVTPTSKAGSGA
REBASTINIB   ..............................................................................................................   21
PF08919      ..............................................................................................................    0
PF00017      ..............................................................................................................   76
PF00018      ..............................................................................................................   47
PF07714      ..............................................................................................................  251
ABL1-202     FGGHKSEKPALPRKRAGENRSDQVTRGTVTPPPRLVKKNEEAADEVFKDIMESSPGSSPPNLTPKPLRRQVTVAPASGLPHKEEAGKGSALGTPAAAEPVTPTSKAGSGA  880
ABL1-203     ..............................................................................................................   64
ABL1-201     FGGHKSEKPALPRKRAGENRSDQVTRGTVTPPPRLVKKNEEAADEVFKDIMESSPGSSPPNLTPKPLRRQVTVAPASGLPHKEEAGKGSALGTPAAAEPVTPTSKAGSGA  861
```

```
logo
              PGGTSKGPAEESRVRRHKHSSESPGRDKGKLSRLKPAPPPPPAASAGKAGGKPSQSPSQEAAGEAVLGAKTKATSLVDAVNSDAAKPSQPGEGLKKPVLPATPKPQSAKP
REBASTINIB    ..............................................................................................................   21
PF08919       ..............................................................................................................    0
PF00017       ..............................................................................................................   76
PF00018       ..............................................................................................................   47
PF07714       ..............................................................................................................  251
ABL1-202      PGGTSKGPAEESRVRRHKHSSESPGRDKGKLSRLKPAPPPPPAASAGKAGGKPSQSPSQEAAGEAVLGAKTKATSLVDAVNSDAAKPSQPGEGLKKPVLPATPKPQSAKP  990
ABL1-203      ..............................................................................................................   64
ABL1-201      PGGTSKGPAEESRVRRHKHSSESPGRDKGKLSRLKPAPPPPPAASAGKAGGKPSQSPSQEAAGEAVLGAKTKATSLVDAVNSDAAKPSQPGEGLKKPVLPATPKPQSAKP  971
```

```
logo
              SGTPISPAPVPSTLPSASSALAGDQPSSTAFIPLISTRVSLRKTRQPPERIASGAITKGVVLDSTEALCLAISRNSEQMASHSAVLEAGKNLYTFCVSYVDSIQQMRNKF
REBASTINIB    ..............................................................................................................   21
PF08919       .......................................................ITKGVVLDSTEALCLAISRNSEQMASHSAVLEAGKNLYTFCVSYVDSIQQMRNKF   55
PF00017       ..............................................................................................................   76
PF00018       ..............................................................................................................   47
PF07714       ..............................................................................................................  251
ABL1-202      SGTPISPAPVPSTLPSASSALAGDQPSSTAFIPLISTRVSLRKTRQPPERIASGAITKGVVLDSTEALCLAISRNSEQMASHSAVLEAGKNLYTFCVSYVDSIQQMRNKF 1100
ABL1-203      ..............................................................................................................   64
ABL1-201      SGTPISPAPVPSTLPSASSALAGDQPSSTAFIPLISTRVSLRKTRQPPERIASGAITKGVVLDSTEALCLAISRNSEQMASHSAVLEAGKNLYTFCVSYVDSIQQMRNKF 1081
```

```
logo
              AFREAINKLENNLRELQICPATAGSGPAATQDFSKLLSSVKEISDIVQR
REBASTINIB    .................................................   21
PF08919       AFREAINKLENNLRELQICPATAGSGPAATQDFSKLLSSVKEISDIVQR  104
PF00017       .................................................   76
PF00018       .................................................   47
PF07714       .................................................  251
ABL1-202      AFREAINKLENNLRELQICPATAGSGPAATQDFSKLLSSVKEISDIVQR 1149
ABL1-203      .................................................   64
ABL1-201      AFREAINKLENNLRELQICPATAGSGPAATQDFSKLLSSVKEISDIVQR 1130
```

- X non conserved
- X similar
- X ≥ 0% conserved
- X ≥ 50% conserved

logo 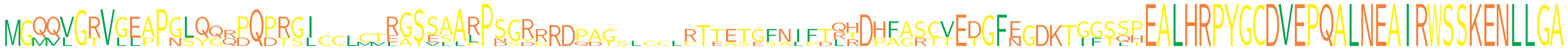

```
IMATINIB  .................................................................................................................    0
PF00017   .................................................................................................................    0
PF08919   .................................................................................................................    0
PF00018   .................................................................................................................    0
PF07714   .................................................................................................................    0
ABL2-203  MGQQVGRVGEAPGLQ.QPQPRGI....RGSSAARPSGR.....................RRDPAGRTTETGFN....IFTQHEALHRPYGCDVEPQALNEAIRWSSKENLLGA   83
ABL2-202  MGQQVGRVGEAPGLQQ.PQPRGI....RGSSAARPSGRRRDPAG....RTTETGFNIFTQH.....................EALHRPYGCDVEPQALNEAIRWSSKENLLGA   83
ABL2-207  MGQQVGRVGEAPGLQ.QPQPRGI....RGSSAARPSGR.....................RRDPAGRTTETGFN....IFTQHEALHRPYGCDVEPQALNEAIRWSSKENLLGA   83
ABL2-211  ..MVLGTVLLPPNSYGRDQDTSLCCLCTEASESALP..D....................LTDHFASCVEDGFEGDKTGGSSPEALHRPYGCDVEPQALNEAIRWSSKENLLGA   89
ABL2-206  ..MVLGTVLLPPNSYGRDQDTSLCCLCTEASESALPD...........................................LTEALHRPYGCDVEPQALNEAIRWSSKENLLGA   68
ABL2-204  MGQQVGRVGEAPGLQQ.PQPRGI....RGSSAARPSGRRRDPAG....RTTETGFNIFTQHDHFASCVEDGFEGDKTGGSSPEALHRPYGCDVEPQALNEAIRWSSKENLLGA  104
ABL2-210  MGQQVGRVGEAPGLQQ.PQPRGI....RGSSAARPSGRRRDPAG....RTTETGFNIFTQHDHFASCVEDGFEGDKTGGSSPEALHRPYGCDVEPQALNEAIRWSSKENLLGA  104
ABL2-201  ........................MVLGTVLLPPNSYGRDQDTSLCCLCTEASESALPDLTDHFASCVEDGFEGDKTGGSSPEALHRPYGCDVEPQALNEAIRWSSKENLLGA   89
```

logo 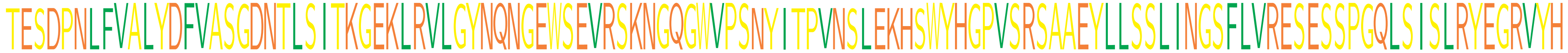

```
IMATINIB  ...................................................................................................................    0
PF00017   ....................................................................WYHGPVSRSAAEYLLSSLINGSFLVRESESSPGQLSISLRYEGRVYH   47
PF08919   ...................................................................................................................    0
PF00018   ........VALYDFVASGDNTLSITKGEKLRVLGYNQNGEWSEVRSKNGQGWVPS............................................................   47
PF07714   ...................................................................................................................    0
ABL2-203  TESDPNLFVALYDFVASGDNTLSITKGEKLRVLGYNQNGEWSEVRSKNGQGWVPSNYITPVNSLEKHSWYHGPVSRSAAEYLLSSLINGSFLVRESESSPGQLSISLRYEGRVYH  198
ABL2-202  TESDPNLFVALYDFVASGDNTLSITKGEKLRVLGYNQNGEWSEVRSKNGQGWVPSNYITPVNSLEKHSWYHGPVSRSAAEYLLSSLINGSFLVRESESSPGQLSISLRYEGRVYH  198
ABL2-207  TESDPNLFVALYDFVASGDNTLSITKGEKLRVLGYNQNGEWSEVRSKNGQGWVPSNYITPVNSLEKHSWYHGPVSRSAAEYLLSSLINGSFLVRESESSPGQLSISLRYEGRVYH  198
ABL2-211  TESDPNLFVALYDFVASGDNTLSITKGEKLRVLGYNQNGEWSEVRSKNGQGWVPSNYITPVNSLEKHSWYHGPVSRSAAEYLLSSLINGSFLVRESESSPGQLSISLRYEGRVYH  204
ABL2-206  TESDPNLFVALYDFVASGDNTLSITKGEKLRVLGYNQNGEWSEVRSKNGQGWVPSNYITPVNSLEKHSWYHGPVSRSAAEYLLSSLINGSFLVRESESSPGQLSISLRYEGRVYH  183
ABL2-204  TESDPNLFVALYDFVASGDNTLSITKGEKLRVLGYNQNGEWSEVRSKNGQGWVPSNYITPVNSLEKHSWYHGPVSRSAAEYLLSSLINGSFLVRESESSPGQLSISLRYEGRVYH  219
ABL2-210  TESDPNLFVALYDFVASGDNTLSITKGEKLRVLGYNQNGEWSEVRSKNGQGWVPSNYITPVNSLEKHSWYHGPVSRSAAEYLLSSLINGSFLVRESESSPGQLSISLRYEGRVYH  219
ABL2-201  TESDPNLFVALYDFVASGDNTLSITKGEKLRVLGYNQNGEWSEVRSKNGQGWVPSNYITPVNSLEKHSWYHGPVSRSAAEYLLSSLINGSFLVRESESSPGQLSISLRYEGRVYH  204
```

logo 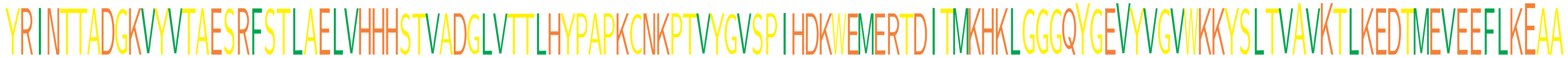

```
IMATINIB  ...............................................................................Y..V............A.K..............E..    5
PF00017   YRINTTADGKVYVTAESRFSTLAELVHHH......................................................................................   76
PF08919   ...................................................................................................................    0
PF00018   ...................................................................................................................   47
PF07714   ....................................................................ITMKHKLGGGQYGEVYVGVWKKYSLTVAVKTLKEDTMEVEEFLKEAA   47
ABL2-203  YRINTTADGKVYVTAESRFSTLAELVHHHSTVADGLVTTLHYPAPKCNKPTVYGVSPIHDKWEMERTDITMKHKLGGGQYGEVYVGVWKKYSLTVAVKTLKEDTMEVEEFLKEAA  313
ABL2-202  YRINTTADGKVYVTAESRFSTLAELVHHHSTVADGLVTTLHYPAPKCNKPTVYGVSPIHDKWEMERTDITMKHKLGGGQYGEVYVGVWKKYSLTVAVKTLKEDTMEVEEFLKEAA  313
ABL2-207  YRINTTADGKVYVTAESRFSTLAELVHHHSTVADGLVTTLHYPAPKCNKPTVYGVSPIHDKWEMERTDITMKHKLGGGQYGEVYVGVWKKYSLTVAVKTLKEDTMEVEEFLKEAA  313
ABL2-211  YRINTTADGKVYVTAESRFSTLAELVHHHSTVADGLVTTLHYPAPKCNKPTVYGVSPIHDKWEMERTDITMKHKLGGGQYGEVYVGVWKKYSLTVAVKTLKEDTMEVEEFLKEAA  319
ABL2-206  YRINTTADGKVYVTAESRFSTLAELVHHHSTVADGLVTTLHYPAPKCNKPTVYGVSPIHDKWEMERTDITMKHKLGGGQYGEVYVGVWKKYSLTVAVKTLKEDTMEVEEFLKEAA  298
ABL2-204  YRINTTADGKVYVTAESRFSTLAELVHHHSTVADGLVTTLHYPAPKCNKPTVYGVSPIHDKWEMERTDITMKHKLGGGQYGEVYVGVWKKYSLTVAVKTLKEDTMEVEEFLKEAA  334
ABL2-210  YRINTTADGKVYVTAESRFSTLAELVHHHSTVADGLVTTLHYPAPKCNKPTVYGVSPIHDKWEMERTDITMKHKLGGGQYGEVYVGVWKKYSLTVAVKTLKEDTMEVEEFLKEAA  334
ABL2-201  YRINTTADGKVYVTAESRFSTLAELVHHHSTVADGLVTTLHYPAPKCNKPTVYGVSPIHDKWEMERTDITMKHKLGGGQYGEVYVGVWKKYSLTVAVKTLKEDTMEVEEFLKEAA  319
```

```
logo      VMKEIKHPNLVQLLGVCTLEPPFYIVTEYMPYGNLLDYLRECNREEVTAVVLLYMATQISSAMEYLEKKNFIHRDLAARNCLVGENHVVKVADFGLSRLMTGDTYTAHAGAKFPI
IMATINIB  .M..I.....V.............I.T.YM..................AV.LL..................IH..................ADF.....................   21
PF00017   ...................................................................................................................   76
PF08919   ...................................................................................................................    0
PF00018   ...................................................................................................................   47
PF07714   VMKEIKHPNLVQLLGVCTLEPPFYIVTEYMPYGNLLDYLRECNREEVTAVVLLYMATQISSAMEYLEKKNFIHRDLAARNCLVGENHVVKVADFGLSRLMTGDTYTAHAGAKFPI  162
ABL2-203  VMKEIKHPNLVQLLGVCTLEPPFYIVTEYMPYGNLLDYLRECNREEVTAVVLLYMATQISSAMEYLEKKNFIHRDLAARNCLVGENHVVKVADFGLSRLMTGDTYTAHAGAKFPI  428
ABL2-202  VMKEIKHPNLVQLLGVCTLEPPFYIVTEYMPYGNLLDYLRECNREEVTAVVLLYMATQISSAMEYLEKKNFIHRDLAARNCLVGENHVVKVADFGLSRLMTGDTYTAHAGAKFPI  428
ABL2-207  VMKEIKHPNLVQLLGVCTLEPPFYIVTEYMPYGNLLDYLRECNREEVTAVVLLYMATQISSAMEYLEKKNFIHRDLAARNCLVGENHVVKVADFGLSRLMTGDTYTAHAGAKFPI  428
ABL2-211  VMKEIKHPNLVQLLGVCTLEPPFYIVTEYMPYGNLLDYLRECNREEVTAVVLLYMATQISSAMEYLEKKNFIHRDLAARNCLVGENHVVKVADFGLSRLMTGDTYTAHAGAKFPI  434
ABL2-206  VMKEIKHPNLVQLLGVCTLEPPFYIVTEYMPYGNLLDYLRECNREEVTAVVLLYMATQISSAMEYLEKKNFIHRDLAARNCLVGENHVVKVADFGLSRLMTGDTYTAHAGAKFPI  413
ABL2-204  VMKEIKHPNLVQLLGVCTLEPPFYIVTEYMPYGNLLDYLRECNREEVTAVVLLYMATQISSAMEYLEKKNFIHRDLAARNCLVGENHVVKVADFGLSRLMTGDTYTAHAGAKFPI  449
ABL2-210  VMKEIKHPNLVQLLGVCTLEPPFYIVTEYMPYGNLLDYLRECNREEVTAVVLLYMATQISSAMEYLEKKNFIHRDLAARNCLVGENHVVKVADFGLSRLMTGDTYTAHAGAKFPI  449
ABL2-201  VMKEIKHPNLVQLLGVCTLEPPFYIVTEYMPYGNLLDYLRECNREEVTAVVLLYMATQISSAMEYLEKKNFIHRDLAARNCLVGENHVVKVADFGLSRLMTGDTYTAHAGAKFPI  434

logo      KWTAPESLAYNTFSIKSDVWAFGVLLWEIATYGMSPYPGIDLSQVYDLLEKGYRMEQPEGCPPKVYELMRACWKWSPADRPSFAETHQAFETMFHDSSISEEVAEELGRAASSSS
IMATINIB  .............................A............................EG.P..V..................................................   26
PF00017   ...................................................................................................................   76
PF08919   ...................................................................................................................    0
PF00018   ...................................................................................................................   47
PF07714   KWTAPESLAYNTFSIKSDVWAFGVLLWEIATYGMSPYPGIDLSQVYDLLEKGYRMEQPEGCPPKVYELMRACWKWSPADRPSFAETHQA..........................  251
ABL2-203  KWTAPESLAYNTFSIKSDVWAFGVLLWEIATYGMSPYPGIDLSQVYDLLEKGYRMEQPEGCPPKVYELMRACWKWSPADRPSFAETHQAFETMFHDSSISEVLLHCANQTCITL.  542
ABL2-202  KWTAPESLAYNTFSIKSDVWAFGVLLWEIATYGMSPYPGIDLSQVYDLLEKGYRMEQPEGCPPKVYELMRACWKWSPADRPSFAETHQAFETMFHDSSISEEVAEELGRAASSSS  543
ABL2-207  KWTAPESLAYNTFSIKSDVWAFGVLLWEIATYGMSPYPGIDLSQVYDLLEKGYRMEQPEGCPPKVYELMRACWKWSPADRPSFAETHQAFETMFHDSSISEEVAEELGRAASSSS  543
ABL2-211  KWTAPESLAYNTFSIKSDVWAFGVLLWEIATYGMSPYPGIDLSQVYDLLEKGYRMEQPEGCPPKVYELMRACWKWSPADRPSFAETHQAFETMFHDSSISEEVAEELGRAASSSS  549
ABL2-206  KWTAPESLAYNTFSIKSDVWAFGVLLWEIATYGMSPYPGIDLSQVYDLLEKGYRMEQPEGCPPKVYELMRACWKWSPADRPSFAETHQAFETMFHDSSISEEVAEELGRAASSSS  528
ABL2-204  KWTAPESLAYNTFSIKSDVWAFGVLLWEIATYGMSPYPGIDLSQVYDLLEKGYRMEQPEGCPPKVYELMRACWKWSPADRPSFAETHQAFETMFHDSSISEEVAEELGRAASSSS  564
ABL2-210  KWTAPESLAYNTFSIKSDVWAFGVLLWEIATYGMSPYPGIDLSQVYDLLEKGYRMEQPEGCPPKVYELMRACWKWSPADRPSFAETHQAFETMFHDSSISEEVAEELGRAASSSS  564
ABL2-201  KWTAPESLAYNTFSIKSDVWAFGVLLWEIATYGMSPYPGIDLSQVYDLLEKGYRMEQPEGCPPKVYELMRACWKWSPADRPSFAETHQAFETMFHDSSISEEVAEELGRAASSSS  549

logo      VVPYLPRLPILPSKTRTLKKQVENKENIEGAQDATENSASSLAPGFIRGAQASSGSPALPRKQRDKSPSSLLEDAKETCFTRDRKGGFFSSFMKKRNAPTPPKRSSSFREMENQP
IMATINIB  ...................................................................................................................   26
PF00017   ...................................................................................................................   76
PF08919   ...................................................................................................................    0
PF00018   ...................................................................................................................   47
PF07714   ...................................................................................................................  251
ABL2-203  ...................................................................................................................  542
ABL2-202  VVPYLPRLPILPSKTRTLKKQVENKENIEGAQDATENSASSLAPGFIRGAQASSGSPALPRKQRDKSPSSLLEDAKETCFTRDRKGGFFSSFMKKRNAPTPPKRSSSFREMENQP  658
ABL2-207  VVPYLPRLPILPSKTRTLKKQVENKENIEGAQDATENSASSLAPGFIRGAQASSGSPALPRKQRDKSPSSLLEDAKETCFTRDRKGGFFSSFMKKRNAPTPPKRSSSFREMENQP  658
ABL2-211  VVPYLPRLPILPSKTRTLKKQVENKENIEGAQDATENSASSLAPGFIRGAQASSGSPALPRKQRDKSPSSLLEDAKETCFTRDRKGGFFSSFMKKRNAPTPPKRSSSFREMENQP  664
ABL2-206  VVPYLPRLPILPSKTRTLKKQVENKENIEGAQDATENSASSLAPGFIRGAQASSGSPALPRKQRDKSPSSLLEDAKETCFTRDRKGGFFSSFMKKRNAPTPPKRSSSFREMENQP  643
ABL2-204  VVPYLPRLPILPSKTRTLKKQVENKENIEGAQDATENSASSLAPGFIRGAQASSGSPALPRKQRDKSPSSLLEDAKETCFTRDRKGGFFSSFMKKRNAPTPPKRSSSFREMENQP  679
ABL2-210  VVPYLPRLPILPSKTRTLKKQVENKENIEGAQDATENSASSLAPGFIRGAQASSGSPALPRKQRDKSPSSLLEDAKETCFTRDRKGGFFSSFMKKRNAPTPPKRSSSFREMENQP  679
ABL2-201  VVPYLPRLPILPSKTRTLKKQVENKENIEGAQDATENSASSLAPGFIRGAQASSGSPALPRKQRDKSPSSLLEDAKETCFTRDRKGGFFSSFMKKRNAPTPPKRSSSFREMENQP  664
```

```
logo        HKKYELTGNFSSVASLQHADGFSFTPAQQEANLVPPKCYGGSFAQRNLCNDDGGGGGGSGTAGGGWSGITGFFTPRLIKKTLGLRAGKPTASDDTSKPFPRSNSTSSMSSGLPEQ
IMATINIB    ...................................................................................................................    26
PF00017     ...................................................................................................................    76
PF08919     ...................................................................................................................     0
PF00018     ...................................................................................................................    47
PF07714     ...................................................................................................................   251
ABL2-203    ...................................................................................................................   542
ABL2-202    HKKYELTGNFSSVASLQHADGFSFTPAQQEANLVPPKCYGGSFAQRNLCNDDGGGGGGSGTAGGGWSGITGFFTPRLIKKTLGLRAGKPTASDDTSKPFPRSNSTSSMSSGLPEQ   773
ABL2-207    HKKYELT.......................................................................................................GLPEQ   670
ABL2-211    HKKYELTGNFSSVASLQHADGFSFTPAQQEANLVPPKCYGGSFAQRNLCNDDGGGGGGSGTAGGGWSGITGFFTPRLIKKTLGLRAGKPTASDDTSKPFPRSNSTSSMSSGLPEQ   779
ABL2-206    HKKYELT.......................................................................................................GLPEQ   655
ABL2-204    HKKYELTGNFSSVASLQHADGFSFTPAQQEANLVPPKCYGGSFAQRNLCNDDGGGGGGSGTAGGGWSGITGFFTPRLIKKTLGLRAGKPTASDDTSKPFPRSNSTSSMSSGLPEQ   794
ABL2-210    HKKYELT.......................................................................................................GLPEQ   691
ABL2-201    HKKYELT.......................................................................................................GLPEQ   676

logo        DRMAMTLPRNCQRSKLQLERTVSTSSQPEENVDRANDMLPKKSEESAAPSRERPKAKLLPRGATALPLRTPSGDLAITEKDPPGVGVAGVAAAPKGKEKNGGARLGMAGVPEDGE
IMATINIB    ...................................................................................................................    26
PF00017     ...................................................................................................................    76
PF08919     ...................................................................................................................     0
PF00018     ...................................................................................................................    47
PF07714     ...................................................................................................................   251
ABL2-203    ...................................................................................................................   542
ABL2-202    DRMAMTLPRNCQRSKLQLERTVSTSSQPEENVDRANDMLPKKSEESAAPSRERPKAKLLPRGATALPLRTPSGDLAITEKDPPGVGVAGVAAAPKGKEKNGGARLGMAGVPEDGE   888
ABL2-207    DRMAMTLPRNCQRSKLQLERTVSTSSQPEENVDRANDMLPKKSEESAAPSRERPKAKLLPRGATALPLRTPSGDLAITEKDPPGVGVAGVAAAPKGKEKNGGARLGMAGVPEDGE   785
ABL2-211    DRMAMTLPRNCQRSKLQLERTVSTSSQPEENVDRANDMLPKKSEESAAPSRERPKAKLLPRGATALPLRTPSGDLAITEKDPPGVGVAGVAAAPKGKEKNGGARLGMAGVPEDGE   894
ABL2-206    DRMAMTLPRNCQRSKLQLERTVSTSSQPEENVDRANDMLPKKSEESAAPSRERPKAKLLPRGATALPLRTPSGDLAITEKDPPGVGVAGVAAAPKGKEKNGGARLGMAGVPEDGE   770
ABL2-204    DRMAMTLPRNCQRSKLQLERTVSTSSQPEENVDRANDMLPKKSEESAAPSRERPKAKLLPRGATALPLRTPSGDLAITEKDPPGVGVAGVAAAPKGKEKNGGARLGMAGVPEDGE   909
ABL2-210    DRMAMTLPRNCQRSKLQLERTVSTSSQPEENVDRANDMLPKKSEESAAPSRERPKAKLLPRGATALPLRTPSGDLAITEKDPPGVGVAGVAAAPKGKEKNGGARLGMAGVPEDGE   806
ABL2-201    DRMAMTLPRNCQRSKLQLERTVSTSSQPEENVDRANDMLPKKSEESAAPSRERPKAKLLPRGATALPLRTPSGDLAITEKDPPGVGVAGVAAAPKGKEKNGGARLGMAGVPEDGE   791

logo        QPGWPSPAKAAPVLPTTHNHKVPVLISPTLKHTPADVQLIGTDSQGNKFKLLSEHQVTSSGDKDRPRRVKPKCAPPPPPVMRLLQHPSICSDPTEEPTALTAGQSTSETQEGGKK
IMATINIB    ...................................................................................................................    26
PF00017     ...................................................................................................................    76
PF08919     ...................................................................................................................     0
PF00018     ...................................................................................................................    47
PF07714     ...................................................................................................................   251
ABL2-203    ...................................................................................................................   542
ABL2-202    QPGWPSPAKAAPVLPTTHNHKVPVLISPTLKHTPADVQLIGTDSQGNKFKLLSEHQVTSSGDKDRPRRVKPKCAPPPPPVMRLLQHPSICSDPTEEPTALTAGQSTSETQEGGKK  1003
ABL2-207    QPGWPSPAKAAPVLPTTHNHKVPVLISPTLKHTPADVQLIGTDSQGNKFKLLSEHQVTSSGDKDRPRRVKPKCAPPPPPVMRLLQHPSICSDPTEEPTALTAGQSTSETQEGGKK   900
ABL2-211    QPGWPSPAKAAPVLPTTHNHKVPVLISPTLKHTPADVQLIGTDSQGNKFKLLSEHQVTSSGDKDRPRRVKPKCAPPPPPVMRLLQHPSICSDPTEEPTALTAGQSTSETQEGGKK  1009
ABL2-206    QPGWPSPAKAAPVLPTTHNHKVPVLISPTLKHTPADVQLIGTDSQGNKFKLLSEHQVTSSGDKDRPRRVKPKCAPPPPPVMRLLQHPSICSDPTEEPTALTAGQSTSETQEGGKK   885
ABL2-204    QPGWPSPAKAAPVLPTTHNHKVPVLISPTLKHTPADVQLIGTDSQGNKFKLLSEHQVTSSGDKDRPRRVKPKCAPPPPPVMRLLQHPSICSDPTEEPTALTAGQSTSETQEGGKK  1024
ABL2-210    QPGWPSPAKAAPVLPTTHNHKVPVLISPTLKHTPADVQLIGTDSQGNKFKLLSEHQVTSSGDKDRPRRVKPKCAPPPPPVMRLLQHPSICSDPTEEPTALTAGQSTSETQEGGKK   921
ABL2-201    QPGWPSPAKAAPVLPTTHNHKVPVLISPTLKHTPADVQLIGTDSQGNKFKLLSEHQVTSSGDKDRPRRVKPKCAPPPPPVMRLLQHPSICSDPTEEPTALTAGQSTSETQEGGKK   906
```

```
logo       AALGAVPISGKAGRPVMPPPQVPLPTSSISPAKMANGTAGTKVALRKTKQAAEKISADKISKEALLECADLLSSALTEPVPNSQLVDTGHQLLDYCSGYVDCIPQTRNKFAFREA
IMATINIB   ...................................................................................................................    26
PF00017    ...................................................................................................................    76
PF08919    ..........................................................KISKEALLECADLLSSALTEPVPNSQLVDTGHQLLDYCSGYVDCIPQTRNKFAFREA    57
PF00018    ...................................................................................................................    47
PF07714    ...................................................................................................................   251
ABL2-203   ...................................................................................................................   542
ABL2-202   AALGAVPISGKAGRPVMPPPQVPLPTSSISPAKMANGTAGTKVALRKTKQAAEKISADKISKEALLECADLLSSALTEPVPNSQLVDTGHQLLDYCSGYVDCIPQTRNKFAFREA  1118
ABL2-207   AALGAVPISGKAGRPVMPPPQVPLPTSSISPAKMANGTAGTKVALRKTKQAAEKISADKISKEALLECADLLSSALTEPVPNSQLVDTGHQLLDYCSGYVDCIPQTRNKFAFREA  1015
ABL2-211   AALGAVPISGKAGRPVMPPPQVPLPTSSISPAKMANGTAGTKVALRKTKQAAEKISADKISKEALLECADLLSSALTEPVPNSQLVDTGHQLLDYCSGYVDCIPQTRNKFAFREA  1124
ABL2-206   AALGAVPISGKAGRPVMPPPQVPLPTSSISPAKMANGTAGTKVALRKTKQAAEKISADKISKEALLECADLLSSALTEPVPNSQLVDTGHQLLDYCSGYVDCIPQTRNKFAFREA  1000
ABL2-204   AALGAVPISGKAGRPVMPPPQVPLPTSSISPAKMANGTAGTKVALRKTKQAAEKISADKISKEALLECADLLSSALTEPVPNSQLVDTGHQLLDYCSGYVDCIPQTRNKFAFREA  1139
ABL2-210   AALGAVPISGKAGRPVMPPPQVPLPTSSISPAKMANGTAGTKVALRKTKQAAEKISADKISKEALLECADLLSSALTEPVPNSQLVDTGHQLLDYCSGYVDCIPQTRNKFAFREA  1036
ABL2-201   AALGAVPISGKAGRPVMPPPQVPLPTSSISPAKMANGTAGTKVALRKTKQAAEKISADKISKEALLECADLLSSALTEPVPNSQLVDTGHQLLDYCSGYVDCIPQTRNKFAFREA  1021

logo       VSKLELSLQELQVSSAAAGVPGTNPVLNNLLSCVQEISDVVQR
IMATINIB   ...........................................    26
PF00017    ...........................................    76
PF08919    VSKLELSLQELQVSSAAAGVPGTNPVLNNLLSCVQEISDVVQR   100
PF00018    ...........................................    47
PF07714    ...........................................   251
ABL2-203   ...........................................   542
ABL2-202   VSKLELSLQELQVSSAAAGVPGTNPVLNNLLSCVQEISDVVQR  1161
ABL2-207   VSKLELSLQELQVSSAAAGVPGTNPVLNNLLSCVQEISDVVQR  1058
ABL2-211   VSKLELSLQELQVSSAAAGVPGTNPVLNNLLSCVQEISDVVQR  1167
ABL2-206   VSKLELSLQELQVSSAAAGVPGTNPVLNNLLSCVQEISDVVQR  1043
ABL2-204   VSKLELSLQELQVSSAAAGVPGTNPVLNNLLSCVQEISDVVQR  1182
ABL2-210   VSKLELSLQELQVSSAAAGVPGTNPVLNNLLSCVQEISDVVQR  1079
ABL2-201   VSKLELSLQELQVSSAAAGVPGTNPVLNNLLSCVQEISDVVQR  1064
```

X non conserved
X similar
X ≥0% conserved
X ≥50% conserved

```
logo
             MLLGWASLLLCAFRLPLAAVGPAATPAQDKAGQPPTAAAAAQPRRRQGEEVQERAEPPGHPHPLAQRRRSKGLVQNIDQLYSGGGKVGYLVYAGGRRFLLDLERDGSVGI
BATIMASTAT   ..............................................................................................................    0
PF01562      ..............................................................PLAQRRRSKGLVQNIDQLYSGGGKVGYLVYAGGRRFLLDLERDGSVGI   48
PF01421      ..............................................................................................................    0
PF05986      ..............................................................................................................    0
PF00090      ..............................................................................................................    0
ADAMTS5-201  MLLGWASLLLCAFRLPLAAVGPAATPAQDKAGQPPTAAAAAQPRRRQGEEVQERAEPPGHPHPLAQRRRSKGLVQNIDQLYSGGGKVGYLVYAGGRRFLLDLERDGSVGI  110

logo
             AGFVPAGGGTSAPWRHRSHCFYRGTVDGSPRSLAVFDLCGGLDGFFAVKHARYTLKPLLRGPWAEEEKGRVYGDGSARILHVYTREGFSFEALPPRASCETPASTPEAHE
BATIMASTAT   ..............................................................................................................    0
PF01562      AGFVPAGGGTSAPWRHRSHCFYRGTVDGSPRSLAVFDLCGGLDGFFAVKHARYTLKPLLRG.................................................  109
PF01421      ..............................................................................................................    0
PF05986      ..............................................................................................................    0
PF00090      ..............................................................................................................    0
ADAMTS5-201  AGFVPAGGGTSAPWRHRSHCFYRGTVDGSPRSLAVFDLCGGLDGFFAVKHARYTLKPLLRGPWAEEEKGRVYGDGSARILHVYTREGFSFEALPPRASCETPASTPEAHE  220

logo
             HAPAHSNPSGRAALASQLLDQSALSPAGGSGPQTWWRRRRRSISRARQVELLLVADASMARLYGRGLQHYLLTLASIANRLYSHASIENHIRLAVVKVVVLGDKDKSLEV
BATIMASTAT   ..............................................................................................................    0
PF01562      ..............................................................................................................  109
PF01421      ...............................................QVELLLVADASMARLYGRGLQHYLLTLASIANRLYSHASIENHIRLAVVKVVVLGDKDKSLEV   63
PF05986      ..............................................................................................................    0
PF00090      ..............................................................................................................    0
ADAMTS5-201  HAPAHSNPSGRAALASQLLDQSALSPAGGSGPQTWWRRRRRSISRARQVELLLVADASMARLYGRGLQHYLLTLASIANRLYSHASIENHIRLAVVKVVVLGDKDKSLEV  330

logo
             SKNAATTLKNFCKWQHQHNQLGDDHEEHYDAAILFTREDLCGHHSCDTLGMADVGTICSPERSCAVIEDDGLHAAFTVAHEIGHLLGLSHDDSKFCEETFGSTEDKRLMS
BATIMASTAT   ..............................................DTLGM............................HE..H.....H...................S   10
PF01562      ..............................................................................................................  109
PF01421      SKNAATTLKNFCKWQHQHNQLGDDHEEHYDAAILFTREDLCGHHSCDTLGMADVGTICSPERSCAVIEDDGLHAAFTVAHEIGHLLGLSHDDSKFCEETFGSTEDKRLMS  173
PF05986      ..............................................................................................................    0
PF00090      ..............................................................................................................    0
ADAMTS5-201  SKNAATTLKNFCKWQHQHNQLGDDHEEHYDAAILFTREDLCGHHSCDTLGMADVGTICSPERSCAVIEDDGLHAAFTVAHEIGHLLGLSHDDSKFCEETFGSTEDKRLMS  440

logo
             SILTSIDASKPWSKCTSATITEFLDDGHGNCLLDLPRKQILGPEELPGQTYDATQQCNLTFGPEYSVCPGMDVCARLWCAVVRQGQMVCLTKKLPAVEGTPCGKGRICLQ
BATIMASTAT   SIL...........................................................................................................   13
PF01562      ..............................................................................................................  109
PF01421      SILTSIDASKPWSKCTSATITEFLDDGHGNCLLDLP..........................................................................  209
PF05986      ..............................................................................................................    0
PF00090      ..............................................................................................................    0
ADAMTS5-201  SILTSIDASKPWSKCTSATITEFLDDGHGNCLLDLPRKQILGPEELPGQTYDATQQCNLTFGPEYSVCPGMDVCARLWCAVVRQGQMVCLTKKLPAVEGTPCGKGRICLQ  550
```

```
logo
             GKCVDKTKKKYYSTSSHGNWGSWGSWGQCSRSCGGGVQFAYRHCNNPAPRNNGRYCTGKRAIYRSCSLMPCPPNGKSFRHEQCEAKNGYQSDAKGVKTFVEWVPKYAGVL
BATIMASTAT   ..............................................................................................................  13
PF01562      ..............................................................................................................  109
PF01421      ..............................................................................................................  209
PF05986      ..............................................................................................................  0
PF00090      .....................SWGSWGQCSRSCGGGVQFAYRHCNNPAPRNNGRYCTGKRAIYRSCSLMPC.......................................  50
ADAMTS5-201  GKCVDKTKKKYYSTSSHGNWGSWGSWGQCSRSCGGGVQFAYRHCNNPAPRNNGRYCTGKRAIYRSCSLMPCPPNGKSFRHEQCEAKNGYQSDAKGVKTFVEWVPKYAGVL  660

logo
             PADVCKLTCRAKGTGYYVVFSPKVTDGTECRLYSNSVCVRGKCVRTGCDGIIGSKLQYDKCGVCGGDNSSCTKIVGTFNKKSKGYTDVVRIPEGATHIKVRQFKAKDQTR
BATIMASTAT   ..............................................................................................................  13
PF01562      ..............................................................................................................  109
PF01421      ..............................................................................................................  209
PF05986      ........................................................................KIVGTFNKKSKGYTDVVRIPEGATHIKVRQFKAKDQTR  38
PF00090      ..............................................................................................................  50
ADAMTS5-201  PADVCKLTCRAKGTGYYVVFSPKVTDGTECRLYSNSVCVRGKCVRTGCDGIIGSKLQYDKCGVCGGDNSSCTKIVGTFNKKSKGYTDVVRIPEGATHIKVRQFKAKDQTR  770

logo
             FTAYLALKKKNGEYLINGKYMISTSETIIDINGTVMNYSGWSHRDDFLHGMGYSATKEILIVQILATDPTKPLDVRYSFFVPKKSTPKVNSVTSHGSNKVGSHTSQPQWV
BATIMASTAT   ..............................................................................................................  13
PF01562      ..............................................................................................................  109
PF01421      ..............................................................................................................  209
PF05986      FTAYLALKKKNGEYLINGKYMISTSETIIDINGTVMNYSGWSHRDDFLHGMGYSATKEILIVQILATDPTKPLDVRYSFFVP............................  120
PF00090      ..............................................................................................................  50
ADAMTS5-201  FTAYLALKKKNGEYLINGKYMISTSETIIDINGTVMNYSGWSHRDDFLHGMGYSATKEILIVQILATDPTKPLDVRYSFFVPKKSTPKVNSVTSHGSNKVGSHTSQPQWV  880

logo
             TGPWLACSRTCDTGWHTRTVQCQDGNRKLAKGCPLSQRPSAFKQCLLKKC
BATIMASTAT   ..................................................  13
PF01562      ..................................................  109
PF01421      ..................................................  209
PF05986      ..................................................  120
PF00090      TGPWLACSRTCDTGWHTRTVQCQDGNRKLAKGCPLSQRPSAFKQCLLKKC  100
ADAMTS5-201  TGPWLACSRTCDTGWHTRTVQCQDGNRKLAKGCPLSQRPSAFKQCLLKKC  930
```

X non conserved
X similar
X $\geq 0\%$ conserved
X $\geq 50\%$ conserved

logo

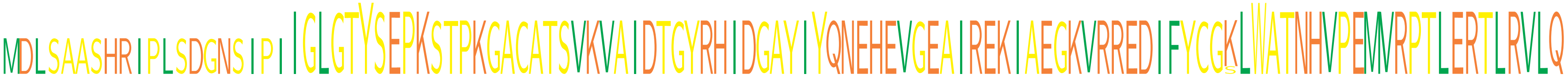

```
FINASTERIDE  .........................Y...............................Y..............................W.....................    3
PF00248.2    ....................IGLGTYSEPKSTPKGACATSVKVAIDTGYRHIDGAYIYQNEHEVGEAIREKIAEGKVRREDIFYCGKLWATNHVPEMVRPTLERTLRVLQ   90
PF00248      ....................IGLGTYSEPKSTPKGACATSVKVAIDTGYRHIDGAYIYQNEHEVGEAIREKIAEGKVRREDIFYCGKLWATNHVPEMVRPTLERTLRVLQ   90
PF00248.1    ....................IGLGTYSEPKSTPKGACATSVKVAIDTGYRHIDGAYIYQNEHEVGEAIREKIAEGKVRREDIFYCGKLWATNHVPEMVRPTLERTLRVLQ   90
AKR1D1-201   MDLSAASHRIPLSDGNSIPIIGLGTYSEPKSTPKGACATSVKVAIDTGYRHIDGAYIYQNEHEVGEAIREKIAEGKVRREDIFYCGKLWATNHVPEMVRPTLERTLRVLQ  110
AKR1D1-203   MDLSAASHRIPLSDGNSIPIIGLGTYSEPKSTPKGACATSVKVAIDTGYRHIDGAYIYQNEHEVGEAIREKIAEGKVRREDIFYCGKLWATNHVPEMVRPTLERTLRVLQ  110
AKR1D1-202   MDLSAASHRIPLSDGNSIPIIGLGTYSEPKSTPKGACATSVKVAIDTGYRHIDGAYIYQNEHEVGEAIREKIAEGKVRREDIFYCGKLWATNHVPEMVRPTLERTLRVLQ  110
AKR1D1-204   MDLSAASHRIPLSDGNSIPIIGLGTYSEPK........................................................SLWATNHVPEMVRPTLERTLRVLQ   54
```

logo

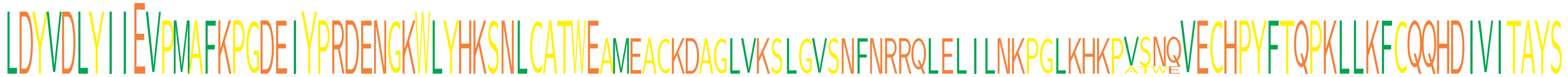

```
FINASTERIDE  .........E...........Y.......W................................................................................    6
PF00248.2    LDYVDLYIIEV....................................................................ATWEVECHPYFTQPKLLKFCQQHDIVITAYS  132
PF00248      LDYVDLYIIEVPMAFKPGDEIYPRDENGKWLYHKSNLCATWEAMEACKDAGLVKSLGVSNFNRRQLELILNKPGLKHKPVSNQVECHPYFTQPKLLKFCQQHDIVITAYS  200
PF00248.1    LDYVDLYIIEVPMAFKPGDEIYPRDENGKWLYHKSNLCATWEAMEACKDAGLVKSLGVSNFNRRQLELILNKPGLKHKPVSNQVECHPYFTQPKLLKFCQQHDIVITAYS  200
AKR1D1-201   LDYVDLYIIEVPMAFKPGDEIYPRDENGKWLYHKSNLCATWEAMEACKDAGLVKSLGVSNFNRRQLELILNKPGLKHKPVSNQVECHPYFTQPKLLKFCQQHDIVITAYS  220
AKR1D1-203   LDYVDLYIIEVPMAFKPGDEIYPRDENGKWLYHKSNLCATWEAMEACKDAGLVKSLGVSNFNRRQLELILNKPGLKHKPVSNQVECHPYFTQPKLLKFCQQHDIVITAYS  220
AKR1D1-202   LDYVDLYIIEVPMAFKPGDEIYPRDENGKWLYHKSNLCATWE.........................................VECHPYFTQPKLLKFCQQHDIVITAYS  179
AKR1D1-204   LDYVDLYIIEVPMAFKPGDEIYPRDENGKWLYHKSNLCATWE....................................................................   96
```

logo

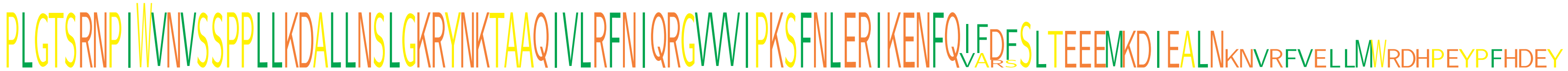

```
FINASTERIDE  .........W..................................................................................MW............    9
PF00248.2    PLGTSRNPIWVNVSSPPLLKDALLNSLGKRYNKTAAQIVLRFNIQRGVVVIPKSFNLERIKENFQIFDFSLTEEEMKDIEALN.......................  215
PF00248      PLGTSRNPIWVNVSSPPLLKDALLNSLGKRYNKTAAQIVLRFNIQRGVVVIPKSFNLERIKENFQIFDFSLTEEEMKDIEALN.......................  283
PF00248.1    PLGTSRNPIWVNVSSPPLLKDALLNSLGKRYNKTAAQIVLRFNIQRGVVVIPKSFNLERIKENFQVA.......................................  267
AKR1D1-201   PLGTSRNPIWVNVSSPPLLKDALLNSLGKRYNKTAAQIVLRFNIQRGVVVIPKSFNLERIKENFQIFDFSLTEEEMKDIEALNKNVRFVELLMWRDHPEYPFHDEY  326
AKR1D1-203   PLGTSRNPIWVNVSSPPLLKDALLNSLGKRYNKTAAQIVLRFNIQRGVVVIPKSFNLERIKENFQVARSS....................................  290
AKR1D1-202   PLGTSRNPIWVNVSSPPLLKDALLNSLGKRYNKTAAQIVLRFNIQRGVVVIPKSFNLERIKENFQIFDFSLTEEEMKDIEALNKNVRFVELLMWRDHPEYPFHDEY  285
AKR1D1-204   ..........................................................................................................   96
```

X non conserved
X similar
X $\geq 0\%$ conserved
X $\geq 50\%$ conserved

```
logo
             MSDVAIVKEGWLHKRGEYIKTWRPRYFLLKNDGTFIGYKERPQDVDQREAPLNNFSVAQCQLMKTERPRPNTFIIRCLQWTTVIERTFHVETPEEREEWTTAIQTVADGL
IPATASERTIB  ..............................................................................................................   0
PF00169      ......VKEGWLHKRGEYIKTWRPRYFLLKNDGTFIGYKERPQDVDQREAPLNNFSVAQCQLMKTERPRPNTFIIRCLQWTTVIERTFHVETPEEREEWTTAIQTV....   100
PF00433      ..............................................................................................................   0
PF00069.1    ..............................................................................................................   0
PF00069.2    ..............................................................................................................   0
PF00169.1    ......VKEGWLHKRGEYIKTWRPRYFLLKNDGTFIGYKERPQDVDQREAPLNNFSVAQCQLMKTERPRPNTFIIRCLQWTTVIERTFH.....................   83
PF00069      ..............................................................................................................   0
PF00433.1    ..............................................................................................................   0
AKT1-211     MSDVAIVKEGWLHKRGEYIKTWRPRYFLLKNDGTFIGYKERPQDVDQREAPLNNFSVAQCQLMKTERPRPNTFIIRCLQWTTVIERTFHVETPEEREEWTTAIQTVADGL   110
AKT1-203     MSDVAIVKEGWLHKRGEYIKTWRPRYFLLKNDGTFIGYKERPQDVDQREAPLNNFSVAQCQLMKTERPRPNTFIIRCLQWTTVIERTFHVETPEEREEWTTAIQTVADGL   110
AKT1-201     MSDVAIVKEGWLHKRGEYIKTWRPRYFLLKNDGTFIGYKERPQDVDQREAPLNNFSVAQCQLMKTERPRPNTFIIRCLQWTTVIERTFHVETPEEREEWTTAIQTVADGL   110
AKT1-215     MSDVAIVKEGWLHKRGEYIKTWRPRYFLLKNDGTFIGYKERPQDVDQREAPLNNFSVAQCQLMKTERPRPNTFIIRCLQWTTVIERTFHVE...................   91
AKT1-208     MSDVAIVKEGWLHKRGEYIKTWRPRYFLLKNDGTFIGYKERPQDVDQREAPLNNFSVAQCQLMKTERPRPNTFIIRCLQWTTVIERTFHVETPEEREEWTTAIQTVADGL   110
AKT1-202     MSDVAIVKEGWLHKRGEYIKTWRPRYFLLKNDGTFIGYKERPQDVDQREAPLNNFSVAQCQLMKTERPRPNTFIIRCLQWTTVIERTFHVETPEEREEWTTAIQTVADGL   110
AKT1-209     ..............................................................................................................   0
AKT1-207     ..............................................................................................................   0
AKT1-214     MSDVAIVKEGWLHKRGEYIKTWRPRYFLLKNDGTFIGYKERPQDVDQREAPLNNFSVAQCQLMKTERPRPNTFIIRCLQWTTVIERTFHVETPEEREEWTTAIQTVADGL   110
AKT1-213     ..............................................................................................................   0

logo
             KKQEEEEMDFRSGSPSDNSGAEEMEVSLAKPKHRVTMNEFEYLKLLGKGTFGKVILVKEKATGRYYAMKILKKEVIVAKDEVAHTLTENRVLQNSRHPFLTALKYSFQTH
IPATASERTIB  .............................................LGKG..GKV............A.K...............................T.........   10
PF00169      ..............................................................................................................   100
PF00433      ..............................................................................................................   0
PF00069.1    ..............................................................................................................   0
PF00069.2    ..............................................................................................................   0
PF00169.1    ..............................................................................................................   83
PF00069      ........................................EYLKLLGKGTFGKVILVKEKATGRYYAMKILKKEVIVAKDEVAHTLTENRVLQNSRHPFLTALKYSFQTH   70
PF00433.1    ..............................................................................................................   0
AKT1-211     KKQEEEEMDFRSGSPSDNSGAEEMEVSLAKPKHRVTMNEFEYLKLLGKGTFGKVILVKEKATGRYYAMKILKKEVIVAKDEVAHTLTENRVLQNSRHPFLTALKYSFQTH   220
AKT1-203     KKQEEEEMDFRSGSPSDNSGAEEMEVSLAKPKHRVTMNEFEYLKLLGKGTFGKVILVKEKATGRYYAMKILKKEVIVAKDEVAHTLTENRVLQNSRHPFLTALKYSFQTH   220
AKT1-201     KKQEEEEMDFRSGSPSDNSGAEEMEVSLAKPKHRVTMNEFEYLKLLGKGTFGKVILVKEKATGRYYAMKILKKEVIVAKDEVAHTLTENRVLQNSRHPFLTALKYSFQTH   220
AKT1-215     ..............................................................................................................   91
AKT1-208     KKQEEEEMDFRSGSPSDNSGAEEMEVSLAKPKHRVTMNEFEYLKLLGKGTFGKVILVKEKATGRYYAMKILKKEVIVAKDEVAHTLTENRVLQNSRHPFLTALKYSFQTH   220
AKT1-202     KKQEEEEMDFRSGSPSDNSGAEEMEVSLAKPKHRVTMNEFEYLKLLGKGTFGKVILVKEKATGRYYAMKILKKEVIVAKDEVAHTLTENRVLQNSRHPFLTALKYSFQTH   220
AKT1-209     ..............................................................................................................   0
AKT1-207     ..............................................................................................................   0
AKT1-214     KKQEEEEMDFRSGSPSDNSGAEEMEVSLAKPKHRVTMNEFEYLKLLGKGTFGKVILVKEKATGRYYAMKILKKEVIVAKDEVAHTLTENRVLQNSRHPFLTALKYSFQTH   220
AKT1-213     ..............................................................................................................   0
```

```
logo         DRLCFVMEYANGGELFFHLSRERVFSEDRARFYGAEIVSALDYLHSEKNVVYRDLKLENLMLDKDGHIKITDFGLCKEGIKDGATMKTFCGTPEYLAPEVLEDNDYGRAV
IPATASERTIB  ......MEYA...E...........................................E..M.........T.......................................  18
PF00169      ..............................................................................................................  100
PF00433      ..............................................................................................................  0
PF00069.1    ............................................HSEKNVVYRDLKLENLMLDKDGHIKITDFGLCKEGIKDGATMKTFCGTPEYLAPE...........  55
PF00069.2    .................................................................................DGATMKTFCGTPEYLAPEVLEDNDYGRAV  29
PF00169.1    ..............................................................................................................  83
PF00069      DRLCFVMEYANGGELFFHLSRERVFSEDRARFYGAEIVSALDYLHSEKNVVYRDLKLENLMLDKDGHIKITDFGLCKEGIKDGATMKTFCGTPEYLAPEVLEDNDYGRAV  180
PF00433.1    ..............................................................................................................  0
AKT1-211     DRLCFVMEYANGGELFFHLSRERVFSEDRARFYGAEIVSALDYLHSEKNVVYRDLKLENLMLDKDGHIKITDFGLCKEGIKDGATMKTFCGTPEYLAPEVLEDNDYGRAV  330
AKT1-203     DRLCFVMEYANGGELFFHLSRERVFSEDRARFYGAEIVSALDYLHSEKNVVYRDLKLENLMLDKDGHIKITDFGLCKEGIKDGATMKTFCGTPEYLAPEVLEDNDYGRAV  330
AKT1-201     DRLCFVMEYANGGELFFHLSRERVFSEDRARFYGAEIVSALDYLHSEKNVVYRDLKLENLMLDKDGHIKITDFGLCKEGIKDGATMKTFCGTPEYLAPEVLEDNDYGRAV  330
AKT1-215     ..............................................................................................................  91
AKT1-208     DRLCFVMEYANGGELFFHLSRERVFSEDRARFYGAEIVSALDYLHSEKNVVYRDLKLENLMLDKDGHIKITDFGLCKEGIKDGATMKTFCGTPEYLAPEVLEDNDYGRAV  330
AKT1-202     DRLCFVMEYANGGELFFHLSRERVFSEDRARFYGAEIVSALDYLHSEKNVVYRDLKLENLMLDKDGHIKITDFGLCKEGIKDGATMKTFCGTPEYLAPEVLEDNDYGRAV  330
AKT1-209     ........................................................LENLMLDKDGHIKITDFGLCKEGIKDGATMKTFCGTPEYLAPEVLEDNDYGRAV  54
AKT1-207     ...........................................XHSEKNVVYRDLKLENLMLDKDGHIKITDFGLCKEGIKDGATMKTFCGTPEYLAPE...........  56
AKT1-214     DRLCFVMEYANGGELFFHLSRERVFSEDRARFYGAEIVSALDYLHSEKNVVYRDLKLENLMLDKDGHIKITDFGLCKEGIKDGATMKTFCGTPEYLAPEVLEDNDYGRAV  330
AKT1-213     .............................................................................XGIKDGATMKTFCGTPEYLAPEVLEDNDYGRAV  33

logo         DWWGLGVVMYEMMCGRLPFYNQDHEKLFELILMEEIRFPRTLGPEAKSLLSGLLKKDPKQRLGGGSEDAKEIMQHRFFAGIVWQHVYEKKLSPPFKPQVTSETDTRYFDE
IPATASERTIB  ..............................................................................................................  18
PF00169      ..............................................................................................................  100
PF00433      ..................................................................................................VTSETDTRYFDE  12
PF00069.1    ..............................................................................................................  55
PF00069.2    DWWGLGVVMYEMMCGRLPFYNQDHEKLFELILMEEIRFPRTLGPEAKSLLSGLLKKDPKQRLGGGSEDAKEIMQHRFF................................  107
PF00169.1    ..............................................................................................................  83
PF00069      DWWGLGVVMYEMMCGRLPFYNQDHEKLFELILMEEIRFPRTLGPEAKSLLSGLLKKDPKQRLGGGSEDAKEIMQHRFF................................  258
PF00433.1    ..................................................................................................VTSETDTRYFDE  12
AKT1-211     DWWGLGVVMYEMMCGRLPFYNQDHEKLFELILMEEIRFPRTLGPEAKSLLSGLLKKDPKQRLGGGSEDAKEIMQHRFFAGIVWQHVYEKKLSPPFKPQVTSETDTRYFDE  440
AKT1-203     DWWGLGVVMYEMMCGRLPFYNQDHEKLFELILMEEIRFPRTLGPEAKSLLSGLLKKDPKQRLGGGSEDAKEIMQHRFFAGIVWQHVYEKKLSPPFKPQVTSETDTRYFDE  440
AKT1-201     DWWGLGVVMYEMMCGRLPFYNQDHEKLFELILMEEIRFPRTLGPEAKSLLSGLLKKDPKQRLGGGSEDAKEIMQHRFFAGIVWQHVYEKKLSPPFKPQVTSETDTRYFDE  440
AKT1-215     ..............................................................................................................  91
AKT1-208     DWWGLGVVMYEMMCGRLPFYNQDHEKLFELILMEEIRFPRTLGPEAKSLLSGLLKKDPKQRLGGGSEDAKEIMQHRFFAGIVWQHVYEKKLSPPFKPQVTSETDTRYFDE  440
AKT1-202     DWWGLGVVMYEMMCGRLPFYNQDHEKLFELILMEEIRFPRTLGPEAKSLLSGLLKKDPKQRLGGGSEDAKEIMQHRFFAGIVWQHVYEKKLSPPFKPQVTSETDTRYFDE  440
AKT1-209     DWWGLGVVMYEMMCGRLPFYNQDHEKLFELILMEEIRFPRTLGPEAKSLLSGLLKKDPKQRLGGGSEDAKEIMQHRFFAGIVWQHVYEKKGP..................  146
AKT1-207     ......................DHEKLFELILMEEIRFPRTLGPEAKSLLSGLLKKDPKQRLGGGSEDAKEIMQHRFFAGIVWQHVYEKKLSPPFKPQVTSETDTRYFDE  144
AKT1-214     DWWGLGVVMYEMMCGRLPFYNQDHEKLFELILMEEIRFPRTLGPEAKSLLSGLLKKDPKQRLGGGSEDAKEIMQHRFFAGIVWQHVYEKKLSPPFKPQVTSETDTRYFDE  440
AKT1-213     DWWGLGVVMYEMMCGRLPFYNQDHEKLFELILMEEIRFPRTLGPEAKSLLSGLLKKDPKQRLGGGSEDAKEIMQHRFFAGIVWQHVYEKKLSPPFKPQVTSETDTRYFDE  143
```

```
logo         EFTAQMITITPPDQDDSMECVDSERRPHFPQFSYSASGTA
IPATASERTIB  ............................F...........   19
PF00169      ........................................  100
PF00433      EFTAQMITITPPDQDDSMECVDSERRPHFPQFSY......   46
PF00069.1    ........................................   55
PF00069.2    ........................................  107
PF00169.1    ........................................   83
PF00069      ........................................  258
PF00433.1    EFTAQMITITPPDQV.........................   27
AKT1-211     EFTAQMITITPPDQDDSMECVDSERRPHFPQFSYSASGTA  480
AKT1-203     EFTAQMITITPPDQDDSMECVDSERRPHFPQFSYSASGTA  480
AKT1-201     EFTAQMITITPPDQDDSMECVDSERRPHFPQFSYSASGTA  480
AKT1-215     ........................................   91
AKT1-208     EFTAQMITITPPDQDDSMECVDSERRPHFPQFSYSASGTA  480
AKT1-202     EFTAQMITITPPDQDDSMECVDSERRPHFPQFSYSASGTA  480
AKT1-209     ........................................  146
AKT1-207     EFTAQMITITPPDQDDSMECVDSERRPHFPQFSYSASGTA  184
AKT1-214     EFTAQMITITPPDQDDSMECVDSERRPHFPQFSYSASGTA  480
AKT1-213     EFTAQMITITPPDQVLGD.CPTGSLPLR............  170
```

X non conserved
X similar
X $\geq$ 0% conserved
X $\geq$ 50% conserved

logo

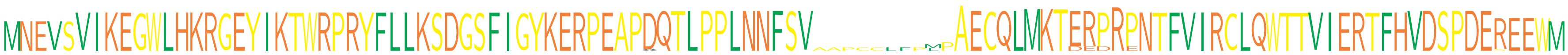

```
A-443654    ..............................................................................................................    0
PF00169.2   .....VIKEGWLHKRGEYIKTWRPRYFLLKSDGSFIGYKERPEAPDQTLPPLNNFSV..........AECQLMKTERPRPNTFVIRCLQWTTVIERTFHVDSPDE.....   90
PF00433     ..............................................................................................................    0
PF00169.1   .....VIKEGWLHKRGEYIKTWRPRYFLLKSDGSFIGYKERPEAPDQTLPPLNNFSVAAPCCLFPLPAECQLMKTERPRPNTFVIRCLQWTTVIERTFHVDSPDEREEWM  105
PF00069.1   ..............................................................................................................    0
PF00169     .....VIKEGWLHKRGEYIKTWRPRYFLLKSDGSFIGYKERPEAPDQTLPPLNNFSV..........AECQLMKTERPRPNTFVIRCLQWTTVIERTFHVDSPDEREEWM   95
PF00069.2   ..............................................................................................................    0
PF00169.3   .....VIKEGWLHKRGEYIKTWRPRYFLLKSDGSFIGYKERPEAPDQTLPPLNNFSV..........AECQLMKT...................................   60
PF00169.4   .....VIKEGWLHKRGEYIKTWRPRYFLLKSDGSFIGYKERPEAPDQTLPPLNNFSV..........AECQLMKTERPRPNTFVIRCLQWTTVIERTFHVDSPDEREEW.   94
PF00433.2   ..............................................................................................................    0
PF00069     ..............................................................................................................    0
PF00069.3   ..............................................................................................................    0
PF00433.1   ..............................................................................................................    0
AKT2-232    ..............................................................................................................    0
AKT2-214    MNEVSVIKEGWLHKRGEYIKTWRPRYFLLKSDGSFIGYKERPEAPDQTLPPLNNFSV..........AECQLMKTERPRPNTFVIRCLQWTTVIERTFHVDSPDEREEWM  100
AKT2-211    MNEVSVIKEGWLHKRGEYIKTWRPRYFLLKSDGSFIGYKERPEAPDQTLPPLNNFSV..........AECQLMKTER.................................   67
AKT2-219    MNEVSVIKEGWLHKRGEYIKTWRPRYFLLKSDGSFIGYKERPEAPDQTLPPLNNFSV..........AECQLMKTERPRPNTFVIRCLQWTTVIERTFHVDSPDEREEWM  100
AKT2-229    MNEVSVIKEGWLHKRGEYIKTWRPRYFLLKSDGSFIGYKERPEAPDQTLPPLNNFSV..........AECQLMKTERPRPNTFVIRCLQWTTVIERTFHVDSPDEREEWM  100
AKT2-224    .................................................................MPA.......DEDRE..............................    8
AKT2-226    ..............................................................................................................    0
AKT2-210    MNEVSVIKEGWLHKRGEYIKTWRPRYFLLKSDGSFIGYKERPEAPDQTLPPLNNFSV..........AECQLMKTERPRPNTFVIRCLQWTTVIERTFHVDSPDEREEWM  100
AKT2-212    MNEVSVIKEGWLHKRGEYIKTWRPRYFLLKSDGSFIGYKERPEAPDQTLPPLNNFSV..........AECQLMKTERPRPNTFVIRCLQWTTVIERTFHVDSPDEREEWM  100
AKT2-241    MNEVSVIKEGWLHKRGEYIKTWRPRYFLLKSDGSFIGYKERPEAPDQTLPPLNNFSV..........AECQLMKTERPRPNTFVIRCLQWTTVIERTFHVDSPDEREEWM  100
AKT2-215    ..............................................................................................................    0
AKT2-234    ........................................................................MKTERPRPNTFVIRCLQWTTVIERTFHVDSPDEREEWM   38
AKT2-213    MNEVSVIKEGWLHKRGEYIKTWRPRYFLLKSDGSFIGYKERPEAPDQTLPPLNNFSV..........AECQLMKTERPRPNTFVIRCLQWTTVIERTFHVDSPDEREEWM  100
AKT2-206    MNEVSVIKEGWLHKRGEYIKTWRPRYFLLKSDGSFIGYKERPEAPDQTLPPLNNFSV..........AECQLMKTERPRPNTFVIRCLQWTTVIERTFHVDSPDEREEWM  100
AKT2-202    MNEVSVIKEGWLHKRGEYIKTWRPRYFLLKSDGSFIGYKERPEAPDQTLPPLNNFSVAAPCCLFPLPAECQLMKTERPRPNTFVIRCLQWTTVIERTFHVDSPDEREEWM  110
AKT2-208    MNEVSVIKEGWLHKRGEYIKTWRPRYFLLKSDGSFIGYKERPEAPDQTLPPLNNFSV..........AECQLMKTERPRPNTFVIRCLQWTTVIERTFHVDSPDE.....   95
AKT2-223    .............................................XQTLPPLNNFSV..........AECQLMKTERPRPNTFVIRCLQWTTVIERTFHVDSPDES....   51
AKT2-209    MNEVSVIKEGWLHKRGEYIKTWRPRYFLLKSDGSFIGYKERPEAPDQTLPPLNNFSV..........AECQLMKTERPRPNTFVIRCLQWTTVIERTFHVDSPDEREEWM  100
AKT2-236    MNEVSVIKEGWLHKRGEYIKTWRPRYFLLKSDGSFIGYKERPEAPDQTLPPLNNFSV..........AECQLMKTERPRPNTFVIRCLQWTTVIERTFHVDSPDE.....   95
AKT2-240    .................................................................MPA.......DEDRE..............................    8
AKT2-207    MNEVSVIKEGWLHKRGEYIKTWRPRYFLLKSDGSFIGYKERPEAPDQTLPPLNNFSV..........AECQLMKTERPRPNTFVIRCLQWTTVIERTFHVDSPDEREEWM  100
AKT2-205    MNEVSVIKEGWLHKRGEYIKTWRPRYFLLKSDGSFIGYKERPEAPDQTLPPLNNFSV..........AECQLMKTERPRPNTFVIRCLQWTTVIERTFHVDSPDEREEWM  100
AKT2-222    ..............................................................................................................    0
AKT2-201    MNEVSVIKEGWLHKRGEYIKTWRPRYFLLKSDGSFIGYKERPEAPDQTLPPLNNFSV..........AECQLMKTERPRPNTFVIRCLQWTTVIERTFHVDSPDEREEWM  100
AKT2-239    MNEVSVIKEGWLHKRGEYIKTWRPRYFLLKSDGSFIGYKERPEAPDQTLPPLNNFSV..........AECQLMKTERPRPNTFVIRCLQWTTVIERTFHVDSPDEREEW.   99
AKT2-231    ..............................................................................................................    0
AKT2-242    ...................................................................................................VDSPDEREEWM   11
AKT2-203    .................................................................MPA.......DEDRE..............................    8
```

logo

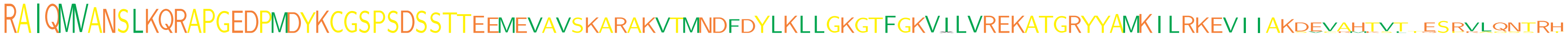

```
A-443654     .........................................................LG...F..V............A.K.............................   6
PF00169.2    ..............................................................................................................  90
PF00433      ..............................................................................................................   0
PF00169.1    RAIQMV........................................................................................................ 111
PF00069.1    .....................................................YLKLLGKGTFGKVILVREKATGRYYAMKILRKEVIIAKDEVAHTVT.ESRVLQNTRH  56
PF00169      RAIQMV........................................................................................................ 101
PF00069.2    ..............................................................................................................   0
PF00169.3    ..............................................................................................................  60
PF00169.4    ..............................................................................................................  94
PF00433.2    ..............................................................................................................   0
PF00069      .....................................................YLKLLGKGTFGKVILVREKATGRYYAMKILRKEVIIAKDEVAHTVT.ESRVLQNTRH  56
PF00069.3    ..............................................................................................................   0
PF00433.1    ..............................................................................................................   0
AKT2-232     ......................KCGSPSDSSTTEEMEVAVSKARAKVTMNDFDYLKLLGKGTFGKVILVREKATGRYYAMKILRKEVIIAK...................  69
AKT2-214     RAIQMVA....................................................................................................... 107
AKT2-211     ..............................................................................................................  67
AKT2-219     RAIQMVANSLKQRAPGEDPMDYKCGSPSDSSTTEEMEVAVSKARAKVTMNDFDYLKL..................................................... 157
AKT2-229     RAIQMVANSLKQRAPGEDPMDYKCGSPSDS................................................................................ 130
AKT2-224     ..............................................................................................................   8
AKT2-226     ..............................................................................................................   0
AKT2-210     RAIQMVANSLKQRAPGEDPMDYKCGSPSDSSTTEEMEVAVSKARAKVTMNDFDYLKLLGKGTFGKVILVREKATGRYYAMKILRKEVIIAKDEVAHTVT.ESRVLQNTRH 209
AKT2-212     RAIQMVANSLKQRAPGEDPMDYKCGSPSDSSTTEEMEVAVSKARAKVTMNDFDYLKLLGKGTFGKVILVREKATGRYYAMKILR.......................... 184
AKT2-241     RAIQMVANSLKQRAPGEDPMDYKCGSPSDSSTTEEMEVAVSKARAKVTMND........................................................... 151
AKT2-215     ..............................................................................................................   0
AKT2-234     RAIQMVANSLKQRAPGEDPMDYKCGSPSDSSTTEEMEVAVSKARAKVTMNDFDYLKLLGKGTFGKVILVREKATGRYYAMKILRKEVIIAKDEVAHTVT.ESRVLQNTRH 147
AKT2-213     RAIQMVANSLKQRAPGEDPMDYKCGSPSDSSTTE............................................................................ 134
AKT2-206     RAIQMVANSLKQRAPGEDPMDYKCGSPSDSSTTEEMEVAVSKARAKVTMNDFDYLKLLGKGTFGKVILVREKATGRYYAMKILRKEVIIAKDEVAHTVT.ESRVLQNTRH 209
AKT2-202     RAIQMVANSLKQRAP............................................................................................... 125
AKT2-208     ..............................................................................................................  95
AKT2-223     ..............................................................................................................  51
AKT2-209     RAIQMVANSLKQRAPGEDP........................................................................................... 119
AKT2-236     ..............................................................................................................  95
AKT2-240     ..............................................................................................................   8
AKT2-207     RAIQMVANSLKQRAPGEDPMDYKCGSPSDSST.............................................................................. 132
AKT2-205     RAIQMVANSLKQRAPGEDPMDYKCGSPSDSSTT............................................................................. 133
AKT2-222     ..............................................................................................................   0
AKT2-201     RAIQMVANSLKQRAPGEDPMDYKCGSPSDSSTTEEMEVAVSKARAKVTMNDFDYLKLLGKGTFGKVILVREKATGRYYAMKILRKEVIIAKDEVAHTVT.ESRVLQNTRH 209
AKT2-239     ..............................................................................................................  99
AKT2-231     ..................................................................XLVREKATGRYYAMKILRKEVIIA....................  24
AKT2-242     RAIQMVANSLKQRAPGEDPMDYKCGSPSDSSTTEEMEVAVSKARAKVTMNDFDYLKLLGKGTFGKVILVREKATGRYYAMKILRKEVIIAKSVCRMKSLTQSPRAGSSRT 121
AKT2-203     ..............................................................................................................   8
```

logo

```
            PFL.TALKYAFQTHDRLCFVMEYANGGELFFHLSRERVFTEERARFYGAEIVSALEYLHSRDVVYRDIKLENLMLDKDGHIKITDFGLCKEGISDGATMKTFCGTPEYLA
A-443654    ....T...............ME.A..............................................EN.M.........TD.........................   15
PF00169.2   ..............................................................................................................   90
PF00433     ..............................................................................................................    0
PF00169.1   ..............................................................................................................  111
PF00069.1   PFL.TALKYAFQTHDRLCFVMEYANGGELFFHLSRERVFTEERARFYGAEIVSALEYLHSRDVVYRDIKLENLMLDKDGHIKITDFGLCKEGISDGATMKTFCGTPEYLA  165
PF00169     ..............................................................................................................  101
PF00069.2   ......LKYAFQTHDRLCFVMEYANGGELFFHLSRERVFTEERARFYGAEIVSALEYLHSRDVVYRDIKLENLMLDKDGHIKITDFGLCKEGISDGATMKTFCGTPEYLA  104
PF00169.3   ..............................................................................................................   60
PF00169.4   ..............................................................................................................   94
PF00433.2   ..............................................................................................................    0
PF00069     PFL.TALKYAFQTHDRLCFVMEYANGGELFFHLSRERVFTEERARFYGAEIVSALEYLHSRDVVYRDIK.........................................  124
PF00069.3   ..............................................................................................................    0
PF00433.1   ..............................................................................................................    0
AKT2-232    .....ALKYAFQTHDRLCFVMEYANGGELFFHLSRERVFTEERARFYGAEIVSALEYLHSRDVVYRDIKLENLMLDKDGHIKITDFGLCKEGISDGATMKTFCGTPEYLA  174
AKT2-214    ..............................................................................................................  107
AKT2-211    ..............................................................................................................   67
AKT2-219    ..............................................................................................................  157
AKT2-229    ..............................................................................................................  130
AKT2-224    ..............................................................................................................    8
AKT2-226    ..............................................................................................................    0
AKT2-210    PFL.TALKYAFQTHDRLCFVMEYANGGELFFHLSRERVFTEERARFYGAEIVSALEYLHSRDVVYRDIK.........................................  277
AKT2-212    ..............................................................................................................  184
AKT2-241    ..............................................................................................................  151
AKT2-215    ..............................................................................................................    0
AKT2-234    PFL.TALKYAFQTHDRLCFVMEYANGGELFFHLSRERVFTEERARFYGAEIVSALEYLHSRDVVYRDIKLENLMLDKDGHIKITDFGLCKEGISDGATMKTFCGTPEYLA  256
AKT2-213    ..............................................................................................................  134
AKT2-206    PFL.TALKYAFQTHDRLCFVMEYANGGELFFHLSRERVFTEERARFYGAEIVSALEYLHSRDVVYRDIKLENLMLDKDGHIKITDFGLCKEGISDGATMKTFCGTPEYLA  318
AKT2-202    ..............................................................................................................  125
AKT2-208    ..............................................................................................................   95
AKT2-223    ..............................................................................................................   51
AKT2-209    ..............................................................................................................  119
AKT2-236    ..............................................................................................................   95
AKT2-240    ..............................................................................................................    8
AKT2-207    ..............................................................................................................  132
AKT2-205    ..............................................................................................................  133
AKT2-222    ..............................................................................................................    0
AKT2-201    PFL.TALKYAFQTHDRLCFVMEYANGGELFFHLSRERVFTEERARFYGAEIVSALEYLHSRDVVYRDIK.........................................  277
AKT2-239    ..............................................................................................................   99
AKT2-231    ....................................................................KLENLMLDKDGHIKITDFGLCKEGISDGA.............   53
AKT2-242    PGTRSSLR......................................................................................................  129
AKT2-203    ..............................................................................................................    8
```

logo

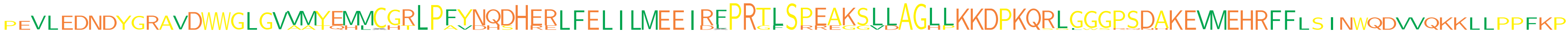

```
A-443654     ..............................................................................................................  15
PF00169.2    ..............................................................................................................  90
PF00433      ..............................................................................................................  0
PF00169.1    ..............................................................................................................  111
PF00069.1    PEVLEDNDYGRAVDWWGLGVVMYEMMCGRLPFYNQDHERLFELILMEEIRFPRTLSPEAKSLLAGLLKKDPKQRLGGGPSDAKEVMEHRFF...................  256
PF00169      ..............................................................................................................  101
PF00069.2    PEVLEDNDYGRAVDWWGLGVVMYEMMCGRLPFYNQDHERLFELILMEEIRFPRTLSPEAKSLLAGLLKKDPKQRLGGGPSDAKEVMEHRFF...................  195
PF00169.3    ..............................................................................................................  60
PF00169.4    ..............................................................................................................  94
PF00433.2    ..............................................................................................................  0
PF00069      ..VLEDNDYGRAVDWWGLGVVMYEMMCGRLPFYNQDHERLFELILMEEIRFPRTLSPEAKSLLAGLLKKDPKQRLGGGPSDAKEVMEHRFF...................  213
PF00069.3    .............DWWGLGVVMYEMMCGRLPFYNQDHERLFELILMEEIRFPRTLSPEAKSLLAGLLKKDPKQRLGGGPSDAKEVMEHRFF...................  78
PF00433.1    ..............................................................................................................  0
AKT2-232     PEVLEDNDYGRAVDWWGLGVVMYEMMCGRLPFYNQDHERLFELILMEEIRFPRTLSPEAKSLLAGLLKKDPKQRLGGGPSDAKEVMEHRFFLSINWQDVVQKKLLPPFKP  284
AKT2-214     ..............................................................................................................  107
AKT2-211     ..............................................................................................................  67
AKT2-219     ..............................................................................................................  157
AKT2-229     ..............................................................................................................  130
AKT2-224     ....................AATQHLCHTLPAVDHSHRE..........DLPRGFSRREGGVDAGHP...........................................  45
AKT2-226     ..................................................XPRTLSPEAKSLLAGLLKKDPKQRLGGGPSDAKEVMEHRFFLSINWQDVVQKKLLPPFKP  60
AKT2-210     ..VLEDNDYGRAVDWWGLGVVMYEMMCGRLPFYNQDHERLFELILMEEIRFPRTLSPEAKSLLAGLLKKDPKQRLGGGPSDAKEVMEHRFFLSINWQDVVQKKLLPPFKP  385
AKT2-212     ..............................................................................................................  184
AKT2-241     ..............................................................................................................  151
AKT2-215     .............DWWGLGVVMYEMMCGRLPFYNQDHERLFELILMEEIRFPRTLSPEAKSLLAGLLKKDPKQRLGGGPSDAKEVMEHRFFLSINWQDVVQKKLLPPFKP  97
AKT2-234     PEVLEDNDYGRAVDWWGLGVVMYEMMCGRLPFYNQDHERLFELILMEEIRFPRTLSPEAKSLLAGLLKKDPKQRLGGGPSDAKEVMEHRFFLSINWQDVVQKKLLPPFKP  366
AKT2-213     ..............................................................................................................  134
AKT2-206     PEVLEDNDYGRAVDWWGLGVVMYEMMCGRLPFYNQDHERLFELILMEEIRFPRTLSPEAKSLLAGLLKKDPKQRLGGGPSDAKEVMEHRFFLSINWQDVVQKKLLPPFKP  428
AKT2-202     ..............................................................................................................  125
AKT2-208     ..............................................................................................................  95
AKT2-223     ..............................................................................................................  51
AKT2-209     ..............................................................................................................  119
AKT2-236     ..............................................................................................................  95
AKT2-240     ....................AATQHLCHTLPAVDHSHRE..........DLPRGFSRREGGVDAGHP...........................................  45
AKT2-207     ..............................................................................................................  132
AKT2-205     ..............................................................................................................  133
AKT2-222     ..........................XGRLPFYNQDHERLFELILMEEIRFPRTLSPEAKSLLAGLLKKDPKQSGLWSRQNH............................  56
AKT2-201     ..VLEDNDYGRAVDWWGLGVVMYEMMCGRLPFYNQDHERLFELILMEEIRFPRTLSPEAKSLLAGLLKKDPKQRLGGGPSDAKEVMEHRFFLSINWQDVVQKKLLPPFKP  385
AKT2-239     ..............................................................................................................  99
AKT2-231     ..............................................................................................................  53
AKT2-242     ..............................................................................................................  129
AKT2-203     ....................AATQHLCHTLPAVDHSHRE..........DLPRGFSRREGGVDAGHP...........................................  45
```

logo

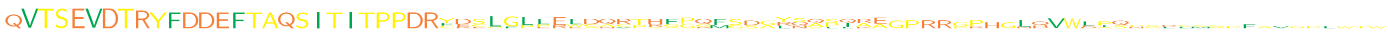

```
A-443654     .............................................................................F........   16
PF00169.2    ......................................................................................   90
PF00433      .VTSEVDTRYFDDEFTAQSITITPPDRYDSLGLLELDQRTHFPQFS..Y.....................................   46
PF00169.1    ......................................................................................  111
PF00069.1    ......................................................................................  256
PF00169      ......................................................................................  101
PF00069.2    ......................................................................................  195
PF00169.3    ......................................................................................   60
PF00169.4    ......................................................................................   94
PF00433.2    .VTSEVDTRYFDDEFTAQSITITPPDRF..........................................................   27
PF00069      ......................................................................................  213
PF00069.3    ......................................................................................   78
PF00433.1    .VTSEVDTRYFDDEFTAQSITITPPDRCECLGPPRWCACPRGGGMGSALN....................................   49
AKT2-232     QVTSEVDT..............................................................................  292
AKT2-214     ......................................................................................  107
AKT2-211     ......................................................................................   67
AKT2-219     ......................................................................................  157
AKT2-229     ......................................................................................  130
AKT2-224     ..............................................DGRQQPQAAGPRRGPHGLQVWLPQ................   69
AKT2-226     QVTSEVDTRYFDDEFTAQSITITPPDRFREGFLEEEANV.SAGR................R..NDVWDASNGRSMA..........  117
AKT2-210     QVTSEVDTRYFDDEFTAQSITITPPDRYDSLGLLELDQRTHFPQFS..YSASIRE...............................  438
AKT2-212     ......................................................................................  184
AKT2-241     ......................................................................................  151
AKT2-215     QVTSEVDTR.............................................................................  106
AKT2-234     QVTSEVDTRYFDDEFTAQSITITPPDRCECLGPPRWCACPRGGGMGSALNSLLKEGPRRPP..LHVWEISPSLLSGPFAVGPLWTW  450
AKT2-213     ......................................................................................  134
AKT2-206     QVTSEVDTRYFDDEFTAQSITITPPDRYDSLGLLELDQRTHFPQFS..YSASIRE...............................  481
AKT2-202     ......................................................................................  125
AKT2-208     ......................................................................................   95
AKT2-223     ......................................................................................   51
AKT2-209     ......................................................................................  119
AKT2-236     ......................................................................................   95
AKT2-240     ..............................................DGRQQPQAAGPRRGPHGLQVWLPQ................   69
AKT2-207     ......................................................................................  132
AKT2-205     ......................................................................................  133
AKT2-222     ......................................................................................   56
AKT2-201     QVTSEVDTRYFDDEFTAQSITITPPDRYDSLGLLELDQRTHFPQFS..YSASIRE...............................  438
AKT2-239     ......................................................................................   99
AKT2-231     ......................................................................................   53
AKT2-242     ......................................................................................  129
AKT2-203     ..............................................DGRQQPQAAGPRRGPHGLQVWLPQ................   69
```

X non conserved
X similar
X $\geq 0\%$ conserved
X $\geq 50\%$ conserved

logo

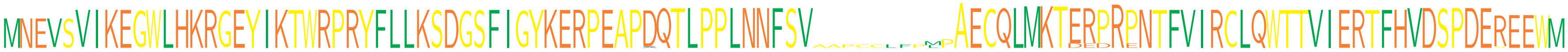

```
CHEMBL428462  ..............................................................................................................    0
PF00169.2     .....VIKEGWLHKRGEYIKTWRPRYFLLKSDGSFIGYKERPEAPDQTLPPLNNFSV..........AECQLMKTERPRPNTFVIRCLQWTTVIERTFHVDSPDE.....   90
PF00433       ..............................................................................................................    0
PF00169.1     .....VIKEGWLHKRGEYIKTWRPRYFLLKSDGSFIGYKERPEAPDQTLPPLNNFSVAAPCCLFPLPAECQLMKTERPRPNTFVIRCLQWTTVIERTFHVDSPDEREEWM  105
PF00069.1     ..............................................................................................................    0
PF00169       .....VIKEGWLHKRGEYIKTWRPRYFLLKSDGSFIGYKERPEAPDQTLPPLNNFSV..........AECQLMKTERPRPNTFVIRCLQWTTVIERTFHVDSPDEREEWM   95
PF00069.2     ..............................................................................................................    0
PF00169.3     .....VIKEGWLHKRGEYIKTWRPRYFLLKSDGSFIGYKERPEAPDQTLPPLNNFSV..........AECQLMKT...................................   60
PF00169.4     .....VIKEGWLHKRGEYIKTWRPRYFLLKSDGSFIGYKERPEAPDQTLPPLNNFSV..........AECQLMKTERPRPNTFVIRCLQWTTVIERTFHVDSPDEREEW.   94
PF00433.2     ..............................................................................................................    0
PF00069       ..............................................................................................................    0
PF00069.3     ..............................................................................................................    0
PF00433.1     ..............................................................................................................    0
AKT2-232      ..............................................................................................................    0
AKT2-214      MNEVSVIKEGWLHKRGEYIKTWRPRYFLLKSDGSFIGYKERPEAPDQTLPPLNNFSV..........AECQLMKTERPRPNTFVIRCLQWTTVIERTFHVDSPDEREEWM  100
AKT2-211      MNEVSVIKEGWLHKRGEYIKTWRPRYFLLKSDGSFIGYKERPEAPDQTLPPLNNFSV..........AECQLMKTER.................................   67
AKT2-219      MNEVSVIKEGWLHKRGEYIKTWRPRYFLLKSDGSFIGYKERPEAPDQTLPPLNNFSV..........AECQLMKTERPRPNTFVIRCLQWTTVIERTFHVDSPDEREEWM  100
AKT2-229      MNEVSVIKEGWLHKRGEYIKTWRPRYFLLKSDGSFIGYKERPEAPDQTLPPLNNFSV..........AECQLMKTERPRPNTFVIRCLQWTTVIERTFHVDSPDEREEWM  100
AKT2-224      .................................................................MPA.......DEDRE..............................    8
AKT2-226      ..............................................................................................................    0
AKT2-210      MNEVSVIKEGWLHKRGEYIKTWRPRYFLLKSDGSFIGYKERPEAPDQTLPPLNNFSV..........AECQLMKTERPRPNTFVIRCLQWTTVIERTFHVDSPDEREEWM  100
AKT2-212      MNEVSVIKEGWLHKRGEYIKTWRPRYFLLKSDGSFIGYKERPEAPDQTLPPLNNFSV..........AECQLMKTERPRPNTFVIRCLQWTTVIERTFHVDSPDEREEWM  100
AKT2-241      MNEVSVIKEGWLHKRGEYIKTWRPRYFLLKSDGSFIGYKERPEAPDQTLPPLNNFSV..........AECQLMKTERPRPNTFVIRCLQWTTVIERTFHVDSPDEREEWM  100
AKT2-215      ..............................................................................................................    0
AKT2-234      ........................................................................MKTERPRPNTFVIRCLQWTTVIERTFHVDSPDEREEWM   38
AKT2-213      MNEVSVIKEGWLHKRGEYIKTWRPRYFLLKSDGSFIGYKERPEAPDQTLPPLNNFSV..........AECQLMKTERPRPNTFVIRCLQWTTVIERTFHVDSPDEREEWM  100
AKT2-206      MNEVSVIKEGWLHKRGEYIKTWRPRYFLLKSDGSFIGYKERPEAPDQTLPPLNNFSV..........AECQLMKTERPRPNTFVIRCLQWTTVIERTFHVDSPDEREEWM  100
AKT2-202      MNEVSVIKEGWLHKRGEYIKTWRPRYFLLKSDGSFIGYKERPEAPDQTLPPLNNFSVAAPCCLFPLPAECQLMKTERPRPNTFVIRCLQWTTVIERTFHVDSPDEREEWM  110
AKT2-208      MNEVSVIKEGWLHKRGEYIKTWRPRYFLLKSDGSFIGYKERPEAPDQTLPPLNNFSV..........AECQLMKTERPRPNTFVIRCLQWTTVIERTFHVDSPDE.....   95
AKT2-223      .............................................XQTLPPLNNFSV..........AECQLMKTERPRPNTFVIRCLQWTTVIERTFHVDSPDES....   51
AKT2-209      MNEVSVIKEGWLHKRGEYIKTWRPRYFLLKSDGSFIGYKERPEAPDQTLPPLNNFSV..........AECQLMKTERPRPNTFVIRCLQWTTVIERTFHVDSPDEREEWM  100
AKT2-236      MNEVSVIKEGWLHKRGEYIKTWRPRYFLLKSDGSFIGYKERPEAPDQTLPPLNNFSV..........AECQLMKTERPRPNTFVIRCLQWTTVIERTFHVDSPDE.....   95
AKT2-240      .................................................................MPA.......DEDRE..............................    8
AKT2-207      MNEVSVIKEGWLHKRGEYIKTWRPRYFLLKSDGSFIGYKERPEAPDQTLPPLNNFSV..........AECQLMKTERPRPNTFVIRCLQWTTVIERTFHVDSPDEREEWM  100
AKT2-205      MNEVSVIKEGWLHKRGEYIKTWRPRYFLLKSDGSFIGYKERPEAPDQTLPPLNNFSV..........AECQLMKTERPRPNTFVIRCLQWTTVIERTFHVDSPDEREEWM  100
AKT2-222      ..............................................................................................................    0
AKT2-201      MNEVSVIKEGWLHKRGEYIKTWRPRYFLLKSDGSFIGYKERPEAPDQTLPPLNNFSV..........AECQLMKTERPRPNTFVIRCLQWTTVIERTFHVDSPDEREEWM  100
AKT2-239      MNEVSVIKEGWLHKRGEYIKTWRPRYFLLKSDGSFIGYKERPEAPDQTLPPLNNFSV..........AECQLMKTERPRPNTFVIRCLQWTTVIERTFHVDSPDEREEW.   99
AKT2-231      ..............................................................................................................    0
AKT2-242      ...................................................................................................VDSPDEREEWM   11
AKT2-203      .................................................................MPA.......DEDRE..............................    8
```

```
logo
              RAIQMVANSLKQRAPGEDPMDYKCGSPSDSSTTEEMEVAVSKARAKVTMNDFDYLKLLGKGTFGKVILVREKATGRYYAMKILRKEVIIAKDEVAHTVT.ESRVLQNTRH
CHEMBL428462  ..........................................................GKG..GKV............A.K.L...........................   9
PF00169.2     ..............................................................................................................  90
PF00433       ..............................................................................................................   0
PF00169.1     RAIQMV........................................................................................................ 111
PF00069.1     .....................................................YLKLLGKGTFGKVILVREKATGRYYAMKILRKEVIIAKDEVAHTVT.ESRVLQNTRH  56
PF00169       RAIQMV........................................................................................................ 101
PF00069.2     ..............................................................................................................   0
PF00169.3     ..............................................................................................................  60
PF00169.4     ..............................................................................................................  94
PF00433.2     ..............................................................................................................   0
PF00069       .....................................................YLKLLGKGTFGKVILVREKATGRYYAMKILRKEVIIAKDEVAHTVT.ESRVLQNTRH  56
PF00069.3     ..............................................................................................................   0
PF00433.1     ..............................................................................................................   0
AKT2-232      ......................KCGSPSDSSTTEEMEVAVSKARAKVTMNDFDYLKLLGKGTFGKVILVREKATGRYYAMKILRKEVIIAK...................  69
AKT2-214      RAIQMVA....................................................................................................... 107
AKT2-211      ..............................................................................................................  67
AKT2-219      RAIQMVANSLKQRAPGEDPMDYKCGSPSDSSTTEEMEVAVSKARAKVTMNDFDYLKL..................................................... 157
AKT2-229      RAIQMVANSLKQRAPGEDPMDYKCGSPSDS................................................................................ 130
AKT2-224      ..............................................................................................................   8
AKT2-226      ..............................................................................................................   0
AKT2-210      RAIQMVANSLKQRAPGEDPMDYKCGSPSDSSTTEEMEVAVSKARAKVTMNDFDYLKLLGKGTFGKVILVREKATGRYYAMKILRKEVIIAKDEVAHTVT.ESRVLQNTRH 209
AKT2-212      RAIQMVANSLKQRAPGEDPMDYKCGSPSDSSTTEEMEVAVSKARAKVTMNDFDYLKLLGKGTFGKVILVREKATGRYYAMKILR.......................... 184
AKT2-241      RAIQMVANSLKQRAPGEDPMDYKCGSPSDSSTTEEMEVAVSKARAKVTMND........................................................... 151
AKT2-215      ..............................................................................................................   0
AKT2-234      RAIQMVANSLKQRAPGEDPMDYKCGSPSDSSTTEEMEVAVSKARAKVTMNDFDYLKLLGKGTFGKVILVREKATGRYYAMKILRKEVIIAKDEVAHTVT.ESRVLQNTRH 147
AKT2-213      RAIQMVANSLKQRAPGEDPMDYKCGSPSDSSTTE............................................................................ 134
AKT2-206      RAIQMVANSLKQRAPGEDPMDYKCGSPSDSSTTEEMEVAVSKARAKVTMNDFDYLKLLGKGTFGKVILVREKATGRYYAMKILRKEVIIAKDEVAHTVT.ESRVLQNTRH 209
AKT2-202      RAIQMVANSLKQRAP............................................................................................... 125
AKT2-208      ..............................................................................................................  95
AKT2-223      ..............................................................................................................  51
AKT2-209      RAIQMVANSLKQRAPGEDP........................................................................................... 119
AKT2-236      ..............................................................................................................  95
AKT2-240      ..............................................................................................................   8
AKT2-207      RAIQMVANSLKQRAPGEDPMDYKCGSPSDSST.............................................................................. 132
AKT2-205      RAIQMVANSLKQRAPGEDPMDYKCGSPSDSSTT............................................................................. 133
AKT2-222      ..............................................................................................................   0
AKT2-201      RAIQMVANSLKQRAPGEDPMDYKCGSPSDSSTTEEMEVAVSKARAKVTMNDFDYLKLLGKGTFGKVILVREKATGRYYAMKILRKEVIIAKDEVAHTVT.ESRVLQNTRH 209
AKT2-239      ..............................................................................................................  99
AKT2-231      ..................................................................XLVREKATGRYYAMKILRKEVIIA....................  24
AKT2-242      RAIQMVANSLKQRAPGEDPMDYKCGSPSDSSTTEEMEVAVSKARAKVTMNDFDYLKLLGKGTFGKVILVREKATGRYYAMKILRKEVIIAKSVCRMKSLTQSPRAGSSRT 121
AKT2-203      ..............................................................................................................   8
```

```
logo
              PFL.TALKYAFQTHDRLCFVMEYANGGELFFHLSRERVFTEERARFYGAEIVSALEYLHSRDVVYRDIKLENLMLDKDGHIKITDFGLCKEGISDGATMKTFCGTPEYLA
CHEMBL428462  ....................MEYA...E..........................................E..M.........TD.........................  18
PF00169.2     ..............................................................................................................  90
PF00433       ..............................................................................................................  0
PF00169.1     ..............................................................................................................  111
PF00069.1     PFL.TALKYAFQTHDRLCFVMEYANGGELFFHLSRERVFTEERARFYGAEIVSALEYLHSRDVVYRDIKLENLMLDKDGHIKITDFGLCKEGISDGATMKTFCGTPEYLA  165
PF00169       ..............................................................................................................  101
PF00069.2     ......LKYAFQTHDRLCFVMEYANGGELFFHLSRERVFTEERARFYGAEIVSALEYLHSRDVVYRDIKLENLMLDKDGHIKITDFGLCKEGISDGATMKTFCGTPEYLA  104
PF00169.3     ..............................................................................................................  60
PF00169.4     ..............................................................................................................  94
PF00433.2     ..............................................................................................................  0
PF00069       PFL.TALKYAFQTHDRLCFVMEYANGGELFFHLSRERVFTEERARFYGAEIVSALEYLHSRDVVYRDIK.........................................  124
PF00069.3     ..............................................................................................................  0
PF00433.1     ..............................................................................................................  0
AKT2-232      .....ALKYAFQTHDRLCFVMEYANGGELFFHLSRERVFTEERARFYGAEIVSALEYLHSRDVVYRDIKLENLMLDKDGHIKITDFGLCKEGISDGATMKTFCGTPEYLA  174
AKT2-214      ..............................................................................................................  107
AKT2-211      ..............................................................................................................  67
AKT2-219      ..............................................................................................................  157
AKT2-229      ..............................................................................................................  130
AKT2-224      ..............................................................................................................  8
AKT2-226      ..............................................................................................................  0
AKT2-210      PFL.TALKYAFQTHDRLCFVMEYANGGELFFHLSRERVFTEERARFYGAEIVSALEYLHSRDVVYRDIK.........................................  277
AKT2-212      ..............................................................................................................  184
AKT2-241      ..............................................................................................................  151
AKT2-215      ..............................................................................................................  0
AKT2-234      PFL.TALKYAFQTHDRLCFVMEYANGGELFFHLSRERVFTEERARFYGAEIVSALEYLHSRDVVYRDIKLENLMLDKDGHIKITDFGLCKEGISDGATMKTFCGTPEYLA  256
AKT2-213      ..............................................................................................................  134
AKT2-206      PFL.TALKYAFQTHDRLCFVMEYANGGELFFHLSRERVFTEERARFYGAEIVSALEYLHSRDVVYRDIKLENLMLDKDGHIKITDFGLCKEGISDGATMKTFCGTPEYLA  318
AKT2-202      ..............................................................................................................  125
AKT2-208      ..............................................................................................................  95
AKT2-223      ..............................................................................................................  51
AKT2-209      ..............................................................................................................  119
AKT2-236      ..............................................................................................................  95
AKT2-240      ..............................................................................................................  8
AKT2-207      ..............................................................................................................  132
AKT2-205      ..............................................................................................................  133
AKT2-222      ..............................................................................................................  0
AKT2-201      PFL.TALKYAFQTHDRLCFVMEYANGGELFFHLSRERVFTEERARFYGAEIVSALEYLHSRDVVYRDIK.........................................  277
AKT2-239      ..............................................................................................................  99
AKT2-231      ....................................................................KLENLMLDKDGHIKITDFGLCKEGISDGA.............  53
AKT2-242      PGTRSSLR......................................................................................................  129
AKT2-203      ..............................................................................................................  8
```

logo

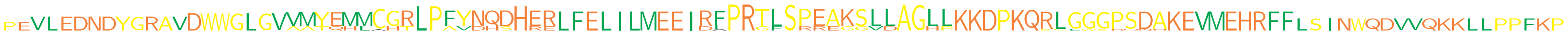

```
CHEMBL428462  .............................................................................................................. 18
PF00169.2     .............................................................................................................. 90
PF00433       .............................................................................................................. 0
PF00169.1     .............................................................................................................. 111
PF00069.1     PEVLEDNDYGRAVDWWGLGVVMYEMMCGRLPFYNQDHERLFELILMEEIRFPRTLSPEAKSLLAGLLKKDPKQRLGGGPSDAKEVMEHRFF................... 256
PF00169       .............................................................................................................. 101
PF00069.2     PEVLEDNDYGRAVDWWGLGVVMYEMMCGRLPFYNQDHERLFELILMEEIRFPRTLSPEAKSLLAGLLKKDPKQRLGGGPSDAKEVMEHRFF................... 195
PF00169.3     .............................................................................................................. 60
PF00169.4     .............................................................................................................. 94
PF00433.2     .............................................................................................................. 0
PF00069       ..VLEDNDYGRAVDWWGLGVVMYEMMCGRLPFYNQDHERLFELILMEEIRFPRTLSPEAKSLLAGLLKKDPKQRLGGGPSDAKEVMEHRFF................... 213
PF00069.3     ..............DWWGLGVVMYEMMCGRLPFYNQDHERLFELILMEEIRFPRTLSPEAKSLLAGLLKKDPKQRLGGGPSDAKEVMEHRFF................... 78
PF00433.1     .............................................................................................................. 0
AKT2-232      PEVLEDNDYGRAVDWWGLGVVMYEMMCGRLPFYNQDHERLFELILMEEIRFPRTLSPEAKSLLAGLLKKDPKQRLGGGPSDAKEVMEHRFFLSINWQDVVQKKLLPPFKP 284
AKT2-214      .............................................................................................................. 107
AKT2-211      .............................................................................................................. 67
AKT2-219      .............................................................................................................. 157
AKT2-229      .............................................................................................................. 130
AKT2-224      ....................AATQHLCHTLPAVDHSHRE..........DLPRGFSRREGGVDAGHP........................................... 45
AKT2-226      ..................................................XPRTLSPEAKSLLAGLLKKDPKQRLGGGPSDAKEVMEHRFFLSINWQDVVQKKLLPPFKP 60
AKT2-210      ..VLEDNDYGRAVDWWGLGVVMYEMMCGRLPFYNQDHERLFELILMEEIRFPRTLSPEAKSLLAGLLKKDPKQRLGGGPSDAKEVMEHRFFLSINWQDVVQKKLLPPFKP 385
AKT2-212      .............................................................................................................. 184
AKT2-241      .............................................................................................................. 151
AKT2-215      ..............DWWGLGVVMYEMMCGRLPFYNQDHERLFELILMEEIRFPRTLSPEAKSLLAGLLKKDPKQRLGGGPSDAKEVMEHRFFLSINWQDVVQKKLLPPFKP 97
AKT2-234      PEVLEDNDYGRAVDWWGLGVVMYEMMCGRLPFYNQDHERLFELILMEEIRFPRTLSPEAKSLLAGLLKKDPKQRLGGGPSDAKEVMEHRFFLSINWQDVVQKKLLPPFKP 366
AKT2-213      .............................................................................................................. 134
AKT2-206      PEVLEDNDYGRAVDWWGLGVVMYEMMCGRLPFYNQDHERLFELILMEEIRFPRTLSPEAKSLLAGLLKKDPKQRLGGGPSDAKEVMEHRFFLSINWQDVVQKKLLPPFKP 428
AKT2-202      .............................................................................................................. 125
AKT2-208      .............................................................................................................. 95
AKT2-223      .............................................................................................................. 51
AKT2-209      .............................................................................................................. 119
AKT2-236      .............................................................................................................. 95
AKT2-240      ....................AATQHLCHTLPAVDHSHRE..........DLPRGFSRREGGVDAGHP........................................... 45
AKT2-207      .............................................................................................................. 132
AKT2-205      .............................................................................................................. 133
AKT2-222      ..........................XGRLPFYNQDHERLFELILMEEIRFPRTLSPEAKSLLAGLLKKDPKQSGLWSRQNH............................ 56
AKT2-201      ..VLEDNDYGRAVDWWGLGVVMYEMMCGRLPFYNQDHERLFELILMEEIRFPRTLSPEAKSLLAGLLKKDPKQRLGGGPSDAKEVMEHRFFLSINWQDVVQKKLLPPFKP 385
AKT2-239      .............................................................................................................. 99
AKT2-231      .............................................................................................................. 53
AKT2-242      .............................................................................................................. 129
AKT2-203      ....................AATQHLCHTLPAVDHSHRE..........DLPRGFSRREGGVDAGHP........................................... 45
```

logo

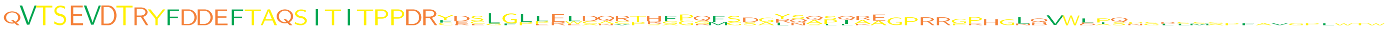

```
CHEMBL428462  ......................................................................................   18
PF00169.2     ......................................................................................   90
PF00433       .VTSEVDTRYFDDEFTAQSITITPPDRYDSLGLLELDQRTHFPQFS..Y.....................................   46
PF00169.1     ......................................................................................  111
PF00069.1     ......................................................................................  256
PF00169       ......................................................................................  101
PF00069.2     ......................................................................................  195
PF00169.3     ......................................................................................   60
PF00169.4     ......................................................................................   94
PF00433.2     .VTSEVDTRYFDDEFTAQSITITPPDRF..........................................................   27
PF00069       ......................................................................................  213
PF00069.3     ......................................................................................   78
PF00433.1     .VTSEVDTRYFDDEFTAQSITITPPDRCECLGPPRWCACPRGGGMGSALN....................................   49
AKT2-232      QVTSEVDT..............................................................................  292
AKT2-214      ......................................................................................  107
AKT2-211      ......................................................................................   67
AKT2-219      ......................................................................................  157
AKT2-229      ......................................................................................  130
AKT2-224      ..............................................DGRQQPQAAGPRRGPHGLQVWLPQ................   69
AKT2-226      QVTSEVDTRYFDDEFTAQSITITPPDRFREGFLEEEANV.SAGR................R..NDVWDASNGRSMA..........  117
AKT2-210      QVTSEVDTRYFDDEFTAQSITITPPDRYDSLGLLELDQRTHFPQFS..YSASIRE...............................  438
AKT2-212      ......................................................................................  184
AKT2-241      ......................................................................................  151
AKT2-215      QVTSEVDTR.............................................................................  106
AKT2-234      QVTSEVDTRYFDDEFTAQSITITPPDRCECLGPPRWCACPRGGGMGSALNSLLKEGPRRPP..LHVWEISPSLLSGPFAVGPLWTW  450
AKT2-213      ......................................................................................  134
AKT2-206      QVTSEVDTRYFDDEFTAQSITITPPDRYDSLGLLELDQRTHFPQFS..YSASIRE...............................  481
AKT2-202      ......................................................................................  125
AKT2-208      ......................................................................................   95
AKT2-223      ......................................................................................   51
AKT2-209      ......................................................................................  119
AKT2-236      ......................................................................................   95
AKT2-240      ..............................................DGRQQPQAAGPRRGPHGLQVWLPQ................   69
AKT2-207      ......................................................................................  132
AKT2-205      ......................................................................................  133
AKT2-222      ......................................................................................   56
AKT2-201      QVTSEVDTRYFDDEFTAQSITITPPDRYDSLGLLELDQRTHFPQFS..YSASIRE...............................  438
AKT2-239      ......................................................................................   99
AKT2-231      ......................................................................................   53
AKT2-242      ......................................................................................  129
AKT2-203      ..............................................DGRQQPQAAGPRRGPHGLQVWLPQ................   69
```

X non conserved
X similar
X ≥ 0% conserved
X ≥ 50% conserved

```
logo
GSK-690693  ..............................................................................................................   0
PF00169.2   .....VIKEGWLHKRGEYIKTWRPRYFLLKSDGSFIGYKERPEAPDQTLPPLNNFSV..........AECQLMKTERPRPNTFVIRCLQWTTVIERTFHVDSPDE.....  90
PF00433     ..............................................................................................................   0
PF00169.1   .....VIKEGWLHKRGEYIKTWRPRYFLLKSDGSFIGYKERPEAPDQTLPPLNNFSVAAPCCLFPLPAECQLMKTERPRPNTFVIRCLQWTTVIERTFHVDSPDEREEWM 105
PF00069.1   ..............................................................................................................   0
PF00169     .....VIKEGWLHKRGEYIKTWRPRYFLLKSDGSFIGYKERPEAPDQTLPPLNNFSV..........AECQLMKTERPRPNTFVIRCLQWTTVIERTFHVDSPDEREEWM  95
PF00069.2   ..............................................................................................................   0
PF00169.3   .....VIKEGWLHKRGEYIKTWRPRYFLLKSDGSFIGYKERPEAPDQTLPPLNNFSV..........AECQLMKT...................................  60
PF00169.4   .....VIKEGWLHKRGEYIKTWRPRYFLLKSDGSFIGYKERPEAPDQTLPPLNNFSV..........AECQLMKTERPRPNTFVIRCLQWTTVIERTFHVDSPDEREEW.  94
PF00433.2   ..............................................................................................................   0
PF00069     ..............................................................................................................   0
PF00069.3   ..............................................................................................................   0
PF00433.1   ..............................................................................................................   0
AKT2-232    ..............................................................................................................   0
AKT2-214    MNEVSVIKEGWLHKRGEYIKTWRPRYFLLKSDGSFIGYKERPEAPDQTLPPLNNFSV..........AECQLMKTERPRPNTFVIRCLQWTTVIERTFHVDSPDEREEWM 100
AKT2-211    MNEVSVIKEGWLHKRGEYIKTWRPRYFLLKSDGSFIGYKERPEAPDQTLPPLNNFSV..........AECQLMKTER.................................  67
AKT2-219    MNEVSVIKEGWLHKRGEYIKTWRPRYFLLKSDGSFIGYKERPEAPDQTLPPLNNFSV..........AECQLMKTERPRPNTFVIRCLQWTTVIERTFHVDSPDEREEWM 100
AKT2-229    MNEVSVIKEGWLHKRGEYIKTWRPRYFLLKSDGSFIGYKERPEAPDQTLPPLNNFSV..........AECQLMKTERPRPNTFVIRCLQWTTVIERTFHVDSPDEREEWM 100
AKT2-224    ................................................................MPA.......DEDRE...............................   8
AKT2-226    ..............................................................................................................   0
AKT2-210    MNEVSVIKEGWLHKRGEYIKTWRPRYFLLKSDGSFIGYKERPEAPDQTLPPLNNFSV..........AECQLMKTERPRPNTFVIRCLQWTTVIERTFHVDSPDEREEWM 100
AKT2-212    MNEVSVIKEGWLHKRGEYIKTWRPRYFLLKSDGSFIGYKERPEAPDQTLPPLNNFSV..........AECQLMKTERPRPNTFVIRCLQWTTVIERTFHVDSPDEREEWM 100
AKT2-241    MNEVSVIKEGWLHKRGEYIKTWRPRYFLLKSDGSFIGYKERPEAPDQTLPPLNNFSV..........AECQLMKTERPRPNTFVIRCLQWTTVIERTFHVDSPDEREEWM 100
AKT2-215    ..............................................................................................................   0
AKT2-234    ........................................................................MKTERPRPNTFVIRCLQWTTVIERTFHVDSPDEREEWM  38
AKT2-213    MNEVSVIKEGWLHKRGEYIKTWRPRYFLLKSDGSFIGYKERPEAPDQTLPPLNNFSV..........AECQLMKTERPRPNTFVIRCLQWTTVIERTFHVDSPDEREEWM 100
AKT2-206    MNEVSVIKEGWLHKRGEYIKTWRPRYFLLKSDGSFIGYKERPEAPDQTLPPLNNFSV..........AECQLMKTERPRPNTFVIRCLQWTTVIERTFHVDSPDEREEWM 100
AKT2-202    MNEVSVIKEGWLHKRGEYIKTWRPRYFLLKSDGSFIGYKERPEAPDQTLPPLNNFSVAAPCCLFPLPAECQLMKTERPRPNTFVIRCLQWTTVIERTFHVDSPDEREEWM 110
AKT2-208    MNEVSVIKEGWLHKRGEYIKTWRPRYFLLKSDGSFIGYKERPEAPDQTLPPLNNFSV..........AECQLMKTERPRPNTFVIRCLQWTTVIERTFHVDSPDE.....  95
AKT2-223    .............................................XQTLPPLNNFSV..........AECQLMKTERPRPNTFVIRCLQWTTVIERTFHVDSPDES....  51
AKT2-209    MNEVSVIKEGWLHKRGEYIKTWRPRYFLLKSDGSFIGYKERPEAPDQTLPPLNNFSV..........AECQLMKTERPRPNTFVIRCLQWTTVIERTFHVDSPDEREEWM 100
AKT2-236    MNEVSVIKEGWLHKRGEYIKTWRPRYFLLKSDGSFIGYKERPEAPDQTLPPLNNFSV..........AECQLMKTERPRPNTFVIRCLQWTTVIERTFHVDSPDE.....  95
AKT2-240    ................................................................MPA.......DEDRE...............................   8
AKT2-207    MNEVSVIKEGWLHKRGEYIKTWRPRYFLLKSDGSFIGYKERPEAPDQTLPPLNNFSV..........AECQLMKTERPRPNTFVIRCLQWTTVIERTFHVDSPDEREEWM 100
AKT2-205    MNEVSVIKEGWLHKRGEYIKTWRPRYFLLKSDGSFIGYKERPEAPDQTLPPLNNFSV..........AECQLMKTERPRPNTFVIRCLQWTTVIERTFHVDSPDEREEWM 100
AKT2-222    ..............................................................................................................   0
AKT2-201    MNEVSVIKEGWLHKRGEYIKTWRPRYFLLKSDGSFIGYKERPEAPDQTLPPLNNFSV..........AECQLMKTERPRPNTFVIRCLQWTTVIERTFHVDSPDEREEWM 100
AKT2-239    MNEVSVIKEGWLHKRGEYIKTWRPRYFLLKSDGSFIGYKERPEAPDQTLPPLNNFSV..........AECQLMKTERPRPNTFVIRCLQWTTVIERTFHVDSPDEREEW.  99
AKT2-231    ..............................................................................................................   0
AKT2-242    ...................................................................................................VDSPDEREEWM  11
AKT2-203    ................................................................MPA.......DEDRE...............................   8
```
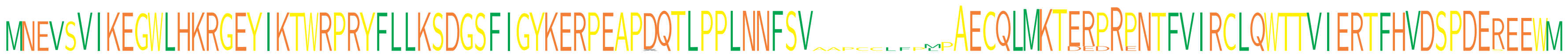

logo

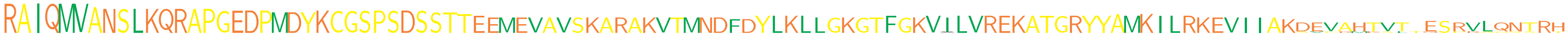

```
GSK-690693  ..............................................................F..V............A.K...................E...L.....    6
PF00169.2   ..............................................................................................................   90
PF00433     ..............................................................................................................    0
PF00169.1   RAIQMV........................................................................................................  111
PF00069.1   .....................................................YLKLLGKGTFGKVILVREKATGRYYAMKILRKEVIIAKDEVAHTVT.ESRVLQNTRH   56
PF00169     RAIQMV........................................................................................................  101
PF00069.2   ..............................................................................................................    0
PF00169.3   ..............................................................................................................   60
PF00169.4   ..............................................................................................................   94
PF00433.2   ..............................................................................................................    0
PF00069     .....................................................YLKLLGKGTFGKVILVREKATGRYYAMKILRKEVIIAKDEVAHTVT.ESRVLQNTRH   56
PF00069.3   ..............................................................................................................    0
PF00433.1   ..............................................................................................................    0
AKT2-232    ......................KCGSPSDSSTTEEMEVAVSKARAKVTMNDFDYLKLLGKGTFGKVILVREKATGRYYAMKILRKEVIIAK...................   69
AKT2-214    RAIQMVA.......................................................................................................  107
AKT2-211    ..............................................................................................................   67
AKT2-219    RAIQMVANSLKQRAPGEDPMDYKCGSPSDSSTTEEMEVAVSKARAKVTMNDFDYLKL.....................................................  157
AKT2-229    RAIQMVANSLKQRAPGEDPMDYKCGSPSDS................................................................................  130
AKT2-224    ..............................................................................................................    8
AKT2-226    ..............................................................................................................    0
AKT2-210    RAIQMVANSLKQRAPGEDPMDYKCGSPSDSSTTEEMEVAVSKARAKVTMNDFDYLKLLGKGTFGKVILVREKATGRYYAMKILRKEVIIAKDEVAHTVT.ESRVLQNTRH  209
AKT2-212    RAIQMVANSLKQRAPGEDPMDYKCGSPSDSSTTEEMEVAVSKARAKVTMNDFDYLKLLGKGTFGKVILVREKATGRYYAMKILR..........................  184
AKT2-241    RAIQMVANSLKQRAPGEDPMDYKCGSPSDSSTTEEMEVAVSKARAKVTMND...........................................................  151
AKT2-215    ..............................................................................................................    0
AKT2-234    RAIQMVANSLKQRAPGEDPMDYKCGSPSDSSTTEEMEVAVSKARAKVTMNDFDYLKLLGKGTFGKVILVREKATGRYYAMKILRKEVIIAKDEVAHTVT.ESRVLQNTRH  147
AKT2-213    RAIQMVANSLKQRAPGEDPMDYKCGSPSDSSTTE............................................................................  134
AKT2-206    RAIQMVANSLKQRAPGEDPMDYKCGSPSDSSTTEEMEVAVSKARAKVTMNDFDYLKLLGKGTFGKVILVREKATGRYYAMKILRKEVIIAKDEVAHTVT.ESRVLQNTRH  209
AKT2-202    RAIQMVANSLKQRAP...............................................................................................  125
AKT2-208    ..............................................................................................................   95
AKT2-223    ..............................................................................................................   51
AKT2-209    RAIQMVANSLKQRAPGEDP...........................................................................................  119
AKT2-236    ..............................................................................................................   95
AKT2-240    ..............................................................................................................    8
AKT2-207    RAIQMVANSLKQRAPGEDPMDYKCGSPSDSST..............................................................................  132
AKT2-205    RAIQMVANSLKQRAPGEDPMDYKCGSPSDSSTT.............................................................................  133
AKT2-222    ..............................................................................................................    0
AKT2-201    RAIQMVANSLKQRAPGEDPMDYKCGSPSDSSTTEEMEVAVSKARAKVTMNDFDYLKLLGKGTFGKVILVREKATGRYYAMKILRKEVIIAKDEVAHTVT.ESRVLQNTRH  209
AKT2-239    ..............................................................................................................   99
AKT2-231    ..................................................................XLVREKATGRYYAMKILRKEVIIA....................   24
AKT2-242    RAIQMVANSLKQRAPGEDPMDYKCGSPSDSSTTEEMEVAVSKARAKVTMNDFDYLKLLGKGTFGKVILVREKATGRYYAMKILRKEVIIAKSVCRMKSLTQSPRAGSSRT  121
AKT2-203    ..............................................................................................................    8
```

```
logo
               PFL.TALKYAFQTHDRLCFVMEYANGGELFFHLSRERVFTEERARFYGAEIVSALEYLHSRDVVYRDIKLENLMLDKDGHIKITDFGLCKEGISDGATMKTFCGTPEYLA
GSK-690693     ..................F.MEYA...E..........................................E..M..........DF........................   16
PF00169.2      ..............................................................................................................   90
PF00433        ..............................................................................................................    0
PF00169.1      ..............................................................................................................  111
PF00069.1      PFL.TALKYAFQTHDRLCFVMEYANGGELFFHLSRERVFTEERARFYGAEIVSALEYLHSRDVVYRDIKLENLMLDKDGHIKITDFGLCKEGISDGATMKTFCGTPEYLA  165
PF00169        ..............................................................................................................  101
PF00069.2      ......LKYAFQTHDRLCFVMEYANGGELFFHLSRERVFTEERARFYGAEIVSALEYLHSRDVVYRDIKLENLMLDKDGHIKITDFGLCKEGISDGATMKTFCGTPEYLA  104
PF00169.3      ..............................................................................................................   60
PF00169.4      ..............................................................................................................   94
PF00433.2      ..............................................................................................................    0
PF00069        PFL.TALKYAFQTHDRLCFVMEYANGGELFFHLSRERVFTEERARFYGAEIVSALEYLHSRDVVYRDIK.........................................  124
PF00069.3      ..............................................................................................................    0
PF00433.1      ..............................................................................................................    0
AKT2-232       .....ALKYAFQTHDRLCFVMEYANGGELFFHLSRERVFTEERARFYGAEIVSALEYLHSRDVVYRDIKLENLMLDKDGHIKITDFGLCKEGISDGATMKTFCGTPEYLA  174
AKT2-214       ..............................................................................................................  107
AKT2-211       ..............................................................................................................   67
AKT2-219       ..............................................................................................................  157
AKT2-229       ..............................................................................................................  130
AKT2-224       ..............................................................................................................    8
AKT2-226       ..............................................................................................................    0
AKT2-210       PFL.TALKYAFQTHDRLCFVMEYANGGELFFHLSRERVFTEERARFYGAEIVSALEYLHSRDVVYRDIK.........................................  277
AKT2-212       ..............................................................................................................  184
AKT2-241       ..............................................................................................................  151
AKT2-215       ..............................................................................................................    0
AKT2-234       PFL.TALKYAFQTHDRLCFVMEYANGGELFFHLSRERVFTEERARFYGAEIVSALEYLHSRDVVYRDIKLENLMLDKDGHIKITDFGLCKEGISDGATMKTFCGTPEYLA  256
AKT2-213       ..............................................................................................................  134
AKT2-206       PFL.TALKYAFQTHDRLCFVMEYANGGELFFHLSRERVFTEERARFYGAEIVSALEYLHSRDVVYRDIKLENLMLDKDGHIKITDFGLCKEGISDGATMKTFCGTPEYLA  318
AKT2-202       ..............................................................................................................  125
AKT2-208       ..............................................................................................................   95
AKT2-223       ..............................................................................................................   51
AKT2-209       ..............................................................................................................  119
AKT2-236       ..............................................................................................................   95
AKT2-240       ..............................................................................................................    8
AKT2-207       ..............................................................................................................  132
AKT2-205       ..............................................................................................................  133
AKT2-222       ..............................................................................................................    0
AKT2-201       PFL.TALKYAFQTHDRLCFVMEYANGGELFFHLSRERVFTEERARFYGAEIVSALEYLHSRDVVYRDIK.........................................  277
AKT2-239       ..............................................................................................................   99
AKT2-231       ....................................................................KLENLMLDKDGHIKITDFGLCKEGISDGA.............   53
AKT2-242       PGTRSSLR......................................................................................................  129
AKT2-203       ..............................................................................................................    8
```

logo

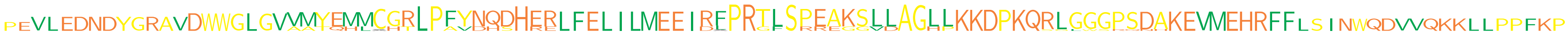

```
GSK-690693   ..............................................................................................................  16
PF00169.2    ..............................................................................................................  90
PF00433      ..............................................................................................................   0
PF00169.1    .............................................................................................................. 111
PF00069.1    PEVLEDNDYGRAVDWWGLGVVMYEMMCGRLPFYNQDHERLFELILMEEIRFPRTLSPEAKSLLAGLLKKDPKQRLGGGPSDAKEVMEHRFF................... 256
PF00169      .............................................................................................................. 101
PF00069.2    PEVLEDNDYGRAVDWWGLGVVMYEMMCGRLPFYNQDHERLFELILMEEIRFPRTLSPEAKSLLAGLLKKDPKQRLGGGPSDAKEVMEHRFF................... 195
PF00169.3    ..............................................................................................................  60
PF00169.4    ..............................................................................................................  94
PF00433.2    ..............................................................................................................   0
PF00069      ..VLEDNDYGRAVDWWGLGVVMYEMMCGRLPFYNQDHERLFELILMEEIRFPRTLSPEAKSLLAGLLKKDPKQRLGGGPSDAKEVMEHRFF................... 213
PF00069.3    .............DWWGLGVVMYEMMCGRLPFYNQDHERLFELILMEEIRFPRTLSPEAKSLLAGLLKKDPKQRLGGGPSDAKEVMEHRFF...................  78
PF00433.1    ..............................................................................................................   0
AKT2-232     PEVLEDNDYGRAVDWWGLGVVMYEMMCGRLPFYNQDHERLFELILMEEIRFPRTLSPEAKSLLAGLLKKDPKQRLGGGPSDAKEVMEHRFFLSINWQDVVQKKLLPPFKP 284
AKT2-214     .............................................................................................................. 107
AKT2-211     ..............................................................................................................  67
AKT2-219     .............................................................................................................. 157
AKT2-229     .............................................................................................................. 130
AKT2-224     ....................AATQHLCHTLPAVDHSHRE..........DLPRGFSRREGGVDAGHP...........................................  45
AKT2-226     ..................................................XPRTLSPEAKSLLAGLLKKDPKQRLGGGPSDAKEVMEHRFFLSINWQDVVQKKLLPPFKP  60
AKT2-210     ..VLEDNDYGRAVDWWGLGVVMYEMMCGRLPFYNQDHERLFELILMEEIRFPRTLSPEAKSLLAGLLKKDPKQRLGGGPSDAKEVMEHRFFLSINWQDVVQKKLLPPFKP 385
AKT2-212     .............................................................................................................. 184
AKT2-241     .............................................................................................................. 151
AKT2-215     .............DWWGLGVVMYEMMCGRLPFYNQDHERLFELILMEEIRFPRTLSPEAKSLLAGLLKKDPKQRLGGGPSDAKEVMEHRFFLSINWQDVVQKKLLPPFKP  97
AKT2-234     PEVLEDNDYGRAVDWWGLGVVMYEMMCGRLPFYNQDHERLFELILMEEIRFPRTLSPEAKSLLAGLLKKDPKQRLGGGPSDAKEVMEHRFFLSINWQDVVQKKLLPPFKP 366
AKT2-213     .............................................................................................................. 134
AKT2-206     PEVLEDNDYGRAVDWWGLGVVMYEMMCGRLPFYNQDHERLFELILMEEIRFPRTLSPEAKSLLAGLLKKDPKQRLGGGPSDAKEVMEHRFFLSINWQDVVQKKLLPPFKP 428
AKT2-202     .............................................................................................................. 125
AKT2-208     ..............................................................................................................  95
AKT2-223     ..............................................................................................................  51
AKT2-209     .............................................................................................................. 119
AKT2-236     ..............................................................................................................  95
AKT2-240     ....................AATQHLCHTLPAVDHSHRE..........DLPRGFSRREGGVDAGHP...........................................  45
AKT2-207     .............................................................................................................. 132
AKT2-205     .............................................................................................................. 133
AKT2-222     ..........................XGRLPFYNQDHERLFELILMEEIRFPRTLSPEAKSLLAGLLKKDPKQSGLWSRQNH............................  56
AKT2-201     ..VLEDNDYGRAVDWWGLGVVMYEMMCGRLPFYNQDHERLFELILMEEIRFPRTLSPEAKSLLAGLLKKDPKQRLGGGPSDAKEVMEHRFFLSINWQDVVQKKLLPPFKP 385
AKT2-239     ..............................................................................................................  99
AKT2-231     ..............................................................................................................  53
AKT2-242     .............................................................................................................. 129
AKT2-203     ....................AATQHLCHTLPAVDHSHRE..........DLPRGFSRREGGVDAGHP...........................................  45
```

logo

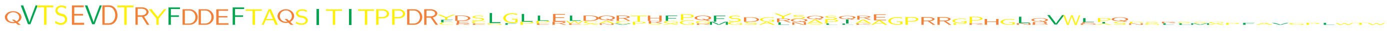

```
GSK-690693    ..........F...F.......................................................................   18
PF00169.2     ......................................................................................   90
PF00433       .VTSEVDTRYFDDEFTAQSITITPPDRYDSLGLLELDQRTHFPQFS..Y.....................................   46
PF00169.1     ......................................................................................  111
PF00069.1     ......................................................................................  256
PF00169       ......................................................................................  101
PF00069.2     ......................................................................................  195
PF00169.3     ......................................................................................   60
PF00169.4     ......................................................................................   94
PF00433.2     .VTSEVDTRYFDDEFTAQSITITPPDRF..........................................................   27
PF00069       ......................................................................................  213
PF00069.3     ......................................................................................   78
PF00433.1     .VTSEVDTRYFDDEFTAQSITITPPDRCECLGPPRWCACPRGGGMGSALN....................................   49
AKT2-232      QVTSEVDT..............................................................................  292
AKT2-214      ......................................................................................  107
AKT2-211      ......................................................................................   67
AKT2-219      ......................................................................................  157
AKT2-229      ......................................................................................  130
AKT2-224      ..............................................DGRQQPQAAGPRRGPHGLQVWLPQ................   69
AKT2-226      QVTSEVDTRYFDDEFTAQSITITPPDRFREGFLEEEANV.SAGR................R..NDVWDASNGRSMA..........  117
AKT2-210      QVTSEVDTRYFDDEFTAQSITITPPDRYDSLGLLELDQRTHFPQFS..YSASIRE...............................  438
AKT2-212      ......................................................................................  184
AKT2-241      ......................................................................................  151
AKT2-215      QVTSEVDTR.............................................................................  106
AKT2-234      QVTSEVDTRYFDDEFTAQSITITPPDRCECLGPPRWCACPRGGGMGSALNSLLKEGPRRPP..LHVWEISPSLLSGPFAVGPLWTW  450
AKT2-213      ......................................................................................  134
AKT2-206      QVTSEVDTRYFDDEFTAQSITITPPDRYDSLGLLELDQRTHFPQFS..YSASIRE...............................  481
AKT2-202      ......................................................................................  125
AKT2-208      ......................................................................................   95
AKT2-223      ......................................................................................   51
AKT2-209      ......................................................................................  119
AKT2-236      ......................................................................................   95
AKT2-240      ..............................................DGRQQPQAAGPRRGPHGLQVWLPQ................   69
AKT2-207      ......................................................................................  132
AKT2-205      ......................................................................................  133
AKT2-222      ......................................................................................   56
AKT2-201      QVTSEVDTRYFDDEFTAQSITITPPDRYDSLGLLELDQRTHFPQFS..YSASIRE...............................  438
AKT2-239      ......................................................................................   99
AKT2-231      ......................................................................................   53
AKT2-242      ......................................................................................  129
AKT2-203      ..............................................DGRQQPQAAGPRRGPHGLQVWLPQ................   69
```

X non conserved
X similar
X $\geq$0% conserved
X $\geq$50% conserved

logo

```
            MGAIGLLWLLPLLLSTAAVGSGMGTGQRAGSPAAGPPLQPREPLSYSRLQRKSLAVDFVVPSLFRVYARDLLLPPSSSELKAGRPEARGSLALDCAPLLRLLGPAPGVSW
ALECTINIB   ..............................................................................................................     0
PF07714     ..............................................................................................................     0
PF00629     ..............................................................................................................     0
PF07714.1   ..............................................................................................................     0
PF12810     ..............................................................................................................     0
PF00629.1   ..............................................................................................................     0
ALK-201     MGAIGLLWLLPLLLSTAAVGSGMGTGQRAGSPAAGPPLQPREPLSYSRLQRKSLAVDFVVPSLFRVYARDLLLPPSSSELKAGRPEARGSLALDCAPLLRLLGPAPGVSW   110
ALK-205     ..............................................................................................................     0
ALK-202     ..............................................................................................................     0
ALK-203     ..............................................................................................................     0
```

logo

```
            TAGSPAPAEARTLSRVLKGGSVRKLRRAKQLVLELGEEAILEGCVGPPGEAAVGLLQFNLSELFSWWIRQGEGRLRIRLMPEKKASEVGREGRLSAAIRASQPRLLFQIF
ALECTINIB   ..............................................................................................................     0
PF07714     ..............................................................................................................     0
PF00629     ..............................................................................................................     0
PF07714.1   ..............................................................................................................     0
PF12810     ..............................................................................................................     0
PF00629.1   ..............................................................................................................     0
ALK-201     TAGSPAPAEARTLSRVLKGGSVRKLRRAKQLVLELGEEAILEGCVGPPGEAAVGLLQFNLSELFSWWIRQGEGRLRIRLMPEKKASEVGREGRLSAAIRASQPRLLFQIF   220
ALK-205     ..............................................................................................................     0
ALK-202     ..............................................................................................................     0
ALK-203     ..............................................................................................................     0
```

logo

```
            GTGHSSLESPTNMPSPSPDYFTWNLTWIMKDSFPFLSHRSRYGLECSFDFPCELEYSPPLHDLRNQSWSWRRIPSEEASQMDLLDGPGAERSKEMPRGSFLLLNTSADSK
ALECTINIB   ..............................................................................................................     0
PF07714     ..............................................................................................................     0
PF00629     .............................................CSFDFPCELEYSPPLHDLRNQSWSWRRIPSEEASQMDLLDGPGAERSKEMPRGSFLLLNTSADSK    65
PF07714.1   ..............................................................................................................     0
PF12810     ..............................................................................................................     0
PF00629.1   ..............................................................................................................     0
ALK-201     GTGHSSLESPTNMPSPSPDYFTWNLTWIMKDSFPFLSHRSRYGLECSFDFPCELEYSPPLHDLRNQSWSWRRIPSEEASQMDLLDGPGAERSKEMPRGSFLLLNTSADSK   330
ALK-205     ..............................................................................................................     0
ALK-202     ..............................................................................................................     0
ALK-203     ..............................................................................................................     0
```

logo

```
            HTILSPWMRSSSEHCTLAVSVHRHLQPSGRYIAQLLPHNEAAREILLMPTPGKHGWTVLQGRIGRPDNPFRVALEYISSGNRSLSAVDFFALKNCSEGTSPGSKMALQSS
ALECTINIB   ..............................................................................................................     0
PF07714     ..............................................................................................................     0
PF00629     HTILSPWMRSSSEHCTLAVSVHRHLQPSGRYIAQLLPHNEAAREILLMPTPGKHGWTVLQGRIGRPDNPFRVALEYISSGNRSLSAVDFFALKNC...............   160
PF07714.1   ..............................................................................................................     0
PF12810     ..............................................................................................................     0
PF00629.1   ..............................................................................................................     0
ALK-201     HTILSPWMRSSSEHCTLAVSVHRHLQPSGRYIAQLLPHNEAAREILLMPTPGKHGWTVLQGRIGRPDNPFRVALEYISSGNRSLSAVDFFALKNCSEGTSPGSKMALQSS   440
ALK-205     ...............................................MPTPGKHGWTVLQGRIGRPDNPFRVALEYISSGNRSLSAVDFFALKNCSEGTSPGSKMALQSS    63
ALK-202     ..............................................................................................................     0
ALK-203     ..............................................................................................................     0
```

```
logo
           FTCWNGTVLQLGQACDFHQDCAQGEDESQMCRKLPVGFYCNFEDGFCGWTQGTLSPHTPQWQVRTLKDARFQDHQDHALLLSTTDVPASESATVTSATFPAPIKSSPCEL
ALECTINIB  ..............................................................................................................   0
PF07714    ..............................................................................................................   0
PF00629    .......................................CNFEDGFCGWTQGTLSPHTPQWQVRTLKDARFQDHQDHALLLSTTDVPASESATVTSATFPAPIKSSPCEL 231
PF07714.1  ..............................................................................................................   0
PF12810    ..............................................................................................................   0
PF00629.1  .......................................CNFEDGFCGWTQGTLSPHTPQWQVRTLKDARFQDHQDHALLLSTTDVPASESATVTSATFPAPIKSSPCEL  71
ALK-201    FTCWNGTVLQLGQACDFHQDCAQGEDESQMCRKLPVGFYCNFEDGFCGWTQGTLSPHTPQWQVRTLKDARFQDHQDHALLLSTTDVPASESATVTSATFPAPIKSSPCEL 550
ALK-205    FTCWNGTVLQLGQACDFHQDCAQGEDESQMCRKLPVGFYCNFEDGFCGWTQGTLSPHTPQWQVRTLKDARFQDHQDHALLLSTTDVPASESATVTSATFPAPIKSSPCEL 173
ALK-202    ..............................................................................................................   0
ALK-203    ..............................................................................................................   0
```

```
logo
           RMSWLIRGVLRGNVSLVLVENKTGKEQGRMVWHVAAYEGLSLWQWMVLPLLDVSDRFWLQMVAWWGQGSRAIVAFDNISISLDCYLTISGEDKILQNTAPKSRNLFERNP
ALECTINIB  ..............................................................................................................   0
PF07714    ..............................................................................................................   0
PF00629    RMSWLIRGVLRGNVSLVLVENKTGKEQGRMVWHVAAYEGLSLWQWMVLPLLDVSDRFWLQMVAWWGQGSRAIVAFDNISISLDC.......................... 315
PF07714.1  ..............................................................................................................   0
PF12810    ..............................................................................................................   0
PF00629.1  RMSWLIRGVLRGNVSLVLVENKTGKEQGRMVWHVAAYEGLSLWQWMVLPLLDVSDRFWLQMVAWWGQGSRAIVAFDNISISLDC.......................... 155
ALK-201    RMSWLIRGVLRGNVSLVLVENKTGKEQGRMVWHVAAYEGLSLWQWMVLPLLDVSDRFWLQMVAWWGQGSRAIVAFDNISISLDCYLTISGEDKILQNTAPKSRNLFERNP 660
ALK-205    RMSWLIRGVLRGNVSLVLVENKTGKEQGRMVWHVAAYEGLSLWQWMVLPLLDVSDRFWLQMVAWWGQGSRAIVAFDNISISLDCYLTISGEDKILQNTAPKSRNLFERNP 283
ALK-202    ..............................................................................................................   0
ALK-203    ..............................................................................................................   0
```

```
logo
           NKELKPGENSPRQTPIFDPTVHWLFTTCGASGPHGPTQAQCNNAYQNSNLSVEVGSEGPLKGIQIWKVPATDTYSISGYGAAGGKGGKNTMMRSHGVSVLGIFNLEKDDM
ALECTINIB  ..............................................................................................................   0
PF07714    ..............................................................................................................   0
PF00629    .............................................................................................................. 315
PF07714.1  ..............................................................................................................   0
PF12810    ......................................................................TDTYSISGYGAAGGKGGKNTMMRSHGVSVLGIFNLEKDDM  40
PF00629.1  .............................................................................................................. 155
ALK-201    NKELKPGENSPRQTPIFDPTVHWLFTTCGASGPHGPTQAQCNNAYQNSNLSVEVGSEGPLKGIQIWKVPATDTYSISGYGAAGGKGGKNTMMRSHGVSVLGIFNLEKDDM 770
ALK-205    NKELKPGENSPRQTPIFDPTVHWLFTTCGASGPHGPTQAQCNNAYQNSNLSVEVGSEGPLKGIQIWKVPATDTYSISGYGAAGGKGGKNTMMRSHGVSVLGIFNLEKDDM 393
ALK-202    ..............................................................................................................   0
ALK-203    ..............................................................................................................   0
```

```
logo
           LYILVGQQGEDACPSTNQLIQKVCIGENNVIEEEIRVNRSVHEWAGGGGGGGGATYVFKMKDGVPVPLIIAAGGGGRAYGAKTDTFHPERLENNSSVLGLNGNSGAAGGG
ALECTINIB  ..............................................................................................................   0
PF07714    ..............................................................................................................   0
PF00629    .............................................................................................................. 315
PF07714.1  ..............................................................................................................   0
PF12810    LYILVGQQGEDACPSTNQLIQKVCIGENNVIEEEIRVNRSVHEWAGGGGGGGGATYVFKMKDGVPVPLIIAAGGGGRAYGAKTDTFHPERLENNSSVLGLNGNSGAAGGG 150
PF00629.1  .............................................................................................................. 155
ALK-201    LYILVGQQGEDACPSTNQLIQKVCIGENNVIEEEIRVNRSVHEWAGGGGGGGGATYVFKMKDGVPVPLIIAAGGGGRAYGAKTDTFHPERLENNSSVLGLNGNSGAAGGG 880
ALK-205    LYILVGQQGEDACPSTNQLIQKVCIGENNVIEEEIRVNRSVHEWAGGGGGGGGATYVFKMKDGVPVPLIIAAGGGGRAYGAKTDTFHPERLENNSSVLGLNGNSGAAGGG 503
ALK-202    ..............................................................................................................   0
ALK-203    ..............................................................................................................   0
```

```
logo
             GGWNDNTSLLWAGKSLQEGATGGHSCPQAMKKWGWETRGGFGGGGGGCSSGGGGGGYIGGNAASNNDPEMDGEDGVSFISPLGILYTPALKVMEGHGEVNIKHYLNCSHC
ALECTINIB    ..............................................................................................................     0
PF07714      ..............................................................................................................     0
PF00629      ..............................................................................................................   315
PF07714.1    ..............................................................................................................     0
PF12810      GGWNDNTSLLWAGKSLQEGATGGHSCPQAMKKWGWETRGGFGGGGGGCSSGGGGGGYIGGNAASNNDPEMDGEDGVSFISP.............................   231
PF00629.1    ..............................................................................................................   155
ALK-201      GGWNDNTSLLWAGKSLQEGATGGHSCPQAMKKWGWETRGGFGGGGGGCSSGGGGGGYIGGNAASNNDPEMDGEDGVSFISPLGILYTPALKVMEGHGEVNIKHYLNCSHC   990
ALK-205      GGWNDNTSLLWAGKSLQEGATGGHSCPQAMKKWGWETRGGFGGGGGGCSSGGGGGGYIGGNAASNNDPEMDGEDGVSFISPLGILYTPALKVMEGHGEVNIKHYLNCSHC   613
ALK-202      ..............................................................................................................     0
ALK-203      ..............................................................................................................     0

logo
             EVDECHMDPESHKVICFCDHGTVLAEDGVSCIVSPTPEPHLPLSLILSVVTSALVAALVLAFSGIMIVYRRKHQELQAMQMELQSPEYKLSKLRTSTIMTDYNPNYCFAG
ALECTINIB    ..............................................................................................................     0
PF07714      ..............................................................................................................     0
PF00629      ..............................................................................................................   315
PF07714.1    ..............................................................................................................     0
PF12810      ..............................................................................................................   231
PF00629.1    ..............................................................................................................   155
ALK-201      EVDECHMDPESHKVICFCDHGTVLAEDGVSCIVSPTPEPHLPLSLILSVVTSALVAALVLAFSGIMIVYRRKHQELQAMQMELQSPEYKLSKLRTSTIMTDYNPNYCFAG  1100
ALK-205      EVDECHMDPESHKVICFCDHGTVLAEDGVSCIVSPTPEPHLPLSLILSVVTSALVAALVLAFSGIMIVYRRKHQELQAMQMELQSPEYKLSKLRTSTIMTDYNPNYCFAG   723
ALK-202      ................................................MVT.TCSAIYTFQF.IRSSVYRRKHQELQAMQMELQSPEYKLSKLRTSTIMTDYNPNYCFAG    60
ALK-203      ..............................................................................................................     0

logo
             KTSSISDLKEVPRKNITLIRGLGHGAFGEVYEGQVSGMPNDPSPLQVAVKTLPEVCSEQDELDFLMEALIISKFNHQNIV  RCIGVSLQ         SLPRFILLELM
ALECTINIB    ...................R.L.........................A.K.............................V..........................LELM     9
PF07714      ................TLIRGLGHGAFGEVYEGQVSGMPNDPSPLQVAVKTLPEVCSEQDELDFLMEALIISKFNHQNIV..RCIGVSLQ.........SLPRFILLELM    83
PF00629      ..............................................................................................................   315
PF07714.1    ................TLIRGLGHGAFGEVYEGQVSGMPNDPSPLQVAVKTLPEVCSEQDELDFLMEALIIS......................................    56
PF12810      ..............................................................................................................   231
PF00629.1    ..............................................................................................................   155
ALK-201      KTSSISDLKEVPRKNITLIRGLGHGAFGEVYEGQVSGMPNDPSPLQVAVKTLPEVCSEQDELDFLMEALIISKFNHQNIV..RCIGVSLQ.........SLPRFILLELM  1199
ALK-205      KTSSISDLKEVPRKNITLIRGLGHGAFGEVYEGQVSGMPNDPSPLQVAVKTLPEVCSEQDELDFLMEALIISKFNHQNIV..RCIGVSLQ.........SLPRFILLELM   822
ALK-202      KTSSISDLKEVPRKNITLIRGLGHGAFGEVYEGQVSGMPNDPSPLQVAVKTLPEVCSEQDELDFLMEALIISKFNHQNIV..RCIGVSLQ.........SLPRFILLELM   159
ALK-203      ..SSISDLKEVPRKNITLIRGLGHGAFGEVYEGQVSGMPNDPSPLQVAVKTLPEVCSEQDELDFLMEALIISLSWKQLFCFTFCLRPSWEKSTMNSSTQSTHQYL..RKT   106

logo
             AGGDLKSFLRETRPRPSQPSSLAMLDLLHVARDIACGCQYLEENHFIHRDIAARNCLLTCPGPGRVAKIGDFGMARDIYRASYYRKGGCAMLPVKWMPPEAFMEGIFTSK
ALECTINIB    A.......................................................L............G........................................    12
PF07714      AGGDLKSFLRETRPRPSQPSSLAMLDLLHVARDIACGCQYLEENHFIHRDIAARNCLLTCPGPGRVAKIGDFGMARDIYRASYYRKGGCAMLPVKWMPPEAFMEGIFTSK   193
PF00629      ..............................................................................................................   315
PF07714.1    ..............................................................................................................    56
PF12810      ..............................................................................................................   231
PF00629.1    ..............................................................................................................   155
ALK-201      AGGDLKSFLRETRPRPSQPSSLAMLDLLHVARDIACGCQYLEENHFIHRDIAARNCLLTCPGPGRVAKIGDFGMARDIYRASYYRKGGCAMLPVKWMPPEAFMEGIFTSK  1309
ALK-205      AGGDLKSFLRETRPRPSQPSSLAMLDLLHVARDIACGCQYLEENHFIHRDIAARNCLLTCPGPGRVAKIGDFGMARDIYRASYYRKGGCAMLPVKWMPPEAFMEGIFTSK   932
ALK-202      AGGDLKSFLRETRPRPSQPSSLAMLDLLHVARDIACGCQYLEENHFIHRDIAARNCLLTCPGPGRVAKIGDFGMARDIYRASYYRKGGCAMLPVKWMPPEAFMEGIFTSK   269
ALK-203      LSCPLRHFLTQVPEQHPHSSSHSPA..........NG...........TPVEPAVVLPTCS.PARF.............................WV.............   152
```

```
logo
             TDTWSFGVLLWEIFSLGYMPYPSKSNQEVLEFVTSGGRMDPPKNCPGPVYRIMTQCWQHQPEDRPNFAIILERIEYCTQDPDVINTALPIEYGPLVEEEEKVPVRPKDPE
ALECTINIB    ..............................................................................................................    12
PF07714      TDTWSFGVLLWEIFSLGYMPYPSKSNQEVLEFVTSGGRMDPPKNCPGPVYRIMTQCWQHQPEDRPNFAIILER.....................................   266
PF00629      ..............................................................................................................   315
PF07714.1    ..............................................................................................................    56
PF12810      ..............................................................................................................   231
PF00629.1    ..............................................................................................................   155
ALK-201      TDTWSFGVLLWEIFSLGYMPYPSKSNQEVLEFVTSGGRMDPPKNCPGPVYRIMTQCWQHQPEDRPNFAIILERIEYCTQDPDVINTALPIEYGPLVEEEEKVPVRPKDPE  1419
ALK-205      TDTWSFGVLLWEIFSLGYMPYPSKSNQEVLEFVTSGGRMDPPKNCPGPVYRIMTQCWQHQPEDRPNFAIILERIEYCTQDPDVINTALPIEYGPLVEEEEKVPVRPKDPE  1042
ALK-202      TDTWSFGVLLWEIFSLGYMPYPSKSNQEVLEFVTSGGRMDPPKNCPGPVYRIMTQCWQHQPEDRPNFAIILERIEYCTQDPDVINTALPIEYGPLVEEEEKVPVRPKDPE   379
ALK-203      ..............................................................................................................   152
```

```
logo
             GVPPLLVSQQAKREEERSPAAPPPLPTTSSGKAAKKPTAAEISVRVPRGPAVEGGHVNMAFSQSNPPSELHKVHGSRNKPTSLWNPTYGSWFTEKPTKKNNPIAKKEPHD
ALECTINIB    ..............................................................................................................    12
PF07714      ..............................................................................................................   266
PF00629      ..............................................................................................................   315
PF07714.1    ..............................................................................................................    56
PF12810      ..............................................................................................................   231
PF00629.1    ..............................................................................................................   155
ALK-201      GVPPLLVSQQAKREEERSPAAPPPLPTTSSGKAAKKPTAAEISVRVPRGPAVEGGHVNMAFSQSNPPSELHKVHGSRNKPTSLWNPTYGSWFTEKPTKKNNPIAKKEPHD  1529
ALK-205      GVPPLLVSQQAKREEERSPAAPPPLPTTSSGKAAKKPTAAEISVRVPRGPAVEGGHVNMAFSQSNPPSELHKVHGSRNKPTSLWNPTYGSWFTEKPTKKNNPIAKKEPHD  1152
ALK-202      GVPPLLVSQQAKREEERSPAAPPPLPTTSSGKAAKKPTAAEISVRVPRGPAVEGGHVNMAFSQSNPPSELHKVHGSRNKPTSLWNPTYGSWFTEKPTKKNNPIAKKEPHD   489
ALK-203      ..............................................................................................................   152
```

```
logo
             RGNLGLEGSCTVPPNVATGRLPGASLLLEPSSLTANMKEVPLFRLRHFPCGNVNYGYQQQGLPLEAATAPGAGHYEDTILKSKNSMNQPGP
ALECTINIB    ...........................................................................................    12
PF07714      ...........................................................................................   266
PF00629      ...........................................................................................   315
PF07714.1    ...........................................................................................    56
PF12810      ...........................................................................................   231
PF00629.1    ...........................................................................................   155
ALK-201      RGNLGLEGSCTVPPNVATGRLPGASLLLEPSSLTANMKEVPLFRLRHFPCGNVNYGYQQQGLPLEAATAPGAGHYEDTILKSKNSMNQPGP  1620
ALK-205      RGNLGLEGSCTVPPNVATGRLPGASLLLEPSSLTANMKEVPLFRLRHFPCGNVNYGYQQQGLPLEAATAPGAGHYEDTILKSKNSMNQPGP  1243
ALK-202      RGNLGLEGSCTVPPNVATGRLPGASLLLEPSSLTANMKEVPLFRLRHFPCGNVNYGYQQQGLPLEAATAPGAGHYEDTILKSKNSMNQPGP   580
ALK-203      ...........................................................................................   152
```

X non conserved

X similar

X $\geq 0\%$ conserved

X $\geq 50\%$ conserved

```
logo
            MGAIGLLWLLPLLLSTAAVGSGMGTGQRAGSPAAGPPLQPREPLSYSRLQRKSLAVDFVVPSLFRVYARDLLLPPSSSELKAGRPEARGSLALDCAPLLRLLGPAPGVSW
BRIGATINIB  ..............................................................................................................    0
PF07714     ..............................................................................................................    0
PF00629     ..............................................................................................................    0
PF07714.1   ..............................................................................................................    0
PF12810     ..............................................................................................................    0
PF00629.1   ..............................................................................................................    0
ALK-201     MGAIGLLWLLPLLLSTAAVGSGMGTGQRAGSPAAGPPLQPREPLSYSRLQRKSLAVDFVVPSLFRVYARDLLLPPSSSELKAGRPEARGSLALDCAPLLRLLGPAPGVSW  110
ALK-205     ..............................................................................................................    0
ALK-202     ..............................................................................................................    0
ALK-203     ..............................................................................................................    0

logo
            TAGSPAPAEARTLSRVLKGGSVRKLRRAKQLVLELGEEAILEGCVGPPGEAAVGLLQFNLSELFSWWIRQGEGRLRIRLMPEKKASEVGREGRLSAAIRASQPRLLFQIF
BRIGATINIB  ..............................................................................................................    0
PF07714     ..............................................................................................................    0
PF00629     ..............................................................................................................    0
PF07714.1   ..............................................................................................................    0
PF12810     ..............................................................................................................    0
PF00629.1   ..............................................................................................................    0
ALK-201     TAGSPAPAEARTLSRVLKGGSVRKLRRAKQLVLELGEEAILEGCVGPPGEAAVGLLQFNLSELFSWWIRQGEGRLRIRLMPEKKASEVGREGRLSAAIRASQPRLLFQIF  220
ALK-205     ..............................................................................................................    0
ALK-202     ..............................................................................................................    0
ALK-203     ..............................................................................................................    0

logo
            GTGHSSLESPTNMPSPSPDYFTWNLTWIMKDSFPFLSHRSRYGLECSFDFPCELEYSPPLHDLRNQSWSWRRIPSEEASQMDLLDGPGAERSKEMPRGSFLLLNTSADSK
BRIGATINIB  ..............................................................................................................    0
PF07714     ..............................................................................................................    0
PF00629     .............................................CSFDFPCELEYSPPLHDLRNQSWSWRRIPSEEASQMDLLDGPGAERSKEMPRGSFLLLNTSADSK   65
PF07714.1   ..............................................................................................................    0
PF12810     ..............................................................................................................    0
PF00629.1   ..............................................................................................................    0
ALK-201     GTGHSSLESPTNMPSPSPDYFTWNLTWIMKDSFPFLSHRSRYGLECSFDFPCELEYSPPLHDLRNQSWSWRRIPSEEASQMDLLDGPGAERSKEMPRGSFLLLNTSADSK  330
ALK-205     ..............................................................................................................    0
ALK-202     ..............................................................................................................    0
ALK-203     ..............................................................................................................    0

logo
            HTILSPWMRSSSEHCTLAVSVHRHLQPSGRYIAQLLPHNEAAREILLMPTPGKHGWTVLQGRIGRPDNPFRVALEYISSGNRSLSAVDFFALKNCSEGTSPGSKMALQSS
BRIGATINIB  ..............................................................................................................    0
PF07714     ..............................................................................................................    0
PF00629     HTILSPWMRSSSEHCTLAVSVHRHLQPSGRYIAQLLPHNEAAREILLMPTPGKHGWTVLQGRIGRPDNPFRVALEYISSGNRSLSAVDFFALKNC...............  160
PF07714.1   ..............................................................................................................    0
PF12810     ..............................................................................................................    0
PF00629.1   ..............................................................................................................    0
ALK-201     HTILSPWMRSSSEHCTLAVSVHRHLQPSGRYIAQLLPHNEAAREILLMPTPGKHGWTVLQGRIGRPDNPFRVALEYISSGNRSLSAVDFFALKNCSEGTSPGSKMALQSS  440
ALK-205     ...............................................MPTPGKHGWTVLQGRIGRPDNPFRVALEYISSGNRSLSAVDFFALKNCSEGTSPGSKMALQSS   63
ALK-202     ..............................................................................................................    0
ALK-203     ..............................................................................................................    0
```

```
logo
            FTCWNGTVLQLGQACDFHQDCAQGEDESQMCRKLPVGFYCNFEDGFCGWTQGTLSPHTPQWQVRTLKDARFQDHQDHALLLSTTDVPASESATVTSATFPAPIKSSPCEL
BRIGATINIB  ..............................................................................................................    0
PF07714     ..............................................................................................................    0
PF00629     .......................................CNFEDGFCGWTQGTLSPHTPQWQVRTLKDARFQDHQDHALLLSTTDVPASESATVTSATFPAPIKSSPCEL  231
PF07714.1   ..............................................................................................................    0
PF12810     ..............................................................................................................    0
PF00629.1   .......................................CNFEDGFCGWTQGTLSPHTPQWQVRTLKDARFQDHQDHALLLSTTDVPASESATVTSATFPAPIKSSPCEL   71
ALK-201     FTCWNGTVLQLGQACDFHQDCAQGEDESQMCRKLPVGFYCNFEDGFCGWTQGTLSPHTPQWQVRTLKDARFQDHQDHALLLSTTDVPASESATVTSATFPAPIKSSPCEL  550
ALK-205     FTCWNGTVLQLGQACDFHQDCAQGEDESQMCRKLPVGFYCNFEDGFCGWTQGTLSPHTPQWQVRTLKDARFQDHQDHALLLSTTDVPASESATVTSATFPAPIKSSPCEL  173
ALK-202     ..............................................................................................................    0
ALK-203     ..............................................................................................................    0

logo
            RMSWLIRGVLRGNVSLVLVENKTGKEQGRMVWHVAAYEGLSLWQWMVLPLLDVSDRFWLQMVAWWGQGSRAIVAFDNISISLDCYLTISGEDKILQNTAPKSRNLFERNP
BRIGATINIB  ..............................................................................................................    0
PF07714     ..............................................................................................................    0
PF00629     RMSWLIRGVLRGNVSLVLVENKTGKEQGRMVWHVAAYEGLSLWQWMVLPLLDVSDRFWLQMVAWWGQGSRAIVAFDNISISLDC..........................  315
PF07714.1   ..............................................................................................................    0
PF12810     ..............................................................................................................    0
PF00629.1   RMSWLIRGVLRGNVSLVLVENKTGKEQGRMVWHVAAYEGLSLWQWMVLPLLDVSDRFWLQMVAWWGQGSRAIVAFDNISISLDC..........................  155
ALK-201     RMSWLIRGVLRGNVSLVLVENKTGKEQGRMVWHVAAYEGLSLWQWMVLPLLDVSDRFWLQMVAWWGQGSRAIVAFDNISISLDCYLTISGEDKILQNTAPKSRNLFERNP  660
ALK-205     RMSWLIRGVLRGNVSLVLVENKTGKEQGRMVWHVAAYEGLSLWQWMVLPLLDVSDRFWLQMVAWWGQGSRAIVAFDNISISLDCYLTISGEDKILQNTAPKSRNLFERNP  283
ALK-202     ..............................................................................................................    0
ALK-203     ..............................................................................................................    0

logo
            NKELKPGENSPRQTPIFDPTVHWLFTTCGASGPHGPTQAQCNNAYQNSNLSVEVGSEGPLKGIQIWKVPATDTYSISGYGAAGGKGGKNTMMRSHGVSVLGIFNLEKDDM
BRIGATINIB  ..............................................................................................................    0
PF07714     ..............................................................................................................    0
PF00629     ..............................................................................................................  315
PF07714.1   ..............................................................................................................    0
PF12810     ......................................................................TDTYSISGYGAAGGKGGKNTMMRSHGVSVLGIFNLEKDDM   40
PF00629.1   ..............................................................................................................  155
ALK-201     NKELKPGENSPRQTPIFDPTVHWLFTTCGASGPHGPTQAQCNNAYQNSNLSVEVGSEGPLKGIQIWKVPATDTYSISGYGAAGGKGGKNTMMRSHGVSVLGIFNLEKDDM  770
ALK-205     NKELKPGENSPRQTPIFDPTVHWLFTTCGASGPHGPTQAQCNNAYQNSNLSVEVGSEGPLKGIQIWKVPATDTYSISGYGAAGGKGGKNTMMRSHGVSVLGIFNLEKDDM  393
ALK-202     ..............................................................................................................    0
ALK-203     ..............................................................................................................    0

logo
            LYILVGQQGEDACPSTNQLIQKVCIGENNVIEEEIRVNRSVHEWAGGGGGGGGATYVFKMKDGVPVPLIIAAGGGGRAYGAKTDTFHPERLENNSSVLGLNGNSGAAGGG
BRIGATINIB  ..............................................................................................................    0
PF07714     ..............................................................................................................    0
PF00629     ..............................................................................................................  315
PF07714.1   ..............................................................................................................    0
PF12810     LYILVGQQGEDACPSTNQLIQKVCIGENNVIEEEIRVNRSVHEWAGGGGGGGGATYVFKMKDGVPVPLIIAAGGGGRAYGAKTDTFHPERLENNSSVLGLNGNSGAAGGG  150
PF00629.1   ..............................................................................................................  155
ALK-201     LYILVGQQGEDACPSTNQLIQKVCIGENNVIEEEIRVNRSVHEWAGGGGGGGGATYVFKMKDGVPVPLIIAAGGGGRAYGAKTDTFHPERLENNSSVLGLNGNSGAAGGG  880
ALK-205     LYILVGQQGEDACPSTNQLIQKVCIGENNVIEEEIRVNRSVHEWAGGGGGGGGATYVFKMKDGVPVPLIIAAGGGGRAYGAKTDTFHPERLENNSSVLGLNGNSGAAGGG  503
ALK-202     ..............................................................................................................    0
ALK-203     ..............................................................................................................    0
```

```
logo
             GGWNDNTSLLWAGKSLQEGATGGHSCPQAMKKWGWETRGGFGGGGGGCSSGGGGGGYIGGNAASNNDPEMDGEDGVSFISPLGILYTPALKVMEGHGEVNIKHYLNCSHC
BRIGATINIB   ..............................................................................................................     0
PF07714      ..............................................................................................................     0
PF00629      ..............................................................................................................   315
PF07714.1    ..............................................................................................................     0
PF12810      GGWNDNTSLLWAGKSLQEGATGGHSCPQAMKKWGWETRGGFGGGGGGCSSGGGGGGYIGGNAASNNDPEMDGEDGVSFISP.............................   231
PF00629.1    ..............................................................................................................   155
ALK-201      GGWNDNTSLLWAGKSLQEGATGGHSCPQAMKKWGWETRGGFGGGGGGCSSGGGGGGYIGGNAASNNDPEMDGEDGVSFISPLGILYTPALKVMEGHGEVNIKHYLNCSHC   990
ALK-205      GGWNDNTSLLWAGKSLQEGATGGHSCPQAMKKWGWETRGGFGGGGGGCSSGGGGGGYIGGNAASNNDPEMDGEDGVSFISPLGILYTPALKVMEGHGEVNIKHYLNCSHC   613
ALK-202      ..............................................................................................................     0
ALK-203      ..............................................................................................................     0

logo
             EVDECHMDPESHKVICFCDHGTVLAEDGVSCIVSPTPEPHLPLSLILSVVTSALVAALVLAFSGIMIVYRRKHQELQAMQMELQSPEYKLSKLRTSTIMTDYNPNYCFAG
BRIGATINIB   ............................................L................................A................................     2
PF07714      ..............................................................................................................     0
PF00629      ..............................................................................................................   315
PF07714.1    ..............................................................................................................     0
PF12810      ..............................................................................................................   231
PF00629.1    ..............................................................................................................   155
ALK-201      EVDECHMDPESHKVICFCDHGTVLAEDGVSCIVSPTPEPHLPLSLILSVVTSALVAALVLAFSGIMIVYRRKHQELQAMQMELQSPEYKLSKLRTSTIMTDYNPNYCFAG  1100
ALK-205      EVDECHMDPESHKVICFCDHGTVLAEDGVSCIVSPTPEPHLPLSLILSVVTSALVAALVLAFSGIMIVYRRKHQELQAMQMELQSPEYKLSKLRTSTIMTDYNPNYCFAG   723
ALK-202      ................................................MVT.TCSAIYTFQF.IRSSVYRRKHQELQAMQMELQSPEYKLSKLRTSTIMTDYNPNYCFAG    60
ALK-203      ..............................................................................................................     0

logo
             KTSSISDLKEVPRKNITLIRGLGHGAFGEVYEGQVSGMPNDPSPLQVAVKTLPEVCSEQDELDFLMEALIISKFNHQNIV RCIGVSLQ SLPRFILLELM
BRIGATINIB   ..........................................................................................................LE.M     5
PF07714      ................TLIRGLGHGAFGEVYEGQVSGMPNDPSPLQVAVKTLPEVCSEQDELDFLMEALIISKFNHQNIV..RCIGVSLQ.........SLPRFILLELM    83
PF00629      ..............................................................................................................   315
PF07714.1    ................TLIRGLGHGAFGEVYEGQVSGMPNDPSPLQVAVKTLPEVCSEQDELDFLMEALIIS......................................    56
PF12810      ..............................................................................................................   231
PF00629.1    ..............................................................................................................   155
ALK-201      KTSSISDLKEVPRKNITLIRGLGHGAFGEVYEGQVSGMPNDPSPLQVAVKTLPEVCSEQDELDFLMEALIISKFNHQNIV..RCIGVSLQ.........SLPRFILLELM  1199
ALK-205      KTSSISDLKEVPRKNITLIRGLGHGAFGEVYEGQVSGMPNDPSPLQVAVKTLPEVCSEQDELDFLMEALIISKFNHQNIV..RCIGVSLQ.........SLPRFILLELM   822
ALK-202      KTSSISDLKEVPRKNITLIRGLGHGAFGEVYEGQVSGMPNDPSPLQVAVKTLPEVCSEQDELDFLMEALIISKFNHQNIV..RCIGVSLQ.........SLPRFILLELM   159
ALK-203      ..SSISDLKEVPRKNITLIRGLGHGAFGEVYEGQVSGMPNDPSPLQVAVKTLPEVCSEQDELDFLMEALIISLSWKQLFCFTFCLRPSWEKSTMNSSTQSTHQYL..RKT   106

logo
             AGGDLKSFLRETRPRPSQPSSLAMLDLLHVARDIACGCQYLEENHFIHRDIAARNCLLTCPGPGRVAKIGDFGMARDIYRASYYRKGGCAMLPVKWMPPEAFMEGIFTSK
BRIGATINIB   A.G.....................................................L.............D.......................................     9
PF07714      AGGDLKSFLRETRPRPSQPSSLAMLDLLHVARDIACGCQYLEENHFIHRDIAARNCLLTCPGPGRVAKIGDFGMARDIYRASYYRKGGCAMLPVKWMPPEAFMEGIFTSK   193
PF00629      ..............................................................................................................   315
PF07714.1    ..............................................................................................................    56
PF12810      ..............................................................................................................   231
PF00629.1    ..............................................................................................................   155
ALK-201      AGGDLKSFLRETRPRPSQPSSLAMLDLLHVARDIACGCQYLEENHFIHRDIAARNCLLTCPGPGRVAKIGDFGMARDIYRASYYRKGGCAMLPVKWMPPEAFMEGIFTSK  1309
ALK-205      AGGDLKSFLRETRPRPSQPSSLAMLDLLHVARDIACGCQYLEENHFIHRDIAARNCLLTCPGPGRVAKIGDFGMARDIYRASYYRKGGCAMLPVKWMPPEAFMEGIFTSK   932
ALK-202      AGGDLKSFLRETRPRPSQPSSLAMLDLLHVARDIACGCQYLEENHFIHRDIAARNCLLTCPGPGRVAKIGDFGMARDIYRASYYRKGGCAMLPVKWMPPEAFMEGIFTSK   269
ALK-203      LSCPLRHFLTQVPEQHPHSSSHSPA..........NG...........TPVEPAVVLPTCS.PARF.............................WV.............   152
```

```
logo
             TDTWSFGVLLWEIFSLGYMPYPSKSNQEVLEFVTSGGRMDPPKNCPGPVYRIMTQCWQHQPEDRPNFAIILERIEYCTQDPDVINTALPIEYGPLVEEEEKVPVRPKDPE
BRIGATINIB   ..............................................................................................................     9
PF07714      TDTWSFGVLLWEIFSLGYMPYPSKSNQEVLEFVTSGGRMDPPKNCPGPVYRIMTQCWQHQPEDRPNFAIILER.....................................   266
PF00629      ..............................................................................................................   315
PF07714.1    ..............................................................................................................    56
PF12810      ..............................................................................................................   231
PF00629.1    ..............................................................................................................   155
ALK-201      TDTWSFGVLLWEIFSLGYMPYPSKSNQEVLEFVTSGGRMDPPKNCPGPVYRIMTQCWQHQPEDRPNFAIILERIEYCTQDPDVINTALPIEYGPLVEEEEKVPVRPKDPE  1419
ALK-205      TDTWSFGVLLWEIFSLGYMPYPSKSNQEVLEFVTSGGRMDPPKNCPGPVYRIMTQCWQHQPEDRPNFAIILERIEYCTQDPDVINTALPIEYGPLVEEEEKVPVRPKDPE  1042
ALK-202      TDTWSFGVLLWEIFSLGYMPYPSKSNQEVLEFVTSGGRMDPPKNCPGPVYRIMTQCWQHQPEDRPNFAIILERIEYCTQDPDVINTALPIEYGPLVEEEEKVPVRPKDPE   379
ALK-203      ..............................................................................................................   152

logo
             GVPPLLVSQQAKREEERSPAAPPPLPTTSSGKAAKKPTAAEISVRVPRGPAVEGGHVNMAFSQSNPPSELHKVHGSRNKPTSLWNPTYGSWFTEKPTKKNNPIAKKEPHD
BRIGATINIB   ..............................................................................................................     9
PF07714      ..............................................................................................................   266
PF00629      ..............................................................................................................   315
PF07714.1    ..............................................................................................................    56
PF12810      ..............................................................................................................   231
PF00629.1    ..............................................................................................................   155
ALK-201      GVPPLLVSQQAKREEERSPAAPPPLPTTSSGKAAKKPTAAEISVRVPRGPAVEGGHVNMAFSQSNPPSELHKVHGSRNKPTSLWNPTYGSWFTEKPTKKNNPIAKKEPHD  1529
ALK-205      GVPPLLVSQQAKREEERSPAAPPPLPTTSSGKAAKKPTAAEISVRVPRGPAVEGGHVNMAFSQSNPPSELHKVHGSRNKPTSLWNPTYGSWFTEKPTKKNNPIAKKEPHD  1152
ALK-202      GVPPLLVSQQAKREEERSPAAPPPLPTTSSGKAAKKPTAAEISVRVPRGPAVEGGHVNMAFSQSNPPSELHKVHGSRNKPTSLWNPTYGSWFTEKPTKKNNPIAKKEPHD   489
ALK-203      ..............................................................................................................   152

logo
             RGNLGLEGSCTVPPNVATGRLPGASLLLEPSSLTANMKEVPLFRLRHFPCGNVNYGYQQQGLPLEAATAPGAGHYEDTILKSKNSMNQPGP
BRIGATINIB   ...........................................................................................     9
PF07714      ...........................................................................................   266
PF00629      ...........................................................................................   315
PF07714.1    ...........................................................................................    56
PF12810      ...........................................................................................   231
PF00629.1    ...........................................................................................   155
ALK-201      RGNLGLEGSCTVPPNVATGRLPGASLLLEPSSLTANMKEVPLFRLRHFPCGNVNYGYQQQGLPLEAATAPGAGHYEDTILKSKNSMNQPGP  1620
ALK-205      RGNLGLEGSCTVPPNVATGRLPGASLLLEPSSLTANMKEVPLFRLRHFPCGNVNYGYQQQGLPLEAATAPGAGHYEDTILKSKNSMNQPGP  1243
ALK-202      RGNLGLEGSCTVPPNVATGRLPGASLLLEPSSLTANMKEVPLFRLRHFPCGNVNYGYQQQGLPLEAATAPGAGHYEDTILKSKNSMNQPGP   580
ALK-203      ...........................................................................................   152
```

X non conserved

X similar

X ≥ 0% conserved

X ≥ 50% conserved

```
logo
           MGAIGLLWLLPLLLSTAAVGSGMGTGQRAGSPAAGPPLQPREPLSYSRLQRKSLAVDFVVPSLFRVYARDLLLPPSSSELKAGRPEARGSLALDCAPLLRLLGPAPGVSW
CERITINIB  ..............................................................................................................    0
PF07714    ..............................................................................................................    0
PF00629    ..............................................................................................................    0
PF07714.1  ..............................................................................................................    0
PF12810    ..............................................................................................................    0
PF00629.1  ..............................................................................................................    0
ALK-201    MGAIGLLWLLPLLLSTAAVGSGMGTGQRAGSPAAGPPLQPREPLSYSRLQRKSLAVDFVVPSLFRVYARDLLLPPSSSELKAGRPEARGSLALDCAPLLRLLGPAPGVSW  110
ALK-205    ..............................................................................................................    0
ALK-202    ..............................................................................................................    0
ALK-203    ..............................................................................................................    0

logo
           TAGSPAPAEARTLSRVLKGGSVRKLRRAKQLVLELGEEAILEGCVGPPGEAAVGLLQFNLSELFSWWIRQGEGRLRIRLMPEKKASEVGREGRLSAAIRASQPRLLFQIF
CERITINIB  ..............................................................................................................    0
PF07714    ..............................................................................................................    0
PF00629    ..............................................................................................................    0
PF07714.1  ..............................................................................................................    0
PF12810    ..............................................................................................................    0
PF00629.1  ..............................................................................................................    0
ALK-201    TAGSPAPAEARTLSRVLKGGSVRKLRRAKQLVLELGEEAILEGCVGPPGEAAVGLLQFNLSELFSWWIRQGEGRLRIRLMPEKKASEVGREGRLSAAIRASQPRLLFQIF  220
ALK-205    ..............................................................................................................    0
ALK-202    ..............................................................................................................    0
ALK-203    ..............................................................................................................    0

logo
           GTGHSSLESPTNMPSPSPDYFTWNLTWIMKDSFPFLSHRSRYGLECSFDFPCELEYSPPLHDLRNQSWSWRRIPSEEASQMDLLDGPGAERSKEMPRGSFLLLNTSADSK
CERITINIB  ..............................................................................................................    0
PF07714    ..............................................................................................................    0
PF00629    .............................................CSFDFPCELEYSPPLHDLRNQSWSWRRIPSEEASQMDLLDGPGAERSKEMPRGSFLLLNTSADSK   65
PF07714.1  ..............................................................................................................    0
PF12810    ..............................................................................................................    0
PF00629.1  ..............................................................................................................    0
ALK-201    GTGHSSLESPTNMPSPSPDYFTWNLTWIMKDSFPFLSHRSRYGLECSFDFPCELEYSPPLHDLRNQSWSWRRIPSEEASQMDLLDGPGAERSKEMPRGSFLLLNTSADSK  330
ALK-205    ..............................................................................................................    0
ALK-202    ..............................................................................................................    0
ALK-203    ..............................................................................................................    0

logo
           HTILSPWMRSSSEHCTLAVSVHRHLQPSGRYIAQLLPHNEAAREILLMPTPGKHGWTVLQGRIGRPDNPFRVALEYISSGNRSLSAVDFFALKNCSEGTSPGSKMALQSS
CERITINIB  ..............................................................................................................    0
PF07714    ..............................................................................................................    0
PF00629    HTILSPWMRSSSEHCTLAVSVHRHLQPSGRYIAQLLPHNEAAREILLMPTPGKHGWTVLQGRIGRPDNPFRVALEYISSGNRSLSAVDFFALKNC...............  160
PF07714.1  ..............................................................................................................    0
PF12810    ..............................................................................................................    0
PF00629.1  ..............................................................................................................    0
ALK-201    HTILSPWMRSSSEHCTLAVSVHRHLQPSGRYIAQLLPHNEAAREILLMPTPGKHGWTVLQGRIGRPDNPFRVALEYISSGNRSLSAVDFFALKNCSEGTSPGSKMALQSS  440
ALK-205    ...............................................MPTPGKHGWTVLQGRIGRPDNPFRVALEYISSGNRSLSAVDFFALKNCSEGTSPGSKMALQSS   63
ALK-202    ..............................................................................................................    0
ALK-203    ..............................................................................................................    0
```

```
logo
            FTCWNGTVLQLGQACDFHQDCAQGEDESQMCRKLPVGFYCNFEDGFCGWTQGTLSPHTPQWQVRTLKDARFQDHQDHALLLSTTDVPASESATVTSATFPAPIKSSPCEL
CERITINIB   ..............................................................................................................   0
PF07714     ..............................................................................................................   0
PF00629     .......................................CNFEDGFCGWTQGTLSPHTPQWQVRTLKDARFQDHQDHALLLSTTDVPASESATVTSATFPAPIKSSPCEL 231
PF07714.1   ..............................................................................................................   0
PF12810     ..............................................................................................................   0
PF00629.1   .......................................CNFEDGFCGWTQGTLSPHTPQWQVRTLKDARFQDHQDHALLLSTTDVPASESATVTSATFPAPIKSSPCEL  71
ALK-201     FTCWNGTVLQLGQACDFHQDCAQGEDESQMCRKLPVGFYCNFEDGFCGWTQGTLSPHTPQWQVRTLKDARFQDHQDHALLLSTTDVPASESATVTSATFPAPIKSSPCEL 550
ALK-205     FTCWNGTVLQLGQACDFHQDCAQGEDESQMCRKLPVGFYCNFEDGFCGWTQGTLSPHTPQWQVRTLKDARFQDHQDHALLLSTTDVPASESATVTSATFPAPIKSSPCEL 173
ALK-202     ..............................................................................................................   0
ALK-203     ..............................................................................................................   0

logo
            RMSWLIRGVLRGNVSLVLVENKTGKEQGRMVWHVAAYEGLSLWQWMVLPLLDVSDRFWLQMVAWWGQGSRAIVAFDNISISLDCYLTISGEDKILQNTAPKSRNLFERNP
CERITINIB   ..............................................................................................................   0
PF07714     ..............................................................................................................   0
PF00629     RMSWLIRGVLRGNVSLVLVENKTGKEQGRMVWHVAAYEGLSLWQWMVLPLLDVSDRFWLQMVAWWGQGSRAIVAFDNISISLDC.......................... 315
PF07714.1   ..............................................................................................................   0
PF12810     ..............................................................................................................   0
PF00629.1   RMSWLIRGVLRGNVSLVLVENKTGKEQGRMVWHVAAYEGLSLWQWMVLPLLDVSDRFWLQMVAWWGQGSRAIVAFDNISISLDC.......................... 155
ALK-201     RMSWLIRGVLRGNVSLVLVENKTGKEQGRMVWHVAAYEGLSLWQWMVLPLLDVSDRFWLQMVAWWGQGSRAIVAFDNISISLDCYLTISGEDKILQNTAPKSRNLFERNP 660
ALK-205     RMSWLIRGVLRGNVSLVLVENKTGKEQGRMVWHVAAYEGLSLWQWMVLPLLDVSDRFWLQMVAWWGQGSRAIVAFDNISISLDCYLTISGEDKILQNTAPKSRNLFERNP 283
ALK-202     ..............................................................................................................   0
ALK-203     ..............................................................................................................   0

logo
            NKELKPGENSPRQTPIFDPTVHWLFTTCGASGPHGPTQAQCNNAYQNSNLSVEVGSEGPLKGIQIWKVPATDTYSISGYGAAGGKGGKNTMMRSHGVSVLGIFNLEKDDM
CERITINIB   ..............................................................................................................   0
PF07714     ..............................................................................................................   0
PF00629     .............................................................................................................. 315
PF07714.1   ..............................................................................................................   0
PF12810     ......................................................................TDTYSISGYGAAGGKGGKNTMMRSHGVSVLGIFNLEKDDM  40
PF00629.1   .............................................................................................................. 155
ALK-201     NKELKPGENSPRQTPIFDPTVHWLFTTCGASGPHGPTQAQCNNAYQNSNLSVEVGSEGPLKGIQIWKVPATDTYSISGYGAAGGKGGKNTMMRSHGVSVLGIFNLEKDDM 770
ALK-205     NKELKPGENSPRQTPIFDPTVHWLFTTCGASGPHGPTQAQCNNAYQNSNLSVEVGSEGPLKGIQIWKVPATDTYSISGYGAAGGKGGKNTMMRSHGVSVLGIFNLEKDDM 393
ALK-202     ..............................................................................................................   0
ALK-203     ..............................................................................................................   0

logo
            LYILVGQQGEDACPSTNQLIQKVCIGENNVIEEEIRVNRSVHEWAGGGGGGGGGATYVFKMKDGVPVPLIIAAGGGGRAYGAKTDTFHPERLENNSSVLGLNGNSGAAGGG
CERITINIB   ..............................................................................................................   0
PF07714     ..............................................................................................................   0
PF00629     .............................................................................................................. 315
PF07714.1   ..............................................................................................................   0
PF12810     LYILVGQQGEDACPSTNQLIQKVCIGENNVIEEEIRVNRSVHEWAGGGGGGGGGATYVFKMKDGVPVPLIIAAGGGGRAYGAKTDTFHPERLENNSSVLGLNGNSGAAGGG 150
PF00629.1   .............................................................................................................. 155
ALK-201     LYILVGQQGEDACPSTNQLIQKVCIGENNVIEEEIRVNRSVHEWAGGGGGGGGGATYVFKMKDGVPVPLIIAAGGGGRAYGAKTDTFHPERLENNSSVLGLNGNSGAAGGG 880
ALK-205     LYILVGQQGEDACPSTNQLIQKVCIGENNVIEEEIRVNRSVHEWAGGGGGGGGGATYVFKMKDGVPVPLIIAAGGGGRAYGAKTDTFHPERLENNSSVLGLNGNSGAAGGG 503
ALK-202     ..............................................................................................................   0
ALK-203     ..............................................................................................................   0
```

logo

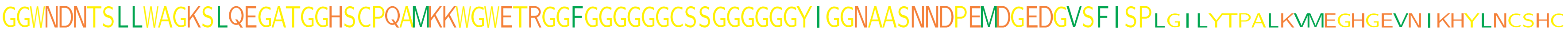

```
CERITINIB   ..............................................................................................................     0
PF07714     ..............................................................................................................     0
PF00629     ..............................................................................................................   315
PF07714.1   ..............................................................................................................     0
PF12810     GGWNDNTSLLWAGKSLQEGATGGHSCPQAMKKWGWETRGGFGGGGGGCSSGGGGGGYIGGNAASNNDPEMDGEDGVSFISP.............................   231
PF00629.1   ..............................................................................................................   155
ALK-201     GGWNDNTSLLWAGKSLQEGATGGHSCPQAMKKWGWETRGGFGGGGGGCSSGGGGGGYIGGNAASNNDPEMDGEDGVSFISPLGILYTPALKVMEGHGEVNIKHYLNCSHC   990
ALK-205     GGWNDNTSLLWAGKSLQEGATGGHSCPQAMKKWGWETRGGFGGGGGGCSSGGGGGGYIGGNAASNNDPEMDGEDGVSFISPLGILYTPALKVMEGHGEVNIKHYLNCSHC   613
ALK-202     ..............................................................................................................     0
ALK-203     ..............................................................................................................     0
```

logo

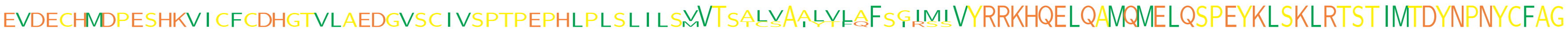

```
CERITINIB   ..............................................................................................................     0
PF07714     ..............................................................................................................     0
PF00629     ..............................................................................................................   315
PF07714.1   ..............................................................................................................     0
PF12810     ..............................................................................................................   231
PF00629.1   ..............................................................................................................   155
ALK-201     EVDECHMDPESHKVICFCDHGTVLAEDGVSCIVSPTPEPHLPLSLILSVVTSALVAALVLAFSGIMIVYRRKHQELQAMQMELQSPEYKLSKLRTSTIMTDYNPNYCFAG  1100
ALK-205     EVDECHMDPESHKVICFCDHGTVLAEDGVSCIVSPTPEPHLPLSLILSVVTSALVAALVLAFSGIMIVYRRKHQELQAMQMELQSPEYKLSKLRTSTIMTDYNPNYCFAG   723
ALK-202     ................................................MVT.TCSAIYTFQF.IRSSVYRRKHQELQAMQMELQSPEYKLSKLRTSTIMTDYNPNYCFAG    60
ALK-203     ..............................................................................................................     0
```

logo

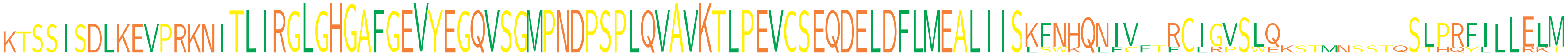

```
CERITINIB   .....................L.HG....V.................A.K........................................................LE.M     9
PF07714     ................TLIRGLGHGAFGEVYEGQVSGMPNDPSPLQVAVKTLPEVCSEQDELDFLMEALIISKFNHQNIV..RCIGVSLQ.........SLPRFILLELM    83
PF00629     ..............................................................................................................   315
PF07714.1   ................TLIRGLGHGAFGEVYEGQVSGMPNDPSPLQVAVKTLPEVCSEQDELDFLMEALIIS......................................    56
PF12810     ..............................................................................................................   231
PF00629.1   ..............................................................................................................   155
ALK-201     KTSSISDLKEVPRKNITLIRGLGHGAFGEVYEGQVSGMPNDPSPLQVAVKTLPEVCSEQDELDFLMEALIISKFNHQNIV..RCIGVSLQ.........SLPRFILLELM  1199
ALK-205     KTSSISDLKEVPRKNITLIRGLGHGAFGEVYEGQVSGMPNDPSPLQVAVKTLPEVCSEQDELDFLMEALIISKFNHQNIV..RCIGVSLQ.........SLPRFILLELM   822
ALK-202     KTSSISDLKEVPRKNITLIRGLGHGAFGEVYEGQVSGMPNDPSPLQVAVKTLPEVCSEQDELDFLMEALIISKFNHQNIV..RCIGVSLQ.........SLPRFILLELM   159
ALK-203     ..SSISDLKEVPRKNITLIRGLGHGAFGEVYEGQVSGMPNDPSPLQVAVKTLPEVCSEQDELDFLMEALIISLSWKQLFCFTFCLRPSWEKSTMNSSTQSTHQYL..RKT   106
```

logo

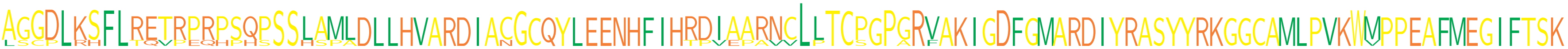

```
CERITINIB   A.GD..S.................................................L.............D.......................................    15
PF07714     AGGDLKSFLRETRPRPSQPSSLAMLDLLHVARDIACGCQYLEENHFIHRDIAARNCLLTCPGPGRVAKIGDFGMARDIYRASYYRKGGCAMLPVKWMPPEAFMEGIFTSK   193
PF00629     ..............................................................................................................   315
PF07714.1   ..............................................................................................................    56
PF12810     ..............................................................................................................   231
PF00629.1   ..............................................................................................................   155
ALK-201     AGGDLKSFLRETRPRPSQPSSLAMLDLLHVARDIACGCQYLEENHFIHRDIAARNCLLTCPGPGRVAKIGDFGMARDIYRASYYRKGGCAMLPVKWMPPEAFMEGIFTSK  1309
ALK-205     AGGDLKSFLRETRPRPSQPSSLAMLDLLHVARDIACGCQYLEENHFIHRDIAARNCLLTCPGPGRVAKIGDFGMARDIYRASYYRKGGCAMLPVKWMPPEAFMEGIFTSK   932
ALK-202     AGGDLKSFLRETRPRPSQPSSLAMLDLLHVARDIACGCQYLEENHFIHRDIAARNCLLTCPGPGRVAKIGDFGMARDIYRASYYRKGGCAMLPVKWMPPEAFMEGIFTSK   269
ALK-203     LSCPLRHFLTQVPEQHPHSSSHSPA..........NG...........TPVEPAVVLPTCS.PARF.............................WV.............   152
```

```
logo
            TDTWSFGVLLWEIFSLGYMPYPSKSNQEVLEFVTSGGRMDPPKNCPGPVYRIMTQCWQHQPEDRPNFAIILERIEYCTQDPDVINTALPIEYGPLVEEEEKVPVRPKDPE
CERITINIB   ..............................................................................................................   15
PF07714     TDTWSFGVLLWEIFSLGYMPYPSKSNQEVLEFVTSGGRMDPPKNCPGPVYRIMTQCWQHQPEDRPNFAIILER.....................................  266
PF00629     ..............................................................................................................  315
PF07714.1   ..............................................................................................................   56
PF12810     ..............................................................................................................  231
PF00629.1   ..............................................................................................................  155
ALK-201     TDTWSFGVLLWEIFSLGYMPYPSKSNQEVLEFVTSGGRMDPPKNCPGPVYRIMTQCWQHQPEDRPNFAIILERIEYCTQDPDVINTALPIEYGPLVEEEEKVPVRPKDPE 1419
ALK-205     TDTWSFGVLLWEIFSLGYMPYPSKSNQEVLEFVTSGGRMDPPKNCPGPVYRIMTQCWQHQPEDRPNFAIILERIEYCTQDPDVINTALPIEYGPLVEEEEKVPVRPKDPE 1042
ALK-202     TDTWSFGVLLWEIFSLGYMPYPSKSNQEVLEFVTSGGRMDPPKNCPGPVYRIMTQCWQHQPEDRPNFAIILERIEYCTQDPDVINTALPIEYGPLVEEEEKVPVRPKDPE  379
ALK-203     ..............................................................................................................  152

logo
            GVPPLLVSQQAKREEERSPAAPPPLPTTSSGKAAKKPTAAEISVRVPRGPAVEGGHVNMAFSQSNPPSELHKVHGSRNKPTSLWNPTYGSWFTEKPTKKNNPIAKKEPHD
CERITINIB   ..............................................................................................................   15
PF07714     ..............................................................................................................  266
PF00629     ..............................................................................................................  315
PF07714.1   ..............................................................................................................   56
PF12810     ..............................................................................................................  231
PF00629.1   ..............................................................................................................  155
ALK-201     GVPPLLVSQQAKREEERSPAAPPPLPTTSSGKAAKKPTAAEISVRVPRGPAVEGGHVNMAFSQSNPPSELHKVHGSRNKPTSLWNPTYGSWFTEKPTKKNNPIAKKEPHD 1529
ALK-205     GVPPLLVSQQAKREEERSPAAPPPLPTTSSGKAAKKPTAAEISVRVPRGPAVEGGHVNMAFSQSNPPSELHKVHGSRNKPTSLWNPTYGSWFTEKPTKKNNPIAKKEPHD 1152
ALK-202     GVPPLLVSQQAKREEERSPAAPPPLPTTSSGKAAKKPTAAEISVRVPRGPAVEGGHVNMAFSQSNPPSELHKVHGSRNKPTSLWNPTYGSWFTEKPTKKNNPIAKKEPHD  489
ALK-203     ..............................................................................................................  152

logo
            RGNLGLEGSCTVPPNVATGRLPGASLLLEPSSLTANMKEVPLFRLRHFPCGNVNYGYQQQGLPLEAATAPGAGHYEDTILKSKNSMNQPGP
CERITINIB   ...........................................................................................   15
PF07714     ...........................................................................................  266
PF00629     ...........................................................................................  315
PF07714.1   ...........................................................................................   56
PF12810     ...........................................................................................  231
PF00629.1   ...........................................................................................  155
ALK-201     RGNLGLEGSCTVPPNVATGRLPGASLLLEPSSLTANMKEVPLFRLRHFPCGNVNYGYQQQGLPLEAATAPGAGHYEDTILKSKNSMNQPGP 1620
ALK-205     RGNLGLEGSCTVPPNVATGRLPGASLLLEPSSLTANMKEVPLFRLRHFPCGNVNYGYQQQGLPLEAATAPGAGHYEDTILKSKNSMNQPGP 1243
ALK-202     RGNLGLEGSCTVPPNVATGRLPGASLLLEPSSLTANMKEVPLFRLRHFPCGNVNYGYQQQGLPLEAATAPGAGHYEDTILKSKNSMNQPGP  580
ALK-203     ...........................................................................................  152
```

X non conserved
X similar
X $\geq 0\%$ conserved
X $\geq 50\%$ conserved

```
logo

             MGAIGLLWLLPLLLSTAAVGSGMGTGQRAGSPAAGPPLQPREPLSYSRLQRKSLAVDFVVPSLFRVYARDLLLPPSSSELKAGRPEARGSLALDCAPLLRLLGPAPGVSW
CRIZOTINIB   ..............................................................................................................     0
PF07714      ..............................................................................................................     0
PF00629      ..............................................................................................................     0
PF07714.1    ..............................................................................................................     0
PF12810      ..............................................................................................................     0
PF00629.1    ..............................................................................................................     0
ALK-201      MGAIGLLWLLPLLLSTAAVGSGMGTGQRAGSPAAGPPLQPREPLSYSRLQRKSLAVDFVVPSLFRVYARDLLLPPSSSELKAGRPEARGSLALDCAPLLRLLGPAPGVSW   110
ALK-205      ..............................................................................................................     0
ALK-202      ..............................................................................................................     0
ALK-203      ..............................................................................................................     0
```

```
logo

             TAGSPAPAEARTLSRVLKGGSVRKLRRAKQLVLELGEEAILEGCVGPPGEAAVGLLQFNLSELFSWWIRQGEGRLRIRLMPEKKASEVGREGRLSAAIRASQPRLLFQIF
CRIZOTINIB   ..............................................................................................................     0
PF07714      ..............................................................................................................     0
PF00629      ..............................................................................................................     0
PF07714.1    ..............................................................................................................     0
PF12810      ..............................................................................................................     0
PF00629.1    ..............................................................................................................     0
ALK-201      TAGSPAPAEARTLSRVLKGGSVRKLRRAKQLVLELGEEAILEGCVGPPGEAAVGLLQFNLSELFSWWIRQGEGRLRIRLMPEKKASEVGREGRLSAAIRASQPRLLFQIF   220
ALK-205      ..............................................................................................................     0
ALK-202      ..............................................................................................................     0
ALK-203      ..............................................................................................................     0
```

```
logo

             GTGHSSLESPTNMPSPSPDYFTWNLTWIMKDSFPFLSHRSRYGLECSFDFPCELEYSPPLHDLRNQSWSWRRIPSEEASQMDLLDGPGAERSKEMPRGSFLLLNTSADSK
CRIZOTINIB   ..............................................................................................................     0
PF07714      ..............................................................................................................     0
PF00629      .............................................CSFDFPCELEYSPPLHDLRNQSWSWRRIPSEEASQMDLLDGPGAERSKEMPRGSFLLLNTSADSK    65
PF07714.1    ..............................................................................................................     0
PF12810      ..............................................................................................................     0
PF00629.1    ..............................................................................................................     0
ALK-201      GTGHSSLESPTNMPSPSPDYFTWNLTWIMKDSFPFLSHRSRYGLECSFDFPCELEYSPPLHDLRNQSWSWRRIPSEEASQMDLLDGPGAERSKEMPRGSFLLLNTSADSK   330
ALK-205      ..............................................................................................................     0
ALK-202      ..............................................................................................................     0
ALK-203      ..............................................................................................................     0
```

```
logo

             HTILSPWMRSSSEHCTLAVSVHRHLQPSGRYIAQLLPHNEAAREILLMPTPGKHGWTVLQGRIGRPDNPFRVALEYISSGNRSLSAVDFFALKNCSEGTSPGSKMALQSS
CRIZOTINIB   ..............................................................................................................     0
PF07714      ..............................................................................................................     0
PF00629      HTILSPWMRSSSEHCTLAVSVHRHLQPSGRYIAQLLPHNEAAREILLMPTPGKHGWTVLQGRIGRPDNPFRVALEYISSGNRSLSAVDFFALKNC...............   160
PF07714.1    ..............................................................................................................     0
PF12810      ..............................................................................................................     0
PF00629.1    ..............................................................................................................     0
ALK-201      HTILSPWMRSSSEHCTLAVSVHRHLQPSGRYIAQLLPHNEAAREILLMPTPGKHGWTVLQGRIGRPDNPFRVALEYISSGNRSLSAVDFFALKNCSEGTSPGSKMALQSS   440
ALK-205      ...............................................MPTPGKHGWTVLQGRIGRPDNPFRVALEYISSGNRSLSAVDFFALKNCSEGTSPGSKMALQSS    63
ALK-202      ..............................................................................................................     0
ALK-203      ..............................................................................................................     0
```

```
logo
            FTCWNGTVLQLGQACDFHQDCAQGEDESQMCRKLPVGFYCNFEDGFCGWTQGTLSPHTPQWQVRTLKDARFQDHQDHALLLSTTDVPASESATVTSATFPAPIKSSPCEL
CRIZOTINIB  ..............................................................................................................    0
PF07714     ..............................................................................................................    0
PF00629     .......................................CNFEDGFCGWTQGTLSPHTPQWQVRTLKDARFQDHQDHALLLSTTDVPASESATVTSATFPAPIKSSPCEL  231
PF07714.1   ..............................................................................................................    0
PF12810     ..............................................................................................................    0
PF00629.1   .......................................CNFEDGFCGWTQGTLSPHTPQWQVRTLKDARFQDHQDHALLLSTTDVPASESATVTSATFPAPIKSSPCEL   71
ALK-201     FTCWNGTVLQLGQACDFHQDCAQGEDESQMCRKLPVGFYCNFEDGFCGWTQGTLSPHTPQWQVRTLKDARFQDHQDHALLLSTTDVPASESATVTSATFPAPIKSSPCEL  550
ALK-205     FTCWNGTVLQLGQACDFHQDCAQGEDESQMCRKLPVGFYCNFEDGFCGWTQGTLSPHTPQWQVRTLKDARFQDHQDHALLLSTTDVPASESATVTSATFPAPIKSSPCEL  173
ALK-202     ..............................................................................................................    0
ALK-203     ..............................................................................................................    0

logo
            RMSWLIRGVLRGNVSLVLVENKTGKEQGRMVWHVAAYEGLSLWQWMVLPLLDVSDRFWLQMVAWWGQGSRAIVAFDNISISLDCYLTISGEDKILQNTAPKSRNLFERNP
CRIZOTINIB  ..............................................................................................................    0
PF07714     ..............................................................................................................    0
PF00629     RMSWLIRGVLRGNVSLVLVENKTGKEQGRMVWHVAAYEGLSLWQWMVLPLLDVSDRFWLQMVAWWGQGSRAIVAFDNISISLDC..........................  315
PF07714.1   ..............................................................................................................    0
PF12810     ..............................................................................................................    0
PF00629.1   RMSWLIRGVLRGNVSLVLVENKTGKEQGRMVWHVAAYEGLSLWQWMVLPLLDVSDRFWLQMVAWWGQGSRAIVAFDNISISLDC..........................  155
ALK-201     RMSWLIRGVLRGNVSLVLVENKTGKEQGRMVWHVAAYEGLSLWQWMVLPLLDVSDRFWLQMVAWWGQGSRAIVAFDNISISLDCYLTISGEDKILQNTAPKSRNLFERNP  660
ALK-205     RMSWLIRGVLRGNVSLVLVENKTGKEQGRMVWHVAAYEGLSLWQWMVLPLLDVSDRFWLQMVAWWGQGSRAIVAFDNISISLDCYLTISGEDKILQNTAPKSRNLFERNP  283
ALK-202     ..............................................................................................................    0
ALK-203     ..............................................................................................................    0

logo
            NKELKPGENSPRQTPIFDPTVHWLFTTCGASGPHGPTQAQCNNAYQNSNLSVEVGSEGPLKGIQIWKVPATDTYSISGYGAAGGKGGKNTMMRSHGVSVLGIFNLEKDDM
CRIZOTINIB  ...........................................................L..................................................    1
PF07714     ..............................................................................................................    0
PF00629     ..............................................................................................................  315
PF07714.1   ..............................................................................................................    0
PF12810     ......................................................................TDTYSISGYGAAGGKGGKNTMMRSHGVSVLGIFNLEKDDM   40
PF00629.1   ..............................................................................................................  155
ALK-201     NKELKPGENSPRQTPIFDPTVHWLFTTCGASGPHGPTQAQCNNAYQNSNLSVEVGSEGPLKGIQIWKVPATDTYSISGYGAAGGKGGKNTMMRSHGVSVLGIFNLEKDDM  770
ALK-205     NKELKPGENSPRQTPIFDPTVHWLFTTCGASGPHGPTQAQCNNAYQNSNLSVEVGSEGPLKGIQIWKVPATDTYSISGYGAAGGKGGKNTMMRSHGVSVLGIFNLEKDDM  393
ALK-202     ..............................................................................................................    0
ALK-203     ..............................................................................................................    0

logo
            LYILVGQQGEDACPSTNQLIQKVCIGENNVIEEEIRVNRSVHEWAGGGGGGGGATYVFKMKDGVPVPLIIAAGGGGRAYGAKTDTFHPERLENNSSVLGLNGNSGAAGGG
CRIZOTINIB  ..............................................................................................................    1
PF07714     ..............................................................................................................    0
PF00629     ..............................................................................................................  315
PF07714.1   ..............................................................................................................    0
PF12810     LYILVGQQGEDACPSTNQLIQKVCIGENNVIEEEIRVNRSVHEWAGGGGGGGGATYVFKMKDGVPVPLIIAAGGGGRAYGAKTDTFHPERLENNSSVLGLNGNSGAAGGG  150
PF00629.1   ..............................................................................................................  155
ALK-201     LYILVGQQGEDACPSTNQLIQKVCIGENNVIEEEIRVNRSVHEWAGGGGGGGGATYVFKMKDGVPVPLIIAAGGGGRAYGAKTDTFHPERLENNSSVLGLNGNSGAAGGG  880
ALK-205     LYILVGQQGEDACPSTNQLIQKVCIGENNVIEEEIRVNRSVHEWAGGGGGGGGATYVFKMKDGVPVPLIIAAGGGGRAYGAKTDTFHPERLENNSSVLGLNGNSGAAGGG  503
ALK-202     ..............................................................................................................    0
ALK-203     ..............................................................................................................    0
```

```
logo        GGWNDNTSLLWAGKSLQEGATGGHSCPQAMKKWGWETRGGFGGGGGGCSSGGGGGGYIGGNAASNNDPEMDGEDGVSFISPLGILYTPALKVMEGHGEVNIKHYLNCSHC
CRIZOTINIB  ..............................................................................................................    1
PF07714     ..............................................................................................................    0
PF00629     ..............................................................................................................  315
PF07714.1   ..............................................................................................................    0
PF12810     GGWNDNTSLLWAGKSLQEGATGGHSCPQAMKKWGWETRGGFGGGGGGCSSGGGGGGYIGGNAASNNDPEMDGEDGVSFISP.............................  231
PF00629.1   ..............................................................................................................  155
ALK-201     GGWNDNTSLLWAGKSLQEGATGGHSCPQAMKKWGWETRGGFGGGGGGCSSGGGGGGYIGGNAASNNDPEMDGEDGVSFISPLGILYTPALKVMEGHGEVNIKHYLNCSHC  990
ALK-205     GGWNDNTSLLWAGKSLQEGATGGHSCPQAMKKWGWETRGGFGGGGGGCSSGGGGGGYIGGNAASNNDPEMDGEDGVSFISPLGILYTPALKVMEGHGEVNIKHYLNCSHC  613
ALK-202     ..............................................................................................................    0
ALK-203     ..............................................................................................................    0

logo        EVDECHMDPESHKVICFCDHGTVLAEDGVSCIVSPTPEPHLPLSLILSVVTSALVAALVLAFSGIMIVYRRKHQELQAMQMELQSPEYKLSKLRTSTIMTDYNPNYCFAG
CRIZOTINIB  ..............................................................................................................    1
PF07714     ..............................................................................................................    0
PF00629     ..............................................................................................................  315
PF07714.1   ..............................................................................................................    0
PF12810     ..............................................................................................................  231
PF00629.1   ..............................................................................................................  155
ALK-201     EVDECHMDPESHKVICFCDHGTVLAEDGVSCIVSPTPEPHLPLSLILSVVTSALVAALVLAFSGIMIVYRRKHQELQAMQMELQSPEYKLSKLRTSTIMTDYNPNYCFAG 1100
ALK-205     EVDECHMDPESHKVICFCDHGTVLAEDGVSCIVSPTPEPHLPLSLILSVVTSALVAALVLAFSGIMIVYRRKHQELQAMQMELQSPEYKLSKLRTSTIMTDYNPNYCFAG  723
ALK-202     ................................................MVT.TCSAIYTFQF.IRSSVYRRKHQELQAMQMELQSPEYKLSKLRTSTIMTDYNPNYCFAG   60
ALK-203     ..............................................................................................................    0

logo        KTSSISDLKEVPRKNITLIRGLGHGAFGEVYEGQVSGMPNDPSPLQVAVKTLPEVCSEQDELDFLMEALIISKFNHQNIV..RCIGVSLQ.........SLPRFILLELM
CRIZOTINIB  .............................V.................A.K........................................................LE.M    7
PF07714     ................TLIRGLGHGAFGEVYEGQVSGMPNDPSPLQVAVKTLPEVCSEQDELDFLMEALIISKFNHQNIV..RCIGVSLQ.........SLPRFILLELM   83
PF00629     ..............................................................................................................  315
PF07714.1   ................TLIRGLGHGAFGEVYEGQVSGMPNDPSPLQVAVKTLPEVCSEQDELDFLMEALIIS......................................   56
PF12810     ..............................................................................................................  231
PF00629.1   ..............................................................................................................  155
ALK-201     KTSSISDLKEVPRKNITLIRGLGHGAFGEVYEGQVSGMPNDPSPLQVAVKTLPEVCSEQDELDFLMEALIISKFNHQNIV..RCIGVSLQ.........SLPRFILLELM 1199
ALK-205     KTSSISDLKEVPRKNITLIRGLGHGAFGEVYEGQVSGMPNDPSPLQVAVKTLPEVCSEQDELDFLMEALIISKFNHQNIV..RCIGVSLQ.........SLPRFILLELM  822
ALK-202     KTSSISDLKEVPRKNITLIRGLGHGAFGEVYEGQVSGMPNDPSPLQVAVKTLPEVCSEQDELDFLMEALIISKFNHQNIV..RCIGVSLQ.........SLPRFILLELM  159
ALK-203     ..SSISDLKEVPRKNITLIRGLGHGAFGEVYEGQVSGMPNDPSPLQVAVKTLPEVCSEQDELDFLMEALIISLSWKQLFCFTFCLRPSWEKSTMNSSTQSTHQYL..RKT  106

logo        AGGDLKSFLRETRPRPSQPSSLAMLDLLHVARDIACGCQYLEENHFIHRDIAARNCLLTCPGPGRVAKIGDFGMARDIYRASYYRKGGCAMLPVKWMPPEAFMEGIFTSK
CRIZOTINIB  AGG..................................................RN.L............GD.......................................   15
PF07714     AGGDLKSFLRETRPRPSQPSSLAMLDLLHVARDIACGCQYLEENHFIHRDIAARNCLLTCPGPGRVAKIGDFGMARDIYRASYYRKGGCAMLPVKWMPPEAFMEGIFTSK  193
PF00629     ..............................................................................................................  315
PF07714.1   ..............................................................................................................   56
PF12810     ..............................................................................................................  231
PF00629.1   ..............................................................................................................  155
ALK-201     AGGDLKSFLRETRPRPSQPSSLAMLDLLHVARDIACGCQYLEENHFIHRDIAARNCLLTCPGPGRVAKIGDFGMARDIYRASYYRKGGCAMLPVKWMPPEAFMEGIFTSK 1309
ALK-205     AGGDLKSFLRETRPRPSQPSSLAMLDLLHVARDIACGCQYLEENHFIHRDIAARNCLLTCPGPGRVAKIGDFGMARDIYRASYYRKGGCAMLPVKWMPPEAFMEGIFTSK  932
ALK-202     AGGDLKSFLRETRPRPSQPSSLAMLDLLHVARDIACGCQYLEENHFIHRDIAARNCLLTCPGPGRVAKIGDFGMARDIYRASYYRKGGCAMLPVKWMPPEAFMEGIFTSK  269
ALK-203     LSCPLRHFLTQVPEQHPHSSSHSPA..........NG...........TPVEPAVVLPTCS.PARF.............................WV.............  152
```

```
logo
             TDTWSFGVLLWEIFSLGYMPYPSKSNQEVLEFVTSGGRMDPPKNCPGPVYRIMTQCWQHQPEDRPNFAIILERIEYCTQDPDVINTALPIEYGPLVEEEEKVPVRPKDPE
CRIZOTINIB   ..............................................................................................................    15
PF07714      TDTWSFGVLLWEIFSLGYMPYPSKSNQEVLEFVTSGGRMDPPKNCPGPVYRIMTQCWQHQPEDRPNFAIILER.....................................   266
PF00629      ..............................................................................................................   315
PF07714.1    ..............................................................................................................    56
PF12810      ..............................................................................................................   231
PF00629.1    ..............................................................................................................   155
ALK-201      TDTWSFGVLLWEIFSLGYMPYPSKSNQEVLEFVTSGGRMDPPKNCPGPVYRIMTQCWQHQPEDRPNFAIILERIEYCTQDPDVINTALPIEYGPLVEEEEKVPVRPKDPE  1419
ALK-205      TDTWSFGVLLWEIFSLGYMPYPSKSNQEVLEFVTSGGRMDPPKNCPGPVYRIMTQCWQHQPEDRPNFAIILERIEYCTQDPDVINTALPIEYGPLVEEEEKVPVRPKDPE  1042
ALK-202      TDTWSFGVLLWEIFSLGYMPYPSKSNQEVLEFVTSGGRMDPPKNCPGPVYRIMTQCWQHQPEDRPNFAIILERIEYCTQDPDVINTALPIEYGPLVEEEEKVPVRPKDPE   379
ALK-203      ..............................................................................................................   152

logo
             GVPPLLVSQQAKREEERSPAAPPPLPTTSSGKAAKKPTAAEISVRVPRGPAVEGGHVNMAFSQSNPPSELHKVHGSRNKPTSLWNPTYGSWFTEKPTKKNNPIAKKEPHD
CRIZOTINIB   ..............................................................................................................    15
PF07714      ..............................................................................................................   266
PF00629      ..............................................................................................................   315
PF07714.1    ..............................................................................................................    56
PF12810      ..............................................................................................................   231
PF00629.1    ..............................................................................................................   155
ALK-201      GVPPLLVSQQAKREEERSPAAPPPLPTTSSGKAAKKPTAAEISVRVPRGPAVEGGHVNMAFSQSNPPSELHKVHGSRNKPTSLWNPTYGSWFTEKPTKKNNPIAKKEPHD  1529
ALK-205      GVPPLLVSQQAKREEERSPAAPPPLPTTSSGKAAKKPTAAEISVRVPRGPAVEGGHVNMAFSQSNPPSELHKVHGSRNKPTSLWNPTYGSWFTEKPTKKNNPIAKKEPHD  1152
ALK-202      GVPPLLVSQQAKREEERSPAAPPPLPTTSSGKAAKKPTAAEISVRVPRGPAVEGGHVNMAFSQSNPPSELHKVHGSRNKPTSLWNPTYGSWFTEKPTKKNNPIAKKEPHD   489
ALK-203      ..............................................................................................................   152

logo
             RGNLGLEGSCTVPPNVATGRLPGASLLLEPSSLTANMKEVPLFRLRHFPCGNVNYGYQQQGLPLEAATAPGAGHYEDTILKSKNSMNQPGP
CRIZOTINIB   ...........................................................................................    15
PF07714      ...........................................................................................   266
PF00629      ...........................................................................................   315
PF07714.1    ...........................................................................................    56
PF12810      ...........................................................................................   231
PF00629.1    ...........................................................................................   155
ALK-201      RGNLGLEGSCTVPPNVATGRLPGASLLLEPSSLTANMKEVPLFRLRHFPCGNVNYGYQQQGLPLEAATAPGAGHYEDTILKSKNSMNQPGP  1620
ALK-205      RGNLGLEGSCTVPPNVATGRLPGASLLLEPSSLTANMKEVPLFRLRHFPCGNVNYGYQQQGLPLEAATAPGAGHYEDTILKSKNSMNQPGP  1243
ALK-202      RGNLGLEGSCTVPPNVATGRLPGASLLLEPSSLTANMKEVPLFRLRHFPCGNVNYGYQQQGLPLEAATAPGAGHYEDTILKSKNSMNQPGP   580
ALK-203      ...........................................................................................   152
```

- X non conserved
- X similar
- X $\geq$0% conserved
- X $\geq$50% conserved

```
logo

              MGAIGLLWLLPLLLSTAAVGSGMGTGQRAGSPAAGPPLQPREPLSYSRLQRKSLAVDFVVPSLFRVYARDLLLPPSSSELKAGRPEARGSLALDCAPLLRLLGPAPGVSW
ENTRECTINIB   ..............................................................................................................    0
PF07714       ..............................................................................................................    0
PF00629       ..............................................................................................................    0
PF07714.1     ..............................................................................................................    0
PF12810       ..............................................................................................................    0
PF00629.1     ..............................................................................................................    0
ALK-201       MGAIGLLWLLPLLLSTAAVGSGMGTGQRAGSPAAGPPLQPREPLSYSRLQRKSLAVDFVVPSLFRVYARDLLLPPSSSELKAGRPEARGSLALDCAPLLRLLGPAPGVSW  110
ALK-205       ..............................................................................................................    0
ALK-202       ..............................................................................................................    0
ALK-203       ..............................................................................................................    0

logo

              TAGSPAPAEARTLSRVLKGGSVRKLRRAKQLVLELGEEAILEGCVGPPGEAAVGLLQFNLSELFSWWIRQGEGRLRIRLMPEKKASEVGREGRLSAAIRASQPRLLFQIF
ENTRECTINIB   ..............................................................................................................    0
PF07714       ..............................................................................................................    0
PF00629       ..............................................................................................................    0
PF07714.1     ..............................................................................................................    0
PF12810       ..............................................................................................................    0
PF00629.1     ..............................................................................................................    0
ALK-201       TAGSPAPAEARTLSRVLKGGSVRKLRRAKQLVLELGEEAILEGCVGPPGEAAVGLLQFNLSELFSWWIRQGEGRLRIRLMPEKKASEVGREGRLSAAIRASQPRLLFQIF  220
ALK-205       ..............................................................................................................    0
ALK-202       ..............................................................................................................    0
ALK-203       ..............................................................................................................    0

logo

              GTGHSSLESPTNMPSPSPDYFTWNLTWIMKDSFPFLSHRSRYGLECSFDFPCELEYSPPLHDLRNQSWSWRRIPSEEASQMDLLDGPGAERSKEMPRGSFLLLNTSADSK
ENTRECTINIB   ..............................................................................................................    0
PF07714       ..............................................................................................................    0
PF00629       .............................................CSFDFPCELEYSPPLHDLRNQSWSWRRIPSEEASQMDLLDGPGAERSKEMPRGSFLLLNTSADSK   65
PF07714.1     ..............................................................................................................    0
PF12810       ..............................................................................................................    0
PF00629.1     ..............................................................................................................    0
ALK-201       GTGHSSLESPTNMPSPSPDYFTWNLTWIMKDSFPFLSHRSRYGLECSFDFPCELEYSPPLHDLRNQSWSWRRIPSEEASQMDLLDGPGAERSKEMPRGSFLLLNTSADSK  330
ALK-205       ..............................................................................................................    0
ALK-202       ..............................................................................................................    0
ALK-203       ..............................................................................................................    0

logo

              HTILSPWMRSSSEHCTLAVSVHRHLQPSGRYIAQLLPHNEAAREILLMPTPGKHGWTVLQGRIGRPDNPFRVALEYISSGNRSLSAVDFFALKNCSEGTSPGSKMALQSS
ENTRECTINIB   ..............................................................................................................    0
PF07714       ..............................................................................................................    0
PF00629       HTILSPWMRSSSEHCTLAVSVHRHLQPSGRYIAQLLPHNEAAREILLMPTPGKHGWTVLQGRIGRPDNPFRVALEYISSGNRSLSAVDFFALKNC...............  160
PF07714.1     ..............................................................................................................    0
PF12810       ..............................................................................................................    0
PF00629.1     ..............................................................................................................    0
ALK-201       HTILSPWMRSSSEHCTLAVSVHRHLQPSGRYIAQLLPHNEAAREILLMPTPGKHGWTVLQGRIGRPDNPFRVALEYISSGNRSLSAVDFFALKNCSEGTSPGSKMALQSS  440
ALK-205       ...............................................MPTPGKHGWTVLQGRIGRPDNPFRVALEYISSGNRSLSAVDFFALKNCSEGTSPGSKMALQSS   63
ALK-202       ..............................................................................................................    0
ALK-203       ..............................................................................................................    0
```

```
logo
              FTCWNGTVLQLGQACDFHQDCAQGEDESQMCRKLPVGFYCNFEDGFCGWTQGTLSPHTPQWQVRTLKDARFQDHQDHALLLSTTDVPASESATVTSATFPAPIKSSPCEL
ENTRECTINIB   ..............................................................................................................     0
PF07714       ..............................................................................................................     0
PF00629       .......................................CNFEDGFCGWTQGTLSPHTPQWQVRTLKDARFQDHQDHALLLSTTDVPASESATVTSATFPAPIKSSPCEL   231
PF07714.1     ..............................................................................................................     0
PF12810       ..............................................................................................................     0
PF00629.1     .......................................CNFEDGFCGWTQGTLSPHTPQWQVRTLKDARFQDHQDHALLLSTTDVPASESATVTSATFPAPIKSSPCEL    71
ALK-201       FTCWNGTVLQLGQACDFHQDCAQGEDESQMCRKLPVGFYCNFEDGFCGWTQGTLSPHTPQWQVRTLKDARFQDHQDHALLLSTTDVPASESATVTSATFPAPIKSSPCEL   550
ALK-205       FTCWNGTVLQLGQACDFHQDCAQGEDESQMCRKLPVGFYCNFEDGFCGWTQGTLSPHTPQWQVRTLKDARFQDHQDHALLLSTTDVPASESATVTSATFPAPIKSSPCEL   173
ALK-202       ..............................................................................................................     0
ALK-203       ..............................................................................................................     0

logo
              RMSWLIRGVLRGNVSLVLVENKTGKEQGRMVWHVAAYEGLSLWQWMVLPLLDVSDRFWLQMVAWWGQGSRAIVAFDNISISLDCYLTISGEDKILQNTAPKSRNLFERNP
ENTRECTINIB   ..............................................................................................................     0
PF07714       ..............................................................................................................     0
PF00629       RMSWLIRGVLRGNVSLVLVENKTGKEQGRMVWHVAAYEGLSLWQWMVLPLLDVSDRFWLQMVAWWGQGSRAIVAFDNISISLDC..........................   315
PF07714.1     ..............................................................................................................     0
PF12810       ..............................................................................................................     0
PF00629.1     RMSWLIRGVLRGNVSLVLVENKTGKEQGRMVWHVAAYEGLSLWQWMVLPLLDVSDRFWLQMVAWWGQGSRAIVAFDNISISLDC..........................   155
ALK-201       RMSWLIRGVLRGNVSLVLVENKTGKEQGRMVWHVAAYEGLSLWQWMVLPLLDVSDRFWLQMVAWWGQGSRAIVAFDNISISLDCYLTISGEDKILQNTAPKSRNLFERNP   660
ALK-205       RMSWLIRGVLRGNVSLVLVENKTGKEQGRMVWHVAAYEGLSLWQWMVLPLLDVSDRFWLQMVAWWGQGSRAIVAFDNISISLDCYLTISGEDKILQNTAPKSRNLFERNP   283
ALK-202       ..............................................................................................................     0
ALK-203       ..............................................................................................................     0

logo
              NKELKPGENSPRQTPIFDPTVHWLFTTCGASGPHGPTQAQCNNAYQNSNLSVEVGSEGPLKGIQIWKVPATDTYSISGYGAAGGKGGKNTMMRSHGVSVLGIFNLEKDDM
ENTRECTINIB   ..............................................................................................................     0
PF07714       ..............................................................................................................     0
PF00629       ..............................................................................................................   315
PF07714.1     ..............................................................................................................     0
PF12810       ......................................................................TDTYSISGYGAAGGKGGKNTMMRSHGVSVLGIFNLEKDDM    40
PF00629.1     ..............................................................................................................   155
ALK-201       NKELKPGENSPRQTPIFDPTVHWLFTTCGASGPHGPTQAQCNNAYQNSNLSVEVGSEGPLKGIQIWKVPATDTYSISGYGAAGGKGGKNTMMRSHGVSVLGIFNLEKDDM   770
ALK-205       NKELKPGENSPRQTPIFDPTVHWLFTTCGASGPHGPTQAQCNNAYQNSNLSVEVGSEGPLKGIQIWKVPATDTYSISGYGAAGGKGGKNTMMRSHGVSVLGIFNLEKDDM   393
ALK-202       ..............................................................................................................     0
ALK-203       ..............................................................................................................     0

logo
              LYILVGQQGEDACPSTNQLIQKVCIGENNVIEEEIRVNRSVHEWAGGGGGGGGATYVFKMKDGVPVPLIIAAGGGGRAYGAKTDTFHPERLENNSSVLGLNGNSGAAGGG
ENTRECTINIB   ..............................................................................................................     0
PF07714       ..............................................................................................................     0
PF00629       ..............................................................................................................   315
PF07714.1     ..............................................................................................................     0
PF12810       LYILVGQQGEDACPSTNQLIQKVCIGENNVIEEEIRVNRSVHEWAGGGGGGGGATYVFKMKDGVPVPLIIAAGGGGRAYGAKTDTFHPERLENNSSVLGLNGNSGAAGGG   150
PF00629.1     ..............................................................................................................   155
ALK-201       LYILVGQQGEDACPSTNQLIQKVCIGENNVIEEEIRVNRSVHEWAGGGGGGGGATYVFKMKDGVPVPLIIAAGGGGRAYGAKTDTFHPERLENNSSVLGLNGNSGAAGGG   880
ALK-205       LYILVGQQGEDACPSTNQLIQKVCIGENNVIEEEIRVNRSVHEWAGGGGGGGGATYVFKMKDGVPVPLIIAAGGGGRAYGAKTDTFHPERLENNSSVLGLNGNSGAAGGG   503
ALK-202       ..............................................................................................................     0
ALK-203       ..............................................................................................................     0
```

logo

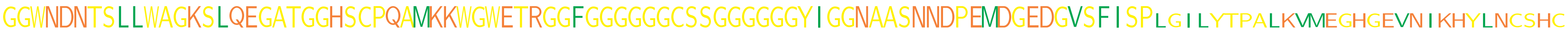

```
ENTRECTINIB  ..............................................................................................................     0
PF07714      ..............................................................................................................     0
PF00629      ..............................................................................................................   315
PF07714.1    ..............................................................................................................     0
PF12810      GGWNDNTSLLWAGKSLQEGATGGHSCPQAMKKWGWETRGGFGGGGGGCSSGGGGGGYIGGNAASNNDPEMDGEDGVSFISP............................   231
PF00629.1    ..............................................................................................................   155
ALK-201      GGWNDNTSLLWAGKSLQEGATGGHSCPQAMKKWGWETRGGFGGGGGGCSSGGGGGGYIGGNAASNNDPEMDGEDGVSFISPLGILYTPALKVMEGHGEVNIKHYLNCSHC   990
ALK-205      GGWNDNTSLLWAGKSLQEGATGGHSCPQAMKKWGWETRGGFGGGGGGCSSGGGGGGYIGGNAASNNDPEMDGEDGVSFISPLGILYTPALKVMEGHGEVNIKHYLNCSHC   613
ALK-202      ..............................................................................................................     0
ALK-203      ..............................................................................................................     0
```

logo

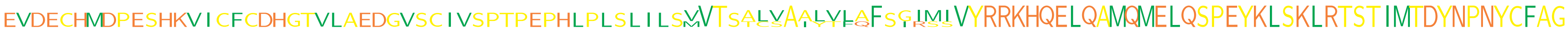

```
ENTRECTINIB  ..............................................................................................................     0
PF07714      ..............................................................................................................     0
PF00629      ..............................................................................................................   315
PF07714.1    ..............................................................................................................     0
PF12810      ..............................................................................................................   231
PF00629.1    ..............................................................................................................   155
ALK-201      EVDECHMDPESHKVICFCDHGTVLAEDGVSCIVSPTPEPHLPLSLILSVVTSALVAALVLAFSGIMIVYRRKHQELQAMQMELQSPEYKLSKLRTSTIMTDYNPNYCFAG  1100
ALK-205      EVDECHMDPESHKVICFCDHGTVLAEDGVSCIVSPTPEPHLPLSLILSVVTSALVAALVLAFSGIMIVYRRKHQELQAMQMELQSPEYKLSKLRTSTIMTDYNPNYCFAG   723
ALK-202      ................................................MVT.TCSAIYTFQF.IRSSVYRRKHQELQAMQMELQSPEYKLSKLRTSTIMTDYNPNYCFAG    60
ALK-203      ..............................................................................................................     0
```

logo

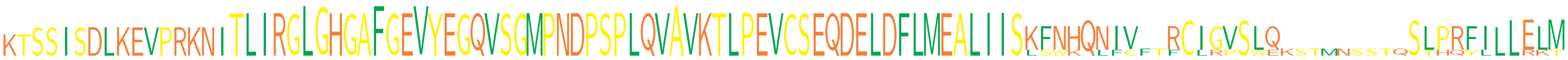

```
ENTRECTINIB  .....................LG...F..V.................A..........................................................LE.M     8
PF07714      ................TLIRGLGHGAFGEVYEGQVSGMPNDPSPLQVAVKTLPEVCSEQDELDFLMEALIISKFNHQNIV..RCIGVSLQ.........SLPRFILLELM    83
PF00629      ..............................................................................................................   315
PF07714.1    ................TLIRGLGHGAFGEVYEGQVSGMPNDPSPLQVAVKTLPEVCSEQDELDFLMEALIIS......................................    56
PF12810      ..............................................................................................................   231
PF00629.1    ..............................................................................................................   155
ALK-201      KTSSISDLKEVPRKNITLIRGLGHGAFGEVYEGQVSGMPNDPSPLQVAVKTLPEVCSEQDELDFLMEALIISKFNHQNIV..RCIGVSLQ.........SLPRFILLELM  1199
ALK-205      KTSSISDLKEVPRKNITLIRGLGHGAFGEVYEGQVSGMPNDPSPLQVAVKTLPEVCSEQDELDFLMEALIISKFNHQNIV..RCIGVSLQ.........SLPRFILLELM   822
ALK-202      KTSSISDLKEVPRKNITLIRGLGHGAFGEVYEGQVSGMPNDPSPLQVAVKTLPEVCSEQDELDFLMEALIISKFNHQNIV..RCIGVSLQ.........SLPRFILLELM   159
ALK-203      ..SSISDLKEVPRKNITLIRGLGHGAFGEVYEGQVSGMPNDPSPLQVAVKTLPEVCSEQDELDFLMEALIISLSWKQLFCFTFCLRPSWEKSTMNSSTQSTHQYL..RKT   106
```

logo

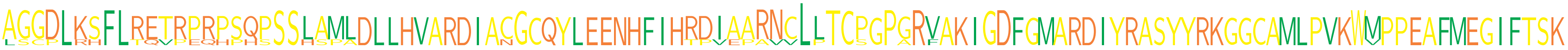

```
ENTRECTINIB  AGGD.................................................RN.L............GD.......................................    17
PF07714      AGGDLKSFLRETRPRPSQPSSLAMLDLLHVARDIACGCQYLEENHFIHRDIAARNCLLTCPGPGRVAKIGDFGMARDIYRASYYRKGGCAMLPVKWMPPEAFMEGIFTSK   193
PF00629      ..............................................................................................................   315
PF07714.1    ..............................................................................................................    56
PF12810      ..............................................................................................................   231
PF00629.1    ..............................................................................................................   155
ALK-201      AGGDLKSFLRETRPRPSQPSSLAMLDLLHVARDIACGCQYLEENHFIHRDIAARNCLLTCPGPGRVAKIGDFGMARDIYRASYYRKGGCAMLPVKWMPPEAFMEGIFTSK  1309
ALK-205      AGGDLKSFLRETRPRPSQPSSLAMLDLLHVARDIACGCQYLEENHFIHRDIAARNCLLTCPGPGRVAKIGDFGMARDIYRASYYRKGGCAMLPVKWMPPEAFMEGIFTSK   932
ALK-202      AGGDLKSFLRETRPRPSQPSSLAMLDLLHVARDIACGCQYLEENHFIHRDIAARNCLLTCPGPGRVAKIGDFGMARDIYRASYYRKGGCAMLPVKWMPPEAFMEGIFTSK   269
ALK-203      LSCPLRHFLTQVPEQHPHSSSHSPA..........NG...........TPVEPAVVLPTCS.PARF.............................WV.............   152
```

```
logo
             TDTWSFGVLLWEIFSLGYMPYPSKSNQEVLEFVTSGGRMDPPKNCPGPVYRIMTQCWQHQPEDRPNFAIILERIEYCTQDPDVINTALPIEYGPLVEEEEKVPVRPKDPE
ENTRECTINIB  ..............................................................................................................    17
PF07714      TDTWSFGVLLWEIFSLGYMPYPSKSNQEVLEFVTSGGRMDPPKNCPGPVYRIMTQCWQHQPEDRPNFAIILER.....................................   266
PF00629      ..............................................................................................................   315
PF07714.1    ..............................................................................................................    56
PF12810      ..............................................................................................................   231
PF00629.1    ..............................................................................................................   155
ALK-201      TDTWSFGVLLWEIFSLGYMPYPSKSNQEVLEFVTSGGRMDPPKNCPGPVYRIMTQCWQHQPEDRPNFAIILERIEYCTQDPDVINTALPIEYGPLVEEEEKVPVRPKDPE  1419
ALK-205      TDTWSFGVLLWEIFSLGYMPYPSKSNQEVLEFVTSGGRMDPPKNCPGPVYRIMTQCWQHQPEDRPNFAIILERIEYCTQDPDVINTALPIEYGPLVEEEEKVPVRPKDPE  1042
ALK-202      TDTWSFGVLLWEIFSLGYMPYPSKSNQEVLEFVTSGGRMDPPKNCPGPVYRIMTQCWQHQPEDRPNFAIILERIEYCTQDPDVINTALPIEYGPLVEEEEKVPVRPKDPE   379
ALK-203      ..............................................................................................................   152

logo
             GVPPLLVSQQAKREEERSPAAPPPLPTTSSGKAAKKPTAAEISVRVPRGPAVEGGHVNMAFSQSNPPSELHKVHGSRNKPTSLWNPTYGSWFTEKPTKKNNPIAKKEPHD
ENTRECTINIB  ..............................................................................................................    17
PF07714      ..............................................................................................................   266
PF00629      ..............................................................................................................   315
PF07714.1    ..............................................................................................................    56
PF12810      ..............................................................................................................   231
PF00629.1    ..............................................................................................................   155
ALK-201      GVPPLLVSQQAKREEERSPAAPPPLPTTSSGKAAKKPTAAEISVRVPRGPAVEGGHVNMAFSQSNPPSELHKVHGSRNKPTSLWNPTYGSWFTEKPTKKNNPIAKKEPHD  1529
ALK-205      GVPPLLVSQQAKREEERSPAAPPPLPTTSSGKAAKKPTAAEISVRVPRGPAVEGGHVNMAFSQSNPPSELHKVHGSRNKPTSLWNPTYGSWFTEKPTKKNNPIAKKEPHD  1152
ALK-202      GVPPLLVSQQAKREEERSPAAPPPLPTTSSGKAAKKPTAAEISVRVPRGPAVEGGHVNMAFSQSNPPSELHKVHGSRNKPTSLWNPTYGSWFTEKPTKKNNPIAKKEPHD   489
ALK-203      ..............................................................................................................   152

logo
             RGNLGLEGSCTVPPNVATGRLPGASLLLEPSSLTANMKEVPLFRLRHFPCGNVNYGYQQQGLPLEAATAPGAGHYEDTILKSKNSMNQPGP
ENTRECTINIB  ...........................................................................................    17
PF07714      ...........................................................................................   266
PF00629      ...........................................................................................   315
PF07714.1    ...........................................................................................    56
PF12810      ...........................................................................................   231
PF00629.1    ...........................................................................................   155
ALK-201      RGNLGLEGSCTVPPNVATGRLPGASLLLEPSSLTANMKEVPLFRLRHFPCGNVNYGYQQQGLPLEAATAPGAGHYEDTILKSKNSMNQPGP  1620
ALK-205      RGNLGLEGSCTVPPNVATGRLPGASLLLEPSSLTANMKEVPLFRLRHFPCGNVNYGYQQQGLPLEAATAPGAGHYEDTILKSKNSMNQPGP  1243
ALK-202      RGNLGLEGSCTVPPNVATGRLPGASLLLEPSSLTANMKEVPLFRLRHFPCGNVNYGYQQQGLPLEAATAPGAGHYEDTILKSKNSMNQPGP   580
ALK-203      ...........................................................................................   152

             X  non conserved
             X  similar
             X  ≥ 0% conserved
             X  ≥ 50% conserved
```

```
logo

             MGAIGLLWLLPLLLSTAAVGSGMGTGQRAGSPAAGPPLQPREPLSYSRLQRKSLAVDFVVPSLFRVYARDLLLPPSSSELKAGRPEARGSLALDCAPLLRLLGPAPGVSW
LORLATINIB   ..............................................................................................................    0
PF07714      ..............................................................................................................    0
PF00629      ..............................................................................................................    0
PF07714.1    ..............................................................................................................    0
PF12810      ..............................................................................................................    0
PF00629.1    ..............................................................................................................    0
ALK-201      MGAIGLLWLLPLLLSTAAVGSGMGTGQRAGSPAAGPPLQPREPLSYSRLQRKSLAVDFVVPSLFRVYARDLLLPPSSSELKAGRPEARGSLALDCAPLLRLLGPAPGVSW  110
ALK-205      ..............................................................................................................    0
ALK-202      ..............................................................................................................    0
ALK-203      ..............................................................................................................    0

logo

             TAGSPAPAEARTLSRVLKGGSVRKLRRAKQLVLELGEEAILEGCVGPPGEAAVGLLQFNLSELFSWWIRQGEGRLRIRLMPEKKASEVGREGRLSAAIRASQPRLLFQIF
LORLATINIB   ..............................................................................................................    0
PF07714      ..............................................................................................................    0
PF00629      ..............................................................................................................    0
PF07714.1    ..............................................................................................................    0
PF12810      ..............................................................................................................    0
PF00629.1    ..............................................................................................................    0
ALK-201      TAGSPAPAEARTLSRVLKGGSVRKLRRAKQLVLELGEEAILEGCVGPPGEAAVGLLQFNLSELFSWWIRQGEGRLRIRLMPEKKASEVGREGRLSAAIRASQPRLLFQIF  220
ALK-205      ..............................................................................................................    0
ALK-202      ..............................................................................................................    0
ALK-203      ..............................................................................................................    0

logo

             GTGHSSLESPTNMPSPSPDYFTWNLTWIMKDSFPFLSHRSRYGLECSFDFPCELEYSPPLHDLRNQSWSWRRIPSEEASQMDLLDGPGAERSKEMPRGSFLLLNTSADSK
LORLATINIB   ..............................................................................................................    0
PF07714      ..............................................................................................................    0
PF00629      .............................................CSFDFPCELEYSPPLHDLRNQSWSWRRIPSEEASQMDLLDGPGAERSKEMPRGSFLLLNTSADSK   65
PF07714.1    ..............................................................................................................    0
PF12810      ..............................................................................................................    0
PF00629.1    ..............................................................................................................    0
ALK-201      GTGHSSLESPTNMPSPSPDYFTWNLTWIMKDSFPFLSHRSRYGLECSFDFPCELEYSPPLHDLRNQSWSWRRIPSEEASQMDLLDGPGAERSKEMPRGSFLLLNTSADSK  330
ALK-205      ..............................................................................................................    0
ALK-202      ..............................................................................................................    0
ALK-203      ..............................................................................................................    0

logo

             HTILSPWMRSSSEHCTLAVSVHRHLQPSGRYIAQLLPHNEAAREILLMPTPGKHGWTVLQGRIGRPDNPFRVALEYISSGNRSLSAVDFFALKNCSEGTSPGSKMALQSS
LORLATINIB   ..............................................................................................................    0
PF07714      ..............................................................................................................    0
PF00629      HTILSPWMRSSSEHCTLAVSVHRHLQPSGRYIAQLLPHNEAAREILLMPTPGKHGWTVLQGRIGRPDNPFRVALEYISSGNRSLSAVDFFALKNC...............  160
PF07714.1    ..............................................................................................................    0
PF12810      ..............................................................................................................    0
PF00629.1    ..............................................................................................................    0
ALK-201      HTILSPWMRSSSEHCTLAVSVHRHLQPSGRYIAQLLPHNEAAREILLMPTPGKHGWTVLQGRIGRPDNPFRVALEYISSGNRSLSAVDFFALKNCSEGTSPGSKMALQSS  440
ALK-205      ...............................................MPTPGKHGWTVLQGRIGRPDNPFRVALEYISSGNRSLSAVDFFALKNCSEGTSPGSKMALQSS   63
ALK-202      ..............................................................................................................    0
ALK-203      ..............................................................................................................    0
```

```
logo
            FTCWNGTVLQLGQACDFHQDCAQGEDESQMCRKLPVGFYCNFEDGFCGWTQGTLSPHTPQWQVRTLKDARFQDHQDHALLLSTTDVPASESATVTSATFPAPIKSSPCEL
LORLATINIB  ..............................................................................................................    0
PF07714     ..............................................................................................................    0
PF00629     .......................................CNFEDGFCGWTQGTLSPHTPQWQVRTLKDARFQDHQDHALLLSTTDVPASESATVTSATFPAPIKSSPCEL  231
PF07714.1   ..............................................................................................................    0
PF12810     ..............................................................................................................    0
PF00629.1   .......................................CNFEDGFCGWTQGTLSPHTPQWQVRTLKDARFQDHQDHALLLSTTDVPASESATVTSATFPAPIKSSPCEL   71
ALK-201     FTCWNGTVLQLGQACDFHQDCAQGEDESQMCRKLPVGFYCNFEDGFCGWTQGTLSPHTPQWQVRTLKDARFQDHQDHALLLSTTDVPASESATVTSATFPAPIKSSPCEL  550
ALK-205     FTCWNGTVLQLGQACDFHQDCAQGEDESQMCRKLPVGFYCNFEDGFCGWTQGTLSPHTPQWQVRTLKDARFQDHQDHALLLSTTDVPASESATVTSATFPAPIKSSPCEL  173
ALK-202     ..............................................................................................................    0
ALK-203     ..............................................................................................................    0
```

```
logo
            RMSWLIRGVLRGNVSLVLVENKTGKEQGRMVWHVAAYEGLSLWQWMVLPLLDVSDRFWLQMVAWWGQGSRAIVAFDNISISLDCYLTISGEDKILQNTAPKSRNLFERNP
LORLATINIB  ..............................................................................................................    0
PF07714     ..............................................................................................................    0
PF00629     RMSWLIRGVLRGNVSLVLVENKTGKEQGRMVWHVAAYEGLSLWQWMVLPLLDVSDRFWLQMVAWWGQGSRAIVAFDNISISLDC..........................  315
PF07714.1   ..............................................................................................................    0
PF12810     ..............................................................................................................    0
PF00629.1   RMSWLIRGVLRGNVSLVLVENKTGKEQGRMVWHVAAYEGLSLWQWMVLPLLDVSDRFWLQMVAWWGQGSRAIVAFDNISISLDC..........................  155
ALK-201     RMSWLIRGVLRGNVSLVLVENKTGKEQGRMVWHVAAYEGLSLWQWMVLPLLDVSDRFWLQMVAWWGQGSRAIVAFDNISISLDCYLTISGEDKILQNTAPKSRNLFERNP  660
ALK-205     RMSWLIRGVLRGNVSLVLVENKTGKEQGRMVWHVAAYEGLSLWQWMVLPLLDVSDRFWLQMVAWWGQGSRAIVAFDNISISLDCYLTISGEDKILQNTAPKSRNLFERNP  283
ALK-202     ..............................................................................................................    0
ALK-203     ..............................................................................................................    0
```

```
logo
            NKELKPGENSPRQTPIFDPTVHWLFTTCGASGPHGPTQAQCNNAYQNSNLSVEVGSEGPLKGIQIWKVPATDTYSISGYGAAGGKGGKNTMMRSHGVSVLGIFNLEKDDM
LORLATINIB  ..............................................................................................................    0
PF07714     ..............................................................................................................    0
PF00629     ..............................................................................................................  315
PF07714.1   ..............................................................................................................    0
PF12810     ......................................................................TDTYSISGYGAAGGKGGKNTMMRSHGVSVLGIFNLEKDDM   40
PF00629.1   ..............................................................................................................  155
ALK-201     NKELKPGENSPRQTPIFDPTVHWLFTTCGASGPHGPTQAQCNNAYQNSNLSVEVGSEGPLKGIQIWKVPATDTYSISGYGAAGGKGGKNTMMRSHGVSVLGIFNLEKDDM  770
ALK-205     NKELKPGENSPRQTPIFDPTVHWLFTTCGASGPHGPTQAQCNNAYQNSNLSVEVGSEGPLKGIQIWKVPATDTYSISGYGAAGGKGGKNTMMRSHGVSVLGIFNLEKDDM  393
ALK-202     ..............................................................................................................    0
ALK-203     ..............................................................................................................    0
```

```
logo
            LYILVGQQGEDACPSTNQLIQKVCIGENNVIEEEIRVNRSVHEWAGGGGGGGGATYVFKMKDGVPVPLIIAAGGGGRAYGAKTDTFHPERLENNSSVLGLNGNSGAAGGG
LORLATINIB  ..............................................................................................................    0
PF07714     ..............................................................................................................    0
PF00629     ..............................................................................................................  315
PF07714.1   ..............................................................................................................    0
PF12810     LYILVGQQGEDACPSTNQLIQKVCIGENNVIEEEIRVNRSVHEWAGGGGGGGGATYVFKMKDGVPVPLIIAAGGGGRAYGAKTDTFHPERLENNSSVLGLNGNSGAAGGG  150
PF00629.1   ..............................................................................................................  155
ALK-201     LYILVGQQGEDACPSTNQLIQKVCIGENNVIEEEIRVNRSVHEWAGGGGGGGGATYVFKMKDGVPVPLIIAAGGGGRAYGAKTDTFHPERLENNSSVLGLNGNSGAAGGG  880
ALK-205     LYILVGQQGEDACPSTNQLIQKVCIGENNVIEEEIRVNRSVHEWAGGGGGGGGATYVFKMKDGVPVPLIIAAGGGGRAYGAKTDTFHPERLENNSSVLGLNGNSGAAGGG  503
ALK-202     ..............................................................................................................    0
ALK-203     ..............................................................................................................    0
```

```
logo
LORLATINIB   ..............................................................................................................   0
PF07714      ..............................................................................................................   0
PF00629      ..............................................................................................................   315
PF07714.1    ..............................................................................................................   0
PF12810      GGWNDNTSLLWAGKSLQEGATGGHSCPQAMKKWGWETRGGFGGGGGGCSSGGGGGGYIGGNAASNNDPEMDGEDGVSFISP.............................   231
PF00629.1    ..............................................................................................................   155
ALK-201      GGWNDNTSLLWAGKSLQEGATGGHSCPQAMKKWGWETRGGFGGGGGGCSSGGGGGGYIGGNAASNNDPEMDGEDGVSFISPLGILYTPALKVMEGHGEVNIKHYLNCSHC   990
ALK-205      GGWNDNTSLLWAGKSLQEGATGGHSCPQAMKKWGWETRGGFGGGGGGCSSGGGGGGYIGGNAASNNDPEMDGEDGVSFISPLGILYTPALKVMEGHGEVNIKHYLNCSHC   613
ALK-202      ..............................................................................................................   0
ALK-203      ..............................................................................................................   0

logo
LORLATINIB   ..............................................................................................................   0
PF07714      ..............................................................................................................   0
PF00629      ..............................................................................................................   315
PF07714.1    ..............................................................................................................   0
PF12810      ..............................................................................................................   231
PF00629.1    ..............................................................................................................   155
ALK-201      EVDECHMDPESHKVICFCDHGTVLAEDGVSCIVSPTPEPHLPLSLILSVVTSALVAALVLAFSGIMIVYRRKHQELQAMQMELQSPEYKLSKLRTSTIMTDYNPNYCFAG   1100
ALK-205      EVDECHMDPESHKVICFCDHGTVLAEDGVSCIVSPTPEPHLPLSLILSVVTSALVAALVLAFSGIMIVYRRKHQELQAMQMELQSPEYKLSKLRTSTIMTDYNPNYCFAG   723
ALK-202      ................................................MVT.TCSAIYTFQF.IRSSVYRRKHQELQAMQMELQSPEYKLSKLRTSTIMTDYNPNYCFAG   60
ALK-203      ..............................................................................................................   0

logo
LORLATINIB   .....................L.......V.................A..........................................................LELM   7
PF07714      ................TLIRGLGHGAFGEVYEGQVSGMPNDPSPLQVAVKTLPEVCSEQDELDFLMEALIISKFNHQNIV..RCIGVSLQ.........SLPRFILLELM   83
PF00629      ..............................................................................................................   315
PF07714.1    ................TLIRGLGHGAFGEVYEGQVSGMPNDPSPLQVAVKTLPEVCSEQDELDFLMEALIIS.......................................   56
PF12810      ..............................................................................................................   231
PF00629.1    ..............................................................................................................   155
ALK-201      KTSSISDLKEVPRKNITLIRGLGHGAFGEVYEGQVSGMPNDPSPLQVAVKTLPEVCSEQDELDFLMEALIISKFNHQNIV..RCIGVSLQ.........SLPRFILLELM   1199
ALK-205      KTSSISDLKEVPRKNITLIRGLGHGAFGEVYEGQVSGMPNDPSPLQVAVKTLPEVCSEQDELDFLMEALIISKFNHQNIV..RCIGVSLQ.........SLPRFILLELM   822
ALK-202      KTSSISDLKEVPRKNITLIRGLGHGAFGEVYEGQVSGMPNDPSPLQVAVKTLPEVCSEQDELDFLMEALIISKFNHQNIV..RCIGVSLQ.........SLPRFILLELM   159
ALK-203      ..SSISDLKEVPRKNITLIRGLGHGAFGEVYEGQVSGMPNDPSPLQVAVKTLPEVCSEQDELDFLMEALIISLSWKQLFCFTFCLRPSWEKSTMNSSTQSTHQYL..RKT   106

logo
LORLATINIB   A.G..................................................RN.L............GD.......................................   14
PF07714      AGGDLKSFLRETRPRPSQPSSLAMLDLLHVARDIACGCQYLEENHFIHRDIAARNCLLTCPGPGRVAKIGDFGMARDIYRASYYRKGGCAMLPVKWMPPEAFMEGIFTSK   193
PF00629      ..............................................................................................................   315
PF07714.1    ..............................................................................................................   56
PF12810      ..............................................................................................................   231
PF00629.1    ..............................................................................................................   155
ALK-201      AGGDLKSFLRETRPRPSQPSSLAMLDLLHVARDIACGCQYLEENHFIHRDIAARNCLLTCPGPGRVAKIGDFGMARDIYRASYYRKGGCAMLPVKWMPPEAFMEGIFTSK   1309
ALK-205      AGGDLKSFLRETRPRPSQPSSLAMLDLLHVARDIACGCQYLEENHFIHRDIAARNCLLTCPGPGRVAKIGDFGMARDIYRASYYRKGGCAMLPVKWMPPEAFMEGIFTSK   932
ALK-202      AGGDLKSFLRETRPRPSQPSSLAMLDLLHVARDIACGCQYLEENHFIHRDIAARNCLLTCPGPGRVAKIGDFGMARDIYRASYYRKGGCAMLPVKWMPPEAFMEGIFTSK   269
ALK-203      LSCPLRHFLTQVPEQHPHSSSHSPA..........NG...........TPVEPAVVLPTCS.PARF............................WV..............   152
```

```
logo
            TDTWSFGVLLWEIFSLGYMPYPSKSNQEVLEFVTSGGRMDPPKNCPGPVYRIMTQCWQHQPEDRPNFAIILERIEYCTQDPDVINTALPIEYGPLVEEEEKVPVRPKDPE
LORLATINIB  ..............................................................................................................    14
PF07714     TDTWSFGVLLWEIFSLGYMPYPSKSNQEVLEFVTSGGRMDPPKNCPGPVYRIMTQCWQHQPEDRPNFAIILER.....................................   266
PF00629     ..............................................................................................................   315
PF07714.1   ..............................................................................................................    56
PF12810     ..............................................................................................................   231
PF00629.1   ..............................................................................................................   155
ALK-201     TDTWSFGVLLWEIFSLGYMPYPSKSNQEVLEFVTSGGRMDPPKNCPGPVYRIMTQCWQHQPEDRPNFAIILERIEYCTQDPDVINTALPIEYGPLVEEEEKVPVRPKDPE  1419
ALK-205     TDTWSFGVLLWEIFSLGYMPYPSKSNQEVLEFVTSGGRMDPPKNCPGPVYRIMTQCWQHQPEDRPNFAIILERIEYCTQDPDVINTALPIEYGPLVEEEEKVPVRPKDPE  1042
ALK-202     TDTWSFGVLLWEIFSLGYMPYPSKSNQEVLEFVTSGGRMDPPKNCPGPVYRIMTQCWQHQPEDRPNFAIILERIEYCTQDPDVINTALPIEYGPLVEEEEKVPVRPKDPE   379
ALK-203     ..............................................................................................................   152

logo
            GVPPLLVSQQAKREEERSPAAPPPLPTTSSGKAAKKPTAAEISVRVPRGPAVEGGHVNMAFSQSNPPSELHKVHGSRNKPTSLWNPTYGSWFTEKPTKKNNPIAKKEPHD
LORLATINIB  ..............................................................................................................    14
PF07714     ..............................................................................................................   266
PF00629     ..............................................................................................................   315
PF07714.1   ..............................................................................................................    56
PF12810     ..............................................................................................................   231
PF00629.1   ..............................................................................................................   155
ALK-201     GVPPLLVSQQAKREEERSPAAPPPLPTTSSGKAAKKPTAAEISVRVPRGPAVEGGHVNMAFSQSNPPSELHKVHGSRNKPTSLWNPTYGSWFTEKPTKKNNPIAKKEPHD  1529
ALK-205     GVPPLLVSQQAKREEERSPAAPPPLPTTSSGKAAKKPTAAEISVRVPRGPAVEGGHVNMAFSQSNPPSELHKVHGSRNKPTSLWNPTYGSWFTEKPTKKNNPIAKKEPHD  1152
ALK-202     GVPPLLVSQQAKREEERSPAAPPPLPTTSSGKAAKKPTAAEISVRVPRGPAVEGGHVNMAFSQSNPPSELHKVHGSRNKPTSLWNPTYGSWFTEKPTKKNNPIAKKEPHD   489
ALK-203     ..............................................................................................................   152

logo
            RGNLGLEGSCTVPPNVATGRLPGASLLLEPSSLTANMKEVPLFRLRHFPCGNVNYGYQQQGLPLEAATAPGAGHYEDTILKSKNSMNQPGP
LORLATINIB  ...........................................................................................    14
PF07714     ...........................................................................................   266
PF00629     ...........................................................................................   315
PF07714.1   ...........................................................................................    56
PF12810     ...........................................................................................   231
PF00629.1   ...........................................................................................   155
ALK-201     RGNLGLEGSCTVPPNVATGRLPGASLLLEPSSLTANMKEVPLFRLRHFPCGNVNYGYQQQGLPLEAATAPGAGHYEDTILKSKNSMNQPGP  1620
ALK-205     RGNLGLEGSCTVPPNVATGRLPGASLLLEPSSLTANMKEVPLFRLRHFPCGNVNYGYQQQGLPLEAATAPGAGHYEDTILKSKNSMNQPGP  1243
ALK-202     RGNLGLEGSCTVPPNVATGRLPGASLLLEPSSLTANMKEVPLFRLRHFPCGNVNYGYQQQGLPLEAATAPGAGHYEDTILKSKNSMNQPGP   580
ALK-203     ...........................................................................................   152
```

X non conserved
X similar
X $\geq$0% conserved
X $\geq$50% conserved

```
logo
                     MEVQLGLGRVYPRPPSKTYRGAFQNLFQSVREVIQNPGPRHPEAASAAPPGASLLLLQQQQQQQQQQQQQQQQQQQQQQQETSPRQQQQQQGEDGSPQAHRRGPT
CYPROTERONE ACETATE  .........................................................................................................   0
PF00104.1            .........................................................................................................   0
PF00105.1            .........................................................................................................   0
PF00105              .........................................................................................................   0
PF02166              .....GLGRVYPRPPSKTYRGAFQNLFQSVREVIQNPGPRHPEAASAAPPGASLLLLQQQQQQQQQQQQQQQQQQQQQQQETSPRQQQQQQGEDGSPQAHRRGPT 100
PF00104.2            .........................................................................................................   0
PF02166.1            .........................................................................................................   0
PF00104              .........................................................................................................   0
AR-209               MEVQLGLGRVYPRPPSKTYRGAFQNLFQSVREVIQNPGPRHPEAASAAPPGASLLLLQQQQQQQQQQQQQQQQQQQQQQQETSPRQQQQQQGEDGSPQAHRRGPT 105
AR-203               MEVQLGLGRVYPRPPSKTYRGAFQNLFQSVREVIQNPGPRHPEAASAAPPGASLLLLQQQQQQQQQQQQQQQQQQQQQQQETSPRQQQQQQGEDGSPQAHRRGPT 105
AR-202               .........................................................................................................   0
AR-204               MEVQLGLGRVYPRPPSKTYRGAFQNLFQSVREVIQNPGPRHPEAASAAPPGASLLLLQQQQQQQQQQQQQQQQQQQQQQQETSPRQQQQQQGEDGSPQAHRRGPT 105
AR-206               MEVQLGLGRVYPRPPSKTYRGAFQNLFQSVREVIQNPGPRHPEAASAAPPGASLLLLQQQQQQQQQQQQQQQQQQQQQQQETSPRQQQQQQGEDGSPQAHRRGPT 105
AR-201               MEVQLGLGRVYPRPPSKTYRGAFQNLFQSVREVIQNPGPRHPEAASAAPPGASLLLLQQQQQQQQQQQQQQQQQQQQQQQETSPRQQQQQQGEDGSPQAHRRGPT 105
AR-207               MEVQLGLGRVYPRPPSKTYRGAFQNLFQSVREVIQNPGPRHPEAASAAPPGASLLLLQQQQQQQQQQQQQQQQQQQQQQQETSPRQQQQQQGEDGSPQAHRRGPT 105
AR-208               .........................................................................................................   0

logo
                     GYLVLDEEQQPSQPQSALECHPERGCVPEPGAAVAASKGLPQQLPAPPDEDDSAAPSTLSLLGPTFPGLSSCSADLKDILSEASTMQLLQQQQQEAVSEGSSSGR
CYPROTERONE ACETATE  .........................................................................................................   0
PF00104.1            .........................................................................................................   0
PF00105.1            .........................................................................................................   0
PF00105              .........................................................................................................   0
PF02166              GYLVLDEEQQPSQPQSALECHPERGCVPEPGAAVAASKGLPQQLPAPPDEDDSAAPSTLSLLGPTFPGLSSCSADLKDILSEASTMQLLQQQQQEAVSEGSSSGR 205
PF00104.2            .........................................................................................................   0
PF02166.1            .........................................................................................QQQQQEAVSEGSSSGR  16
PF00104              .........................................................................................................   0
AR-209               GYLVLDEEQQPSQPQSALECHPERGCVPEPGAAVAASKGLPQQLPAPPDEDDSAAPSTLSLLGPTFPGLSSCSADLKDILSEASTMQLLQQQQQEAVSEGSSSGR 210
AR-203               GYLVLDEEQQPSQPQSALECHPERGCVPEPGAAVAASKGLPQQLPAPPDEDDSAAPSTLSLLGPTFPGLSSCSADLKDILSEASTMQLLQQQQQEAVSEGSSSGR 210
AR-202               .........................................................................................................   0
AR-204               GYLVLDEEQQPSQPQSALECHPERGCVPEPGAAVAASKGLPQQLPAPPDEDDSAAPSTLSLLGPTFPGLSSCSADLKDILSEASTMQLLQQQQQEAVSEGSSSGR 210
AR-206               GYLVLDEEQQPSQPQSALECHPERGCVPEPGAAVAASKGLPQQLPAPPDEDDSAAPSTLSLLGPTFPGLSSCSADLKDILSEASTMQLLQQQQQEAVSEGSSSGR 210
AR-201               GYLVLDEEQQPSQPQSALECHPERGCVPEPGAAVAASKGLPQQLPAPPDEDDSAAPSTLSLLGPTFPGLSSCSADLKDILSEASTMQLLQQQQQEAVSEGSSSGR 210
AR-207               GYLVLDEEQQPSQPQSALECHPERGCVPEPGAAVAASKGLPQQLPAPPDEDDSAAPSTLSLLGPTFPGLSSCSADLKDILSEASTMQLLQQQQQEAVSEGSSSGR 210
AR-208               .....................................................................................MQLLQQQQQEAVSEGSSSGR  20
```

```
logo                  AREASGAPTSSKDNYLGGTSTISDNAKELCKAVSVSMGLGVEALEHLSPGEQLRGDCMYAPLLGVPPAVRPTPCAPLAECKGSLLDDSAGKSTEDTAEYSPFKGG
CYPROTERONE ACETATE   .........................................................................................................    0
PF00104.1             .........................................................................................................    0
PF00105.1             .........................................................................................................    0
PF00105               .........................................................................................................    0
PF02166               AREASGAPTSSKDNYLGGTSTISDNAKELCKAVSVSMGLGVEALEHLSPGEQLRGDCMYAPLLGVPPAVRPTPCAPLAECKGSLLDDSAGKSTEDTAEYSPFKGG  310
PF00104.2             .........................................................................................................    0
PF02166.1             AREASGAPTSSKDNYLGGTSTISDNAKELCKAVSVSMGLGVEALEHLSPGEQLRGDCMYAPLLGVPPAVRPTPCAPLAECKGSLLDDSAGKSTEDTAEYSPFKGG  121
PF00104               .........................................................................................................    0
AR-209                AREASGAPTSSKDNYLGGTSTISDNAKELCKAVSVSMGLGVEALEHLSPGEQLRGDCMYAPLLGVPPAVRPTPCAPLAECKGSLLDDSAGKSTEDTAEYSPFKGG  315
AR-203                AREASGAPTSSKDNYLGGTSTISDNAKELCKAVSVSMGLGVEALEHLSPGEQLRGDCMYAPLLGVPPAVRPTPCAPLAECKGSLLDDSAGKSTEDTAEYSPFKGG  315
AR-202                .........................................................................................................    0
AR-204                AREASGAPTSSKDNYLGGTSTISDNAKELCKAVSVSMGLGVEALEHLSPGEQLRGDCMYAPLLGVPPAVRPTPCAPLAECKGSLLDDSAGKSTEDTAEYSPFKGG  315
AR-206                AREASGAPTSSKDNYLGGTSTISDNAKELCKAVSVSMGLGVEALEHLSPGEQLRGDCMYAPLLGVPPAVRPTPCAPLAECKGSLLDDSAGKSTEDTAEYSPFKGG  315
AR-201                AREASGAPTSSKDNYLGGTSTISDNAKELCKAVSVSMGLGVEALEHLSPGEQLRGDCMYAPLLGVPPAVRPTPCAPLAECKGSLLDDSAGKSTEDTAEYSPFKGG  315
AR-207                AREASGAPTSSKDNYLGGTSTISDNAKELCKAVSVSMGLGVEALEHLSPGEQLRGDCMYAPLLGVPPAVRPTPCAPLAECKGSLLDDSAGKSTEDTAEYSPFKGG  315
AR-208                AREASGAPTSSKDNYLGGTSTISDNAKELCKAVSVSMGLGVEALEHLSPGEQLRGDCMYAPLLGVPPAVRPTPCAPLAECKGSLLDDSAGKSTEDTAEYSPFKGG  125
```

```
logo                  YTKGLEGESLGCSGSAAAGSSGTLELPSTLSLYKSGALDEAAAYQSRDYYNFPLALAGPPPPPPPPHPHARIKLENPLDYGSAWAAAAAQCRYGDLASLHGAGAA
CYPROTERONE ACETATE   .........................................................................................................    0
PF00104.1             .........................................................................................................    0
PF00105.1             .........................................................................................................    0
PF00105               .........................................................................................................    0
PF02166               YTKGLEGESLGCSGSAAAGSSGTLELPSTLSLYKSGALDEAAAYQSRDYYNFPLALAGPPPPPPPPHPHARIKLENPLDYGSAWAAAAAQCRYGDLASLHGAGAA  415
PF00104.2             .........................................................................................................    0
PF02166.1             YTKGLEGESLGCSGSAAAGSSGTLELPSTLSLYKSGALDEAAAYQSRDYYNFPLALAGPPPPPPPPHPHARIKLENPLDYGSAWAAAAAQCRYGDLASLHGAGAA  226
PF00104               .........................................................................................................    0
AR-209                YTKGLEGESLGCSGSAAAGSSGTLELPSTLSLYKSGALDEAAAYQSRDYYNFPLALAGPPPPPPPPHPHARIKLENPLDYGSAWAAAAAQCRYGDLASLHGAGAA  420
AR-203                YTKGLEGESLGCSGSAAAGSSGTLELPSTLSLYKSGALDEAAAYQSRDYYNFPLALAGPPPPPPPPHPHARIKLENPLDYGSAWAAAAAQCRYGDLASLHGAGAA  420
AR-202                .........................................................................................................    0
AR-204                YTKGLEGESLGCSGSAAAGSSGTLELPSTLSLYKSGALDEAAAYQSRDYYNFPLALAGPPPPPPPPHPHARIKLENPLDYGSAWAAAAAQCRYGDLASLHGAGAA  420
AR-206                YTKGLEGESLGCSGSAAAGSSGTLELPSTLSLYKSGALDEAAAYQSRDYYNFPLALAGPPPPPPPPHPHARIKLENPLDYGSAWAAAAAQCRYGDLASLHGAGAA  420
AR-201                YTKGLEGESLGCSGSAAAGSSGTLELPSTLSLYKSGALDEAAAYQSRDYYNFPLALAGPPPPPPPPHPHARIKLENPLDYGSAWAAAAAQCRYGDLASLHGAGAA  420
AR-207                YTKGLEGESLGCSGSAAAGSSGTLELPSTLSLYKSGALDEAAAYQSRDYYNFPLALAGPPPPPPPPHPHARIKLENPLDYGSAWAAAAAQCRYGDLASLHGAGAA  420
AR-208                YTKGLEGESLGCSGSAAAGSSGTLELPSTLSLYKSGALDEAAAYQSRDYYNFPLALAGPPPPPPPPHPHARIKLENPLDYGSAWAAAAAQCRYGDLASLHGAGAA  230
```

logo

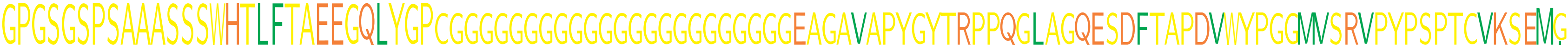

```
CYPROTERONE ACETATE  .........................................................................................................    0
PF00104.1            .........................................................................................................    0
PF00105.1            .........................................................................................................    0
PF00105              .........................................................................................................    0
PF02166              GPGSGSPSAAASSSWHTLFTAEEGQLYGP............................................................................  444
PF00104.2            .........................................................................................................    0
PF02166.1            GPGSGSPSAAASSSWHTLFTAEEGQLYGP............................................................................  255
PF00104              .........................................................................................................    0
AR-209               GPGSGSPSAAASSSWHTLFTAEEGQLYGPCGGGGGGGGGGGGGGGGGGGGGGGEAGAVAPYGYTRPPQGLAGQESDFTAPDVWYPGGMVSRVPYPSPTCVKSEMG  525
AR-203               GPGSGSPSAAASSSWHTLFTAEEGQLYGPCGGGGGGGGGGGGGGGGGGGGGGGEAGAVAPYGYTRPPQGLAGQESDFTAPDVWYPGGMVSRVPYPSPTCVKSEMG  525
AR-202               .......................................................................................................MI    2
AR-204               GPGSGSPSAAASSSWHTLFTAEEGQLYGPCGGGGGGGGGGGGGGGGGGGGGGGEAGAVAPYGYTRPPQGLAGQESDFTAPDVWYPGGMVSRVPYPSPTCVKSEMG  525
AR-206               GPGSGSPSAAASSSWHTLFTAEEGQLYGPCGGGGGGGGGGGGGGGGGGGGGGGEAGAVAPYGYTRPPQGLAGQESDFTAPDVWYPGGMVSRVPYPSPTCVKSEMG  525
AR-201               GPGSGSPSAAASSSWHTLFTAEEGQLYGPCGGGGGGGGGGGGGGGGGGGGGGGEAGAVAPYGYTRPPQGLAGQESDFTAPDVWYPGGMVSRVPYPSPTCVKSEMG  525
AR-207               GPGSGSPSAAASSSWHTLFTAEEGQLYGPCGGGGGGGGGGGGGGGGGGGGGGGEAGAVAPYGYTRPPQGLAGQESDFTAPDVWYPGGMVSRVPYPSPTCVKSEMG  525
AR-208               GPGSGSPSAAASSSWHTLFTAEEGQLYGPCGGGGGGGGGGGGGGGGGGGGGGGEAGAVAPYGYTRPPQGLAGQESDFTAPDVWYPGGMVSRVPYPSPTCVKSEMG  335
```

logo

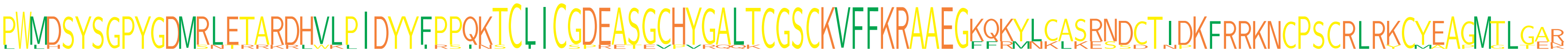

```
CYPROTERONE ACETATE  .........................................................................................................    0
PF00104.1            .........................................................................................................    0
PF00105.1            .................................TCLICGDEASGCHYGALTCGSCKVFFKRAAEG........................................   32
PF00105              .................................TCLICGDEASGCHYGALTCGSCKVFFKRAAEGKQKYLCASRNDCTIDKFRRKNCPSCRLRKCYEAGMT....   68
PF02166              .........................................................................................................  444
PF00104.2            .........................................................................................................    0
PF02166.1            .........................................................................................................  255
PF00104              .........................................................................................................    0
AR-209               PWMDSYSGPYGDMRNTRRKRLWKLI...IRSINSCI.CSPRETEVPVRQQK......................................................  572
AR-203               PWMDSYSGPYGDMRLETARDHVLPIDYYFPPQKTCLICGDEASGCHYGALTCGSCKVFFKRAAEGKQKYLCASRNDCTIDKFRRKNCPSCRLRKCYEAGMTLGAR  630
AR-202               LWLH.........SLETARDHVLPIDYYFPPQKTCLICGDEASGCHYGALTCGSCKVFFKRAAEGKQKYLCASRNDCTIDKFRRKNCPSCRLRKCYEAGMTLGAR   98
AR-204               PWMDSYSGPYGDMRLETARDHVLPIDYYFPPQKTCLICGDEASGCHYGALTCGSCKVFFKRAAEGKQKYLCASRNDCTIDKFRRKNCPSCRLRKCYEAGMTLGEK  630
AR-206               PWMDSYSGPYGDMRLETARDHVLPIDYYFPPQKTCLICGDEASGCHYGALTCGSCKVFFKRAAEGFFRMNKLKES..............................  600
AR-201               PWMDSYSGPYGDMRLETARDHVLPIDYYFPPQKTCLICGDEASGCHYGALTCGSCKVFFKRAAEGKQKYLCASRNDCTIDKFRRKNCPSCRLRKCYEAGMTLGAR  630
AR-207               PWMDSYSGPYGDMRLETARDHVLPIDYYFPPQKTCLICGDEASGCHYGALTCGSCKVFFKRAAEGFFRMNKLKESSDTNPK......P.....YCMAAPMGLTEN  619
AR-208               PWMDSYSGPYGDMRLETARDHVLPIDYYFPPQKTCLICGDEASGCHYGALTCGSCKVFFKRAAEGKQKYLCASRNDCTIDKFRRKNCPSCRLRKCYEAGMTLGAR  440
```

logo

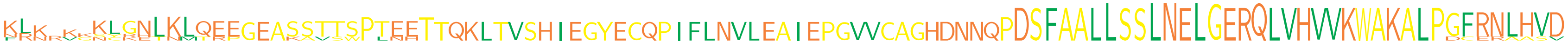

```
CYPROTERONE ACETATE  ..........................................................................L..LN.LG..Q....................    6
PF00104.1            ...................................................................PDSFAALLSSLNELGERQLVHVVKWAKALPGFRNLHVD   38
PF00105.1            .........................................................................................................   32
PF00105              .........................................................................................................   68
PF02166              .........................................................................................................  444
PF00104.2            ....................................................................DSFAALLSSLNELGERQLVHVVKWAKALPGFRNLHVD   37
PF02166.1            .........................................................................................................  255
PF00104              ....................................................................DSFAALLSSLNELGERQLVHVVKWAKALPGFRNLHVD   37
AR-209               .........................................................................................................  572
AR-203               KL..K.KLGNLKLQEEGEASSTTSPTEETTQKLTVSHIEGYECQPIFLNVLEAIEPGVVCAGHDNNQPDSFAALLSSLNELGERQLVHVVKWAKALPDCERAASV  732
AR-202               KLK...KLGNLKLQEEGEASSTTSPTEETTQKLTVSHIEGYECQPIFLNVLEAIEPGVVCAGHDNNQPDSFAALLSSLNELGERQLVHVVKWAKALPGFRNLHVD  200
AR-204               FR..VGNCKHLKMTRP.........................................................................................  644
AR-206               .........................................................................................................  600
AR-201               KLK...KLGNLKLQEEGEASSTTSPTEETTQKLTVSHIEGYECQPIFLNVLEAIEPGVVCAGHDNNQPDSFAALLSSLNELGERQLVHVVKWAKALPGFRNLHVD  732
AR-207               NRNRKKSYRETNL......KAVSWPLNHT............................................................................  642
AR-208               KLK...KLGNLKLQEEGEASSTTSPTEETTQKLTVSHIEGYECQPIFLNVLEAIEPGVVCAGHDNNQPDSFAALLSSLNELGERQLVHVVKWAKALPGFRNLHVD  542
```

logo

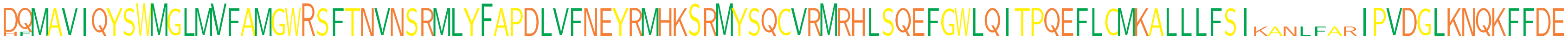

```
CYPROTERONE ACETATE  .........WM...V.....R...........F.............S.M......M.................................................   14
PF00104.1            DQMAVIQYSWMGLMVFAMGWRSFTNVNSRMLYFAPDLVFNEYRMHKSRMYSQCVRMRHLSQEFGWLQITPQEFLCMKALLLFSI.......IPVDGLKNQKFFDE  136
PF00105.1            .........................................................................................................   32
PF00105              .........................................................................................................   68
PF02166              .........................................................................................................  444
PF00104.2            DQMAVIQYSWMGLMVFAMGWRSFTNVNSRMLYFAPDLVFNEYRMHKSRMYSQCVRMRHLSQEFGWLQITPQEFLCMKALLLFSIKANLFARIPVDGLKNQKFFDE  142
PF02166.1            .........................................................................................................  255
PF00104              DQMAVIQYSWMGLMVFAMGWRSFTNVNSRMLYFAPDLVFNEYRMHKSRMYSQCVRMRHLSQEFGWLQITPQEFLCMKALLLFSI.......IPVDGLKNQKFFDE  135
AR-209               .........................................................................................................  572
AR-203               HF.......................................................................................................  734
AR-202               DQMAVIQYSWMGLMVFAMGWRSFTNVNSRMLYFAPDLVFNEYRMHKSRMYSQCVRMRHLSQEFGWLQITPQEFLCMKALLLFSI.......IPVDGLKNQKFFDE  298
AR-204               .........................................................................................................  644
AR-206               .........................................................................................................  600
AR-201               DQMAVIQYSWMGLMVFAMGWRSFTNVNSRMLYFAPDLVFNEYRMHKSRMYSQCVRMRHLSQEFGWLQITPQEFLCMKALLLFSI.......IPVDGLKNQKFFDE  830
AR-207               .........................................................................................................  642
AR-208               DQMAVIQYSWMGLMVFAMGWRSFTNVNSRMLYFAPDLVFNEYRMHKSRMYSQCVRMRHLSQEFGWLQITPQEFLCMKALLLFSIKANLFARIPVDGLKNQKFFDE  647
```

logo

```
                      LRMNYIKELDRIIACKRKNPTSCSRRFYQLTKLLDSVQPIARELHQFTFDLLIKSHMVSVDFPEMMAEIISVQVPKILSGKVKPIYFHTQ
CYPROTERONE ACETATE   ..............................................FA..L..........F...M........................   19
PF00104.1             LRMNYIKELDRIIACKRKNPTSCSRRFYQLTKLLDSVQPIARELHQFTF.........................................  185
PF00105.1             ..........................................................................................   32
PF00105               ..........................................................................................   68
PF02166               ..........................................................................................  444
PF00104.2             LRMNYIKELDRIIACKRKNPTSCSRRFYQLTKLLDSVQPIARELHQFTF.........................................  191
PF02166.1             ..........................................................................................  255
PF00104               LRMNYIKELDRIIACKRKNPTSCSRRFYQLTKLLDSVQPIARELHQFTF.........................................  184
AR-209                ..........................................................................................  572
AR-203                ..........................................................................................  734
AR-202                LRMNYIKELDRIIACKRKNPTSCSRRFYQLTKLLDSVQPIARELHQFTFDLLIKSHMVSVDFPEMMAEIISVQVPKILSGKVKPIYFHTQ  388
AR-204                ..........................................................................................  644
AR-206                ..........................................................................................  600
AR-201                LRMNYIKELDRIIACKRKNPTSCSRRFYQLTKLLDSVQPIARELHQFTFDLLIKSHMVSVDFPEMMAEIISVQVPKILSGKVKPIYFHTQ  920
AR-207                ..........................................................................................  642
AR-208                LRMNYIKELDRIIACKRKNPTSCSRRFYQLTKLLDSVQPIARELHQFTFDLLIKSHMVSVDFPEMMAEIISVQVPKILSGKVKPIYFHTQ  737
```

X non conserved

X similar

X $\geq$ 0% conserved

X $\geq$ 50% conserved

```
logo
            MEVQLGLGRVYPRPPSKTYRGAFQNLFQSVREVIQNPGPRHPEAASAAPPGASLLLLQQQQQQQQQQQQQQQQQQQQQQQETSPRQQQQQQGEDGSPQAHRRGPTGYLVL
ENOBOSARM   ..............................................................................................................   0
PF00104.1   ..............................................................................................................   0
PF00105.1   ..............................................................................................................   0
PF00105     ..............................................................................................................   0
PF02166     .....GLGRVYPRPPSKTYRGAFQNLFQSVREVIQNPGPRHPEAASAAPPGASLLLLQQQQQQQQQQQQQQQQQQQQQQQETSPRQQQQQQGEDGSPQAHRRGPTGYLVL   105
PF00104.2   ..............................................................................................................   0
PF02166.1   ..............................................................................................................   0
PF00104     ..............................................................................................................   0
AR-209      MEVQLGLGRVYPRPPSKTYRGAFQNLFQSVREVIQNPGPRHPEAASAAPPGASLLLLQQQQQQQQQQQQQQQQQQQQQQQETSPRQQQQQQGEDGSPQAHRRGPTGYLVL   110
AR-203      MEVQLGLGRVYPRPPSKTYRGAFQNLFQSVREVIQNPGPRHPEAASAAPPGASLLLLQQQQQQQQQQQQQQQQQQQQQQQETSPRQQQQQQGEDGSPQAHRRGPTGYLVL   110
AR-202      ..............................................................................................................   0
AR-204      MEVQLGLGRVYPRPPSKTYRGAFQNLFQSVREVIQNPGPRHPEAASAAPPGASLLLLQQQQQQQQQQQQQQQQQQQQQQQETSPRQQQQQQGEDGSPQAHRRGPTGYLVL   110
AR-206      MEVQLGLGRVYPRPPSKTYRGAFQNLFQSVREVIQNPGPRHPEAASAAPPGASLLLLQQQQQQQQQQQQQQQQQQQQQQQETSPRQQQQQQGEDGSPQAHRRGPTGYLVL   110
AR-201      MEVQLGLGRVYPRPPSKTYRGAFQNLFQSVREVIQNPGPRHPEAASAAPPGASLLLLQQQQQQQQQQQQQQQQQQQQQQQETSPRQQQQQQGEDGSPQAHRRGPTGYLVL   110
AR-207      MEVQLGLGRVYPRPPSKTYRGAFQNLFQSVREVIQNPGPRHPEAASAAPPGASLLLLQQQQQQQQQQQQQQQQQQQQQQQETSPRQQQQQQGEDGSPQAHRRGPTGYLVL   110
AR-208      ..............................................................................................................   0

logo
            DEEQQPSQPQSALECHPERGCVPEPGAAVAASKGLPQQLPAPPDEDDSAAPSTLSLLGPTFPGLSSCSADLKDILSEASTMQLLQQQQQEAVSEGSSSGRAREASGAPTS
ENOBOSARM   ..............................................................................................................   0
PF00104.1   ..............................................................................................................   0
PF00105.1   ..............................................................................................................   0
PF00105     ..............................................................................................................   0
PF02166     DEEQQPSQPQSALECHPERGCVPEPGAAVAASKGLPQQLPAPPDEDDSAAPSTLSLLGPTFPGLSSCSADLKDILSEASTMQLLQQQQQEAVSEGSSSGRAREASGAPTS   215
PF00104.2   ..............................................................................................................   0
PF02166.1   ....................................................................................QQQQQEAVSEGSSSGRAREASGAPTS   26
PF00104     ..............................................................................................................   0
AR-209      DEEQQPSQPQSALECHPERGCVPEPGAAVAASKGLPQQLPAPPDEDDSAAPSTLSLLGPTFPGLSSCSADLKDILSEASTMQLLQQQQQEAVSEGSSSGRAREASGAPTS   220
AR-203      DEEQQPSQPQSALECHPERGCVPEPGAAVAASKGLPQQLPAPPDEDDSAAPSTLSLLGPTFPGLSSCSADLKDILSEASTMQLLQQQQQEAVSEGSSSGRAREASGAPTS   220
AR-202      ..............................................................................................................   0
AR-204      DEEQQPSQPQSALECHPERGCVPEPGAAVAASKGLPQQLPAPPDEDDSAAPSTLSLLGPTFPGLSSCSADLKDILSEASTMQLLQQQQQEAVSEGSSSGRAREASGAPTS   220
AR-206      DEEQQPSQPQSALECHPERGCVPEPGAAVAASKGLPQQLPAPPDEDDSAAPSTLSLLGPTFPGLSSCSADLKDILSEASTMQLLQQQQQEAVSEGSSSGRAREASGAPTS   220
AR-201      DEEQQPSQPQSALECHPERGCVPEPGAAVAASKGLPQQLPAPPDEDDSAAPSTLSLLGPTFPGLSSCSADLKDILSEASTMQLLQQQQQEAVSEGSSSGRAREASGAPTS   220
AR-207      DEEQQPSQPQSALECHPERGCVPEPGAAVAASKGLPQQLPAPPDEDDSAAPSTLSLLGPTFPGLSSCSADLKDILSEASTMQLLQQQQQEAVSEGSSSGRAREASGAPTS   220
AR-208      ................................................................................MQLLQQQQQEAVSEGSSSGRAREASGAPTS   30
```

```
logo        SKDNYLGGTSTISDNAKELCKAVSVSMGLGVEALEHLSPGEQLRGDCMYAPLLGVPPAVRPTPCAPLAECKGSLLDDSAGKSTEDTAEYSPFKGGYTKGLEGESLGCSGS
ENOBOSARM   ..............................................................................................................    0
PF00104.1   ..............................................................................................................    0
PF00105.1   ..............................................................................................................    0
PF00105     ..............................................................................................................    0
PF02166     SKDNYLGGTSTISDNAKELCKAVSVSMGLGVEALEHLSPGEQLRGDCMYAPLLGVPPAVRPTPCAPLAECKGSLLDDSAGKSTEDTAEYSPFKGGYTKGLEGESLGCSGS  325
PF00104.2   ..............................................................................................................    0
PF02166.1   SKDNYLGGTSTISDNAKELCKAVSVSMGLGVEALEHLSPGEQLRGDCMYAPLLGVPPAVRPTPCAPLAECKGSLLDDSAGKSTEDTAEYSPFKGGYTKGLEGESLGCSGS  136
PF00104     ..............................................................................................................    0
AR-209      SKDNYLGGTSTISDNAKELCKAVSVSMGLGVEALEHLSPGEQLRGDCMYAPLLGVPPAVRPTPCAPLAECKGSLLDDSAGKSTEDTAEYSPFKGGYTKGLEGESLGCSGS  330
AR-203      SKDNYLGGTSTISDNAKELCKAVSVSMGLGVEALEHLSPGEQLRGDCMYAPLLGVPPAVRPTPCAPLAECKGSLLDDSAGKSTEDTAEYSPFKGGYTKGLEGESLGCSGS  330
AR-202      ..............................................................................................................    0
AR-204      SKDNYLGGTSTISDNAKELCKAVSVSMGLGVEALEHLSPGEQLRGDCMYAPLLGVPPAVRPTPCAPLAECKGSLLDDSAGKSTEDTAEYSPFKGGYTKGLEGESLGCSGS  330
AR-206      SKDNYLGGTSTISDNAKELCKAVSVSMGLGVEALEHLSPGEQLRGDCMYAPLLGVPPAVRPTPCAPLAECKGSLLDDSAGKSTEDTAEYSPFKGGYTKGLEGESLGCSGS  330
AR-201      SKDNYLGGTSTISDNAKELCKAVSVSMGLGVEALEHLSPGEQLRGDCMYAPLLGVPPAVRPTPCAPLAECKGSLLDDSAGKSTEDTAEYSPFKGGYTKGLEGESLGCSGS  330
AR-207      SKDNYLGGTSTISDNAKELCKAVSVSMGLGVEALEHLSPGEQLRGDCMYAPLLGVPPAVRPTPCAPLAECKGSLLDDSAGKSTEDTAEYSPFKGGYTKGLEGESLGCSGS  330
AR-208      SKDNYLGGTSTISDNAKELCKAVSVSMGLGVEALEHLSPGEQLRGDCMYAPLLGVPPAVRPTPCAPLAECKGSLLDDSAGKSTEDTAEYSPFKGGYTKGLEGESLGCSGS  140

logo        AAAGSSGTLELPSTLSLYKSGALDEAAAYQSRDYYNFPLALAGPPPPPPPPHPHARIKLENPLDYGSAWAAAAAQCRYGDLASLHGAGAAGPGSGSPSAAASSSWHTLFT
ENOBOSARM   ..............................................................................................................    0
PF00104.1   ..............................................................................................................    0
PF00105.1   ..............................................................................................................    0
PF00105     ..............................................................................................................    0
PF02166     AAAGSSGTLELPSTLSLYKSGALDEAAAYQSRDYYNFPLALAGPPPPPPPPHPHARIKLENPLDYGSAWAAAAAQCRYGDLASLHGAGAAGPGSGSPSAAASSSWHTLFT  435
PF00104.2   ..............................................................................................................    0
PF02166.1   AAAGSSGTLELPSTLSLYKSGALDEAAAYQSRDYYNFPLALAGPPPPPPPPHPHARIKLENPLDYGSAWAAAAAQCRYGDLASLHGAGAAGPGSGSPSAAASSSWHTLFT  246
PF00104     ..............................................................................................................    0
AR-209      AAAGSSGTLELPSTLSLYKSGALDEAAAYQSRDYYNFPLALAGPPPPPPPPHPHARIKLENPLDYGSAWAAAAAQCRYGDLASLHGAGAAGPGSGSPSAAASSSWHTLFT  440
AR-203      AAAGSSGTLELPSTLSLYKSGALDEAAAYQSRDYYNFPLALAGPPPPPPPPHPHARIKLENPLDYGSAWAAAAAQCRYGDLASLHGAGAAGPGSGSPSAAASSSWHTLFT  440
AR-202      ..............................................................................................................    0
AR-204      AAAGSSGTLELPSTLSLYKSGALDEAAAYQSRDYYNFPLALAGPPPPPPPPHPHARIKLENPLDYGSAWAAAAAQCRYGDLASLHGAGAAGPGSGSPSAAASSSWHTLFT  440
AR-206      AAAGSSGTLELPSTLSLYKSGALDEAAAYQSRDYYNFPLALAGPPPPPPPPHPHARIKLENPLDYGSAWAAAAAQCRYGDLASLHGAGAAGPGSGSPSAAASSSWHTLFT  440
AR-201      AAAGSSGTLELPSTLSLYKSGALDEAAAYQSRDYYNFPLALAGPPPPPPPPHPHARIKLENPLDYGSAWAAAAAQCRYGDLASLHGAGAAGPGSGSPSAAASSSWHTLFT  440
AR-207      AAAGSSGTLELPSTLSLYKSGALDEAAAYQSRDYYNFPLALAGPPPPPPPPHPHARIKLENPLDYGSAWAAAAAQCRYGDLASLHGAGAAGPGSGSPSAAASSSWHTLFT  440
AR-208      AAAGSSGTLELPSTLSLYKSGALDEAAAYQSRDYYNFPLALAGPPPPPPPPHPHARIKLENPLDYGSAWAAAAAQCRYGDLASLHGAGAAGPGSGSPSAAASSSWHTLFT  250
```

```
logo
```

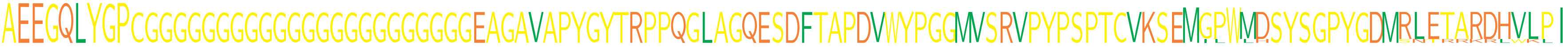

```
ENOBOSARM   ..............................................................................................................   0
PF00104.1   ..............................................................................................................   0
PF00105.1   ..............................................................................................................   0
PF00105     ..............................................................................................................   0
PF02166     AEEGQLYGP.....................................................................................................   444
PF00104.2   ..............................................................................................................   0
PF02166.1   AEEGQLYGP.....................................................................................................   255
PF00104     ..............................................................................................................   0
AR-209      AEEGQLYGPCGGGGGGGGGGGGGGGGGGGGGGGGEAGAVAPYGYTRPPQGLAGQESDFTAPDVWYPGGMVSRVPYPSPTCVKSEMGPWMDSYSGPYGDMRNTRRKRLWKLI   550
AR-203      AEEGQLYGPCGGGGGGGGGGGGGGGGGGGGGGGGEAGAVAPYGYTRPPQGLAGQESDFTAPDVWYPGGMVSRVPYPSPTCVKSEMGPWMDSYSGPYGDMRLETARDHVLPI   550
AR-202      ...................................................................................MILWLH.........SLETARDHVLPI   18
AR-204      AEEGQLYGPCGGGGGGGGGGGGGGGGGGGGGGGGEAGAVAPYGYTRPPQGLAGQESDFTAPDVWYPGGMVSRVPYPSPTCVKSEMGPWMDSYSGPYGDMRLETARDHVLPI   550
AR-206      AEEGQLYGPCGGGGGGGGGGGGGGGGGGGGGGGGEAGAVAPYGYTRPPQGLAGQESDFTAPDVWYPGGMVSRVPYPSPTCVKSEMGPWMDSYSGPYGDMRLETARDHVLPI   550
AR-201      AEEGQLYGPCGGGGGGGGGGGGGGGGGGGGGGGGEAGAVAPYGYTRPPQGLAGQESDFTAPDVWYPGGMVSRVPYPSPTCVKSEMGPWMDSYSGPYGDMRLETARDHVLPI   550
AR-207      AEEGQLYGPCGGGGGGGGGGGGGGGGGGGGGGGGEAGAVAPYGYTRPPQGLAGQESDFTAPDVWYPGGMVSRVPYPSPTCVKSEMGPWMDSYSGPYGDMRLETARDHVLPI   550
AR-208      AEEGQLYGPCGGGGGGGGGGGGGGGGGGGGGGGGEAGAVAPYGYTRPPQGLAGQESDFTAPDVWYPGGMVSRVPYPSPTCVKSEMGPWMDSYSGPYGDMRLETARDHVLPI   360
```

```
logo
```

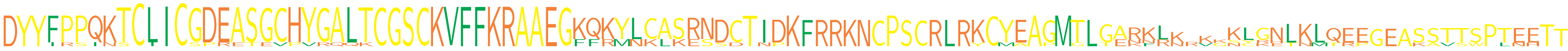

```
ENOBOSARM   ..............................................................................................................   0
PF00104.1   ..............................................................................................................   0
PF00105.1   ........TCLICGDEASGCHYGALTCGSCKVFFKRAAEG......................................................................   32
PF00105     ........TCLICGDEASGCHYGALTCGSCKVFFKRAAEGKQKYLCASRNDCTIDKFRRKNCPSCRLRKCYEAGMT..................................   68
PF02166     ..............................................................................................................   444
PF00104.2   ..............................................................................................................   0
PF02166.1   ..............................................................................................................   255
PF00104     ..............................................................................................................   0
AR-209      ...IRSINSCI.CSPRETEVPVRQQK....................................................................................   572
AR-203      DYYFPPQKTCLICGDEASGCHYGALTCGSCKVFFKRAAEGKQKYLCASRNDCTIDKFRRKNCPSCRLRKCYEAGMTLGARKL..K.KLGNLKLQEEGEASSTTSPTEETT   657
AR-202      DYYFPPQKTCLICGDEASGCHYGALTCGSCKVFFKRAAEGKQKYLCASRNDCTIDKFRRKNCPSCRLRKCYEAGMTLGARKLK...KLGNLKLQEEGEASSTTSPTEETT   125
AR-204      DYYFPPQKTCLICGDEASGCHYGALTCGSCKVFFKRAAEGKQKYLCASRNDCTIDKFRRKNCPSCRLRKCYEAGMTLGEKFR..VGNCKHLKMTRP..............   644
AR-206      DYYFPPQKTCLICGDEASGCHYGALTCGSCKVFFKRAAEGFFRMNKLKES............................................................   600
AR-201      DYYFPPQKTCLICGDEASGCHYGALTCGSCKVFFKRAAEGKQKYLCASRNDCTIDKFRRKNCPSCRLRKCYEAGMTLGARKLK...KLGNLKLQEEGEASSTTSPTEETT   657
AR-207      DYYFPPQKTCLICGDEASGCHYGALTCGSCKVFFKRAAEGFFRMNKLKESSDTNPK......P.....YCMAAPMGLTENNRNRKKSYRETNL......KAVSWPLNHT.   642
AR-208      DYYFPPQKTCLICGDEASGCHYGALTCGSCKVFFKRAAEGKQKYLCASRNDCTIDKFRRKNCPSCRLRKCYEAGMTLGARKLK...KLGNLKLQEEGEASSTTSPTEETT   467
```

logo

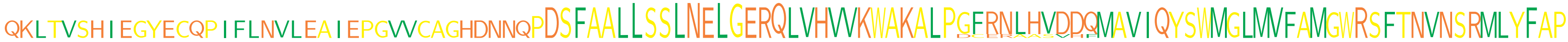


```
ENOBOSARM   ............................................L..LN.LG..Q..........................Q..WM..MV..M..R...........F..   14
PF00104.1   .....................................PDSFAALLSSLNELGERQLVHVVKWAKALPGFRNLHVDDQMAVIQYSWMGLMVFAMGWRSFTNVNSRMLYFAP   73
PF00105.1   ..............................................................................................................   32
PF00105     ..............................................................................................................   68
PF02166     ..............................................................................................................  444
PF00104.2   ......................................DSFAALLSSLNELGERQLVHVVKWAKALPGFRNLHVDDQMAVIQYSWMGLMVFAMGWRSFTNVNSRMLYFAP   72
PF02166.1   ..............................................................................................................  255
PF00104     ......................................DSFAALLSSLNELGERQLVHVVKWAKALPGFRNLHVDDQMAVIQYSWMGLMVFAMGWRSFTNVNSRMLYFAP   72
AR-209      ..............................................................................................................  572
AR-203      QKLTVSHIEGYECQPIFLNVLEAIEPGVVCAGHDNNQPDSFAALLSSLNELGERQLVHVVKWAKALPDCERAASVHF.................................  734
AR-202      QKLTVSHIEGYECQPIFLNVLEAIEPGVVCAGHDNNQPDSFAALLSSLNELGERQLVHVVKWAKALPGFRNLHVDDQMAVIQYSWMGLMVFAMGWRSFTNVNSRMLYFAP  235
AR-204      ..............................................................................................................  644
AR-206      ..............................................................................................................  600
AR-201      QKLTVSHIEGYECQPIFLNVLEAIEPGVVCAGHDNNQPDSFAALLSSLNELGERQLVHVVKWAKALPGFRNLHVDDQMAVIQYSWMGLMVFAMGWRSFTNVNSRMLYFAP  767
AR-207      ..............................................................................................................  642
AR-208      QKLTVSHIEGYECQPIFLNVLEAIEPGVVCAGHDNNQPDSFAALLSSLNELGERQLVHVVKWAKALPGFRNLHVDDQMAVIQYSWMGLMVFAMGWRSFTNVNSRMLYFAP  577
```

logo

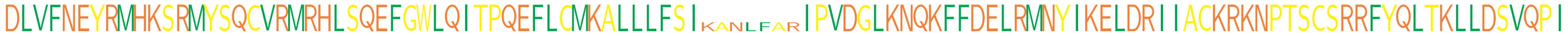


```
ENOBOSARM   ..............................................................................................................   14
PF00104.1   DLVFNEYRMHKSRMYSQCVRMRHLSQEFGWLQITPQEFLCMKALLLFSI.......IPVDGLKNQKFFDELRMNYIKELDRIIACKRKNPTSCSRRFYQLTKLLDSVQPI  176
PF00105.1   ..............................................................................................................   32
PF00105     ..............................................................................................................   68
PF02166     ..............................................................................................................  444
PF00104.2   DLVFNEYRMHKSRMYSQCVRMRHLSQEFGWLQITPQEFLCMKALLLFSIKANLFARIPVDGLKNQKFFDELRMNYIKELDRIIACKRKNPTSCSRRFYQLTKLLDSVQPI  182
PF02166.1   ..............................................................................................................  255
PF00104     DLVFNEYRMHKSRMYSQCVRMRHLSQEFGWLQITPQEFLCMKALLLFSI.......IPVDGLKNQKFFDELRMNYIKELDRIIACKRKNPTSCSRRFYQLTKLLDSVQPI  175
AR-209      ..............................................................................................................  572
AR-203      ..............................................................................................................  734
AR-202      DLVFNEYRMHKSRMYSQCVRMRHLSQEFGWLQITPQEFLCMKALLLFSI.......IPVDGLKNQKFFDELRMNYIKELDRIIACKRKNPTSCSRRFYQLTKLLDSVQPI  338
AR-204      ..............................................................................................................  644
AR-206      ..............................................................................................................  600
AR-201      DLVFNEYRMHKSRMYSQCVRMRHLSQEFGWLQITPQEFLCMKALLLFSI.......IPVDGLKNQKFFDELRMNYIKELDRIIACKRKNPTSCSRRFYQLTKLLDSVQPI  870
AR-207      ..............................................................................................................  642
AR-208      DLVFNEYRMHKSRMYSQCVRMRHLSQEFGWLQITPQEFLCMKALLLFSIKANLFARIPVDGLKNQKFFDELRMNYIKELDRIIACKRKNPTSCSRRFYQLTKLLDSVQPI  687
```

logo

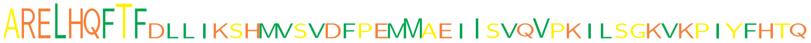


```
ENOBOSARM    ...L...T.................M...I...V................    19
PF00104.1    ARELHQFTF.........................................   185
PF00105.1    ..................................................    32
PF00105      ..................................................    68
PF02166      ..................................................   444
PF00104.2    ARELHQFTF.........................................   191
PF02166.1    ..................................................   255
PF00104      ARELHQFTF.........................................   184
AR-209       ..................................................   572
AR-203       ..................................................   734
AR-202       ARELHQFTFDLLIKSHMVSVDFPEMMAEIISVQVPKILSGKVKPIYFHTQ   388
AR-204       ..................................................   644
AR-206       ..................................................   600
AR-201       ARELHQFTFDLLIKSHMVSVDFPEMMAEIISVQVPKILSGKVKPIYFHTQ   920
AR-207       ..................................................   642
AR-208       ARELHQFTFDLLIKSHMVSVDFPEMMAEIISVQVPKILSGKVKPIYFHTQ   737
```

- X non conserved
- X similar
- X $\geq$ 0% conserved
- X $\geq$ 50% conserved

```
logo
             MEVQLGLGRVYPRPPSKTYRGAFQNLFQSVREVIQNPGPRHPEAASAAPPGASLLLLQQQQQQQQQQQQQQQQQQQQQQQETSPRQQQQQQGEDGSPQAHRRGPTGYLVL
FLUTAMIDE    ..............................................................................................................   0
PF00104.1    ..............................................................................................................   0
PF00105.1    ..............................................................................................................   0
PF00105      ..............................................................................................................   0
PF02166      .....GLGRVYPRPPSKTYRGAFQNLFQSVREVIQNPGPRHPEAASAAPPGASLLLLQQQQQQQQQQQQQQQQQQQQQQQETSPRQQQQQQGEDGSPQAHRRGPTGYLVL   105
PF00104.2    ..............................................................................................................   0
PF02166.1    ..............................................................................................................   0
PF00104      ..............................................................................................................   0
AR-209       MEVQLGLGRVYPRPPSKTYRGAFQNLFQSVREVIQNPGPRHPEAASAAPPGASLLLLQQQQQQQQQQQQQQQQQQQQQQQETSPRQQQQQQGEDGSPQAHRRGPTGYLVL   110
AR-203       MEVQLGLGRVYPRPPSKTYRGAFQNLFQSVREVIQNPGPRHPEAASAAPPGASLLLLQQQQQQQQQQQQQQQQQQQQQQQETSPRQQQQQQGEDGSPQAHRRGPTGYLVL   110
AR-202       ..............................................................................................................   0
AR-204       MEVQLGLGRVYPRPPSKTYRGAFQNLFQSVREVIQNPGPRHPEAASAAPPGASLLLLQQQQQQQQQQQQQQQQQQQQQQQETSPRQQQQQQGEDGSPQAHRRGPTGYLVL   110
AR-206       MEVQLGLGRVYPRPPSKTYRGAFQNLFQSVREVIQNPGPRHPEAASAAPPGASLLLLQQQQQQQQQQQQQQQQQQQQQQQETSPRQQQQQQGEDGSPQAHRRGPTGYLVL   110
AR-201       MEVQLGLGRVYPRPPSKTYRGAFQNLFQSVREVIQNPGPRHPEAASAAPPGASLLLLQQQQQQQQQQQQQQQQQQQQQQQETSPRQQQQQQGEDGSPQAHRRGPTGYLVL   110
AR-207       MEVQLGLGRVYPRPPSKTYRGAFQNLFQSVREVIQNPGPRHPEAASAAPPGASLLLLQQQQQQQQQQQQQQQQQQQQQQQETSPRQQQQQQGEDGSPQAHRRGPTGYLVL   110
AR-208       ..............................................................................................................   0

logo
             DEEQQPSQPQSALECHPERGCVPEPGAAVAASKGLPQQLPAPPDEDDSAAPSTLSLLGPTFPGLSSCSADLKDILSEASTMQLLQQQQQEAVSEGSSSGRAREASGAPTS
FLUTAMIDE    ..............................................................................................................   0
PF00104.1    ..............................................................................................................   0
PF00105.1    ..............................................................................................................   0
PF00105      ..............................................................................................................   0
PF02166      DEEQQPSQPQSALECHPERGCVPEPGAAVAASKGLPQQLPAPPDEDDSAAPSTLSLLGPTFPGLSSCSADLKDILSEASTMQLLQQQQQEAVSEGSSSGRAREASGAPTS   215
PF00104.2    ..............................................................................................................   0
PF02166.1    ....................................................................................QQQQQEAVSEGSSSGRAREASGAPTS   26
PF00104      ..............................................................................................................   0
AR-209       DEEQQPSQPQSALECHPERGCVPEPGAAVAASKGLPQQLPAPPDEDDSAAPSTLSLLGPTFPGLSSCSADLKDILSEASTMQLLQQQQQEAVSEGSSSGRAREASGAPTS   220
AR-203       DEEQQPSQPQSALECHPERGCVPEPGAAVAASKGLPQQLPAPPDEDDSAAPSTLSLLGPTFPGLSSCSADLKDILSEASTMQLLQQQQQEAVSEGSSSGRAREASGAPTS   220
AR-202       ..............................................................................................................   0
AR-204       DEEQQPSQPQSALECHPERGCVPEPGAAVAASKGLPQQLPAPPDEDDSAAPSTLSLLGPTFPGLSSCSADLKDILSEASTMQLLQQQQQEAVSEGSSSGRAREASGAPTS   220
AR-206       DEEQQPSQPQSALECHPERGCVPEPGAAVAASKGLPQQLPAPPDEDDSAAPSTLSLLGPTFPGLSSCSADLKDILSEASTMQLLQQQQQEAVSEGSSSGRAREASGAPTS   220
AR-201       DEEQQPSQPQSALECHPERGCVPEPGAAVAASKGLPQQLPAPPDEDDSAAPSTLSLLGPTFPGLSSCSADLKDILSEASTMQLLQQQQQEAVSEGSSSGRAREASGAPTS   220
AR-207       DEEQQPSQPQSALECHPERGCVPEPGAAVAASKGLPQQLPAPPDEDDSAAPSTLSLLGPTFPGLSSCSADLKDILSEASTMQLLQQQQQEAVSEGSSSGRAREASGAPTS   220
AR-208       ................................................................................MQLLQQQQQEAVSEGSSSGRAREASGAPTS   30
```

```
logo        SKDNYLGGTSTISDNAKELCKAVSVSMGLGVEALEHLSPGEQLRGDCMYAPLLGVPPAVRPTPCAPLAECKGSLLDDSAGKSTEDTAEYSPFKGGYTKGLEGESLGCSGS
FLUTAMIDE   ..............................................................................................................   0
PF00104.1   ..............................................................................................................   0
PF00105.1   ..............................................................................................................   0
PF00105     ..............................................................................................................   0
PF02166     SKDNYLGGTSTISDNAKELCKAVSVSMGLGVEALEHLSPGEQLRGDCMYAPLLGVPPAVRPTPCAPLAECKGSLLDDSAGKSTEDTAEYSPFKGGYTKGLEGESLGCSGS 325
PF00104.2   ..............................................................................................................   0
PF02166.1   SKDNYLGGTSTISDNAKELCKAVSVSMGLGVEALEHLSPGEQLRGDCMYAPLLGVPPAVRPTPCAPLAECKGSLLDDSAGKSTEDTAEYSPFKGGYTKGLEGESLGCSGS 136
PF00104     ..............................................................................................................   0
AR-209      SKDNYLGGTSTISDNAKELCKAVSVSMGLGVEALEHLSPGEQLRGDCMYAPLLGVPPAVRPTPCAPLAECKGSLLDDSAGKSTEDTAEYSPFKGGYTKGLEGESLGCSGS 330
AR-203      SKDNYLGGTSTISDNAKELCKAVSVSMGLGVEALEHLSPGEQLRGDCMYAPLLGVPPAVRPTPCAPLAECKGSLLDDSAGKSTEDTAEYSPFKGGYTKGLEGESLGCSGS 330
AR-202      ..............................................................................................................   0
AR-204      SKDNYLGGTSTISDNAKELCKAVSVSMGLGVEALEHLSPGEQLRGDCMYAPLLGVPPAVRPTPCAPLAECKGSLLDDSAGKSTEDTAEYSPFKGGYTKGLEGESLGCSGS 330
AR-206      SKDNYLGGTSTISDNAKELCKAVSVSMGLGVEALEHLSPGEQLRGDCMYAPLLGVPPAVRPTPCAPLAECKGSLLDDSAGKSTEDTAEYSPFKGGYTKGLEGESLGCSGS 330
AR-201      SKDNYLGGTSTISDNAKELCKAVSVSMGLGVEALEHLSPGEQLRGDCMYAPLLGVPPAVRPTPCAPLAECKGSLLDDSAGKSTEDTAEYSPFKGGYTKGLEGESLGCSGS 330
AR-207      SKDNYLGGTSTISDNAKELCKAVSVSMGLGVEALEHLSPGEQLRGDCMYAPLLGVPPAVRPTPCAPLAECKGSLLDDSAGKSTEDTAEYSPFKGGYTKGLEGESLGCSGS 330
AR-208      SKDNYLGGTSTISDNAKELCKAVSVSMGLGVEALEHLSPGEQLRGDCMYAPLLGVPPAVRPTPCAPLAECKGSLLDDSAGKSTEDTAEYSPFKGGYTKGLEGESLGCSGS 140
```

```
logo        AAAGSSGTLELPSTLSLYKSGALDEAAAYQSRDYYNFPLALAGPPPPPPPPHPHARIKLENPLDYGSAWAAAAAQCRYGDLASLHGAGAAGPGSGSPSAAASSSWHTLFT
FLUTAMIDE   ..............................................................................................................   0
PF00104.1   ..............................................................................................................   0
PF00105.1   ..............................................................................................................   0
PF00105     ..............................................................................................................   0
PF02166     AAAGSSGTLELPSTLSLYKSGALDEAAAYQSRDYYNFPLALAGPPPPPPPPHPHARIKLENPLDYGSAWAAAAAQCRYGDLASLHGAGAAGPGSGSPSAAASSSWHTLFT 435
PF00104.2   ..............................................................................................................   0
PF02166.1   AAAGSSGTLELPSTLSLYKSGALDEAAAYQSRDYYNFPLALAGPPPPPPPPHPHARIKLENPLDYGSAWAAAAAQCRYGDLASLHGAGAAGPGSGSPSAAASSSWHTLFT 246
PF00104     ..............................................................................................................   0
AR-209      AAAGSSGTLELPSTLSLYKSGALDEAAAYQSRDYYNFPLALAGPPPPPPPPHPHARIKLENPLDYGSAWAAAAAQCRYGDLASLHGAGAAGPGSGSPSAAASSSWHTLFT 440
AR-203      AAAGSSGTLELPSTLSLYKSGALDEAAAYQSRDYYNFPLALAGPPPPPPPPHPHARIKLENPLDYGSAWAAAAAQCRYGDLASLHGAGAAGPGSGSPSAAASSSWHTLFT 440
AR-202      ..............................................................................................................   0
AR-204      AAAGSSGTLELPSTLSLYKSGALDEAAAYQSRDYYNFPLALAGPPPPPPPPHPHARIKLENPLDYGSAWAAAAAQCRYGDLASLHGAGAAGPGSGSPSAAASSSWHTLFT 440
AR-206      AAAGSSGTLELPSTLSLYKSGALDEAAAYQSRDYYNFPLALAGPPPPPPPPHPHARIKLENPLDYGSAWAAAAAQCRYGDLASLHGAGAAGPGSGSPSAAASSSWHTLFT 440
AR-201      AAAGSSGTLELPSTLSLYKSGALDEAAAYQSRDYYNFPLALAGPPPPPPPPHPHARIKLENPLDYGSAWAAAAAQCRYGDLASLHGAGAAGPGSGSPSAAASSSWHTLFT 440
AR-207      AAAGSSGTLELPSTLSLYKSGALDEAAAYQSRDYYNFPLALAGPPPPPPPPHPHARIKLENPLDYGSAWAAAAAQCRYGDLASLHGAGAAGPGSGSPSAAASSSWHTLFT 440
AR-208      AAAGSSGTLELPSTLSLYKSGALDEAAAYQSRDYYNFPLALAGPPPPPPPPHPHARIKLENPLDYGSAWAAAAAQCRYGDLASLHGAGAAGPGSGSPSAAASSSWHTLFT 250
```

logo

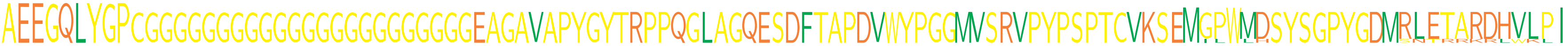

```
FLUTAMIDE   ..............................................................................................................   0
PF00104.1   ..............................................................................................................   0
PF00105.1   ..............................................................................................................   0
PF00105     ..............................................................................................................   0
PF02166     AEEGQLYGP.....................................................................................................   444
PF00104.2   ..............................................................................................................   0
PF02166.1   AEEGQLYGP.....................................................................................................   255
PF00104     ..............................................................................................................   0
AR-209      AEEGQLYGPCGGGGGGGGGGGGGGGGGGGGGGGEAGAVAPYGYTRPPQGLAGQESDFTAPDVWYPGGMVSRVPYPSPTCVKSEMGPWMDSYSGPYGDMRNTRRKRLWKLI   550
AR-203      AEEGQLYGPCGGGGGGGGGGGGGGGGGGGGGGGEAGAVAPYGYTRPPQGLAGQESDFTAPDVWYPGGMVSRVPYPSPTCVKSEMGPWMDSYSGPYGDMRLETARDHVLPI   550
AR-202      ...................................................................................MILWLH.........SLETARDHVLPI   18
AR-204      AEEGQLYGPCGGGGGGGGGGGGGGGGGGGGGGGEAGAVAPYGYTRPPQGLAGQESDFTAPDVWYPGGMVSRVPYPSPTCVKSEMGPWMDSYSGPYGDMRLETARDHVLPI   550
AR-206      AEEGQLYGPCGGGGGGGGGGGGGGGGGGGGGGGEAGAVAPYGYTRPPQGLAGQESDFTAPDVWYPGGMVSRVPYPSPTCVKSEMGPWMDSYSGPYGDMRLETARDHVLPI   550
AR-201      AEEGQLYGPCGGGGGGGGGGGGGGGGGGGGGGGEAGAVAPYGYTRPPQGLAGQESDFTAPDVWYPGGMVSRVPYPSPTCVKSEMGPWMDSYSGPYGDMRLETARDHVLPI   550
AR-207      AEEGQLYGPCGGGGGGGGGGGGGGGGGGGGGGGEAGAVAPYGYTRPPQGLAGQESDFTAPDVWYPGGMVSRVPYPSPTCVKSEMGPWMDSYSGPYGDMRLETARDHVLPI   550
AR-208      AEEGQLYGPCGGGGGGGGGGGGGGGGGGGGGGGEAGAVAPYGYTRPPQGLAGQESDFTAPDVWYPGGMVSRVPYPSPTCVKSEMGPWMDSYSGPYGDMRLETARDHVLPI   360
```

logo

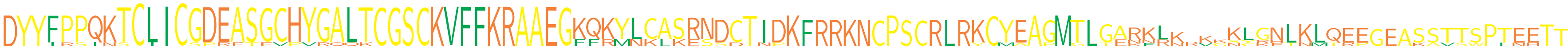

```
FLUTAMIDE   ...............................................................................................................   0
PF00104.1   ...............................................................................................................   0
PF00105.1   ........TCLICGDEASGCHYGALTCGSCKVFFKRAAEG.......................................................................   32
PF00105     ........TCLICGDEASGCHYGALTCGSCKVFFKRAAEGKQKYLCASRNDCTIDKFRRKNCPSCRLRKCYEAGMT...................................   68
PF02166     ...............................................................................................................   444
PF00104.2   ...............................................................................................................   0
PF02166.1   ...............................................................................................................   255
PF00104     ...............................................................................................................   0
AR-209      ...IRSINSCI.CSPRETEVPVRQQK.....................................................................................   572
AR-203      DYYFPPQKTCLICGDEASGCHYGALTCGSCKVFFKRAAEGKQKYLCASRNDCTIDKFRRKNCPSCRLRKCYEAGMTLGARKL..K.KLGNLKLQEEGEASSTTSPTEETT   657
AR-202      DYYFPPQKTCLICGDEASGCHYGALTCGSCKVFFKRAAEGKQKYLCASRNDCTIDKFRRKNCPSCRLRKCYEAGMTLGARKLK...KLGNLKLQEEGEASSTTSPTEETT   125
AR-204      DYYFPPQKTCLICGDEASGCHYGALTCGSCKVFFKRAAEGKQKYLCASRNDCTIDKFRRKNCPSCRLRKCYEAGMTLGEKFR..VGNCKHLKMTRP..............   644
AR-206      DYYFPPQKTCLICGDEASGCHYGALTCGSCKVFFKRAAEGFFRMNKLKES.............................................................   600
AR-201      DYYFPPQKTCLICGDEASGCHYGALTCGSCKVFFKRAAEGKQKYLCASRNDCTIDKFRRKNCPSCRLRKCYEAGMTLGARKLK...KLGNLKLQEEGEASSTTSPTEETT   657
AR-207      DYYFPPQKTCLICGDEASGCHYGALTCGSCKVFFKRAAEGFFRMNKLKESSDTNPK......P.....YCMAAPMGLTENNRNRKKSYRETNL......KAVSWPLNHT.   642
AR-208      DYYFPPQKTCLICGDEASGCHYGALTCGSCKVFFKRAAEGKQKYLCASRNDCTIDKFRRKNCPSCRLRKCYEAGMTLGARKLK...KLGNLKLQEEGEASSTTSPTEETT   467
```

```
logo
             QKLTVSHIEGYECQPIFLNVLEAIEPGVVCAGHDNNQPDSFAALLSSLNELGERQLVHVVKWAKALPGFRNLHVDDQMAVIQYSWMGLMVFAMGWRSFTNVNSRMLYFAP
FLUTAMIDE    ............................................L..LN.LG..Q..............................M..MV..M..R...........F..   12
PF00104.1    .....................................PDSFAALLSSLNELGERQLVHVVKWAKALPGFRNLHVDDQMAVIQYSWMGLMVFAMGWRSFTNVNSRMLYFAP   73
PF00105.1    ..............................................................................................................   32
PF00105      ..............................................................................................................   68
PF02166      ..............................................................................................................  444
PF00104.2    ......................................DSFAALLSSLNELGERQLVHVVKWAKALPGFRNLHVDDQMAVIQYSWMGLMVFAMGWRSFTNVNSRMLYFAP   72
PF02166.1    ..............................................................................................................  255
PF00104      ......................................DSFAALLSSLNELGERQLVHVVKWAKALPGFRNLHVDDQMAVIQYSWMGLMVFAMGWRSFTNVNSRMLYFAP   72
AR-209       ..............................................................................................................  572
AR-203       QKLTVSHIEGYECQPIFLNVLEAIEPGVVCAGHDNNQPDSFAALLSSLNELGERQLVHVVKWAKALPDCERAASVHF.................................  734
AR-202       QKLTVSHIEGYECQPIFLNVLEAIEPGVVCAGHDNNQPDSFAALLSSLNELGERQLVHVVKWAKALPGFRNLHVDDQMAVIQYSWMGLMVFAMGWRSFTNVNSRMLYFAP  235
AR-204       ..............................................................................................................  644
AR-206       ..............................................................................................................  600
AR-201       QKLTVSHIEGYECQPIFLNVLEAIEPGVVCAGHDNNQPDSFAALLSSLNELGERQLVHVVKWAKALPGFRNLHVDDQMAVIQYSWMGLMVFAMGWRSFTNVNSRMLYFAP  767
AR-207       ..............................................................................................................  642
AR-208       QKLTVSHIEGYECQPIFLNVLEAIEPGVVCAGHDNNQPDSFAALLSSLNELGERQLVHVVKWAKALPGFRNLHVDDQMAVIQYSWMGLMVFAMGWRSFTNVNSRMLYFAP  577

logo
             DLVFNEYRMHKSRMYSQCVRMRHLSQEFGWLQITPQEFLCMKALLLFSIKANLFARIPVDGLKNQKFFDELRMNYIKELDRIIACKRKNPTSCSRRFYQLTKLLDSVQPI
FLUTAMIDE    ....................M.........................................................................................   13
PF00104.1    DLVFNEYRMHKSRMYSQCVRMRHLSQEFGWLQITPQEFLCMKALLLFSI.......IPVDGLKNQKFFDELRMNYIKELDRIIACKRKNPTSCSRRFYQLTKLLDSVQPI  176
PF00105.1    ..............................................................................................................   32
PF00105      ..............................................................................................................   68
PF02166      ..............................................................................................................  444
PF00104.2    DLVFNEYRMHKSRMYSQCVRMRHLSQEFGWLQITPQEFLCMKALLLFSIKANLFARIPVDGLKNQKFFDELRMNYIKELDRIIACKRKNPTSCSRRFYQLTKLLDSVQPI  182
PF02166.1    ..............................................................................................................  255
PF00104      DLVFNEYRMHKSRMYSQCVRMRHLSQEFGWLQITPQEFLCMKALLLFSI.......IPVDGLKNQKFFDELRMNYIKELDRIIACKRKNPTSCSRRFYQLTKLLDSVQPI  175
AR-209       ..............................................................................................................  572
AR-203       ..............................................................................................................  734
AR-202       DLVFNEYRMHKSRMYSQCVRMRHLSQEFGWLQITPQEFLCMKALLLFSI.......IPVDGLKNQKFFDELRMNYIKELDRIIACKRKNPTSCSRRFYQLTKLLDSVQPI  338
AR-204       ..............................................................................................................  644
AR-206       ..............................................................................................................  600
AR-201       DLVFNEYRMHKSRMYSQCVRMRHLSQEFGWLQITPQEFLCMKALLLFSI.......IPVDGLKNQKFFDELRMNYIKELDRIIACKRKNPTSCSRRFYQLTKLLDSVQPI  870
AR-207       ..............................................................................................................  642
AR-208       DLVFNEYRMHKSRMYSQCVRMRHLSQEFGWLQITPQEFLCMKALLLFSIKANLFARIPVDGLKNQKFFDELRMNYIKELDRIIACKRKNPTSCSRRFYQLTKLLDSVQPI  687
```

logo

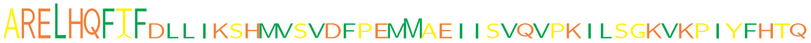


```
FLUTAMIDE    ...L...A.................M........................   16
PF00104.1    ARELHQFTF.........................................  185
PF00105.1    ..................................................   32
PF00105      ..................................................   68
PF02166      ..................................................  444
PF00104.2    ARELHQFTF.........................................  191
PF02166.1    ..................................................  255
PF00104      ARELHQFTF.........................................  184
AR-209       ..................................................  572
AR-203       ..................................................  734
AR-202       ARELHQFTFDLLIKSHMVSVDFPEMMAEIISVQVPKILSGKVKPIYFHTQ  388
AR-204       ..................................................  644
AR-206       ..................................................  600
AR-201       ARELHQFTFDLLIKSHMVSVDFPEMMAEIISVQVPKILSGKVKPIYFHTQ  920
AR-207       ..................................................  642
AR-208       ARELHQFTFDLLIKSHMVSVDFPEMMAEIISVQVPKILSGKVKPIYFHTQ  737
```

- X non conserved
- X similar
- X ≥ 0% conserved
- X ≥ 50% conserved

```
logo
```
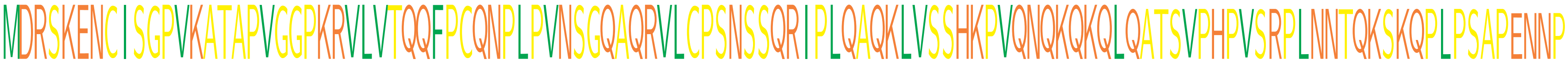

```
DANUSERTIB  ..............................................................................................................    0
PF00069     ..............................................................................................................    0
PF00069.2   ..............................................................................................................    0
PF00069.1   ..............................................................................................................    0
AURKA-212   MDRSKENCISGPVKATAPVGGPKRVLVTQQFPCQNPLPVNSGQAQRVLCPSNSSQRIPLQAQKLVSSHKPVQNQKQKQL...............................   79
AURKA-206   MDRSKENCISGPVKATAPVGGPKRVLVTQQFPCQNPLPVNSGQAQRVLCPSNSSQRIPLQAQKLVSSHKPVQNQKQKQLQATSVPHPVSRPLNNTQKSKQPLPSAPENNP  110
AURKA-209   MDRSKENCISGPVKATAPVGGPKRVLVTQQFPCQNPLPVNSGQAQRVLCPSNSSQRIPLQAQKLVSSHKPVQNQKQKQLQATSVPHPVSRPLNNTQKSKQPLPSAPENNP  110
AURKA-201   MDRSKENCISGPVKATAPVGGPKRVLVTQQFPCQNPLPVNSGQAQRVLCPSNSSQRIPLQAQKLVSSHKPVQNQKQKQLQATSVPHPVSRPLNNTQKSKQPLPSAPENNP  110
AURKA-205   MDRSKENCISGPVKATAPVGGPKRVLVTQQFPCQNPLPVNSGQAQRVLCPSNSSQRIPLQAQKLVSSHKPVQNQKQKQLQATSVPHPVSRPLNNTQKSKQPLPSAPENNP  110
AURKA-213   MDRSKENCISGPVKATAPVGGPKRVLVTQQFPCQNPLPVNSGQAQRVLCPSNSSQRIPLQAQKLVSSHKPVQNQKQKQLQATSVPHPVSRPLNNTQKSKQPLPSAP....  106
AURKA-211   MDRSKENCISGPVKATAPVGGPKRVLVTQQFPCQNPLPVNSGQAQRVLCPSNSSQRIPLQAQKLVSSHKPVQNQKQKQLQATSVPHPVSRPLNNTQKSKQPLPSAPENNP  110
AURKA-204   MDRSKENCISGPVKATAPVGGPKRVLVTQQFPCQNPLPVNSGQAQRVLCPSNSSQRIPLQAQKLVSSHKPVQNQKQKQLQATSVPHPVSRPLNNTQKSKQPLPSAPENNP  110
AURKA-207   MDRSKENCISGPVKATAPVGGPKRVLVTQQFPCQNPLPVNSGQAQRVLCPSNSSQRIPLQAQKLVSSHKPVQNQKQKQLQATSVPHPVSRPLNNTQKSKQPLPSAPENNP  110
AURKA-203   MDRSKENCISGPVKATAPVGGPKRVLVTQQFPCQNPLPVNSGQAQRVLCPSNSSQRIPLQAQKLVSSHKPVQNQKQKQLQATSVPHPVSRPLNNTQKSKQPLPSAPENNP  110
AURKA-202   MDRSKENCISGPVKATAPVGGPKRVLVTQQFPCQNPLPVNSGQAQRVLCPSNSSQRIPLQAQKLVSSHKPVQNQKQKQLQATSVPHPVSRPLNNTQKSKQPLPSAPENNP  110
AURKA-208   MDRSKENCISGPVKATAPVGGPKRVLVTQQFPCQNPLPVNSGQAQRVLCPSNSSQRIPLQAQKLVSSHKPVQNQKQKQLQATSVPHPVSRPLNNTQKSKQPLPSAPENNP  110
AURKA-210   MDRSKENCISGPVKATAPVGGPKRVLVTQQFPCQNPLPVNSGQAQRVLCPSNSSQRIPLQAQKLVSSHKPVQNQKQKQLQATSVPHPVSRPLNNTQKSKQPLPSAPENNP  110
```

```
logo
```
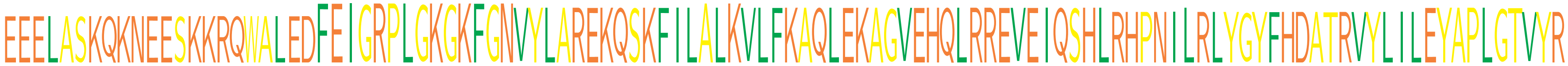

```
DANUSERTIB  .................................................A.K...............................L................EYAPLGT...   10
PF00069     ......................FEIGRPLGKGKFGNVYLAREKQSKFILALKVLFKAQLEKAGVEHQLRREVEIQSHLRHPNILRLYGYFHDATRVYLILEYAPLGTVYR   88
PF00069.2   ......................FEIGRPLGKGKFGNVYLAREKQSKFILALKVLFKAQLEKAGVEHQLRREVEIQSHLRHPNILRLYGYFHDATRVYLILEYAPLGTVYR   88
PF00069.1   ......................FEIGRPLGKGKFGNVYLAREKQSKFILALKVLFKAQLEKAGVEHQLRREVEIQSHLRHPNILRLYGYFHDATRVYLILEYAPLGTVYR   88
AURKA-212   ..............................................................................................................   79
AURKA-206   EEELASKQKNEESKKRQWALEDFEIGRPLGKGKFGNVYLAREKQSKFILALKVLFKAQLEKAGVEHQLRREVEIQSHLRHPNILRLYGYFHDATRVYLILEYAPLGTVYR  220
AURKA-209   EEELASKQKNEESKKRQWALEDFEIGRPLGKGKFGNVYLAREKQSKFILALKVLFKAQLEKAGVEHQLRREVEIQSHL................................  188
AURKA-201   EEELASKQKNEESKKRQWALEDFEIGRPLGKGKFGNVYLAREKQSKFILALKVLFKAQLEKAGVEHQLRREVEIQSHLRHPNILRLYGYFHDATRVYLILEYAPLGTVYR  220
AURKA-205   EEELASKQKNEESKKRQWALEDFEIGRPLGKGKFGNVYLAREKQSKFILALKVLFKAQLEKAGVEHQLRREVEIQSHLRHPNILRLYGYFHDATRVYLILEYAPLGTVYR  220
AURKA-213   ..............................................................................................................  106
AURKA-211   EEELASKQKNEESKKRQWALEDFEIGRPLGKGKFGNVYLAREKQSKFILALKVLFKAQLEKAGVEHQLRREVEIQSHLRHPNILRLYGYFHDATRVYLILEYAPLGTVYR  220
AURKA-204   EEELASKQKNEESKKRQWALEDFEIGRPLGKGKFGNVYLAREKQSKFILALKVLFKAQLEKAGVEHQLRREVEIQSHLRHPNILRLYGYFHDATRVYLILEYAPLGTVYR  220
AURKA-207   EEELASKQKNEESKKRQWALEDFEIGRPLGKGKFGNVYLAREKQSKFILALKVLFKAQLEKAGVEHQLRREVEIQSHLRHPNILRLYGYFHDATRVYLILEYAPLGTVYR  220
AURKA-203   EEELASKQKNEESKKRQWALEDFEIGRPLGKGKFGNVYLAREKQSKFILALKVLFKAQLEKAGVEHQLRREVEIQSHLRHPNILRLYGYFHDATRVYLILEYAPLGTVYR  220
AURKA-202   EEELASKQKNEESKKRQWALEDFEIGRPLGKGKFGNVYLAREKQSKFILALKVLFKAQLEKAGVEHQLRREVEIQSHLRHPNILRLYGYFHDATRVYLILEYAPLGTVYR  220
AURKA-208   EEELASKQKNEESKKRQWALEDFEIGRPLGKGKFGNVYLAREKQSKFILALKVLFKAQLEKAGVEHQLRREVEIQSHLRHPNILRLYGYFHDATRVYLILEYAPLGTVYR  220
AURKA-210   EEELASKQKNEESKKRQWALEDFEIGRPLGKGKFGN..........................................................................  146
```

```
logo        ELQKLSKFDEQRTATYITELANALSYCHSKRVIHRDIKPENLLLGSAGELKIADFGWSVHAPSSRRTTLCGTLDYLPPEMIEGRMHDEKVDLWSLGVLCYEFLVGKPPFE
DANUSERTIB  .......................................E..L.........A.F.......................................................  14
PF00069     ELQKLSKFDEQRTATYITELANALSYCHSKRVIHRDIKPENLLLGSAGELKIADFGWSVHAPSSRRTTLCGTLDYLPPEMIEGRMHDEKVDLWSLGVLCYEFLVGKPPFE 198
PF00069.2   ELQKLSKFDEQRTATYITELANALSYCHSKRVIHRDIKPENLLLGSAGELK........................................................... 139
PF00069.1   ELQKLSKFDEQRTATYITELANALSYCHSKRVIHRDIKPENLLLGSAGELKIADFGWSVHAPSSRRTTLCGTLDYLPPEMIEGRMHDEKVDLWSLGVLCYEFLVGKPPFE 198
AURKA-212   ..............................................................................................................  79
AURKA-206   ELQKLSKFDEQRTATYITELANALSYCHSKRVIHRDIKPENLLLGSAGELKIADFGWSVHAPSSRRTTLCGTLDYLPPEMIEGRMHDEKVDLWSLGVLCYEFLVGKPPFE 330
AURKA-209   .............................................................................................................. 188
AURKA-201   ELQKLSKFDEQRTATYITELANALSYCHSKRVIHRDIKPENLLLGSAGELKIADFGWSVHAPSSRRTTLCGTLDYLPPEMIEGRMHDEKVDLWSLGVLCYEFLVGKPPFE 330
AURKA-205   ELQKLSKFDEQRTATYITELANALSYCHSKRVIHRDIKPENLLLGSAGELKIADFGWSVHAPSSRRTTLCGTLDYLPPEMIEGRMHDEKVDLWSLGVLCYEFLVGKPPFE 330
AURKA-213   .............................................................................................................. 106
AURKA-211   ELQKLSKFDEQRTATYITELANALSYCHSKRVIHRDIKPENLLLGSAGELK........................................................... 271
AURKA-204   ELQKLSKFDEQRTATYITELANALSYCHSKRVIHRDIKPENLLLGSAGELKIADFGWSVHAPSSRRTTLCGTLDYLPPEMIEGRMHDEKVDLWSLGVLCYEFLVGKPPFE 330
AURKA-207   ELQKLSKFDEQRTATYITELANALSYCHSKRVIHRDIKPENLLLGSAGELKIADFGWSVHAPSSRRTTLCGTLDYLPPEMIEGRMHDEKVDLWSLGVLCYEFLVGKPPFE 330
AURKA-203   ELQKLSKFDEQRTATYITELANALSYCHSKRVIHRDIKPENLLLGSAGELKIADFGWSVHAPSSRRTTLCGTLDYLPPEMIEGRMHDEKVDLWSLGVLCYEFLVGKPPFE 330
AURKA-202   ELQKLSKFDEQRTATYITELANALSYCHSKRVIHRDIKPENLLLGSAGELKIADFGWSVHAPSSRRTTLCGTLDYLPPEMIEGRMHDEKVDLWSLGVLCYEFLVGKPPFE 330
AURKA-208   ELQKLSKFDEQRTATYITELANALSYCHSKRVIHRDIKPENLLLGSAGELKIADFGWSVHAPSSRRTTLCGTLDYLPPEMIEGRMHDEKVDLWSLGVLCYEFLVGKPPFE 330
AURKA-210   .............................................................................................................. 146

logo        ANTYQETYKRISRVEFTFPDFVTEGARDLISRLLKHNPSQRPMLREVLEHPWITANSSKPSNCQNKESASKQS
DANUSERTIB  .........................................................................  14
PF00069     ANTYQETYKRISRVEFTFPDFVTEGARDLISRLLKHNPSQRPMLREVLEHPWI.................... 251
PF00069.2   ......................................................................... 139
PF00069.1   ANTYQETYKRI.............................................................. 209
AURKA-212   .........................................................................  79
AURKA-206   ANTYQETYKRISRVEFTFPDFVTEGARDLISRLLKHNPSQRPMLREVLEHPWITANSSKPSNCQNKESASKQS 403
AURKA-209   ......................................................................... 188
AURKA-201   ANTYQETYKRISRVEFTFPDFVTEGARDLISRLLKHNPSQRPMLREVLEHPWITANSSKPSNCQNKESASKQS 403
AURKA-205   ANTYQETYKRISRVEFTFPDFVTEGARDLISRLLKHNPSQRPMLREVLEHPWITANSSKPSNCQNKESASKQS 403
AURKA-213   ......................................................................... 106
AURKA-211   ......................................................................... 271
AURKA-204   ANTYQETYKRISRVRRN........................................................ 347
AURKA-207   ANTYQETYKRISRVEFTFPDFVTEGARDLISRLLKHNPSQRPMLREVLEHPWITANSSKPSNCQNKESASKQS 403
AURKA-203   ANTYQETYKRISRVEFTFPDFVTEGARDLISRLLKHNPSQRPMLREVLEHPWITANSSKPSNCQNKESASKQS 403
AURKA-202   ANTYQETYKRISRVEFTFPDFVTEGARDLISRLLKHNPSQRPMLREVLEHPWITANSSKPSNCQNKESASKQS 403
AURKA-208   ANTYQETYKRISRVEFTFPDFVTEGARDLISRLLKHNPSQRPMLREVLEHPWITANSSKPSNCQNKESASKQS 403
AURKA-210   ......................................................................... 146
```

X non conserved
X similar
X ≥ 0% conserved
X ≥ 50% conserved

logo

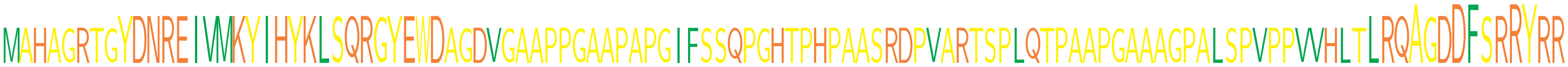

```
NAVITOCLAX  ...................................................................................................A..DF..RY..   5
PF02180     ........YDNREIVMKYIHYKLSQRGYEWD...............................................................................  23
PF00452     ................................................................................................LRQAGDDFSRRYRR  14
BCL2-201    MAHAGRTGYDNREIVMKYIHYKLSQRGYEWDAGDVGAAPPGAAPAPGIFSSQPGHTPHPAASRDPVARTSPLQTPAAPGAAAGPALSPVPPVVHLTLRQAGDDFSRRYRR 110
BCL2-203    MAHAGRTGYDNREIVMKYIHYKLSQRGYEWDAGDVGAAPPGAAPAPGIFSSQPGHTPHPAASRDPVARTSPLQTPAAPGAAAGPALSPVPPVVHLTLRQAGDDFSRRYRR 110
BCL2-202    MAHAGRTGYDNREIVMKYIHYKLSQRGYEWDAGDVGAAPPGAAPAPGIFSSQPGHTPHPAASRDPVARTSPLQTPAAPGAAAGPALSPVPPVVHLTLRQAGDDFSRRYRR 110
```

logo

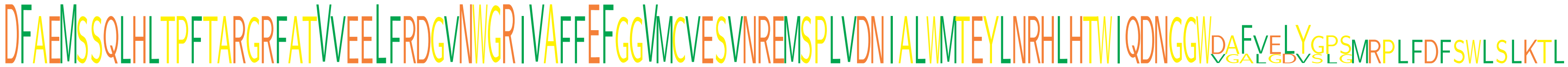

```
NAVITOCLAX  DF..M.................V...L.....NWGR.VA..EF..V.........................................F..LY..................  22
PF02180     ..............................................................................................................  23
PF00452     DFAEMSSQLHLTPFTARGRFATVVEELFRDGVNWGRIVAFFEFGGVMCVESVNREMSPLVDNIALWMTEYLNRHLHTWIQDNGGW.........................  99
BCL2-201    DFAEMSSQLHLTPFTARGRFATVVEELFRDGVNWGRIVAFFEFGGVMCVESVNREMSPLVDNIALWMTEYLNRHLHTWIQDNGGWDAFVELYGPSMRPLFDFSWLSLKTL 220
BCL2-203    DFAEMSSQLHLTPFTARGRFATVVEELFRDGVNWGRIVAFFEFGGVMCVESVNREMSPLVDNIALWMTEYLNRHLHTWIQDNGGWVGALGDVSLG............... 205
BCL2-202    DFAEMSSQLHLTPFTARGRFATVVEELFRDGVNWGRIVAFFEFGGVMCVESVNREMSPLVDNIALWMTEYLNRHLHTWIQDNGGWDAFVELYGPSMRPLFDFSWLSLKTL 220
```

logo

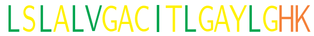

```
NAVITOCLAX  ...................  22
PF02180     ...................  23
PF00452     ...................  99
BCL2-201    LSLALVGACITLGAYLGHK 239
BCL2-203    ................... 205
BCL2-202    LSLALVGACITLGAYLGHK 239
```

X non conserved
X similar
X ≥ 0% conserved
X ≥ 50% conserved

```
logo
             MAALSGGGGGGGAEPGQALFNGDMEPEAGAGAGAAASSAADPAIPEEVWNIKQMIKLTQEHIEALLDKFGGEHNPPSIYLEAYEEYTSKLDALQQREQQLLESLGNGTDFS
CHIR-265     ..............................................................................................................    0
PF02196      ..............................................................................................................    0
PF00130.1    ..............................................................................................................    0
PF07714      ..............................................................................................................    0
PF02196.1    ..............................................................................................................    0
PF00130      ..............................................................................................................    0
BRAF-204     ..............................................................................................................    0
BRAF-205     ...............QALFNGDMEPEAGAGAGAAASSAADPAIPEEVWNIKQMIKLTQEHIEALLDKFGGEHNPPSIYLEAYEEYTSKLDALQQREQQLLESLGNGTDFS   95
BRAF-201     MAALSGGGGGGGAEPGQALFNGDMEPEAGAGAGAAASSAADPAIPEEVWNIKQMIKLTQEHIEALLDKFGGEHNPPSIYLEAYEEYTSKLDALQQREQQLLESLGNGTDFS  110
BRAF-203     ..............................................................................................................    0

logo
             VSSSASMDTVTSSSSSSLSVLPSSLSVFQNPTDVARSNPKSPQKPIVRVFLPNKQRTVVPARCGVTVRDSLKKALMMRGLIPECCAVYRIQDGEKKPIGWDTDISWLTGE
CHIR-265     ..............................................................................................................    0
PF02196      ..............................................VRVFLPNKQRTVVPARCGVTVRDSLKKALMMRGLIPECCAVYRIQDGEKKPIGWDTDISWLTGE   64
PF00130.1    ..............................................................................................................    0
PF07714      ..............................................................................................................    0
PF02196.1    ..............................................................................................................    0
PF00130      ..............................................................................................................    0
BRAF-204     ..............................................................................................................    0
BRAF-205     VSSSASMDTVTSSSSSSLSVLPSSLSVFQNPTDVARSNPKSPQKPIVRVFLPNKQRTVVPARCGVTVRDSLKKALMMRGLIPECCAVYRIQDGSFLELT...........  194
BRAF-201     VSSSASMDTVTSSSSSSLSVLPSSLSVFQNPTDVARSNPKSPQKPIVRVFLPNKQRTVVPARCGVTVRDSLKKALMMRGLIPECCAVYRIQDGEKKPIGWDTDISWLTGE  220
BRAF-203     ..............................................................................................................    0

logo
             ELHVEVLENVPLTTHNFVRKTFFTLAFCDFCRKLLFQGFRCQTCGYKFHQRCSTEVPLMCVNYDQLDLLFVSKFFEHHPIPQEEASLAETALTSGSSPSAPASDSIGPQI
CHIR-265     ..............................................................................................................    0
PF02196      ELHVE.........................................................................................................   69
PF00130.1    ..............................................................................................................    0
PF07714      ..............................................................................................................    0
PF02196.1    ..............................................................................................................    0
PF00130      ..............HNFVRKTFFTLAFCDFCRKLLFQGFRCQTCGYKFHQRCSTEVPLMC..................................................   46
BRAF-204     ..............................................................................................................    0
BRAF-205     ..............................................................................................................  194
BRAF-201     ELHVEVLENVPLTTHNFVRKTFFTLAFCDFCRKLLFQGFRCQTCGYKFHQRCSTEVPLMCVNYDQLDLLFVSKFFEHHPIPQEEASLAETALTSGSSPSAPASDSIGPQI  330
BRAF-203     ..............................................................................................................    0

logo
             LTSPSPSKSIPIPQPFRPADEDHRNQFGQRDRSSSAPNVHINTIEPVNIDDLIRDQGFRGDGXSTTGLSATPPASLPGSLTNVKALQKSPGPQRERKSSSSSEDRNRMKT
CHIR-265     ..............................................................................................................    0
PF02196      ..............................................................................................................   69
PF00130.1    ..............................................................................................................    0
PF07714      ..............................................................................................................    0
PF02196.1    ..............................................................................................................    0
PF00130      ..............................................................................................................   46
BRAF-204     ..............................................................XSTTGLSATPPASLPGSLTNVKALQKSPGPQRERKSSSSSEDRNRMKT   48
BRAF-205     ..............................................................................................................  194
BRAF-201     LTSPSPSKSIPIPQPFRPADEDHRNQFGQRDRSSSAPNVHINTIEPVNIDDLIRDQGFRGDGGSTTGLSATPPASLPGSLTNVKALQKSPGPQRERKSSSSSEDRNRMKT  440
BRAF-203     ..............................................................................................................    0
```

```
logo         LGRRDSSDDWEIPDGQITVGQRIGSGSFGTVYKGKWHGDVAVKMLNVTAPTPQQLQAFKNEVGVLRKTRHVNILLFMGYSTKPQLAIVTQWCEGSSLYHHLHIIETKFEM
CHIR-265     ......................I.......V.........A.K.................E...L.......IL............I.TQWC.G................   14
PF02196      ..............................................................................................................   69
PF00130.1    ..............................................................................................................    0
PF07714      .........................................................FKNEVGVLRKTRHVNILLFMGYSTKPQLAIVTQWCEGSSLYHHLHIIETKFEM   53
PF02196.1    ............................................................................................................EM    2
PF00130      ..............................................................................................................   46
BRAF-204     LGRRDSSDDWEIPDGQITVGQRIGSGSFGTVYKGKWHGDVAVKMLNVTAPTPQQLQAFKNEVGVLRKTRHVNILLFMGYSTKPQLAIVTQWCEGSSLYHHLHIIETKFEM  158
BRAF-205     ..............................................................................................................  194
BRAF-201     LGRRDSSDDWEIPDGQITVGQRIGSGSFGTVYKGKWHGDVAVKMLNVTAPTPQQLQAFKNEVGVLRKTRHVNILLFMGYSTKPQLAIVTQWCEGSSLYHHLHIIETKFEM  550
BRAF-203     ..............................................................................................................    0

logo         IKLIDIARQTAQGMDYLHAKSIIHRDLKSNNIFLHEDLTVKIGDFGLATVKSRWSGSHQFEQLSGSILWMAPEVIRMQDKNPYSFQSDVYAFGIVLYELMTGQLPYSNIN
CHIR-265     ................L......H........F........IGDF.................................................................   21
PF02196      ..............................................................................................................   69
PF00130.1    ............................................................................MQDKNPYSFQSDVYAFGIVLYELMTGQLPYSNIN   34
PF07714      IKLIDIARQTAQGMDYLHAKSIIHRDLKSNNIFLHEDLTVKIGDFGLATVKSRWSGSHQFEQLSGSILWMAPEVIRMQDKNPYSFQSDVYAFGIVLYELMTGQLPYSNIN  163
PF02196.1    IKLIDIARQTAQGMDYLHAKSIIHRDLKSNNIFLHEDLTVKIGDFGLATVKSRWSGSHQFEQLSGSI...........................................   69
PF00130      ..............................................................................................................   46
BRAF-204     IKLIDIARQTAQGMDYLHAKSIIHRDLKSNNIFLHEDLTVKIGDFGLATVKSRWSGSHQFEQLSGSILWMAPEVIRMQDKNPYSFQSDVYAFGIVLYELMTGQLPYSNIN  268
BRAF-205     ..............................................................................................................  194
BRAF-201     IKLIDIARQTAQGMDYLHAKSIIHRDLKSNNIFLHEDLTVKIGDFGLATVKSRWSGSHQFEQLSGSILWMAPEVIRMQDKNPYSFQSDVYAFGIVLYELMTGQLPYSNIN  660
BRAF-203     .......................IHRDLKSNNIFLHEDLTVKIGDFGLATVKSRWSGSHQFEQLSGSILWMAPEVIRMQDKNPYSFQSDVYAFGIVLYELMTGQLPYSNIN   88

logo         NRDQIIFMVGRGYLSPDLSKVRSNCPKAMKRLMAECLKKKRDERPLFPQILASIELLARSLPKIHRSASEPSLNRAGFQTEDFSLYACASPKTPIQAGGYG
CHIR-265     ...........................................................................................................   21
PF02196      ...........................................................................................................   69
PF00130.1    NRDQIIFMVGRG...............................................................................................   46
PF07714      NRDQIIFMVGRGYLSPDLSKVRSNCPKAMKRLMAECLKKKRDERPLFPQILASIELLARSLPKIHRSASEPSLNRAGFQTEDFSLYACASPK...............  255
PF02196.1    ...........................................................................................................   69
PF00130      ...........................................................................................................   46
BRAF-204     NRDQIIFMVGRGYLSPDLSKVRSNCPKAMKRLMAECLKKKRDERPLFPQILASIELLARSLPKIHRSASEPSLNRAGFQTEDFSLYACASPKTPIQAGGYGEFAAFK  375
BRAF-205     ...........................................................................................................  194
BRAF-201     NRDQIIFMVGRGYLSPDLSKVRSNCPKAMKRLMAECLKKKRDERPLFPQILASIELLARSLPKIHRSASEPSLNRAGFQTEDFSLYACASPKTPIQAGGYGAFPVH.  766
BRAF-203     NRDQVLCPPWE.YNK............................................................................................  102
```

- X non conserved
- X similar
- X $\geq 0\%$ conserved
- X $\geq 50\%$ conserved

```
logo
```
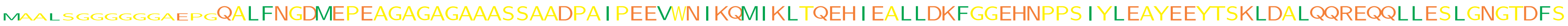

```
DABRAFENIB   ..............................................................................................................    0
PF02196      ..............................................................................................................    0
PF00130.1    ..............................................................................................................    0
PF07714      ..............................................................................................................    0
PF02196.1    ..............................................................................................................    0
PF00130      ..............................................................................................................    0
BRAF-204     ..............................................................................................................    0
BRAF-205     ...............QALFNGDMEPEAGAGAGAAASSAADPAIPEEVWNIKQMIKLTQEHIEALLDKFGGEHNPPSIYLEAYEEYTSKLDALQQREQQLLESLGNGTDFS   95
BRAF-201     MAALSGGGGGGAEPGQALFNGDMEPEAGAGAGAAASSAADPAIPEEVWNIKQMIKLTQEHIEALLDKFGGEHNPPSIYLEAYEEYTSKLDALQQREQQLLESLGNGTDFS  110
BRAF-203     ..............................................................................................................    0
```

```
logo
```
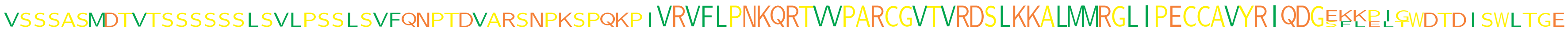

```
DABRAFENIB   ..............................................................................................................    0
PF02196      ..............................................VRVFLPNKQRTVVPARCGVTVRDSLKKALMMRGLIPECCAVYRIQDGEKKPIGWDTDISWLTGE   64
PF00130.1    ..............................................................................................................    0
PF07714      ..............................................................................................................    0
PF02196.1    ..............................................................................................................    0
PF00130      ..............................................................................................................    0
BRAF-204     ..............................................................................................................    0
BRAF-205     VSSSASMDTVTSSSSSSLSVLPSSLSVFQNPTDVARSNPKSPQKPIVRVFLPNKQRTVVPARCGVTVRDSLKKALMMRGLIPECCAVYRIQDGSFLELT...........  194
BRAF-201     VSSSASMDTVTSSSSSSLSVLPSSLSVFQNPTDVARSNPKSPQKPIVRVFLPNKQRTVVPARCGVTVRDSLKKALMMRGLIPECCAVYRIQDGEKKPIGWDTDISWLTGE  220
BRAF-203     ..............................................................................................................    0
```

```
logo
```
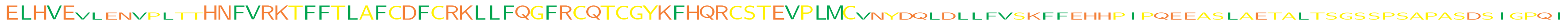

```
DABRAFENIB   ..............................................................................................................    0
PF02196      ELHVE.........................................................................................................   69
PF00130.1    ..............................................................................................................    0
PF07714      ..............................................................................................................    0
PF02196.1    ..............................................................................................................    0
PF00130      ..............HNFVRKTFFTLAFCDFCRKLLFQGFRCQTCGYKFHQRCSTEVPLMC..................................................   46
BRAF-204     ..............................................................................................................    0
BRAF-205     ..............................................................................................................  194
BRAF-201     ELHVEVLENVPLTTHNFVRKTFFTLAFCDFCRKLLFQGFRCQTCGYKFHQRCSTEVPLMCVNYDQLDLLFVSKFFEHHPIPQEEASLAETALTSGSSPSAPASDSIGPQI  330
BRAF-203     ..............................................................................................................    0
```

```
logo
```
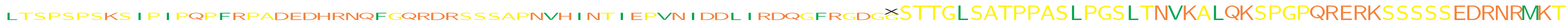

```
DABRAFENIB   ..............................................................................................................    0
PF02196      ..............................................................................................................   69
PF00130.1    ..............................................................................................................    0
PF07714      ..............................................................................................................    0
PF02196.1    ..............................................................................................................    0
PF00130      ..............................................................................................................   46
BRAF-204     ..............................................................XSTTGLSATPPASLPGSLTNVKALQKSPGPQRERKSSSSSEDRNRMKT   48
BRAF-205     ..............................................................................................................  194
BRAF-201     LTSPSPSKSIPIPQPFRPADEDHRNQFGQRDRSSSAPNVHINTIEPVNIDDLIRDQGFRGDGGSTTGLSATPPASLPGSLTNVKALQKSPGPQRERKSSSSSEDRNRMKT  440
BRAF-203     ..............................................................................................................    0
```

```
logo
DABRAFENIB   ......................IGSG.F..V.........A.K....................VL........L.F..........I.TQWC..................   17
PF02196      ..............................................................................................................   69
PF00130.1    ..............................................................................................................    0
PF07714      .........................................................FKNEVGVLRKTRHVNILLFMGYSTKPQLAIVTQWCEGSSLYHHLHIIETKFEM   53
PF02196.1    ............................................................................................................EM    2
PF00130      ..............................................................................................................   46
BRAF-204     LGRRDSSDDWEIPDGQITVGQRIGSGSFGTVYKGKWHGDVAVKMLNVTAPTPQQLQAFKNEVGVLRKTRHVNILLFMGYSTKPQLAIVTQWCEGSSLYHHLHIIETKFEM  158
BRAF-205     ..............................................................................................................  194
BRAF-201     LGRRDSSDDWEIPDGQITVGQRIGSGSFGTVYKGKWHGDVAVKMLNVTAPTPQQLQAFKNEVGVLRKTRHVNILLFMGYSTKPQLAIVTQWCEGSSLYHHLHIIETKFEM  550
BRAF-203     ..............................................................................................................    0

logo
DABRAFENIB   ................................F.........GDF.................................................................   21
PF02196      ..............................................................................................................   69
PF00130.1    ............................................................................MQDKNPYSFQSDVYAFGIVLYELMTGQLPYSNIN   34
PF07714      IKLIDIARQTAQGMDYLHAKSIIHRDLKSNNIFLHEDLTVKIGDFGLATVKSRWSGSHQFEQLSGSILWMAPEVIRMQDKNPYSFQSDVYAFGIVLYELMTGQLPYSNIN  163
PF02196.1    IKLIDIARQTAQGMDYLHAKSIIHRDLKSNNIFLHEDLTVKIGDFGLATVKSRWSGSHQFEQLSGSI...........................................   69
PF00130      ..............................................................................................................   46
BRAF-204     IKLIDIARQTAQGMDYLHAKSIIHRDLKSNNIFLHEDLTVKIGDFGLATVKSRWSGSHQFEQLSGSILWMAPEVIRMQDKNPYSFQSDVYAFGIVLYELMTGQLPYSNIN  268
BRAF-205     ..............................................................................................................  194
BRAF-201     IKLIDIARQTAQGMDYLHAKSIIHRDLKSNNIFLHEDLTVKIGDFGLATVKSRWSGSHQFEQLSGSILWMAPEVIRMQDKNPYSFQSDVYAFGIVLYELMTGQLPYSNIN  660
BRAF-203     ......................IHRDLKSNNIFLHEDLTVKIGDFGLATVKSRWSGSHQFEQLSGSILWMAPEVIRMQDKNPYSFQSDVYAFGIVLYELMTGQLPYSNIN   88

logo
DABRAFENIB   ...........................................................................................................   21
PF02196      ...........................................................................................................   69
PF00130.1    NRDQIIFMVGRG...............................................................................................   46
PF07714      NRDQIIFMVGRGYLSPDLSKVRSNCPKAMKRLMAECLKKKRDERPLFPQILASIELLARSLPKIHRSASEPSLNRAGFQTEDFSLYACASPK...............  255
PF02196.1    ...........................................................................................................   69
PF00130      ...........................................................................................................   46
BRAF-204     NRDQIIFMVGRGYLSPDLSKVRSNCPKAMKRLMAECLKKKRDERPLFPQILASIELLARSLPKIHRSASEPSLNRAGFQTEDFSLYACASPKTPIQAGGYGEFAAFK  375
BRAF-205     ...........................................................................................................  194
BRAF-201     NRDQIIFMVGRGYLSPDLSKVRSNCPKAMKRLMAECLKKKRDERPLFPQILASIELLARSLPKIHRSASEPSLNRAGFQTEDFSLYACASPKTPIQAGGYGAFPVH.  766
BRAF-203     NRDQVLCPPWE.YNK............................................................................................  102
```

- X non conserved
- X similar
- X $\geq 0\%$ conserved
- X $\geq 50\%$ conserved

```
logo
           MAALSGGGGGGAEPGQALFNGDMEPEAGAGAGAAASSAADPAIPEEVWNIKQMIKLTQEHIEALLDKFGGEHNPPSIYLEAYEEYTSKLDALQQREQQLLESLGNGTDFS
PLX-4720   ..............................................................................................................    0
PF02196    ..............................................................................................................    0
PF00130.1  ..............................................................................................................    0
PF07714    ..............................................................................................................    0
PF02196.1  ..............................................................................................................    0
PF00130    ..............................................................................................................    0
BRAF-204   ..............................................................................................................    0
BRAF-205   ...............QALFNGDMEPEAGAGAGAAASSAADPAIPEEVWNIKQMIKLTQEHIEALLDKFGGEHNPPSIYLEAYEEYTSKLDALQQREQQLLESLGNGTDFS   95
BRAF-201   MAALSGGGGGGAEPGQALFNGDMEPEAGAGAGAAASSAADPAIPEEVWNIKQMIKLTQEHIEALLDKFGGEHNPPSIYLEAYEEYTSKLDALQQREQQLLESLGNGTDFS  110
BRAF-203   ..............................................................................................................    0

logo
           VSSSASMDTVTSSSSSSLSVLPSSLSVFQNPTDVARSNPKSPQKPIVRVFLPNKQRTVVPARCGVTVRDSLKKALMMRGLIPECCAVYRIQDGSFLELIGWDTDISWLTGE
PLX-4720   ..............................................................................................................    0
PF02196    ..............................................VRVFLPNKQRTVVPARCGVTVRDSLKKALMMRGLIPECCAVYRIQDGEKKPIGWDTDISWLTGE   64
PF00130.1  ..............................................................................................................    0
PF07714    ..............................................................................................................    0
PF02196.1  ..............................................................................................................    0
PF00130    ..............................................................................................................    0
BRAF-204   ..............................................................................................................    0
BRAF-205   VSSSASMDTVTSSSSSSLSVLPSSLSVFQNPTDVARSNPKSPQKPIVRVFLPNKQRTVVPARCGVTVRDSLKKALMMRGLIPECCAVYRIQDGSFLELT...........  194
BRAF-201   VSSSASMDTVTSSSSSSLSVLPSSLSVFQNPTDVARSNPKSPQKPIVRVFLPNKQRTVVPARCGVTVRDSLKKALMMRGLIPECCAVYRIQDGEKKPIGWDTDISWLTGE  220
BRAF-203   ..............................................................................................................    0

logo
           ELHVEVLENVPLTTHNFVRKTFFTLAFCDFCRKLLFQGFRCQTCGYKFHQRCSTEVPLMCVNYDQLDLLFVSKFFEHHPIPQEEASLAETALTSGSSPSAPASDSIGPQI
PLX-4720   ..............................................................................................................    0
PF02196    ELHVE.........................................................................................................   69
PF00130.1  ..............................................................................................................    0
PF07714    ..............................................................................................................    0
PF02196.1  ..............................................................................................................    0
PF00130    ..............HNFVRKTFFTLAFCDFCRKLLFQGFRCQTCGYKFHQRCSTEVPLMC..................................................   46
BRAF-204   ..............................................................................................................    0
BRAF-205   ..............................................................................................................  194
BRAF-201   ELHVEVLENVPLTTHNFVRKTFFTLAFCDFCRKLLFQGFRCQTCGYKFHQRCSTEVPLMCVNYDQLDLLFVSKFFEHHPIPQEEASLAETALTSGSSPSAPASDSIGPQI  330
BRAF-203   ..............................................................................................................    0

logo
           LTSPSPSKSIPIPQPFRPADEDHRNQFGQRDRSSSAPNVHINTIEPVNIDDLIRDQGFRGDGXSTTGLSATPPASLPGSLTNVKALQKSPGPQRERKSSSSSEDRNRMKT
PLX-4720   ..............................................................................................................    0
PF02196    ..............................................................................................................   69
PF00130.1  ..............................................................................................................    0
PF07714    ..............................................................................................................    0
PF02196.1  ..............................................................................................................    0
PF00130    ..............................................................................................................   46
BRAF-204   ..............................................................XSTTGLSATPPASLPGSLTNVKALQKSPGPQRERKSSSSSEDRNRMKT   48
BRAF-205   ..............................................................................................................  194
BRAF-201   LTSPSPSKSIPIPQPFRPADEDHRNQFGQRDRSSSAPNVHINTIEPVNIDDLIRDQGFRGDGGSTTGLSATPPASLPGSLTNVKALQKSPGPQRERKSSSSSEDRNRMKT  440
BRAF-203   ..............................................................................................................    0
```

```
logo        LGRRDSSDDWEIPDGQITVGQRIGSGSFGTVYKGKWHGDVAVKMLNVTAPTPQQLQAFKNEVGVLRKTRHVNILLFMGYSTKPQLAIVTQWCEGSSLYHHLHIIETKFEM
PLX-4720    ......................I.......V.........A.K.................E...L........L.F..........I.TQWC..S...............     14
PF02196     ..............................................................................................................     69
PF00130.1   ..............................................................................................................      0
PF07714     .........................................................FKNEVGVLRKTRHVNILLFMGYSTKPQLAIVTQWCEGSSLYHHLHIIETKFEM     53
PF02196.1   ............................................................................................................EM      2
PF00130     ..............................................................................................................     46
BRAF-204    LGRRDSSDDWEIPDGQITVGQRIGSGSFGTVYKGKWHGDVAVKMLNVTAPTPQQLQAFKNEVGVLRKTRHVNILLFMGYSTKPQLAIVTQWCEGSSLYHHLHIIETKFEM    158
BRAF-205    ..............................................................................................................    194
BRAF-201    LGRRDSSDDWEIPDGQITVGQRIGSGSFGTVYKGKWHGDVAVKMLNVTAPTPQQLQAFKNEVGVLRKTRHVNILLFMGYSTKPQLAIVTQWCEGSSLYHHLHIIETKFEM    550
BRAF-203    ..............................................................................................................      0

logo        IKLIDIARQTAQGMDYLHAKSIIHRDLKSNNIFLHEDLTVKIGDFGLATVKSRWSGSHQFEQLSGSILWMAPEVIRMQDKNPYSFQSDVYAFGIVLYELMTGQLPYSNIN
PLX-4720    ................................F.........GDFG................................................................     19
PF02196     ..............................................................................................................     69
PF00130.1   ............................................................................MQDKNPYSFQSDVYAFGIVLYELMTGQLPYSNIN     34
PF07714     IKLIDIARQTAQGMDYLHAKSIIHRDLKSNNIFLHEDLTVKIGDFGLATVKSRWSGSHQFEQLSGSILWMAPEVIRMQDKNPYSFQSDVYAFGIVLYELMTGQLPYSNIN    163
PF02196.1   IKLIDIARQTAQGMDYLHAKSIIHRDLKSNNIFLHEDLTVKIGDFGLATVKSRWSGSHQFEQLSGSI...........................................     69
PF00130     ..............................................................................................................     46
BRAF-204    IKLIDIARQTAQGMDYLHAKSIIHRDLKSNNIFLHEDLTVKIGDFGLATVKSRWSGSHQFEQLSGSILWMAPEVIRMQDKNPYSFQSDVYAFGIVLYELMTGQLPYSNIN    268
BRAF-205    ..............................................................................................................    194
BRAF-201    IKLIDIARQTAQGMDYLHAKSIIHRDLKSNNIFLHEDLTVKIGDFGLATVKSRWSGSHQFEQLSGSILWMAPEVIRMQDKNPYSFQSDVYAFGIVLYELMTGQLPYSNIN    660
BRAF-203    ......................IHRDLKSNNIFLHEDLTVKIGDFGLATVKSRWSGSHQFEQLSGSILWMAPEVIRMQDKNPYSFQSDVYAFGIVLYELMTGQLPYSNIN     88

logo        NRDQIIFMVGRGYLSPDLSKVRSNCPKAMKRLMAECLKKKRDERPLFPQILASIELLARSLPKIHRSASEPSLNRAGFQTEDFSLYACASPKTPIQAGGYGEFAAFK
PLX-4720    ...........................................................................................................     19
PF02196     ...........................................................................................................     69
PF00130.1   NRDQIIFMVGRG...............................................................................................     46
PF07714     NRDQIIFMVGRGYLSPDLSKVRSNCPKAMKRLMAECLKKKRDERPLFPQILASIELLARSLPKIHRSASEPSLNRAGFQTEDFSLYACASPK...............    255
PF02196.1   ...........................................................................................................     69
PF00130     ...........................................................................................................     46
BRAF-204    NRDQIIFMVGRGYLSPDLSKVRSNCPKAMKRLMAECLKKKRDERPLFPQILASIELLARSLPKIHRSASEPSLNRAGFQTEDFSLYACASPKTPIQAGGYGEFAAFK    375
BRAF-205    ...........................................................................................................    194
BRAF-201    NRDQIIFMVGRGYLSPDLSKVRSNCPKAMKRLMAECLKKKRDERPLFPQILASIELLARSLPKIHRSASEPSLNRAGFQTEDFSLYACASPKTPIQAGGYGAFPVH.    766
BRAF-203    NRDQVLCPPWE.YNK............................................................................................    102
```

X non conserved
X similar
X ≥ 0% conserved
X ≥ 50% conserved

```
logo
             MAALSGGGGGGAEPGQALFNGDMEPEAGAGAGAAASSAADPAIPEEVWNIKQMIKLTQEHIEALLDKFGGEHNPPSIYLEAYEEYTSKLDALQQREQQLLESLGNGTDFS
SORAFENIB    ..............................................................................................................    0
PF02196      ..............................................................................................................    0
PF00130.1    ..............................................................................................................    0
PF07714      ..............................................................................................................    0
PF02196.1    ..............................................................................................................    0
PF00130      ..............................................................................................................    0
BRAF-204     ..............................................................................................................    0
BRAF-205     ...............QALFNGDMEPEAGAGAGAAASSAADPAIPEEVWNIKQMIKLTQEHIEALLDKFGGEHNPPSIYLEAYEEYTSKLDALQQREQQLLESLGNGTDFS   95
BRAF-201     MAALSGGGGGGAEPGQALFNGDMEPEAGAGAGAAASSAADPAIPEEVWNIKQMIKLTQEHIEALLDKFGGEHNPPSIYLEAYEEYTSKLDALQQREQQLLESLGNGTDFS  110
BRAF-203     ..............................................................................................................    0

logo
             VSSSASMDTVTSSSSSSLSVLPSSLSVFQNPTDVARSNPKSPQKPIVRVFLPNKQRTVVPARCGVTVRDSLKKALMMRGLIPECCAVYRIQDGEKKPIGWDTDISWLTGE
SORAFENIB    ..............................................................................................................    0
PF02196      ..............................................VRVFLPNKQRTVVPARCGVTVRDSLKKALMMRGLIPECCAVYRIQDGEKKPIGWDTDISWLTGE   64
PF00130.1    ..............................................................................................................    0
PF07714      ..............................................................................................................    0
PF02196.1    ..............................................................................................................    0
PF00130      ..............................................................................................................    0
BRAF-204     ..............................................................................................................    0
BRAF-205     VSSSASMDTVTSSSSSSLSVLPSSLSVFQNPTDVARSNPKSPQKPIVRVFLPNKQRTVVPARCGVTVRDSLKKALMMRGLIPECCAVYRIQDGSFLELT...........  194
BRAF-201     VSSSASMDTVTSSSSSSLSVLPSSLSVFQNPTDVARSNPKSPQKPIVRVFLPNKQRTVVPARCGVTVRDSLKKALMMRGLIPECCAVYRIQDGEKKPIGWDTDISWLTGE  220
BRAF-203     ..............................................................................................................    0

logo
             ELHVEVLENVPLTTTHNFVRKTFFTLAFCDFCRKLLFQGFRCQTCGYKFHQRCSTEVPLMCVNYDQLDLLFVSKFFEHHPIPQEEASLAETALTSGSSPSAPASDSIGPQI
SORAFENIB    ..............................................................................................................    0
PF02196      ELHVE.........................................................................................................   69
PF00130.1    ..............................................................................................................    0
PF07714      ..............................................................................................................    0
PF02196.1    ..............................................................................................................    0
PF00130      ...............HNFVRKTFFTLAFCDFCRKLLFQGFRCQTCGYKFHQRCSTEVPLMC.................................................   46
BRAF-204     ..............................................................................................................    0
BRAF-205     ..............................................................................................................  194
BRAF-201     ELHVEVLENVPLTTTHNFVRKTFFTLAFCDFCRKLLFQGFRCQTCGYKFHQRCSTEVPLMCVNYDQLDLLFVSKFFEHHPIPQEEASLAETALTSGSSPSAPASDSIGPQI  330
BRAF-203     ..............................................................................................................    0

logo
             LTSPSPSKSIPIPQPFRPADEDHRNQFGQRDRSSSAPNVHINTIEPVNIDDLIRDQGFRGDGXSTTGLSATPPASLPGSLTNVKALQKSPGPQRERKSSSSSEDRNRMKT
SORAFENIB    ..............................................................................................................    0
PF02196      ..............................................................................................................   69
PF00130.1    ..............................................................................................................    0
PF07714      ..............................................................................................................    0
PF02196.1    ..............................................................................................................    0
PF00130      ..............................................................................................................   46
BRAF-204     ..............................................................XSTTGLSATPPASLPGSLTNVKALQKSPGPQRERKSSSSSEDRNRMKT   48
BRAF-205     ..............................................................................................................  194
BRAF-201     LTSPSPSKSIPIPQPFRPADEDHRNQFGQRDRSSSAPNVHINTIEPVNIDDLIRDQGFRGDGGSTTGLSATPPASLPGSLTNVKALQKSPGPQRERKSSSSSEDRNRMKT  440
BRAF-203     ..............................................................................................................    0
```

logo

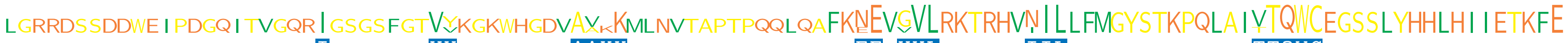

```
SORAFENIB   ......................I.......VV........AAKK................EE.VVL......IIL.............TTQWC.................   20
PF02196     ..............................................................................................................   69
PF00130.1   ..............................................................................................................    0
PF07714     ..........................................................FKNEVGVLRKTRHVNILLFMGYSTKPQLAIVTQWCEGSSLYHHLHIIETKFE   52
PF02196.1   .............................................................................................................E    1
PF00130     ..............................................................................................................   46
BRAF-204    LGRRDSSDDWEIPDGQITVGQRIGSGSFGTVYKGKWHGDVAV.KMLNVTAPTPQQLQAFKNEVGVLRKTRHVNILLFMGYSTKPQLAIVTQWCEGSSLYHHLHIIETKFE  157
BRAF-205    ..............................................................................................................  194
BRAF-201    LGRRDSSDDWEIPDGQITVGQRIGSGSFGTVYKGKWHGDVAV.KMLNVTAPTPQQLQAFKNEVGVLRKTRHVNILLFMGYSTKPQLAIVTQWCEGSSLYHHLHIIETKFE  549
BRAF-203    ..............................................................................................................    0
```

logo

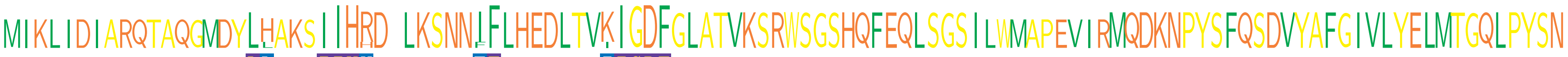

```
SORAFENIB   .................LL...IIHH.......FF.......IIGDF...............................................................   33
PF02196     ..............................................................................................................   69
PF00130.1   ..............................................................................MQDKNPYSFQSDVYAFGIVLYELMTGQLPYSN   32
PF07714     MIKLIDIARQTAQGMDYLHAKSIIHRD.LKSNNIFLHEDLTVKIGDFGLATVKSRWSGSHQFEQLSGSILWMAPEVIRMQDKNPYSFQSDVYAFGIVLYELMTGQLPYSN  161
PF02196.1   MIKLIDIARQTAQGMDYLHAKSIIHRD.LKSNNIFLHEDLTVKIGDFGLATVKSRWSGSHQFEQLSGSI.........................................   69
PF00130     ..............................................................................................................   46
BRAF-204    MIKLIDIARQTAQGMDYLHAKSIIHRD.LKSNNIFLHEDLTVKIGDFGLATVKSRWSGSHQFEQLSGSILWMAPEVIRMQDKNPYSFQSDVYAFGIVLYELMTGQLPYSN  266
BRAF-205    ..............................................................................................................  194
BRAF-201    MIKLIDIARQTAQGMDYLHAKSIIHRD.LKSNNIFLHEDLTVKIGDFGLATVKSRWSGSHQFEQLSGSILWMAPEVIRMQDKNPYSFQSDVYAFGIVLYELMTGQLPYSN  658
BRAF-203    .......................IHRD.LKSNNIFLHEDLTVKIGDFGLATVKSRWSGSHQFEQLSGSILWMAPEVIRMQDKNPYSFQSDVYAFGIVLYELMTGQLPYSN   86
```

logo

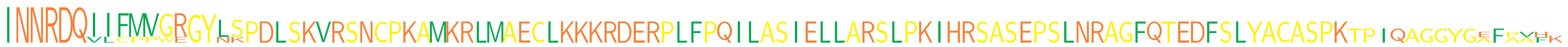

```
SORAFENIB   .............................................................................................................   33
PF02196     .............................................................................................................   69
PF00130.1   INNRDQIIFMVGRG...............................................................................................   46
PF07714     INNRDQIIFMVGRGYLSPDLSKVRSNCPKAMKRLMAECLKKKRDERPLFPQILASIELLARSLPKIHRSASEPSLNRAGFQTEDFSLYACASPK...............  255
PF02196.1   .............................................................................................................   69
PF00130     .............................................................................................................   46
BRAF-204    INNRDQIIFMVGRGYLSPDLSKVRSNCPKAMKRLMAECLKKKRDERPLFPQILASIELLARSLPKIHRSASEPSLNRAGFQTEDFSLYACASPKTPIQAGGYGEFAAFK  375
BRAF-205    .............................................................................................................  194
BRAF-201    INNRDQIIFMVGRGYLSPDLSKVRSNCPKAMKRLMAECLKKKRDERPLFPQILASIELLARSLPKIHRSASEPSLNRAGFQTEDFSLYACASPKTPIQAGGYGAFPVH.  766
BRAF-203    INNRDQVLCPPWE.YNK............................................................................................  102
```

- X non conserved
- X similar
- X ≥ 0% conserved
- X ≥ 50% conserved

```
logo
             MAALSGGGGGGGAEPGQALFNGDMEPEAGAGAGAAASSAADPAIPEEVWNIKQMIKLTQEHIEALLDKFGGEHNPPSIYLEAYEEYTSKLDALQQREQQLLESLGNGTDFS
VEMURAFENIB  ..............................................................................................................     0
PF02196      ..............................................................................................................     0
PF00130.1    ..............................................................................................................     0
PF07714      ..............................................................................................................     0
PF02196.1    ..............................................................................................................     0
PF00130      ..............................................................................................................     0
BRAF-204     ..............................................................................................................     0
BRAF-205     ...............QALFNGDMEPEAGAGAGAAASSAADPAIPEEVWNIKQMIKLTQEHIEALLDKFGGEHNPPSIYLEAYEEYTSKLDALQQREQQLLESLGNGTDFS    95
BRAF-201     MAALSGGGGGGGAEPGQALFNGDMEPEAGAGAGAAASSAADPAIPEEVWNIKQMIKLTQEHIEALLDKFGGEHNPPSIYLEAYEEYTSKLDALQQREQQLLESLGNGTDFS   110
BRAF-203     ..............................................................................................................     0

logo
             VSSSASMDTVTSSSSSSLSVLPSSLSVFQNPTDVARSNPKSPQKPIVRVFLPNKQRTVVPARCGVTVRDSLKKALMMRGLIPECCAVYRIQDGEKKPIGWDTDISWLTGE
VEMURAFENIB  ..............................................................................................................     0
PF02196      ..............................................VRVFLPNKQRTVVPARCGVTVRDSLKKALMMRGLIPECCAVYRIQDGEKKPIGWDTDISWLTGE    64
PF00130.1    ..............................................................................................................     0
PF07714      ..............................................................................................................     0
PF02196.1    ..............................................................................................................     0
PF00130      ..............................................................................................................     0
BRAF-204     ..............................................................................................................     0
BRAF-205     VSSSASMDTVTSSSSSSLSVLPSSLSVFQNPTDVARSNPKSPQKPIVRVFLPNKQRTVVPARCGVTVRDSLKKALMMRGLIPECCAVYRIQDGSFLELT...........   194
BRAF-201     VSSSASMDTVTSSSSSSLSVLPSSLSVFQNPTDVARSNPKSPQKPIVRVFLPNKQRTVVPARCGVTVRDSLKKALMMRGLIPECCAVYRIQDGEKKPIGWDTDISWLTGE   220
BRAF-203     ..............................................................................................................     0

logo
             ELHVEVLENVPLTTHNFVRKTFFTLAFCDFCRKLLFQGFRCQTCGYKFHQRCSTEVPLMCVNYDQLDLLFVSKFFEHHPIPQEEASLAETALTSGSSPSAPASDSIGPQI
VEMURAFENIB  ..............................................................................................................     0
PF02196      ELHVE.........................................................................................................    69
PF00130.1    ..............................................................................................................     0
PF07714      ..............................................................................................................     0
PF02196.1    ..............................................................................................................     0
PF00130      ..............HNFVRKTFFTLAFCDFCRKLLFQGFRCQTCGYKFHQRCSTEVPLMC..................................................    46
BRAF-204     ..............................................................................................................     0
BRAF-205     ..............................................................................................................   194
BRAF-201     ELHVEVLENVPLTTHNFVRKTFFTLAFCDFCRKLLFQGFRCQTCGYKFHQRCSTEVPLMCVNYDQLDLLFVSKFFEHHPIPQEEASLAETALTSGSSPSAPASDSIGPQI   330
BRAF-203     ..............................................................................................................     0

logo
             LTSPSPSKSIPIPQPFRPADEDHRNQFGQRDRSSSAPNVHINTIEPVNIDDLIRDQGFRGDGGSTTGLSATPPASLPGSLTNVKALQKSPGPQRERKSSSSSEDRNRMKT
VEMURAFENIB  ..............................................................................................................     0
PF02196      ..............................................................................................................    69
PF00130.1    ..............................................................................................................     0
PF07714      ..............................................................................................................     0
PF02196.1    ..............................................................................................................     0
PF00130      ..............................................................................................................    46
BRAF-204     ..............................................................XSTTGLSATPPASLPGSLTNVKALQKSPGPQRERKSSSSSEDRNRMKT    48
BRAF-205     ..............................................................................................................   194
BRAF-201     LTSPSPSKSIPIPQPFRPADEDHRNQFGQRDRSSSAPNVHINTIEPVNIDDLIRDQGFRGDGGSTTGLSATPPASLPGSLTNVKALQKSPGPQRERKSSSSSEDRNRMKT   440
BRAF-203     ..............................................................................................................     0
```

logo

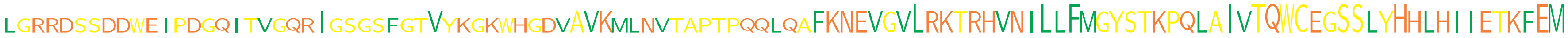

```
VEMURAFENIB  ......................I.......V.........AVK.....................L........L.F..........I.TQWC..SS..H...........   16
PF02196      ..............................................................................................................   69
PF00130.1    ..............................................................................................................    0
PF07714      .........................................................FKNEVGVLRKTRHVNILLFMGYSTKPQLAIVTQWCEGSSLYHHLHIIETKFEM   53
PF02196.1    ............................................................................................................EM    2
PF00130      ..............................................................................................................   46
BRAF-204     LGRRDSSDDWEIPDGQITVGQRIGSGSFGTVYKGKWHGDVAVKMLNVTAPTPQQLQAFKNEVGVLRKTRHVNILLFMGYSTKPQLAIVTQWCEGSSLYHHLHIIETKFEM  158
BRAF-205     ..............................................................................................................  194
BRAF-201     LGRRDSSDDWEIPDGQITVGQRIGSGSFGTVYKGKWHGDVAVKMLNVTAPTPQQLQAFKNEVGVLRKTRHVNILLFMGYSTKPQLAIVTQWCEGSSLYHHLHIIETKFEM  550
BRAF-203     ..............................................................................................................    0
```

logo

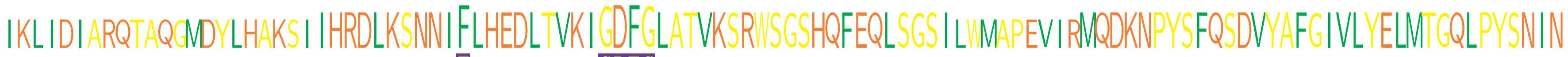

```
VEMURAFENIB  ................................F.........GDFG................................................................   21
PF02196      ..............................................................................................................   69
PF00130.1    .............................................................................MQDKNPYSFQSDVYAFGIVLYELMTGQLPYSNIN   34
PF07714      IKLIDIARQTAQGMDYLHAKSIIHRDLKSNNIFLHEDLTVKIGDFGLATVKSRWSGSHQFEQLSGSILWMAPEVIRMQDKNPYSFQSDVYAFGIVLYELMTGQLPYSNIN  163
PF02196.1    IKLIDIARQTAQGMDYLHAKSIIHRDLKSNNIFLHEDLTVKIGDFGLATVKSRWSGSHQFEQLSGSI...........................................   69
PF00130      ..............................................................................................................   46
BRAF-204     IKLIDIARQTAQGMDYLHAKSIIHRDLKSNNIFLHEDLTVKIGDFGLATVKSRWSGSHQFEQLSGSILWMAPEVIRMQDKNPYSFQSDVYAFGIVLYELMTGQLPYSNIN  268
BRAF-205     ..............................................................................................................  194
BRAF-201     IKLIDIARQTAQGMDYLHAKSIIHRDLKSNNIFLHEDLTVKIGDFGLATVKSRWSGSHQFEQLSGSILWMAPEVIRMQDKNPYSFQSDVYAFGIVLYELMTGQLPYSNIN  660
BRAF-203     ......................IHRDLKSNNIFLHEDLTVKIGDFGLATVKSRWSGSHQFEQLSGSILWMAPEVIRMQDKNPYSFQSDVYAFGIVLYELMTGQLPYSNIN   88
```

logo

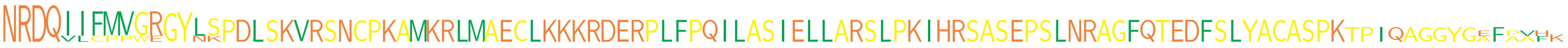

```
VEMURAFENIB  ...........................................................................................................   21
PF02196      ...........................................................................................................   69
PF00130.1    NRDQIIFMVGRG...............................................................................................   46
PF07714      NRDQIIFMVGRGYLSPDLSKVRSNCPKAMKRLMAECLKKKRDERPLFPQILASIELLARSLPKIHRSASEPSLNRAGFQTEDFSLYACASPK...............  255
PF02196.1    ...........................................................................................................   69
PF00130      ...........................................................................................................   46
BRAF-204     NRDQIIFMVGRGYLSPDLSKVRSNCPKAMKRLMAECLKKKRDERPLFPQILASIELLARSLPKIHRSASEPSLNRAGFQTEDFSLYACASPKTPIQAGGYGEFAAFK  375
BRAF-205     ...........................................................................................................  194
BRAF-201     NRDQIIFMVGRGYLSPDLSKVRSNCPKAMKRLMAECLKKKRDERPLFPQILASIELLARSLPKIHRSASEPSLNRAGFQTEDFSLYACASPKTPIQAGGYGAFPVH.  766
BRAF-203     NRDQVLCPPWE.YNK............................................................................................  102
```

X non conserved
X similar
X $\geq 0\%$ conserved
X $\geq 50\%$ conserved

```
logo         MSAESGPGTRLRNLPVMGDGLETSQMSTTQAQAQPQPANAASTNPPPPETSNPNKPKRQTNQLQYLLRVVLKTLWKHQFAWPFQQPVDAVKLNLPDYYKIIKTPMDMGTI
FEDRATINIB   ................................................................................WPF...V....L.L................     6
PF00439.1    .....................................................................VLKTLWKHQFAWPFQQPVDAVKLNLPDYYKIIKTPM.....    36
PF00439.2    .....................................................................VLKTLWKHQFAWPFQQPVDAVKLNLPDYYKIIKTPMDMGTI    41
PF17105      ..............................................................................................................     0
PF00439      .....................................................................VLKTLWKHQFAWPFQQPVDAVKLNLPDYYKIIKTPMDMGTI    41
PF17035      ..............................................................................................................     0
BRD4-201     MSAESGPGTRLRNLPVMGDGLETSQMSTTQAQAQPQPANAASTNPPPPETSNPNKPKRQTNQLQYLLRVVLKTLWKHQFAWPFQQPVDAVKLNLPDYYKIIKTPMDMGTI   110
BRD4-205     MSAESGPGTRLRNLPVMGDGLETSQMSTTQAQAQPQPANAASTNPPPPETSNPNKPKRQTNQLQYLLRVVLKTLWKHQFAWPFQQPVDAVKLNLPDYYKIIKTPMDMGTI   110
BRD4-212     MSAESGPGTRLRNLPVMGDGLETSQMSTTQAQAQPQPANAASTNPPPPETSNPNKPKRQTNQLQYLLRVVLKTLWKHQFAWPFQQPVDAVKLNLPDYYKIIKTPMDMGTI   110
BRD4-208     MSAESGPGTRLRNLPVMGDGLETSQMSTTQAQAQPQPANAASTNPPPPETSNPNKPKRQTNQLQYLLRVVLKTLWKHQFAWPFQQPVDAVKLNLPDYYKIIKTPMDMGTI   110
BRD4-210     MSAESGPGTRLRNLPVMGDGLETSQMSTTQAQAQPQPANAASTNPPPPETSNPNKPKRQTNQLQYLLRVVLKTLWKHQFAWPFQQPVDAVKLNLPDYYKIIKTPM.....   105
BRD4-202     MSAESGPGTRLRNLPVMGDGLETSQMSTTQAQAQPQPANAASTNPPPPETSNPNKPKRQTNQLQYLLRVVLKTLWKHQFAWPFQQPVDAVKLNLPDYYKIIKTPMDMGTI   110
BRD4-203     MSAESGPGTRLRNLPVMGDGLETSQMSTTQAQAQPQPANAASTNPPPPETSNPNKPKRQTNQLQYLLRVVLKTLWKHQFAWPFQQPVDAVKLNLPDYYKIIKTPMDMGTI   110
```

```
logo         KKRLENNYYWNAQECIQDFNTMFTNCYIYNKPGDDIVLMAEALEKLFLQKINELPTEETEIMIVQAKGRGRGRKETGTAKPGVSTVPNTTQASTPPQTQTPQPNPPPVQA
FEDRATINIB   .............................N................................................................................     7
PF00439.1    ..............................................................................................................    36
PF00439.2    KKRLENNYYWNAQECIQDFNTMFTNCYIYNKF..............................................................................    73
PF17105      ..............................................................................................................     0
PF00439      KKRLENNYYWNAQECIQDFNTMFTNCYIYNKPGDDIVLMA......................................................................    81
PF17035      ..............................................................................................................     0
BRD4-201     KKRLENNYYWNAQECIQDFNTMFTNCYIYNKPGDDIVLMAEALEKLFLQKINELPTEETEIMIVQAKGRGRGRKETGTAKPGVSTVPNTTQASTPPQTQTPQPNPPPVQA   220
BRD4-205     KKRLENNYYWNAQECIQDFNTMFTNCYIYNKPGDDIVLMAEALEKLFLQKINELPTEETEIMIVQAKGRGRGRKETGTAKPGVSTVPNTTQASTPPQTQTPQPNPPPVQA   220
BRD4-212     KKRLENNYYWNAQECIQDFNTMFTNCYIYNKFRPQM..........................................................................   146
BRD4-208     KKRLENNYYWNAQECIQDFNTMFTNCYIYNKFRPQM..........................................................................   146
BRD4-210     ..............................................................................................................   105
BRD4-202     KKRLENNYYWNAQECIQDFNTMFTNCYIYNKPGDDIVLMAEALEKLFLQKINELPTEETEIMIVQAKGRGRGRKETGTAKPGVSTVPNTTQASTPPQTQTPQPNPPPVQA   220
BRD4-203     KKRLENNYYWNAQECIQDFNTMFTNCYIYNKPGDDIVLMAEALEKLFLQKINELPTEETEIMIVQAKGRGRGRKETGTAKPGVSTVPNTTQASTPPQTQTPQPNPPPVQA   220
```

```
logo         TPHPFPAVTPDLIVQTPVMTVVPPQPLQTPPPVPPQPQPPPAPAPQPVQSHPPIIAATPQPVKTKKGVKRKADTTTPTTIDPIHEPPSLPPEPKTTKLGQRRESSRPVKP
FEDRATINIB   ..............................................................................................................     7
PF00439.1    ..............................................................................................................    36
PF00439.2    ..............................................................................................................    73
PF17105      ..............................................................................................................     0
PF00439      ..............................................................................................................    81
PF17035      ..............................................................................................................     0
BRD4-201     TPHPFPAVTPDLIVQTPVMTVVPPQPLQTPPPVPPQPQPPPAPAPQPVQSHPPIIAATPQPVKTKKGVKRKADTTTPTTIDPIHEPPSLPPEPKTTKLGQRRESSRPVKP   330
BRD4-205     TPHPFPAVTPDLIVQTPVMTVVPPQPLQTPPPVPPQPQPPPAPAPQPVQSHPPIIAATPQPVKTKKGVKRKADTTTPTTIDPIHEPPSLPPEPKTTKLGQRRESSRPVKP   330
BRD4-212     ..............................................................................................................   146
BRD4-208     ..............................................................................................................   146
BRD4-210     ..............................................................................................................   105
BRD4-202     TPHPFPAVTPDLIVQTPVMTVVPPQPLQTPPPVPPQPQPPPAPAPQPVQSHPPIIAATPQPVKTKKGVKRKADTTTPTTIDPIHEPPSLPPEPKTTKLGQRRESSRPVKP   330
BRD4-203     TPHPFPAVTPDLIVQTPVMTVVPPQPLQTPPPVPPQPQPPPAPAPQPVQSHPPIIAATPQPVKTKKGVKRKADTTTPTTIDPIHEPPSLPPEPKTTKLGQRRESSRPVKP   330
```

```
logo
             PKKDVPDSQQHPAPEKSSKVSEQLKCCSGILKEMFAKKHAAYAWPFYKPVDVEALGLHDYCDIIKHPMDMSTIKSKLEAREYRDAQEFGADVRLMFSNCYKYNPPDHEVV
FEDRATINIB   ..............................................................................................................   7
PF00439.1    ..............................................................................................................  36
PF00439.2    ..............................................................................................................  73
PF17105      ..............................................................................................................   0
PF00439      ..........................CSGILKEMFAKKHAAYAWPFYKPVDVEALGLHDYCDIIKHPMDMSTIKSKLEAREYRDAQEFGADVRLMFSNCYKYNPPDHEVV 165
PF17035      ..............................................................................................................   0
BRD4-201     PKKDVPDSQQHPAPEKSSKVSEQLKCCSGILKEMFAKKHAAYAWPFYKPVDVEALGLHDYCDIIKHPMDMSTIKSKLEAREYRDAQEFGADVRLMFSNCYKYNPPDHEVV 440
BRD4-205     PKKDVPDSQQHPAPEKSSKVSEQLKCCSGILKEMFAKKHAAYAWPFYKPVDVEALGLHDYCDIIKHPMDMSTIKSKLEAREYRDAQEFGADVRLMFSNCYKYNPPDHEVV 440
BRD4-212     .............................................................................................................. 146
BRD4-208     .............................................................................................................. 146
BRD4-210     .............................................................................................................. 105
BRD4-202     PKKDVPDSQQHPAPEKSSKVSEQLKCCSGILKEMFAKKHAAYAWPFYKPVDVEALGLHDYCDIIKHPMDMSTIKSKLEAREYRDAQEFGADVRLMFSNCYKYNPPDHEVV 440
BRD4-203     PKKDVPDSQQHPAPEKSSKVSEQLKCCSGILKEMFAKKHAAYAWPFYKPVDVEALGLHDYCDIIKHPMDMSTIKSKLEAREYRDAQEFGADVRLMFSNCYKYNPPDHEVV 440

logo
             AMARKLQDVFEMRFAKMPDEPEEPVVAVSSPAVPPPTKVVAPPSSSDSSSDSSSDSDSSTDDSEEERAQRLAELQEQLKAVHEQLAALSQPQQNKPKKKEKDKKEKKKEK
FEDRATINIB   ..............................................................................................................   7
PF00439.1    ..............................................................................................................  36
PF00439.2    ..............................................................................................................  73
PF17105      ..............................................................................................................   0
PF00439      AMAR.......................................................................................................... 169
PF17035      ..............................................................................................................   0
BRD4-201     AMARKLQDVFEMRFAKMPDEPEEPVVAVSSPAVPPPTKVVAPPSSSDSSSDSSSDSDSSTDDSEEERAQRLAELQEQLKAVHEQLAALSQPQQNKPKKKEKDKKEKKKEK 550
BRD4-205     AMARKLQDVFEMRFAKMPDEPEEPVVAVSSPAVPPPTKVVAPPSSSDSSSDSSSDSDSSTDDSEEERAQRLAELQEQLKAVHEQLAALSQPQQNKPKKKEKDKKEKKKEK 550
BRD4-212     .............................................................................................................. 146
BRD4-208     .............................................................................................................. 146
BRD4-210     .............................................................................................................. 105
BRD4-202     AMARKLQDVFEMRFAKMPDEPEEPVVAVSSPAVPPPTKVVAPPSSSDSSSDSSSDSDSSTDDSEEERAQRLAELQEQLKAVHEQLAALSQPQQNKPKKKEKDKKEKKKEK 550
BRD4-203     AMARKLQDVFEMRFAKMPDEPEEPVVAVSSPAVPPPTKVVAPPSSSDSSSDSSSDSDSSTDDSEEERAQRLAELQEQLKAVHEQLAALSQPQQNKPKKKEKDKKEKKKEK 550

logo
             HKRKEEVEENKKSKAKEPPPKKTKKNNSSNSNVSKKEPAPMKSKPPPTYESEEEDKCKPMSYEEKRQLSLDINKLPGEKLGRVVHIIQSREPSLKNSNPDEIEIDFETLK
FEDRATINIB   ..............................................................................................................   7
PF00439.1    ..............................................................................................................  36
PF00439.2    ..............................................................................................................  73
PF17105      ..............................................................................................................   0
PF00439      .............................................................................................................. 169
PF17035      ..........................................................PMSYEEKRQLSLDINKLPGEKLGRVVHIIQSREPSLKNSNPDEIEIDFETLK  52
BRD4-201     HKRKEEVEENKKSKAKEPPPKKTKKNNSSNSNVSKKEPAPMKSKPPPTYESEEEDKCKPMSYEEKRQLSLDINKLPGEKLGRVVHIIQSREPSLKNSNPDEIEIDFETLK 660
BRD4-205     HKRKEEVEENKKSKAKEPPPKK........................................................................................ 572
BRD4-212     .............................................................................................................. 146
BRD4-208     .............................................................................................................. 146
BRD4-210     .............................................................................................................. 105
BRD4-202     HKRKEEVEENKKSKAKEPPPKKTKKNNSSNSNVSKKEPAPMKSKPPPTYESEEEDKCKPMSYEEKRQLSLDINKLPGEKLGRVVHIIQSREPSLKNSNPDEIEIDFETLK 660
BRD4-203     HKRKEEVEENKKSKAKEPPPKKTKKNNSSNSNVSKKEPAPMKSKPPPTYESEEEDKCKPMSYEEKRQLSLDINKLPGEKLGRVVHIIQSREPSLKNSNPDEIEIDFETLK 660
```

logo

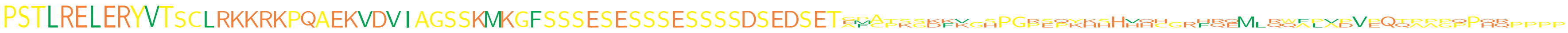

```
FEDRATINIB   ..............................................................................................................     7
PF00439.1    ..............................................................................................................    36
PF00439.2    ..............................................................................................................    73
PF17105      ..............................................................................................................     0
PF00439      ..............................................................................................................   169
PF17035      PSTLRELERYVT..................................................................................................    64
BRD4-201     PSTLRELERYVTSCLRKKRKPQAEKVDVIAGSSKMKGFSSSESESSSESSSSDSEDSETEMAPKSKKKGHPGREQKKHHHHH..HQQM.QQAPAPVPQQPPPPPQQPPPP   767
BRD4-205     ..............................................................................................................   572
BRD4-212     ..............................................................................................................   146
BRD4-208     ..............................................................................................................   146
BRD4-210     ..............................................................................................................   105
BRD4-202     PSTLRELERYVTSCLRKKRKPQAEKVDVIAGSSKMKGFSSSESESSSESSSSDSEDSETAFCTSGDFV.SPGPSPYHSHVQCGRFREMLRWFLVDVEQTAAGQPHR....   765
BRD4-203     PSTLRELERYVTSCLRKKRKPQAEKVDVIAGSSKMKGFSSSESESSSESSSSDSEDSETGPA................................................   722
```

logo

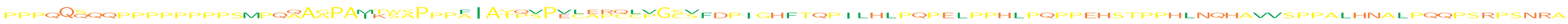

```
FEDRATINIB   ..............................................................................................................     7
PF00439.1    ..............................................................................................................    36
PF00439.2    ..............................................................................................................    73
PF17105      ..............................................................................................................     0
PF00439      ..............................................................................................................   169
PF17035      ..............................................................................................................    64
BRD4-201     PPPQQQQQPPPPPPPPPSMPQQAAPAMKSSPPPFIATQVPVLEPQLPGSVFDPIGHFTQPILHLPQPELPPHLPQPPEHSTPPHLNQHAVVSPPALHNALPQQPSRPSNRA   877
BRD4-205     ..............................................................................................................   572
BRD4-212     ..............................................................................................................   146
BRD4-208     ..............................................................................................................   146
BRD4-210     ..............................................................................................................   105
BRD4-202     ....QS..............AAGPAITWAP..AIAYPSPECARCCVGCS.............................................................   794
BRD4-203     ..............................................................................................................   722
```

logo


```
FEDRATINIB   ..............................................................................................................     7
PF00439.1    ..............................................................................................................    36
PF00439.2    ..............................................................................................................    73
PF17105      ..............................................................................................................     0
PF00439      ..............................................................................................................   169
PF17035      ..............................................................................................................    64
BRD4-201     AALPPKPARPPAVSPALTQTPLLPQPPMAQPPQVLLEDEEPPAPPLTSMQMQLYLQQLQKVQPPTPLLPSVKVQSQPPPPLPPPPHPSVQQQLQQQPPPPPPPQPQPPPQ   987
BRD4-205     ..............................................................................................................   572
BRD4-212     ..............................................................................................................   146
BRD4-208     ..............................................................................................................   146
BRD4-210     ..............................................................................................................   105
BRD4-202     ..............................................................................................................   794
BRD4-203     ..............................................................................................................   722
```

```
logo

            QQHQPPPRPVHLQPMQFSTHIQQPPPPQGQQPPHPPPGQQPPPPQPAKPQQVIQHHHSPRHHKSDPYSTGHLREAPSPLMIHSPQMSQFQSLTHQSPPQQNVQPKKQELR
FEDRATINIB  ..............................................................................................................    7
PF00439.1   ..............................................................................................................   36
PF00439.2   ..............................................................................................................   73
PF17105     ..............................................................................................................    0
PF00439     ..............................................................................................................  169
PF17035     ..............................................................................................................   64
BRD4-201    QQHQPPPRPVHLQPMQFSTHIQQPPPPQGQQPPHPPPGQQPPPPQPAKPQQVIQHHHSPRHHKSDPYSTGHLREAPSPLMIHSPQMSQFQSLTHQSPPQQNVQPKKQELR 1097
BRD4-205    ..............................................................................................................  572
BRD4-212    ..............................................................................................................  146
BRD4-208    ..............................................................................................................  146
BRD4-210    ..............................................................................................................  105
BRD4-202    ..............................................................................................................  794
BRD4-203    ..............................................................................................................  722
```

```
logo

            AASVVQPQPLVVVKEEKIHSPIIRSEPFSPSLRPEPPKHPESIKAPVHLPQRPEMKPVDVGRPVIRPPEQNAPPPGAPDKDKQKQEPKTPVAPKKDLKIKNMGSWASLVQ
FEDRATINIB  ..............................................................................................................    7
PF00439.1   ..............................................................................................................   36
PF00439.2   ..............................................................................................................   73
PF17105     ..............................................................................................................    0
PF00439     ..............................................................................................................  169
PF17035     ..............................................................................................................   64
BRD4-201    AASVVQPQPLVVVKEEKIHSPIIRSEPFSPSLRPEPPKHPESIKAPVHLPQRPEMKPVDVGRPVIRPPEQNAPPPGAPDKDKQKQEPKTPVAPKKDLKIKNMGSWASLVQ 1207
BRD4-205    ..............................................................................................................  572
BRD4-212    ..............................................................................................................  146
BRD4-208    ..............................................................................................................  146
BRD4-210    ..............................................................................................................  105
BRD4-202    ..............................................................................................................  794
BRD4-203    ..............................................................................................................  722
```

```
logo

            KHPTTPSSTAKSSSDSFEQFRRAAREKEEREKALKAQAEHAEKEKERLRQERMRSREDEDALEQARRAHEEARRRQEQQQQQRQEQQQQQQQQAAAVAAAATPQAQSSQP
FEDRATINIB  ..............................................................................................................    7
PF00439.1   ..............................................................................................................   36
PF00439.2   ..............................................................................................................   73
PF17105     ..............................................................................................................    0
PF00439     ..............................................................................................................  169
PF17035     ..............................................................................................................   64
BRD4-201    KHPTTPSSTAKSSSDSFEQFRRAAREKEEREKALKAQAEHAEKEKERLRQERMRSREDEDALEQARRAHEEARRRQEQQQQQRQEQQQQQQQQAAAVAAAATPQAQSSQP 1317
BRD4-205    ..............................................................................................................  572
BRD4-212    ..............................................................................................................  146
BRD4-208    ..............................................................................................................  146
BRD4-210    ..............................................................................................................  105
BRD4-202    ..............................................................................................................  794
BRD4-203    ..............................................................................................................  722
```

logo

```
             QSMLDQQRELARKREQERRRREAMAATIDMNFQSDLLSIFEENLF
FEDRATINIB   .............................................     7
PF00439.1    .............................................    36
PF00439.2    .............................................    73
PF17105      .SMLDQQRELARKREQERRRREAMAATIDMNFQSDLLSIFEENLF    44
PF00439      .............................................   169
PF17035      .............................................    64
BRD4-201     QSMLDQQRELARKREQERRRREAMAATIDMNFQSDLLSIFEENLF  1362
BRD4-205     .............................................   572
BRD4-212     .............................................   146
BRD4-208     .............................................   146
BRD4-210     .............................................   105
BRD4-202     .............................................   794
BRD4-203     .............................................   722
```

X non conserved

X similar

X $\geq$ 0% conserved

X $\geq$ 50% conserved

```
logo         MSAESGPGTRLRNLPVMGDGLETSQMSTTQAQAQPQPANAASTNPPPPETSNPNKPKRQTNQLQYLLRVVLKTLWKHQFAWPFQQPVDAVKLNLPDYYKIIKTPMDMGTI
LY-294002    ................................................................................WPF....V....L.L...............      6
PF00439.1    .....................................................................VLKTLWKHQFAWPFQQPVDAVKLNLPDYYKIIKTPM.....     36
PF00439.2    .....................................................................VLKTLWKHQFAWPFQQPVDAVKLNLPDYYKIIKTPMDMGTI     41
PF17105      ..............................................................................................................      0
PF00439      .....................................................................VLKTLWKHQFAWPFQQPVDAVKLNLPDYYKIIKTPMDMGTI     41
PF17035      ..............................................................................................................      0
BRD4-201     MSAESGPGTRLRNLPVMGDGLETSQMSTTQAQAQPQPANAASTNPPPPETSNPNKPKRQTNQLQYLLRVVLKTLWKHQFAWPFQQPVDAVKLNLPDYYKIIKTPMDMGTI    110
BRD4-205     MSAESGPGTRLRNLPVMGDGLETSQMSTTQAQAQPQPANAASTNPPPPETSNPNKPKRQTNQLQYLLRVVLKTLWKHQFAWPFQQPVDAVKLNLPDYYKIIKTPMDMGTI    110
BRD4-212     MSAESGPGTRLRNLPVMGDGLETSQMSTTQAQAQPQPANAASTNPPPPETSNPNKPKRQTNQLQYLLRVVLKTLWKHQFAWPFQQPVDAVKLNLPDYYKIIKTPMDMGTI    110
BRD4-208     MSAESGPGTRLRNLPVMGDGLETSQMSTTQAQAQPQPANAASTNPPPPETSNPNKPKRQTNQLQYLLRVVLKTLWKHQFAWPFQQPVDAVKLNLPDYYKIIKTPMDMGTI    110
BRD4-210     MSAESGPGTRLRNLPVMGDGLETSQMSTTQAQAQPQPANAASTNPPPPETSNPNKPKRQTNQLQYLLRVVLKTLWKHQFAWPFQQPVDAVKLNLPDYYKIIKTPM.....    105
BRD4-202     MSAESGPGTRLRNLPVMGDGLETSQMSTTQAQAQPQPANAASTNPPPPETSNPNKPKRQTNQLQYLLRVVLKTLWKHQFAWPFQQPVDAVKLNLPDYYKIIKTPMDMGTI    110
BRD4-203     MSAESGPGTRLRNLPVMGDGLETSQMSTTQAQAQPQPANAASTNPPPPETSNPNKPKRQTNQLQYLLRVVLKTLWKHQFAWPFQQPVDAVKLNLPDYYKIIKTPMDMGTI    110

logo         KKRLENNYYWNAQECIQDFNTMFTNCYIYNKPGDDIVLMAEALEKLFLQKINELPTEETEIMIVQAKGRGRGRKETGTAKPGVSTVPNTTQASTPPQTQTPQPNPPPVQA
                                            FRPQM
LY-294002    .............................N.....I..........................................................................      8
PF00439.1    ..............................................................................................................     36
PF00439.2    KKRLENNYYWNAQECIQDFNTMFTNCYIYNKF..............................................................................     73
PF17105      ..............................................................................................................      0
PF00439      KKRLENNYYWNAQECIQDFNTMFTNCYIYNKPGDDIVLMA......................................................................     81
PF17035      ..............................................................................................................      0
BRD4-201     KKRLENNYYWNAQECIQDFNTMFTNCYIYNKPGDDIVLMAEALEKLFLQKINELPTEETEIMIVQAKGRGRGRKETGTAKPGVSTVPNTTQASTPPQTQTPQPNPPPVQA    220
BRD4-205     KKRLENNYYWNAQECIQDFNTMFTNCYIYNKPGDDIVLMAEALEKLFLQKINELPTEETEIMIVQAKGRGRGRKETGTAKPGVSTVPNTTQASTPPQTQTPQPNPPPVQA    220
BRD4-212     KKRLENNYYWNAQECIQDFNTMFTNCYIYNKFRPQM..........................................................................    146
BRD4-208     KKRLENNYYWNAQECIQDFNTMFTNCYIYNKFRPQM..........................................................................    146
BRD4-210     ..............................................................................................................    105
BRD4-202     KKRLENNYYWNAQECIQDFNTMFTNCYIYNKPGDDIVLMAEALEKLFLQKINELPTEETEIMIVQAKGRGRGRKETGTAKPGVSTVPNTTQASTPPQTQTPQPNPPPVQA    220
BRD4-203     KKRLENNYYWNAQECIQDFNTMFTNCYIYNKPGDDIVLMAEALEKLFLQKINELPTEETEIMIVQAKGRGRGRKETGTAKPGVSTVPNTTQASTPPQTQTPQPNPPPVQA    220

logo         TPHPFPAVTPDLIVQTPVMTVVPPQPLQTPPPVPPQPQPPPAPAPQPVQSHPPIIAATPQPVKTKKGVKRKADTTTPTTIDPIHEPPSLPPEPKTTKLGQRRESSRPVKP
LY-294002    ..............................................................................................................      8
PF00439.1    ..............................................................................................................     36
PF00439.2    ..............................................................................................................     73
PF17105      ..............................................................................................................      0
PF00439      ..............................................................................................................     81
PF17035      ..............................................................................................................      0
BRD4-201     TPHPFPAVTPDLIVQTPVMTVVPPQPLQTPPPVPPQPQPPPAPAPQPVQSHPPIIAATPQPVKTKKGVKRKADTTTPTTIDPIHEPPSLPPEPKTTKLGQRRESSRPVKP    330
BRD4-205     TPHPFPAVTPDLIVQTPVMTVVPPQPLQTPPPVPPQPQPPPAPAPQPVQSHPPIIAATPQPVKTKKGVKRKADTTTPTTIDPIHEPPSLPPEPKTTKLGQRRESSRPVKP    330
BRD4-212     ..............................................................................................................    146
BRD4-208     ..............................................................................................................    146
BRD4-210     ..............................................................................................................    105
BRD4-202     TPHPFPAVTPDLIVQTPVMTVVPPQPLQTPPPVPPQPQPPPAPAPQPVQSHPPIIAATPQPVKTKKGVKRKADTTTPTTIDPIHEPPSLPPEPKTTKLGQRRESSRPVKP    330
BRD4-203     TPHPFPAVTPDLIVQTPVMTVVPPQPLQTPPPVPPQPQPPPAPAPQPVQSHPPIIAATPQPVKTKKGVKRKADTTTPTTIDPIHEPPSLPPEPKTTKLGQRRESSRPVKP    330
```

```
logo
           PKKDVPDSQQHPAPEKSSKVSEQLKCCSGILKEMFAKKHAAYAWPFYKPVDVEALGLHDYCDIIKHPMDMSTIKSKLEAREYRDAQEFGADVRLMFSNCYKYNPPDHEVV
LY-294002  ..............................................................................................................   8
PF00439.1  ..............................................................................................................  36
PF00439.2  ..............................................................................................................  73
PF17105    ..............................................................................................................   0
PF00439    ..........................CSGILKEMFAKKHAAYAWPFYKPVDVEALGLHDYCDIIKHPMDMSTIKSKLEAREYRDAQEFGADVRLMFSNCYKYNPPDHEVV 165
PF17035    ..............................................................................................................   0
BRD4-201   PKKDVPDSQQHPAPEKSSKVSEQLKCCSGILKEMFAKKHAAYAWPFYKPVDVEALGLHDYCDIIKHPMDMSTIKSKLEAREYRDAQEFGADVRLMFSNCYKYNPPDHEVV 440
BRD4-205   PKKDVPDSQQHPAPEKSSKVSEQLKCCSGILKEMFAKKHAAYAWPFYKPVDVEALGLHDYCDIIKHPMDMSTIKSKLEAREYRDAQEFGADVRLMFSNCYKYNPPDHEVV 440
BRD4-212   .............................................................................................................. 146
BRD4-208   .............................................................................................................. 146
BRD4-210   .............................................................................................................. 105
BRD4-202   PKKDVPDSQQHPAPEKSSKVSEQLKCCSGILKEMFAKKHAAYAWPFYKPVDVEALGLHDYCDIIKHPMDMSTIKSKLEAREYRDAQEFGADVRLMFSNCYKYNPPDHEVV 440
BRD4-203   PKKDVPDSQQHPAPEKSSKVSEQLKCCSGILKEMFAKKHAAYAWPFYKPVDVEALGLHDYCDIIKHPMDMSTIKSKLEAREYRDAQEFGADVRLMFSNCYKYNPPDHEVV 440

logo
           AMARKLQDVFEMRFAKMPDEPEEPVVAVSSPAVPPPTKVVAPPSSSDSSSDSSSDSDSSTDDSEEERAQRLAELQEQLKAVHEQLAALSQPQQNKPKKKEKDKKEKKKEK
LY-294002  ..............................................................................................................   8
PF00439.1  ..............................................................................................................  36
PF00439.2  ..............................................................................................................  73
PF17105    ..............................................................................................................   0
PF00439    AMAR.......................................................................................................... 169
PF17035    ..............................................................................................................   0
BRD4-201   AMARKLQDVFEMRFAKMPDEPEEPVVAVSSPAVPPPTKVVAPPSSSDSSSDSSSDSDSSTDDSEEERAQRLAELQEQLKAVHEQLAALSQPQQNKPKKKEKDKKEKKKEK 550
BRD4-205   AMARKLQDVFEMRFAKMPDEPEEPVVAVSSPAVPPPTKVVAPPSSSDSSSDSSSDSDSSTDDSEEERAQRLAELQEQLKAVHEQLAALSQPQQNKPKKKEKDKKEKKKEK 550
BRD4-212   .............................................................................................................. 146
BRD4-208   .............................................................................................................. 146
BRD4-210   .............................................................................................................. 105
BRD4-202   AMARKLQDVFEMRFAKMPDEPEEPVVAVSSPAVPPPTKVVAPPSSSDSSSDSSSDSDSSTDDSEEERAQRLAELQEQLKAVHEQLAALSQPQQNKPKKKEKDKKEKKKEK 550
BRD4-203   AMARKLQDVFEMRFAKMPDEPEEPVVAVSSPAVPPPTKVVAPPSSSDSSSDSSSDSDSSTDDSEEERAQRLAELQEQLKAVHEQLAALSQPQQNKPKKKEKDKKEKKKEK 550

logo
           HKRKEEVEENKKSKAKEPPPKKTKKNNSSNSNVSKKEPAPMKSKPPPTYESEEEDKCKPMSYEEKRQLSLDINKLPGEKLGRVVHIIQSREPSLKNSNPDEIEIDFETLK
LY-294002  ..............................................................................................................   8
PF00439.1  ..............................................................................................................  36
PF00439.2  ..............................................................................................................  73
PF17105    ..............................................................................................................   0
PF00439    .............................................................................................................. 169
PF17035    ..........................................................PMSYEEKRQLSLDINKLPGEKLGRVVHIIQSREPSLKNSNPDEIEIDFETLK  52
BRD4-201   HKRKEEVEENKKSKAKEPPPKKTKKNNSSNSNVSKKEPAPMKSKPPPTYESEEEDKCKPMSYEEKRQLSLDINKLPGEKLGRVVHIIQSREPSLKNSNPDEIEIDFETLK 660
BRD4-205   HKRKEEVEENKKSKAKEPPPKK........................................................................................ 572
BRD4-212   .............................................................................................................. 146
BRD4-208   .............................................................................................................. 146
BRD4-210   .............................................................................................................. 105
BRD4-202   HKRKEEVEENKKSKAKEPPPKKTKKNNSSNSNVSKKEPAPMKSKPPPTYESEEEDKCKPMSYEEKRQLSLDINKLPGEKLGRVVHIIQSREPSLKNSNPDEIEIDFETLK 660
BRD4-203   HKRKEEVEENKKSKAKEPPPKKTKKNNSSNSNVSKKEPAPMKSKPPPTYESEEEDKCKPMSYEEKRQLSLDINKLPGEKLGRVVHIIQSREPSLKNSNPDEIEIDFETLK 660
```

logo

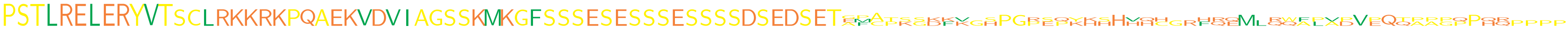

```
LY-294002    ..............................................................................................................    8
PF00439.1    ..............................................................................................................   36
PF00439.2    ..............................................................................................................   73
PF17105      ..............................................................................................................    0
PF00439      ..............................................................................................................  169
PF17035      PSTLRELERYVT..................................................................................................   64
BRD4-201     PSTLRELERYVTSCLRKKRKPQAEKVDVIAGSSKMKGFSSSESESSSESSSSDSEDSETEMAPKSKKKGHPGREQKKHHHHH..HQQM.QQAPAPVPQQPPPPPQQPPPP  767
BRD4-205     ..............................................................................................................  572
BRD4-212     ..............................................................................................................  146
BRD4-208     ..............................................................................................................  146
BRD4-210     ..............................................................................................................  105
BRD4-202     PSTLRELERYVTSCLRKKRKPQAEKVDVIAGSSKMKGFSSSESESSSESSSSDSEDSETAFCTSGDFV.SPGPSPYHSHVQCGRFREMLRWFLVDVEQTAAGQPHR....  765
BRD4-203     PSTLRELERYVTSCLRKKRKPQAEKVDVIAGSSKMKGFSSSESESSSESSSSDSEDSETGPA................................................  722
```

logo

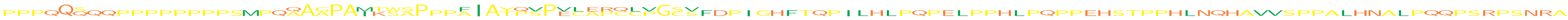

```
LY-294002    ..............................................................................................................    8
PF00439.1    ..............................................................................................................   36
PF00439.2    ..............................................................................................................   73
PF17105      ..............................................................................................................    0
PF00439      ..............................................................................................................  169
PF17035      ..............................................................................................................   64
BRD4-201     PPPQQQQQPPPPPPPPSMPQQAAPAMKSSPPPFIATQVPVLEPQLPGSVFDPIGHFTQPILHLPQPELPPHLPQPPEHSTPPHLNQHAVVSPPALHNALPQQPSRPSNRA  877
BRD4-205     ..............................................................................................................  572
BRD4-212     ..............................................................................................................  146
BRD4-208     ..............................................................................................................  146
BRD4-210     ..............................................................................................................  105
BRD4-202     ....QS..............AAGPAITWAP..AIAYPSPECARCCVGCS.............................................................  794
BRD4-203     ..............................................................................................................  722
```

logo


```
LY-294002    ..............................................................................................................    8
PF00439.1    ..............................................................................................................   36
PF00439.2    ..............................................................................................................   73
PF17105      ..............................................................................................................    0
PF00439      ..............................................................................................................  169
PF17035      ..............................................................................................................   64
BRD4-201     AALPPKPARPPAVSPALTQTPLLPQPPMAQPPQVLLEDEEPPAPPLTSMQMQLYLQQLQKVQPPTPLLPSVKVQSQPPPPLPPPPHPSVQQQLQQQPPPPPPPQPQPPPQ  987
BRD4-205     ..............................................................................................................  572
BRD4-212     ..............................................................................................................  146
BRD4-208     ..............................................................................................................  146
BRD4-210     ..............................................................................................................  105
BRD4-202     ..............................................................................................................  794
BRD4-203     ..............................................................................................................  722
```

```
logo

              QQHQPPPRPVHLQPMQFSTHIQQPPPPQGQQPPHPPPGQQPPPPQPAKPQQVIQHHHSPRHHKSDPYSTGHLREAPSPLMIHSPQMSQFQSLTHQSPPQQNVQPKKQELR
LY-294002     ..............................................................................................................    8
PF00439.1     ..............................................................................................................   36
PF00439.2     ..............................................................................................................   73
PF17105       ..............................................................................................................    0
PF00439       ..............................................................................................................  169
PF17035       ..............................................................................................................   64
BRD4-201      QQHQPPPRPVHLQPMQFSTHIQQPPPPQGQQPPHPPPGQQPPPPQPAKPQQVIQHHHSPRHHKSDPYSTGHLREAPSPLMIHSPQMSQFQSLTHQSPPQQNVQPKKQELR 1097
BRD4-205      ..............................................................................................................  572
BRD4-212      ..............................................................................................................  146
BRD4-208      ..............................................................................................................  146
BRD4-210      ..............................................................................................................  105
BRD4-202      ..............................................................................................................  794
BRD4-203      ..............................................................................................................  722

logo

              AASVVQPQPLVVVKEEKIHSPIIRSEPFSPSLRPEPPKHPESIKAPVHLPQRPEMKPVDVGRPVIRPPEQNAPPPGAPDKDKQKQEPKTPVAPKKDLKIKNMGSWASLVQ
LY-294002     ..............................................................................................................    8
PF00439.1     ..............................................................................................................   36
PF00439.2     ..............................................................................................................   73
PF17105       ..............................................................................................................    0
PF00439       ..............................................................................................................  169
PF17035       ..............................................................................................................   64
BRD4-201      AASVVQPQPLVVVKEEKIHSPIIRSEPFSPSLRPEPPKHPESIKAPVHLPQRPEMKPVDVGRPVIRPPEQNAPPPGAPDKDKQKQEPKTPVAPKKDLKIKNMGSWASLVQ 1207
BRD4-205      ..............................................................................................................  572
BRD4-212      ..............................................................................................................  146
BRD4-208      ..............................................................................................................  146
BRD4-210      ..............................................................................................................  105
BRD4-202      ..............................................................................................................  794
BRD4-203      ..............................................................................................................  722

logo

              KHPTTPSSTAKSSSDSFEQFRRAAREKEEREKALKAQAEHAEKEKERLRQERMRSREDEDALEQARRAHEEARRRQEQQQQQRQEQQQQQQQQAAAVAAAATPQAQSSQP
LY-294002     ..............................................................................................................    8
PF00439.1     ..............................................................................................................   36
PF00439.2     ..............................................................................................................   73
PF17105       ..............................................................................................................    0
PF00439       ..............................................................................................................  169
PF17035       ..............................................................................................................   64
BRD4-201      KHPTTPSSTAKSSSDSFEQFRRAAREKEEREKALKAQAEHAEKEKERLRQERMRSREDEDALEQARRAHEEARRRQEQQQQQRQEQQQQQQQQAAAVAAAATPQAQSSQP 1317
BRD4-205      ..............................................................................................................  572
BRD4-212      ..............................................................................................................  146
BRD4-208      ..............................................................................................................  146
BRD4-210      ..............................................................................................................  105
BRD4-202      ..............................................................................................................  794
BRD4-203      ..............................................................................................................  722
```

logo

```
             QSMLDQQRELARKREQERRRREAMAATIDMNFQSDLLSIFEENLF
LY-294002    .............................................     8
PF00439.1    .............................................    36
PF00439.2    .............................................    73
PF17105      .SMLDQQRELARKREQERRRREAMAATIDMNFQSDLLSIFEENLF    44
PF00439      .............................................   169
PF17035      .............................................    64
BRD4-201     QSMLDQQRELARKREQERRRREAMAATIDMNFQSDLLSIFEENLF  1362
BRD4-205     .............................................   572
BRD4-212     .............................................   146
BRD4-208     .............................................   146
BRD4-210     .............................................   105
BRD4-202     .............................................   794
BRD4-203     .............................................   722
```

X non conserved

X similar

X $\geq$ 0% conserved

X $\geq$ 50% conserved

```
logo
             MASWSIQQMVIGCPLCGRHCSGGEHTGELQKEEAMAAVILESIFLKRSQQKKKTSPLNFKKRLFLLTVHKLSYYEYDFERGRRGSKKGSIDVEKITCVETVVPEKNPPPE
IBRUTINIB    ..............................................................................................................     0
PF00017.1    ..............................................................................................................     0
PF07714      ..............................................................................................................     0
PF00779      ..............................................................................................................     0
PF00169      ......................................ILESIFLKRSQQKKKTSPLNFKKRLFLLTVHKLSYYEYDFERGRRGSKKGSIDVEKITCVETVVPEKNPPPE    72
PF00017      ..............................................................................................................     0
PF00018      ..............................................................................................................     0
PF07714.2    ..............................................................................................................     0
PF07714.1    ..............................................................................................................     0
BTK-210      MASWSIQQMVIGCPLCGRHCSGGEHTGELQKEEAMAAVILESIFLKRSQQKKKTSPLNFKKRLFLLTVHKLSYYEYDFERGRRGSKKGSIDVEKITCVETVVPEKNPPPE   110
BTK-202      ..................................MAAVILESIFLKRSQQKKKTSPLNFKKRLFLLTVHKLSYYEYDFERGRRGSKKGSIDVEKITCVETVVPEKNPPPE    76
BTK-201      ..................................MAAVILESIFLKRSQQKKKTSPLNFKKRLFLLTVHKLSYYEYDFERGRRGSKKGSIDVEKITCVETVVPEKNPPPE    76
BTK-209      ..................................MAAVILESIFLKRSQQKKKTSPLNFKKRLFLLTVHKLSYYEYDFERGRRGSKKGSIDVEKITCVETVVPEKNPPPE    76

logo
             RQIPRRGEESSEMEQISIIERFPYPFQVVYDEGPLYVFSPTEELRKRWIHQLKNVIRYNSDLVQKYHPCFWIDGQYLCCSQTAKNAMGCQILENRNGSLKPGSSHRKTKK
IBRUTINIB    ..............................................................................................................     0
PF00017.1    ..............................................................................................................     0
PF07714      ..............................................................................................................     0
PF00779      ................................................................KYHPCFWIDGQYLCCSQTAKNAMGCQI...................    27
PF00169      RQIPRRGEESSEMEQISIIERFPYPFQVVYDEGPLYVFSPTEELRKRWIHQLKNVIR.....................................................   129
PF00017      ..............................................................................................................     0
PF00018      ..............................................................................................................     0
PF07714.2    ..............................................................................................................     0
PF07714.1    ..............................................................................................................     0
BTK-210      RQIPRRGEESSEMEQISIIERFPYPFQVVYDEGPLYVFSPTEELRKRWIHQLKNVIRYNSDLVQKYHPCFWIDGQYLCCSQTAKNAMGCQILENRNGSLKPGSSHRKTKK   220
BTK-202      RQIPRRGEESSEMEQISIIERFPYPFQVVYDEGPLYVFSPTEELRKRWIHQLKNVIRYNSDLVQKYHPCFWIDGQYLCCSQTAKNAMGCQILENRNGSLKPGSSHRKTKK   186
BTK-201      RQIPRRGEESSEMEQISIIERFPYPFQVVYDEGPLYVFSPTEELRKRWIHQLKNVIRYNSDLVQKYHPCFWIDGQYLCCSQTAKNAMGCQILENRNGSLKPGSSHRKTKK   186
BTK-209      RQIPRRGEESSEMEQISIIERFPYPFQVVYDEGPLYVFSPTEELRKRWIHQLKNVIRYNSDLVQKYHPCFWIDGQYLCCSQTAKNAMGCQILENRNGSLKPGSSHRKTKK   186

logo
             PLPPTPEEDQILKKPLPPEPAAAPVSTSELKKVVALYDYMPMNANDLQLRKGDEYFILEESNLPWWRARDKNGQEGYIPSNYVTEAEDSIEMYEWYSKHMTRSQAEQLLK
IBRUTINIB    ..............................................................................................................     0
PF00017.1    ..............................................................................................WYSKHMTRSQAEQLLK    16
PF07714      ..............................................................................................................     0
PF00779      ..............................................................................................................    27
PF00169      ..............................................................................................................   129
PF00017      ..............................................................................................WYSKHMTRSQAEQLLK    16
PF00018      .................................VALYDYMPMNANDLQLRKGDEYFILEESNLPWWRARDKNGQEGYIPS..............................    47
PF07714.2    ..............................................................................................................     0
PF07714.1    ..............................................................................................................     0
BTK-210      PLPPTPEEDQILKKPLPPEPAAAPVSTSELKKVVALYDYMPMNANDLQLRKGDEYFILEESNLPWWRARDKNGQEGYIPSNYVTEAEDSIEMYEWYSKHMTRSQAEQLLK   330
BTK-202      PLPPTPEEDQILKKPLPPEPAAAPVSTSELKKVVALYDYMPMNANDLQLRKGDEYFILEESNLPWWRARDKNGQEGYIPSNYVTEAEDSIEMYEWYSKHMTRSQAEQLLK   296
BTK-201      PLPPTPEEDQILKKPLPPEPAAAPVSTSELKKVVALYDYMPMNANDLQLRKGDEYFILEESNLPWWRARDKNGQEGYIPSNYVTEAEDSIEMYEWYSKHMTRSQAEQLLK   296
BTK-209      PLPPTPEEDQILKKPLPPEPAAAPVSTSELKKVVALYDYMPMNANDLQLRKGDEYFILEESNLPWWRARDKNGQEGYIPSNYVTEAEDSIEMYEWYSKHMTRSQAEQLLK   296
```

```
logo         QEGKEGGFIVRDSSKAGKYTVSVFAKSTGDPQGVIRHYVVCSTPQSQYYLAEKHLFSTIPELINYHQHNSAGLISRLKYPVSQQNKNAPSTAGLGYGSWEIDPKDLTFLK
IBRUTINIB    ..............................................................................................................     0
PF00017.1    QEGKEGGFIVRDSSKAGKYTVSVFAKSTGDPQGVIRHYVVCSTPQSQYYLA............................................................    67
PF07714      .........................................................................................................LTFLK     5
PF00779      ..............................................................................................................    27
PF00169      ..............................................................................................................   129
PF00017      QEGKEGGFIVRDSSKAGKYTVSVFAKSTGDPQGVIRHYVVCSTPQSQYYLAEKHLFSTIPELINYH............................................    82
PF00018      ..............................................................................................................    47
PF07714.2    .........................................................................................................LTFLK     5
PF07714.1    ..............................................................................................................     0
BTK-210      QEGKEGGFIVRDSSKAGKYTVSVFAKSTGDPQGVIRHYVVCSTPQSQYYLAEKHLFSTIPELINYHQHNSAGLISRLKYPVSQQNKNAPSTAGLGYGSWEIDPKDLTFLK   440
BTK-202      QEGKEGGFIVRDSSKAGKYTVSVFAKSTGDPQGVIRHYVVCSTPQSQYYLA............................................................   347
BTK-201      QEGKEGGFIVRDSSKAGKYTVSVFAKSTGDPQGVIRHYVVCSTPQSQYYLAEKHLFSTIPELINYHQHNSAGLISRLKYPVSQQNKNAPSTAGLGYGSWEIDPKDLTFLK   406
BTK-209      QEGKEGGFIVRDSSKAGKYTVSVFAKSTGDPQGVIRHYVVCSTPQSQYYLAEKHLFSTIPELINYHQHNSAGLISRLKYPVSQQNKNAPSTAGLGYGSWEIDPKDLTFLK   406

logo         ELGTGQFGVVKYGKWRGQYDVAIKMIKEGSMSEDEFIEEAKVMMNLSHEKLVQLYGVCTKQRPIFIITEYMANGCLLNYLREMRHRFQTQQLLEMCKDVCEAMEYLESKQ
IBRUTINIB    .L.......V...........A.K..................M........................TEYM..GC..N................................    12
PF00017.1    ..............................................................................................................    67
PF07714      ELGTGQFGVVKYGKWRGQYDVAIKMIKEGSMSEDEFIEEAKVMMNLSHEKLVQLYGVCTKQRPIFIITEYMANGCLLNYLREMRHRFQTQQLLEMCKDVCEAMEYLESKQ   115
PF00779      ..............................................................................................................    27
PF00169      ..............................................................................................................   129
PF00017      ..............................................................................................................    82
PF00018      ..............................................................................................................    47
PF07714.2    ELGTGQFGVVKYGKWRGQYDVAIKMIKEGSMSEDEFIEEAKVMMNLSHEKLVQLYGVCTKQRPIFIITEYMANGCLLNYLREMRHRFQTQQLLEMCKDVCEAMEYLESKQ   115
PF07714.1    ..............................................................................................................     0
BTK-210      ELGTGQFGVVKYGKWRGQYDVAIKMIKEGSMSEDEFIEEAKVMMNLSHEKLVQLYGVCTKQRPIFIITEYMANGCLLNYLREMRHRFQTQQLLEMCKDVCEAMEYLESKQ   550
BTK-202      ..............................................................................................................   347
BTK-201      ELGTGQFGVVKYGKWRGQYDVAIKMIKEGSMSEDEFIEEAKVMMNLSHEKLVQLYGVCTKQRPIFIITEYMANGCLLNYLREMRHRFQTQQLLEMCKDVCEAMEYLESKQ   516
BTK-209      ELGTGQFGVVKYGKWRGQYDVAIKMIKEGSMSEDEFIEEAKVMMNLSHEKLVQLYGVCTKQRPIFIITEYMANGCLLNYLREMRHRFQTQQLLEMCKDVCEAMEYLESKQ   516

logo         FLHRDLAARNCLVNDQGVVKVSDFGLSRYVLDDEYTSSVGSKFPVRWSPPEVLMYSKFSSKSDIWAFGVLMWEIYSLGKMPYERFTNSETAEHIAQGLRLYRPHLASEKV
IBRUTINIB    ...........L.........SDF......................................................................................    16
PF00017.1    .......ARN....................................................................................................    70
PF07714      FLHRDLAARNCLVNDQGVVKVSDFGLSRYVLDDEYTSSVGSKFPVRWSPPEVLMYSKFSSKSDIWAFGVLMWEIYSLGKMPYERFTNSETAEHIAQGLRLYRPHLASEKV   225
PF00779      ..............................................................................................................    27
PF00169      ..............................................................................................................   129
PF00017      ..............................................................................................................    82
PF00018      ..............................................................................................................    47
PF07714.2    FLHRDLAARNCLVNDQGVVKVSDFGLSRYVLDDEYTSSVGSKFPVRWSPPES...........................................................   167
PF07714.1    .....LAARNCLVNDQGVVKVSDFGLSRYVLDDEYTSSVGSKFPVRWSPPEVLMYSKFSSKSDIWAFGVLMWEIYSLGKMPYERFTNSETAEHIAQGLRLYRPHLASEKV   105
BTK-210      FLHRDLAARNCLVNDQGVVKVSDFGLSRYVLDDEYTSSVGSKFPVRWSPPEVLMYSKFSSKSDIWAFGVLMWEIYSLGKMPYERFTNSETAEHIAQGLRLYRPHLASEKV   660
BTK-202      .......ARNCLVNDQGVVKVSDFGLSRYVLDDEYTSSVGSKFPVRWSPPEVLMYSKFSSKSDIWAFGVLMWEIYSLGKMPYERFTNSETAEHIAQGLRLYRPHLASEKV   450
BTK-201      FLHRDLAARNCLVNDQGVVKVSDFGLSRYVLDDEYTSSVGSKFPVRWSPPEVLMYSKFSSKSDIWAFGVLMWEIYSLGKMPYERFTNSETAEHIAQGLRLYRPHLASEKV   626
BTK-209      FLHRDLAARNCLVNDQGVVKVSDFGLSRYVLDDEYTSSVGSKFPVRWSPPES...........................................................   568
```

```
logo
             YTIMYSCWHEKADERPTFKILLSNILDVMDEES
IBRUTINIB    .................................   16
PF00017.1    .................................   70
PF07714      YTIMYSCWHEKADERPTFKILLSN.........  249
PF00779      .................................   27
PF00169      .................................  129
PF00017      .................................   82
PF00018      .................................   47
PF07714.2    .................................  167
PF07714.1    YTIMYSCWHEKADERPTFKILLSN.........  129
BTK-210      YTIMYSCWHEKADERPTFKILLSNILDVMDEES  693
BTK-202      YTIMYSCWHEKADERPTFKILLSNILDVMDEES  483
BTK-201      YTIMYSCWHEKADERPTFKILLSNILDVMDEES  659
BTK-209      .................................  568
```

- X non conserved
- X similar
- X $\geq 0\%$ conserved
- X $\geq 50\%$ conserved

logo

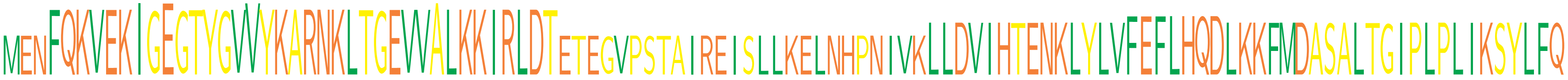

```
BMS-387032   .........I.E.....V............A................................................FEFLHQD........................   11
PF00069.2    ...FQKVEKIGEGTYGVVYKARNKLTGEVVALKKIRLDT..........................LLDVIHTENKLYLVFEFLHQDLKKFMDASALTGIPLPLIKSYLFQ   81
PF00069.3    ...FQKVEKIGEGTYGVVYKARNKLTGEVVALKKIRLDTETEGVPSTAIREISLLKELNHPNIVKLLDVIHTENKLYLVFEFLHQDLKKFMDASALTGIPLPLIKSYLFQ  107
PF00069.1    ...FQKVEKIGEGTYGVVYKARNKLTGEVVALKKIRLDTETEGVPSTAIREISLLKELNHPNIVKLLDVIHTENKLYLVFEFLHQDLKKFMDASALTGIPLPLIKSYLFQ  107
PF00069      ...FQKVEKIGEGTYGVVYKARNKLTGEVVALKKIRLDTETEGVPSTAIREISLLKELNHPNIVKLLDVIHTENKLYLVFEFLHQDLKKFMDASALTGIPLPLIKSYLFQ  107
CDK2-208     MENFQKVEKIGEGTYGVVYKARNKLTGEVVALKKIRLDT.......................................................................   39
CDK2-203     MENFQKVEKIGEGTYGVVYKARNKLTGEVVALKKIRLDT..........................LLDVIHTENKLYLVFEFLHQDLKKFMDASALTGIPLPLIKSYLFQ   84
CDK2-204     MENFQKVEKIGEGTYGVVYKARNKLTGEVVALKKIRLDTETEGVPSTAIREISLLKELNHPNIVKLLDVIHTENKLYLVFEFLHQDLKKFMDASALTGIPLPLIKSYLFQ  110
CDK2-201     MENFQKVEKIGEGTYGVVYKARNKLTGEVVALKKIRLDTETEGVPSTAIREISLLKELNHPNIVKLLDVIHTENKLYLVFEFLHQDLKKFMDASALTGIPLPLIKSYLFQ  110
CDK2-202     MENFQKVEKIGEGTYGVVYKARNKLTGEVVALKKIRLDTETEGVPSTAIREISLLKELNHPNIVKLLDVIHTENKLYLVFEFLHQDLKKFMDASALTGIPLPLIKSYLFQ  110
CDK2-207     MENFQKVEKIGEGTYGVVYKARNKLTGEVVALKKIRLDT.......................................................................   39
```

logo

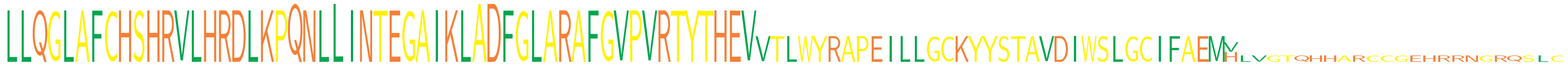

```
BMS-387032   ....................Q..L.........AD...........................................................................   15
PF00069.2    LLQGLAFCHSHRVLHRDLKPQNLLINTEGAIKLADFGLARAFGVPVRTYTHEV.........................................................  134
PF00069.3    LLQGLAFCHSHRVLHRDLKPQNLLINTEGAIKLADFGLARAFGVPVRTYTHEVVTLWYRAPEILLGCKYYSTAVDIWSLGCIFAEMH.......................  194
PF00069.1    LLQGLAFCHSHRVLHRDLKPQNLLINTEGAIKLADFGLARAFGVPVRTYTHEVVTLWYRAPEILLGCKYYSTAVDIWSLGCIFAEMV.......................  194
PF00069      LLQGLAFCHSHRVLHRDLKPQNLLINTEGAIKLADFGLARAFGVPVRTYTHEV.........................................................  160
CDK2-208     ..............................................................................................................   39
CDK2-203     LLQGLAFCHSHRVLHRDLKPQNLLINTEGAIKLADFGLARAFGVPVRTYTHEV.........................................................  137
CDK2-204     LLQGLAFCHSHRVLHRDLKPQNLLINTEGAIKLADFGLARAFGVPVRTYTHEVVTLWYRAPEILLGCKYYSTAVDIWSLGCIFAEMHLVGTQHHARCCGEHRRNGRQSLC  220
CDK2-201     LLQGLAFCHSHRVLHRDLKPQNLLINTEGAIKLADFGLARAFGVPVRTYTHEVVTLWYRAPEILLGCKYYSTAVDIWSLGCIFAEMV.......................  197
CDK2-202     LLQGLAFCHSHRVLHRDLKPQNLLINTEGAIKLADFGLARAFGVPVRTYTHEV.........................................................  163
CDK2-207     ..............................................................................................................   39
```

logo

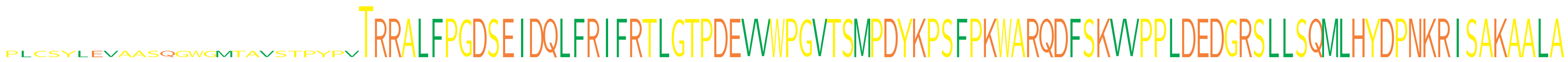

```
BMS-387032   ..............................................................................................................   15
PF00069.2    .........................T....................................................................................  135
PF00069.3    .........................TRRALFPGDSEIDQLFRIFRTLGTPDEVVWPGVTSMPDYKPSFPKWARQDFSKVVPPLDEDGRSLLSQMLHYDPNKRISAKAALA  279
PF00069.1    .........................TRRALFPGDSEIDQLFRIFRTLGTPDEVVWPGVTSMPDYKPSFPKWARQDFSKVVPPLDEDGRSLLSQMLHYDPNKRISAKAALA  279
PF00069      .........................T....................................................................................  161
CDK2-208     ..............................................................................................................   39
CDK2-203     .........................TRRALFPGDSEIDQLFRIFRTLGTPDEVVWPGVTSMPDYKPSFPKWARQDFSKVVPPLDEDGRSLLSQMLHYDPNKRISAKAALA  222
CDK2-204     PLCSYLEVAASQGWGMTAVSTPYPVTRRALFPGDSEIDQLFRIFRTLGTPDEVVWPGVTSMPDYKPSFPKWARQDFSKVVPPLDEDGRSLLSQMLHYDPNKRISAKAALA  330
CDK2-201     .........................TRRALFPGDSEIDQLFRIFRTLGTPDEVVWPGVTSMPDYKPSFPKWARQDFSKVVPPLDEDGRSLLSQMLHYDPNKRISAKAALA  282
CDK2-202     .........................TRRALFPGDSEIDQLFRIFRTLGTPDEVVWPGVTSMPDYKPSFPKWARQDFSKVVPPLDEDGRSLLSQMLHYDPNKRISAKAALA  248
CDK2-207     ..............................................................................................................   39
```

```
logo          HPFFQDVTKPVPHLRL
BMS-387032    ................     15
PF00069.2     ................    135
PF00069.3     HPFF............    283
PF00069.1     HPFF............    283
PF00069       ................    161
CDK2-208      ................     39
CDK2-203      HPFFQDVTKPVPHLRL    238
CDK2-204      HPFFQDVTKPVPHLRL    346
CDK2-201      HPFFQDVTKPVPHLRL    298
CDK2-202      HPFFQDVTKPVPHLRL    264
CDK2-207      ................     39
```

X non conserved
X similar
X $\geq$ 0% conserved
X $\geq$ 50% conserved

```
logo
CHEMBL303958  .........I.E.....V............A.K..............................V................EFL...........................   9
PF00069.2     ...FQKVEKIGEGTYGVVYKARNKLTGEVVALKKIRLDT..........................LLDVIHTENKLYLVFEFLHQDLKKFMDASALTGIPLPLIKSYLFQ  81
PF00069.3     ...FQKVEKIGEGTYGVVYKARNKLTGEVVALKKIRLDTETEGVPSTAIREISLLKELNHPNIVKLLDVIHTENKLYLVFEFLHQDLKKFMDASALTGIPLPLIKSYLFQ 107
PF00069.1     ...FQKVEKIGEGTYGVVYKARNKLTGEVVALKKIRLDTETEGVPSTAIREISLLKELNHPNIVKLLDVIHTENKLYLVFEFLHQDLKKFMDASALTGIPLPLIKSYLFQ 107
PF00069       ...FQKVEKIGEGTYGVVYKARNKLTGEVVALKKIRLDTETEGVPSTAIREISLLKELNHPNIVKLLDVIHTENKLYLVFEFLHQDLKKFMDASALTGIPLPLIKSYLFQ 107
CDK2-208      MENFQKVEKIGEGTYGVVYKARNKLTGEVVALKKIRLDT.......................................................................  39
CDK2-203      MENFQKVEKIGEGTYGVVYKARNKLTGEVVALKKIRLDT..........................LLDVIHTENKLYLVFEFLHQDLKKFMDASALTGIPLPLIKSYLFQ  84
CDK2-204      MENFQKVEKIGEGTYGVVYKARNKLTGEVVALKKIRLDTETEGVPSTAIREISLLKELNHPNIVKLLDVIHTENKLYLVFEFLHQDLKKFMDASALTGIPLPLIKSYLFQ 110
CDK2-201      MENFQKVEKIGEGTYGVVYKARNKLTGEVVALKKIRLDTETEGVPSTAIREISLLKELNHPNIVKLLDVIHTENKLYLVFEFLHQDLKKFMDASALTGIPLPLIKSYLFQ 110
CDK2-202      MENFQKVEKIGEGTYGVVYKARNKLTGEVVALKKIRLDTETEGVPSTAIREISLLKELNHPNIVKLLDVIHTENKLYLVFEFLHQDLKKFMDASALTGIPLPLIKSYLFQ 110
CDK2-207      MENFQKVEKIGEGTYGVVYKARNKLTGEVVALKKIRLDT.......................................................................  39

logo
CHEMBL303958  ............................................................................................................L.  10
PF00069.2     LLQGLAFCHSHRVLHRDLKPQNLLINTEGAIKLADFGLARAFGVPVRTYTHEV......................................................... 134
PF00069.3     LLQGLAFCHSHRVLHRDLKPQNLLINTEGAIKLADFGLARAFGVPVRTYTHEVVTLWYRAPEILLGCKYYSTAVDIWSLGCIFAEMH........................ 194
PF00069.1     LLQGLAFCHSHRVLHRDLKPQNLLINTEGAIKLADFGLARAFGVPVRTYTHEVVTLWYRAPEILLGCKYYSTAVDIWSLGCIFAEMV........................ 194
PF00069       LLQGLAFCHSHRVLHRDLKPQNLLINTEGAIKLADFGLARAFGVPVRTYTHEV......................................................... 160
CDK2-208      ..............................................................................................................  39
CDK2-203      LLQGLAFCHSHRVLHRDLKPQNLLINTEGAIKLADFGLARAFGVPVRTYTHEV......................................................... 137
CDK2-204      LLQGLAFCHSHRVLHRDLKPQNLLINTEGAIKLADFGLARAFGVPVRTYTHEVVTLWYRAPEILLGCKYYSTAVDIWSLGCIFAEMHLVGTQHHARCCGEHRRNGRQSLC 220
CDK2-201      LLQGLAFCHSHRVLHRDLKPQNLLINTEGAIKLADFGLARAFGVPVRTYTHEVVTLWYRAPEILLGCKYYSTAVDIWSLGCIFAEMV........................ 197
CDK2-202      LLQGLAFCHSHRVLHRDLKPQNLLINTEGAIKLADFGLARAFGVPVRTYTHEV......................................................... 163
CDK2-207      ..............................................................................................................  39

logo
CHEMBL303958  ........A.....................................................................................................  11
PF00069.2     .........................T.................................................................................... 135
PF00069.3     .........................TRRALFPGDSEIDQLFRIFRTLGTPDEVVWPGVTSMPDYKPSFPKWARQDFSKVVPPLDEDGRSLLSQMLHYDPNKRISAKAALA 279
PF00069.1     .........................TRRALFPGDSEIDQLFRIFRTLGTPDEVVWPGVTSMPDYKPSFPKWARQDFSKVVPPLDEDGRSLLSQMLHYDPNKRISAKAALA 279
PF00069       .........................T.................................................................................... 161
CDK2-208      ..............................................................................................................  39
CDK2-203      .........................TRRALFPGDSEIDQLFRIFRTLGTPDEVVWPGVTSMPDYKPSFPKWARQDFSKVVPPLDEDGRSLLSQMLHYDPNKRISAKAALA 222
CDK2-204      PLCSYLEVAASQGWGMTAVSTPYPVTRRALFPGDSEIDQLFRIFRTLGTPDEVVWPGVTSMPDYKPSFPKWARQDFSKVVPPLDEDGRSLLSQMLHYDPNKRISAKAALA 330
CDK2-201      .........................TRRALFPGDSEIDQLFRIFRTLGTPDEVVWPGVTSMPDYKPSFPKWARQDFSKVVPPLDEDGRSLLSQMLHYDPNKRISAKAALA 282
CDK2-202      .........................TRRALFPGDSEIDQLFRIFRTLGTPDEVVWPGVTSMPDYKPSFPKWARQDFSKVVPPLDEDGRSLLSQMLHYDPNKRISAKAALA 248
CDK2-207      ..............................................................................................................  39
```

logo	HPFFQDVTKPVPHLRL	
CHEMBL303958	....	11
PF00069.2	....	135
PF00069.3	HPFF............	283
PF00069.1	HPFF............	283
PF00069	....	161
CDK2-208	....	39
CDK2-203	HPFFQDVTKPVPHLRL	238
CDK2-204	HPFFQDVTKPVPHLRL	346
CDK2-201	HPFFQDVTKPVPHLRL	298
CDK2-202	HPFFQDVTKPVPHLRL	264
CDK2-207	....	39

X	non conserved
X	similar
X	$\geq$ 0% conserved
X	$\geq$ 50% conserved

```
logo         MENFQKVEKIGEGTYGVVYKARNKLTGEVVALKKIRLDTETEGVPSTAIREISLLKELNHPNIVKLLDVIHTENKLYLVFEFLHQDLKKFMDASALTGIPLPLIKSYLFQ
RG-547       .................V............A.K..............................V...............FEFLHQD..K.....................  12
PF00069.2    ...FQKVEKIGEGTYGVVYKARNKLTGEVVALKKIRLDT..........................LLDVIHTENKLYLVFEFLHQDLKKFMDASALTGIPLPLIKSYLFQ  81
PF00069.3    ...FQKVEKIGEGTYGVVYKARNKLTGEVVALKKIRLDTETEGVPSTAIREISLLKELNHPNIVKLLDVIHTENKLYLVFEFLHQDLKKFMDASALTGIPLPLIKSYLFQ  107
PF00069.1    ...FQKVEKIGEGTYGVVYKARNKLTGEVVALKKIRLDTETEGVPSTAIREISLLKELNHPNIVKLLDVIHTENKLYLVFEFLHQDLKKFMDASALTGIPLPLIKSYLFQ  107
PF00069      ...FQKVEKIGEGTYGVVYKARNKLTGEVVALKKIRLDTETEGVPSTAIREISLLKELNHPNIVKLLDVIHTENKLYLVFEFLHQDLKKFMDASALTGIPLPLIKSYLFQ  107
CDK2-208     MENFQKVEKIGEGTYGVVYKARNKLTGEVVALKKIRLDT.......................................................................  39
CDK2-203     MENFQKVEKIGEGTYGVVYKARNKLTGEVVALKKIRLDT..........................LLDVIHTENKLYLVFEFLHQDLKKFMDASALTGIPLPLIKSYLFQ  84
CDK2-204     MENFQKVEKIGEGTYGVVYKARNKLTGEVVALKKIRLDTETEGVPSTAIREISLLKELNHPNIVKLLDVIHTENKLYLVFEFLHQDLKKFMDASALTGIPLPLIKSYLFQ  110
CDK2-201     MENFQKVEKIGEGTYGVVYKARNKLTGEVVALKKIRLDTETEGVPSTAIREISLLKELNHPNIVKLLDVIHTENKLYLVFEFLHQDLKKFMDASALTGIPLPLIKSYLFQ  110
CDK2-202     MENFQKVEKIGEGTYGVVYKARNKLTGEVVALKKIRLDTETEGVPSTAIREISLLKELNHPNIVKLLDVIHTENKLYLVFEFLHQDLKKFMDASALTGIPLPLIKSYLFQ  110
CDK2-207     MENFQKVEKIGEGTYGVVYKARNKLTGEVVALKKIRLDT.......................................................................  39

logo         LLQGLAFCHSHRVLHRDLKPQNLLINTEGAIKLADFGLARAFGVPVRTYTHEVVTLWYRAPEILLGCKYYSTAVDIWSLGCIFAEMHLVGTQHHARCCGEHRRNGRQSLC
RG-547       ....................QN.L.........AD...........................................................................  17
PF00069.2    LLQGLAFCHSHRVLHRDLKPQNLLINTEGAIKLADFGLARAFGVPVRTYTHEV.........................................................  134
PF00069.3    LLQGLAFCHSHRVLHRDLKPQNLLINTEGAIKLADFGLARAFGVPVRTYTHEVVTLWYRAPEILLGCKYYSTAVDIWSLGCIFAEMH.......................  194
PF00069.1    LLQGLAFCHSHRVLHRDLKPQNLLINTEGAIKLADFGLARAFGVPVRTYTHEVVTLWYRAPEILLGCKYYSTAVDIWSLGCIFAEMV.......................  194
PF00069      LLQGLAFCHSHRVLHRDLKPQNLLINTEGAIKLADFGLARAFGVPVRTYTHEV.........................................................  160
CDK2-208     ..............................................................................................................  39
CDK2-203     LLQGLAFCHSHRVLHRDLKPQNLLINTEGAIKLADFGLARAFGVPVRTYTHEV.........................................................  137
CDK2-204     LLQGLAFCHSHRVLHRDLKPQNLLINTEGAIKLADFGLARAFGVPVRTYTHEVVTLWYRAPEILLGCKYYSTAVDIWSLGCIFAEMHLVGTQHHARCCGEHRRNGRQSLC  220
CDK2-201     LLQGLAFCHSHRVLHRDLKPQNLLINTEGAIKLADFGLARAFGVPVRTYTHEVVTLWYRAPEILLGCKYYSTAVDIWSLGCIFAEMV.......................  197
CDK2-202     LLQGLAFCHSHRVLHRDLKPQNLLINTEGAIKLADFGLARAFGVPVRTYTHEV.........................................................  163
CDK2-207     ..............................................................................................................  39

logo         PLCSYLEVAASQGWGMTAVSTPYPVTRRALFPGDSEIDQLFRIFRTLGTPDEVVWPGVTSMPDYKPSFPKWARQDFSKVVPPLDEDGRSLLSQMLHYDPNKRISAKAALA
RG-547       ..............................................................................................................  17
PF00069.2    .........................T....................................................................................  135
PF00069.3    .........................TRRALFPGDSEIDQLFRIFRTLGTPDEVVWPGVTSMPDYKPSFPKWARQDFSKVVPPLDEDGRSLLSQMLHYDPNKRISAKAALA  279
PF00069.1    .........................TRRALFPGDSEIDQLFRIFRTLGTPDEVVWPGVTSMPDYKPSFPKWARQDFSKVVPPLDEDGRSLLSQMLHYDPNKRISAKAALA  279
PF00069      .........................T....................................................................................  161
CDK2-208     ..............................................................................................................  39
CDK2-203     .........................TRRALFPGDSEIDQLFRIFRTLGTPDEVVWPGVTSMPDYKPSFPKWARQDFSKVVPPLDEDGRSLLSQMLHYDPNKRISAKAALA  222
CDK2-204     PLCSYLEVAASQGWGMTAVSTPYPVTRRALFPGDSEIDQLFRIFRTLGTPDEVVWPGVTSMPDYKPSFPKWARQDFSKVVPPLDEDGRSLLSQMLHYDPNKRISAKAALA  330
CDK2-201     .........................TRRALFPGDSEIDQLFRIFRTLGTPDEVVWPGVTSMPDYKPSFPKWARQDFSKVVPPLDEDGRSLLSQMLHYDPNKRISAKAALA  282
CDK2-202     .........................TRRALFPGDSEIDQLFRIFRTLGTPDEVVWPGVTSMPDYKPSFPKWARQDFSKVVPPLDEDGRSLLSQMLHYDPNKRISAKAALA  248
CDK2-207     ..............................................................................................................  39
```

```
logo         HPFFQDVTKPVPHLRL
RG-547       ................    17
PF00069.2    ................   135
PF00069.3    HPFF............   283
PF00069.1    HPFF............   283
PF00069      ................   161
CDK2-208     ................    39
CDK2-203     HPFFQDVTKPVPHLRL   238
CDK2-204     HPFFQDVTKPVPHLRL   346
CDK2-201     HPFFQDVTKPVPHLRL   298
CDK2-202     HPFFQDVTKPVPHLRL   264
CDK2-207     ................    39
```

X non conserved

X similar

X ≥ 0% conserved

X ≥ 50% conserved

logo

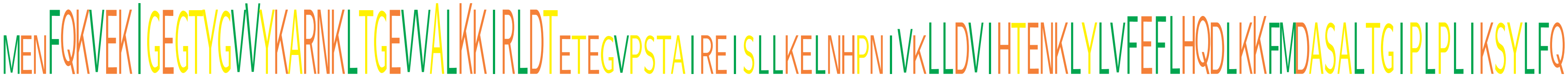

```
SELICICLIB   .........I.......V............A.K..............................V...............FEFLHQ...K.....................   12
PF00069.2    ...FQKVEKIGEGTYGVVYKARNKLTGEVVALKKIRLDT..........................LLDVIHTENKLYLVFEFLHQDLKKFMDASALTGIPLPLIKSYLFQ   81
PF00069.3    ...FQKVEKIGEGTYGVVYKARNKLTGEVVALKKIRLDTETEGVPSTAIREISLLKELNHPNIVKLLDVIHTENKLYLVFEFLHQDLKKFMDASALTGIPLPLIKSYLFQ  107
PF00069.1    ...FQKVEKIGEGTYGVVYKARNKLTGEVVALKKIRLDTETEGVPSTAIREISLLKELNHPNIVKLLDVIHTENKLYLVFEFLHQDLKKFMDASALTGIPLPLIKSYLFQ  107
PF00069      ...FQKVEKIGEGTYGVVYKARNKLTGEVVALKKIRLDTETEGVPSTAIREISLLKELNHPNIVKLLDVIHTENKLYLVFEFLHQDLKKFMDASALTGIPLPLIKSYLFQ  107
CDK2-208     MENFQKVEKIGEGTYGVVYKARNKLTGEVVALKKIRLDT.......................................................................   39
CDK2-203     MENFQKVEKIGEGTYGVVYKARNKLTGEVVALKKIRLDT..........................LLDVIHTENKLYLVFEFLHQDLKKFMDASALTGIPLPLIKSYLFQ   84
CDK2-204     MENFQKVEKIGEGTYGVVYKARNKLTGEVVALKKIRLDTETEGVPSTAIREISLLKELNHPNIVKLLDVIHTENKLYLVFEFLHQDLKKFMDASALTGIPLPLIKSYLFQ  110
CDK2-201     MENFQKVEKIGEGTYGVVYKARNKLTGEVVALKKIRLDTETEGVPSTAIREISLLKELNHPNIVKLLDVIHTENKLYLVFEFLHQDLKKFMDASALTGIPLPLIKSYLFQ  110
CDK2-202     MENFQKVEKIGEGTYGVVYKARNKLTGEVVALKKIRLDTETEGVPSTAIREISLLKELNHPNIVKLLDVIHTENKLYLVFEFLHQDLKKFMDASALTGIPLPLIKSYLFQ  110
CDK2-207     MENFQKVEKIGEGTYGVVYKARNKLTGEVVALKKIRLDT.......................................................................   39
```

logo

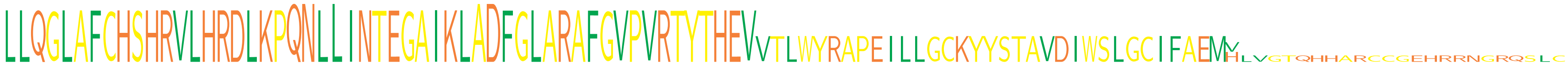

```
SELICICLIB   ....................QN.L.........AD...........................................................................   17
PF00069.2    LLQGLAFCHSHRVLHRDLKPQNLLINTEGAIKLADFGLARAFGVPVRTYTHEV.........................................................  134
PF00069.3    LLQGLAFCHSHRVLHRDLKPQNLLINTEGAIKLADFGLARAFGVPVRTYTHEVVTLWYRAPEILLGCKYYSTAVDIWSLGCIFAEMH.......................  194
PF00069.1    LLQGLAFCHSHRVLHRDLKPQNLLINTEGAIKLADFGLARAFGVPVRTYTHEVVTLWYRAPEILLGCKYYSTAVDIWSLGCIFAEMV.......................  194
PF00069      LLQGLAFCHSHRVLHRDLKPQNLLINTEGAIKLADFGLARAFGVPVRTYTHEV.........................................................  160
CDK2-208     ..............................................................................................................   39
CDK2-203     LLQGLAFCHSHRVLHRDLKPQNLLINTEGAIKLADFGLARAFGVPVRTYTHEV.........................................................  137
CDK2-204     LLQGLAFCHSHRVLHRDLKPQNLLINTEGAIKLADFGLARAFGVPVRTYTHEVVTLWYRAPEILLGCKYYSTAVDIWSLGCIFAEMHLVGTQHHARCCGEHRRNGRQSLC  220
CDK2-201     LLQGLAFCHSHRVLHRDLKPQNLLINTEGAIKLADFGLARAFGVPVRTYTHEVVTLWYRAPEILLGCKYYSTAVDIWSLGCIFAEMV.......................  197
CDK2-202     LLQGLAFCHSHRVLHRDLKPQNLLINTEGAIKLADFGLARAFGVPVRTYTHEV.........................................................  163
CDK2-207     ..............................................................................................................   39
```

logo

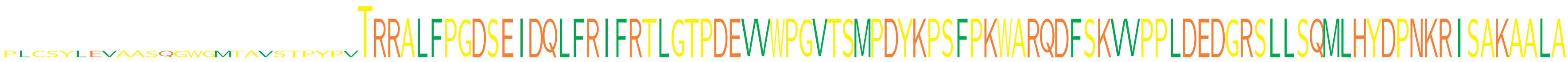

```
SELICICLIB   ..............................................................................................................   17
PF00069.2    .........................T....................................................................................  135
PF00069.3    .........................TRRALFPGDSEIDQLFRIFRTLGTPDEVVWPGVTSMPDYKPSFPKWARQDFSKVVPPLDEDGRSLLSQMLHYDPNKRISAKAALA  279
PF00069.1    .........................TRRALFPGDSEIDQLFRIFRTLGTPDEVVWPGVTSMPDYKPSFPKWARQDFSKVVPPLDEDGRSLLSQMLHYDPNKRISAKAALA  279
PF00069      .........................T....................................................................................  161
CDK2-208     ..............................................................................................................   39
CDK2-203     .........................TRRALFPGDSEIDQLFRIFRTLGTPDEVVWPGVTSMPDYKPSFPKWARQDFSKVVPPLDEDGRSLLSQMLHYDPNKRISAKAALA  222
CDK2-204     PLCSYLEVAASQGWGMTAVSTPYPVTRRALFPGDSEIDQLFRIFRTLGTPDEVVWPGVTSMPDYKPSFPKWARQDFSKVVPPLDEDGRSLLSQMLHYDPNKRISAKAALA  330
CDK2-201     .........................TRRALFPGDSEIDQLFRIFRTLGTPDEVVWPGVTSMPDYKPSFPKWARQDFSKVVPPLDEDGRSLLSQMLHYDPNKRISAKAALA  282
CDK2-202     .........................TRRALFPGDSEIDQLFRIFRTLGTPDEVVWPGVTSMPDYKPSFPKWARQDFSKVVPPLDEDGRSLLSQMLHYDPNKRISAKAALA  248
CDK2-207     ..............................................................................................................   39
```

```
logo          HPFFQDVTKPVPHLRL
SELICICLIB    ................    17
PF00069.2     ................   135
PF00069.3     HPFF............   283
PF00069.1     HPFF............   283
PF00069       ................   161
CDK2-208      ................    39
CDK2-203      HPFFQDVTKPVPHLRL   238
CDK2-204      HPFFQDVTKPVPHLRL   346
CDK2-201      HPFFQDVTKPVPHLRL   298
CDK2-202      HPFFQDVTKPVPHLRL   264
CDK2-207      ................    39
```

X non conserved
X similar
X $\geq 0\%$ conserved
X $\geq 50\%$ conserved

logo

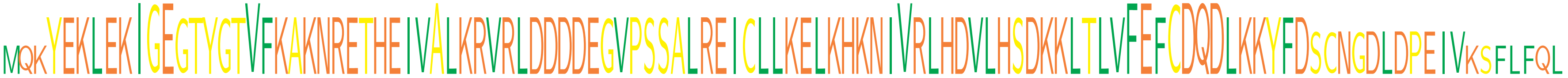

```
SELICICLIB  .........IGE.....V............A................................V...............FE.CDQD........................   12
PF00069     ...YEKLEKIGEGTYGTVFKAKNRETHEIVALKRVRLDDDDEGVPSSALREICLLKELKHKNIVRLHDVLHSDKKLTLVFEFCDQDLKKYFD..................   89
PF00069.1   ...YEKLEKIGEGTYGTVFKAKNRETHEIVALKRVRLDDDDEGVPSSALREICLLKELKHKNIVRLHDVLHSDKKLTLVFEFCDQDLKKYFDSCNGDLDPEIVKSFLFQL  107
CDK5-201    MQKYEKLEKIGEGTYGTVFKAKNRETHEIVALKRVRLDDDDEGVPSSALREICLLKELKHKNIVRLHDVLHSDKKLTLVFEFCDQDLKKYFDSCNGDLDPEIV.......  103
CDK5-204    MQKYEKLEKIGEGTYGTVFKAKNRETHEIVALKRVRLDDDDEGVPSSALREICLLKELKHKNIVRLHDVLHSDKKLTLVFEFCDQDLKKYFDSCNGDLDPEIVKSFLFQL  110
```

logo

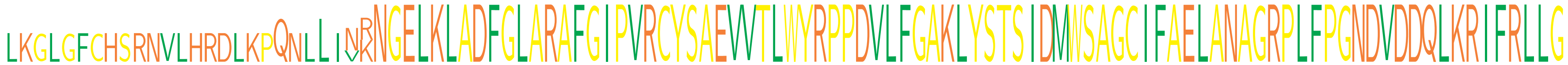

```
SELICICLIB  ...................Q..L.......................................................................................   14
PF00069     ........................VKNGELKLADFGLARAFGIPVRCYSAEVVTLWYRPPDVLFGAKLYSTSIDMWSAGCIFAELANAGRPLFPGNDVDDQLKRIFRLLG  175
PF00069.1   LKGLGFCHSRNVLHRDLKPQNLLINRNGELKLADFGLARAFGIPVRCYSAEVVTLWYRPPDVLFGAKLYSTSIDMWSAGCIFAELANAGRPLFPGNDVDDQLKRIFRLLG  217
CDK5-201    .........................KNGELKLADFGLARAFGIPVRCYSAEVVTLWYRPPDVLFGAKLYSTSIDMWSAGCIFAELANAGRPLFPGNDVDDQLKRIFRLLG  188
CDK5-204    LKGLGFCHSRNVLHRDLKPQNLLINRNGELKLADFGLARAFGIPVRCYSAEVVTLWYRPPDVLFGAKLYSTSIDMWSAGCIFAELANAGRPLFPGNDVDDQLKRIFRLLG  220
```

logo

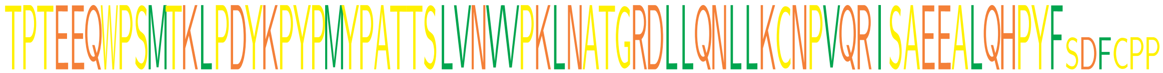

```
SELICICLIB  ........................................................................   14
PF00069     TPTEEQWPSMTKLPDYKPYPMYPATTSLVNVVPKLNATGRDLLQNLLKCNPVQRISAEEALQHPYF......  241
PF00069.1   TPTEEQWPSMTKLPDYKPYPMYPATTSLVNVVPKLNATGRDLLQNLLKCNPVQRISAEEALQHPYF......  283
CDK5-201    TPTEEQWPSMTKLPDYKPYPMYPATTSLVNVVPKLNATGRDLLQNLLKCNPVQRISAEEALQHPYFSDFCPP  260
CDK5-204    TPTEEQWPSMTKLPDYKPYPMYPATTSLVNVVPKLNATGRDLLQNLLKCNPVQRISAEEALQHPYFSDFCPP  292
```

X non conserved
X similar
X $\geq 0\%$ conserved
X $\geq 50\%$ conserved

```
logo         MEKDGLCRADQQYECVAEIGEGAYGKVFKARDLKNGGRFVALKRVRVQTGEEGMPLSTIREVAVLRHLETFEHPNVVRLFDVCTVSRTDRETKLTLVFEHVDQDLTTYLD
ABEMACICLIB  ..................IG...Y..V.............A.K.................................V....................FEHV..D..T...    13
PF00069      ............YECVAEIGEGAYGKVFKARDLKNGGRFVALKRVRVQTGEEGMPLSTIREVAVLRHLETFEHPNVVRLFDVCTVSRTDRETKLTLVFEHVDQDLTTYLD    98
CDK6-202     MEKDGLCRADQQYECVAEIGEGAYGKVFKARDLKNGGRFVALKRVRVQTGEEGMPLSTIREVAVLRHLETFEHPNVVRLFDVCTVSRTDRETKLTLVFEHVDQDLTTYLD   110
CDK6-201     MEKDGLCRADQQYECVAEIGEGAYGKVFKARDLKNGGRFVALKRVRVQTGEEGMPLSTIREVAVLRHLETFEHPNVVRLFDVCTVSRTDRETKLTLVFEHVDQDLTTYLD   110

logo         KVPEPGVPTETIKDMMFQLLRGLDFLHSHRVVHRDLKPQNILVTSSGQIKLADFGLARIYSFQMALTSVVVTLWYRAPEVLLQSSYATPVDLWSVGCIFAEMFRRKPLFR
ABEMACICLIB  ......................................Q..L..........D.........................................................    16
PF00069      KVPEPGVPTETIKDMMFQLLRGLDFLHSHRVVHRDLKPQNILVTSSGQIKLADFGLARIYSFQMALTSVVVTLWYRAPEVLLQSSYATPVDLWSVGCIFAEMFRRKPLFR   208
CDK6-202     KVPEPGVPTETIKDMMFQLLRGLDFLHSHRVVHRDLKPQNILVTSSGQIKLADFGLARIYSFQMALTSVVVTLWYRAPEVLLQSSYATPVDLWSVGCIFAEMFRRKPLFR   220
CDK6-201     KVPEPGVPTETIKDMMFQLLRGLDFLHSHRVVHRDLKPQNILVTSSGQIKLADFGLARIYSFQMALTSVVVTLWYRAPEVLLQSSYATPVDLWSVGCIFAEMFRRKPLFR   220

logo         GSSDVDQLGKILDVIGLPGEEDWPRDVALPRQAFHSKSAQPIEKFVTDIDELGKDLLLKCLTFNPAKRISAYSALSHPYFQDLERCKENLDSHLPPSQNTSELNTA
ABEMACICLIB  ..........................................................................................................    16
PF00069      GSSDVDQLGKILDVIGLPGEEDWPRDVALPRQAFHSKSAQPIEKFVTDIDELGKDLLLKCLTFNPAKRISAYSALSHPYF..........................   288
CDK6-202     GSSDVDQLGKILDVIGLPGEEDWPRDVALPRQAFHSKSAQPIEKFVTDIDELGKDLLLKCLTFNPAKRISAYSALSHPYFQDLERCKENLDSHLPPSQNTSELNTA   326
CDK6-201     GSSDVDQLGKILDVIGLPGEEDWPRDVALPRQAFHSKSAQPIEKFVTDIDELGKDLLLKCLTFNPAKRISAYSALSHPYFQDLERCKENLDSHLPPSQNTSELNTA   326
```

X non conserved
X similar
X ≥ 0% conserved
X ≥ 50% conserved

```
logo
PALBOCICLIB  ..................I....Y..V.............A.K................................V....................FE.VDQD...T...    13
PF00069      ............YECVAEIGEGAYGKVFKARDLKNGGRFVALKRVRVQTGEEGMPLSTIREVAVLRHLETFEHPNVVRLFDVCTVSRTDRETKLTLVFEHVDQDLTTYLD    98
CDK6-202     MEKDGLCRADQQYECVAEIGEGAYGKVFKARDLKNGGRFVALKRVRVQTGEEGMPLSTIREVAVLRHLETFEHPNVVRLFDVCTVSRTDRETKLTLVFEHVDQDLTTYLD   110
CDK6-201     MEKDGLCRADQQYECVAEIGEGAYGKVFKARDLKNGGRFVALKRVRVQTGEEGMPLSTIREVAVLRHLETFEHPNVVRLFDVCTVSRTDRETKLTLVFEHVDQDLTTYLD   110

logo
PALBOCICLIB  ......................................QN.L.........AD.........................................................    18
PF00069      KVPEPGVPTETIKDMMFQLLRGLDFLHSHRVVHRDLKPQNILVTSSGQIKLADFGLARIYSFQMALTSVVVTLWYRAPEVLLQSSYATPVDLWSVGCIFAEMFRRKPLFR   208
CDK6-202     KVPEPGVPTETIKDMMFQLLRGLDFLHSHRVVHRDLKPQNILVTSSGQIKLADFGLARIYSFQMALTSVVVTLWYRAPEVLLQSSYATPVDLWSVGCIFAEMFRRKPLFR   220
CDK6-201     KVPEPGVPTETIKDMMFQLLRGLDFLHSHRVVHRDLKPQNILVTSSGQIKLADFGLARIYSFQMALTSVVVTLWYRAPEVLLQSSYATPVDLWSVGCIFAEMFRRKPLFR   220

logo
PALBOCICLIB  ..........................................................................................................    18
PF00069      GSSDVDQLGKILDVIGLPGEEDWPRDVALPRQAFHSKSAQPIEKFVTDIDELGKDLLLKCLTFNPAKRISAYSALSHPYF..........................   288
CDK6-202     GSSDVDQLGKILDVIGLPGEEDWPRDVALPRQAFHSKSAQPIEKFVTDIDELGKDLLLKCLTFNPAKRISAYSALSHPYFQDLERCKENLDSHLPPSQNTSELNTA   326
CDK6-201     GSSDVDQLGKILDVIGLPGEEDWPRDVALPRQAFHSKSAQPIEKFVTDIDELGKDLLLKCLTFNPAKRISAYSALSHPYFQDLERCKENLDSHLPPSQNTSELNTA   326
```

X non conserved
X similar
X ≥ 0% conserved
X ≥ 50% conserved

```
logo        MEKDGLCRADQQYECVAEIGEGAYGKVFKARDLKNGGRFVALKRVRVQTGEEGMPLSTIREVAVLRHLETFEHPNVVRLFDVCTVSRTDRETKLTLVFEHVDQDLTTYLD
RIBOCICLIB  ..................I.......V.............A.K.................................V....................FE.V..D..T...    10
PF00069     ............YECVAEIGEGAYGKVFKARDLKNGGRFVALKRVRVQTGEEGMPLSTIREVAVLRHLETFEHPNVVRLFDVCTVSRTDRETKLTLVFEHVDQDLTTYLD    98
CDK6-202    MEKDGLCRADQQYECVAEIGEGAYGKVFKARDLKNGGRFVALKRVRVQTGEEGMPLSTIREVAVLRHLETFEHPNVVRLFDVCTVSRTDRETKLTLVFEHVDQDLTTYLD   110
CDK6-201    MEKDGLCRADQQYECVAEIGEGAYGKVFKARDLKNGGRFVALKRVRVQTGEEGMPLSTIREVAVLRHLETFEHPNVVRLFDVCTVSRTDRETKLTLVFEHVDQDLTTYLD   110

logo        KVPEPGVPTETIKDMMFQLLRGLDFLHSHRVVHRDLKPQNILVTSSGQIKLADFGLARIYSFQMALTSVVVTLWYRAPEVLLQSSYATPVDLWSVGCIFAEMFRRKPLFR
RIBOCICLIB  ......................................Q..L.........AD.........................................................    14
PF00069     KVPEPGVPTETIKDMMFQLLRGLDFLHSHRVVHRDLKPQNILVTSSGQIKLADFGLARIYSFQMALTSVVVTLWYRAPEVLLQSSYATPVDLWSVGCIFAEMFRRKPLFR   208
CDK6-202    KVPEPGVPTETIKDMMFQLLRGLDFLHSHRVVHRDLKPQNILVTSSGQIKLADFGLARIYSFQMALTSVVVTLWYRAPEVLLQSSYATPVDLWSVGCIFAEMFRRKPLFR   220
CDK6-201    KVPEPGVPTETIKDMMFQLLRGLDFLHSHRVVHRDLKPQNILVTSSGQIKLADFGLARIYSFQMALTSVVVTLWYRAPEVLLQSSYATPVDLWSVGCIFAEMFRRKPLFR   220

logo        GSSDVDQLGKILDVIGLPGEEDWPRDVALPRQAFHSKSAQPIEKFVTDIDELGKDLLLKCLTFNPAKRISAYSALSHPYFQDLERCKENLDSHLPPSQNTSELNTA
RIBOCICLIB  ..........................................................................................................    14
PF00069     GSSDVDQLGKILDVIGLPGEEDWPRDVALPRQAFHSKSAQPIEKFVTDIDELGKDLLLKCLTFNPAKRISAYSALSHPYF..........................   288
CDK6-202    GSSDVDQLGKILDVIGLPGEEDWPRDVALPRQAFHSKSAQPIEKFVTDIDELGKDLLLKCLTFNPAKRISAYSALSHPYFQDLERCKENLDSHLPPSQNTSELNTA   326
CDK6-201    GSSDVDQLGKILDVIGLPGEEDWPRDVALPRQAFHSKSAQPIEKFVTDIDELGKDLLLKCLTFNPAKRISAYSALSHPYFQDLERCKENLDSHLPPSQNTSELNTA   326
```

X non conserved
X similar
X $\geq$ 0% conserved
X $\geq$ 50% conserved

logo

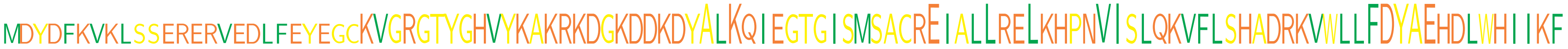

```
SORAFENIB   .................................................A.K.............E...L..L....VI.................FDYAE.........   12
PF00069.1   ..............................................................................................................   0
PF00069     .........................KVGRGTYGHVYKAKRKDGKDDKDYALKQIEGTGISMSACREIALLRELKHPNVISLQKVFLSHADRKVWLLFDYAEHDLWHIIKF   85
CDK8-201    MDYDFKVKLSSERERVEDLFEYEGCKVGRGTYGHVYKAKRKDGKDDKDYALKQIEGTGISMSACREIALLRELKHPNVISLQKVFLSHADRKVWLLFDYAEHDLWHIIKF   110
CDK8-206    ..............................................................................................................   0
CDK8-205    MDYDFKVKLSSERERVEDLFEYEGCKVGRGTYGHVYKAKRKDGKDDKDYALKQIEGTGISMSACREIALLRELKHPNVISLQKVFLSHADRKVWLLFDYAEHDLWHIIKF   110
CDK8-202    ..............................................................................................................   0
```

logo

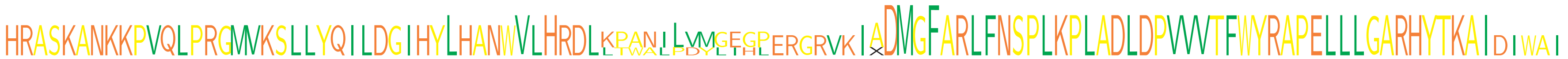

```
SORAFENIB   ...............................L....V.H........L............IADM.F............................................   21
PF00069.1   ..............................................................DMGFARLFNSPLKPLADLDPVVVTFWYRAPELLLGARHYTKAI.....   43
PF00069     HRASKANKKPVQLPRGMVKSLLYQILDGIHYLHANWVLHRDLKPANILVMGEGPERGRVKIADMGFARLFNSPLKPLADLDPVVVTFWYRAPELLLGARHYTKAIDIWAI   195
CDK8-201    HRASKANKKPVQLPRGMVKSLLYQILDGIHYLHANWVLHRDLKPANILVMGEGPERGRVKIADMGFARLFNSPLKPLADLDPVVVTFWYRAPELLLGARHYTKAIDIWAI   220
CDK8-206    ..............................................................................................................   0
CDK8-205    HRASKANKKPVQLPRGMVKSLLYQILDGIHYLHANWVLHRDLLTWALPDYLIHL........................................................   164
CDK8-202    .............................................................XDMGFARLFNSPLKPLADLDPVVVTFWYRAPELLLGARHYTKAI.....   44
```

logo

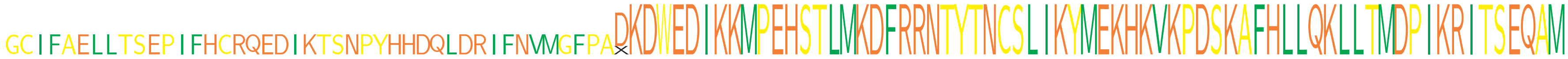

```
SORAFENIB   ..............................................................................................................   21
PF00069.1   ...........................................DKDWEDIKKMPEHSTLMKDFRRNTYTNCSLIKYMEKHKVKPDSKAFHLLQKLLTMDPIKRITSEQAM   110
PF00069     GCIFAELLTSEPIFHCRQEDIKTSNPYHHDQLDRIFNVMGFPADKDWEDIKKMPEHSTLMKDFRRNTYTNCSLIKYMEKHKVKPDSKAFHLLQKLLTMDPIKRITSEQAM   305
CDK8-201    GCIFAELLTSEPIFHCRQEDIKTSNPYHHDQLDRIFNVMGFPADKDWEDIKKMPEHSTLMKDFRRNTYTNCSLIKYMEKHKVKPDSKAFHLLQKLLTMDPIKRITSEQAM   330
CDK8-206    ...........................................XKDWEDIKKMPEHSTLMKDFRRNTYTNCSLIKYMEKHKVKPDSKAFHLLQKLLTMDPIKRITSEQAM   67
CDK8-205    ..............................................................................................................   164
CDK8-202    ...........................................DKDWEDIKKMPEHSTLMKDFRRNTYTNCSLIKYMEKHKVKPDSKAFHLLQKLLTMDPIKRITSEQAM   111
```

logo

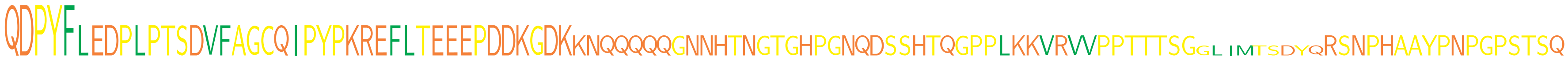

```
SORAFENIB   ..............................................................................................................   21
PF00069.1   QDPYF.........................................................................................................   115
PF00069     QDPYF.........................................................................................................   310
CDK8-201    QDPYFLEDPLPTSDVFAGCQIPYPKREFLTEEEPDDKGDKKNQQQQQGNNHTNGTGHPGNQDSSHTQGPPLKKVRVVPPTTTSGGLIMTSDYQRSNPHAAYPNPGPSTSQ   440
CDK8-206    QDPYFLEDPLPTSDVFAGCQIPYPKREFLTEEEPDDKGDK.....................................................RSNPHAAYPNPGPSTSQ   124
CDK8-205    ..............................................................................................................   164
CDK8-202    QDPYFLEDPLPTSDVFAGCQIPYPKREFLTEEEPDDKGDKKNQQQQQGNNHTNGTGHPGNQDSSHTQGPPLKKVRVVPPTTTSG..........................   195
```

logo

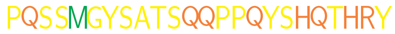

```
SORAFENIB   ........................   21
PF00069.1   ........................   115
PF00069     ........................   310
CDK8-201    PQSSMGYSATSQQPPQYSHQTHRY   464
CDK8-206    PQSSMGYSATSQQPPQYSHQTHRY   148
CDK8-205    ........................   164
CDK8-202    ........................   195
```

X non conserved
X similar
X $\geq 0\%$ conserved
X $\geq 50\%$ conserved

```
logo
             MQRDAPPRAPAPAPRLPAPPIGAAASSGGGGGGGSGGGGGGASAAPAPPGLSGTTSPRGPGGGRRAEEAGSAPRGRKWPWRRKWRGRGGAWSAAAAGPGAGAAAAATGGG
ALVOCIDIB    ..............................................................................................................     0
PF00069      ..............................................................................................................     0
PF00069.1    ..............................................................................................................     0
PF12330      ..............................................................................................................     0
CDK9-201     ..............................................................................................................     0
CDK9-202     MQRDAPPRAPAPAPRLPAPPIGAAASSGGGGGGGSGGGGGGASAAPAPPGLSGTTSPRGPGGGRRAEEAGSAPRGRKWPWRRKWRGRGGAWSAAAAGPGAGAAAAATGGG   110

logo
             GGALEAAMAKQYDSVECPFCDEVSKYEKLAKIGQGTFGEVFKARHRKTGQKVALKKVLMENEKEGFPITALREIKILQLLKHENVVNLIEICRTKASPYNRCKGSIYLVF
ALVOCIDIB    ...............................I....F..V............A.K..............................V.......................F     7
PF00069      .........................YEKLAKIGQGTFGEVFKARHRKTGQKVALKKVLMENEKEGFPITALREIKILQLLKHENVVNLIEICRTKASPYNRCKGSIYLVF    85
PF00069.1    .........................YEKLAKIGQGTFGEVFKARHRKTGQKVALKKVLMENEKEGFPITALREIKILQLLKHEN..........................    59
PF12330      .........................YEKLAKIGQGTFGEVFKARHRKTGQKVALKKVLMENEKEGFPITALREIKILQLLKHENVVNLIEICRTKASPYNRCKGSIYLVF    85
CDK9-201     .......MAKQYDSVECPFCDEVSKYEKLAKIGQGTFGEVFKARHRKTGQKVALKKVLMENEKEGFPITALREIKILQLLKHENVVNLIEICRTKASPYNRCKGSIYLVF   103
CDK9-202     GGALEAAMAKQYDSVECPFCDEVSKYEKLAKIGQGTFGEVFKARHRKTGQKVALKKVLMENEKEGFPITALREIKILQLLKHEN..........................   194

logo
             DFCEHDLAGLLSNVLVKFTLSEIKRVMQMLLNGLYYIHRNKILHRDMKAANVLITRDGVLKLADFGLARAFSLAKNSQPNRYTNRVVTLWYRPPELLLGERDYGPPIDLW
ALVOCIDIB    ..CE.............................................AN.L..........D..............................................    13
PF00069      DFCEHDLAGLLSNVLVKFTLSEIKRVMQMLLNGLYYIHRNKILHRDMKAANVLITRDGVLKLADFGLARAFSLAKNSQPNRYTNRVVTLWYRPPELLLGERDYGPPIDLW   195
PF00069.1    ..............................................................................................................    59
PF12330      DFCEHDLAGLLSNVLVKFTLSEIKRVMQMLLNGLYYIHRNKILHRDMKAANVLITRDGVLKLADFGLARAFSLAK...................................   160
CDK9-201     DFCEHDLAGLLSNVLVKFTLSEIKRVMQMLLNGLYYIHRNKILHRDMKAANVLITRDGVLKLADFGLARAFSLAKNSQPNRYTNRVVTLWYRPPELLLGERDYGPPIDLW   213
CDK9-202     ..............................................................................................................   194

logo
             GAGCIMAEMWTRSPIMQGNTEQHQLALISQLCGSITPEVWPNVDNYELYEKLELVKGQKRKVKDRLKAYVRDPYALDLIDKLLVLDPAQRIDSDDALNHDFFWSDPMPSD
ALVOCIDIB    ..............................................................................................................    13
PF00069      GAGCIMAEMWTRSPIMQGNTEQHQLALISQLCGSITPEVWPNVDNYELYEKLELVKGQKRKVKDRLKAYVRDPYALDLIDKLLVLDPAQRIDSDDALNHDFF........   297
PF00069.1    ..............................................................................................................    59
PF12330      ..............................................................................................................   160
CDK9-201     GAGCIMAEMWTRSPIMQGNTEQHQLALISQLCGSITPEVWPNVDNYELYEKLELVKGQKRKVKDRLKAYVRDPYALDLIDKLLVLDPAQRIDSDDALNHDFFWSDPMPSD   323
CDK9-202     ..............................................................................................................   194

logo
             LKGMLSTHLTSMFEYLAPPRRKGSQITQQSTNQSRNPATTNQTEFERVF
ALVOCIDIB    .................................................    13
PF00069      .................................................   297
PF00069.1    .................................................    59
PF12330      .................................................   160
CDK9-201     LKGMLSTHLTSMFEYLAPPRRKGSQITQQSTNQSRNPATTNQTEFERVF   372
CDK9-202     .................................................   194
```

- X non conserved
- X similar
- X $\geq 0\%$ conserved
- X $\geq 50\%$ conserved

```
logo
```

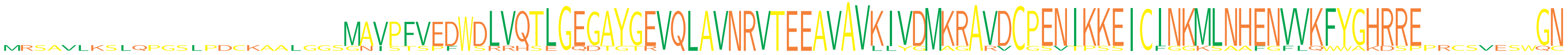

```
AZD-7762     ......................................L....Y...............A..................................................    3
PF00069.4    ..................................LVQTLGEGAYGEVQLAVNRVTEEAVAVKIVDMKRAVDCPENIKKEICINKMLNHENVVKFYGHRRE........GN   68
PF00069.7    .........................................................................ENIKKEICINKMLNHENVVKFYGHRRE........GN   29
PF00069.5    ..............................................................................................................    0
PF00069.6    ..............................................................................................................    0
PF00069.2    ..................................LVQTLGEGAYGEVQLAVNRVTEEAVAVKIVDMKRAVDCPENIKKEICINKMLNHENVVKFYGHRRE........GN   68
PF00069.1    ..............................................................................................................    0
PF00069.3    ..................................LVQTLGEGAYGEVQLAVNRVTEEAVAVKIVDMKRAVDCPENIKKEICINKMLNHENVVKFYGHRRE........GN   68
PF00069      ..................................LVQTLGEGAYGEVQLAVNRVTEEAVAVKIVDMKRAVDCPENIKKEICINKMLNHENVVKFYGHRRE........GN   68
CHEK1-204    ........................MAVPFVEDWDLVQTLGEGAYGEVQLAVNRVTEEAVAVKIVDMKRAVDCPENIKKEICINKMLNHENVVKFYGHRRE........GN   78
CHEK1-206    ........................MAVPFVEDWDLVQTLGEGAYGEVQLAVNRVTEEAVAVKIVDMKRAVDCPENIKKEICINKMLNHENVVKFYGHRRE........GN   78
CHEK1-205    ..............................................................................................................    0
CHEK1-201    ........................MAVPFVEDWDLVQTLGEGAYGEVQLAVNRVTEEAVAVKIVDMKRAVDCPENIKKEICINKMLNHENVVKFYGHRRE........GN   78
CHEK1-217    ........................MAVPFVEDWDLVQTLGEGAYGEVQLAVNRVTEEAVAVKIVDMKRAVDCPENIKKEICINKMLNHENVVKFYGHRRE........GN   78
CHEK1-203    ........................MAVPFVEDWDLVQTLGEGAYGEVQLAVNRVTEEAVAVKIVDMKRAVDCPENIKKEICINKMLNHENVVKFYGHRRE........GN   78
CHEK1-213    ........................MAVPFVEDWDLVQTLGEGAYGEVQLAVNRVTEEAVAVKIVDMKRAVDCPENIKKEICINKMLNHENVVKFYGHRRE........GN   78
CHEK1-208    ........................MAVPFVEDWDLVQTLGEGAYGEVQLAVNRVTEEAVAVKIVDMKRAVDCPENIKKEICINKMLNHENVVKFYGHRRE........GN   78
CHEK1-207    MRSAVLKSLQPGSLPDCKAALGGSGNISTSPFWSRRHSEGQDTGTR.............AVLLYG.AGARVCGSVTPSS..FGGKSAAFGFLQWWAKDSPPRCSVESWQC   94
CHEK1-202    MRSAVLKSLQPGSLPDCKAALGGSGNISTSPFWSRRHSEGQDTGTR.............AVLLYG.AGARVCGSVTPSS..FGGKSAAFGFLQWWAKDSPPRCSVESWQC   94
CHEK1-209    ........................MAVPFVEDWDLVQTLGEGAYGEVQLAVNRVTEEAVAVKIVDMKRAVDCPENIKKEICINKMLNHENVVKFYGHRRE........GN   78
CHEK1-216    ........................MAVPFVEDWDLVQTLGEGAYGEVQLAVNRVTEEAVAVKIVDMKRAVDCPENIKKEICINKMLNHENVVKFYGHRRE........GN   78
CHEK1-218    ..............................................................................................................    0
CHEK1-214    ..............................................................................................................    0
CHEK1-215    ........................MAVPFVEDWDLVQTLGEGAYGEVQLAVNRVTEEAVAVKIVDM............................................   42
CHEK1-212    .................................................................MKRAVDCPENIKKEICINKMLNHENVVKFYGHRRE........GN   37
```

```
logo
```

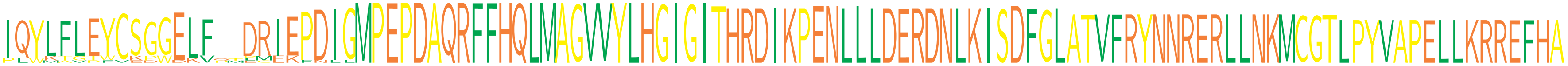

```
AZD-7762     ......EYCS.GE............................................EN.L..........D......................................   13
PF00069.4    IQYLFLEYCSGGELF..DRIEPDIGMPEPDAQRFFHQLMAGVVYLHGIGI............................................................  116
PF00069.7    IQYLFLEYCSGGELF..DRIEPDIGMPEPDAQRFFHQLMAGVVYLHGIGITHRDIKPENLLLDERDNLKISDFGL...................................  102
PF00069.5    ......................DIGMPEPDAQRFFHQLMAGVVYLHGIGITHRDIKPENLLLDERDNLKISDFGLATVFRYNNRERLLNKMCGTLPYVAPELLKRREFHA   88
PF00069.6    .........................MPEPDAQRFFHQLMAGVVYLHGIGITHRDIKPENLLLDERDNLKISDFGLATVFRYNNRERLLNKMCGTLPYVAPELLKRREFHA   85
PF00069.2    IQYLFLEYCSGGELF..DRIEPDIGMPEPDAQRFFHQLMAGVVYLHGIGITHRDIKPENLLLDERDNLKISDFGLATVFRYNNRERLLNKMCGTLPYVAPELLKRREFHA  176
PF00069.1    ........................GMPEPDAQRFFHQLMAGVVYLHGIGITHRDIKPENLLLDERDNLKISDFGLATVFRYNNRERLLNKMCGTLPYVAPELLKRREFHA   86
PF00069.3    IQYLFLEYCSGGELF..DRIEPDIGMPEPDAQRFFHQLMAGVVYLHGIGITHRDIKPENLLLDERDNLKISDFGLATVFRYNNRERLLNKMCGT................  160
PF00069      IQYLFLEYCSGGELF..DRIEPDIGMPEPDAQRFFHQLMAGVVYLHGIGITHRDIKPENLLLDERDNLKISDFGLATVFRYNNRERLLNKMCGTLPYVAPELLKRREFHA  176
CHEK1-204    IQYLFLEYCSGGELF..DRIEPDIGMPEPDAQRFFHQLMAGVVYLHGIGITHRDIKPENLLLDERDNLKISDFGLATVFRYNNRERLLNKMCGTLPYVAPELLKRREFHA  186
CHEK1-206    IQYLFLEYCSGGELF..DRIEPDIGMPEPDAQRFFHQLMAGVVYLHGIGITHRDIKPENLLLDERDNLKISDFGLATVFRYNNRERLLNKMCGTLPYVAPELLKRREFHA  186
CHEK1-205    ..............................................................................................................    0
CHEK1-201    IQYLFLEYCSGGELF..DRIEPDIGMPEPDAQRFFHQLMAGVVYLHGIGITHRDIKPENLLLDERDNLKISDFGLATVFRYNNRERLLNKMCGTLPYVAPELLKRREFHA  186
CHEK1-217    IQYLFLEYCSGGELF..DRIEPDIGMPEPDAQRFFHQLMAGVVYLHGIGIT...........................................................  127
CHEK1-203    IQYLFLEYCSGGELF..DRIEPDIGMPEPDAQRFFHQLMAGVVYLHGIGITHRDIKPENLLLDERDNLKISDFGLATVFRYNNRERLLNKMCGTLPYVAPELLKRREFHA  186
CHEK1-213    IQYLFLEYCSGGELF..DRIEPDIGMPEPDAQRFFHQLMAGVVYLHGIGITHRDIKPENLLLDERDNLKISDFGLATVFRYNNRERLLNKMCGTLPYVAPELLKRREFHA  186
CHEK1-208    IQYLFLEYCSGGELF..DRIEPDIGMPEPDAQRFFHQLMAGVVYLHGIGITHRDIKPENLLLDERDNLKISDFGLATVFRYNNRERLLNKMCGT................  170
CHEK1-207    PLWKTGTWCKPWEKVPME..KFNLL.....................................................................................  117
CHEK1-202    PLWKTGTWCKPWEKV..PMEKPDIGMPEPDAQRFFHQLMAGVVYLHGIGITHRDIKPENLLLDERDNLKISDFGLATVFRYNNRERLLNKMCGTLPYVAPELLKRREFHA  202
CHEK1-209    IQYLFLEYCSGGELF..DRIEPDIGMPEPDAQRFFHQLMAGVVYLHGIGITHRDIKPENLLLDERDNLKISDFGLATVFRYNNRERLLNKMCGTLPYVAPELLKRREFHA  186
CHEK1-216    IQYLFLEYCSGGELF..DRIEPDIGMPEPDAQRFFHQLMAGVVYLHGIGITHRDIKPENLLLDERDNLKISDFGLATVFRYNNRERLLNKMCGTLPYVAPELLKRREFHA  186
CHEK1-218    ..................MEKPDIGMPEPDAQRFFHQLMAGVVYLHGIGITHRDIKPENLLLDERDNLKISDFGLATVFRYNNRERLLNKMCGTLPYVAPELLKRREFHA   92
CHEK1-214    ...MAVPFVEDWDLVQTLGEEPDIGMPEPDAQRFFHQLMAGVVYLHGIGITHRDIKPENLLLDERDNLKISDFGLATVFRYNNRERLLNKMCGTLPYVAPELLKRREFHA  107
CHEK1-215    ..............................................................................................................   42
CHEK1-212    IQYLFLEYCSGGELF..DRIEPDIGMPEPDAQRFFHQLMAGVVYLHGIGITHRDIKPENLLLDERDNLKISDFGL...................................  110
```

```
logo
           EPVDVWSCGIVLTAMLAGELPWDQPSDSCQEYSDWKEKKTYLNPWKKIDSAPLALLHKILVENPSARITIPDIKKDRWYNKPLKKGAKRPRVTSGGVSESPSGFSKHIQS
AZD-7762   ..............................................................................................................  13
PF00069.4  ..............................................................................................................  116
PF00069.7  ..............................................................................................................  102
PF00069.5  EPVDVWSCGIVLTAMLAGELPWDQPSDSCQEYSDWKEKKTYLNPWKKIDSAPLALLHKILVENPSARITIPDIKKDRW................................  166
PF00069.6  EPVDVWSCGIVLTAMLAGELPWDQPSDSCQEYSDWKEKKTYLNPWKKIDSAPLALLHKILVENPSARITIPDIKKDRW................................  163
PF00069.2  EPVDVWSCGIVLTAMLAG............................................................................................  194
PF00069.1  EPVDVWSCGIVLTAMLAGELPWDQPSDSCQEYSDWKEKKTYLNPWKKIDSAPLALLHKILVENPSARITIPDIKKDRW................................  164
PF00069.3  ..............................................................................................................  160
PF00069    EPVDVWSCGIVLTAMLAGELPWDQPSDSCQEYSDWKEKKTYLNPWKKIDSAPLALLHKILVENPSARITIPDIKKDRW................................  254
CHEK1-204  EPVDVWSCGIVLTAMLAGELPWDQPSDSCQEYSDWKEKKTYLNPWKKIDSAPLALLHKILVENPSARITIPDIKKDRWYNKPLKKGAKRPRVTSGGVSESPSGFSKHIQS  296
CHEK1-206  EPVDVWSCGIVLTAMLAGELPWDQPSDSCQEYSDWKEKKTYLNPWKKIDSAPLALLHKILVENPSARITIPDIKKDRWYNKPLKKGAKRPRVTSGGVSESPSGFSKHIQS  296
CHEK1-205  ..............................................................................................................  0
CHEK1-201  EPVDVWSCGIVLTAMLAGELPWDQPSDSCQEYSDWKEKKTYLNPWKKIDSAPLALLHKILVENPSARITIPDIKKDRWYNKPLKKGAKRPRVTSGGVSESPSGFSKHIQS  296
CHEK1-217  ..............................................................................................................  127
CHEK1-203  EPVDVWSCGIVLTAMLAGELPWDQPSDSCQEYSDWKEKKTYLNPWKKIDSAPLALLHKILVENPSARITIPDIKKDRWYNKPLKKGAKRPRVTSGGVSESPSGFSKHIQS  296
CHEK1-213  EPVDVWSCGIVLTAMLAGELPWDQPSDSCQEYSDWKEKKTYLNPWKKIDSAPLALLHKILVENPSARITIPDIKKDRWYNKPLKKGAKRPRVTSGGVSESPSGFSKHIQS  296
CHEK1-208  ..............................................................................................................  170
CHEK1-207  ..............................................................................................................  117
CHEK1-202  EPVDVWSCGIVLTAMLAGELPWDQPSDSCQEYSDWKEKKTYLNPWKKIDSAPLALLHKILVENPSARITIPDIKKDRWYNKPLKKGAKRPRVTSGGVSESPSGFSKHIQS  312
CHEK1-209  EPVDVWSCGIVLTAMLAG............................................................................................  204
CHEK1-216  EPVDVWSCGIVLTAMLAGELPWDQPSDSCQEYSDWKEKKTYLNPWKKIDSAPLALLHKILVENPSARITIPDIKKDRWYNKPLKKGAKRPRVTSGGVSESPSGFSKHIQS  296
CHEK1-218  EPVDVWSCGIVLTAMLAGELPWDQPSDSCQEYSDWKEKKTYLNPWKKIDSAPLALLHKILVENPSARITIPDIKKDRWYNKPLKKGAKRPRVTSGGVSESPSGFSKHIQS  202
CHEK1-214  EPVDVWSCGIVLTAMLAGELPWDQPSDSCQEYSDWKEKKTYLNPWKKIDSAPLALLHKILVENPSARITIPDIKKDRWYNKPLKK.........................  192
CHEK1-215  ..............................................................................................................  42
CHEK1-212  ..............................................................................................................  110

logo
           NLDFSPVNSASSEENVKYSSSQPEPRTGLSLWDTSPSYIDKLVQGISFSQPTCPDHMLLNSQLLGTPGSSQNPWQRLVKRMTRFFTKLDADKSYQCLKETCEKLGYQWKK
AZD-7762   ..............................................................................................................  13
PF00069.4  ..............................................................................................................  116
PF00069.7  ..............................................................................................................  102
PF00069.5  ..............................................................................................................  166
PF00069.6  ..............................................................................................................  163
PF00069.2  ..............................................................................................................  194
PF00069.1  ..............................................................................................................  164
PF00069.3  ..............................................................................................................  160
PF00069    ..............................................................................................................  254
CHEK1-204  NLDFSPVNSASSEENVKYSSSQPEPRTGLSLWDTSPSYIDKLVQGISFSQPTCPDHMLLNSQLLGTPGSSQNPWQRLVKRMTRFFTKLDADKSYQCLKETCEKLGYQWKK  406
CHEK1-206  NLDFSPVNSASSEENVKYSSSQPEPRTGLSLWDTSPSYIDKLVQGISFSQPTCPDHMLLNSQLLGTPGSSQNPWQRLVKRMTRFFTKLDADKSYQCLKETCEKLGYQWKK  406
CHEK1-205  ...........................................................................................KSYQCLKETCEKLGYQWKK  19
CHEK1-201  NLDFSPVNSASSEENVKYSSSQPEPRTGLSLWDTSPSYIDKLVQGISFSQPTCPDHMLLNSQLLGTPGSSQ.......................................  367
CHEK1-217  ..............................................................................................................  127
CHEK1-203  NLDFSPVNSASSEENVKYSSSQPEPRTGLSLWDTSPSYIDKLVQGISFSQPTCPDHMLLNSQLLGTPGSSQNPWQRLVKRMTRFFTKLDADKSYQCLKETCEKLGYQWKK  406
CHEK1-213  NLDFSPVNSASSEENVKYSSSQPEPRTGLSLWDTSPSYIDKLVQGISFSQPTCPDHMLLNSQLLGTPGSSQNPWQRLVKRMTRFFTKLDADKSYQCLKETCEKLGYQWKK  406
CHEK1-208  ..............................................................................................................  170
CHEK1-207  ..............................................................................................................  117
CHEK1-202  NLDFSPVNSASSEENVKYSSSQPEPRTGLSLWDTSPSYIDKLVQGISFSQPTCPDHMLLNSQLLGTPGSSQNPWQRLVKRMTRFFTKLDADKSYQCLKETCEKLGYQWKK  422
CHEK1-209  ..............................................................................................................  204
CHEK1-216  NLDFSPVNSASSEENVKYSSSQPEPRTGLSLWDTSPSYIDKLVQGISFSQPTCPDHMLLNSQLLGTPGSSQNPWQRLVKRMTRFFTKLDADKSYQCLKETCEKLGYQWKK  406
CHEK1-218  NLDFSPVNSASSEENVKYSSSQPEPRTGLSLWDTSPSYIDKLVQGISFSQPTCPDHMLLNSQLLGTPGSSQNPWQRLVKRMTRFFTKLDADKSYQCLKETCEKLGYQWKK  312
CHEK1-214  ..............................................................................................................  192
CHEK1-215  ..............................................................................................................  42
CHEK1-212  ..............................................................................................................  110
```

```
logo
            SCMNQVTISTTDRRNNKLIFKVNLLEMDDKILVDFRLSKGDGLEFKRHFLKIKGKLIDIVSSQKIWLPAT
AZD-7762    ......................................................................   13
PF00069.4   ......................................................................  116
PF00069.7   ......................................................................  102
PF00069.5   ......................................................................  166
PF00069.6   ......................................................................  163
PF00069.2   ......................................................................  194
PF00069.1   ......................................................................  164
PF00069.3   ......................................................................  160
PF00069     ......................................................................  254
CHEK1-204   SCMNQVTISTTDRRNNKLIFKVNLLEMDDKILVDFRLSKGDGLEFKRHFLKIKGKLIDIVSSQKIWLPAT  476
CHEK1-206   SCMNQVTISTTDRRNNKLIFKVNLLEMDDKILVDFRLSKGDGLEFKRHFLKIKGKLIDIVSSQKIWLPAT  476
CHEK1-205   SCMNQVTISTTDRRNNKLIFKVNLLEMDDKILVDFRLSKGDGLEFKRHFLKIKGKLIDIVSSQKIWLPAT   89
CHEK1-201   .....VTISTTDRRNNKLIFKVNLLEMDDKILVDFRLSKGDGLEFKRHFLKIKGKLIDIVSSQKIWLPAT  432
CHEK1-217   ......................................................................  127
CHEK1-203   SCMNQVTISTTDRRNNKLIFKVNLLEMDDKILVDFRLSKGDGLEFKRHFLKIKGKLIDIVSSQKIWLPAT  476
CHEK1-213   SCMNQ..................................GDGLEFKRHFLKIKGKLIDIVSSQKIWLPAT  442
CHEK1-208   ......................................................................  170
CHEK1-207   ......................................................................  117
CHEK1-202   SCMNQVTISTTDRRNNKLIFKVNLLEMDDKILVDFRLSKGDGLEFKRHFLKIKGKLIDIVSSQKIWLPAT  492
CHEK1-209   ......................................................................  204
CHEK1-216   SCMNQVTISTTDRRNNKLIFKVNLLEMDDKILVDFRLSKGDGLEFKRHFLKIKGKLIDIVSSQKIWLPAT  476
CHEK1-218   SCMNQVTISTTDRRNNKLIFKVNLLEMDDKILVDFRLSKGDGLEFKRHFLKIKGKLIDIVSSQKIWLPAT  382
CHEK1-214   ......................................................................  192
CHEK1-215   ......................................................................   42
CHEK1-212   ......................................................................  110
```

X non conserved
X similar
X $\geq$ 0% conserved
X $\geq$ 50% conserved

logo

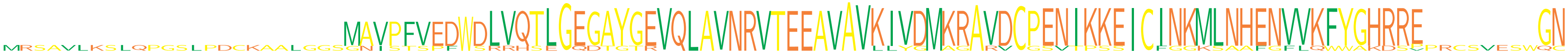

```
CHEMBL1236539  ......................................LG...Y..V............A.K.....................................V..........    7
PF00069.4      ..................................LVQTLGEGAYGEVQLAVNRVTEEAVAVKIVDMKRAVDCPENIKKEICINKMLNHENVVKFYGHRRE........GN   68
PF00069.7      .........................................................................ENIKKEICINKMLNHENVVKFYGHRRE........GN   29
PF00069.5      ..............................................................................................................    0
PF00069.6      ..............................................................................................................    0
PF00069.2      ..................................LVQTLGEGAYGEVQLAVNRVTEEAVAVKIVDMKRAVDCPENIKKEICINKMLNHENVVKFYGHRRE........GN   68
PF00069.1      ..............................................................................................................    0
PF00069.3      ..................................LVQTLGEGAYGEVQLAVNRVTEEAVAVKIVDMKRAVDCPENIKKEICINKMLNHENVVKFYGHRRE........GN   68
PF00069        ..................................LVQTLGEGAYGEVQLAVNRVTEEAVAVKIVDMKRAVDCPENIKKEICINKMLNHENVVKFYGHRRE........GN   68
CHEK1-204      ........................MAVPFVEDWDLVQTLGEGAYGEVQLAVNRVTEEAVAVKIVDMKRAVDCPENIKKEICINKMLNHENVVKFYGHRRE........GN   78
CHEK1-206      ........................MAVPFVEDWDLVQTLGEGAYGEVQLAVNRVTEEAVAVKIVDMKRAVDCPENIKKEICINKMLNHENVVKFYGHRRE........GN   78
CHEK1-205      ..............................................................................................................    0
CHEK1-201      ........................MAVPFVEDWDLVQTLGEGAYGEVQLAVNRVTEEAVAVKIVDMKRAVDCPENIKKEICINKMLNHENVVKFYGHRRE........GN   78
CHEK1-217      ........................MAVPFVEDWDLVQTLGEGAYGEVQLAVNRVTEEAVAVKIVDMKRAVDCPENIKKEICINKMLNHENVVKFYGHRRE........GN   78
CHEK1-203      ........................MAVPFVEDWDLVQTLGEGAYGEVQLAVNRVTEEAVAVKIVDMKRAVDCPENIKKEICINKMLNHENVVKFYGHRRE........GN   78
CHEK1-213      ........................MAVPFVEDWDLVQTLGEGAYGEVQLAVNRVTEEAVAVKIVDMKRAVDCPENIKKEICINKMLNHENVVKFYGHRRE........GN   78
CHEK1-208      ........................MAVPFVEDWDLVQTLGEGAYGEVQLAVNRVTEEAVAVKIVDMKRAVDCPENIKKEICINKMLNHENVVKFYGHRRE........GN   78
CHEK1-207      MRSAVLKSLQPGSLPDCKAALGGSGNISTSPFWSRRHSEGQDTGTR.............AVLLYG.AGARVCGSVTPSS..FGGKSAAFGFLQWWAKDSPPRCSVESWQC   94
CHEK1-202      MRSAVLKSLQPGSLPDCKAALGGSGNISTSPFWSRRHSEGQDTGTR.............AVLLYG.AGARVCGSVTPSS..FGGKSAAFGFLQWWAKDSPPRCSVESWQC   94
CHEK1-209      ........................MAVPFVEDWDLVQTLGEGAYGEVQLAVNRVTEEAVAVKIVDMKRAVDCPENIKKEICINKMLNHENVVKFYGHRRE........GN   78
CHEK1-216      ........................MAVPFVEDWDLVQTLGEGAYGEVQLAVNRVTEEAVAVKIVDMKRAVDCPENIKKEICINKMLNHENVVKFYGHRRE........GN   78
CHEK1-218      ..............................................................................................................    0
CHEK1-214      ..............................................................................................................    0
CHEK1-215      ........................MAVPFVEDWDLVQTLGEGAYGEVQLAVNRVTEEAVAVKIVDM............................................   42
CHEK1-212      .................................................................MKRAVDCPENIKKEICINKMLNHENVVKFYGHRRE........GN   37
```

logo

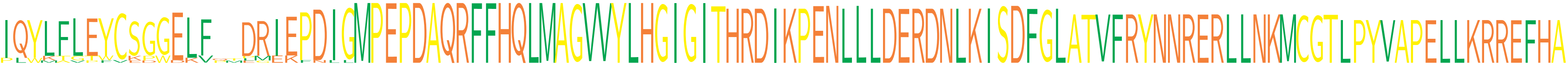

```
CHEMBL1236539  .....LEYC..GE............................................EN.L.........SD......................................   18
PF00069.4      IQYLFLEYCSGGELF..DRIEPDIGMPEPDAQRFFHQLMAGVVYLHGIGI............................................................  116
PF00069.7      IQYLFLEYCSGGELF..DRIEPDIGMPEPDAQRFFHQLMAGVVYLHGIGITHRDIKPENLLLDERDNLKISDFGL...................................  102
PF00069.5      ......................DIGMPEPDAQRFFHQLMAGVVYLHGIGITHRDIKPENLLLDERDNLKISDFGLATVFRYNNRERLLNKMCGTLPYVAPELLKRREFHA   88
PF00069.6      .........................MPEPDAQRFFHQLMAGVVYLHGIGITHRDIKPENLLLDERDNLKISDFGLATVFRYNNRERLLNKMCGTLPYVAPELLKRREFHA   85
PF00069.2      IQYLFLEYCSGGELF..DRIEPDIGMPEPDAQRFFHQLMAGVVYLHGIGITHRDIKPENLLLDERDNLKISDFGLATVFRYNNRERLLNKMCGTLPYVAPELLKRREFHA  176
PF00069.1      ........................GMPEPDAQRFFHQLMAGVVYLHGIGITHRDIKPENLLLDERDNLKISDFGLATVFRYNNRERLLNKMCGTLPYVAPELLKRREFHA   86
PF00069.3      IQYLFLEYCSGGELF..DRIEPDIGMPEPDAQRFFHQLMAGVVYLHGIGITHRDIKPENLLLDERDNLKISDFGLATVFRYNNRERLLNKMCGT................  160
PF00069        IQYLFLEYCSGGELF..DRIEPDIGMPEPDAQRFFHQLMAGVVYLHGIGITHRDIKPENLLLDERDNLKISDFGLATVFRYNNRERLLNKMCGTLPYVAPELLKRREFHA  176
CHEK1-204      IQYLFLEYCSGGELF..DRIEPDIGMPEPDAQRFFHQLMAGVVYLHGIGITHRDIKPENLLLDERDNLKISDFGLATVFRYNNRERLLNKMCGTLPYVAPELLKRREFHA  186
CHEK1-206      IQYLFLEYCSGGELF..DRIEPDIGMPEPDAQRFFHQLMAGVVYLHGIGITHRDIKPENLLLDERDNLKISDFGLATVFRYNNRERLLNKMCGTLPYVAPELLKRREFHA  186
CHEK1-205      ..............................................................................................................    0
CHEK1-201      IQYLFLEYCSGGELF..DRIEPDIGMPEPDAQRFFHQLMAGVVYLHGIGITHRDIKPENLLLDERDNLKISDFGLATVFRYNNRERLLNKMCGTLPYVAPELLKRREFHA  186
CHEK1-217      IQYLFLEYCSGGELF..DRIEPDIGMPEPDAQRFFHQLMAGVVYLHGIGIT...........................................................  127
CHEK1-203      IQYLFLEYCSGGELF..DRIEPDIGMPEPDAQRFFHQLMAGVVYLHGIGITHRDIKPENLLLDERDNLKISDFGLATVFRYNNRERLLNKMCGTLPYVAPELLKRREFHA  186
CHEK1-213      IQYLFLEYCSGGELF..DRIEPDIGMPEPDAQRFFHQLMAGVVYLHGIGITHRDIKPENLLLDERDNLKISDFGLATVFRYNNRERLLNKMCGTLPYVAPELLKRREFHA  186
CHEK1-208      IQYLFLEYCSGGELF..DRIEPDIGMPEPDAQRFFHQLMAGVVYLHGIGITHRDIKPENLLLDERDNLKISDFGLATVFRYNNRERLLNKMCGT................  170
CHEK1-207      PLWKTGTWCKPWEKVPME..KFNLL.....................................................................................  117
CHEK1-202      PLWKTGTWCKPWEKV..PMEKPDIGMPEPDAQRFFHQLMAGVVYLHGIGITHRDIKPENLLLDERDNLKISDFGLATVFRYNNRERLLNKMCGTLPYVAPELLKRREFHA  202
CHEK1-209      IQYLFLEYCSGGELF..DRIEPDIGMPEPDAQRFFHQLMAGVVYLHGIGITHRDIKPENLLLDERDNLKISDFGLATVFRYNNRERLLNKMCGTLPYVAPELLKRREFHA  186
CHEK1-216      IQYLFLEYCSGGELF..DRIEPDIGMPEPDAQRFFHQLMAGVVYLHGIGITHRDIKPENLLLDERDNLKISDFGLATVFRYNNRERLLNKMCGTLPYVAPELLKRREFHA  186
CHEK1-218      ..................MEKPDIGMPEPDAQRFFHQLMAGVVYLHGIGITHRDIKPENLLLDERDNLKISDFGLATVFRYNNRERLLNKMCGTLPYVAPELLKRREFHA   92
CHEK1-214      ...MAVPFVEDWDLVQTLGEEPDIGMPEPDAQRFFHQLMAGVVYLHGIGITHRDIKPENLLLDERDNLKISDFGLATVFRYNNRERLLNKMCGTLPYVAPELLKRREFHA  107
CHEK1-215      ..............................................................................................................   42
CHEK1-212      IQYLFLEYCSGGELF..DRIEPDIGMPEPDAQRFFHQLMAGVVYLHGIGITHRDIKPENLLLDERDNLKISDFGL...................................  110
```

```
logo            EPVDVWSCGIVLTAMLAGELPWDQPSDSCQEYSDWKEKKTYLNPWKKIDSAPLALLHKILVENPSARITIPDIKKDRWYNKPLKKGAKRPRVTSGGVSESPSGFSKHIQS
CHEMBL1236539   ..............................................................................................................  18
PF00069.4       ..............................................................................................................  116
PF00069.7       ..............................................................................................................  102
PF00069.5       EPVDVWSCGIVLTAMLAGELPWDQPSDSCQEYSDWKEKKTYLNPWKKIDSAPLALLHKILVENPSARITIPDIKKDRW................................  166
PF00069.6       EPVDVWSCGIVLTAMLAGELPWDQPSDSCQEYSDWKEKKTYLNPWKKIDSAPLALLHKILVENPSARITIPDIKKDRW................................  163
PF00069.2       EPVDVWSCGIVLTAMLAG............................................................................................  194
PF00069.1       EPVDVWSCGIVLTAMLAGELPWDQPSDSCQEYSDWKEKKTYLNPWKKIDSAPLALLHKILVENPSARITIPDIKKDRW................................  164
PF00069.3       ..............................................................................................................  160
PF00069         EPVDVWSCGIVLTAMLAGELPWDQPSDSCQEYSDWKEKKTYLNPWKKIDSAPLALLHKILVENPSARITIPDIKKDRW................................  254
CHEK1-204       EPVDVWSCGIVLTAMLAGELPWDQPSDSCQEYSDWKEKKTYLNPWKKIDSAPLALLHKILVENPSARITIPDIKKDRWYNKPLKKGAKRPRVTSGGVSESPSGFSKHIQS  296
CHEK1-206       EPVDVWSCGIVLTAMLAGELPWDQPSDSCQEYSDWKEKKTYLNPWKKIDSAPLALLHKILVENPSARITIPDIKKDRWYNKPLKKGAKRPRVTSGGVSESPSGFSKHIQS  296
CHEK1-205       ..............................................................................................................  0
CHEK1-201       EPVDVWSCGIVLTAMLAGELPWDQPSDSCQEYSDWKEKKTYLNPWKKIDSAPLALLHKILVENPSARITIPDIKKDRWYNKPLKKGAKRPRVTSGGVSESPSGFSKHIQS  296
CHEK1-217       ..............................................................................................................  127
CHEK1-203       EPVDVWSCGIVLTAMLAGELPWDQPSDSCQEYSDWKEKKTYLNPWKKIDSAPLALLHKILVENPSARITIPDIKKDRWYNKPLKKGAKRPRVTSGGVSESPSGFSKHIQS  296
CHEK1-213       EPVDVWSCGIVLTAMLAGELPWDQPSDSCQEYSDWKEKKTYLNPWKKIDSAPLALLHKILVENPSARITIPDIKKDRWYNKPLKKGAKRPRVTSGGVSESPSGFSKHIQS  296
CHEK1-208       ..............................................................................................................  170
CHEK1-207       ..............................................................................................................  117
CHEK1-202       EPVDVWSCGIVLTAMLAGELPWDQPSDSCQEYSDWKEKKTYLNPWKKIDSAPLALLHKILVENPSARITIPDIKKDRWYNKPLKKGAKRPRVTSGGVSESPSGFSKHIQS  312
CHEK1-209       EPVDVWSCGIVLTAMLAG............................................................................................  204
CHEK1-216       EPVDVWSCGIVLTAMLAGELPWDQPSDSCQEYSDWKEKKTYLNPWKKIDSAPLALLHKILVENPSARITIPDIKKDRWYNKPLKKGAKRPRVTSGGVSESPSGFSKHIQS  296
CHEK1-218       EPVDVWSCGIVLTAMLAGELPWDQPSDSCQEYSDWKEKKTYLNPWKKIDSAPLALLHKILVENPSARITIPDIKKDRWYNKPLKKGAKRPRVTSGGVSESPSGFSKHIQS  202
CHEK1-214       EPVDVWSCGIVLTAMLAGELPWDQPSDSCQEYSDWKEKKTYLNPWKKIDSAPLALLHKILVENPSARITIPDIKKDRWYNKPLKK.........................  192
CHEK1-215       ..............................................................................................................  42
CHEK1-212       ..............................................................................................................  110

logo            NLDFSPVNSASSEENVKYSSSQPEPRTGLSLWDTSPSYIDKLVQGISFSQPTCPDHMLLNSQLLGTPGSSQNPWQRLVKRMTRFFTKLDADKSYQCLKETCEKLGYQWKK
CHEMBL1236539   ..............................................................................................................  18
PF00069.4       ..............................................................................................................  116
PF00069.7       ..............................................................................................................  102
PF00069.5       ..............................................................................................................  166
PF00069.6       ..............................................................................................................  163
PF00069.2       ..............................................................................................................  194
PF00069.1       ..............................................................................................................  164
PF00069.3       ..............................................................................................................  160
PF00069         ..............................................................................................................  254
CHEK1-204       NLDFSPVNSASSEENVKYSSSQPEPRTGLSLWDTSPSYIDKLVQGISFSQPTCPDHMLLNSQLLGTPGSSQNPWQRLVKRMTRFFTKLDADKSYQCLKETCEKLGYQWKK  406
CHEK1-206       NLDFSPVNSASSEENVKYSSSQPEPRTGLSLWDTSPSYIDKLVQGISFSQPTCPDHMLLNSQLLGTPGSSQNPWQRLVKRMTRFFTKLDADKSYQCLKETCEKLGYQWKK  406
CHEK1-205       ...........................................................................................KSYQCLKETCEKLGYQWKK  19
CHEK1-201       NLDFSPVNSASSEENVKYSSSQPEPRTGLSLWDTSPSYIDKLVQGISFSQPTCPDHMLLNSQLLGTPGSSQ.......................................  367
CHEK1-217       ..............................................................................................................  127
CHEK1-203       NLDFSPVNSASSEENVKYSSSQPEPRTGLSLWDTSPSYIDKLVQGISFSQPTCPDHMLLNSQLLGTPGSSQNPWQRLVKRMTRFFTKLDADKSYQCLKETCEKLGYQWKK  406
CHEK1-213       NLDFSPVNSASSEENVKYSSSQPEPRTGLSLWDTSPSYIDKLVQGISFSQPTCPDHMLLNSQLLGTPGSSQNPWQRLVKRMTRFFTKLDADKSYQCLKETCEKLGYQWKK  406
CHEK1-208       ..............................................................................................................  170
CHEK1-207       ..............................................................................................................  117
CHEK1-202       NLDFSPVNSASSEENVKYSSSQPEPRTGLSLWDTSPSYIDKLVQGISFSQPTCPDHMLLNSQLLGTPGSSQNPWQRLVKRMTRFFTKLDADKSYQCLKETCEKLGYQWKK  422
CHEK1-209       ..............................................................................................................  204
CHEK1-216       NLDFSPVNSASSEENVKYSSSQPEPRTGLSLWDTSPSYIDKLVQGISFSQPTCPDHMLLNSQLLGTPGSSQNPWQRLVKRMTRFFTKLDADKSYQCLKETCEKLGYQWKK  406
CHEK1-218       NLDFSPVNSASSEENVKYSSSQPEPRTGLSLWDTSPSYIDKLVQGISFSQPTCPDHMLLNSQLLGTPGSSQNPWQRLVKRMTRFFTKLDADKSYQCLKETCEKLGYQWKK  312
CHEK1-214       ..............................................................................................................  192
CHEK1-215       ..............................................................................................................  42
CHEK1-212       ..............................................................................................................  110
```

```
logo
                SCMNQVTISTTDRRNNKLIFKVNLLEMDDKILVDFRLSKGDGLEFKRHFLKIKGKLIDIVSSQKIWLPAT
CHEMBL1236539   ......................................................................   18
PF00069.4       ......................................................................  116
PF00069.7       ......................................................................  102
PF00069.5       ......................................................................  166
PF00069.6       ......................................................................  163
PF00069.2       ......................................................................  194
PF00069.1       ......................................................................  164
PF00069.3       ......................................................................  160
PF00069         ......................................................................  254
CHEK1-204       SCMNQVTISTTDRRNNKLIFKVNLLEMDDKILVDFRLSKGDGLEFKRHFLKIKGKLIDIVSSQKIWLPAT  476
CHEK1-206       SCMNQVTISTTDRRNNKLIFKVNLLEMDDKILVDFRLSKGDGLEFKRHFLKIKGKLIDIVSSQKIWLPAT  476
CHEK1-205       SCMNQVTISTTDRRNNKLIFKVNLLEMDDKILVDFRLSKGDGLEFKRHFLKIKGKLIDIVSSQKIWLPAT   89
CHEK1-201       .....VTISTTDRRNNKLIFKVNLLEMDDKILVDFRLSKGDGLEFKRHFLKIKGKLIDIVSSQKIWLPAT  432
CHEK1-217       ......................................................................  127
CHEK1-203       SCMNQVTISTTDRRNNKLIFKVNLLEMDDKILVDFRLSKGDGLEFKRHFLKIKGKLIDIVSSQKIWLPAT  476
CHEK1-213       SCMNQ..................................GDGLEFKRHFLKIKGKLIDIVSSQKIWLPAT  442
CHEK1-208       ......................................................................  170
CHEK1-207       ......................................................................  117
CHEK1-202       SCMNQVTISTTDRRNNKLIFKVNLLEMDDKILVDFRLSKGDGLEFKRHFLKIKGKLIDIVSSQKIWLPAT  492
CHEK1-209       ......................................................................  204
CHEK1-216       SCMNQVTISTTDRRNNKLIFKVNLLEMDDKILVDFRLSKGDGLEFKRHFLKIKGKLIDIVSSQKIWLPAT  476
CHEK1-218       SCMNQVTISTTDRRNNKLIFKVNLLEMDDKILVDFRLSKGDGLEFKRHFLKIKGKLIDIVSSQKIWLPAT  382
CHEK1-214       ......................................................................  192
CHEK1-215       ......................................................................   42
CHEK1-212       ......................................................................  110
```

X non conserved
X similar
X ≥0% conserved
X ≥50% conserved

```
logo
             MGPGVLLLLLVATAWHGQGIPVIEPSVPELVVKPGATVTLRCVGNGSVEWDGPPSPHWTLYSDGSSSILSTNNATFQNTGTYRCTEPGDPLGGSAAIHLYVKDPARPWNV
IMATINIB     ..............................................................................................................     0
PF00047      ..............................................................................................................     0
PF13927      ..............................................................................................................     0
PF07714      ..............................................................................................................     0
CSF1R-201    MGPGVLLLLLVATAWHGQGIPVIEPSVPELVVKPGATVTLRCVGNGSVEWDGPPSPHWTLYSDGSSSILSTNNATFQNTGTYRCTEPGDPLGGSAAIHLYVKDPARPWNV   110
CSF1R-205    ..............................................................................................................     0
CSF1R-203    MGPGVLLLLLVATAWHGQGIPVIEPSVPELVVKPGATVTLRCVGNGSVEWDGPPSPHWTLYSDGSSSILSTNNATFQNTGTYRCTEPGDPLGGSAAIHLYVKDPARPWNV   110
CSF1R-209    MGPGVLLLLLVATAWHGQGIPVIEPSVPELVVKPGATVTLRCVGNGSVEWDGPPSPHWTLYSDGSSSILSTNNATFQNTGTYRCTEPGDPLGGSAAIHLYVKDPARPWNV   110
CSF1R-207    ..............................................................................................................     0

logo
             LAQEVVVFEDQDALLPCLLTDPVLEAGVSLVRVRGRPLMRHTNYSFSPWHGFTIHRAKFIQSQDYQCSALMGGRKVMSISIRLKVQKVIPGPPALTLVPAELVRIRGEAA
IMATINIB     ..............................................................................................................     0
PF00047      ..................................................................................................PAELVRIRGEAA    12
PF13927      ..............................................................................................................     0
PF07714      ..............................................................................................................     0
CSF1R-201    LAQEVVVFEDQDALLPCLLTDPVLEAGVSLVRVRGRPLMRHTNYSFSPWHGFTIHRAKFIQSQDYQCSALMGGRKVMSISIRLKVQKVIPGPPALTLVPAELVRIRGEAA   220
CSF1R-205    ......................................MRHTNYSFSPWHGFTIHRAKFIQSQDYQCSALMGGRKVMSISIRLKVQK.......................    49
CSF1R-203    LAQEVVVFEDQDALLPCLLTDPVLEAGVSLVRVRGRPLMRHTNYSFSPWHGFTIHRAKFIQSQDYQCSALMGGRKVMSISIRLKVQKVIPGPPALTLVPAELVRIRGEAA   220
CSF1R-209    LAQEVVVFEDQDALLPCLLTDPVLEAGVSLVRVRGRPLMRHTNYSFSPWHGFTIHRAKFIQSQDYQCSALMGGRKVMSISIRLKVQKVIPGPPALTLVPAELVRIRGEAA   220
CSF1R-207    ..............................................................................................................     0

logo
             QIVCSASSVDVNFDVFLQHNNTKLAIPQQSDFHNNRYQKVLTLNLDQVDFQHAGNYSCVASNVQGKHSTSMFFRVVESAYLNLSSEQNLIQEVTVGEGLNLKVMVEAYPG
IMATINIB     ..............................................................................................................     0
PF00047      QIVCSASSVDVNFDVFLQHNNTKLAIPQQSDFHNNRYQKVLTLNLDQVDFQHAGNYSCVASNVQGKHSTSMF......................................    84
PF13927      ..............................................................................................................     0
PF07714      ..............................................................................................................     0
CSF1R-201    QIVCSASSVDVNFDVFLQHNNTKLAIPQQSDFHNNRYQKVLTLNLDQVDFQHAGNYSCVASNVQGKHSTSMFFRVVESAYLNLSSEQNLIQEVTVGEGLNLKVMVEAYPG   330
CSF1R-205    ..............................................................................................................    49
CSF1R-203    QIVCSASSVDVNFDVFLQHNNTKLAIPQQSDFHNNRYQKVLTLNLDQVDFQHAGNYSCVASNVQGKHSTSMFFRVVESAYLNLSSEQNLIQEVTVGEGLNLKVMVEAYPG   330
CSF1R-209    QIVCSASSVDVNFDVFLQHNNTKLAIPQQSDFHNNRYQKVLTLNLDQVDFQHAGNYSCVASNVQGKHSTSMFFRVVGTPSPSLCPA........................   306
CSF1R-207    ..............................................................................................................     0

logo
             LQGFNWTYLGPFSDHQPEPKLANATTKDTYRHTFTLSLPRLKPSEAGRYSFLARNPGGWRALTFELTLRYPPEVSVIWTFINGSGTLLCAASGYPQPNVTWLQCSGHTDR
IMATINIB     ..............................................................................................................     0
PF00047      ..............................................................................................................    84
PF13927      ..............................................................................TFINGSGTLLCAASGYPQPNVTWLQCSGHTDR    32
PF07714      ..............................................................................................................     0
CSF1R-201    LQGFNWTYLGPFSDHQPEPKLANATTKDTYRHTFTLSLPRLKPSEAGRYSFLARNPGGWRALTFELTLRYPPEVSVIWTFINGSGTLLCAASGYPQPNVTWLQCSGHTDR   440
CSF1R-205    ..............................................................................................................    49
CSF1R-203    LQGFNWTYLGPFSDHQPEPKLANATTKDTYRHTFTLSLPRLKPSEAGRYSFLARNPGGWRALTFELTLRYPPEVSVIWTFINGSGTLLCAASGYPQPNVTWLQCSGHTDR   440
CSF1R-209    ..............................................................................................................   306
CSF1R-207    ..............................................................................................................     0
```

logo

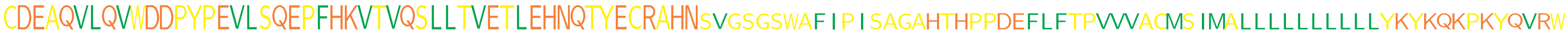

```
IMATINIB   ..............................................................................................................   0
PF00047    ..............................................................................................................  84
PF13927    CDEAQVLQVWDDPYPEVLSQEPFHKVTVQSLLTVETLEHNQTYECRAHN.............................................................  81
PF07714    ..............................................................................................................   0
CSF1R-201  CDEAQVLQVWDDPYPEVLSQEPFHKVTVQSLLTVETLEHNQTYECRAHNSVGSGSWAFIPISAGAHTHPPDEFLFTPVVVACMSIMALLLLLLLLLLYKYKQKPKYQVRW 550
CSF1R-205  ..............................................................................................................  49
CSF1R-203  CDEAQVLQVWDDPYPEVLSQEPFHKVTVQSLLTVETLEHNQTYECRAHNSVGSGSWAFIPISAGAHTHPPDEFLFTPVVVACMSIMALLLLLLLLLLYKYKQKPKYQVRW 550
CSF1R-209  .............................................................................................................. 306
CSF1R-207  ..............................................................................................................   0
```

logo

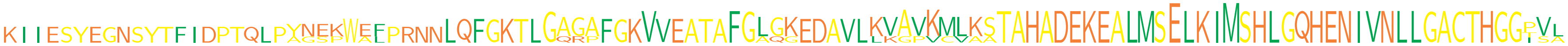

```
IMATINIB   .............................................V.................A.K................E...M.......................   5
PF00047    ..............................................................................................................  84
PF13927    ..............................................................................................................  81
PF07714    ...............................LQFGKTLGAGAFGKVVEATAFGLGKEDAVLKVAVKMLKSTAHADEKEALMSELKIMSHLGQHENIVNLLGACTHGGPVL  79
CSF1R-201  KIIESYEGNSYTFIDPTQLPYNEKWEFPRNNLQFGKTLGAGAFGKVVEATAFGLGKEDAVLKVAVKMLKSTAHADEKEALMSELKIMSHLGQHENIVNLLGACTHGGPVL 660
CSF1R-205  ..............................................................................................................  49
CSF1R-203  KIIESYEGNSYTFIDPTQLPYNEKWEFPRNNLQFGKTLGAGAFGKVVEATAFGLGKEDAVLKVAVKMLKSTAHADEKEALMSELKIMSHLGQHENIVNLLGACTHGGTVA 660
CSF1R-209  .............................................................................................................. 306
CSF1R-207  ....................AGSPWAL...........GQRP.........FGAQG.....LKGPVCVAATAHADEKEALMSELKIMSHLGQHENIVNLLGACTHGGIS.  64
```

logo

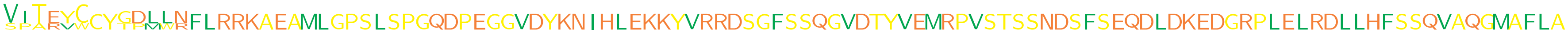

```
IMATINIB   V.T..C........................................................................................................   8
PF00047    ..............................................................................................................  84
PF13927    ..............................................................................................................  81
PF07714    VITEYCCYGDLLNFLRRKAEAMLGPSLSPGQDPEGGVDYKNIHLEKKYVRRDSGFSSQGVDTYVEMRPVSTSSNDSFSEQDLDKEDGRPLELRDLLHFSSQVAQGMAFLA 189
CSF1R-201  VITEYCCYGDLLNFLRRKAEAMLGPSLSPGQDPEGGVDYKNIHLEKKYVRRDSGFSSQGVDTYVEMRPVSTSSNDSFSEQDLDKEDGRPLELRDLLHFSSQVAQGMAFLA 770
CSF1R-205  ..............................................................................................................  49
CSF1R-203  SPARVW..TPMWR................................................................................................. 671
CSF1R-209  .............................................................................................................. 306
CSF1R-207  ..............................................................................................................  64
```

logo

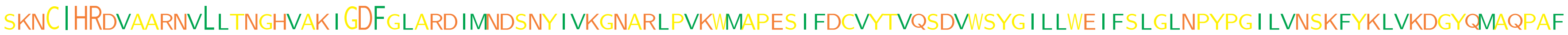

```
IMATINIB   ...CIHR.......L.........GDF...................................................................................  16
PF00047    ..............................................................................................................  84
PF13927    ..............................................................................................................  81
PF07714    SKNCIHRDVAARNVLLTNGHVAKIGDFGLARDIMNDSNYIVKGNARLPVKWMAPESIFDCVYTVQSDVWSYGILLWEIFSLGLNPYPGILVNSKFYKLVKDGYQMAQPAF 299
CSF1R-201  SKNCIHRDVAARNVLLTNGHVAKIGDFGLARDIMNDSNYIVKGNARLPVKWMAPESIFDCVYTVQSDVWSYGILLWEIFSLGLNPYPGILVNSKFYKLVKDGYQMAQPAF 880
CSF1R-205  ..............................................................................................................  49
CSF1R-203  .............................................................................................................. 671
CSF1R-209  .............................................................................................................. 306
CSF1R-207  ..............................................................................................................  64
```

logo

```
           APKNIYSIMQACWALEPTHRPTFQQICSFLQEQAQEDRRERDYTNLPSSSRSGGSGSSSSELEEESSSEHLTCCEQGDIAQPLLQPNNYQFC
IMATINIB   ............................................................................................    16
PF00047    ............................................................................................    84
PF13927    ............................................................................................    81
PF07714    APKNIYSIMQACWALEPTHRPTFQQICSFL..............................................................   329
CSF1R-201  APKNIYSIMQACWALEPTHRPTFQQICSFLQEQAQEDRRERDYTNLPSSSRSGGSGSSSSELEEESSSEHLTCCEQGDIAQPLLQPNNYQFC   972
CSF1R-205  ............................................................................................    49
CSF1R-203  ............................................................................................   671
CSF1R-209  ............................................................................................   306
CSF1R-207  ............................................................................................    64
```

X non conserved
X similar
X $\geq 0\%$ conserved
X $\geq 50\%$ conserved

logo

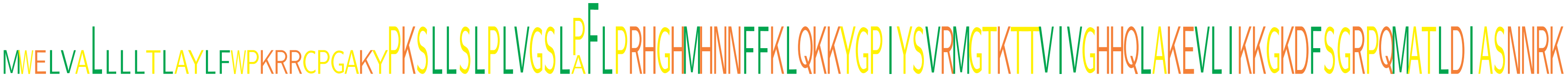

```
ABIRATERONE  ......L.................................AF....................................................................   3
PF00067      ...........................PKSLLSLPLVGSLPFLPRHGHMHNNFFKLQKKYGPIYSVRMGTKTTVIVGHHQLAKEVLIKKGKDFSGRPQMATLDIASNNRK  83
CYP17A1-201  MWELVALLLLTLAYLFWPKRRCPGAKYPKSLLSLPLVGSLPFLPRHGHMHNNFFKLQKKYGPIYSVRMGTKTTVIVGHHQLAKEVLIKKGKDFSGRPQMATLDIASNNRK 110
```

logo

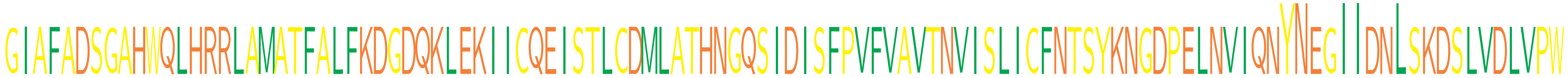

```
ABIRATERONE  ..........................................................................................YN..II..L...........   8
PF00067      GIAFADSGAHWQLHRRLAMATFALFKDGDQKLEKIICQEISTLCDMLATHNGQSIDISFPVFVAVTNVISLICFNTSYKNGDPELNVIQNYNEGIIDNLSKDSLVDLVPW 193
CYP17A1-201  GIAFADSGAHWQLHRRLAMATFALFKDGDQKLEKIICQEISTLCDMLATHNGQSIDISFPVFVAVTNVISLICFNTSYKNGDPELNVIQNYNEGIIDNLSKDSLVDLVPW 220
```

logo

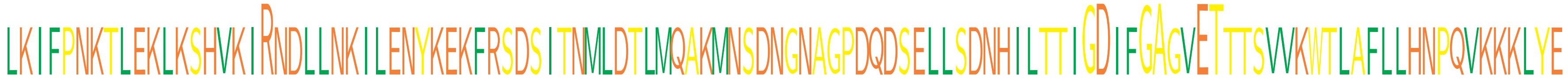

```
ABIRATERONE  ..................R.........................................................GD..GA..ET........................  15
PF00067      LKIFPNKTLEKLKSHVKIRNDLLNKILENYKEKFRSDSITNMLDTLMQAKMNSDNGNAGPDQDSELLSDNHILTTIGDIFGAGVETTTSVVKWTLAFLLHNPQVKKKLYE 303
CYP17A1-201  LKIFPNKTLEKLKSHVKIRNDLLNKILENYKEKFRSDSITNMLDTLMQAKMNSDNGNAGPDQDSELLSDNHILTTIGDIFGAGVETTTSVVKWTLAFLLHNPQVKKKLYE 330
```

logo

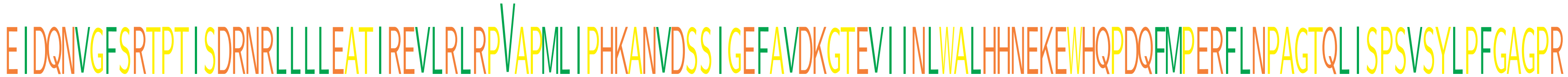

```
ABIRATERONE  ...................................V..........................................................................  16
PF00067      EIDQNVGFSRTPTISDRNRLLLLEATIREVLRLRPVAPMLIPHKANVDSSIGEFAVDKGTEVIINLWALHHNEKEWHQPDQFMPERFLNPAGTQLISPSVSYLPFGAGPR 413
CYP17A1-201  EIDQNVGFSRTPTISDRNRLLLLEATIREVLRLRPVAPMLIPHKANVDSSIGEFAVDKGTEVIINLWALHHNEKEWHQPDQFMPERFLNPAGTQLISPSVSYLPFGAGPR 440
```

logo

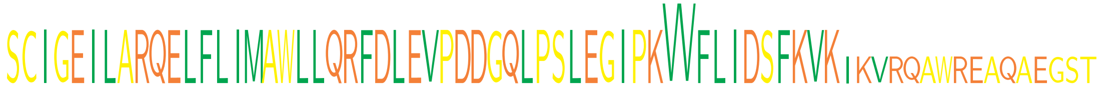

```
ABIRATERONE  .........................................VV.........................   18
PF00067      SCIGEILARQELFLIMAWLLQRFDLEVPDDGQLPSLEGIPKVVFLIDSFKVK................  465
CYP17A1-201  SCIGEILARQELFLIMAWLLQRFDLEVPDDGQLPSLEGIPKVVFLIDSFKVKIKVRQAWREAQAEGST  508
```

X non conserved
X similar
X $\geq 0\%$ conserved
X $\geq 50\%$ conserved

logo

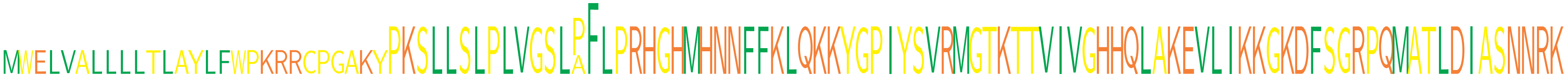

```
GALETERONE    ........................................AF....................................................................     2
PF00067       ...........................PKSLLSLPLVGSLPFLPRHGHMHNNFFKLQKKYGPIYSVRMGTKTTVIVGHHQLAKEVLIKKGKDFSGRPQMATLDIASNNRK    83
CYP17A1-201   MWELVALLLLTLAYLFWPKRRCPGAKYPKSLLSLPLVGSLPFLPRHGHMHNNFFKLQKKYGPIYSVRMGTKTTVIVGHHQLAKEVLIKKGKDFSGRPQMATLDIASNNRK   110
```

logo

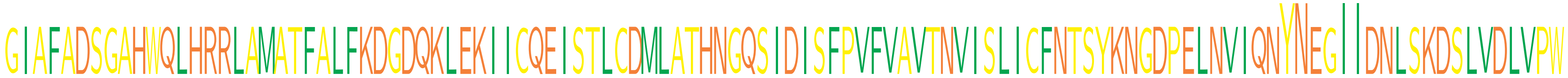

```
GALETERONE    ..........................................................................................YN..II..............     6
PF00067       GIAFADSGAHWQLHRRLAMATFALFKDGDQKLEKIICQEISTLCDMLATHNGQSIDISFPVFVAVTNVISLICFNTSYKNGDPELNVIQNYNEGIIDNLSKDSLVDLVPW   193
CYP17A1-201   GIAFADSGAHWQLHRRLAMATFALFKDGDQKLEKIICQEISTLCDMLATHNGQSIDISFPVFVAVTNVISLICFNTSYKNGDPELNVIQNYNEGIIDNLSKDSLVDLVPW   220
```

logo

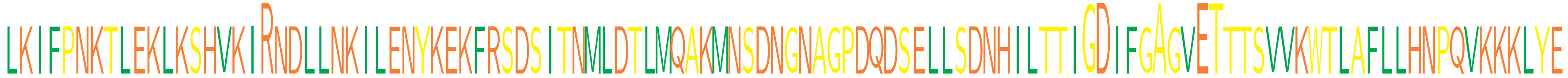

```
GALETERONE    ..................R..........................................................GD....A.ET........................    12
PF00067       LKIFPNKTLEKLKSHVKIRNDLLNKILENYKEKFRSDSITNMLDTLMQAKMNSDNGNAGPDQDSELLSDNHILTTIGDIFGAGVETTTSVVKWTLAFLLHNPQVKKKLYE   303
CYP17A1-201   LKIFPNKTLEKLKSHVKIRNDLLNKILENYKEKFRSDSITNMLDTLMQAKMNSDNGNAGPDQDSELLSDNHILTTIGDIFGAGVETTTSVVKWTLAFLLHNPQVKKKLYE   330
```

logo

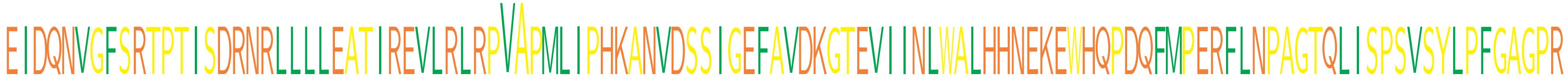

```
GALETERONE    ...................................VA.........................................................................    14
PF00067       EIDQNVGFSRTPTISDRNRLLLLEATIREVLRLRPVAPMLIPHKANVDSSIGEFAVDKGTEVIINLWALHHNEKEWHQPDQFMPERFLNPAGTQLISPSVSYLPFGAGPR   413
CYP17A1-201   EIDQNVGFSRTPTISDRNRLLLLEATIREVLRLRPVAPMLIPHKANVDSSIGEFAVDKGTEVIINLWALHHNEKEWHQPDQFMPERFLNPAGTQLISPSVSYLPFGAGPR   440
```

logo

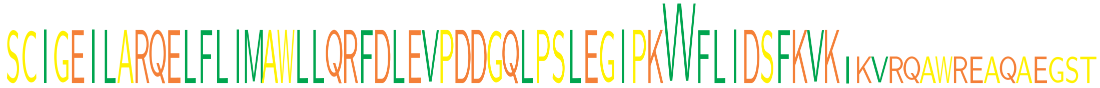

```
GALETERONE    .........................................VV.........................    16
PF00067       SCIGEILARQELFLIMAWLLQRFDLEVPDDGQLPSLEGIPKVVFLIDSFKVK................   465
CYP17A1-201   SCIGEILARQELFLIMAWLLQRFDLEVPDDGQLPSLEGIPKVVFLIDSFKVKIKVRQAWREAQAEGST   508
```

- X non conserved
- X similar
- X $\geq$0% conserved
- X $\geq$50% conserved

```
logo
ORTERONEL    ............A.................................................................................................    1
PF00067      ...........................PKSLLSLPLVGSLPFLPRHGHMHNNFFKLQKKYGPIYSVRMGTKTTVIVGHHQLAKEVLIKKGKDFSGRPQMATLDIASNNRK   83
CYP17A1-201  MWELVALLLLTLAYLFWPKRRCPGAKYPKSLLSLPLVGSLPFLPRHGHMHNNFFKLQKKYGPIYSVRMGTKTTVIVGHHQLAKEVLIKKGKDFSGRPQMATLDIASNNRK  110

logo
ORTERONEL    ....................................................................I.........................................    2
PF00067      GIAFADSGAHWQLHRRLAMATFALFKDGDQKLEKIICQEISTLCDMLATHNGQSIDISFPVFVAVTNVISLICFNTSYKNGDPELNVIQNYNEGIIDNLSKDSLVDLVPW  193
CYP17A1-201  GIAFADSGAHWQLHRRLAMATFALFKDGDQKLEKIICQEISTLCDMLATHNGQSIDISFPVFVAVTNVISLICFNTSYKNGDPELNVIQNYNEGIIDNLSKDSLVDLVPW  220

logo
ORTERONEL    ..................R......................................................T..GD..GA...T........................    9
PF00067      LKIFPNKTLEKLKSHVKIRNDLLNKILENYKEKFRSDSITNMLDTLMQAKMNSDNGNAGPDQDSELLSDNHILTTTIGDIFGAGVETTTSVVKWTLAFLLHNPQVKKKLYE  303
CYP17A1-201  LKIFPNKTLEKLKSHVKIRNDLLNKILENYKEKFRSDSITNMLDTLMQAKMNSDNGNAGPDQDSELLSDNHILTTTIGDIFGAGVETTTSVVKWTLAFLLHNPQVKKKLYE  330

logo
ORTERONEL    ...................................V..........................................................................   10
PF00067      EIDQNVGFSRTPTISDRNRLLLLEATIREVLRLRPVAPMLIPHKANVDSSIGEFAVDKGTEVIINLWALHHNEKEWHQPDQFMPERFLNPAGTQLISPSVSYLPFGAGPR  413
CYP17A1-201  EIDQNVGFSRTPTISDRNRLLLLEATIREVLRLRPVAPMLIPHKANVDSSIGEFAVDKGTEVIINLWALHHNEKEWHQPDQFMPERFLNPAGTQLISPSVSYLPFGAGPR  440

logo
ORTERONEL    .........................................VV.........................   12
PF00067      SCIGEILARQELFLIMAWLLQRFDLEVPDDGQLPSLEGIPKVVFLIDSFKVK................  465
CYP17A1-201  SCIGEILARQELFLIMAWLLQRFDLEVPDDGQLPSLEGIPKVVFLIDSFKVKIKVRQAWREAQAEGST  508
```

X non conserved

X similar

X ≥ 0% conserved

X ≥ 50% conserved

logo

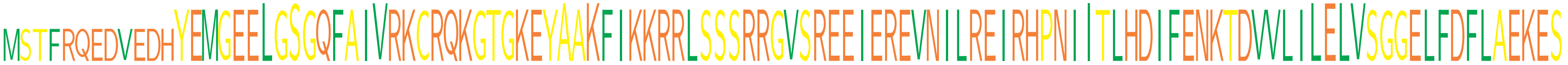

```
CHEMBL21156   ..................L.S.....V............A.K..................................I...............LELV..............   10
PF00069.2     ..............................................................................................................    0
PF00069.3     ............YEMGEELGSGQFAIVRKCRQKGTGKEYAAKFIKKRRLSSSRRGVSREEIEREVNILREIRHPNIITLHDIFENKTDVVLILELVSGGELFDFLAEKES   98
PF00069       ............YEMGEELGSGQFAIVRKCRQKGTGKEYAAKFIKKRRLSSSRRGVSREEIEREVNILREIRHPNIITLHDIFENKTDVVLILELVSGGELFDFLAEKES   98
PF00069.1     ............YEMGEELGSGQFAIVRKCRQKGTGKEYAAKFIKKRRLSSSRRGVSREEIEREVNILREIRHPNIITLHDIFENKTDVVLILELVSGGELFDFLAEKES   98
DAPK3-203     MSTFRQEDVEDHYEMGEELGSGQFAIVRKCRQKGTGKEYAAKFIKKRRLSSSRRGVSREEIEREVNILREIRHPNIITLHDIFENKTDVVLILELVSGGELFDFLAEKES  110
DAPK3-206     MSTFRQEDVEDHYEMGEELGSGQFAIVRKCRQKGTGKEYAAKFIKKRRLSSSRRGVSREEIEREVNILREIRHPNIITLHDIFENKTDVVLILELVSGGELFDFLAEKES  110
DAPK3-202     MSTFRQEDVEDHYEMGEELGSGQFAIVRKCRQKGTGKEYAAKFIKKRRLSSSRRGVSREEIEREVNILREIRHPNIITLHDIFENKTDVVLILELVSGGELFDFLAEKES  110
DAPK3-204     ..............................................................................................................    0
DAPK3-207     MSTFRQEDVEDHYEMGEELGSGQFAIVRKCRQKGTGKEYAAKFIKKRRLSSSRRGVSREEIEREVNILREIRHPNIITLHDIFENKTDVVLILELVSGGELFDFLAEKES  110
DAPK3-201     MSTFRQEDVEDHYEMGEELGSGQFAIVRKCRQKGTGKEYAAKFIKKRRLSSSRRGVSREEIEREVNILREIRHPNIITLHDIFENKTDVVLILELVSGGELFDFLAEKES  110
```

logo

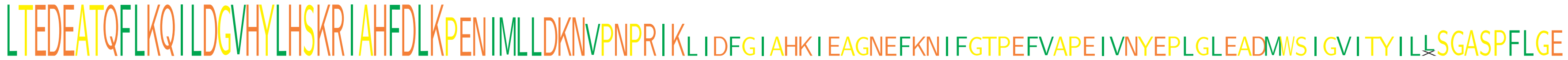

```
CHEMBL21156   ..............................................................................................................   10
PF00069.2     ..............................................................................................................    0
PF00069.3     LTEDEATQFLKQILDGVHYLHSKRIAHFDLKPENIMLLDKN.....................................................................  139
PF00069       LTEDEATQFLKQILDGVHYLHSKRIAHFDLKPENIMLLDKNVPNPRIKLIDFGIAHKIEAGNEFKNIFGTPEFVAPEIVNYEPLGLEADMWSIGVITYILLSGASPFLGE  208
PF00069.1     LTEDEATQFLKQILDGVHYLHSKRIAHFDLK...............................................................................  129
DAPK3-203     LTEDEATQFLKQILDGVHYLHSKRIAHFDLK...............................................................................  141
DAPK3-206     LTEDEATQFLKQILDGVHYLHSKRIAHFDLKPENIMLLDKNVPNPRIK..............................................................  158
DAPK3-202     LTEDEATQFLKQILDGVHYLHSKRIAHFDLKPENIMLLDKNVPNPRIKLIDFGIAHKIEAGNEFKNIFGTPEFVAPEIVNYEPLGLEADMWSIGVITYILLSGASPFLGE  220
DAPK3-204     ....................................................................................................XSGASPFLGE   10
DAPK3-207     LTEDEATQFLKQILDGVHYLHSKRIAHFDLKPENIMLLDKNVPNPRIK..............................................................  158
DAPK3-201     LTEDEATQFLKQILDGVHYLHSKRIAHFDLKPENIMLLDKNVPNPRIKLIDFGIAHKIEAGNEFKNIFGTPEFVAPEIVNYEPLGLEADMWSIGVITYILLSGASPFLGE  220
```

logo

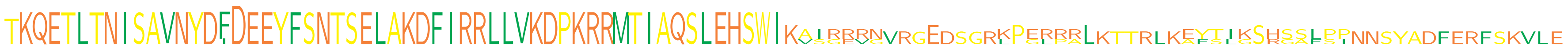

```
CHEMBL21156   ...............ID.............................................................................................   12
PF00069.2     .KQETLTNISAVNYDFDEEYFSNTSELAKDFIRRLLVKDPKRRMTIAQSLEHSWI.......................................................   54
PF00069.3     ..............................................................................................................  139
PF00069       TKQETLTNISAVNYDFDEEYFSNTSELAKDFIRRLLVKDPKRRMTIAQSLEHSWI.......................................................  263
PF00069.1     ..............................................................................................................  129
DAPK3-203     ..............................................................................................................  141
DAPK3-206     ..............................................................................................................  158
DAPK3-202     TKQETLTNISAVNYDFDEEYFSNTSELAKDFIRRLLVKDPKRRMTIAQSLEHSWIKAIRRRNVRGEDSGRKPERRRLKTTRLKEYTIKSHSSLPPNNSYADFERFSKVLE  330
DAPK3-204     TKQETLTNISAVNYDFDEEYFSNTSELAKDFIRRLLVKDPKRRMTIAQSLEHSWIKVSGEVG...E....LPGLPAL......AFSLGSRGAFSI...............   92
DAPK3-207     ..............................................................................................................  158
DAPK3-201     TKQETLTNISAVNYDFDEEYFSNTSELAKDFIRRLLVKDPKRRMTIAQSLEHSWIKAIRRRNVRGEDSGRKPERRRLKTTRLKEYTIKSHSSLPPNNSYADFERFSKVLE  330
```

```
logo
             EAAAAEEGLRELQRSRRLCHEDVEALAAIYEEKEAWYREESDSLGQDLRRLRQELLKTEALKRQAQEEAKGALLGTSGLKRRFSRLENRYEALAKQVASEMRFVQDLVRA
CHEMBL21156  ..............................................................................................................   12
PF00069.2    ..............................................................................................................   54
PF00069.3    ..............................................................................................................  139
PF00069      ..............................................................................................................  263
PF00069.1    ..............................................................................................................  129
DAPK3-203    ..............................................................................................................  141
DAPK3-206    ..............................................................................................................  158
DAPK3-202    EAAAAEEGLRELQRSRRLCHEDVEALAAIYEEKEAWYREESDSLGQDLRRLRQELLKTEALKRQAQEEAKGALLGTSGLKRRFSRLENRYEALAKQVASEMRFVQDLVRA  440
DAPK3-204    ..............................................................................................................   92
DAPK3-207    ..............................................................................................................  158
DAPK3-201    EAAAAEEGLRELQRSRRLCHEDVEALAAIYEEKEAWYREESDSLGQDLRRLRQELLKTEALKRQAQEEAKGALLGTSGLKRRFSRLENRYEALAKQVASEMRFVQDLVRA  440
```

```
logo
             LEQEKLQGVECGLR
CHEMBL21156  ..............   12
PF00069.2    ..............   54
PF00069.3    ..............  139
PF00069      ..............  263
PF00069.1    ..............  129
DAPK3-203    ..............  141
DAPK3-206    ..............  158
DAPK3-202    LEQEKLQGVECGLR  454
DAPK3-204    ..............   92
DAPK3-207    ..............  158
DAPK3-201    LEQEKLQGVECGLR  454
```

X non conserved
X similar
X $\geq$ 0% conserved
X $\geq$ 50% conserved

logo

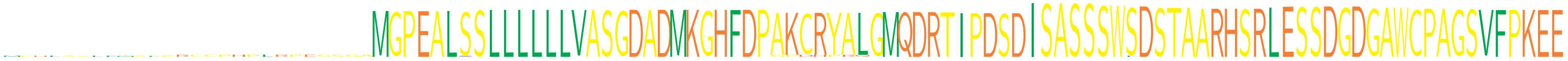


```
IMATINIB     ..............................................................................................................   0
PF00754.6    ........................................................................ISASSSWSDSTAARHSRLESSDGDGAWCPAGSVFPKEE  38
PF00754.9    ........................................................................ISASSSWSDSTAARHSRLESSDGDGAWCPAGSVFPKEE  38
PF07714.1    ..............................................................................................................   0
PF00754.2    ........................................................................ISASSSWSDSTAARHSRLESSDGDGAWCPAGSVFPKEE  38
PF07714.3    ..............................................................................................................   0
PF00754.3    .........................................................................................LESSDGDGAWCPAGSVFPKEE  21
PF00754      ........................................................................ISASSSWSDSTAARHSRLESSDGDGAWCPAGSVFPKEE  38
PF07714      ..............................................................................................................   0
PF07714.5    ..............................................................................................................   0
PF00754.8    ........................................................................ISASSSWSDSTAARHSRLESSDGDGAWCPAGSVFPKEE  38
PF00754.4    ........................................................................ISASSSWSDSTAARHSRLESSDGDGAWCPAGSVFPKEE  38
PF07714.4    ..............................................................................................................   0
PF00754.1    ........................................................................ISASSSWSDSTAARHSRLESSDGDGAWCPAGSVFPKEE  38
PF00754.5    ........................................................................ISASSSWSDSTAARHSRLESSDGDGAWCPAGSVFPKEE  38
PF07714.2    ..............................................................................................................   0
PF00754.7    ........................................................................ISASSSWSDSTAARHSRLESSDGDGAWCPAGSVFPKEE  38
DDR1-229     ..........................MGPEALSSLLLLLLVASGDADMKGHFDPAKCRYALGMQDRTIPDSDISASSSWSDSTAARHSRLESSDGDGAWCPAGSVFPKEE  84
DDR1-262     ........MSLPRCCPHPLRPEGSGAMGPEALSSLLLLLLVASGDADMKGHFDPAKCRYALGMQDRTIPDSDISASSSWSDSTAARHSRLESSDGDGAWCPAGSVFPKEE 102
DDR1-234     ................................XSLLLLLLVASGDADMKGHFDPAKCRYALGMQDRTIPDSDISASSSWL..........ESSDGDGAWCPAGSVFPKEE  68
DDR1-250     ..........................MGPEALSSLLLLLLVASGDADMKGHFDPGPDHPRQ.................................................  35
DDR1-277     ..............................................................................................................   0
DDR1-245     ..............................................................................................................   0
DDR1-244     ..........................MGPEALSSLLLLLLVASGDADMKGHFDPAKCRYALGMQDRTIPDSDISASSSWSDSTAARHSRLESSDGDGAWCPAGSVFPKEE  84
DDR1-259     ..........................MGPEALSSLLLLLLVASGDADMKGHFDPAKCRYALGMQDRTIPDSDISASSSWSDSTAARHSRLESSDGD..............  70
DDR1-248     ..........................MGPEALSSLLLLLLVASGDADMKGHFDPAKCRYALGMQDRTIPDSDISASS.................................  51
DDR1-274     ..........................MGPEALSSLLLLLLVASGDADMKGHFDPGPDHPRQ.................................................  35
DDR1-253     ..........................MGPEALSSLLLLLLVASGDADMKGHFDPAKCRYALGMQDRTIPDSDISASSSWSDSTAARHSRLESSDGDGAW...........  73
DDR1-265     ..........................MGPEALSSLLLLLLVASGDADMKGHFDPAKCRYALGMQDRTIPDSDISASSSWSDSTAARHSRLESSDGDGAWCPAGSVFPKEE  84
DDR1-231     ..............................................................................................................   0
DDR1-254     ..........................MGPEALSSLLLLLLVASGDADMKGHFDPAKCRYALGMQDRTIPDSDISASSSWSDSTAARHSRLESSDGDGAWCPAGSVFPKEE  84
DDR1-228     ..........................MGPEALSSLLLLLLVASGDADMKGHFDPAKCRYALGMQDRTIPDSDISASSSWSDSTAARHSRLESSDGDGAWCPAGSVFPKEE  84
DDR1-240     ..........................MGPEALSSLLLLLLVASGDADMKGHFDPAKCRYALGMQDRTIPDSDISASSSWSDSTAARHSRLESSDGDGAWCPAGSVFPKEE  84
DDR1-223     ..........................MGPEALSSLLLLLLVASGDADMKGHFDPAKCRYALGMQDRTIPDSDISASSSWSDSTAARHSRLESSDGDGAWCPAGSVFPKEE  84
DDR1-251     ..........................MGPEALSSLLLLLLVASGDADMKGHFDPAKCRYALGMQDRT...........................................  41
DDR1-280     ..........................MGPEALSSLLLLLLVASGDADMKGHFDPAKCRYALGMQDRTIPDSDISASSSWSDSTAARHSRLESSDGDGAWCPAGSVFPKEE  84
DDR1-275     ..............................................................................................................   0
DDR1-266     ..........................MGPEALSSLLLLLLVASGDADMKGHFDPAKCRYALGMQDRTIPDSDISASSSWSDSTAARHSRLESSDGDGAWCPAGSVFPKEE  84
DDR1-271     ..........................MGPEALSSLLLLLLVASGDADMKGHFDPAKCRYALGMQDRTIPDSDISASSSWSDSTAARHSRLESSDGDGAWCPAGSVFPKEE  84
DDR1-247     ..........................MGPEALSSLLLLLLVASGDADMKGHFDPAKCRYALGMQDRTIPDSDISASSSWSD.............................  55
DDR1-269     ..........................MGPEALSSLLLLLLVASGDADMKGHFDPAKCRYALGMQDRTIPDSDISASSSWSDSTAARHSRLESSDGDGAWCPAGSVFPKEE  84
DDR1-237     ..........................MGPEALSSLLLLLLVASGDADMKGHFDPAKCRYALGMQDRTIPDSDISASSSWSDSTAARHSRLESSDGDGAWCPAGSVFPKEE  84
DDR1-238     ..........................MGPEALSSLLLLLLVASGDADMKGHFDPAKCRYALGMQDRTIPDSDISASSSWSDSTAARHSRLESSDGDGAWCPAGSVFPKEE  84
DDR1-233     ..........................MGPEALSSLLLLLLVASGDADMKGHFDPAKCRYALGMQDRTIPDSDISASSSWSDSTAARHSRLESSDGDGAWCPAGSVFPKEE  84
DDR1-255     ..........................MGPEALSSLLLLLLVASGDADMKGHFDPAKCRYALGMQDRTIPDSDISASSSWSDSTAARHSRLESSDGDGAWCPAGSVFPKEE  84
DDR1-226     ..........................MGPEALSSLLLLLLVASGDADMKGHFDPAKCRYALGMQDRTIPDSDISASSSWSDSTAARHSRLESSDGDGAWCPAGSVFPKEE  84
DDR1-256     ..........................MGPEALSSLLLLLLVASGDADMKGHFDPAKCRYALGMQDRTIPDSDISASSSWSDSTAARHSRLESSDGDGAWCPAGSVFPKEE  84
DDR1-263     ..........................MGPEALSSLLLLLLVASGDADMKGHFDPAKCRYALGMQDRTIPDSDISASSSWSDSTAARHSRLESSDGDGAWCPAGSVFPKEE  84
DDR1-268     ..........................MGPEALSSLLLLLLVASGDADMKGHFDPAKCRYALGMQDRTIPDSDISASSSWSDSTAARHSRLESSDGDGAWCPAGSVFPKEE  84
DDR1-239     ..........................MGPEALSSLLLLLLVASGDADMKGHFDPAKCRYALGMQDRTIPDSDISASSSWSDSTAARHSRLESSDGDGAWCPAGSVFPKEE  84
DDR1-270     ..........................MGPEALSSLLLLLLVASGDADMKGHFDPAKCRYALGMQDRTIPDSDISASS.................................  51
DDR1-267     ..........................MGPEALSSLLLLLLVASGDADMKGHFDPAKCRYALGMQDRTIPDSDISASSSWSDSTAARHSRLESSDGDGAWCPAGSVFPKEE  84
DDR1-261     ..........................MGPEALSSLLLLLLVASGDADMKGHFDPAKCRYALGMQDRTIPDSDISASSSWSDSTAARHSRLESSDGDGAWCPAGS......  78
DDR1-241     ..........................MGPEALSSLLLLLLVASGDADMKGHFDPAKCRYALGMQDRTIPDSDISASSSWSDSTAARHSRLESSDGDGAWCPAGSVFPKEE  84
DDR1-225     ..........................MGPEALSSLLLLLLVASGDADMKGHFDPAKCRYALGMQDRTIPDSDISASSSWSDSTAARHSRLESSDGDGAWCPAGSVFPKEE  84
DDR1-257     ..........................MGPEALSSLLLLLLVASGDADMKGHFDPAKCRYALGMQDRTIPDSDISASSSWSDSTAARHSRLESSDGDGAWCPAGSVFPKEE  84
DDR1-235     ..........................MGPEALSSLLLLLLVASGDADMKGHFDPAKCRYALGMQDRTIPDSDISASSSWSDSTAARHSRLESSDGDGAWCPAGSVFPKEE  84
DDR1-230     ..........................MGPEALSSLLLLLLVASGDADMKGHFDPAKCRYALGMQDRTIPDSDISASSSWSDSTA..........................  58
```

```
logo         EYLQVDLQRLHLVALVGTQGRHAGGLGKEFSRSYRLRYSRDGRRWMGWKDRWGQEVISGNEDPEGVVLKDLGPPMVARLVRFYPRADRVMSVCLRVELYGCLWRDGLLSY
IMATINIB     ..............................................................................................................   0
PF00754.6    EYLQVDLQRLHLVALVGTQGRHAGGLGKEFSRSYRLRYSRDGRRWMGWKDRWGQEVISGNEDPEGVVLKDLGPPMVARLVRFYPR.........................   123
PF00754.9    EYLQVDLQRLHLVALVGTQGRHAGGLGKEFSRSYRLRYSRDGRRWMGWKDRWGQEVISGNEDPEGVVLKDLGPPM...................................   113
PF07714.1    ..............................................................................................................   0
PF00754.2    EYLQVDLQRLHLVALVGTQGRHAGGLGKEFSRSYRLRYSRDGRRWMGWKDRWGQEVISGNEDPE..............................................   102
PF07714.3    ..............................................................................................................   0
PF00754.3    EYLQVDLQRLHLVALVGTQGRHAGGLGKEFSRSYRLRYSRDGRRWMGWKDRWGQEVISGNEDPEGVVLKDLGPPMVARLVRFYPRADRVMSVCLRVEL............   119
PF00754      EYLQVDLQRLHLVALVGTQGRHAGGLGKEFSRSYRLRYSRDGRRWMGWKDRWGQEVISGNEDPEGVVLKDLGPPMVARLVRFYPRADRVMSVCLRVEL............   136
PF07714      ..............................................................................................................   0
PF07714.5    ..............................................................................................................   0
PF00754.8    EYLQVDLQRLHLVALVGTQGRHAGGLGKEFSRSYRLRYSRDG....................................................................   80
PF00754.4    EYLQVDLQRLHLVALVGTQGRHAGGLGKEFSRSYRLRYSRDGRRWM................................................................   84
PF07714.4    ..............................................................................................................   0
PF00754.1    EYLQVDLQRLHLVALVGTQGRHAGGLGKEFSRSYRLRYSRDGRRWMGWKDRWGQEVISGNED................................................   100
PF00754.5    EYLQVDLQRLHLVALVGTQGRHAGGLGKEFSRSYRLRYSRDGRRWMGWKD............................................................   88
PF07714.2    ..............................................................................................................   0
PF00754.7    EYLQVDLQRLHLVALVGTQGRHAGGLGKEFSRSYRLRY........................................................................   76
DDR1-229     EYLQVDLQRLHLVALVGTQGRHAGGLGKEFSRSYRLRYSRDGRRWMGWKDRWGQEVISGNEDP...............................................   147
DDR1-262     EYLQVDLQRLHLVALVGTQGRHAGGLGKEFSRSYRLRYSRDGRRWMGWKDRWGQEVISGNEDPEGVVLKDLGPPMVARLVRFYPRADRVMSVCLRVELYGCLWRDGLLSY   212
DDR1-234     EYLQVDLQRLHLVALVGTQGRHAGGLGKEFSRSYRLRYSRDGRRWMGWKDRWGQEVISGNEDPEGVVLKDLGPPMVARLVRFYPRADRVMSVCLRVELYGCLWRDGLLSY   178
DDR1-250     ..............................................................................................................   35
DDR1-277     ..........................................................................MVARLVRFYPRADRVMSVCLRVELYGCLWRDGLLSY   36
DDR1-245     ..............................................................................................................   0
DDR1-244     EYLQVDLQRLHLVALVGTQGRHAGGLGKEFSRSYRLRYSRDGRRWMGWKDRWGQEVISGNEDPEGVVLKDLGPPMVARLVRFYPRADRVMSVCLRVELYGCLWRDCSMGV   194
DDR1-259     ..............................................................................................................   70
DDR1-248     ..............................................................................................................   51
DDR1-274     ..............................................................................................................   35
DDR1-253     ..............................................................................................................   73
DDR1-265     EYLQVDLQRLHLVALVGTQGRHAGGLGKEF................................................................................   114
DDR1-231     ..............................................................................................................   0
DDR1-254     EYLQVDLQRLHLVALVGTQGRHAGGLGKEFSRSYRLRY........................................................................   122
DDR1-228     EYLQVDLQRLHLVALVGTQGRHAGGLGKEFSRSYRLRYSRDGRRWMGWKDRWGQEVISGNEDPEGVVLKDLGPPMVARLVRFYPRADRVMSVCLRVELYGCLWRDCSMGV   194
DDR1-240     EYLQVDLQRLHLVALVGTQGRHAGGLGKEFSRSYRLRYSRDGRRWMGWKDRWGQEVISGNEDPEGVVLKDLGPPMVARLVRFYPRADRVMSVCLRVELYGCLWRDGLLSY   194
DDR1-223     EYLQVDLQRLHLVALVGTQGRHAGGLGKEFSRSYRLRYSRDGRRWMGWKDRWGQEVISGNEDPEGVVLKDLGPPMVARLVRFYPRADRVMSVCLRVELYGCLWRDGLLSY   194
DDR1-251     ..............................................................................................................   41
DDR1-280     E.............................................................................................................   85
DDR1-275     ..............................................................................................................   0
DDR1-266     EYLQVDLQRLHLVALVGTQGRHAGGLGKEFSRSYRLRYSRDGRRWMGWKDRWGQEVIS....................................................   142
DDR1-271     EYLQVDLQRLHLVALVGTQGRHAGGLGKEFSRSYRLRYSRDGRRWMGWKDRWGQEVISGNEDPEGVVLKDLGPPMVARLVRFYPRADRVMSVCLRVELYGCLWRDGLLSY   194
DDR1-247     ..............................................................................................................   55
DDR1-269     EYLQVDLQRLHLVALVGTQGRHAGGLGKEFSRSYRLRYSRDGRRWMGWKDRWGQ........................................................   138
DDR1-237     EYLQVDLQRLHLVALVGTQGRHAGGLGKEFSRSYRLRYSRDGRRWMGWKDRWGQEVISGNEDPEGVVLK........................................   153
DDR1-238     EYLQVDLQRLHLVALVGTQGRHAGGLGKEFSRSYRLRYSRDGRRWMGWKDRWGQEVISGNEDPEGVVLKDLGPPMVARLVRFYPRADRVMSVCLRVELYGCLWRDGLLSY   194
DDR1-233     EYLQVDLQRLHLVALVGTQGRHAGGLGKEFSRSYRLRYSRDGRRWMGWKDRWGQEVISGNEDPEGVVLKDLGPPMVARLVRFYPRADRVMSVCLRVELYGCLWRDGLLSY   194
DDR1-255     EYLQVDLQRLHLVALVGTQGRHAGGLGKEFSRSYRLRYSRDGRRWMGWKDRWGQEVISGNEDPEGVVLKDLGPPMV..................................   160
DDR1-226     EYLQVDLQRLHLVALVGTQGRHAGGLGKEFSRSYRLRYSRDGRRWMGWKDRWGQEVISGNEDPEGVVLKDLGPPMVARLVRFYPRADRVMSVCLRVELYGCLWRDGLLSY   194
DDR1-256     EYLQVDLQRLHLVALVGTQGRHAGGLGKEFSRSYRLRYSRDGRRWMGW..............................................................   132
DDR1-263     EYLQVDLQRLHLVALVGTQGRHAGGLGKEFSRSYRLRYSRDG....................................................................   126
DDR1-268     EYLQVDLQRLHLVALVGTQGRHAGGLGKEFSRSYRLRYSRDGRRWMGWKDRWGQEVISGNEDPEGVVLKDLGPPMVARLVRFYPRA........................   170
DDR1-239     EYLQVDLQRLHLVALVGTQGRHAGGLGKEFSRSYRLRYSRDGRRWMGWKDRWGQEVISGNEDPEGVVLKDLGPPMVARLVRFYPRADRVMSVCLRVELYGCLWRDGLLSY   194
DDR1-270     ..............................................................................................................   51
DDR1-267     EYLQVDLQRLHLVALVGTQGRHAGGLGK..................................................................................   112
DDR1-261     ..............................................................................................................   78
DDR1-241     EYLQVDLQRLHLVALVGTQGRHAGGLGKEFSRSYRLRYSRDGRRWMGWKDRWGQEVISGNEDPEGVVLKDLGPPMVARLVRFYPRADRVMSVCLRVELYGCLWRDGLLSY   194
DDR1-225     EYLQVDLQRLHLVALVGTQGRHAGGLGKEFSRSYRLRYSRDGRRWMGWKDRWGQEVISGNEDPEGVVLKDLGPPMVARLVRFYPRADRVMSVCLRVELYGCLWRDGLLSY   194
DDR1-257     EYLQVDLQRLHLVALV..............................................................................................   100
DDR1-235     EYLQVDLQRLHLVALVGTQGRHAGGLGKEFSRSYRLRYSRDGRRWMGWKDRWGQEVISGNEDPEGVVLKDLGPPMVARLVRFYPRADRVMSVCLRVELYGCLWRDGLLSY   194
DDR1-230     ..............................................................................................................   58
```

logo

```
             TAPVGQTMYLSEAVYLNDSTYDGHTVGGLQYGGLGQLADGVVGLDDFRKSQELRVWPGYDYV      GWSNHSFSSGYVEMEFEFDRLRAFQAMQVHCNNMHTLGARLP
IMATINIB     ..............................................................................................................   0
PF00754.6    .............................................................................................................. 123
PF00754.9    .............................................................................................................. 113
PF07714.1    ..............................................................................................................   0
PF00754.2    .............................................................................................................. 102
PF07714.3    ..............................................................................................................   0
PF00754.3    .............................................................................................................. 119
PF00754      .............................................................................................................. 136
PF07714      ..............................................................................................................   0
PF07714.5    ..............................................................................................................   0
PF00754.8    ..............................................................................................................  80
PF00754.4    ..............................................................................................................  84
PF07714.4    ..............................................................................................................   0
PF00754.1    .............................................................................................................. 100
PF00754.5    ..............................................................................................................  88
PF07714.2    ..............................................................................................................   0
PF00754.7    ..............................................................................................................  76
DDR1-229     .............................................................................................................. 147
DDR1-262     TAPVGQTMYLSEAVYLNDSTYDGHTVGGLQYGGLGQLADGVVGLDDFRKSQELRVWPGYDYV......GWSNHSFSSGYVEMEFEFDRLRAFQAMQVHCNNMHTLGARLP 316
DDR1-234     TAPVGQTMYLSEAVYLND............................................................................................ 196
DDR1-250     ..............................................................................................................  35
DDR1-277     TAPVGQTMYLSEAVYLNDSTYDGHTVGGLQYGG.............................................................................  69
DDR1-245     ..............................................................................................................   0
DDR1-244     WASWQMVWW............GWMTLGRVRSCGSG...............QAMTMWDGATTASPVAMWRWSLSL..TG................................ 243
DDR1-259     ..............................................................................................................  70
DDR1-248     ..............................................................................................................  51
DDR1-274     ..............................................................................................................  35
DDR1-253     ..............................................................................................................  73
DDR1-265     .............................................................................................................. 114
DDR1-231     ..........SEAVYLNDSTYDGHTVGGLQYGGLGQLADGVVGLDDFRKSQELRVWPGYDYV......GWSNHSFSSGYVEMEFEFDRLRAFQAMQVHCNNMHTLGARLP  94
DDR1-254     .............................................................................................................. 122
DDR1-228     WASWQMVWW............GWMTLGRVRSCG...............SGQAMTMWDGATTASPVAMWRWSLSLTG.................................. 243
DDR1-240     TAPVGQTMYLSEAVYLNDSTYDGHTVGGLQYGGLGQLADGVVGLDDFRKSQELRVWPGYDYV......GWSNHSFSSGYVEMEFEFDRLRAFQAMQVHCNNMHTLGARLP 298
DDR1-223     TAPVGQTMYLSEAVYLNDSTYDGHTVGGLQYGGLGQLADGVVGLDDFRKSQELRVWPGYDYV......GWSNHSFSSGYVEMEFEFDRLRAFQAMQVHCNNMHTLGARLP 298
DDR1-251     ..............................................................................................................  41
DDR1-280     ..............................................................................................................  85
DDR1-275     ..............................................................................................................   0
DDR1-266     .............................................................................................................. 142
DDR1-271     TAPVGQTMYLSEAVYLNDSTYDGHTVGGLQYGGLGQLADGVVGLDDFRKSQELRVWPGYDYV......GWSNHSFSSGYVEMEFEFDRLRAFQAMQVHCNNMHTLGARLP 298
DDR1-247     ..............................................................................................................  55
DDR1-269     .............................................................................................................. 138
DDR1-237     .............................................................................................................. 153
DDR1-238     TAPVGQTMYLSEAVYLNDSTYDGHTVGGLQYGGLGQLADGVVGLDDFRKSQELRVWPGYDYV......GWSNHSFSSGYVEMEFEFDRLRAFQAMQVHCNNMHTLGARLP 298
DDR1-233     TAPVGQTMYLSEAVY............................................................................................... 209
DDR1-255     .............................................................................................................. 160
DDR1-226     TAPVGQTMYLSEAVYLNDSTYDGHTVGGLQYGGLGQLADGVVGLDDFRKSQELRVWPGYDYV......GWSNHSFSSGYVEMEFEFDRLRAFQAMQVHCNNMHTLGARLP 298
DDR1-256     .............................................................................................................. 132
DDR1-263     .............................................................................................................. 126
DDR1-268     .............................................................................................................. 170
DDR1-239     TAPVGQTMYLSEAVYLNDSTYDGHTVGGLQYGGLGQLADGVVGLDDFRKSQELRVWPGYDYV......GWSNHSFSSGYVEMEFEFDRLRAFQAMQVHCNNMHTLGARLP 298
DDR1-270     ..............................................................................................................  51
DDR1-267     .............................................................................................................. 112
DDR1-261     ..............................................................................................................  78
DDR1-241     TAPVGQTMYLSEAVYLNDSTYDGHTVGGLQYGGLGQLADGVVGLDDFRKSQELRVWPGYDYV......GWSNHSFSSGYVEMEFEFDRLRAFQAMQVH............ 286
DDR1-225     TAPVGQTMYLSEAVYLNDSTYDGHTVGGLQYGGLGQLADGVVGLDDFRKSQELRVWPGYDYV......GWSNHSFSSGYVEMEFEFDRLRAFQAMQVHCNNMHTLGARLP 298
DDR1-257     .............................................................................................................. 100
DDR1-235     TAPVGQTMYLSEAVYLNDSTYDGHTVGGLQYGGLGQLADGVVGLDDFRKSQELRVWPGYDYV......GWSNHSFSSGY............................... 267
DDR1-230     ..............................................................................................................  58
```

```
logo
             GGVECRFRRGPAMAWEGEPMRHNLGGNLGDPRARAVSVPLGGRVARFLQCRFLFAGPWLLFSEISFISDVVNNSSPALGGTFPPAPWWPPGPPPTNFSSLELEPRGQQPV
IMATINIB     ..............................................................................................................   0
PF00754.6    ..............................................................................................................   123
PF00754.9    ..............................................................................................................   113
PF07714.1    ..............................................................................................................   0
PF00754.2    ..............................................................................................................   102
PF07714.3    ..............................................................................................................   0
PF00754.3    ..............................................................................................................   119
PF00754      ..............................................................................................................   136
PF07714      ..............................................................................................................   0
PF07714.5    ..............................................................................................................   0
PF00754.8    ..............................................................................................................   80
PF00754.4    ..............................................................................................................   84
PF07714.4    ..............................................................................................................   0
PF00754.1    ..............................................................................................................   100
PF00754.5    ..............................................................................................................   88
PF07714.2    ..............................................................................................................   0
PF00754.7    ..............................................................................................................   76
DDR1-229     ..............................................................................................................   147
DDR1-262     GGVECRFRRGPAMAWEGEPMRHNLGGNLGDPRARAVSVPLGGRVARFLQCRFLFAGPWLLFSEISFISDVVNNSSPALGGTFPPAPWWPPGPPPTNFSSLELEPRGQQPV   426
DDR1-234     ..............................................................................................................   196
DDR1-250     ..............................................................................................................   35
DDR1-277     ..............................................................................................................   69
DDR1-245     ..............................................................................................................   0
DDR1-244     ..............................................................................................................   243
DDR1-259     ..............................................................................................................   70
DDR1-248     ..............................................................................................................   51
DDR1-274     ..............................................................................................................   35
DDR1-253     ..............................................................................................................   73
DDR1-265     ..............................................................................................................   114
DDR1-231     GGVECRFRRGPAMAWEGEPMRHNLGGNLGDPRARAVSVPLGGRVARFLQCRFLFAGPWLLFSEISFISDVVNNSSPALGGTFPPAPWWPPGPPPTNFSSLELEPRGQQPV   204
DDR1-254     ..............................................................................................................   122
DDR1-228     ..............................................................................................................   243
DDR1-240     GGVECRFRRGPAMAWEGEPMRHNLGGNLGDPRARAVSVPLGGRVARFLQCRFLFAGPWLLFSEISFISDVVNNSSPALGGTFPPAPWWPPGPPPTNFSSLELEPRGQQPV   408
DDR1-223     GGVECRFRRGPAMAWEGEPMRHNLGGNLGDPRARAVSVPLGGRVARFLQCRFLFAGPWLLFSEISFISDVVNNSSPALGGTFPPAPWWPPGPPPTNFSSLELEPRGQQPV   408
DDR1-251     ..............................................................................................................   41
DDR1-280     ..............................................................................................................   85
DDR1-275     ..............................................................................................................   0
DDR1-266     ..............................................................................................................   142
DDR1-271     GGVECRFRRGPAMAWEGEPMRHNLGGNLGDPRARAVSVPLGGRVARFLQCRFLFAGPWLLFSEISFISDVVNNSSPALGGTFPPAPWWPPGPPPTNFSSLELEPRGQQPV   408
DDR1-247     ..............................................................................................................   55
DDR1-269     ..............................................................................................................   138
DDR1-237     ..............................................................................................................   153
DDR1-238     GGVECRFRRGPAMAWEGEPMRHNLGGNLGDPRARAVSVPLGGRVARFLQCRFLFAGPWLLFSEISFISDVVNNSSPALGGTFPPAPWWPPGPPPTNFSSLELEPRGQQPV   408
DDR1-233     ..............................................................................................................   209
DDR1-255     ..............................................................................................................   160
DDR1-226     GGVECRFRRGPAMAWEGEPMRHNLGGNLGDPRARAVSVPLGGRVARFLQCRFLFAGPWLLFSEISFISDVVNNSSPALGGTFPPAPWWPPGPPPTNFSSLELEPRGQQPV   408
DDR1-256     ..............................................................................................................   132
DDR1-263     ..............................................................................................................   126
DDR1-268     ..............................................................................................................   170
DDR1-239     GGVECRFRRGPAMAWEGEPMRHNLGGNLGDPRARAVSVPLGGRVARFLQCRFLFAGPWLLFSEISFISDVVNNSSPALGGTFPPAPWWPPGPPPTNFSSLELEPRGQQPV   408
DDR1-270     ..............................................................................................................   51
DDR1-267     ..............................................................................................................   112
DDR1-261     ..............................................................................................................   78
DDR1-241     ..............................................................................................................   286
DDR1-225     GGVECRFRRGPAMAWEGEPMRHNLGGNLGDPRARAVSVPLGGRVARFLQCRFLFAGPWLLFSEISFISDVVNNSSPALGGTFPPAPWWPPGPPPTNFSSLELEPRGQQPV   408
DDR1-257     ..............................................................................................................   100
DDR1-235     ..............................................................................................................   267
DDR1-230     ..............................................................................................................   58
```

logo

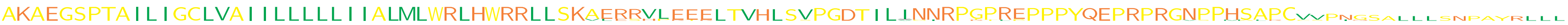

```
IMATINIB     ..............................................................................................................  0
PF00754.6    ..............................................................................................................  123
PF00754.9    ..............................................................................................................  113
PF07714.1    ..............................................................................................................  0
PF00754.2    ..............................................................................................................  102
PF07714.3    ..............................................................................................................  0
PF00754.3    ..............................................................................................................  119
PF00754      ..............................................................................................................  136
PF07714      ..............................................................................................................  0
PF07714.5    ..............................................................................................................  0
PF00754.8    ..............................................................................................................  80
PF00754.4    ..............................................................................................................  84
PF07714.4    ..............................................................................................................  0
PF00754.1    ..............................................................................................................  100
PF00754.5    ..............................................................................................................  88
PF07714.2    ..............................................................................................................  0
PF00754.7    ..............................................................................................................  76
DDR1-229     ..............................................................................................................  147
DDR1-262     AKAEGSPTAILIGCLVAIILLLLLIIALMLWRLHWRRLLSKAERRVLEEELTVHLSVPGDTILINNRPGPREPPPYQEPRPRGNPPHSAPCV..................  518
DDR1-234     ..............................................................................................................  196
DDR1-250     ..............................................................................................................  35
DDR1-277     ..............................................................................................................  69
DDR1-245     ..............................................................................................................  0
DDR1-244     ..............................................................................................................  243
DDR1-259     ..............................................................................................................  70
DDR1-248     ..............................................................................................................  51
DDR1-274     ..............................................................................................................  35
DDR1-253     ..............................................................................................................  73
DDR1-265     ..............................................................................................................  114
DDR1-231     AKAEGSPTAILIGCLVAIILLLLLIIALMLWRLHWRRLLSKVLESHPRTR......SPG...LVGIRPTPLP...........VSPMALRCCSPIQ..............  280
DDR1-254     ..............................................................................................................  122
DDR1-228     ..............................................................................................................  243
DDR1-240     AKAEGSPTAILIGCLVAIILLLLLIIALMLWRLHWRRLLSKAERRVLEEELTVHLSVPGDTILINNRPGPREPPPYQEPRPRGNPPHSAPCV..................  500
DDR1-223     AKAEGSPTAILIGCLVAIILLLLLIIALMLWRLHWRRLLSKAERRVLEEELTVHLSVPGDTILINNRPGPREPPPYQEPRPRGNPPHSAPC.VPNGSALLLSNPAYRLLL  517
DDR1-251     ..............................................................................................................  41
DDR1-280     ..............................................................................................................  85
DDR1-275     ...............................................................XNNRPGPREPPPYQEPRPRGNPPHSAPC.VPNGS.............  33
DDR1-266     ..............................................................................................................  142
DDR1-271     AKAEGSPTAILIGCLVAIILLLLLIIALMLWRLHWRRLLSKAERRVLEEELTVHLSVPGDTILINNRPGPREPPPYQEPRPRGNPPHSAPC.VPNGSALLLSNPAYRLLL  517
DDR1-247     ..............................................................................................................  55
DDR1-269     ..............................................................................................................  138
DDR1-237     ..............................................................................................................  153
DDR1-238     AKAEGSPTAILIGCLVAIILLLLLIIALMLWRLHWRRLLSKVLESHP.....................RTRS......................................  459
DDR1-233     ..............................................................................................................  209
DDR1-255     ..............................................................................................................  160
DDR1-226     AKAEGSPTAILIGCLVAIILLLLLIIALMLWRLHWRRLLSKAERRVLEEELTVHLSVPGDTILINNRPGPREPPPYQEPRPRGNPPHSAPCV..................  500
DDR1-256     ..............................................................................................................  132
DDR1-263     ..............................................................................................................  126
DDR1-268     ..............................................................................................................  170
DDR1-239     AKAEGSPTAILIGCLVAIILLLLLIIALMLWRLHWRRLLSKAERRVLEEELTVHLSVPGDTILINNRPGPREPPPYQEPRPRGNPPHSAPC.VPNGSALLLSNPAYRLLL  517
DDR1-270     ..............................................................................................................  51
DDR1-267     ..............................................................................................................  112
DDR1-261     ..............................................................................................................  78
DDR1-241     ..............................................................................................................  286
DDR1-225     AKAEGSPTAILIGCLVAIILLLLLIIALMLWRLHWRRLLSKAERRVLEEELTVHLSVPGDTILINNRPGPREPPPYQEPRPRGNPPHSAPC.VPNGSALLLSNPAYRLLL  517
DDR1-257     ..............................................................................................................  100
DDR1-235     ..............................................................................................................  267
DDR1-230     ..............................................................................................................  58
```

logo

```
IMATINIB     ..........................................................................................................V...  1
PF00754.6    ..............................................................................................................  123
PF00754.9    ..............................................................................................................  113
PF07714.1    ............................................................................................LRFKEKLGEGQFGEVHLC  18
PF00754.2    ..............................................................................................................  102
PF07714.3    ..............................................................................................................  0
PF00754.3    ..............................................................................................................  119
PF00754      ..............................................................................................................  136
PF07714      ............................................................................................LRFKEKLGEGQFGEVHLC  18
PF07714.5    ............................................................................................LRFKEKLGEGQFGEVHLC  18
PF00754.8    ..............................................................................................................  80
PF00754.4    ..............................................................................................................  84
PF07714.4    ............................................................................................LRFKEKLGEGQFGEVHLC  18
PF00754.1    ..............................................................................................................  100
PF00754.5    ..............................................................................................................  88
PF07714.2    ..............................................................................................................  0
PF00754.7    ..............................................................................................................  76
DDR1-229     ..............................................................................................................  147
DDR1-262     ....................PNGSAYSGDYMEPEKPGAPLLPPPPQNSVPHYAEADIVTLQGVTGGNTYAVPALPPGAVGDGPPRVDFPRSRLRFKEKLGEGQFGEVHLC  608
DDR1-234     ..............................................................................................................  196
DDR1-250     ..............................................................................................................  35
DDR1-277     ..............................................................................................................  69
DDR1-245     ..............................................................................................................  0
DDR1-244     ..............................................................................................................  243
DDR1-259     ..............................................................................................................  70
DDR1-248     ..............................................................................................................  51
DDR1-274     ..............................................................................................................  35
DDR1-253     ..............................................................................................................  73
DDR1-265     ..............................................................................................................  114
DDR1-231     ...........PTASFWPLTP..........VPLEARA..........PPHPPGPNPPTPRGVTGGNTYAVPALPPGAVGDGPPRVDFPRSRLRFKEKLGEGQFGEVHLC  359
DDR1-254     ..............................................................................................................  122
DDR1-228     ..............................................................................................................  243
DDR1-240     ....................PNGSAYSGDYMEPEKPGAPLLPPPPQNSVPHYAEADIVTLQGVTGGNTYAVPALPPGAVGDGPPRVDFPRSRLRFKEKLGEGQFGEVHLC  590
DDR1-223     ATYARPPRGPGPPTPAWAKPTNTQAYSGDYMEPEKPGAPLLPPPPQNSVPHYAEADIVTLQGVTGGNTYAVPALPPGAVGDGPPRVDFPRSRLRFKEKLGEGQFGEVHLC  627
DDR1-251     ..............................................................................................................  41
DDR1-280     ..............................................................................................................  85
DDR1-275     ........................AYSGDYMEPEKPGAPLLPPPPQNSVPHYAEADIVTLQGVTGGNTYAVPALPPGAVGDGPPRVDFPRSRLRFKEKLGEGQFGEVHLC  119
DDR1-266     ..............................................................................................................  142
DDR1-271     ATYARPPRGPGPPTPAWAKPTNTQAYSGDYMEPEKPGAPLLPPPPQNSVPHYAEADIVTLQGVTGGNTYAVPALPPGAVGDGPPRVDFPRSRLRFKEKLGEGQFGEVHLC  627
DDR1-247     ..............................................................................................................  55
DDR1-269     ..............................................................................................................  138
DDR1-237     ..............................................................................................................  153
DDR1-238     ...........................................................................PGLVGIRPT.............PLPVSPMALVHLC  481
DDR1-233     ..............................................................................................................  209
DDR1-255     ..............................................................................................................  160
DDR1-226     ....................PNGSAYSGDYMEPEKPGAPLLPPPPQNSVPHYAEADIVTLQGVTGGNTYAVPALPPGAVGDGPPRVDFPRSRLRFKEKLGEGQFGEVHLC  590
DDR1-256     ..............................................................................................................  132
DDR1-263     ..............................................................................................................  126
DDR1-268     ..............................................................................................................  170
DDR1-239     ATYARPPRGPGPPTPAWAKPTNTQAYSGDYMEPEKPGAPLLPPPPQNSVPHYAEADIVTLQGVTGGNTYAVPALPPGAVGDGPPRVDFPRSRLRFKEKLGEGQFGEVHLC  627
DDR1-270     ..............................................................................................................  51
DDR1-267     ..............................................................................................................  112
DDR1-261     ..............................................................................................................  78
DDR1-241     ..............................................................................................................  286
DDR1-225     ATYARPPRGPGPPTPAWAKPTNTQAYSGDYMEPEKPGAPLLPPPPQNSVPHYAEADIVTLQGVTGGNTYAVPALPPGAVGDGPPRVDFPRSRLRFKEKLGEGQFGEVHLC  627
DDR1-257     ..............................................................................................................  100
DDR1-235     ..............................................................................................................  267
DDR1-230     ..............................................................................................................  58
```

```
logo
             EVDSPQDLVSLDFPLNVRKGHPLLVAVKILRPDATKNASFSLFSRNDFLKEVKIMSRLKDPNIIRLLGVCVQDDPLCMITDYMENGDLNQFLSAHQLEDKAAEGAPGDGQ
IMATINIB     .........................AVK......................E..IM........I.............M.T.YM...........................   12
PF00754.6    ..............................................................................................................  123
PF00754.9    ..............................................................................................................  113
PF07714.1    EVDSPQDLVSLDFPLNVRKGHPLLVAVKILRPDATKNA......RNDFLKEVKIMSRLKDPNIIRLLGVCVQDDPLCMITDYMENGDLNQFLSAHQLEDKAAEGAPGDGQ  122
PF00754.2    ..............................................................................................................  102
PF07714.3    ....................HPLLVAVKILRPDATKNA......RNDFLKEVKIMSRLKDPNIIRLLGVCVQDDPLCMITDYMENGDLNQFLSAHQLEDKAAEGAPGDGQ   84
PF00754.3    ..............................................................................................................  119
PF00754      ..............................................................................................................  136
PF07714      EVDSPQDLVSLDFPLNVRKGHPLLVAVKILRPDATKNA......RNDFLKEVKIMSRLKDPNIIRLLGVCVQDDPLCMITDYMENGDLNQFLSAHQLEDKAAEGAPGDGQ  122
PF07714.5    EVDSPQDLVSLDFPLNVRKGHPLLVAVKILRPDATKNASFSLFSRNDFLKEVKIMSRLKDPNIIRLLGVCVQDDPLCMITDYMENGDLNQFLSAHQLEDKAAEGAPGDGQ  128
PF00754.8    ..............................................................................................................   80
PF00754.4    ..............................................................................................................   84
PF07714.4    EVDSPQDLVSLDFPLNVRKGHPLLVAVKILRPDATKNASFSLFSRNDFLKE...........................................................   69
PF00754.1    ..............................................................................................................  100
PF00754.5    ..............................................................................................................   88
PF07714.2    ....................HPLLVAVKILRPDATKNA......RNDFLKEVKIMSRLKDPNIIRLLGVCVQDDPLCMITDYMENGDLNQFLSAHQLEDKAAEGAPGDGQ   84
PF00754.7    ..............................................................................................................   76
DDR1-229     ..............................................................................................................  147
DDR1-262     EVDSPQDLVSLDFPLNVRKGHPLLVAVKILRPDATKNA......RNDFLKEVKIMSRLKDPNIIRLLGVCVQDDPLCMITDYMENGDLNQFLSAHQLEDKAAEGAPGDGQ  712
DDR1-234     ..............................................................................................................  196
DDR1-250     ..............................................................................................................   35
DDR1-277     ..............................................................................................................   69
DDR1-245     ................XRKGHPLLVAVKILRPDATKNA......RNDFLKEVKIMSRLKDPNIIRLLGVCVQDDPLCMITDYMENGDLNQFLSAHQLEDKAAEGAPGDGQ   88
DDR1-244     ..............................................................................................................  243
DDR1-259     ..............................................................................................................   70
DDR1-248     ..............................................................................................................   51
DDR1-274     ..............................................................................................................   35
DDR1-253     ..............................................................................................................   73
DDR1-265     ..............................................................................................................  114
DDR1-231     EVDSPQDLVSLDFPLNVRKGHPLLVAVKILRPDATKNA......RNDFLKEVKIMSRLKDPNIIRLLGVCVQDDPLCMITDYMENGDLNQFLSAHQLEDKAAEGAPGDGQ  463
DDR1-254     ..............................................................................................................  122
DDR1-228     ..............................................................................................................  243
DDR1-240     EVDSPQDLVSLDFPLNVRKGHPLLVAVKILRPDATKNA......RNDFLKEVKIMSRLKDPNIIRLLGVCVQDDPLCMITDYMENGDLNQFLSAHQLEDKAAEGAPGDGQ  694
DDR1-223     EVDSPQDLVSLDFPLNVRKGHPLLVAVKILRPDATKNA......RNDFLKEVKIMSRLKDPNIIRLLGVCVQDDPLCMITDYMENGDLNQFLSAHQLEDKAAEGAPGDGQ  731
DDR1-251     ..............................................................................................................   41
DDR1-280     ..............................................................................................................   85
DDR1-275     EVDSPQDLVSLDFPLNVRKGHPLLVAVKILRPDATKNASFSLFSRNDFLKEVK.........................................................  172
DDR1-266     ..............................................................................................................  142
DDR1-271     EVDSPQDLVSLDFPLNVRKGHPLLVAVKILRPDATKNASFSLFSRNDFLKEVKIMSRLKDPNIIRLLGVCVQDDPLCMITDYMENGDLNQFLSAHQLEDKAAEGAPGDGQ  737
DDR1-247     ..............................................................................................................   55
DDR1-269     ..............................................................................................................  138
DDR1-237     ..............................................................................................................  153
DDR1-238     EVDSPQDLVSLDFPLNVRKGHPLLVAVKILRPDATKNA......RNDFLKEVKIMSRLKDPNIIRLLGVCVQDDPLCMITDYMENGDLNQFLSAHQLEDKAAEGAPGDGQ  585
DDR1-233     ..............................................................................................................  209
DDR1-255     ..............................................................................................................  160
DDR1-226     EVDSPQDLVSLDFPLNVRKGHPLLVAVKILRPDATKNA......RNDFLKEVKIMSRLKDPNIIRLLGVCVQDDPLCMITDYMENGDLNQFLSAHQLEDKAAEGAPGDGQ  694
DDR1-256     ..............................................................................................................  132
DDR1-263     ..............................................................................................................  126
DDR1-268     ..............................................................................................................  170
DDR1-239     EVDSPQDLVSLDFPLNVRKGHPLLVAVKILRPDATKNA......RNDFLKEVKIMSRLKDPNIIRLLGVCVQDDPLCMITDYMENGDLNQFLSAHQLEDKAAEGAPGDGQ  731
DDR1-270     ..............................................................................................................   51
DDR1-267     ..............................................................................................................  112
DDR1-261     ..............................................................................................................   78
DDR1-241     ..............................................................................................................  286
DDR1-225     EVDSPQDLVSLDFPLNVRKGHPLLVAVKILRPDATKNA......RNDFLKEVKIMSRLKDPNIIRLLGVCVQDDPLCMITDYMENGDLNQFLSAHQLEDKAAEGAPGDGQ  731
DDR1-257     ..............................................................................................................  100
DDR1-235     ..............................................................................................................  267
DDR1-230     ..............................................................................................................   58
```

```
logo
             AAQGPTISYPMLLHVAAQIASGMRYLATLNFVHRDLATRNCLVGENFTIKIADFGMSRNLYAGDYYRVQGRAVLPIRWMAWECILMGKFTTASDVWAFGVTLWEVLMLCR
IMATINIB     ...............................VHR.......L.........ADF........................................................   19
PF00754.6    ..............................................................................................................  123
PF00754.9    ..............................................................................................................  113
PF07714.1    AAQGPTISYPMLLHVAAQIASGMRYLAT..................................................................................  150
PF00754.2    ..............................................................................................................  102
PF07714.3    AAQGPTISYPMLLHVAAQIASGMRYLATLNFVHRDLATRNCLVGENFTIKIADFGMSRNLYAGDYYRVQGRAVLPIRWMAWECILMGKFTTASDVWAFGVTLWEVLMLCR  194
PF00754.3    ..............................................................................................................  119
PF00754      ..............................................................................................................  136
PF07714      AAQGPTISYPMLLHVAAQIASGMRYLATLNFVHRDLATRNCLVGENFTIKIADFGMSRNLYAGDYYRVQGRAVLPIRWMAWECILMGKFTTASDVWAFGVTLWEVLMLCR  232
PF07714.5    AAQGPTISYPMLLHVAAQIASGMRYLATLNFVHRDLATRNCLVGENFTIKIADFGMSRNLYAGDYYRVQGRAVLPIRWMAWECILMGKFTTASDVWAFGVTLWEVLMLCR  238
PF00754.8    ..............................................................................................................   80
PF00754.4    ..............................................................................................................   84
PF07714.4    ..............................................................................................................   69
PF00754.1    ..............................................................................................................  100
PF00754.5    ..............................................................................................................   88
PF07714.2    AAQGPTISYPMLLHVAAQIASGMRYLATLNFVHRDLATRNCLVGENFTIKIADFGMSRNLYAGDYYRVQGRAVLPIRWMAWECILMGKFTTASDVWAFGVTLWEVLMLCR  194
PF00754.7    ..............................................................................................................   76
DDR1-229     ..............................................................................................................  147
DDR1-262     AAQGPTISYPMLLHVAAQIASGMRYLATLNFVHRDLATRNCLVGENFTIKIADFGMSRNLYAGDYYRVQGRAVLPIRWMAWECILMGKFTTASDVWAFGVTLWEVLMLCR  822
DDR1-234     ..............................................................................................................  196
DDR1-250     ..............................................................................................................   35
DDR1-277     ..............................................................................................................   69
DDR1-245     AAQGPTISYPMLLHVAAQIASGMRYLATLNFVHRDLATRNCLVGENFTIKIADFGMSRNLYAGDYYRVQGRAVLPIRWMAWECILMGKFTTASDVWAFGVTLWEVLMLCR  198
DDR1-244     ..............................................................................................................  243
DDR1-259     ..............................................................................................................   70
DDR1-248     ..............................................................................................................   51
DDR1-274     ..............................................................................................................   35
DDR1-253     ..............................................................................................................   73
DDR1-265     ..............................................................................................................  114
DDR1-231     AAQGPTISYPMLLHVAAQIASGMRYLATLN................................................................................  493
DDR1-254     ..............................................................................................................  122
DDR1-228     ..............................................................................................................  243
DDR1-240     AAQGPTISYPMLLHVAAQIASGMRYLATLNFVHRDLATRNCLVGENFTIKIADFGMSRNLYAGDYYRVQGRAVLPIRWMAWECILMGKFTTASDVWAFGVTLWEVLMLCR  804
DDR1-223     AAQGPTISYPMLLHVAAQIASGMRYLATLNFVHRDLATRNCLVGENFTIKIADFGMSRNLYAGDYYRVQGRAVLPIRWMAWECILMGKFTTASDVWAFGVTLWEVLMLCR  841
DDR1-251     ..............................................................................................................   41
DDR1-280     ..............................................................................................................   85
DDR1-275     ..............................................................................................................  172
DDR1-266     ..............................................................................................................  142
DDR1-271     AAQGPTISYPMLLHVAAQIASGMRYLATLNFVHRDLATRNCLVGENFTIKIADFGMSRNLYAGDYYRVQGRAVLPIRWMAWECILMGKFTTASDVWAFGVTLWEVLMLCR  847
DDR1-247     ..............................................................................................................   55
DDR1-269     ..............................................................................................................  138
DDR1-237     ..............................................................................................................  153
DDR1-238     AAQGPTISYPMLLHVAAQIASGMRYLATLNFVHRDLATRNCLVGENFTIKIADFGMSRNLYAGDYYRVQGRAVLPIRWMAWECILMGKFTTASDVWAFGVTLWEVLMLCR  695
DDR1-233     ..............................................................................................................  209
DDR1-255     ..............................................................................................................  160
DDR1-226     AAQGPTISYPMLLHVAAQIASGMRYLATLNFVHRDLATRNCLVGENFTIKIADFGMSRNLYAGDYYRVQGRAVLPIRWMAWECILMGKFTTASDVWAFGVTLWEVLMLCR  804
DDR1-256     ..............................................................................................................  132
DDR1-263     ..............................................................................................................  126
DDR1-268     ..............................................................................................................  170
DDR1-239     AAQGPTISYPMLLHVAAQIASGMRYLATLNFVHRDLATRNCLVGENFTIKIADFGMSRNLYAGDYYRVQGRAVLPIRWMAWECILMGKFTTASDVWAFGVTLWEVLMLCR  841
DDR1-270     ..............................................................................................................   51
DDR1-267     ..............................................................................................................  112
DDR1-261     ..............................................................................................................   78
DDR1-241     ..............................................................................................................  286
DDR1-225     AAQGPTISYPMLLHVAAQIASGMRYLATLNFVHRDLATRNCLVGENFTIKIADFGMSRNLYAGDYYRVQGRAVLPIRWMAWECILMGKFTTASDVWAFGVTLWEVLMLCR  841
DDR1-257     ..............................................................................................................  100
DDR1-235     ..............................................................................................................  267
DDR1-230     ..............................................................................................................   58
```

```
logo
             AQPFGQLTDEQVIENAGEFFRDQGRQVYLSRPPACPQGLYELMLRCWSRESEQRPPFSQLHRFLAEDALNTV
IMATINIB     .........................................................................................   19
PF00754.6    .........................................................................................  123
PF00754.9    .........................................................................................  113
PF07714.1    .........................................................................................  150
PF00754.2    .........................................................................................  102
PF07714.3    AQPFGQLTDEQVIENA.........................................................................  210
PF00754.3    .........................................................................................  119
PF00754      .........................................................................................  136
PF07714      AQPFGQLTDEQVIENAGEFFRDQGRQVYLSRPPACPQGLYELMLRCWSRESEQRPPFSQLHRFL.........................  296
PF07714.5    AQPFGQLTDEQVIENAGEFFRDQGRQVYLSRPPACPQGLYELMLRCWSRESEQRPPFSQLHRFL.........................  302
PF00754.8    .........................................................................................   80
PF00754.4    .........................................................................................   84
PF07714.4    .........................................................................................   69
PF00754.1    .........................................................................................  100
PF00754.5    .........................................................................................   88
PF07714.2    AQPFGQLTDEQVIENAGEFFRDQGRQVYLSRPPACPQGLYELMLRCWSRESEQRPPFSQLHRFL.........................  258
PF00754.7    .........................................................................................   76
DDR1-229     .........................................................................................  147
DDR1-262     AQPFGQLTDEQVIENAGEFFRDQGRQVYLSRPPACPQGLYELMLRCWSRESEQRPPFSQLHRFLAEDALNTV.................  894
DDR1-234     .........................................................................................  196
DDR1-250     .........................................................................................   35
DDR1-277     .........................................................................................   69
DDR1-245     AQPFGQLTDEQVIENAGEFFRDQGRQVRVEEREDGSEAGDR..........RGRVVILETKEYLFPDSHPHCHNAGVPVPAACLPAGPI  277
DDR1-244     .........................................................................................  243
DDR1-259     .........................................................................................   70
DDR1-248     .........................................................................................   51
DDR1-274     .........................................................................................   35
DDR1-253     .........................................................................................   73
DDR1-265     .........................................................................................  114
DDR1-231     .........................................................................................  493
DDR1-254     .........................................................................................  122
DDR1-228     .........................................................................................  243
DDR1-240     AQPFGQLTDEQVIENAGEFFRDQGRQVYLSRPPACPQGLYELMLRCWSRESEQRPPFSQLHRFLAEDALNTV.................  876
DDR1-223     AQPFGQLTDEQVIENAGEFFRDQGRQVYLSRPPACPQGLYELMLRCWSRESEQRPPFSQLHRFLAEDALNTV.................  913
DDR1-251     .........................................................................................   41
DDR1-280     .........................................................................................   85
DDR1-275     .........................................................................................  172
DDR1-266     .........................................................................................  142
DDR1-271     AQPFGQLTDEQVIENAGEFFRDQGRQVYLSRPPACPQGLYELMLRCWSRESEQRPPFSQLHRFLAEDALNTV.................  919
DDR1-247     .........................................................................................   55
DDR1-269     .........................................................................................  138
DDR1-237     .........................................................................................  153
DDR1-238     AQPFGQLTDEQVIENAGEFFRDQGRQVYLSRPPACPQGLYELMLRCWSRESEQRPPFSQLHRFLAEDALNTV.................  767
DDR1-233     .........................................................................................  209
DDR1-255     .........................................................................................  160
DDR1-226     AQPFGQLTDEQVIENAGEFFRDQGRQVYLSRPPACPQGLYELMLRCWSRESEQRPPFSQLHRFLAEDALNTV.................  876
DDR1-256     .........................................................................................  132
DDR1-263     .........................................................................................  126
DDR1-268     .........................................................................................  170
DDR1-239     AQPFGQLTDEQVIENAGEFFRDQGRQVYLSRPPACPQGLYELMLRCWSRESEQRPPFSQLHRFLAEDALNTV.................  913
DDR1-270     .........................................................................................   51
DDR1-267     .........................................................................................  112
DDR1-261     .........................................................................................   78
DDR1-241     .........................................................................................  286
DDR1-225     AQPFGQLTDEQVIENAGEFFRDQGRQVYLSRPPACPQGLYELMLRCWSRESEQRPPFSQLHRFLAEDALNTV.................  913
DDR1-257     .........................................................................................  100
DDR1-235     .........................................................................................  267
DDR1-230     .........................................................................................   58
```

X non conserved
X similar
X $\geq 0\%$ conserved
X $\geq 50\%$ conserved

logo

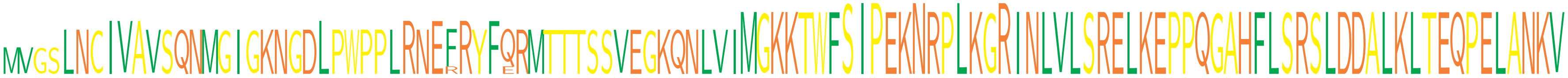

```
METHOTREXATE  .......IVA............L.....R.ERR.FE.......................SIP..N..L..R.......................................   16
PF00186.1     ....................................................MGKKTWFSIPEKNRPLKGRINLVLSRELKEPPQGAHFLSRSLDDALKLTEQPELANKV   58
PF00186       ....LNCIVAVSQNMGIGKNGDLPWPPLRNEFRYFQRMTTTSSVEGKQNLVIMGKKTWFSIPEKNRPLKGRINLVLSRELKEPPQGAHFLSRSLDDALKLTEQPELANKV  106
PF00186.2     ....LNCIVAVSQNMGIGKNGDLPWPPLRNEFRYFQRMTTTSSVEGKQNLVIMGKKTWFSIPEKNRPLKGRINLVLSRELKEPPQGAHFLSRSLDDALKLTEQPELANKV  106
DHFR-205      MVGSLNCIVAVSQNMGIGKNGDLPWPPLRNEFRYFQRMTTTSSVEGKQNLVIMGKKTWFSIPEKNRPLKGRINLVLSRELKEPPQGAHFLSRSLDDALKLTEQPELANKV  110
DHFR-201      MVGSLNCIVAVSQNMGIGKNGDLPWPPLRNEFRYFQRMTTTSSVEGKQNLVIMGKKTWFSIPEKNRPLKGRINLVLSRELKEPPQGAHFLSRSLDDALKLTEQPELANKV  110
DHFR-203      MVGSLNCIVAVSQNMGIGKNGDLPWPPLRNEFRYFQRMTTTSSVEGKQNLVIMGKKTWFSIPEKNRPLKGRINLVLSRELKEPPQGAHFLSRSLDDALKLTEQPELANKV  110
DHFR-202      ....................................................MGKKTWFSIPEKNRPLKGRINLVLSRELKEPPQGAHFLSRSLDDALKLTEQPELANKV   58
```

logo

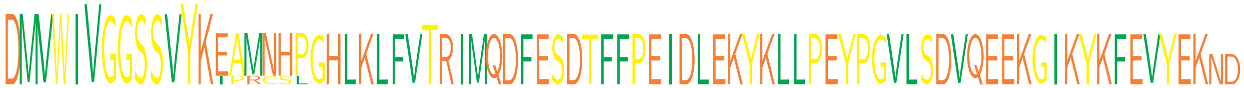

```
METHOTREXATE  .....V.....Y..............T..................................................   19
PF00186.1     DMVWIVGGSSVYKEAMNHPGHLKLFVTRIMQDFESDTFFPEIDLEKYKLLPEYPGVLSDVQEEKGIKYKFEVYEK..  133
PF00186       DMVWIVGGSSVYKEAMNHPGHLKLFVTRIMQDFESDTFFPEIDLEKYKLLPEYPGVLSDVQEEKGIKYKFEVYEK..  181
PF00186.2     DMVWIVGGSSVYK................................................................  119
DHFR-205      DMVWIVGGSSVYKIPRCSL..........................................................  129
DHFR-201      DMVWIVGGSSVYKEAMNHPGHLKLFVTRIMQDFESDTFFPEIDLEKYKLLPEYPGVLSDVQEEKGIKYKFEVYEKND  187
DHFR-203      DMVWIVGGSSVYKEAMNHPGHLKLFVTRIMQDFESDTFFPEIDLEKYKLLPEYPGVLSDVQEEKGIKYKFEVYEKND  187
DHFR-202      DMVWIVGGSSVYKEAMNHPGHLKLFVTRIMQDFESDTFFPEIDLEKYKLLPEYPGVLSDVQEEKGIKYKFEVYEKND  135
```

- X non conserved
- X similar
- X $\geq 0\%$ conserved
- X $\geq 50\%$ conserved

logo

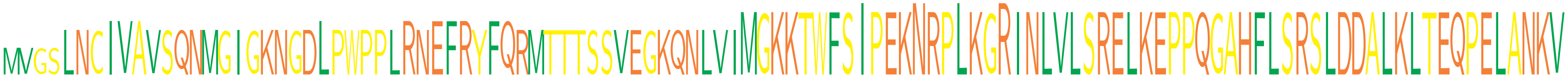

```
TALOTREXIN  .......IVA............L.....R.EFR.FQ........................I...N..L..R.......................................  14
PF00186.1   ....................................................MGKKTWFSIPEKNRPLKGRINLVLSRELKEPPQGAHFLSRSLDDALKLTEQPELANKV  58
PF00186     ....LNCIVAVSQNMGIGKNGDLPWPPLRNEFRYFQRMTTTSSVEGKQNLVIMGKKTWFSIPEKNRPLKGRINLVLSRELKEPPQGAHFLSRSLDDALKLTEQPELANKV  106
PF00186.2   ....LNCIVAVSQNMGIGKNGDLPWPPLRNEFRYFQRMTTTSSVEGKQNLVIMGKKTWFSIPEKNRPLKGRINLVLSRELKEPPQGAHFLSRSLDDALKLTEQPELANKV  106
DHFR-205    MVGSLNCIVAVSQNMGIGKNGDLPWPPLRNEFRYFQRMTTTSSVEGKQNLVIMGKKTWFSIPEKNRPLKGRINLVLSRELKEPPQGAHFLSRSLDDALKLTEQPELANKV  110
DHFR-201    MVGSLNCIVAVSQNMGIGKNGDLPWPPLRNEFRYFQRMTTTSSVEGKQNLVIMGKKTWFSIPEKNRPLKGRINLVLSRELKEPPQGAHFLSRSLDDALKLTEQPELANKV  110
DHFR-203    MVGSLNCIVAVSQNMGIGKNGDLPWPPLRNEFRYFQRMTTTSSVEGKQNLVIMGKKTWFSIPEKNRPLKGRINLVLSRELKEPPQGAHFLSRSLDDALKLTEQPELANKV  110
DHFR-202    ....................................................MGKKTWFSIPEKNRPLKGRINLVLSRELKEPPQGAHFLSRSLDDALKLTEQPELANKV  58
```

logo

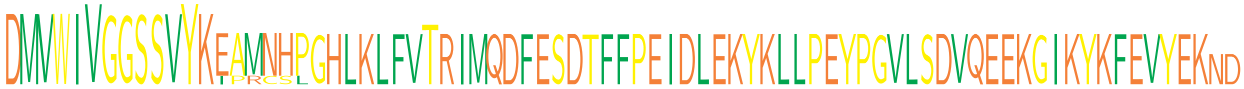

```
TALOTREXIN  .....V.....Y..............T..................................................  17
PF00186.1   DMVWIVGGSSVYKEAMNHPGHLKLFVTRIMQDFESDTFFPEIDLEKYKLLPEYPGVLSDVQEEKGIKYKFEVYEK..  133
PF00186     DMVWIVGGSSVYKEAMNHPGHLKLFVTRIMQDFESDTFFPEIDLEKYKLLPEYPGVLSDVQEEKGIKYKFEVYEK..  181
PF00186.2   DMVWIVGGSSVYK................................................................  119
DHFR-205    DMVWIVGGSSVYKIPRCSL..........................................................  129
DHFR-201    DMVWIVGGSSVYKEAMNHPGHLKLFVTRIMQDFESDTFFPEIDLEKYKLLPEYPGVLSDVQEEKGIKYKFEVYEKND  187
DHFR-203    DMVWIVGGSSVYKEAMNHPGHLKLFVTRIMQDFESDTFFPEIDLEKYKLLPEYPGVLSDVQEEKGIKYKFEVYEKND  187
DHFR-202    DMVWIVGGSSVYKEAMNHPGHLKLFVTRIMQDFESDTFFPEIDLEKYKLLPEYPGVLSDVQEEKGIKYKFEVYEKND  135
```

- X non conserved
- X similar
- X $\geq$ 0% conserved
- X $\geq$ 50% conserved

```
logo        MRPSGTAGAALLALLAALCPASRALEEKKVCQGTSNKLTQLGTFEDHFLSLQRMFNNCEVVLGNLEITYVQRNYDLSFLKTIQEVAGYVLIALNTVERIPLENLQIIRGN
AEE-788     ..............................................................................................................    0
PF01030     ........................................................NCEVVLGNLEITYVQRNYDLSFLKTIQEVAGYVLIALNTVERIPLENLQIIRGN   54
PF00757     ..............................................................................................................    0
PF14843.2   ..............................................................................................................    0
PF07714     ..............................................................................................................    0
PF14843.1   ..............................................................................................................    0
PF14843     ..............................................................................................................    0
PF01030.2   ........................................................NCEVVLGNLEITYVQRNYDLSFLKTIQEVAGYVLIALNTVERIPLENLQIIRGN   54
PF01030.1   ........................................................NCEVVLGNLEITYVQRNYDLSFLKTIQEVAGYVLIALNTVERIPLENLQIIRGN   54
PF00757.1   ..............................................................................................................    0
EGFR-206    MRPSGTAGAALLALLAALCPASRALEEKKVCQGTSNKLTQLGTFEDHFLSLQRMFNNCEVVLGNLEITYVQRNYDLSFLKTIQEVAGYVLIALNTVERIPLENLQIIRGN  110
EGFR-203    MRPSGTAGAALLALLAALCPASRALEEKKVCQGTSNKLTQLGTFEDHFLSLQRMFNNCEVVLGNLEITYVQRNYDLSFLKTIQEVAGYVLIALNTVERIPLENLQIIRGN  110
EGFR-207    MRPSGTAGAALLALLAALCPASRALEEKKVCQGTSNKLTQLGTFEDHFLSLQRMFNNCEVVLGNLEITYVQRNYDLSFLKTIQEVAGYVLIALNTVERIPLENLQIIRGN  110
EGFR-205    .....................................................MFNNCEVVLGNLEITYVQRNYDLSFLKTIQEVAGYVLIALNTVERIPLENLQIIRGN   57
EGFR-202    MRPSGTAGAALLALLAALCPASRALEEKKVCQGTSNKLTQLGTFEDHFLSLQRMFNNCEVVLGNLEITYVQRNYDLSFLKTIQEVAGYVLIALNTVERIPLENLQIIRGN  110
EGFR-204    MRPSGTAGAALLALLAALCPASRALEEKKVCQGTSNKLTQLGTFEDHFLSLQRMFNNCEVVLGNLEITYVQRNYDLSFLKTIQEVAGYVLIALNTVERIPLENLQIIRGN  110
EGFR-201    MRPSGTAGAALLALLAALCPASRALEEKKVCQGTSNKLTQLGTFEDHFLSLQRMFNNCEVVLGNLEITYVQRNYDLSFLKTIQEVAGYVLIALNTVERIPLENLQIIRGN  110

logo        MYYENSYALAVLSNYDANKTGLKELPMRNLQEILHGAVRFSNNPALCNVESIQWRDIVSSDFLSNMSMDFQNHLGSCQKCDPSCPNGSCWGAGEENCQKLTKIICAQQCS
AEE-788     ..............................................................................................................    0
PF01030     MYYENSYALAVLSNYDANKTGLKELPMRNLQEILHGAVRFSNNPALCNVESIQWRDI.....................................................  111
PF00757     ............................................................................GSCQKCDPSCPNGSCWGAGEENCQKLTKIICAQQCS   36
PF14843.2   ..............................................................................................................    0
PF07714     ..............................................................................................................    0
PF14843.1   ..............................................................................................................    0
PF14843     ..............................................................................................................    0
PF01030.2   MYYENSYALAVLSNYDANKTGLKELPMRNLQEILHGAVRFSNNPALCNVESIQWRDI.....................................................  111
PF01030.1   MYYENSYALAVLSNYDANKTGLKELPMRNLQ...............................................................................   85
PF00757.1   ..............................QG.............................................QKCDPSCPNGSCWGAGEENCQKLTKIICAQQCS   35
EGFR-206    MYYENSYALAVLSNYDANKTGLKELPMRNLQG.............................................QKCDPSCPNGSCWGAGEENCQKLTKIICAQQCS  175
EGFR-203    MYYENSYALAVLSNYDANKTGLKELPMRNLQEILHGAVRFSNNPALCNVESIQWRDIVSSDFLSNMSMDFQNHLGSCQKCDPSCPNGSCWGAGEENCQKLTKIICAQQCS  220
EGFR-207    MYYENSYALAVLSNYDANKTGLKELPMRNLQG.............................................QKCDPSCPNGSCWGAGEENCQKLTKIICAQQCS  175
EGFR-205    MYYENSYALAVLSNYDANKTGLKELPMRNLQEILHGAVRFSNNPALCNVESIQWRDIVSSDFLSNMSMDFQ.......................................  128
EGFR-202    MYYENSYALAVLSNYDANKTGLKELPMRNLQEILHGAVRFSNNPALCNVESIQWRDIVSSDFLSNMSMDFQNHLGSCQKCDPSCPNGSCWGAGEENCQKLTKIICAQQCS  220
EGFR-204    MYYENSYALAVLSNYDANKTGLKELPMRNLQEILHGAVRFSNNPALCNVESIQWRDIVSSDFLSNMSMDFQNHLGSCQKCDPSCPNGSCWGAGEENCQKLTKIICAQQCS  220
EGFR-201    MYYENSYALAVLSNYDANKTGLKELPMRNLQEILHGAVRFSNNPALCNVESIQWRDIVSSDFLSNMSMDFQNHLGSCQKCDPSCPNGSCWGAGEENCQKLTKIICAQQCS  220
```

```
logo        GRCRGKSPSDCCHNQCAAGCTGPRESDCLVCRKFRDEATCKDTCPPLMLYNPTTYQMDVNPEGKYSFGATCVKKCPRNYVVTDHGSCVRACGADSYEMEEDGVRKCKKCE
AEE-788     ..............................................................................................................    0
PF01030     ..............................................................................................................  111
PF00757     GRCRGKSPSDCCHNQCAAGCTGPRESDCLVCRKFRDEATCKDTCPPLMLYNPTTYQMDVNPEGKYSFGATCVKKCPRNYVVTDHGSCVRACGADSYEMEEDGVRKCKKCE  146
PF14843.2   ..............................................................................................................    0
PF07714     ..............................................................................................................    0
PF14843.1   ..............................................................................................................    0
PF14843     ..............................................................................................................    0
PF01030.2   ..............................................................................................................  111
PF01030.1   ..............................................................................................................   85
PF00757.1   GRCRGKSPSDCCHNQCAAGCTGPRESDCLVCRKFRDEATCKDTCPPLMLYNPTTYQMDVNPEGKYSFGATCVKKCPRNYVVTDHGSCVRACGADSYEMEEDGVRKCKKCE  145
EGFR-206    GRCRGKSPSDCCHNQCAAGCTGPRESDCLVCRKFRDEATCKDTCPPLMLYNPTTYQMDVNPEGKYSFGATCVKKCPRNYVVTDHGSCVRACGADSYEMEEDGVRKCKKCE  285
EGFR-203    GRCRGKSPSDCCHNQCAAGCTGPRESDCLVCRKFRDEATCKDTCPPLMLYNPTTYQMDVNPEGKYSFGATCVKKCPRNYVVTDHGSCVRACGADSYEMEEDGVRKCKKCE  330
EGFR-207    GRCRGKSPSDCCHNQCAAGCTGPRESDCLVCRKFRDEATCKDTCPPLMLYNPTTYQMDVNPEGKYSFGATCVKKCPRNYVVTDHGSCVRACGADSYEMEEDGVRKCKKCE  285
EGFR-205    ..............................................................................................................  128
EGFR-202    GRCRGKSPSDCCHNQCAAGCTGPRESDCLVCRKFRDEATCKDTCPPLMLYNPTTYQMDVNPEGKYSFGATCVKKCPRNYVVTDHGSCVRACGADSYEMEEDGVRKCKKCE  330
EGFR-204    GRCRGKSPSDCCHNQCAAGCTGPRESDCLVCRKFRDEATCKDTCPPLMLYNPTTYQMDVNPEGKYSFGATCVKKCPRNYVVTDHGSCVRACGADSYEMEEDGVRKCKKCE  330
EGFR-201    GRCRGKSPSDCCHNQCAAGCTGPRESDCLVCRKFRDEATCKDTCPPLMLYNPTTYQMDVNPEGKYSFGATCVKKCPRNYVVTDHGSCVRACGADSYEMEEDGVRKCKKCE  330

logo        GPCRKVCNGIGIGEFKDSLSINATNIKHFKNCTSISGDLHILPVAFRGDSFTHTPPLDPQELDILKTVKEITGFLLIQAWPENRTDLHAFENLEIIRGRTKQHGQFSLAV
AEE-788     ..............................................................................................................    0
PF01030     ..............................NCTSISGDLHILPVAFRGDSFTHTPPLDPQELDILKTVKEITGFLLIQAWPENRTDLHAFENLEIIRGRTKQHGQFSLAV  191
PF00757     GPCRKVCN......................................................................................................  154
PF14843.2   ..............................................................................................................    0
PF07714     ..............................................................................................................    0
PF14843.1   ..............................................................................................................    0
PF14843     ..............................................................................................................    0
PF01030.2   ..............................................................................................................  111
PF01030.1   ..............................NCTSISGDLHILPVAFRGDSFTHTPPLDPQELDILKTVKEITGFLLIQAWPENRTDLHAFENLEIIRGRTKQHGQFSLAV  165
PF00757.1   GPCRKVCN......................................................................................................  153
EGFR-206    GPCRKVCNGIGIGEFKDSLSINATNIKHFKNCTSISGDLHILPVAFRGDSFTHTPPLDPQELDILKTVKEITGFLLIQAWPENRTDLHAFENLEIIRGRTKQHGQFSLAV  395
EGFR-203    GPCRKVCNGIGIGEFKDSLSINATNIKHFKNCTSISGDLHILPVAFRGDSFTHTPPLDPQELDILKTVKEITGFLLIQAWPENRTDLHAFENLEIIRGRTKQHGQFSLAV  440
EGFR-207    GPCRKVCNGIGIGEFKDSLSINATNIKHFKNCTSISGDLHILPVAFRGDSFTHTPPLDPQELDILKTVKEITGFLLIQAWPENRTDLHAFENLEIIRGRTKQHGQFSLAV  395
EGFR-205    ..............................................................................................................  128
EGFR-202    GPCRKVCNGIGIGEFKDSLSINATNIKHFKNCTSISGDLHILPVAFRGDSFTHTPPLDPQELDILKTVKEITGFLLIQAWPENRTDLHAFENLEIIRGRTKQHGQFSLAV  440
EGFR-204    GPCRKVCNGIGIGEFKDSLSINATNIKHFKNCTSISGDLHILPVAFRGDSFTHTPPLDPQELDILKTVKEITGLS...................................  405
EGFR-201    GPCRKVCNGIGIGEFKDSLSINATNIKHFKNCTSISGDLHILPVAFRGDSFTHTPPLDPQELDILKTVKEITGFLLIQAWPENRTDLHAFENLEIIRGRTKQHGQFSLAV  440
```

```
logo        VSLNITSLGLRSLKEISDGDVIISGNKNLCYANTINWKKLFGTSGQKTKIISNRGENSCKATGQVCHALCSPEGCWGPEPRDCVSCRNVSRGRECVDKCNLLEGEPREFV
AEE-788     ..............................................................................................................    0
PF01030     VSLNITSLGLRSLKEISDGDVIISGNKNLCYANTINWKKL......................................................................  231
PF00757     ..............................................................................................................  154
PF14843.2   ................................................................VCHALCSPEGCWGPEPRDCVSCRNVSRGRECVDKCNLLEGEPREFV   46
PF07714     ..............................................................................................................    0
PF14843.1   ................................................................VCHALCSPEGCWGPEPRDCVSCRNVSRGRECVDKCNLLEGEPREFV   46
PF14843     ................................................................VCHALCSPEGCWGPEPRDCVSCRNVSRGRECVDKCNLLEGEPREFV   46
PF01030.2   ..............................................................................................................  111
PF01030.1   VSLNITSLGLRSLKEISDGDVIISGNKNLCYANTINWKKL......................................................................  205
PF00757.1   ..............................................................................................................  153
EGFR-206    VSLNITSLGLRSLKEISDGDVIISGNKNLCYANTINWKKLFGTSGQKTKIISNRGENSCKATGQVCHALCSPEGCWGPEPRDCVSCRNVSRGRECVDKCNLLEGEPREFV  505
EGFR-203    VSLNITSLGLRSLKEISDGDVIISGNKNLCYANTINWKKLFGTSGQKTKIISNRGENSCKATGQVCHALCSPEGCWGPEPRDCVSCRNVSRGRECVDKCNLLEGEPREFV  550
EGFR-207    VSLNITSLGLRSLKEISDGDVIISGNKNLCYANTINWKKLFGTSGQKTKIISNRGENSCKATGQVCHALCSPEGCWGPEPRDCVSCRNVSRGRECVDKCNLLEGEPREFV  505
EGFR-205    ..............................................................................................................  128
EGFR-202    VSLNITSLGLRSLKEISDGDVIISGNKNLCYANTINWKKLFGTSGQKTKIISNRGENSCKATGQVCHALCSPEGCWGPEPRDCVSCRNVSRGRECVDKCNLLEGEPREFV  550
EGFR-204    ..............................................................................................................  405
EGFR-201    VSLNITSLGLRSLKEISDGDVIISGNKNLCYANTINWKKLFGTSGQKTKIISNRGENSCKATGQVCHALCSPEGCWGPEPRDCVSCRNVSRGRECVDKCNLLEGEPREFV  550

logo        ENSECIQCHPECLPQAMNITCTGRGPDNCIQCAHYIDGPHCVKTCPAGVMGENNTLVWKYADAGHVCHLCHPNCTYGCTGPGLEGCPTNGPKI
AEE-788     ..............................................................................................................    0
PF01030     ..............................................................................................................  231
PF00757     ..............................................................................................................  154
PF14843.2   ENSECIQCHPECLPQAMNITCTGRGPDNCIQCAHYIDGPHCVKTCPAGVMGENNTLVWKYADAGHVCHLCHPNCTYGPG...............................  125
PF07714     ..............................................................................................................    0
PF14843.1   ENSECIQCHPECLPQAMNITCTGRGPDNCIQCAHYIDGPHCVKTCPAGVMGENNTLVWKYADAGHVCHLCHPNCTYG.................................  123
PF14843     ENSECIQCHPECLPQAMNITCTGRGPDNCIQCAHYIDGPHCVKTCPAGVMGENNTLVWKYADAGHVCHLCHPNCTYGCTGPGLEGC........................  132
PF01030.2   ..............................................................................................................  111
PF01030.1   ..............................................................................................................  205
PF00757.1   ..............................................................................................................  153
EGFR-206    ENSECIQCHPECLPQAMNITCTGRGPDNCIQCAHYIDGPHCVKTCPAGVMGENNTLVWKYADAGHVCHLCHPNCTYGCTGPGLEGCPTNGPKI................  598
EGFR-203    ENSECIQCHPECLPQAMNITCTGRGPDNCIQCAHYIDGPHCVKTCPAGVMGENNTLVWKYADAGHVCHLCHPNCTYGPGNESLKAMLFCLFKLSSCNQSNDGSVSHQSGS  660
EGFR-207    ENSECIQCHPECLPQAMNITCTGRGPDNCIQCAHYIDGPHCVKTCPAGVMGENNTLVWKYADAGHVCHLCHPNCTYGCTGPGLEGCPTNGPKI................  598
EGFR-205    ..............................................................................................................  128
EGFR-202    ENSECIQCHPECLPQAMNITCTGRGPDNCIQCAHYIDGPHCVKTCPAGVMGENNTLVWKYADAGHVCHLCHPNCTYGS................................  628
EGFR-204    ..............................................................................................................  405
EGFR-201    ENSECIQCHPECLPQAMNITCTGRGPDNCIQCAHYIDGPHCVKTCPAGVMGENNTLVWKYADAGHVCHLCHPNCTYGCTGPGLEGCPTNGPKI................  643
```

```
logo
            PSIATGMVGALLLLLVVALGIGLFMRRRHIVRKRTLRRLLQERELVEPLTPSGEAPNQALLRILKETEFKKIKVLGSGAFGTVYKGLWIPEGEKVKIPVAIKELREATSP
AEE-788     ..........................................................................L.......V................AIK........    5
PF01030     ..............................................................................................................  231
PF00757     ..............................................................................................................  154
PF14843.2   ..............................................................................................................  125
PF07714     ......................................................................KIKVLGSGAFGTVYKGLWIPEGEKVKIPVAIKELREATSP   40
PF14843.1   ..............................................................................................................  123
PF14843     ..............................................................................................................  132
PF01030.2   ..............................................................................................................  111
PF01030.1   ..............................................................................................................  205
PF00757.1   ..............................................................................................................  153
EGFR-206    PSIATGMVGALLLLLVVALGIGLFMRRRHIVRKRTLRRLLQERELVEPLTPSGEAPNQALLRILKETEFKKIKVLGSGAFGTVYKGLWIPEGEKVKIPVAIKELREATSP  708
EGFR-203    PAAQESCLGWIPSLLPSEFQLG......................................................WGG..CSHLHAWPSASVIITASS...........  703
EGFR-207    PSIATGMVGALLLLLVVALGIGLFMRRRHIVRKRTLRRLLQERELVEPLTPSGEAPNQALLRILKETEFKKIKVLGSGAFGTVYKGLWIPEGEKVKIPVAIKELREATSP  708
EGFR-205    ..............................................................................................................  128
EGFR-202    ..............................................................................................................  628
EGFR-204    ..............................................................................................................  405
EGFR-201    PSIATGMVGALLLLLVVALGIGLFMRRRHIVRKRTLRRLLQERELVEPLTPSGEAPNQALLRILKETEFKKIKVLGSGAFGTVYKGLWIPEGEKVKIPVAIKELREATSP  753
```

```
logo
            KANKEILDEAYVMASVDNPHVCRLLGICLTSTVQLITQLMPFGCLLDYVREHKDNIGSQYLLNWCVQIAKGMNYLEDRRLVHRDLAARNVLVKTPQHVKITDFGLAKLLG
AEE-788     ............M.....................L.MQLMP.G...D...E.......................................L.........TD........   18
PF01030     ..............................................................................................................  231
PF00757     ..............................................................................................................  154
PF14843.2   ..............................................................................................................  125
PF07714     KANKEILDEAYVMASVDNPHVCRLLGICLTSTVQLITQLMPFGCLLDYVREHKDNIGSQYLLNWCVQIAKGMNYLEDRRLVHRDLAARNVLVKTPQHVKITDFGLAKLLG  150
PF14843.1   ..............................................................................................................  123
PF14843     ..............................................................................................................  132
PF01030.2   ..............................................................................................................  111
PF01030.1   ..............................................................................................................  205
PF00757.1   ..............................................................................................................  153
EGFR-206    KANKEILDEAYVMASVDNPHVCRLLGICLTSTVQLITQLMPFGCLLDYVREHKDNIGSQYLLNWCVQIAKGMNYLEDRRLVHRDLAARNVLVKTPQHVKITDFGLAKLLG  818
EGFR-203    .....................CH.......................................................................................  705
EGFR-207    KANKEILDEAYVMASVDNPHVCRLLGICLTSTVQLITQLMPFGCLLDYVREHKDNIGSQYLLNWCVQIAKGMNYLEDRRLVHRDLAARNVLVKTPQHVKITDFGLAKLLG  818
EGFR-205    ..............................................................................................................  128
EGFR-202    ..............................................................................................................  628
EGFR-204    ..............................................................................................................  405
EGFR-201    KANKEILDEAYVMASVDNPHVCRLLGICLTSTVQLITQLMPFGCLLDYVREHKDNIGSQYLLNWCVQIAKGMNYLEDRRLVHRDLAARNVLVKTPQHVKITDFGLAKLLG  863
```

```
logo
           AEEKEYHAEGGKVPIKWMALESILHRIYTHQSDVWSYGVTVWELMTFGSKPYDGIPASEISSILEKGERLPQPPICTIDVYMIMVKCWMIDADSRPKFRELIIEFSKMAR
AEE-788    ..............................................................................................................   18
PF01030    ..............................................................................................................  231
PF00757    ..............................................................................................................  154
PF14843.2  ..............................................................................................................  125
PF07714    AEEKEYHAEGGKVPIKWMALESILHRIYTHQSDVWSYGVTVWELMTFGSKPYDGIPASEISSILEKGERLPQPPICTIDVYMIMVKCWMIDADSRPKFRELI.........  252
PF14843.1  ..............................................................................................................  123
PF14843    ..............................................................................................................  132
PF01030.2  ..............................................................................................................  111
PF01030.1  ..............................................................................................................  205
PF00757.1  ..............................................................................................................  153
EGFR-206   AEEKEYHAEGGKVPIKWMALESILHRIYTHQSDVWSYGVTVWELMTFGSKPYDGIPASEISSILEKGERLPQPPICTIDVYMIMVKCWMIDADSRPKFRELIIEFSKMAR  928
EGFR-203   ..............................................................................................................  705
EGFR-207   AEEKEYHAEGGKVPIKWMALESILHRIYTHQSDVWSYGVTVWELMTFGSKPYDGIPASEISSILEKGERLPQPPICTIDVYMIMVKCWMIDADSRPKFRELIIEFSKMAR  928
EGFR-205   ..............................................................................................................  128
EGFR-202   ..............................................................................................................  628
EGFR-204   ..............................................................................................................  405
EGFR-201   AEEKEYHAEGGKVPIKWMALESILHRIYTHQSDVWSYGVTVWELMTFGSKPYDGIPASEISSILEKGERLPQPPICTIDVYMIMVKCWMIDADSRPKFRELIIEFSKMAR  973

logo
           DPQRYLVIQGDERMHLPSPTDSNFYRALMDEEDMDDVVDADEYLIPQQGFFSSPSTSRTPLLSSLSATSNNSTVACIDRNGLQSCPIKEDSFLQRYSSDPTGALTEDSID
AEE-788    ..............................................................................................................   18
PF01030    ..............................................................................................................  231
PF00757    ..............................................................................................................  154
PF14843.2  ..............................................................................................................  125
PF07714    ..............................................................................................................  252
PF14843.1  ..............................................................................................................  123
PF14843    ..............................................................................................................  132
PF01030.2  ..............................................................................................................  111
PF01030.1  ..............................................................................................................  205
PF00757.1  ..............................................................................................................  153
EGFR-206   DPQRYLVIQGDERMHLPSPTDSNFYRALMDEEDMDDVVDADEYLIPQQGFFSSPSTSRTPLLSSLSATSNNSTVACIDRNGLQSCPIKEDSFLQRYSSDPTGALTEDSID 1038
EGFR-203   ..............................................................................................................  705
EGFR-207   DPQRYLVIQGDERMHLPSPTDSNFYRALMDEEDMDDVVDADEYLIPQQGFFSSPSTSRTPLLSSLSATSNNSTVACIDRNGLQSCPIKEDSFLQRYSSDPTGALTEDSID 1038
EGFR-205   ..............................................................................................................  128
EGFR-202   ..............................................................................................................  628
EGFR-204   ..............................................................................................................  405
EGFR-201   DPQRYLVIQGDERMHLPSPTDSNFYRALMDEEDMDDVVDADEYLIPQQGFFSSPSTSRTPLLSSLSATSNNSTVACIDRNGLQSCPIKEDSFLQRYSSDPTGALTEDSID 1083
```

logo

```
            [illegible]
AEE-788     ..............................................................................................................  18
PF01030     ..............................................................................................................  231
PF00757     ..............................................................................................................  154
PF14843.2   ..............................................................................................................  125
PF07714     ..............................................................................................................  252
PF14843.1   ..............................................................................................................  123
PF14843     ..............................................................................................................  132
PF01030.2   ..............................................................................................................  111
PF01030.1   ..............................................................................................................  205
PF00757.1   ..............................................................................................................  153
EGFR-206    DTFLPVPEYINQSVP........KRPAGSVQNPVYHNQPLNPAPSRDPHYQDPHSTAVGNPEYLNTVQPTCVNSTFDSPAHWAQKGSHQISLDNPDYQQDFFPKEAKPNG  1140
EGFR-203    ..............................................................................................................  705
EGFR-207    DTFLPVPGEWLVWKQSCSSTSSTHSAAASLQCPSQVLPPASPEGETVADLQTQ.........................................................  1091
EGFR-205    ..............................................................................................................  128
EGFR-202    ..............................................................................................................  628
EGFR-204    ..............................................................................................................  405
EGFR-201    DTFLPVPEYINQSVP........KRPAGSVQNPVYHNQPLNPAPSRDPHYQDPHSTAVGNPEYLNTVQPTCVNSTFDSPAHWAQKGSHQISLDNPDYQQDFFPKEAKPNG  1185
```

logo

```
            IFKGSTAENAEYLRVAPQSSEFIGA
AEE-788     .........................    18
PF01030     .........................   231
PF00757     .........................   154
PF14843.2   .........................   125
PF07714     .........................   252
PF14843.1   .........................   123
PF14843     .........................   132
PF01030.2   .........................   111
PF01030.1   .........................   205
PF00757.1   .........................   153
EGFR-206    IFKGSTAENAEYLRVAPQSSEFIGA  1165
EGFR-203    .........................   705
EGFR-207    ......................... 1091
EGFR-205    .........................   128
EGFR-202    .........................   628
EGFR-204    .........................   405
EGFR-201    IFKGSTAENAEYLRVAPQSSEFIGA  1210
```

X non conserved
X similar
X $\geq 0\%$ conserved
X $\geq 50\%$ conserved

logo
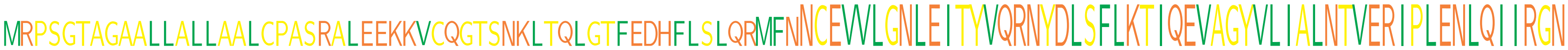

```
AFATINIB    ..............................................................................................................   0
PF01030     ........................................................NCEVVLGNLEITYVQRNYDLSFLKTIQEVAGYVLIALNTVERIPLENLQIIRGN  54
PF00757     ..............................................................................................................   0
PF14843.2   ..............................................................................................................   0
PF07714     ..............................................................................................................   0
PF14843.1   ..............................................................................................................   0
PF14843     ..............................................................................................................   0
PF01030.2   ........................................................NCEVVLGNLEITYVQRNYDLSFLKTIQEVAGYVLIALNTVERIPLENLQIIRGN  54
PF01030.1   ........................................................NCEVVLGNLEITYVQRNYDLSFLKTIQEVAGYVLIALNTVERIPLENLQIIRGN  54
PF00757.1   ..............................................................................................................   0
EGFR-206    MRPSGTAGAALLALLAALCPASRALEEKKVCQGTSNKLTQLGTFEDHFLSLQRMFNNCEVVLGNLEITYVQRNYDLSFLKTIQEVAGYVLIALNTVERIPLENLQIIRGN 110
EGFR-203    MRPSGTAGAALLALLAALCPASRALEEKKVCQGTSNKLTQLGTFEDHFLSLQRMFNNCEVVLGNLEITYVQRNYDLSFLKTIQEVAGYVLIALNTVERIPLENLQIIRGN 110
EGFR-207    MRPSGTAGAALLALLAALCPASRALEEKKVCQGTSNKLTQLGTFEDHFLSLQRMFNNCEVVLGNLEITYVQRNYDLSFLKTIQEVAGYVLIALNTVERIPLENLQIIRGN 110
EGFR-205    .....................................................MFNNCEVVLGNLEITYVQRNYDLSFLKTIQEVAGYVLIALNTVERIPLENLQIIRGN  57
EGFR-202    MRPSGTAGAALLALLAALCPASRALEEKKVCQGTSNKLTQLGTFEDHFLSLQRMFNNCEVVLGNLEITYVQRNYDLSFLKTIQEVAGYVLIALNTVERIPLENLQIIRGN 110
EGFR-204    MRPSGTAGAALLALLAALCPASRALEEKKVCQGTSNKLTQLGTFEDHFLSLQRMFNNCEVVLGNLEITYVQRNYDLSFLKTIQEVAGYVLIALNTVERIPLENLQIIRGN 110
EGFR-201    MRPSGTAGAALLALLAALCPASRALEEKKVCQGTSNKLTQLGTFEDHFLSLQRMFNNCEVVLGNLEITYVQRNYDLSFLKTIQEVAGYVLIALNTVERIPLENLQIIRGN 110
```

logo
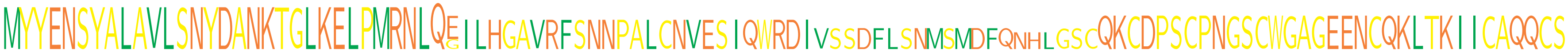

```
AFATINIB    ..............................................................................................................   0
PF01030     MYYENSYALAVLSNYDANKTGLKELPMRNLQEILHGAVRFSNNPALCNVESIQWRDI..................................................... 111
PF00757     ..........................................................................GSCQKCDPSCPNGSCWGAGEENCQKLTKIICAQQCS  36
PF14843.2   ..............................................................................................................   0
PF07714     ..............................................................................................................   0
PF14843.1   ..............................................................................................................   0
PF14843     ..............................................................................................................   0
PF01030.2   MYYENSYALAVLSNYDANKTGLKELPMRNLQEILHGAVRFSNNPALCNVESIQWRDI..................................................... 111
PF01030.1   MYYENSYALAVLSNYDANKTGLKELPMRNLQ...............................................................................  85
PF00757.1   ..............................QG.............................................QKCDPSCPNGSCWGAGEENCQKLTKIICAQQCS  35
EGFR-206    MYYENSYALAVLSNYDANKTGLKELPMRNLQG.............................................QKCDPSCPNGSCWGAGEENCQKLTKIICAQQCS 175
EGFR-203    MYYENSYALAVLSNYDANKTGLKELPMRNLQEILHGAVRFSNNPALCNVESIQWRDIVSSDFLSNMSMDFQNHLGSCQKCDPSCPNGSCWGAGEENCQKLTKIICAQQCS 220
EGFR-207    MYYENSYALAVLSNYDANKTGLKELPMRNLQG.............................................QKCDPSCPNGSCWGAGEENCQKLTKIICAQQCS 175
EGFR-205    MYYENSYALAVLSNYDANKTGLKELPMRNLQEILHGAVRFSNNPALCNVESIQWRDIVSSDFLSNMSMDFQ....................................... 128
EGFR-202    MYYENSYALAVLSNYDANKTGLKELPMRNLQEILHGAVRFSNNPALCNVESIQWRDIVSSDFLSNMSMDFQNHLGSCQKCDPSCPNGSCWGAGEENCQKLTKIICAQQCS 220
EGFR-204    MYYENSYALAVLSNYDANKTGLKELPMRNLQEILHGAVRFSNNPALCNVESIQWRDIVSSDFLSNMSMDFQNHLGSCQKCDPSCPNGSCWGAGEENCQKLTKIICAQQCS 220
EGFR-201    MYYENSYALAVLSNYDANKTGLKELPMRNLQEILHGAVRFSNNPALCNVESIQWRDIVSSDFLSNMSMDFQNHLGSCQKCDPSCPNGSCWGAGEENCQKLTKIICAQQCS 220
```

```
logo
            GRCRGKSPSDCCHNQCAAGCTGPRESDCLVCRKFRDEATCKDTCPPLMLYNPTTYQMDVNPEGKYSFGATCVKKCPRNYVVTDHGSCVRACGADSYEMEEDGVRKCKKCE
AFATINIB    ..............................................................................................................     0
PF01030     ..............................................................................................................   111
PF00757     GRCRGKSPSDCCHNQCAAGCTGPRESDCLVCRKFRDEATCKDTCPPLMLYNPTTYQMDVNPEGKYSFGATCVKKCPRNYVVTDHGSCVRACGADSYEMEEDGVRKCKKCE   146
PF14843.2   ..............................................................................................................     0
PF07714     ..............................................................................................................     0
PF14843.1   ..............................................................................................................     0
PF14843     ..............................................................................................................     0
PF01030.2   ..............................................................................................................   111
PF01030.1   ..............................................................................................................    85
PF00757.1   GRCRGKSPSDCCHNQCAAGCTGPRESDCLVCRKFRDEATCKDTCPPLMLYNPTTYQMDVNPEGKYSFGATCVKKCPRNYVVTDHGSCVRACGADSYEMEEDGVRKCKKCE   145
EGFR-206    GRCRGKSPSDCCHNQCAAGCTGPRESDCLVCRKFRDEATCKDTCPPLMLYNPTTYQMDVNPEGKYSFGATCVKKCPRNYVVTDHGSCVRACGADSYEMEEDGVRKCKKCE   285
EGFR-203    GRCRGKSPSDCCHNQCAAGCTGPRESDCLVCRKFRDEATCKDTCPPLMLYNPTTYQMDVNPEGKYSFGATCVKKCPRNYVVTDHGSCVRACGADSYEMEEDGVRKCKKCE   330
EGFR-207    GRCRGKSPSDCCHNQCAAGCTGPRESDCLVCRKFRDEATCKDTCPPLMLYNPTTYQMDVNPEGKYSFGATCVKKCPRNYVVTDHGSCVRACGADSYEMEEDGVRKCKKCE   285
EGFR-205    ..............................................................................................................   128
EGFR-202    GRCRGKSPSDCCHNQCAAGCTGPRESDCLVCRKFRDEATCKDTCPPLMLYNPTTYQMDVNPEGKYSFGATCVKKCPRNYVVTDHGSCVRACGADSYEMEEDGVRKCKKCE   330
EGFR-204    GRCRGKSPSDCCHNQCAAGCTGPRESDCLVCRKFRDEATCKDTCPPLMLYNPTTYQMDVNPEGKYSFGATCVKKCPRNYVVTDHGSCVRACGADSYEMEEDGVRKCKKCE   330
EGFR-201    GRCRGKSPSDCCHNQCAAGCTGPRESDCLVCRKFRDEATCKDTCPPLMLYNPTTYQMDVNPEGKYSFGATCVKKCPRNYVVTDHGSCVRACGADSYEMEEDGVRKCKKCE   330
```

```
logo
            GPCRKVCNGIGIGEFKDSLSINATNIKHFKNCTSISGDLHILPVAFRGDSFTHTPPLDPQELDILKTVKEITGFLLIQAWPENRTDLHAFENLEIIRGRTKQHGQFSLAV
AFATINIB    ..............................................................................................................     0
PF01030     ..............................NCTSISGDLHILPVAFRGDSFTHTPPLDPQELDILKTVKEITGFLLIQAWPENRTDLHAFENLEIIRGRTKQHGQFSLAV   191
PF00757     GPCRKVCN......................................................................................................   154
PF14843.2   ..............................................................................................................     0
PF07714     ..............................................................................................................     0
PF14843.1   ..............................................................................................................     0
PF14843     ..............................................................................................................     0
PF01030.2   ..............................................................................................................   111
PF01030.1   ..............................NCTSISGDLHILPVAFRGDSFTHTPPLDPQELDILKTVKEITGFLLIQAWPENRTDLHAFENLEIIRGRTKQHGQFSLAV   165
PF00757.1   GPCRKVCN......................................................................................................   153
EGFR-206    GPCRKVCNGIGIGEFKDSLSINATNIKHFKNCTSISGDLHILPVAFRGDSFTHTPPLDPQELDILKTVKEITGFLLIQAWPENRTDLHAFENLEIIRGRTKQHGQFSLAV   395
EGFR-203    GPCRKVCNGIGIGEFKDSLSINATNIKHFKNCTSISGDLHILPVAFRGDSFTHTPPLDPQELDILKTVKEITGFLLIQAWPENRTDLHAFENLEIIRGRTKQHGQFSLAV   440
EGFR-207    GPCRKVCNGIGIGEFKDSLSINATNIKHFKNCTSISGDLHILPVAFRGDSFTHTPPLDPQELDILKTVKEITGFLLIQAWPENRTDLHAFENLEIIRGRTKQHGQFSLAV   395
EGFR-205    ..............................................................................................................   128
EGFR-202    GPCRKVCNGIGIGEFKDSLSINATNIKHFKNCTSISGDLHILPVAFRGDSFTHTPPLDPQELDILKTVKEITGFLLIQAWPENRTDLHAFENLEIIRGRTKQHGQFSLAV   440
EGFR-204    GPCRKVCNGIGIGEFKDSLSINATNIKHFKNCTSISGDLHILPVAFRGDSFTHTPPLDPQELDILKTVKEITGLS...................................   405
EGFR-201    GPCRKVCNGIGIGEFKDSLSINATNIKHFKNCTSISGDLHILPVAFRGDSFTHTPPLDPQELDILKTVKEITGFLLIQAWPENRTDLHAFENLEIIRGRTKQHGQFSLAV   440
```

```
logo         VSLNITSLGLRSLKEISDGDVIISGNKNLCYANTINWKKLFGTSGQKTKIISNRGENSCKATGQVCHALCSPEGCWGPEPRDCVSCRNVSRGRECVDKCNLLEGEPREFV
AFATINIB     ............................................................................................................   0
PF01030      VSLNITSLGLRSLKEISDGDVIISGNKNLCYANTINWKKL....................................................................   231
PF00757      ............................................................................................................   154
PF14843.2    ................................................................VCHALCSPEGCWGPEPRDCVSCRNVSRGRECVDKCNLLEGEPREFV   46
PF07714      ............................................................................................................   0
PF14843.1    ................................................................VCHALCSPEGCWGPEPRDCVSCRNVSRGRECVDKCNLLEGEPREFV   46
PF14843      ................................................................VCHALCSPEGCWGPEPRDCVSCRNVSRGRECVDKCNLLEGEPREFV   46
PF01030.2    ............................................................................................................   111
PF01030.1    VSLNITSLGLRSLKEISDGDVIISGNKNLCYANTINWKKL....................................................................   205
PF00757.1    ............................................................................................................   153
EGFR-206     VSLNITSLGLRSLKEISDGDVIISGNKNLCYANTINWKKLFGTSGQKTKIISNRGENSCKATGQVCHALCSPEGCWGPEPRDCVSCRNVSRGRECVDKCNLLEGEPREFV   505
EGFR-203     VSLNITSLGLRSLKEISDGDVIISGNKNLCYANTINWKKLFGTSGQKTKIISNRGENSCKATGQVCHALCSPEGCWGPEPRDCVSCRNVSRGRECVDKCNLLEGEPREFV   550
EGFR-207     VSLNITSLGLRSLKEISDGDVIISGNKNLCYANTINWKKLFGTSGQKTKIISNRGENSCKATGQVCHALCSPEGCWGPEPRDCVSCRNVSRGRECVDKCNLLEGEPREFV   505
EGFR-205     ............................................................................................................   128
EGFR-202     VSLNITSLGLRSLKEISDGDVIISGNKNLCYANTINWKKLFGTSGQKTKIISNRGENSCKATGQVCHALCSPEGCWGPEPRDCVSCRNVSRGRECVDKCNLLEGEPREFV   550
EGFR-204     ............................................................................................................   405
EGFR-201     VSLNITSLGLRSLKEISDGDVIISGNKNLCYANTINWKKLFGTSGQKTKIISNRGENSCKATGQVCHALCSPEGCWGPEPRDCVSCRNVSRGRECVDKCNLLEGEPREFV   550

logo         ENSECIQCHPECLPQAMNITCTGRGPDNCIQCAHYIDGPHCVKTCPAGVMGENNTLVWKYADAGHVCHLCHPNCTYG
AFATINIB     ............................................................................................................   0
PF01030      ............................................................................................................   231
PF00757      ............................................................................................................   154
PF14843.2    ENSECIQCHPECLPQAMNITCTGRGPDNCIQCAHYIDGPHCVKTCPAGVMGENNTLVWKYADAGHVCHLCHPNCTYGPG.............................   125
PF07714      ............................................................................................................   0
PF14843.1    ENSECIQCHPECLPQAMNITCTGRGPDNCIQCAHYIDGPHCVKTCPAGVMGENNTLVWKYADAGHVCHLCHPNCTYG...............................   123
PF14843      ENSECIQCHPECLPQAMNITCTGRGPDNCIQCAHYIDGPHCVKTCPAGVMGENNTLVWKYADAGHVCHLCHPNCTYGCTGPGLEGC......................   132
PF01030.2    ............................................................................................................   111
PF01030.1    ............................................................................................................   205
PF00757.1    ............................................................................................................   153
EGFR-206     ENSECIQCHPECLPQAMNITCTGRGPDNCIQCAHYIDGPHCVKTCPAGVMGENNTLVWKYADAGHVCHLCHPNCTYGCTGPGLEGCPTNGPKI................   598
EGFR-203     ENSECIQCHPECLPQAMNITCTGRGPDNCIQCAHYIDGPHCVKTCPAGVMGENNTLVWKYADAGHVCHLCHPNCTYGPGNESLKAMLFCLFKLSSCNQSNDGSVSHQSGS   660
EGFR-207     ENSECIQCHPECLPQAMNITCTGRGPDNCIQCAHYIDGPHCVKTCPAGVMGENNTLVWKYADAGHVCHLCHPNCTYGCTGPGLEGCPTNGPKI................   598
EGFR-205     ............................................................................................................   128
EGFR-202     ENSECIQCHPECLPQAMNITCTGRGPDNCIQCAHYIDGPHCVKTCPAGVMGENNTLVWKYADAGHVCHLCHPNCTYGS..............................   628
EGFR-204     ............................................................................................................   405
EGFR-201     ENSECIQCHPECLPQAMNITCTGRGPDNCIQCAHYIDGPHCVKTCPAGVMGENNTLVWKYADAGHVCHLCHPNCTYGCTGPGLEGCPTNGPKI................   643
```

```
logo
             PSIATGMVGALLLLLVVALGIGLFMRRRHIVRKRTLRRLLQERELVEPLTPSGEAPNQALLRILKETEFKKIKVLGSGAFGTVYKGLWIPEGEKVKIPVAIKELREATSP
AFATINIB     ..............................................................................................................     0
PF01030      ..............................................................................................................   231
PF00757      ..............................................................................................................   154
PF14843.2    ..............................................................................................................   125
PF07714      ......................................................................KIKVLGSGAFGTVYKGLWIPEGEKVKIPVAIKELREATSP    40
PF14843.1    ..............................................................................................................   123
PF14843      ..............................................................................................................   132
PF01030.2    ..............................................................................................................   111
PF01030.1    ..............................................................................................................   205
PF00757.1    ..............................................................................................................   153
EGFR-206     PSIATGMVGALLLLLVVALGIGLFMRRRHIVRKRTLRRLLQERELVEPLTPSGEAPNQALLRILKETEFKKIKVLGSGAFGTVYKGLWIPEGEKVKIPVAIKELREATSP   708
EGFR-203     PAAQESCLGWIPSLLPSEFQLG......................................................WGG..CSHLHAWPSASVIITASS...........   703
EGFR-207     PSIATGMVGALLLLLVVALGIGLFMRRRHIVRKRTLRRLLQERELVEPLTPSGEAPNQALLRILKETEFKKIKVLGSGAFGTVYKGLWIPEGEKVKIPVAIKELREATSP   708
EGFR-205     ..............................................................................................................   128
EGFR-202     ..............................................................................................................   628
EGFR-204     ..............................................................................................................   405
EGFR-201     PSIATGMVGALLLLLVVALGIGLFMRRRHIVRKRTLRRLLQERELVEPLTPSGEAPNQALLRILKETEFKKIKVLGSGAFGTVYKGLWIPEGEKVKIPVAIKELREATSP   753

logo
             KANKEILDEAYVMASVDNPHVCRLLGICLTSTVQLITQLMPFGCLLDYVREHKDNIGSQYLLNWCVQIAKGMNYLEDRRLVHRDLAARNVLVKTPQHVKITDFGLAKLLG
AFATINIB     .........................................F.....Y...H..NI...Y..................................P...............     7
PF01030      ..............................................................................................................   231
PF00757      ..............................................................................................................   154
PF14843.2    ..............................................................................................................   125
PF07714      KANKEILDEAYVMASVDNPHVCRLLGICLTSTVQLITQLMPFGCLLDYVREHKDNIGSQYLLNWCVQIAKGMNYLEDRRLVHRDLAARNVLVKTPQHVKITDFGLAKLLG   150
PF14843.1    ..............................................................................................................   123
PF14843      ..............................................................................................................   132
PF01030.2    ..............................................................................................................   111
PF01030.1    ..............................................................................................................   205
PF00757.1    ..............................................................................................................   153
EGFR-206     KANKEILDEAYVMASVDNPHVCRLLGICLTSTVQLITQLMPFGCLLDYVREHKDNIGSQYLLNWCVQIAKGMNYLEDRRLVHRDLAARNVLVKTPQHVKITDFGLAKLLG   818
EGFR-203     .....................CH.......................................................................................   705
EGFR-207     KANKEILDEAYVMASVDNPHVCRLLGICLTSTVQLITQLMPFGCLLDYVREHKDNIGSQYLLNWCVQIAKGMNYLEDRRLVHRDLAARNVLVKTPQHVKITDFGLAKLLG   818
EGFR-205     ..............................................................................................................   128
EGFR-202     ..............................................................................................................   628
EGFR-204     ..............................................................................................................   405
EGFR-201     KANKEILDEAYVMASVDNPHVCRLLGICLTSTVQLITQLMPFGCLLDYVREHKDNIGSQYLLNWCVQIAKGMNYLEDRRLVHRDLAARNVLVKTPQHVKITDFGLAKLLG   863
```

```
logo
             AEEKEYHAEGGKVPIKWMALESILHRIYTHQSDVWSYGVTVWELMTFGSKPYDGIPASEISSILEKGERLPQPPICTIDVYMIMVKCWMIDADSRPKFRELIIEFSKMAR
AFATINIB     ..............................................................................................................    7
PF01030      ..............................................................................................................  231
PF00757      ..............................................................................................................  154
PF14843.2    ..............................................................................................................  125
PF07714      AEEKEYHAEGGKVPIKWMALESILHRIYTHQSDVWSYGVTVWELMTFGSKPYDGIPASEISSILEKGERLPQPPICTIDVYMIMVKCWMIDADSRPKFRELI.........  252
PF14843.1    ..............................................................................................................  123
PF14843      ..............................................................................................................  132
PF01030.2    ..............................................................................................................  111
PF01030.1    ..............................................................................................................  205
PF00757.1    ..............................................................................................................  153
EGFR-206     AEEKEYHAEGGKVPIKWMALESILHRIYTHQSDVWSYGVTVWELMTFGSKPYDGIPASEISSILEKGERLPQPPICTIDVYMIMVKCWMIDADSRPKFRELIIEFSKMAR  928
EGFR-203     ..............................................................................................................  705
EGFR-207     AEEKEYHAEGGKVPIKWMALESILHRIYTHQSDVWSYGVTVWELMTFGSKPYDGIPASEISSILEKGERLPQPPICTIDVYMIMVKCWMIDADSRPKFRELIIEFSKMAR  928
EGFR-205     ..............................................................................................................  128
EGFR-202     ..............................................................................................................  628
EGFR-204     ..............................................................................................................  405
EGFR-201     AEEKEYHAEGGKVPIKWMALESILHRIYTHQSDVWSYGVTVWELMTFGSKPYDGIPASEISSILEKGERLPQPPICTIDVYMIMVKCWMIDADSRPKFRELIIEFSKMAR  973

logo
             DPQRYLVIQGDERMHLPSPTDSNFYRALMDEEDMDDVVDADEYLIPQQGFFSSPSTSRTPLLSSLSATSNNSTVACIDRNGLQSCPIKEDSFLQRYSSDPTGALTEDSID
AFATINIB     ............RMHL..............................................................................................   11
PF01030      ..............................................................................................................  231
PF00757      ..............................................................................................................  154
PF14843.2    ..............................................................................................................  125
PF07714      ..............................................................................................................  252
PF14843.1    ..............................................................................................................  123
PF14843      ..............................................................................................................  132
PF01030.2    ..............................................................................................................  111
PF01030.1    ..............................................................................................................  205
PF00757.1    ..............................................................................................................  153
EGFR-206     DPQRYLVIQGDERMHLPSPTDSNFYRALMDEEDMDDVVDADEYLIPQQGFFSSPSTSRTPLLSSLSATSNNSTVACIDRNGLQSCPIKEDSFLQRYSSDPTGALTEDSID 1038
EGFR-203     ..............................................................................................................  705
EGFR-207     DPQRYLVIQGDERMHLPSPTDSNFYRALMDEEDMDDVVDADEYLIPQQGFFSSPSTSRTPLLSSLSATSNNSTVACIDRNGLQSCPIKEDSFLQRYSSDPTGALTEDSID 1038
EGFR-205     ..............................................................................................................  128
EGFR-202     ..............................................................................................................  628
EGFR-204     ..............................................................................................................  405
EGFR-201     DPQRYLVIQGDERMHLPSPTDSNFYRALMDEEDMDDVVDADEYLIPQQGFFSSPSTSRTPLLSSLSATSNNSTVACIDRNGLQSCPIKEDSFLQRYSSDPTGALTEDSID 1083
```

```
logo
            DTFLPVPEYINQSVP........KRPAGSVQNPVYHNQPLNPAPSRDPHYQDPHSTAVGNPEYLNTVQPTCVNSTFDSPAHWAQKGSHQISLDNPDYQQDFFPKEAKPNG
AFATINIB    .........................................................................................................   11
PF01030     .........................................................................................................  231
PF00757     .........................................................................................................  154
PF14843.2   .........................................................................................................  125
PF07714     .........................................................................................................  252
PF14843.1   .........................................................................................................  123
PF14843     .........................................................................................................  132
PF01030.2   .........................................................................................................  111
PF01030.1   .........................................................................................................  205
PF00757.1   .........................................................................................................  153
EGFR-206    DTFLPVPEYINQSVP........KRPAGSVQNPVYHNQPLNPAPSRDPHYQDPHSTAVGNPEYLNTVQPTCVNSTFDSPAHWAQKGSHQISLDNPDYQQDFFPKEAKPNG 1140
EGFR-203    .........................................................................................................  705
EGFR-207    DTFLPVPGEWLVWKQSCSSTSSTHSAAASLQCPSQVLPPASPEGETVADLQTQ.................................................... 1091
EGFR-205    .........................................................................................................  128
EGFR-202    .........................................................................................................  628
EGFR-204    .........................................................................................................  405
EGFR-201    DTFLPVPEYINQSVP........KRPAGSVQNPVYHNQPLNPAPSRDPHYQDPHSTAVGNPEYLNTVQPTCVNSTFDSPAHWAQKGSHQISLDNPDYQQDFFPKEAKPNG 1185
```

```
logo
            IFKGSTAENAEYLRVAPQSSEFIGA
AFATINIB    .........................   11
PF01030     .........................  231
PF00757     .........................  154
PF14843.2   .........................  125
PF07714     .........................  252
PF14843.1   .........................  123
PF14843     .........................  132
PF01030.2   .........................  111
PF01030.1   .........................  205
PF00757.1   .........................  153
EGFR-206    IFKGSTAENAEYLRVAPQSSEFIGA 1165
EGFR-203    .........................  705
EGFR-207    ......................... 1091
EGFR-205    .........................  128
EGFR-202    .........................  628
EGFR-204    .........................  405
EGFR-201    IFKGSTAENAEYLRVAPQSSEFIGA 1210
```

X non conserved
X similar
X $\geq 0\%$ conserved
X $\geq 50\%$ conserved

```
logo           MRPSGTAGAALLALLAALCPASRALEEKKVCQGTSNKLTQLGTFEDHFLSLQRMFNNCEVVLGNLEITYVQRNYDLSFLKTIQEVAGYVLIALNTVERIPLENLQIIRGN
DACOMITINIB    ..............................................................................................................    0
PF01030        ........................................................NCEVVLGNLEITYVQRNYDLSFLKTIQEVAGYVLIALNTVERIPLENLQIIRGN   54
PF00757        ..............................................................................................................    0
PF14843.2      ..............................................................................................................    0
PF07714        ..............................................................................................................    0
PF14843.1      ..............................................................................................................    0
PF14843        ..............................................................................................................    0
PF01030.2      ........................................................NCEVVLGNLEITYVQRNYDLSFLKTIQEVAGYVLIALNTVERIPLENLQIIRGN   54
PF01030.1      ........................................................NCEVVLGNLEITYVQRNYDLSFLKTIQEVAGYVLIALNTVERIPLENLQIIRGN   54
PF00757.1      ..............................................................................................................    0
EGFR-206       MRPSGTAGAALLALLAALCPASRALEEKKVCQGTSNKLTQLGTFEDHFLSLQRMFNNCEVVLGNLEITYVQRNYDLSFLKTIQEVAGYVLIALNTVERIPLENLQIIRGN  110
EGFR-203       MRPSGTAGAALLALLAALCPASRALEEKKVCQGTSNKLTQLGTFEDHFLSLQRMFNNCEVVLGNLEITYVQRNYDLSFLKTIQEVAGYVLIALNTVERIPLENLQIIRGN  110
EGFR-207       MRPSGTAGAALLALLAALCPASRALEEKKVCQGTSNKLTQLGTFEDHFLSLQRMFNNCEVVLGNLEITYVQRNYDLSFLKTIQEVAGYVLIALNTVERIPLENLQIIRGN  110
EGFR-205       .....................................................MFNNCEVVLGNLEITYVQRNYDLSFLKTIQEVAGYVLIALNTVERIPLENLQIIRGN   57
EGFR-202       MRPSGTAGAALLALLAALCPASRALEEKKVCQGTSNKLTQLGTFEDHFLSLQRMFNNCEVVLGNLEITYVQRNYDLSFLKTIQEVAGYVLIALNTVERIPLENLQIIRGN  110
EGFR-204       MRPSGTAGAALLALLAALCPASRALEEKKVCQGTSNKLTQLGTFEDHFLSLQRMFNNCEVVLGNLEITYVQRNYDLSFLKTIQEVAGYVLIALNTVERIPLENLQIIRGN  110
EGFR-201       MRPSGTAGAALLALLAALCPASRALEEKKVCQGTSNKLTQLGTFEDHFLSLQRMFNNCEVVLGNLEITYVQRNYDLSFLKTIQEVAGYVLIALNTVERIPLENLQIIRGN  110

logo           MYYENSYALAVLSNYDANKTGLKELPMRNLQEILHGAVRFSNNPALCNVESIQWRDIVSSDFLSNMSMDFQNHLGSCQKCDPSCPNGSCWGAGEENCQKLTKIICAQQCS
DACOMITINIB    ..............................................................................................................    0
PF01030        MYYENSYALAVLSNYDANKTGLKELPMRNLQEILHGAVRFSNNPALCNVESIQWRDI.....................................................  111
PF00757        ..........................................................................GSCQKCDPSCPNGSCWGAGEENCQKLTKIICAQQCS   36
PF14843.2      ..............................................................................................................    0
PF07714        ..............................................................................................................    0
PF14843.1      ..............................................................................................................    0
PF14843        ..............................................................................................................    0
PF01030.2      MYYENSYALAVLSNYDANKTGLKELPMRNLQEILHGAVRFSNNPALCNVESIQWRDI.....................................................  111
PF01030.1      MYYENSYALAVLSNYDANKTGLKELPMRNLQ...............................................................................   85
PF00757.1      ..............................QG.............................................QKCDPSCPNGSCWGAGEENCQKLTKIICAQQCS   35
EGFR-206       MYYENSYALAVLSNYDANKTGLKELPMRNLQG.............................................QKCDPSCPNGSCWGAGEENCQKLTKIICAQQCS  175
EGFR-203       MYYENSYALAVLSNYDANKTGLKELPMRNLQEILHGAVRFSNNPALCNVESIQWRDIVSSDFLSNMSMDFQNHLGSCQKCDPSCPNGSCWGAGEENCQKLTKIICAQQCS  220
EGFR-207       MYYENSYALAVLSNYDANKTGLKELPMRNLQG.............................................QKCDPSCPNGSCWGAGEENCQKLTKIICAQQCS  175
EGFR-205       MYYENSYALAVLSNYDANKTGLKELPMRNLQEILHGAVRFSNNPALCNVESIQWRDIVSSDFLSNMSMDFQ.......................................  128
EGFR-202       MYYENSYALAVLSNYDANKTGLKELPMRNLQEILHGAVRFSNNPALCNVESIQWRDIVSSDFLSNMSMDFQNHLGSCQKCDPSCPNGSCWGAGEENCQKLTKIICAQQCS  220
EGFR-204       MYYENSYALAVLSNYDANKTGLKELPMRNLQEILHGAVRFSNNPALCNVESIQWRDIVSSDFLSNMSMDFQNHLGSCQKCDPSCPNGSCWGAGEENCQKLTKIICAQQCS  220
EGFR-201       MYYENSYALAVLSNYDANKTGLKELPMRNLQEILHGAVRFSNNPALCNVESIQWRDIVSSDFLSNMSMDFQNHLGSCQKCDPSCPNGSCWGAGEENCQKLTKIICAQQCS  220
```

```
logo
             GRCRGKSPSDCCHNQCAAGCTGPRESDCLVCRKFRDEATCKDTCPPLMLYNPTTYQMDVNPEGKYSFGATCVKKCPRNYVVTDHGSCVRACGADSYEMEEDGVRKCKKCE
DACOMITINIB  ..............................................................................................................   0
PF01030      ..............................................................................................................   111
PF00757      GRCRGKSPSDCCHNQCAAGCTGPRESDCLVCRKFRDEATCKDTCPPLMLYNPTTYQMDVNPEGKYSFGATCVKKCPRNYVVTDHGSCVRACGADSYEMEEDGVRKCKKCE   146
PF14843.2    ..............................................................................................................   0
PF07714      ..............................................................................................................   0
PF14843.1    ..............................................................................................................   0
PF14843      ..............................................................................................................   0
PF01030.2    ..............................................................................................................   111
PF01030.1    ..............................................................................................................   85
PF00757.1    GRCRGKSPSDCCHNQCAAGCTGPRESDCLVCRKFRDEATCKDTCPPLMLYNPTTYQMDVNPEGKYSFGATCVKKCPRNYVVTDHGSCVRACGADSYEMEEDGVRKCKKCE   145
EGFR-206     GRCRGKSPSDCCHNQCAAGCTGPRESDCLVCRKFRDEATCKDTCPPLMLYNPTTYQMDVNPEGKYSFGATCVKKCPRNYVVTDHGSCVRACGADSYEMEEDGVRKCKKCE   285
EGFR-203     GRCRGKSPSDCCHNQCAAGCTGPRESDCLVCRKFRDEATCKDTCPPLMLYNPTTYQMDVNPEGKYSFGATCVKKCPRNYVVTDHGSCVRACGADSYEMEEDGVRKCKKCE   330
EGFR-207     GRCRGKSPSDCCHNQCAAGCTGPRESDCLVCRKFRDEATCKDTCPPLMLYNPTTYQMDVNPEGKYSFGATCVKKCPRNYVVTDHGSCVRACGADSYEMEEDGVRKCKKCE   285
EGFR-205     ..............................................................................................................   128
EGFR-202     GRCRGKSPSDCCHNQCAAGCTGPRESDCLVCRKFRDEATCKDTCPPLMLYNPTTYQMDVNPEGKYSFGATCVKKCPRNYVVTDHGSCVRACGADSYEMEEDGVRKCKKCE   330
EGFR-204     GRCRGKSPSDCCHNQCAAGCTGPRESDCLVCRKFRDEATCKDTCPPLMLYNPTTYQMDVNPEGKYSFGATCVKKCPRNYVVTDHGSCVRACGADSYEMEEDGVRKCKKCE   330
EGFR-201     GRCRGKSPSDCCHNQCAAGCTGPRESDCLVCRKFRDEATCKDTCPPLMLYNPTTYQMDVNPEGKYSFGATCVKKCPRNYVVTDHGSCVRACGADSYEMEEDGVRKCKKCE   330

logo
             GPCRKVCNGIGIGEFKDSLSINATNIKHFKNCTSISGDLHILPVAFRGDSFTHTPPLDPQELDILKTVKEITGFLLIQAWPENRTDLHAFENLEIIRGRTKQHGQFSLAV
DACOMITINIB  ..............................................................................................................   0
PF01030      ..............................NCTSISGDLHILPVAFRGDSFTHTPPLDPQELDILKTVKEITGFLLIQAWPENRTDLHAFENLEIIRGRTKQHGQFSLAV   191
PF00757      GPCRKVCN......................................................................................................   154
PF14843.2    ..............................................................................................................   0
PF07714      ..............................................................................................................   0
PF14843.1    ..............................................................................................................   0
PF14843      ..............................................................................................................   0
PF01030.2    ..............................................................................................................   111
PF01030.1    ..............................NCTSISGDLHILPVAFRGDSFTHTPPLDPQELDILKTVKEITGFLLIQAWPENRTDLHAFENLEIIRGRTKQHGQFSLAV   165
PF00757.1    GPCRKVCN......................................................................................................   153
EGFR-206     GPCRKVCNGIGIGEFKDSLSINATNIKHFKNCTSISGDLHILPVAFRGDSFTHTPPLDPQELDILKTVKEITGFLLIQAWPENRTDLHAFENLEIIRGRTKQHGQFSLAV   395
EGFR-203     GPCRKVCNGIGIGEFKDSLSINATNIKHFKNCTSISGDLHILPVAFRGDSFTHTPPLDPQELDILKTVKEITGFLLIQAWPENRTDLHAFENLEIIRGRTKQHGQFSLAV   440
EGFR-207     GPCRKVCNGIGIGEFKDSLSINATNIKHFKNCTSISGDLHILPVAFRGDSFTHTPPLDPQELDILKTVKEITGFLLIQAWPENRTDLHAFENLEIIRGRTKQHGQFSLAV   395
EGFR-205     ..............................................................................................................   128
EGFR-202     GPCRKVCNGIGIGEFKDSLSINATNIKHFKNCTSISGDLHILPVAFRGDSFTHTPPLDPQELDILKTVKEITGFLLIQAWPENRTDLHAFENLEIIRGRTKQHGQFSLAV   440
EGFR-204     GPCRKVCNGIGIGEFKDSLSINATNIKHFKNCTSISGDLHILPVAFRGDSFTHTPPLDPQELDILKTVKEITGLS...................................   405
EGFR-201     GPCRKVCNGIGIGEFKDSLSINATNIKHFKNCTSISGDLHILPVAFRGDSFTHTPPLDPQELDILKTVKEITGFLLIQAWPENRTDLHAFENLEIIRGRTKQHGQFSLAV   440
```

```
logo         VSLNITSLGLRSLKEISDGDVIISGNKNLCYANTINWKKLFGTSGQKTKIISNRGENSCKATGQVCHALCSPEGCWGPEPRDCVSCRNVSRGRECVDKCNLLEGEPREFV
DACOMITINIB  ..............................................................................................................    0
PF01030      VSLNITSLGLRSLKEISDGDVIISGNKNLCYANTINWKKL......................................................................  231
PF00757      ..............................................................................................................  154
PF14843.2    ................................................................VCHALCSPEGCWGPEPRDCVSCRNVSRGRECVDKCNLLEGEPREFV   46
PF07714      ..............................................................................................................    0
PF14843.1    ................................................................VCHALCSPEGCWGPEPRDCVSCRNVSRGRECVDKCNLLEGEPREFV   46
PF14843      ................................................................VCHALCSPEGCWGPEPRDCVSCRNVSRGRECVDKCNLLEGEPREFV   46
PF01030.2    ..............................................................................................................  111
PF01030.1    VSLNITSLGLRSLKEISDGDVIISGNKNLCYANTINWKKL......................................................................  205
PF00757.1    ..............................................................................................................  153
EGFR-206     VSLNITSLGLRSLKEISDGDVIISGNKNLCYANTINWKKLFGTSGQKTKIISNRGENSCKATGQVCHALCSPEGCWGPEPRDCVSCRNVSRGRECVDKCNLLEGEPREFV  505
EGFR-203     VSLNITSLGLRSLKEISDGDVIISGNKNLCYANTINWKKLFGTSGQKTKIISNRGENSCKATGQVCHALCSPEGCWGPEPRDCVSCRNVSRGRECVDKCNLLEGEPREFV  550
EGFR-207     VSLNITSLGLRSLKEISDGDVIISGNKNLCYANTINWKKLFGTSGQKTKIISNRGENSCKATGQVCHALCSPEGCWGPEPRDCVSCRNVSRGRECVDKCNLLEGEPREFV  505
EGFR-205     ..............................................................................................................  128
EGFR-202     VSLNITSLGLRSLKEISDGDVIISGNKNLCYANTINWKKLFGTSGQKTKIISNRGENSCKATGQVCHALCSPEGCWGPEPRDCVSCRNVSRGRECVDKCNLLEGEPREFV  550
EGFR-204     ..............................................................................................................  405
EGFR-201     VSLNITSLGLRSLKEISDGDVIISGNKNLCYANTINWKKLFGTSGQKTKIISNRGENSCKATGQVCHALCSPEGCWGPEPRDCVSCRNVSRGRECVDKCNLLEGEPREFV  550

logo         ENSECIQCHPECLPQAMNITCTGRGPDNCIQCAHYIDGPHCVKTCPAGVMGENNTLVWKYADAGHVCHLCHPNCTYGCTGPGLEGCPTNGPKI
DACOMITINIB  ..............................................................................................................    0
PF01030      ..............................................................................................................  231
PF00757      ..............................................................................................................  154
PF14843.2    ENSECIQCHPECLPQAMNITCTGRGPDNCIQCAHYIDGPHCVKTCPAGVMGENNTLVWKYADAGHVCHLCHPNCTYGPG..............................  125
PF07714      ..............................................................................................................    0
PF14843.1    ENSECIQCHPECLPQAMNITCTGRGPDNCIQCAHYIDGPHCVKTCPAGVMGENNTLVWKYADAGHVCHLCHPNCTYG.................................  123
PF14843      ENSECIQCHPECLPQAMNITCTGRGPDNCIQCAHYIDGPHCVKTCPAGVMGENNTLVWKYADAGHVCHLCHPNCTYGCTGPGLEGC........................  132
PF01030.2    ..............................................................................................................  111
PF01030.1    ..............................................................................................................  205
PF00757.1    ..............................................................................................................  153
EGFR-206     ENSECIQCHPECLPQAMNITCTGRGPDNCIQCAHYIDGPHCVKTCPAGVMGENNTLVWKYADAGHVCHLCHPNCTYGCTGPGLEGCPTNGPKI.................  598
EGFR-203     ENSECIQCHPECLPQAMNITCTGRGPDNCIQCAHYIDGPHCVKTCPAGVMGENNTLVWKYADAGHVCHLCHPNCTYGPGNESLKAMLFCLFKLSSCNQSNDGSVSHQSGS  660
EGFR-207     ENSECIQCHPECLPQAMNITCTGRGPDNCIQCAHYIDGPHCVKTCPAGVMGENNTLVWKYADAGHVCHLCHPNCTYGCTGPGLEGCPTNGPKI.................  598
EGFR-205     ..............................................................................................................  128
EGFR-202     ENSECIQCHPECLPQAMNITCTGRGPDNCIQCAHYIDGPHCVKTCPAGVMGENNTLVWKYADAGHVCHLCHPNCTYGS................................  628
EGFR-204     ..............................................................................................................  405
EGFR-201     ENSECIQCHPECLPQAMNITCTGRGPDNCIQCAHYIDGPHCVKTCPAGVMGENNTLVWKYADAGHVCHLCHPNCTYGCTGPGLEGCPTNGPKI.................  643
```

```
logo
              PSIATGMVGALLLLLVVALGIGLFMRRRHIVRKRTLRRLLQERELVEPLTPSGEAPNQALLRILKETEFKKIKVLGSGAFGTVYKGLWIPEGEKVKIPVAIKELREATSP
DACOMITINIB   ..........................................................................L........................AIK........     4
PF01030       ..............................................................................................................   231
PF00757       ..............................................................................................................   154
PF14843.2     ..............................................................................................................   125
PF07714       ......................................................................KIKVLGSGAFGTVYKGLWIPEGEKVKIPVAIKELREATSP    40
PF14843.1     ..............................................................................................................   123
PF14843       ..............................................................................................................   132
PF01030.2     ..............................................................................................................   111
PF01030.1     ..............................................................................................................   205
PF00757.1     ..............................................................................................................   153
EGFR-206      PSIATGMVGALLLLLVVALGIGLFMRRRHIVRKRTLRRLLQERELVEPLTPSGEAPNQALLRILKETEFKKIKVLGSGAFGTVYKGLWIPEGEKVKIPVAIKELREATSP   708
EGFR-203      PAAQESCLGWIPSLLPSEFQLG......................................................WGG..CSHLHAWPSASVIITASS...........   703
EGFR-207      PSIATGMVGALLLLLVVALGIGLFMRRRHIVRKRTLRRLLQERELVEPLTPSGEAPNQALLRILKETEFKKIKVLGSGAFGTVYKGLWIPEGEKVKIPVAIKELREATSP   708
EGFR-205      ..............................................................................................................   128
EGFR-202      ..............................................................................................................   628
EGFR-204      ..............................................................................................................   405
EGFR-201      PSIATGMVGALLLLLVVALGIGLFMRRRHIVRKRTLRRLLQERELVEPLTPSGEAPNQALLRILKETEFKKIKVLGSGAFGTVYKGLWIPEGEKVKIPVAIKELREATSP   753

logo
              KANKEILDEAYVMASVDNPHVCRLLGICLTSTVQLITQLMPFGCLLDYVREHKDNIGSQYLLNWCVQIAKGMNYLEDRRLVHRDLAARNVLVKTPQHVKITDFGLAKLLG
DACOMITINIB   ............M.....................L.MQLMP.GC..D...........................................L.........TD........    17
PF01030       ..............................................................................................................   231
PF00757       ..............................................................................................................   154
PF14843.2     ..............................................................................................................   125
PF07714       KANKEILDEAYVMASVDNPHVCRLLGICLTSTVQLITQLMPFGCLLDYVREHKDNIGSQYLLNWCVQIAKGMNYLEDRRLVHRDLAARNVLVKTPQHVKITDFGLAKLLG   150
PF14843.1     ..............................................................................................................   123
PF14843       ..............................................................................................................   132
PF01030.2     ..............................................................................................................   111
PF01030.1     ..............................................................................................................   205
PF00757.1     ..............................................................................................................   153
EGFR-206      KANKEILDEAYVMASVDNPHVCRLLGICLTSTVQLITQLMPFGCLLDYVREHKDNIGSQYLLNWCVQIAKGMNYLEDRRLVHRDLAARNVLVKTPQHVKITDFGLAKLLG   818
EGFR-203      .....................CH.......................................................................................   705
EGFR-207      KANKEILDEAYVMASVDNPHVCRLLGICLTSTVQLITQLMPFGCLLDYVREHKDNIGSQYLLNWCVQIAKGMNYLEDRRLVHRDLAARNVLVKTPQHVKITDFGLAKLLG   818
EGFR-205      ..............................................................................................................   128
EGFR-202      ..............................................................................................................   628
EGFR-204      ..............................................................................................................   405
EGFR-201      KANKEILDEAYVMASVDNPHVCRLLGICLTSTVQLITQLMPFGCLLDYVREHKDNIGSQYLLNWCVQIAKGMNYLEDRRLVHRDLAARNVLVKTPQHVKITDFGLAKLLG   863
```

```
logo
               AEEKEYHAEGGKVPIKWMALESILHRIYTHQSDVWSYGVTVWELMTFGSKPYDGIPASEISSILEKGERLPQPPICTIDVYMIMVKCWMIDADSRPKFRELIIEFSKMAR
DACOMITINIB    ..............................................................................................................   17
PF01030        ..............................................................................................................  231
PF00757        ..............................................................................................................  154
PF14843.2      ..............................................................................................................  125
PF07714        AEEKEYHAEGGKVPIKWMALESILHRIYTHQSDVWSYGVTVWELMTFGSKPYDGIPASEISSILEKGERLPQPPICTIDVYMIMVKCWMIDADSRPKFRELI........  252
PF14843.1      ..............................................................................................................  123
PF14843        ..............................................................................................................  132
PF01030.2      ..............................................................................................................  111
PF01030.1      ..............................................................................................................  205
PF00757.1      ..............................................................................................................  153
EGFR-206       AEEKEYHAEGGKVPIKWMALESILHRIYTHQSDVWSYGVTVWELMTFGSKPYDGIPASEISSILEKGERLPQPPICTIDVYMIMVKCWMIDADSRPKFRELIIEFSKMAR  928
EGFR-203       ..............................................................................................................  705
EGFR-207       AEEKEYHAEGGKVPIKWMALESILHRIYTHQSDVWSYGVTVWELMTFGSKPYDGIPASEISSILEKGERLPQPPICTIDVYMIMVKCWMIDADSRPKFRELIIEFSKMAR  928
EGFR-205       ..............................................................................................................  128
EGFR-202       ..............................................................................................................  628
EGFR-204       ..............................................................................................................  405
EGFR-201       AEEKEYHAEGGKVPIKWMALESILHRIYTHQSDVWSYGVTVWELMTFGSKPYDGIPASEISSILEKGERLPQPPICTIDVYMIMVKCWMIDADSRPKFRELIIEFSKMAR  973

logo
               DPQRYLVIQGDERMHLPSPTDSNFYRALMDEEDMDDVVDADEYLIPQQGFFSSPSTSRTPLLSSLSATSNNSTVACIDRNGLQSCPIKEDSFLQRYSSDPTGALTEDSID
DACOMITINIB    ..............................................................................................................   17
PF01030        ..............................................................................................................  231
PF00757        ..............................................................................................................  154
PF14843.2      ..............................................................................................................  125
PF07714        ..............................................................................................................  252
PF14843.1      ..............................................................................................................  123
PF14843        ..............................................................................................................  132
PF01030.2      ..............................................................................................................  111
PF01030.1      ..............................................................................................................  205
PF00757.1      ..............................................................................................................  153
EGFR-206       DPQRYLVIQGDERMHLPSPTDSNFYRALMDEEDMDDVVDADEYLIPQQGFFSSPSTSRTPLLSSLSATSNNSTVACIDRNGLQSCPIKEDSFLQRYSSDPTGALTEDSID 1038
EGFR-203       ..............................................................................................................  705
EGFR-207       DPQRYLVIQGDERMHLPSPTDSNFYRALMDEEDMDDVVDADEYLIPQQGFFSSPSTSRTPLLSSLSATSNNSTVACIDRNGLQSCPIKEDSFLQRYSSDPTGALTEDSID 1038
EGFR-205       ..............................................................................................................  128
EGFR-202       ..............................................................................................................  628
EGFR-204       ..............................................................................................................  405
EGFR-201       DPQRYLVIQGDERMHLPSPTDSNFYRALMDEEDMDDVVDADEYLIPQQGFFSSPSTSRTPLLSSLSATSNNSTVACIDRNGLQSCPIKEDSFLQRYSSDPTGALTEDSID 1083
```

```
logo

             DTFLPVPEYINQSVP........KRPAGSVQNPVYHNQPLNPAPSRDPHYQDPHSTAVGNPEYLNTVQPTCVNSTFDSPAHWAQKGSHQISLDNPDYQQDFFPKEAKPNG
DACOMITINIB  ..............................................................................................................   17
PF01030      ..............................................................................................................  231
PF00757      ..............................................................................................................  154
PF14843.2    ..............................................................................................................  125
PF07714      ..............................................................................................................  252
PF14843.1    ..............................................................................................................  123
PF14843      ..............................................................................................................  132
PF01030.2    ..............................................................................................................  111
PF01030.1    ..............................................................................................................  205
PF00757.1    ..............................................................................................................  153
EGFR-206     DTFLPVPEYINQSVP........KRPAGSVQNPVYHNQPLNPAPSRDPHYQDPHSTAVGNPEYLNTVQPTCVNSTFDSPAHWAQKGSHQISLDNPDYQQDFFPKEAKPNG 1140
EGFR-203     ..............................................................................................................  705
EGFR-207     DTFLPVPGEWLVWKQSCSSTSSTHSAAASLQCPSQVLPPASPEGETVADLQTQ......................................................... 1091
EGFR-205     ..............................................................................................................  128
EGFR-202     ..............................................................................................................  628
EGFR-204     ..............................................................................................................  405
EGFR-201     DTFLPVPEYINQSVP........KRPAGSVQNPVYHNQPLNPAPSRDPHYQDPHSTAVGNPEYLNTVQPTCVNSTFDSPAHWAQKGSHQISLDNPDYQQDFFPKEAKPNG 1185


logo

             IFKGSTAENAEYLRVAPQSSEFIGA
DACOMITINIB  .........................   17
PF01030      .........................  231
PF00757      .........................  154
PF14843.2    .........................  125
PF07714      .........................  252
PF14843.1    .........................  123
PF14843      .........................  132
PF01030.2    .........................  111
PF01030.1    .........................  205
PF00757.1    .........................  153
EGFR-206     IFKGSTAENAEYLRVAPQSSEFIGA 1165
EGFR-203     .........................  705
EGFR-207     ......................... 1091
EGFR-205     .........................  128
EGFR-202     .........................  628
EGFR-204     .........................  405
EGFR-201     IFKGSTAENAEYLRVAPQSSEFIGA 1210
```

- X non conserved
- X similar
- X $\geq 0\%$ conserved
- X $\geq 50\%$ conserved

```
logo
```
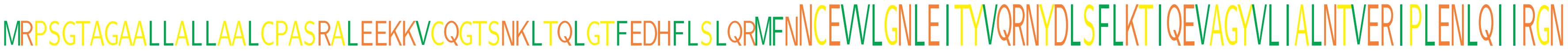

```
ERLOTINIB    ..............................................................................................................   0
PF01030      ........................................................NCEVVLGNLEITYVQRNYDLSFLKTIQEVAGYVLIALNTVERIPLENLQIIRGN  54
PF00757      ..............................................................................................................   0
PF14843.2    ..............................................................................................................   0
PF07714      ..............................................................................................................   0
PF14843.1    ..............................................................................................................   0
PF14843      ..............................................................................................................   0
PF01030.2    ........................................................NCEVVLGNLEITYVQRNYDLSFLKTIQEVAGYVLIALNTVERIPLENLQIIRGN  54
PF01030.1    ........................................................NCEVVLGNLEITYVQRNYDLSFLKTIQEVAGYVLIALNTVERIPLENLQIIRGN  54
PF00757.1    ..............................................................................................................   0
EGFR-206     MRPSGTAGAALLALLAALCPASRALEEKKVCQGTSNKLTQLGTFEDHFLSLQRMFNNCEVVLGNLEITYVQRNYDLSFLKTIQEVAGYVLIALNTVERIPLENLQIIRGN 110
EGFR-203     MRPSGTAGAALLALLAALCPASRALEEKKVCQGTSNKLTQLGTFEDHFLSLQRMFNNCEVVLGNLEITYVQRNYDLSFLKTIQEVAGYVLIALNTVERIPLENLQIIRGN 110
EGFR-207     MRPSGTAGAALLALLAALCPASRALEEKKVCQGTSNKLTQLGTFEDHFLSLQRMFNNCEVVLGNLEITYVQRNYDLSFLKTIQEVAGYVLIALNTVERIPLENLQIIRGN 110
EGFR-205     .....................................................MFNNCEVVLGNLEITYVQRNYDLSFLKTIQEVAGYVLIALNTVERIPLENLQIIRGN  57
EGFR-202     MRPSGTAGAALLALLAALCPASRALEEKKVCQGTSNKLTQLGTFEDHFLSLQRMFNNCEVVLGNLEITYVQRNYDLSFLKTIQEVAGYVLIALNTVERIPLENLQIIRGN 110
EGFR-204     MRPSGTAGAALLALLAALCPASRALEEKKVCQGTSNKLTQLGTFEDHFLSLQRMFNNCEVVLGNLEITYVQRNYDLSFLKTIQEVAGYVLIALNTVERIPLENLQIIRGN 110
EGFR-201     MRPSGTAGAALLALLAALCPASRALEEKKVCQGTSNKLTQLGTFEDHFLSLQRMFNNCEVVLGNLEITYVQRNYDLSFLKTIQEVAGYVLIALNTVERIPLENLQIIRGN 110
```

```
logo
```
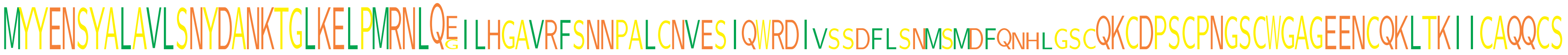

```
ERLOTINIB    ..............................................................................................................   0
PF01030      MYYENSYALAVLSNYDANKTGLKELPMRNLQEILHGAVRFSNNPALCNVESIQWRDI..................................................... 111
PF00757      ..........................................................................GSCQKCDPSCPNGSCWGAGEENCQKLTKIICAQQCS  36
PF14843.2    ..............................................................................................................   0
PF07714      ..............................................................................................................   0
PF14843.1    ..............................................................................................................   0
PF14843      ..............................................................................................................   0
PF01030.2    MYYENSYALAVLSNYDANKTGLKELPMRNLQEILHGAVRFSNNPALCNVESIQWRDI..................................................... 111
PF01030.1    MYYENSYALAVLSNYDANKTGLKELPMRNLQ...............................................................................  85
PF00757.1    ..............................QG.............................................QKCDPSCPNGSCWGAGEENCQKLTKIICAQQCS  35
EGFR-206     MYYENSYALAVLSNYDANKTGLKELPMRNLQG.............................................QKCDPSCPNGSCWGAGEENCQKLTKIICAQQCS 175
EGFR-203     MYYENSYALAVLSNYDANKTGLKELPMRNLQEILHGAVRFSNNPALCNVESIQWRDIVSSDFLSNMSMDFQNHLGSCQKCDPSCPNGSCWGAGEENCQKLTKIICAQQCS 220
EGFR-207     MYYENSYALAVLSNYDANKTGLKELPMRNLQG.............................................QKCDPSCPNGSCWGAGEENCQKLTKIICAQQCS 175
EGFR-205     MYYENSYALAVLSNYDANKTGLKELPMRNLQEILHGAVRFSNNPALCNVESIQWRDIVSSDFLSNMSMDFQ....................................... 128
EGFR-202     MYYENSYALAVLSNYDANKTGLKELPMRNLQEILHGAVRFSNNPALCNVESIQWRDIVSSDFLSNMSMDFQNHLGSCQKCDPSCPNGSCWGAGEENCQKLTKIICAQQCS 220
EGFR-204     MYYENSYALAVLSNYDANKTGLKELPMRNLQEILHGAVRFSNNPALCNVESIQWRDIVSSDFLSNMSMDFQNHLGSCQKCDPSCPNGSCWGAGEENCQKLTKIICAQQCS 220
EGFR-201     MYYENSYALAVLSNYDANKTGLKELPMRNLQEILHGAVRFSNNPALCNVESIQWRDIVSSDFLSNMSMDFQNHLGSCQKCDPSCPNGSCWGAGEENCQKLTKIICAQQCS 220
```

```
logo
            GRCRGKSPSDCCHNQCAAGCTGPRESDCLVCRKFRDEATCKDTCPPLMLYNPTTYQMDVNPEGKYSFGATCVKKCPRNYVVTDHGSCVRACGADSYEMEEDGVRKCKKCE
ERLOTINIB   ..............................................................................................................     0
PF01030     ..............................................................................................................   111
PF00757     GRCRGKSPSDCCHNQCAAGCTGPRESDCLVCRKFRDEATCKDTCPPLMLYNPTTYQMDVNPEGKYSFGATCVKKCPRNYVVTDHGSCVRACGADSYEMEEDGVRKCKKCE   146
PF14843.2   ..............................................................................................................     0
PF07714     ..............................................................................................................     0
PF14843.1   ..............................................................................................................     0
PF14843     ..............................................................................................................     0
PF01030.2   ..............................................................................................................   111
PF01030.1   ..............................................................................................................    85
PF00757.1   GRCRGKSPSDCCHNQCAAGCTGPRESDCLVCRKFRDEATCKDTCPPLMLYNPTTYQMDVNPEGKYSFGATCVKKCPRNYVVTDHGSCVRACGADSYEMEEDGVRKCKKCE   145
EGFR-206    GRCRGKSPSDCCHNQCAAGCTGPRESDCLVCRKFRDEATCKDTCPPLMLYNPTTYQMDVNPEGKYSFGATCVKKCPRNYVVTDHGSCVRACGADSYEMEEDGVRKCKKCE   285
EGFR-203    GRCRGKSPSDCCHNQCAAGCTGPRESDCLVCRKFRDEATCKDTCPPLMLYNPTTYQMDVNPEGKYSFGATCVKKCPRNYVVTDHGSCVRACGADSYEMEEDGVRKCKKCE   330
EGFR-207    GRCRGKSPSDCCHNQCAAGCTGPRESDCLVCRKFRDEATCKDTCPPLMLYNPTTYQMDVNPEGKYSFGATCVKKCPRNYVVTDHGSCVRACGADSYEMEEDGVRKCKKCE   285
EGFR-205    ..............................................................................................................   128
EGFR-202    GRCRGKSPSDCCHNQCAAGCTGPRESDCLVCRKFRDEATCKDTCPPLMLYNPTTYQMDVNPEGKYSFGATCVKKCPRNYVVTDHGSCVRACGADSYEMEEDGVRKCKKCE   330
EGFR-204    GRCRGKSPSDCCHNQCAAGCTGPRESDCLVCRKFRDEATCKDTCPPLMLYNPTTYQMDVNPEGKYSFGATCVKKCPRNYVVTDHGSCVRACGADSYEMEEDGVRKCKKCE   330
EGFR-201    GRCRGKSPSDCCHNQCAAGCTGPRESDCLVCRKFRDEATCKDTCPPLMLYNPTTYQMDVNPEGKYSFGATCVKKCPRNYVVTDHGSCVRACGADSYEMEEDGVRKCKKCE   330

logo
            GPCRKVCNGIGIGEFKDSLSINATNIKHFKNCTSISGDLHILPVAFRGDSFTHTPPLDPQELDILKTVKEITGFLLIQAWPENRTDLHAFENLEIIRGRTKQHGQFSLAV
ERLOTINIB   ..............................................................................................................     0
PF01030     ..............................NCTSISGDLHILPVAFRGDSFTHTPPLDPQELDILKTVKEITGFLLIQAWPENRTDLHAFENLEIIRGRTKQHGQFSLAV   191
PF00757     GPCRKVCN......................................................................................................   154
PF14843.2   ..............................................................................................................     0
PF07714     ..............................................................................................................     0
PF14843.1   ..............................................................................................................     0
PF14843     ..............................................................................................................     0
PF01030.2   ..............................................................................................................   111
PF01030.1   ..............................NCTSISGDLHILPVAFRGDSFTHTPPLDPQELDILKTVKEITGFLLIQAWPENRTDLHAFENLEIIRGRTKQHGQFSLAV   165
PF00757.1   GPCRKVCN......................................................................................................   153
EGFR-206    GPCRKVCNGIGIGEFKDSLSINATNIKHFKNCTSISGDLHILPVAFRGDSFTHTPPLDPQELDILKTVKEITGFLLIQAWPENRTDLHAFENLEIIRGRTKQHGQFSLAV   395
EGFR-203    GPCRKVCNGIGIGEFKDSLSINATNIKHFKNCTSISGDLHILPVAFRGDSFTHTPPLDPQELDILKTVKEITGFLLIQAWPENRTDLHAFENLEIIRGRTKQHGQFSLAV   440
EGFR-207    GPCRKVCNGIGIGEFKDSLSINATNIKHFKNCTSISGDLHILPVAFRGDSFTHTPPLDPQELDILKTVKEITGFLLIQAWPENRTDLHAFENLEIIRGRTKQHGQFSLAV   395
EGFR-205    ..............................................................................................................   128
EGFR-202    GPCRKVCNGIGIGEFKDSLSINATNIKHFKNCTSISGDLHILPVAFRGDSFTHTPPLDPQELDILKTVKEITGFLLIQAWPENRTDLHAFENLEIIRGRTKQHGQFSLAV   440
EGFR-204    GPCRKVCNGIGIGEFKDSLSINATNIKHFKNCTSISGDLHILPVAFRGDSFTHTPPLDPQELDILKTVKEITGLS...................................   405
EGFR-201    GPCRKVCNGIGIGEFKDSLSINATNIKHFKNCTSISGDLHILPVAFRGDSFTHTPPLDPQELDILKTVKEITGFLLIQAWPENRTDLHAFENLEIIRGRTKQHGQFSLAV   440
```

```
logo         VSLNITSLGLRSLKEISDGDVIISGNKNLCYANTINWKKLFGTSGQKTKIISNRGENSCKATGQVCHALCSPEGCWGPEPRDCVSCRNVSRGRECVDKCNLLEGEPREFV
ERLOTINIB    ..............................................................................................................    0
PF01030      VSLNITSLGLRSLKEISDGDVIISGNKNLCYANTINWKKL......................................................................  231
PF00757      ..............................................................................................................  154
PF14843.2    ................................................................VCHALCSPEGCWGPEPRDCVSCRNVSRGRECVDKCNLLEGEPREFV   46
PF07714      ..............................................................................................................    0
PF14843.1    ................................................................VCHALCSPEGCWGPEPRDCVSCRNVSRGRECVDKCNLLEGEPREFV   46
PF14843      ................................................................VCHALCSPEGCWGPEPRDCVSCRNVSRGRECVDKCNLLEGEPREFV   46
PF01030.2    ..............................................................................................................  111
PF01030.1    VSLNITSLGLRSLKEISDGDVIISGNKNLCYANTINWKKL......................................................................  205
PF00757.1    ..............................................................................................................  153
EGFR-206     VSLNITSLGLRSLKEISDGDVIISGNKNLCYANTINWKKLFGTSGQKTKIISNRGENSCKATGQVCHALCSPEGCWGPEPRDCVSCRNVSRGRECVDKCNLLEGEPREFV  505
EGFR-203     VSLNITSLGLRSLKEISDGDVIISGNKNLCYANTINWKKLFGTSGQKTKIISNRGENSCKATGQVCHALCSPEGCWGPEPRDCVSCRNVSRGRECVDKCNLLEGEPREFV  550
EGFR-207     VSLNITSLGLRSLKEISDGDVIISGNKNLCYANTINWKKLFGTSGQKTKIISNRGENSCKATGQVCHALCSPEGCWGPEPRDCVSCRNVSRGRECVDKCNLLEGEPREFV  505
EGFR-205     ..............................................................................................................  128
EGFR-202     VSLNITSLGLRSLKEISDGDVIISGNKNLCYANTINWKKLFGTSGQKTKIISNRGENSCKATGQVCHALCSPEGCWGPEPRDCVSCRNVSRGRECVDKCNLLEGEPREFV  550
EGFR-204     ..............................................................................................................  405
EGFR-201     VSLNITSLGLRSLKEISDGDVIISGNKNLCYANTINWKKLFGTSGQKTKIISNRGENSCKATGQVCHALCSPEGCWGPEPRDCVSCRNVSRGRECVDKCNLLEGEPREFV  550

logo         ENSECIQCHPECLPQAMNITCTGRGPDNCIQCAHYIDGPHCVKTCPAGVMGENNTLVWKYADAGHVCHLCHPNCTYGCTGPGLEGCPTNGPKI
ERLOTINIB    ..............................................................................................................    0
PF01030      ..............................................................................................................  231
PF00757      ..............................................................................................................  154
PF14843.2    ENSECIQCHPECLPQAMNITCTGRGPDNCIQCAHYIDGPHCVKTCPAGVMGENNTLVWKYADAGHVCHLCHPNCTYGPG...............................  125
PF07714      ..............................................................................................................    0
PF14843.1    ENSECIQCHPECLPQAMNITCTGRGPDNCIQCAHYIDGPHCVKTCPAGVMGENNTLVWKYADAGHVCHLCHPNCTYG.................................  123
PF14843      ENSECIQCHPECLPQAMNITCTGRGPDNCIQCAHYIDGPHCVKTCPAGVMGENNTLVWKYADAGHVCHLCHPNCTYGCTGPGLEGC........................  132
PF01030.2    ..............................................................................................................  111
PF01030.1    ..............................................................................................................  205
PF00757.1    ..............................................................................................................  153
EGFR-206     ENSECIQCHPECLPQAMNITCTGRGPDNCIQCAHYIDGPHCVKTCPAGVMGENNTLVWKYADAGHVCHLCHPNCTYGCTGPGLEGCPTNGPKI.................  598
EGFR-203     ENSECIQCHPECLPQAMNITCTGRGPDNCIQCAHYIDGPHCVKTCPAGVMGENNTLVWKYADAGHVCHLCHPNCTYGPGNESLKAMLFCLFKLSSCNQSNDGSVSHQSGS  660
EGFR-207     ENSECIQCHPECLPQAMNITCTGRGPDNCIQCAHYIDGPHCVKTCPAGVMGENNTLVWKYADAGHVCHLCHPNCTYGCTGPGLEGCPTNGPKI.................  598
EGFR-205     ..............................................................................................................  128
EGFR-202     ENSECIQCHPECLPQAMNITCTGRGPDNCIQCAHYIDGPHCVKTCPAGVMGENNTLVWKYADAGHVCHLCHPNCTYGS................................  628
EGFR-204     ..............................................................................................................  405
EGFR-201     ENSECIQCHPECLPQAMNITCTGRGPDNCIQCAHYIDGPHCVKTCPAGVMGENNTLVWKYADAGHVCHLCHPNCTYGCTGPGLEGCPTNGPKI.................  643
```

```
logo
             PSIATGMVGALLLLLVVALGIGLFMRRRHIVRKRTLRRLLQERELVEPLTPSGEAPNQALLRILKETEFKKIKVLGSGAFGTVYKGLWIPEGEKVKIPVAIKELREATSP
ERLOTINIB    ...............................................................L..................V................A.K........   4
PF01030      ..............................................................................................................   231
PF00757      ..............................................................................................................   154
PF14843.2    ..............................................................................................................   125
PF07714      ......................................................................KIKVLGSGAFGTVYKGLWIPEGEKVKIPVAIKELREATSP   40
PF14843.1    ..............................................................................................................   123
PF14843      ..............................................................................................................   132
PF01030.2    ..............................................................................................................   111
PF01030.1    ..............................................................................................................   205
PF00757.1    ..............................................................................................................   153
EGFR-206     PSIATGMVGALLLLLVVALGIGLFMRRRHIVRKRTLRRLLQERELVEPLTPSGEAPNQALLRILKETEFKKIKVLGSGAFGTVYKGLWIPEGEKVKIPVAIKELREATSP   708
EGFR-203     PAAQESCLGWIPSLLPSEFQLG......................................................WGG..CSHLHAWPSASVIITASS...........   703
EGFR-207     PSIATGMVGALLLLLVVALGIGLFMRRRHIVRKRTLRRLLQERELVEPLTPSGEAPNQALLRILKETEFKKIKVLGSGAFGTVYKGLWIPEGEKVKIPVAIKELREATSP   708
EGFR-205     ..............................................................................................................   128
EGFR-202     ..............................................................................................................   628
EGFR-204     ..............................................................................................................   405
EGFR-201     PSIATGMVGALLLLLVVALGIGLFMRRRHIVRKRTLRRLLQERELVEPLTPSGEAPNQALLRILKETEFKKIKVLGSGAFGTVYKGLWIPEGEKVKIPVAIKELREATSP   753

logo
             KANKEILDEAYVMASVDNPHVCRLLGICLTSTVQLITQLMPFGCLLDYVREHKDNIGSQYLLNWCVQIAKGMNYLEDRRLVHRDLAARNVLVKTPQHVKITDFGLAKLLG
ERLOTINIB    ..................................L.TQLMPFGC..D...........................................L.........TD........   17
PF01030      ..............................................................................................................   231
PF00757      ..............................................................................................................   154
PF14843.2    ..............................................................................................................   125
PF07714      KANKEILDEAYVMASVDNPHVCRLLGICLTSTVQLITQLMPFGCLLDYVREHKDNIGSQYLLNWCVQIAKGMNYLEDRRLVHRDLAARNVLVKTPQHVKITDFGLAKLLG   150
PF14843.1    ..............................................................................................................   123
PF14843      ..............................................................................................................   132
PF01030.2    ..............................................................................................................   111
PF01030.1    ..............................................................................................................   205
PF00757.1    ..............................................................................................................   153
EGFR-206     KANKEILDEAYVMASVDNPHVCRLLGICLTSTVQLITQLMPFGCLLDYVREHKDNIGSQYLLNWCVQIAKGMNYLEDRRLVHRDLAARNVLVKTPQHVKITDFGLAKLLG   818
EGFR-203     .....................CH.......................................................................................   705
EGFR-207     KANKEILDEAYVMASVDNPHVCRLLGICLTSTVQLITQLMPFGCLLDYVREHKDNIGSQYLLNWCVQIAKGMNYLEDRRLVHRDLAARNVLVKTPQHVKITDFGLAKLLG   818
EGFR-205     ..............................................................................................................   128
EGFR-202     ..............................................................................................................   628
EGFR-204     ..............................................................................................................   405
EGFR-201     KANKEILDEAYVMASVDNPHVCRLLGICLTSTVQLITQLMPFGCLLDYVREHKDNIGSQYLLNWCVQIAKGMNYLEDRRLVHRDLAARNVLVKTPQHVKITDFGLAKLLG   863
```

```
logo
             AEEKEYHAEGGKVPIKWMALESILHRIYTHQSDVWSYGVTVWELMTFGSKPYDGIPASEISSILEKGERLPQPPICTIDVYMIMVKCWMIDADSRPKFRELIIEFSKMAR
ERLOTINIB    ..............................................................................................................   17
PF01030      ..............................................................................................................  231
PF00757      ..............................................................................................................  154
PF14843.2    ..............................................................................................................  125
PF07714      AEEKEYHAEGGKVPIKWMALESILHRIYTHQSDVWSYGVTVWELMTFGSKPYDGIPASEISSILEKGERLPQPPICTIDVYMIMVKCWMIDADSRPKFRELI.........  252
PF14843.1    ..............................................................................................................  123
PF14843      ..............................................................................................................  132
PF01030.2    ..............................................................................................................  111
PF01030.1    ..............................................................................................................  205
PF00757.1    ..............................................................................................................  153
EGFR-206     AEEKEYHAEGGKVPIKWMALESILHRIYTHQSDVWSYGVTVWELMTFGSKPYDGIPASEISSILEKGERLPQPPICTIDVYMIMVKCWMIDADSRPKFRELIIEFSKMAR  928
EGFR-203     ..............................................................................................................  705
EGFR-207     AEEKEYHAEGGKVPIKWMALESILHRIYTHQSDVWSYGVTVWELMTFGSKPYDGIPASEISSILEKGERLPQPPICTIDVYMIMVKCWMIDADSRPKFRELIIEFSKMAR  928
EGFR-205     ..............................................................................................................  128
EGFR-202     ..............................................................................................................  628
EGFR-204     ..............................................................................................................  405
EGFR-201     AEEKEYHAEGGKVPIKWMALESILHRIYTHQSDVWSYGVTVWELMTFGSKPYDGIPASEISSILEKGERLPQPPICTIDVYMIMVKCWMIDADSRPKFRELIIEFSKMAR  973
```

```
logo
             DPQRYLVIQGDERMHLPSPTDSNFYRALMDEEDMDDVVDADEYLIPQQGFFSSPSTSRTPLLSSLSATSNNSTVACIDRNGLQSCPIKEDSFLQRYSSDPTGALTEDSID
ERLOTINIB    ..............................................................................................................   17
PF01030      ..............................................................................................................  231
PF00757      ..............................................................................................................  154
PF14843.2    ..............................................................................................................  125
PF07714      ..............................................................................................................  252
PF14843.1    ..............................................................................................................  123
PF14843      ..............................................................................................................  132
PF01030.2    ..............................................................................................................  111
PF01030.1    ..............................................................................................................  205
PF00757.1    ..............................................................................................................  153
EGFR-206     DPQRYLVIQGDERMHLPSPTDSNFYRALMDEEDMDDVVDADEYLIPQQGFFSSPSTSRTPLLSSLSATSNNSTVACIDRNGLQSCPIKEDSFLQRYSSDPTGALTEDSID 1038
EGFR-203     ..............................................................................................................  705
EGFR-207     DPQRYLVIQGDERMHLPSPTDSNFYRALMDEEDMDDVVDADEYLIPQQGFFSSPSTSRTPLLSSLSATSNNSTVACIDRNGLQSCPIKEDSFLQRYSSDPTGALTEDSID 1038
EGFR-205     ..............................................................................................................  128
EGFR-202     ..............................................................................................................  628
EGFR-204     ..............................................................................................................  405
EGFR-201     DPQRYLVIQGDERMHLPSPTDSNFYRALMDEEDMDDVVDADEYLIPQQGFFSSPSTSRTPLLSSLSATSNNSTVACIDRNGLQSCPIKEDSFLQRYSSDPTGALTEDSID 1083
```

```
logo

            [illegible]
ERLOTINIB   ..............................................................................................................    17
PF01030     ..............................................................................................................   231
PF00757     ..............................................................................................................   154
PF14843.2   ..............................................................................................................   125
PF07714     ..............................................................................................................   252
PF14843.1   ..............................................................................................................   123
PF14843     ..............................................................................................................   132
PF01030.2   ..............................................................................................................   111
PF01030.1   ..............................................................................................................   205
PF00757.1   ..............................................................................................................   153
EGFR-206    DTFLPVPEYINQSVP........KRPAGSVQNPVYHNQPLNPAPSRDPHYQDPHSTAVGNPEYLNTVQPTCVNSTFDSPAHWAQKGSHQISLDNPDYQQDFFPKEAKPNG  1140
EGFR-203    ..............................................................................................................   705
EGFR-207    DTFLPVPGEWLVWKQSCSSTSSTHSAAASLQCPSQVLPPASPEGETVADLQTQ.........................................................  1091
EGFR-205    ..............................................................................................................   128
EGFR-202    ..............................................................................................................   628
EGFR-204    ..............................................................................................................   405
EGFR-201    DTFLPVPEYINQSVP........KRPAGSVQNPVYHNQPLNPAPSRDPHYQDPHSTAVGNPEYLNTVQPTCVNSTFDSPAHWAQKGSHQISLDNPDYQQDFFPKEAKPNG  1185


logo

            IFKGSTAENAEYLRVAPQSSEFIGA
ERLOTINIB   .........................    17
PF01030     .........................   231
PF00757     .........................   154
PF14843.2   .........................   125
PF07714     .........................   252
PF14843.1   .........................   123
PF14843     .........................   132
PF01030.2   .........................   111
PF01030.1   .........................   205
PF00757.1   .........................   153
EGFR-206    IFKGSTAENAEYLRVAPQSSEFIGA  1165
EGFR-203    .........................   705
EGFR-207    .........................  1091
EGFR-205    .........................   128
EGFR-202    .........................   628
EGFR-204    .........................   405
EGFR-201    IFKGSTAENAEYLRVAPQSSEFIGA  1210
```

X non conserved

X similar

X ≥ 0% conserved

X ≥ 50% conserved

```
logo         MRPSGTAGAALLALLAALCPASRALEEKKVCQGTSNKLTQLGTFEDHFLSLQRMFNNCEWLGNLEITYVQRNYDLSFLKTIQEVAGYVLIALNTVERIPLENLQIIRGN
GEFITINIB    ..............................................................................................................    0
PF01030      ........................................................NCEVVLGNLEITYVQRNYDLSFLKTIQEVAGYVLIALNTVERIPLENLQIIRGN   54
PF00757      ..............................................................................................................    0
PF14843.2    ..............................................................................................................    0
PF07714      ..............................................................................................................    0
PF14843.1    ..............................................................................................................    0
PF14843      ..............................................................................................................    0
PF01030.2    ........................................................NCEVVLGNLEITYVQRNYDLSFLKTIQEVAGYVLIALNTVERIPLENLQIIRGN   54
PF01030.1    ........................................................NCEVVLGNLEITYVQRNYDLSFLKTIQEVAGYVLIALNTVERIPLENLQIIRGN   54
PF00757.1    ..............................................................................................................    0
EGFR-206     MRPSGTAGAALLALLAALCPASRALEEKKVCQGTSNKLTQLGTFEDHFLSLQRMFNNCEVVLGNLEITYVQRNYDLSFLKTIQEVAGYVLIALNTVERIPLENLQIIRGN  110
EGFR-203     MRPSGTAGAALLALLAALCPASRALEEKKVCQGTSNKLTQLGTFEDHFLSLQRMFNNCEVVLGNLEITYVQRNYDLSFLKTIQEVAGYVLIALNTVERIPLENLQIIRGN  110
EGFR-207     MRPSGTAGAALLALLAALCPASRALEEKKVCQGTSNKLTQLGTFEDHFLSLQRMFNNCEVVLGNLEITYVQRNYDLSFLKTIQEVAGYVLIALNTVERIPLENLQIIRGN  110
EGFR-205     .....................................................MFNNCEVVLGNLEITYVQRNYDLSFLKTIQEVAGYVLIALNTVERIPLENLQIIRGN   57
EGFR-202     MRPSGTAGAALLALLAALCPASRALEEKKVCQGTSNKLTQLGTFEDHFLSLQRMFNNCEVVLGNLEITYVQRNYDLSFLKTIQEVAGYVLIALNTVERIPLENLQIIRGN  110
EGFR-204     MRPSGTAGAALLALLAALCPASRALEEKKVCQGTSNKLTQLGTFEDHFLSLQRMFNNCEVVLGNLEITYVQRNYDLSFLKTIQEVAGYVLIALNTVERIPLENLQIIRGN  110
EGFR-201     MRPSGTAGAALLALLAALCPASRALEEKKVCQGTSNKLTQLGTFEDHFLSLQRMFNNCEVVLGNLEITYVQRNYDLSFLKTIQEVAGYVLIALNTVERIPLENLQIIRGN  110

logo         MYYENSYALAVLSNYDANKTGLKELPMRNLQEILHGAVRFSNNPALCNVESIQWRDIVSSDFLSNMSMDFQNHLGSCQKCDPSCPNGSCWGAGEENCQKLTKIICAQQCS
GEFITINIB    ..............................................................................................................    0
PF01030      MYYENSYALAVLSNYDANKTGLKELPMRNLQEILHGAVRFSNNPALCNVESIQWRDI.....................................................  111
PF00757      ..........................................................................GSCQKCDPSCPNGSCWGAGEENCQKLTKIICAQQCS   36
PF14843.2    ..............................................................................................................    0
PF07714      ..............................................................................................................    0
PF14843.1    ..............................................................................................................    0
PF14843      ..............................................................................................................    0
PF01030.2    MYYENSYALAVLSNYDANKTGLKELPMRNLQEILHGAVRFSNNPALCNVESIQWRDI.....................................................  111
PF01030.1    MYYENSYALAVLSNYDANKTGLKELPMRNLQ...............................................................................   85
PF00757.1    ...............................QG............................................QKCDPSCPNGSCWGAGEENCQKLTKIICAQQCS   35
EGFR-206     MYYENSYALAVLSNYDANKTGLKELPMRNLQG.............................................QKCDPSCPNGSCWGAGEENCQKLTKIICAQQCS  175
EGFR-203     MYYENSYALAVLSNYDANKTGLKELPMRNLQEILHGAVRFSNNPALCNVESIQWRDIVSSDFLSNMSMDFQNHLGSCQKCDPSCPNGSCWGAGEENCQKLTKIICAQQCS  220
EGFR-207     MYYENSYALAVLSNYDANKTGLKELPMRNLQG.............................................QKCDPSCPNGSCWGAGEENCQKLTKIICAQQCS  175
EGFR-205     MYYENSYALAVLSNYDANKTGLKELPMRNLQEILHGAVRFSNNPALCNVESIQWRDIVSSDFLSNMSMDFQ.......................................  128
EGFR-202     MYYENSYALAVLSNYDANKTGLKELPMRNLQEILHGAVRFSNNPALCNVESIQWRDIVSSDFLSNMSMDFQNHLGSCQKCDPSCPNGSCWGAGEENCQKLTKIICAQQCS  220
EGFR-204     MYYENSYALAVLSNYDANKTGLKELPMRNLQEILHGAVRFSNNPALCNVESIQWRDIVSSDFLSNMSMDFQNHLGSCQKCDPSCPNGSCWGAGEENCQKLTKIICAQQCS  220
EGFR-201     MYYENSYALAVLSNYDANKTGLKELPMRNLQEILHGAVRFSNNPALCNVESIQWRDIVSSDFLSNMSMDFQNHLGSCQKCDPSCPNGSCWGAGEENCQKLTKIICAQQCS  220
```

```
logo
             GRCRGKSPSDCCHNQCAAGCTGPRESDCLVCRKFRDEATCKDTCPPLMLYNPTTYQMDVNPEGKYSFGATCVKKCPRNYVVTDHGSCVRACGADSYEMEEDGVRKCKKCE
GEFITINIB    ..............................................................................................................   0
PF01030      ..............................................................................................................   111
PF00757      GRCRGKSPSDCCHNQCAAGCTGPRESDCLVCRKFRDEATCKDTCPPLMLYNPTTYQMDVNPEGKYSFGATCVKKCPRNYVVTDHGSCVRACGADSYEMEEDGVRKCKKCE   146
PF14843.2    ..............................................................................................................   0
PF07714      ..............................................................................................................   0
PF14843.1    ..............................................................................................................   0
PF14843      ..............................................................................................................   0
PF01030.2    ..............................................................................................................   111
PF01030.1    ..............................................................................................................   85
PF00757.1    GRCRGKSPSDCCHNQCAAGCTGPRESDCLVCRKFRDEATCKDTCPPLMLYNPTTYQMDVNPEGKYSFGATCVKKCPRNYVVTDHGSCVRACGADSYEMEEDGVRKCKKCE   145
EGFR-206     GRCRGKSPSDCCHNQCAAGCTGPRESDCLVCRKFRDEATCKDTCPPLMLYNPTTYQMDVNPEGKYSFGATCVKKCPRNYVVTDHGSCVRACGADSYEMEEDGVRKCKKCE   285
EGFR-203     GRCRGKSPSDCCHNQCAAGCTGPRESDCLVCRKFRDEATCKDTCPPLMLYNPTTYQMDVNPEGKYSFGATCVKKCPRNYVVTDHGSCVRACGADSYEMEEDGVRKCKKCE   330
EGFR-207     GRCRGKSPSDCCHNQCAAGCTGPRESDCLVCRKFRDEATCKDTCPPLMLYNPTTYQMDVNPEGKYSFGATCVKKCPRNYVVTDHGSCVRACGADSYEMEEDGVRKCKKCE   285
EGFR-205     ..............................................................................................................   128
EGFR-202     GRCRGKSPSDCCHNQCAAGCTGPRESDCLVCRKFRDEATCKDTCPPLMLYNPTTYQMDVNPEGKYSFGATCVKKCPRNYVVTDHGSCVRACGADSYEMEEDGVRKCKKCE   330
EGFR-204     GRCRGKSPSDCCHNQCAAGCTGPRESDCLVCRKFRDEATCKDTCPPLMLYNPTTYQMDVNPEGKYSFGATCVKKCPRNYVVTDHGSCVRACGADSYEMEEDGVRKCKKCE   330
EGFR-201     GRCRGKSPSDCCHNQCAAGCTGPRESDCLVCRKFRDEATCKDTCPPLMLYNPTTYQMDVNPEGKYSFGATCVKKCPRNYVVTDHGSCVRACGADSYEMEEDGVRKCKKCE   330

logo
             GPCRKVCNGIGIGEFKDSLSINATNIKHFKNCTSISGDLHILPVAFRGDSFTHTPPLDPQELDILKTVKEITGFLLIQAWPENRTDLHAFENLEIIRGRTKQHGQFSLAV
GEFITINIB    ..............................................................................................................   0
PF01030      ..............................NCTSISGDLHILPVAFRGDSFTHTPPLDPQELDILKTVKEITGFLLIQAWPENRTDLHAFENLEIIRGRTKQHGQFSLAV   191
PF00757      GPCRKVCN......................................................................................................   154
PF14843.2    ..............................................................................................................   0
PF07714      ..............................................................................................................   0
PF14843.1    ..............................................................................................................   0
PF14843      ..............................................................................................................   0
PF01030.2    ..............................................................................................................   111
PF01030.1    ..............................NCTSISGDLHILPVAFRGDSFTHTPPLDPQELDILKTVKEITGFLLIQAWPENRTDLHAFENLEIIRGRTKQHGQFSLAV   165
PF00757.1    GPCRKVCN......................................................................................................   153
EGFR-206     GPCRKVCNGIGIGEFKDSLSINATNIKHFKNCTSISGDLHILPVAFRGDSFTHTPPLDPQELDILKTVKEITGFLLIQAWPENRTDLHAFENLEIIRGRTKQHGQFSLAV   395
EGFR-203     GPCRKVCNGIGIGEFKDSLSINATNIKHFKNCTSISGDLHILPVAFRGDSFTHTPPLDPQELDILKTVKEITGFLLIQAWPENRTDLHAFENLEIIRGRTKQHGQFSLAV   440
EGFR-207     GPCRKVCNGIGIGEFKDSLSINATNIKHFKNCTSISGDLHILPVAFRGDSFTHTPPLDPQELDILKTVKEITGFLLIQAWPENRTDLHAFENLEIIRGRTKQHGQFSLAV   395
EGFR-205     ..............................................................................................................   128
EGFR-202     GPCRKVCNGIGIGEFKDSLSINATNIKHFKNCTSISGDLHILPVAFRGDSFTHTPPLDPQELDILKTVKEITGFLLIQAWPENRTDLHAFENLEIIRGRTKQHGQFSLAV   440
EGFR-204     GPCRKVCNGIGIGEFKDSLSINATNIKHFKNCTSISGDLHILPVAFRGDSFTHTPPLDPQELDILKTVKEITGLS...................................   405
EGFR-201     GPCRKVCNGIGIGEFKDSLSINATNIKHFKNCTSISGDLHILPVAFRGDSFTHTPPLDPQELDILKTVKEITGFLLIQAWPENRTDLHAFENLEIIRGRTKQHGQFSLAV   440
```

```
logo         VSLNITSLGLRSLKEISDGDVIISGNKNLCYANTINWKKLFGTSGQKTKIISNRGENSCKATGQVCHALCSPEGCWGPEPRDCVSCRNVSRGRECVDKCNLLEGEPREFV
GEFITINIB    ..............................................................................................................     0
PF01030      VSLNITSLGLRSLKEISDGDVIISGNKNLCYANTINWKKL......................................................................   231
PF00757      ..............................................................................................................   154
PF14843.2    ................................................................VCHALCSPEGCWGPEPRDCVSCRNVSRGRECVDKCNLLEGEPREFV    46
PF07714      ..............................................................................................................     0
PF14843.1    ................................................................VCHALCSPEGCWGPEPRDCVSCRNVSRGRECVDKCNLLEGEPREFV    46
PF14843      ................................................................VCHALCSPEGCWGPEPRDCVSCRNVSRGRECVDKCNLLEGEPREFV    46
PF01030.2    ..............................................................................................................   111
PF01030.1    VSLNITSLGLRSLKEISDGDVIISGNKNLCYANTINWKKL......................................................................   205
PF00757.1    ..............................................................................................................   153
EGFR-206     VSLNITSLGLRSLKEISDGDVIISGNKNLCYANTINWKKLFGTSGQKTKIISNRGENSCKATGQVCHALCSPEGCWGPEPRDCVSCRNVSRGRECVDKCNLLEGEPREFV   505
EGFR-203     VSLNITSLGLRSLKEISDGDVIISGNKNLCYANTINWKKLFGTSGQKTKIISNRGENSCKATGQVCHALCSPEGCWGPEPRDCVSCRNVSRGRECVDKCNLLEGEPREFV   550
EGFR-207     VSLNITSLGLRSLKEISDGDVIISGNKNLCYANTINWKKLFGTSGQKTKIISNRGENSCKATGQVCHALCSPEGCWGPEPRDCVSCRNVSRGRECVDKCNLLEGEPREFV   505
EGFR-205     ..............................................................................................................   128
EGFR-202     VSLNITSLGLRSLKEISDGDVIISGNKNLCYANTINWKKLFGTSGQKTKIISNRGENSCKATGQVCHALCSPEGCWGPEPRDCVSCRNVSRGRECVDKCNLLEGEPREFV   550
EGFR-204     ..............................................................................................................   405
EGFR-201     VSLNITSLGLRSLKEISDGDVIISGNKNLCYANTINWKKLFGTSGQKTKIISNRGENSCKATGQVCHALCSPEGCWGPEPRDCVSCRNVSRGRECVDKCNLLEGEPREFV   550

logo         ENSECIQCHPECLPQAMNITCTGRGPDNCIQCAHYIDGPHCVKTCPAGVMGENNTLVWKYADAGHVCHLCHPNCTYGCTGPGLEGCPTNGPKI
GEFITINIB    ..............................................................................................................     0
PF01030      ..............................................................................................................   231
PF00757      ..............................................................................................................   154
PF14843.2    ENSECIQCHPECLPQAMNITCTGRGPDNCIQCAHYIDGPHCVKTCPAGVMGENNTLVWKYADAGHVCHLCHPNCTYGPG...............................   125
PF07714      ..............................................................................................................     0
PF14843.1    ENSECIQCHPECLPQAMNITCTGRGPDNCIQCAHYIDGPHCVKTCPAGVMGENNTLVWKYADAGHVCHLCHPNCTYG.................................   123
PF14843      ENSECIQCHPECLPQAMNITCTGRGPDNCIQCAHYIDGPHCVKTCPAGVMGENNTLVWKYADAGHVCHLCHPNCTYGCTGPGLEGC........................   132
PF01030.2    ..............................................................................................................   111
PF01030.1    ..............................................................................................................   205
PF00757.1    ..............................................................................................................   153
EGFR-206     ENSECIQCHPECLPQAMNITCTGRGPDNCIQCAHYIDGPHCVKTCPAGVMGENNTLVWKYADAGHVCHLCHPNCTYGCTGPGLEGCPTNGPKI.................   598
EGFR-203     ENSECIQCHPECLPQAMNITCTGRGPDNCIQCAHYIDGPHCVKTCPAGVMGENNTLVWKYADAGHVCHLCHPNCTYGPGNESLKAMLFCLFKLSSCNQSNDGSVHSQSGS   660
EGFR-207     ENSECIQCHPECLPQAMNITCTGRGPDNCIQCAHYIDGPHCVKTCPAGVMGENNTLVWKYADAGHVCHLCHPNCTYGCTGPGLEGCPTNGPKI.................   598
EGFR-205     ..............................................................................................................   128
EGFR-202     ENSECIQCHPECLPQAMNITCTGRGPDNCIQCAHYIDGPHCVKTCPAGVMGENNTLVWKYADAGHVCHLCHPNCTYGS................................   628
EGFR-204     ..............................................................................................................   405
EGFR-201     ENSECIQCHPECLPQAMNITCTGRGPDNCIQCAHYIDGPHCVKTCPAGVMGENNTLVWKYADAGHVCHLCHPNCTYGCTGPGLEGCPTNGPKI.................   643
```

logo

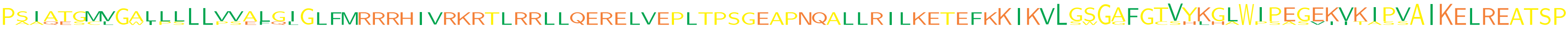

```
GEFITINIB    ..........................................................................LS......V................AIK........   6
PF01030      ..............................................................................................................   231
PF00757      ..............................................................................................................   154
PF14843.2    ..............................................................................................................   125
PF07714      ......................................................................KIKVLGSGAFGTVYKGLWIPEGEKVKIPVAIKELREATSP   40
PF14843.1    ..............................................................................................................   123
PF14843      ..............................................................................................................   132
PF01030.2    ..............................................................................................................   111
PF01030.1    ..............................................................................................................   205
PF00757.1    ..............................................................................................................   153
EGFR-206     PSIATGMVGALLLLLVVALGIGLFMRRRHIVRKRTLRRLLQERELVEPLTPSGEAPNQALLRILKETEFKKIKVLGSGAFGTVYKGLWIPEGEKVKIPVAIKELREATSP   708
EGFR-203     PAAQESCLGWIPSLLPSEFQLG......................................................WGG..CSHLHAWPSASVIITASS...........   703
EGFR-207     PSIATGMVGALLLLLVVALGIGLFMRRRHIVRKRTLRRLLQERELVEPLTPSGEAPNQALLRILKETEFKKIKVLGSGAFGTVYKGLWIPEGEKVKIPVAIKELREATSP   708
EGFR-205     ..............................................................................................................   128
EGFR-202     ..............................................................................................................   628
EGFR-204     ..............................................................................................................   405
EGFR-201     PSIATGMVGALLLLLVVALGIGLFMRRRHIVRKRTLRRLLQERELVEPLTPSGEAPNQALLRILKETEFKKIKVLGSGAFGTVYKGLWIPEGEKVKIPVAIKELREATSP   753
```

logo

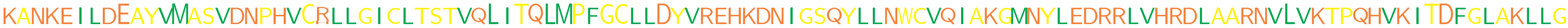

```
GEFITINIB    ........E...M.....................L.TQLMP.GC..D...........................................L.........TD........   20
PF01030      ..............................................................................................................   231
PF00757      ..............................................................................................................   154
PF14843.2    ..............................................................................................................   125
PF07714      KANKEILDEAYVMASVDNPHVCRLLGICLTSTVQLITQLMPFGCLLDYVREHKDNIGSQYLLNWCVQIAKGMNYLEDRRLVHRDLAARNVLVKTPQHVKITDFGLAKLLG   150
PF14843.1    ..............................................................................................................   123
PF14843      ..............................................................................................................   132
PF01030.2    ..............................................................................................................   111
PF01030.1    ..............................................................................................................   205
PF00757.1    ..............................................................................................................   153
EGFR-206     KANKEILDEAYVMASVDNPHVCRLLGICLTSTVQLITQLMPFGCLLDYVREHKDNIGSQYLLNWCVQIAKGMNYLEDRRLVHRDLAARNVLVKTPQHVKITDFGLAKLLG   818
EGFR-203     .....................CH.......................................................................................   705
EGFR-207     KANKEILDEAYVMASVDNPHVCRLLGICLTSTVQLITQLMPFGCLLDYVREHKDNIGSQYLLNWCVQIAKGMNYLEDRRLVHRDLAARNVLVKTPQHVKITDFGLAKLLG   818
EGFR-205     ..............................................................................................................   128
EGFR-202     ..............................................................................................................   628
EGFR-204     ..............................................................................................................   405
EGFR-201     KANKEILDEAYVMASVDNPHVCRLLGICLTSTVQLITQLMPFGCLLDYVREHKDNIGSQYLLNWCVQIAKGMNYLEDRRLVHRDLAARNVLVKTPQHVKITDFGLAKLLG   863
```

```
logo
             AEEKEYHAEGGKVPIKWMALESILHRIYTHQSDVWSYGVTVWELMTFGSKPYDGIPASEISSILEKGERLPQPPICTIDVYMIMVKCWMIDADSRPKFRELIIEFSKMAR
GEFITINIB    ..............................................................................................................    20
PF01030      ..............................................................................................................   231
PF00757      ..............................................................................................................   154
PF14843.2    ..............................................................................................................   125
PF07714      AEEKEYHAEGGKVPIKWMALESILHRIYTHQSDVWSYGVTVWELMTFGSKPYDGIPASEISSILEKGERLPQPPICTIDVYMIMVKCWMIDADSRPKFRELI.........   252
PF14843.1    ..............................................................................................................   123
PF14843      ..............................................................................................................   132
PF01030.2    ..............................................................................................................   111
PF01030.1    ..............................................................................................................   205
PF00757.1    ..............................................................................................................   153
EGFR-206     AEEKEYHAEGGKVPIKWMALESILHRIYTHQSDVWSYGVTVWELMTFGSKPYDGIPASEISSILEKGERLPQPPICTIDVYMIMVKCWMIDADSRPKFRELIIEFSKMAR   928
EGFR-203     ..............................................................................................................   705
EGFR-207     AEEKEYHAEGGKVPIKWMALESILHRIYTHQSDVWSYGVTVWELMTFGSKPYDGIPASEISSILEKGERLPQPPICTIDVYMIMVKCWMIDADSRPKFRELIIEFSKMAR   928
EGFR-205     ..............................................................................................................   128
EGFR-202     ..............................................................................................................   628
EGFR-204     ..............................................................................................................   405
EGFR-201     AEEKEYHAEGGKVPIKWMALESILHRIYTHQSDVWSYGVTVWELMTFGSKPYDGIPASEISSILEKGERLPQPPICTIDVYMIMVKCWMIDADSRPKFRELIIEFSKMAR   973

logo
             DPQRYLVIQGDERMHLPSPTDSNFYRALMDEEDMDDVVDADEYLIPQQGFFSSPSTSRTPLLSSLSATSNNSTVACIDRNGLQSCPIKEDSFLQRYSSDPTGALTEDSID
GEFITINIB    ..............................................................................................................    20
PF01030      ..............................................................................................................   231
PF00757      ..............................................................................................................   154
PF14843.2    ..............................................................................................................   125
PF07714      ..............................................................................................................   252
PF14843.1    ..............................................................................................................   123
PF14843      ..............................................................................................................   132
PF01030.2    ..............................................................................................................   111
PF01030.1    ..............................................................................................................   205
PF00757.1    ..............................................................................................................   153
EGFR-206     DPQRYLVIQGDERMHLPSPTDSNFYRALMDEEDMDDVVDADEYLIPQQGFFSSPSTSRTPLLSSLSATSNNSTVACIDRNGLQSCPIKEDSFLQRYSSDPTGALTEDSID  1038
EGFR-203     ..............................................................................................................   705
EGFR-207     DPQRYLVIQGDERMHLPSPTDSNFYRALMDEEDMDDVVDADEYLIPQQGFFSSPSTSRTPLLSSLSATSNNSTVACIDRNGLQSCPIKEDSFLQRYSSDPTGALTEDSID  1038
EGFR-205     ..............................................................................................................   128
EGFR-202     ..............................................................................................................   628
EGFR-204     ..............................................................................................................   405
EGFR-201     DPQRYLVIQGDERMHLPSPTDSNFYRALMDEEDMDDVVDADEYLIPQQGFFSSPSTSRTPLLSSLSATSNNSTVACIDRNGLQSCPIKEDSFLQRYSSDPTGALTEDSID  1083
```

```
logo

GEFITINIB   ..............................................................................................................   20
PF01030     ..............................................................................................................  231
PF00757     ..............................................................................................................  154
PF14843.2   ..............................................................................................................  125
PF07714     ..............................................................................................................  252
PF14843.1   ..............................................................................................................  123
PF14843     ..............................................................................................................  132
PF01030.2   ..............................................................................................................  111
PF01030.1   ..............................................................................................................  205
PF00757.1   ..............................................................................................................  153
EGFR-206    DTFLPVPEYINQSVP........KRPAGSVQNPVYHNQPLNPAPSRDPHYQDPHSTAVGNPEYLNTVQPTCVNSTFDSPAHWAQKGSHQISLDNPDYQQDFFPKEAKPNG 1140
EGFR-203    ..............................................................................................................  705
EGFR-207    DTFLPVPGEWLVWKQSCSSTSSTHSAAASLQCPSQVLPPASPEGETVADLQTQ......................................................... 1091
EGFR-205    ..............................................................................................................  128
EGFR-202    ..............................................................................................................  628
EGFR-204    ..............................................................................................................  405
EGFR-201    DTFLPVPEYINQSVP........KRPAGSVQNPVYHNQPLNPAPSRDPHYQDPHSTAVGNPEYLNTVQPTCVNSTFDSPAHWAQKGSHQISLDNPDYQQDFFPKEAKPNG 1185
```

```
logo

GEFITINIB   .........................   20
PF01030     .........................  231
PF00757     .........................  154
PF14843.2   .........................  125
PF07714     .........................  252
PF14843.1   .........................  123
PF14843     .........................  132
PF01030.2   .........................  111
PF01030.1   .........................  205
PF00757.1   .........................  153
EGFR-206    IFKGSTAENAEYLRVAPQSSEFIGA 1165
EGFR-203    .........................  705
EGFR-207    ......................... 1091
EGFR-205    .........................  128
EGFR-202    .........................  628
EGFR-204    .........................  405
EGFR-201    IFKGSTAENAEYLRVAPQSSEFIGA 1210
```

- X non conserved
- X similar
- X $\geq$ 0% conserved
- X $\geq$ 50% conserved

logo

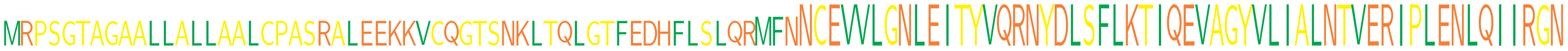

```
LAPATINIB   ..............................................................................................................   0
PF01030     ........................................................NCEVVLGNLEITYVQRNYDLSFLKTIQEVAGYVLIALNTVERIPLENLQIIRGN   54
PF00757     ..............................................................................................................   0
PF14843.2   ..............................................................................................................   0
PF07714     ..............................................................................................................   0
PF14843.1   ..............................................................................................................   0
PF14843     ..............................................................................................................   0
PF01030.2   ........................................................NCEVVLGNLEITYVQRNYDLSFLKTIQEVAGYVLIALNTVERIPLENLQIIRGN   54
PF01030.1   ........................................................NCEVVLGNLEITYVQRNYDLSFLKTIQEVAGYVLIALNTVERIPLENLQIIRGN   54
PF00757.1   ..............................................................................................................   0
EGFR-206    MRPSGTAGAALLALLAALCPASRALEEKKVCQGTSNKLTQLGTFEDHFLSLQRMFNNCEVVLGNLEITYVQRNYDLSFLKTIQEVAGYVLIALNTVERIPLENLQIIRGN   110
EGFR-203    MRPSGTAGAALLALLAALCPASRALEEKKVCQGTSNKLTQLGTFEDHFLSLQRMFNNCEVVLGNLEITYVQRNYDLSFLKTIQEVAGYVLIALNTVERIPLENLQIIRGN   110
EGFR-207    MRPSGTAGAALLALLAALCPASRALEEKKVCQGTSNKLTQLGTFEDHFLSLQRMFNNCEVVLGNLEITYVQRNYDLSFLKTIQEVAGYVLIALNTVERIPLENLQIIRGN   110
EGFR-205    .....................................................MFNNCEVVLGNLEITYVQRNYDLSFLKTIQEVAGYVLIALNTVERIPLENLQIIRGN   57
EGFR-202    MRPSGTAGAALLALLAALCPASRALEEKKVCQGTSNKLTQLGTFEDHFLSLQRMFNNCEVVLGNLEITYVQRNYDLSFLKTIQEVAGYVLIALNTVERIPLENLQIIRGN   110
EGFR-204    MRPSGTAGAALLALLAALCPASRALEEKKVCQGTSNKLTQLGTFEDHFLSLQRMFNNCEVVLGNLEITYVQRNYDLSFLKTIQEVAGYVLIALNTVERIPLENLQIIRGN   110
EGFR-201    MRPSGTAGAALLALLAALCPASRALEEKKVCQGTSNKLTQLGTFEDHFLSLQRMFNNCEVVLGNLEITYVQRNYDLSFLKTIQEVAGYVLIALNTVERIPLENLQIIRGN   110
```

logo

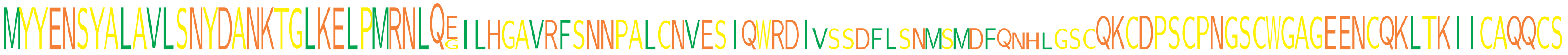

```
LAPATINIB   ..............................................................................................................   0
PF01030     MYYENSYALAVLSNYDANKTGLKELPMRNLQEILHGAVRFSNNPALCNVESIQWRDI......................................................   111
PF00757     ..........................................................................GSCQKCDPSCPNGSCWGAGEENCQKLTKIICAQQCS   36
PF14843.2   ..............................................................................................................   0
PF07714     ..............................................................................................................   0
PF14843.1   ..............................................................................................................   0
PF14843     ..............................................................................................................   0
PF01030.2   MYYENSYALAVLSNYDANKTGLKELPMRNLQEILHGAVRFSNNPALCNVESIQWRDI......................................................   111
PF01030.1   MYYENSYALAVLSNYDANKTGLKELPMRNLQ...............................................................................   85
PF00757.1   ..............................QG.............................................QKCDPSCPNGSCWGAGEENCQKLTKIICAQQCS   35
EGFR-206    MYYENSYALAVLSNYDANKTGLKELPMRNLQG.............................................QKCDPSCPNGSCWGAGEENCQKLTKIICAQQCS   175
EGFR-203    MYYENSYALAVLSNYDANKTGLKELPMRNLQEILHGAVRFSNNPALCNVESIQWRDIVSSDFLSNMSMDFQNHLGSCQKCDPSCPNGSCWGAGEENCQKLTKIICAQQCS   220
EGFR-207    MYYENSYALAVLSNYDANKTGLKELPMRNLQG.............................................QKCDPSCPNGSCWGAGEENCQKLTKIICAQQCS   175
EGFR-205    MYYENSYALAVLSNYDANKTGLKELPMRNLQEILHGAVRFSNNPALCNVESIQWRDIVSSDFLSNMSMDFQ.......................................   128
EGFR-202    MYYENSYALAVLSNYDANKTGLKELPMRNLQEILHGAVRFSNNPALCNVESIQWRDIVSSDFLSNMSMDFQNHLGSCQKCDPSCPNGSCWGAGEENCQKLTKIICAQQCS   220
EGFR-204    MYYENSYALAVLSNYDANKTGLKELPMRNLQEILHGAVRFSNNPALCNVESIQWRDIVSSDFLSNMSMDFQNHLGSCQKCDPSCPNGSCWGAGEENCQKLTKIICAQQCS   220
EGFR-201    MYYENSYALAVLSNYDANKTGLKELPMRNLQEILHGAVRFSNNPALCNVESIQWRDIVSSDFLSNMSMDFQNHLGSCQKCDPSCPNGSCWGAGEENCQKLTKIICAQQCS   220
```

```
logo
            GRCRGKSPSDCCHNQCAAGCTGPRESDCLVCRKFRDEATCKDTCPPLMLYNPTTYQMDVNPEGKYSFGATCVKKCPRNYVVTDHGSCVRACGADSYEMEEDGVRKCKKCE
LAPATINIB   ..............................................................................................................   0
PF01030     ..............................................................................................................   111
PF00757     GRCRGKSPSDCCHNQCAAGCTGPRESDCLVCRKFRDEATCKDTCPPLMLYNPTTYQMDVNPEGKYSFGATCVKKCPRNYVVTDHGSCVRACGADSYEMEEDGVRKCKKCE   146
PF14843.2   ..............................................................................................................   0
PF07714     ..............................................................................................................   0
PF14843.1   ..............................................................................................................   0
PF14843     ..............................................................................................................   0
PF01030.2   ..............................................................................................................   111
PF01030.1   ..............................................................................................................   85
PF00757.1   GRCRGKSPSDCCHNQCAAGCTGPRESDCLVCRKFRDEATCKDTCPPLMLYNPTTYQMDVNPEGKYSFGATCVKKCPRNYVVTDHGSCVRACGADSYEMEEDGVRKCKKCE   145
EGFR-206    GRCRGKSPSDCCHNQCAAGCTGPRESDCLVCRKFRDEATCKDTCPPLMLYNPTTYQMDVNPEGKYSFGATCVKKCPRNYVVTDHGSCVRACGADSYEMEEDGVRKCKKCE   285
EGFR-203    GRCRGKSPSDCCHNQCAAGCTGPRESDCLVCRKFRDEATCKDTCPPLMLYNPTTYQMDVNPEGKYSFGATCVKKCPRNYVVTDHGSCVRACGADSYEMEEDGVRKCKKCE   330
EGFR-207    GRCRGKSPSDCCHNQCAAGCTGPRESDCLVCRKFRDEATCKDTCPPLMLYNPTTYQMDVNPEGKYSFGATCVKKCPRNYVVTDHGSCVRACGADSYEMEEDGVRKCKKCE   285
EGFR-205    ..............................................................................................................   128
EGFR-202    GRCRGKSPSDCCHNQCAAGCTGPRESDCLVCRKFRDEATCKDTCPPLMLYNPTTYQMDVNPEGKYSFGATCVKKCPRNYVVTDHGSCVRACGADSYEMEEDGVRKCKKCE   330
EGFR-204    GRCRGKSPSDCCHNQCAAGCTGPRESDCLVCRKFRDEATCKDTCPPLMLYNPTTYQMDVNPEGKYSFGATCVKKCPRNYVVTDHGSCVRACGADSYEMEEDGVRKCKKCE   330
EGFR-201    GRCRGKSPSDCCHNQCAAGCTGPRESDCLVCRKFRDEATCKDTCPPLMLYNPTTYQMDVNPEGKYSFGATCVKKCPRNYVVTDHGSCVRACGADSYEMEEDGVRKCKKCE   330

logo
            GPCRKVCNGIGIGEFKDSLSINATNIKHFKNCTSISGDLHILPVAFRGDSFTHTPPLDPQELDILKTVKEITGFLLIQAWPENRTDLHAFENLEIIRGRTKQHGQFSLAV
LAPATINIB   ..............................................................................................................   0
PF01030     ..............................NCTSISGDLHILPVAFRGDSFTHTPPLDPQELDILKTVKEITGFLLIQAWPENRTDLHAFENLEIIRGRTKQHGQFSLAV   191
PF00757     GPCRKVCN......................................................................................................   154
PF14843.2   ..............................................................................................................   0
PF07714     ..............................................................................................................   0
PF14843.1   ..............................................................................................................   0
PF14843     ..............................................................................................................   0
PF01030.2   ..............................................................................................................   111
PF01030.1   ..............................NCTSISGDLHILPVAFRGDSFTHTPPLDPQELDILKTVKEITGFLLIQAWPENRTDLHAFENLEIIRGRTKQHGQFSLAV   165
PF00757.1   GPCRKVCN......................................................................................................   153
EGFR-206    GPCRKVCNGIGIGEFKDSLSINATNIKHFKNCTSISGDLHILPVAFRGDSFTHTPPLDPQELDILKTVKEITGFLLIQAWPENRTDLHAFENLEIIRGRTKQHGQFSLAV   395
EGFR-203    GPCRKVCNGIGIGEFKDSLSINATNIKHFKNCTSISGDLHILPVAFRGDSFTHTPPLDPQELDILKTVKEITGFLLIQAWPENRTDLHAFENLEIIRGRTKQHGQFSLAV   440
EGFR-207    GPCRKVCNGIGIGEFKDSLSINATNIKHFKNCTSISGDLHILPVAFRGDSFTHTPPLDPQELDILKTVKEITGFLLIQAWPENRTDLHAFENLEIIRGRTKQHGQFSLAV   395
EGFR-205    ..............................................................................................................   128
EGFR-202    GPCRKVCNGIGIGEFKDSLSINATNIKHFKNCTSISGDLHILPVAFRGDSFTHTPPLDPQELDILKTVKEITGFLLIQAWPENRTDLHAFENLEIIRGRTKQHGQFSLAV   440
EGFR-204    GPCRKVCNGIGIGEFKDSLSINATNIKHFKNCTSISGDLHILPVAFRGDSFTHTPPLDPQELDILKTVKEITGLS...................................   405
EGFR-201    GPCRKVCNGIGIGEFKDSLSINATNIKHFKNCTSISGDLHILPVAFRGDSFTHTPPLDPQELDILKTVKEITGFLLIQAWPENRTDLHAFENLEIIRGRTKQHGQFSLAV   440
```

```
logo
             VSLNITSLGLRSLKEISDGDVIISGNKNLCYANTINWKKLFGTSGQKTKIISNRGENSCKATGQVCHALCSPEGCWGPEPRDCVSCRNVSRGRECVDKCNLLEGEPREFV
LAPATINIB    ..............................................................................................................    0
PF01030      VSLNITSLGLRSLKEISDGDVIISGNKNLCYANTINWKKL......................................................................  231
PF00757      ..............................................................................................................  154
PF14843.2    ................................................................VCHALCSPEGCWGPEPRDCVSCRNVSRGRECVDKCNLLEGEPREFV   46
PF07714      ..............................................................................................................    0
PF14843.1    ................................................................VCHALCSPEGCWGPEPRDCVSCRNVSRGRECVDKCNLLEGEPREFV   46
PF14843      ................................................................VCHALCSPEGCWGPEPRDCVSCRNVSRGRECVDKCNLLEGEPREFV   46
PF01030.2    ..............................................................................................................  111
PF01030.1    VSLNITSLGLRSLKEISDGDVIISGNKNLCYANTINWKKL......................................................................  205
PF00757.1    ..............................................................................................................  153
EGFR-206     VSLNITSLGLRSLKEISDGDVIISGNKNLCYANTINWKKLFGTSGQKTKIISNRGENSCKATGQVCHALCSPEGCWGPEPRDCVSCRNVSRGRECVDKCNLLEGEPREFV  505
EGFR-203     VSLNITSLGLRSLKEISDGDVIISGNKNLCYANTINWKKLFGTSGQKTKIISNRGENSCKATGQVCHALCSPEGCWGPEPRDCVSCRNVSRGRECVDKCNLLEGEPREFV  550
EGFR-207     VSLNITSLGLRSLKEISDGDVIISGNKNLCYANTINWKKLFGTSGQKTKIISNRGENSCKATGQVCHALCSPEGCWGPEPRDCVSCRNVSRGRECVDKCNLLEGEPREFV  505
EGFR-205     ..............................................................................................................  128
EGFR-202     VSLNITSLGLRSLKEISDGDVIISGNKNLCYANTINWKKLFGTSGQKTKIISNRGENSCKATGQVCHALCSPEGCWGPEPRDCVSCRNVSRGRECVDKCNLLEGEPREFV  550
EGFR-204     ..............................................................................................................  405
EGFR-201     VSLNITSLGLRSLKEISDGDVIISGNKNLCYANTINWKKLFGTSGQKTKIISNRGENSCKATGQVCHALCSPEGCWGPEPRDCVSCRNVSRGRECVDKCNLLEGEPREFV  550

logo
             ENSECIQCHPECLPQAMNITCTGRGPDNCIQCAHYIDGPHCVKTCPAGVMGENNTLVWKYADAGHVCHLCHPNCTYGCTGPGLEGCPTNGPKI
LAPATINIB    ..............................................................................................................    0
PF01030      ..............................................................................................................  231
PF00757      ..............................................................................................................  154
PF14843.2    ENSECIQCHPECLPQAMNITCTGRGPDNCIQCAHYIDGPHCVKTCPAGVMGENNTLVWKYADAGHVCHLCHPNCTYGPG...............................  125
PF07714      ..............................................................................................................    0
PF14843.1    ENSECIQCHPECLPQAMNITCTGRGPDNCIQCAHYIDGPHCVKTCPAGVMGENNTLVWKYADAGHVCHLCHPNCTYG.................................  123
PF14843      ENSECIQCHPECLPQAMNITCTGRGPDNCIQCAHYIDGPHCVKTCPAGVMGENNTLVWKYADAGHVCHLCHPNCTYGCTGPGLEGC........................  132
PF01030.2    ..............................................................................................................  111
PF01030.1    ..............................................................................................................  205
PF00757.1    ..............................................................................................................  153
EGFR-206     ENSECIQCHPECLPQAMNITCTGRGPDNCIQCAHYIDGPHCVKTCPAGVMGENNTLVWKYADAGHVCHLCHPNCTYGCTGPGLEGCPTNGPKI.................  598
EGFR-203     ENSECIQCHPECLPQAMNITCTGRGPDNCIQCAHYIDGPHCVKTCPAGVMGENNTLVWKYADAGHVCHLCHPNCTYGPGNESLKAMLFCLFKLSSCNQSNDGSVSHQSGS  660
EGFR-207     ENSECIQCHPECLPQAMNITCTGRGPDNCIQCAHYIDGPHCVKTCPAGVMGENNTLVWKYADAGHVCHLCHPNCTYGCTGPGLEGCPTNGPKI.................  598
EGFR-205     ..............................................................................................................  128
EGFR-202     ENSECIQCHPECLPQAMNITCTGRGPDNCIQCAHYIDGPHCVKTCPAGVMGENNTLVWKYADAGHVCHLCHPNCTYGS................................  628
EGFR-204     ..............................................................................................................  405
EGFR-201     ENSECIQCHPECLPQAMNITCTGRGPDNCIQCAHYIDGPHCVKTCPAGVMGENNTLVWKYADAGHVCHLCHPNCTYGCTGPGLEGCPTNGPKI.................  643
```

```
logo
             PSIATGMVGALLLLLVVALGIGLFMRRRHIVRKRTLRRLLQERELVEPLTPSGEAPNQALLRILKETEFKKIKVLGSGAFGTVYKGLWIPEGEKVKIPVAIKELREATSP
LAPATINIB    ..........................................................................L........................A.K........   3
PF01030      .............................................................................................................. 231
PF00757      .............................................................................................................. 154
PF14843.2    .............................................................................................................. 125
PF07714      ......................................................................KIKVLGSGAFGTVYKGLWIPEGEKVKIPVAIKELREATSP  40
PF14843.1    .............................................................................................................. 123
PF14843      .............................................................................................................. 132
PF01030.2    .............................................................................................................. 111
PF01030.1    .............................................................................................................. 205
PF00757.1    .............................................................................................................. 153
EGFR-206     PSIATGMVGALLLLLVVALGIGLFMRRRHIVRKRTLRRLLQERELVEPLTPSGEAPNQALLRILKETEFKKIKVLGSGAFGTVYKGLWIPEGEKVKIPVAIKELREATSP 708
EGFR-203     PAAQESCLGWIPSLLPSEFQLG......................................................WGG..CSHLHAWPSASVIITASS........... 703
EGFR-207     PSIATGMVGALLLLLVVALGIGLFMRRRHIVRKRTLRRLLQERELVEPLTPSGEAPNQALLRILKETEFKKIKVLGSGAFGTVYKGLWIPEGEKVKIPVAIKELREATSP 708
EGFR-205     .............................................................................................................. 128
EGFR-202     .............................................................................................................. 628
EGFR-204     .............................................................................................................. 405
EGFR-201     PSIATGMVGALLLLLVVALGIGLFMRRRHIVRKRTLRRLLQERELVEPLTPSGEAPNQALLRILKETEFKKIKVLGSGAFGTVYKGLWIPEGEKVKIPVAIKELREATSP 753

logo
             KANKEILDEAYVMASVDNPHVCRLLGICLTSTVQLITQLMPFGCLLDYVREHKDNIGSQYLLNWCVQIAKGMNYLEDRRLVHRDLAARNVLVKTPQHVKITDFGLAKLLG
LAPATINIB    ............M........CRL..........L.TQLM..GC.LD...........................................L.........TDF.L.....  21
PF01030      .............................................................................................................. 231
PF00757      .............................................................................................................. 154
PF14843.2    .............................................................................................................. 125
PF07714      KANKEILDEAYVMASVDNPHVCRLLGICLTSTVQLITQLMPFGCLLDYVREHKDNIGSQYLLNWCVQIAKGMNYLEDRRLVHRDLAARNVLVKTPQHVKITDFGLAKLLG 150
PF14843.1    .............................................................................................................. 123
PF14843      .............................................................................................................. 132
PF01030.2    .............................................................................................................. 111
PF01030.1    .............................................................................................................. 205
PF00757.1    .............................................................................................................. 153
EGFR-206     KANKEILDEAYVMASVDNPHVCRLLGICLTSTVQLITQLMPFGCLLDYVREHKDNIGSQYLLNWCVQIAKGMNYLEDRRLVHRDLAARNVLVKTPQHVKITDFGLAKLLG 818
EGFR-203     .....................CH....................................................................................... 705
EGFR-207     KANKEILDEAYVMASVDNPHVCRLLGICLTSTVQLITQLMPFGCLLDYVREHKDNIGSQYLLNWCVQIAKGMNYLEDRRLVHRDLAARNVLVKTPQHVKITDFGLAKLLG 818
EGFR-205     .............................................................................................................. 128
EGFR-202     .............................................................................................................. 628
EGFR-204     .............................................................................................................. 405
EGFR-201     KANKEILDEAYVMASVDNPHVCRLLGICLTSTVQLITQLMPFGCLLDYVREHKDNIGSQYLLNWCVQIAKGMNYLEDRRLVHRDLAARNVLVKTPQHVKITDFGLAKLLG 863
```

```
logo
           AEEKEYHAEGGKVPIKWMALESILHRIYTHQSDVWSYGVTVWELMTFGSKPYDGIPASEISSILEKGERLPQPPICTIDVYMIMVKCWMIDADSRPKFRELIIEFSKMAR
LAPATINIB  ..............................................................................................................   21
PF01030    ..............................................................................................................  231
PF00757    ..............................................................................................................  154
PF14843.2  ..............................................................................................................  125
PF07714    AEEKEYHAEGGKVPIKWMALESILHRIYTHQSDVWSYGVTVWELMTFGSKPYDGIPASEISSILEKGERLPQPPICTIDVYMIMVKCWMIDADSRPKFRELI.........  252
PF14843.1  ..............................................................................................................  123
PF14843    ..............................................................................................................  132
PF01030.2  ..............................................................................................................  111
PF01030.1  ..............................................................................................................  205
PF00757.1  ..............................................................................................................  153
EGFR-206   AEEKEYHAEGGKVPIKWMALESILHRIYTHQSDVWSYGVTVWELMTFGSKPYDGIPASEISSILEKGERLPQPPICTIDVYMIMVKCWMIDADSRPKFRELIIEFSKMAR  928
EGFR-203   ..............................................................................................................  705
EGFR-207   AEEKEYHAEGGKVPIKWMALESILHRIYTHQSDVWSYGVTVWELMTFGSKPYDGIPASEISSILEKGERLPQPPICTIDVYMIMVKCWMIDADSRPKFRELIIEFSKMAR  928
EGFR-205   ..............................................................................................................  128
EGFR-202   ..............................................................................................................  628
EGFR-204   ..............................................................................................................  405
EGFR-201   AEEKEYHAEGGKVPIKWMALESILHRIYTHQSDVWSYGVTVWELMTFGSKPYDGIPASEISSILEKGERLPQPPICTIDVYMIMVKCWMIDADSRPKFRELIIEFSKMAR  973

logo
           DPQRYLVIQGDERMHLPSPTDSNFYRALMDEEDMDDVVDADEYLIPQQGFFSSPSTSRTPLLSSLSATSNNSTVACIDRNGLQSCPIKEDSFLQRYSSDPTGALTEDSID
LAPATINIB  ..............................................................................................................   21
PF01030    ..............................................................................................................  231
PF00757    ..............................................................................................................  154
PF14843.2  ..............................................................................................................  125
PF07714    ..............................................................................................................  252
PF14843.1  ..............................................................................................................  123
PF14843    ..............................................................................................................  132
PF01030.2  ..............................................................................................................  111
PF01030.1  ..............................................................................................................  205
PF00757.1  ..............................................................................................................  153
EGFR-206   DPQRYLVIQGDERMHLPSPTDSNFYRALMDEEDMDDVVDADEYLIPQQGFFSSPSTSRTPLLSSLSATSNNSTVACIDRNGLQSCPIKEDSFLQRYSSDPTGALTEDSID 1038
EGFR-203   ..............................................................................................................  705
EGFR-207   DPQRYLVIQGDERMHLPSPTDSNFYRALMDEEDMDDVVDADEYLIPQQGFFSSPSTSRTPLLSSLSATSNNSTVACIDRNGLQSCPIKEDSFLQRYSSDPTGALTEDSID 1038
EGFR-205   ..............................................................................................................  128
EGFR-202   ..............................................................................................................  628
EGFR-204   ..............................................................................................................  405
EGFR-201   DPQRYLVIQGDERMHLPSPTDSNFYRALMDEEDMDDVVDADEYLIPQQGFFSSPSTSRTPLLSSLSATSNNSTVACIDRNGLQSCPIKEDSFLQRYSSDPTGALTEDSID 1083
```

```
logo

            DTFLPVP[illegible]KRPAGSVQNPV[illegible]HSTAVGNPEYLNTVQPTCVNSTFDSPAHWAQKGSHQISLDNPDYQQDFFPKEAKPNG
LAPATINIB   ..............................................................................................................    21
PF01030     ..............................................................................................................   231
PF00757     ..............................................................................................................   154
PF14843.2   ..............................................................................................................   125
PF07714     ..............................................................................................................   252
PF14843.1   ..............................................................................................................   123
PF14843     ..............................................................................................................   132
PF01030.2   ..............................................................................................................   111
PF01030.1   ..............................................................................................................   205
PF00757.1   ..............................................................................................................   153
EGFR-206    DTFLPVPEYINQSVP........KRPAGSVQNPVYHNQPLNPAPSRDPHYQDPHSTAVGNPEYLNTVQPTCVNSTFDSPAHWAQKGSHQISLDNPDYQQDFFPKEAKPNG  1140
EGFR-203    ..............................................................................................................   705
EGFR-207    DTFLPVPGEWLVWKQSCSSTSSTHSAAASLQCPSQVLPPASPEGETVADLQTQ.........................................................  1091
EGFR-205    ..............................................................................................................   128
EGFR-202    ..............................................................................................................   628
EGFR-204    ..............................................................................................................   405
EGFR-201    DTFLPVPEYINQSVP........KRPAGSVQNPVYHNQPLNPAPSRDPHYQDPHSTAVGNPEYLNTVQPTCVNSTFDSPAHWAQKGSHQISLDNPDYQQDFFPKEAKPNG  1185


logo

            IFKGSTAENAEYLRVAPQSSEFIGA
LAPATINIB   .........................    21
PF01030     .........................   231
PF00757     .........................   154
PF14843.2   .........................   125
PF07714     .........................   252
PF14843.1   .........................   123
PF14843     .........................   132
PF01030.2   .........................   111
PF01030.1   .........................   205
PF00757.1   .........................   153
EGFR-206    IFKGSTAENAEYLRVAPQSSEFIGA  1165
EGFR-203    .........................   705
EGFR-207    .........................  1091
EGFR-205    .........................   128
EGFR-202    .........................   628
EGFR-204    .........................   405
EGFR-201    IFKGSTAENAEYLRVAPQSSEFIGA  1210
```

X non conserved
X similar
X ≥ 0% conserved
X ≥ 50% conserved

```
logo         MRPSGTAGAALLALLAALCPASRALEEKKVCQGTSNKLTQLGTFEDHFLSLQRMFNNCEVVLGNLEITYVQRNYDLSFLKTIQEVAGYVLIALNTVERIPLENLQIIRGN
OSIMERTINIB  ..............................................................................................................     0
PF01030      ........................................................NCEVVLGNLEITYVQRNYDLSFLKTIQEVAGYVLIALNTVERIPLENLQIIRGN    54
PF00757      ..............................................................................................................     0
PF14843.2    ..............................................................................................................     0
PF07714      ..............................................................................................................     0
PF14843.1    ..............................................................................................................     0
PF14843      ..............................................................................................................     0
PF01030.2    ........................................................NCEVVLGNLEITYVQRNYDLSFLKTIQEVAGYVLIALNTVERIPLENLQIIRGN    54
PF01030.1    ........................................................NCEVVLGNLEITYVQRNYDLSFLKTIQEVAGYVLIALNTVERIPLENLQIIRGN    54
PF00757.1    ..............................................................................................................     0
EGFR-206     MRPSGTAGAALLALLAALCPASRALEEKKVCQGTSNKLTQLGTFEDHFLSLQRMFNNCEVVLGNLEITYVQRNYDLSFLKTIQEVAGYVLIALNTVERIPLENLQIIRGN   110
EGFR-203     MRPSGTAGAALLALLAALCPASRALEEKKVCQGTSNKLTQLGTFEDHFLSLQRMFNNCEVVLGNLEITYVQRNYDLSFLKTIQEVAGYVLIALNTVERIPLENLQIIRGN   110
EGFR-207     MRPSGTAGAALLALLAALCPASRALEEKKVCQGTSNKLTQLGTFEDHFLSLQRMFNNCEVVLGNLEITYVQRNYDLSFLKTIQEVAGYVLIALNTVERIPLENLQIIRGN   110
EGFR-205     .....................................................MFNNCEVVLGNLEITYVQRNYDLSFLKTIQEVAGYVLIALNTVERIPLENLQIIRGN    57
EGFR-202     MRPSGTAGAALLALLAALCPASRALEEKKVCQGTSNKLTQLGTFEDHFLSLQRMFNNCEVVLGNLEITYVQRNYDLSFLKTIQEVAGYVLIALNTVERIPLENLQIIRGN   110
EGFR-204     MRPSGTAGAALLALLAALCPASRALEEKKVCQGTSNKLTQLGTFEDHFLSLQRMFNNCEVVLGNLEITYVQRNYDLSFLKTIQEVAGYVLIALNTVERIPLENLQIIRGN   110
EGFR-201     MRPSGTAGAALLALLAALCPASRALEEKKVCQGTSNKLTQLGTFEDHFLSLQRMFNNCEVVLGNLEITYVQRNYDLSFLKTIQEVAGYVLIALNTVERIPLENLQIIRGN   110

logo         MYYENSYALAVLSNYDANKTGLKELPMRNLQEILHGAVRFSNNPALCNVESIQWRDIVSSDFLSNMSMDFQNHLGSCQKCDPSCPNGSCWGAGEENCQKLTKIICAQQCS
OSIMERTINIB  ..............................................................................................................     0
PF01030      MYYENSYALAVLSNYDANKTGLKELPMRNLQEILHGAVRFSNNPALCNVESIQWRDI.....................................................   111
PF00757      ..........................................................................GSCQKCDPSCPNGSCWGAGEENCQKLTKIICAQQCS    36
PF14843.2    ..............................................................................................................     0
PF07714      ..............................................................................................................     0
PF14843.1    ..............................................................................................................     0
PF14843      ..............................................................................................................     0
PF01030.2    MYYENSYALAVLSNYDANKTGLKELPMRNLQEILHGAVRFSNNPALCNVESIQWRDI.....................................................   111
PF01030.1    MYYENSYALAVLSNYDANKTGLKELPMRNLQ...............................................................................    85
PF00757.1    ...............................QG............................................QKCDPSCPNGSCWGAGEENCQKLTKIICAQQCS    35
EGFR-206     MYYENSYALAVLSNYDANKTGLKELPMRNLQG.............................................QKCDPSCPNGSCWGAGEENCQKLTKIICAQQCS   175
EGFR-203     MYYENSYALAVLSNYDANKTGLKELPMRNLQEILHGAVRFSNNPALCNVESIQWRDIVSSDFLSNMSMDFQNHLGSCQKCDPSCPNGSCWGAGEENCQKLTKIICAQQCS   220
EGFR-207     MYYENSYALAVLSNYDANKTGLKELPMRNLQG.............................................QKCDPSCPNGSCWGAGEENCQKLTKIICAQQCS   175
EGFR-205     MYYENSYALAVLSNYDANKTGLKELPMRNLQEILHGAVRFSNNPALCNVESIQWRDIVSSDFLSNMSMDFQ.......................................   128
EGFR-202     MYYENSYALAVLSNYDANKTGLKELPMRNLQEILHGAVRFSNNPALCNVESIQWRDIVSSDFLSNMSMDFQNHLGSCQKCDPSCPNGSCWGAGEENCQKLTKIICAQQCS   220
EGFR-204     MYYENSYALAVLSNYDANKTGLKELPMRNLQEILHGAVRFSNNPALCNVESIQWRDIVSSDFLSNMSMDFQNHLGSCQKCDPSCPNGSCWGAGEENCQKLTKIICAQQCS   220
EGFR-201     MYYENSYALAVLSNYDANKTGLKELPMRNLQEILHGAVRFSNNPALCNVESIQWRDIVSSDFLSNMSMDFQNHLGSCQKCDPSCPNGSCWGAGEENCQKLTKIICAQQCS   220
```

```
logo         GRCRGKSPSDCCHNQCAAGCTGPRESDCLVCRKFRDEATCKDTCPPLMLYNPTTYQMDVNPEGKYSFGATCVKKCPRNYVVTDHGSCVRACGADSYEMEEDGVRKCKKCE
OSIMERTINIB  ..............................................................................................................    0
PF01030      ..............................................................................................................  111
PF00757      GRCRGKSPSDCCHNQCAAGCTGPRESDCLVCRKFRDEATCKDTCPPLMLYNPTTYQMDVNPEGKYSFGATCVKKCPRNYVVTDHGSCVRACGADSYEMEEDGVRKCKKCE  146
PF14843.2    ..............................................................................................................    0
PF07714      ..............................................................................................................    0
PF14843.1    ..............................................................................................................    0
PF14843      ..............................................................................................................    0
PF01030.2    ..............................................................................................................  111
PF01030.1    ..............................................................................................................   85
PF00757.1    GRCRGKSPSDCCHNQCAAGCTGPRESDCLVCRKFRDEATCKDTCPPLMLYNPTTYQMDVNPEGKYSFGATCVKKCPRNYVVTDHGSCVRACGADSYEMEEDGVRKCKKCE  145
EGFR-206     GRCRGKSPSDCCHNQCAAGCTGPRESDCLVCRKFRDEATCKDTCPPLMLYNPTTYQMDVNPEGKYSFGATCVKKCPRNYVVTDHGSCVRACGADSYEMEEDGVRKCKKCE  285
EGFR-203     GRCRGKSPSDCCHNQCAAGCTGPRESDCLVCRKFRDEATCKDTCPPLMLYNPTTYQMDVNPEGKYSFGATCVKKCPRNYVVTDHGSCVRACGADSYEMEEDGVRKCKKCE  330
EGFR-207     GRCRGKSPSDCCHNQCAAGCTGPRESDCLVCRKFRDEATCKDTCPPLMLYNPTTYQMDVNPEGKYSFGATCVKKCPRNYVVTDHGSCVRACGADSYEMEEDGVRKCKKCE  285
EGFR-205     ..............................................................................................................  128
EGFR-202     GRCRGKSPSDCCHNQCAAGCTGPRESDCLVCRKFRDEATCKDTCPPLMLYNPTTYQMDVNPEGKYSFGATCVKKCPRNYVVTDHGSCVRACGADSYEMEEDGVRKCKKCE  330
EGFR-204     GRCRGKSPSDCCHNQCAAGCTGPRESDCLVCRKFRDEATCKDTCPPLMLYNPTTYQMDVNPEGKYSFGATCVKKCPRNYVVTDHGSCVRACGADSYEMEEDGVRKCKKCE  330
EGFR-201     GRCRGKSPSDCCHNQCAAGCTGPRESDCLVCRKFRDEATCKDTCPPLMLYNPTTYQMDVNPEGKYSFGATCVKKCPRNYVVTDHGSCVRACGADSYEMEEDGVRKCKKCE  330

logo         GPCRKVCNGIGIGEFKDSLSINATNIKHFKNCTSISGDLHILPVAFRGDSFTHTPPLDPQELDILKTVKEITGFLLIQAWPENRTDLHAFENLEIIRGRTKQHGQFSLAV
OSIMERTINIB  ..............................................................................................................    0
PF01030      ..............................NCTSISGDLHILPVAFRGDSFTHTPPLDPQELDILKTVKEITGFLLIQAWPENRTDLHAFENLEIIRGRTKQHGQFSLAV  191
PF00757      GPCRKVCN......................................................................................................  154
PF14843.2    ..............................................................................................................    0
PF07714      ..............................................................................................................    0
PF14843.1    ..............................................................................................................    0
PF14843      ..............................................................................................................    0
PF01030.2    ..............................................................................................................  111
PF01030.1    ..............................NCTSISGDLHILPVAFRGDSFTHTPPLDPQELDILKTVKEITGFLLIQAWPENRTDLHAFENLEIIRGRTKQHGQFSLAV  165
PF00757.1    GPCRKVCN......................................................................................................  153
EGFR-206     GPCRKVCNGIGIGEFKDSLSINATNIKHFKNCTSISGDLHILPVAFRGDSFTHTPPLDPQELDILKTVKEITGFLLIQAWPENRTDLHAFENLEIIRGRTKQHGQFSLAV  395
EGFR-203     GPCRKVCNGIGIGEFKDSLSINATNIKHFKNCTSISGDLHILPVAFRGDSFTHTPPLDPQELDILKTVKEITGFLLIQAWPENRTDLHAFENLEIIRGRTKQHGQFSLAV  440
EGFR-207     GPCRKVCNGIGIGEFKDSLSINATNIKHFKNCTSISGDLHILPVAFRGDSFTHTPPLDPQELDILKTVKEITGFLLIQAWPENRTDLHAFENLEIIRGRTKQHGQFSLAV  395
EGFR-205     ..............................................................................................................  128
EGFR-202     GPCRKVCNGIGIGEFKDSLSINATNIKHFKNCTSISGDLHILPVAFRGDSFTHTPPLDPQELDILKTVKEITGFLLIQAWPENRTDLHAFENLEIIRGRTKQHGQFSLAV  440
EGFR-204     GPCRKVCNGIGIGEFKDSLSINATNIKHFKNCTSISGDLHILPVAFRGDSFTHTPPLDPQELDILKTVKEITGLS...................................  405
EGFR-201     GPCRKVCNGIGIGEFKDSLSINATNIKHFKNCTSISGDLHILPVAFRGDSFTHTPPLDPQELDILKTVKEITGFLLIQAWPENRTDLHAFENLEIIRGRTKQHGQFSLAV  440
```

```
logo
```
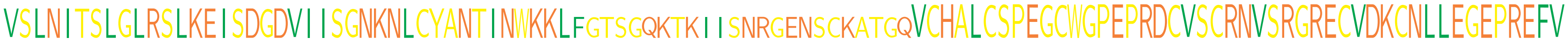

```
OSIMERTINIB  ..............................................................................................................   0
PF01030      VSLNITSLGLRSLKEISDGDVIISGNKNLCYANTINWKKL......................................................................  231
PF00757      ..............................................................................................................  154
PF14843.2    ................................................................VCHALCSPEGCWGPEPRDCVSCRNVSRGRECVDKCNLLEGEPREFV   46
PF07714      ..............................................................................................................    0
PF14843.1    ................................................................VCHALCSPEGCWGPEPRDCVSCRNVSRGRECVDKCNLLEGEPREFV   46
PF14843      ................................................................VCHALCSPEGCWGPEPRDCVSCRNVSRGRECVDKCNLLEGEPREFV   46
PF01030.2    ..............................................................................................................  111
PF01030.1    VSLNITSLGLRSLKEISDGDVIISGNKNLCYANTINWKKL......................................................................  205
PF00757.1    ..............................................................................................................  153
EGFR-206     VSLNITSLGLRSLKEISDGDVIISGNKNLCYANTINWKKLFGTSGQKTKIISNRGENSCKATGQVCHALCSPEGCWGPEPRDCVSCRNVSRGRECVDKCNLLEGEPREFV  505
EGFR-203     VSLNITSLGLRSLKEISDGDVIISGNKNLCYANTINWKKLFGTSGQKTKIISNRGENSCKATGQVCHALCSPEGCWGPEPRDCVSCRNVSRGRECVDKCNLLEGEPREFV  550
EGFR-207     VSLNITSLGLRSLKEISDGDVIISGNKNLCYANTINWKKLFGTSGQKTKIISNRGENSCKATGQVCHALCSPEGCWGPEPRDCVSCRNVSRGRECVDKCNLLEGEPREFV  505
EGFR-205     ..............................................................................................................  128
EGFR-202     VSLNITSLGLRSLKEISDGDVIISGNKNLCYANTINWKKLFGTSGQKTKIISNRGENSCKATGQVCHALCSPEGCWGPEPRDCVSCRNVSRGRECVDKCNLLEGEPREFV  550
EGFR-204     ..............................................................................................................  405
EGFR-201     VSLNITSLGLRSLKEISDGDVIISGNKNLCYANTINWKKLFGTSGQKTKIISNRGENSCKATGQVCHALCSPEGCWGPEPRDCVSCRNVSRGRECVDKCNLLEGEPREFV  550
```

```
logo
```
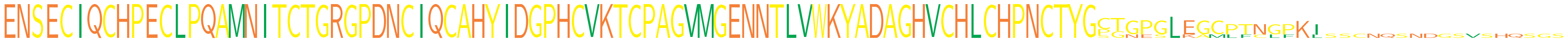

```
OSIMERTINIB  ..............................................................................................................   0
PF01030      ..............................................................................................................  231
PF00757      ..............................................................................................................  154
PF14843.2    ENSECIQCHPECLPQAMNITCTGRGPDNCIQCAHYIDGPHCVKTCPAGVMGENNTLVWKYADAGHVCHLCHPNCTYGPG...............................  125
PF07714      ..............................................................................................................    0
PF14843.1    ENSECIQCHPECLPQAMNITCTGRGPDNCIQCAHYIDGPHCVKTCPAGVMGENNTLVWKYADAGHVCHLCHPNCTYG.................................  123
PF14843      ENSECIQCHPECLPQAMNITCTGRGPDNCIQCAHYIDGPHCVKTCPAGVMGENNTLVWKYADAGHVCHLCHPNCTYGCTGPGLEGC.........................  132
PF01030.2    ..............................................................................................................  111
PF01030.1    ..............................................................................................................  205
PF00757.1    ..............................................................................................................  153
EGFR-206     ENSECIQCHPECLPQAMNITCTGRGPDNCIQCAHYIDGPHCVKTCPAGVMGENNTLVWKYADAGHVCHLCHPNCTYGCTGPGLEGCPTNGPKI..................  598
EGFR-203     ENSECIQCHPECLPQAMNITCTGRGPDNCIQCAHYIDGPHCVKTCPAGVMGENNTLVWKYADAGHVCHLCHPNCTYGPGNESLKAMLFCLFKLSSCNQSNDGSVSHQSGS  660
EGFR-207     ENSECIQCHPECLPQAMNITCTGRGPDNCIQCAHYIDGPHCVKTCPAGVMGENNTLVWKYADAGHVCHLCHPNCTYGCTGPGLEGCPTNGPKI..................  598
EGFR-205     ..............................................................................................................  128
EGFR-202     ENSECIQCHPECLPQAMNITCTGRGPDNCIQCAHYIDGPHCVKTCPAGVMGENNTLVWKYADAGHVCHLCHPNCTYGS................................  628
EGFR-204     ..............................................................................................................  405
EGFR-201     ENSECIQCHPECLPQAMNITCTGRGPDNCIQCAHYIDGPHCVKTCPAGVMGENNTLVWKYADAGHVCHLCHPNCTYGCTGPGLEGCPTNGPKI..................  643
```

```
logo
             PSIATGMVGALLLLLVVALGIGLFMRRRHIVRKRTLRRLLQERELVEPLTPSGEAPNQALLRILKETEFKKIKVLGSGAFGTVYKGLWIPEGEKVKIPVAIKELREATSP
OSIMERTINIB  ..........................................................................LG......V................A..........    4
PF01030      ..............................................................................................................  231
PF00757      ..............................................................................................................  154
PF14843.2    ..............................................................................................................  125
PF07714      ......................................................................KIKVLGSGAFGTVYKGLWIPEGEKVKIPVAIKELREATSP   40
PF14843.1    ..............................................................................................................  123
PF14843      ..............................................................................................................  132
PF01030.2    ..............................................................................................................  111
PF01030.1    ..............................................................................................................  205
PF00757.1    ..............................................................................................................  153
EGFR-206     PSIATGMVGALLLLLVVALGIGLFMRRRHIVRKRTLRRLLQERELVEPLTPSGEAPNQALLRILKETEFKKIKVLGSGAFGTVYKGLWIPEGEKVKIPVAIKELREATSP  708
EGFR-203     PAAQESCLGWIPSLLPSEFQLG......................................................WGG..CSHLHAWPSASVIITASS...........  703
EGFR-207     PSIATGMVGALLLLLVVALGIGLFMRRRHIVRKRTLRRLLQERELVEPLTPSGEAPNQALLRILKETEFKKIKVLGSGAFGTVYKGLWIPEGEKVKIPVAIKELREATSP  708
EGFR-205     ..............................................................................................................  128
EGFR-202     ..............................................................................................................  628
EGFR-204     ..............................................................................................................  405
EGFR-201     PSIATGMVGALLLLLVVALGIGLFMRRRHIVRKRTLRRLLQERELVEPLTPSGEAPNQALLRILKETEFKKIKVLGSGAFGTVYKGLWIPEGEKVKIPVAIKELREATSP  753

logo
             KANKEILDEAYVMASVDNPHVCRLLGICLTSTVQLITQLMPFGCLLDYVREHKDNIGSQYLLNWCVQIAKGMNYLEDRRLVHRDLAARNVLVKTPQHVKITDFGLAKLLG
OSIMERTINIB  .......................................MP.GC..................................................................    8
PF01030      ..............................................................................................................  231
PF00757      ..............................................................................................................  154
PF14843.2    ..............................................................................................................  125
PF07714      KANKEILDEAYVMASVDNPHVCRLLGICLTSTVQLITQLMPFGCLLDYVREHKDNIGSQYLLNWCVQIAKGMNYLEDRRLVHRDLAARNVLVKTPQHVKITDFGLAKLLG  150
PF14843.1    ..............................................................................................................  123
PF14843      ..............................................................................................................  132
PF01030.2    ..............................................................................................................  111
PF01030.1    ..............................................................................................................  205
PF00757.1    ..............................................................................................................  153
EGFR-206     KANKEILDEAYVMASVDNPHVCRLLGICLTSTVQLITQLMPFGCLLDYVREHKDNIGSQYLLNWCVQIAKGMNYLEDRRLVHRDLAARNVLVKTPQHVKITDFGLAKLLG  818
EGFR-203     .....................CH.......................................................................................  705
EGFR-207     KANKEILDEAYVMASVDNPHVCRLLGICLTSTVQLITQLMPFGCLLDYVREHKDNIGSQYLLNWCVQIAKGMNYLEDRRLVHRDLAARNVLVKTPQHVKITDFGLAKLLG  818
EGFR-205     ..............................................................................................................  128
EGFR-202     ..............................................................................................................  628
EGFR-204     ..............................................................................................................  405
EGFR-201     KANKEILDEAYVMASVDNPHVCRLLGICLTSTVQLITQLMPFGCLLDYVREHKDNIGSQYLLNWCVQIAKGMNYLEDRRLVHRDLAARNVLVKTPQHVKITDFGLAKLLG  863
```

```
logo
               AEEKEYHAEGGKVPIKWMALESILHRIYTHQSDVWSYGVTVWELMTFGSKPYDGIPASEISSILEKGERLPQPPICTIDVYMIMVKCWMIDADSRPKFRELIIEFSKMAR
OSIMERTINIB    ..............................................................................................................    8
PF01030        ..............................................................................................................  231
PF00757        ..............................................................................................................  154
PF14843.2      ..............................................................................................................  125
PF07714        AEEKEYHAEGGKVPIKWMALESILHRIYTHQSDVWSYGVTVWELMTFGSKPYDGIPASEISSILEKGERLPQPPICTIDVYMIMVKCWMIDADSRPKFRELI.........  252
PF14843.1      ..............................................................................................................  123
PF14843        ..............................................................................................................  132
PF01030.2      ..............................................................................................................  111
PF01030.1      ..............................................................................................................  205
PF00757.1      ..............................................................................................................  153
EGFR-206       AEEKEYHAEGGKVPIKWMALESILHRIYTHQSDVWSYGVTVWELMTFGSKPYDGIPASEISSILEKGERLPQPPICTIDVYMIMVKCWMIDADSRPKFRELIIEFSKMAR  928
EGFR-203       ..............................................................................................................  705
EGFR-207       AEEKEYHAEGGKVPIKWMALESILHRIYTHQSDVWSYGVTVWELMTFGSKPYDGIPASEISSILEKGERLPQPPICTIDVYMIMVKCWMIDADSRPKFRELIIEFSKMAR  928
EGFR-205       ..............................................................................................................  128
EGFR-202       ..............................................................................................................  628
EGFR-204       ..............................................................................................................  405
EGFR-201       AEEKEYHAEGGKVPIKWMALESILHRIYTHQSDVWSYGVTVWELMTFGSKPYDGIPASEISSILEKGERLPQPPICTIDVYMIMVKCWMIDADSRPKFRELIIEFSKMAR  973

logo
               DPQRYLVIQGDERMHLPSPTDSNFYRALMDEEDMDDVVDADEYLIPQQGFFSSPSTSRTPLLSSLSATSNNSTVACIDRNGLQSCPIKEDSFLQRYSSDPTGALTEDSID
OSIMERTINIB    ..............................................................................................................    8
PF01030        ..............................................................................................................  231
PF00757        ..............................................................................................................  154
PF14843.2      ..............................................................................................................  125
PF07714        ..............................................................................................................  252
PF14843.1      ..............................................................................................................  123
PF14843        ..............................................................................................................  132
PF01030.2      ..............................................................................................................  111
PF01030.1      ..............................................................................................................  205
PF00757.1      ..............................................................................................................  153
EGFR-206       DPQRYLVIQGDERMHLPSPTDSNFYRALMDEEDMDDVVDADEYLIPQQGFFSSPSTSRTPLLSSLSATSNNSTVACIDRNGLQSCPIKEDSFLQRYSSDPTGALTEDSID 1038
EGFR-203       ..............................................................................................................  705
EGFR-207       DPQRYLVIQGDERMHLPSPTDSNFYRALMDEEDMDDVVDADEYLIPQQGFFSSPSTSRTPLLSSLSATSNNSTVACIDRNGLQSCPIKEDSFLQRYSSDPTGALTEDSID 1038
EGFR-205       ..............................................................................................................  128
EGFR-202       ..............................................................................................................  628
EGFR-204       ..............................................................................................................  405
EGFR-201       DPQRYLVIQGDERMHLPSPTDSNFYRALMDEEDMDDVVDADEYLIPQQGFFSSPSTSRTPLLSSLSATSNNSTVACIDRNGLQSCPIKEDSFLQRYSSDPTGALTEDSID 1083
```

```
logo
             DTFLPVPEYINQSVPSCSSTSSTKRPAGSVQNPVYHNQPLNPAPSRDPHYQDPHSTAVGNPEYLNTVQPTCVNSTFDSPAHWAQKGSHQISLDNPDYQQDFFPKEAKPNG
OSIMERTINIB  ..............................................................................................................     8
PF01030      ..............................................................................................................   231
PF00757      ..............................................................................................................   154
PF14843.2    ..............................................................................................................   125
PF07714      ..............................................................................................................   252
PF14843.1    ..............................................................................................................   123
PF14843      ..............................................................................................................   132
PF01030.2    ..............................................................................................................   111
PF01030.1    ..............................................................................................................   205
PF00757.1    ..............................................................................................................   153
EGFR-206     DTFLPVPEYINQSVP........KRPAGSVQNPVYHNQPLNPAPSRDPHYQDPHSTAVGNPEYLNTVQPTCVNSTFDSPAHWAQKGSHQISLDNPDYQQDFFPKEAKPNG  1140
EGFR-203     ..............................................................................................................   705
EGFR-207     DTFLPVPGEWLVWKQSCSSTSSTHSAAASLQCPSQVLPPASPEGETVADLQTQ.........................................................  1091
EGFR-205     ..............................................................................................................   128
EGFR-202     ..............................................................................................................   628
EGFR-204     ..............................................................................................................   405
EGFR-201     DTFLPVPEYINQSVP........KRPAGSVQNPVYHNQPLNPAPSRDPHYQDPHSTAVGNPEYLNTVQPTCVNSTFDSPAHWAQKGSHQISLDNPDYQQDFFPKEAKPNG  1185
```

```
logo
             IFKGSTAENAEYLRVAPQSSEFIGA
OSIMERTINIB  .........................     8
PF01030      .........................   231
PF00757      .........................   154
PF14843.2    .........................   125
PF07714      .........................   252
PF14843.1    .........................   123
PF14843      .........................   132
PF01030.2    .........................   111
PF01030.1    .........................   205
PF00757.1    .........................   153
EGFR-206     IFKGSTAENAEYLRVAPQSSEFIGA  1165
EGFR-203     .........................   705
EGFR-207     .........................  1091
EGFR-205     .........................   128
EGFR-202     .........................   628
EGFR-204     .........................   405
EGFR-201     IFKGSTAENAEYLRVAPQSSEFIGA  1210
```

X non conserved
X similar
X $\geq$ 0% conserved
X $\geq$ 50% conserved

```
logo
           MELQAARACFALLWGCALAAAAAAQGKEVVLLDFAAAGGELGWLTHPYGKGWDLMQNIMNDMPIYMYSVCNVMSGDQDNWLRTNWVYRGEAERIFIELKFTVRDCNSFPG
DASATINIB  ..............................................................................................................    0
PF07714    ..............................................................................................................    0
PF00041    ..............................................................................................................    0
PF14575    ..............................................................................................................    0
PF01404    ............................VVLLDFAAAGGELGWLTHPYGKGWDLMQNIMNDMPIYMYSVCNVMSGDQDNWLRTNWVYRGEAERIFIELKFTVRDCNSFPG   82
PF00536    ..............................................................................................................    0
EPHA2-201  MELQAARACFALLWGCALAAAAAAQGKEVVLLDFAAAGGELGWLTHPYGKGWDLMQNIMNDMPIYMYSVCNVMSGDQDNWLRTNWVYRGEAERIFIELKFTVRDCNSFPG  110

logo
           GASSCKETFNLYYAESDLDYGTNFQKRLFTKIDTIAPDEITVSSDFEARHVKLNVEERSVGPLTRKGFYLAFQDIGACVALLSVRVYYKKCPELLQGLAHFPETIAGSDA
DASATINIB  ..............................................................................................................    0
PF07714    ..............................................................................................................    0
PF00041    ..............................................................................................................    0
PF14575    ..............................................................................................................    0
PF01404    GASSCKETFNLYYAESDLDYGTNFQKRLFTKIDTIAPDEITVSSDFEARHVKLNVEERSVGPLTRKGFYLAFQDIGACVALLSVRVYYKKC...................  173
PF00536    ..............................................................................................................    0
EPHA2-201  GASSCKETFNLYYAESDLDYGTNFQKRLFTKIDTIAPDEITVSSDFEARHVKLNVEERSVGPLTRKGFYLAFQDIGACVALLSVRVYYKKCPELLQGLAHFPETIAGSDA  220

logo
           PSLATVAGTCVDHAVVPPGGEEPRMHCAVDGEWLVPIGQCLCQAGYEKVEDACQACSPGFFKFEASESPCLECPEHTLPSPEGATSCECEEGFFRAPQDPASMPCTRPPS
DASATINIB  ..............................................................................................................    0
PF07714    ..............................................................................................................    0
PF00041    .............................................................................................................S    1
PF14575    ..............................................................................................................    0
PF01404    ..............................................................................................................  173
PF00536    ..............................................................................................................    0
EPHA2-201  PSLATVAGTCVDHAVVPPGGEEPRMHCAVDGEWLVPIGQCLCQAGYEKVEDACQACSPGFFKFEASESPCLECPEHTLPSPEGATSCECEEGFFRAPQDPASMPCTRPPS  330

logo
           APHYLTAVGMGAKVELRWTPPQDSGGREDIVYSVTCEQCWPESGECGPCEASVRYSEPPHGLTRTSVTVSDLEPHMNYTFTVEARNGVSGLVTSRSFRTASVSINQTEPP
DASATINIB  ..............................................................................................................    0
PF07714    ..............................................................................................................    0
PF00041    APHYLTAVGMGAKVELRWTPPQDSGGREDIVYSVTCEQCWPESGECGPCEASVRYSEPPHGLTRTSVTVSDLEPHMNYTFTVEARNGVS..................EPP   93
PF14575    ..............................................................................................................    0
PF01404    ..............................................................................................................  173
PF00536    ..............................................................................................................    0
EPHA2-201  APHYLTAVGMGAKVELRWTPPQDSGGREDIVYSVTCEQCWPESGECGPCEASVRYSEPPHGLTRTSVTVSDLEPHMNYTFTVEARNGVSGLVTSRSFRTASVSINQTEPP  440

logo
           KVRLEGRSTTSLSVSWSIPPPQQSRVWKYEVTYRKKGDSNSYNVRRTEGFSVTLDDLAPDTTYLVQVQALTQEGQGAGSKVHEFQTLSPEGSGNLAVIGGVAVGVVLLLV
DASATINIB  ..............................................................................................................    0
PF07714    ..............................................................................................................    0
PF00041    KVRLEGRSTTSLSVSWSIPPPQQSRVWKYEVTYRKKGDSNSYNVRRTEGFSVTLDDLAPDTTYLVQVQALTQEGQGAGS...............................  172
PF14575    ................................................................................................VIGGVAVGVVLLLV   14
PF01404    ..............................................................................................................  173
PF00536    ..............................................................................................................    0
EPHA2-201  KVRLEGRSTTSLSVSWSIPPPQQSRVWKYEVTYRKKGDSNSYNVRRTEGFSVTLDDLAPDTTYLVQVQALTQEGQGAGSKVHEFQTLSPEGSGNLAVIGGVAVGVVLLLV  550
```

```
logo
           LAGVGFFIHRRRKNQRARQSPEDVYFSKSEQLKPLKTYVDPHTYEDPNQAVLKFTTEIHPSCVTRQKVIGAGEFGEVYKGMLKTSSGKKEVPVAIKTLKAGYTEKQRVDF
DASATINIB  ....................................................................I........................AIK..............    4
PF07714    ................................................................RQKVIGAGEFGEVYKGMLKTSSGKKEVPVAIKTLKAGYTEKQRVDF   46
PF00041    ..............................................................................................................  172
PF14575    LAGVGFFIHRRRKNQRARQSPEDVYFSKSEQLKPLKTYVDPHTYEDPNQAVLKFTTEIHP..................................................   74
PF01404    ..............................................................................................................  173
PF00536    ..............................................................................................................    0
EPHA2-201  LAGVGFFIHRRRKNQRARQSPEDVYFSKSEQLKPLKTYVDPHTYEDPNQAVLKFTTEIHPSCVTRQKVIGAGEFGEVYKGMLKTSSGKKEVPVAIKTLKAGYTEKQRVDF  660

logo
           LGEAGIMGQFSHHNIIRLEGVISKYKPMMIITEYMENGALDKFLREKDGEFSVLQLVGMLRGIAAGMKYLANMNYVHRDLAARNILVNSNLVCKVSDFGLSRVLEDDPEA
DASATINIB  ..E...M........I.............I.TEYME.G...K...E.......................................L.........S..............   18
PF07714    LGEAGIMGQFSHHNIIRLEGVISKYKPMMIITEYMENGALDKFLREKDGEFSVLQLVGMLRGIAAGMKYLANMNYVHRDLAARNILVNSNLVCKVSDFGLSRVLEDDPEA  156
PF00041    ..............................................................................................................  172
PF14575    ..............................................................................................................   74
PF01404    ..............................................................................................................  173
PF00536    ..............................................................................................................    0
EPHA2-201  LGEAGIMGQFSHHNIIRLEGVISKYKPMMIITEYMENGALDKFLREKDGEFSVLQLVGMLRGIAAGMKYLANMNYVHRDLAARNILVNSNLVCKVSDFGLSRVLEDDPEA  770

logo
           TYTTSGGKIPIRWTAPEAISYRKFTSASDVWSFGIVMWEVMTYGERPYWELSNHEVMKAINDGFRLPTPMDCPSAIYQLMMQCWQQERARRPKFADIVSILDKLIRAPDS
DASATINIB  ..............................................................................................................   18
PF07714    TYTTSGGKIPIRWTAPEAISYRKFTSASDVWSFGIVMWEVMTYGERPYWELSNHEVMKAINDGFRLPTPMDCPSAIYQLMMQCWQQERARRPKFADIVSIL.........  257
PF00041    ..............................................................................................................  172
PF14575    ..............................................................................................................   74
PF01404    ..............................................................................................................  173
PF00536    ..............................................................................................................    0
EPHA2-201  TYTTSGGKIPIRWTAPEAISYRKFTSASDVWSFGIVMWEVMTYGERPYWELSNHEVMKAINDGFRLPTPMDCPSAIYQLMMQCWQQERARRPKFADIVSILDKLIRAPDS  880

logo
           LKTLADFDPRVSIRLPSTSGSEGVPFRTVSEWLESIKMQQYTEHFMAAGYTAIEKVVQMTNDDIKRIGVRLPGHQKRIAYSLLGLKDQVNTVGIPI
DASATINIB  ................................................................................................   18
PF07714    ................................................................................................  257
PF00041    ................................................................................................  172
PF14575    ................................................................................................   74
PF01404    ................................................................................................  173
PF00536    .......................VPFRTVSEWLESIKMQQYTEHFMAAGYTAIEKVVQMTNDDIKRIGVRLPGHQKRIAYSLLGLK..........   63
EPHA2-201  LKTLADFDPRVSIRLPSTSGSEGVPFRTVSEWLESIKMQQYTEHFMAAGYTAIEKVVQMTNDDIKRIGVRLPGHQKRIAYSLLGLKDQVNTVGIPI  976
```

X non conserved
X similar
X ≥ 0% conserved
X ≥ 50% conserved

logo

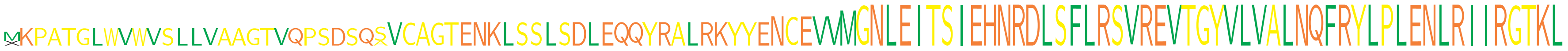

```
LAPATINIB    ..............................................................................................................    0
PF01030.1    ............................................................GNLEITSIEHNRDLSFLRSVREVTGYVLVALNQFRYLPLENLRIIRGTKL   50
PF00757      ..............................................................................................................    0
PF14843.1    ..............................................................................................................    0
PF07714      ..............................................................................................................    0
PF01030      ......................................................NCEVVMGNLEITSIEHNRDLSFLRSVREVTGYVLVALNQFRYLPLENLRIIRGTKL   56
PF14843      ..............................................................................................................    0
ERBB4-203    ..........................AVCAGTENKLSSLSDLEQQYRALRKYYENCEVVMGNLEITSIEHNRDLSFLRSVREVTGYVLVALNQFRYLPLENLRIIRGTKL   84
ERBB4-201    XKPATGLWVWVSLLVAAGTVQPSDSQSVCAGTENKLSSLSDLEQQYRALRKYYENCEVVMGNLEITSIEHNRDLSFLRSVREVTGYVLVALNQFRYLPLENLRIIRGTKL  110
ERBB4-204    ...........................................................MGNLEITSIEHNRDLSFLRSVREVTGYVLVALNQFRYLPLENLRIIRGTKL   51
ERBB4-202    MKPATGLWVWVSLLVAAGTVQPSDSQSVCAGTENKLSSLSDLEQQYRALRKYYENCEVVMGNLEITSIEHNRDLSFLRSVREVTGYVLVALNQFRYLPLENLRIIRGTKL  110
ERBB4-205    MKPATGLWVWVSLLVAAGTVQPSDSQSVCAGTENKLSSLSDLEQQYRALRKYYENCEVVMGNLEITSIEHNRDLSFLRSVREVTGYVLVALNQFRYLPLENLRIIRGTKL  110
```

logo

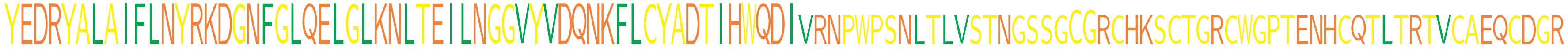

```
LAPATINIB    ..............................................................................................................    0
PF01030.1    YEDRYALAIFLNYRKDGNFGLQELGLKNLTEILNGGVYVDQNKFLCYADTIHWQDI......................................................  106
PF00757      ...........................................................................CGRCHKSCTGRCWGPTENHCQTLTRTVCAEQCDGR   35
PF14843.1    ..............................................................................................................    0
PF07714      ..............................................................................................................    0
PF01030      YEDRYALAIFLNYRKDGNFGLQELGLKNLTEILNGGVYVDQNKFLCYADTIHWQDI......................................................  112
PF14843      ..............................................................................................................    0
ERBB4-203    YEDRYALAIFLNYRKDGNFGLQELGLKNLTEILNGGVYVDQNKFLCYADTIHWQDIVRNPWPSNLTLVSTNGSSGCGRCHKSCTGRCWGPTENHCQTLTRTVCAEQCDGR  194
ERBB4-201    YEDRYALAIFLNYRKDGNFGLQELGLKNLTEILNGGVYVDQNKFLCYADTIHWQDIVRNPWPSNLTLVSTNGSSGCGRCHKSCTGRCWGPTENHCQTLTRTVCAEQCDGR  220
ERBB4-204    YEDRYALAIFLNYRKDGNFGLQELGLKNLTEILNGGVYVDQNKFLCYADTIHWQDIVRNPWPSNLTLVSTNGSSGCG.................................  128
ERBB4-202    YEDRYALAIFLNYRKDGNFGLQELGLKNLTEILNGGVYVDQNKFLCYADTIHWQDIVRNPWPSNLTLVSTNGSSGCGRCHKSCTGRCWGPTENHCQTLTRTVCAEQCDGR  220
ERBB4-205    YEDRYALAIFLNYRKDGNFGLQELGLKNLTEILNGGVYVDQNKFLCYADTIHWQDIVRNPWPSNLTLVSTNGSSGCGRCHKSCTGRCWGPTENHCQTLTRTVCAEQCDGR  220
```

logo

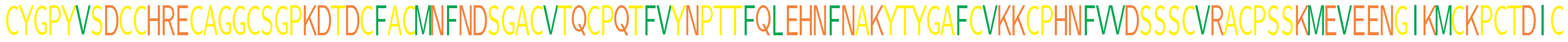

```
LAPATINIB    ..............................................................................................................    0
PF01030.1    ..............................................................................................................  106
PF00757      CYGPYVSDCCHRECAGGCSGPKDTDCFACMNFNDSGACVTQCPQTFVYNPTTFQLEHNFNAKYTYGAFCVKKCPHNFVVDSSSCVRACPSSKMEVEENGIKMCKPCTDIC  145
PF14843.1    ..............................................................................................................    0
PF07714      ..............................................................................................................    0
PF01030      ..............................................................................................................  112
PF14843      ..............................................................................................................    0
ERBB4-203    CYGPYVSDCCHRECAGGCSGPKDTDCFACMNFNDSGACVTQCPQTFVYNPTTFQLEHNFNAKYTYGAFCVKKCPHNFVVDSSSCVRACPSSKMEVEENGIKMCKPCTDIC  304
ERBB4-201    CYGPYVSDCCHRECAGGCSGPKDTDCFACMNFNDSGACVTQCPQTFVYNPTTFQLEHNFNAKYTYGAFCVKKCPHNFVVDSSSCVRACPSSKMEVEENGIKMCKPCTDIC  330
ERBB4-204    ..............................................................................................................  128
ERBB4-202    CYGPYVSDCCHRECAGGCSGPKDTDCFACMNFNDSGACVTQCPQTFVYNPTTFQLEHNFNAKYTYGAFCVKKCPHNFVVDSSSCVRACPSSKMEVEENGIKMCKPCTDIC  330
ERBB4-205    CYGPYVSDCCHRECAGGCSGPKDTDCFACMNFNDSGACVTQCPQTFVYNPTTFQLEHNFNAKYTYGAFCVKKCPHNFVVDSSSCVRACPSSKMEVEENGIKMCKPCTDIC  330
```

```
logo
```

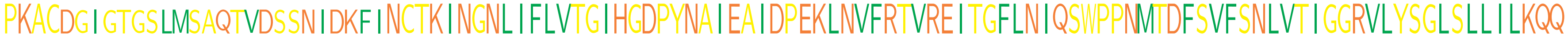

```
LAPATINIB    ..............................................................................................................    0
PF01030.1    ..............................................................................................................  106
PF00757      PKAC..........................................................................................................  149
PF14843.1    ..............................................................................................................    0
PF07714      ..............................................................................................................    0
PF01030      ...........................NCTKINGNLIFLVTGIHGDPYNAIEAIDPEKLNVFRTVREITGFLNIQSWPPNMTDFSVFSNLVTIGGRVLYSGLSLLILKQQ  195
PF14843      ..............................................................................................................    0
ERBB4-203    PKACDGIGTGSLMSAQTVDSSNIDKFINCTKINGNLIFLVTGIHGDPYNAIEAIDPEKLNVFRTVREITGFLNIQSWPPNMTDFSVFSNLVTIGGRVLYSGLSLLILKQQ  414
ERBB4-201    PKACDGIGTGSLMSAQTVDSSNIDKFINCTKINGNLIFLVTGIHGDPYNAIEAIDPEKLNVFRTVREITGFLNIQSWPPNMTDFSVFSNLVTIGGRVLYSGLSLLILKQQ  440
ERBB4-204    ..............................................................................................................  128
ERBB4-202    PKACDGIGTGSLMSAQTVDSSNIDKFINCTKINGNLIFLVTGIHGDPYNAIEAIDPEKLNVFRTVREITGFLNIQSWPPNMTDFSVFSNLVTIGGRVLYSGLSLLILKQQ  440
ERBB4-205    PKACDGIGTGSLMSAQTVDSSNIDKFINCTKINGNLIFLVTGIHGDPYNAIEAIDPEKLNVFRTVREITGFLNIQSWPPNMTDFSVFSNLVTIGGRVLYSGLSLLILKQQ  440
```

```
logo
```

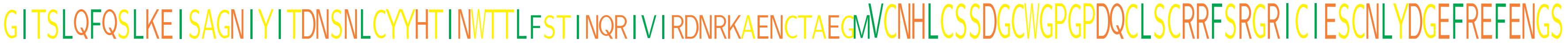

```
LAPATINIB    ..............................................................................................................    0
PF01030.1    ..............................................................................................................  106
PF00757      ..............................................................................................................  149
PF14843.1    .............................................................VCNHLCSSDGCWGPGPDQCLSCRRFSRGRICIESCNLYDGEFREFENGS   49
PF07714      ..............................................................................................................    0
PF01030      GITSLQFQSLKEISAGNIYITDNSNLCYYHTINWTTL.........................................................................  232
PF14843      .............................................................VCNHLCSSDGCWGPGPDQCLSCRRFSRGRICIESCNLYDGEFREFENGS   49
ERBB4-203    GITSLQFQSLKEISAGNIYITDNSNLCYYHTINWTTLFSTINQRIVIRDNRKAENCTAEGMVCNHLCSSDGCWGPGPDQCLSCRRFSRGRICIESCNLYDGEFREFENGS  524
ERBB4-201    GITSLQFQSLKEISAGNIYITDNSNLCYYHTINWTTLFSTINQRIVIRDNRKAENCTAEGMVCNHLCSSDGCWGPGPDQCLSCRRFSRGRICIESCNLYDGEFREFENGS  550
ERBB4-204    ..............................................................................................................  128
ERBB4-202    GITSLQFQSLKEISAGNIYITDNSNLCYYHTINWTTLFSTINQRIVIRDNRKAENCTAEGMVCNHLCSSDGCWGPGPDQCLSCRRFSRGRICIESCNLYDGEFREFENGS  550
ERBB4-205    GITSLQFQSLKEISAGNIYITDNSNLCYYHTINWTTLFSTINQRIVIRDNRKAENCTAEGMVCNHLCSSDGCWGPGPDQCLSCRRFSRGRICIESCNLYDGEFREFENGS  550
```

```
logo
```

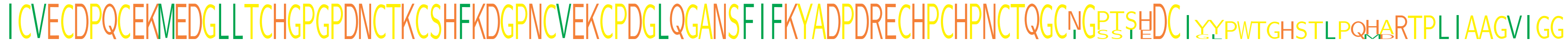

```
LAPATINIB    ..............................................................................................................    0
PF01030.1    ..............................................................................................................  106
PF00757      ..............................................................................................................  149
PF14843.1    ICVECDPQCEKMEDGLLTCHGPGPDNCTKCSHFKDGPNCVEKCPDGLQGANSFIFKYADPDRECHPCHPNCTQGCNGPTSHDC...........................  132
PF07714      ..............................................................................................................    0
PF01030      ..............................................................................................................  232
PF14843      ICVECDPQCEKMEDGLLTCHGPGPDNCTKCSHFKDGPNCVEKCPDGLQGANSFIFKYADPDRECHPCHPNCTQGCIGSSIEDC...........................  132
ERBB4-203    ICVECDPQCEKMEDGLLTCHGPGPDNCTKCSHFKDGPNCVEKCPDGLQGANSFIFKYADPDRECHPCHPNCTQGCNGPTSHDCIYYPWTGHSTLPQHARTPLIAAGVIGG  634
ERBB4-201    ICVECDPQCEKMEDGLLTCHGPGPDNCTKCSHFKDGPNCVEKCPDGLQGANSFIFKYADPDRECHPCHPNCTQGCIGSSIEDCIGL..........MDRTPLIAAGVIGG  650
ERBB4-204    ..............................................................................................................  128
ERBB4-202    ICVECDPQCEKMEDGLLTCHGPGPDNCTKCSHFKDGPNCVEKCPDGLQGANSFIFKYADPDRECHPCHPNCTQGCNGPTSHDCIYYPWTGHSTLPQHARTPLIAAGVIGG  660
ERBB4-205    ICVECDPQCEKMEDGLLTCHGPGPDNCTKCSHFKDGPNCVEKCPDGLQGANSFIFKYADPDRECHPCHPNCTQGCNGPTSHDCIYYPWTGHSTLPQHARTPLIAAGVIGG  660
```

```
logo
            LFILVIVGLTFAVYVRRKSIKKKRALRRFLETELVEPLTPSGTAPNQAQLRILKETELKRVKVLGSGAFGTVYKGIWVPEGETVKIPVAIKILNETTGPKANVEFMDEAL
LAPATINIB   .......................................................................V................AIK...................    4
PF01030.1   ..............................................................................................................  106
PF00757     ..............................................................................................................  149
PF14843.1   ..............................................................................................................  132
PF07714     ..........................................................KRVKVLGSGAFGTVYKGIWVPEGETVKIPVAIKILNETTGPKANVEFMDEAL   52
PF01030     ..............................................................................................................  232
PF14843     ..............................................................................................................  132
ERBB4-203   LFILVIVGLTFAVYVRRKSIKKKRALRRFLETELVEPLTPSGTAPNQAQLRILKETELKRVKVLGSGAFGTVYKGIWVPEGETVKIPVAIKILNETTGPKANVEFMDEAL  744
ERBB4-201   LFILVIVGLTFAVYVRRKSIKKKRALRRFLETELVEPLTPSGTAPNQAQLRILKETELKRVKVLGSGAFGTVYKGIWVPEG.............................  731
ERBB4-204   ..............................................................................................................  128
ERBB4-202   LFILVIVGLTFAVYVRRKSIKKKRALRRFLETELVEPLTPSGTAPNQAQLRILKETELKRVKVLGSGAFGTVYKGIWVPEGETVKIPVAIKILNETTGPKANVEFMDEAL  770
ERBB4-205   LFILVIVGLTFAVYVRRKSIKKKRALRRFLETELVEPLTPSGTAPNQAQLRILKETELKRVKVLGSGAFGTVYKGIWVPEGETVKIPVAIKILNETTGPKANVEFMDEAL  770

logo
            IMASMDHPHLVRLLGVCLSPTIQLVTQLMPHGCLLEYVHEHKDNIGSQLLLNWCVQIAKGMMYLEERRLVHRDLAARNVLVKSPNHVKITDFGLARLLEGDEKEYNADGG
LAPATINIB   .M........V............L.T..M..G...............................................L.........TDF.L................   15
PF01030.1   ..............................................................................................................  106
PF00757     ..............................................................................................................  149
PF14843.1   ..............................................................................................................  132
PF07714     IMASMDHPHLVRLLGVCLSPTIQLVTQLMPHGCLLEYVHEHKDNIGSQLLLNWCVQIAKGMMYLEERRLVHRDLAARNVLVKSPNHVKITDFGLARLLEGDEKEYNADGG  162
PF01030     ..............................................................................................................  232
PF14843     ..............................................................................................................  132
ERBB4-203   IMASMDHPHLVRLLGVCLSPTIQLVTQLMPHGCLLEYVHEHKDNIGSQLLLNWCVQIAKGMMYLEERRLVHRDLAARNVLVKSPNHVKITDFGLARLLEGDEKEYNADGG  854
ERBB4-201   ..............................................................................................................  731
ERBB4-204   ..............................................................................................................  128
ERBB4-202   IMASMDHPHLVRLLGVCLSPTIQLVTQLMPHGCLLEYVHEHKDNIGSQLLLNWCVQIAKGMMYLEERRLVHRDLAARNVLVKSPNHVKITDFGLARLLEGDEKEYNADGG  880
ERBB4-205   IMASMDHPHLVRLLGVCLSPTIQLVTQLMPHGCLLEYVHEHKDNIGSQLLLNWCVQIAKGMMYLEERRLVHRDLAARNVLVKSPNHVKITDFGLARLLEGDEKEYNADGG  880

logo
            KMPIKWMALECIHYRKFTHQSDVWSYGVTIWELMTFGGKPYDGIPTREIPDLLEKGERLPQPPICTIDVYMVMVKCWMIDADSRPKFKELAAEFSRMARDPQRYLVIQGD
LAPATINIB   ..............................................................................................................   15
PF01030.1   ..............................................................................................................  106
PF00757     ..............................................................................................................  149
PF14843.1   ..............................................................................................................  132
PF07714     KMPIKWMALECIHYRKFTHQSDVWSYGVTIWELMTFGGKPYDGIPTREIPDLLEKGERLPQPPICTIDVYMVMVKCWMIDADSRPKFKELAAE.................  255
PF01030     ..............................................................................................................  232
PF14843     ..............................................................................................................  132
ERBB4-203   KMPIKWMALECIHYRKFTHQSDVWSYGVTIWELMTFGGKPYDGIPTREIPDLLEKGERLPQPPICTIDVYMVMVKCWMIDADSRPKFKELAAEFSRMARDPQRYLVIQGD  964
ERBB4-201   ..............................................................................................................  731
ERBB4-204   ..............................................................................................................  128
ERBB4-202   KMPIKWMALECIHYRKFTHQSDVWSYGVTIWELMTFGGKPYDGIPTREIPDLLEKGERLPQPPICTIDVYMVMVKCWMIDADSRPKFKELAAEFSRMARDPQRYLVIQGD  990
ERBB4-205   KMPIKWMALECIHYRKFTHQSDVWSYGVTIWELMTFGGKPYDGIPTREIPDLLEKGERLPQPPICTIDVYMVMVKCWMIDADSRPKFKELAAEFSRMARDPQRYLVIQGD  990
```

```
logo
             DRMKLPSPNDSKFFQNLLDEEDLEDMMDAEEYLVPQAFNIPPPIYTSRARIDSNRSEIGHSPPPAYTPMSGNQFVYRDGGFAAEQGVSVPYRAPTSTIPEAPVAQGATAE
LAPATINIB    ..............................................................................................................    15
PF01030.1    ..............................................................................................................   106
PF00757      ..............................................................................................................   149
PF14843.1    ..............................................................................................................   132
PF07714      ..............................................................................................................   255
PF01030      ..............................................................................................................   232
PF14843      ..............................................................................................................   132
ERBB4-203    DRMKLPSPNDSKFFQNLLDEEDLEDMMDAEEYLVPQAFNIPPPIYTSRARIDSNR................NQFVYRDGGFAAEQGVSVPYRAPTSTIPEAPVAQGATAE  1058
ERBB4-201    ..............................................................................................................   731
ERBB4-204    ..............................................................................................................   128
ERBB4-202    DRMKLPSPNDSKFFQNLLDEEDLEDMMDAEEYLVPQAFNIPPPIYTSRARIDSNRSEIGHSPPPAYTPMSGNQFVYRDGGFAAEQGVSVPYRAPTSTIPEAPVAQGATAE  1100
ERBB4-205    DRMKLPSPNDSKFFQNLLDEEDLEDMMDAEEYLVPQAFNIPPPIYTSRARIDSNR................NQFVYRDGGFAAEQGVSVPYRAPTSTIPEAPVAQGATAE  1084

logo
             IFDDSCCNGTLRKPVAPHVQEDSSTQRYSADPTVFAPERSPRGELDEEGYMTPMRDKPKQEYLNPVEENPFVSRRKNGDLQALDNPEYHNASNGPPKAEDEYVNEPLYLN
LAPATINIB    ..............................................................................................................    15
PF01030.1    ..............................................................................................................   106
PF00757      ..............................................................................................................   149
PF14843.1    ..............................................................................................................   132
PF07714      ..............................................................................................................   255
PF01030      ..............................................................................................................   232
PF14843      ..............................................................................................................   132
ERBB4-203    IFDDSCCNGTLRKPVAPHVQEDSSTQRYSADPTVFAPERSPRGELDEEGYMTPMRDKPKQEYLNPVEENPFVSRRKNGDLQALDNPEYHNASNGPPKAEDEYVNEPLYLN  1168
ERBB4-201    ..............................................................................................................   731
ERBB4-204    ..............................................................................................................   128
ERBB4-202    IFDDSCCNGTLRKPVAPHVQEDSSTQRYSADPTVFAPERSPRGELDEEGYMTPMRDKPKQEYLNPVEENPFVSRRKNGDLQALDNPEYHNASNGPPKAEDEYVNEPLYLN  1210
ERBB4-205    IFDDSCCNGTLRKPVAPHVQEDSSTQRYSADPTVFAPERSPRGELDEEGYMTPMRDKPKQEYLNPVEENPFVSRRKNGDLQALDNPEYHNASNGPPKAEDEYVNEPLYLN  1194

logo
             TFANTLGKAEYLKNNILSMPEKAKKAFDNPDYWNHSLPPRSTLQHPDYLQEYSTKYFYKQNGRIRPIVAENPEYLSEFSLKPGTVLPPPPYRHRNTVV
LAPATINIB    ..................................................................................................    15
PF01030.1    ..................................................................................................   106
PF00757      ..................................................................................................   149
PF14843.1    ..................................................................................................   132
PF07714      ..................................................................................................   255
PF01030      ..................................................................................................   232
PF14843      ..................................................................................................   132
ERBB4-203    TFANTLGKAEYLKNNILSMPEKAKKAFDNPDYWNHSLPPRSTLQHPDYLQEYSTKYFYKQNGRIRPIVAENPEYLSEFSLKPGTVLPPPPYRHRNTVV  1266
ERBB4-201    ..................................................................................................   731
ERBB4-204    ..................................................................................................   128
ERBB4-202    TFANTLGKAEYLKNNILSMPEKAKKAFDNPDYWNHSLPPRSTLQHPDYLQEYSTKYFYKQNGRIRPIVAENPEYLSEFSLKPGTVLPPPPYRHRNTVV  1308
ERBB4-205    TFANTLGKAEYLKNNILSMPEKAKKAFDNPDYWNHSLPPRSTLQHPDYLQEYSTKYFYKQNGRIRPIVAENPEYLSEFSLKPGTVLPPPPYRHRNTVV  1292
```

X non conserved
X similar
X ≥ 0% conserved
X ≥ 50% conserved

```
logo                       MTMTLHTKASGMALLHQIQGNELEPLNRPQLKIPLERPLGEVYLDSSKPAVYNYPEGAAYEFNAAAAANAQVYGQTGLPYGPGSEAAAFGSNGLGGFPPL
AFIMOXIFENE (CHEMBL489)    ....................................................................................................   0
PF02159.3                  .........................................VYLDSSKPAVYNYPEGAAYEFNAAAAANAQVYGQTGLPYGPGSEAAAFGSNGLGGFPPL  59
PF00104.3                  ....................................................................................................   0
PF00105.1                  ....................................................................................................   0
PF02159.2                  .........................................VYLDSSKPAVYNYPEGAAYEFNAAAAANAQVYGQTGLPYGPGS................  43
PF00104.2                  ....................................................................................................   0
PF02159.4                  .........................................VYLDSSKPAVYNYPEGAAYEFNAAAAANAQVYGQTGLPYGPGSEAAAFGSNGLGGFPPL  59
PF12743                    ....................................................................................................   0
PF02159                    .........................................VYLDSSKPAVYNYPEGAAYEFNAAAAANAQVYGQTGLPYGPGSEAAAFGSNGLGGFPPL  59
PF00104.1                  ....................................................................................................   0
PF00105                    ....................................................................................................   0
PF00104                    ....................................................................................................   0
PF02159.1                  .........................................VYLDSSKPAVYNYPEGAAYEFNAAAAANAQVYGQTGLPYGPGSEAAAFGSNGLGGFPPL  59
ESR1-202                   MTMTLHTKASGMALLHQIQGNELEPLNRPQLKIPLERPLGEVYLDSSKPAVYNYPEGAAYEFNAAAAANAQVYGQTGLPYGPGSEAAAFGSNGLGGFPPL 100
ESR1-203                   MTMTLHTKASGMALLHQIQGNELEPLNRPQLKIPLERPLGEVYLDSSKPAVYNYPEGAAYEFNAAAAANAQVYGQTGLPYGPGS................  84
ESR1-207                   MTMTLHTKASGMALLHQIQGNELEPLNRPQLKIPLERPLGEVYLDSSKPAVYNYPEGAAYEFNAAAAANAQVYGQTGLPYGPGSEAAAFGSNGLGGFPPL 100
ESR1-204                   MTMTLHTKASGMALLHQIQGNELEPLNRPQLKIPLERPLGEVYLDSSKPAVYNYPEGAAYEFNAAAAANAQVYGQTGLPYGPGSEAAAFGSNGLGGFPPL 100
ESR1-205                   ....................................................................................................   0
ESR1-206                   ....................................................................................................   0
ESR1-210                   MTMTLHTKASGMALLHQIQGNELEPLNRPQLKIPLERPLGEVYLDSSKPAVYNYPEGAAYEFNAAAAANAQVYGQTGLPYGPGSEAAAFGSNGLGGFPPL 100
ESR1-201                   MTMTLHTKASGMALLHQIQGNELEPLNRPQLKIPLERPLGEVYLDSSKPAVYNYPEGAAYEFNAAAAANAQVYGQTGLPYGPGSEAAAFGSNGLGGFPPL 100
ESR1-208                   MTMTLHTKASGMALLHQIQGNELEPLNRPQLKIPLERPLGEVYLDSSKPAVYNYPEGAAYEFNAAAAANAQVYGQTGLPYGPGSEAAAFGSNGLGGFPPL 100
ESR1-209                   MTMTLHTKASGMALLHQIQGNELEPLNRPQLKIPLERPLGEVYLDSSKPAVYNYPEGAAYEFNAAAAANAQVYGQTGLPYGPGSEAAAFGSNGLGGFPPL 100

logo                       NSVSPSPLMLLHPPPQLSPFLQPHGQQVPYYLENEPSGYTVREAGPPAFYRPNSDNRRQGGRERLASTNDKGSMAMESAKETRYCAVCNDYASGYHYGVW
AFIMOXIFENE (CHEMBL489)    ....................................................................................................   0
PF02159.3                  NSVSPSPLMLLHPPP.....................................................................................  74
PF00104.3                  ....................................................................................................   0
PF00105.1                  ....................................................................................................   0
PF02159.2                  ....................................................................................................  43
PF00104.2                  ....................................................................................................   0
PF02159.4                  NSVSPSPLMLLHPPPQLSPFLQPHGQQVPYYLENEPSGYTVREAGPPAFYR................................................. 110
PF12743                    ....................................................................................................   0
PF02159                    NSVSPSPLMLLHPPPQLSPFLQPHGQQVPYYLENEPSGYTVREAGPPAFYRPNSDNRRQGGRERLASTNDKGSMAMESAKE................... 140
PF00104.1                  ....................................................................................................   0
PF00105                    ...................................................................................YCAVCNDYASGYHYGVW  17
PF00104                    ....................................................................................................   0
PF02159.1                  NSVSPSPLMLLHPPPQLSPFLQPHGQQVPYYLENEPSGYTVREAGPPAFYRNQ............................................... 112
ESR1-202                   NSVSPSPLMLLHPPPQLSPFLQPHGQQVPYYLENEPSGYTVREAGPPAFYRPNSDNRRQGGRERLASTNDKGSMAMESAKETRYCAVCNDYASGYHYGVW 200
ESR1-203                   ....................................................................................................  84
ESR1-207                   NSVSPSPLMLLHPPPQLSPFLQPHGQQVPYYLENEPSGYTVREAGPPAFYRPNSDNRRQGGRERLASTNDKGSMAMESAKETRYCAVCNDYASGYHYGVW 200
ESR1-204                   NSVSPSPLMLLHPPPQLSPFLQPHGQQVPYYLENEPSGYTVREAGPPAFYR................................................. 151
ESR1-205                   ....................................................................................................   0
ESR1-206                   .........................................................................MAMESAKETRYCAVCNDYASGYHYGVW  27
ESR1-210                   NSVSPSPLMLLHPPPQLSPFLQPHGQQVPYYLENEPSGYTVREAGPPAFYRT................................................ 152
ESR1-201                   NSVSPSPLMLLHPPPQLSPFLQPHGQQVPYYLENEPSGYTVREAGPPAFYRPNSDNRRQGGRERLASTNDKGSMAMESAKETRYCAVCNDYASGYHYGVW 200
ESR1-208                   NSVSPSPLMLLHPPPQLSPFLQPHGQQVPYYLENEPSGYTVREAGPPAFYRPNSDNRRQGGRERLASTNDKGSMAMESAKETRYCAVCNDYASGYHYGVW 200
ESR1-209                   NSVSPSPLMLLHPPP..................................................................................... 115
```

```
logo
                             SCEGCKAFFKRSIQGHNDYMCPATNQCTIDKNRRKSCQACRLRKCYEVGMMKGGIRKDRRGGRMLKHKRQRDDGEGRGEVGSAGDMRAANLWPSPLMIKR
AFIMOXIFENE (CHEMBL489)      ....................................................................................................   0
PF02159.3                    ....................................................................................................  74
PF00104.3                    ....................................................................................................   0
PF00105.1                    ................NDYMCPATNQCTIDKNRRKSCQACRLRKCYEVGMMK................................................  36
PF02159.2                    ....................................................................................................  43
PF00104.2                    .....................PFSEASMMGLL..TNL...............................................................  14
PF02159.4                    .................................................................................................... 110
PF12743                      ....................................................................................................   0
PF02159                      .................................................................................................... 140
PF00104.1                    ....................................................................................................   0
PF00105                      SCEGCKAFFKRSIQGHNDYMCPATNQCTIDKNRRKSCQACRLRKCYEVGMMK................................................  69
PF00104                      .....................PFSEASMMGLL..TNL...............................................................  14
PF02159.1                    .................................................................................................... 112
ESR1-202                     SCEGCKAFFKRSIQGHNDYMCPATNQCTIDKNRRKSCQACRLRKCYEVGMMKGGIRKDRRGGRMLKHKRQRDDGEGRGEVGSAGDMRAANLWPSPLMIKR 300
ESR1-203                     ....................................................................................................  84
ESR1-207                     SCEGCKAFFKRSIQGHNDYMCPATNQCTIDKNRRKSCQACRLRKCYEVGMMKGGIRKDRRGGRMLKHKRQRDDGEGRGEVGSAGDMRAANLWPSPLMIKR 300
ESR1-204                     .................................................................................................... 151
ESR1-205                     ...............HNDYMCPATNQCTIDKNRRKSC...............................................................  22
ESR1-206                     SCEGCKAFFKRSIQGHNDYMCPATNQCTIDKNRRKSCQACRLRKCYEVGMMKGGIRKDRRGGRMLKHKRQRDDGEGRGEVGSAGDMRAANLWPSPLMIKR 127
ESR1-210                     .................................................................................................... 152
ESR1-201                     SCEGCKAFFKRSIQGHNDYMCPATNQCTIDKNRRKSCQACRLRKCYEVGMMKGGIRKDRRGGRMLKHKRQRDDGEGRGEVGSAGDMRAANLWPSPLMIKR 300
ESR1-208                     SCEGCKAFFKRSIQGHNDYMCPATNQCTIDKNRRKSCQACRLRKCYEVGMMKGGIRKDRRGGRMLKHKRQRDDGEGRGEVGSAGDMRAANLWPSPLMIKR 300
ESR1-209                     .................................................................................................... 115

logo
                             SKKNSLALSLTADQMVSALLDAEPPILYSEYDPTRPFSEASMMGLLTNLADRELVHMINWAKRVPGFVDLTLHDQVHLLECAWLEILMIGLVWRSMEHPG
AFIMOXIFENE (CHEMBL489)      ..........................................M..LT.LAD.EL............................WL..LM..L..R......  14
PF02159.3                    ....................................................................................................  74
PF00104.3                    .................................................................GFVDLTLHDQVHLLECAWLEILMIGLVWRSMEHPG  35
PF00105.1                    ....................................................................................................  36
PF02159.2                    ....................................................................................................  43
PF00104.2                    .................................................ADRELVHMINWAKRVPGFVDLTLHDQVHLLECAWLEILMIGLVWRSMEHPG  65
PF02159.4                    .................................................................................................... 110
PF12743                      ....................................................................................................   0
PF02159                      .................................................................................................... 140
PF00104.1                    ....................................................................................................   0
PF00105                      ....................................................................................................  69
PF00104                      .................................................ADRELVHMINWAKRVPGFVDLTLHDQVHLLECAWLEILMIGLVWRSMEHPG  65
PF02159.1                    .................................................................................................... 112
ESR1-202                     SKKNSLALSLTADQMVSALLDAEPPILYSEYDPTRPFSEASMMGLLTNLADRELVHMINWAKRVPGFVDLTLHDQVHLLECAWLEILMIGLVWRSMEHPG 400
ESR1-203                     ....................................................................................................  84
ESR1-207                     SKKNSLALSLTADQMVSALLDAEPPILYSEYDPTRPFSEASMMGLLTNLADRELVHMINWAKRVPGFVDLTLHDQVHLLECAWLEILMIGLVWRSMEHPG 400
ESR1-204                     .................................................................................................... 151
ESR1-205                     .................................................QACRLRKCYEVGMMKGGFVDLTLHDQVHLLECAWLEILMIGLVWRSMEHPG  73
ESR1-206                     SKKNSLALSLTADQMVSALLDAEPPILYSEYDPTRPFSEASMMGLLTNLADRELVHMINWAKRVPGFVDLTLHDQVHLLECAWLEILMIGLVWRSMEHPG 227
ESR1-210                     .................................................................................................... 152
ESR1-201                     SKKNSLALSLTADQMVSALLDAEPPILYSEYDPTRPFSEASMMGLLTNLADRELVHMINWAKRVPGFVDLTLHDQVHLLECAWLEILMIGLVWRSMEHPG 400
ESR1-208                     SKKNSLALSLTADQMVSALLDAEPPILYSEYDPTRPFSEASMMGLLTNLADRELVHMINWAKRVPGFVDLTLHDQVHLLECAWLEILMIGLVWRSMEHPG 400
ESR1-209                     .................................................................................................... 115
```

```
logo
                             KLLFAPNLLLDRNQGKCVEGMVEIFDMLLATSSRFRMMNLQGEEFVCLKSIILLNSGVYTFLSSTLKSLEEKDHIHRVLDKITDTLIHLMAKAGLTLQQQ
AFIMOXIFENE (CHEMBL489)      ...F................M..IF..L........................................................................   19
PF02159.3                    ....................................................................................................   74
PF00104.3                    KLLFAPNLLLDRNQGKCVEGMVEI............................................................................   59
PF00105.1                    ....................................................................................................   36
PF02159.2                    ....................................................................................................   43
PF00104.2                    KLLFAPNLLLDRNQGKCVEGMVEIFDMLLATSSRFRMMNLQGEEFVCLKSIILLNS............................................  121
PF02159.4                    ....................................................................................................  110
PF12743                      ....................................................................................................    0
PF02159                      ....................................................................................................  140
PF00104.1                    ............NQGKCVEGMVEIFDMLLATSSRFRMMNLQGEEFVCLKSIILLNSGVYTFLSSTLKSLEEKDHIHRVLDKITDTLIHLMAKAGLTLQQQ   88
PF00105                      ....................................................................................................   69
PF00104                      KLLFAPNLLLDRNQGKCVEGMVEIFDMLLATSSRFRMMNLQGEEFVCLKSIILLNSGVYTFLSSTLKSLEEKDHIHRVLDKITDTLIHLMAKAGLTLQQQ  165
PF02159.1                    ....................................................................................................  112
ESR1-202                     KLLFAPNLLLDRNQGKCVEGMVEIFDMLLATSSRFRMMNLQGEEFVCLKSIILLNSGVYTFLSSTLKSLEEKDHIHRVLDKITDTLIHLMAKAGLTLQQQ  500
ESR1-203                     ....................................................................................................   84
ESR1-207                     KLLFAPNLLLDRNQGKCVEGMVEIFDMLLATSSRFRMMNLQGEEFVCLKSIILLNSGVYTFLSSTLKSLEEKDHIHRVLDKITDTLIHLMAKAGLTLQQQ  500
ESR1-204                     ............NQGKCVEGMVEIFDMLLATSSRFRMMNLQGEEFVCLKSIILLNSGVYTFLSSTLKSLEEKDHIHRVLDKITDTLIHLMAKAGLTLQQQ  239
ESR1-205                     KLLFAPNLLLDRNQGKCVEGMVEIFDMLLATSSR..................................................................  107
ESR1-206                     KLLFAPNLLLDRNQGKCVEGMVEIFDMLLATSSRFRMMNLQGEEFVCLKSIILLNSGISHVEAK........KRILNLHPKIFGNKWF..PRV.......  310
ESR1-210                     ....................................................................................................  152
ESR1-201                     KLLFAPNLLLDRNQGKCVEGMVEIFDMLLATSSRFRMMNLQGEEFVCLKSIILLNSGVYTFLSSTLKSLEEKDHIHRVLDKITDTLIHLMAKAGLTLQQQ  500
ESR1-208                     KLLFAPNLLLDRNQGKCVEGMVEIFDMLLATSSRFRMMNLQGEEFVCLKSIILLNSGVYTFLSSTLKSLEEKDHIHRVLDKITDTLIHLMAKAGLTLQQQ  500
ESR1-209                     ....................................................................................................  115

logo
                             HQRLAQLLLILSHIRHMSNKGMEHLYSMKCKNVVPLYDLLLEMLDAHRLHAPTSRGGASVEETDQSHLATAGSTSSHSLQKYYITGEAEGFPATV
AFIMOXIFENE (CHEMBL489)      ....................G..HL.......V.P............................................................   24
PF02159.3                    ...............................................................................................   74
PF00104.3                    ...............................................................................................   59
PF00105.1                    ...............................................................................................   36
PF02159.2                    ...............................................................................................   43
PF00104.2                    ...............................................................................................  121
PF02159.4                    ...............................................................................................  110
PF12743                      ...................................................PTSRGGASVEETDQSHLATAGSTSSHSLQKYYITGEAEGFPATV   44
PF02159                      ...............................................................................................  140
PF00104.1                    HQRLAQLLLILSHIRHMSNKGMEHLYSMKC.................................................................  118
PF00105                      ...............................................................................................   69
PF00104                      HQRLAQLLLILSHIRHMSNKGMEHLYSMKC.................................................................  195
PF02159.1                    ...............................................................................................  112
ESR1-202                     HQRLAQLLLILSHIRHMSNKGMEHLYSMKCKNVVPLYDLLLEMLDAHRLHAPTSRGGASVEETDQSHLATAGSTSSHSLQKYYITGEAEGFPATV  595
ESR1-203                     ...............................................................................................   84
ESR1-207                     HQRLAQLLLILSHIRHMSNKGMEHLYSMKCKNVVPLYDLLLEMLDAHRLHAPTSRGGASVEETDQSHLATAGSTSSHSLQKYYITGEAEGFPATV  595
ESR1-204                     HQRLAQLLLILSHIRHMSNKGMEHLYSMKCKNVVPLYDLLLEMLDAHRLHAPTSRGGASVEETDQSHLATAGSTSSHSLQKYYITGEAEGFPATV  334
ESR1-205                     ...............................................................................................  107
ESR1-206                     ...............................................................................................  310
ESR1-210                     ...............................................................................................  152
ESR1-201                     HQRLAQLLLILSHIRHMSNKGMEHLYSMKCKNVVPLYDLLLEMLDAHRLHAPTSRGGASVEETDQSHLATAGSTSSHSLQKYYITGEAEGFPATV  595
ESR1-208                     HQRLAQLLLILSHIRHMSNKGMEHLYSMKCKNVVPLYDLLLEMLDAHRLHAPTSRGGASVEETDQSHLATAGSTSSHSLQKYYITGEAEGFPATV  595
ESR1-209                     ...............................................................................................  115
```

X non conserved
X similar
X $\geq 0\%$ conserved
X $\geq 50\%$ conserved

```
logo                MTMTLHTKASGMALLHQIQGNELEPLNRPQLKIPLERPLGEVYLDSSKPAVYNYPEGAAYEFNAAAAANAQVYGQTGLPYGPGSEAAAFGSNGLGGFPPLNSVSP
DIETHYLSTILBESTROL  .........................................................................................................   0
PF02159.3           .........................................VYLDSSKPAVYNYPEGAAYEFNAAAAANAQVYGQTGLPYGPGSEAAAFGSNGLGGFPPLNSVSP  64
PF00104.3           .........................................................................................................   0
PF00105.1           .........................................................................................................   0
PF02159.2           .........................................VYLDSSKPAVYNYPEGAAYEFNAAAAANAQVYGQTGLPYGPGS.....................  43
PF00104.2           .........................................................................................................   0
PF02159.4           .........................................VYLDSSKPAVYNYPEGAAYEFNAAAAANAQVYGQTGLPYGPGSEAAAFGSNGLGGFPPLNSVSP  64
PF12743             .........................................................................................................   0
PF02159             .........................................VYLDSSKPAVYNYPEGAAYEFNAAAAANAQVYGQTGLPYGPGSEAAAFGSNGLGGFPPLNSVSP  64
PF00104.1           .........................................................................................................   0
PF00105             .........................................................................................................   0
PF00104             .........................................................................................................   0
PF02159.1           .........................................VYLDSSKPAVYNYPEGAAYEFNAAAAANAQVYGQTGLPYGPGSEAAAFGSNGLGGFPPLNSVSP  64
ESR1-202            MTMTLHTKASGMALLHQIQGNELEPLNRPQLKIPLERPLGEVYLDSSKPAVYNYPEGAAYEFNAAAAANAQVYGQTGLPYGPGSEAAAFGSNGLGGFPPLNSVSP 105
ESR1-203            MTMTLHTKASGMALLHQIQGNELEPLNRPQLKIPLERPLGEVYLDSSKPAVYNYPEGAAYEFNAAAAANAQVYGQTGLPYGPGS.....................  84
ESR1-207            MTMTLHTKASGMALLHQIQGNELEPLNRPQLKIPLERPLGEVYLDSSKPAVYNYPEGAAYEFNAAAAANAQVYGQTGLPYGPGSEAAAFGSNGLGGFPPLNSVSP 105
ESR1-204            MTMTLHTKASGMALLHQIQGNELEPLNRPQLKIPLERPLGEVYLDSSKPAVYNYPEGAAYEFNAAAAANAQVYGQTGLPYGPGSEAAAFGSNGLGGFPPLNSVSP 105
ESR1-205            .........................................................................................................   0
ESR1-206            .........................................................................................................   0
ESR1-210            MTMTLHTKASGMALLHQIQGNELEPLNRPQLKIPLERPLGEVYLDSSKPAVYNYPEGAAYEFNAAAAANAQVYGQTGLPYGPGSEAAAFGSNGLGGFPPLNSVSP 105
ESR1-201            MTMTLHTKASGMALLHQIQGNELEPLNRPQLKIPLERPLGEVYLDSSKPAVYNYPEGAAYEFNAAAAANAQVYGQTGLPYGPGSEAAAFGSNGLGGFPPLNSVSP 105
ESR1-208            MTMTLHTKASGMALLHQIQGNELEPLNRPQLKIPLERPLGEVYLDSSKPAVYNYPEGAAYEFNAAAAANAQVYGQTGLPYGPGSEAAAFGSNGLGGFPPLNSVSP 105
ESR1-209            MTMTLHTKASGMALLHQIQGNELEPLNRPQLKIPLERPLGEVYLDSSKPAVYNYPEGAAYEFNAAAAANAQVYGQTGLPYGPGSEAAAFGSNGLGGFPPLNSVSP 105

logo                SPLMLLHPPPQLSPFLQPHGQQVPYYLENEPSGYTVREAGPPAFYRPNSDNRRQGGRERLASTNDKGSMAMESAKETRYCAVCNDYASGYHYGVWSCEGCKAFFK
DIETHYLSTILBESTROL  .........................................................................................................   0
PF02159.3           SPLMLLHPPP...............................................................................................  74
PF00104.3           .........................................................................................................   0
PF00105.1           .........................................................................................................   0
PF02159.2           .........................................................................................................  43
PF00104.2           .........................................................................................................   0
PF02159.4           SPLMLLHPPPQLSPFLQPHGQQVPYYLENEPSGYTVREAGPPAFYR........................................................... 110
PF12743             .........................................................................................................   0
PF02159             SPLMLLHPPPQLSPFLQPHGQQVPYYLENEPSGYTVREAGPPAFYRPNSDNRRQGGRERLASTNDKGSMAMESAKE............................. 140
PF00104.1           .........................................................................................................   0
PF00105             ..............................................................................YCAVCNDYASGYHYGVWSCEGCKAFFK  27
PF00104             .........................................................................................................   0
PF02159.1           SPLMLLHPPPQLSPFLQPHGQQVPYYLENEPSGYTVREAGPPAFYRNQ......................................................... 112
ESR1-202            SPLMLLHPPPQLSPFLQPHGQQVPYYLENEPSGYTVREAGPPAFYRPNSDNRRQGGRERLASTNDKGSMAMESAKETRYCAVCNDYASGYHYGVWSCEGCKAFFK 210
ESR1-203            .........................................................................................................  84
ESR1-207            SPLMLLHPPPQLSPFLQPHGQQVPYYLENEPSGYTVREAGPPAFYRPNSDNRRQGGRERLASTNDKGSMAMESAKETRYCAVCNDYASGYHYGVWSCEGCKAFFK 210
ESR1-204            SPLMLLHPPPQLSPFLQPHGQQVPYYLENEPSGYTVREAGPPAFYR........................................................... 151
ESR1-205            .........................................................................................................   0
ESR1-206            ....................................................................MAMESAKETRYCAVCNDYASGYHYGVWSCEGCKAFFK  37
ESR1-210            SPLMLLHPPPQLSPFLQPHGQQVPYYLENEPSGYTVREAGPPAFYRT.......................................................... 152
ESR1-201            SPLMLLHPPPQLSPFLQPHGQQVPYYLENEPSGYTVREAGPPAFYRPNSDNRRQGGRERLASTNDKGSMAMESAKETRYCAVCNDYASGYHYGVWSCEGCKAFFK 210
ESR1-208            SPLMLLHPPPQLSPFLQPHGQQVPYYLENEPSGYTVREAGPPAFYRPNSDNRRQGGRERLASTNDKGSMAMESAKETRYCAVCNDYASGYHYGVWSCEGCKAFFK 210
ESR1-209            SPLMLLHPPP............................................................................................... 115
```

logo

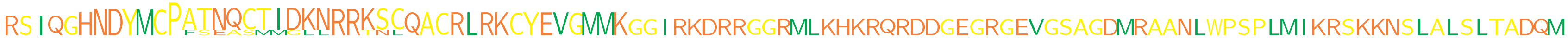

```
DIETHYLSTILBESTROL  .........................................................................................................    0
PF02159.3           .........................................................................................................   74
PF00104.3           .........................................................................................................    0
PF00105.1           ......NDYMCPATNQCTIDKNRRKSCQACRLRKCYEVGMMK...............................................................   36
PF02159.2           .........................................................................................................   43
PF00104.2           ...........PFSEASMMGLL..TNL..............................................................................   14
PF02159.4           .........................................................................................................  110
PF12743             .........................................................................................................    0
PF02159             .........................................................................................................  140
PF00104.1           .........................................................................................................    0
PF00105             RSIQGHNDYMCPATNQCTIDKNRRKSCQACRLRKCYEVGMMK...............................................................   69
PF00104             ...........PFSEASMMGLL..TNL..............................................................................   14
PF02159.1           .........................................................................................................  112
ESR1-202            RSIQGHNDYMCPATNQCTIDKNRRKSCQACRLRKCYEVGMMKGGIRKDRRGGRMLKHKRQRDDGEGRGEVGSAGDMRAANLWPSPLMIKRSKKNSLALSLTADQM  315
ESR1-203            .........................................................................................................   84
ESR1-207            RSIQGHNDYMCPATNQCTIDKNRRKSCQACRLRKCYEVGMMKGGIRKDRRGGRMLKHKRQRDDGEGRGEVGSAGDMRAANLWPSPLMIKRSKKNSLALSLTADQM  315
ESR1-204            .........................................................................................................  151
ESR1-205            .....HNDYMCPATNQCTIDKNRRKSC..............................................................................   22
ESR1-206            RSIQGHNDYMCPATNQCTIDKNRRKSCQACRLRKCYEVGMMKGGIRKDRRGGRMLKHKRQRDDGEGRGEVGSAGDMRAANLWPSPLMIKRSKKNSLALSLTADQM  142
ESR1-210            .........................................................................................................  152
ESR1-201            RSIQGHNDYMCPATNQCTIDKNRRKSCQACRLRKCYEVGMMKGGIRKDRRGGRMLKHKRQRDDGEGRGEVGSAGDMRAANLWPSPLMIKRSKKNSLALSLTADQM  315
ESR1-208            RSIQGHNDYMCPATNQCTIDKNRRKSCQACRLRKCYEVGMMKGGIRKDRRGGRMLKHKRQRDDGEGRGEVGSAGDMRAANLWPSPLMIKRSKKNSLALSLTADQM  315
ESR1-209            .........................................................................................................  115
```

logo

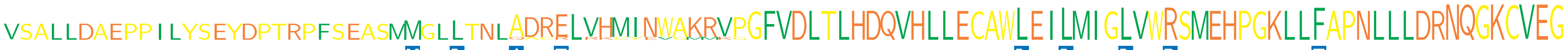

```
DIETHYLSTILBESTROL  ...........................M..L...A..E..............................L..L...L..R.........F................    9
PF02159.3           .........................................................................................................   74
PF00104.3           ..................................................GFVDLTLHDQVHLLECAWLEILMIGLVWRSMEHPGKLLFAPNLLLDRNQGKCVEG   55
PF00105.1           .........................................................................................................   36
PF02159.2           .........................................................................................................   43
PF00104.2           ..................................ADRELVHMINWAKRVPGFVDLTLHDQVHLLECAWLEILMIGLVWRSMEHPGKLLFAPNLLLDRNQGKCVEG   85
PF02159.4           .........................................................................................................  110
PF12743             .........................................................................................................    0
PF02159             .........................................................................................................  140
PF00104.1           .................................................................................................NQGKCVEG    8
PF00105             .........................................................................................................   69
PF00104             ..................................ADRELVHMINWAKRVPGFVDLTLHDQVHLLECAWLEILMIGLVWRSMEHPGKLLFAPNLLLDRNQGKCVEG   85
PF02159.1           .........................................................................................................  112
ESR1-202            VSALLDAEPPILYSEYDPTRPFSEASMMGLLTNLADRELVHMINWAKRVPGFVDLTLHDQVHLLECAWLEILMIGLVWRSMEHPGKLLFAPNLLLDRNQGKCVEG  420
ESR1-203            .........................................................................................................   84
ESR1-207            VSALLDAEPPILYSEYDPTRPFSEASMMGLLTNLADRELVHMINWAKRVPGFVDLTLHDQVHLLECAWLEILMIGLVWRSMEHPGKLLFAPNLLLDRNQGKCVEG  420
ESR1-204            .................................................................................................NQGKCVEG  159
ESR1-205            ..................................QACRLRKCYEVGMMKGGFVDLTLHDQVHLLECAWLEILMIGLVWRSMEHPGKLLFAPNLLLDRNQGKCVEG   93
ESR1-206            VSALLDAEPPILYSEYDPTRPFSEASMMGLLTNLADRELVHMINWAKRVPGFVDLTLHDQVHLLECAWLEILMIGLVWRSMEHPGKLLFAPNLLLDRNQGKCVEG  247
ESR1-210            .........................................................................................................  152
ESR1-201            VSALLDAEPPILYSEYDPTRPFSEASMMGLLTNLADRELVHMINWAKRVPGFVDLTLHDQVHLLECAWLEILMIGLVWRSMEHPGKLLFAPNLLLDRNQGKCVEG  420
ESR1-208            VSALLDAEPPILYSEYDPTRPFSEASMMGLLTNLADRELVHMINWAKRVPGFVDLTLHDQVHLLECAWLEILMIGLVWRSMEHPGKLLFAPNLLLDRNQGKCVEG  420
ESR1-209            .........................................................................................................  115
```

```
logo                 MVEIFDMLLATSSRFRMMNLQGEEFVCLKSIILLNSGVYTFLSSTLKSLEEKDHIHRVLDKITDTLIHLMAKAGLTLQQQHQRLAQLLLILSHIRHMSNKGMEHL
DIETHYLSTILBESTROL   M...................................................................................................G..HL   13
PF02159.3            .........................................................................................................   74
PF00104.3            MVEI.....................................................................................................   59
PF00105.1            .........................................................................................................   36
PF02159.2            .........................................................................................................   43
PF00104.2            MVEIFDMLLATSSRFRMMNLQGEEFVCLKSIILLNS.....................................................................  121
PF02159.4            .........................................................................................................  110
PF12743              .........................................................................................................    0
PF02159              .........................................................................................................  140
PF00104.1            MVEIFDMLLATSSRFRMMNLQGEEFVCLKSIILLNSGVYTFLSSTLKSLEEKDHIHRVLDKITDTLIHLMAKAGLTLQQQHQRLAQLLLILSHIRHMSNKGMEHL  113
PF00105              .........................................................................................................   69
PF00104              MVEIFDMLLATSSRFRMMNLQGEEFVCLKSIILLNSGVYTFLSSTLKSLEEKDHIHRVLDKITDTLIHLMAKAGLTLQQQHQRLAQLLLILSHIRHMSNKGMEHL  190
PF02159.1            .........................................................................................................  112
ESR1-202             MVEIFDMLLATSSRFRMMNLQGEEFVCLKSIILLNSGVYTFLSSTLKSLEEKDHIHRVLDKITDTLIHLMAKAGLTLQQQHQRLAQLLLILSHIRHMSNKGMEHL  525
ESR1-203             .........................................................................................................   84
ESR1-207             MVEIFDMLLATSSRFRMMNLQGEEFVCLKSIILLNSGVYTFLSSTLKSLEEKDHIHRVLDKITDTLIHLMAKAGLTLQQQHQRLAQLLLILSHIRHMSNKGMEHL  525
ESR1-204             MVEIFDMLLATSSRFRMMNLQGEEFVCLKSIILLNSGVYTFLSSTLKSLEEKDHIHRVLDKITDTLIHLMAKAGLTLQQQHQRLAQLLLILSHIRHMSNKGMEHL  264
ESR1-205             MVEIFDMLLATSSR...........................................................................................  107
ESR1-206             MVEIFDMLLATSSRFRMMNLQGEEFVCLKSIILLNSGISHVEAK........KRILNLHPKIFGNKWF..PRV................................  310
ESR1-210             .........................................................................................................  152
ESR1-201             MVEIFDMLLATSSRFRMMNLQGEEFVCLKSIILLNSGVYTFLSSTLKSLEEKDHIHRVLDKITDTLIHLMAKAGLTLQQQHQRLAQLLLILSHIRHMSNKGMEHL  525
ESR1-208             MVEIFDMLLATSSRFRMMNLQGEEFVCLKSIILLNSGVYTFLSSTLKSLEEKDHIHRVLDKITDTLIHLMAKAGLTLQQQHQRLAQLLLILSHIRHMSNKGMEHL  525
ESR1-209             .........................................................................................................  115

logo                 YSMKCKNVVPLYDLLLEMLDAHRLHAPTSRGGASVEETDQSHLATAGSTSSHSLQKYYITGEAEGFPATV
DIETHYLSTILBESTROL   ..M...................................................................   14
PF02159.3            ......................................................................   74
PF00104.3            ......................................................................   59
PF00105.1            ......................................................................   36
PF02159.2            ......................................................................   43
PF00104.2            ......................................................................  121
PF02159.4            ......................................................................  110
PF12743              ..........................PTSRGGASVEETDQSHLATAGSTSSHSLQKYYITGEAEGFPATV   44
PF02159              ......................................................................  140
PF00104.1            YSMKC.................................................................  118
PF00105              ......................................................................   69
PF00104              YSMKC.................................................................  195
PF02159.1            ......................................................................  112
ESR1-202             YSMKCKNVVPLYDLLLEMLDAHRLHAPTSRGGASVEETDQSHLATAGSTSSHSLQKYYITGEAEGFPATV  595
ESR1-203             ......................................................................   84
ESR1-207             YSMKCKNVVPLYDLLLEMLDAHRLHAPTSRGGASVEETDQSHLATAGSTSSHSLQKYYITGEAEGFPATV  595
ESR1-204             YSMKCKNVVPLYDLLLEMLDAHRLHAPTSRGGASVEETDQSHLATAGSTSSHSLQKYYITGEAEGFPATV  334
ESR1-205             ......................................................................  107
ESR1-206             ......................................................................  310
ESR1-210             ......................................................................  152
ESR1-201             YSMKCKNVVPLYDLLLEMLDAHRLHAPTSRGGASVEETDQSHLATAGSTSSHSLQKYYITGEAEGFPATV  595
ESR1-208             YSMKCKNVVPLYDLLLEMLDAHRLHAPTSRGGASVEETDQSHLATAGSTSSHSLQKYYITGEAEGFPATV  595
ESR1-209             ......................................................................  115
```

- X non conserved
- X similar
- X $\geq$ 0% conserved
- X $\geq$ 50% conserved

```
logo         MTMTLHTKASGMALLHQIQGNELEPLNRPQLKIPLERPLGEVYLDSSKPAVYNYPEGAAYEFNAAAAANAQVYGQTGLPYGPGSEAAAFGSNGLGGFPPLNSVSPSPLML
GENISTEIN    ..............................................................................................................   0
PF02159.3    .........................................VYLDSSKPAVYNYPEGAAYEFNAAAAANAQVYGQTGLPYGPGSEAAAFGSNGLGGFPPLNSVSPSPLML  69
PF00104.3    ..............................................................................................................   0
PF00105.1    ..............................................................................................................   0
PF02159.2    .........................................VYLDSSKPAVYNYPEGAAYEFNAAAAANAQVYGQTGLPYGPGS..........................  43
PF00104.2    ..............................................................................................................   0
PF02159.4    .........................................VYLDSSKPAVYNYPEGAAYEFNAAAAANAQVYGQTGLPYGPGSEAAAFGSNGLGGFPPLNSVSPSPLML  69
PF12743      ..............................................................................................................   0
PF02159      .........................................VYLDSSKPAVYNYPEGAAYEFNAAAAANAQVYGQTGLPYGPGSEAAAFGSNGLGGFPPLNSVSPSPLML  69
PF00104.1    ..............................................................................................................   0
PF00105      ..............................................................................................................   0
PF00104      ..............................................................................................................   0
PF02159.1    .........................................VYLDSSKPAVYNYPEGAAYEFNAAAAANAQVYGQTGLPYGPGSEAAAFGSNGLGGFPPLNSVSPSPLML  69
ESR1-202     MTMTLHTKASGMALLHQIQGNELEPLNRPQLKIPLERPLGEVYLDSSKPAVYNYPEGAAYEFNAAAAANAQVYGQTGLPYGPGSEAAAFGSNGLGGFPPLNSVSPSPLML 110
ESR1-203     MTMTLHTKASGMALLHQIQGNELEPLNRPQLKIPLERPLGEVYLDSSKPAVYNYPEGAAYEFNAAAAANAQVYGQTGLPYGPGS..........................  84
ESR1-207     MTMTLHTKASGMALLHQIQGNELEPLNRPQLKIPLERPLGEVYLDSSKPAVYNYPEGAAYEFNAAAAANAQVYGQTGLPYGPGSEAAAFGSNGLGGFPPLNSVSPSPLML 110
ESR1-204     MTMTLHTKASGMALLHQIQGNELEPLNRPQLKIPLERPLGEVYLDSSKPAVYNYPEGAAYEFNAAAAANAQVYGQTGLPYGPGSEAAAFGSNGLGGFPPLNSVSPSPLML 110
ESR1-205     ..............................................................................................................   0
ESR1-206     ..............................................................................................................   0
ESR1-210     MTMTLHTKASGMALLHQIQGNELEPLNRPQLKIPLERPLGEVYLDSSKPAVYNYPEGAAYEFNAAAAANAQVYGQTGLPYGPGSEAAAFGSNGLGGFPPLNSVSPSPLML 110
ESR1-201     MTMTLHTKASGMALLHQIQGNELEPLNRPQLKIPLERPLGEVYLDSSKPAVYNYPEGAAYEFNAAAAANAQVYGQTGLPYGPGSEAAAFGSNGLGGFPPLNSVSPSPLML 110
ESR1-208     MTMTLHTKASGMALLHQIQGNELEPLNRPQLKIPLERPLGEVYLDSSKPAVYNYPEGAAYEFNAAAAANAQVYGQTGLPYGPGSEAAAFGSNGLGGFPPLNSVSPSPLML 110
ESR1-209     MTMTLHTKASGMALLHQIQGNELEPLNRPQLKIPLERPLGEVYLDSSKPAVYNYPEGAAYEFNAAAAANAQVYGQTGLPYGPGSEAAAFGSNGLGGFPPLNSVSPSPLML 110

logo         LHPPPQLSPFLQPHGQQVPYYLENEPSGYTVREAGPPAFYRPNSDNRRQGGRERLASTNDKGSMAMESAKETRYCAVCNDYASGYHYGVWSCEGCKAFFKRSIQGHNDYM
GENISTEIN    ..............................................................................................................   0
PF02159.3    LHPPP.........................................................................................................  74
PF00104.3    ..............................................................................................................   0
PF00105.1    ..........................................................................................................NDYM   4
PF02159.2    ..............................................................................................................  43
PF00104.2    ..............................................................................................................   0
PF02159.4    LHPPPQLSPFLQPHGQQVPYYLENEPSGYTVREAGPPAFYR..................................................................... 110
PF12743      ..............................................................................................................   0
PF02159      LHPPPQLSPFLQPHGQQVPYYLENEPSGYTVREAGPPAFYRPNSDNRRQGGRERLASTNDKGSMAMESAKE....................................... 140
PF00104.1    ..............................................................................................................   0
PF00105      .........................................................................YCAVCNDYASGYHYGVWSCEGCKAFFKRSIQGHNDYM  37
PF00104      ..............................................................................................................   0
PF02159.1    LHPPPQLSPFLQPHGQQVPYYLENEPSGYTVREAGPPAFYRNQ................................................................... 112
ESR1-202     LHPPPQLSPFLQPHGQQVPYYLENEPSGYTVREAGPPAFYRPNSDNRRQGGRERLASTNDKGSMAMESAKETRYCAVCNDYASGYHYGVWSCEGCKAFFKRSIQGHNDYM 220
ESR1-203     ..............................................................................................................  84
ESR1-207     LHPPPQLSPFLQPHGQQVPYYLENEPSGYTVREAGPPAFYRPNSDNRRQGGRERLASTNDKGSMAMESAKETRYCAVCNDYASGYHYGVWSCEGCKAFFKRSIQGHNDYM 220
ESR1-204     LHPPPQLSPFLQPHGQQVPYYLENEPSGYTVREAGPPAFYR..................................................................... 151
ESR1-205     .........................................................................................................HNDYM   5
ESR1-206     ...............................................................MAMESAKETRYCAVCNDYASGYHYGVWSCEGCKAFFKRSIQGHNDYM  47
ESR1-210     LHPPPQLSPFLQPHGQQVPYYLENEPSGYTVREAGPPAFYRT.................................................................... 152
ESR1-201     LHPPPQLSPFLQPHGQQVPYYLENEPSGYTVREAGPPAFYRPNSDNRRQGGRERLASTNDKGSMAMESAKETRYCAVCNDYASGYHYGVWSCEGCKAFFKRSIQGHNDYM 220
ESR1-208     LHPPPQLSPFLQPHGQQVPYYLENEPSGYTVREAGPPAFYRPNSDNRRQGGRERLASTNDKGSMAMESAKETRYCAVCNDYASGYHYGVWSCEGCKAFFKRSIQGHNDYM 220
ESR1-209     LHPPP......................................................................................................... 115
```

```
logo
             CPATNQCTIDKNRRKSCQACRLRKCYEVGMMKGGIRKDRRGGRMLKHKRQRDDGEGRGEVGSAGDMRAANLWPSPLMIKRSKKNSLALSLTADQMVSALLDAEPPILYSE
GENISTEIN    ..............................................................................................................   0
PF02159.3    ..............................................................................................................  74
PF00104.3    ..............................................................................................................   0
PF00105.1    CPATNQCTIDKNRRKSCQACRLRKCYEVGMMK..............................................................................  36
PF02159.2    ..............................................................................................................  43
PF00104.2    .PFSEASMMGLL..TNL.............................................................................................  14
PF02159.4    .............................................................................................................. 110
PF12743      ..............................................................................................................   0
PF02159      .............................................................................................................. 140
PF00104.1    ..............................................................................................................   0
PF00105      CPATNQCTIDKNRRKSCQACRLRKCYEVGMMK..............................................................................  69
PF00104      .PFSEASMMGLL..TNL.............................................................................................  14
PF02159.1    .............................................................................................................. 112
ESR1-202     CPATNQCTIDKNRRKSCQACRLRKCYEVGMMKGGIRKDRRGGRMLKHKRQRDDGEGRGEVGSAGDMRAANLWPSPLMIKRSKKNSLALSLTADQMVSALLDAEPPILYSE 330
ESR1-203     ..............................................................................................................  84
ESR1-207     CPATNQCTIDKNRRKSCQACRLRKCYEVGMMKGGIRKDRRGGRMLKHKRQRDDGEGRGEVGSAGDMRAANLWPSPLMIKRSKKNSLALSLTADQMVSALLDAEPPILYSE 330
ESR1-204     .............................................................................................................. 151
ESR1-205     CPATNQCTIDKNRRKSC.............................................................................................  22
ESR1-206     CPATNQCTIDKNRRKSCQACRLRKCYEVGMMKGGIRKDRRGGRMLKHKRQRDDGEGRGEVGSAGDMRAANLWPSPLMIKRSKKNSLALSLTADQMVSALLDAEPPILYSE 157
ESR1-210     .............................................................................................................. 152
ESR1-201     CPATNQCTIDKNRRKSCQACRLRKCYEVGMMKGGIRKDRRGGRMLKHKRQRDDGEGRGEVGSAGDMRAANLWPSPLMIKRSKKNSLALSLTADQMVSALLDAEPPILYSE 330
ESR1-208     CPATNQCTIDKNRRKSCQACRLRKCYEVGMMKGGIRKDRRGGRMLKHKRQRDDGEGRGEVGSAGDMRAANLWPSPLMIKRSKKNSLALSLTADQMVSALLDAEPPILYSE 330
ESR1-209     .............................................................................................................. 115

logo
             YDPTRPFSEASMMGLLTNLADRELVHMINWAKRVPGFVDLTLHDQVHLLECAWLEILMIGLVWRSMEHPGKLLFAPNLLLDRNQGKCVEGMVEIFDMLLATSSRFRMMNL
GENISTEIN    ............M..L...A..E.................................L......R..........................M..I................   8
PF02159.3    ..............................................................................................................  74
PF00104.3    ....................................GFVDLTLHDQVHLLECAWLEILMIGLVWRSMEHPGKLLFAPNLLLDRNQGKCVEGMVEI................  59
PF00105.1    ..............................................................................................................  36
PF02159.2    ..............................................................................................................  43
PF00104.2    ...................ADRELVHMINWAKRVPGFVDLTLHDQVHLLECAWLEILMIGLVWRSMEHPGKLLFAPNLLLDRNQGKCVEGMVEIFDMLLATSSRFRMMNL 105
PF02159.4    .............................................................................................................. 110
PF12743      ..............................................................................................................   0
PF02159      .............................................................................................................. 140
PF00104.1    ..................................................................................NQGKCVEGMVEIFDMLLATSSRFRMMNL  28
PF00105      ..............................................................................................................  69
PF00104      ...................ADRELVHMINWAKRVPGFVDLTLHDQVHLLECAWLEILMIGLVWRSMEHPGKLLFAPNLLLDRNQGKCVEGMVEIFDMLLATSSRFRMMNL 105
PF02159.1    .............................................................................................................. 112
ESR1-202     YDPTRPFSEASMMGLLTNLADRELVHMINWAKRVPGFVDLTLHDQVHLLECAWLEILMIGLVWRSMEHPGKLLFAPNLLLDRNQGKCVEGMVEIFDMLLATSSRFRMMNL 440
ESR1-203     ..............................................................................................................  84
ESR1-207     YDPTRPFSEASMMGLLTNLADRELVHMINWAKRVPGFVDLTLHDQVHLLECAWLEILMIGLVWRSMEHPGKLLFAPNLLLDRNQGKCVEGMVEIFDMLLATSSRFRMMNL 440
ESR1-204     ..................................................................................NQGKCVEGMVEIFDMLLATSSRFRMMNL 179
ESR1-205     ...................QACRLRKCYEVGMMKGGFVDLTLHDQVHLLECAWLEILMIGLVWRSMEHPGKLLFAPNLLLDRNQGKCVEGMVEIFDMLLATSSR...... 107
ESR1-206     YDPTRPFSEASMMGLLTNLADRELVHMINWAKRVPGFVDLTLHDQVHLLECAWLEILMIGLVWRSMEHPGKLLFAPNLLLDRNQGKCVEGMVEIFDMLLATSSRFRMMNL 267
ESR1-210     .............................................................................................................. 152
ESR1-201     YDPTRPFSEASMMGLLTNLADRELVHMINWAKRVPGFVDLTLHDQVHLLECAWLEILMIGLVWRSMEHPGKLLFAPNLLLDRNQGKCVEGMVEIFDMLLATSSRFRMMNL 440
ESR1-208     YDPTRPFSEASMMGLLTNLADRELVHMINWAKRVPGFVDLTLHDQVHLLECAWLEILMIGLVWRSMEHPGKLLFAPNLLLDRNQGKCVEGMVEIFDMLLATSSRFRMMNL 440
ESR1-209     .............................................................................................................. 115
```

```
logo
            QGEEFVCLKSIILLNSGVYTFLSSTLKSLEEKDHIHRVLDKITDTLIHLMAKAGLTLQQQHQRLAQLLLILSHIRHMSNKGMEHLYSMKCKNVVPLYDLLLEMLDAHRLH
GENISTEIN   ..............................................................................................................   8
PF02159.3   ..............................................................................................................  74
PF00104.3   ..............................................................................................................  59
PF00105.1   ..............................................................................................................  36
PF02159.2   ..............................................................................................................  43
PF00104.2   QGEEFVCLKSIILLNS..............................................................................................  121
PF02159.4   ..............................................................................................................  110
PF12743     ..............................................................................................................   0
PF02159     ..............................................................................................................  140
PF00104.1   QGEEFVCLKSIILLNSGVYTFLSSTLKSLEEKDHIHRVLDKITDTLIHLMAKAGLTLQQQHQRLAQLLLILSHIRHMSNKGMEHLYSMKC....................  118
PF00105     ..............................................................................................................  69
PF00104     QGEEFVCLKSIILLNSGVYTFLSSTLKSLEEKDHIHRVLDKITDTLIHLMAKAGLTLQQQHQRLAQLLLILSHIRHMSNKGMEHLYSMKC....................  195
PF02159.1   ..............................................................................................................  112
ESR1-202    QGEEFVCLKSIILLNSGVYTFLSSTLKSLEEKDHIHRVLDKITDTLIHLMAKAGLTLQQQHQRLAQLLLILSHIRHMSNKGMEHLYSMKCKNVVPLYDLLLEMLDAHRLH  550
ESR1-203    ..............................................................................................................  84
ESR1-207    QGEEFVCLKSIILLNSGVYTFLSSTLKSLEEKDHIHRVLDKITDTLIHLMAKAGLTLQQQHQRLAQLLLILSHIRHMSNKGMEHLYSMKCKNVVPLYDLLLEMLDAHRLH  550
ESR1-204    QGEEFVCLKSIILLNSGVYTFLSSTLKSLEEKDHIHRVLDKITDTLIHLMAKAGLTLQQQHQRLAQLLLILSHIRHMSNKGMEHLYSMKCKNVVPLYDLLLEMLDAHRLH  289
ESR1-205    ..............................................................................................................  107
ESR1-206    QGEEFVCLKSIILLNSGISHVEAK........KRILNLHPKIFGNKWF..PRV.........................................................  310
ESR1-210    ..............................................................................................................  152
ESR1-201    QGEEFVCLKSIILLNSGVYTFLSSTLKSLEEKDHIHRVLDKITDTLIHLMAKAGLTLQQQHQRLAQLLLILSHIRHMSNKGMEHLYSMKCKNVVPLYDLLLEMLDAHRLH  550
ESR1-208    QGEEFVCLKSIILLNSGVYTFLSSTLKSLEEKDHIHRVLDKITDTLIHLMAKAGLTLQQQHQRLAQLLLILSHIRHMSNKGMEHLYSMKCKNVVPLYDLLLEMLDAHRLH  550
ESR1-209    ..............................................................................................................  115

logo
            APTSRGGASVEETDQSHLATAGSTSSHSLQKYYITGEAEGFPATV
GENISTEIN   ................HL...........................   10
PF02159.3   .............................................   74
PF00104.3   .............................................   59
PF00105.1   .............................................   36
PF02159.2   .............................................   43
PF00104.2   .............................................  121
PF02159.4   .............................................  110
PF12743     .PTSRGGASVEETDQSHLATAGSTSSHSLQKYYITGEAEGFPATV   44
PF02159     .............................................  140
PF00104.1   .............................................  118
PF00105     .............................................   69
PF00104     .............................................  195
PF02159.1   .............................................  112
ESR1-202    APTSRGGASVEETDQSHLATAGSTSSHSLQKYYITGEAEGFPATV  595
ESR1-203    .............................................   84
ESR1-207    APTSRGGASVEETDQSHLATAGSTSSHSLQKYYITGEAEGFPATV  595
ESR1-204    APTSRGGASVEETDQSHLATAGSTSSHSLQKYYITGEAEGFPATV  334
ESR1-205    .............................................  107
ESR1-206    .............................................  310
ESR1-210    .............................................  152
ESR1-201    APTSRGGASVEETDQSHLATAGSTSSHSLQKYYITGEAEGFPATV  595
ESR1-208    APTSRGGASVEETDQSHLATAGSTSSHSLQKYYITGEAEGFPATV  595
ESR1-209    .............................................  115
```

X non conserved
X similar
X $\geq 0\%$ conserved
X $\geq 50\%$ conserved

```
logo                    MDIKNSPSSLNSPSSYNCSQSILPLEHGSIYIPSSYVDSHHEYPAMTFYSPAVMNYSIPSNVTNLEGGPGRQTTSPNVLWPTPGHLSPLVVHRQLSHLYA
AFIMOXIFENE (CHEMBL489) ....................................................................................................   0
PF00104                 ....................................................................................................   0
PF00104.4               ....................................................................................................   0
PF00104.1               ....................................................................................................   0
PF12497                 ...........SPSSYNCSQSILPLEHGSIYIPSSYVDSHHEYPAMTFYSPAVMNYSIPSNVTNLEGGPGRQTTSPNVLWPTPGHLSPLVVHRQLSHLYA  89
PF00104.2               ....................................................................................................   0
PF00105                 ....................................................................................................   0
PF00104.3               ....................................................................................................   0
ESR2-203                MDIKNSPSSLNSPSSYNCSQSILPLEHGSIYIPSSYVDSHHEYPAMTFYSPAVMNYSIPSNVTNLEGGPGRQTTSPNVLWPTPGHLSPLVVHRQLSHLYA 100
ESR2-204                MDIKNSPSSLNSPSSYNCSQSILPLEHGSIYIPSSYVDSHHEYPAMTFYSPAVMNYSIPSNVTNLEGGPGRQTTSPNVLWPTPGHLSPLVVHRQLSHLYA 100
ESR2-209                MDIKNSPSSLNSPSSYNCSQSILPLEHGSIYIPSSYVDSHHEYPAMTFYSPAVMNYSIPSNVTNLEGGPGRQTTSPNVLWPTPGHLSPLVVHRQLSHLYA 100
ESR2-212                MDIKNSPSSLNSPSSYNCSQSILPLEHGSIYIPSSYVDSHHEYPAMTFYSPAVMNYSIPSNVTNLEGGPGRQTTSPNVLWPTPGHLSPLVVHRQLSHLYA 100
ESR2-205                MDIKNSPSSLNSPSSYNCSQSILPLEHGSIYIPSSYVDSHHEYPAMTFYSPAVMNYSIPSNVTNLEGGPGRQTTSPNVLWPTPGHLSPLVVHRQLSHLYA 100
ESR2-201                MDIKNSPSSLNSPSSYNCSQSILPLEHGSIYIPSSYVDSHHEYPAMTFYSPAVMNYSIPSNVTNLEGGPGRQTTSPNVLWPTPGHLSPLVVHRQLSHLYA 100
ESR2-202                MDIKNSPSSLNSPSSYNCSQSILPLEHGSIYIPSSYVDSHHEYPAMTFYSPAVMNYSIPSNVTNLEGGPGRQTTSPNVLWPTPGHLSPLVVHRQLSHLYA 100
ESR2-206                MDIKNSPSSLNSPSSYNCSQSILPLEHGSIYIPSSYVDSHHEYPAMTFYSPAVMNYSIPSNVTNLEGGPGRQTTSPNVLWPTPGHLSPLVVHRQLSHLYA 100
ESR2-208                MDIKNSPSSLNSPSSYNCSQSILPLEHGSIYIPSSYVDSHHEYPAMTFYSPAVMNYSIPSNVTNLEGGPGRQTTSPNVLWPTPGHLSPLVVHRQLSHLYA 100
ESR2-213                MDIKNSPSSLNSPSSYNCSQSILPLEHGSIYIPSSYVDSHHEYPAMTFYSPAVMNYSIPSNVTNLEGGPGRQTTSPNVLWPTPGHLSPLVVHRQLSHLYA 100
```

```
logo                    EPQKSPWCEARSLEHTLPVNRETLKRKVSGNRCASPVTGPGSKRDAHFCAVCSDYASGYHYGVWSCEGCKAFFKRSIQGHNDYICPATNQCTIDKNRRKS
AFIMOXIFENE (CHEMBL489) ....................................................................................................   0
PF00104                 ....................................................................................................   0
PF00104.4               ....................................................................................................   0
PF00104.1               ....................................................................................................   0
PF12497                 EPQKSPWCEARSLEHTLPVNRETLK........................................................................... 114
PF00104.2               ....................................................................................................   0
PF00105                 ...............................................FCAVCSDYASGYHYGVWSCEGCKAFFKRSIQGHNDYICPATNQCTIDKNRRKS  53
PF00104.3               ....................................................................................................   0
ESR2-203                EPQKSPWCEARSLEHTLPVNRETLKRKVSGNRCASPVTGPGSKRDAHFCAVCSDYASGYHYGVWSCEGCKAFFKRSIQGHNDYICPATNQCTIDKNRRKS 200
ESR2-204                EPQKSPWCEARSLEHTLPVNRETLKRKVSGNRCASPVTGPGSKRDAHFCAVCSDYASGYHYGVWSCEGCKAFFKRSIQGHNDYICPATNQCTIDKNRRKS 200
ESR2-209                EPQKSPWCEARSLEHTLPVNRETLKRKVSGNRCASPVTGPGSKRDAHFCAVCSDYASGYHYGVWSCEGCKAFFKRSIQGHNDYICPATNQCTIDKNRRKS 200
ESR2-212                EPQKSPWCEARSLEHTLPVNRETLKRKVSGNRCASPVTGPGSKRDAHFCAVCSDYASGYHYGVWSCEGCKAFFKRSIQGHNDYICPATNQCTIDKNRRKS 200
ESR2-205                EPQKSPWCEARSLEHTLPVNRETLKRKVSGNRCASPVTGPGSKRDAHFCAVCSDYASGYHYGVWSCEGCKAFFKRSIQGHNDYICPATNQCTIDKNRRKS 200
ESR2-201                EPQKSPWCEARSLEHTLPVNRETLKRKVSGNRCASPVTGPGSKRDAHFCAVCSDYASGYHYGVWSCEGCKAFFKRSIQGHNDYICPATNQCTIDKNRRKS 200
ESR2-202                EPQKSPWCEARSLEHTLPVNRETLKRKVSGNRCASPVTGPGSKRDAHFCAVCSDYASGYHYGVWSCEGCKAFFKRSIQGHNDYICPATNQCTIDKNRRKS 200
ESR2-206                EPQKSPWCEARSLEHTLPVNRETLKRKVSGNRCASPVTGPGSKRDAHFCAVCSDYASGYHYGVWSCEGCKAFFKRSIQGHNDYICPATNQCTIDKNRRKS 200
ESR2-208                EPQKSPWCEARSLEHTLPVNRETLKRKVSGNRCASPVTGPGSKRDAHFCAVCSDYASGYHYGVWSCEGCKAFFKRSIQGHNDYICPATNQCTIDKNRRKS 200
ESR2-213                EPQKSPWCEARSLEHTLPVNRETLKRKVSGNRCASPVTGPGSKRDAHFCAVCSDYASGYHYGVWSCEGCKAFFKRSIQGHNDYICPATNQCTIDKNRRKS 200
```

logo

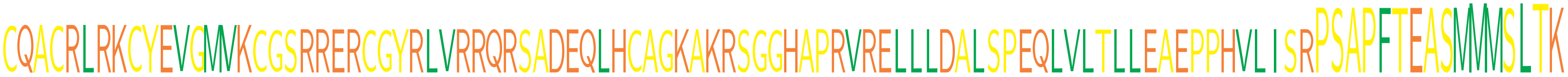

```
AFIMOXIFENE (CHEMBL489)  ..............................................................................................M..LT.    3
PF00104                  ....................................................................................PSAPFTEASMMMSLTK   16
PF00104.4                ....................................................................................PSAPFTEASMMMSLTK   16
PF00104.1                ....................................................................................PSAPFTEASMMMSLTK   16
PF12497                  ....................................................................................................  114
PF00104.2                ....................................................................................PSAPFTEASMMMSLTK   16
PF00105                  CQACRLRKCYEVGMVK....................................................................................   69
PF00104.3                ....................................................................................PSAPFTEASMMMSLTK   16
ESR2-203                 CQACRLRKCYEVGMVKCGSRRERCGYRLVRRQRSADEQLHCAGKAKRSGGHAPRVRELLLDALSPEQLVLTLLEAEPPHVLISRPSAPFTEASMMMSLTK  300
ESR2-204                 CQACRLRKCYEVGMVKCGSRRERCGYRLVRRQRSADEQLHCAGKAKRSGGHAPRVRELLLDALSPEQLVLTLLEAEPPHVLISRPSAPFTEASMMMSLTK  300
ESR2-209                 CQACRLRKCYEVGMVKCGSRRERCGYRLVRRQRSADEQLHCAGKAKRSGGHAPRVRELLLDALSPEQLVLTLLEAEPPHVLISRPSAPFTEASMMMSLTK  300
ESR2-212                 CQACRLRKCYEVGMVKCGSRRERCGYRLVRRQRSADEQLHCAGKAKRSGGHAPRVRELLLDALSPEQLVLTLLEAEPPHVLISRPSAPFTEASMMMSLTK  300
ESR2-205                 CQACRLRKCYEVGMVKCGSRRERCGYRLVRRQRSADEQLHCAGKAKRSGGHAPRVRELLLDALSPEQLVLTLLEAEPPHVLISRPSAPFTEASMMMSLTK  300
ESR2-201                 CQACRLRKCYEVGMVKCGSRRERCGYRLVRRQRSADEQLHCAGKAKRSGGHAPRVRELLLDALSPEQLVLTLLEAEPPHVLISRPSAPFTEASMMMSLTK  300
ESR2-202                 CQACRLRKCYEVGMVKCGSRRERCGYRLVRRQRSADEQLHCAGKAKRSGGHAPRVRELLLDALSPEQLVLTLLEAEPPHVLISRPSAPFTEASMMMSLTK  300
ESR2-206                 CQACRLRKCYEVGMVKCGSRRERCGYRLVRRQRSADEQLHCAGKAKRSGGHAPRVRELLLDALSPEQLVLTLLEAEPPHVLISRPSAPFTEASMMMSLTK  300
ESR2-208                 CQACRLRKCYEVGMVKCGSRRERCGYRLVRRQRSADEQLHCAGKAKRSGGHAPRVRELLLDALSPEQLVLTLLEAEPPHVLISRPSAPFTEASMMMSLTK  300
ESR2-213                 CQACRLRKCYEVGMVKCGSRRERCGYRLVRRQRSADEQLHCAGKAKRSGGHAPRVRELLLDALSPEQLVLTLLEAEPPHVLISRPSAPFTEASMMMSLTK  300
```

logo

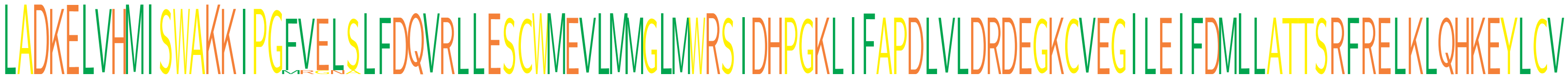

```
AFIMOXIFENE (CHEMBL489)  LAD.EL..MI....K........L..QV..LE..WM..LM..L..R.........F................I..I...L....................   26
PF00104                  LADKELVHMISWAKKIPGFVELSLFDQVRLLESCWMEVLMMGLMWRSIDHPGKLIFAPDLVLDRDEGKCVEGILEIFDMLLATTSRFRELKLQHKEYLCV  116
PF00104.4                LADKELVHMISWAKKIPGFVELSLFDQVRLLESCWMEVLMMGLMWRSIDHPGKLIFAPDLVLDRDEGKCVEGILEIFDMLLATTSRFRELKLQHKEYLCV  116
PF00104.1                LADKELVHMISWAKKIPGFVELSLFDQVRLLESCWMEVLMMGLMWRSIDHPGKLIFAPDLVLDRDEGKCVEGILEIFDMLLATTSRFRELKLQHKEYLCV  116
PF12497                  ....................................................................................................  114
PF00104.2                LADKELVHMISWAKKIPGFVELSLFDQVRLLESCWMEVLMMGLMWRSIDHPGKLIFAPDLVLDRDEGKCVEGILEIFDMLLATTSRFRELKLQHKEYLCV  116
PF00105                  ....................................................................................................   69
PF00104.3                LADKELVHMISWAKKIPGFVELSLFDQVRLLESCWMEVLMMGLMWRSIDHPGKLIFAPDLVLDRDEGKCVEGILEIFDMLLATTSRFRELKLQHKEYLCV  116
ESR2-203                 LADKELVHMISWAKKIPGMRGNA.............................................................................  323
ESR2-204                 LADKELVHMISWAKKIPGFVELSLFDQVRLLESCWMEVLMMGLMWRSIDHPGKLIFAPDLVLDRDEGKCVEGILEIFDMLLATTSRFRELKLQHKEYLCV  400
ESR2-209                 LADKELVHMISWAKKIPGFVELSLFDQVRLLESCWMEVLMMGLMWRSIDHPGKLIFAPDLVLDRDEGKCVEGILEIFDMLLATTSRFRELKLQHKEYLCV  400
ESR2-212                 LADKELVHMISWAKKIPGFVELSLFDQVRLLESCWMEVLMMGLMWRSIDHPGKLIFAPDLVLDRDEGKCVEGILEIFDMLLATTSRFRELKLQHKEYLCV  400
ESR2-205                 LADKELVHMISWAKKIPGFVELSLFDQVRLLESCWMEVLMMGLMWRSIDHPGKLIFAPDLVLDRDEGKCVEGILEIFDMLLATTSRFRELKLQHKEYLCV  400
ESR2-201                 LADKELVHMISWAKKIPG..................................................................................  318
ESR2-202                 LADKELVHMISWAKKIPGFVELSLFDQVRLLESCWMEVLMMGLMWRSIDHPGKLIFAPDLVLDRDEGKCVEGILEIFDMLLATTSRFRELKLQHKEYLCV  400
ESR2-206                 LADKELVHMISWAKKIPGFVELSLFDQVRLLESCWMEVLMMGLMWRSIDHPGKLIFAPDLVLDRDEGKCVEGILEIFDMLLATTSRFRELKLQHKEYLCV  400
ESR2-208                 LADKELVHMISWAKKIPGFVELSLFDQVRLLESCWMEVLMMGLMWRSIDHPGKLIFAPDLVLDRDEGKCVEGILEIFDMLLATTSRFRELKLQHKEYLCV  400
ESR2-213                 LADKELVHMISWAKKIPGFVELSLFDQVRLLESCWMEVLMMGLMWRSIDHPGKLIFAPDLVLDRDEGKCVEGILEIFDMLLATTSRFRELKLQHKEYLCV  400
```

logo

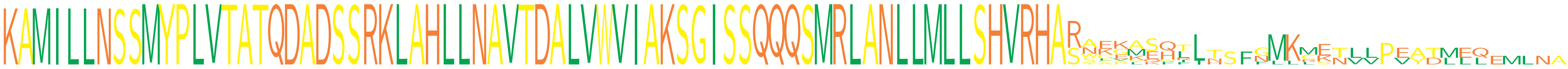

```
AFIMOXIFENE (CHEMBL489)  ....................................................................................................  26
PF00104                  KAMILLNSSMYPLVTATQDADSSRKLAHLLNAVTDALVWVIAKSGISSQQQSMRLANLLMLLSHVRHARAEKAS..........................  190
PF00104.4                KAMILLNSSMYPLVTATQDADSSRKLAHLLNAVTDALVWVIAKSGISSQQQSMRLANLLMLLSHVRHAS...............................  185
PF00104.1                KAMILLNSSMYPLVTATQDADSSRKLAHLLNAVTDALVWVIAKSGISSQQQSMRLANLLMLLSHVRHASNKGMEHLL...NMK.................  196
PF12497                  ....................................................................................................  114
PF00104.2                KAMILLNSSMYPLVTATQDADSSRKLAHLLNAVTDALVWVIAKSGISSQQQSMRLANLLMLLSHVRHARWGEKQFI........................  192
PF00105                  ....................................................................................................  69
PF00104.3                KAMILLNSSMYPLVTATQDADSSRKLAHLLNAVTDALVWVIAKSGISSQQQSMRLANLLMLLSHVRHA................................  184
ESR2-203                 ....................................................................................................  323
ESR2-204                 KAMILLNSSMYPLVTATQDADSSRKLAHLLNAVTDALVWVIAKSGISSQQQSMRLANLLMLLSHVRHARAEKASQTLTSFGMKMETLLPEATMEQ.....  495
ESR2-209                 KAMILLNSSMYPLVTATQDADSSRKLAHLLNAVTDALVWVIAKSGISSQQQSMRLANLLMLLSHVRHARWGEK.QFIH...LKLS...............  481
ESR2-212                 KAMILLNSSMYPLVTATQDADSSRKLAHLLNAVTDALVWVIAKSGISSQQQSMRLANLLMLLSHVRHASSLSLSWRL...FMLRE...............  482
ESR2-205                 KAMILLNSSMYPLVTATQDADSSRKLAHLLNAVTDALVWVIAKSGISSQQQSMRLANLLMLLSHVRHARAEKASQTLTSFGMKMETLLPEATMEQ.....  495
ESR2-201                 .........MYPLVTATQDADSSRKLAHLLNAVTDALVWVIAKSGISSQQQSMRLANLLMLLSHVRHASNKGMEHLLN...MKCKNVVPVYDLLLEMLNA  406
ESR2-202                 KAMILLNSSMYPLVTATQDADSSRKLAHLLNAVTDALVWVIAKSGISSQQQSMRLANLLMLLSHVRHASNKGMEHLL...NMKCKNVVPVYDLLLEMLNA  497
ESR2-206                 KAMILLNSSMYPLVTATQDADSSRKLAHLLNAVTDALVWVIAKSGISSQQQSMRLANLLMLLSHVRHARSCVYK..........................  474
ESR2-208                 KAMILLNSSMYPLVTATQDADSSRKLAHLLNAVTDALVWVIAKSGISSQQQSMRLANLLMLLSHVRHARAEKASQTLTSFGMKMETLLPEATMEQ.....  495
ESR2-213                 KAMILLNSSMYPLVTATQDADSSRKLAHLLNAVTDALVWVIAKSGISSQQQSMRLANLLMLLSHVRHARYAP............................  472
```

logo

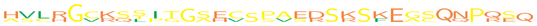

```
AFIMOXIFENE (CHEMBL489)  ....G...L........................  28
PF00104                  .................................  190
PF00104.4                .................................  185
PF00104.1                .................................  196
PF12497                  .................................  114
PF00104.2                .................................  192
PF00105                  .................................  69
PF00104.3                .................................  184
ESR2-203                 .................................  323
ESR2-204                 .................................  495
ESR2-209                 .................................  481
ESR2-212                 ASCHGVRQTPGGAHV.SVSRSRSFEACQQPRE.  513
ESR2-205                 .................................  495
ESR2-201                 HVLRGCKSSITGSECSPAEDSKSKEGSQNPQSQ  439
ESR2-202                 HVLRGCKSSITGSECSPAEDSKSKEGSQNPQSQ  530
ESR2-206                 .................................  474
ESR2-208                 .................................  495
ESR2-213                 .................................  472
```

- X non conserved
- X similar
- X $\geq 0\%$ conserved
- X $\geq 50\%$ conserved

```
logo        MDIKNSPSSLNSPSSYNCSQSILPLEHGSIYIPSSYVDSHHEYPAMTFYSPAVMNYSIPSNVTNLEGGPGRQTTSPNVLWPTPGHLSPLVVHRQLSHLYAEPQKSPWCEA
GENISTEIN   ..............................................................................................................    0
PF00104     ..............................................................................................................    0
PF00104.4   ..............................................................................................................    0
PF00104.1   ..............................................................................................................    0
PF12497     ...........SPSSYNCSQSILPLEHGSIYIPSSYVDSHHEYPAMTFYSPAVMNYSIPSNVTNLEGGPGRQTTSPNVLWPTPGHLSPLVVHRQLSHLYAEPQKSPWCEA   99
PF00104.2   ..............................................................................................................    0
PF00105     ..............................................................................................................    0
PF00104.3   ..............................................................................................................    0
ESR2-203    MDIKNSPSSLNSPSSYNCSQSILPLEHGSIYIPSSYVDSHHEYPAMTFYSPAVMNYSIPSNVTNLEGGPGRQTTSPNVLWPTPGHLSPLVVHRQLSHLYAEPQKSPWCEA  110
ESR2-204    MDIKNSPSSLNSPSSYNCSQSILPLEHGSIYIPSSYVDSHHEYPAMTFYSPAVMNYSIPSNVTNLEGGPGRQTTSPNVLWPTPGHLSPLVVHRQLSHLYAEPQKSPWCEA  110
ESR2-209    MDIKNSPSSLNSPSSYNCSQSILPLEHGSIYIPSSYVDSHHEYPAMTFYSPAVMNYSIPSNVTNLEGGPGRQTTSPNVLWPTPGHLSPLVVHRQLSHLYAEPQKSPWCEA  110
ESR2-212    MDIKNSPSSLNSPSSYNCSQSILPLEHGSIYIPSSYVDSHHEYPAMTFYSPAVMNYSIPSNVTNLEGGPGRQTTSPNVLWPTPGHLSPLVVHRQLSHLYAEPQKSPWCEA  110
ESR2-205    MDIKNSPSSLNSPSSYNCSQSILPLEHGSIYIPSSYVDSHHEYPAMTFYSPAVMNYSIPSNVTNLEGGPGRQTTSPNVLWPTPGHLSPLVVHRQLSHLYAEPQKSPWCEA  110
ESR2-201    MDIKNSPSSLNSPSSYNCSQSILPLEHGSIYIPSSYVDSHHEYPAMTFYSPAVMNYSIPSNVTNLEGGPGRQTTSPNVLWPTPGHLSPLVVHRQLSHLYAEPQKSPWCEA  110
ESR2-202    MDIKNSPSSLNSPSSYNCSQSILPLEHGSIYIPSSYVDSHHEYPAMTFYSPAVMNYSIPSNVTNLEGGPGRQTTSPNVLWPTPGHLSPLVVHRQLSHLYAEPQKSPWCEA  110
ESR2-206    MDIKNSPSSLNSPSSYNCSQSILPLEHGSIYIPSSYVDSHHEYPAMTFYSPAVMNYSIPSNVTNLEGGPGRQTTSPNVLWPTPGHLSPLVVHRQLSHLYAEPQKSPWCEA  110
ESR2-208    MDIKNSPSSLNSPSSYNCSQSILPLEHGSIYIPSSYVDSHHEYPAMTFYSPAVMNYSIPSNVTNLEGGPGRQTTSPNVLWPTPGHLSPLVVHRQLSHLYAEPQKSPWCEA  110
ESR2-213    MDIKNSPSSLNSPSSYNCSQSILPLEHGSIYIPSSYVDSHHEYPAMTFYSPAVMNYSIPSNVTNLEGGPGRQTTSPNVLWPTPGHLSPLVVHRQLSHLYAEPQKSPWCEA  110

logo        RSLEHTLPVNRETLKRKVSGNRCASPVTGPGSKRDAHFCAVCSDYASGYHYGVWSCEGCKAFFKRSIQGHNDYICPATNQCTIDKNRRKSCQACRLRKCYEVGMVKCGSR
GENISTEIN   ..............................................................................................................    0
PF00104     ..............................................................................................................    0
PF00104.4   ..............................................................................................................    0
PF00104.1   ..............................................................................................................    0
PF12497     RSLEHTLPVNRETLK...............................................................................................  114
PF00104.2   ..............................................................................................................    0
PF00105     .....................................FCAVCSDYASGYHYGVWSCEGCKAFFKRSIQGHNDYICPATNQCTIDKNRRKSCQACRLRKCYEVGMVK....   69
PF00104.3   ..............................................................................................................    0
ESR2-203    RSLEHTLPVNRETLKRKVSGNRCASPVTGPGSKRDAHFCAVCSDYASGYHYGVWSCEGCKAFFKRSIQGHNDYICPATNQCTIDKNRRKSCQACRLRKCYEVGMVKCGSR  220
ESR2-204    RSLEHTLPVNRETLKRKVSGNRCASPVTGPGSKRDAHFCAVCSDYASGYHYGVWSCEGCKAFFKRSIQGHNDYICPATNQCTIDKNRRKSCQACRLRKCYEVGMVKCGSR  220
ESR2-209    RSLEHTLPVNRETLKRKVSGNRCASPVTGPGSKRDAHFCAVCSDYASGYHYGVWSCEGCKAFFKRSIQGHNDYICPATNQCTIDKNRRKSCQACRLRKCYEVGMVKCGSR  220
ESR2-212    RSLEHTLPVNRETLKRKVSGNRCASPVTGPGSKRDAHFCAVCSDYASGYHYGVWSCEGCKAFFKRSIQGHNDYICPATNQCTIDKNRRKSCQACRLRKCYEVGMVKCGSR  220
ESR2-205    RSLEHTLPVNRETLKRKVSGNRCASPVTGPGSKRDAHFCAVCSDYASGYHYGVWSCEGCKAFFKRSIQGHNDYICPATNQCTIDKNRRKSCQACRLRKCYEVGMVKCGSR  220
ESR2-201    RSLEHTLPVNRETLKRKVSGNRCASPVTGPGSKRDAHFCAVCSDYASGYHYGVWSCEGCKAFFKRSIQGHNDYICPATNQCTIDKNRRKSCQACRLRKCYEVGMVKCGSR  220
ESR2-202    RSLEHTLPVNRETLKRKVSGNRCASPVTGPGSKRDAHFCAVCSDYASGYHYGVWSCEGCKAFFKRSIQGHNDYICPATNQCTIDKNRRKSCQACRLRKCYEVGMVKCGSR  220
ESR2-206    RSLEHTLPVNRETLKRKVSGNRCASPVTGPGSKRDAHFCAVCSDYASGYHYGVWSCEGCKAFFKRSIQGHNDYICPATNQCTIDKNRRKSCQACRLRKCYEVGMVKCGSR  220
ESR2-208    RSLEHTLPVNRETLKRKVSGNRCASPVTGPGSKRDAHFCAVCSDYASGYHYGVWSCEGCKAFFKRSIQGHNDYICPATNQCTIDKNRRKSCQACRLRKCYEVGMVKCGSR  220
ESR2-213    RSLEHTLPVNRETLKRKVSGNRCASPVTGPGSKRDAHFCAVCSDYASGYHYGVWSCEGCKAFFKRSIQGHNDYICPATNQCTIDKNRRKSCQACRLRKCYEVGMVKCGSR  220
```

```
logo        RERCGYRLVRRQRSADEQLHCAGKAKRSGGHAPRVRELLLDALSPEQLVLTLLEAEPPHVLISRPSAPFTEASMMMSLTKLADKELVHMISWAKKIPGFVELSLFDQVRL
GENISTEIN   ..........................................................................M..L..L...E.........................   4
PF00104     ................................................................PSAPFTEASMMMSLTKLADKELVHMISWAKKIPGFVELSLFDQVRL  46
PF00104.4   ................................................................PSAPFTEASMMMSLTKLADKELVHMISWAKKIPGFVELSLFDQVRL  46
PF00104.1   ................................................................PSAPFTEASMMMSLTKLADKELVHMISWAKKIPGFVELSLFDQVRL  46
PF12497     .............................................................................................................. 114
PF00104.2   ................................................................PSAPFTEASMMMSLTKLADKELVHMISWAKKIPGFVELSLFDQVRL  46
PF00105     ..............................................................................................................  69
PF00104.3   ................................................................PSAPFTEASMMMSLTKLADKELVHMISWAKKIPGFVELSLFDQVRL  46
ESR2-203    RERCGYRLVRRQRSADEQLHCAGKAKRSGGHAPRVRELLLDALSPEQLVLTLLEAEPPHVLISRPSAPFTEASMMMSLTKLADKELVHMISWAKKIPGMRGNA....... 323
ESR2-204    RERCGYRLVRRQRSADEQLHCAGKAKRSGGHAPRVRELLLDALSPEQLVLTLLEAEPPHVLISRPSAPFTEASMMMSLTKLADKELVHMISWAKKIPGFVELSLFDQVRL 330
ESR2-209    RERCGYRLVRRQRSADEQLHCAGKAKRSGGHAPRVRELLLDALSPEQLVLTLLEAEPPHVLISRPSAPFTEASMMMSLTKLADKELVHMISWAKKIPGFVELSLFDQVRL 330
ESR2-212    RERCGYRLVRRQRSADEQLHCAGKAKRSGGHAPRVRELLLDALSPEQLVLTLLEAEPPHVLISRPSAPFTEASMMMSLTKLADKELVHMISWAKKIPGFVELSLFDQVRL 330
ESR2-205    RERCGYRLVRRQRSADEQLHCAGKAKRSGGHAPRVRELLLDALSPEQLVLTLLEAEPPHVLISRPSAPFTEASMMMSLTKLADKELVHMISWAKKIPGFVELSLFDQVRL 330
ESR2-201    RERCGYRLVRRQRSADEQLHCAGKAKRSGGHAPRVRELLLDALSPEQLVLTLLEAEPPHVLISRPSAPFTEASMMMSLTKLADKELVHMISWAKKIPG............ 318
ESR2-202    RERCGYRLVRRQRSADEQLHCAGKAKRSGGHAPRVRELLLDALSPEQLVLTLLEAEPPHVLISRPSAPFTEASMMMSLTKLADKELVHMISWAKKIPGFVELSLFDQVRL 330
ESR2-206    RERCGYRLVRRQRSADEQLHCAGKAKRSGGHAPRVRELLLDALSPEQLVLTLLEAEPPHVLISRPSAPFTEASMMMSLTKLADKELVHMISWAKKIPGFVELSLFDQVRL 330
ESR2-208    RERCGYRLVRRQRSADEQLHCAGKAKRSGGHAPRVRELLLDALSPEQLVLTLLEAEPPHVLISRPSAPFTEASMMMSLTKLADKELVHMISWAKKIPGFVELSLFDQVRL 330
ESR2-213    RERCGYRLVRRQRSADEQLHCAGKAKRSGGHAPRVRELLLDALSPEQLVLTLLEAEPPHVLISRPSAPFTEASMMMSLTKLADKELVHMISWAKKIPGFVELSLFDQVRL 330

logo        LESCWMEVLMMGLMWRSIDHPGKLIFAPDLVLDRDEGKCVEGILEIFDMLLATTSRFRELKLQHKEYLCVKAMILLNSSMYPLVTATQDADSSRKLAHLLNAVTDALVWV
GENISTEIN   ........L......R.........F................I..I................................................................   9
PF00104     LESCWMEVLMMGLMWRSIDHPGKLIFAPDLVLDRDEGKCVEGILEIFDMLLATTSRFRELKLQHKEYLCVKAMILLNSSMYPLVTATQDADSSRKLAHLLNAVTDALVWV 156
PF00104.4   LESCWMEVLMMGLMWRSIDHPGKLIFAPDLVLDRDEGKCVEGILEIFDMLLATTSRFRELKLQHKEYLCVKAMILLNSSMYPLVTATQDADSSRKLAHLLNAVTDALVWV 156
PF00104.1   LESCWMEVLMMGLMWRSIDHPGKLIFAPDLVLDRDEGKCVEGILEIFDMLLATTSRFRELKLQHKEYLCVKAMILLNSSMYPLVTATQDADSSRKLAHLLNAVTDALVWV 156
PF12497     .............................................................................................................. 114
PF00104.2   LESCWMEVLMMGLMWRSIDHPGKLIFAPDLVLDRDEGKCVEGILEIFDMLLATTSRFRELKLQHKEYLCVKAMILLNSSMYPLVTATQDADSSRKLAHLLNAVTDALVWV 156
PF00105     ..............................................................................................................  69
PF00104.3   LESCWMEVLMMGLMWRSIDHPGKLIFAPDLVLDRDEGKCVEGILEIFDMLLATTSRFRELKLQHKEYLCVKAMILLNSSMYPLVTATQDADSSRKLAHLLNAVTDALVWV 156
ESR2-203    .............................................................................................................. 323
ESR2-204    LESCWMEVLMMGLMWRSIDHPGKLIFAPDLVLDRDEGKCVEGILEIFDMLLATTSRFRELKLQHKEYLCVKAMILLNSSMYPLVTATQDADSSRKLAHLLNAVTDALVWV 440
ESR2-209    LESCWMEVLMMGLMWRSIDHPGKLIFAPDLVLDRDEGKCVEGILEIFDMLLATTSRFRELKLQHKEYLCVKAMILLNSSMYPLVTATQDADSSRKLAHLLNAVTDALVWV 440
ESR2-212    LESCWMEVLMMGLMWRSIDHPGKLIFAPDLVLDRDEGKCVEGILEIFDMLLATTSRFRELKLQHKEYLCVKAMILLNSSMYPLVTATQDADSSRKLAHLLNAVTDALVWV 440
ESR2-205    LESCWMEVLMMGLMWRSIDHPGKLIFAPDLVLDRDEGKCVEGILEIFDMLLATTSRFRELKLQHKEYLCVKAMILLNSSMYPLVTATQDADSSRKLAHLLNAVTDALVWV 440
ESR2-201    ...............................................................................MYPLVTATQDADSSRKLAHLLNAVTDALVWV 349
ESR2-202    LESCWMEVLMMGLMWRSIDHPGKLIFAPDLVLDRDEGKCVEGILEIFDMLLATTSRFRELKLQHKEYLCVKAMILLNSSMYPLVTATQDADSSRKLAHLLNAVTDALVWV 440
ESR2-206    LESCWMEVLMMGLMWRSIDHPGKLIFAPDLVLDRDEGKCVEGILEIFDMLLATTSRFRELKLQHKEYLCVKAMILLNSSMYPLVTATQDADSSRKLAHLLNAVTDALVWV 440
ESR2-208    LESCWMEVLMMGLMWRSIDHPGKLIFAPDLVLDRDEGKCVEGILEIFDMLLATTSRFRELKLQHKEYLCVKAMILLNSSMYPLVTATQDADSSRKLAHLLNAVTDALVWV 440
ESR2-213    LESCWMEVLMMGLMWRSIDHPGKLIFAPDLVLDRDEGKCVEGILEIFDMLLATTSRFRELKLQHKEYLCVKAMILLNSSMYPLVTATQDADSSRKLAHLLNAVTDALVWV 440
```

logo 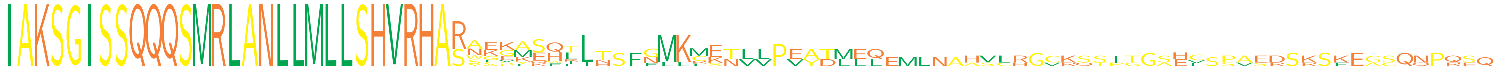

```
GENISTEIN   .........................................................................HL..................   11
PF00104     IAKSGISSQQQSMRLANLLMLLSHVRHARAEKAS...........................................................  190
PF00104.4   IAKSGISSQQQSMRLANLLMLLSHVRHAS................................................................  185
PF00104.1   IAKSGISSQQQSMRLANLLMLLSHVRHASNKGMEHLL...NMK..................................................  196
PF12497     .............................................................................................  114
PF00104.2   IAKSGISSQQQSMRLANLLMLLSHVRHARWGEKQFI.........................................................  192
PF00105     .............................................................................................   69
PF00104.3   IAKSGISSQQQSMRLANLLMLLSHVRHA.................................................................  184
ESR2-203    .............................................................................................  323
ESR2-204    IAKSGISSQQQSMRLANLLMLLSHVRHARAEKASQTLTSFGMKMETLLPEATMEQ......................................  495
ESR2-209    IAKSGISSQQQSMRLANLLMLLSHVRHARWGEK.QFIH...LKLS................................................  481
ESR2-212    IAKSGISSQQQSMRLANLLMLLSHVRHASSLSLSWRL...FMLRE...............ASCHGVRQTPGGAHV.SVSRSRSFEACQQPRE.  513
ESR2-205    IAKSGISSQQQSMRLANLLMLLSHVRHARAEKASQTLTSFGMKMETLLPEATMEQ......................................  495
ESR2-201    IAKSGISSQQQSMRLANLLMLLSHVRHASNKGMEHLLN...MKCKNVVPVYDLLLEMLNAHVLRGCKSSITGSECSPAEDSKSKEGSQNPQSQ  439
ESR2-202    IAKSGISSQQQSMRLANLLMLLSHVRHASNKGMEHLL...NMKCKNVVPVYDLLLEMLNAHVLRGCKSSITGSECSPAEDSKSKEGSQNPQSQ  530
ESR2-206    IAKSGISSQQQSMRLANLLMLLSHVRHARSCVYK...........................................................  474
ESR2-208    IAKSGISSQQQSMRLANLLMLLSHVRHARAEKASQTLTSFGMKMETLLPEATMEQ......................................  495
ESR2-213    IAKSGISSQQQSMRLANLLMLLSHVRHARYAP.............................................................  472
```

- X non conserved
- X similar
- X $\geq$ 0% conserved
- X $\geq$ 50% conserved

```
logo
```

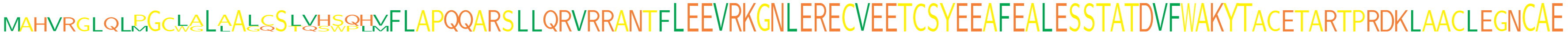

```
SURAMIN      ..............................................................................................................    0
PF00089.1    ..............................................................................................................    0
PF09396      ..............................................................................................................    0
PF09396.1    ..............................................................................................................    0
PF00051      ...........................................................................................................CAE    3
PF00594      ...............................................LEEVRKGNLERECVEETCSYEEAFEALESSTATDVFWAKYT......................   41
PF00089      ..............................................................................................................    0
F2-205       MAHVRGLQLPGCLALAALCSLVHSQHVFLAPQQARSLLQRVRRANTFLEEVRKGNLERECVEETCSYEEAFEALESSTATDVFWAKYTACETARTPRDKLAACLEGNCAE  110
F2-201       MAHVRGLQLPGCLALAALCSLVHSQHVFLAPQQARSLLQRVRRANTFLEEVRKGNLERECVEETCSYEEAFEALESSTATDVFWAKYTACETARTPRDKLAACLEGNCAE  110
F2-202       .........MG.WGLLAGQSTQSWPLMFLAPQQARSLLQRVRRANTFLEEVRKGNLERECVEETCSYEEAFEALESSTATDVFWAKYTACETARTPRDKLAACLEGNCAE  100
```

```
logo
```

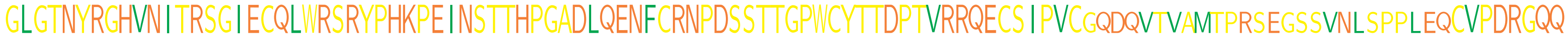

```
SURAMIN      ..............................................................................................................    0
PF00089.1    ..............................................................................................................    0
PF09396      ..............................................................................................................    0
PF09396.1    ..............................................................................................................    0
PF00051      GLGTNYRGHVNITRSGIECQLWRSRYPHKPEINSTTHPGADLQENFCRNPDSSTTGPWCYTTDPTVRRQECSIPVC..........................CVPDRGQQ   87
PF00594      ..............................................................................................................   41
PF00089      ..............................................................................................................    0
F2-205       GLGTNYRGHVNITRSGIECQLWRSRYPHKPEINSTTHPGADLQENFCRNPDSSTTGPWCYTTDPTVRRQECSIPVCGQDQVTVAMTPRSEGSSVNLSPPLEQCVPDRGQQ  220
F2-201       GLGTNYRGHVNITRSGIECQLWRSRYPHKPEINSTTHPGADLQENFCRNPDSSTTGPWCYTTDPTVRRQECSIPVCGQDQVTVAMTPRSEGSSVNLSPPLEQCVPDRGQQ  220
F2-202       GLGTNYRGHVNITRSGIECQLWRSRYPHKPEINSTTHPGADLQENFCRNPDSSTTGPWCYTTDPTVRRQECSIPVCGQDQVTVAMTPRSEGSSVNLSPPLEQCVPDRGQQ  210
```

```
logo
```

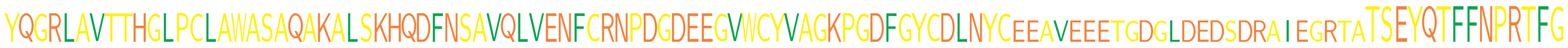

```
SURAMIN      ..............................................................................................................    0
PF00089.1    ..............................................................................................................    0
PF09396      ................................................................................................TSEYQTFFNPRTFG   14
PF09396.1    ................................................................................................TSEYQTFFNPRTFG   14
PF00051      YQGRLAVTTHGLPCLAWASAQAKALSKHQDFNSAVQLVENFCRNPDGDEEGVWCYVAGKPGDFGYCDLNYC.......................................  158
PF00594      ..............................................................................................................   41
PF00089      ..............................................................................................................    0
F2-205       YQGRLAVTTHGLPCLAWASAQAKALSKHQDFNSAVQLVENFCRNPDGDEEGVWCYVAGKPGDFGYCDLNYCEEAVEEETGDGLDEDSDRAIEGRTATSEYQTFFNPRTFG  330
F2-201       YQGRLAVTTHGLPCLAWASAQAKALSKHQDFNSAVQLVENFCRNPDGDEEGVWCYVAGKPGDFGYCDLNYCEEAVEEETGDGLDEDSDRAIEGRTATSEYQTFFNPRTFG  330
F2-202       YQGRLAVTTHGLPCLAWASAQAKALSKHQDFNSAVQLVENFCRNPDGDEEGVWCYVAGKPGDFGYCDLNYCEEAVEEETGDGLDEDSDRAIEGRTATSEYQTFFNPRTFG  320
```

```
logo
```

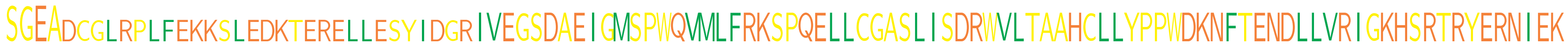

```
SURAMIN      ..............................................................................................................    0
PF00089.1    .................................IVEGSDAEIGMSPWQVMLFRKSPQELLCGASLISDRWVLTAAHCLLYPPWDKNFTENDLLVRIGKHSRTRYERNIEK   77
PF09396      SGEADCGLRPLFEKKSLEDKTERELLESYIDGR.............................................................................   47
PF09396.1    SGEA..........................................................................................................   18
PF00051      ..............................................................................................................  158
PF00594      ..............................................................................................................   41
PF00089      .................................IVEGSDAEIGMSPWQVMLFRKSPQELLCGASLISDRWVLTAAHCLLYPPWDKNFTENDLLVRIGKHSRTRYERNIEK   77
F2-205       SGEADCGLRPLFEKKSLEDKTERELLESYIDGRIVEGSDAEIGMSPWQVMLFRKSPQELLCGASLISDRWVLTAAHCLLYPPWDKNFTENDLLVRIGKHSRTRYERNIEK  440
F2-201       SGEADCGLRPLFEKKSLEDKTERELLESYIDGRIVEGSDAEIGMSPWQVMLFRKSPQELLCGASLISDRWVLTAAHCLLYPPWDKNFTENDLLVRIGKHSRTRYERNIEK  440
F2-202       SGEA..........................................................................................................  324
```

```
logo        ISMLEKIYIHPRYNWRENLRRDIALMKLKKPVAFSDYIHPVCLPDRETAASLLQAGYKGRVTGWGNLKETWTANVGKGQPSVLQVVNLPIVERPVCKDSTRIRITDNMFC
SURAMIN     .........HPR.......R..........................................................................................   4
PF00089.1   ISMLEKIYIHP.......................................SLLQAGYKGRVTGWGNLKETWTANVGKGQPSVLQVVNLPIVERPVCKDSTRIRITDNMFC 148
PF09396     ..............................................................................................................  47
PF09396.1   ..............................................................................................................  18
PF00051     .............................................................................................................. 158
PF00594     ..............................................................................................................  41
PF00089     ISMLEKIYIHPRYNWRENLDRDIALMKLKKPVAFSDYIHPVCLPDRETAASLLQAGYKGRVTGWGNLKETWTANVGKGQPSVLQVVNLPIVERPVCKDSTRIRITDNMFC 187
F2-205      ISMLEKIYIHP.......................................SLLQAGYKGRVTGWGNLKETWTANVGKGQPSVLQVVNLPIVERPVCKDSTRIRITDNMFC 511
F2-201      ISMLEKIYIHPRYNWRENLDRDIALMKLKKPVAFSDYIHPVCLPDRETAASLLQAGYKGRVTGWGNLKETWTANVGKGQPSVLQVVNLPIVERPVCKDSTRIRITDNMFC 550
F2-202      .............................................................................................................. 324

logo        AGYKPDEGKRGDACEGDSGGPFVMKSPFNNRWYQMGIVSWGEGCDRDGKYGFYTHVFRLKKWIQKVIDQFGE
SURAMIN     ............................................................KW..KV.DQ.GE  12
PF00089.1   AGYKPDEGKRGDACEGDSGGPFVMKSPFNNRWYQMGIVSWGEGCDRDGKYGFYTHVFRLKKWI......... 211
PF09396     ........................................................................  47
PF09396.1   ........................................................................  18
PF00051     ........................................................................ 158
PF00594     ........................................................................  41
PF00089     AGYKPDEGKRGDACEGDSGGPFVMKSPFNNRWYQMGIVSWGEGCDRDGKYGFYTHVFRLKKWI......... 250
F2-205      AGYKPDEGKRGDACEGDSGGPFVMKSPFNNRWYQMGIVSWGEGCDRDGKYGFYTHVFRLKKWIQKVIDQFGE 583
F2-201      AGYKPDEGKRGDACEGDSGGPFVMKSPFNNRWYQMGIVSWGEGCDRDGKYGFYTHVFRLKKWIQKVIDQFGE 622
F2-202      ........................................................................ 324
```

X non conserved
X similar
X ≥ 0% conserved
X ≥ 50% conserved

```
logo
MINODRONIC ACID  .........................................................................................................  0
PF00348.2        .........................................................................................................  0
PF00348.3        .........................................................................................................  0
PF00348          .........................................................................................................  0
PF00348.1        .........................................................................................................  0
FDPS-220         .........................................................................................................  0
FDPS-221         ..................................................................MNGDQNSDVYAQEKQDFVQHFSQIVRVLTEDEMGHPEIG  39
FDPS-203         ..................................................................MNGDQNSDVYAQEKQDFVQHFSQIVRVLTEDEMGHPEIG  39
FDPS-201         MPLSRWLRSVGVFLLPAPYWAPRERWLGSLRRPSLVHGYPVLAWHSARCWCQAWTEEPRALCSSLRMNGDQNSDVYAQEKQDFVQHFSQIVRVLTEDEMGHPEIG  105
FDPS-206         ..................................................................MNGDQNSDVYAQEKQDFVQHFSQIVRVLTEDEMGHPEIG  39
FDPS-216         MPLSRWLRSVGVFLLPAPYWAPRERWLGSLRRPSLVHGYPVLAWHSARCWCQAWTEEPRALCSSLRMNGDQNSDVYAQEKQDFVQHFSQIVRVLTEDEMGHPEIG  105
FDPS-205         MPLSRWLRSVGVFLLPAPYWAPRERWLGSLRRPSLVHGYPVLAWHSARCWCQAWTEEPRALCSSLRMNGDQNSDVYAQEKQDFVQHFSQIVRVLTEDEMGHPEIG  105
FDPS-210         ..................................................................MNGDQNSDVYAQEKQDFVQHFSQIVRVLTEDEMGHPEIG  39
FDPS-202         MPLSRWLRSVGVFLLPAPYWAPRERWLGSLRRPSLVHGYPVLAWHSARCWCQAWTEEPRALCSSLRMNGDQNSDVYAQEKQDFVQHFSQIVRVLTEDEMGHPEIG  105

logo
MINODRONIC ACID  ...........................................................FL..D...D....R................................  5
PF00348.2        ..................................................................MDSSLTRRGQICWYQKPGVGLDAINDANLLEACIYRLLK  39
PF00348.3        ....RLKEVLEYNAIGGKYNRGLTVVVAFRELVEPRKQDADSLQRAWTVGWCVELLQAFFLVADDIMDS....................................  65
PF00348          ....RLKEVLEYNAIGGKYNRGLTVVVAFRELVEPRKQDADSLQRAWTVGWCVELLQAFFLVADDIMDSSLTRRGQICWYQKPGVGLDAINDANLLEACIYRLLK  101
PF00348.1        ....RLKEVLEYNAIGGKYNRGLTVVVAFRELVEPRKQDADSLQRAWTVGWCVELLQAFFLVADDIMDSSLTRRGQICWYQKPGVGLDAINDANLLEACIYRLLK  101
FDPS-220         ..................................................................MDSSLTRRGQICWYQKPGVGLDAINDANLLEACIYRLLK  39
FDPS-221         DAIARLKEVLEYNAIGGKYNRGLTVVVAFRELVEPRKQDADSLQRAWTVGWCVELLQAFFLVADDIMDSSLTRRGQICWYQKPGVGLDAINDANLLEACIYRLLK  144
FDPS-203         DAIARLKEVLEYNAIGGKYNRGLTVVVAFRELVEPRKQDADSLQRAWTVGWCVELLQAFFLVADDIMDSSLTRRGQICWYQKPGVGLDAINDANLLEACIYRLLK  144
FDPS-201         DAIARLKEVLEYNAIGGKYNRGLTVVVAFRELVEPRKQDADSLQRAWTVGWCVELLQAFFLVADDIMDSSLTRRGQICWYQKPGVGLDAINDANLLEACIYRLLK  210
FDPS-206         DAIARLKEVLEYNAIGGKYNRGLTVVVAFRELVEPRKQDADSLQRAWTVGWCVELLQAFFLVADDIMDSSLTRRGQICWYQKPGVGLDAINDANLLEACIYRLLK  144
FDPS-216         DAIARLKEVLEYNAIGGKYNRGLTVVVAFRELVEPRKQDADSLQRAWTVGWCVELLQAFFLVADDIMDSSLTRRGQICWYQKPGVGLDAINDANLLEACIYRLLK  210
FDPS-205         DAIARLKEVLEYNAIGGKYNRGLTVVVAFRELVEPRKQDADSLQRAWTVGWCVELLQAFFLVADDIMDS....................................  174
FDPS-210         DAIARLKESLALSPG..LECDGVISACCNLHLLGSSDSPAS....AF..........................................................  80
FDPS-202         DAIARLKEVLEYNAIGGKYNRGLTVVVAFRELVEPRKQDADSLQRAWTVGWCVELLQAFFLVADDIMDSSLTRRGQICWYQKPGVGLDAINDANLLEACIYRLLK  210

logo
MINODRONIC ACID  ......................T...Q............................KT..Y...................................Q..D......  12
PF00348.2        LYCREQPYYLNLIELFLQSSYQTEIGQTLDLLTAPQGNVDLVRFTEKRYKSIVKYKTAFYSFYLPIAAAMYMAGIDGEKEHANAKKILLEMGEFFQIQDDYLDLF  144
PF00348.3        .........................................................................................................  65
PF00348          LYCREQPYYLNLIELFLQSSYQTEIGQTLDLLTAPQGNVDLVRFTEKRYKSIVKYKTAFYSFYLPIAAAMYMAGIDGEKEHANAKKILLEMGEFFQIQDDYLDLF  206
PF00348.1        LYCREQPYYLNLIELFLQSSYQTEIGQTLDLLTAPQGNVDLVRFTEKRYKSIVKYKTAFYSFYL.........................................  165
FDPS-220         LYCREQPYYLNLIELFLQSSYQTEIGQTLDLLTAPQGNVDLVRFTEKRYKSIVKYKTAFYSFYLPIAAAMYMAGIDGEKEHANAKKILLEMGEFFQIQDDYLDLF  144
FDPS-221         LYCREQPYYLNLIELFLQSSYQTEIGQTLDLLTAPQGNVDLVRFTEKRYKSIVKYKTAFYSFYLPIAAAMYMAGIDGEKEHANAKKILLEMGEFFQIQDDYLDLF  249
FDPS-203         LYCREQPYYLNLIELFLQSSYQTEIGQTLDLLTAPQGNVDLVRFTEKRYKSIVKYKTAFYSFYLPIAAAMYMAGIDGEKEHANAKKILLEMGEFFQIQDDYLDLF  249
FDPS-201         LYCREQPYYLNLIELFLQSSYQTEIGQTLDLLTAPQGNVDLVRFTEKRYKSIVKYKTAFYSFYLPIAAAMYMAGIDGEKEHANAKKILLEMGEFFQIQDDYLDLF  315
FDPS-206         LYCREQPYYLNLIELFLQSSYQTEIGQTLDLLTAPQGNVDLVRFTEKRYKSIVKYKTAFYSFYLPIAAAMYMAGIDGEKEHANAKKILLEMGEFFQIQDDYLDLF  249
FDPS-216         LYCREQPYYLNLIELFLQSSYQTEIGQTLDLLTAPQGNVDLVRFTEKRYKSIVKYKTAFYSFYL.........................................  274
FDPS-205         .........................................................................................................  174
FDPS-210         .........................................................................................................  80
FDPS-202         LYCREQPYYLNLIELFLQSSYQTEIGQTLDLLTAPQGNVDLVRFTEKRYKSIVKYKTAFYSFYLPIAAAMYMAGIDGEKEHANAKKILLEMGEFFQIQDDYLDLF  315
```

logo

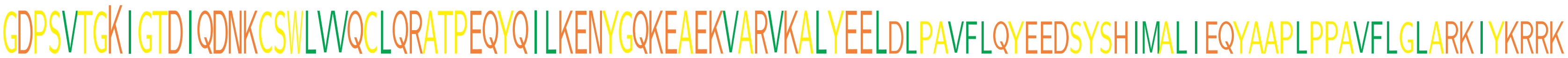

```
MINODRONIC ACID  .......K................................................................................................  13
PF00348.2        GDPSVTGKIGTDIQDNKCSWLVVQCLQRATPEQYQILKENYGQKEAEKVARVKALYEEL.............................................  203
PF00348.3        ........................................................................................................  65
PF00348          GDPSVTGKIGTDIQDNKCSWLVVQCLQRATPEQYQILKENYGQKEAEKVARVKALYEEL.............................................  265
PF00348.1        ........................................................................................................  165
FDPS-220         GDPSVTGKIGTDIQDNKCSWLVVQCLQRATPEQYQILKENYGQKEAEKVARVKALYEELDLPAVFLQYEEDSYSHIMALIEQYAAPLPPAVFLGLARKIYKRRK  248
FDPS-221         GDPSVTGKIGTDIQDNKCSWLVVQCLQRATPEQYQILKENYGQKEAEKVARVKALYEELDLPAVFLQYEEDSYSHIMALIEQYAAPLPPAVFLGLARKIYKRRK  353
FDPS-203         GDPSVTGKIGTDIQDNKCSWLVVQCLQRATPEQYQILKENYGQKEAEKVARVKALYEELDLPAVFLQYEEDSYSHIMALIEQYAAPLPPAVFLGLARKIYKRRK  353
FDPS-201         GDPSVTGKIGTDIQDNKCSWLVVQCLQRATPEQYQILKENYGQKEAEKVARVKALYEELDLPAVFLQYEEDSYSHIMALIEQYAAPLPPAVFLGLARKIYKRRK  419
FDPS-206         GDPSVTGKIGTDIQDNKCSWLVVQCLQRATPEQYQILKENYGQKEAEKVARVKALYEELDLPAVFLQYEEDSYSHIMALIEQYAAPLPPAVFLGLARKIYKRRK  353
FDPS-216         ........................................................................................................  274
FDPS-205         ........................................................................................................  174
FDPS-210         ........................................................................................................  80
FDPS-202         GDPSVTGKIGTDIQDNKCSWLVVQCLQRATPEQYQILKENYGQKEAEKVARVKALYEELDLPAVFLQYEEDSYSHIMALIEQYAAPLPPAVFLGLARKIYKRRK  419
```

- X non conserved
- X similar
- X $\geq$ 0% conserved
- X $\geq$ 50% conserved

logo 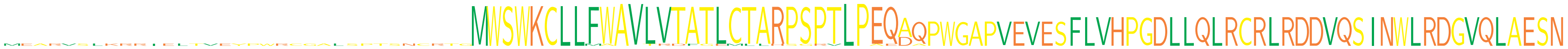

```
DOVITINIB    ..............................................................................................................    0
PF07679.1    ..............................................................................................................    0
PF07679.4    ..............................................................................................................    0
PF07679.3    ..............................................................................................................    0
PF07679.2    ..............................................................................................................    0
PF00047      ...........................................................................FLVHPGDLLQLRCRLRDDVQSINWLRDGVQLAESN   35
PF00047.1    ...........................................................................FLVHPGDLLQLRCRLRDDVQSINWLRDGVQLAESN   35
PF07714      ..............................................................................................................    0
PF07679      ..............................................................................................................    0
FGFR1-236    ..............................................................................................................    0
FGFR1-214    .................................MWSWKCLLFWAVLVTATLCTARPSPTLPEQAQPWGAPVEVESFLVHPGDLLQLRCRLRDDVQSINWLRDGVQLAESN   77
FGFR1-202    .........................................MAAVTRDFGEMLLHSGRVLPAEAQPWGAPVEVESFLVHPGDLLQLRCRLRDDVQSINWLRDGVQLAESN   69
FGFR1-201    .................................MWSWKCLLFWAVLVTATLCTARPSPTLPEQ...............................................   30
FGFR1-241    .................................MWSWKCLLFWAVLVTATLCTARPSPTLPEQ...............................................   30
FGFR1-209    .................................MWSWKCLLFWAVLVTATLCTARPSPTLPEQAQPWGAPVEVESFLVHPGDLLQLRCRLRDDVQSINWLRDGVQLAESN   77
FGFR1-238    .................................MWSWKCLLFWAVLVTATLCTARPSPTLPEQAQPWGAPVEVESFLVHPGDLLQLRCRLRDDVQSINWLRDGVQLAESN   77
FGFR1-210    .................................MWSWKCLLFWAVLVTATLCTARPSPTLPEQAQPWGAPVEVESFLVHPGDLLQLRCRLRDDVQSINWLRDGVQLAESN   77
FGFR1-208    .................................MWSWKCLLFWAVLVTATLCTARPSPTLPEQAQPWGAPVEVESFLVHPGDLLQLRCRLRDDVQSINWLRDGVQLAESN   77
FGFR1-212    .................................MWSWKCLLFWAVLVTATLCTARPSPTLPEQDA.............................................   32
FGFR1-204    .................................MWSWKCLLFWAVLVTATLCTARPSPTLPEQD..............................................   31
FGFR1-221    .................................MWSWKCLLFWAVLVTATLCTARPSPTLPEQAQPWGAPVEVESFLVHPGDLLQLRCRLRDDVQSINWLRDGVQLAESN   77
FGFR1-207    .................................MWSWKCLLFWAVLVTATLCTARPSPTLPEQD..............................................   31
FGFR1-213    ..............................................................................................................    0
FGFR1-226    .................................MWSWKCLLFWAVLVTATLCTARPSPTLPEQAQPWGAPVEVESFLVHPGDLLQLRCRLRDDVQSINWLRDGVQLAESN   77
FGFR1-229    .................................MWSWKCLLFWAVLVTATLCTARPSPTLPEQD..............................................   31
FGFR1-211    MEARVSLKRRIELTVEYPWRCGALSPTSNCRTGMWSWKCLLFWAVLVTATLCTARPSPTLPEQAQPWGAPVEVESFLVHPGDLLQLRCRLRDDVQSINWLRDGVQLAESN  110
FGFR1-234    .................................MWSWKCLLFWAVLVTATLCTARPSPTLPEQD..............................................   31
FGFR1-203    .................................MWSWKCLLFWAVLVTATLCTARPSPTLPEQAQPWGAPVEVESFLVHPGDLLQLRCRLRDDVQSINWLRDGVQLAESN   77
FGFR1-240    ..............................................................................................................    0
FGFR1-222    ..............................................................................................................    0
FGFR1-233    .................................MWSWKCLLFWAVLVTATLCTARPSPTLPEQ...............................................   30
FGFR1-206    .................................MWSWKCLLFWAVLVTATLCTARPSPTLPEQAQPWGAPVEVESFLVHPGDLLQLRCRLRDDVQSINWLRDGVQLAESN   77
```

logo
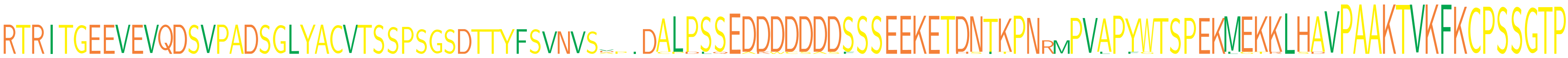

```
DOVITINIB   ..............................................................................................................   0
PF07679.1   ..............................................................................................PAAKTVKFKCPSSGTP  16
PF07679.4   .............................................................................................VPAAKTVKFKCPSSGTP  17
PF07679.3   ..............................................................................................PAAKTVKFKCPSSGTP  16
PF07679.2   .............................................................................................VPAAKTVKFKCPSSGTP  17
PF00047     RTRITGEEVEVQDSVPADSGLYACVTSSPSGSDTTYFS........................................................................  73
PF00047.1   RTRITGEEVEVQDSVPADSGLYACVTS...................................................................................  62
PF07714     ..............................................................................................................   0
PF07679     ..............................................................................................PAAKTVKFKCPSSGTP  16
FGFR1-236   ..............................................................................................................   0
FGFR1-214   RTRITGEEVEVQDSVPADSGLYACVTSSPSGSDTTYFSVNVS...DALPSSEDDDDDDDSSSEEKETDNTKPNRMPVAPYWTSPEKMEKKLHAVPAAKTVKFKCPSSGTP 184
FGFR1-202   RTRITGEEVEVQDSVPADSGLYACVTSSPSGSDTTYFSVNVSD...ALPSSEDDDDDDDSSSEEKETDNTKPNRMPVAPYWTSPEKMEKKLHAVPAAKTVKFKCPSSGTP 176
FGFR1-201   .............................................DALPSSEDDDDDDDSSSEEKETDNTKPN..PVAPYWTSPEKMEKKLHAVPAAKTVKFKCPSSGTP  93
FGFR1-241   .............................................DALPSSEDDDDDDDSSSEEKETDNTKPN..PVAPYWTSPEKMEKKLHAVPAAKTVKFKCPSSGTP  93
FGFR1-209   RTRITGEEVEVQDSVPADSGLYACVTSSPSGSDTTYFSVNVS...DALPSSEDDDDDDDSSSEEKETDNTKPN..PVAPYWTSPEKMEKKLHAVPAAKTVKFKCPSSGTP 182
FGFR1-238   RTRITGEEVEVQDSVPADSGLYACVTSSPSGSDTTYFSVNVS...DALPSSEDDDDDDDSSSEEKETDNTKPNRMPVAPYWTSPEKMEKKLHAVPAAKTVKFKCPSSGTP 184
FGFR1-210   RTRITGEEVEVQDSVPADSGLYACVTSSP................................................................................. 106
FGFR1-208   RTRITGEEVEVQDSVPADSGLYACVTSSPSGSDTTYFSVNVS...DALPSSEDDDDDDDSSSEEKETDNTKPN..PVAPYWTSPEKMEKKLHAVPAAKTVKFKCPSSGTP 182
FGFR1-212   ...............................................LPSSEDDDDDDDSSSEEKETDN.........................................  54
FGFR1-204   ..............................................ALPSSEDDDDDDDSSSEEKETDNTKPNRMPVAPYWTSPEKMEKKLHAVPAAKTVKFKCPSSGTP  95
FGFR1-221   RTRITGEEVEVQDSVPADSGLYACVTSSPSGSDTTYFSVNVSA...CPDLQEAKWCSASF.......HSITP.....LPFG.....LGTRLSD................. 150
FGFR1-207   ..............................................ALPSSEDDDDDDDSSSEEKETDNTKPN..PVAPYWTSPEKMEKKLHAVPAAKTVKFKCPSSGTP  93
FGFR1-213   ..............................................................................................................   0
FGFR1-226   RTRITGEEVEVQDSVPADSGLYACVTSSPSGSDTTYFSVNVS...DALPSSEDDDDDDDSSSEEKETDNTKPNRMPVAPYWTSPEKMEKKLHAVPAAKTVKFKCPSSGTP 184
FGFR1-229   ..............................................ALPSSEDDDDDDDSSSEEKETDNTKPN..PVAPYWTSPEKMEKKLHAVPAAKTVKFKCPSSGTP  93
FGFR1-211   RTRITGEEVEVQDSVPADSGLYACVTSSPSGSDTTYFSVNVS...DALPSSEDDDDDDDSSSEEKETDNTKPN..PVAPYWTSPEKMEKKLHAVPAAKTVKFKCPSSGTP 215
FGFR1-234   ..............................................ALPSSEDDDDDDDSSSEEKETDNTKPN..PVAPYWTSPEKMEKKLHAVPAAKTVKF........  85
FGFR1-203   RTRITGEEVEVQDSVPADSGLYACVTSSPSGSDTTYFSVNVSVPIDALPSSEDDDDDDDSSSEEKETDNTKPN..PVAPYWTSPEKMEKKLHAVPAAKTVKFKCPSSGTP 185
FGFR1-240   ......................................................................................MEKKLHAVPAAKTVKFKCPSSGTP  24
FGFR1-222   ..............................................................................................................   0
FGFR1-233   .............................................DALPSSEDDDDDDDSSSEEKETDNTKPNRMPVAPYWTSPEKMEKKLHAVPAAKTVKFKCPSSGTP  95
FGFR1-206   RTRITGEEVEVQDSVPADSGLYACVTSSPSGSDTTYFSVNVS...DALPSSEDDDDDDDSSSEEKETDNTKPN..PVAPYWTSPEKMEKKLHAVPAAKTVKFKCPSSGTP 182
```

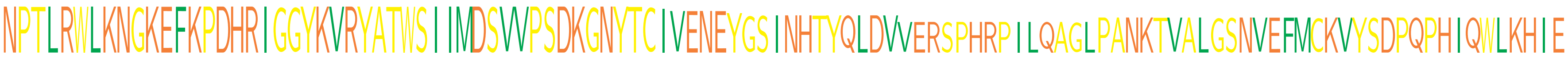

```
logo
DOVITINIB    ..............................................................................................................   0
PF07679.1    NPTLRWLKNGKEFKPDHRIGGYKVRYATWSIIMDSVVPSDKGNYTCIVENEYGSINHTYQLDV..............PANKTVALGSNVEFMCKVYSDPQPHIQWLKHIE 112
PF07679.4    NPTLRWLKNGKEFKPDHRIGGYKVRYATWSIIMDSVVPSDKGNYTC................................................................  63
PF07679.3    NPTLRWLKNGKEFKPDHRIGGYKVRYATWSIIMDSVVPSDKGNYTCIVENEYGSINHTYQLDV...............................................  79
PF07679.2    NPTLRWLKNGKEFKPDHRIGGYKVRYATWSIIMDSVVPSDKGNYTCIVENE...........................................................  68
PF00047      ..............................................................................................................  73
PF00047.1    ..............................................................................................................  62
PF07714      ..............................................................................................................   0
PF07679      NPTLRWLKNGKEFKPDHRIGGYKVRYATWSIIMDSVVPSDKGNYTCIVENEYGSINHTYQLDV..............PANKTVALGSNVEFMCKVYSDPQPHIQWLKHIE 112
FGFR1-236    ..............................................................................................................   0
FGFR1-214    NPTLRWLKNGKEFKPDHRIGGYKVRYATWSIIMDSVVPSDKGNYTCIVENEYGSINHTYQLDVVERSPHRPILQAGLPANKTVALGSNVEFMCKVYSDPQPHIQWLKHIE 294
FGFR1-202    NPTLRWLKNGKEFKPDHRIGGYKVRYATWSIIMDSVVPSDKGNYTCIVENEYGSINHTYQLDVVERSPHRPILQAGLPANKTVALGSNVEFMCKVYSDPQPHIQWLKHIE 286
FGFR1-201    NPTLRWLKNGKEFKPDHRIGGYKVRYATWSIIMDSVVPSDKGNYTCIVENEYGSINHTYQLDVVERSPHRPILQAGLPANKTVALGSNVEFMCKVYSDPQPHIQWLKHIE 203
FGFR1-241    NPTLRWLKNGKEFKPDHRIGGYKVRYATWSIIMDSVVPSDKGNYTCIVENEYGSINHTYQLDVVERSPHRPILQAGLPANKTVALGSNVEFMCKVYSDPQPHIQWLKHIE 203
FGFR1-209    NPTLRWLKNGKEFKPDHRIGGYKVRYATWSIIMDSVVPSDKGNYTCIVENEYGSINHTYQLDVVERSPHRPILQAGLPANKTVALGSNVEFMCKVYSDPQPHIQWLKHIE 292
FGFR1-238    NPTLRWLKNGKEFKPDHRIGGYKVRYATWSIIMDSVVPSDKGNYTCIVENEYGSINHTYQLDVVERSPHRPILQAGLPANKTVALGSNVEFMCKVYSDPQPHIQWLKHIE 294
FGFR1-210    .............................................................................................................. 106
FGFR1-208    NPTLRWLKNGKEFKPDHRIGGYKVRYATWSIIMDSVVPSDKGNYTCIVENEYGSINHTYQLDVVERSPHRPILQAGLPANKTVALGSNVEFMCKVYSDPQPHIQWLKHIE 292
FGFR1-212    ..............................................................................................................  54
FGFR1-204    NPTLRWLKNGKEFKPDHRIGGYKVRYATWSIIMDSVVPSDKGNYTCIVENEYGSINHTYQLDVVERSPHRPILQAGLPANKTVALGSNVEFMCKVYSDPQPHIQWLKHIE 205
FGFR1-221    .............................................................................................................. 150
FGFR1-207    NPTLRWLKNGKEFKPDHRIGGYKVRYATWSIIMDSVVPSDKGNYTCIVENEYGSINHTYQLDVVERSPHRPILQAGLPANKTVALGSNVEFMCKVYSDPQPHIQWLKHIE 203
FGFR1-213    ..............................................................................................................   0
FGFR1-226    NPTLRWLKNGKEFKPDHRIGGYKVRYATWSIIMDSVVPSDKGNYTCIVENEYGSINHTYQLDVVERSPHRPILQAGLPANKTVALGSNVEFMCKVYSDPQPHIQWLKHIE 294
FGFR1-229    NPTLRWLKNGKEFKPDHRIGGYKVRYATWSIIMDSVVPSDKGNYTCIVENEY.......................................................... 145
FGFR1-211    NPTLRWLKNGKEFKPDHRIGGYKVRYATWSIIMDSVVPSDKGNYTCIVENEYGSINHTYQLDVVERSPHRPILQAGLPANKTVALGSNVEFMCKVYSDPQPHIQWLKHIE 325
FGFR1-234    ..............................................................................................................  85
FGFR1-203    NPTLRWLKNGKEFKPDHRIGGYKVRYATWSIIMDSVVPSDKGNYTCIVENEYGSINHTYQLDVVERSPHRPILQAGLPANKTVALGSNVEFMCKVYSDPQPHIQWLKHIE 295
FGFR1-240    NPTLRWLKNGKEFKPDHRIGGYKVRYATWSIIMDSVVPSDKGNYTCIVENEYGSINHTYQLDVVERSPHRPILQAGLPANKTVALGSNVEFMCKVYSDPQPHIQWLKHIE 134
FGFR1-222    ..............................................................................................................   0
FGFR1-233    NPTLRWLKNGKEFKPDHRIGGYKVRYATWSIIMDSVVPSDKGNYTCI............................................................... 142
FGFR1-206    NPTLRWLKNGKEFKPDHRIGGYKVRYATWSIIMDSVVPSDKGNYTCIVENEYGSINHTYQLDVVERSPHRPILQAGLPANKTVALGSNVEFMCKVYSDPQPHIQWLKHIE 292
```

logo

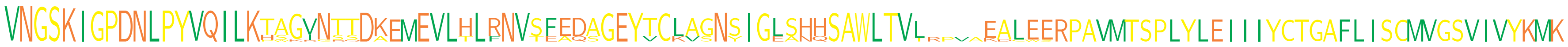

```
DOVITINIB    ..............................................................................................................    0
PF07679.1    VNGSKIGPDNLPYVQILKHSGINSSDA..EVLTLFNVTEAQSGEYVCKVSNYIGEANQSAWLTV..............................................  174
PF07679.4    ..............................................................................................................   63
PF07679.3    ..............................................................................................................   79
PF07679.2    ..............................................................................................................   68
PF00047      ..............................................................................................................   73
PF00047.1    ..............................................................................................................   62
PF07714      ..............................................................................................................    0
PF07679      VNGSKIGPDNLPYVQILKTAGVNTTDKEMEVLHLRNVSFEDAGEYTCLAGNSIGLSHHSAWLTV..............................................  176
FGFR1-236    ..............................................................................................................    0
FGFR1-214    VNGSKIGPDNLPYVQILKTAGVNTTDKEMEVLHLRNVSFEDAGEYTCLAGNSIGLSHHSAWLTVL....EALEERPAVMTSPLYLEIIIYCTGAFLISCMVGSVIVYKMK  400
FGFR1-202    VNGSKIGPDNLPYVQILKTAGVNTTDKEMEVLHLRNVSFEDAGEYTCLAGNSIGLSHHSAWLTVL....EALEERPAVMTSPLYLEIIIYCTGAFLISCMVGSVIVYKMK  392
FGFR1-201    VNGSKIGPDNLPYVQILKTAGVNTTDKEMEVLHLRNVSFEDAGEYTCLAGNSIGLSHHSAWLTVL....EALEERPAVMTSPLYLEIIIYCTGAFLISCMVGSVIVYKMK  309
FGFR1-241    VNGSKIGPDNLPYVQILKPWKRGRQ.....................................................................................  228
FGFR1-209    VNGSKIGPDNLPYVQILKTAGVNTTDKEMEVLHLRNVSFEDAGEYTCLAGNSIGLSHHSAWLTVL....EALEERPAVMTSPLYLEIIIYCTGAFLISCMVGSVIVYKMK  398
FGFR1-238    VNGSKIGPDNLPYVQILKTAGVNTTDKEMEVLHLRNVSFEDAGEYTCLAGNSIGLSHHSAWLTVL....EALEERPAVMTSPLYLEIIIYCTGAFLISCMVGSVIVYKMK  400
FGFR1-210    ..............................................................................................................  106
FGFR1-208    VNGSKIGPDNLPYVQILKTAGVNTTDKEMEVLHLRNVSFEDAGEYTCLAGNSIGLSHHSAWLTVL....EALEERPAVMTSPLYLEIIIYCTGAFLISCMVGSVIVYKMK  398
FGFR1-212    ..............................................................................................................   54
FGFR1-204    VNGSKIGPDNLPYVQILKTAGVNTTDKEMEVLHLRNVSFEDAGEYTCLAGNSIGLSHHSAWLTVL....EALEERPAVMTSPLYLEIIIYCTGAFLISCMVGSVIVYKMK  311
FGFR1-221    ..............................................................................................................  150
FGFR1-207    VNGSKIGPDNLPYVQILKHSGINSSDA..EVLTLFNVTEAQSGEYVCKVSNYIGEANQSAWLTVTRPVAKALEERPAVMTSPLYLEIIIYCTGAFLISCMVGSVIVYKMK  311
FGFR1-213    ..............................................................................................................    0
FGFR1-226    VNGS..........................................................................................................  298
FGFR1-229    ..............................................................................................................  145
FGFR1-211    VNGSKIGPDNLPYVQILKTAGVNTTDKEMEVLHLRNVSFEDAGEYTCLAGNSIGLSHHSAWLTVL....EALEERPAVMTSPLYLEIIIYCTGAFLISCMVGSVIVYKMK  431
FGFR1-234    ..............................................................................................................   85
FGFR1-203    VNGSKIGPDNLPYVQILKHSGINSSDA..EVLTLFNVTEAQSGEYVCKVSNYIGEANQSAWLTVTRPVAKDCWS....................................  367
FGFR1-240    VNGSK.........................................................................................................  139
FGFR1-222    ..............................................................................................................    0
FGFR1-233    ..............................................................................................................  142
FGFR1-206    VNGSKIGPDNLPYVQILKTAGVNTTDKEMEVLHLRNVSFEDAGEYTCLAGNSIGLSHHSAWLTVL....EALEERPAVMTSPLYLEIIIYCTGAFLISCMVGSVIVYKMK  398
```

```
logo
               SGTKKSDFHSQMAVHKLAKSIPLRRQVTVSADSSASMNSGVLLVRPSRLSSSGTPMLAGVSEYELPEDPRWELPRDRLVLGKPLGEGCFGQVVLAEAIGLDKDKPNRVTK
DOVITINIB      ..............................................................................................................     0
PF07679.1      ..............................................................................................................   174
PF07679.4      ..............................................................................................................    63
PF07679.3      ..............................................................................................................    79
PF07679.2      ..............................................................................................................    68
PF00047        ..............................................................................................................    73
PF00047.1      ..............................................................................................................    62
PF07714        ..............................................................................VLGKPLGEGCFGQVVLAEAIGLDKDKPNRVTK    32
PF07679        ..............................................................................................................   176
FGFR1-236      ..............................................................................................................     0
FGFR1-214      SGTKKSDFHSQMAVHKLAKSIPLRRQVTVSADSSASMNSGVLLVRPSRLSSSGTPMLAGVSEYELPEDPRWELPRDRLVLGKPLGEGCFGQVVLAEAIGLDKDKPNRVTK   510
FGFR1-202      SGTKKSDFHSQMAVHKLAKSIPLRRQV..SADSSASMNSGVLLVRPSRLSSSGTPMLAGVSEYELPEDPRWELPRDRLVLGKPLGEGCFGQVVLAEAIGLDKDKPNRVTK   500
FGFR1-201      SGTKKSDFHSQMAVHKLAKSIPLRRQVTVSADSSASMNSGVLLVRPSRLSSSGTPMLAGVSEYELPEDPRWELPRDRLVLGKPLGEGCFGQVVLAEAIGLDKDKPNRVTK   419
FGFR1-241      ..............................................................................................................   228
FGFR1-209      SGTKKSDFHSQMAVHKLAKSIPLRRQVTVSADSSASMNSGVLLVRPSRLSSSGTPMLAGVSEYELPEDPRWELPRDRLVLGKPLGEGCFGQVVLAEAIGLDKDKPNRVTK   508
FGFR1-238      SGTKKSDFHSQMAVHKLAKSIPLRRQV..SADSSASMNSGVLLVRPSRLSSSGTPMLAGVSEYELPEDPRWELPRDRLVLGKPLGEGCFGQVVLAEAIGLDKDKPNRVTK   508
FGFR1-210      ..............................................................................................................   106
FGFR1-208      SGTKKSDFHSQMAVHKLAKSIPLRRQVTVSADSSASMNSGVLLVRPSRLSSSGTPMLAGVSEYELPEDPRWELPRDRLVLGKPLGEGCFGQVVLAEAIGLDKDKPNRVTK   508
FGFR1-212      ..............................................................................................................    54
FGFR1-204      SGTKKSDFHSQMAVHKLAKSIPLRRQVTVSADSSASMNSGVLLVRPSRLSSSGTPMLAGVSEYELPEDPRWELPRDRLVLGKPLGEGCFGQVVLAEAIGLDKDKPNRVTK   421
FGFR1-221      ..............................................................................................................   150
FGFR1-207      SGTKKSDFHSQMAVHKLAKSIPLRRQVTVSADSSASMNSGVLLVRPSRLSSSGTPMLAGVSEYELPEDPRWELPRDRLVLGKPLGEGCFGQVVLAEAIGLDKDKPNRVTK   421
FGFR1-213      ..............................................................................................................     0
FGFR1-226      ..............................................................................................................   298
FGFR1-229      ..............................................................................................................   145
FGFR1-211      SGTKKSDFHSQMAVHKLAKSIPLRRQVTVSADSSASMNSGVLLVRPSRLSSSGTPMLAGVSEYELPEDPRWELPRDRLVLGKPLGEGCFGQVVLAEAIGLDKDKPNRVTK   541
FGFR1-234      ..............................................................................................................    85
FGFR1-203      ..............................................................................................................   367
FGFR1-240      ..............................................................................................................   139
FGFR1-222      ..............................................................................................................     0
FGFR1-233      ..............................................................................................................   142
FGFR1-206      SGTKKSDFHSQMAVHKLAKSIPLRRQVTVSADSSASMNSGVLLVRPSRLSSSGTPMLAGVSEYELPEDPRWELPRDRLVLGKPLGEGCFGQVVLAEAIGLDKDKPNRVTK   508
```

```
logo
                VAVKMLKSDATEKDLSDLISEMEMMKMIGKHKNIINLLGACTQDGPLYVIVEYASKGNLREYLQARRPPGLEYCYNPSHNPEEQLSSKDLVSCAYQV      ARGMEYL
DOVITINIB       ..................................................................................L.......V...................    2
PF07679.1       ..............................................................................................................  174
PF07679.4       ..............................................................................................................   63
PF07679.3       ..............................................................................................................   79
PF07679.2       ..............................................................................................................   68
PF00047         ..............................................................................................................   73
PF00047.1       ..............................................................................................................   62
PF07714         VAVKMLKSDATEKDLSDLISEMEMMKMIGKHKNIINLLGACTQDGPLYVIVEYASKGNLREYLQARRPPGLEYCYNPSHNPEEQLSSKDLVSCAYQV......ARGMEYL  136
PF07679         ..............................................................................................................  176
FGFR1-236       ..........................................................................................VSCAYQV......ARGMEYL   14
FGFR1-214       VAVKMLKSDATEKDLSDLISEMEMMKMIGKHKNIINLLGACTQDGPLYVIVEYASKGNLREYLQARRPPGLEYCYNPSHNPEEQLSSKDLVSCAYQV......ARGMEYL  614
FGFR1-202       VAVKMLKSDATEKDLSDLISEMEMMKMIGKHKNIINLLGACTQDGPLYVIVEYASKGNLREYLQARRPPGLEYCYNPSHNPEEQLSSKDLVSCAYQV......ARGMEYL  604
FGFR1-201       VAVKMLKSDATEKDLSDLISEMEMMKMIGKHKNIINLLGACTQDGPLYVIVEYASKGNLREYLQARRPPGLEYCYNPSHNPEEQLSSKDLVSCAYQV......ARGMEYL  523
FGFR1-241       ..............................................................................................................  228
FGFR1-209       VAVKMLKSDATEKDLSDLISEMEMMKMIGKHKNIINLLGACTQDGPLYVIVEYASKGNLREYLQARRPPGLEYCYNPSHNPEEQLSSKDLVSCAYQV......ARGMEYL  612
FGFR1-238       VAVKMLKSDATEKDLSDLISEMEMMKMIGKHKNIINLLGACTQDGPLYVIVEYASKGNLREYLQARRPPGLEYCYNPSHNPEEQLSSKDLVSCAYQV......ARGMEYL  612
FGFR1-210       ..............................................................................................................  106
FGFR1-208       VAVKMLKSDATEKDLSDLISEMEMMKMIGKHKNIINLLGACTQDGPLYVIVEYASKGNLREYLQARRPPGLEYCYNPSHNPEEQLSSKDLVSCAYQV......ARGMEYL  612
FGFR1-212       ..............................................................................................................   54
FGFR1-204       VAVKMLKSDATEKDLSDLISEMEMMKMIGKHKNIINLLGACTQDGPLYVIVEYASKGNLREYLQARRPPGLEYCYNPSHNPEEQLSSKDLVSCAYQV......ARGMEYL  525
FGFR1-221       ..............................................................................................................  150
FGFR1-207       VAVKMLKSDATEKDLSDLISEMEMMKMIGKHKNIINLLGACTQDGPLYVIVEYASKGNLREYLQARRPPGLEYCYNPSHNPEEQLSSKDLVSCAYQV......ARGMEYL  525
FGFR1-213       ..............................................................................................................    0
FGFR1-226       ..............................................................................................................  298
FGFR1-229       ..............................................................................................................  145
FGFR1-211       VAVKMLKSDATEKDLSDLISEMEMMKMIGKHKNIINLLGACTQDGPLYVIVEYASKGNLREYLQARRPPGLEYCYNPSHNPEEQLSSKDLVSCAYQV......ARGMEYL  645
FGFR1-234       ..............................................................................................................   85
FGFR1-203       ..............................................................................................................  367
FGFR1-240       ..............................................................................................................  139
FGFR1-222       ..............................................................................................................    0
FGFR1-233       ..............................................................................................................  142
FGFR1-206       VAVKMLKSDATEKDLSDLISEMEMMKMIGKHKNIINLLGACTQDGPLYVIVEYASKGNLREYLQARRPPGLEYCYNPSHNPEEQLSSKDLVSCAYQV......ARGMEYL  612
```

```
logo
             ASKKCIHRDLAARN         VLVTEDNVMKIAD    FGLARDIHHIDYYKKTTNGRLPVKWMAPEALFDRIYTHQSDVWSFG           VLLWEIFTLG
DOVITINIB    A.K..............................I...............MEYASKG......................................................   12
PF07679.1    ..............................................................................................................  174
PF07679.4    ..............................................................................................................   63
PF07679.3    ..............................................................................................................   79
PF07679.2    ..............................................................................................................   68
PF00047      ..............................................................................................................   73
PF00047.1    ..............................................................................................................   62
PF07714      ASKKCIHRDLAARN.........VLVTEDNVMKIAD....FGLARDIHHIDYYKKTTNGRLPVKWMAPEALFDRIYTHQSDVWSFG...........VLLWEIFTLG...  219
PF07679      ..............................................................................................................  176
FGFR1-236    ASKKCIHRDLAARN.........VLVTEDNVMKIAD....FGLARDIHHIDYYKKTTNGRLPVKWMAPEALFDRIYTHQSDVWSFG...........VLLWEIFTLG...   97
FGFR1-214    ASKKCIHRDLAARN.........VLVTEDNVMKIAD....FGLARDIHHIDYYKKTTNGRLPVKWMAPEALFDRIYTHQSDVWSFG...........VLLWEIFTLG...  697
FGFR1-202    ASKKCIHRDLAARN.........VLVTEDNVMKIAD....FGLARDIHHIDYYKKTTNGRLPVKWMAPEALFDRIYTHQSDVWSFG...........VLLWEIFTLG...  687
FGFR1-201    ASKKCIHRDLAARN.........VLVTEDNVMKIAD....FGLARDIHHIDYYKKTTNGRLPVKWMAPEALFDRIYTHQSDVWSFG...........VLLWEIFTLG...  606
FGFR1-241    ..............................................................................................................  228
FGFR1-209    ASKKCIHRDLAARN.........VLVTEDNVMKIAD....FGLARDIHHIDYYKKTTNGRLPVKWMAPEALFDRIYTHQSDVWSFG...........VLLWEIFTLG...  695
FGFR1-238    ASKKCIHRDLAARN.........VLVTEDNVMKIAD....FGLARDIHHIDYYKKTTNGRLPVKWMAPEALFDRIYTHQSDVWSFG...........VLLWEIFTLG...  695
FGFR1-210    ..............................................................................................................  106
FGFR1-208    ASKKCIHRDLAARN.........VLVTEDNVMKIAD....FGLARDIHHIDYYKKTTNGRLPVKWMAPEALFDRIYTHQSDVWSFG...........VLLWEIFTLG...  695
FGFR1-212    ..............................................................................................................   54
FGFR1-204    ASKKCIHRDLAARN.........VLVTEDNVMKIAD....FGLARDIHHIDYYKKTTNGRLPVKWMAPEALFDRIYTHQSDVWSFG...........VLLWEIFTLG...  608
FGFR1-221    ..............................................................................................................  150
FGFR1-207    ASKKCIHRDLAARN.........VLVTEDNVMKIAD....FGLARDIHHIDYYKKTTNGRLPVKWMAPEALFDRIYTHQSDVWSFG...........VLLWEIFTLG...  608
FGFR1-213    .................................................M............................................................    1
FGFR1-226    ..............................................................................................................  298
FGFR1-229    ..............................................................................................................  145
FGFR1-211    ASKKCIHRDLAARN.........VLVTEDNVMKIAD....FGLARDIHHIDYYKKTTNGRLPVKWMAPEALFDRIYTHQSDVWSFG...........VLLWEIFTLG...  728
FGFR1-234    ..............................................................................................................   85
FGFR1-203    ..............................................................................................................  367
FGFR1-240    ..............................................................................................................  139
FGFR1-222    .................................................................................MWSWK..........CLLFWAVLVTATLC   19
FGFR1-233    ..............................................................................................................  142
FGFR1-206    ASKKCIHRDLAARN.........VLVTEDNVMKIAD....FGLARDIHHIDYYKKTTNGRLPVKWMAPEALFDRIYTHQSDVWSFG...........VLLWEIFTLG...  695
```

```
logo
           .GSPYPGVPVEELFKLLKEGHRMDKPSNCTNELYMMMRDCWHAVPSQRPTFKQLVEDLDRIVALTSNQEYLDLSMPLDQYSPSFPDTRSSTCSSGEDSVFSHEPLPEEPC
DOVITINIB  ............L..........D......................................................................................   14
PF07679.1  ..............................................................................................................  174
PF07679.4  ..............................................................................................................   63
PF07679.3  ..............................................................................................................   79
PF07679.2  ..............................................................................................................   68
PF00047    ..............................................................................................................   73
PF00047.1  ..............................................................................................................   62
PF07714    .GSPYPGVPVEELFKLLKEGHRMDKPSNCTNELYMMMRDCWHAVPSQRPTFKQLVEDL....................................................  276
PF07679    ..............................................................................................................  176
FGFR1-236  .GSPYPGVPVEELFKLLKEGHRMDKPSNCTNELPAA.......GT.................................................................  134
FGFR1-214  .GSPYPGVPVEELFKLLKEGHRMDKPSNCTNELYMMMRDCWHAVPSQRPTFKQLVEDLDRIVALTSNQEYLDLSMPLDQYSPSFPDTRSSTCSSGEDSVFSHEPLPEEPC  806
FGFR1-202  .GSPYPGVPVEELFKLLKEGHRMDKPSNCTNELYMMMRDCWHAVPSQRPTFKQLVEDLDRIVALTSNQEYLDLSMPLDQYSPSFPDTRSSTCSSGEDSVFSHEPLPEEPC  796
FGFR1-201  .GSPYPGVPVEELFKLLKEGHRMDKPSNCTNELYMMMRDCWHAVPSQRPTFKQLVEDLDRIVALTSNQEYLDLSMPLDQYSPSFPDTRSSTCSSGEDSVFSHEPLPEEPC  715
FGFR1-241  ..............................................................................................................  228
FGFR1-209  .GSPYPGVPVEELFKLLKEGHRMDKPSNCTNELYMMMRDCWHAVPSQRPTFKQLVEDLDRIVALTSNQEYLDLSMPLDQYSPSFPDTRSSTCSSGEDSVFSHEPLPEEPC  804
FGFR1-238  .GSPYPGVPVEELFKLLKEGHRMDKPSNCTNELYMMMRDCWHAVPSQRPTFKQLVEDLDRIVALTSNQEYLDLSMPLDQYSPSFPDTRSSTCSSGEDSVFSHEPLPEEPC  804
FGFR1-210  ..............................................................................................................  106
FGFR1-208  .GSPYPGVPVEELFKLLKEGHRMDKPSNCTNELYMMMRDCWHAVPSQRPTFKQLVEDLDRIVALTSNQEYLDLSMPLDQYSPSFPDTRSSTCSSGEDSVFSHEPLPEEPC  804
FGFR1-212  ..............................................................................................................   54
FGFR1-204  .GSPYPGVPVEELFKLLKEGHRMDKPSNCTNELYMMMRDCWHAVPSQRPTFKQLVEDLDRIVALTSNQEYLDLSMPLDQYSPSFPDTRSSTCSSGEDSVFSHEPLPEEPC  717
FGFR1-221  ..............................................................................................................  150
FGFR1-207  .GSPYPGVPVEELFKLLKEGHRMDKPSNCTNELYMMMRDCWHAVPSQRPTFKQLVEDLDRIVALTSNQEYLDLSMPLDQYSPSFPDTRSSTCSSGEDSVFSHEPLPEEPC  717
FGFR1-213  ..............................................................................................................    1
FGFR1-226  ..............................................................................................................  298
FGFR1-229  ..............................................................................................................  145
FGFR1-211  .GSPYPGVPVEELFKLLKEGHRMDKPSNCTNELYMMMRDCWHAVPSQRPTFKQLVEDLDRIVALTSNQEYLDLSMPLDQYSPSFPDTRSSTCSSGEDSVFSHEPLPEEPC  837
FGFR1-234  ..............................................................................................................   85
FGFR1-203  ..............................................................................................................  367
FGFR1-240  ..............................................................................................................  139
FGFR1-222  TARPSPTLPEQACPDL.......QEAKWCSASFHSITPLPFGLGTR........LSD.....................................................   61
FGFR1-233  ..............................................................................................................  142
FGFR1-206  .GSPYPGVPVEELFKLLKEGHRMDKPSNCTNELYMMMRDCWHAVPSQRPTFKQLVEDLDRIVALTSNQEYLDLSMPLDQYSPSFPDTRSSTCSSGEDSVFSHEPLPEEPC  804
```

```
logo
             LPRHPAQLANGGLKRR
DOVITINIB    ................    14
PF07679.1    ................   174
PF07679.4    ................    63
PF07679.3    ................    79
PF07679.2    ................    68
PF00047      ................    73
PF00047.1    ................    62
PF07714      ................   276
PF07679      ................   176
FGFR1-236    ................   134
FGFR1-214    LPRHPAQLANGGLKRR   822
FGFR1-202    LPRHPAQLANGGLKRR   812
FGFR1-201    LPRHPAQLANGGLKRR   731
FGFR1-241    ................   228
FGFR1-209    LPRHPAQLANGGLKRR   820
FGFR1-238    LPRHPAQLANGGLKRR   820
FGFR1-210    ................   106
FGFR1-208    LPRHPAQLANGGLKRR   820
FGFR1-212    ................    54
FGFR1-204    LPRHPAQLANGGLKRR   733
FGFR1-221    ................   150
FGFR1-207    LPRHPAQLANGGLKRR   733
FGFR1-213    ................     1
FGFR1-226    ................   298
FGFR1-229    ................   145
FGFR1-211    LPRHPAQLANGGLKRR   853
FGFR1-234    ................    85
FGFR1-203    ................   367
FGFR1-240    ................   139
FGFR1-222    ................    61
FGFR1-233    ................   142
FGFR1-206    LPRHPAQLANGGLKRR   820
```

X non conserved
X similar
X ≥ 0% conserved
X ≥ 50% conserved

logo

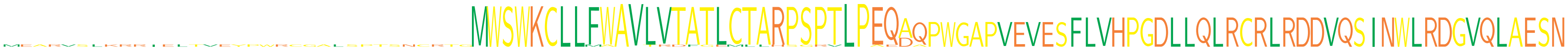

```
INFIGRATINIB   ..............................................................................................................   0
PF07679.1      ..............................................................................................................   0
PF07679.4      ..............................................................................................................   0
PF07679.3      ..............................................................................................................   0
PF07679.2      ..............................................................................................................   0
PF00047        ...........................................................................FLVHPGDLLQLRCRLRDDVQSINWLRDGVQLAESN  35
PF00047.1      ...........................................................................FLVHPGDLLQLRCRLRDDVQSINWLRDGVQLAESN  35
PF07714        ..............................................................................................................   0
PF07679        ..............................................................................................................   0
FGFR1-236      ..............................................................................................................   0
FGFR1-214      .................................MWSWKCLLFWAVLVTATLCTARPSPTLPEQAQPWGAPVEVESFLVHPGDLLQLRCRLRDDVQSINWLRDGVQLAESN  77
FGFR1-202      .........................................MAAVTRDFGEMLLHSGRVLPAEAQPWGAPVEVESFLVHPGDLLQLRCRLRDDVQSINWLRDGVQLAESN  69
FGFR1-201      .................................MWSWKCLLFWAVLVTATLCTARPSPTLPEQ...............................................  30
FGFR1-241      .................................MWSWKCLLFWAVLVTATLCTARPSPTLPEQ...............................................  30
FGFR1-209      .................................MWSWKCLLFWAVLVTATLCTARPSPTLPEQAQPWGAPVEVESFLVHPGDLLQLRCRLRDDVQSINWLRDGVQLAESN  77
FGFR1-238      .................................MWSWKCLLFWAVLVTATLCTARPSPTLPEQAQPWGAPVEVESFLVHPGDLLQLRCRLRDDVQSINWLRDGVQLAESN  77
FGFR1-210      .................................MWSWKCLLFWAVLVTATLCTARPSPTLPEQAQPWGAPVEVESFLVHPGDLLQLRCRLRDDVQSINWLRDGVQLAESN  77
FGFR1-208      .................................MWSWKCLLFWAVLVTATLCTARPSPTLPEQAQPWGAPVEVESFLVHPGDLLQLRCRLRDDVQSINWLRDGVQLAESN  77
FGFR1-212      .................................MWSWKCLLFWAVLVTATLCTARPSPTLPEQDA.............................................  32
FGFR1-204      .................................MWSWKCLLFWAVLVTATLCTARPSPTLPEQD..............................................  31
FGFR1-221      .................................MWSWKCLLFWAVLVTATLCTARPSPTLPEQAQPWGAPVEVESFLVHPGDLLQLRCRLRDDVQSINWLRDGVQLAESN  77
FGFR1-207      .................................MWSWKCLLFWAVLVTATLCTARPSPTLPEQD..............................................  31
FGFR1-213      ..............................................................................................................   0
FGFR1-226      .................................MWSWKCLLFWAVLVTATLCTARPSPTLPEQAQPWGAPVEVESFLVHPGDLLQLRCRLRDDVQSINWLRDGVQLAESN  77
FGFR1-229      .................................MWSWKCLLFWAVLVTATLCTARPSPTLPEQD..............................................  31
FGFR1-211      MEARVSLKRRIELTVEYPWRCGALSPTSNCRTGMWSWKCLLFWAVLVTATLCTARPSPTLPEQAQPWGAPVEVESFLVHPGDLLQLRCRLRDDVQSINWLRDGVQLAESN 110
FGFR1-234      .................................MWSWKCLLFWAVLVTATLCTARPSPTLPEQD..............................................  31
FGFR1-203      .................................MWSWKCLLFWAVLVTATLCTARPSPTLPEQAQPWGAPVEVESFLVHPGDLLQLRCRLRDDVQSINWLRDGVQLAESN  77
FGFR1-240      ..............................................................................................................   0
FGFR1-222      ..............................................................................................................   0
FGFR1-233      .................................MWSWKCLLFWAVLVTATLCTARPSPTLPEQ...............................................  30
FGFR1-206      .................................MWSWKCLLFWAVLVTATLCTARPSPTLPEQAQPWGAPVEVESFLVHPGDLLQLRCRLRDDVQSINWLRDGVQLAESN  77
```

logo

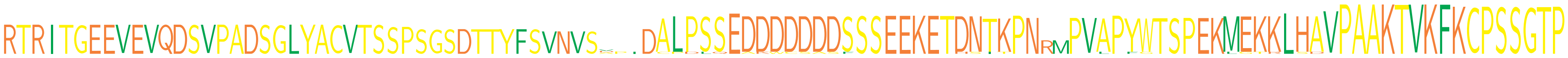

```
INFIGRATINIB  ..............................................................................................................   0
PF07679.1     ..............................................................................................PAAKTVKFKCPSSGTP  16
PF07679.4     .............................................................................................VPAAKTVKFKCPSSGTP  17
PF07679.3     ..............................................................................................PAAKTVKFKCPSSGTP  16
PF07679.2     .............................................................................................VPAAKTVKFKCPSSGTP  17
PF00047       RTRITGEEVEVQDSVPADSGLYACVTSSPSGSDTTYFS.........................................................................  73
PF00047.1     RTRITGEEVEVQDSVPADSGLYACVTS....................................................................................  62
PF07714       ..............................................................................................................   0
PF07679       ..............................................................................................PAAKTVKFKCPSSGTP  16
FGFR1-236     ..............................................................................................................   0
FGFR1-214     RTRITGEEVEVQDSVPADSGLYACVTSSPSGSDTTYFSVNVS...DALPSSEDDDDDDDSSSEEKETDNTKPNRMPVAPYWTSPEKMEKKLHAVPAAKTVKFKCPSSGTP 184
FGFR1-202     RTRITGEEVEVQDSVPADSGLYACVTSSPSGSDTTYFSVNVSD...ALPSSEDDDDDDDSSSEEKETDNTKPNRMPVAPYWTSPEKMEKKLHAVPAAKTVKFKCPSSGTP 176
FGFR1-201     ..............................................DALPSSEDDDDDDDSSSEEKETDNTKPN..PVAPYWTSPEKMEKKLHAVPAAKTVKFKCPSSGTP  93
FGFR1-241     ..............................................DALPSSEDDDDDDDSSSEEKETDNTKPN..PVAPYWTSPEKMEKKLHAVPAAKTVKFKCPSSGTP  93
FGFR1-209     RTRITGEEVEVQDSVPADSGLYACVTSSPSGSDTTYFSVNVS...DALPSSEDDDDDDDSSSEEKETDNTKPN..PVAPYWTSPEKMEKKLHAVPAAKTVKFKCPSSGTP 182
FGFR1-238     RTRITGEEVEVQDSVPADSGLYACVTSSPSGSDTTYFSVNVS...DALPSSEDDDDDDDSSSEEKETDNTKPNRMPVAPYWTSPEKMEKKLHAVPAAKTVKFKCPSSGTP 184
FGFR1-210     RTRITGEEVEVQDSVPADSGLYACVTSSP................................................................................. 106
FGFR1-208     RTRITGEEVEVQDSVPADSGLYACVTSSPSGSDTTYFSVNVS...DALPSSEDDDDDDDSSSEEKETDNTKPN..PVAPYWTSPEKMEKKLHAVPAAKTVKFKCPSSGTP 182
FGFR1-212     ...............................................LPSSEDDDDDDDSSSEEKETDN.........................................  54
FGFR1-204     ...............................................ALPSSEDDDDDDDSSSEEKETDNTKPNRMPVAPYWTSPEKMEKKLHAVPAAKTVKFKCPSSGTP  95
FGFR1-221     RTRITGEEVEVQDSVPADSGLYACVTSSPSGSDTTYFSVNVSA...CPDLQEAKWCSASF.......HSITP.....LPFG.....LGTRLSD................. 150
FGFR1-207     ...............................................ALPSSEDDDDDDDSSSEEKETDNTKPN..PVAPYWTSPEKMEKKLHAVPAAKTVKFKCPSSGTP  93
FGFR1-213     ..............................................................................................................   0
FGFR1-226     RTRITGEEVEVQDSVPADSGLYACVTSSPSGSDTTYFSVNVS...DALPSSEDDDDDDDSSSEEKETDNTKPNRMPVAPYWTSPEKMEKKLHAVPAAKTVKFKCPSSGTP 184
FGFR1-229     ...............................................ALPSSEDDDDDDDSSSEEKETDNTKPN..PVAPYWTSPEKMEKKLHAVPAAKTVKFKCPSSGTP  93
FGFR1-211     RTRITGEEVEVQDSVPADSGLYACVTSSPSGSDTTYFSVNVS...DALPSSEDDDDDDDSSSEEKETDNTKPN..PVAPYWTSPEKMEKKLHAVPAAKTVKFKCPSSGTP 215
FGFR1-234     ...............................................ALPSSEDDDDDDDSSSEEKETDNTKPN..PVAPYWTSPEKMEKKLHAVPAAKTVKF........  85
FGFR1-203     RTRITGEEVEVQDSVPADSGLYACVTSSPSGSDTTYFSVNVSVPIDALPSSEDDDDDDDSSSEEKETDNTKPN..PVAPYWTSPEKMEKKLHAVPAAKTVKFKCPSSGTP 185
FGFR1-240     ......................................................................................MEKKLHAVPAAKTVKFKCPSSGTP  24
FGFR1-222     ..............................................................................................................   0
FGFR1-233     ..............................................DALPSSEDDDDDDDSSSEEKETDNTKPNRMPVAPYWTSPEKMEKKLHAVPAAKTVKFKCPSSGTP  95
FGFR1-206     RTRITGEEVEVQDSVPADSGLYACVTSSPSGSDTTYFSVNVS...DALPSSEDDDDDDDSSSEEKETDNTKPN..PVAPYWTSPEKMEKKLHAVPAAKTVKFKCPSSGTP 182
```

```
logo          NPTLRWLKNGKEFKPDHRIGGYKVRYATWSIIMDSVVPSDKGNYTCIVENEYGSINHTYQLDVVERSPHRPILQAGLPANKTVALGSNVEFMCKVYSDPQPHIQWLKHIE
INFIGRATINIB  ..............................................................................................................   0
PF07679.1     NPTLRWLKNGKEFKPDHRIGGYKVRYATWSIIMDSVVPSDKGNYTCIVENEYGSINHTYQLDV..............PANKTVALGSNVEFMCKVYSDPQPHIQWLKHIE 112
PF07679.4     NPTLRWLKNGKEFKPDHRIGGYKVRYATWSIIMDSVVPSDKGNYTC................................................................  63
PF07679.3     NPTLRWLKNGKEFKPDHRIGGYKVRYATWSIIMDSVVPSDKGNYTCIVENEYGSINHTYQLDV...............................................  79
PF07679.2     NPTLRWLKNGKEFKPDHRIGGYKVRYATWSIIMDSVVPSDKGNYTCIVENE...........................................................  68
PF00047       ..............................................................................................................  73
PF00047.1     ..............................................................................................................  62
PF07714       ..............................................................................................................   0
PF07679       NPTLRWLKNGKEFKPDHRIGGYKVRYATWSIIMDSVVPSDKGNYTCIVENEYGSINHTYQLDV..............PANKTVALGSNVEFMCKVYSDPQPHIQWLKHIE 112
FGFR1-236     ..............................................................................................................   0
FGFR1-214     NPTLRWLKNGKEFKPDHRIGGYKVRYATWSIIMDSVVPSDKGNYTCIVENEYGSINHTYQLDVVERSPHRPILQAGLPANKTVALGSNVEFMCKVYSDPQPHIQWLKHIE 294
FGFR1-202     NPTLRWLKNGKEFKPDHRIGGYKVRYATWSIIMDSVVPSDKGNYTCIVENEYGSINHTYQLDVVERSPHRPILQAGLPANKTVALGSNVEFMCKVYSDPQPHIQWLKHIE 286
FGFR1-201     NPTLRWLKNGKEFKPDHRIGGYKVRYATWSIIMDSVVPSDKGNYTCIVENEYGSINHTYQLDVVERSPHRPILQAGLPANKTVALGSNVEFMCKVYSDPQPHIQWLKHIE 203
FGFR1-241     NPTLRWLKNGKEFKPDHRIGGYKVRYATWSIIMDSVVPSDKGNYTCIVENEYGSINHTYQLDVVERSPHRPILQAGLPANKTVALGSNVEFMCKVYSDPQPHIQWLKHIE 203
FGFR1-209     NPTLRWLKNGKEFKPDHRIGGYKVRYATWSIIMDSVVPSDKGNYTCIVENEYGSINHTYQLDVVERSPHRPILQAGLPANKTVALGSNVEFMCKVYSDPQPHIQWLKHIE 292
FGFR1-238     NPTLRWLKNGKEFKPDHRIGGYKVRYATWSIIMDSVVPSDKGNYTCIVENEYGSINHTYQLDVVERSPHRPILQAGLPANKTVALGSNVEFMCKVYSDPQPHIQWLKHIE 294
FGFR1-210     .............................................................................................................. 106
FGFR1-208     NPTLRWLKNGKEFKPDHRIGGYKVRYATWSIIMDSVVPSDKGNYTCIVENEYGSINHTYQLDVVERSPHRPILQAGLPANKTVALGSNVEFMCKVYSDPQPHIQWLKHIE 292
FGFR1-212     ..............................................................................................................  54
FGFR1-204     NPTLRWLKNGKEFKPDHRIGGYKVRYATWSIIMDSVVPSDKGNYTCIVENEYGSINHTYQLDVVERSPHRPILQAGLPANKTVALGSNVEFMCKVYSDPQPHIQWLKHIE 205
FGFR1-221     .............................................................................................................. 150
FGFR1-207     NPTLRWLKNGKEFKPDHRIGGYKVRYATWSIIMDSVVPSDKGNYTCIVENEYGSINHTYQLDVVERSPHRPILQAGLPANKTVALGSNVEFMCKVYSDPQPHIQWLKHIE 203
FGFR1-213     ..............................................................................................................   0
FGFR1-226     NPTLRWLKNGKEFKPDHRIGGYKVRYATWSIIMDSVVPSDKGNYTCIVENEYGSINHTYQLDVVERSPHRPILQAGLPANKTVALGSNVEFMCKVYSDPQPHIQWLKHIE 294
FGFR1-229     NPTLRWLKNGKEFKPDHRIGGYKVRYATWSIIMDSVVPSDKGNYTCIVENEY.......................................................... 145
FGFR1-211     NPTLRWLKNGKEFKPDHRIGGYKVRYATWSIIMDSVVPSDKGNYTCIVENEYGSINHTYQLDVVERSPHRPILQAGLPANKTVALGSNVEFMCKVYSDPQPHIQWLKHIE 325
FGFR1-234     ..............................................................................................................  85
FGFR1-203     NPTLRWLKNGKEFKPDHRIGGYKVRYATWSIIMDSVVPSDKGNYTCIVENEYGSINHTYQLDVVERSPHRPILQAGLPANKTVALGSNVEFMCKVYSDPQPHIQWLKHIE 295
FGFR1-240     NPTLRWLKNGKEFKPDHRIGGYKVRYATWSIIMDSVVPSDKGNYTCIVENEYGSINHTYQLDVVERSPHRPILQAGLPANKTVALGSNVEFMCKVYSDPQPHIQWLKHIE 134
FGFR1-222     ..............................................................................................................   0
FGFR1-233     NPTLRWLKNGKEFKPDHRIGGYKVRYATWSIIMDSVVPSDKGNYTCI............................................................... 142
FGFR1-206     NPTLRWLKNGKEFKPDHRIGGYKVRYATWSIIMDSVVPSDKGNYTCIVENEYGSINHTYQLDVVERSPHRPILQAGLPANKTVALGSNVEFMCKVYSDPQPHIQWLKHIE 292
```

logo

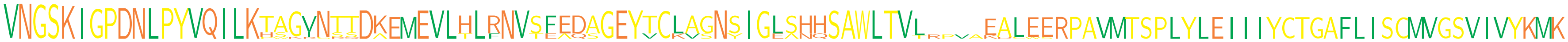

```
INFIGRATINIB  .............................................................................................................. 0
PF07679.1     VNGSKIGPDNLPYVQILKHSGINSSDA..EVLTLFNVTEAQSGEYVCKVSNYIGEANQSAWLTV.............................................. 174
PF07679.4     .............................................................................................................. 63
PF07679.3     .............................................................................................................. 79
PF07679.2     .............................................................................................................. 68
PF00047       .............................................................................................................. 73
PF00047.1     .............................................................................................................. 62
PF07714       .............................................................................................................. 0
PF07679       VNGSKIGPDNLPYVQILKTAGVNTTDKEMEVLHLRNVSFEDAGEYTCLAGNSIGLSHHSAWLTV.............................................. 176
FGFR1-236     .............................................................................................................. 0
FGFR1-214     VNGSKIGPDNLPYVQILKTAGVNTTDKEMEVLHLRNVSFEDAGEYTCLAGNSIGLSHHSAWLTVL....EALEERPAVMTSPLYLEIIIYCTGAFLISCMVGSVIVYKMK 400
FGFR1-202     VNGSKIGPDNLPYVQILKTAGVNTTDKEMEVLHLRNVSFEDAGEYTCLAGNSIGLSHHSAWLTVL....EALEERPAVMTSPLYLEIIIYCTGAFLISCMVGSVIVYKMK 392
FGFR1-201     VNGSKIGPDNLPYVQILKTAGVNTTDKEMEVLHLRNVSFEDAGEYTCLAGNSIGLSHHSAWLTVL....EALEERPAVMTSPLYLEIIIYCTGAFLISCMVGSVIVYKMK 309
FGFR1-241     VNGSKIGPDNLPYVQILKPWKRGRQ..................................................................................... 228
FGFR1-209     VNGSKIGPDNLPYVQILKTAGVNTTDKEMEVLHLRNVSFEDAGEYTCLAGNSIGLSHHSAWLTVL....EALEERPAVMTSPLYLEIIIYCTGAFLISCMVGSVIVYKMK 398
FGFR1-238     VNGSKIGPDNLPYVQILKTAGVNTTDKEMEVLHLRNVSFEDAGEYTCLAGNSIGLSHHSAWLTVL....EALEERPAVMTSPLYLEIIIYCTGAFLISCMVGSVIVYKMK 400
FGFR1-210     .............................................................................................................. 106
FGFR1-208     VNGSKIGPDNLPYVQILKTAGVNTTDKEMEVLHLRNVSFEDAGEYTCLAGNSIGLSHHSAWLTVL....EALEERPAVMTSPLYLEIIIYCTGAFLISCMVGSVIVYKMK 398
FGFR1-212     .............................................................................................................. 54
FGFR1-204     VNGSKIGPDNLPYVQILKTAGVNTTDKEMEVLHLRNVSFEDAGEYTCLAGNSIGLSHHSAWLTVL....EALEERPAVMTSPLYLEIIIYCTGAFLISCMVGSVIVYKMK 311
FGFR1-221     .............................................................................................................. 150
FGFR1-207     VNGSKIGPDNLPYVQILKHSGINSSDA..EVLTLFNVTEAQSGEYVCKVSNYIGEANQSAWLTVTRPVAKALEERPAVMTSPLYLEIIIYCTGAFLISCMVGSVIVYKMK 311
FGFR1-213     .............................................................................................................. 0
FGFR1-226     VNGS.......................................................................................................... 298
FGFR1-229     .............................................................................................................. 145
FGFR1-211     VNGSKIGPDNLPYVQILKTAGVNTTDKEMEVLHLRNVSFEDAGEYTCLAGNSIGLSHHSAWLTVL....EALEERPAVMTSPLYLEIIIYCTGAFLISCMVGSVIVYKMK 431
FGFR1-234     .............................................................................................................. 85
FGFR1-203     VNGSKIGPDNLPYVQILKHSGINSSDA..EVLTLFNVTEAQSGEYVCKVSNYIGEANQSAWLTVTRPVAKDCWS.................................... 367
FGFR1-240     VNGSK......................................................................................................... 139
FGFR1-222     .............................................................................................................. 0
FGFR1-233     .............................................................................................................. 142
FGFR1-206     VNGSKIGPDNLPYVQILKTAGVNTTDKEMEVLHLRNVSFEDAGEYTCLAGNSIGLSHHSAWLTVL....EALEERPAVMTSPLYLEIIIYCTGAFLISCMVGSVIVYKMK 398
```

```
logo
               SGTKKSDFHSQMAVHKLAKSIPLRRQVTVSADSSASMNSGVLLVRPSRLSSSGTPMLAGVSEYELPEDPRWELPRDRLVLGKPLGEGCFGQVVLAEAIGLDKDKPNRVTK
INFIGRATINIB   ..............................................................................................................   0
PF07679.1      ..............................................................................................................   174
PF07679.4      ..............................................................................................................   63
PF07679.3      ..............................................................................................................   79
PF07679.2      ..............................................................................................................   68
PF00047        ..............................................................................................................   73
PF00047.1      ..............................................................................................................   62
PF07714        ..............................................................................VLGKPLGEGCFGQVVLAEAIGLDKDKPNRVTK   32
PF07679        ..............................................................................................................   176
FGFR1-236      ..............................................................................................................   0
FGFR1-214      SGTKKSDFHSQMAVHKLAKSIPLRRQVTVSADSSASMNSGVLLVRPSRLSSSGTPMLAGVSEYELPEDPRWELPRDRLVLGKPLGEGCFGQVVLAEAIGLDKDKPNRVTK   510
FGFR1-202      SGTKKSDFHSQMAVHKLAKSIPLRRQV..SADSSASMNSGVLLVRPSRLSSSGTPMLAGVSEYELPEDPRWELPRDRLVLGKPLGEGCFGQVVLAEAIGLDKDKPNRVTK   500
FGFR1-201      SGTKKSDFHSQMAVHKLAKSIPLRRQVTVSADSSASMNSGVLLVRPSRLSSSGTPMLAGVSEYELPEDPRWELPRDRLVLGKPLGEGCFGQVVLAEAIGLDKDKPNRVTK   419
FGFR1-241      ..............................................................................................................   228
FGFR1-209      SGTKKSDFHSQMAVHKLAKSIPLRRQVTVSADSSASMNSGVLLVRPSRLSSSGTPMLAGVSEYELPEDPRWELPRDRLVLGKPLGEGCFGQVVLAEAIGLDKDKPNRVTK   508
FGFR1-238      SGTKKSDFHSQMAVHKLAKSIPLRRQV..SADSSASMNSGVLLVRPSRLSSSGTPMLAGVSEYELPEDPRWELPRDRLVLGKPLGEGCFGQVVLAEAIGLDKDKPNRVTK   508
FGFR1-210      ..............................................................................................................   106
FGFR1-208      SGTKKSDFHSQMAVHKLAKSIPLRRQVTVSADSSASMNSGVLLVRPSRLSSSGTPMLAGVSEYELPEDPRWELPRDRLVLGKPLGEGCFGQVVLAEAIGLDKDKPNRVTK   508
FGFR1-212      ..............................................................................................................   54
FGFR1-204      SGTKKSDFHSQMAVHKLAKSIPLRRQVTVSADSSASMNSGVLLVRPSRLSSSGTPMLAGVSEYELPEDPRWELPRDRLVLGKPLGEGCFGQVVLAEAIGLDKDKPNRVTK   421
FGFR1-221      ..............................................................................................................   150
FGFR1-207      SGTKKSDFHSQMAVHKLAKSIPLRRQVTVSADSSASMNSGVLLVRPSRLSSSGTPMLAGVSEYELPEDPRWELPRDRLVLGKPLGEGCFGQVVLAEAIGLDKDKPNRVTK   421
FGFR1-213      ..............................................................................................................   0
FGFR1-226      ..............................................................................................................   298
FGFR1-229      ..............................................................................................................   145
FGFR1-211      SGTKKSDFHSQMAVHKLAKSIPLRRQVTVSADSSASMNSGVLLVRPSRLSSSGTPMLAGVSEYELPEDPRWELPRDRLVLGKPLGEGCFGQVVLAEAIGLDKDKPNRVTK   541
FGFR1-234      ..............................................................................................................   85
FGFR1-203      ..............................................................................................................   367
FGFR1-240      ..............................................................................................................   139
FGFR1-222      ..............................................................................................................   0
FGFR1-233      ..............................................................................................................   142
FGFR1-206      SGTKKSDFHSQMAVHKLAKSIPLRRQVTVSADSSASMNSGVLLVRPSRLSSSGTPMLAGVSEYELPEDPRWELPRDRLVLGKPLGEGCFGQVVLAEAIGLDKDKPNRVTK   508
```

```
logo
                VAVKMLKSDATEKDLSDLISEMEMMKMIGKHKNIINLLGACTQDGPLYVIVEYASKGNLREYLQARRPPGLEYCYNPSHNPEEQLSSKDLVSCAYQV      ARGMEYL
INFIGRATINIB    ..................................................................................L.......V...................    2
PF07679.1       ..............................................................................................................  174
PF07679.4       ..............................................................................................................   63
PF07679.3       ..............................................................................................................   79
PF07679.2       ..............................................................................................................   68
PF00047         ..............................................................................................................   73
PF00047.1       ..............................................................................................................   62
PF07714         VAVKMLKSDATEKDLSDLISEMEMMKMIGKHKNIINLLGACTQDGPLYVIVEYASKGNLREYLQARRPPGLEYCYNPSHNPEEQLSSKDLVSCAYQV......ARGMEYL  136
PF07679         ..............................................................................................................  176
FGFR1-236       ..........................................................................................VSCAYQV......ARGMEYL   14
FGFR1-214       VAVKMLKSDATEKDLSDLISEMEMMKMIGKHKNIINLLGACTQDGPLYVIVEYASKGNLREYLQARRPPGLEYCYNPSHNPEEQLSSKDLVSCAYQV......ARGMEYL  614
FGFR1-202       VAVKMLKSDATEKDLSDLISEMEMMKMIGKHKNIINLLGACTQDGPLYVIVEYASKGNLREYLQARRPPGLEYCYNPSHNPEEQLSSKDLVSCAYQV......ARGMEYL  604
FGFR1-201       VAVKMLKSDATEKDLSDLISEMEMMKMIGKHKNIINLLGACTQDGPLYVIVEYASKGNLREYLQARRPPGLEYCYNPSHNPEEQLSSKDLVSCAYQV......ARGMEYL  523
FGFR1-241       ..............................................................................................................  228
FGFR1-209       VAVKMLKSDATEKDLSDLISEMEMMKMIGKHKNIINLLGACTQDGPLYVIVEYASKGNLREYLQARRPPGLEYCYNPSHNPEEQLSSKDLVSCAYQV......ARGMEYL  612
FGFR1-238       VAVKMLKSDATEKDLSDLISEMEMMKMIGKHKNIINLLGACTQDGPLYVIVEYASKGNLREYLQARRPPGLEYCYNPSHNPEEQLSSKDLVSCAYQV......ARGMEYL  612
FGFR1-210       ..............................................................................................................  106
FGFR1-208       VAVKMLKSDATEKDLSDLISEMEMMKMIGKHKNIINLLGACTQDGPLYVIVEYASKGNLREYLQARRPPGLEYCYNPSHNPEEQLSSKDLVSCAYQV......ARGMEYL  612
FGFR1-212       ..............................................................................................................   54
FGFR1-204       VAVKMLKSDATEKDLSDLISEMEMMKMIGKHKNIINLLGACTQDGPLYVIVEYASKGNLREYLQARRPPGLEYCYNPSHNPEEQLSSKDLVSCAYQV......ARGMEYL  525
FGFR1-221       ..............................................................................................................  150
FGFR1-207       VAVKMLKSDATEKDLSDLISEMEMMKMIGKHKNIINLLGACTQDGPLYVIVEYASKGNLREYLQARRPPGLEYCYNPSHNPEEQLSSKDLVSCAYQV......ARGMEYL  525
FGFR1-213       ..............................................................................................................    0
FGFR1-226       ..............................................................................................................  298
FGFR1-229       ..............................................................................................................  145
FGFR1-211       VAVKMLKSDATEKDLSDLISEMEMMKMIGKHKNIINLLGACTQDGPLYVIVEYASKGNLREYLQARRPPGLEYCYNPSHNPEEQLSSKDLVSCAYQV......ARGMEYL  645
FGFR1-234       ..............................................................................................................   85
FGFR1-203       ..............................................................................................................  367
FGFR1-240       ..............................................................................................................  139
FGFR1-222       ..............................................................................................................    0
FGFR1-233       ..............................................................................................................  142
FGFR1-206       VAVKMLKSDATEKDLSDLISEMEMMKMIGKHKNIINLLGACTQDGPLYVIVEYASKGNLREYLQARRPPGLEYCYNPSHNPEEQLSSKDLVSCAYQV......ARGMEYL  612
```

```
logo
              ASKKCIHRDL AARNVLVTEDNVMKIADFGLARDIHHIDYYKKTTNGRLPVKWMAPEALFDRIYTHQSDVWSFGVL LWEIFTLGGS    PYPGVPVEELFKLLKEGHR
INFIGRATINIB  A.K................E...M..........I.............V.VEYA..G...E.................................................  14
PF07679.1     ..............................................................................................................  174
PF07679.4     ..............................................................................................................  63
PF07679.3     ..............................................................................................................  79
PF07679.2     ..............................................................................................................  68
PF00047       ..............................................................................................................  73
PF00047.1     ..............................................................................................................  62
PF07714       ASKKCIHRDL.AARNVLVTEDNVMKIADFGLARDIHHIDYYKKTTNGRLPVKWMAPEALFDRIYTHQSDVWSFGVL.LWEIFTLGGS....PYPGVPVEELFKLLKEGHR  240
PF07679       ..............................................................................................................  176
FGFR1-236     ASKKCIHRDL.AARNVLVTEDNVMKIADFGLARDIHHIDYYKKTTNGRLPVKWMAPEALFDRIYTHQSDVWSFGVL.LWEIFTLGGS....PYPGVPVEELFKLLKEGHR  118
FGFR1-214     ASKKCIHRDL.AARNVLVTEDNVMKIADFGLARDIHHIDYYKKTTNGRLPVKWMAPEALFDRIYTHQSDVWSFGVL.LWEIFTLGGS....PYPGVPVEELFKLLKEGHR  718
FGFR1-202     ASKKCIHRDL.AARNVLVTEDNVMKIADFGLARDIHHIDYYKKTTNGRLPVKWMAPEALFDRIYTHQSDVWSFGVL.LWEIFTLGGS....PYPGVPVEELFKLLKEGHR  708
FGFR1-201     ASKKCIHRDL.AARNVLVTEDNVMKIADFGLARDIHHIDYYKKTTNGRLPVKWMAPEALFDRIYTHQSDVWSFGVL.LWEIFTLGGS....PYPGVPVEELFKLLKEGHR  627
FGFR1-241     ..............................................................................................................  228
FGFR1-209     ASKKCIHRDL.AARNVLVTEDNVMKIADFGLARDIHHIDYYKKTTNGRLPVKWMAPEALFDRIYTHQSDVWSFGVL.LWEIFTLGGS....PYPGVPVEELFKLLKEGHR  716
FGFR1-238     ASKKCIHRDL.AARNVLVTEDNVMKIADFGLARDIHHIDYYKKTTNGRLPVKWMAPEALFDRIYTHQSDVWSFGVL.LWEIFTLGGS....PYPGVPVEELFKLLKEGHR  716
FGFR1-210     ..............................................................................................................  106
FGFR1-208     ASKKCIHRDL.AARNVLVTEDNVMKIADFGLARDIHHIDYYKKTTNGRLPVKWMAPEALFDRIYTHQSDVWSFGVL.LWEIFTLGGS....PYPGVPVEELFKLLKEGHR  716
FGFR1-212     ..............................................................................................................  54
FGFR1-204     ASKKCIHRDL.AARNVLVTEDNVMKIADFGLARDIHHIDYYKKTTNGRLPVKWMAPEALFDRIYTHQSDVWSFGVL.LWEIFTLGGS....PYPGVPVEELFKLLKEGHR  629
FGFR1-221     ..............................................................................................................  150
FGFR1-207     ASKKCIHRDL.AARNVLVTEDNVMKIADFGLARDIHHIDYYKKTTNGRLPVKWMAPEALFDRIYTHQSDVWSFGVL.LWEIFTLGGS....PYPGVPVEELFKLLKEGHR  629
FGFR1-213     .......................M......................................................................................  1
FGFR1-226     ..............................................................................................................  298
FGFR1-229     ..............................................................................................................  145
FGFR1-211     ASKKCIHRDL.AARNVLVTEDNVMKIADFGLARDIHHIDYYKKTTNGRLPVKWMAPEALFDRIYTHQSDVWSFGVL.LWEIFTLGGS....PYPGVPVEELFKLLKEGHR  749
FGFR1-234     ..............................................................................................................  85
FGFR1-203     ..............................................................................................................  367
FGFR1-240     ..............................................................................................................  139
FGFR1-222     .....................................................................MWSWKCLLFWAVLVTATLCTARPSPTLPEQACPDLQE....  37
FGFR1-233     ..............................................................................................................  142
FGFR1-206     ASKKCIHRDL.AARNVLVTEDNVMKIADFGLARDIHHIDYYKKTTNGRLPVKWMAPEALFDRIYTHQSDVWSFGVL.LWEIFTLGGS....PYPGVPVEELFKLLKEGHR  716
```

```
logo          MDKPSNCTNELYMMMRDCWHAVPSQRPTFKQLVEDLDRIVALTSNQEYLDLSMPLDQYSPSFPDTRSSTCSSGEDSVFSHEPLPEEPCLPRHPAQLANGGLKRR
INFIGRATINIB  ..........L......................AD.....................................................................   17
PF07679.1     ........................................................................................................  174
PF07679.4     ........................................................................................................   63
PF07679.3     ........................................................................................................   79
PF07679.2     ........................................................................................................   68
PF00047       ........................................................................................................   73
PF00047.1     ........................................................................................................   62
PF07714       MDKPSNCTNELYMMMRDCWHAVPSQRPTFKQLVEDL....................................................................  276
PF07679       ........................................................................................................  176
FGFR1-236     MDKPSNCTNELPAA.....GT...................................................................................  134
FGFR1-214     MDKPSNCTNELYMMMRDCWHAVPSQRPTFKQLVEDLDRIVALTSNQEYLDLSMPLDQYSPSFPDTRSSTCSSGEDSVFSHEPLPEEPCLPRHPAQLANGGLKRR  822
FGFR1-202     MDKPSNCTNELYMMMRDCWHAVPSQRPTFKQLVEDLDRIVALTSNQEYLDLSMPLDQYSPSFPDTRSSTCSSGEDSVFSHEPLPEEPCLPRHPAQLANGGLKRR  812
FGFR1-201     MDKPSNCTNELYMMMRDCWHAVPSQRPTFKQLVEDLDRIVALTSNQEYLDLSMPLDQYSPSFPDTRSSTCSSGEDSVFSHEPLPEEPCLPRHPAQLANGGLKRR  731
FGFR1-241     ........................................................................................................  228
FGFR1-209     MDKPSNCTNELYMMMRDCWHAVPSQRPTFKQLVEDLDRIVALTSNQEYLDLSMPLDQYSPSFPDTRSSTCSSGEDSVFSHEPLPEEPCLPRHPAQLANGGLKRR  820
FGFR1-238     MDKPSNCTNELYMMMRDCWHAVPSQRPTFKQLVEDLDRIVALTSNQEYLDLSMPLDQYSPSFPDTRSSTCSSGEDSVFSHEPLPEEPCLPRHPAQLANGGLKRR  820
FGFR1-210     ........................................................................................................  106
FGFR1-208     MDKPSNCTNELYMMMRDCWHAVPSQRPTFKQLVEDLDRIVALTSNQEYLDLSMPLDQYSPSFPDTRSSTCSSGEDSVFSHEPLPEEPCLPRHPAQLANGGLKRR  820
FGFR1-212     ........................................................................................................   54
FGFR1-204     MDKPSNCTNELYMMMRDCWHAVPSQRPTFKQLVEDLDRIVALTSNQEYLDLSMPLDQYSPSFPDTRSSTCSSGEDSVFSHEPLPEEPCLPRHPAQLANGGLKRR  733
FGFR1-221     ........................................................................................................  150
FGFR1-207     MDKPSNCTNELYMMMRDCWHAVPSQRPTFKQLVEDLDRIVALTSNQEYLDLSMPLDQYSPSFPDTRSSTCSSGEDSVFSHEPLPEEPCLPRHPAQLANGGLKRR  733
FGFR1-213     ........................................................................................................    1
FGFR1-226     ........................................................................................................  298
FGFR1-229     ........................................................................................................  145
FGFR1-211     MDKPSNCTNELYMMMRDCWHAVPSQRPTFKQLVEDLDRIVALTSNQEYLDLSMPLDQYSPSFPDTRSSTCSSGEDSVFSHEPLPEEPCLPRHPAQLANGGLKRR  853
FGFR1-234     ........................................................................................................   85
FGFR1-203     ........................................................................................................  367
FGFR1-240     ........................................................................................................  139
FGFR1-222     ...AKWCSASFHSITPLPFGLGTR.......L.SD.....................................................................   61
FGFR1-233     ........................................................................................................  142
FGFR1-206     MDKPSNCTNELYMMMRDCWHAVPSQRPTFKQLVEDLDRIVALTSNQEYLDLSMPLDQYSPSFPDTRSSTCSSGEDSVFSHEPLPEEPCLPRHPAQLANGGLKRR  820
```

X non conserved

X similar

X $\geq$ 0% conserved

X $\geq$ 50% conserved

logo 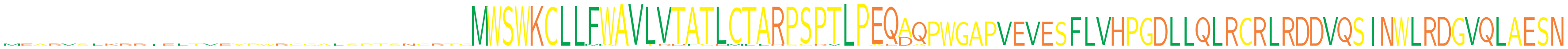

```
JNJ-42756493  ..............................................................................................................    0
PF07679.1     ..............................................................................................................    0
PF07679.4     ..............................................................................................................    0
PF07679.3     ..............................................................................................................    0
PF07679.2     ..............................................................................................................    0
PF00047       ...........................................................................FLVHPGDLLQLRCRLRDDVQSINWLRDGVQLAESN   35
PF00047.1     ...........................................................................FLVHPGDLLQLRCRLRDDVQSINWLRDGVQLAESN   35
PF07714       ..............................................................................................................    0
PF07679       ..............................................................................................................    0
FGFR1-236     ..............................................................................................................    0
FGFR1-214     .................................MWSWKCLLFWAVLVTATLCTARPSPTLPEQAQPWGAPVEVESFLVHPGDLLQLRCRLRDDVQSINWLRDGVQLAESN   77
FGFR1-202     .........................................MAAVTRDFGEMLLHSGRVLPAEAQPWGAPVEVESFLVHPGDLLQLRCRLRDDVQSINWLRDGVQLAESN   69
FGFR1-201     .................................MWSWKCLLFWAVLVTATLCTARPSPTLPEQ...............................................   30
FGFR1-241     .................................MWSWKCLLFWAVLVTATLCTARPSPTLPEQ...............................................   30
FGFR1-209     .................................MWSWKCLLFWAVLVTATLCTARPSPTLPEQAQPWGAPVEVESFLVHPGDLLQLRCRLRDDVQSINWLRDGVQLAESN   77
FGFR1-238     .................................MWSWKCLLFWAVLVTATLCTARPSPTLPEQAQPWGAPVEVESFLVHPGDLLQLRCRLRDDVQSINWLRDGVQLAESN   77
FGFR1-210     .................................MWSWKCLLFWAVLVTATLCTARPSPTLPEQAQPWGAPVEVESFLVHPGDLLQLRCRLRDDVQSINWLRDGVQLAESN   77
FGFR1-208     .................................MWSWKCLLFWAVLVTATLCTARPSPTLPEQAQPWGAPVEVESFLVHPGDLLQLRCRLRDDVQSINWLRDGVQLAESN   77
FGFR1-212     .................................MWSWKCLLFWAVLVTATLCTARPSPTLPEQDA.............................................   32
FGFR1-204     .................................MWSWKCLLFWAVLVTATLCTARPSPTLPEQD..............................................   31
FGFR1-221     .................................MWSWKCLLFWAVLVTATLCTARPSPTLPEQAQPWGAPVEVESFLVHPGDLLQLRCRLRDDVQSINWLRDGVQLAESN   77
FGFR1-207     .................................MWSWKCLLFWAVLVTATLCTARPSPTLPEQD..............................................   31
FGFR1-213     ..............................................................................................................    0
FGFR1-226     .................................MWSWKCLLFWAVLVTATLCTARPSPTLPEQAQPWGAPVEVESFLVHPGDLLQLRCRLRDDVQSINWLRDGVQLAESN   77
FGFR1-229     .................................MWSWKCLLFWAVLVTATLCTARPSPTLPEQD..............................................   31
FGFR1-211     MEARVSLKRRIELTVEYPWRCGALSPTSNCRTGMWSWKCLLFWAVLVTATLCTARPSPTLPEQAQPWGAPVEVESFLVHPGDLLQLRCRLRDDVQSINWLRDGVQLAESN  110
FGFR1-234     .................................MWSWKCLLFWAVLVTATLCTARPSPTLPEQD..............................................   31
FGFR1-203     .................................MWSWKCLLFWAVLVTATLCTARPSPTLPEQAQPWGAPVEVESFLVHPGDLLQLRCRLRDDVQSINWLRDGVQLAESN   77
FGFR1-240     ..............................................................................................................    0
FGFR1-222     ..............................................................................................................    0
FGFR1-233     .................................MWSWKCLLFWAVLVTATLCTARPSPTLPEQ...............................................   30
FGFR1-206     .................................MWSWKCLLFWAVLVTATLCTARPSPTLPEQAQPWGAPVEVESFLVHPGDLLQLRCRLRDDVQSINWLRDGVQLAESN   77
```

logo

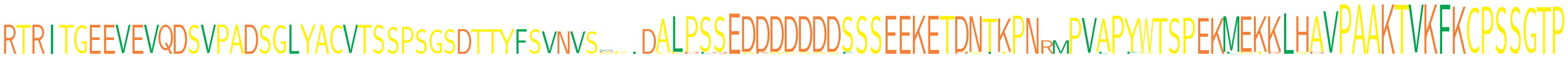

```
JNJ-42756493  ..............................................................................................................   0
PF07679.1     ..............................................................................................PAAKTVKFKCPSSGTP   16
PF07679.4     .............................................................................................VPAAKTVKFKCPSSGTP   17
PF07679.3     ..............................................................................................PAAKTVKFKCPSSGTP   16
PF07679.2     .............................................................................................VPAAKTVKFKCPSSGTP   17
PF00047       RTRITGEEVEVQDSVPADSGLYACVTSSPSGSDTTYFS........................................................................   73
PF00047.1     RTRITGEEVEVQDSVPADSGLYACVTS...................................................................................   62
PF07714       ..............................................................................................................   0
PF07679       ..............................................................................................PAAKTVKFKCPSSGTP   16
FGFR1-236     ..............................................................................................................   0
FGFR1-214     RTRITGEEVEVQDSVPADSGLYACVTSSPSGSDTTYFSVNVS...DALPSSEDDDDDDDSSSEEKETDNTKPNRMPVAPYWTSPEKMEKKLHAVPAAKTVKFKCPSSGTP   184
FGFR1-202     RTRITGEEVEVQDSVPADSGLYACVTSSPSGSDTTYFSVNVSD...ALPSSEDDDDDDDSSSEEKETDNTKPNRMPVAPYWTSPEKMEKKLHAVPAAKTVKFKCPSSGTP   176
FGFR1-201     .............................................DALPSSEDDDDDDDSSSEEKETDNTKPN..PVAPYWTSPEKMEKKLHAVPAAKTVKFKCPSSGTP   93
FGFR1-241     .............................................DALPSSEDDDDDDDSSSEEKETDNTKPN..PVAPYWTSPEKMEKKLHAVPAAKTVKFKCPSSGTP   93
FGFR1-209     RTRITGEEVEVQDSVPADSGLYACVTSSPSGSDTTYFSVNVS...DALPSSEDDDDDDDSSSEEKETDNTKPN..PVAPYWTSPEKMEKKLHAVPAAKTVKFKCPSSGTP   182
FGFR1-238     RTRITGEEVEVQDSVPADSGLYACVTSSPSGSDTTYFSVNVS...DALPSSEDDDDDDDSSSEEKETDNTKPNRMPVAPYWTSPEKMEKKLHAVPAAKTVKFKCPSSGTP   184
FGFR1-210     RTRITGEEVEVQDSVPADSGLYACVTSSP.................................................................................   106
FGFR1-208     RTRITGEEVEVQDSVPADSGLYACVTSSPSGSDTTYFSVNVS...DALPSSEDDDDDDDSSSEEKETDNTKPN..PVAPYWTSPEKMEKKLHAVPAAKTVKFKCPSSGTP   182
FGFR1-212     ...............................................LPSSEDDDDDDDSSSEEKETDN.........................................   54
FGFR1-204     ..............................................ALPSSEDDDDDDDSSSEEKETDNTKPNRMPVAPYWTSPEKMEKKLHAVPAAKTVKFKCPSSGTP   95
FGFR1-221     RTRITGEEVEVQDSVPADSGLYACVTSSPSGSDTTYFSVNVSA...CPDLQEAKWCSASF......HSITP.....LPFG.....LGTRLSD..................   150
FGFR1-207     ..............................................ALPSSEDDDDDDDSSSEEKETDNTKPN..PVAPYWTSPEKMEKKLHAVPAAKTVKFKCPSSGTP   93
FGFR1-213     ..............................................................................................................   0
FGFR1-226     RTRITGEEVEVQDSVPADSGLYACVTSSPSGSDTTYFSVNVS...DALPSSEDDDDDDDSSSEEKETDNTKPNRMPVAPYWTSPEKMEKKLHAVPAAKTVKFKCPSSGTP   184
FGFR1-229     ..............................................ALPSSEDDDDDDDSSSEEKETDNTKPN..PVAPYWTSPEKMEKKLHAVPAAKTVKFKCPSSGTP   93
FGFR1-211     RTRITGEEVEVQDSVPADSGLYACVTSSPSGSDTTYFSVNVS...DALPSSEDDDDDDDSSSEEKETDNTKPN..PVAPYWTSPEKMEKKLHAVPAAKTVKFKCPSSGTP   215
FGFR1-234     ..............................................ALPSSEDDDDDDDSSSEEKETDNTKPN..PVAPYWTSPEKMEKKLHAVPAAKTVKF........   85
FGFR1-203     RTRITGEEVEVQDSVPADSGLYACVTSSPSGSDTTYFSVNVSVPIDALPSSEDDDDDDDSSSEEKETDNTKPN..PVAPYWTSPEKMEKKLHAVPAAKTVKFKCPSSGTP   185
FGFR1-240     ......................................................................................MEKKLHAVPAAKTVKFKCPSSGTP   24
FGFR1-222     ..............................................................................................................   0
FGFR1-233     .............................................DALPSSEDDDDDDDSSSEEKETDNTKPNRMPVAPYWTSPEKMEKKLHAVPAAKTVKFKCPSSGTP   95
FGFR1-206     RTRITGEEVEVQDSVPADSGLYACVTSSPSGSDTTYFSVNVS...DALPSSEDDDDDDDSSSEEKETDNTKPN..PVAPYWTSPEKMEKKLHAVPAAKTVKFKCPSSGTP   182
```

```
logo           NPTLRWLKNGKEFKPDHRIGGYKVRYATWSIIMDSVVPSDKGNYTCIVENEYGSINHTYQLDVVERSPHRPILQAGLPANKTVALGSNVEFMCKVYSDPQPHIQWLKHIE
JNJ-42756493   ..............................................................................................................   0
PF07679.1      NPTLRWLKNGKEFKPDHRIGGYKVRYATWSIIMDSVVPSDKGNYTCIVENEYGSINHTYQLDV..............PANKTVALGSNVEFMCKVYSDPQPHIQWLKHIE 112
PF07679.4      NPTLRWLKNGKEFKPDHRIGGYKVRYATWSIIMDSVVPSDKGNYTC................................................................  63
PF07679.3      NPTLRWLKNGKEFKPDHRIGGYKVRYATWSIIMDSVVPSDKGNYTCIVENEYGSINHTYQLDV...............................................  79
PF07679.2      NPTLRWLKNGKEFKPDHRIGGYKVRYATWSIIMDSVVPSDKGNYTCIVENE...........................................................  68
PF00047        ..............................................................................................................  73
PF00047.1      ..............................................................................................................  62
PF07714        ..............................................................................................................   0
PF07679        NPTLRWLKNGKEFKPDHRIGGYKVRYATWSIIMDSVVPSDKGNYTCIVENEYGSINHTYQLDV..............PANKTVALGSNVEFMCKVYSDPQPHIQWLKHIE 112
FGFR1-236      ..............................................................................................................   0
FGFR1-214      NPTLRWLKNGKEFKPDHRIGGYKVRYATWSIIMDSVVPSDKGNYTCIVENEYGSINHTYQLDVVERSPHRPILQAGLPANKTVALGSNVEFMCKVYSDPQPHIQWLKHIE 294
FGFR1-202      NPTLRWLKNGKEFKPDHRIGGYKVRYATWSIIMDSVVPSDKGNYTCIVENEYGSINHTYQLDVVERSPHRPILQAGLPANKTVALGSNVEFMCKVYSDPQPHIQWLKHIE 286
FGFR1-201      NPTLRWLKNGKEFKPDHRIGGYKVRYATWSIIMDSVVPSDKGNYTCIVENEYGSINHTYQLDVVERSPHRPILQAGLPANKTVALGSNVEFMCKVYSDPQPHIQWLKHIE 203
FGFR1-241      NPTLRWLKNGKEFKPDHRIGGYKVRYATWSIIMDSVVPSDKGNYTCIVENEYGSINHTYQLDVVERSPHRPILQAGLPANKTVALGSNVEFMCKVYSDPQPHIQWLKHIE 203
FGFR1-209      NPTLRWLKNGKEFKPDHRIGGYKVRYATWSIIMDSVVPSDKGNYTCIVENEYGSINHTYQLDVVERSPHRPILQAGLPANKTVALGSNVEFMCKVYSDPQPHIQWLKHIE 292
FGFR1-238      NPTLRWLKNGKEFKPDHRIGGYKVRYATWSIIMDSVVPSDKGNYTCIVENEYGSINHTYQLDVVERSPHRPILQAGLPANKTVALGSNVEFMCKVYSDPQPHIQWLKHIE 294
FGFR1-210      .............................................................................................................. 106
FGFR1-208      NPTLRWLKNGKEFKPDHRIGGYKVRYATWSIIMDSVVPSDKGNYTCIVENEYGSINHTYQLDVVERSPHRPILQAGLPANKTVALGSNVEFMCKVYSDPQPHIQWLKHIE 292
FGFR1-212      ..............................................................................................................  54
FGFR1-204      NPTLRWLKNGKEFKPDHRIGGYKVRYATWSIIMDSVVPSDKGNYTCIVENEYGSINHTYQLDVVERSPHRPILQAGLPANKTVALGSNVEFMCKVYSDPQPHIQWLKHIE 205
FGFR1-221      .............................................................................................................. 150
FGFR1-207      NPTLRWLKNGKEFKPDHRIGGYKVRYATWSIIMDSVVPSDKGNYTCIVENEYGSINHTYQLDVVERSPHRPILQAGLPANKTVALGSNVEFMCKVYSDPQPHIQWLKHIE 203
FGFR1-213      ..............................................................................................................   0
FGFR1-226      NPTLRWLKNGKEFKPDHRIGGYKVRYATWSIIMDSVVPSDKGNYTCIVENEYGSINHTYQLDVVERSPHRPILQAGLPANKTVALGSNVEFMCKVYSDPQPHIQWLKHIE 294
FGFR1-229      NPTLRWLKNGKEFKPDHRIGGYKVRYATWSIIMDSVVPSDKGNYTCIVENEY.......................................................... 145
FGFR1-211      NPTLRWLKNGKEFKPDHRIGGYKVRYATWSIIMDSVVPSDKGNYTCIVENEYGSINHTYQLDVVERSPHRPILQAGLPANKTVALGSNVEFMCKVYSDPQPHIQWLKHIE 325
FGFR1-234      ..............................................................................................................  85
FGFR1-203      NPTLRWLKNGKEFKPDHRIGGYKVRYATWSIIMDSVVPSDKGNYTCIVENEYGSINHTYQLDVVERSPHRPILQAGLPANKTVALGSNVEFMCKVYSDPQPHIQWLKHIE 295
FGFR1-240      NPTLRWLKNGKEFKPDHRIGGYKVRYATWSIIMDSVVPSDKGNYTCIVENEYGSINHTYQLDVVERSPHRPILQAGLPANKTVALGSNVEFMCKVYSDPQPHIQWLKHIE 134
FGFR1-222      ..............................................................................................................   0
FGFR1-233      NPTLRWLKNGKEFKPDHRIGGYKVRYATWSIIMDSVVPSDKGNYTCI............................................................... 142
FGFR1-206      NPTLRWLKNGKEFKPDHRIGGYKVRYATWSIIMDSVVPSDKGNYTCIVENEYGSINHTYQLDVVERSPHRPILQAGLPANKTVALGSNVEFMCKVYSDPQPHIQWLKHIE 292
```

logo

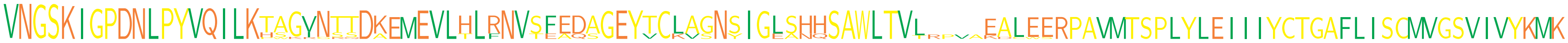

```
JNJ-42756493  ..............................................................................................................    0
PF07679.1     VNGSKIGPDNLPYVQILKHSGINSSDA..EVLTLFNVTEAQSGEYVCKVSNYIGEANQSAWLTV..............................................  174
PF07679.4     ..............................................................................................................   63
PF07679.3     ..............................................................................................................   79
PF07679.2     ..............................................................................................................   68
PF00047       ..............................................................................................................   73
PF00047.1     ..............................................................................................................   62
PF07714       ..............................................................................................................    0
PF07679       VNGSKIGPDNLPYVQILKTAGVNTTDKEMEVLHLRNVSFEDAGEYTCLAGNSIGLSHHSAWLTV..............................................  176
FGFR1-236     ..............................................................................................................    0
FGFR1-214     VNGSKIGPDNLPYVQILKTAGVNTTDKEMEVLHLRNVSFEDAGEYTCLAGNSIGLSHHSAWLTVL....EALEERPAVMTSPLYLEIIIYCTGAFLISCMVGSVIVYKMK  400
FGFR1-202     VNGSKIGPDNLPYVQILKTAGVNTTDKEMEVLHLRNVSFEDAGEYTCLAGNSIGLSHHSAWLTVL....EALEERPAVMTSPLYLEIIIYCTGAFLISCMVGSVIVYKMK  392
FGFR1-201     VNGSKIGPDNLPYVQILKTAGVNTTDKEMEVLHLRNVSFEDAGEYTCLAGNSIGLSHHSAWLTVL....EALEERPAVMTSPLYLEIIIYCTGAFLISCMVGSVIVYKMK  309
FGFR1-241     VNGSKIGPDNLPYVQILKPWKRGRQ.....................................................................................  228
FGFR1-209     VNGSKIGPDNLPYVQILKTAGVNTTDKEMEVLHLRNVSFEDAGEYTCLAGNSIGLSHHSAWLTVL....EALEERPAVMTSPLYLEIIIYCTGAFLISCMVGSVIVYKMK  398
FGFR1-238     VNGSKIGPDNLPYVQILKTAGVNTTDKEMEVLHLRNVSFEDAGEYTCLAGNSIGLSHHSAWLTVL....EALEERPAVMTSPLYLEIIIYCTGAFLISCMVGSVIVYKMK  400
FGFR1-210     ..............................................................................................................  106
FGFR1-208     VNGSKIGPDNLPYVQILKTAGVNTTDKEMEVLHLRNVSFEDAGEYTCLAGNSIGLSHHSAWLTVL....EALEERPAVMTSPLYLEIIIYCTGAFLISCMVGSVIVYKMK  398
FGFR1-212     ..............................................................................................................   54
FGFR1-204     VNGSKIGPDNLPYVQILKTAGVNTTDKEMEVLHLRNVSFEDAGEYTCLAGNSIGLSHHSAWLTVL....EALEERPAVMTSPLYLEIIIYCTGAFLISCMVGSVIVYKMK  311
FGFR1-221     ..............................................................................................................  150
FGFR1-207     VNGSKIGPDNLPYVQILKHSGINSSDA..EVLTLFNVTEAQSGEYVCKVSNYIGEANQSAWLTVTRPVAKALEERPAVMTSPLYLEIIIYCTGAFLISCMVGSVIVYKMK  311
FGFR1-213     ..............................................................................................................    0
FGFR1-226     VNGS..........................................................................................................  298
FGFR1-229     ..............................................................................................................  145
FGFR1-211     VNGSKIGPDNLPYVQILKTAGVNTTDKEMEVLHLRNVSFEDAGEYTCLAGNSIGLSHHSAWLTVL....EALEERPAVMTSPLYLEIIIYCTGAFLISCMVGSVIVYKMK  431
FGFR1-234     ..............................................................................................................   85
FGFR1-203     VNGSKIGPDNLPYVQILKHSGINSSDA..EVLTLFNVTEAQSGEYVCKVSNYIGEANQSAWLTVTRPVAKDCWS....................................  367
FGFR1-240     VNGSK.........................................................................................................  139
FGFR1-222     ..............................................................................................................    0
FGFR1-233     ..............................................................................................................  142
FGFR1-206     VNGSKIGPDNLPYVQILKTAGVNTTDKEMEVLHLRNVSFEDAGEYTCLAGNSIGLSHHSAWLTVL....EALEERPAVMTSPLYLEIIIYCTGAFLISCMVGSVIVYKMK  398
```

```
logo
                 SGTKKSDFHSQMAVHKLAKSIPLRRQVTVSADSSASMNSGVLLVRPSRLSSSGTPMLAGVSEYELPEDPRWELPRDRLVLGKPLGEGCFGQVVLAEAIGLDKDKPNRVTK
JNJ-42756493     ..............................................................................................................    0
PF07679.1        ..............................................................................................................  174
PF07679.4        ..............................................................................................................   63
PF07679.3        ..............................................................................................................   79
PF07679.2        ..............................................................................................................   68
PF00047          ..............................................................................................................   73
PF00047.1        ..............................................................................................................   62
PF07714          ..............................................................................VLGKPLGEGCFGQVVLAEAIGLDKDKPNRVTK   32
PF07679          ..............................................................................................................  176
FGFR1-236        ..............................................................................................................    0
FGFR1-214        SGTKKSDFHSQMAVHKLAKSIPLRRQVTVSADSSASMNSGVLLVRPSRLSSSGTPMLAGVSEYELPEDPRWELPRDRLVLGKPLGEGCFGQVVLAEAIGLDKDKPNRVTK  510
FGFR1-202        SGTKKSDFHSQMAVHKLAKSIPLRRQV..SADSSASMNSGVLLVRPSRLSSSGTPMLAGVSEYELPEDPRWELPRDRLVLGKPLGEGCFGQVVLAEAIGLDKDKPNRVTK  500
FGFR1-201        SGTKKSDFHSQMAVHKLAKSIPLRRQVTVSADSSASMNSGVLLVRPSRLSSSGTPMLAGVSEYELPEDPRWELPRDRLVLGKPLGEGCFGQVVLAEAIGLDKDKPNRVTK  419
FGFR1-241        ..............................................................................................................  228
FGFR1-209        SGTKKSDFHSQMAVHKLAKSIPLRRQVTVSADSSASMNSGVLLVRPSRLSSSGTPMLAGVSEYELPEDPRWELPRDRLVLGKPLGEGCFGQVVLAEAIGLDKDKPNRVTK  508
FGFR1-238        SGTKKSDFHSQMAVHKLAKSIPLRRQV..SADSSASMNSGVLLVRPSRLSSSGTPMLAGVSEYELPEDPRWELPRDRLVLGKPLGEGCFGQVVLAEAIGLDKDKPNRVTK  508
FGFR1-210        ..............................................................................................................  106
FGFR1-208        SGTKKSDFHSQMAVHKLAKSIPLRRQVTVSADSSASMNSGVLLVRPSRLSSSGTPMLAGVSEYELPEDPRWELPRDRLVLGKPLGEGCFGQVVLAEAIGLDKDKPNRVTK  508
FGFR1-212        ..............................................................................................................   54
FGFR1-204        SGTKKSDFHSQMAVHKLAKSIPLRRQVTVSADSSASMNSGVLLVRPSRLSSSGTPMLAGVSEYELPEDPRWELPRDRLVLGKPLGEGCFGQVVLAEAIGLDKDKPNRVTK  421
FGFR1-221        ..............................................................................................................  150
FGFR1-207        SGTKKSDFHSQMAVHKLAKSIPLRRQVTVSADSSASMNSGVLLVRPSRLSSSGTPMLAGVSEYELPEDPRWELPRDRLVLGKPLGEGCFGQVVLAEAIGLDKDKPNRVTK  421
FGFR1-213        ..............................................................................................................    0
FGFR1-226        ..............................................................................................................  298
FGFR1-229        ..............................................................................................................  145
FGFR1-211        SGTKKSDFHSQMAVHKLAKSIPLRRQVTVSADSSASMNSGVLLVRPSRLSSSGTPMLAGVSEYELPEDPRWELPRDRLVLGKPLGEGCFGQVVLAEAIGLDKDKPNRVTK  541
FGFR1-234        ..............................................................................................................   85
FGFR1-203        ..............................................................................................................  367
FGFR1-240        ..............................................................................................................  139
FGFR1-222        ..............................................................................................................    0
FGFR1-233        ..............................................................................................................  142
FGFR1-206        SGTKKSDFHSQMAVHKLAKSIPLRRQVTVSADSSASMNSGVLLVRPSRLSSSGTPMLAGVSEYELPEDPRWELPRDRLVLGKPLGEGCFGQVVLAEAIGLDKDKPNRVTK  508
```

```
logo
              VAVKMLKSDATEKDLSDLISEMEMMKMIGKHKNIINLLGACTQDGPLYVIVEYASKGNLREYLQARRPPGLEYCYNPSHNPEEQLSSKDLVSCAYQV      ARGMEYL
JNJ-42756493  ..................................................................................L.......V...................    2
PF07679.1     ..............................................................................................................  174
PF07679.4     ..............................................................................................................   63
PF07679.3     ..............................................................................................................   79
PF07679.2     ..............................................................................................................   68
PF00047       ..............................................................................................................   73
PF00047.1     ..............................................................................................................   62
PF07714       VAVKMLKSDATEKDLSDLISEMEMMKMIGKHKNIINLLGACTQDGPLYVIVEYASKGNLREYLQARRPPGLEYCYNPSHNPEEQLSSKDLVSCAYQV......ARGMEYL  136
PF07679       ..............................................................................................................  176
FGFR1-236     ..........................................................................................VSCAYQV......ARGMEYL   14
FGFR1-214     VAVKMLKSDATEKDLSDLISEMEMMKMIGKHKNIINLLGACTQDGPLYVIVEYASKGNLREYLQARRPPGLEYCYNPSHNPEEQLSSKDLVSCAYQV......ARGMEYL  614
FGFR1-202     VAVKMLKSDATEKDLSDLISEMEMMKMIGKHKNIINLLGACTQDGPLYVIVEYASKGNLREYLQARRPPGLEYCYNPSHNPEEQLSSKDLVSCAYQV......ARGMEYL  604
FGFR1-201     VAVKMLKSDATEKDLSDLISEMEMMKMIGKHKNIINLLGACTQDGPLYVIVEYASKGNLREYLQARRPPGLEYCYNPSHNPEEQLSSKDLVSCAYQV......ARGMEYL  523
FGFR1-241     ..............................................................................................................  228
FGFR1-209     VAVKMLKSDATEKDLSDLISEMEMMKMIGKHKNIINLLGACTQDGPLYVIVEYASKGNLREYLQARRPPGLEYCYNPSHNPEEQLSSKDLVSCAYQV......ARGMEYL  612
FGFR1-238     VAVKMLKSDATEKDLSDLISEMEMMKMIGKHKNIINLLGACTQDGPLYVIVEYASKGNLREYLQARRPPGLEYCYNPSHNPEEQLSSKDLVSCAYQV......ARGMEYL  612
FGFR1-210     ..............................................................................................................  106
FGFR1-208     VAVKMLKSDATEKDLSDLISEMEMMKMIGKHKNIINLLGACTQDGPLYVIVEYASKGNLREYLQARRPPGLEYCYNPSHNPEEQLSSKDLVSCAYQV......ARGMEYL  612
FGFR1-212     ..............................................................................................................   54
FGFR1-204     VAVKMLKSDATEKDLSDLISEMEMMKMIGKHKNIINLLGACTQDGPLYVIVEYASKGNLREYLQARRPPGLEYCYNPSHNPEEQLSSKDLVSCAYQV......ARGMEYL  525
FGFR1-221     ..............................................................................................................  150
FGFR1-207     VAVKMLKSDATEKDLSDLISEMEMMKMIGKHKNIINLLGACTQDGPLYVIVEYASKGNLREYLQARRPPGLEYCYNPSHNPEEQLSSKDLVSCAYQV......ARGMEYL  525
FGFR1-213     ..............................................................................................................    0
FGFR1-226     ..............................................................................................................  298
FGFR1-229     ..............................................................................................................  145
FGFR1-211     VAVKMLKSDATEKDLSDLISEMEMMKMIGKHKNIINLLGACTQDGPLYVIVEYASKGNLREYLQARRPPGLEYCYNPSHNPEEQLSSKDLVSCAYQV......ARGMEYL  645
FGFR1-234     ..............................................................................................................   85
FGFR1-203     ..............................................................................................................  367
FGFR1-240     ..............................................................................................................  139
FGFR1-222     ..............................................................................................................    0
FGFR1-233     ..............................................................................................................  142
FGFR1-206     VAVKMLKSDATEKDLSDLISEMEMMKMIGKHKNIINLLGACTQDGPLYVIVEYASKGNLREYLQARRPPGLEYCYNPSHNPEEQLSSKDLVSCAYQV......ARGMEYL  612
```

```
logo
               ASKKCIHRDL.AARNVLVTEDNVMKIADFGLARDIHHIDYYKKTTNGRLPVKWMAPEALFDRIYTHQSDVWSFGVL.LWEIFTLGGS....PYPGVPVEELFKLLKEGHR
JNJ-42756493   A.K................E...M..........I.............V.VEYA..G.....................................................   13
PF07679.1      ..............................................................................................................  174
PF07679.4      ..............................................................................................................   63
PF07679.3      ..............................................................................................................   79
PF07679.2      ..............................................................................................................   68
PF00047        ..............................................................................................................   73
PF00047.1      ..............................................................................................................   62
PF07714        ASKKCIHRDL.AARNVLVTEDNVMKIADFGLARDIHHIDYYKKTTNGRLPVKWMAPEALFDRIYTHQSDVWSFGVL.LWEIFTLGGS....PYPGVPVEELFKLLKEGHR  240
PF07679        ..............................................................................................................  176
FGFR1-236      ASKKCIHRDL.AARNVLVTEDNVMKIADFGLARDIHHIDYYKKTTNGRLPVKWMAPEALFDRIYTHQSDVWSFGVL.LWEIFTLGGS....PYPGVPVEELFKLLKEGHR  118
FGFR1-214      ASKKCIHRDL.AARNVLVTEDNVMKIADFGLARDIHHIDYYKKTTNGRLPVKWMAPEALFDRIYTHQSDVWSFGVL.LWEIFTLGGS....PYPGVPVEELFKLLKEGHR  718
FGFR1-202      ASKKCIHRDL.AARNVLVTEDNVMKIADFGLARDIHHIDYYKKTTNGRLPVKWMAPEALFDRIYTHQSDVWSFGVL.LWEIFTLGGS....PYPGVPVEELFKLLKEGHR  708
FGFR1-201      ASKKCIHRDL.AARNVLVTEDNVMKIADFGLARDIHHIDYYKKTTNGRLPVKWMAPEALFDRIYTHQSDVWSFGVL.LWEIFTLGGS....PYPGVPVEELFKLLKEGHR  627
FGFR1-241      ..............................................................................................................  228
FGFR1-209      ASKKCIHRDL.AARNVLVTEDNVMKIADFGLARDIHHIDYYKKTTNGRLPVKWMAPEALFDRIYTHQSDVWSFGVL.LWEIFTLGGS....PYPGVPVEELFKLLKEGHR  716
FGFR1-238      ASKKCIHRDL.AARNVLVTEDNVMKIADFGLARDIHHIDYYKKTTNGRLPVKWMAPEALFDRIYTHQSDVWSFGVL.LWEIFTLGGS....PYPGVPVEELFKLLKEGHR  716
FGFR1-210      ..............................................................................................................  106
FGFR1-208      ASKKCIHRDL.AARNVLVTEDNVMKIADFGLARDIHHIDYYKKTTNGRLPVKWMAPEALFDRIYTHQSDVWSFGVL.LWEIFTLGGS....PYPGVPVEELFKLLKEGHR  716
FGFR1-212      ..............................................................................................................   54
FGFR1-204      ASKKCIHRDL.AARNVLVTEDNVMKIADFGLARDIHHIDYYKKTTNGRLPVKWMAPEALFDRIYTHQSDVWSFGVL.LWEIFTLGGS....PYPGVPVEELFKLLKEGHR  629
FGFR1-221      ..............................................................................................................  150
FGFR1-207      ASKKCIHRDL.AARNVLVTEDNVMKIADFGLARDIHHIDYYKKTTNGRLPVKWMAPEALFDRIYTHQSDVWSFGVL.LWEIFTLGGS....PYPGVPVEELFKLLKEGHR  629
FGFR1-213      .......................M......................................................................................    1
FGFR1-226      ..............................................................................................................  298
FGFR1-229      ..............................................................................................................  145
FGFR1-211      ASKKCIHRDL.AARNVLVTEDNVMKIADFGLARDIHHIDYYKKTTNGRLPVKWMAPEALFDRIYTHQSDVWSFGVL.LWEIFTLGGS....PYPGVPVEELFKLLKEGHR  749
FGFR1-234      ..............................................................................................................   85
FGFR1-203      ..............................................................................................................  367
FGFR1-240      ..............................................................................................................  139
FGFR1-222      .....................................................................MWSWKCLLFWAVLVTATLCTARPSPTLPEQACPDLQEA...   38
FGFR1-233      ..............................................................................................................  142
FGFR1-206      ASKKCIHRDL.AARNVLVTEDNVMKIADFGLARDIHHIDYYKKTTNGRLPVKWMAPEALFDRIYTHQSDVWSFGVL.LWEIFTLGGS....PYPGVPVEELFKLLKEGHR  716
```

logo

```
                 MDKPSNCTNELYMMMRDCWHAVPSQRPTFKQLVEDLDRIVALTSNQEYLDLSMPLDQYSPSFPDTRSSTCSSGEDSVFSHEPLPEEPCLPRHPAQLANGGLKRR
JNJ-42756493     .......RN.L...............ADF...........................................................................  19
PF07679.1        ........................................................................................................  174
PF07679.4        ........................................................................................................  63
PF07679.3        ........................................................................................................  79
PF07679.2        ........................................................................................................  68
PF00047          ........................................................................................................  73
PF00047.1        ........................................................................................................  62
PF07714          MDKPSNCTNELYMMMRDCWHAVPSQRPTFKQLVEDL....................................................................  276
PF07679          ........................................................................................................  176
FGFR1-236        MDKPSNCTNELPAA.....GT...................................................................................  134
FGFR1-214        MDKPSNCTNELYMMMRDCWHAVPSQRPTFKQLVEDLDRIVALTSNQEYLDLSMPLDQYSPSFPDTRSSTCSSGEDSVFSHEPLPEEPCLPRHPAQLANGGLKRR  822
FGFR1-202        MDKPSNCTNELYMMMRDCWHAVPSQRPTFKQLVEDLDRIVALTSNQEYLDLSMPLDQYSPSFPDTRSSTCSSGEDSVFSHEPLPEEPCLPRHPAQLANGGLKRR  812
FGFR1-201        MDKPSNCTNELYMMMRDCWHAVPSQRPTFKQLVEDLDRIVALTSNQEYLDLSMPLDQYSPSFPDTRSSTCSSGEDSVFSHEPLPEEPCLPRHPAQLANGGLKRR  731
FGFR1-241        ........................................................................................................  228
FGFR1-209        MDKPSNCTNELYMMMRDCWHAVPSQRPTFKQLVEDLDRIVALTSNQEYLDLSMPLDQYSPSFPDTRSSTCSSGEDSVFSHEPLPEEPCLPRHPAQLANGGLKRR  820
FGFR1-238        MDKPSNCTNELYMMMRDCWHAVPSQRPTFKQLVEDLDRIVALTSNQEYLDLSMPLDQYSPSFPDTRSSTCSSGEDSVFSHEPLPEEPCLPRHPAQLANGGLKRR  820
FGFR1-210        ........................................................................................................  106
FGFR1-208        MDKPSNCTNELYMMMRDCWHAVPSQRPTFKQLVEDLDRIVALTSNQEYLDLSMPLDQYSPSFPDTRSSTCSSGEDSVFSHEPLPEEPCLPRHPAQLANGGLKRR  820
FGFR1-212        ........................................................................................................  54
FGFR1-204        MDKPSNCTNELYMMMRDCWHAVPSQRPTFKQLVEDLDRIVALTSNQEYLDLSMPLDQYSPSFPDTRSSTCSSGEDSVFSHEPLPEEPCLPRHPAQLANGGLKRR  733
FGFR1-221        ........................................................................................................  150
FGFR1-207        MDKPSNCTNELYMMMRDCWHAVPSQRPTFKQLVEDLDRIVALTSNQEYLDLSMPLDQYSPSFPDTRSSTCSSGEDSVFSHEPLPEEPCLPRHPAQLANGGLKRR  733
FGFR1-213        ........................................................................................................  1
FGFR1-226        ........................................................................................................  298
FGFR1-229        ........................................................................................................  145
FGFR1-211        MDKPSNCTNELYMMMRDCWHAVPSQRPTFKQLVEDLDRIVALTSNQEYLDLSMPLDQYSPSFPDTRSSTCSSGEDSVFSHEPLPEEPCLPRHPAQLANGGLKRR  853
FGFR1-234        ........................................................................................................  85
FGFR1-203        ........................................................................................................  367
FGFR1-240        ........................................................................................................  139
FGFR1-222        ....KWCSASFHSITPLPFGLG.TRLSD............................................................................  61
FGFR1-233        ........................................................................................................  142
FGFR1-206        MDKPSNCTNELYMMMRDCWHAVPSQRPTFKQLVEDLDRIVALTSNQEYLDLSMPLDQYSPSFPDTRSSTCSSGEDSVFSHEPLPEEPCLPRHPAQLANGGLKRR  820
```

X non conserved
X similar
X ≥0% conserved
X ≥50% conserved

logo

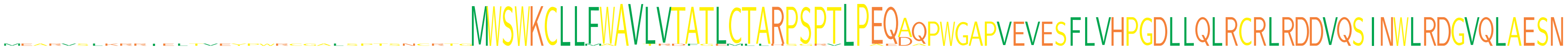

```
LUCITANIB   ..............................................................................................................   0
PF07679.1   ..............................................................................................................   0
PF07679.4   ..............................................................................................................   0
PF07679.3   ..............................................................................................................   0
PF07679.2   ..............................................................................................................   0
PF00047     ...........................................................................FLVHPGDLLQLRCRLRDDVQSINWLRDGVQLAESN  35
PF00047.1   ...........................................................................FLVHPGDLLQLRCRLRDDVQSINWLRDGVQLAESN  35
PF07714     ..............................................................................................................   0
PF07679     ..............................................................................................................   0
FGFR1-236   ..............................................................................................................   0
FGFR1-214   .................................MWSWKCLLFWAVLVTATLCTARPSPTLPEQAQPWGAPVEVESFLVHPGDLLQLRCRLRDDVQSINWLRDGVQLAESN  77
FGFR1-202   .........................................MAAVTRDFGEMLLHSGRVLPAEAQPWGAPVEVESFLVHPGDLLQLRCRLRDDVQSINWLRDGVQLAESN  69
FGFR1-201   .................................MWSWKCLLFWAVLVTATLCTARPSPTLPEQ...............................................  30
FGFR1-241   .................................MWSWKCLLFWAVLVTATLCTARPSPTLPEQ...............................................  30
FGFR1-209   .................................MWSWKCLLFWAVLVTATLCTARPSPTLPEQAQPWGAPVEVESFLVHPGDLLQLRCRLRDDVQSINWLRDGVQLAESN  77
FGFR1-238   .................................MWSWKCLLFWAVLVTATLCTARPSPTLPEQAQPWGAPVEVESFLVHPGDLLQLRCRLRDDVQSINWLRDGVQLAESN  77
FGFR1-210   .................................MWSWKCLLFWAVLVTATLCTARPSPTLPEQAQPWGAPVEVESFLVHPGDLLQLRCRLRDDVQSINWLRDGVQLAESN  77
FGFR1-208   .................................MWSWKCLLFWAVLVTATLCTARPSPTLPEQAQPWGAPVEVESFLVHPGDLLQLRCRLRDDVQSINWLRDGVQLAESN  77
FGFR1-212   .................................MWSWKCLLFWAVLVTATLCTARPSPTLPEQDA.............................................  32
FGFR1-204   .................................MWSWKCLLFWAVLVTATLCTARPSPTLPEQD..............................................  31
FGFR1-221   .................................MWSWKCLLFWAVLVTATLCTARPSPTLPEQAQPWGAPVEVESFLVHPGDLLQLRCRLRDDVQSINWLRDGVQLAESN  77
FGFR1-207   .................................MWSWKCLLFWAVLVTATLCTARPSPTLPEQD..............................................  31
FGFR1-213   ..............................................................................................................   0
FGFR1-226   .................................MWSWKCLLFWAVLVTATLCTARPSPTLPEQAQPWGAPVEVESFLVHPGDLLQLRCRLRDDVQSINWLRDGVQLAESN  77
FGFR1-229   .................................MWSWKCLLFWAVLVTATLCTARPSPTLPEQD..............................................  31
FGFR1-211   MEARVSLKRRIELTVEYPWRCGALSPTSNCRTGMWSWKCLLFWAVLVTATLCTARPSPTLPEQAQPWGAPVEVESFLVHPGDLLQLRCRLRDDVQSINWLRDGVQLAESN 110
FGFR1-234   .................................MWSWKCLLFWAVLVTATLCTARPSPTLPEQD..............................................  31
FGFR1-203   .................................MWSWKCLLFWAVLVTATLCTARPSPTLPEQAQPWGAPVEVESFLVHPGDLLQLRCRLRDDVQSINWLRDGVQLAESN  77
FGFR1-240   ..............................................................................................................   0
FGFR1-222   ..............................................................................................................   0
FGFR1-233   .................................MWSWKCLLFWAVLVTATLCTARPSPTLPEQ...............................................  30
FGFR1-206   .................................MWSWKCLLFWAVLVTATLCTARPSPTLPEQAQPWGAPVEVESFLVHPGDLLQLRCRLRDDVQSINWLRDGVQLAESN  77
```

logo

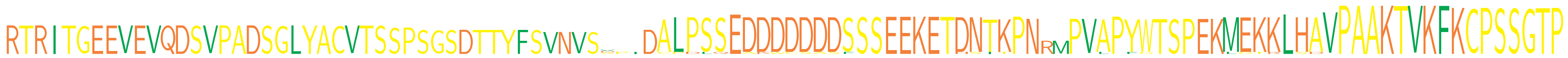

```
LUCITANIB   ..............................................................................................................   0
PF07679.1   ..............................................................................................PAAKTVKFKCPSSGTP   16
PF07679.4   .............................................................................................VPAAKTVKFKCPSSGTP   17
PF07679.3   ..............................................................................................PAAKTVKFKCPSSGTP   16
PF07679.2   .............................................................................................VPAAKTVKFKCPSSGTP   17
PF00047     RTRITGEEVEVQDSVPADSGLYACVTSSPSGSDTTYFS........................................................................   73
PF00047.1   RTRITGEEVEVQDSVPADSGLYACVTS...................................................................................   62
PF07714     ..............................................................................................................   0
PF07679     ..............................................................................................PAAKTVKFKCPSSGTP   16
FGFR1-236   ..............................................................................................................   0
FGFR1-214   RTRITGEEVEVQDSVPADSGLYACVTSSPSGSDTTYFSVNVS...DALPSSEDDDDDDDSSSEEKETDNTKPNRMPVAPYWTSPEKMEKKLHAVPAAKTVKFKCPSSGTP   184
FGFR1-202   RTRITGEEVEVQDSVPADSGLYACVTSSPSGSDTTYFSVNVSD...ALPSSEDDDDDDDSSSEEKETDNTKPNRMPVAPYWTSPEKMEKKLHAVPAAKTVKFKCPSSGTP   176
FGFR1-201   .............................................DALPSSEDDDDDDDSSSEEKETDNTKPN..PVAPYWTSPEKMEKKLHAVPAAKTVKFKCPSSGTP   93
FGFR1-241   .............................................DALPSSEDDDDDDDSSSEEKETDNTKPN..PVAPYWTSPEKMEKKLHAVPAAKTVKFKCPSSGTP   93
FGFR1-209   RTRITGEEVEVQDSVPADSGLYACVTSSPSGSDTTYFSVNVS...DALPSSEDDDDDDDSSSEEKETDNTKPN..PVAPYWTSPEKMEKKLHAVPAAKTVKFKCPSSGTP   182
FGFR1-238   RTRITGEEVEVQDSVPADSGLYACVTSSPSGSDTTYFSVNVS...DALPSSEDDDDDDDSSSEEKETDNTKPNRMPVAPYWTSPEKMEKKLHAVPAAKTVKFKCPSSGTP   184
FGFR1-210   RTRITGEEVEVQDSVPADSGLYACVTSSP.................................................................................   106
FGFR1-208   RTRITGEEVEVQDSVPADSGLYACVTSSPSGSDTTYFSVNVS...DALPSSEDDDDDDDSSSEEKETDNTKPN..PVAPYWTSPEKMEKKLHAVPAAKTVKFKCPSSGTP   182
FGFR1-212   ...............................................LPSSEDDDDDDDSSSEEKETDN.........................................   54
FGFR1-204   ..............................................ALPSSEDDDDDDDSSSEEKETDNTKPNRMPVAPYWTSPEKMEKKLHAVPAAKTVKFKCPSSGTP   95
FGFR1-221   RTRITGEEVEVQDSVPADSGLYACVTSSPSGSDTTYFSVNVSA...CPDLQEAKWCSASF.......HSITP.....LPFG.....LGTRLSD.................   150
FGFR1-207   ..............................................ALPSSEDDDDDDDSSSEEKETDNTKPN..PVAPYWTSPEKMEKKLHAVPAAKTVKFKCPSSGTP   93
FGFR1-213   ..............................................................................................................   0
FGFR1-226   RTRITGEEVEVQDSVPADSGLYACVTSSPSGSDTTYFSVNVS...DALPSSEDDDDDDDSSSEEKETDNTKPNRMPVAPYWTSPEKMEKKLHAVPAAKTVKFKCPSSGTP   184
FGFR1-229   ..............................................ALPSSEDDDDDDDSSSEEKETDNTKPN..PVAPYWTSPEKMEKKLHAVPAAKTVKFKCPSSGTP   93
FGFR1-211   RTRITGEEVEVQDSVPADSGLYACVTSSPSGSDTTYFSVNVS...DALPSSEDDDDDDDSSSEEKETDNTKPN..PVAPYWTSPEKMEKKLHAVPAAKTVKFKCPSSGTP   215
FGFR1-234   ..............................................ALPSSEDDDDDDDSSSEEKETDNTKPN..PVAPYWTSPEKMEKKLHAVPAAKTVKF........   85
FGFR1-203   RTRITGEEVEVQDSVPADSGLYACVTSSPSGSDTTYFSVNVSVPIDALPSSEDDDDDDDSSSEEKETDNTKPN..PVAPYWTSPEKMEKKLHAVPAAKTVKFKCPSSGTP   185
FGFR1-240   ......................................................................................MEKKLHAVPAAKTVKFKCPSSGTP   24
FGFR1-222   ..............................................................................................................   0
FGFR1-233   .............................................DALPSSEDDDDDDDSSSEEKETDNTKPNRMPVAPYWTSPEKMEKKLHAVPAAKTVKFKCPSSGTP   95
FGFR1-206   RTRITGEEVEVQDSVPADSGLYACVTSSPSGSDTTYFSVNVS...DALPSSEDDDDDDDSSSEEKETDNTKPN..PVAPYWTSPEKMEKKLHAVPAAKTVKFKCPSSGTP   182
```

```
logo        NPTLRWLKNGKEFKPDHRIGGYKVRYATWSIIMDSVVPSDKGNYTCIVENEYGSINHTYQLDVVERSPHRPILQAGLPANKTVALGSNVEFMCKVYSDPQPHIQWLKHIE
LUCITANIB   ..............................................................................................................    0
PF07679.1   NPTLRWLKNGKEFKPDHRIGGYKVRYATWSIIMDSVVPSDKGNYTCIVENEYGSINHTYQLDV..............PANKTVALGSNVEFMCKVYSDPQPHIQWLKHIE  112
PF07679.4   NPTLRWLKNGKEFKPDHRIGGYKVRYATWSIIMDSVVPSDKGNYTC................................................................   63
PF07679.3   NPTLRWLKNGKEFKPDHRIGGYKVRYATWSIIMDSVVPSDKGNYTCIVENEYGSINHTYQLDV...............................................   79
PF07679.2   NPTLRWLKNGKEFKPDHRIGGYKVRYATWSIIMDSVVPSDKGNYTCIVENE...........................................................   68
PF00047     ..............................................................................................................   73
PF00047.1   ..............................................................................................................   62
PF07714     ..............................................................................................................    0
PF07679     NPTLRWLKNGKEFKPDHRIGGYKVRYATWSIIMDSVVPSDKGNYTCIVENEYGSINHTYQLDV..............PANKTVALGSNVEFMCKVYSDPQPHIQWLKHIE  112
FGFR1-236   ..............................................................................................................    0
FGFR1-214   NPTLRWLKNGKEFKPDHRIGGYKVRYATWSIIMDSVVPSDKGNYTCIVENEYGSINHTYQLDVVERSPHRPILQAGLPANKTVALGSNVEFMCKVYSDPQPHIQWLKHIE  294
FGFR1-202   NPTLRWLKNGKEFKPDHRIGGYKVRYATWSIIMDSVVPSDKGNYTCIVENEYGSINHTYQLDVVERSPHRPILQAGLPANKTVALGSNVEFMCKVYSDPQPHIQWLKHIE  286
FGFR1-201   NPTLRWLKNGKEFKPDHRIGGYKVRYATWSIIMDSVVPSDKGNYTCIVENEYGSINHTYQLDVVERSPHRPILQAGLPANKTVALGSNVEFMCKVYSDPQPHIQWLKHIE  203
FGFR1-241   NPTLRWLKNGKEFKPDHRIGGYKVRYATWSIIMDSVVPSDKGNYTCIVENEYGSINHTYQLDVVERSPHRPILQAGLPANKTVALGSNVEFMCKVYSDPQPHIQWLKHIE  203
FGFR1-209   NPTLRWLKNGKEFKPDHRIGGYKVRYATWSIIMDSVVPSDKGNYTCIVENEYGSINHTYQLDVVERSPHRPILQAGLPANKTVALGSNVEFMCKVYSDPQPHIQWLKHIE  292
FGFR1-238   NPTLRWLKNGKEFKPDHRIGGYKVRYATWSIIMDSVVPSDKGNYTCIVENEYGSINHTYQLDVVERSPHRPILQAGLPANKTVALGSNVEFMCKVYSDPQPHIQWLKHIE  294
FGFR1-210   ..............................................................................................................  106
FGFR1-208   NPTLRWLKNGKEFKPDHRIGGYKVRYATWSIIMDSVVPSDKGNYTCIVENEYGSINHTYQLDVVERSPHRPILQAGLPANKTVALGSNVEFMCKVYSDPQPHIQWLKHIE  292
FGFR1-212   ..............................................................................................................   54
FGFR1-204   NPTLRWLKNGKEFKPDHRIGGYKVRYATWSIIMDSVVPSDKGNYTCIVENEYGSINHTYQLDVVERSPHRPILQAGLPANKTVALGSNVEFMCKVYSDPQPHIQWLKHIE  205
FGFR1-221   ..............................................................................................................  150
FGFR1-207   NPTLRWLKNGKEFKPDHRIGGYKVRYATWSIIMDSVVPSDKGNYTCIVENEYGSINHTYQLDVVERSPHRPILQAGLPANKTVALGSNVEFMCKVYSDPQPHIQWLKHIE  203
FGFR1-213   ..............................................................................................................    0
FGFR1-226   NPTLRWLKNGKEFKPDHRIGGYKVRYATWSIIMDSVVPSDKGNYTCIVENEYGSINHTYQLDVVERSPHRPILQAGLPANKTVALGSNVEFMCKVYSDPQPHIQWLKHIE  294
FGFR1-229   NPTLRWLKNGKEFKPDHRIGGYKVRYATWSIIMDSVVPSDKGNYTCIVENEY..........................................................  145
FGFR1-211   NPTLRWLKNGKEFKPDHRIGGYKVRYATWSIIMDSVVPSDKGNYTCIVENEYGSINHTYQLDVVERSPHRPILQAGLPANKTVALGSNVEFMCKVYSDPQPHIQWLKHIE  325
FGFR1-234   ..............................................................................................................   85
FGFR1-203   NPTLRWLKNGKEFKPDHRIGGYKVRYATWSIIMDSVVPSDKGNYTCIVENEYGSINHTYQLDVVERSPHRPILQAGLPANKTVALGSNVEFMCKVYSDPQPHIQWLKHIE  295
FGFR1-240   NPTLRWLKNGKEFKPDHRIGGYKVRYATWSIIMDSVVPSDKGNYTCIVENEYGSINHTYQLDVVERSPHRPILQAGLPANKTVALGSNVEFMCKVYSDPQPHIQWLKHIE  134
FGFR1-222   ..............................................................................................................    0
FGFR1-233   NPTLRWLKNGKEFKPDHRIGGYKVRYATWSIIMDSVVPSDKGNYTCI...............................................................  142
FGFR1-206   NPTLRWLKNGKEFKPDHRIGGYKVRYATWSIIMDSVVPSDKGNYTCIVENEYGSINHTYQLDVVERSPHRPILQAGLPANKTVALGSNVEFMCKVYSDPQPHIQWLKHIE  292
```

logo

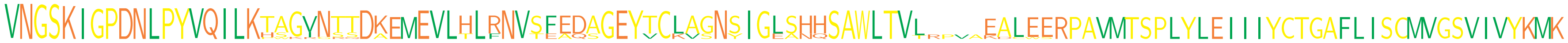

```
LUCITANIB    ..............................................................................................................   0
PF07679.1    VNGSKIGPDNLPYVQILKHSGINSSDA..EVLTLFNVTEAQSGEYVCKVSNYIGEANQSAWLTV..............................................   174
PF07679.4    ..............................................................................................................   63
PF07679.3    ..............................................................................................................   79
PF07679.2    ..............................................................................................................   68
PF00047      ..............................................................................................................   73
PF00047.1    ..............................................................................................................   62
PF07714      ..............................................................................................................   0
PF07679      VNGSKIGPDNLPYVQILKTAGVNTTDKEMEVLHLRNVSFEDAGEYTCLAGNSIGLSHHSAWLTV..............................................   176
FGFR1-236    ..............................................................................................................   0
FGFR1-214    VNGSKIGPDNLPYVQILKTAGVNTTDKEMEVLHLRNVSFEDAGEYTCLAGNSIGLSHHSAWLTVL....EALEERPAVMTSPLYLEIIIYCTGAFLISCMVGSVIVYKMK   400
FGFR1-202    VNGSKIGPDNLPYVQILKTAGVNTTDKEMEVLHLRNVSFEDAGEYTCLAGNSIGLSHHSAWLTVL....EALEERPAVMTSPLYLEIIIYCTGAFLISCMVGSVIVYKMK   392
FGFR1-201    VNGSKIGPDNLPYVQILKTAGVNTTDKEMEVLHLRNVSFEDAGEYTCLAGNSIGLSHHSAWLTVL....EALEERPAVMTSPLYLEIIIYCTGAFLISCMVGSVIVYKMK   309
FGFR1-241    VNGSKIGPDNLPYVQILKPWKRGRQ.....................................................................................   228
FGFR1-209    VNGSKIGPDNLPYVQILKTAGVNTTDKEMEVLHLRNVSFEDAGEYTCLAGNSIGLSHHSAWLTVL....EALEERPAVMTSPLYLEIIIYCTGAFLISCMVGSVIVYKMK   398
FGFR1-238    VNGSKIGPDNLPYVQILKTAGVNTTDKEMEVLHLRNVSFEDAGEYTCLAGNSIGLSHHSAWLTVL....EALEERPAVMTSPLYLEIIIYCTGAFLISCMVGSVIVYKMK   400
FGFR1-210    ..............................................................................................................   106
FGFR1-208    VNGSKIGPDNLPYVQILKTAGVNTTDKEMEVLHLRNVSFEDAGEYTCLAGNSIGLSHHSAWLTVL....EALEERPAVMTSPLYLEIIIYCTGAFLISCMVGSVIVYKMK   398
FGFR1-212    ..............................................................................................................   54
FGFR1-204    VNGSKIGPDNLPYVQILKTAGVNTTDKEMEVLHLRNVSFEDAGEYTCLAGNSIGLSHHSAWLTVL....EALEERPAVMTSPLYLEIIIYCTGAFLISCMVGSVIVYKMK   311
FGFR1-221    ..............................................................................................................   150
FGFR1-207    VNGSKIGPDNLPYVQILKHSGINSSDA..EVLTLFNVTEAQSGEYVCKVSNYIGEANQSAWLTVTRPVAKALEERPAVMTSPLYLEIIIYCTGAFLISCMVGSVIVYKMK   311
FGFR1-213    ..............................................................................................................   0
FGFR1-226    VNGS..........................................................................................................   298
FGFR1-229    ..............................................................................................................   145
FGFR1-211    VNGSKIGPDNLPYVQILKTAGVNTTDKEMEVLHLRNVSFEDAGEYTCLAGNSIGLSHHSAWLTVL....EALEERPAVMTSPLYLEIIIYCTGAFLISCMVGSVIVYKMK   431
FGFR1-234    ..............................................................................................................   85
FGFR1-203    VNGSKIGPDNLPYVQILKHSGINSSDA..EVLTLFNVTEAQSGEYVCKVSNYIGEANQSAWLTVTRPVAKDCWS....................................   367
FGFR1-240    VNGSK.........................................................................................................   139
FGFR1-222    ..............................................................................................................   0
FGFR1-233    ..............................................................................................................   142
FGFR1-206    VNGSKIGPDNLPYVQILKTAGVNTTDKEMEVLHLRNVSFEDAGEYTCLAGNSIGLSHHSAWLTVL....EALEERPAVMTSPLYLEIIIYCTGAFLISCMVGSVIVYKMK   398
```

```
logo
             SGTKKSDFHSQMAVHKLAKSIPLRRQVTvSADSSASMNSGVLLVRPSRLSSSGTPMLAGVSEYELPEDPRWELPRDRLVLGKPLGEGCFGQVVLAEAIGLDKDKPNRVTK
LUCITANIB    ..............................................................................................................   0
PF07679.1    ..............................................................................................................   174
PF07679.4    ..............................................................................................................   63
PF07679.3    ..............................................................................................................   79
PF07679.2    ..............................................................................................................   68
PF00047      ..............................................................................................................   73
PF00047.1    ..............................................................................................................   62
PF07714      ..............................................................................VLGKPLGEGCFGQVVLAEAIGLDKDKPNRVTK   32
PF07679      ..............................................................................................................   176
FGFR1-236    ..............................................................................................................   0
FGFR1-214    SGTKKSDFHSQMAVHKLAKSIPLRRQVTVSADSSASMNSGVLLVRPSRLSSSGTPMLAGVSEYELPEDPRWELPRDRLVLGKPLGEGCFGQVVLAEAIGLDKDKPNRVTK   510
FGFR1-202    SGTKKSDFHSQMAVHKLAKSIPLRRQV..SADSSASMNSGVLLVRPSRLSSSGTPMLAGVSEYELPEDPRWELPRDRLVLGKPLGEGCFGQVVLAEAIGLDKDKPNRVTK   500
FGFR1-201    SGTKKSDFHSQMAVHKLAKSIPLRRQVTVSADSSASMNSGVLLVRPSRLSSSGTPMLAGVSEYELPEDPRWELPRDRLVLGKPLGEGCFGQVVLAEAIGLDKDKPNRVTK   419
FGFR1-241    ..............................................................................................................   228
FGFR1-209    SGTKKSDFHSQMAVHKLAKSIPLRRQVTVSADSSASMNSGVLLVRPSRLSSSGTPMLAGVSEYELPEDPRWELPRDRLVLGKPLGEGCFGQVVLAEAIGLDKDKPNRVTK   508
FGFR1-238    SGTKKSDFHSQMAVHKLAKSIPLRRQV..SADSSASMNSGVLLVRPSRLSSSGTPMLAGVSEYELPEDPRWELPRDRLVLGKPLGEGCFGQVVLAEAIGLDKDKPNRVTK   508
FGFR1-210    ..............................................................................................................   106
FGFR1-208    SGTKKSDFHSQMAVHKLAKSIPLRRQVTVSADSSASMNSGVLLVRPSRLSSSGTPMLAGVSEYELPEDPRWELPRDRLVLGKPLGEGCFGQVVLAEAIGLDKDKPNRVTK   508
FGFR1-212    ..............................................................................................................   54
FGFR1-204    SGTKKSDFHSQMAVHKLAKSIPLRRQVTVSADSSASMNSGVLLVRPSRLSSSGTPMLAGVSEYELPEDPRWELPRDRLVLGKPLGEGCFGQVVLAEAIGLDKDKPNRVTK   421
FGFR1-221    ..............................................................................................................   150
FGFR1-207    SGTKKSDFHSQMAVHKLAKSIPLRRQVTVSADSSASMNSGVLLVRPSRLSSSGTPMLAGVSEYELPEDPRWELPRDRLVLGKPLGEGCFGQVVLAEAIGLDKDKPNRVTK   421
FGFR1-213    ..............................................................................................................   0
FGFR1-226    ..............................................................................................................   298
FGFR1-229    ..............................................................................................................   145
FGFR1-211    SGTKKSDFHSQMAVHKLAKSIPLRRQVTVSADSSASMNSGVLLVRPSRLSSSGTPMLAGVSEYELPEDPRWELPRDRLVLGKPLGEGCFGQVVLAEAIGLDKDKPNRVTK   541
FGFR1-234    ..............................................................................................................   85
FGFR1-203    ..............................................................................................................   367
FGFR1-240    ..............................................................................................................   139
FGFR1-222    ..............................................................................................................   0
FGFR1-233    ..............................................................................................................   142
FGFR1-206    SGTKKSDFHSQMAVHKLAKSIPLRRQVTVSADSSASMNSGVLLVRPSRLSSSGTPMLAGVSEYELPEDPRWELPRDRLVLGKPLGEGCFGQVVLAEAIGLDKDKPNRVTK   508
```

```
logo
             VAVKMLKSDATEKDLSDLISEMEMMKMIGKHKNIINLLGACTQDGPLYVIVEYASKGNLREYLQARRPPGLEYCYNPSHNPEEQLSSKDLVSCAYQV      ARGMEYL
LUCITANIB    ..................................................................................L.......V...................    2
PF07679.1    ..............................................................................................................  174
PF07679.4    ..............................................................................................................   63
PF07679.3    ..............................................................................................................   79
PF07679.2    ..............................................................................................................   68
PF00047      ..............................................................................................................   73
PF00047.1    ..............................................................................................................   62
PF07714      VAVKMLKSDATEKDLSDLISEMEMMKMIGKHKNIINLLGACTQDGPLYVIVEYASKGNLREYLQARRPPGLEYCYNPSHNPEEQLSSKDLVSCAYQV......ARGMEYL  136
PF07679      ..............................................................................................................  176
FGFR1-236    ..........................................................................................VSCAYQV......ARGMEYL   14
FGFR1-214    VAVKMLKSDATEKDLSDLISEMEMMKMIGKHKNIINLLGACTQDGPLYVIVEYASKGNLREYLQARRPPGLEYCYNPSHNPEEQLSSKDLVSCAYQV......ARGMEYL  614
FGFR1-202    VAVKMLKSDATEKDLSDLISEMEMMKMIGKHKNIINLLGACTQDGPLYVIVEYASKGNLREYLQARRPPGLEYCYNPSHNPEEQLSSKDLVSCAYQV......ARGMEYL  604
FGFR1-201    VAVKMLKSDATEKDLSDLISEMEMMKMIGKHKNIINLLGACTQDGPLYVIVEYASKGNLREYLQARRPPGLEYCYNPSHNPEEQLSSKDLVSCAYQV......ARGMEYL  523
FGFR1-241    ..............................................................................................................  228
FGFR1-209    VAVKMLKSDATEKDLSDLISEMEMMKMIGKHKNIINLLGACTQDGPLYVIVEYASKGNLREYLQARRPPGLEYCYNPSHNPEEQLSSKDLVSCAYQV......ARGMEYL  612
FGFR1-238    VAVKMLKSDATEKDLSDLISEMEMMKMIGKHKNIINLLGACTQDGPLYVIVEYASKGNLREYLQARRPPGLEYCYNPSHNPEEQLSSKDLVSCAYQV......ARGMEYL  612
FGFR1-210    ..............................................................................................................  106
FGFR1-208    VAVKMLKSDATEKDLSDLISEMEMMKMIGKHKNIINLLGACTQDGPLYVIVEYASKGNLREYLQARRPPGLEYCYNPSHNPEEQLSSKDLVSCAYQV......ARGMEYL  612
FGFR1-212    ..............................................................................................................   54
FGFR1-204    VAVKMLKSDATEKDLSDLISEMEMMKMIGKHKNIINLLGACTQDGPLYVIVEYASKGNLREYLQARRPPGLEYCYNPSHNPEEQLSSKDLVSCAYQV......ARGMEYL  525
FGFR1-221    ..............................................................................................................  150
FGFR1-207    VAVKMLKSDATEKDLSDLISEMEMMKMIGKHKNIINLLGACTQDGPLYVIVEYASKGNLREYLQARRPPGLEYCYNPSHNPEEQLSSKDLVSCAYQV......ARGMEYL  525
FGFR1-213    ..............................................................................................................    0
FGFR1-226    ..............................................................................................................  298
FGFR1-229    ..............................................................................................................  145
FGFR1-211    VAVKMLKSDATEKDLSDLISEMEMMKMIGKHKNIINLLGACTQDGPLYVIVEYASKGNLREYLQARRPPGLEYCYNPSHNPEEQLSSKDLVSCAYQV......ARGMEYL  645
FGFR1-234    ..............................................................................................................   85
FGFR1-203    ..............................................................................................................  367
FGFR1-240    ..............................................................................................................  139
FGFR1-222    ..............................................................................................................    0
FGFR1-233    ..............................................................................................................  142
FGFR1-206    VAVKMLKSDATEKDLSDLISEMEMMKMIGKHKNIINLLGACTQDGPLYVIVEYASKGNLREYLQARRPPGLEYCYNPSHNPEEQLSSKDLVSCAYQV......ARGMEYL  612
```

logo

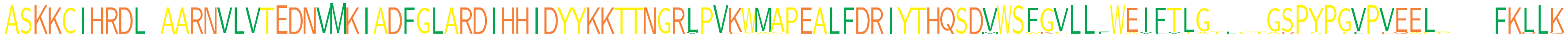

```
LUCITANIB    A.K................E...M........................V.VEYAS.......................................................  12
PF07679.1    .............................................................................................................. 174
PF07679.4    ..............................................................................................................  63
PF07679.3    ..............................................................................................................  79
PF07679.2    ..............................................................................................................  68
PF00047      ..............................................................................................................  73
PF00047.1    ..............................................................................................................  62
PF07714      ASKKCIHRDL.AARNVLVTEDNVMKIADFGLARDIHHIDYYKKTTNGRLPVKWMAPEALFDRIYTHQSDVWSFGVLL.WEIFTLG....GSPYPGVPVEEL....FKLLK 236
PF07679      .............................................................................................................. 176
FGFR1-236    ASKKCIHRDL.AARNVLVTEDNVMKIADFGLARDIHHIDYYKKTTNGRLPVKWMAPEALFDRIYTHQSDVWSFGVLL.WEIFTLG....GSPYPGVPVEEL....FKLLK 114
FGFR1-214    ASKKCIHRDL.AARNVLVTEDNVMKIADFGLARDIHHIDYYKKTTNGRLPVKWMAPEALFDRIYTHQSDVWSFGVLL.WEIFTLG....GSPYPGVPVEEL....FKLLK 714
FGFR1-202    ASKKCIHRDL.AARNVLVTEDNVMKIADFGLARDIHHIDYYKKTTNGRLPVKWMAPEALFDRIYTHQSDVWSFGVLL.WEIFTLG....GSPYPGVPVEEL....FKLLK 704
FGFR1-201    ASKKCIHRDL.AARNVLVTEDNVMKIADFGLARDIHHIDYYKKTTNGRLPVKWMAPEALFDRIYTHQSDVWSFGVLL.WEIFTLG....GSPYPGVPVEEL....FKLLK 623
FGFR1-241    .............................................................................................................. 228
FGFR1-209    ASKKCIHRDL.AARNVLVTEDNVMKIADFGLARDIHHIDYYKKTTNGRLPVKWMAPEALFDRIYTHQSDVWSFGVLL.WEIFTLG....GSPYPGVPVEEL....FKLLK 712
FGFR1-238    ASKKCIHRDL.AARNVLVTEDNVMKIADFGLARDIHHIDYYKKTTNGRLPVKWMAPEALFDRIYTHQSDVWSFGVLL.WEIFTLG....GSPYPGVPVEEL....FKLLK 712
FGFR1-210    .............................................................................................................. 106
FGFR1-208    ASKKCIHRDL.AARNVLVTEDNVMKIADFGLARDIHHIDYYKKTTNGRLPVKWMAPEALFDRIYTHQSDVWSFGVLL.WEIFTLG....GSPYPGVPVEEL....FKLLK 712
FGFR1-212    ..............................................................................................................  54
FGFR1-204    ASKKCIHRDL.AARNVLVTEDNVMKIADFGLARDIHHIDYYKKTTNGRLPVKWMAPEALFDRIYTHQSDVWSFGVLL.WEIFTLG....GSPYPGVPVEEL....FKLLK 625
FGFR1-221    .............................................................................................................. 150
FGFR1-207    ASKKCIHRDL.AARNVLVTEDNVMKIADFGLARDIHHIDYYKKTTNGRLPVKWMAPEALFDRIYTHQSDVWSFGVLL.WEIFTLG....GSPYPGVPVEEL....FKLLK 625
FGFR1-213    .......................M......................................................................................   1
FGFR1-226    .............................................................................................................. 298
FGFR1-229    .............................................................................................................. 145
FGFR1-211    ASKKCIHRDL.AARNVLVTEDNVMKIADFGLARDIHHIDYYKKTTNGRLPVKWMAPEALFDRIYTHQSDVWSFGVLL.WEIFTLG....GSPYPGVPVEEL....FKLLK 745
FGFR1-234    ..............................................................................................................  85
FGFR1-203    .............................................................................................................. 367
FGFR1-240    .............................................................................................................. 139
FGFR1-222    .....................................................................MWSWKCLLFWAVLVTATLCTARPSPTLPEQACP.....DLQ  36
FGFR1-233    .............................................................................................................. 142
FGFR1-206    ASKKCIHRDL.AARNVLVTEDNVMKIADFGLARDIHHIDYYKKTTNGRLPVKWMAPEALFDRIYTHQSDVWSFGVLL.WEIFTLG....GSPYPGVPVEEL....FKLLK 712
```

```
logo
            EGHRMDKPSNCTNELYMMMRDCWHAVPSQRPTFKQLVEDLDRIVALTSNQEYLDLSMPLDQYSPSFPDTRSSTCSSGEDSVFSHEPLPEEPCLPRHPAQLANGGLKRR
LUCITANIB   ..............L.........A.DF.......L........................................................................   17
PF07679.1   ............................................................................................................  174
PF07679.4   ............................................................................................................   63
PF07679.3   ............................................................................................................   79
PF07679.2   ............................................................................................................   68
PF00047     ............................................................................................................   73
PF00047.1   ............................................................................................................   62
PF07714     EGHRMDKPSNCTNELYMMMRDCWHAVPSQRPTFKQLVEDL....................................................................  276
PF07679     ............................................................................................................  176
FGFR1-236   EGHRMDKPSNCTNELPAAGT........................................................................................  134
FGFR1-214   EGHRMDKPSNCTNELYMMMRDCWHAVPSQRPTFKQLVEDLDRIVALTSNQEYLDLSMPLDQYSPSFPDTRSSTCSSGEDSVFSHEPLPEEPCLPRHPAQLANGGLKRR  822
FGFR1-202   EGHRMDKPSNCTNELYMMMRDCWHAVPSQRPTFKQLVEDLDRIVALTSNQEYLDLSMPLDQYSPSFPDTRSSTCSSGEDSVFSHEPLPEEPCLPRHPAQLANGGLKRR  812
FGFR1-201   EGHRMDKPSNCTNELYMMMRDCWHAVPSQRPTFKQLVEDLDRIVALTSNQEYLDLSMPLDQYSPSFPDTRSSTCSSGEDSVFSHEPLPEEPCLPRHPAQLANGGLKRR  731
FGFR1-241   ............................................................................................................  228
FGFR1-209   EGHRMDKPSNCTNELYMMMRDCWHAVPSQRPTFKQLVEDLDRIVALTSNQEYLDLSMPLDQYSPSFPDTRSSTCSSGEDSVFSHEPLPEEPCLPRHPAQLANGGLKRR  820
FGFR1-238   EGHRMDKPSNCTNELYMMMRDCWHAVPSQRPTFKQLVEDLDRIVALTSNQEYLDLSMPLDQYSPSFPDTRSSTCSSGEDSVFSHEPLPEEPCLPRHPAQLANGGLKRR  820
FGFR1-210   ............................................................................................................  106
FGFR1-208   EGHRMDKPSNCTNELYMMMRDCWHAVPSQRPTFKQLVEDLDRIVALTSNQEYLDLSMPLDQYSPSFPDTRSSTCSSGEDSVFSHEPLPEEPCLPRHPAQLANGGLKRR  820
FGFR1-212   ............................................................................................................   54
FGFR1-204   EGHRMDKPSNCTNELYMMMRDCWHAVPSQRPTFKQLVEDLDRIVALTSNQEYLDLSMPLDQYSPSFPDTRSSTCSSGEDSVFSHEPLPEEPCLPRHPAQLANGGLKRR  733
FGFR1-221   ............................................................................................................  150
FGFR1-207   EGHRMDKPSNCTNELYMMMRDCWHAVPSQRPTFKQLVEDLDRIVALTSNQEYLDLSMPLDQYSPSFPDTRSSTCSSGEDSVFSHEPLPEEPCLPRHPAQLANGGLKRR  733
FGFR1-213   ............................................................................................................    1
FGFR1-226   ............................................................................................................  298
FGFR1-229   ............................................................................................................  145
FGFR1-211   EGHRMDKPSNCTNELYMMMRDCWHAVPSQRPTFKQLVEDLDRIVALTSNQEYLDLSMPLDQYSPSFPDTRSSTCSSGEDSVFSHEPLPEEPCLPRHPAQLANGGLKRR  853
FGFR1-234   ............................................................................................................   85
FGFR1-203   ............................................................................................................  367
FGFR1-240   ............................................................................................................  139
FGFR1-222   E......AKWCSASFHSIT....PL.PFG......LGTRLSD..................................................................   61
FGFR1-233   ............................................................................................................  142
FGFR1-206   EGHRMDKPSNCTNELYMMMRDCWHAVPSQRPTFKQLVEDLDRIVALTSNQEYLDLSMPLDQYSPSFPDTRSSTCSSGEDSVFSHEPLPEEPCLPRHPAQLANGGLKRR  820
```

X non conserved
X similar
X ≥ 0% conserved
X ≥ 50% conserved

logo

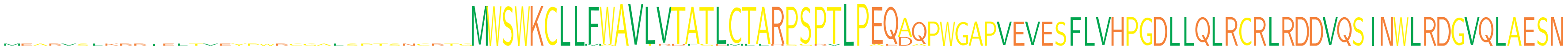

```
PONATINIB    ..............................................................................................................   0
PF07679.1    ..............................................................................................................   0
PF07679.4    ..............................................................................................................   0
PF07679.3    ..............................................................................................................   0
PF07679.2    ..............................................................................................................   0
PF00047      ...........................................................................FLVHPGDLLQLRCRLRDDVQSINWLRDGVQLAESN  35
PF00047.1    ...........................................................................FLVHPGDLLQLRCRLRDDVQSINWLRDGVQLAESN  35
PF07714      ..............................................................................................................   0
PF07679      ..............................................................................................................   0
FGFR1-236    ..............................................................................................................   0
FGFR1-214    .................................MWSWKCLLFWAVLVTATLCTARPSPTLPEQAQPWGAPVEVESFLVHPGDLLQLRCRLRDDVQSINWLRDGVQLAESN  77
FGFR1-202    .........................................MAAVTRDFGEMLLHSGRVLPAEAQPWGAPVEVESFLVHPGDLLQLRCRLRDDVQSINWLRDGVQLAESN  69
FGFR1-201    .................................MWSWKCLLFWAVLVTATLCTARPSPTLPEQ...............................................  30
FGFR1-241    .................................MWSWKCLLFWAVLVTATLCTARPSPTLPEQ...............................................  30
FGFR1-209    .................................MWSWKCLLFWAVLVTATLCTARPSPTLPEQAQPWGAPVEVESFLVHPGDLLQLRCRLRDDVQSINWLRDGVQLAESN  77
FGFR1-238    .................................MWSWKCLLFWAVLVTATLCTARPSPTLPEQAQPWGAPVEVESFLVHPGDLLQLRCRLRDDVQSINWLRDGVQLAESN  77
FGFR1-210    .................................MWSWKCLLFWAVLVTATLCTARPSPTLPEQAQPWGAPVEVESFLVHPGDLLQLRCRLRDDVQSINWLRDGVQLAESN  77
FGFR1-208    .................................MWSWKCLLFWAVLVTATLCTARPSPTLPEQAQPWGAPVEVESFLVHPGDLLQLRCRLRDDVQSINWLRDGVQLAESN  77
FGFR1-212    .................................MWSWKCLLFWAVLVTATLCTARPSPTLPEQDA.............................................  32
FGFR1-204    .................................MWSWKCLLFWAVLVTATLCTARPSPTLPEQD..............................................  31
FGFR1-221    .................................MWSWKCLLFWAVLVTATLCTARPSPTLPEQAQPWGAPVEVESFLVHPGDLLQLRCRLRDDVQSINWLRDGVQLAESN  77
FGFR1-207    .................................MWSWKCLLFWAVLVTATLCTARPSPTLPEQD..............................................  31
FGFR1-213    ..............................................................................................................   0
FGFR1-226    .................................MWSWKCLLFWAVLVTATLCTARPSPTLPEQAQPWGAPVEVESFLVHPGDLLQLRCRLRDDVQSINWLRDGVQLAESN  77
FGFR1-229    .................................MWSWKCLLFWAVLVTATLCTARPSPTLPEQD..............................................  31
FGFR1-211    MEARVSLKRRIELTVEYPWRCGALSPTSNCRTGMWSWKCLLFWAVLVTATLCTARPSPTLPEQAQPWGAPVEVESFLVHPGDLLQLRCRLRDDVQSINWLRDGVQLAESN 110
FGFR1-234    .................................MWSWKCLLFWAVLVTATLCTARPSPTLPEQD..............................................  31
FGFR1-203    .................................MWSWKCLLFWAVLVTATLCTARPSPTLPEQAQPWGAPVEVESFLVHPGDLLQLRCRLRDDVQSINWLRDGVQLAESN  77
FGFR1-240    ..............................................................................................................   0
FGFR1-222    ..............................................................................................................   0
FGFR1-233    .................................MWSWKCLLFWAVLVTATLCTARPSPTLPEQ...............................................  30
FGFR1-206    .................................MWSWKCLLFWAVLVTATLCTARPSPTLPEQAQPWGAPVEVESFLVHPGDLLQLRCRLRDDVQSINWLRDGVQLAESN  77
```

logo

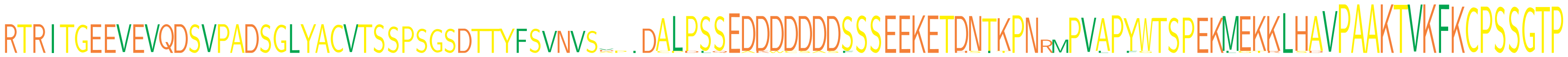

```
PONATINIB   ..............................................................................................................   0
PF07679.1   ..............................................................................................PAAKTVKFKCPSSGTP  16
PF07679.4   .............................................................................................VPAAKTVKFKCPSSGTP  17
PF07679.3   ..............................................................................................PAAKTVKFKCPSSGTP  16
PF07679.2   .............................................................................................VPAAKTVKFKCPSSGTP  17
PF00047     RTRITGEEVEVQDSVPADSGLYACVTSSPSGSDTTYFS........................................................................  73
PF00047.1   RTRITGEEVEVQDSVPADSGLYACVTS...................................................................................  62
PF07714     ..............................................................................................................   0
PF07679     ..............................................................................................PAAKTVKFKCPSSGTP  16
FGFR1-236   ..............................................................................................................   0
FGFR1-214   RTRITGEEVEVQDSVPADSGLYACVTSSPSGSDTTYFSVNVS...DALPSSEDDDDDDDSSSEEKETDNTKPNRMPVAPYWTSPEKMEKKLHAVPAAKTVKFKCPSSGTP 184
FGFR1-202   RTRITGEEVEVQDSVPADSGLYACVTSSPSGSDTTYFSVNVSD...ALPSSEDDDDDDDSSSEEKETDNTKPNRMPVAPYWTSPEKMEKKLHAVPAAKTVKFKCPSSGTP 176
FGFR1-201   .............................................DALPSSEDDDDDDDSSSEEKETDNTKPN..PVAPYWTSPEKMEKKLHAVPAAKTVKFKCPSSGTP  93
FGFR1-241   .............................................DALPSSEDDDDDDDSSSEEKETDNTKPN..PVAPYWTSPEKMEKKLHAVPAAKTVKFKCPSSGTP  93
FGFR1-209   RTRITGEEVEVQDSVPADSGLYACVTSSPSGSDTTYFSVNVS...DALPSSEDDDDDDDSSSEEKETDNTKPN..PVAPYWTSPEKMEKKLHAVPAAKTVKFKCPSSGTP 182
FGFR1-238   RTRITGEEVEVQDSVPADSGLYACVTSSPSGSDTTYFSVNVS...DALPSSEDDDDDDDSSSEEKETDNTKPNRMPVAPYWTSPEKMEKKLHAVPAAKTVKFKCPSSGTP 184
FGFR1-210   RTRITGEEVEVQDSVPADSGLYACVTSSP................................................................................. 106
FGFR1-208   RTRITGEEVEVQDSVPADSGLYACVTSSPSGSDTTYFSVNVS...DALPSSEDDDDDDDSSSEEKETDNTKPN..PVAPYWTSPEKMEKKLHAVPAAKTVKFKCPSSGTP 182
FGFR1-212   ...............................................LPSSEDDDDDDDSSSEEKETDN.........................................  54
FGFR1-204   ..............................................ALPSSEDDDDDDDSSSEEKETDNTKPNRMPVAPYWTSPEKMEKKLHAVPAAKTVKFKCPSSGTP  95
FGFR1-221   RTRITGEEVEVQDSVPADSGLYACVTSSPSGSDTTYFSVNVSA...CPDLQEAKWCSASF.......HSITP.....LPFG.....LGTRLSD................. 150
FGFR1-207   ..............................................ALPSSEDDDDDDDSSSEEKETDNTKPN..PVAPYWTSPEKMEKKLHAVPAAKTVKFKCPSSGTP  93
FGFR1-213   ..............................................................................................................   0
FGFR1-226   RTRITGEEVEVQDSVPADSGLYACVTSSPSGSDTTYFSVNVS...DALPSSEDDDDDDDSSSEEKETDNTKPNRMPVAPYWTSPEKMEKKLHAVPAAKTVKFKCPSSGTP 184
FGFR1-229   ..............................................ALPSSEDDDDDDDSSSEEKETDNTKPN..PVAPYWTSPEKMEKKLHAVPAAKTVKFKCPSSGTP  93
FGFR1-211   RTRITGEEVEVQDSVPADSGLYACVTSSPSGSDTTYFSVNVS...DALPSSEDDDDDDDSSSEEKETDNTKPN..PVAPYWTSPEKMEKKLHAVPAAKTVKFKCPSSGTP 215
FGFR1-234   ..............................................ALPSSEDDDDDDDSSSEEKETDNTKPN..PVAPYWTSPEKMEKKLHAVPAAKTVKF........  85
FGFR1-203   RTRITGEEVEVQDSVPADSGLYACVTSSPSGSDTTYFSVNVSVPIDALPSSEDDDDDDDSSSEEKETDNTKPN..PVAPYWTSPEKMEKKLHAVPAAKTVKFKCPSSGTP 185
FGFR1-240   ......................................................................................MEKKLHAVPAAKTVKFKCPSSGTP  24
FGFR1-222   ..............................................................................................................   0
FGFR1-233   .............................................DALPSSEDDDDDDDSSSEEKETDNTKPNRMPVAPYWTSPEKMEKKLHAVPAAKTVKFKCPSSGTP  95
FGFR1-206   RTRITGEEVEVQDSVPADSGLYACVTSSPSGSDTTYFSVNVS...DALPSSEDDDDDDDSSSEEKETDNTKPN..PVAPYWTSPEKMEKKLHAVPAAKTVKFKCPSSGTP 182
```

```
logo         NPTLRWLKNGKEFKPDHRIGGYKVRYATWSIIMDSVVPSDKGNYTCIVENEYGSINHTYQLDVVERSPHRPILQAGLPANKTVALGSNVEFMCKVYSDPQPHIQWLKHIE
PONATINIB    ..............................................................................................................    0
PF07679.1    NPTLRWLKNGKEFKPDHRIGGYKVRYATWSIIMDSVVPSDKGNYTCIVENEYGSINHTYQLDV..............PANKTVALGSNVEFMCKVYSDPQPHIQWLKHIE  112
PF07679.4    NPTLRWLKNGKEFKPDHRIGGYKVRYATWSIIMDSVVPSDKGNYTC................................................................   63
PF07679.3    NPTLRWLKNGKEFKPDHRIGGYKVRYATWSIIMDSVVPSDKGNYTCIVENEYGSINHTYQLDV...............................................   79
PF07679.2    NPTLRWLKNGKEFKPDHRIGGYKVRYATWSIIMDSVVPSDKGNYTCIVENE...........................................................   68
PF00047      ..............................................................................................................   73
PF00047.1    ..............................................................................................................   62
PF07714      ..............................................................................................................    0
PF07679      NPTLRWLKNGKEFKPDHRIGGYKVRYATWSIIMDSVVPSDKGNYTCIVENEYGSINHTYQLDV..............PANKTVALGSNVEFMCKVYSDPQPHIQWLKHIE  112
FGFR1-236    ..............................................................................................................    0
FGFR1-214    NPTLRWLKNGKEFKPDHRIGGYKVRYATWSIIMDSVVPSDKGNYTCIVENEYGSINHTYQLDVVERSPHRPILQAGLPANKTVALGSNVEFMCKVYSDPQPHIQWLKHIE  294
FGFR1-202    NPTLRWLKNGKEFKPDHRIGGYKVRYATWSIIMDSVVPSDKGNYTCIVENEYGSINHTYQLDVVERSPHRPILQAGLPANKTVALGSNVEFMCKVYSDPQPHIQWLKHIE  286
FGFR1-201    NPTLRWLKNGKEFKPDHRIGGYKVRYATWSIIMDSVVPSDKGNYTCIVENEYGSINHTYQLDVVERSPHRPILQAGLPANKTVALGSNVEFMCKVYSDPQPHIQWLKHIE  203
FGFR1-241    NPTLRWLKNGKEFKPDHRIGGYKVRYATWSIIMDSVVPSDKGNYTCIVENEYGSINHTYQLDVVERSPHRPILQAGLPANKTVALGSNVEFMCKVYSDPQPHIQWLKHIE  203
FGFR1-209    NPTLRWLKNGKEFKPDHRIGGYKVRYATWSIIMDSVVPSDKGNYTCIVENEYGSINHTYQLDVVERSPHRPILQAGLPANKTVALGSNVEFMCKVYSDPQPHIQWLKHIE  292
FGFR1-238    NPTLRWLKNGKEFKPDHRIGGYKVRYATWSIIMDSVVPSDKGNYTCIVENEYGSINHTYQLDVVERSPHRPILQAGLPANKTVALGSNVEFMCKVYSDPQPHIQWLKHIE  294
FGFR1-210    ..............................................................................................................  106
FGFR1-208    NPTLRWLKNGKEFKPDHRIGGYKVRYATWSIIMDSVVPSDKGNYTCIVENEYGSINHTYQLDVVERSPHRPILQAGLPANKTVALGSNVEFMCKVYSDPQPHIQWLKHIE  292
FGFR1-212    ..............................................................................................................   54
FGFR1-204    NPTLRWLKNGKEFKPDHRIGGYKVRYATWSIIMDSVVPSDKGNYTCIVENEYGSINHTYQLDVVERSPHRPILQAGLPANKTVALGSNVEFMCKVYSDPQPHIQWLKHIE  205
FGFR1-221    ..............................................................................................................  150
FGFR1-207    NPTLRWLKNGKEFKPDHRIGGYKVRYATWSIIMDSVVPSDKGNYTCIVENEYGSINHTYQLDVVERSPHRPILQAGLPANKTVALGSNVEFMCKVYSDPQPHIQWLKHIE  203
FGFR1-213    ..............................................................................................................    0
FGFR1-226    NPTLRWLKNGKEFKPDHRIGGYKVRYATWSIIMDSVVPSDKGNYTCIVENEYGSINHTYQLDVVERSPHRPILQAGLPANKTVALGSNVEFMCKVYSDPQPHIQWLKHIE  294
FGFR1-229    NPTLRWLKNGKEFKPDHRIGGYKVRYATWSIIMDSVVPSDKGNYTCIVENEY..........................................................  145
FGFR1-211    NPTLRWLKNGKEFKPDHRIGGYKVRYATWSIIMDSVVPSDKGNYTCIVENEYGSINHTYQLDVVERSPHRPILQAGLPANKTVALGSNVEFMCKVYSDPQPHIQWLKHIE  325
FGFR1-234    ..............................................................................................................   85
FGFR1-203    NPTLRWLKNGKEFKPDHRIGGYKVRYATWSIIMDSVVPSDKGNYTCIVENEYGSINHTYQLDVVERSPHRPILQAGLPANKTVALGSNVEFMCKVYSDPQPHIQWLKHIE  295
FGFR1-240    NPTLRWLKNGKEFKPDHRIGGYKVRYATWSIIMDSVVPSDKGNYTCIVENEYGSINHTYQLDVVERSPHRPILQAGLPANKTVALGSNVEFMCKVYSDPQPHIQWLKHIE  134
FGFR1-222    ..............................................................................................................    0
FGFR1-233    NPTLRWLKNGKEFKPDHRIGGYKVRYATWSIIMDSVVPSDKGNYTCI...............................................................  142
FGFR1-206    NPTLRWLKNGKEFKPDHRIGGYKVRYATWSIIMDSVVPSDKGNYTCIVENEYGSINHTYQLDVVERSPHRPILQAGLPANKTVALGSNVEFMCKVYSDPQPHIQWLKHIE  292
```

logo

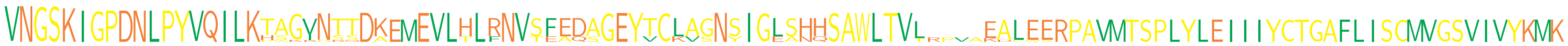

```
PONATINIB    .............................................................................................................. 0
PF07679.1    VNGSKIGPDNLPYVQILKHSGINSSDA..EVLTLFNVTEAQSGEYVCKVSNYIGEANQSAWLTV.............................................. 174
PF07679.4    .............................................................................................................. 63
PF07679.3    .............................................................................................................. 79
PF07679.2    .............................................................................................................. 68
PF00047      .............................................................................................................. 73
PF00047.1    .............................................................................................................. 62
PF07714      .............................................................................................................. 0
PF07679      VNGSKIGPDNLPYVQILKTAGVNTTDKEMEVLHLRNVSFEDAGEYTCLAGNSIGLSHHSAWLTV.............................................. 176
FGFR1-236    .............................................................................................................. 0
FGFR1-214    VNGSKIGPDNLPYVQILKTAGVNTTDKEMEVLHLRNVSFEDAGEYTCLAGNSIGLSHHSAWLTVL....EALEERPAVMTSPLYLEIIIYCTGAFLISCMVGSVIVYKMK 400
FGFR1-202    VNGSKIGPDNLPYVQILKTAGVNTTDKEMEVLHLRNVSFEDAGEYTCLAGNSIGLSHHSAWLTVL....EALEERPAVMTSPLYLEIIIYCTGAFLISCMVGSVIVYKMK 392
FGFR1-201    VNGSKIGPDNLPYVQILKTAGVNTTDKEMEVLHLRNVSFEDAGEYTCLAGNSIGLSHHSAWLTVL....EALEERPAVMTSPLYLEIIIYCTGAFLISCMVGSVIVYKMK 309
FGFR1-241    VNGSKIGPDNLPYVQILKPWKRGRQ..................................................................................... 228
FGFR1-209    VNGSKIGPDNLPYVQILKTAGVNTTDKEMEVLHLRNVSFEDAGEYTCLAGNSIGLSHHSAWLTVL....EALEERPAVMTSPLYLEIIIYCTGAFLISCMVGSVIVYKMK 398
FGFR1-238    VNGSKIGPDNLPYVQILKTAGVNTTDKEMEVLHLRNVSFEDAGEYTCLAGNSIGLSHHSAWLTVL....EALEERPAVMTSPLYLEIIIYCTGAFLISCMVGSVIVYKMK 400
FGFR1-210    .............................................................................................................. 106
FGFR1-208    VNGSKIGPDNLPYVQILKTAGVNTTDKEMEVLHLRNVSFEDAGEYTCLAGNSIGLSHHSAWLTVL....EALEERPAVMTSPLYLEIIIYCTGAFLISCMVGSVIVYKMK 398
FGFR1-212    .............................................................................................................. 54
FGFR1-204    VNGSKIGPDNLPYVQILKTAGVNTTDKEMEVLHLRNVSFEDAGEYTCLAGNSIGLSHHSAWLTVL....EALEERPAVMTSPLYLEIIIYCTGAFLISCMVGSVIVYKMK 311
FGFR1-221    .............................................................................................................. 150
FGFR1-207    VNGSKIGPDNLPYVQILKHSGINSSDA..EVLTLFNVTEAQSGEYVCKVSNYIGEANQSAWLTVTRPVAKALEERPAVMTSPLYLEIIIYCTGAFLISCMVGSVIVYKMK 311
FGFR1-213    .............................................................................................................. 0
FGFR1-226    VNGS.......................................................................................................... 298
FGFR1-229    .............................................................................................................. 145
FGFR1-211    VNGSKIGPDNLPYVQILKTAGVNTTDKEMEVLHLRNVSFEDAGEYTCLAGNSIGLSHHSAWLTVL....EALEERPAVMTSPLYLEIIIYCTGAFLISCMVGSVIVYKMK 431
FGFR1-234    .............................................................................................................. 85
FGFR1-203    VNGSKIGPDNLPYVQILKHSGINSSDA..EVLTLFNVTEAQSGEYVCKVSNYIGEANQSAWLTVTRPVAKDCWS................................... 367
FGFR1-240    VNGSK......................................................................................................... 139
FGFR1-222    .............................................................................................................. 0
FGFR1-233    .............................................................................................................. 142
FGFR1-206    VNGSKIGPDNLPYVQILKTAGVNTTDKEMEVLHLRNVSFEDAGEYTCLAGNSIGLSHHSAWLTVL....EALEERPAVMTSPLYLEIIIYCTGAFLISCMVGSVIVYKMK 398
```

logo

```
            SGTKKSDFHSQMAVHKLAKSIPLRRQVTvSADSSASMNSGVLLVRPSRLSSSGTPMLAGVSEYELPEDPRWELPRDRLVLGKPLGEGCFGQVVLAEAIGLDKDKPNRVTK
PONATINIB   ..............................................................................................................     0
PF07679.1   ..............................................................................................................   174
PF07679.4   ..............................................................................................................    63
PF07679.3   ..............................................................................................................    79
PF07679.2   ..............................................................................................................    68
PF00047     ..............................................................................................................    73
PF00047.1   ..............................................................................................................    62
PF07714     ..............................................................................VLGKPLGEGCFGQVVLAEAIGLDKDKPNRVTK    32
PF07679     ..............................................................................................................   176
FGFR1-236   ..............................................................................................................     0
FGFR1-214   SGTKKSDFHSQMAVHKLAKSIPLRRQVTVSADSSASMNSGVLLVRPSRLSSSGTPMLAGVSEYELPEDPRWELPRDRLVLGKPLGEGCFGQVVLAEAIGLDKDKPNRVTK   510
FGFR1-202   SGTKKSDFHSQMAVHKLAKSIPLRRQV..SADSSASMNSGVLLVRPSRLSSSGTPMLAGVSEYELPEDPRWELPRDRLVLGKPLGEGCFGQVVLAEAIGLDKDKPNRVTK   500
FGFR1-201   SGTKKSDFHSQMAVHKLAKSIPLRRQVTVSADSSASMNSGVLLVRPSRLSSSGTPMLAGVSEYELPEDPRWELPRDRLVLGKPLGEGCFGQVVLAEAIGLDKDKPNRVTK   419
FGFR1-241   ..............................................................................................................   228
FGFR1-209   SGTKKSDFHSQMAVHKLAKSIPLRRQVTVSADSSASMNSGVLLVRPSRLSSSGTPMLAGVSEYELPEDPRWELPRDRLVLGKPLGEGCFGQVVLAEAIGLDKDKPNRVTK   508
FGFR1-238   SGTKKSDFHSQMAVHKLAKSIPLRRQV..SADSSASMNSGVLLVRPSRLSSSGTPMLAGVSEYELPEDPRWELPRDRLVLGKPLGEGCFGQVVLAEAIGLDKDKPNRVTK   508
FGFR1-210   ..............................................................................................................   106
FGFR1-208   SGTKKSDFHSQMAVHKLAKSIPLRRQVTVSADSSASMNSGVLLVRPSRLSSSGTPMLAGVSEYELPEDPRWELPRDRLVLGKPLGEGCFGQVVLAEAIGLDKDKPNRVTK   508
FGFR1-212   ..............................................................................................................    54
FGFR1-204   SGTKKSDFHSQMAVHKLAKSIPLRRQVTVSADSSASMNSGVLLVRPSRLSSSGTPMLAGVSEYELPEDPRWELPRDRLVLGKPLGEGCFGQVVLAEAIGLDKDKPNRVTK   421
FGFR1-221   ..............................................................................................................   150
FGFR1-207   SGTKKSDFHSQMAVHKLAKSIPLRRQVTVSADSSASMNSGVLLVRPSRLSSSGTPMLAGVSEYELPEDPRWELPRDRLVLGKPLGEGCFGQVVLAEAIGLDKDKPNRVTK   421
FGFR1-213   ..............................................................................................................     0
FGFR1-226   ..............................................................................................................   298
FGFR1-229   ..............................................................................................................   145
FGFR1-211   SGTKKSDFHSQMAVHKLAKSIPLRRQVTVSADSSASMNSGVLLVRPSRLSSSGTPMLAGVSEYELPEDPRWELPRDRLVLGKPLGEGCFGQVVLAEAIGLDKDKPNRVTK   541
FGFR1-234   ..............................................................................................................    85
FGFR1-203   ..............................................................................................................   367
FGFR1-240   ..............................................................................................................   139
FGFR1-222   ..............................................................................................................     0
FGFR1-233   ..............................................................................................................   142
FGFR1-206   SGTKKSDFHSQMAVHKLAKSIPLRRQVTVSADSSASMNSGVLLVRPSRLSSSGTPMLAGVSEYELPEDPRWELPRDRLVLGKPLGEGCFGQVVLAEAIGLDKDKPNRVTK   508
```

```
logo
              VAVKMLKSDATEKDLSDLISEMEMMKMIGKHKNIINLLGACTQDGPLYVIVEYASKGNLREYLQARRPPGLEYCYNPSHNPEEQLSSKDLVSCAYQV      ARGMEYL
PONATINIB     ..................................................................................L.......V...................     2
PF07679.1     ..............................................................................................................   174
PF07679.4     ..............................................................................................................    63
PF07679.3     ..............................................................................................................    79
PF07679.2     ..............................................................................................................    68
PF00047       ..............................................................................................................    73
PF00047.1     ..............................................................................................................    62
PF07714       VAVKMLKSDATEKDLSDLISEMEMMKMIGKHKNIINLLGACTQDGPLYVIVEYASKGNLREYLQARRPPGLEYCYNPSHNPEEQLSSKDLVSCAYQV......ARGMEYL   136
PF07679       ..............................................................................................................   176
FGFR1-236     ..........................................................................................VSCAYQV......ARGMEYL    14
FGFR1-214     VAVKMLKSDATEKDLSDLISEMEMMKMIGKHKNIINLLGACTQDGPLYVIVEYASKGNLREYLQARRPPGLEYCYNPSHNPEEQLSSKDLVSCAYQV......ARGMEYL   614
FGFR1-202     VAVKMLKSDATEKDLSDLISEMEMMKMIGKHKNIINLLGACTQDGPLYVIVEYASKGNLREYLQARRPPGLEYCYNPSHNPEEQLSSKDLVSCAYQV......ARGMEYL   604
FGFR1-201     VAVKMLKSDATEKDLSDLISEMEMMKMIGKHKNIINLLGACTQDGPLYVIVEYASKGNLREYLQARRPPGLEYCYNPSHNPEEQLSSKDLVSCAYQV......ARGMEYL   523
FGFR1-241     ..............................................................................................................   228
FGFR1-209     VAVKMLKSDATEKDLSDLISEMEMMKMIGKHKNIINLLGACTQDGPLYVIVEYASKGNLREYLQARRPPGLEYCYNPSHNPEEQLSSKDLVSCAYQV......ARGMEYL   612
FGFR1-238     VAVKMLKSDATEKDLSDLISEMEMMKMIGKHKNIINLLGACTQDGPLYVIVEYASKGNLREYLQARRPPGLEYCYNPSHNPEEQLSSKDLVSCAYQV......ARGMEYL   612
FGFR1-210     ..............................................................................................................   106
FGFR1-208     VAVKMLKSDATEKDLSDLISEMEMMKMIGKHKNIINLLGACTQDGPLYVIVEYASKGNLREYLQARRPPGLEYCYNPSHNPEEQLSSKDLVSCAYQV......ARGMEYL   612
FGFR1-212     ..............................................................................................................    54
FGFR1-204     VAVKMLKSDATEKDLSDLISEMEMMKMIGKHKNIINLLGACTQDGPLYVIVEYASKGNLREYLQARRPPGLEYCYNPSHNPEEQLSSKDLVSCAYQV......ARGMEYL   525
FGFR1-221     ..............................................................................................................   150
FGFR1-207     VAVKMLKSDATEKDLSDLISEMEMMKMIGKHKNIINLLGACTQDGPLYVIVEYASKGNLREYLQARRPPGLEYCYNPSHNPEEQLSSKDLVSCAYQV......ARGMEYL   525
FGFR1-213     ..............................................................................................................     0
FGFR1-226     ..............................................................................................................   298
FGFR1-229     ..............................................................................................................   145
FGFR1-211     VAVKMLKSDATEKDLSDLISEMEMMKMIGKHKNIINLLGACTQDGPLYVIVEYASKGNLREYLQARRPPGLEYCYNPSHNPEEQLSSKDLVSCAYQV......ARGMEYL   645
FGFR1-234     ..............................................................................................................    85
FGFR1-203     ..............................................................................................................   367
FGFR1-240     ..............................................................................................................   139
FGFR1-222     ..............................................................................................................     0
FGFR1-233     ..............................................................................................................   142
FGFR1-206     VAVKMLKSDATEKDLSDLISEMEMMKMIGKHKNIINLLGACTQDGPLYVIVEYASKGNLREYLQARRPPGLEYCYNPSHNPEEQLSSKDLVSCAYQV......ARGMEYL   612
```

```
logo
            ASKKCIHRDLA ARNVLVTEDNVMKIADFGLARDIHHIDYYKKTTNGRLPVKWMAPEALFDRIYTHQSDVWSFGVLL.WEIFTLG....GSPYPGVPVEELFKLLKEGHR
PONATINIB   AVK................E..MM..........I...............VEYA........................................................  13
PF07679.1   ..............................................................................................................  174
PF07679.4   ..............................................................................................................  63
PF07679.3   ..............................................................................................................  79
PF07679.2   ..............................................................................................................  68
PF00047     ..............................................................................................................  73
PF00047.1   ..............................................................................................................  62
PF07714     ASKKCIHRDLA.ARNVLVTEDNVMKIADFGLARDIHHIDYYKKTTNGRLPVKWMAPEALFDRIYTHQSDVWSFGVLL.WEIFTLG....GSPYPGVPVEELFKLLKEGHR  240
PF07679     ..............................................................................................................  176
FGFR1-236   ASKKCIHRDLA.ARNVLVTEDNVMKIADFGLARDIHHIDYYKKTTNGRLPVKWMAPEALFDRIYTHQSDVWSFGVLL.WEIFTLG....GSPYPGVPVEELFKLLKEGHR  118
FGFR1-214   ASKKCIHRDLA.ARNVLVTEDNVMKIADFGLARDIHHIDYYKKTTNGRLPVKWMAPEALFDRIYTHQSDVWSFGVLL.WEIFTLG....GSPYPGVPVEELFKLLKEGHR  718
FGFR1-202   ASKKCIHRDLA.ARNVLVTEDNVMKIADFGLARDIHHIDYYKKTTNGRLPVKWMAPEALFDRIYTHQSDVWSFGVLL.WEIFTLG....GSPYPGVPVEELFKLLKEGHR  708
FGFR1-201   ASKKCIHRDLA.ARNVLVTEDNVMKIADFGLARDIHHIDYYKKTTNGRLPVKWMAPEALFDRIYTHQSDVWSFGVLL.WEIFTLG....GSPYPGVPVEELFKLLKEGHR  627
FGFR1-241   ..............................................................................................................  228
FGFR1-209   ASKKCIHRDLA.ARNVLVTEDNVMKIADFGLARDIHHIDYYKKTTNGRLPVKWMAPEALFDRIYTHQSDVWSFGVLL.WEIFTLG....GSPYPGVPVEELFKLLKEGHR  716
FGFR1-238   ASKKCIHRDLA.ARNVLVTEDNVMKIADFGLARDIHHIDYYKKTTNGRLPVKWMAPEALFDRIYTHQSDVWSFGVLL.WEIFTLG....GSPYPGVPVEELFKLLKEGHR  716
FGFR1-210   ..............................................................................................................  106
FGFR1-208   ASKKCIHRDLA.ARNVLVTEDNVMKIADFGLARDIHHIDYYKKTTNGRLPVKWMAPEALFDRIYTHQSDVWSFGVLL.WEIFTLG....GSPYPGVPVEELFKLLKEGHR  716
FGFR1-212   ..............................................................................................................  54
FGFR1-204   ASKKCIHRDLA.ARNVLVTEDNVMKIADFGLARDIHHIDYYKKTTNGRLPVKWMAPEALFDRIYTHQSDVWSFGVLL.WEIFTLG....GSPYPGVPVEELFKLLKEGHR  629
FGFR1-221   ..............................................................................................................  150
FGFR1-207   ASKKCIHRDLA.ARNVLVTEDNVMKIADFGLARDIHHIDYYKKTTNGRLPVKWMAPEALFDRIYTHQSDVWSFGVLL.WEIFTLG....GSPYPGVPVEELFKLLKEGHR  629
FGFR1-213   ......................M.......................................................................................  1
FGFR1-226   ..............................................................................................................  298
FGFR1-229   ..............................................................................................................  145
FGFR1-211   ASKKCIHRDLA.ARNVLVTEDNVMKIADFGLARDIHHIDYYKKTTNGRLPVKWMAPEALFDRIYTHQSDVWSFGVLL.WEIFTLG....GSPYPGVPVEELFKLLKEGHR  749
FGFR1-234   ..............................................................................................................  85
FGFR1-203   ..............................................................................................................  367
FGFR1-240   ..............................................................................................................  139
FGFR1-222   .....................................................................MWSWKCLLFWAVLVTATLCTARPSPTLPEQACPDL......  35
FGFR1-233   ..............................................................................................................  142
FGFR1-206   ASKKCIHRDLA.ARNVLVTEDNVMKIADFGLARDIHHIDYYKKTTNGRLPVKWMAPEALFDRIYTHQSDVWSFGVLL.WEIFTLG....GSPYPGVPVEELFKLLKEGHR  716
```

```
logo
             MDKPSNCTNELYMMMRDCWHAVPSQRPTFKQLVEDLDRIVALTSNQEYLDLSMPLDQYSPSFPDTRSSTCSSGEDSVFSHEPLPEEPCLPRHPAQLANGGLKRR
PONATINIB    .L....CIHR.......L........IADFGL........................................................................   25
PF07679.1    ........................................................................................................  174
PF07679.4    ........................................................................................................   63
PF07679.3    ........................................................................................................   79
PF07679.2    ........................................................................................................   68
PF00047      ........................................................................................................   73
PF00047.1    ........................................................................................................   62
PF07714      MDKPSNCTNELYMMMRDCWHAVPSQRPTFKQLVEDL....................................................................  276
PF07679      ........................................................................................................  176
FGFR1-236    MDKPSNCTNELPAAG................T........................................................................  134
FGFR1-214    MDKPSNCTNELYMMMRDCWHAVPSQRPTFKQLVEDLDRIVALTSNQEYLDLSMPLDQYSPSFPDTRSSTCSSGEDSVFSHEPLPEEPCLPRHPAQLANGGLKRR  822
FGFR1-202    MDKPSNCTNELYMMMRDCWHAVPSQRPTFKQLVEDLDRIVALTSNQEYLDLSMPLDQYSPSFPDTRSSTCSSGEDSVFSHEPLPEEPCLPRHPAQLANGGLKRR  812
FGFR1-201    MDKPSNCTNELYMMMRDCWHAVPSQRPTFKQLVEDLDRIVALTSNQEYLDLSMPLDQYSPSFPDTRSSTCSSGEDSVFSHEPLPEEPCLPRHPAQLANGGLKRR  731
FGFR1-241    ........................................................................................................  228
FGFR1-209    MDKPSNCTNELYMMMRDCWHAVPSQRPTFKQLVEDLDRIVALTSNQEYLDLSMPLDQYSPSFPDTRSSTCSSGEDSVFSHEPLPEEPCLPRHPAQLANGGLKRR  820
FGFR1-238    MDKPSNCTNELYMMMRDCWHAVPSQRPTFKQLVEDLDRIVALTSNQEYLDLSMPLDQYSPSFPDTRSSTCSSGEDSVFSHEPLPEEPCLPRHPAQLANGGLKRR  820
FGFR1-210    ........................................................................................................  106
FGFR1-208    MDKPSNCTNELYMMMRDCWHAVPSQRPTFKQLVEDLDRIVALTSNQEYLDLSMPLDQYSPSFPDTRSSTCSSGEDSVFSHEPLPEEPCLPRHPAQLANGGLKRR  820
FGFR1-212    ........................................................................................................   54
FGFR1-204    MDKPSNCTNELYMMMRDCWHAVPSQRPTFKQLVEDLDRIVALTSNQEYLDLSMPLDQYSPSFPDTRSSTCSSGEDSVFSHEPLPEEPCLPRHPAQLANGGLKRR  733
FGFR1-221    ........................................................................................................  150
FGFR1-207    MDKPSNCTNELYMMMRDCWHAVPSQRPTFKQLVEDLDRIVALTSNQEYLDLSMPLDQYSPSFPDTRSSTCSSGEDSVFSHEPLPEEPCLPRHPAQLANGGLKRR  733
FGFR1-213    ........................................................................................................    1
FGFR1-226    ........................................................................................................  298
FGFR1-229    ........................................................................................................  145
FGFR1-211    MDKPSNCTNELYMMMRDCWHAVPSQRPTFKQLVEDLDRIVALTSNQEYLDLSMPLDQYSPSFPDTRSSTCSSGEDSVFSHEPLPEEPCLPRHPAQLANGGLKRR  853
FGFR1-234    ........................................................................................................   85
FGFR1-203    ........................................................................................................  367
FGFR1-240    ........................................................................................................  139
FGFR1-222    .QEAKWCSASFHSI...........TPLPFGLGTRLSD..................................................................   61
FGFR1-233    ........................................................................................................  142
FGFR1-206    MDKPSNCTNELYMMMRDCWHAVPSQRPTFKQLVEDLDRIVALTSNQEYLDLSMPLDQYSPSFPDTRSSTCSSGEDSVFSHEPLPEEPCLPRHPAQLANGGLKRR  820
```

X non conserved

X similar

X ≥ 0% conserved

X ≥ 50% conserved

```
logo        MRLLLALLGVLLSVPGPPVLSLEASEEVELEPCLAPSLEQQEQELTVALGQPVRLCCGRAERGGHWYKEGSRLAPAGRVRGWRGRLEIASFLPEDAGRYLCLARGSMIVL
DOVITINIB   ..............................................................................................................    0
PF13927     ..............................................................................................................    0
PF07679.1   ..............................................................................................................    0
PF07714.1   ..............................................................................................................    0
PF07679     ..............................................................................................................    0
PF07714     ..............................................................................................................    0
FGFR4-208   MRLLLALLGVLLSVPGPPVLSLEASEEVELEPCLAPSLEQQEQELTVALGQPVRLCCGRAERGGHWYKEGSRLAPAGRVRGWRGRLEIASFLPEDAGRYLCLARGSMIVL  110
FGFR4-203   MRLLLALLGVLLSVPGPPVLSLEASEEVELEPCLAPSLEQQEQELTVALGQPVRLCCGRAERGGHWYKEGSRLAPAGRVRGWRGRLEIASFLPEDAGRYLCLARGSMIVL  110
FGFR4-214   MRLLLALLGVLLSVPGPPVLSLEASEEVELEPCLAPSLEQQEQELTVALGQPVRLCCGRAERGGHWYKEGSRLAPAGRVRGWRGRLEIASFLPEDAGRYLCLARGSMI..  108
FGFR4-216   MRLLLALLGVLLSVPGPPVLSLEASEEVELEPCLAPSLEQQEQELTVALGQPVRLCCGRAERGGHWYKEGSRLAPAGRVRGWRGRLEIASFLPEDAGRYLCLARGSMIVL  110
FGFR4-202   MRLLLALLGVLLSVPGPPVLSLEASEEVELEPCLAPSLEQQEQELTVALGQPVRLCCGRAERGGHWYKEGSRLAPAGRVRGWRGRLEIASFLPEDAGRYLCLARGSMIVL  110
FGFR4-207   MRLLLALLGVLLSVPGPPVLSLEASEEVELEPCLAPSLEQQEQELTVALGQPVRLCCGRAERGGHWYKEGSRLAPAGRVRGWRGRLEIASFLPEDAGRYLCLARGSMIVL  110
FGFR4-201   MRLLLALLGVLLSVPGPPVLSLEASEEVELEPCLAPSLEQQEQELTVALGQPVRLCCGRAERGGHWYKEGSRLAPAGRVRGWRGRLEIASFLPEDAGRYLCLARGSMIVL  110
FGFR4-212   MRLLLALLGVLLSVPGPPVLSLEASEEVELEPCLAPSLEQQEQELTVALGQPVRLCCGRAERGGHWYKEGSRLAPAGRVRGWRGRLE.......................   87
FGFR4-213   ..............................................................................................................    0
FGFR4-205   MRLLLALLGVLLSVPGPPVLSLEASEEVELASIHLSLCLRERT..............GLAG...............RRALSWAAELVSPAWLPAWSSKSRS.........   72

logo        QNLTLITGDSLTSSNDDEDPKSHRDPSNRHSYPQQAPYWTHPQRMEKKLHAVPAGNTVKFRCPAAGNPTPTIRWLKDGQAFHGENRIGGIRLRHQHWSLVMESVVPSDRG
DOVITINIB   ..............................................................................................................    0
PF13927     ..............................................................................................................    0
PF07679.1   ..................................................AVPAGNTVKFRCPAAGNPTPTIRWLKDGQAFHGENR........................   36
PF07714.1   ..............................................................................................................    0
PF07679     ..................................................AVPAGNTVKFRCPAAGNPTPTIRWLKDGQAFHGENRIGGIRLRHQHWSLVMESVVPSDRG   60
PF07714     ..............................................................................................................    0
FGFR4-208   QNLTLITGDSLTSSNDDEDPKSHRDPSNRHSYPQQAPYWTHPQRMEKKLHAVPAGNTVKFRCPAAGNPTPTIRWLKDGQAFHGENRIGGIR...................  201
FGFR4-203   QNLTLITGDSLTSSNDDEDPKSHRDPSNRHSYPQQAPYWTHPQRMEKKLHAVPAGNTVKFRCPAAGNPTPTIRWLKDGQAFHGENRIGGIRLRHQHWSLVMESVVPSDRG  220
FGFR4-214   ..............................................................................................................  108
FGFR4-216   QNLT..........................................................................................................  114
FGFR4-202   QNLTLITGDSLTSSNDDEDPKSHRDPSNRHSYPQQAPYWTHPQRMEKKLHAVPAGNTVKFRCPAAGNPTPTIRWLKDGQAFHGENRIGGIRLRHQHWSLVMESVVPSDRG  220
FGFR4-207   QNLTLITGDSLTSSNDDEDPKSHRDPSNRHSYPQQAPYWTHPQRMEKKLHAVPAGNTVKFRCPAAGNPTPTIRWLKDGQAFHGENRIGGIRLRHQHWSLVMESVVPSDRG  220
FGFR4-201   QNLTLITGDSLTSSNDDEDPKSHRDPSNRHSYPQQAPYWTHPQRMEKKLHAVPAGNTVKFRCPAAGNPTPTIRWLKDGQAFHGENRIGGIRLRHQHWSLVMESVVPSDRG  220
FGFR4-212   ..............................................................................................................   87
FGFR4-213   ..............................................................................................................    0
FGFR4-205   ..............................................................................................................   72
```

```
logo
```

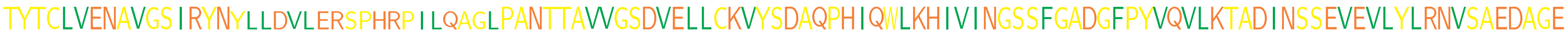

```
DOVITINIB    ..............................................................................................................    0
PF13927      ...................................PANTTAVVGSDVELLCKVYSDAQPHIQWLKHIVINGSSFGADGFPYVQVLKTADINSSEVEVLYLRNVSAEDAGE   75
PF07679.1    ..............................................................................................................   36
PF07714.1    ..............................................................................................................    0
PF07679      TYTCLVENAVGSIRYN..............................................................................................   76
PF07714      ..............................................................................................................    0
FGFR4-208    ..............................................................................................................  201
FGFR4-203    TYTCLVENAVGSIRYNYLLDVLERSPHRPILQAGLPANTTAVVGSDVELLCKVYSDAQPHIQWLKHIVINGSSFGADGFPYVQVLKTADINSSEVEVLYLRNVSAEDAGE  330
FGFR4-214    ..............................................................................................................  108
FGFR4-216    ..............................................................................................................  114
FGFR4-202    TYTCLVENAVGSIRYNYLLDVLERSPHRPILQAGLPANTTAVVGSDVELLCKVYSDAQPHIQWLKHIVINGSSFGADGFPYVQVLKTADINSSEVEVLYLRNVSAEDAGE  330
FGFR4-207    TYTCLVENAVGSIRYNYLLDVLERSPHRPILQAGLPANTTAVVGSDVELLCKVYSDAQPHIQWLKHIVINGSSFGADGFPYVQVLKTADINSSEVEVLYLRNVSAEDAGE  330
FGFR4-201    TYTCLVENAVGSIRYNYLLDVLERSPHRPILQAGLPANTTAVVGSDVELLCKVYSDAQPHIQWLKHIVINGSSFGADGFPYVQVLKTADINSSEVEVLYLRNVSAEDAGE  330
FGFR4-212    ..............................................................................................................   87
FGFR4-213    ..............................................................................................................    0
FGFR4-205    ..............................................................................................................   72
```

```
logo
```

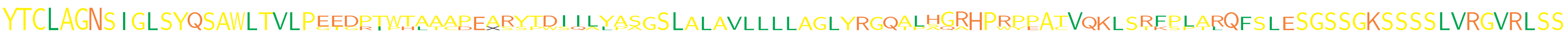

```
DOVITINIB    ..............................................................................................................    0
PF13927      YTCLAGN.......................................................................................................   82
PF07679.1    ..............................................................................................................   36
PF07714.1    ..............................................................................................................    0
PF07679      ..............................................................................................................   76
PF07714      ..............................................................................................................    0
FGFR4-208    ..............................................................................................................  201
FGFR4-203    YTCLAGNSIGLSYQSAWLTVLPEEDPTWTAAAPEASSPWSQALPA....................SQAHPWYEACV..................................  386
FGFR4-214    ..............................................................................................................  108
FGFR4-216    ..............................................................................................................  114
FGFR4-202    YTCLAGNSIGLSYQSAWLTVLPGTGRIPHLTCD.............SL...............TPAGR............TKSPTLQFSLESGSSGKSSSSLVRGVRLSS  400
FGFR4-207    YTCLAGNSIGLSYQSAWLTVLPEEDPTWTAAAPEARYTDIILYASGSLALAVLLLLAGLYRGQALHGRHPRPPATVQKLSRFPLARQFSLESGSSGKSSSSLVRGVRLSS  440
FGFR4-201    YTCLAGNSIGLSYQSAWLTVLPEEDPTWTAAAPEARYTDIILYASGSLALAVLLLLAGLYRGQALHGRHPRPPATVQKLSRFPLARQFSLESGSSGKSSSSLVRGVRLSS  440
FGFR4-212    ..............................................................................................................   87
FGFR4-213    ..................................XRYTDIILYASGSLALAVLLLLAGLYRGQALHGRHPRPPATVQKLSRFPLARQ....SGSSGKSSSSLVRGVRLSS   72
FGFR4-205    ..............................................................................................................   72
```

```
logo
           SGPALLAGLVSLDLPLDPLWEFPRDRLVLGKPLGEGCFGQVVRAEAFGMDPARPDQASTVAVKMLKDNASDKDLADLVSEMEVMKLIGRHKNIINLLGVCTQEGPLYVIV
DOVITINIB  .............................................................................................I...............V    2
PF13927    ..............................................................................................................   82
PF07679.1  ..............................................................................................................   36
PF07714.1  ...........................VLGKPLGEGCFGQVVRAEAFGMDPARPDQASTVAVKMLKDNASDKDLADLVSEMEVMKLIGRHKNIINLLGVCTQEGPLYVIV   83
PF07679    ..............................................................................................................   76
PF07714    ...........................VLGKPLGEGCFGQVVRAEAFGMDPARPDQASTVAVKMLKDNASDKDLADLVSEMEVMKLIGRHKNIINLLGVCTQEGPLYVIV   83
FGFR4-208  ..............................................................................................................  201
FGFR4-203  ............SPPAA..PPCSPASLVLGKPLGEGCFGQVVRAEAFGMDPARPDQASTVAVKMLKDNASDKDLADLVSEMEVMKLIGRHKNIINLLGVCTQEGPLYVIV  482
FGFR4-214  ..............................................................................................................  108
FGFR4-216  ..............................................................................................................  114
FGFR4-202  SGPALLAGLVSLDLPLDPLWEFPRDRLVLGKPLGEGCFGQVVRAEAFGMDPARPDQASTVAVKMLKDNASDKDLADLVSEMEVMKLIGRHKNIINLLGVCTQEGPLYVIV  510
FGFR4-207  SGPALLAGLVSLDLPLDPLWEFPRDRLVLGKPLGEGCFGQVVRAEAFGMDPARPDQASTVAVKMLKDNASDKDLADLVSEMEVMKLIGRHKNIINLLGVCTQEGPLYVIV  550
FGFR4-201  SGPALLAGLVSLDLPLDPLWEFPRDRLVLGKPLGEGCFGQVVRAEAFGMDPARPDQASTVAVKMLKDNASDKDLADLVSEMEVMKLIGRHKNIINLLGVCTQEGPLYVIV  550
FGFR4-212  ..............................................................................................................   87
FGFR4-213  SGPALLAGLVSLDLPLDPLWEFPRDRLVLGKPLGEGCFGQVVRAEAFGMDPARPDQASTVAVKMLKDNASDKDLADLVSEMEVMKLIGRHKNIINLLGVCTQEGPLYVIV  182
FGFR4-205  ..............................................................................................................   72

logo
           ECAAKGNLREFLRARRPPGPDLSPDGPRSSEGPLSFPVLVSCAYQVARGMQYLESRKCIHRDLAARNVLVTEDNVMKIADFGLARGVHHIDYYKKTSNGRLPVKWMAPEA
DOVITINIB  .CAA.G............................................................................L...........................    7
PF13927    ..............................................................................................................   82
PF07679.1  ..............................................................................................................   36
PF07714.1  ECAAKGNLREFLRARRPPGPDLSPDGPRSSEGPLSFPVLVSCAYQVARGMQYLESRKCIHRDLAARNVLVTEDNVMKIADFGLARGVHHIDYYKKTS.............  180
PF07679    ..............................................................................................................   76
PF07714    ECAAKGNLREFLRARRPPGPDLSPDGPRSSEGPLSFPVLVSCAYQVARGMQYLESRKCIHRDLAARNVLVTEDNVMKIADFGLARGVHHIDYYKKTSNGRLPVKWMAPEA  193
FGFR4-208  ..............................................................................................................  201
FGFR4-203  ECAAKGNLREFLRARRPPGPDLSPDGPRSSEGPLSFPVLVSCAYQVARGMQYLESRKCIHRDLAARNVLVTEDNVMKIADFGLARGVHHIDYYKKTSNGRLPVKWMAPEA  592
FGFR4-214  ..............................................................................................................  108
FGFR4-216  ..............................................................................................................  114
FGFR4-202  ECAAKGNLREFLRARRPPGPDLSPDGPRSSEGPLSFPVLVSCAYQVARGMQYLESRKCIHRDLAARNVLVTEDNVMKIADFGLARGVHHIDYYKKTSNGRLPVKWMAPEA  620
FGFR4-207  ECAAKGNLREFLRARRPPGPDLSPDGPRSSEGPLSFPVLVSCAYQVARGMQYLESRKCIHRDLAARNVLVTEDNVMKIADFGLARGVHHIDYYKKTSNGRLPVKWMAPEA  660
FGFR4-201  ECAAKGNLREFLRARRPPGPDLSPDGPRSSEGPLSFPVLVSCAYQVARGMQYLESRKCIHRDLAARNVLVTEDNVMKIADFGLARGVHHIDYYKKTSNGRLPVKWMAPEA  660
FGFR4-212  ..............................................................................................................   87
FGFR4-213  ECAAKGNLREFLRARRPPGPDLSPDGPRSSEGPLSFPVLVSCAYQVARGMQYLESRKCIHRDLAARNVLVTEDNVMKIADFGLARGVHHIDYYKKTSN............  280
FGFR4-205  ..............................................................................................................   72
```

```
logo
             LFDRVYTHQSDVWSFGILLWEIFTLGGSPYPGIPVEELFSLLREGHRMDRPPHCPPELYGLMRECWHAAPSQRPTFKQLVEALDKVLLAVSEEYLDLRLTFGPYSPSGGD
DOVITINIB    ..............................................................................................................    7
PF13927      ..............................................................................................................   82
PF07679.1    ..............................................................................................................   36
PF07714.1    ..............................................................................................................  180
PF07679      ..............................................................................................................   76
PF07714      LFDRVYTHQSDVWSFGILLWEIFTLGGSPYPGIPVEELFSLLREGHRMDRPPHCPPELYGLMRECWHAAPSQRPTFKQLVEAL...........................  276
FGFR4-208    ..............................................................................................................  201
FGFR4-203    LFDRVYTHQSDVWSFGILLWEIFTLGGSPYPGIPVEELFSLLREGHRMDRPPHCPPELYGLMRECWHAAPSQRPTFKQLVEALDKVLLAVSEEYLDLRLTFGPYSPSGGD  702
FGFR4-214    ..............................................................................................................  108
FGFR4-216    ..............................................................................................................  114
FGFR4-202    LFDRVYTHQSDVWSFGILLWEIFTLGGSPYPGIPVEELFSLLREGHRMDRPPHCPPELYGLMRECWHAAPSQRPTFKQLVEALDKVLLAVSEEYLDLRLTFGPYSPSGGD  730
FGFR4-207    LFDRVYTHQSDVWSFGILLWEIFTLGGSPYPGIPVEELFSLLREGHRMDRPPHCPPELYGLMRECWHAAPSQRPTFKQLVEALDKVLLAVSEEYLDLRLTFGPYSPSGGD  770
FGFR4-201    LFDRVYTHQSDVWSFGILLWEIFTLGGSPYPGIPVEELFSLLREGHRMDRPPHCPPELYGLMRECWHAAPSQRPTFKQLVEALDKVLLAVSEEYLDLRLTFGPYSPSGGD  770
FGFR4-212    ..............................................................................................................   87
FGFR4-213    ..............................................................................................................  280
FGFR4-205    ..............................................................................................................   72
```

```
logo
             ASSTCSSSDSVFSHDPLPLGSSSFPFGSGVQT
DOVITINIB    ................................    7
PF13927      ................................   82
PF07679.1    ................................   36
PF07714.1    ................................  180
PF07679      ................................   76
PF07714      ................................  276
FGFR4-208    ................................  201
FGFR4-203    ASSTCSSSDSVFSHDPLPLGSSSFPFGSGVQT  734
FGFR4-214    ................................  108
FGFR4-216    ................................  114
FGFR4-202    ASSTCSSSDSVFSHDPLPLGSSSFPFGSGVQT  762
FGFR4-207    ASSTCSSSDSVFSHDPLPLGSSSFPFGSGVQT  802
FGFR4-201    ASSTCSSSDSVFSHDPLPLGSSSFPFGSGVQT  802
FGFR4-212    ................................   87
FGFR4-213    ................................  280
FGFR4-205    ................................   72
```

X non conserved
X similar
X $\geq$ 0% conserved
X $\geq$ 50% conserved

```
logo         MRLLLALLGVLLSVPGPPVLSLEASEEVELEPCLAPSLEQQEQELTVALGQPVRLCCGRAERGGHWYKEGSRLAPAGRVRGWRGRLEIASFLPEDAGRYLCLARGSMIVL
PONATINIB    ..............................................................................................................     0
PF13927      ..............................................................................................................     0
PF07679.1    ..............................................................................................................     0
PF07714.1    ..............................................................................................................     0
PF07679      ..............................................................................................................     0
PF07714      ..............................................................................................................     0
FGFR4-208    MRLLLALLGVLLSVPGPPVLSLEASEEVELEPCLAPSLEQQEQELTVALGQPVRLCCGRAERGGHWYKEGSRLAPAGRVRGWRGRLEIASFLPEDAGRYLCLARGSMIVL   110
FGFR4-203    MRLLLALLGVLLSVPGPPVLSLEASEEVELEPCLAPSLEQQEQELTVALGQPVRLCCGRAERGGHWYKEGSRLAPAGRVRGWRGRLEIASFLPEDAGRYLCLARGSMIVL   110
FGFR4-214    MRLLLALLGVLLSVPGPPVLSLEASEEVELEPCLAPSLEQQEQELTVALGQPVRLCCGRAERGGHWYKEGSRLAPAGRVRGWRGRLEIASFLPEDAGRYLCLARGSMI..   108
FGFR4-216    MRLLLALLGVLLSVPGPPVLSLEASEEVELEPCLAPSLEQQEQELTVALGQPVRLCCGRAERGGHWYKEGSRLAPAGRVRGWRGRLEIASFLPEDAGRYLCLARGSMIVL   110
FGFR4-202    MRLLLALLGVLLSVPGPPVLSLEASEEVELEPCLAPSLEQQEQELTVALGQPVRLCCGRAERGGHWYKEGSRLAPAGRVRGWRGRLEIASFLPEDAGRYLCLARGSMIVL   110
FGFR4-207    MRLLLALLGVLLSVPGPPVLSLEASEEVELEPCLAPSLEQQEQELTVALGQPVRLCCGRAERGGHWYKEGSRLAPAGRVRGWRGRLEIASFLPEDAGRYLCLARGSMIVL   110
FGFR4-201    MRLLLALLGVLLSVPGPPVLSLEASEEVELEPCLAPSLEQQEQELTVALGQPVRLCCGRAERGGHWYKEGSRLAPAGRVRGWRGRLEIASFLPEDAGRYLCLARGSMIVL   110
FGFR4-212    MRLLLALLGVLLSVPGPPVLSLEASEEVELEPCLAPSLEQQEQELTVALGQPVRLCCGRAERGGHWYKEGSRLAPAGRVRGWRGRLE.......................    87
FGFR4-213    ..............................................................................................................     0
FGFR4-205    MRLLLALLGVLLSVPGPPVLSLEASEEVELASIHLSLCLRERT..............GLAG...............RRALSWAAELVSPAWLPAWSSKSRS..........    72

logo         QNLTLITGDSLTSSNDDEDPKSHRDPSNRHSYPQQAPYWTHPQRMEKKLHAVPAGNTVKFRCPAAGNPTPTIRWLKDGQAFHGENRIGGIRLRHQHWSLVMESVVPSDRG
PONATINIB    ..............................................................................................................     0
PF13927      ..............................................................................................................     0
PF07679.1    ..................................................AVPAGNTVKFRCPAAGNPTPTIRWLKDGQAFHGENR........................    36
PF07714.1    ..............................................................................................................     0
PF07679      ..................................................AVPAGNTVKFRCPAAGNPTPTIRWLKDGQAFHGENRIGGIRLRHQHWSLVMESVVPSDRG    60
PF07714      ..............................................................................................................     0
FGFR4-208    QNLTLITGDSLTSSNDDEDPKSHRDPSNRHSYPQQAPYWTHPQRMEKKLHAVPAGNTVKFRCPAAGNPTPTIRWLKDGQAFHGENRIGGIR...................   201
FGFR4-203    QNLTLITGDSLTSSNDDEDPKSHRDPSNRHSYPQQAPYWTHPQRMEKKLHAVPAGNTVKFRCPAAGNPTPTIRWLKDGQAFHGENRIGGIRLRHQHWSLVMESVVPSDRG   220
FGFR4-214    ..............................................................................................................   108
FGFR4-216    QNLT..........................................................................................................   114
FGFR4-202    QNLTLITGDSLTSSNDDEDPKSHRDPSNRHSYPQQAPYWTHPQRMEKKLHAVPAGNTVKFRCPAAGNPTPTIRWLKDGQAFHGENRIGGIRLRHQHWSLVMESVVPSDRG   220
FGFR4-207    QNLTLITGDSLTSSNDDEDPKSHRDPSNRHSYPQQAPYWTHPQRMEKKLHAVPAGNTVKFRCPAAGNPTPTIRWLKDGQAFHGENRIGGIRLRHQHWSLVMESVVPSDRG   220
FGFR4-201    QNLTLITGDSLTSSNDDEDPKSHRDPSNRHSYPQQAPYWTHPQRMEKKLHAVPAGNTVKFRCPAAGNPTPTIRWLKDGQAFHGENRIGGIRLRHQHWSLVMESVVPSDRG   220
FGFR4-212    ..............................................................................................................    87
FGFR4-213    ..............................................................................................................     0
FGFR4-205    ..............................................................................................................    72
```

logo

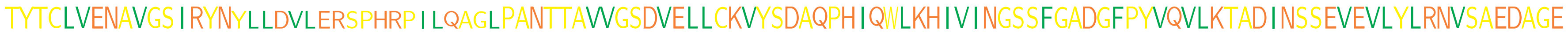

```
PONATINIB   ..............................................................................................................   0
PF13927     ...................................PANTTAVVGSDVELLCKVYSDAQPHIQWLKHIVINGSSFGADGFPYVQVLKTADINSSEVEVLYLRNVSAEDAGE  75
PF07679.1   ..............................................................................................................  36
PF07714.1   ..............................................................................................................   0
PF07679     TYTCLVENAVGSIRYN..............................................................................................  76
PF07714     ..............................................................................................................   0
FGFR4-208   .............................................................................................................. 201
FGFR4-203   TYTCLVENAVGSIRYNYLLDVLERSPHRPILQAGLPANTTAVVGSDVELLCKVYSDAQPHIQWLKHIVINGSSFGADGFPYVQVLKTADINSSEVEVLYLRNVSAEDAGE 330
FGFR4-214   .............................................................................................................. 108
FGFR4-216   .............................................................................................................. 114
FGFR4-202   TYTCLVENAVGSIRYNYLLDVLERSPHRPILQAGLPANTTAVVGSDVELLCKVYSDAQPHIQWLKHIVINGSSFGADGFPYVQVLKTADINSSEVEVLYLRNVSAEDAGE 330
FGFR4-207   TYTCLVENAVGSIRYNYLLDVLERSPHRPILQAGLPANTTAVVGSDVELLCKVYSDAQPHIQWLKHIVINGSSFGADGFPYVQVLKTADINSSEVEVLYLRNVSAEDAGE 330
FGFR4-201   TYTCLVENAVGSIRYNYLLDVLERSPHRPILQAGLPANTTAVVGSDVELLCKVYSDAQPHIQWLKHIVINGSSFGADGFPYVQVLKTADINSSEVEVLYLRNVSAEDAGE 330
FGFR4-212   ..............................................................................................................  87
FGFR4-213   ..............................................................................................................   0
FGFR4-205   ..............................................................................................................  72
```

logo

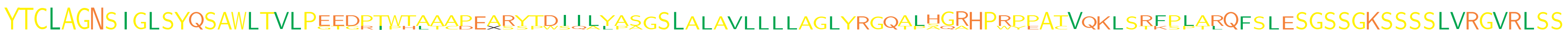

```
PONATINIB   ..............................................................................................................   0
PF13927     YTCLAGN.......................................................................................................  82
PF07679.1   ..............................................................................................................  36
PF07714.1   ..............................................................................................................   0
PF07679     ..............................................................................................................  76
PF07714     ..............................................................................................................   0
FGFR4-208   .............................................................................................................. 201
FGFR4-203   YTCLAGNSIGLSYQSAWLTVLPEEDPTWTAAAPEASSPWSQALPA....................SQAHPWYEACV.................................. 386
FGFR4-214   .............................................................................................................. 108
FGFR4-216   .............................................................................................................. 114
FGFR4-202   YTCLAGNSIGLSYQSAWLTVLPGTGRIPHLTCD.............SL...............TPAGR............TKSPTLQFSLESGSSGKSSSSLVRGVRLSS 400
FGFR4-207   YTCLAGNSIGLSYQSAWLTVLPEEDPTWTAAAPEARYTDIILYASGSLALAVLLLLAGLYRGQALHGRHPRPPATVQKLSRFPLARQFSLESGSSGKSSSSLVRGVRLSS 440
FGFR4-201   YTCLAGNSIGLSYQSAWLTVLPEEDPTWTAAAPEARYTDIILYASGSLALAVLLLLAGLYRGQALHGRHPRPPATVQKLSRFPLARQFSLESGSSGKSSSSLVRGVRLSS 440
FGFR4-212   ..............................................................................................................  87
FGFR4-213   ..................................XRYTDIILYASGSLALAVLLLLAGLYRGQALHGRHPRPPATVQKLSRFPLARQ....SGSSGKSSSSLVRGVRLSS  72
FGFR4-205   ..............................................................................................................  72
```

```
logo
           SGPALLAGLVSLDLPLDPLWEFPRDRLVLGKPLGEGCFGQVVRAEAFGMDPARPDQASTVAVKMLKDNASDKDLADLVSEMEVMKLIGRHKNIINLLGVCTQEGPLYVIV
PONATINIB  ................................L.......V...................AVK................E...M..I.....II...............V   11
PF13927    ..............................................................................................................   82
PF07679.1  ..............................................................................................................   36
PF07714.1  ...........................VLGKPLGEGCFGQVVRAEAFGMDPARPDQASTVAVKMLKDNASDKDLADLVSEMEVMKLIGRHKNIINLLGVCTQEGPLYVIV   83
PF07679    ..............................................................................................................   76
PF07714    ...........................VLGKPLGEGCFGQVVRAEAFGMDPARPDQASTVAVKMLKDNASDKDLADLVSEMEVMKLIGRHKNIINLLGVCTQEGPLYVIV   83
FGFR4-208  ..............................................................................................................  201
FGFR4-203  ............SPPAA..PPCSPASLVLGKPLGEGCFGQVVRAEAFGMDPARPDQASTVAVKMLKDNASDKDLADLVSEMEVMKLIGRHKNIINLLGVCTQEGPLYVIV  482
FGFR4-214  ..............................................................................................................  108
FGFR4-216  ..............................................................................................................  114
FGFR4-202  SGPALLAGLVSLDLPLDPLWEFPRDRLVLGKPLGEGCFGQVVRAEAFGMDPARPDQASTVAVKMLKDNASDKDLADLVSEMEVMKLIGRHKNIINLLGVCTQEGPLYVIV  510
FGFR4-207  SGPALLAGLVSLDLPLDPLWEFPRDRLVLGKPLGEGCFGQVVRAEAFGMDPARPDQASTVAVKMLKDNASDKDLADLVSEMEVMKLIGRHKNIINLLGVCTQEGPLYVIV  550
FGFR4-201  SGPALLAGLVSLDLPLDPLWEFPRDRLVLGKPLGEGCFGQVVRAEAFGMDPARPDQASTVAVKMLKDNASDKDLADLVSEMEVMKLIGRHKNIINLLGVCTQEGPLYVIV  550
FGFR4-212  ..............................................................................................................   87
FGFR4-213  SGPALLAGLVSLDLPLDPLWEFPRDRLVLGKPLGEGCFGQVVRAEAFGMDPARPDQASTVAVKMLKDNASDKDLADLVSEMEVMKLIGRHKNIINLLGVCTQEGPLYVIV  182
FGFR4-205  ..............................................................................................................   72

logo
           ECAAKGNLREFLRARRPPGPDLSPDGPRSSEGPLSFPVLVSCAYQVARGMQYLESRKCIHRDLAARNVLVTEDNVMKIADFGLARGVHHIDYYKKTSNGRLPVKWMAPEA
PONATINIB  ECA.................................................L....CIHR.......L........IADFGL...........................   26
PF13927    ..............................................................................................................   82
PF07679.1  ..............................................................................................................   36
PF07714.1  ECAAKGNLREFLRARRPPGPDLSPDGPRSSEGPLSFPVLVSCAYQVARGMQYLESRKCIHRDLAARNVLVTEDNVMKIADFGLARGVHHIDYYKKTS.............  180
PF07679    ..............................................................................................................   76
PF07714    ECAAKGNLREFLRARRPPGPDLSPDGPRSSEGPLSFPVLVSCAYQVARGMQYLESRKCIHRDLAARNVLVTEDNVMKIADFGLARGVHHIDYYKKTSNGRLPVKWMAPEA  193
FGFR4-208  ..............................................................................................................  201
FGFR4-203  ECAAKGNLREFLRARRPPGPDLSPDGPRSSEGPLSFPVLVSCAYQVARGMQYLESRKCIHRDLAARNVLVTEDNVMKIADFGLARGVHHIDYYKKTSNGRLPVKWMAPEA  592
FGFR4-214  ..............................................................................................................  108
FGFR4-216  ..............................................................................................................  114
FGFR4-202  ECAAKGNLREFLRARRPPGPDLSPDGPRSSEGPLSFPVLVSCAYQVARGMQYLESRKCIHRDLAARNVLVTEDNVMKIADFGLARGVHHIDYYKKTSNGRLPVKWMAPEA  620
FGFR4-207  ECAAKGNLREFLRARRPPGPDLSPDGPRSSEGPLSFPVLVSCAYQVARGMQYLESRKCIHRDLAARNVLVTEDNVMKIADFGLARGVHHIDYYKKTSNGRLPVKWMAPEA  660
FGFR4-201  ECAAKGNLREFLRARRPPGPDLSPDGPRSSEGPLSFPVLVSCAYQVARGMQYLESRKCIHRDLAARNVLVTEDNVMKIADFGLARGVHHIDYYKKTSNGRLPVKWMAPEA  660
FGFR4-212  ..............................................................................................................   87
FGFR4-213  ECAAKGNLREFLRARRPPGPDLSPDGPRSSEGPLSFPVLVSCAYQVARGMQYLESRKCIHRDLAARNVLVTEDNVMKIADFGLARGVHHIDYYKKTSN............  280
FGFR4-205  ..............................................................................................................   72
```

```
logo
             LFDRVYTHQSDVWSFGILLWEIFTLGGSPYPGIPVEELFSLLREGHRMDRPPHCPPELYGLMRECWHAAPSQRPTFKQLVEALDKVLLAVSEEYLDLRLTFGPYSPSGGD
PONATINIB    ..............................................................................................................   26
PF13927      ..............................................................................................................   82
PF07679.1    ..............................................................................................................   36
PF07714.1    ..............................................................................................................  180
PF07679      ..............................................................................................................   76
PF07714      LFDRVYTHQSDVWSFGILLWEIFTLGGSPYPGIPVEELFSLLREGHRMDRPPHCPPELYGLMRECWHAAPSQRPTFKQLVEAL...........................  276
FGFR4-208    ..............................................................................................................  201
FGFR4-203    LFDRVYTHQSDVWSFGILLWEIFTLGGSPYPGIPVEELFSLLREGHRMDRPPHCPPELYGLMRECWHAAPSQRPTFKQLVEALDKVLLAVSEEYLDLRLTFGPYSPSGGD  702
FGFR4-214    ..............................................................................................................  108
FGFR4-216    ..............................................................................................................  114
FGFR4-202    LFDRVYTHQSDVWSFGILLWEIFTLGGSPYPGIPVEELFSLLREGHRMDRPPHCPPELYGLMRECWHAAPSQRPTFKQLVEALDKVLLAVSEEYLDLRLTFGPYSPSGGD  730
FGFR4-207    LFDRVYTHQSDVWSFGILLWEIFTLGGSPYPGIPVEELFSLLREGHRMDRPPHCPPELYGLMRECWHAAPSQRPTFKQLVEALDKVLLAVSEEYLDLRLTFGPYSPSGGD  770
FGFR4-201    LFDRVYTHQSDVWSFGILLWEIFTLGGSPYPGIPVEELFSLLREGHRMDRPPHCPPELYGLMRECWHAAPSQRPTFKQLVEALDKVLLAVSEEYLDLRLTFGPYSPSGGD  770
FGFR4-212    ..............................................................................................................   87
FGFR4-213    ..............................................................................................................  280
FGFR4-205    ..............................................................................................................   72
```

```
logo
             ASSTCSSSDSVFSHDPLPLGSSSFPFGSGVQT
PONATINIB    ................................   26
PF13927      ................................   82
PF07679.1    ................................   36
PF07714.1    ................................  180
PF07679      ................................   76
PF07714      ................................  276
FGFR4-208    ................................  201
FGFR4-203    ASSTCSSSDSVFSHDPLPLGSSSFPFGSGVQT  734
FGFR4-214    ................................  108
FGFR4-216    ................................  114
FGFR4-202    ASSTCSSSDSVFSHDPLPLGSSSFPFGSGVQT  762
FGFR4-207    ASSTCSSSDSVFSHDPLPLGSSSFPFGSGVQT  802
FGFR4-201    ASSTCSSSDSVFSHDPLPLGSSSFPFGSGVQT  802
FGFR4-212    ................................   87
FGFR4-213    ................................  280
FGFR4-205    ................................   72
```

X non conserved
X similar
X $\geq$ 0% conserved
X $\geq$ 50% conserved

logo

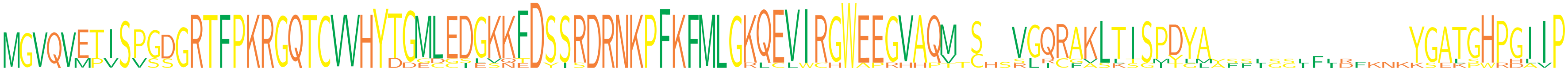

```
SIROLIMUS    ..........................Y.........FD........F......QEVI..W......................Y.....................H.....   11
PF00254.2    .............RTFPKRGQTCVVHYTGMLEDGKKFDSSRDRNKPFKFMLGKQEVIRGWEEGVAQV.C.........................................   55
PF00254      .............RTFPKRGQTCVVHYTGMLEDGKKFDSSRDRNKPFKFMLGKQEVIRGWEEGVAQM.S..VGQRAKLTISPDYA..............YGATGHPGIIP   80
PF00254.1    ...........................DGMLEDGKKFDSSRDRNKPFKFMLGKQEVIRGWEEGVAQM.S..VGQRAKLTISPDYA..............YGATGHPGIIP   66
FKBP1A-210   MGVQVETISPGDGRTFPKRGQTCVVHYTGMLEDGKKFDSSRDRNKPFKFMLGKQEVIRGWEEGVAQV.C..SLICFVLLSSIYLLYFFIGGIFIRFKNKKSEKSHPHAIP  107
FKBP1A-202   MGVQVETISPGDGRTFPKRGQTCVVHYTGMLEDGKKFDSSRDRNKPFKFMLGKQEVIRGWEEGVAQM.S..VGQRAKLTISPDYA..............YGATGHPGIIP   93
FKBP1A-204   MGVQVETISPGDGRTFPKRGQTCVVHYTGMLEDGKKFDSSRDRNKPFKFMLGKQEVIRGWEEGVAQM.S..VGQRAKLTISPDYA..............YGATGHPGIIP   93
FKBP1A-205   MGVQVETISPG................DGMLEDGKKFDSSRDRNKPFKFMLGKQEVIRGWEEGVAQM.S..VGQRAKLTISPDYA..............YGATGHPGIIP   77
FKBP1A-201   .....MPVSVSSGRTFPKRGQTCVVHYTGMLEDGKKFDSSRDRNKPFKFMLGKQEVIRGWEEGVAQM.S..VGQRAKLTISPDYA..............YGATGHPGIIP   88
FKBP1A-209   MGVQVETISPGDGRTFPKRGQTCVVHYTGGDCILVNE.........................................................................   37
FKBP1A-203   .....MPVSVSSGRTFPKRGQTCVVHYTGMLEDGKKFDSSRDRNKPFKFMLGKQEVIRGWEEGVAQM.S..VGQRAKLTISPDYA..............YGATGHPGIIP   88
FKBP1A-211   MGVQVETISPGDGRTFPKRGQTCVVHYTDECGSESQTDYIS...........RLCLWCHWAPRHHPTTCHSRLRCGASKTGMTGMASSLSSLFLD.......LPWRDLVP   92
```

logo

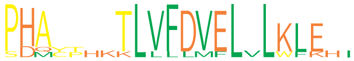

```
SIROLIMUS    ..........F...........    12
PF00254.2    ......................    55
PF00254      PHA....TLVFDVEL.L.....    92
PF00254.1    PHA....TLVFDVEL.L.....    78
FKBP1A-210   SDQYPHKKLLLLMFLVLWFKHI   129
FKBP1A-202   PHA....TLVFDVEL.LKLE..   108
FKBP1A-204   PHA....TLVFDVEL.LKLE..   108
FKBP1A-205   PHA....TLVFDVEL.LKLE..    92
FKBP1A-201   PHA....TLVFDVEL.LKLE..   103
FKBP1A-209   ......................    37
FKBP1A-203   PHA....TLVFDVEL.LKLE..   103
FKBP1A-211   PDMCT.................    97
```

X non conserved
X similar
X $\geq$ 0% conserved
X $\geq$ 50% conserved

```
logo
```
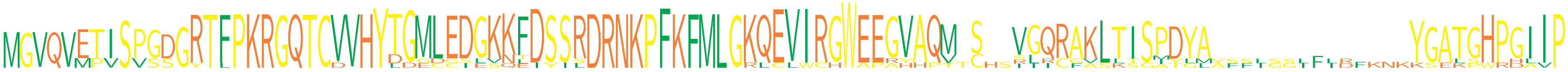

```
TACROLIMUS   .............Y.L.......D..YL.......NLI..L.....F.......EVI..W..AY.......II........A................Y....H...I.   22
PF00254.2    .............RTFPKRGQTCVVHYTGMLEDGKKFDSSRDRNKPFKFMLGKQEVIRGWEEGVAQV.C........................................   55
PF00254      .............RTFPKRGQTCVVHYTGMLEDGKKFDSSRDRNKPFKFMLGKQEVIRGWEEGVAQM.S..VGQRAKLTISPDYA.............YGATGHPGIIP   80
PF00254.1    ...........................DGMLEDGKKFDSSRDRNKPFKFMLGKQEVIRGWEEGVAQM.S..VGQRAKLTISPDYA.............YGATGHPGIIP   66
FKBP1A-210   MGVQVETISPGDGRTFPKRGQTCVVHYTGMLEDGKKFDSSRDRNKPFKFMLGKQEVIRGWEEGVAQV.C..SLICFVLLSSIYLLYFFIGGIFIRFKNKKSEKSHPHAIP  107
FKBP1A-202   MGVQVETISPGDGRTFPKRGQTCVVHYTGMLEDGKKFDSSRDRNKPFKFMLGKQEVIRGWEEGVAQM.S..VGQRAKLTISPDYA.............YGATGHPGIIP   93
FKBP1A-204   MGVQVETISPGDGRTFPKRGQTCVVHYTGMLEDGKKFDSSRDRNKPFKFMLGKQEVIRGWEEGVAQM.S..VGQRAKLTISPDYA.............YGATGHPGIIP   93
FKBP1A-205   MGVQVETISPG................DGMLEDGKKFDSSRDRNKPFKFMLGKQEVIRGWEEGVAQM.S..VGQRAKLTISPDYA.............YGATGHPGIIP   77
FKBP1A-201   .....MPVSVSSGRTFPKRGQTCVVHYTGMLEDGKKFDSSRDRNKPFKFMLGKQEVIRGWEEGVAQM.S..VGQRAKLTISPDYA.............YGATGHPGIIP   88
FKBP1A-209   MGVQVETISPGDGRTFPKRGQTCVVHYTGGDCILVNE........................................................................   37
FKBP1A-203   .....MPVSVSSGRTFPKRGQTCVVHYTGMLEDGKKFDSSRDRNKPFKFMLGKQEVIRGWEEGVAQM.S..VGQRAKLTISPDYA.............YGATGHPGIIP   88
FKBP1A-211   MGVQVETISPGDGRTFPKRGQTCVVHYTDECGSESQTDYIS...........RLCLWCHWAPRHHPTTCHSRLRCGASKTGMTGMASSLSLFLD.......LPWRDLVP   92
```

```
logo
```
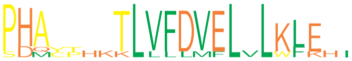

```
TACROLIMUS   ..................F...     23
PF00254.2    ......................     55
PF00254      PHA....TLVFDVEL.L.....     92
PF00254.1    PHA....TLVFDVEL.L.....     78
FKBP1A-210   SDQYPHKKLLLLMFLVLWFKHI    129
FKBP1A-202   PHA....TLVFDVEL.LKLE..    108
FKBP1A-204   PHA....TLVFDVEL.LKLE..    108
FKBP1A-205   PHA....TLVFDVEL.LKLE..     92
FKBP1A-201   PHA....TLVFDVEL.LKLE..    103
FKBP1A-209   ......................     37
FKBP1A-203   PHA....TLVFDVEL.LKLE..    103
FKBP1A-211   PDMCT.................     97
```

X non conserved
X similar
X $\geq$ 0% conserved
X $\geq$ 50% conserved

```
logo
SIROLIMUS   ........................................................Y.........FD........F......GEVI..W....................   9
PF00254.1   ................................................IGDRVFVHYTGWLLDGTKFDSSLDRKDKFSFDLGKGEVIKAWDIAIATMKVGEVCHITCKPE  62
PF00254     ...........................................TEMPMIGDRVFVHYTGWLLDGTKFDSSLDRKDKFSFDLGKGEVIKAWDIAIATMKVGEVCHITCKPE  67
PF07719     ..............................................................................................................   0
PF00515     ..............................................................................................................   0
FKBP4-203   ..............................................................................................................   0
FKBP4-202   .............................................MPMIGDRVFVHYTGWLLDGTKFDSSLDRKDKFSFDLGKGEVIKAWDIAIATMKVGEVCHITCKPE  65
FKBP4-208   MTAEEMKATESGAQSAPLPMEGVDISPKQDEGVLKIIGDLKRLLQRLSCGM...........................................................  51
FKBP4-207   ..............................................................................................................   0
FKBP4-206   MTAEEMKATESGAQSAPLPMEGVDISPKQDEGVLKIIGDLKRLLQRLSCGM...........................................................  51
FKBP4-201   MTAEEMKATESGAQSAPLPMEGVDISPKQDEGVLKVIKREGTGTEMPMIGDRVFVHYTGWLLDGTKFDSSLDRKDKFSFDLGKGEVIKAWDIAIATMKVGEVCHITCKPE 110

logo
SIROLIMUS   ..Y.......KI........F.........................................................................................  13
PF00254.1   YAYGSAGSPPKIPPNATLVFE.........................................................................................  83
PF00254     YAYGSAGSPPKIPPNATLVFEVEL...........................AKPN....EG.AI.VEV.ALE.GYYK.DKLF.DQR.ELRF.EIGEG.ENLD.LPYG.LE 135
PF07719     ..............................................................................................................   0
PF00515     ..............................................................................................................   0
FKBP4-203   ..............................................................................................................   0
FKBP4-202   YAYGSAGSPPKIPPNATLVFE.........................................................................................  86
FKBP4-208   ..............................................................................................................  51
FKBP4-207   ..............................................................................................................   0
FKBP4-206   ..............................................................................................................  51
FKBP4-201   YAYGSAGSPPKIPPNATLVFEVELFEFKGEDLTEEEDGGIIRRIQTRGEGYAKPN....EG.AI.VEV.ALE.GYYK.DKLF.DQR.ELRF.EIGEG.ENLD.LPYG.LE 205

logo
SIROLIMUS   ..............................................................................................................  13
PF00254.1   ..............................................................................................................  83
PF00254     RA.IQRM.EKGE.HSIVY.LKPS.YAF.GSVG.KEKF.QIPP.NAEL.KYEL.HL....................................................... 179
PF07719     ..............................................................................................................   0
PF00515     ..............................................................................................................   0
FKBP4-203   ..............................................................................................................   0
FKBP4-202   ..............................................................................................................  86
FKBP4-208   ..............................................................................................................  51
FKBP4-207   ..............................................................................................................   0
FKBP4-206   ..............................................................................................................  51
FKBP4-201   RA.IQRM.EKGE.HSIVY.LKPS.YAF.GSVG.KEKF.QIPP.NAEL.KYEL.HLKS.FEKA.KESW.EMNS.EEKL.EQST.IVKE....................... 275
```

logo

SIROLIMUS 13
PF00254.1 83
PF00254 179
PF07719 0
PF00515 0
FKBP4-203 0
FKBP4-202 86
FKBP4-208 51
FKBP4-207 0
FKBP4-206 51
FKBP4-201 275

logo

SIROLIMUS 13
PF00254.1 83
PF00254 179
PF07719 0
PF00515 0
FKBP4-203 0
FKBP4-202 86
FKBP4-208 51
FKBP4-207 0
FKBP4-206 51
FKBP4-201 275

logo

SIROLIMUS 13
PF00254.1 83
PF00254 179
PF07719 0
PF00515 0
FKBP4-203 0
FKBP4-202 86
FKBP4-208 51
FKBP4-207 0
FKBP4-206 51
FKBP4-201 275

logo

SIROLIMUS 13
PF00254.1 83
PF00254 179
PF07719 0
PF00515 0
FKBP4-203 0
FKBP4-202 86
FKBP4-208 51
FKBP4-207 0
FKBP4-206 51
FKBP4-201 275

logo

SIROLIMUS 13
PF00254.1 83
PF00254 179
PF07719 0
PF00515 0
FKBP4-203 0
FKBP4-202 86
FKBP4-208 51
FKBP4-207 0
FKBP4-206 51
FKBP4-201 275

logo

SIROLIMUS 13
PF00254.1 83
PF00254 179
PF07719 0
PF00515 0
FKBP4-203 0
FKBP4-202 86
FKBP4-208 51
FKBP4-207 0
FKBP4-206 51
FKBP4-201 275

```
logo

SIROLIMUS  ..............................................................................................................   13
PF00254.1  ..............................................................................................................   83
PF00254    ..............................................................................................................  179
PF07719    ..............................................................................................................    0
PF00515    ..............................................................................................................    0
FKBP4-203  ..............................................................................................................    0
FKBP4-202  ..............................................................................................................   86
FKBP4-208  ..............................................................................................................   51
FKBP4-207  ..............................................................................................................    0
FKBP4-206  ..............................................................................................................   51
FKBP4-201  ..............................................................................................................  275
```

```
logo
                                                                                                                        R
SIROLIMUS  ..............................................................................................................   13
PF00254.1  ..............................................................................................................   83
PF00254    ..............................................................................................................  179
PF07719    ..............................................................................................................    0
PF00515    ..............................................................................................................    0
FKBP4-203  ..............................................................................................................    0
FKBP4-202  ..............................................................................................................   86
FKBP4-208  ..............................................................................................................   51
FKBP4-207  ..............................................................................................................    0
FKBP4-206  ..............................................................................................................   51
FKBP4-201  .............................................................................................................R  276
```

```
logo
                      GT            VYFKEG  KYKQALLQYKKIVSWLEYESS                                FSNEEAQKAQALRLASHLN
SIROLIMUS  ..............................................................................................................   13
PF00254.1  ..............................................................................................................   83
PF00254    ..............................................................................................................  179
PF07719    ..............................................................................................................    0
PF00515    .....................................................................................................SHLN.....    4
FKBP4-203  ..............................................................................................................    0
FKBP4-202  ..............................................................................................................   86
FKBP4-208  ..............................................................................................................   51
FKBP4-207  ..............................................................................................................    0
FKBP4-206  ..............................................................................................................   51
FKBP4-201  ...........GT............VYFKEG..KYKQALLQYKKIVSWLEYESS................................FSNEEAQKAQALRLASHLN.....  324
```

```
logo
                          L                  AMCHLKLQAFSAAIESCNKALELDSNN                    EKGLFRRGEA
SIROLIMUS   ..............................................................................................................   13
PF00254.1   ..............................................................................................................   83
PF00254     ..............................................................................................................  179
PF07719     ................................................................................KGLFRRGEA.....................    9
PF00515     .............L..................AMCHLKLQAFSAAIESCNKALELDSNN...................................................   32
FKBP4-203   ..............................................................................................................    0
FKBP4-202   ..............................................................................................................   86
FKBP4-208   ..............................................................................................................   51
FKBP4-207   ..............................................................................................................    0
FKBP4-206   ..............................................................................................................   51
FKBP4-201   .............L..................AMCHLKLQAFSAAIESCNKALELDSNN....................EKGLFRRGEA.....................  362

logo
             HLAVND                FELA RA  DFQKVLQLYPNNKAAKTQLAVCQQRIR                 R
SIROLIMUS   ..............................................................................................................   13
PF00254.1   ..............................................................................................................   83
PF00254     ..............................................................................................................  179
PF07719     .HLAVND................FELA.RA..DFQKVLQLYPNN..................................................................   33
PF00515     ..............................................................................................................   32
FKBP4-203   ...XVND................FELA.RA..DFQKVLQLYPNNKAAKTQLAVCQQRIR.................R.................................   38
FKBP4-202   ..............................................................................................................   86
FKBP4-208   ..............................................................................................................   51
FKBP4-207   ..............................................................................................................    0
FKBP4-206   ..............................................................................................................   51
FKBP4-201   .HLAVND................FELA.RA..DFQKVLQLYPNNKAAKTQLAVCQQRIR.................R.................................  402

logo
                Q             LARE           KKLYANMFERLA     EEE                      NKA                 PWL   T   S
SIROLIMUS   ..............................................................................................................   13
PF00254.1   ..............................................................................................................   83
PF00254     ..............................................................................................................  179
PF07719     ..............................................................................................................   33
PF00515     ..............................................................................................................   32
FKBP4-203   ....Q.............LARE...........KKLYANMFERLA.....EEE......................NKA.................PWL...T...P....   66
FKBP4-202   ..............................................................................................................   86
FKBP4-208   ..............................................................................................................   51
FKBP4-207   ..............................................................................................................    0
FKBP4-206   ..............................................................................................................   51
FKBP4-201   ....Q.............LARE...........KKLYANMFERLA.....EEE......................NKA.................KAE...A...S....  430
```

```
logo

SIROLIMUS   ..............................................................................................................   13
PF00254.1   ..............................................................................................................   83
PF00254     ..............................................................................................................  179
PF07719     ..............................................................................................................   33
PF00515     ..............................................................................................................   32
FKBP4-203   A.....................IP..A..L..CRA................................K..........................................   75
FKBP4-202   ..............................................................................................................   86
FKBP4-208   ..............................................................................................................   51
FKBP4-207   ..............................................................................................................    0
FKBP4-206   ..............................................................................................................   51
FKBP4-201   S.....................GD..H..P..TDT................................E..........................................  439


logo

SIROLIMUS   ..............................................................................................................   13
PF00254.1   ..............................................................................................................   83
PF00254     ..............................................................................................................  179
PF07719     ..............................................................................................................   33
PF00515     ..............................................................................................................   32
FKBP4-203   ..............................................................................................................   75
FKBP4-202   ..............................................................................................................   86
FKBP4-208   ..............................................................................................................   51
FKBP4-207   ..............................................................................................................    0
FKBP4-206   ..............................................................................................................   51
FKBP4-201   ..............................................................................................................  439


logo

SIROLIMUS   ..............................................................................................................   13
PF00254.1   ..............................................................................................................   83
PF00254     ..............................................................................................................  179
PF07719     ..............................................................................................................   33
PF00515     ..............................................................................................................   32
FKBP4-203   ..............................................................................................................   75
FKBP4-202   ..............................................................................................................   86
FKBP4-208   ..............................................................................................................   51
FKBP4-207   ..............................................................................................................    0
FKBP4-206   ..............................................................................................................   51
FKBP4-201   ..............................................................................................................  439
```

```
logo

SIROLIMUS    ..................................................LE..S...F....................................................  17
PF00254.1    ...............................................................................................................  83
PF00254      ............................................................................................................... 179
PF07719      ...............................................................................................................  33
PF00515      ...............................................................................................................  32
FKBP4-203    ..............................V........GGSLEPSR................................................................  84
FKBP4-202    ...............................................................................................................  86
FKBP4-208    ...............................................................................................................  51
FKBP4-207    ..............................XKEEQKSNTAGSQSQIRKPRLRS..........................................................  23
FKBP4-206    ...............................................................................................................  51
FKBP4-201    ..............................MKEEQKSNTAGSQSQVETEA............................................................. 459


logo

SIROLIMUS    .......T..WD..Y..F   22
PF00254.1    ..................   83
PF00254      ..................  179
PF07719      ..................   33
PF00515      ..................   32
FKBP4-203    ..................   84
FKBP4-202    ..................   86
FKBP4-208    ..................   51
FKBP4-207    ..................   23
FKBP4-206    ..................   51
FKBP4-201    ..................  459
```

X non conserved

X similar

X ≥ 0% conserved

X ≥ 50% conserved

```
logo         MPALARDGGQLPLLVVFSAMIFGTITNQDLPVIKCVLINHKNNDSSVGKSSSYPMVSESPEDLGCALRPQSSGTVYEAAAVEVDVSASITLQVLVDAPGNISCLWVFKHS
QUIZARTINIB  ..............................................................................................................   0
PF07714      ..............................................................................................................   0
PF00047      ..............................................................................................................   0
FLT3-201     MPALARDGGQLPLLVVFSAMIFGTITNQDLPVIKCVLINHKNNDSSVGKSSSYPMVSESPEDLGCALRPQSSGTVYEAAAVEVDVSASITLQVLVDAPGNISCLWVFKHS 110
FLT3-202     MPALARDGGQLPLLVVFSAMIFGTITNQDLPVIKCVLINHKNNDSSVGKSSSYPMVSESPEDLGCALRPQSSGTVYEAAAVEVDVSASITLQVLVDAPGNISCLWVFKHS 110

logo         SLNCQPHFDLQNRGVVSMVILKMTETQAGEYLLFIQSEATNYTILFTVSIRNTLLYTLRRPYFRKMENQDALVCISESVPEPIVEWVLCDSQGESCKEESPAVVKKEEKV
QUIZARTINIB  ..............................................................................................................   0
PF07714      ..............................................................................................................   0
PF00047      ..............................................................................................................   0
FLT3-201     SLNCQPHFDLQNRGVVSMVILKMTETQAGEYLLFIQSEATNYTILFTVSIRNTLLYTLRRPYFRKMENQDALVCISESVPEPIVEWVLCDSQGESCKEESPAVVKKEEKV 220
FLT3-202     SLNCQPHFDLQNRGVVSMVILKMTETQAGEYLLFIQSEATNYTILFTVSIRNTLLYTLRRPYFRKMENQDALVCISESVPEPIVEWVLCDSQGESCKEESPAVVKKEEKV 220

logo         LHELFGTDIRCCARNELGRECTRLFTIDLNQTPQTTLPQLFLKVGEPLWIRCKAVHVNHGFGLTWELENKALEEGNYFEMSTYSTNRTMIRILFAFVSSVARNDTGYYTC
QUIZARTINIB  ..............................................................................................................   0
PF07714      ..............................................................................................................   0
PF00047      ....................................LPQLFLKVGEPLWIRCKAVHVNHGFGLTWELENKALEEGNYFEMSTYSTNRTMIRILFAFVSSVARNDTGYYTC  74
FLT3-201     LHELFGTDIRCCARNELGRECTRLFTIDLNQTPQTTLPQLFLKVGEPLWIRCKAVHVNHGFGLTWELENKALEEGNYFEMSTYSTNRTMIRILFAFVSSVARNDTGYYTC 330
FLT3-202     LHELFGTDIRCCARNELGRECTRLFTIDLNQTPQTTLPQLFLKVGEPLWIRCKAVHVNHGFGLTWELENKALEEGNYFEMSTYSTNRTMIRILFAFVSSVARNDTGYYTC 330

logo         SSSKHPSQSALVTIVEKGFINATNSSEDYEIDQYEEFCFSVRFKAYPQIRCTWTFSRKSFPCEQKGLDNGYSISKFCNHKHQPGEYIFHAENDDAQFTKMFTLNIRRKPQ
QUIZARTINIB  ..............................................................................................................   0
PF07714      ..............................................................................................................   0
PF00047      SSSKHPSQSALVTI................................................................................................  88
FLT3-201     SSSKHPSQSALVTIVEKGFINATNSSEDYEIDQYEEFCFSVRFKAYPQIRCTWTFSRKSFPCEQKGLDNGYSISKFCNHKHQPGEYIFHAENDDAQFTKMFTLNIRRKPQ 440
FLT3-202     SSSKHPSQSALVTIVEKGFINATNSSEDYEIDQYEEFCFSVRFKAYPQIRCTWTFSRKSFPCEQKGLDNGYSISKFCNHKHQPGEYIFHAENDDAQFTKMFTLNIRRKPQ 440

logo         VLAEASASQASCFSDGYPLPSWTWKKCSDKSPNCTEEITEGVWNRKANRKVFGQWVSSSTLNMSEAIKGFLVKCCAYNSLGTSCETILLNSPGPFPFIQDNISFYATIGV
QUIZARTINIB  ..............................................................................................................   0
PF07714      ..............................................................................................................   0
PF00047      ..............................................................................................................  88
FLT3-201     VLAEASASQASCFSDGYPLPSWTWKKCSDKSPNCTEEITEGVWNRKANRKVFGQWVSSSTLNMSEAIKGFLVKCCAYNSLGTSCETILLNSPGPFPFIQDNISFYATIGV 550
FLT3-202     VLAEASASQASCFSDGYPLPSWTWKKCSDKSPNCTEEITEGVWNRKANRKVFGQWVSSSTLNMSEAIKGFLVKCCAYNSLGTSCETILLNSPGPFPFIQDNISFYATIGV 550

logo         CLLFIVVLTLLICHKYKKQFRYESQLQMVQVTGSSDNEYFYVDFREYEYDLKWEFPRENLEFGKVLGSGAFGKVMNATAYGISKTGVSIQVAVKMLKEKADSSEREALMS
QUIZARTINIB  .................................................................L........V................A.K................   4
PF07714      ...........................................................LEFGKVLGSGAFGKVMNATAYGISKTGVSIQVAVKMLKEKADSSEREALMS  51
PF00047      ..............................................................................................................  88
FLT3-201     CLLFIVVLTLLICHKYKKQFRYESQLQMVQVTGSSDNEYFYVDFREYEYDLKWEFPRENLEFGKVLGSGAFGKVMNATAYGISKTGVSIQVAVKMLKEKADSSEREALMS 660
FLT3-202     CLLFIVVLTLLICHKYKKQFRYESQLQMVQVTGSSDNEYFYVDFREYEYDLKWEFPRENLEFGKVLGSGAFGKVMNATAYGISKTGVSIQVAVKMLKEKADSSEREALMS 660
```

```
logo         ELKMMTQLGSHENIVNLLGACTLSGPIYLIFEYCCYGDLLNYLRSKREKFHRTWTEIFKEHNFSFYPTFQSHPNSSMPGSREVQIHPDSDQISGLHGNSFHSEDEIEYEN
                                                                                          KKK
QUIZARTINIB  E..MM..L.....IV................F.YCCYG.........................................................................   16
PF07714      ELKMMTQLGSHENIVNLLGACTLSGPIYLIFEYCCYGDLLNYLRSKREKFHRTWTEIFKEHNFSFYPTFQSHPNSSMPGSREVQIHPDSDQISGLHGNSFHSEDEIEYEN  161
PF00047      ...............................................................................................................   88
FLT3-201     ELKMMTQLGSHENIVNLLGACTLSGPIYLIFEYCCYGDLLNYLRSKREKFHRTWTEIFKEHNFSFYPTFQSHPNSSMPGSREVQIHPDSDQISGLHGNSFHSEDEIEYEN  770
FLT3-202     ELKMMTQLGSHENIVNLLGACTLSGPIYLIFEYCCYGDLLNYLRSKREKFHRTWTEIFKEHNFSFYPTFQSHPNSSKKK................................  739

logo         QKRLEEEEDLNVLTFEDLLCFAYQVAKGMEFLEFKSCVHRDLAARNVLVTHGKVVKICDFGLARDIMSDSNYVVRGNARLPVKWMAPESLFEGIYTIKSDVWSYGILLWE
QUIZARTINIB  ......................................H........L........ICDF.................................................   22
PF07714      QKRLEEEEDLNVLTFEDLLCFAYQVAKGMEFLEFKSCVHRDLAARNVLVTHGKVVKICDFGLARDIMSDSNYVVRGNARLPVKWMAPESLFEGIYTIKSDVWSYGILLWE  271
PF00047      ...............................................................................................................   88
FLT3-201     QKRLEEEEDLNVLTFEDLLCFAYQVAKGMEFLEFKSCVHRDLAARNVLVTHGKVVKICDFGLARDIMSDSNYVVRGNARLPVKWMAPESLFEGIYTIKSDVWSYGILLWE  880
FLT3-202     ...............................................................................................................  739

logo         IFSLGVNPYPGIPVDANFYKLIQNGFKMDQPFYATEEIYIIMQSCWAFDSRKRPSFPNLTSFLGCQLADAEEAMYQNVDGRVSECPHTYQNRRPFSREMDLGLLSPQAQV
QUIZARTINIB  ...............................................................................................................   22
PF07714      IFSLGVNPYPGIPVDANFYKLIQNGFKMDQPFYATEEIYIIMQSCWAFDSRKRPSFPNLTSFL................................................  334
PF00047      ...............................................................................................................   88
FLT3-201     IFSLGVNPYPGIPVDANFYKLIQNGFKMDQPFYATEEIYIIMQSCWAFDSRKRPSFPNLTSFLGCQLADAEEAMYQNVDGRVSECPHTYQNRRPFSREMDLGLLSPQAQV  990
FLT3-202     ...............................................................................................................  739

logo         EDS
QUIZARTINIB  ...    22
PF07714      ...   334
PF00047      ...    88
FLT3-201     EDS   993
FLT3-202     ...   739
```

X non conserved
X similar
X ≥ 0% conserved
X ≥ 50% conserved

logo

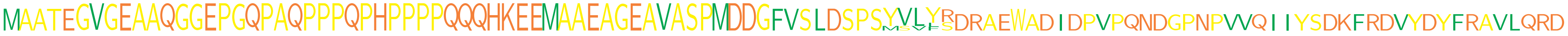

```
TIPIFARNIB   .................................................................L.....W......................................   2
PF01239.3    ..............................................................................................................   0
PF01239.2    ..............................................................................................................   0
PF01239      ..............................................................................................................   0
PF01239.1    ..............................................................................................................   0
FNTA-204     ..............................................................................................................   0
FNTA-214     MAATEGVGEAAQGGEPGQPAQPPPQPHPPPPQQQHKEEMAAEAGEAVASPMDDGFVSLDSPSYVLYR...........................................  67
FNTA-210     ..............................................................MSVFRDRAEWADIDPVPQNDGPNPVVQIIYSDKFRDVYDYFRAVLQRD  48
FNTA-201     MAATEGVGEAAQGGEPGQPAQPPPQPHPPPPQQQHKEEMAAEAGEAVASPMDDGFVSLDSPSYVLYRDRAEWADIDPVPQNDGPNPVVQIIYSDKFRDVYDYFRAVLQRD 110
FNTA-209     .....GVGEAAQGGEPGQPAQPPPQPHPPPPQQQHKEEMAAEAGEAVASPMDDG.............DRAEWADIDPVPQNDGPNPVVQIIYSDKFRDVYDYFRAVLQRD  92
FNTA-208     ..............................................................................................................   0
FNTA-205     MAATEGVGEAAQGGEPGQPAQPPPQPHPPPPQQQHKEEMAAEAGEAVASPMDDGFVSLDSPSYVLYS...........................................  67
FNTA-212     MAATEGVGEAAQGGEPGQPAQPPPQPHPPPPQQQHKEEMAAEAGEAVASPMDDGFVSLDSPSYVLYS...........................................  67
```

logo

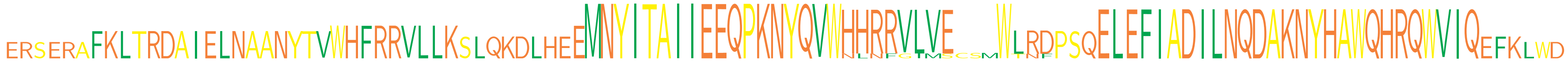

```
TIPIFARNIB   .......................W......................................................................................   3
PF01239.3    .........................................MNYITAIIEEQPKNYQVWHHRRVLVE...W......ELEFIADILNQDAKNYHAWQHRQWVIQ......  54
PF01239.2    .........................................MNYITAIIEEQPKNYQVWHHRRVLVE...W......ELEFIADILNQDAKNYHAWQHRQWVIQ......  54
PF01239      ......FKLTRDAIELNAANYTVWHFRRVLLK........EMNYITAIIEEQPKNYQVWHHRRVLVE...W......ELEFIADILNQDAKNYHAWQHRQWVIQ......  81
PF01239.1    ......FKLTRDAIELNAANYTVWHFRRVLLK........EMNYITAIIEEQPKNYQVWHHRRVLVE...W......ELEFIADILNQDAKNYHAWQHRQWVIQ......  81
FNTA-204     .........................................MNYITAIIEEQPKNYQVWHHRRVLVE...WLRDPSQELEFIADILNQDAKNYHAWQHRQWVIQ......  60
FNTA-214     ........................HFRRVLLKSLQKDLHEEMNYITAIIEEQPKNYQVWNLNFGIMSCSMWTNF.................................... 117
FNTA-210     ERSERAFKLTRDAIELNAANYTVWHFRRVLLKSLQKDLHEEMNYITAIIEEQPKNYQVWHHRRVLVE...WLRDPSQELEFIADILNQDAKNYHAWQHRQWVIQEFKLWD 155
FNTA-201     ERSERAFKLTRDAIELNAANYTVWHFRRVLLKSLQKDLHEEMNYITAIIEEQPKNYQVWHHRRVLVE...WLRDPSQELEFIADILNQDAKNYHAWQHRQWVIQEFKLWD 217
FNTA-209     ERSERAFKLTRDAIELNAANYTVWHFRRVLLKSLQKDLHEEMNYITAIIEEQPKNYQVWHHRRVLVE...WLRDPSQELEFIADILNQDAKNYHAWQHRQWVIQEFK... 196
FNTA-208     .........................................MNYITAIIEEQPKNYQVWHHRRVLVE...WLRDPSQELEFIADILNQDAKNYHAWQHRQWVIQEFKLWD  66
FNTA-205     ..............................................................................................................  67
FNTA-212     ..............................................................................................................  67
```

logo

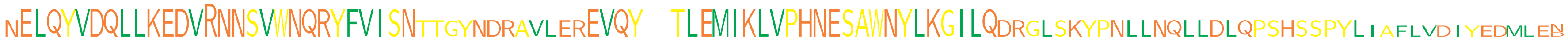

```
TIPIFARNIB   ..............R...............................................................................................D   5
PF01239.3    .ELQYVDQLLKEDVRNNSVWNQRYFVISN............EVQY..TLEMIKLVPHNESAWNYLKGILQ........................................ 109
PF01239.2    ..............................................................................................................  54
PF01239      .ELQYVDQLLKEDVRNNSVWNQRYFVISN............EVQY..TLEMIKLVPHNESAWNYLKGILQ........................................ 136
PF01239.1    ..............................................................................................................  81
FNTA-204     ..............................................................................................................  60
FNTA-214     .............................................................................................................. 117
FNTA-210     NELQYVDQLLKEDVRNNSVWNQRYFVISNTTGYNDRAVLEREVQY..TLEMIKLVPHNESAWNYLKGILQDRGLSKYPNLLNQLLDLQPSHSSPYL.............. 249
FNTA-201     NELQYVDQLLKEDVRNNSVWNQRYFVISNTTGYNDRAVLEREVQY..TLEMIKLVPHNESAWNYLKGILQDRGLSKYPNLLNQLLDLQPSHSSPYLIAFLVDIYEDMLEN 325
FNTA-209     .............................................................................................................. 196
FNTA-208     NELQYVDQLLKEDVRNNSVWNQRYFVISNTTGYNDRAVLEREVQY..TLEMIKLVPHNESAWNYLKGILQDRGLSKYPNLLNQLLDLQPSHSSPYLIAFLVDIYEDMLEN 174
FNTA-205     ..............................................................................................................  67
FNTA-212     ..............................................................................................................  67
```

logo

```
               QCDNKEDILNKALELCEILAKEKDTIRKEYWRYIGRSLQSKHSTENDSP        TNVQQ  YH
TIPIFARNIB     .CY..........................................................D.YH    10
PF01239.3      .................................................................   109
PF01239.2      .................................................................    54
PF01239        .................................................................   136
PF01239.1      .................................................................    81
FNTA-204       .................................................................    60
FNTA-214       .................................................................   117
FNTA-210       .................................................................   249
FNTA-201       QCDNKEDILNKALELCEILAKEKDTIRKEYWRYIGRSLQSKHSTENDSP........TNVQQ...   379
FNTA-209       .................................................................   196
FNTA-208       QCDNKEDILNKALELCEILAKEKDTIRKEYWRYIGRSLQSKHSTENDSP........TNVQQ...   228
FNTA-205       .................................................................    67
FNTA-212       .................................................................    67
```

- X non conserved
- X similar
- X $\geq$ 0% conserved
- X $\geq$ 50% conserved

```
logo        MASPSSFTYYCPPSSSPVWSEPLYSLRPEHARERLQDDSVETVTSIEQAKVEEKIQEVFSSYKFNHLVPRLVLQREKHFHYLKRGLRQLTDAYECLDASRPWLCYWILHS
TIPIFARNIB  ...............................................................................................L.....W...W....    3
PF00432     ..............................................................................................................    0
FNTB-201    MASPSSFTYYCPPSSSPVWSEPLYSLRPEHARERLQDDSVETVTSIEQAKVEEKIQEVFSSYKFNHLVPRLVLQREKHFHYLKRGLRQLTDAYECLDASRPWLCYWILHS  110

logo        LELLDEPIPQIVATDVCQFLELCQSPEGGFGGGPGQYPHLAPTYAAVNALCIIGTEEAYDIINREKLLQYLYSLKQPDGSFLMHVGGEVDVRSAYCAASVASLTNIITPD
TIPIFARNIB  ...........................................................................................R..................    4
PF00432     ...............VCQFLELCQSPEGGFGGGPGQYPHLAPTYAAVNALCIIG.......INREKLLQYLYSLKQPDGSFLMHVGGEVDVRSAYCAASVASL.......   81
FNTB-201    LELLDEPIPQIVATDVCQFLELCQSPEGGFGGGPGQYPHLAPTYAAVNALCIIGTEEAYDIINREKLLQYLYSLKQPDGSFLMHVGGEVDVRSAYCAASVASLTNIITPD  220

logo        LFEGTAEWIARCQNWEGGIGGVPGMEAHGGYTFCGLAALVILKRERSLNLKSLLQWVTSRQMRFEGGFQGRCNKLVDGCYSFWQAGLLPLLHRALHAQGDPALSMSHWMF
TIPIFARNIB  ............................................................................D.CY..............................    7
PF00432     ....TAEWIARCQNWEGGIGGVPGMEAHGGYTFCGLAALVIL......NLKSLLQWVTSRQMRFEGGFQGRCNKLVDGCYSFWQAGLLPLL...................  162
FNTB-201    LFEGTAEWIARCQNWEGGIGGVPGMEAHGGYTFCGLAALVILKRERSLNLKSLLQWVTSRQMRFEGGFQGRCNKLVDGCYSFWQAGLLPLLHRALHAQGDPALSMSHWMF  330

logo        HQQALQEYILMCCQCPAGGLLDKPGKSRDFYHTCYCLSGLSIAQHFGSGAMLHDVVLGVPENALQPTHPVYNIGPDKVIQATTYFLQKPVPGFEELKDETSAEPATD
TIPIFARNIB  ............................D.YH...........................................................................   10
PF00432     HQQALQEYILMCCQCPAGGLLDKPGKSRDFYHTCYCLSGLSIA................................................................  205
FNTB-201    HQQALQEYILMCCQCPAGGLLDKPGKSRDFYHTCYCLSGLSIAQHFGSGAMLHDVVLGVPENALQPTHPVYNIGPDKVIQATTYFLQKPVPGFEELKDETSAEPATD  437
```

X non conserved

X similar

X $\geq$ 0% conserved

X $\geq$ 50% conserved

logo

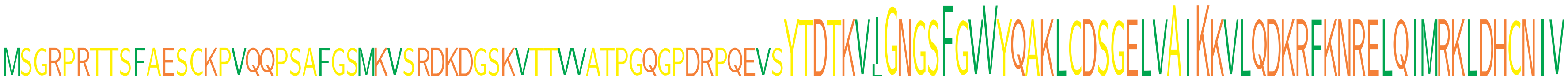

```
A-443654     .............................................................LG...F..V............A.K.........................   6
PF00069.1    .......................................................YTDTKVIGNGSFGVVYQAKLCDSGELVAIKKVLQDKRFKNRELQIMRKLDHCNIV  55
PF00069      .......................................................YTDTKVIGNGSFGVVYQAKLCDSGELVAIKKVLQDKRFKNRELQIMRKLDHCNIV  55
GSK3B-202    MSGRPRTTSFAESCKPVQQPSAFGSMKVSRDKDGSKVTTVVATPGQGPDRPQEVSYTDTKVIGNGSFGVVYQAKLCDSGELVAIKKVLQDKRFKNRELQIMRKLDHCNIV 110
GSK3B-201    MSGRPRTTSFAESCKPVQQPSAFGSMKVSRDKDGSKVTTVVATPGQGPDRPQEVSYTDTKVIGNGSFGVVYQAKLCDSGELVAIKKVLQDKRFKNRELQIMRKLDHCNIV 110
```

logo

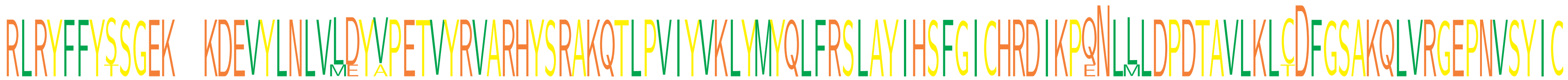

```
A-443654     .......T...............ME.A.................................................EN.M..........TD..................  15
PF00069.1    RLRYFFYSSGEK..KDEVYLNLVLDYVPETVYRVARHYSRAKQTLPVIYVKLYMYQLFRSLAYIHSFGICHRDIKPQNLLLDPDTAVLKLCDFGSAKQLVRGEPNVSYIC 163
PF00069      RLRYFFYSSGEK..KDEVYLNLVLDYVPETVYRVARHYSRAKQTLPVIYVKLYMYQLFRSLAYIHSFGICHRDIKPQNLLLDPDTAVLKLCDFGSAKQLVRGEPNVSYIC 163
GSK3B-202    RLRYFFYSSGEK..KDEVYLNLVLDYVPETVYRVARHYSRAKQTLPVIYVKLYMYQLFRSLAYIHSFGICHRDIKPQNLLLDPDTAVLKLCDFGSAKQLVRGEPNVSYIC 218
GSK3B-201    RLRYFFYSSGEK..KDEVYLNLVLDYVPETVYRVARHYSRAKQTLPVIYVKLYMYQLFRSLAYIHSFGICHRDIKPQNLLLDPDTAVLKLCDFGSAKQLVRGEPNVSYIC 218
```

logo

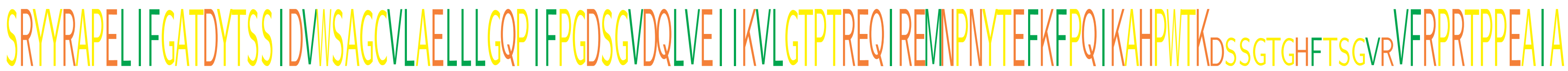

```
A-443654     ..............................................................................................................  15
PF00069.1    SRYYRAPELIFGATDYTSSIDVWSAGCVLAELLLGQPIFPGDSGVDQLVEIIKVLGTPTREQIREMNPNYTEFKFPQIKAHPWTKDSSGTGHFTSGVRVFRPRTPPEAIA 273
PF00069      SRYYRAPELIFGATDYTSSIDVWSAGCVLAELLLGQPIFPGDSGVDQLVEIIKVLGTPTREQIREMNPNYTEFKFPQIKAHPWTK.............VFRPRTPPEAIA 260
GSK3B-202    SRYYRAPELIFGATDYTSSIDVWSAGCVLAELLLGQPIFPGDSGVDQLVEIIKVLGTPTREQIREMNPNYTEFKFPQIKAHPWTKDSSGTGHFTSGVRVFRPRTPPEAIA 328
GSK3B-201    SRYYRAPELIFGATDYTSSIDVWSAGCVLAELLLGQPIFPGDSGVDQLVEIIKVLGTPTREQIREMNPNYTEFKFPQIKAHPWTK.............VFRPRTPPEAIA 315
```

logo

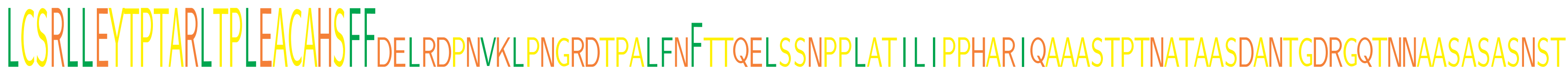

```
A-443654     ..............................................F..........................................................  16
PF00069.1    LCSRLLEYTPTARLTPLEACAHSFF................................................................................  298
PF00069      LCSRLLEYTPTARLTPLEACAHSFF................................................................................  285
GSK3B-202    LCSRLLEYTPTARLTPLEACAHSFFDELRDPNVKLPNGRDTPALFNFTTQELSSNPPLATILIPPHARIQAAASTPTNATAASDANTGDRGQTNNAASASASNST  433
GSK3B-201    LCSRLLEYTPTARLTPLEACAHSFFDELRDPNVKLPNGRDTPALFNFTTQELSSNPPLATILIPPHARIQAAASTPTNATAASDANTGDRGQTNNAASASASNST  420
```

- X non conserved
- X similar
- X ≥ 0% conserved
- X ≥ 50% conserved

```
logo          MSGRPRTTSFAESCKPVQQPSAFGSMKVSRDKDGSKVTTVVATPGQGPDRPQEVSYTDTKVIGNGSFGVVYQAKLCDSGELVAIKKVLQDKRFKNRELQIMRKLDHCNI
CHEMBL428462  ..............................................................GKG..GKV............A.K.L.......................    9
PF00069.1     .......................................................YTDTKVIGNGSFGVVYQAKLCDSGELVAIKKVLQDKRFKNRELQIMRKLDHCNI.   54
PF00069       .......................................................YTDTKVIGNGSFGVVYQAKLCDSGELVAIKKVLQDKRFKNRELQIMRKLDHCNI.   54
GSK3B-202     MSGRPRTTSFAESCKPVQQPSAFGSMKVSRDKDGSKVTTVVATPGQGPDRPQEVSYTDTKVIGNGSFGVVYQAKLCDSGELVAIKKVLQDKRFKNRELQIMRKLDHCNI.  109
GSK3B-201     MSGRPRTTSFAESCKPVQQPSAFGSMKVSRDKDGSKVTTVVATPGQGPDRPQEVSYTDTKVIGNGSFGVVYQAKLCDSGELVAIKKVLQDKRFKNRELQIMRKLDHCNI.  109

logo          VRLRYFFYSSGEKKDEVYLNLVLDYVPETVYRVARHYSRAKQTLPVIYVKLYMYQLFRSLAYIHSFGICHRDIKPQNLLLDPDTAVLKLCDFGSAKQLVRGEPNVSYICS
CHEMBL428462  ......................MEYA...E..............................................E..M..............................   16
PF00069.1     VRLRYFFYSSGEKKDEVYLNLVLDYVPETVYRVARHYSRAKQTLPVIYVKLYMYQLFRSLAYIHSFGICHRDIKPQNLLLDPDTAVLKLCDFGSAKQLVRGEPNVSYICS  164
PF00069       VRLRYFFYSSGEKKDEVYLNLVLDYVPETVYRVARHYSRAKQTLPVIYVKLYMYQLFRSLAYIHSFGICHRDIKPQNLLLDPDTAVLKLCDFGSAKQLVRGEPNVSYICS  164
GSK3B-202     VRLRYFFYSSGEKKDEVYLNLVLDYVPETVYRVARHYSRAKQTLPVIYVKLYMYQLFRSLAYIHSFGICHRDIKPQNLLLDPDTAVLKLCDFGSAKQLVRGEPNVSYICS  219
GSK3B-201     VRLRYFFYSSGEKKDEVYLNLVLDYVPETVYRVARHYSRAKQTLPVIYVKLYMYQLFRSLAYIHSFGICHRDIKPQNLLLDPDTAVLKLCDFGSAKQLVRGEPNVSYICS  219

logo          RYYRAPELIFGATDYTSSIDVWSAGCVLAELLLGQPIFPGDSGVDQLVEIIKVLGTPTREQIREMNPNYTEFKFPQIKAHPWTKDSSGTGHFTSGVRVFRPRTPPEAIAL
CHEMBL428462  ............TD................................................................................................   18
PF00069.1     RYYRAPELIFGATDYTSSIDVWSAGCVLAELLLGQPIFPGDSGVDQLVEIIKVLGTPTREQIREMNPNYTEFKFPQIKAHPWTKDSSGTGHFTSGVRVFRPRTPPEAIAL  274
PF00069       RYYRAPELIFGATDYTSSIDVWSAGCVLAELLLGQPIFPGDSGVDQLVEIIKVLGTPTREQIREMNPNYTEFKFPQIKAHPWTK.............VFRPRTPPEAIAL  261
GSK3B-202     RYYRAPELIFGATDYTSSIDVWSAGCVLAELLLGQPIFPGDSGVDQLVEIIKVLGTPTREQIREMNPNYTEFKFPQIKAHPWTKDSSGTGHFTSGVRVFRPRTPPEAIAL  329
GSK3B-201     RYYRAPELIFGATDYTSSIDVWSAGCVLAELLLGQPIFPGDSGVDQLVEIIKVLGTPTREQIREMNPNYTEFKFPQIKAHPWTK.............VFRPRTPPEAIAL  316

logo          CSRLLEYTPTARLTPLEACAHSFFDELRDPNVKLPNGRDTPALFNFTTQELSSNPPLATILIPPHARIQAAASTPTNATAASDANTGDRGQTNNAASASASNST
CHEMBL428462  ........................................................................................................   18
PF00069.1     CSRLLEYTPTARLTPLEACAHSFF................................................................................  298
PF00069       CSRLLEYTPTARLTPLEACAHSFF................................................................................  285
GSK3B-202     CSRLLEYTPTARLTPLEACAHSFFDELRDPNVKLPNGRDTPALFNFTTQELSSNPPLATILIPPHARIQAAASTPTNATAASDANTGDRGQTNNAASASASNST  433
GSK3B-201     CSRLLEYTPTARLTPLEACAHSFFDELRDPNVKLPNGRDTPALFNFTTQELSSNPPLATILIPPHARIQAAASTPTNATAASDANTGDRGQTNNAASASASNST  420
```

X non conserved

X similar

X $\geq 0\%$ conserved

X $\geq 50\%$ conserved

```
logo
CHEMBL1081312  ..............................................................................................................    0
PF00018        ..........................................................................................VALYDYEAIHHEDLSFQKGD   20
PF07714        ..............................................................................................................    0
PF00017        ..............................................................................................................    0
HCK-203        LGGRSSCEDPGCPRDEERAPRMGCMKS.KFLQVGG.NTFSKTE....TSASPHCPVYYVPDPTSTIKPGPNSHNSNTPGIRE.GSEDIIVVALYDYEAIHHEDLSFQKGD  102
HCK-207        .....................MGCMKS.KFLQVGG.NTFSKTE....TSASPHCPVYYVPDPTSTIKPGPNSHNSNTPGIREAGSEDIIVVALYDYEAIHHEDLSFQKGD   82
HCK-210        .....................MGCMKS.KFLQVGG.NTFSKTE....TSASPHCPVYYVPDPTSTIKPGPNSHNSNTPGIREAGSEDIIVVALYDYEAIHHEDLSFQKGD   82
HCK-205        LGGRSSCEDPGCPRDEERAPRQTKQNRS.SLEVANLSQFPPEEDGVHEVQVPPGRRQYILKN................................................   61
HCK-201        LGGRSSCEDPGCPRDEERAPRGNRCHELQSFQD..............VPLKMDCI.......................................................   41
HCK-202        LGGRSSCEDPGCPRDEERAPRMGCMKS.KFLQVGG.NTFSKTE....TSASPHCPVYYVPDPTSTIKPGPNSHNSNTPGIREAGSEDIIVVALYDYEAIHHEDLSFQKGD  103
HCK-206        .....................MGCMKS.KFLQVGG.NTFSKTE....TSASPHCPVYYVPDPTSTIKPGPNSHNSNTPGIRE.GSEDIIVVALYDYEAIHHEDLSFQKGD   81
HCK-208        LGGRSSCEDPGCPRDEERAPRMGCMKS.KFLQVGG.NTFSKTE....TSASPHCPVYYVPDPTSTIKPGPNSHNSNTPGIREAGSEDIIVVALYDYEAIHHEDLSFQKGD  103
HCK-209        .....................MGCMKS.KFLQVGG.NTFSKTE....TSASPHCPVYYVPDPTSTIKPGPNSHNSNTPGIREAGSEDIIVVALYDYEAIHHEDLSFQKGD   82

logo
CHEMBL1081312  ..............................................................................................................    0
PF00018        QMVVLEESGEWWKARSLATRKEGYIPS...................................................................................   47
PF07714        ..............................................................................................................    0
PF00017        ........................................WFFKGISRKDAERQLLAPGNMLGSFMIRDSETTKGSYSLSVRDYDPRQGDTVKHYKIRTLDNGGFYISPR   70
HCK-203        QMVVLEESGEWWKARSLATRKEGYIPSNYVARVDSLETEEWFFKGISRKDAERQLLAPGNMLGSFMIRDSETTKGSYSLSVRDYDPRQGDTVKHYKIRTLDNGGFYISPR  212
HCK-207        QMVVLEESGEWWKARSLATRKEGYIPSNYVARVDSLETEEWFFKGISRKDAERQLLAPGNMLGSFMIRDSETTKGSYSLSVRDYDPRQGDTVKHYKIRTLDNGGFYISPR  192
HCK-210        QMVVLEESGEWWKARSLATRKEGYIPSNYVARVDSLETEEWFFKGISRKDAERQLLAPGNMLGSFMIRDSETTKGSYSLSVRDYDPRQGDTVKHYKIRTLDNGGFYISPR  192
HCK-205        ..............................................................................................................   61
HCK-201        ..............................................................................................................   41
HCK-202        QMVVLEESGEWWKARSLATRKEGYIPSNYVARVDSLETEEWFFKGISRKDAERQLLAPGNMLGSFMIRDSETTKGSYSLSVRDYDPRQGDTVKHYKIRTLDNGGFYISPR  213
HCK-206        QMVVLEESGEWWKARSLATRKEGYIPSNYVARVDSLETEEWFFKGISRKDAERQLLAPGNMLGSFMIRDSETTKGSYSLSVRDYDPRQGDTVKHYKIRTLDNGGFYISPR  191
HCK-208        QMVVLEESGEWWKARSLATRKEGYIPSNYVARVDSLETEEWFFKGISRKDAERQLLAPGNMLGSFMIRDSETTKGSYSLSVRDYDPRQGDTVKHYKIRTLDNGGFYISPR  213
HCK-209        QMVVLEESGEWWKARSLATRKEGYIPSNYVARVDSLETEEWFFKGISRKDAERQLLAPGNMLGSFMIRDSETTKGSYSLSVRDYDPRQGDTVKHYKIRTLDNGGFYISPR  192

logo
CHEMBL1081312  ......................................................L.......V...........A.K.................................    4
PF00018        ..............................................................................................................   47
PF07714        ................................................LKLEKKLGAGQFGEVWMATYNKHTKVAVKTMKPGSMSVEAFLAEANVMKTLQHDKLVKLHAV   62
PF00017        STFSTLQELVDHY.................................................................................................   83
HCK-203        STFSTLQELVDHYKKGNDGLCQKLSVPCMSSKPQKPWEKDAWEIPRESLKLEKKLGAGQFGEVWMATYNKHTKVAVKTMKPGSMSVEAFLAEANVMKTLQHDKLVKLHAV  322
HCK-207        STFSTLQELVDHYKKGNDGLCQKLSVPCMSSKPQKPWEKDAWEIPRESLKLEKKLGAGQFGEVWMATYNKHTKVAVKTMKPGSMSVEAFLAEANVMKTLQHDKLVKLHAV  302
HCK-210        STFSTLQELVDHYKKGNDGLCQKLSVPCMSSKPQKPWEKDAWEIPRESLKLEKKLGAGQFGEVWMATYNKHTKVAVKTMKPGSMSVEAFLAEANVMKTLQHDKLVKLHAV  302
HCK-205        ..............................................................................................................   61
HCK-201        ..............................................................................................................   41
HCK-202        STFSTLQELVDHYKKGNDGLCQKLSVPCMSSKPQKPWEKDAWEIPRESLKLEKKLGAGQFGEVWMATYNKHTKVAVKTMKPGSMSVEAFLAEANVMKTLQHDKLVKLHAV  323
HCK-206        STFSTLQELVDHYKKGNDGLCQKLSVPCMSSKPQKPWEKDAWEIPRESLKLEKKLGAGQFGEVWMATYNKHTKVAVKTMKPGSMSVEAFLAEANVMKTLQHDKLVKLHAV  301
HCK-208        STFSTLQELVDHYKKGNDGLCQKLSVPCMSSKPQKPWEKDAWEIPRESLKLEKKLGAGQFGEVWMATYNKHTKVAVKTMKPGSMSVEAFLAEANVMKTLQHDKLVKLHAV  323
HCK-209        STFSTLQELVDHYKKGNDGLCQKLSVPCMSSKPQKPWEKDAWEIPRESLKLEKKLGAGQFGEVWMATYNKHTKVAVKTMKPGSMSVEAFLAEANVMKTLQHDKLVKLHAV  302
```

```
logo            VTKEPIYIITEFMAKGSLLDFLKSDEGSKQPLPKLIDFSAQIAEGMAFIEQRNYIHRDLRAANILVSASLVCKIADFGLARVIEDNEYTAREGAKFPIKWTAPEAINFGS
CHEMBL1081312   .......I.TEFM..GS...............................................L.........AD..................................  14
PF00018         ..............................................................................................................  47
PF07714         VTKEPIYIITEFMAKGSLLDFLKSDEGSKQPLPKLIDFSAQIAEGMAFIEQRNYIHRDLRAANILVSASLVCKIADFGLARVIEDNEYTAREGAKFPIKWTAPEAINFGS  172
PF00017         ..............................................................................................................  83
HCK-203         VTKEPIYIITEFMAKGSLLDFLKSDEGSKQPLPKLIDFSAQIAEGMAFIEQRNYIHRDLRAANILVSASLVCKIADFGLARVIEDNEYTAREGAKFPIKWTAPEAINFGS  432
HCK-207         VTKEPIYIITEFMAKGSLLDFLKSDEGSKQPLPKLIDFSAQIAEGMAFIEQRNYIHRDLRAANILVSASLVCKIADFGLARVIEDNEYTAREGAKFPIKWTAPEAINFGS  412
HCK-210         VTKEPIYIITEFMAKGSLLDFLKSDEGSKQPLPKLIDFSAQIAEGMAFIEQRNYIHRDLRAANILVSASLVCKIADFGLARVIEDNEYTAREGAKFPIKWTAPEAINFGS  412
HCK-205         ..............................................................................................................  61
HCK-201         ..............................................................................................................  41
HCK-202         VTKEPIYIITEFMAKGSLLDFLKSDEGSKQPLPKLIDFSAQIAEGMAFIEQRNYIHRDLRAANILVSASLVCKIADFGLARVIEDNEYTAREGAKFPIKWTAPEAINFGS  433
HCK-206         VTKEPIYIITEFMAKGSLLDFLKSDEGSKQPLPKLIDFSAQIAEGMAFIEQRNYIHRDLRAANILVSASLVCKIADFGLARVIEDNEYTAREGAKFPIKWTAPEAINFGS  411
HCK-208         VTKEPIYIITEFMAKGSLLDFLKSDEGSKQPLPKLIDFSAQIAEGMAFIEQRNYIHRDLRAANILVSASLVCKIADFGLARVIEDNEYTAREGAKFPIKWTAPEAINFGS  433
HCK-209         VTKEPIYIITEFMAKGSLLDFLKSDEGSKQPLPKLIDFSAQIAEGMAFIEQRNYIHRDLRAANILVSASLVCKIADFGLARVIEDNEYTAREGAKFPIKWTAPEAINFGS  412

logo            FTIKSDVWSFGILLMEIVTYGRIPYPGMSNPEVIRALERGYRMPRPENCPEELYNIMMRCWKNRPEERPTFEYIQSVLDDFYTATESQYQQQP
CHEMBL1081312   .............................................................................................  14
PF00018         .............................................................................................  47
PF07714         FTIKSDVWSFGILLMEIVTYGRIPYPGMSNPEVIRALERGYRMPRPENCPEELYNIMMRCWKNRPEERPTFEYIQSV................  249
PF00017         .............................................................................................  83
HCK-203         FTIKSDVWSFGILLMEIVTYGRIPYPGMSNPEVIRALERGYRMPRPENCPEELYNIMMRCWKNRPEERPTFEYIQSVLDDFYTATESQYQQQP  525
HCK-207         FTIKSDVWSFGILLMEIVTYGRIPYPGMSNPEVIRALERGYRMPRPENCPEELYNIMMRCWKNRPEERPTFEYIQSVLDDFYTATESQYQQQP  505
HCK-210         FTIKSDVWSFGILLMEIVTYGRIPYPGMSNPEVIRALERGYRMPRPENCPEELYNIMMRCWKNRPEERPTFEYIQSVLDDFYTATESQYQQQP  505
HCK-205         .............................................................................................  61
HCK-201         .............................................................................................  41
HCK-202         FTIKSDVWSFGILLMEIVTYGRIPYPGMSNPEVIRALERGYRMPRPENCPEELYNIMMRCWKNRPEERPTFEYIQSVLDDFYTATESQYQQQP  526
HCK-206         FTIKSDVWSFGILLMEIVTYGRIPYPGMSNPEVIRALERGYRMPRPENCPEELYNIMMRCWKNRPEERPTFEYIQSVLDDFYTATESQYQQQP  504
HCK-208         FTIKSDVWSFGILLMEIVTYGRIPYPGMSNPEVIRALERGYRMPRPENCPEELYNIMMRCWKNRPEERPTFEYIQSVLDDFYTATESQYQQQP  526
HCK-209         FTIKSDVWSFGILLMEIVTYGRIPYPGMSNPEVIRALERGYRMPRPENCPEELYNIMMRCWKNRPEERPTFEYIQSVLDDFYTATESQYQQQP  505
```

X non conserved
X similar
X $\geq$ 0% conserved
X $\geq$ 50% conserved

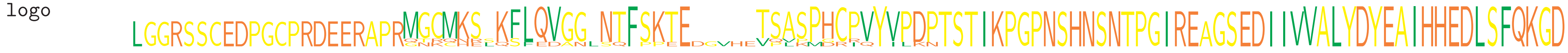

```
QUERCETIN  ..............................................................................................................   0
PF00018    ..........................................................................................VALYDYEAIHHEDLSFQKGD  20
PF07714    ..............................................................................................................   0
PF00017    ..............................................................................................................   0
HCK-203    LGGRSSCEDPGCPRDEERAPRMGCMKS.KFLQVGG.NTFSKTE.....TSASPHCPVYVPDPTSTIKPGPNSHNSNTPGIRE.GSEDIIVVALYDYEAIHHEDLSFQKGD 102
HCK-207    .....................MGCMKS.KFLQVGG.NTFSKTE.....TSASPHCPVYVPDPTSTIKPGPNSHNSNTPGIREAGSEDIIVVALYDYEAIHHEDLSFQKGD  82
HCK-210    .....................MGCMKS.KFLQVGG.NTFSKTE.....TSASPHCPVYVPDPTSTIKPGPNSHNSNTPGIREAGSEDIIVVALYDYEAIHHEDLSFQKGD  82
HCK-205    LGGRSSCEDPGCPRDEERAPRQTKQNRS.SLEVANLSQFPPEEDGVHEVQVPPGRRQYILKN................................................  61
HCK-201    LGGRSSCEDPGCPRDEERAPRGNRCHELQSFQD...............VPLKMDCI......................................................  41
HCK-202    LGGRSSCEDPGCPRDEERAPRMGCMKS.KFLQVGG.NTFSKTE.....TSASPHCPVYVPDPTSTIKPGPNSHNSNTPGIREAGSEDIIVVALYDYEAIHHEDLSFQKGD 103
HCK-206    .....................MGCMKS.KFLQVGG.NTFSKTE.....TSASPHCPVYVPDPTSTIKPGPNSHNSNTPGIRE.GSEDIIVVALYDYEAIHHEDLSFQKGD  81
HCK-208    LGGRSSCEDPGCPRDEERAPRMGCMKS.KFLQVGG.NTFSKTE.....TSASPHCPVYVPDPTSTIKPGPNSHNSNTPGIREAGSEDIIVVALYDYEAIHHEDLSFQKGD 103
HCK-209    .....................MGCMKS.KFLQVGG.NTFSKTE.....TSASPHCPVYVPDPTSTIKPGPNSHNSNTPGIREAGSEDIIVVALYDYEAIHHEDLSFQKGD  82
```

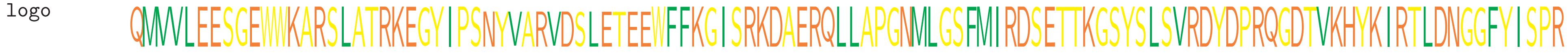

```
QUERCETIN  ..............................................................................................................   0
PF00018    QMVVLEESGEWWKARSLATRKEGYIPS...................................................................................  47
PF07714    ..............................................................................................................   0
PF00017    ........................................WFFKGISRKDAERQLLAPGNMLGSFMIRDSETTKGSYSLSVRDYDPRQGDTVKHYKIRTLDNGGFYISPR  70
HCK-203    QMVVLEESGEWWKARSLATRKEGYIPSNYVARVDSLETEEWFFKGISRKDAERQLLAPGNMLGSFMIRDSETTKGSYSLSVRDYDPRQGDTVKHYKIRTLDNGGFYISPR 212
HCK-207    QMVVLEESGEWWKARSLATRKEGYIPSNYVARVDSLETEEWFFKGISRKDAERQLLAPGNMLGSFMIRDSETTKGSYSLSVRDYDPRQGDTVKHYKIRTLDNGGFYISPR 192
HCK-210    QMVVLEESGEWWKARSLATRKEGYIPSNYVARVDSLETEEWFFKGISRKDAERQLLAPGNMLGSFMIRDSETTKGSYSLSVRDYDPRQGDTVKHYKIRTLDNGGFYISPR 192
HCK-205    ..............................................................................................................  61
HCK-201    ..............................................................................................................  41
HCK-202    QMVVLEESGEWWKARSLATRKEGYIPSNYVARVDSLETEEWFFKGISRKDAERQLLAPGNMLGSFMIRDSETTKGSYSLSVRDYDPRQGDTVKHYKIRTLDNGGFYISPR 213
HCK-206    QMVVLEESGEWWKARSLATRKEGYIPSNYVARVDSLETEEWFFKGISRKDAERQLLAPGNMLGSFMIRDSETTKGSYSLSVRDYDPRQGDTVKHYKIRTLDNGGFYISPR 191
HCK-208    QMVVLEESGEWWKARSLATRKEGYIPSNYVARVDSLETEEWFFKGISRKDAERQLLAPGNMLGSFMIRDSETTKGSYSLSVRDYDPRQGDTVKHYKIRTLDNGGFYISPR 213
HCK-209    QMVVLEESGEWWKARSLATRKEGYIPSNYVARVDSLETEEWFFKGISRKDAERQLLAPGNMLGSFMIRDSETTKGSYSLSVRDYDPRQGDTVKHYKIRTLDNGGFYISPR 192
```

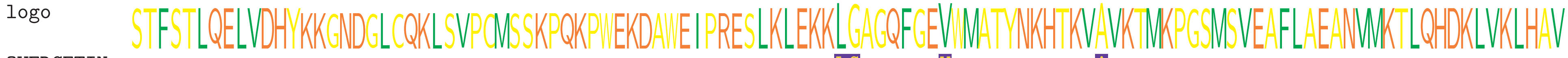

```
QUERCETIN  ......................................................LG......V...........A...................................   4
PF00018    ..............................................................................................................  47
PF07714    ................................................LKLEKKLGAGQFGEVWMATYNKHTKVAVKTMKPGSMSVEAFLAEANVMKTLQHDKLVKLHAV  62
PF00017    STFSTLQELVDHY.................................................................................................  83
HCK-203    STFSTLQELVDHYKKGNDGLCQKLSVPCMSSKPQKPWEKDAWEIPRESLKLEKKLGAGQFGEVWMATYNKHTKVAVKTMKPGSMSVEAFLAEANVMKTLQHDKLVKLHAV 322
HCK-207    STFSTLQELVDHYKKGNDGLCQKLSVPCMSSKPQKPWEKDAWEIPRESLKLEKKLGAGQFGEVWMATYNKHTKVAVKTMKPGSMSVEAFLAEANVMKTLQHDKLVKLHAV 302
HCK-210    STFSTLQELVDHYKKGNDGLCQKLSVPCMSSKPQKPWEKDAWEIPRESLKLEKKLGAGQFGEVWMATYNKHTKVAVKTMKPGSMSVEAFLAEANVMKTLQHDKLVKLHAV 302
HCK-205    ..............................................................................................................  61
HCK-201    ..............................................................................................................  41
HCK-202    STFSTLQELVDHYKKGNDGLCQKLSVPCMSSKPQKPWEKDAWEIPRESLKLEKKLGAGQFGEVWMATYNKHTKVAVKTMKPGSMSVEAFLAEANVMKTLQHDKLVKLHAV 323
HCK-206    STFSTLQELVDHYKKGNDGLCQKLSVPCMSSKPQKPWEKDAWEIPRESLKLEKKLGAGQFGEVWMATYNKHTKVAVKTMKPGSMSVEAFLAEANVMKTLQHDKLVKLHAV 301
HCK-208    STFSTLQELVDHYKKGNDGLCQKLSVPCMSSKPQKPWEKDAWEIPRESLKLEKKLGAGQFGEVWMATYNKHTKVAVKTMKPGSMSVEAFLAEANVMKTLQHDKLVKLHAV 323
HCK-209    STFSTLQELVDHYKKGNDGLCQKLSVPCMSSKPQKPWEKDAWEIPRESLKLEKKLGAGQFGEVWMATYNKHTKVAVKTMKPGSMSVEAFLAEANVMKTLQHDKLVKLHAV 302
```

```
logo       VTKEPIYIITEFMAKGSLLDFLKSDEGSKQPLPKLIDFSAQIAEGMAFIEQRNYIHRDLRAANILVSASLVCKIADFGLARVIEDNEYTAREGAKFPIKWTAPEAINFGS
QUERCETIN  .........TEFM..G...D..........................................................L...............................   11
PF00018    ..............................................................................................................   47
PF07714    VTKEPIYIITEFMAKGSLLDFLKSDEGSKQPLPKLIDFSAQIAEGMAFIEQRNYIHRDLRAANILVSASLVCKIADFGLARVIEDNEYTAREGAKFPIKWTAPEAINFGS  172
PF00017    ..............................................................................................................   83
HCK-203    VTKEPIYIITEFMAKGSLLDFLKSDEGSKQPLPKLIDFSAQIAEGMAFIEQRNYIHRDLRAANILVSASLVCKIADFGLARVIEDNEYTAREGAKFPIKWTAPEAINFGS  432
HCK-207    VTKEPIYIITEFMAKGSLLDFLKSDEGSKQPLPKLIDFSAQIAEGMAFIEQRNYIHRDLRAANILVSASLVCKIADFGLARVIEDNEYTAREGAKFPIKWTAPEAINFGS  412
HCK-210    VTKEPIYIITEFMAKGSLLDFLKSDEGSKQPLPKLIDFSAQIAEGMAFIEQRNYIHRDLRAANILVSASLVCKIADFGLARVIEDNEYTAREGAKFPIKWTAPEAINFGS  412
HCK-205    ..............................................................................................................   61
HCK-201    ..............................................................................................................   41
HCK-202    VTKEPIYIITEFMAKGSLLDFLKSDEGSKQPLPKLIDFSAQIAEGMAFIEQRNYIHRDLRAANILVSASLVCKIADFGLARVIEDNEYTAREGAKFPIKWTAPEAINFGS  433
HCK-206    VTKEPIYIITEFMAKGSLLDFLKSDEGSKQPLPKLIDFSAQIAEGMAFIEQRNYIHRDLRAANILVSASLVCKIADFGLARVIEDNEYTAREGAKFPIKWTAPEAINFGS  411
HCK-208    VTKEPIYIITEFMAKGSLLDFLKSDEGSKQPLPKLIDFSAQIAEGMAFIEQRNYIHRDLRAANILVSASLVCKIADFGLARVIEDNEYTAREGAKFPIKWTAPEAINFGS  433
HCK-209    VTKEPIYIITEFMAKGSLLDFLKSDEGSKQPLPKLIDFSAQIAEGMAFIEQRNYIHRDLRAANILVSASLVCKIADFGLARVIEDNEYTAREGAKFPIKWTAPEAINFGS  412

logo       FTIKSDVWSFGILLMEIVTYGRIPYPGMSNPEVIRALERGYRMPRPENCPEELYNIMMRCWKNRPEERPTFEYIQSVLDDFYTATESQYQQQP
QUERCETIN  .............................................................................................   11
PF00018    .............................................................................................   47
PF07714    FTIKSDVWSFGILLMEIVTYGRIPYPGMSNPEVIRALERGYRMPRPENCPEELYNIMMRCWKNRPEERPTFEYIQSV................  249
PF00017    .............................................................................................   83
HCK-203    FTIKSDVWSFGILLMEIVTYGRIPYPGMSNPEVIRALERGYRMPRPENCPEELYNIMMRCWKNRPEERPTFEYIQSVLDDFYTATESQYQQQP  525
HCK-207    FTIKSDVWSFGILLMEIVTYGRIPYPGMSNPEVIRALERGYRMPRPENCPEELYNIMMRCWKNRPEERPTFEYIQSVLDDFYTATESQYQQQP  505
HCK-210    FTIKSDVWSFGILLMEIVTYGRIPYPGMSNPEVIRALERGYRMPRPENCPEELYNIMMRCWKNRPEERPTFEYIQSVLDDFYTATESQYQQQP  505
HCK-205    .............................................................................................   61
HCK-201    .............................................................................................   41
HCK-202    FTIKSDVWSFGILLMEIVTYGRIPYPGMSNPEVIRALERGYRMPRPENCPEELYNIMMRCWKNRPEERPTFEYIQSVLDDFYTATESQYQQQP  526
HCK-206    FTIKSDVWSFGILLMEIVTYGRIPYPGMSNPEVIRALERGYRMPRPENCPEELYNIMMRCWKNRPEERPTFEYIQSVLDDFYTATESQYQQQP  504
HCK-208    FTIKSDVWSFGILLMEIVTYGRIPYPGMSNPEVIRALERGYRMPRPENCPEELYNIMMRCWKNRPEERPTFEYIQSVLDDFYTATESQYQQQP  526
HCK-209    FTIKSDVWSFGILLMEIVTYGRIPYPGMSNPEVIRALERGYRMPRPENCPEELYNIMMRCWKNRPEERPTFEYIQSVLDDFYTATESQYQQQP  505
```

X non conserved
X similar
X $\geq$ 0% conserved
X $\geq$ 50% conserved

```
logo
VORINOSTAT   ...................................HP.....................................................................D...    3
PF00850.4    ......................................KPHRIRMTHNLLLNYGLYRKMEIYRPHKATAEEMTKYHSDEYIKFLRSIRPDNMSEYSKQMQRFNVGEDCPV   72
PF00850.9    ...................................HPMKPHRIRMTHNLLLNYGLYRKMEIYRPHKATAEEMTKYHSDEYIKFLRSIRPDNMSEYSKQMQRFNVGEDCPV   75
PF00850.6    ......................................KPHRIRMTHNLLLNYGLYRKMEIYRPHKATAEEMTKYHSDEYIKFLRSIRPD....................   52
PF00850.3    ...................................HPMKPHRIRMTHNLLLNYGLYRKMEIYRPHKATAEEMTKYHSDEYIKFLRSIRPDNMSEYSKQMQ..........   65
PF00850.2    ......................................KPHRIRMTHNLLLNYGLYRKMEIYRPHKATAEEMTKYHSDEYIKFLRSIRPDNMSEYSKQMQRFNVGEDCPV   72
PF00850      ......................................KPHRIRMTHNLLLNYGLYRKMEIYRPHKATAEEMTKYHSDEYIKFLRSIRPDNMSEYSKQMQRFNVGEDCPV   72
PF00850.1    ...................................HPMKPHRIRMTHNLLLNYGLYRKMEIYRPHKATAEEMTKYHSDEYIKFLRSIRPDNMSEYSKQMQRFNVGEDCPV   75
PF00850.8    ......................................KPHRIRMTHNLLLNYGLYRKMEIYRPHKATAEEMTKYHSDEYIKFLRSIRPDNMSEYSKQMQRFNVGEDCPV   72
PF00850.7    ......................................KPHRIRMTHNLLLNYGLYRKMEIYRPHKATAEEMTKYHSDEYIKFLRSIRPDNMSE................   56
PF00850.5    ......................................KPHRIRMTHNLLLNYGLYRKMEIYRPHKATAEEMTKYHSDEYIKFLRSIRPDNMSEYSKQMQ..........   62
HDAC2-206    .....................................MKPHRIRMTHNLLLNYGLYRKMEIYRPHKATAEEMTKYHSDEYIKFLRSIRPDNMSEYSKQMQRFNVGEDCPV   73
HDAC2-202    ..........KVCYYYD.......GDIGNYYYGQGHPMKPHRIRMTHNLLLNYGLYRKMEIYRPHKATAEEMTKYHSDEYIKFLRSIRPDNMSEYSKQMQRFNVGEDCPV   93
HDAC2-217    .....................................MKPHRIRMTHNLLLNYGLYRKMEIYRPHKATAEEMTKYHSDEYIKFLRSIRP.....................   52
HDAC2-203    .....................................MKPHRIRMTHNLLLNYGLYRKMEIYRPHKATAEEMTKYHSDEYIKFLRSIRPDNMSEYSKQMQRFNVGEDC..   71
HDAC2-201    .....................................MKPHRIRMTHNLLLNYGLYRKMEIYRPHKATAEEMTKYHSDEYIKFLRSIRPDNMSEYSKQMQRFNVGEDCPV   73
HDAC2-210    MAYSQGGGKKKVCYYYDVCVFSILGDIGNYYYGQGHPMKPHRIRMTHNLLLNYGLYRKMEIYRPHKATAEEMTKYHSDEYIKFLRSIRPDNMSEYSKQMQRFNVGEDCP.  109
HDAC2-205    MAYSQGGGKKKVCYYYD.......GDIGNYYYGQGHPMKPHRIRMTHNLLLNYGLYRKMEIYRPHKATAEEMTKYHSDEYIKFLRSIRPDNMSEYSKQMQRFNVGEDCPV  103
HDAC2-209    .....................................MKPHRIRMTHNLLLNYGLYRKMEIYRPHKATAEEMTKYHSDEYIKFLRSIRPDNMSEYSK.............   60
HDAC2-213    .....................................MKPHRIRMTHNLLLNYGLYRKMEIYRPHKATAEEMTKYHSDEYIKFLRSIRPDNMSEYSKQMQRFNVGEDCPV   73
HDAC2-212    .....................................MKPHRIRMTHNLLLNYGLYRKMEIYRPHKATAEEMTKYHSDEYIKFLRSIRPDNMSEYSKQMQRFNVGEDCPV   73
HDAC2-214    .....................................MKPHRIRMTHNLLLNYGLYRKMEIYRPHKATAEEMTKYHSDEYIKFLRSIRPDNMSEYSKQMQRF........   65
HDAC2-216    .....................................MKPHRIRMTHNLLLNYGLYRKMEIYRPHKATAEEMTKYHSDEYIKFLRSIRPDNMSEYSKQMQRFNVGEDCPV   73
```

```
logo
VORINOSTAT   .....................................HH.......GF.........................D.H..................................    9
PF00850.4    FDGLFEFCQLSTG.................................................................................................   85
PF00850.9    FDGLFEFCQLSTGGSVAGAVKLNRQQTDMAVNWAGGLHHAKKSEASGFCYVNDIVLAILELLKYHQRVLYIDIDIHHGDGVEEAFYTTDRVMTVSFHKYGEYFPGTGDLR  185
PF00850.6    ..............................................................................................................   52
PF00850.3    ..............................................................................................................   65
PF00850.2    FDGLFEFCQLSTGGSVAGAVKLNRQQ....................................................................................   98
PF00850      FDGLFEFCQLSTGGSVAGAVKLNRQQTDMAVNWAGGLHHAKKSEASGFCYVNDIVLAILELLKYHQRVLYIDIDIHHGDGVEEAFYTTDRVMTVSFHKYGEYFPGTGDLR  182
PF00850.1    FDGLFEFCQLSTGGSVAGAVKLNRQQTDMAVNWAGGLHHAKKSEASGFCYVNDIVLAILELLK...............................................  138
PF00850.8    FDGLFEFCQLSTGGSVAGAVKLNRQ.....................................................................................   97
PF00850.7    ..............................................................................................................   56
PF00850.5    ..............................................................................................................   62
HDAC2-206    FDGLFEFCQLSTGGSVAGAVKLNRQQTDMAVNWAGGLHHAKKSEASGFCYVNDIVLAILELLKYHQRVLYIDIDIHHGDGVEEAFYTTDRVMTVSFHKYGEYFPGTGDLR  183
HDAC2-202    FDGLFEFCQLSTGGSVAGAVKLNRQQTDMAVNWAGGLHHAKKSEASGFCYVNDIVLAILELLKYVNLY..........................................  161
HDAC2-217    ..............................................................................................................   52
HDAC2-203    ..............................................................................................................   71
HDAC2-201    FDGLFEFCQLSTGGSVAGAVKLNRQQTDMAVNWAGGLHHAKKSEASGFCYVNDIVLAILELLKYHQRVLYIDIDIHHGDGVEEAFYTTDRVMTVSFHKYGEYFPGTGDLR  183
HDAC2-210    ..............................................................................................................  109
HDAC2-205    FDGLFEFCQLSTGGSVAGAVKLNRQQTDMAVNWAGGLHHAKKSEASGFCYVNDIVLAILELLKYHQRVLYIDIDIHHGDGVEEAFYTTDRVMTVSFHKYGEYFPGTGDLR  213
HDAC2-209    ..............................................................................................................   60
HDAC2-213    FDGLFEFCQLSTG.................................................................................................   86
HDAC2-212    FDGLFEFCQLSTGGSVAGAVKLNRQQT...................................................................................  100
HDAC2-214    ..............................................................................................................   65
HDAC2-216    FDGLFEFCQLSTGGSVAGAVKLNRQQTDMA................................................................................  103
```

```
logo
              DIGAGKGKYYAVNFPMRDGIDDESYGQIFKPIISKVMEMYQPSAVVLQCGADSLSGDRLGCFNLTVKGHAKCVEVVKTFNLPLLMLGGGGYTIRNVARCWTYETAVALDC
VORINOSTAT    .............F...........................................................D....................................   11
PF00850.4     ..............................................................................................................   85
PF00850.9     DIGAGKGKYYAVNFPMRDGIDDESYGQIFKPIISKVMEMYQPSAVVLQCGADSLSGDRLGCFNLTVKGHAKCVEVVKTFNLPLLMLGGGGYTIRNVARCWTYETA.....  290
PF00850.6     ..............................................................................................................   52
PF00850.3     ..............................................................................................................   65
PF00850.2     ..............................................................................................................   98
PF00850       DIGAGKGKYYAVNFPMRDGIDDESYGQIFKPIISKVMEMYQPSAVVLQCGADSLSGDRLGCFNLTVKGHAKCVEVVKTFNLPLLMLGGGGYTIRNVARCWTYETA.....  287
PF00850.1     ..............................................................................................................  138
PF00850.8     ..............................................................................................................   97
PF00850.7     ..............................................................................................................   56
PF00850.5     ..............................................................................................................   62
HDAC2-206     DIGAGKGKYYAVNFPMRDGIDDESYGQIFKPIISKVMEMYQPSAVVLQCGADSLSGDRLGCFNLTVKGHAKCVEVVKTFNLPLLMLGGGGYTIRNVARCWTYETAVALDC  293
HDAC2-202     ..............................................................................................................  161
HDAC2-217     ..............................................................................................................   52
HDAC2-203     ..............................................................................................................   71
HDAC2-201     DIGAGKGKYYAVNFPMRDGIDDESYGQIFKPIISKVMEMYQPSAVVLQCGADSLSGDRLGCFNLTVKGHAKCVEVVKTFNLPLLMLGGGGYTIRNVARCWTYETAVALDC  293
HDAC2-210     ..............................................................................................................  109
HDAC2-205     DIGAGKGKYYAVNFPMRDGIDDESYGQIFKPIISKVMEMYQPSAVVLQCGADSLSGDRLGCFNLTVKGHAKCVEVVKTFNLPLLMLGGGGYTIRNVARCWTYETAVALDC  323
HDAC2-209     ..............................................................................................................   60
HDAC2-213     ..............................................................................................................   86
HDAC2-212     ..............................................................................................................  100
HDAC2-214     ..............................................................................................................   65
HDAC2-216     ..............................................................................................................  103

logo
              EIPNELPYNDYFEYFGPDFKLHISPSNMTNQNTPEYMEKIKQRLFENLRMLPHAPGVQMQAIPEDAVHEDSGDEDGEDPDKRISIRASDKRIACDEEFSDSEDEGEGGRR
VORINOSTAT    ...................................Y..........................................................................   12
PF00850.4     ..............................................................................................................   85
PF00850.9     ..............................................................................................................  290
PF00850.6     ..............................................................................................................   52
PF00850.3     ..............................................................................................................   65
PF00850.2     ..............................................................................................................   98
PF00850       ..............................................................................................................  287
PF00850.1     ..............................................................................................................  138
PF00850.8     ..............................................................................................................   97
PF00850.7     ..............................................................................................................   56
PF00850.5     ..............................................................................................................   62
HDAC2-206     EIPNELPYNDYFEYFGPDFKLHISPSNMTNQNTPEYMEKIKQRLFENLRMLPHAPGVQMQAIPEDAVHEDSGDEDGEDPDKRISIRASDKRIACDEEFSDSEDEGEGGRR  403
HDAC2-202     ..............................................................................................................  161
HDAC2-217     ..............................................................................................................   52
HDAC2-203     ..............................................................................................................   71
HDAC2-201     EIPNELPYNDYFEYFGPDFKLHISPSNMTNQNTPEYMEKIKQRLFENLRMLPHAPGVQMQAIPEDAVHEDSGDEDGEDPDKRISIRASDKRIACDEEFSDSEDEGEGGRR  403
HDAC2-210     ..............................................................................................................  109
HDAC2-205     EIPNELPYNDYFEYFGPDFKLHISPSNMTNQNTPEYMEKIKQRLFENLRMLPHAPGVQMQAIPEDAVHEDSGDEDGEDPDKRISIRASDKRIACDEEFSDSEDEGEGGRR  433
HDAC2-209     ..............................................................................................................   60
HDAC2-213     ..............................................................................................................   86
HDAC2-212     ..............................................................................................................  100
HDAC2-214     ..............................................................................................................   65
HDAC2-216     ..............................................................................................................  103
```

logo

```
             NVADHKKGAKKARIEEDKKETEDKKTDVKEEDKSKDNSGEKTDTKGTKSEQLSNP
VORINOSTAT   .......................................................   12
PF00850.4    .......................................................   85
PF00850.9    .......................................................  290
PF00850.6    .......................................................   52
PF00850.3    .......................................................   65
PF00850.2    .......................................................   98
PF00850      .......................................................  287
PF00850.1    .......................................................  138
PF00850.8    .......................................................   97
PF00850.7    .......................................................   56
PF00850.5    .......................................................   62
HDAC2-206    NVADHKKGAKKARIEEDKKETEDKKTDVKEEDKSKDNSGEKTDTKGTKSEQLSNP  458
HDAC2-202    .......................................................  161
HDAC2-217    .......................................................   52
HDAC2-203    .......................................................   71
HDAC2-201    NVADHKKGAKKARIEEDKKETEDKKTDVKEEDKSKDNSGEKTDTKGTKSEQLSNP  458
HDAC2-210    .......................................................  109
HDAC2-205    NVADHKKGAKKARIEEDKKETEDKKTDVKEEDKSKDNSGEKTDTKGTKSEQLSNP  488
HDAC2-209    .......................................................   60
HDAC2-213    .......................................................   86
HDAC2-212    .......................................................  100
HDAC2-214    .......................................................   65
HDAC2-216    .......................................................  103
```

X non conserved
X similar
X $\geq$ 0% conserved
X $\geq$ 50% conserved

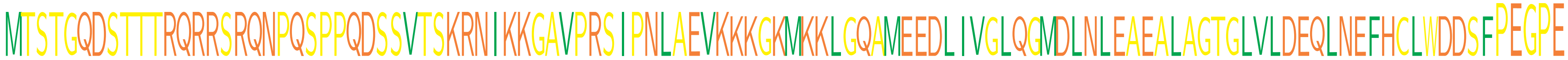

```
logo
TRICHOSTATIN  ..............................................................................................................    0
PF00850       .........................................................................................................PEGPE    5
PF02148       ..............................................................................................................    0
PF00850.2     .........................................................................................................PEGPE    5
PF00850.1     .........................................................................................................PEGPE    5
HDAC6-203     MTSTGQDSTTTRQRRSRQNPQSPPQDSSVTSKRNIKKGAVPRSIPNLAEVKKKGKMKKLGQAMEEDLIVGLQGMDLNLEAEALAGTGLVLDEQLNEFHCLWDDSFPEGPE  110
HDAC6-205     MTSTGQDSTTTRQRRSRQNPQSPPQDSSVTSKRNIKKGAVPRSIPNLAEVKKKGKMKKLGQAMEEDLIVGLQGMDLNLEAEALAGTGLVLDEQLNEFHCLWDDSFPEGPE  110
HDAC6-201     MTSTGQDSTTTRQRRSRQNPQSPPQDSSVTSKRNIKKGAVPRSIPNLAEVKKKGKMKKLGQAMEEDLIVGLQGMDLNLEAEALAGTGLVLDEQLNEFHCLWDDSFPEGPE  110
HDAC6-204     MTSTGQDSTTTRQRRSRQNPQSPPQDSSVTSKRNIKKGAVPRSIPNLAEVKKKGKMKKLGQAMEEDLIVGLQGMDLNLEAEALAGTGLVLDEQLNEFHCLWDDSFPEGPE  110
HDAC6-202     MTSTGQDSTTTRQRRSRQNPQSPPQDSSVTSKRNIKKGAVPRSIPNLAEVKKKGKMKKLGQAMEEDLIVGLQGMDLNLEAEALAGTGLVLDEQLNEFHCLWDDSFPEGPE  110
HDAC6-206     MTSTGQDSTTTRQRRSRQNPQSPPQDSSVTSKRNIKKGAVPRSIPNLAEVKKKGKMKKLGQAMEEDLIVGLQGMDLNLEAEALAGTGLVLDEQLNEFHCLWDDSFPEGPE  110
HDAC6-211     MTSTGQDSTTTRQRRSRQNPQSPPQDSSVTSKRNIKKGAVPRSIPNLAEVKKKGKMKKLGQAMEEDLIVGLQGMDLNLEAEALAGTGLVLDEQLNEFHCLWDDSFPEGPE  110
HDAC6-207     ..............................................................................................................    0
HDAC6-209     MTSTGQDSTTTRQRRSRQNPQSPPQDSSVTSKRNIKKGAVPRSIPNLAEVKKKGKMKKLGQAMEEDLIVGLQGMDLNLEAEALAGTGLVLDEQLNEFHCLWDDSFPEGPE  110
HDAC6-210     MTSTGQDSTTTRQRRSRQNPQSPPQDSSVTSKRNIKKGAVPRSIPNLAEVKKKGKMKKLGQAMEEDLIVGLQGMDLNLEAEALAGTGLVLDEQLNEFHCLWDDSFPEGPE  110
HDAC6-208     MTSTGQDSTTTRQRRSRQNPQSPPQDSSVTSKRNIKKGAVPRSIPNLAEVK...........................................................   51
```

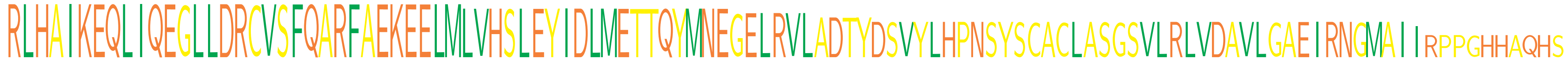

```
logo
TRICHOSTATIN  ..............................................................................................................    0
PF00850       RLHAIKEQLIQEGLLDRCVSFQARFAEKEELMLVHSLEYIDLMETTQYMNEGELRVLADTYDSVYLHPNSYSCACLASGSVLRLVDAVLGAEIRNGMAIIRPPGHHAQHS  115
PF02148       ..............................................................................................................    0
PF00850.2     RLHAIKEQLIQEGLLDRCVSFQARFAEKEELMLVHSLEYIDLMETTQYMNEGELRVLADTYDSVYLHPNSYSCACLASGSVLRLVDAVLGAEIRNGMAIIRPPGHHAQHS  115
PF00850.1     RLHAIKEQLIQEGLLDRCVSFQARFAEKEELMLVHSLEYIDLMETTQYMNEGELRVLADTYDSVYLHPNSYSCACLASGSVLRLVDAVLGAEIRNGMAI...........  104
HDAC6-203     RLHAIKEQLIQEGLLDRCVSFQARFAEKEELMLVHSLEYIDLMETTQYMNEGELRVLADTYDSVYLHPNSYSCACLASGSVLRLVDAVLGAEIRNGMAIIRPPGHHAQHS  220
HDAC6-205     RLHAIKEQLIQEGLLDRCVSFQARFAEKEELM..............................................................................  142
HDAC6-201     RLHAIKEQLIQEGLLDRCVSFQARFAEKEELMLVHSLEYIDLMETTQYMNEGELRVLADTYDSVYLHPNSYSCACLASGSVLRLVDAVLGAEIRNGMAIIRPPGHHAQHS  220
HDAC6-204     RLHAIKEQLIQEGLLDRCVSFQARFAEKEELMLVHSLEYIDLMETTQYMNEGELRVLADTYDSVYLHPNSYSCACLASGSVLRLVDAVLGAEIRNGMAII..........  210
HDAC6-202     RLHAIKEQLIQEGLLDRCVSFQARFAEKEELMLVHSLEYIDLMETTQYMNEGELRVLADTYDSVYLHPNSYSCACLASGSVLRLVDAVLGAEIRNGMAII..........  210
HDAC6-206     RLHAIKEQLIQEGLLDRCVSFQARFAEKEELMLVHSLEYIDLMETTQYMNEGELRVLADTYDSVYLHPNSYSCACLASGSVLRLVDAVLGAEIRNGMAIIRPPGHHAQHS  220
HDAC6-211     RLHAIKEQLIQEGLLDRCVSFQARFAEKEELMLVHSLEYIDLMETTQYMNEGELRVL.....................................................  167
HDAC6-207     ..............................................................................................................    0
HDAC6-209     RLHAIKEQLIQEGLLDRCVSFQARFAEKEELMLVHSLEYIDLMETTQYMNEGELRVLADTY.................................................  171
HDAC6-210     RLHAIKEQLIQEGLLDRCVSFQARFAEKEELMLVHSLEYIDLMETTQYMNE...........................................................  161
HDAC6-208     ..............................................................................................................   51
```

```
logo
                LMDGYCMFNHVAVAARYAQQKHRIRRVLIVDWDVHHGQGTQFTFDQDPSVLYFSIHRYEQGRFWPHLKASNWSTTGFGQGQGYTINVPWNQVGMRDADYIAAFLHVLLPV
TRICHOSTATIN    ..............................................................................................................    0
PF00850         LMDGYCMFNHVAVAARYAQQKHRIRRVLIVDWDVHHGQGTQFTFDQDPSVLYFSIHRYEQGRFWPHLKASNWSTTGFGQGQGYTINVPWNQVGMRDADYIAAFLHVLLPV  225
PF02148         ..............................................................................................................    0
PF00850.2       LMDGYCMFNHVAVAARYAQQKHRIRRVLIVDWDVHHGQGTQFTFDQDPR.............................................................  164
PF00850.1       ..............................................................................................................  104
HDAC6-203       LMDGYCMFNHVAVAARYAQQKHRIRRVLIVDWDVHHGQGTQFTFDQDPSVLYFSIHRYEQGRFWPHLKASNWSTTGFGQGQGYTINVPWNQVGMRDADYIAAFLHVLLPV  330
HDAC6-205       ..............................................................................................................  142
HDAC6-201       LMDGYCMFNHVAVAARYAQQKHRIRRVLIVDWDVHHGQGTQFTFDQDPSVLYFSIHRYEQGRFWPHLKASNWSTTGFGQGQGYTINVPWNQVGMRDADYIAAFLHVLLPV  330
HDAC6-204       ..............................................................................................................  210
HDAC6-202       ..............................................................................................................  210
HDAC6-206       LMDGYCMFNHVAVAARYAQQKHRIRRVLIVDWDVHHGQGTQFTFDQDPR.............................................................  269
HDAC6-211       ..............................................................................................................  167
HDAC6-207       ..............................................................................................................    0
HDAC6-209       ..............................................................................................................  171
HDAC6-210       ..............................................................................................................  161
HDAC6-208       ..............................................................................................................   51

logo
                ALEFQPQLVLVAAGFDALQGDPKGEMAATPAGFAQLTHLLMGLAGGKLILSLEGGYNLRALAEGVSASLHTLLGDPCPMLESPGAPCRSAQASVSCALEALEPFWEVLVR
TRICHOSTATIN    ..............................................................................................................    0
PF00850         ALEFQPQLVLVAAGFDALQGDPKGEMAATPAGFAQLTHLLMGLAGGKLILSLEGGYNLRALAEGVSASLHT.......................................  296
PF02148         ..............................................................................................................    0
PF00850.2       ..............................................................................................................  164
PF00850.1       ..............................................................................................................  104
HDAC6-203       ALEFQPQLVLVAAGFDALQGDPKGEMAATPAGFAQLTHLLMGLAGGKLILSLEGGYNLRALAEGVSASLHTLLGDPCPMLESPGAPCRSAQASVSCALEALEPFWEVLVR  440
HDAC6-205       ..............................................................................................................  142
HDAC6-201       ALEFQPQLVLVAAGFDALQGDPKGEMAATPAGFAQLTHLLMGLAGGKLILSLEGGYNLRALAEGVSASLHTLLGDPCPMLESPGAPCRSAQASVSCALEALEPFWEVLVR  440
HDAC6-204       ..............................................................................................................  210
HDAC6-202       ..............................................................................................................  210
HDAC6-206       ..............................................................................................................  269
HDAC6-211       ..............................................................................................................  167
HDAC6-207       ..............................................................................................................    0
HDAC6-209       ..............................................................................................................  171
HDAC6-210       ..............................................................................................................  161
HDAC6-208       ..............................................................................................................   51
```

```
logo
             STETVERDNMEEDNVEESEEEGPWEPPVLPILTWPVLQSRTGLVYDQNMMNHCNLWDSHHPEVPQRILRIMCRLEELGLAGRCLTLTPRPATEAELLTCHSAEYVGHLRA
TRICHOSTATIN ...........................................................HP.................................................     2
PF00850      ...........................................................HPEVPQRILRIMCRLEELGLAGRCLTLTPRPATEAELLTCHSAEYVGHLRA   347
PF02148      ..............................................................................................................     0
PF00850.2    ..............................................................................................................   164
PF00850.1    ..............................................................................................................   104
HDAC6-203    STETVERDNMEEDNVEESEEEGPWEPPVLPILTWPVLQSRTGLVYDQNMMNHCNLWDSHHPEVPQRILRIMCRLEELGLAGRCLTLTPRPATEAELLTCHSAEYVGHLRA   550
HDAC6-205    ..............................................................................................................   142
HDAC6-201    STETVERDNMEEDNVEESEEEGPWEPPVLPILTWPVLQSRTGLVYDQNMMNHCNLWDSHHPEVPQRILRIMCRLEELGLAGRCLTLTPRPATEAELLTCHSAEYVGHLRA   550
HDAC6-204    ..............................................................................................................   210
HDAC6-202    ..............................................................................................................   210
HDAC6-206    ..............................................................................................................   269
HDAC6-211    ..............................................................................................................   167
HDAC6-207    ..............................................................................................................     0
HDAC6-209    ..............................................................................................................   171
HDAC6-210    ..............................................................................................................   161
HDAC6-208    ..............................................................................................................    51

logo
             TEKMKTRELHRESSNFDSIYICPSTFACAQLATGAACRLVEAVLSGEVLNGAAVVRPPGHHAEQDAACGFCFFNSVAVAARHAQTISGHALRILIVDWDVHHGNGTQHMF
TRICHOSTATIN ...........................................................HH.......GF............................D.H.........     8
PF00850      TEKMKTRELHRESSNFDSIYICPSTFACAQLATGAACRLVEAVLSGEVLNGAAVVRPPGHHAEQDAACGFCFFNSVAVAARHAQTISGHALRILIVDWDVHHGNGTQHMF   457
PF02148      ..............................................................................................................     0
PF00850.2    ..............................................................................................................   164
PF00850.1    ..............................................................................................................   104
HDAC6-203    TEKMKTRELHRESSNFDSIYICPSTFACAQLATGAACRLVEAVLSGEVLNGAAVVRPPGHHAEQDAACGFCFFNSVAVAARHAQTISGHALRILIVDWDVHHGNGTQHMF   660
HDAC6-205    ..............................................................................................................   142
HDAC6-201    TEKMKTRELHRESSNFDSIYICPSTFACAQLATGAACRLVEAVLSGEVLNGAAVVRPPGHHAEQDAACGFCFFNSVAVAARHAQTISGHALRILIVDWDVHHGNGTQHMF   660
HDAC6-204    ..............................................................................................................   210
HDAC6-202    ..............................................................................................................   210
HDAC6-206    ..............................................................................................................   269
HDAC6-211    ..............................................................................................................   167
HDAC6-207    ..............................................................................................................     0
HDAC6-209    ..............................................................................................................   171
HDAC6-210    ..............................................................................................................   161
HDAC6-208    ..............................................................................................................    51
```

```
logo
              EDDPSVLYVSLHRYDHGTFFPMGDEGASSQIGRAAGTGFTVNVAWNGPRMGDADYLAAWHRLVLPIAYEFNPELVLVSAGFDAARGDPLGGCQVSPEGYAHLTHLLMGLA
TRICHOSTATIN  ...................F.............................................................D......L.....................   11
PF00850       EDDPSVLYVSLHRYDHGTFFPMGDEGASSQIGRAAGTGFTVNVAWNGPRMGDADYLAAWHRLVLPIAYEFNPELVLVSAGFDAARGDPLGGCQVSPEGYAHLTHLLMGLA  567
PF02148       ..............................................................................................................    0
PF00850.2     ..............................................................................................................  164
PF00850.1     ..............................................................................................................  104
HDAC6-203     EDDPSVLYVSLHRYDHGTFFPMGDEGASSQIGRAAGTGFTVNVAWNGPRMGDADYLAAWHRLVLPIAYEFNPELVLVSAGFDAARGDPLGGCQVSPEGYAHLTHLLMGLA  770
HDAC6-205     ..............................................................................................................  142
HDAC6-201     EDDPSVLYVSLHRYDHGTFFPMGDEGASSQIGRAAGTGFTVNVAWNGPRMGDADYLAAWHRLVLPIAYEFNPELVLVSAGFDAARGDPLGGCQVSPEGYAHLTHLLMGLA  770
HDAC6-204     ..............................................................................................................  210
HDAC6-202     ..............................................................................................................  210
HDAC6-206     ..............................................................................................................  269
HDAC6-211     ..............................................................................................................  167
HDAC6-207     ..............................................................................................................    0
HDAC6-209     ..............................................................................................................  171
HDAC6-210     ..............................................................................................................  161
HDAC6-208     ..............................................................................................................   51

logo
              SGRIILILEGGYNLTSISESMAACTRSLLGDPPPLLTLPRPPLSGALASITETIQVHRRYWRSLRVMKVEDREGPSSSKLVTKKAPQPAKPRLAERMTTREKKVLEAGMG
TRICHOSTATIN  ...........................................................Y..................................................   12
PF00850       SGRIILILEGGYNLTSISESMAACTRS...................................................................................  594
PF02148       ..............................................................................................................    0
PF00850.2     ..............................................................................................................  164
PF00850.1     ..............................................................................................................  104
HDAC6-203     SGRIILILEGGYNLTSISESMAACTRSLLGDPPPLLTLPRPPLSGALASITETIQVHRRYWRSLRVMKVEDREGPSSSKLVTKKAPQPAKPRLAERMTTREKKVLEAGMG  880
HDAC6-205     ..............................................................................................................  142
HDAC6-201     SGRIILILEGGYNLTSISESMAACTRSLLGDPPPLLTLPRPPLSGALASITETIQVHRRYWRSLRVMKVEDREGPSSSKLVTKKAPQPAKPRLAERMTTREKKVLEAGMG  880
HDAC6-204     ..............................................................................................................  210
HDAC6-202     ..............................................................................................................  210
HDAC6-206     ..............................................................................................................  269
HDAC6-211     ..............................................................................................................  167
HDAC6-207     ..............................................................................................................    0
HDAC6-209     ..............................................................................................................  171
HDAC6-210     ..............................................................................................................  161
HDAC6-208     ..............................................................................................................   51
```

```
logo

                KVTSASFGEESTPGQTNSETAVVALTQDQPSEAATGGATLAQTISEAAIGGAMLGQTTSEEAVGGATPDQTTSEETVGGAILDQTTSEDAVGGATLGQTTSEEAVGGATL
TRICHOSTATIN    ..............................................................................................................   12
PF00850         ..............................................................................................................  594
PF02148         ..............................................................................................................    0
PF00850.2       ..............................................................................................................  164
PF00850.1       ..............................................................................................................  104
HDAC6-203       KVTSASFGEESTPGQTNSETAVVALTQDQPSEAATGGATLAQTISEAAIGGAMLGQTTSEEAVGGATPDQTTSEETVGGAILDQTTSEDAVGGATLGQTTSEEAVGGATL  990
HDAC6-205       ..............................................................................................................  142
HDAC6-201       KVTSASFGEESTPGQTNSETAVVALTQDQPSEAATGGATLAQTISEAAIGGAMLGQTTSEEAVGGATPDQTTSEETVGGAILDQTTSEDAVGGATLGQTTSEEAVGGATL  990
HDAC6-204       ..............................................................................................................  210
HDAC6-202       ..............................................................................................................  210
HDAC6-206       ..............................................................................................................  269
HDAC6-211       ..............................................................................................................  167
HDAC6-207       ..............................................................................................................    0
HDAC6-209       ..............................................................................................................  171
HDAC6-210       ..............................................................................................................  161
HDAC6-208       ..............................................................................................................   51
```

```
logo

                AQTTSEAAMEGATLDQTTSEEAPGGTELIQTPLASSTDHQTPPTSPVQGTTPQISPSTLIGSLRTLELGSESQGASESQAPGEENLLGEAAGGQDMADSMLMQGSRGLTD
TRICHOSTATIN    ..............................................................................................................   12
PF00850         ..............................................................................................................  594
PF02148         ..............................................................................................................    0
PF00850.2       ..............................................................................................................  164
PF00850.1       ..............................................................................................................  104
HDAC6-203       AQTTSEAAMEGATLDQTTSEEAPGGTELIQTPLASSTDHQTPPTSPVQGTTPQISPSTLIGSLRTLELGSESQGASESQAPGEENLLGEAAGGQDMADSMLMQGSRGLTD 1100
HDAC6-205       ..............................................................................................................  142
HDAC6-201       AQTTSEAAMEGATLDQTTSEEAPGGTELIQTPLASSTDHQTPPTSPVQGTTPQISPSTLIGSLRTLELGSESQGASESQAPGEENLLGEAAGGQDMADSMLMQGSRGLTD 1100
HDAC6-204       ..............................................................................................................  210
HDAC6-202       ..............................................................................................................  210
HDAC6-206       ..............................................................................................................  269
HDAC6-211       ..............................................................................................................  167
HDAC6-207       .................................................XTPQISPSTLIGSLRTLELGSESQGASESQAPGEENLLGEAAGGQDMADSMLMQGSRGLTD   61
HDAC6-209       ..............................................................................................................  171
HDAC6-210       ..............................................................................................................  161
HDAC6-208       ..............................................................................................................   51
```

```
logo
              QAIFYAVTPLPWCPHLVAVCPIPAAGLDVTQPCGDCGTIQENWVCLSCYQVYCGRYINGHMLQHHGNSGHPLVLSYIDLSAWCYYCQAYVHHQALLDVKNIAHQNKFGED
TRICHOSTATIN  ..............................................................................................................    12
PF00850       ..............................................................................................................   594
PF02148       ................................CGDCGTIQENWVCLSCYQVYCGRYINGHMLQHHGNSGHPLVLSYIDLSAWCYYCQAYVHHQ.................    61
PF00850.2     ..............................................................................................................   164
PF00850.1     ..............................................................................................................   104
HDAC6-203     QAIFYAVTPLPWCPHLVAVCPIPAAGLDVTQPCGDCGTIQENWVCLSCYQVYCGRYINGHMLQHHGNSGHPLVLSYIDLSAWCYYCQAYVHHQALLDVKNIAHQNKFGED  1210
HDAC6-205     ..............................................................................................................   142
HDAC6-201     QAIFYAVTPLPWCPHLVAVCPIPAAGLDVTQPCGDCGTIQENWVCLSCYQVYCGRYINGHMLQHHGNSGHPLVLSYIDLSAWCYYCQAYVHHQALLDVKNIAHQNKFGED  1210
HDAC6-204     ..............................................................................................................   210
HDAC6-202     ..............................................................................................................   210
HDAC6-206     ..............................................................................................................   269
HDAC6-211     ..............................................................................................................   167
HDAC6-207     QA............................................................................................LLDVKNIAHQNKFGED    79
HDAC6-209     ..............................................................................................................   171
HDAC6-210     ..............................................................................................................   161
HDAC6-208     ..............................................................................................................    51


logo
              MPHPH
TRICHOSTATIN  .....    12
PF00850       .....   594
PF02148       .....    61
PF00850.2     .....   164
PF00850.1     .....   104
HDAC6-203     MPHPH  1215
HDAC6-205     .....   142
HDAC6-201     MPHPH  1215
HDAC6-204     .....   210
HDAC6-202     .....   210
HDAC6-206     .....   269
HDAC6-211     .....   167
HDAC6-207     MPHPH    84
HDAC6-209     .....   171
HDAC6-210     .....   161
HDAC6-208     .....    51
```

X non conserved

X similar

X ≥ 0% conserved

X ≥ 50% conserved

logo

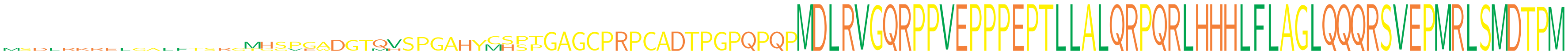

```
TRICHOSTATIN  ..............................................................................................................    0
PF00850.1     ..............................................................................................................    0
PF00850       ..............................................................................................................    0
HDAC7-224     ..............................................................................................................    0
HDAC7-214     ........................................................MDLRVGQRPPVEPPPEPTLLALQRPQRLHHHLFLAGLQQQRSVEPMRLSMD...   51
HDAC7-208     ..........................MHSPGA..............................................................................    6
HDAC7-203     MSDLRKRELGALFTSRGTGGVEWDGTQVSPGAHYCSPTGAGCPRPCADTPGPQPQPMDLRVGQRPPVEPPPEPTLLALQRPQRLHHHLFLAGLQQQRSVEPMRLSMDTPM  110
HDAC7-205     ..................................MHSPGAGCPRPCADTPGPQPQPMDLRVGQRPPVEPPPEPTLLALQRPQRLHHHLFLAGLQQQRSVEPMRLSMDTPM   76
HDAC7-226     ..............................................................................................................    0
HDAC7-215     ........................................................MDLRVGQRPPVEPPPEPTLLALQRPQRLHHHLFLAGLQQQRSVEPMRLSMDTPM   54
HDAC7-227     ..............................................................................................................    0
HDAC7-206     ........................................................MDLRVGQRPPVEPPPEPTLLALQRPQRLHHHLFLAGLQQQRSVEPMRLSMDTPM   54
HDAC7-204     ........................................................MDLRVGQRPPVEPPPEPTLLALQRPQRLHHHLFLAGLQQQRSVEPMRLSMDTPM   54
HDAC7-207     ........................................................MDLRVGQRPPVEPPPEPTLLALQRPQRLHHHLFLAGLQQQRSVEPMRLSMDTPM   54
HDAC7-212     ........................................................MDLRVGQRPPVEPPPEPTLLALQRPQRLHHHLFLAGLQQQRSVEPMRLSMDTPM   54
HDAC7-221     ........................................................MDLRVGQRPPVEPPPEPTLLALQRPQRLHHHLFLAGLQQQRSVEPMRLSMDTPM   54
HDAC7-210     .................MHSPGADGTQVSPGAHYCSPTGAGCPRPCADTPGPQPQPMDLRVGQRPPVEPPPEPTLLALQRPQRLHHHLFLAGLQQQRSVEPMRLSMDTPM   93
HDAC7-209     ........................................................MDLRVGQRPPVEPPPEPTLLALQRPQRLHHHLFLAGLQQQRSVEPMRLSMDTPM   54
HDAC7-230     ..................................MHSPGAGCPRPCADTPGPQPQPMDLRVGQRPPVEPPPEPTLLALQRPQRLHHHLFLAGLQQQRSVEPMRLSMDTPM   76
HDAC7-223     ..............................................................................................................    0
HDAC7-211     ........................................................MDLRVGQRPPVEPPPEPTLLALQRPQRLHHHLFLAGLQQQRSVEPMRLSMDTPM   54
HDAC7-202     .................MHSPGADGTQVSPGAHYCSPTGAGCPRPCADTPGPQPQPMDLRVGQRPPVEPPPEPTLLALQRPQRLHHHLFLAGLQQQRSVEPMRLSMDTPM   93
HDAC7-228     ..............................................................................................................    0
HDAC7-216     ........................................................MDLRVGQRPPVEPPPEPTLLALQRPQRLHHHLFLAGLQQQRSVEPMRLSMDTPM   54
HDAC7-201     .................MHSPGADGTQVSPGAHYCSPTGAGCPRPCADTPGPQPQPMDLRVGQRPPVEPPPEPTLLALQRPQRLHHHLFLAGLQQQRSVEPMRLSMDTPM   93
HDAC7-213     ..................................MHSPGAGCPRPCADTPGPQPQPMDLRVGQRPPVEPPPEPTLLALQRPQRLHHHLFLAGLQQQRSVEPMRLSMDTPM   76
```

logo

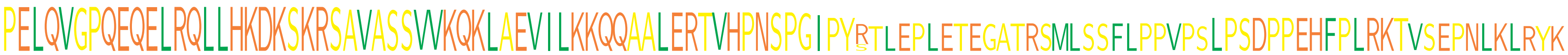

```
TRICHOSTATIN  ..............................................................................................................   0
PF00850.1     ..............................................................................................................   0
PF00850       ..............................................................................................................   0
HDAC7-224     ..............................................................................................................   0
HDAC7-214     ..............................................................................................................  51
HDAC7-208     ..............................................................................................................   6
HDAC7-203     PELQVGPQEQELRQLLHKDKSKRSAVASSVVKQKLAEVILKKQQAALERTVHPNSPGIPYRTLEPLETEGATRSMLSSFLPPVPSLPSDPPEHFPLRKTVSEPNLKLRYK 220
HDAC7-205     PELQVGPQEQELRQLLHKDKSKRSAVASSVVKQKLAEVILKKQQAALERTVHPNSPG..................................................... 133
HDAC7-226     ..............................................................................................................   0
HDAC7-215     PELQVGPQEQELRQLLHKDKSKRSAVASSVVKQKLAEVILKKQQAALERTVHPNSPGIPYRTLEPLETEGATRSMLSSFLPPVPSLPSDPPEHFPLRKT........... 153
HDAC7-227     ..............................................................................................................   0
HDAC7-206     PELQVGPQEQELRQLLHKDKSKRSAVASSVVKQKLAEVILKKQQAALERTVHPNSPGIPYRTLEPLETEGATRSMLSSFLPPVP.......................... 138
HDAC7-204     PELQVGPQEQELRQLLHKDKSKRS......................................................................................  78
HDAC7-207     PELQVGPQEQELRQLLHKDKSKRSAVASSVVKQKLAEVILKKQQAALERTVHPNSPGIPYS........................LPSDPPEHFPLRKTVSEPNLKLRYK 140
HDAC7-212     PELQVGPQEQELRQLLHKDKSKRSAVASSVVKQKLAEVILKKQQAALERTVHPNSPGIPYRTLEPLETEGATRSMLSSFLPPVPSLPSDPPEHFPLRKT........... 153
HDAC7-221     PELQVGPQEQELRQLLHKDKSKRSAVASSVVKQKLAEVILKKQQAALERTVHPNSPGIPYRTLEPLETEGATRSMLSSFLPPVPSLPSDPPEHFPLRKTVSEPNLKLRYK 164
HDAC7-210     PELQVGPQEQELRQLLHKDKSKRSAVASSVVKQKLAEVILKKQQAALERTVHPNSPGIPYS........................LPSDPPEHFPLRKTVSEPNLKLRYK 179
HDAC7-209     PELQVGPQEQELRQLLHKDKSKRSAVASSVVKQKLAEVILKKQQAALERTVHPNSPGIPYRTLEPLETEGATRSMLSSFLPPVPSLPSDPPEHFPLRKTVSEPNLKLRYK 164
HDAC7-230     PELQVGPQEQELRQLLHKDKSKRSAVASSVVKQKLAEVILKKQQAALERTVHPNSPGIPYRTLEPLETEGATRSMLSSFLPPVPSLPSDPPEHFPLRKTVSEPNLKLRYK 186
HDAC7-223     ...............................................................EPLETEGATRSMLSSFLPPVPSLPSDPPEHFPLRKTVSEPNLKLRYK  47
HDAC7-211     PELQVGPQEQELRQLLHKDKSKRSAVASSVVKQK............................................................................  88
HDAC7-202     PELQVGPQEQELRQLLHKDKSKRSAVASSVVKQKLAEVILKKQQAALERTVHPNSPGIPYRTLEPLETEGATRSMLSSFLPPVPSLPSDPPEHFPLRKTVSEPNLKLRYK 203
HDAC7-228     ..............................................................................................................   0
HDAC7-216     PELQVGPQEQELRQLLHKDKSKRSAVASSVVKQKLAEVILKKQQAALERTVHPNSPGI.................................................... 112
HDAC7-201     PELQVGPQEQELRQLLHKDKSKRSAVASSVVKQKLAEVILKKQQAALERTVHPNSPGIPYRTLEPLETEGATRSMLSSFLPPVPSLPSDPPEHFPLRKTVSEPNLKLRYK 203
HDAC7-213     PELQVGPQEQELRQLLHKDKSKRSAVASSVVKQKLAEVILKKQQAALERTVHPNSPGIPYS........................LPSDPPEHFPLRKTVSEPNLKL... 159
```

```
logo
              PKKSLERRKNPLLRKESAPPSLRRRPAETLGDSSPSSSSTPASGCSSPNDSEHGPNPILGSEALLGQRLRLQETSVAPFALPTVSLLPAITLGLPAPARADSDRRTHPTL
TRICHOSTATIN  ..............................................................................................................    0
PF00850.1     ..............................................................................................................    0
PF00850       ..............................................................................................................    0
HDAC7-224     ..............................................................................................................    0
HDAC7-214     ..............................................................................................................   51
HDAC7-208     ..............................................................................................................    6
HDAC7-203     PKKSLERRKNPLLRKESAPPSLRRRPAETLGDSSPSSSSTPASGCSSPNDSEHGPNPILGSEALLGQRLRLQETSVAPFALPTVSLLPAITLGLPAPARADSDRRTHPTL  330
HDAC7-205     ..............................................................................................................  133
HDAC7-226     ..............................................................................................................    0
HDAC7-215     ..............................................................................................................  153
HDAC7-227     ..............................................................................................................    0
HDAC7-206     ..............................................................................................................  138
HDAC7-204     ..............................................................................................................   78
HDAC7-207     P.............................................................................................................  141
HDAC7-212     ..............................................................................................................  153
HDAC7-221     PKKSLERRKNPLLRKESAPPSLRRRPAETLGDSSPSSSSTPASGCSSPNDSEHGPNPILG..................................................  224
HDAC7-210     PKKSLERRKNPLLRKESAPPSLRRRPAETLGDSSPSSSSTPASGCSSPNDSEHGPNPILGSE.....................................ADSDRRTHPTL  252
HDAC7-209     PKKSLERRKNPLLRKESAPPSLRRRPAETLGDSSPSSSSTPASGCSSPNDSEHGPNPILGSEALLGQRLRLQETSVAPFALPTVSLLPAITLGLPAPARADSDRRTHPTL  274
HDAC7-230     PKKSLERRKNPLLRKESAPPSLRRRPAETLGDSSPSSSSTPASGCSSPNDSEHGPNPILGSEALLGQRLRLQETSVAPFALPTVSLLPAITLGLPAPARADSDRRTHPTL  296
HDAC7-223     PKKSLERRKNPLLRKESAPPSLRRRPAETLGDSSPSSSSTPASGCSSPNDSEHGPNPILGSEALLGQRLRLQETSVAPFALPTVSLLPAITLGLPAPARALMPRC.....  152
HDAC7-211     ..............................................................................................................   88
HDAC7-202     PKKSLERRKNPLLRKESAPPSLRRRPAETLGDSSPSSSSTPASGCSSPNDSEHGPNPILGSE.....................................ADSDRRTHPTL  276
HDAC7-228     ..............................................................................................................    0
HDAC7-216     ..............................................................................................................  112
HDAC7-201     PKKSLERRKNPLLRKESAPPSLRRRPAETLGDSSPSSSSTPASGCSSPNDSEHGPNPILGSEALLGQRLRLQETSVAPFALPTVSLLPAITLGLPAPARADSDRRTHPTL  313
HDAC7-213     ..............................................................................................................  159
```

logo

```
             GPRGPILGSPHTPLFLPHGLEPEAGGTLPSRLQPILLLDPSGSHAPLLTVPGLGPLPFHFAQSLMTTERLSGSGLHWPLSRTRSEPLPPSATAPPPPGPMQPRLEQLKTH
TRICHOSTATIN ..............................................................................................................   0
PF00850.1    ..............................................................................................................   0
PF00850      ..............................................................................................................   0
HDAC7-224    ..............................................................................................................   0
HDAC7-214    ..............................................................................................................  51
HDAC7-208    .........................................................................A....................................   7
HDAC7-203    GPRGPILGSPHTPLFLPHGLEPEAGGTLPSRLQPILLLDPSGSHAPLLTVPGLGPLPFHFAQSLMTTERLSGSGLHWPLSRTRSEPLPPSATAPPPPGPMQPRLEQLKTH 440
HDAC7-205    .............................................................................................................. 133
HDAC7-226    ..............................................................................................................   0
HDAC7-215    .............................................................................................................. 153
HDAC7-227    ..............................................................................................................   0
HDAC7-206    .............................................................................................................. 138
HDAC7-204    ..............................................................................................................  78
HDAC7-207    .............................................................................................................. 141
HDAC7-212    .............................................................................................................. 153
HDAC7-221    .............................................................................................................. 224
HDAC7-210    GPRGPILGSPHTPLFLPHGLEP........................................................................................ 274
HDAC7-209    GPRGPILGSPHTPLFLPHGLEPEAGGTLPSRLQPILLLDPSGSHAPLLTVPGLGPLPFHFAQSLMTTERLSGSGLHWPLSRTRSEPLPPSATAPPPPGPMQPRLEQLKTH 384
HDAC7-230    GPRGPILGSPHTPLFLPHGLEPEAGGTLPSRLQPILLLDPSGSHAPLLTVPGLGPLPFHFAQSLMTTERLSGSGLHWPLSRTRSEPLPPSATAPPPPGPMQPRLEQLKTH 406
HDAC7-223    .............................................................................................................. 152
HDAC7-211    ..............................................................................................................  88
HDAC7-202    GPRGPILGSPHTPLFLPHGLEPEAGGTLPSRLQPILLLDPSGSHAPLLTVPGLGPLPFHFAQSLMTTERLSGSGLHWPLSRTRSEPLPPSATAPPPPGPMQPRLEQLKTH 386
HDAC7-228    ..............................................................................................................   0
HDAC7-216    .............................................................................................................. 112
HDAC7-201    GPRGPILGSPHTPLFLPHGLEPEAGGTLPSRLQPILLLDPSGSHAPLLTVPGLGPLPFHFAQSLMTTERLSGSGLHWPLSRTRSEPLPPSATAPPPPGPMQPRLEQLKTH 423
HDAC7-213    .............................................................................................................. 159
```

```
logo
              VQVIKRSAKPSEKPRLRQIPSAEDLETDGGGPGQVVDDGLEHRELGHGQPEARGPAPLQQHPQVLLWEQQRLAGRLPRGSTGDTVLLPLAQGGHRPLSRAQSSPAAPASL
TRICHOSTATIN  ..............................................................................................................    0
PF00850.1     ..............................................................................................................    0
PF00850       ..............................................................................................................    0
HDAC7-224     ..............................................................................................................    0
HDAC7-214     ..............................................................................................................   51
HDAC7-208     ..................LHGHADARVAGGTPGTRAAAAS................P.QGQEQAKCCSQQRGQAEASG...GDSE...KTAGGPRKNSP...........   65
HDAC7-203     VQVIKRSAKPSEKPRLRQIPSAEDLETDGGGPGQVVDDGLEHRELGHGQPEARGPAPLQQHPQVLLWEQQRLAGRLPRGSTGDTVLLPLAQGGHRPLSRAQSSPAAPASL  550
HDAC7-205     ..............................................................................................................  133
HDAC7-226     ..............................................................................................................    0
HDAC7-215     ..............................................................................................................  153
HDAC7-227     ..............................................................................................................    0
HDAC7-206     ..............................................................................................................  138
HDAC7-204     ..............................................................................................................   78
HDAC7-207     ..............................................................................................................  141
HDAC7-212     ..............................................................................................................  153
HDAC7-221     ..............................................................................................................  224
HDAC7-210     ..............................................................................................................  274
HDAC7-209     VQVIKRSAKPSEKPRLRQIPSAEDLETDGGGPGQVVDDGLEHRELGHGQPEARGPAPLQQHPQVLLWEQQRLAGRLPRGSTGDTVLLPLAQGGHRPLSRAQSSPAAPASL  494
HDAC7-230     VQVIKRSAKPSEKPRLRQIPSAEDLETDGGGPGQVVDDGLEHRELGHGQPEARGPAPLQQHPQVLLWEQQRLAGRLPRGSTGDTVLLPLAQGGHRPLSRAQSSPAAPASL  516
HDAC7-223     ..............................................................................................................  152
HDAC7-211     ..............................................................................................................   88
HDAC7-202     VQVIKRSAKPSEKPRLRQIPSAEDLETDGGGPGQVVDDGLEHRELGHGQPEARGPAPLQQHPQVLLWEQQRLAGRLPRGSTGDTVLLPLAQGGHRPLSRAQSSPAAPASL  496
HDAC7-228     ..............................................................................XSTGDTVLLPLAQGGHRPLSRAQSSPAAPASL   32
HDAC7-216     ..............................................................................................................  112
HDAC7-201     VQVIKRSAKPSEKPRLRQIPSAEDLETDGGGPGQVVDDGLEHRELGHGQPEARGPAPLQQHPQVLLWEQQRLAGRLPRGSTGDTVLLPLAQGGHRPLSRAQSSPAAPASL  533
HDAC7-213     ..............................................................................................................  159
```

logo

```
             SAPEPASQARVLSSSETPARTLPFTTGLIYDSVMLKHQCSCGDNSRHPEHAGRIQSIWSRLQERGLRSQCECLRGRKASLEELQSVHSERHVLLYGTNPLSRLKLDNGKL
TRICHOSTATIN .............................................R.P..............................................................   2
PF00850.1    ..............................................................................................................   0
PF00850      ..............................................HPEHAGRIQSIWSRLQERGLRSQCECLRGRKASLEELQSVHSERHVLLYGTNPLSRLKLDNGKL  64
HDAC7-224    ...........................XIYDSVMLKHQCSCGDNSRHPEHAGRIQSIWSRLQERGLRSQCECLRGRKASLEELQSVHSERHVLLYGTNPLSRLKLDNGKL  83
HDAC7-214    ..............................................................................................................  51
HDAC7-208    ..............SQQPRH.............SLQNPGAPGDGRSHPLHAQQLFASCSQPAQ............................................... 101
HDAC7-203    SAPEPASQARVLSSSETPARTLPFTTGLIYDSVMLKHQCSCGDNSRHPEHAGRIQSIWSRLQERGLRSQCECLRGRKASLEELQSVHSERHVLLYGTNPLSRLKLDNGKL 660
HDAC7-205    .............................................................................................................. 133
HDAC7-226    ..............................................................................................................   0
HDAC7-215    .............................................................................................................. 153
HDAC7-227    ..........................XLIYDSVMLKHQCSCGDNSRHPEHAGRIQSIWSRLQERGLRSQCECLRGRKASLEELQSVHSERHVLLYGTNPLSRLKLDNGKL  84
HDAC7-206    .............................................................................................................. 138
HDAC7-204    ..............................................................................................................  78
HDAC7-207    .............................................................................................................. 141
HDAC7-212    .............................................................................................................. 153
HDAC7-221    .............................................................................................................. 224
HDAC7-210    .............................................................................................................. 274
HDAC7-209    SAPEPASQARVLSSSETPARTLPFTTGLIYDSVMLKHQCSCGDNSRHPEHAGRIQSIWSRLQERGLRSQCECLRGRKASLEELQSVHSERHVLLYGTNPLSRLKLDNGKL 604
HDAC7-230    SAPEPASQARVLSSSETPARTLPFTTGLIYDSVMLKHQCSCGDNSRHPEHAGRIQSIWSRLQERGLRSQCECLRGRKASLEELQSVHSERHVLLYGTNPLSRLKLDNGKL 626
HDAC7-223    .............................................................................................................. 152
HDAC7-211    ..............................................................................................................  88
HDAC7-202    SAPEPASQARVLSSSETPARTLPFTTGLIYDSVMLKHQCSCGDNSRHPEHAGRIQSIWSRLQERGLRSQCECLRGRKASLEELQSVHSERHVLLYGTNPLSRLKLDNGKL 606
HDAC7-228    SAPEPASQARVLSSSETPARTLPFTTGSL.................................................................................  61
HDAC7-216    .............................................................................................................. 112
HDAC7-201    SAPEPASQARVLSSSETPARTLPFTTGLIYDSVMLKHQCSCGDNSRHPEHAGRIQSIWSRLQERGLRSQCECLRGRKASLEELQSVHSERHVLLYGTNPLSRLKLDNGKL 643
HDAC7-213    .............................................................................................................. 159
```

```
logo
             AGLLAQRMFVMLPCGGVGVDTDTIWNELHSSNAARWAAGSVTDLAFKVASRELKNGFAVVRPPGHHADHSTAMGFCFFNSVAIACRQLQQQSKASKILIVDWDVHHGNGT
TRICHOSTATIN .....................D..........................................HH.......GF...........................D.H.....   9
PF00850.1    ......................................................................................................DVHHGNGT   8
PF00850      AGLLAQRMFVMLPCGGVGVDTDTIWNELHSSNAARWAAGSVTDLAFKVASRELKNGFAVVRPPGHHADHSTAMGFCFFNSVAIACRQLQQQSKASKILIVDWDVHHGNGT 174
HDAC7-224    AGLLAQRMFVMLPCGGVGVDTDTIWNELHSSNAARWAAGSVTDLAFKVASRELKNGFAVVRPPGHHADHSTAMGFCFFNSVAIACRQLQQQSKASKILIVDWVVMIRIIA 193
HDAC7-214    .............................................................................................................. 51
HDAC7-208    .............................................................................................................. 101
HDAC7-203    AGLLAQRMFVMLPCGGVGVDTDTIWNELHSSNAARWAAGSVTDLAFKVASRELKNGFAVVRPPGHHADHSTAMGFCFFNSVAIACRQLQQQSKASKILIVDWDVHHGNGT 770
HDAC7-205    .............................................................................................................. 133
HDAC7-226    .............................................................................................................. 0
HDAC7-215    .............................................................................................................. 153
HDAC7-227    AGLLAQRMFVMLPCGGVGVDTDTIWNELHSSNAARWAAGSVTDLAFKVASRELK................................................DVHHGNGT 146
HDAC7-206    .............................................................................................................. 138
HDAC7-204    .............................................................................................................. 78
HDAC7-207    .............................................................................................................. 141
HDAC7-212    .............................................................................................................. 153
HDAC7-221    .............................................................................................................. 224
HDAC7-210    .............................................................................................................. 274
HDAC7-209    AGLLAQRMFVMLPCGGVGVDTDTIWNELHSSNAARWAAGSVTDLAFKVASRELKNGFAVVRPPGHHADHSTAMGFCFFNSVAIACRQLQQQSKASKILIVDWDVHHGNGT 714
HDAC7-230    AGLLAQRMFVMLPCGGVGVDTDTIWNELHSSNAARWAAGSVTDLAFKVASRELKNGFAVVRPPGHHADHSTAMGFCFFNSVAIACRQLQQQSKASKILIVDWDVHHGNGT 736
HDAC7-223    .............................................................................................................. 152
HDAC7-211    .............................................................................................................. 88
HDAC7-202    AGLLAQRMFVMLPCGGVGVDTDTIWNELHSSNAARWAAGSVTDLAFKVASRELKNGFAVVRPPGHHADHSTAMGFCFFNSVAIACRQLQQQSKASKILIVDWDVHHGNGT 716
HDAC7-228    .............................................................................................................. 61
HDAC7-216    .............................................................................................................. 112
HDAC7-201    AGLLAQRMFVMLPCGGVGVDTDTIWNELHSSNAARWAAGSVTDLAFKVASRELKNGFAVVRPPGHHADHSTAMGFCFFNSVAIACRQLQQQSKASKILIVDWDVHHGNGT 753
HDAC7-213    .............................................................................................................. 159
```

```
logo
               QQTFYQDPSVLYISLHRHDDGNFFPGSGAVDEVGAGSGEGFNVNVAWAGGLDPPMGDPEYLAAFRIVVMPIAREFSPDLVLVSAGFDAAEGHPAPLGGYHVSAKCFGYMT
TRICHOSTATIN   .......................F..............................................................D.......PL..............  13
PF00850.1      QQTFYQDPSVLYISLHRHDDGNFFPGSGAVDEVGAGSGEGFNVNVAWAGGLDPPMGDPEYLAAFRIVVMPIAREFSPDLVLVSAGFDAAEGHPAPLGGYHVSAKCFGYMT  118
PF00850        QQTFYQDPSVLYISLHRHDDGNFFPGSGAVDEVGAGSGEGFNVNVAWAGGLDPPMGDPEYLAAFRIVVMPIAREFSPDLVLVSAGFDAAEGHPAPLGGYHVSAKCFGYMT  284
HDAC7-224      DNI.....STPLI.TSRH............................................................................................  205
HDAC7-214      ..............................................................................................................  51
HDAC7-208      ..............................................................................................................  101
HDAC7-203      QQTFYQDPSVLYISLHRHDDGNFFPGSGAVDEVGAGSGEGFNVNVAWAGGLDPPMGDPEYLAAFRIVVMPIAREFSPDLVLVSAGFDAAEGHPAPLGGYHVSAKCFGYMT  880
HDAC7-205      ..............................................................................................................  133
HDAC7-226      ............................................................................XDLVLVSAGFDAAEGHPAPLGGYHVSAKCFGYMT  34
HDAC7-215      ..............................................................................................................  153
HDAC7-227      QQTFYQDPSVLYISLHRHDDGNFFPGSGAVDEVGAGSGEGFNVNVAWAGGLDPPMGDPEYLAAFRIVVMPIAREFSPDLVLVSAGFDAAEGHPAPLGGYHVSAKCFGYMT  256
HDAC7-206      ..............................................................................................................  138
HDAC7-204      ..............................................................................................................  78
HDAC7-207      ..............................................................................................................  141
HDAC7-212      ..............................................................................................................  153
HDAC7-221      ..............................................................................................................  224
HDAC7-210      ..............................................................................................................  274
HDAC7-209      QQTFYQDPSVLYISLHRHDDGNFFPGSGAVDEVGAGSGEGFNVNVAWAGGLDPPMGDPEYLAAFRIVVMPIAREFSPDLVLVSAGFDAAEGHPAPLGGYHVSAKCFGYMT  824
HDAC7-230      QQTFYQDPSVLYISLHRHDDGNFFPGSGAVDEVGAGSGEGFNVNVAWAGGLDPPMGDPEYLAAFRIVVMPIAREFSPDLVLVSAGFDAAEGHPAPLGGYHVSAKCFGYMT  846
HDAC7-223      ..............................................................................................................  152
HDAC7-211      ..............................................................................................................  88
HDAC7-202      QQTFYQDPSVLYISLHRHDDGNFFPGSGAVDEVGAGSGEGFNVNVAWAGGLDPPMGDPEYLAAFRIVVMPIAREFSPDLVLVSAGFDAAEGHPAPLGGYHVSAKCFGYMT  826
HDAC7-228      ..............................................................................................................  61
HDAC7-216      ..............................................................................................................  112
HDAC7-201      QQTFYQDPSVLYISLHRHDDGNFFPGSGAVDEVGAGSGEGFNVNVAWAGGLDPPMGDPEYLAAFRIVVMPIAREFSPDLVLVSAGFDAAEGHPAPLGGYHVSAKCFGYMT  863
HDAC7-213      ..............................................................................................................  159
```

logo

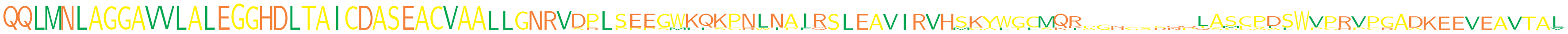

```
TRICHOSTATIN   ................G.............................................................................................   14
PF00850.1      QQLMNLAGGAVVLALEGGHDLTAICDASEACVAA............................................................................  152
PF00850        QQLMNLAGGAVVLALEGGHDLTAICDASEACVAA............................................................................  318
HDAC7-224      ..............................................................................................................  205
HDAC7-214      ..............................................................................................................   51
HDAC7-208      ..............................................................................................................  101
HDAC7-203      QQLMNLAGGAVVLALEGGHDLTAICDASEACVAALLGNRVDPLSEEGWKQKPNLNAIRSLEAVIRVHMGALTLSQIPGHGSSQQQAGGAFSWPGHPCRAA.........V  981
HDAC7-205      ..............................................................................................................  133
HDAC7-226      QQLMNLAGGAVVLALEGGHDLTAICDASEACVAALLGNRVSRLPPPSMLLSGRWIPFQ................KKAGN..RNPTSMPSALW..................  108
HDAC7-215      ..............................................................................................................  153
HDAC7-227      QQLMNLAGGAVVLALEGGHDLTAICDASEACVAALLGNRVDPLSEEGWKQKPNLNAIRSLEAVIRVHSKYWGCMQR........LASCPDSWVPRVPGADKEEVEAVTAL  358
HDAC7-206      ..............................................................................................................  138
HDAC7-204      ..............................................................................................................   78
HDAC7-207      ..............................................................................................................  141
HDAC7-212      ..............................................................................................................  153
HDAC7-221      ..............................................................................................................  224
HDAC7-210      ..............................................................................................................  274
HDAC7-209      QQLMNLAGGAVVLALEGGHDLTAICDASEACVAALLGNRVDPLSEEGWKQKPNLNAIRSLEAVIRVHSKYWGCMQR........LASCPDSWVPRVPGADKEEVEAVTAL  926
HDAC7-230      QQLMNLAGGAVVLALEGGHDLTAICDASEACVAALLGNRVDPLSEEGWKQKPNLNAIRSLEAVIRVHSKYWGCMQR........LASCPDSWVPRVPGADKEEVEAVTAL  948
HDAC7-223      ..............................................................................................................  152
HDAC7-211      ..............................................................................................................   88
HDAC7-202      QQLMNLAGGAVVLALEGGHDLTAICDASEACVAALLGNRVDPLSEEGWKQKPNLNAIRSLEAVIRVHSKYWGCMQR........LASCPDSWVPRVPGADKEEVEAVTAL  928
HDAC7-228      ..............................................................................................................   61
HDAC7-216      ..............................................................................................................  112
HDAC7-201      QQLMNLAGGAVVLALEGGHDLTAICDASEACVAALLGNRVDPLSEEGWKQKPNLNAIRSLEAVIRVHSKYWGCMQR........LASCPDSWVPRVPGADKEEVEAVTAL  965
HDAC7-213      ..............................................................................................................  159
```

logo

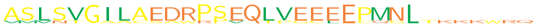

```
TRICHOSTATIN  .................................    14
PF00850.1     .................................   152
PF00850       .................................   318
HDAC7-224     .................................   205
HDAC7-214     .................................    51
HDAC7-208     .................................   101
HDAC7-203     VMVNTGAACSAWPPVQTPGCLECQGLTKKKWRQ  1014
HDAC7-205     .................................   133
HDAC7-226     ............RP...................   110
HDAC7-215     .................................   153
HDAC7-227     ASLSVGILAEDRPSEQLVEEEEPMNL.......   384
HDAC7-206     .................................   138
HDAC7-204     .................................    78
HDAC7-207     .................................   141
HDAC7-212     .................................   153
HDAC7-221     .................................   224
HDAC7-210     .................................   274
HDAC7-209     ASLSVGILAEDRPSEQLVEEEEPMNL.......   952
HDAC7-230     ASLSVGILAEDRPSEQLVEEEEPMNL.......   974
HDAC7-223     .................................   152
HDAC7-211     .................................    88
HDAC7-202     ASLSVGILAEDRPSEQLVEEEEPMNL.......   954
HDAC7-228     .................................    61
HDAC7-216     .................................   112
HDAC7-201     ASLSVGILAEDRPSEQLVEEEEPMNL.......   991
HDAC7-213     .................................   159
```

X non conserved
X similar
X $\geq$ 0% conserved
X $\geq$ 50% conserved

logo

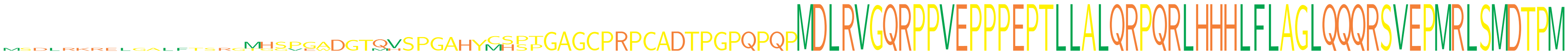

```
VORINOSTAT   ..............................................................................................................   0
PF00850.1    ..............................................................................................................   0
PF00850      ..............................................................................................................   0
HDAC7-224    ..............................................................................................................   0
HDAC7-214    ........................................................MDLRVGQRPPVEPPPEPTLLALQRPQRLHHHLFLAGLQQQRSVEPMRLSMD...  51
HDAC7-208    ..........................MHSPGA..............................................................................   6
HDAC7-203    MSDLRKRELGALFTSRGTGGVEWDGTQVSPGAHYCSPTGAGCPRPCADTPGPQPQPMDLRVGQRPPVEPPPEPTLLALQRPQRLHHHLFLAGLQQQRSVEPMRLSMDTPM 110
HDAC7-205    ..................................MHSPGAGCPRPCADTPGPQPQPMDLRVGQRPPVEPPPEPTLLALQRPQRLHHHLFLAGLQQQRSVEPMRLSMDTPM  76
HDAC7-226    ..............................................................................................................   0
HDAC7-215    ........................................................MDLRVGQRPPVEPPPEPTLLALQRPQRLHHHLFLAGLQQQRSVEPMRLSMDTPM  54
HDAC7-227    ..............................................................................................................   0
HDAC7-206    ........................................................MDLRVGQRPPVEPPPEPTLLALQRPQRLHHHLFLAGLQQQRSVEPMRLSMDTPM  54
HDAC7-204    ........................................................MDLRVGQRPPVEPPPEPTLLALQRPQRLHHHLFLAGLQQQRSVEPMRLSMDTPM  54
HDAC7-207    ........................................................MDLRVGQRPPVEPPPEPTLLALQRPQRLHHHLFLAGLQQQRSVEPMRLSMDTPM  54
HDAC7-212    ........................................................MDLRVGQRPPVEPPPEPTLLALQRPQRLHHHLFLAGLQQQRSVEPMRLSMDTPM  54
HDAC7-221    ........................................................MDLRVGQRPPVEPPPEPTLLALQRPQRLHHHLFLAGLQQQRSVEPMRLSMDTPM  54
HDAC7-210    .................MHSPGADGTQVSPGAHYCSPTGAGCPRPCADTPGPQPQPMDLRVGQRPPVEPPPEPTLLALQRPQRLHHHLFLAGLQQQRSVEPMRLSMDTPM  93
HDAC7-209    ........................................................MDLRVGQRPPVEPPPEPTLLALQRPQRLHHHLFLAGLQQQRSVEPMRLSMDTPM  54
HDAC7-230    ..................................MHSPGAGCPRPCADTPGPQPQPMDLRVGQRPPVEPPPEPTLLALQRPQRLHHHLFLAGLQQQRSVEPMRLSMDTPM  76
HDAC7-223    ..............................................................................................................   0
HDAC7-211    ........................................................MDLRVGQRPPVEPPPEPTLLALQRPQRLHHHLFLAGLQQQRSVEPMRLSMDTPM  54
HDAC7-202    .................MHSPGADGTQVSPGAHYCSPTGAGCPRPCADTPGPQPQPMDLRVGQRPPVEPPPEPTLLALQRPQRLHHHLFLAGLQQQRSVEPMRLSMDTPM  93
HDAC7-228    ..............................................................................................................   0
HDAC7-216    ........................................................MDLRVGQRPPVEPPPEPTLLALQRPQRLHHHLFLAGLQQQRSVEPMRLSMDTPM  54
HDAC7-201    .................MHSPGADGTQVSPGAHYCSPTGAGCPRPCADTPGPQPQPMDLRVGQRPPVEPPPEPTLLALQRPQRLHHHLFLAGLQQQRSVEPMRLSMDTPM  93
HDAC7-213    ..................................MHSPGAGCPRPCADTPGPQPQPMDLRVGQRPPVEPPPEPTLLALQRPQRLHHHLFLAGLQQQRSVEPMRLSMDTPM  76
```

logo

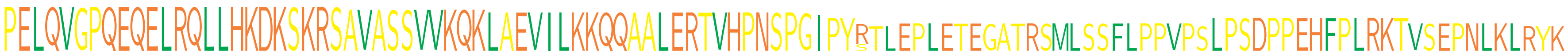

```
VORINOSTAT   ..............................................................................................................   0
PF00850.1    ..............................................................................................................   0
PF00850      ..............................................................................................................   0
HDAC7-224    ..............................................................................................................   0
HDAC7-214    ..............................................................................................................   51
HDAC7-208    ..............................................................................................................   6
HDAC7-203    PELQVGPQEQELRQLLHKDKSKRSAVASSVVKQKLAEVILKKQQAALERTVHPNSPGIPYRTLEPLETEGATRSMLSSFLPPVPSLPSDPPEHFPLRKTVSEPNLKLRYK   220
HDAC7-205    PELQVGPQEQELRQLLHKDKSKRSAVASSVVKQKLAEVILKKQQAALERTVHPNSPG.....................................................   133
HDAC7-226    ..............................................................................................................   0
HDAC7-215    PELQVGPQEQELRQLLHKDKSKRSAVASSVVKQKLAEVILKKQQAALERTVHPNSPGIPYRTLEPLETEGATRSMLSSFLPPVPSLPSDPPEHFPLRKT...........   153
HDAC7-227    ..............................................................................................................   0
HDAC7-206    PELQVGPQEQELRQLLHKDKSKRSAVASSVVKQKLAEVILKKQQAALERTVHPNSPGIPYRTLEPLETEGATRSMLSSFLPPVP..........................   138
HDAC7-204    PELQVGPQEQELRQLLHKDKSKRS......................................................................................   78
HDAC7-207    PELQVGPQEQELRQLLHKDKSKRSAVASSVVKQKLAEVILKKQQAALERTVHPNSPGIPYS........................LPSDPPEHFPLRKTVSEPNLKLRYK   140
HDAC7-212    PELQVGPQEQELRQLLHKDKSKRSAVASSVVKQKLAEVILKKQQAALERTVHPNSPGIPYRTLEPLETEGATRSMLSSFLPPVPSLPSDPPEHFPLRKT...........   153
HDAC7-221    PELQVGPQEQELRQLLHKDKSKRSAVASSVVKQKLAEVILKKQQAALERTVHPNSPGIPYRTLEPLETEGATRSMLSSFLPPVPSLPSDPPEHFPLRKTVSEPNLKLRYK   164
HDAC7-210    PELQVGPQEQELRQLLHKDKSKRSAVASSVVKQKLAEVILKKQQAALERTVHPNSPGIPYS........................LPSDPPEHFPLRKTVSEPNLKLRYK   179
HDAC7-209    PELQVGPQEQELRQLLHKDKSKRSAVASSVVKQKLAEVILKKQQAALERTVHPNSPGIPYRTLEPLETEGATRSMLSSFLPPVPSLPSDPPEHFPLRKTVSEPNLKLRYK   164
HDAC7-230    PELQVGPQEQELRQLLHKDKSKRSAVASSVVKQKLAEVILKKQQAALERTVHPNSPGIPYRTLEPLETEGATRSMLSSFLPPVPSLPSDPPEHFPLRKTVSEPNLKLRYK   186
HDAC7-223    ...............................................................EPLETEGATRSMLSSFLPPVPSLPSDPPEHFPLRKTVSEPNLKLRYK   47
HDAC7-211    PELQVGPQEQELRQLLHKDKSKRSAVASSVVKQK............................................................................   88
HDAC7-202    PELQVGPQEQELRQLLHKDKSKRSAVASSVVKQKLAEVILKKQQAALERTVHPNSPGIPYRTLEPLETEGATRSMLSSFLPPVPSLPSDPPEHFPLRKTVSEPNLKLRYK   203
HDAC7-228    ..............................................................................................................   0
HDAC7-216    PELQVGPQEQELRQLLHKDKSKRSAVASSVVKQKLAEVILKKQQAALERTVHPNSPGI....................................................   112
HDAC7-201    PELQVGPQEQELRQLLHKDKSKRSAVASSVVKQKLAEVILKKQQAALERTVHPNSPGIPYRTLEPLETEGATRSMLSSFLPPVPSLPSDPPEHFPLRKTVSEPNLKLRYK   203
HDAC7-213    PELQVGPQEQELRQLLHKDKSKRSAVASSVVKQKLAEVILKKQQAALERTVHPNSPGIPYS........................LPSDPPEHFPLRKTVSEPNLKL...   159
```

```
logo
               PKKSLERRKNPLLRKESAPPSLRRRPAETLGDSSPSSSSTPASGCSSPNDSEHGPNPILGSEALLGQRLRLQETSVAPFALPTVSLLPAITLGLPAPARADSDRRTHPTL
VORINOSTAT     ..............................................................................................................    0
PF00850.1      ..............................................................................................................    0
PF00850        ..............................................................................................................    0
HDAC7-224      ..............................................................................................................    0
HDAC7-214      ..............................................................................................................   51
HDAC7-208      ..............................................................................................................    6
HDAC7-203      PKKSLERRKNPLLRKESAPPSLRRRPAETLGDSSPSSSSTPASGCSSPNDSEHGPNPILGSEALLGQRLRLQETSVAPFALPTVSLLPAITLGLPAPARADSDRRTHPTL  330
HDAC7-205      ..............................................................................................................  133
HDAC7-226      ..............................................................................................................    0
HDAC7-215      ..............................................................................................................  153
HDAC7-227      ..............................................................................................................    0
HDAC7-206      ..............................................................................................................  138
HDAC7-204      ..............................................................................................................   78
HDAC7-207      P.............................................................................................................  141
HDAC7-212      ..............................................................................................................  153
HDAC7-221      PKKSLERRKNPLLRKESAPPSLRRRPAETLGDSSPSSSSTPASGCSSPNDSEHGPNPILG..................................................  224
HDAC7-210      PKKSLERRKNPLLRKESAPPSLRRRPAETLGDSSPSSSSTPASGCSSPNDSEHGPNPILGSE.....................................ADSDRRTHPTL  252
HDAC7-209      PKKSLERRKNPLLRKESAPPSLRRRPAETLGDSSPSSSSTPASGCSSPNDSEHGPNPILGSEALLGQRLRLQETSVAPFALPTVSLLPAITLGLPAPARADSDRRTHPTL  274
HDAC7-230      PKKSLERRKNPLLRKESAPPSLRRRPAETLGDSSPSSSSTPASGCSSPNDSEHGPNPILGSEALLGQRLRLQETSVAPFALPTVSLLPAITLGLPAPARADSDRRTHPTL  296
HDAC7-223      PKKSLERRKNPLLRKESAPPSLRRRPAETLGDSSPSSSSTPASGCSSPNDSEHGPNPILGSEALLGQRLRLQETSVAPFALPTVSLLPAITLGLPAPARALMPRC.....  152
HDAC7-211      ..............................................................................................................   88
HDAC7-202      PKKSLERRKNPLLRKESAPPSLRRRPAETLGDSSPSSSSTPASGCSSPNDSEHGPNPILGSE.....................................ADSDRRTHPTL  276
HDAC7-228      ..............................................................................................................    0
HDAC7-216      ..............................................................................................................  112
HDAC7-201      PKKSLERRKNPLLRKESAPPSLRRRPAETLGDSSPSSSSTPASGCSSPNDSEHGPNPILGSEALLGQRLRLQETSVAPFALPTVSLLPAITLGLPAPARADSDRRTHPTL  313
HDAC7-213      ..............................................................................................................  159
```

```
logo
              GPRGPILGSPHTPLFLPHGLEPEAGGTLPSRLQPILLLDPSGSHAPLLTVPGLGPLPFHFAQSLMTTERLSGSGLHWPLSRTRSEPLPPSATAPPPPGPMQPRLEQLKTH
VORINOSTAT    ..............................................................................................................     0
PF00850.1     ..............................................................................................................     0
PF00850       ..............................................................................................................     0
HDAC7-224     ..............................................................................................................     0
HDAC7-214     ..............................................................................................................    51
HDAC7-208     .........................................................................A....................................     7
HDAC7-203     GPRGPILGSPHTPLFLPHGLEPEAGGTLPSRLQPILLLDPSGSHAPLLTVPGLGPLPFHFAQSLMTTERLSGSGLHWPLSRTRSEPLPPSATAPPPPGPMQPRLEQLKTH   440
HDAC7-205     ..............................................................................................................   133
HDAC7-226     ..............................................................................................................     0
HDAC7-215     ..............................................................................................................   153
HDAC7-227     ..............................................................................................................     0
HDAC7-206     ..............................................................................................................   138
HDAC7-204     ..............................................................................................................    78
HDAC7-207     ..............................................................................................................   141
HDAC7-212     ..............................................................................................................   153
HDAC7-221     ..............................................................................................................   224
HDAC7-210     GPRGPILGSPHTPLFLPHGLEP........................................................................................   274
HDAC7-209     GPRGPILGSPHTPLFLPHGLEPEAGGTLPSRLQPILLLDPSGSHAPLLTVPGLGPLPFHFAQSLMTTERLSGSGLHWPLSRTRSEPLPPSATAPPPPGPMQPRLEQLKTH   384
HDAC7-230     GPRGPILGSPHTPLFLPHGLEPEAGGTLPSRLQPILLLDPSGSHAPLLTVPGLGPLPFHFAQSLMTTERLSGSGLHWPLSRTRSEPLPPSATAPPPPGPMQPRLEQLKTH   406
HDAC7-223     ..............................................................................................................   152
HDAC7-211     ..............................................................................................................    88
HDAC7-202     GPRGPILGSPHTPLFLPHGLEPEAGGTLPSRLQPILLLDPSGSHAPLLTVPGLGPLPFHFAQSLMTTERLSGSGLHWPLSRTRSEPLPPSATAPPPPGPMQPRLEQLKTH   386
HDAC7-228     ..............................................................................................................     0
HDAC7-216     ..............................................................................................................   112
HDAC7-201     GPRGPILGSPHTPLFLPHGLEPEAGGTLPSRLQPILLLDPSGSHAPLLTVPGLGPLPFHFAQSLMTTERLSGSGLHWPLSRTRSEPLPPSATAPPPPGPMQPRLEQLKTH   423
HDAC7-213     ..............................................................................................................   159
```

```
logo
               VQVIKRSAKPSEKPRLRQIPSAEDLETDGGGPGQVVDDGLEHRELGHGQPEARGPAPLQQHPQVLLWEQQRLAGRLPRGSTGDTVLLPLAQGGHRPLSRAQSSPAAPASL
VORINOSTAT     ..............................................................................................................    0
PF00850.1      ..............................................................................................................    0
PF00850        ..............................................................................................................    0
HDAC7-224      ..............................................................................................................    0
HDAC7-214      ..............................................................................................................   51
HDAC7-208      ..................LHGHADARVAGGTPGTRAAAAS................P.QGGQEQAKCCSQQRGQAEASG...GDSE...KTAGGPRKNSP..........   65
HDAC7-203      VQVIKRSAKPSEKPRLRQIPSAEDLETDGGGPGQVVDDGLEHRELGHGQPEARGPAPLQQHPQVLLWEQQRLAGRLPRGSTGDTVLLPLAQGGHRPLSRAQSSPAAPASL  550
HDAC7-205      ..............................................................................................................  133
HDAC7-226      ..............................................................................................................    0
HDAC7-215      ..............................................................................................................  153
HDAC7-227      ..............................................................................................................    0
HDAC7-206      ..............................................................................................................  138
HDAC7-204      ..............................................................................................................   78
HDAC7-207      ..............................................................................................................  141
HDAC7-212      ..............................................................................................................  153
HDAC7-221      ..............................................................................................................  224
HDAC7-210      ..............................................................................................................  274
HDAC7-209      VQVIKRSAKPSEKPRLRQIPSAEDLETDGGGPGQVVDDGLEHRELGHGQPEARGPAPLQQHPQVLLWEQQRLAGRLPRGSTGDTVLLPLAQGGHRPLSRAQSSPAAPASL  494
HDAC7-230      VQVIKRSAKPSEKPRLRQIPSAEDLETDGGGPGQVVDDGLEHRELGHGQPEARGPAPLQQHPQVLLWEQQRLAGRLPRGSTGDTVLLPLAQGGHRPLSRAQSSPAAPASL  516
HDAC7-223      ..............................................................................................................  152
HDAC7-211      ..............................................................................................................   88
HDAC7-202      VQVIKRSAKPSEKPRLRQIPSAEDLETDGGGPGQVVDDGLEHRELGHGQPEARGPAPLQQHPQVLLWEQQRLAGRLPRGSTGDTVLLPLAQGGHRPLSRAQSSPAAPASL  496
HDAC7-228      ..............................................................................XSTGDTVLLPLAQGGHRPLSRAQSSPAAPASL   32
HDAC7-216      ..............................................................................................................  112
HDAC7-201      VQVIKRSAKPSEKPRLRQIPSAEDLETDGGGPGQVVDDGLEHRELGHGQPEARGPAPLQQHPQVLLWEQQRLAGRLPRGSTGDTVLLPLAQGGHRPLSRAQSSPAAPASL  533
HDAC7-213      ..............................................................................................................  159
```

```
logo
             SAPEPASQARVLSSSETPARTLPFTTGLIYDSVMLKHQCSCGDNSRHPEHAGRIQSIWSRLQERGLRSQCECLRGRKASLEELQSVHSERHVLLYGTNPLSRLKLDNGKL
VORINOSTAT   .............................................................................................................. 0
PF00850.1    .............................................................................................................. 0
PF00850      ..............................................HPEHAGRIQSIWSRLQERGLRSQCECLRGRKASLEELQSVHSERHVLLYGTNPLSRLKLDNGKL 64
HDAC7-224    ...........................XIYDSVMLKHQCSCGDNSRHPEHAGRIQSIWSRLQERGLRSQCECLRGRKASLEELQSVHSERHVLLYGTNPLSRLKLDNGKL 83
HDAC7-214    .............................................................................................................. 51
HDAC7-208    ..............SQQPRH.............SLQNPGAPGDGRSHPLHAQQLFASCSQPAQ............................................... 101
HDAC7-203    SAPEPASQARVLSSSETPARTLPFTTGLIYDSVMLKHQCSCGDNSRHPEHAGRIQSIWSRLQERGLRSQCECLRGRKASLEELQSVHSERHVLLYGTNPLSRLKLDNGKL 660
HDAC7-205    .............................................................................................................. 133
HDAC7-226    .............................................................................................................. 0
HDAC7-215    .............................................................................................................. 153
HDAC7-227    ..........................XLIYDSVMLKHQCSCGDNSRHPEHAGRIQSIWSRLQERGLRSQCECLRGRKASLEELQSVHSERHVLLYGTNPLSRLKLDNGKL 84
HDAC7-206    .............................................................................................................. 138
HDAC7-204    .............................................................................................................. 78
HDAC7-207    .............................................................................................................. 141
HDAC7-212    .............................................................................................................. 153
HDAC7-221    .............................................................................................................. 224
HDAC7-210    .............................................................................................................. 274
HDAC7-209    SAPEPASQARVLSSSETPARTLPFTTGLIYDSVMLKHQCSCGDNSRHPEHAGRIQSIWSRLQERGLRSQCECLRGRKASLEELQSVHSERHVLLYGTNPLSRLKLDNGKL 604
HDAC7-230    SAPEPASQARVLSSSETPARTLPFTTGLIYDSVMLKHQCSCGDNSRHPEHAGRIQSIWSRLQERGLRSQCECLRGRKASLEELQSVHSERHVLLYGTNPLSRLKLDNGKL 626
HDAC7-223    .............................................................................................................. 152
HDAC7-211    .............................................................................................................. 88
HDAC7-202    SAPEPASQARVLSSSETPARTLPFTTGLIYDSVMLKHQCSCGDNSRHPEHAGRIQSIWSRLQERGLRSQCECLRGRKASLEELQSVHSERHVLLYGTNPLSRLKLDNGKL 606
HDAC7-228    SAPEPASQARVLSSSETPARTLPFTTGSL................................................................................. 61
HDAC7-216    .............................................................................................................. 112
HDAC7-201    SAPEPASQARVLSSSETPARTLPFTTGLIYDSVMLKHQCSCGDNSRHPEHAGRIQSIWSRLQERGLRSQCECLRGRKASLEELQSVHSERHVLLYGTNPLSRLKLDNGKL 643
HDAC7-213    .............................................................................................................. 159
```

```
logo
            AGLLAQRMFVMLPCGGVGVDTDTIWNELHSSNAARWAAGSVTDLAFKVASRELKNGFAVVRPPGHHADHSTAMGFCFFNSVAIACRQLQQQSKASKILIVDWDVHHGNGT
VORINOSTAT  ................................................................HH.......GF...........................D.H.....     6
PF00850.1   ......................................................................................................DVHHGNGT     8
PF00850     AGLLAQRMFVMLPCGGVGVDTDTIWNELHSSNAARWAAGSVTDLAFKVASRELKNGFAVVRPPGHHADHSTAMGFCFFNSVAIACRQLQQQSKASKILIVDWDVHHGNGT   174
HDAC7-224   AGLLAQRMFVMLPCGGVGVDTDTIWNELHSSNAARWAAGSVTDLAFKVASRELKNGFAVVRPPGHHADHSTAMGFCFFNSVAIACRQLQQQSKASKILIVDWVVMIRIIA   193
HDAC7-214   ..............................................................................................................    51
HDAC7-208   ..............................................................................................................   101
HDAC7-203   AGLLAQRMFVMLPCGGVGVDTDTIWNELHSSNAARWAAGSVTDLAFKVASRELKNGFAVVRPPGHHADHSTAMGFCFFNSVAIACRQLQQQSKASKILIVDWDVHHGNGT   770
HDAC7-205   ..............................................................................................................   133
HDAC7-226   ..............................................................................................................     0
HDAC7-215   ..............................................................................................................   153
HDAC7-227   AGLLAQRMFVMLPCGGVGVDTDTIWNELHSSNAARWAAGSVTDLAFKVASRELK................................................DVHHGNGT   146
HDAC7-206   ..............................................................................................................   138
HDAC7-204   ..............................................................................................................    78
HDAC7-207   ..............................................................................................................   141
HDAC7-212   ..............................................................................................................   153
HDAC7-221   ..............................................................................................................   224
HDAC7-210   ..............................................................................................................   274
HDAC7-209   AGLLAQRMFVMLPCGGVGVDTDTIWNELHSSNAARWAAGSVTDLAFKVASRELKNGFAVVRPPGHHADHSTAMGFCFFNSVAIACRQLQQQSKASKILIVDWDVHHGNGT   714
HDAC7-230   AGLLAQRMFVMLPCGGVGVDTDTIWNELHSSNAARWAAGSVTDLAFKVASRELKNGFAVVRPPGHHADHSTAMGFCFFNSVAIACRQLQQQSKASKILIVDWDVHHGNGT   736
HDAC7-223   ..............................................................................................................   152
HDAC7-211   ..............................................................................................................    88
HDAC7-202   AGLLAQRMFVMLPCGGVGVDTDTIWNELHSSNAARWAAGSVTDLAFKVASRELKNGFAVVRPPGHHADHSTAMGFCFFNSVAIACRQLQQQSKASKILIVDWDVHHGNGT   716
HDAC7-228   ..............................................................................................................    61
HDAC7-216   ..............................................................................................................   112
HDAC7-201   AGLLAQRMFVMLPCGGVGVDTDTIWNELHSSNAARWAAGSVTDLAFKVASRELKNGFAVVRPPGHHADHSTAMGFCFFNSVAIACRQLQQQSKASKILIVDWDVHHGNGT   753
HDAC7-213   ..............................................................................................................   159
```

```
logo
                QQTFYQDPSVLYISLHRHDDGNFFPGSGAVDEVGAGSGEGFNVNVAWAGGLDPPMGDPEYLAAFRIVVMPIAREFSPDLVLVSAGFDAAEGHPAPLGGYHVSAKCFGYMT
VORINOSTAT      .......................F..............................................................................   7
PF00850.1       QQTFYQDPSVLYISLHRHDDGNFFPGSGAVDEVGAGSGEGFNVNVAWAGGLDPPMGDPEYLAAFRIVVMPIAREFSPDLVLVSAGFDAAEGHPAPLGGYHVSAKCFGYMT 118
PF00850         QQTFYQDPSVLYISLHRHDDGNFFPGSGAVDEVGAGSGEGFNVNVAWAGGLDPPMGDPEYLAAFRIVVMPIAREFSPDLVLVSAGFDAAEGHPAPLGGYHVSAKCFGYMT 284
HDAC7-224       DNI.....STPLI.TSRH............................................................................................ 205
HDAC7-214       ..............................................................................................................  51
HDAC7-208       .............................................................................................................. 101
HDAC7-203       QQTFYQDPSVLYISLHRHDDGNFFPGSGAVDEVGAGSGEGFNVNVAWAGGLDPPMGDPEYLAAFRIVVMPIAREFSPDLVLVSAGFDAAEGHPAPLGGYHVSAKCFGYMT 880
HDAC7-205       .............................................................................................................. 133
HDAC7-226       ............................................................................XDLVLVSAGFDAAEGHPAPLGGYHVSAKCFGYMT  34
HDAC7-215       .............................................................................................................. 153
HDAC7-227       QQTFYQDPSVLYISLHRHDDGNFFPGSGAVDEVGAGSGEGFNVNVAWAGGLDPPMGDPEYLAAFRIVVMPIAREFSPDLVLVSAGFDAAEGHPAPLGGYHVSAKCFGYMT 256
HDAC7-206       .............................................................................................................. 138
HDAC7-204       ..............................................................................................................  78
HDAC7-207       .............................................................................................................. 141
HDAC7-212       .............................................................................................................. 153
HDAC7-221       .............................................................................................................. 224
HDAC7-210       .............................................................................................................. 274
HDAC7-209       QQTFYQDPSVLYISLHRHDDGNFFPGSGAVDEVGAGSGEGFNVNVAWAGGLDPPMGDPEYLAAFRIVVMPIAREFSPDLVLVSAGFDAAEGHPAPLGGYHVSAKCFGYMT 824
HDAC7-230       QQTFYQDPSVLYISLHRHDDGNFFPGSGAVDEVGAGSGEGFNVNVAWAGGLDPPMGDPEYLAAFRIVVMPIAREFSPDLVLVSAGFDAAEGHPAPLGGYHVSAKCFGYMT 846
HDAC7-223       .............................................................................................................. 152
HDAC7-211       ..............................................................................................................  88
HDAC7-202       QQTFYQDPSVLYISLHRHDDGNFFPGSGAVDEVGAGSGEGFNVNVAWAGGLDPPMGDPEYLAAFRIVVMPIAREFSPDLVLVSAGFDAAEGHPAPLGGYHVSAKCFGYMT 826
HDAC7-228       ..............................................................................................................  61
HDAC7-216       .............................................................................................................. 112
HDAC7-201       QQTFYQDPSVLYISLHRHDDGNFFPGSGAVDEVGAGSGEGFNVNVAWAGGLDPPMGDPEYLAAFRIVVMPIAREFSPDLVLVSAGFDAAEGHPAPLGGYHVSAKCFGYMT 863
HDAC7-213       .............................................................................................................. 159
```

logo

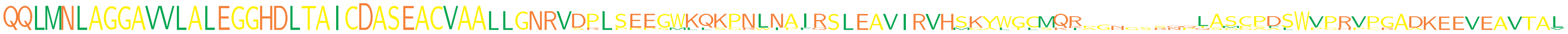

```
VORINOSTAT   .........................D....................................................................................   8
PF00850.1    QQLMNLAGGAVVLALEGGHDLTAICDASEACVAA............................................................................ 152
PF00850      QQLMNLAGGAVVLALEGGHDLTAICDASEACVAA............................................................................ 318
HDAC7-224    .............................................................................................................. 205
HDAC7-214    ..............................................................................................................  51
HDAC7-208    .............................................................................................................. 101
HDAC7-203    QQLMNLAGGAVVLALEGGHDLTAICDASEACVAALLGNRVDPLSEEGWKQKPNLNAIRSLEAVIRVHMGALTLSQIPGHGSSQQQAGGAFSWPGHPCRAA.........V 981
HDAC7-205    .............................................................................................................. 133
HDAC7-226    QQLMNLAGGAVVLALEGGHDLTAICDASEACVAALLGNRVSRLPPPSMLLSGRWIPFQ................KKAGN..RNPTSMPSALW.................. 108
HDAC7-215    .............................................................................................................. 153
HDAC7-227    QQLMNLAGGAVVLALEGGHDLTAICDASEACVAALLGNRVDPLSEEGWKQKPNLNAIRSLEAVIRVHSKYWGCMQR........LASCPDSWVPRVPGADKEEVEAVTAL 358
HDAC7-206    .............................................................................................................. 138
HDAC7-204    ..............................................................................................................  78
HDAC7-207    .............................................................................................................. 141
HDAC7-212    .............................................................................................................. 153
HDAC7-221    .............................................................................................................. 224
HDAC7-210    .............................................................................................................. 274
HDAC7-209    QQLMNLAGGAVVLALEGGHDLTAICDASEACVAALLGNRVDPLSEEGWKQKPNLNAIRSLEAVIRVHSKYWGCMQR........LASCPDSWVPRVPGADKEEVEAVTAL 926
HDAC7-230    QQLMNLAGGAVVLALEGGHDLTAICDASEACVAALLGNRVDPLSEEGWKQKPNLNAIRSLEAVIRVHSKYWGCMQR........LASCPDSWVPRVPGADKEEVEAVTAL 948
HDAC7-223    .............................................................................................................. 152
HDAC7-211    ..............................................................................................................  88
HDAC7-202    QQLMNLAGGAVVLALEGGHDLTAICDASEACVAALLGNRVDPLSEEGWKQKPNLNAIRSLEAVIRVHSKYWGCMQR........LASCPDSWVPRVPGADKEEVEAVTAL 928
HDAC7-228    ..............................................................................................................  61
HDAC7-216    .............................................................................................................. 112
HDAC7-201    QQLMNLAGGAVVLALEGGHDLTAICDASEACVAALLGNRVDPLSEEGWKQKPNLNAIRSLEAVIRVHSKYWGCMQR........LASCPDSWVPRVPGADKEEVEAVTAL 965
HDAC7-213    .............................................................................................................. 159
```

logo

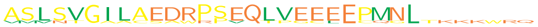

```
VORINOSTAT    .................................     8
PF00850.1     .................................   152
PF00850       .................................   318
HDAC7-224     .................................   205
HDAC7-214     .................................    51
HDAC7-208     .................................   101
HDAC7-203     VMVNTGAACSAWPPVQTPGCLECQGLTKKKWRQ  1014
HDAC7-205     .................................   133
HDAC7-226     ............RP...................   110
HDAC7-215     .................................   153
HDAC7-227     ASLSVGILAEDRPSEQLVEEEEPMNL.......   384
HDAC7-206     .................................   138
HDAC7-204     .................................    78
HDAC7-207     .................................   141
HDAC7-212     .................................   153
HDAC7-221     .................................   224
HDAC7-210     .................................   274
HDAC7-209     ASLSVGILAEDRPSEQLVEEEEPMNL.......   952
HDAC7-230     ASLSVGILAEDRPSEQLVEEEEPMNL.......   974
HDAC7-223     .................................   152
HDAC7-211     .................................    88
HDAC7-202     ASLSVGILAEDRPSEQLVEEEEPMNL.......   954
HDAC7-228     .................................    61
HDAC7-216     .................................   112
HDAC7-201     ASLSVGILAEDRPSEQLVEEEEPMNL.......   991
HDAC7-213     .................................   159
```

X non conserved
X similar
X $\geq$0% conserved
X $\geq$50% conserved

```
logo          MEEPEEPADSGQSLVPVYIYSPEYVSMCDSLAKIPKRASMVHSLIEAYALHKQMRIVKPKVASMEEMATFHTDAYLQHLQKVSQEGDDDHPDSIEYGLGYDCPATEGIFD
TRICHOSTATIN  ................................KIP.R..............................................................YD.........    6
PF00850.1     ..................................PKRASMVHSLIEAYALHKQMRIVKPKVASMEEMATFHTDAYLQHLQKVSQEGDDDHPDSIEYGLGYDCPATEGIFD   76
PF00850.3     ..................................PKRASMVHSLIEAYALHKQMRIVKPKVASMEEMATFHTDAYLQHLQKVSQEGDDDHPDSIEYGLGYDCPATEGIFD   76
PF00850       ..................................PKRASMVHSLIEAYALHKQMRIVKPKVASMEEMATFHTDAYLQHLQKVSQEGDDDHPDSIEYGLGYDCPATEGIFD   76
PF00850.6     ..................................PKRASMVHSLIEAYALHKQMRIVKPKVASMEEMATFHTDAYLQHLQKVSQEGDDDHPDSIEYGLGYDCPATEGIFD   76
PF00850.5     ..................................PKRASMVHSLIEAYALHKQMRIVKPKVASMEEMATFHTDAYLQHLQKVSQEGDDDHPDSIEYGLGYDCPATEGIFD   76
PF00850.9     ..................................PKRASMVHSLIEAYALHKQMRIVKPKVASMEEMATFHTDAYLQHLQKVSQEGDDDHPDSIEYGLGYDCPATEGIFD   76
PF00850.8     .......................................MVHSLIEAYALHKQMRIVKPKVASMEEMATFHTDAYLQHLQKVSQEGDDDHPDSIEYGLGYDCPATEGIFD   71
PF00850.2     ................................................ALHKQMR.......................................................    7
PF00850.4     ................................................ALHKQMR.......................................................    7
PF00850.7     ..................................PKRASMVHSLIEAYALHKQMRIVKPKVASMEEMATFHTDAYLQHLQKVSQEGDDDHPDSIEYGLGYDCPATEGIFD   76
HDAC8-203     MEEPEEPADSGQSLVPVYIYSPEYVSMCDSLAKIPKRASMVHSLIEAYALHKQMR.......................................................   55
HDAC8-211     MEEPEEPADSGQSLVPVYIYSPEYVSMCDSLAKIPKRASMVHSLIEAYALHKQMRIVKPKVASMEEMATFHTDAYLQHLQKVSQEGDDDHPDSIEYGLGYDCPATEGIFD  110
HDAC8-209     MEEPEEPADSGQSLVPVYIYSPEYVSMCDSLAKIPKRASMVHSLIEAYALHKQMR.......................................................   55
HDAC8-207     MEEPEEPADSGQSLVPVYIYSPEYVSMCDSLAKIPKRASMVHSLIEAYALHKQMRIVKPKVASMEEMATFHTDAYLQHLQKVSQEGDDDHPDSIEYGLGYDCPATEGIFD  110
HDAC8-205     MEEPEEPADSGQSLVPVYIYSPEYVSMCDSLAKIPKRASMVHSLIEAYALHKQMR.......................................................   55
HDAC8-215     MEEPEEPADSGQSLVPVYIYSPEYVSMCDSLAKIPKRASMVHSLIEAYALHKQMS.......................................................   55
HDAC8-210     MEEPEEPADSGQSLVPVYIYSPEYVSMCDSLAKIPKRASMVHSLIEAYALHKQMR.......................................................   55
HDAC8-208     MEEPEEPADSGQSLVPVYIYSPEYVSMCDSLAKIPKRASMVHSLIEAYALHKQMR.......................................................   55
HDAC8-214     MEEPEEPADSGQSLVPVYIYSPEYVSMCDSLAKIPKRASMVHSLIEAYALHKQMRIVKPKVASMEEMATFHTDAYLQHLQKVSQEGDDDHPDSIEYGLGYDCPATEGIFD  110
HDAC8-202     MEEPEEPADSGQSLVPVYIYSPEYVSMCDSLAKIPKRASMVHSLIEAYALHKQMRIVKPKVASMEEMATFHTDAYLQHLQKVSQEGDDDHPDSIEYGLGYDCPATEGIFD  110
HDAC8-213     MEEPEEPADSGQSLVPVYIYSPEYVSMCDSLAKIPKRASMVHSLIEAYALHKQMRIVKPKVASMEEMATFHTDAYLQHLQKVSQEGDDDHPDSIEYGLGYDCPATEGIFD  110
HDAC8-206     MEEPEEPADSGQSLVPVYIYSPEYVSMCDSLAKIPKRASMVHSLIEAYALHKQMRIVKPKVASMEEMATFHTDAYLQHLQKVSQEGDDDHPDSIEYGLGYDCPATEGIFD  110
HDAC8-204     MEEPEEPADSGQSLVPVYIYSPEYVSMCDSLAKIPKRASMVHSLIEAYALHKQMRIVKPKVASMEEMATFHTDAYLQHLQKVSQEGDDDHPDSIEYGLGYDCPATEGIFD  110
HDAC8-201     MEEPEEPADSGQSLVPVYIYSPEYVSMCDSLAKIPKRASMVHSLIEAYALHKQMRIVKPKVASMEEMATFHTDAYLQHLQKVSQEGDDDHPDSIEYGLGYDCPATEGIFD  110
HDAC8-212     .......................................MVHSLIEAYALHKQMRIVKPKVASMEEMATFHTDAYLQHLQKVSQEGDDDHPDSIEYGLGYDCPATEGIFD   71
```

logo 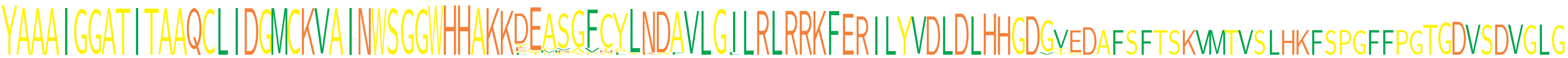

```
TRICHOSTATIN  Y.............................WHH.......GF.........................D.H........................................  14
PF00850.1     YAAAIGGATITAAQCLIDGMCKVAINWSGGWHHAKKE......................................................................... 113
PF00850.3     YAAAIGGATITAAQCLIDGMCKVAINWSGGWHHAKKYE........................................................................ 114
PF00850       YAAAIGGATITAAQCLIDGMCKVAINWSGGWHHAK........................................................................... 111
PF00850.6     YAAAIGGATITAAQCLIDGMCKVAINWSGGWHHAKKDEASGFCYLNDAVLGILRLRRKFERILYVDLDLHHGDGVEDAFSFTSKVMTVSLHKFSPGFFPGTGDVSDVGLG 186
PF00850.5     YAAAIGGATITAAQCLIDGMCKVAINWSGGWHHAKKDEASGFCYLNDAVLGILRLRRKFERILYVDLDLHHGDGVEDAFSFTSKVMTVSLHKFSPGFFPGTGDVSDVGLG 186
PF00850.9     YAAAIGGATITAAQCLIDGMCKVAINWSGGWHHAKKDEASGFCYLNDAVLGILRLRRKFERILYVDLDLHHGDGVEDAFSFTSKVMTVSLHKFSPGFFPGTGDVSDVGLG 186
PF00850.8     YAAAIGGATITAAQCLIDGMCKVAINWSGGWHHAKKDEASGFCYLNDAVLGILRLRRKF................................................... 130
PF00850.2     ....................................DEASGFCYLNDAVLGILRLRRKFERILYVDLDLHHGDG..........................TGD.......  48
PF00850.4     ....................................DEASGFCYLNDAVLGILRLRRKFERILYVDLDLHHGDGVEDAFSFTSKVMTVSLHKFSPGFFPGTGDVSDVGLG  81
PF00850.7     YAAAIGGATITAAQCLIDGMCKVAINWSGGWHHAKKDEASGFCYLNDAVLGILRLRRKFERILYVDLDLHHGDGTGD................................. 153
HDAC8-203     ....................................DEASGFCYLNDAVLGILRLRRKFERILYVDLDLHHGDG..........................TGDVSDVGLG 103
HDAC8-211     YAAAIGGATITAAQCLIDGMCKVAINWSGGWHHAKKDEASGFCYLNDAVLGILRLRRKFERILYVDLDLHHGDGT..........................GDVSDVGLG 194
HDAC8-209     ....................................DEASGFCYLNDAVLGILRLRRKFERILYVDLDLHHGDGVEDAFSFTSKVMTVSLHKFSPGFFPGTGDVSDVGLG 129
HDAC8-207     YAAAIGGATITAAQCLIDGMCKVAINWSGGWHHAKKDEASGFCYLNDAVLGILRLRRKFERILYVDLDLHHGDGVEDAFSFTSKVMTVSLHKFSPGFFPGTGDVSDVGLG 220
HDAC8-205     ....................................DEASGFCYLNDAVLGILRLRRKFERILYVDLDLHHGDGVEDAFSFTSKVMTVSLHKFSPGFFPGTGDVSDVGLG 129
HDAC8-215     ..............................................................................................................  55
HDAC8-210     ....................................L.........................................................................  56
HDAC8-208     ....................................SWMRAVTSL.................................................................  64
HDAC8-214     YAAAIGGATITAAQCLIDGMCKVAINWSGGWHHAKKDEASGFCYLNDAVLGILRLRRKFERILYVDLDLHHGDGVEDAFSFTSKVMTVSLHKFSPGFFPGTGDVSDVGLG 220
HDAC8-202     YAAAIGGATITAAQCLIDGMCKVAINWSGGWHHAKKETCVYVALYKAF.............................................................. 158
HDAC8-213     YAAAIGGATITAAQCLIDGMCKVAINWSGGWHHAKKDEASGFCYLNDAVLGILRLRRKFERILYVDLDLHHGDVY................................... 185
HDAC8-206     YAAAIGGATITAAQCLIDGMCKVAINWSGGWHHAKKDEASGFCYLNDAVLGILRLRRKFERILYVDLDLHHGDGVEDAFSFTSKVMTVSLHKFSPGFFPGTGDVSDVGLG 220
HDAC8-204     YAAAIGGATITAAQCLIDGMCKVAINWSGGWHHAKKYEPPAPNP......GL.......................................................... 156
HDAC8-201     YAAAIGGATITAAQCLIDGMCKVAINWSGGWHHAKK.......................................................................... 146
HDAC8-212     YAAAIGGATITAAQCLIDGMCKVAINWSGGWHHAKKDEASGFCYLNDAVLGILRLRRKFE.................................................. 131
```

logo

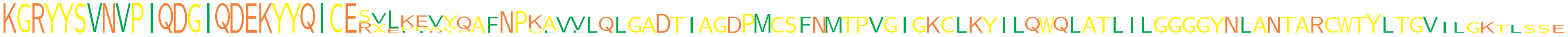

```
TRICHOSTATIN  ..............................................D.....PM..........................................Y.............   18
PF00850.1     ..............................................................................................................  113
PF00850.3     ..............................................................................................................  114
PF00850       ..............................................................................................................  111
PF00850.6     KGRYYSVNVPIQDGIQDEKYYQICESVLKEVYQAFNPKAVVLQLGADTIAGDPMCSFNMTPVGIGKCLKYILQWQLATLILGGGGYNLANTARCWTYLTGV.........  287
PF00850.5     KGRYYSVNVPIQDGIQDEKYYQICERAL..................................................................................  214
PF00850.9     KGRYYSVNVPIQDGIQDEKYYQICERY...................................................................................  213
PF00850.8     ..............................................................................................................  130
PF00850.2     ..............................................................................................................   48
PF00850.4     KGRYYSVNVPIQDGIQDEKYYQICESVLKEVYQAFNPKAVVLQLGADTIAGDPMCSFNMTPVGIGKCLKYILQWQLATLILGGGGYNLANTARCWTYLTGV.........  182
PF00850.7     ..............................................................................................................  153
HDAC8-203     KGRYYSVNVPIQDGIQDEKYYQICERYEPPAP...NPGL.......................................................................  139
HDAC8-211     KGRYYSVNVPIQDGIQDEKYYQICESVLKEVYQAFNPKAVVLQLGADTIAGDPMCSFNMTPVGIGKCLKYILQWQLATLILGGGGYNLANTARCWTYLTGVILGK.....  299
HDAC8-209     KGRYYSVNVPIQDGIQDEKYYQICESVLKEVYQAFNPKAVVLQLGADTIAGDPMCSFNMTPVGIGKCLKYILQWQLATLILGGGGYNLANTARCWTYLTGVILGKTLSSE  239
HDAC8-207     KGRYYSVNVPIQDGIQDEKYYQICESVLKEVYQAFNPKAVVLQLGADTIAGDPMCSFNMTPVGIGKCLKYILQWQLATLILGGGGYNLANTARCWTYLTGVILGKTLSSE  330
HDAC8-205     KGRYYSVNVPIQDGIQDEKYYQICESVLKEVYQAFNPKAVVLQLGADTIAGDPMCSFNMTPVGIGKCLKYILQWQLATLILGGGGYNLANTARCWTYLTGVILGKTLSSE  239
HDAC8-215     ..............................................................................................................   55
HDAC8-210     ..............................................................................................................   56
HDAC8-208     ..............................................................................................................   64
HDAC8-214     KGRYYSVNVPIQDGIQDEKYYQICERYEPPAP...NPG..L.....................................................................  256
HDAC8-202     ..............................................................................................................  158
HDAC8-213     ..............................................................................................................  185
HDAC8-206     KGRYYSVNVPIQDGIQDEKYYQICERALFIRTP.............................................................................  253
HDAC8-204     ..............................................................................................................  156
HDAC8-201     ..............................................................................................................  146
HDAC8-212     ..............................................................................................................  131
```

logo

```
              IPDHEFFTAYGPDYVLEITPSCRPDRNEPHRIQQILNYIKGNLKHVV
TRICHOSTATIN  ...............................................   18
PF00850.1     ...............................................  113
PF00850.3     ...............................................  114
PF00850       ...............................................  111
PF00850.6     ...............................................  287
PF00850.5     ...............................................  214
PF00850.9     ...............................................  213
PF00850.8     ...............................................  130
PF00850.2     ...............................................   48
PF00850.4     ...............................................  182
PF00850.7     ...............................................  153
HDAC8-203     ...............................................  139
HDAC8-211     ...............................................  299
HDAC8-209     IPDHEFFTAYGPDYVLEITPSCRPDRNEPHRIQQILNYIKGNLKHVV  286
HDAC8-207     IPDHEFFTAYGPDYVLEITPSCRPDRNEPHRIQQILNYIKGNLKHVV  377
HDAC8-205     IPDHEFFTA......................................  248
HDAC8-215     ...............................................   55
HDAC8-210     ...............................................   56
HDAC8-208     ...............................................   64
HDAC8-214     ...............................................  256
HDAC8-202     ...............................................  158
HDAC8-213     ...............................................  185
HDAC8-206     ...............................................  253
HDAC8-204     ...............................................  156
HDAC8-201     ...............................................  146
HDAC8-212     ...............................................  131
```

X non conserved
X similar
X ≥ 0% conserved
X ≥ 50% conserved

```
logo        MEEPEEPADSGQSLVPVYIYSPEYVSMCDSLAKIPKRASMVHSLIEAYALHKQMRIVKPKVASMEEMATFHTDAYLQHLQKVSQEGDDDHPDSIEYGLGYDCPATEGIFD
VORINOSTAT  .......................Y......................................................................................   1
PF00850.1   ..................................PKRASMVHSLIEAYALHKQMRIVKPKVASMEEMATFHTDAYLQHLQKVSQEGDDDHPDSIEYGLGYDCPATEGIFD  76
PF00850.3   ..................................PKRASMVHSLIEAYALHKQMRIVKPKVASMEEMATFHTDAYLQHLQKVSQEGDDDHPDSIEYGLGYDCPATEGIFD  76
PF00850     ..................................PKRASMVHSLIEAYALHKQMRIVKPKVASMEEMATFHTDAYLQHLQKVSQEGDDDHPDSIEYGLGYDCPATEGIFD  76
PF00850.6   ..................................PKRASMVHSLIEAYALHKQMRIVKPKVASMEEMATFHTDAYLQHLQKVSQEGDDDHPDSIEYGLGYDCPATEGIFD  76
PF00850.5   ..................................PKRASMVHSLIEAYALHKQMRIVKPKVASMEEMATFHTDAYLQHLQKVSQEGDDDHPDSIEYGLGYDCPATEGIFD  76
PF00850.9   ..................................PKRASMVHSLIEAYALHKQMRIVKPKVASMEEMATFHTDAYLQHLQKVSQEGDDDHPDSIEYGLGYDCPATEGIFD  76
PF00850.8   .......................................MVHSLIEAYALHKQMRIVKPKVASMEEMATFHTDAYLQHLQKVSQEGDDDHPDSIEYGLGYDCPATEGIFD  71
PF00850.2   ................................................ALHKQMR.......................................................   7
PF00850.4   ................................................ALHKQMR.......................................................   7
PF00850.7   ..................................PKRASMVHSLIEAYALHKQMRIVKPKVASMEEMATFHTDAYLQHLQKVSQEGDDDHPDSIEYGLGYDCPATEGIFD  76
HDAC8-203   MEEPEEPADSGQSLVPVYIYSPEYVSMCDSLAKIPKRASMVHSLIEAYALHKQMR.......................................................  55
HDAC8-211   MEEPEEPADSGQSLVPVYIYSPEYVSMCDSLAKIPKRASMVHSLIEAYALHKQMRIVKPKVASMEEMATFHTDAYLQHLQKVSQEGDDDHPDSIEYGLGYDCPATEGIFD 110
HDAC8-209   MEEPEEPADSGQSLVPVYIYSPEYVSMCDSLAKIPKRASMVHSLIEAYALHKQMR.......................................................  55
HDAC8-207   MEEPEEPADSGQSLVPVYIYSPEYVSMCDSLAKIPKRASMVHSLIEAYALHKQMRIVKPKVASMEEMATFHTDAYLQHLQKVSQEGDDDHPDSIEYGLGYDCPATEGIFD 110
HDAC8-205   MEEPEEPADSGQSLVPVYIYSPEYVSMCDSLAKIPKRASMVHSLIEAYALHKQMR.......................................................  55
HDAC8-215   MEEPEEPADSGQSLVPVYIYSPEYVSMCDSLAKIPKRASMVHSLIEAYALHKQMS.......................................................  55
HDAC8-210   MEEPEEPADSGQSLVPVYIYSPEYVSMCDSLAKIPKRASMVHSLIEAYALHKQMR.......................................................  55
HDAC8-208   MEEPEEPADSGQSLVPVYIYSPEYVSMCDSLAKIPKRASMVHSLIEAYALHKQMR.......................................................  55
HDAC8-214   MEEPEEPADSGQSLVPVYIYSPEYVSMCDSLAKIPKRASMVHSLIEAYALHKQMRIVKPKVASMEEMATFHTDAYLQHLQKVSQEGDDDHPDSIEYGLGYDCPATEGIFD 110
HDAC8-202   MEEPEEPADSGQSLVPVYIYSPEYVSMCDSLAKIPKRASMVHSLIEAYALHKQMRIVKPKVASMEEMATFHTDAYLQHLQKVSQEGDDDHPDSIEYGLGYDCPATEGIFD 110
HDAC8-213   MEEPEEPADSGQSLVPVYIYSPEYVSMCDSLAKIPKRASMVHSLIEAYALHKQMRIVKPKVASMEEMATFHTDAYLQHLQKVSQEGDDDHPDSIEYGLGYDCPATEGIFD 110
HDAC8-206   MEEPEEPADSGQSLVPVYIYSPEYVSMCDSLAKIPKRASMVHSLIEAYALHKQMRIVKPKVASMEEMATFHTDAYLQHLQKVSQEGDDDHPDSIEYGLGYDCPATEGIFD 110
HDAC8-204   MEEPEEPADSGQSLVPVYIYSPEYVSMCDSLAKIPKRASMVHSLIEAYALHKQMRIVKPKVASMEEMATFHTDAYLQHLQKVSQEGDDDHPDSIEYGLGYDCPATEGIFD 110
HDAC8-201   MEEPEEPADSGQSLVPVYIYSPEYVSMCDSLAKIPKRASMVHSLIEAYALHKQMRIVKPKVASMEEMATFHTDAYLQHLQKVSQEGDDDHPDSIEYGLGYDCPATEGIFD 110
HDAC8-212   .......................................MVHSLIEAYALHKQMRIVKPKVASMEEMATFHTDAYLQHLQKVSQEGDDDHPDSIEYGLGYDCPATEGIFD  71
```

logo

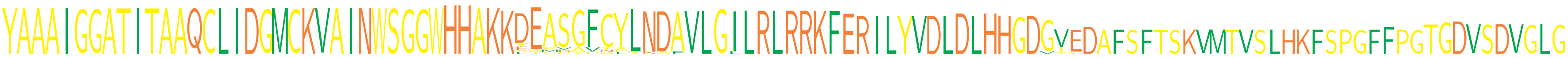

```
VORINOSTAT  ...............................HH.......GF.........................D.H...........................F............  8
PF00850.1   YAAAIGGATITAAQCLIDGMCKVAINWSGGWHHAKKE.........................................................................  113
PF00850.3   YAAAIGGATITAAQCLIDGMCKVAINWSGGWHHAKKYE........................................................................  114
PF00850     YAAAIGGATITAAQCLIDGMCKVAINWSGGWHHAK...........................................................................  111
PF00850.6   YAAAIGGATITAAQCLIDGMCKVAINWSGGWHHAKKDEASGFCYLNDAVLGILRLRRKFERILYVDLDLHHGDGVEDAFSFTSKVMTVSLHKFSPGFFPGTGDVSDVGLG  186
PF00850.5   YAAAIGGATITAAQCLIDGMCKVAINWSGGWHHAKKDEASGFCYLNDAVLGILRLRRKFERILYVDLDLHHGDGVEDAFSFTSKVMTVSLHKFSPGFFPGTGDVSDVGLG  186
PF00850.9   YAAAIGGATITAAQCLIDGMCKVAINWSGGWHHAKKDEASGFCYLNDAVLGILRLRRKFERILYVDLDLHHGDGVEDAFSFTSKVMTVSLHKFSPGFFPGTGDVSDVGLG  186
PF00850.8   YAAAIGGATITAAQCLIDGMCKVAINWSGGWHHAKKDEASGFCYLNDAVLGILRLRRKF...................................................  130
PF00850.2   ....................................DEASGFCYLNDAVLGILRLRRKFERILYVDLDLHHGDG..........................TGD.......  48
PF00850.4   ....................................DEASGFCYLNDAVLGILRLRRKFERILYVDLDLHHGDGVEDAFSFTSKVMTVSLHKFSPGFFPGTGDVSDVGLG  81
PF00850.7   YAAAIGGATITAAQCLIDGMCKVAINWSGGWHHAKKDEASGFCYLNDAVLGILRLRRKFERILYVDLDLHHGDGTGD.................................  153
HDAC8-203   ....................................DEASGFCYLNDAVLGILRLRRKFERILYVDLDLHHGDG..........................TGDVSDVGLG  103
HDAC8-211   YAAAIGGATITAAQCLIDGMCKVAINWSGGWHHAKKDEASGFCYLNDAVLGILRLRRKFERILYVDLDLHHGDGT..........................GDVSDVGLG  194
HDAC8-209   ....................................DEASGFCYLNDAVLGILRLRRKFERILYVDLDLHHGDGVEDAFSFTSKVMTVSLHKFSPGFFPGTGDVSDVGLG  129
HDAC8-207   YAAAIGGATITAAQCLIDGMCKVAINWSGGWHHAKKDEASGFCYLNDAVLGILRLRRKFERILYVDLDLHHGDGVEDAFSFTSKVMTVSLHKFSPGFFPGTGDVSDVGLG  220
HDAC8-205   ....................................DEASGFCYLNDAVLGILRLRRKFERILYVDLDLHHGDGVEDAFSFTSKVMTVSLHKFSPGFFPGTGDVSDVGLG  129
HDAC8-215   ..............................................................................................................  55
HDAC8-210   ....................................L.........................................................................  56
HDAC8-208   ....................................SWMRAVTSL.................................................................  64
HDAC8-214   YAAAIGGATITAAQCLIDGMCKVAINWSGGWHHAKKDEASGFCYLNDAVLGILRLRRKFERILYVDLDLHHGDGVEDAFSFTSKVMTVSLHKFSPGFFPGTGDVSDVGLG  220
HDAC8-202   YAAAIGGATITAAQCLIDGMCKVAINWSGGWHHAKKETCVYVALYKAF..............................................................  158
HDAC8-213   YAAAIGGATITAAQCLIDGMCKVAINWSGGWHHAKKDEASGFCYLNDAVLGILRLRRKFERILYVDLDLHHGDVY...................................  185
HDAC8-206   YAAAIGGATITAAQCLIDGMCKVAINWSGGWHHAKKDEASGFCYLNDAVLGILRLRRKFERILYVDLDLHHGDGVEDAFSFTSKVMTVSLHKFSPGFFPGTGDVSDVGLG  220
HDAC8-204   YAAAIGGATITAAQCLIDGMCKVAINWSGGWHHAKKYEPPAPNP......GL..........................................................  156
HDAC8-201   YAAAIGGATITAAQCLIDGMCKVAINWSGGWHHAKK..........................................................................  146
HDAC8-212   YAAAIGGATITAAQCLIDGMCKVAINWSGGWHHAKKDEASGFCYLNDAVLGILRLRRKFE..................................................  131
```

logo

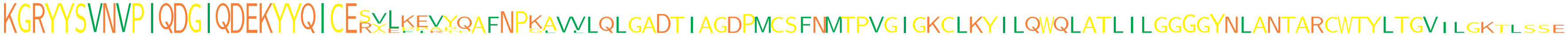

```
VORINOSTAT   ..............................................D.....P..............................G.Y........................  12
PF00850.1    ..............................................................................................................  113
PF00850.3    ..............................................................................................................  114
PF00850      ..............................................................................................................  111
PF00850.6    KGRYYSVNVPIQDGIQDEKYYQICESVLKEVYQAFNPKAVVLQLGADTIAGDPMCSFNMTPVGIGKCLKYILQWQLATLILGGGGYNLANTARCWTYLTGV..........  287
PF00850.5    KGRYYSVNVPIQDGIQDEKYYQICERAL..................................................................................  214
PF00850.9    KGRYYSVNVPIQDGIQDEKYYQICERY...................................................................................  213
PF00850.8    ..............................................................................................................  130
PF00850.2    ..............................................................................................................  48
PF00850.4    KGRYYSVNVPIQDGIQDEKYYQICESVLKEVYQAFNPKAVVLQLGADTIAGDPMCSFNMTPVGIGKCLKYILQWQLATLILGGGGYNLANTARCWTYLTGV..........  182
PF00850.7    ..............................................................................................................  153
HDAC8-203    KGRYYSVNVPIQDGIQDEKYYQICERYEPPAP...NPGL.......................................................................  139
HDAC8-211    KGRYYSVNVPIQDGIQDEKYYQICESVLKEVYQAFNPKAVVLQLGADTIAGDPMCSFNMTPVGIGKCLKYILQWQLATLILGGGGYNLANTARCWTYLTGVILGK.....  299
HDAC8-209    KGRYYSVNVPIQDGIQDEKYYQICESVLKEVYQAFNPKAVVLQLGADTIAGDPMCSFNMTPVGIGKCLKYILQWQLATLILGGGGYNLANTARCWTYLTGVILGKTLSSE  239
HDAC8-207    KGRYYSVNVPIQDGIQDEKYYQICESVLKEVYQAFNPKAVVLQLGADTIAGDPMCSFNMTPVGIGKCLKYILQWQLATLILGGGGYNLANTARCWTYLTGVILGKTLSSE  330
HDAC8-205    KGRYYSVNVPIQDGIQDEKYYQICESVLKEVYQAFNPKAVVLQLGADTIAGDPMCSFNMTPVGIGKCLKYILQWQLATLILGGGGYNLANTARCWTYLTGVILGKTLSSE  239
HDAC8-215    ..............................................................................................................  55
HDAC8-210    ..............................................................................................................  56
HDAC8-208    ..............................................................................................................  64
HDAC8-214    KGRYYSVNVPIQDGIQDEKYYQICERYEPPAP...NPG..L.....................................................................  256
HDAC8-202    ..............................................................................................................  158
HDAC8-213    ..............................................................................................................  185
HDAC8-206    KGRYYSVNVPIQDGIQDEKYYQICERALFIRTP.............................................................................  253
HDAC8-204    ..............................................................................................................  156
HDAC8-201    ..............................................................................................................  146
HDAC8-212    ..............................................................................................................  131
```

logo

```
             IPDHEFFTAYGPDYVLEITPSCRPDRNEPHRIQQILNYIKGNLKHVV
VORINOSTAT   ...............................................   12
PF00850.1    ...............................................  113
PF00850.3    ...............................................  114
PF00850      ...............................................  111
PF00850.6    ...............................................  287
PF00850.5    ...............................................  214
PF00850.9    ...............................................  213
PF00850.8    ...............................................  130
PF00850.2    ...............................................   48
PF00850.4    ...............................................  182
PF00850.7    ...............................................  153
HDAC8-203    ...............................................  139
HDAC8-211    ...............................................  299
HDAC8-209    IPDHEFFTAYGPDYVLEITPSCRPDRNEPHRIQQILNYIKGNLKHVV  286
HDAC8-207    IPDHEFFTAYGPDYVLEITPSCRPDRNEPHRIQQILNYIKGNLKHVV  377
HDAC8-205    IPDHEFFTA......................................  248
HDAC8-215    ...............................................   55
HDAC8-210    ...............................................   56
HDAC8-208    ...............................................   64
HDAC8-214    ...............................................  256
HDAC8-202    ...............................................  158
HDAC8-213    ...............................................  185
HDAC8-206    ...............................................  253
HDAC8-204    ...............................................  156
HDAC8-201    ...............................................  146
HDAC8-212    ...............................................  131
```

X non conserved
X similar
X $\geq$ 0% conserved
X $\geq$ 50% conserved

logo
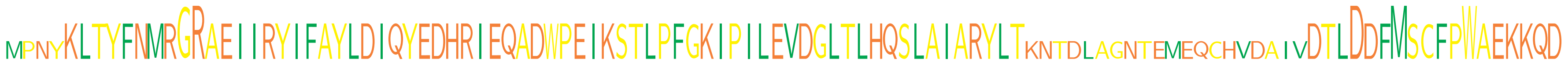

```
NOCODAZOLE  ............GR.................................................................................D..M....W......      5
PF14497     ............................................................................................DTLDDFMSCFPWAEKKQD     18
PF02798     ....KLTYFNMRGRAEIIRYIFAYLDIQYEDHRIEQADWPEIKSTLPFGKIPILEVDGLTLHQSLAIARYLT......................................     68
HPGDS-201   MPNYKLTYFNMRGRAEIIRYIFAYLDIQYEDHRIEQADWPEIKSTLPFGKIPILEVDGLTLHQSLAIARYLTKNTDLAGNTEMEQCHVDAIVDTLDDFMSCFPWAEKKQD    110
```

logo
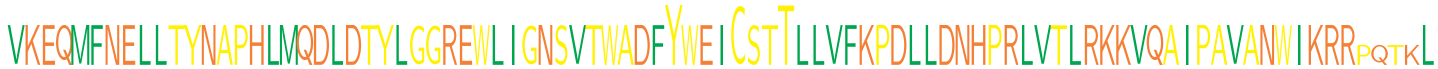

```
NOCODAZOLE  .........................................Y...C..T.......................................L      9
PF14497     VKEQMFNELLTYNAPHLMQDLDTYLGGREWLIGNSVTWADFYWEICSTTLLVFKPDLLDNHPRLVTLRKKVQAIPAVANWIKRR.....    102
PF02798     .........................................................................................     68
HPGDS-201   VKEQMFNELLTYNAPHLMQDLDTYLGGREWLIGNSVTWADFYWEICSTTLLVFKPDLLDNHPRLVTLRKKVQAIPAVANWIKRRPQTKL    199
```

- X non conserved
- X similar
- X $\geq 0\%$ conserved
- X $\geq 50\%$ conserved

logo

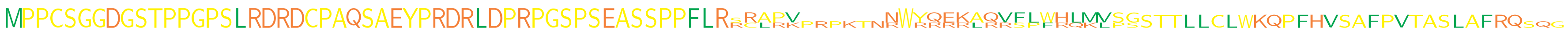

```
ALVESPIMYCIN  ..............................................................................................................    0
PF00183       ..............................................................................................................    0
PF02518       ..............................................................................................................    0
PF02518.1     ..............................................................................................................    0
PF00183.1     ..............................................................................................................    0
HSP90AA1-203  ..............................................................................................................    0
HSP90AA1-209  MPPCSGGDGSTPPGPSLRDRDCPAQSAEYPRDRLDPRPGSPSEASSPPFLR.RAPV......NWYQEKAQVFLWHLMVSGSTTLLCLWKQPFHVSAFPVTASLAFRQ...  100
HSP90AA1-208  MPPCSGGDGSTPPGPSLRDRDCPAQSAEYPRDRLDPRPGSPSEASSPPFLRRCLRKPRPKTNRWRRRRLRRSPFRQKLPS..............................   80
HSP90AA1-202  MPPCSGGDGSTPPGPSLRDRDCPAQSAEYPRDRLDPRPGSPSEASSPPFLRSRAPV......NWYQEKAQVFLWHLMVSGSTTLLCLWKQPFHVSAFPVTASLAFRQSQG  104
HSP90AA1-201  ..............................................................................................................    0
HSP90AA1-204  ..............................................................................................................    0
```

logo

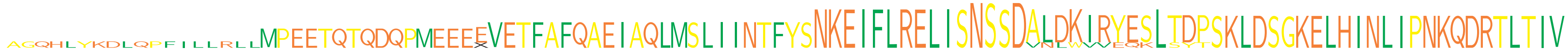

```
ALVESPIMYCIN  ....................................................................NS.DA..K..................................    5
PF00183       ..............................................................................................................    0
PF02518       .........................................................NKEIFLRELISNSSDALDKIRYESLTDPSKLDSGKELHINLIPNKQDRTLTIV   53
PF02518.1     .........................................................NKEIFLRELISNSSDALDKIRYESLTDPSKLDSGKELHINLIPNKQDRTLTIV   53
PF00183.1     ..............................................................................................................    0
HSP90AA1-203  ..................MPEETQTQDQPMEEEEVETFAFQAEIAQLMSLIINTFYSNKEIFLRELISNSSDALDKIRYESLTDPSKLDSGKELHINLIPNKQDRTLTIV   92
HSP90AA1-209  ..............................................................................................................  100
HSP90AA1-208  ..............................................................................................................   80
HSP90AA1-202  AGQHLYKDLQPFILLRLLMPEETQTQDQPMEEEEVETFAFQAEIAQLMSLIINTFYSNKEIFLRELISNSSDALDKIRYESLTDPSKLDSGKELHINLIPNKQDRTLTIV  214
HSP90AA1-201  ..................MPEETQTQDQPMEEEEVETFAFQAEIAQLMSLIINTFYSNKEIFLRELISNSSDALDKIRYESLTDPSKLDSGKELHINLIPNKQDRTLTIV   92
HSP90AA1-204  .................................XVETFAFQAEIAQLMSLIINTFYSNKEIFLRELISNSSDVNLWVVEQKLSYT.........................   52
```

logo

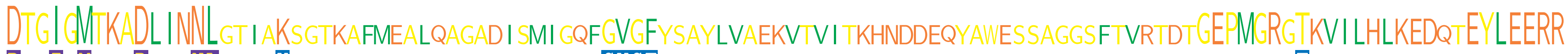

```
ALVESPIMYCIN  D..I.M...D...NL....K......................GVGF.............................................T..................   17
PF00183       .......................................................................................................EYLEERR    7
PF02518       DTGIGMTKADLINNLGTIAKSGTKAFMEALQAGADISMIGQFGVGFYSAYLVAEKVTVITKHNDDEQYAWESSAGGSFTVRTDTGEPMGRGTKVILHLKED.........  154
PF02518.1     DTGIGMTKADLINNL.....................................................................GEPMGR....................   74
PF00183.1     .......................................................................................................EYLEERR    7
HSP90AA1-203  DTGIGMTKADLINNL.....................................................................GEPMGRGTKVILHLKEDQTEYLEERR  133
HSP90AA1-209  ..............................................................................................................  100
HSP90AA1-208  ..............................................................................................................   80
HSP90AA1-202  DTGIGMTKADLINNLGTIAKSGTKAFMEALQAGADISMIGQFGVGFYSAYLVAEKVTVITKHNDDEQYAWESSAGGSFTVRTDTGEPMGRGTKVILHLKEDQTEYLEERR  324
HSP90AA1-201  DTGIGMTKADLINNLGTIAKSGTKAFMEALQAGADISMIGQFGVGFYSAYLVAEKVTVITKHNDDEQYAWESSAGGSFTVRTDTGEPMGRGTKVILHLKEDQTEYLEERR  202
HSP90AA1-204  ..............................................................................................................   52
```

```
logo
                IKEIVKKHSQFIGYPITLFVEKERDKEVSDDEAEEKEDKEEEKEKEEKESEDKPEIEDVGSDEEEEKKDGDKKKKKKIKEKYIDQEELNKTKPIWTRNPDDITNEEYGEF
ALVESPIMYCIN    ..............................................................................................................   17
PF00183         IKEIVKKHSQFIGYPITLFVEKERDKEVSDDEAEEKEDKEEEKEKEEKESEDKPEIEDVGSDEEEEKKDGDKKKKKKIKEKYIDQEELNKTKPIWTRNPDDITNEEYGEF  117
PF02518         ..............................................................................................................  154
PF02518.1       ..............................................................................................................   74
PF00183.1       IKEIVKKHSQFIGYPITLFVEKERDKEVSDDEAEEKEDKE......................................................................   47
HSP90AA1-203    IKEIVKKHSQFIGYPITLFVEKERDKEVSDDEAEEKEDKEE.....................................................................  174
HSP90AA1-209    ..............................................................................................................  100
HSP90AA1-208    ..............................................................................................................   80
HSP90AA1-202    IKEIVKKHSQFIGYPITLFVEKERDKEVSDDEAEEKEDKEEEKEKEEKESEDKPEIEDVGSDEEEEKKDGDKKKKKKIKEKYIDQEELNKTKPIWTRNPDDITNEEYGEF  434
HSP90AA1-201    IKEIVKKHSQFIGYPITLFVEKERDKEVSDDEAEEKEDKEEEKEKEEKESEDKPEIEDVGSDEEEEKKDGDKKKKKKIKEKYIDQEELNKTKPIWTRNPDDITNEEYGEF  312
HSP90AA1-204    ..............................................................................................................   52

logo
                YKSLTNDWEDHLAVKHFSVEGQLEFRALLFVPRRAPFDLFENRKKKNNIKLYVRRVFIMDNCEELIPEYLNFIRGVVDSEDLPLNISREMLQQSKILKVIRKNLVKKCLE
ALVESPIMYCIN    ..............................................................................................................   17
PF00183         YKSLTNDWEDHLAVKHFSVEGQLEFRALLFVPRRAPFDLFENRKKKNNIKLYVRRVFIMDNCEELIPEYLNFIRGVVDSEDLPLNISREMLQQSKILKVIRKNLVKKCLE  227
PF02518         ..............................................................................................................  154
PF02518.1       ..............................................................................................................   74
PF00183.1       ..............................................................................................................   47
HSP90AA1-203    ..............................................................................................................  174
HSP90AA1-209    ..............................................................................................................  100
HSP90AA1-208    ..............................................................................................................   80
HSP90AA1-202    YKSLTNDWEDHLAVKHFSVEGQLEFRALLFVPRRAPFDLFENRKKKNNIKLYVRRVFIMDNCEELIPEYLNFIRGVVDSEDLPLNISREMLQQSKILKVIRKNLVKKCLE  544
HSP90AA1-201    YKSLTNDWEDHLAVKHFSVEGQLEFRALLFVPRRAPFDLFENRKKKNNIKLYVRRVFIMDNCEELIPEYLNFIRGVVDSEDLPLNISREMLQQSKILKVIRKNLVKKCLE  422
HSP90AA1-204    ..............................................................................................................   52

logo
                LFTELAEDKENYKKFYEQFSKNIKLGIHEDSQNRKKLSELLRYYTSASGDEMVSLKDYCTRMKENQKHIYYITGETKDQVANSAFVERLRKHGLEVIYMIEPIDEYCVQQ
ALVESPIMYCIN    ..............................................................................................................   17
PF00183         LFTELAEDKENYKKFYEQFSKNIKLGIHEDSQNRKKLSELLRYYTSASGDEMVSLKDYCTRMKENQKHIYYITGETKDQVANSAFVERLRKHGLEVIYMIEPIDEYCVQQ  337
PF02518         ..............................................................................................................  154
PF02518.1       ..............................................................................................................   74
PF00183.1       ..............................................................................................................   47
HSP90AA1-203    ..............................................................................................................  174
HSP90AA1-209    ..............................................................................................................  100
HSP90AA1-208    ..............................................................................................................   80
HSP90AA1-202    LFTELAEDKENYKKFYEQFSKNIKLGIHEDSQNRKKLSELLRYYTSASGDEMVSLKDYCTRMKENQKHIYYITGETKDQVANSAFVERLRKHGLEVIYMIEPIDEYCVQQ  654
HSP90AA1-201    LFTELAEDKENYKKFYEQFSKNIKLGIHEDSQNRKKLSELLRYYTSASGDEMVSLKDYCTRMKENQKHIYYITGETKDQVANSAFVERLRKHGLEVIYMIEPIDEYCVQQ  532
HSP90AA1-204    ..............................................................................................................   52
```

```
logo
               LKEFEGKTLVSVTKEGLELPEDEEEKKKQEEKKTKFENLCKIMKDILEKKVEKVVVSNRLVTSPCCIVTSTYGWTANMERIMKAQALRDNSTMGYMAAKKHLEINPDHSI
ALVESPIMYCIN   ..................................................................................................................   17
PF00183        LKEFEGKTLVSVTKEGLELPEDEEEKKKQEEKKTKFENLCKIMKDILEKKVEKVVVSNRLVTSPCCIVTSTYGWTANMERIMKAQALRDNSTMGYMAAKKHLEINPDHSI  447
PF02518        ..................................................................................................................  154
PF02518.1      ..................................................................................................................   74
PF00183.1      ..................................................................................................................   47
HSP90AA1-203   ..................................................................................................................  174
HSP90AA1-209   ..................................................................................................................  100
HSP90AA1-208   ..................................................................................................................   80
HSP90AA1-202   LKEFEGKTLVSVTKEGLELPEDEEEKKKQEEKKTKFENLCKIMKDILEKKVEKVVVSNRLVTSPCCIVTSTYGWTANMERIMKAQALRDNSTMGYMAAKKHLEINPDHSI  764
HSP90AA1-201   LKEFEGKTLVSVTKEGLELPEDEEEKKKQEEKKTKFENLCKIMKDILEKKVEKVVVSNRLVTSPCCIVTSTYGWTANMERIMKAQALRDNSTMGYMAAKKHLEINPDHSI  642
HSP90AA1-204   ..................................................................................................................   52

logo
               IETLRQKAEADKNDKSVKDLVILLYETALLSSGFSLEDPQTHANRIYRMIKLGLGIDEDDPTADDTSAAVTEEMPPLEGDDDTSRMEEVD
ALVESPIMYCIN   .........................................................................................   17
PF00183        IETLRQKAEADKNDKSVKDLVILLYETALLSSGFSLEDPQTHANRIYRMIKLGLGIDEDDPTADDTSAAVTE.................  519
PF02518        .........................................................................................  154
PF02518.1      .........................................................................................   74
PF00183.1      .........................................................................................   47
HSP90AA1-203   .........................................................................................  174
HSP90AA1-209   .........................................................................................  100
HSP90AA1-208   .........................................................................................   80
HSP90AA1-202   IETLRQKAEADKNDKSVKDLVILLYETALLSSGFSLEDPQTHANRIYRMIKLGLGIDEDDPTADDTSAAVTEEMPPLEGDDDTSRMEEVD  854
HSP90AA1-201   IETLRQKAEADKNDKSVKDLVILLYETALLSSGFSLEDPQTHANRIYRMIKLGLGIDEDDPTADDTSAAVTEEMPPLEGDDDTSRMEEVD  732
HSP90AA1-204   .........................................................................................   52
```

X non conserved
X similar
X $\geq$ 0% conserved
X $\geq$ 50% conserved

logo

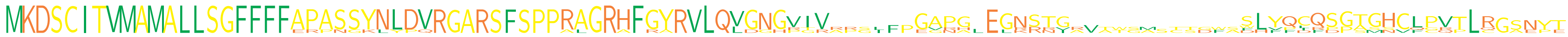

```
LOVASTATIN   .................................................L............................................................   1
PF08441      ..............................................................................................................   0
PF08441.1    ..............................................................................................................   0
PF00357      ..............................................................................................................   0
PF01839      ..............................................................................................................   0
PF00092.2    ..............................................................................................................   0
PF00092.1    ..............................................................................................................   0
PF00092      .......................................................................................DLVFLFDGSMS.....LQ.....  13
ITGAL-202    MKDSCITVMAMALLSGFFFFAPASSYNLDVRGARSFSPPRAGRHFGYRVLQVGNGVIV......GAPG.EGNSTG............SLYQCQSGTGHCLPVTLRGSNYT  91
ITGAL-206    MKDSCITVMAMALLSGFFFFAPASSYNLDVRGARSFSPPRAGRHFGYRVLQVGNGVIV......GAPG.EGNSTG............SLYQCQSGTGHCLPVTLRGSNYT  91
ITGAL-212    MKDSCITVMAMALLSGFFFFAPASSYNLDVRGARSFSPPRAGRHFGYRVLQVGNG.......................................................  55
ITGAL-219    ..............................................................................................................   0
ITGAL-203    ..............................................................................................................   0
ITGAL-208    MKDSCITVMAMALLSGFFFFAPASSYNLDVRGARSFSPPRAGRHFGYRVLQVGNGVIV......GAPG.EGNSTG............SLYQCQSGTGHCLPVTLR.....  86
ITGAL-205    MKDSCITVMAMALLSGFFFFERPNGKLYPQ.........ALGRAFRA....LG.GPGVAPSIFPEVNALEGKRRVRVAWGA.TSGWAW...DRPPAGHVQGR........  84
ITGAL-201    MKDSCITVMAMALLSGFFFFAPASSYNLDVRGARSFSPPRAGRHFGYRVLQVGNGVIV......GAPG.EGNSTG............SLYQCQSGTGHCLPVTLRGSNYT  91
ITGAL-207    ..............................................................................................................   0
ITGAL-216    MKDSCITVMAMALLSGFFFFAPASSYNLDVRGARSFSPPRAGRHFGYRVLQLDGHRTRRRGLFPGGRH.ELRRNIAVTTSMSCTDFSFHFPIQSPASNCFCQPLCGAEPE 109
```

logo

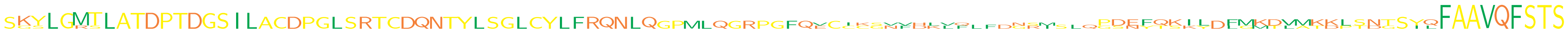

```
LOVASTATIN   ..............................................................................................................   1
PF08441      ..............................................................................................................   0
PF08441.1    ..............................................................................................................   0
PF00357      ..............................................................................................................   0
PF01839      ..............................................................................................................   0
PF00092.2    .....................................................................................................FAAVQFSTS   9
PF00092.1    .....................................................................................................FAAVQFSTS   9
PF00092      .............................................................................PDEFQKILDFMKDVMKKLSNTSYQFAAVQFSTS  46
ITGAL-202    SKYLGMTLATDPTDGSIL...................................................................................FAAVQFSTS 118
ITGAL-206    SKYLGMTLATDPTDGSILACDPGLSRTCDQNTYLSGLCYLFRQNLQGPMLQGRPGFQVCCCSVFHKLQ...NRI.................................... 162
ITGAL-212    ..............................................................................................................  55
ITGAL-219    .....MTLATDPTDGSILACDPGLSRTCDQNTYLSGLCYLFRQNLQ................................................................  41
ITGAL-203    ..............................................................................................................   0
ITGAL-208    .............................................................................GSNYTSKY.LGMTLATDPTDGSILFAAVQFSTS 118
ITGAL-205    .GSLGRS.......................................................................................................  90
ITGAL-201    SKYLGMTLATDPTDGSILACDPGLSRTCDQNTYLSGLCYLFRQNLQGPMLQGRPGFQECIKGNVDLVFLFDGSMSLQPDEFQKILDFMKDVMKKLSNTSYQFAAVQFSTS 201
ITGAL-207    ..............................................................................................................   0
ITGAL-216    .............................................................................................................. 109
```

```
logo
            YKTEFDFSDYVKRKDPDALLKHVKHMLLLTNTFGAINYVATEVFREELGARPDATKVLIIITDGEATDSGNIDAAKDIIRYIIGIGKHFQTKESQETLHKFASKPASEFV
LOVASTATIN  ..............................................................................................................    1
PF08441     ..............................................................................................................    0
PF08441.1   ..............................................................................................................    0
PF00357     ..............................................................................................................    0
PF01839     ..............................................................................................................    0
PF00092.2   YKTEFDFSDYVKRKDPDALLKHVKHMLLLTNTFGAINYVATEVFREELGARPDATKVLIIITDGEATDSGNIDAAKD.................................   86
PF00092.1   YKTEFDFSDYVKRKDPDALLKHVKHMLLLTNTFGAINYVATEVFREELGARPDATKVLIIITDGEATDSGNIDAAKDIIRYIIGIGKHFQTKESQETLHKFASKPASEFV  119
PF00092     YKTEFDFSDYVKRKDPDALLKHVKHMLLLTNTFGAINYVATEVFREELGARPDATKVLIIITDGEATDSGNIDAAKDIIRYIIGIGKHFQTKESQETLHKFASKPASEFV  156
ITGAL-202   YKTEFDFSDYVKRKDPDALLKHVKHMLLLTNTFGAINYVATEVFREELGARPDATKVLIIITDGEATDSGNIDAAKDIIRYIIGIGKHFQTKESQETLHKFASKPASEFV  228
ITGAL-206   ..............................................................................................................  162
ITGAL-212   ..............................................................................................................   55
ITGAL-219   ..............................................................................................................   41
ITGAL-203   ..............................................................................................................    0
ITGAL-208   YKTEFDFSDYVKRKDPDALLKHVKHMLLLTNTFGAINYVATEVFREELGARPDATKVLIIITDGEATDSGNIDAAKDIIRYIIGV.........................  203
ITGAL-205   ..............................................................................................................   90
ITGAL-201   YKTEFDFSDYVKRKDPDALLKHVKHMLLLTNTFGAINYVATEVFREELGARPDATKVLIIITDGEATDSGNIDAAKDIIRYIIGIGKHFQTKESQETLHKFASKPASEFV  311
ITGAL-207   ..............................................................................................................    0
ITGAL-216   ..............................................................................................................  109

logo
            KILDTFEKLKDLFTELQKKIYVIEGTSKQDLTSFNMELSSSGISADLSRGHAVVGAVGAKDWAGGFLDLKADLQDDTFIGNEPLTPEVRAGYLGYTVTWLPSRQKTSLLA
LOVASTATIN  ..............................................................................................................    1
PF08441     ..............................................................................................................    0
PF08441.1   ..............................................................................................................    0
PF00357     ..............................................................................................................    0
PF01839     ..............................................................................................................    0
PF00092.2   ..............................................................................................................   86
PF00092.1   KILDTFEKLKDLFT................................................................................................  133
PF00092     KILDTFEKLKDLFT................................................................................................  170
ITGAL-202   KILDTFEKLKDLFTELQKKIYVIEGTSKQDLTSFNMELSSSGISADLSRGHAVVGAVGAKDWAGGFLDLKADLQDDTFIGNEPLTPEVRAGYLGYTVTWLPSRQKTSLLA  338
ITGAL-206   ..............................................................................................................  162
ITGAL-212   ..............................................................................................................   55
ITGAL-219   ..............................................................................................................   41
ITGAL-203   ..............................................................................................................    0
ITGAL-208   ..............................................................................................................  203
ITGAL-205   ..............................................................................................................   90
ITGAL-201   KILDTFEKLKDLFTELQKKIYVIEGTSKQDLTSFNMELSSSGISADLSRGHAVVGAVGAKDWAGGFLDLKADLQDDTFIGNEPLTPEVRAGYLGYTVTWLPSRQKTSLLA  421
ITGAL-207   ..............................................................................................................    0
ITGAL-216   ..............................................................................................................  109
```

```
logo
            SGAPRYQHMGRVLLFQEPQGGGHWSQVQTIHGTQIGSYFGGELCGVDVDQDGETELLLIGAPLFYGEQRGGRVFIYQRRQLGFEEVSELQGDPGYPLGRFGEAITALTDI
LOVASTATIN  ..............................................................................................................   1
PF08441     ..............................................................................................................   0
PF08441.1   ..............................................................................................................   0
PF00357     ..............................................................................................................   0
PF01839     .......................................................................................................ITALTDI   7
PF00092.2   ..............................................................................................................  86
PF00092.1   .............................................................................................................. 133
PF00092     .............................................................................................................. 170
ITGAL-202   SGAPRYQHMGRVLLFQEPQGGGHWSQVQTIHGTQIGSYFGGELCGVDVDQDGETELLLIGAPLFYGEQRGGRVFIYQRRQLGFEEVSELQGDPGYPLGRFGEAITALTDI 448
ITGAL-206   .............................................................................................................. 162
ITGAL-212   ..............................................................................................................  55
ITGAL-219   ..............................................................................................................  41
ITGAL-203   ..............................................................................................................   0
ITGAL-208   .............................................................................................................. 203
ITGAL-205   ..............................................................................................................  90
ITGAL-201   SGAPRYQHMGRVLLFQEPQGGGHWSQVQTIHGTQIGSYFGGELCGVDVDQDGETELLLIGAPLFYGEQRGGRVFIYQRRQLGFEEVSELQGDPGYPLGRFGEAITALTDI 531
ITGAL-207   ..............................................................................................................   0
ITGAL-216   .............................................................................................................. 109
```

```
logo
            NGDGLVDVAVGAPLEEQGAVYIFNGRHGGLSPQPSQRIEGTQVLSGIQWFGRSIHGVKDLEGDGLADVAVGAESQMIVLSSRPVVDMVTLMSFSPAEIPVHEVECSYSTS
LOVASTATIN  ..............................................................................................................   1
PF08441     ................................................................................SRPVVDMVTLMSFSPAEIPVHEVECSYSTS  30
PF08441.1   ................................................................................SRPVVDMVTLMSFSPAEIPVHEVECSYSTS  30
PF00357     ..............................................................................................................   0
PF01839     NGDGLVDVAVGAPLEEQGAVYIF.......................................................................................  30
PF00092.2   ..............................................................................................................  86
PF00092.1   .............................................................................................................. 133
PF00092     .............................................................................................................. 170
ITGAL-202   NGDGLVDVAVGAPLEEQGAVYIFNGRHGGLSPQPSQRIEGTQVLSGIQWFGRSIHGVKDLEGDGLADVAVGAESQMIVLSSRPVVDMVTLMSFSPAEIPVHEVECSYSTS 558
ITGAL-206   .............................................................................................................. 162
ITGAL-212   ..............................................................................................................  55
ITGAL-219   ..............................................................................................................  41
ITGAL-203   ................................................................MKD......SCITVMA...MALLSGFFFFAPASSYNLDVRGARSFS  37
ITGAL-208   .............................................................................................................. 203
ITGAL-205   ..............................................................................................................  90
ITGAL-201   NGDGLVDVAVGAPLEEQGAVYIFNGRHGGLSPQPSQRIEGTQVLSGIQWFGRSIHGVKDLEGDGLADVAVGAESQMIVLSSRPVVDMVTLMSFSPAEIPVHEVECSYSTS 641
ITGAL-207   ..............................................................................................................   0
ITGAL-216   .............................................................................................................. 109
```

```
logo
            NKMKEGVNITICFQIKSLIPQFQGRLVANLTYTLQLDGHRTRRRGLFPGGRHELRRNIAVTTSMSCTDFSFHFPVCVQDLISPINVSLNFSLWEEEGTPRDQRAQGKDIP
LOVASTATIN  ..............................................................................................................    1
PF08441     NKMKEGVNITICFQIKSLIPQFQGRLVANLTYTLQLDGHRTRRRGLFPGGRHELRRNIAVTTSMSCTDFSFHFPVCVQDLISPINVSLNFSLWEEEGTPRDQRAQGKDIP  140
PF08441.1   NKMKEGVNITICFQIKSLIPQFQGRLVANLTYTLQLDGHRTRRRGLFPGGRHELRRNIAVTTSMSCTDFSFHFPVCVQDLISPINVSLNFSLWEEEGTPRDQRA.GKDIP  139
PF00357     ..............................................................................................................    0
PF01839     ..............................................................................................................   30
PF00092.2   ..............................................................................................................   86
PF00092.1   ..............................................................................................................  133
PF00092     ..............................................................................................................  170
ITGAL-202   NKMKEGVNITICFQIKSLIPQFQGRLVANLTYTLQLDGHRTRRRGLFPGGRHELRRNIAVTTSMSCTDFSFHFPVCVQDLISPINVSLNFSLWEEEGTPRDQRA.GKDIP  667
ITGAL-206   ..............................................................................................................  162
ITGAL-212   ..............................................................................................................   55
ITGAL-219   ..............................................................................................................   41
ITGAL-203   PPRAGRHFGYRVLQIKSLIPQFQGRLVANLTYTLQLDGHRTRRRGLFPGGRHELRRNIAVTTSMSCTDFSFHFP....................................  111
ITGAL-208   ..............................................................................................................  203
ITGAL-205   ..............................................................................................................   90
ITGAL-201   NKMKEGVNITICFQIKSLIPQFQGRLVANLTYTLQLDGHRTRRRGLFPGGRHELRRNIAVTTSMSCTDFSFHFPVCVQDLISPINVSLNFSLWEEEGTPRDQRAQGKDIP  751
ITGAL-207   ..XKEGVNITICFQIKSLIPQFQAGWPPDQKT............GVVPRRET..........................................................   38
ITGAL-216   ..............................................................................................................  109


logo
            PILRPSLHSETWEIPFEKNCGEDKKCEANLRVSFSPARSRALRLTAFASLSVELSLSNLEEDAYWVQLDLHFPPGLSFRKVEMLKPHSQIPVSCEELPEESRLLSRALSC
LOVASTATIN  ..............................................................................................................    1
PF08441     PILRPSLHSETWEIPFEKNCGEDKKCEANLRVSFSPARSRALRLTAFASLSVELSLSNLEEDAYWVQLDLHFPPGLSFRKVEMLKPHSQIPVSCEELPEESRLLSRALSC  250
PF08441.1   PILRPSLHSETWEIPFEKNCGEDKKCEANLRVSFSPARSRALRLTAFASLSVELSLSNLEEDAYWVQLDLHFPPGLSFRKVEMLKPHSQIPVSCEELPEESRLLSRALSC  249
PF00357     ..............................................................................................................    0
PF01839     ..............................................................................................................   30
PF00092.2   ..............................................................................................................   86
PF00092.1   ..............................................................................................................  133
PF00092     ..............................................................................................................  170
ITGAL-202   PILRPSLHSETWEIPFEKNCGEDKKCEANLRVSFSPARSRALRLTAFASLSVELSLSNLEEDAYWVQLDLHFPPGLSFRKVEMLKPHSQIPVSCEELPEESRLLSRALSC  777
ITGAL-206   ..............................................................................................................  162
ITGAL-212   ..............................................................................................................   55
ITGAL-219   ..............................................................................................................   41
ITGAL-203   ..............................................................................................................  111
ITGAL-208   ..............................................................................................................  203
ITGAL-205   ..............................................................................................................   90
ITGAL-201   PILRPSLHSETWEIPFEKNCGEDKKCEANLRVSFSPARSRALRLTAFASLSVELSLSNLEEDAYWVQLDLHFPPGLSFRKVEMLKPHSQIPVSCEELPEESRLLSRALSC  861
ITGAL-207   ..............................................................................................................   38
ITGAL-216   ..............................................................................................................  109
```

```
logo
             NVSSPIFKAGHSVALQMMFNTLVNSSWGDSVELHANVTCNNEDSDLLEDNSATTIIPILYPINILIQDQEDSTLYVSFTPKGPKIHQVKHMYQVRIQPSIHDHNIPTLEA
LOVASTATIN   ........................................................................................................I.....    2
PF08441      NVSSPIFKAGHSVALQMMFNTLVNSSWGDSVELHANVTCNNEDSDLLEDNSATTIIPILYPINILIQDQE........................................  320
PF08441.1    NVSSPIFKAGHSVALQMMFNTLVNSSWGDSVELHANVTCNNEDSDLLEDNSATTIIPILYPINILIQDQE........................................  319
PF00357      ..............................................................................................................    0
PF01839      ..............................................................................................................   30
PF00092.2    ..............................................................................................................   86
PF00092.1    ..............................................................................................................  133
PF00092      ..............................................................................................................  170
ITGAL-202    NVSSPIFKAGHSVALQMMFNTLVNSSWGDSVELHANVTCNNEDSDLLEDNSATTIIPILYPINILIQDQEDSTLYVSFTPKGPKIHQVKHMYQVRIQPSIHDHNIPTLEA  887
ITGAL-206    ..............................................................................................................  162
ITGAL-212    ..............................................................................................................   55
ITGAL-219    ..............................................................................................................   41
ITGAL-203    ................MMFNTLVNSSWGDSVELHANVTCNNEDSDLLEDNSATTIIPILYPINILIQDQEDSTLYVSFTPKGPKIHQVKHMYQVRIQPSIHDHNIPTLEA  205
ITGAL-208    ..............................................................................................................  203
ITGAL-205    ..............................................................................................................   90
ITGAL-201    NVSSPIFKAGHSVALQMMFNTLVNSSWGDSVELHANVTCNNEDSDLLEDNSATTIIPILYPINILIQDQEDSTLYVSFTPKGPKIHQVKHMYQVRIQPSIHDHNIPTLEA  971
ITGAL-207    ..............................................................................................................   38
ITGAL-216    ..............................................................................................................  109

logo
             VVGVPQPPSEGPITHQWSVQMEPPVPCHYEDLERLPDAAEPCLPGALFRCPVVFRQEILVQVIGTLELVGEIEASSMFSLCSSLSISFNSSKHFHLYGSNASLAQVVMKV
LOVASTATIN   ............................Y.................................................................................    3
PF08441      ..............................................................................................................  320
PF08441.1    ..............................................................................................................  319
PF00357      ..............................................................................................................    0
PF01839      ..............................................................................................................   30
PF00092.2    ..............................................................................................................   86
PF00092.1    ..............................................................................................................  133
PF00092      ..............................................................................................................  170
ITGAL-202    VVGVPQPPSEGPITHQWSVQMEPPVPCHYEDLERLPDAAEPCLPGALFRCPVVFRQEILVQVIGTLELVGEIEASSMFSLCSSLSISFNSSKHFHLYGSNASLAQVVMKV  997
ITGAL-206    ..............................................................................................................  162
ITGAL-212    ..............................................................................................................   55
ITGAL-219    ..............................................................................................................   41
ITGAL-203    VVGVPQPPSEGPITHQWSVQMEPPVPCHYEDLERLPDAAEPCLPGALFRCPVVFRQEILVQVIGTLELVGEIEASSMFSLCSSLSISFNSSKHFHLYGSNASLAQVVMKV  315
ITGAL-208    ..............................................................................................................  203
ITGAL-205    ..............................................................................................................   90
ITGAL-201    VVGVPQPPSEGPITHQWSVQMEPPVPCHYEDLERLPDAAEPCLPGALFRCPVVFRQEILVQVIGTLELVGEIEASSMFSLCSSLSISFNSSKHFHLYGSNASLAQVVMKV 1081
ITGAL-207    ..............................................................................................................   38
ITGAL-216    ..............................................................................................................  109
```

```
logo
              DVVYEKQMLYLYVLSGIGGLLLLLLIFIVLYKVGFFKRNLKEKMEAGRGVPNGIPAEDSEQLASGQEAGDPGCLKPLHEKDSESGGGKD
LOVASTATIN    ...................L....L................................................................       5
PF08441       .........................................................................................     320
PF08441.1     .........................................................................................     319
PF00357       ...............................KVGFFKRNLKEKMEA...........................................      15
PF01839       .........................................................................................      30
PF00092.2     .........................................................................................      86
PF00092.1     .........................................................................................     133
PF00092       .........................................................................................     170
ITGAL-202     DVVYEKQMLYLYVLSGIGGLLLLLLIFIVLYKVGFFKRNLKEKMEAGRGVPNGIPAEDSEQLASGQEAGDPGCLKPLHEKDSESGGGKD    1086
ITGAL-206     .........................................................................................     162
ITGAL-212     .........................................................................................      55
ITGAL-219     .........................................................................................      41
ITGAL-203     DVVYEKQMLYLYVLSGIGGLLLLLLIFIVLYKVGFFKRNLKEKMEAGRGVPNGIPAEDSEQLASGQEAGDPGCLKPLHEKDSESGGGKD     404
ITGAL-208     .........................................................................................     203
ITGAL-205     .........................................................................................      90
ITGAL-201     DVVYEKQMLYLYVLSGIGGLLLLLLIFIVLYKVGFFKRNLKEKMEAGRGVPNGIPAEDSEQLASGQEAGDPGCLKPLHEKDSESGGGKD    1170
ITGAL-207     .........................................................................................      38
ITGAL-216     .........................................................................................     109
```

- X non conserved
- X similar
- X $\geq$ 0% conserved
- X $\geq$ 50% conserved

```
logo
               MQYLNIKEDCNAMAFCAKMRSSKKTEVNLEAPEPGVEVIFYLSDREPLRLGSGEYTAEELCIRAAQACRISPLCHNLFALYDENTKLWYAPNRTITVDDKMSLRLHYRMR
CHEMBL21156    ..............................................................................................................     0
PF07714        ..............................................................................................................     0
JAK1-201       MQYLNIKEDCNAMAFCAKMRSSKKTEVNLEAPEPGVEVIFYLSDREPLRLGSGEYTAEELCIRAAQACRISPLCHNLFALYDENTKLWYAPNRTITVDDKMSLRLHYRMR   110

logo
               FYFTNWHGTNDNEQSVWRHSPKKQKNGYEKKKIPDATPLLDASSLEYLFAQGQYDLVKCLAPIRDPKTEQDGHDIENECLGMAVLAISHYAMMKKMQLPELPKDISYKRY
CHEMBL21156    ..............................................................................................................     0
PF07714        ..............................................................................................................     0
JAK1-201       FYFTNWHGTNDNEQSVWRHSPKKQKNGYEKKKIPDATPLLDASSLEYLFAQGQYDLVKCLAPIRDPKTEQDGHDIENECLGMAVLAISHYAMMKKMQLPELPKDISYKRY   220

logo
               IPETLNKSIRQRNLLTRMRINNVFKDFLKEFNNKTICDSSVSTHDLKVKYLATLETLTKHYGAEIFETSMLLISSENEMNWFHSNDGGNVLYYEVMVTGNLGIQWRHKPN
CHEMBL21156    ..............................................................................................................     0
PF07714        ..............................................................................................................     0
JAK1-201       IPETLNKSIRQRNLLTRMRINNVFKDFLKEFNNKTICDSSVSTHDLKVKYLATLETLTKHYGAEIFETSMLLISSENEMNWFHSNDGGNVLYYEVMVTGNLGIQWRHKPN   330

logo
               VVSVEKEKNKLKRKKLENKHKKDEEKNKIREEWNNFSYFPEITHIVIKESVVSINKQDNKKMELKLSSHEEALSFVSLVDGYFRLTADAHHYLCTDVAPPLIVHNIQNGC
CHEMBL21156    ..............................................................................................................     0
PF07714        ..............................................................................................................     0
JAK1-201       VVSVEKEKNKLKRKKLENKHKKDEEKNKIREEWNNFSYFPEITHIVIKESVVSINKQDNKKMELKLSSHEEALSFVSLVDGYFRLTADAHHYLCTDVAPPLIVHNIQNGC   440

logo
               HGPICTEYAINKLRQEGSEEGMYVLRWSCTDFDNILMTVTCFEKSEQVQGAQKQFKNFQIEVQKGRYSLHGSDRSFPSLGDLMSHLKKQILRTDNISFMLKRCCQPKPRE
CHEMBL21156    ..............................................................................................................     0
PF07714        ..............................................................................................................     0
JAK1-201       HGPICTEYAINKLRQEGSEEGMYVLRWSCTDFDNILMTVTCFEKSEQVQGAQKQFKNFQIEVQKGRYSLHGSDRSFPSLGDLMSHLKKQILRTDNISFMLKRCCQPKPRE   550

logo
               ISNLLVATKKAQEWQPVYPMSQLSFDRILKKDLVQGEHLGRGTRTHIYSGTLMDYKDDEGTSEEKKIKVILKVLDPSHRDISLAFFEAASMMRQVSHKHIVYLYGVCVRD
CHEMBL21156    ..............................................................................................................     0
PF07714        .................................VQGEHLGRGTRTHIYSGTLMDYKDDEGTSEEKKIKVILKVLDPSHRDISLAFFEAASMMRQVSHKHIVYLYGVCVRD    77
JAK1-201       ISNLLVATKKAQEWQPVYPMSQLSFDRILKKDLVQGEHLGRGTRTHIYSGTLMDYKDDEGTSEEKKIKVILKVLDPSHRDISLAFFEAASMMRQVSHKHIVYLYGVCVRD   660

logo
               VENIMVEEFVEGGPLDLFMHRKSDVLTTPWKFKVAKQLASALSYLEDKDLVHGNVCTKNLLLAREGIDSECGPFIKLSDPGIPITVLSRQECIERIPWIAPECVEDSKNL
CHEMBL21156    ..............................................................................................................     0
PF07714        VENIMVEEFVEGGPLDLFMHRKSDVLTTPWKFKVAKQLASALSYLEDKDLVHGNVCTKNLLLAREGIDSECGPFIKLSDPGIPITVLSRQECIERIPWIAPECVEDSKNL   187
JAK1-201       VENIMVEEFVEGGPLDLFMHRKSDVLTTPWKFKVAKQLASALSYLEDKDLVHGNVCTKNLLLAREGIDSECGPFIKLSDPGIPITVLSRQECIERIPWIAPECVEDSKNL   770
```

```
logo
```
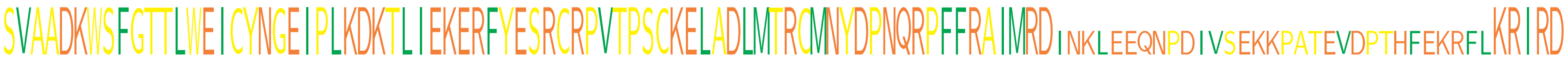

```
CHEMBL21156  ..............................................................................................................     0
PF07714      SVAADKWSFGTTLWEICYNGEIPLKDKTLIEKERFYESRCRPVTPSCKELADLMTRCMNYDPNQRPFFRAIMRD...............................KRIRD   266
JAK1-201     SVAADKWSFGTTLWEICYNGEIPLKDKTLIEKERFYESRCRPVTPSCKELADLMTRCMNYDPNQRPFFRAIMRDINKLEEQNPDIVSEKKPATEVDPTHFEKRFLKRIRD   880
```

```
logo
```
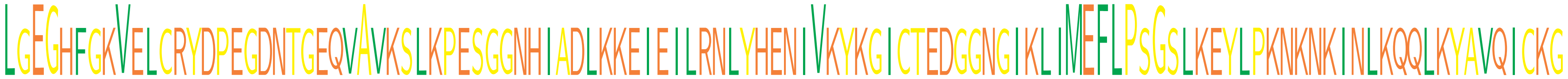

```
CHEMBL21156  L.EG....V................A...............................V.................MEFLP.G............................    12
PF07714      LGEGHFGKVELCRYDPEGDNTGEQVAVKSLKPESGGNHIADLKKEIEILRNLYHENIVKYKGICTEDGGNGIKLIMEFLPSGSLKEYLPKNKNKINLKQQLKYAVQICKG   376
JAK1-201     LGEGHFGKVELCRYDPEGDNTGEQVAVKSLKPESGGNHIADLKKEIEILRNLYHENIVKYKGICTEDGGNGIKLIMEFLPSGSLKEYLPKNKNKINLKQQLKYAVQICKG   990
```

```
logo
```
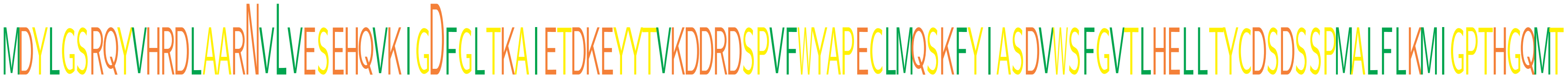

```
CHEMBL21156  .................N.L..........D...............................................................................    15
PF07714      MDYLGSRQYVHRDLAARNVLVESEHQVKIGDFGLTKAIETDKEYYTVKDDRDSPVFWYAPECLMQSKFYIASDVWSFGVTLHELLTYCDSDSSPMALFLKMIGPTHGQMT   486
JAK1-201     MDYLGSRQYVHRDLAARNVLVESEHQVKIGDFGLTKAIETDKEYYTVKDDRDSPVFWYAPECLMQSKFYIASDVWSFGVTLHELLTYCDSDSSPMALFLKMIGPTHGQMT  1100
```

```
logo
```
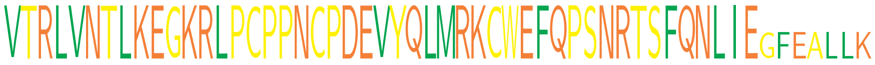

```
CHEMBL21156  ......................................................    15
PF07714      VTRLVNTLKEGKRLPCPPNCPDEVYQLMRKCWEFQPSNRTSFQNLIE.......   533
JAK1-201     VTRLVNTLKEGKRLPCPPNCPDEVYQLMRKCWEFQPSNRTSFQNLIEGFEALLK  1154
```

- X non conserved
- X similar
- X ≥ 0% conserved
- X ≥ 50% conserved

```
logo
             MQYLNIKEDCNAMAFCAKMRSSKKTEVNLEAPEPGVEVIFYLSDREPLRLGSGEYTAEELCIRAAQACRISPLCHNLFALYDENTKLWYAPNRTITVDDKMSLRLHYRMR
TOFACITINIB  ..............................................................................................................    0
PF07714      ..............................................................................................................    0
JAK1-201     MQYLNIKEDCNAMAFCAKMRSSKKTEVNLEAPEPGVEVIFYLSDREPLRLGSGEYTAEELCIRAAQACRISPLCHNLFALYDENTKLWYAPNRTITVDDKMSLRLHYRMR  110

logo
             FYFTNWHGTNDNEQSVWRHSPKKQKNGYEKKKIPDATPLLDASSLEYLFAQGQYDLVKCLAPIRDPKTEQDGHDIENECLGMAVLAISHYAMMKKMQLPELPKDISYKRY
TOFACITINIB  ..............................................................................................................    0
PF07714      ..............................................................................................................    0
JAK1-201     FYFTNWHGTNDNEQSVWRHSPKKQKNGYEKKKIPDATPLLDASSLEYLFAQGQYDLVKCLAPIRDPKTEQDGHDIENECLGMAVLAISHYAMMKKMQLPELPKDISYKRY  220

logo
             IPETLNKSIRQRNLLTRMRINNVFKDFLKEFNNKTICDSSVSTHDLKVKYLATLETLTKHYGAEIFETSMLLISSENEMNWFHSNDGGNVLYYEVMVTGNLGIQWRHKPN
TOFACITINIB  ..............................................................................................................    0
PF07714      ..............................................................................................................    0
JAK1-201     IPETLNKSIRQRNLLTRMRINNVFKDFLKEFNNKTICDSSVSTHDLKVKYLATLETLTKHYGAEIFETSMLLISSENEMNWFHSNDGGNVLYYEVMVTGNLGIQWRHKPN  330

logo
             VVSVEKEKNKLKRKKLENKHKKDEEKNKIREEWNNFSYFPEITHIVIKESVVSINKQDNKKMELKLSSHEEALSFVSLVDGYFRLTADAHHYLCTDVAPPLIVHNIQNGC
TOFACITINIB  ..............................................................................................................    0
PF07714      ..............................................................................................................    0
JAK1-201     VVSVEKEKNKLKRKKLENKHKKDEEKNKIREEWNNFSYFPEITHIVIKESVVSINKQDNKKMELKLSSHEEALSFVSLVDGYFRLTADAHHYLCTDVAPPLIVHNIQNGC  440

logo
             HGPICTEYAINKLRQEGSEEGMYVLRWSCTDFDNILMTVTCFEKSEQVQGAQKQFKNFQIEVQKGRYSLHGSDRSFPSLGDLMSHLKKQILRTDNISFMLKRCCQPKPRE
TOFACITINIB  ..............................................................................................................    0
PF07714      ..............................................................................................................    0
JAK1-201     HGPICTEYAINKLRQEGSEEGMYVLRWSCTDFDNILMTVTCFEKSEQVQGAQKQFKNFQIEVQKGRYSLHGSDRSFPSLGDLMSHLKKQILRTDNISFMLKRCCQPKPRE  550

logo
             ISNLLVATKKAQEWQPVYPMSQLSFDRILKKDLVQGEHLGRGTRTHIYSGTLMDYKDDEGTSEEKKIKVILKVLDPSHRDISLAFFEAASMMRQVSHKHIVYLYGVCVRD
TOFACITINIB  ..............................................................................................................    0
PF07714      .................................VQGEHLGRGTRTHIYSGTLMDYKDDEGTSEEKKIKVILKVLDPSHRDISLAFFEAASMMRQVSHKHIVYLYGVCVRD   77
JAK1-201     ISNLLVATKKAQEWQPVYPMSQLSFDRILKKDLVQGEHLGRGTRTHIYSGTLMDYKDDEGTSEEKKIKVILKVLDPSHRDISLAFFEAASMMRQVSHKHIVYLYGVCVRD  660

logo
             VENIMVEEFVEGGPLDLFMHRKSDVLTTPWKFKVAKQLASALSYLEDKDLVHGNVCTKNLLLAREGIDSECGPFIKLSDPGIPITVLSRQECIERIPWIAPECVEDSKNL
TOFACITINIB  ..............................................................................................................    0
PF07714      VENIMVEEFVEGGPLDLFMHRKSDVLTTPWKFKVAKQLASALSYLEDKDLVHGNVCTKNLLLAREGIDSECGPFIKLSDPGIPITVLSRQECIERIPWIAPECVEDSKNL  187
JAK1-201     VENIMVEEFVEGGPLDLFMHRKSDVLTTPWKFKVAKQLASALSYLEDKDLVHGNVCTKNLLLAREGIDSECGPFIKLSDPGIPITVLSRQECIERIPWIAPECVEDSKNL  770
```

logo 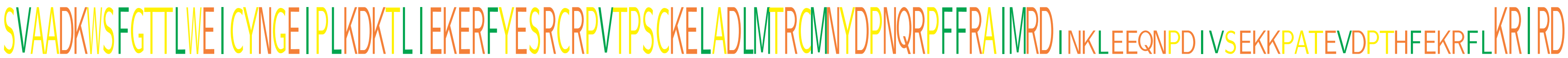

```
TOFACITINIB  ..............................................................................................................     0
PF07714      SVAADKWSFGTTLWEICYNGEIPLKDKTLIEKERFYESRCRPVTPSCKELADLMTRCMNYDPNQRPFFRAIMRD...............................KRIRD   266
JAK1-201     SVAADKWSFGTTLWEICYNGEIPLKDKTLIEKERFYESRCRPVTPSCKELADLMTRCMNYDPNQRPFFRAIMRDINKLEEQNPDIVSEKKPATEVDPTHFEKRFLKRIRD   880
```

logo 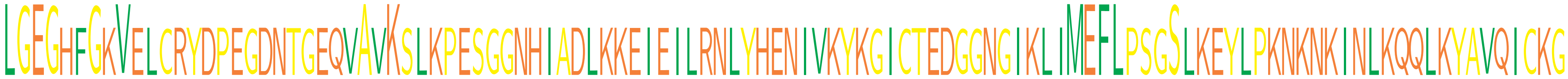

```
TOFACITINIB  LGEG..G.V................A.K...............................................MEFL...S...........................    13
PF07714      LGEGHFGKVELCRYDPEGDNTGEQVAVKSLKPESGGNHIADLKKEIEILRNLYHENIVKYKGICTEDGGNGIKLIMEFLPSGSLKEYLPKNKNKINLKQQLKYAVQICKG   376
JAK1-201     LGEGHFGKVELCRYDPEGDNTGEQVAVKSLKPESGGNHIADLKKEIEILRNLYHENIVKYKGICTEDGGNGIKLIMEFLPSGSLKEYLPKNKNKINLKQQLKYAVQICKG   990
```

logo 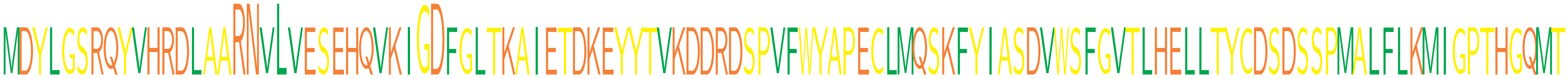

```
TOFACITINIB  ................RN.L.........GD...............................................................................    18
PF07714      MDYLGSRQYVHRDLAARNVLVESEHQVKIGDFGLTKAIETDKEYYTVKDDRDSPVFWYAPECLMQSKFYIASDVWSFGVTLHELLTYCDSDSSPMALFLKMIGPTHGQMT   486
JAK1-201     MDYLGSRQYVHRDLAARNVLVESEHQVKIGDFGLTKAIETDKEYYTVKDDRDSPVFWYAPECLMQSKFYIASDVWSFGVTLHELLTYCDSDSSPMALFLKMIGPTHGQMT  1100
```

logo 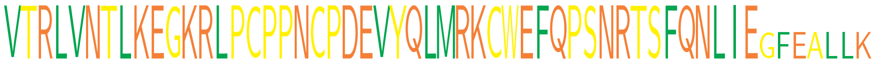

```
TOFACITINIB  ......................................................    18
PF07714      VTRLVNTLKEGKRLPCPPNCPDEVYQLMRKCWEFQPSNRTSFQNLIE.......   533
JAK1-201     VTRLVNTLKEGKRLPCPPNCPDEVYQLMRKCWEFQPSNRTSFQNLIEGFEALLK  1154
```

X non conserved
X similar
X $\geq$ 0% conserved
X $\geq$ 50% conserved

```
logo
             MGMACLTMTEMEGTSTSSIYQNGDISGNANSMKQIDPVLQVYLYHSLGKSEADYLTFPSGEYVAEEICIAASKACGITPVYHNMFALMSETERIWYPPNHVFHIDESTRH
AZD-1480     ..............................................................................................................   0
PF07714.1    ..............................................................................................................   0
PF07714      ..............................................................................................................   0
PF00017      ..............................................................................................................   0
JAK2-202     MGMACLTMTEMEGTSTSSIYQNGDIS....................................................................................  26
JAK2-201     MGMACLTMTEMEGTSTSSIYQNGDISGNANSMKQIDPVLQVYLYHSLGKSEADYLTFPSGEYVAEEICIAASKACGITPVYHNMFALMSETERIWYPPNHVFHIDESTRH 110
JAK2-204     MGMACLTMTEMEGTSTSSIYQNGDISGNANSMKQIDPVLQVYLYHSLGKSEADYLTFPSGEYVAEEICIAASKACGITPVYHNMFALMSETERIWYPPNHVFHIDESTRH 110

logo
             NVLYRIRFYFPRWYCSGSNRAYRHGISRGAEAPLLDDFVMSYLFAQWRHDFVHGWIKVPVTHETQEECLGMAVLDMMRIAKENDQTPLAIYNSISYKTFLPKCIRAKIQD
AZD-1480     ..............................................................................................................   0
PF07714.1    ..............................................................................................................   0
PF07714      ..............................................................................................................   0
PF00017      ..............................................................................................................   0
JAK2-202     ..............................................................................................................  26
JAK2-201     NVLYRIRFYFPRWYCSGSNRAYRHGISRGAEAPLLDDFVMSYLFAQWRHDFVHGWIKVPVTHETQEECLGMAVLDMMRIAKENDQTPLAIYNSISYKTFLPKCIRAKIQD 220
JAK2-204     NVLYRIRFYFPRWYCSGSNRAYRHGISRGAEAPLLDDFVMSYLFAQWRHDFVHGWIKVPVTHETQEECLGMAVLDMMRIAKENDQTPLAIYNSISYKTFLPKCIRAKIQD 220

logo
             YHILTRKRIRYRFRRFIQQFSQCKATARNLKLKYLINLETLQSAFYTEKFEVKEPGSGPSGEEIFATIIITGNGGIQWSRGKHKESETLTEQDLQLYCDFPNIIDVSIKQ
AZD-1480     ..............................................................................................................   0
PF07714.1    ..............................................................................................................   0
PF07714      ..............................................................................................................   0
PF00017      ..............................................................................................................   0
JAK2-202     ..............................................................................................................  26
JAK2-201     YHILTRKRIRYRFRRFIQQFSQCKATARNLKLKYLINLETLQSAFYTEKFEVKEPGSGPSGEEIFATIIITGNGGIQWSRGKHKESETLTEQDLQLYCDFPNIIDVSIKQ 330
JAK2-204     YHILTRKRIRYRFRRFIQQFSQCKATARNLKLKYLINLETLQSAFYTEKFEVKEPGSGPSGEEIFATIIITGNGGIQWSRGKHKESETLTEQDLQLYCDFPNIIDVSIKQ 330

logo
             ANQEGSNESRVVTIHKQDGKNLEIELSSLREALSFVSLIDGYYRLTADAHHYLCKEVAPPAVLENIQSNCHGPISMDFAISKLKKAGNQTGLYVLRCSPKDFNKYFLTFA
AZD-1480     ..............................................................................................................   0
PF07714.1    ..............................................................................................................   0
PF07714      ..............................................................................................................   0
PF00017      ......................................................................HGPISMDFAISKLKKAGNQTGLYVLRCSPKDFNKYFLTFA  40
JAK2-202     ..............................................................................................................  26
JAK2-201     ANQEGSNESRVVTIHKQDGKNLEIELSSLREALSFVSLIDGYYRLTADAHHYLCKEVAPPAVLENIQSNCHGPISMDFAISKLKKAGNQTGLYVLRCSPKDFNKYFLTFA 440
JAK2-204     ANQEGSNESRVVTIHKQDGKNLEIELSSLREALSFVSLIDGYYRLTADAHHYLCKEVAPPAVLENIQSNCHGPISMDFAISKLKKAGNQTGLYVLRCSPKDFNKYFLTFA 440

logo
             VERENVIEYKHCLITKNENEEYNLSGTKKNFSSLKDLLNCYQMETVRSDNIIFQFTKCCPPKPKDKSNLLVFRTNGVSDVPTSPTLQRPTHMNQMVFHKIRNEDLIFNES
AZD-1480     ..............................................................................................................   0
PF07714.1    .........................................................................................................IFNES   5
PF07714      .........................................................................................................IFNES   5
PF00017      VERENVIEYKHCLITKNENEEYNLSGTKKNFSSLKDLLNCY.....................................................................  81
JAK2-202     ..............................................................................................................  26
JAK2-201     VERENVIEYKHCLITKNENEEYNLSGTKKNFSSLKDLLNCYQMETVRSDNIIFQFTKCCPPKPKDKSNLLVFRTNGVSDVPTSPTLQRPTHMNQMVFHKIRNEDLIFNES 550
JAK2-204     VERENVIEYKHCLITKNENEEYNLSGTKKNFSSLKDLLNCYQMETVRSDNIIFQFTKCCPPKPKDKSNLLVFRTNGVSDVPTSPTLQRPTHMNQMVFHKIRNEDLIFNES 550
```

```
logo       LGQGTFTKIFKGVRREVGDYGQLHETEVLLKVLDKAHRNYSESFFEAASMMSKLSHKHLVLNYGVCVCGDENILVQEFVKFGSLDTYLKKNKNCINILWKLEVAKQLAWA
AZD-1480   ..............................................................................................................    0
PF07714.1  LGQGTFTKIFKGVRREVGDYGQLHETEVLLKVLDKAHRNYSESFFEAASMMSKLSHKHLVLNYGVCVCGDENILVQEFVKFGSLDTYLKKNKNCINILWKLEVAKQLAWA  115
PF07714    LGQGTFTKIFKGVRREVGDYGQLHETEVLLKVLDKAHRNYSESFFEAASMMSKLSHKHLVLNYGVCVCGDENILVQEFVKFGSLDTYLKKNKNCINILWKLEVAKQLAWA  115
PF00017    ..............................................................................................................   81
JAK2-202   ..............................................................................................................   26
JAK2-201   LGQGTFTKIFKGVRREVGDYGQLHETEVLLKVLDKAHRNYSESFFEAASMMSKLSHKHLVLNYGVCVCGDENILVQEFVKFGSLDTYLKKNKNCINILWKLEVAKQLAWA  660
JAK2-204   LGQGTFTKIFKGVRREVGDYGQLHETEVLLKVLDKAHRNYSESFFEAASMMSKLSHKHLVLNYGVCVCGDENILVQEFVKFGSLDTYLKKNKNCINILWKLEVAKQLAWA  660

logo       MHFLEENTLIHGNVCAKNILLIREEDRKTGNPPFIKLSDPGISITVLPKDILQERIPWVPPECIENPKNLNLATDKWSFGTTLWEICSGGDKPLSALDSQRKLQFYEDRH
AZD-1480   ..............................................................................................................    0
PF07714.1  MHFLEENTLIHGNVCAKNILLIREEDRKTGNPPFIKLSDPGISITV................................................................  161
PF07714    MHFLEENTLIHGNVCAKNILLIREEDRKTGNPPFIKLSDPGISITVLPKDILQERIPWVPPECIENPKNLNLATDKWSFGTTLWEICSGGDKPLSALDSQRKLQFYEDRH  225
PF00017    ..............................................................................................................   81
JAK2-202   ..............................................................................................................   26
JAK2-201   MHFLEENTLIHGNVCAKNILLIREEDRKTGNPPFIKLSDPGISITVLPKDILQERIPWVPPECIENPKNLNLATDKWSFGTTLWEICSGGDKPLSALDSQRKLQFYEDRH  770
JAK2-204   MHFLEENTLIHGNVCAKNILLIREEDRKTGNPPFIKLSDPGISITVLPKD............................................................  710

logo       QLPAPKWAELANLINNCMDYEPDFRPSFRAIIRDLNSLFTPDYELLTENDMLPNMRIGALGFSGAFEDRDPTQFEERHLKFLQQLGKGNFGSVEMCRYDPLQDNTGEVVA
AZD-1480   ....................................................................................L.......V................A    3
PF07714.1  ..............................................................................................................  161
PF07714    QLPAPKWAELANLINNCMDYEPDFRPSFRAIIRDL...........................................LKFLQQLGKGNFGSVEMCRYDPLQDNTGEVVA  292
PF00017    ..............................................................................................................   81
JAK2-202   ..............................................................................................................   26
JAK2-201   QLPAPKWAELANLINNCMDYEPDFRPSFRAIIRDLNSLFTPDYELLTENDMLPNMRIGALGFSGAFEDRDPTQFEERHLKFLQQLGKGNFGSVEMCRYDPLQDNTGEVVA  880
JAK2-204   ..............................................................................................................  710

logo       VKKLQHSTEEHLRDFEREIEILKSLQHDNIVKYKGVCYSAGRRNLKLIMEYLPYGSLRDYLQKHKERIDHIKLLQYTSQICKGMEYLGTKRYIHRDLATRNILVENENRV
AZD-1480   ................................................MEYL..G............................................RN.L.......   11
PF07714.1  ..............................................................................................................  161
PF07714    VKKLQHSTEEHLRDFEREIEILKSLQHDNIVKYKGVCYSAGRRNLKLIMEYLPYGSLRDYLQKHKERIDHIKLLQYTSQICKGMEYLGTKRYIHRDLATRNILVENENRV  402
PF00017    ..............................................................................................................   81
JAK2-202   ..............................................................................................................   26
JAK2-201   VKKLQHSTEEHLRDFEREIEILKSLQHDNIVKYKGVCYSAGRRNLKLIMEYLPYGSLRDYLQKHKERIDHIKLLQYTSQICKGMEYLGTKRYIHRDLATRNILVENENRV  990
JAK2-204   ..............................................................................................................  710

logo       KIGDFGLTKVLPQDKEYYKVKEPGESPIFWYAPESLTESKFSVASDVWSFGVVLYELFTYIEKSKSPPAEFMRMIGNDKQGQMIVFHLIELLKNNGRLPRPDGCPDEIYM
AZD-1480   ..G...........................................................................................................   12
PF07714.1  ..............................................................................................................  161
PF07714    KIGDFGLTKVLPQDKEYYKVKEPGESPIFWYAPESLTESKFSVASDVWSFGVVLYELFTYIEKSKSPPAEFMRMIGNDKQGQMIVFHLIELLKNNGRLPRPDGCPDEIYM  512
PF00017    ..............................................................................................................   81
JAK2-202   ..............................................................................................................   26
JAK2-201   KIGDFGLTKVLPQDKEYYKVKEPGESPIFWYAPESLTESKFSVASDVWSFGVVLYELFTYIEKSKSPPAEFMRMIGNDKQGQMIVFHLIELLKNNGRLPRPDGCPDEIYM 1100
JAK2-204   ..............................................................................................................  710
```

```
logo
           IMTECWNNNVNQRPSFRDLALRVDQIRDNMAG
AZD-1480   ................................    12
PF07714.1  ................................   161
PF07714    IMTECWNNNVNQRPSFRDLA............   532
PF00017    ................................    81
JAK2-202   ................................    26
JAK2-201   IMTECWNNNVNQRPSFRDLALRVDQIRDNMAG  1132
JAK2-204   ................................   710
```

X non conserved
X similar
X ≥ 0% conserved
X ≥ 50% conserved

```
logo
            MGMACLTMTEMEGTSTSSIYQNGDISGNANSMKQIDPVLQVYLYHSLGKSEADYLTFPSGEYVAEEICIAASKACGITPVYHNMFALMSETERIWYPPNHVFHIDESTRH
CHEMBL21156 ..............................................................................................................    0
PF07714.1   ..............................................................................................................    0
PF07714     ..............................................................................................................    0
PF00017     ..............................................................................................................    0
JAK2-202    MGMACLTMTEMEGTSTSSIYQNGDIS....................................................................................   26
JAK2-201    MGMACLTMTEMEGTSTSSIYQNGDISGNANSMKQIDPVLQVYLYHSLGKSEADYLTFPSGEYVAEEICIAASKACGITPVYHNMFALMSETERIWYPPNHVFHIDESTRH  110
JAK2-204    MGMACLTMTEMEGTSTSSIYQNGDISGNANSMKQIDPVLQVYLYHSLGKSEADYLTFPSGEYVAEEICIAASKACGITPVYHNMFALMSETERIWYPPNHVFHIDESTRH  110

logo
            NVLYRIRFYFPRWYCSGSNRAYRHGISRGAEAPLLDDFVMSYLFAQWRHDFVHGWIKVPVTHETQEECLGMAVLDMMRIAKENDQTPLAIYNSISYKTFLPKCIRAKIQD
CHEMBL21156 ..............................................................................................................    0
PF07714.1   ..............................................................................................................    0
PF07714     ..............................................................................................................    0
PF00017     ..............................................................................................................    0
JAK2-202    ..............................................................................................................   26
JAK2-201    NVLYRIRFYFPRWYCSGSNRAYRHGISRGAEAPLLDDFVMSYLFAQWRHDFVHGWIKVPVTHETQEECLGMAVLDMMRIAKENDQTPLAIYNSISYKTFLPKCIRAKIQD  220
JAK2-204    NVLYRIRFYFPRWYCSGSNRAYRHGISRGAEAPLLDDFVMSYLFAQWRHDFVHGWIKVPVTHETQEECLGMAVLDMMRIAKENDQTPLAIYNSISYKTFLPKCIRAKIQD  220

logo
            YHILTRKRIRYRFRRFIQQFSQCKATARNLKLKYLINLETLQSAFYTEKFEVKEPGSGPSGEEIFATIIITGNGGIQWSRGKHKESETLTEQDLQLYCDFPNIIDVSIKQ
CHEMBL21156 ..............................................................................................................    0
PF07714.1   ..............................................................................................................    0
PF07714     ..............................................................................................................    0
PF00017     ..............................................................................................................    0
JAK2-202    ..............................................................................................................   26
JAK2-201    YHILTRKRIRYRFRRFIQQFSQCKATARNLKLKYLINLETLQSAFYTEKFEVKEPGSGPSGEEIFATIIITGNGGIQWSRGKHKESETLTEQDLQLYCDFPNIIDVSIKQ  330
JAK2-204    YHILTRKRIRYRFRRFIQQFSQCKATARNLKLKYLINLETLQSAFYTEKFEVKEPGSGPSGEEIFATIIITGNGGIQWSRGKHKESETLTEQDLQLYCDFPNIIDVSIKQ  330

logo
            ANQEGSNESRVVTIHKQDGKNLEIELSSLREALSFVSLIDGYYRLTADAHHYLCKEVAPPAVLENIQSNCHGPISMDFAISKLKKAGNQTGLYVLRCSPKDFNKYFLTFA
CHEMBL21156 ..............................................................................................................    0
PF07714.1   ..............................................................................................................    0
PF07714     ..............................................................................................................    0
PF00017     ......................................................................HGPISMDFAISKLKKAGNQTGLYVLRCSPKDFNKYFLTFA   40
JAK2-202    ..............................................................................................................   26
JAK2-201    ANQEGSNESRVVTIHKQDGKNLEIELSSLREALSFVSLIDGYYRLTADAHHYLCKEVAPPAVLENIQSNCHGPISMDFAISKLKKAGNQTGLYVLRCSPKDFNKYFLTFA  440
JAK2-204    ANQEGSNESRVVTIHKQDGKNLEIELSSLREALSFVSLIDGYYRLTADAHHYLCKEVAPPAVLENIQSNCHGPISMDFAISKLKKAGNQTGLYVLRCSPKDFNKYFLTFA  440

logo
            VERENVIEYKHCLITKNENEEYNLSGTKKNFSSLKDLLNCYQMETVRSDNIIFQFTKCCPPKPKDKSNLLVFRTNGVSDVPTSPTLQRPTHMNQMVFHKIRNEDLIFNES
CHEMBL21156 ..............................................................................................................    0
PF07714.1   .........................................................................................................IFNES    5
PF07714     .........................................................................................................IFNES    5
PF00017     VERENVIEYKHCLITKNENEEYNLSGTKKNFSSLKDLLNCY.....................................................................   81
JAK2-202    ..............................................................................................................   26
JAK2-201    VERENVIEYKHCLITKNENEEYNLSGTKKNFSSLKDLLNCYQMETVRSDNIIFQFTKCCPPKPKDKSNLLVFRTNGVSDVPTSPTLQRPTHMNQMVFHKIRNEDLIFNES  550
JAK2-204    VERENVIEYKHCLITKNENEEYNLSGTKKNFSSLKDLLNCYQMETVRSDNIIFQFTKCCPPKPKDKSNLLVFRTNGVSDVPTSPTLQRPTHMNQMVFHKIRNEDLIFNES  550
```

```
logo         LGQGTFTKIFKGVRREVGDYGQLHETEVLLKVLDKAHRNYSESFFEAASMMSKLSHKHLVLNYGVCVCGDENILVQEFVKFGSLDTYLKKNKNCINILWKLEVAKQLAWA
CHEMBL21156  ..............................................................................................................     0
PF07714.1    LGQGTFTKIFKGVRREVGDYGQLHETEVLLKVLDKAHRNYSESFFEAASMMSKLSHKHLVLNYGVCVCGDENILVQEFVKFGSLDTYLKKNKNCINILWKLEVAKQLAWA   115
PF07714      LGQGTFTKIFKGVRREVGDYGQLHETEVLLKVLDKAHRNYSESFFEAASMMSKLSHKHLVLNYGVCVCGDENILVQEFVKFGSLDTYLKKNKNCINILWKLEVAKQLAWA   115
PF00017      ..............................................................................................................    81
JAK2-202     ..............................................................................................................    26
JAK2-201     LGQGTFTKIFKGVRREVGDYGQLHETEVLLKVLDKAHRNYSESFFEAASMMSKLSHKHLVLNYGVCVCGDENILVQEFVKFGSLDTYLKKNKNCINILWKLEVAKQLAWA   660
JAK2-204     LGQGTFTKIFKGVRREVGDYGQLHETEVLLKVLDKAHRNYSESFFEAASMMSKLSHKHLVLNYGVCVCGDENILVQEFVKFGSLDTYLKKNKNCINILWKLEVAKQLAWA   660

logo         MHFLEENTLIHGNVCAKNILLIREEDRKTGNPPFIKLSDPGISITVLPKDILQERIPWVPPECIENPKNLNLATDKWSFGTTLWEICSGGDKPLSALDSQRKLQFYEDRH
CHEMBL21156  ..............................................................................................................     0
PF07714.1    MHFLEENTLIHGNVCAKNILLIREEDRKTGNPPFIKLSDPGISITV................................................................   161
PF07714      MHFLEENTLIHGNVCAKNILLIREEDRKTGNPPFIKLSDPGISITVLPKDILQERIPWVPPECIENPKNLNLATDKWSFGTTLWEICSGGDKPLSALDSQRKLQFYEDRH   225
PF00017      ..............................................................................................................    81
JAK2-202     ..............................................................................................................    26
JAK2-201     MHFLEENTLIHGNVCAKNILLIREEDRKTGNPPFIKLSDPGISITVLPKDILQERIPWVPPECIENPKNLNLATDKWSFGTTLWEICSGGDKPLSALDSQRKLQFYEDRH   770
JAK2-204     MHFLEENTLIHGNVCAKNILLIREEDRKTGNPPFIKLSDPGISITVLPKD............................................................   710

logo         QLPAPKWAELANLINNCMDYEPDFRPSFRAIIRDLNSLFTPDYELLTENDMLPNMRIGALGFSGAFEDRDPTQFEERHLKFLQQLGKGNFGSVEMCRYDPLQDNTGEVVA
CHEMBL21156  ....................................................................................LGK.....V................A     5
PF07714.1    ..............................................................................................................   161
PF07714      QLPAPKWAELANLINNCMDYEPDFRPSFRAIIRDL...........................................LKFLQQLGKGNFGSVEMCRYDPLQDNTGEVVA   292
PF00017      ..............................................................................................................    81
JAK2-202     ..............................................................................................................    26
JAK2-201     QLPAPKWAELANLINNCMDYEPDFRPSFRAIIRDLNSLFTPDYELLTENDMLPNMRIGALGFSGAFEDRDPTQFEERHLKFLQQLGKGNFGSVEMCRYDPLQDNTGEVVA   880
JAK2-204     ..............................................................................................................   710

logo         VKKLQHSTEEHLRDFEREIEILKSLQHDNIVKYKGVCYSAGRRNLKLIMEYLPYGSLRDYLQKHKERIDHIKLLQYTSQICKGMEYLGTKRYIHRDLATRNILVENENRV
CHEMBL21156  ................................................MEYLP.G............................................RN.L.......    14
PF07714.1    ..............................................................................................................   161
PF07714      VKKLQHSTEEHLRDFEREIEILKSLQHDNIVKYKGVCYSAGRRNLKLIMEYLPYGSLRDYLQKHKERIDHIKLLQYTSQICKGMEYLGTKRYIHRDLATRNILVENENRV   402
PF00017      ..............................................................................................................    81
JAK2-202     ..............................................................................................................    26
JAK2-201     VKKLQHSTEEHLRDFEREIEILKSLQHDNIVKYKGVCYSAGRRNLKLIMEYLPYGSLRDYLQKHKERIDHIKLLQYTSQICKGMEYLGTKRYIHRDLATRNILVENENRV   990
JAK2-204     ..............................................................................................................   710

logo         KIGDFGLTKVLPQDKEYYKVKEPGESPIFWYAPESLTESKFSVASDVWSFGVVLYELFTYIEKSKSPPAEFMRMIGNDKQGQMIVFHLIELLKNNGRLPRPDGCPDEIYM
CHEMBL21156  ...D..........................................................................................................    15
PF07714.1    ..............................................................................................................   161
PF07714      KIGDFGLTKVLPQDKEYYKVKEPGESPIFWYAPESLTESKFSVASDVWSFGVVLYELFTYIEKSKSPPAEFMRMIGNDKQGQMIVFHLIELLKNNGRLPRPDGCPDEIYM   512
PF00017      ..............................................................................................................    81
JAK2-202     ..............................................................................................................    26
JAK2-201     KIGDFGLTKVLPQDKEYYKVKEPGESPIFWYAPESLTESKFSVASDVWSFGVVLYELFTYIEKSKSPPAEFMRMIGNDKQGQMIVFHLIELLKNNGRLPRPDGCPDEIYM  1100
JAK2-204     ..............................................................................................................   710
```

```
logo
              IMTECWNNNVNQRPSFRDLALRVDQIRDNMAG
CHEMBL21156   ................................    15
PF07714.1     ................................   161
PF07714       IMTECWNNNVNQRPSFRDLA............   532
PF00017       ................................    81
JAK2-202      ................................    26
JAK2-201      IMTECWNNNVNQRPSFRDLALRVDQIRDNMAG  1132
JAK2-204      ................................   710
```

X non conserved

X similar

X $\geq$ 0% conserved

X $\geq$ 50% conserved

```
logo
             MGMACLTMTEMEGTSTSSIYQNGDISGNANSMKQIDPVLQVYLYHSLGKSEADYLTFPSGEYVAEEICIAASKACGITPVYHNMFALMSETERIWYPPNHVFHIDESTRH
TOFACITINIB  ..............................................................................................................    0
PF07714.1    ..............................................................................................................    0
PF07714      ..............................................................................................................    0
PF00017      ..............................................................................................................    0
JAK2-202     MGMACLTMTEMEGTSTSSIYQNGDIS....................................................................................   26
JAK2-201     MGMACLTMTEMEGTSTSSIYQNGDISGNANSMKQIDPVLQVYLYHSLGKSEADYLTFPSGEYVAEEICIAASKACGITPVYHNMFALMSETERIWYPPNHVFHIDESTRH  110
JAK2-204     MGMACLTMTEMEGTSTSSIYQNGDISGNANSMKQIDPVLQVYLYHSLGKSEADYLTFPSGEYVAEEICIAASKACGITPVYHNMFALMSETERIWYPPNHVFHIDESTRH  110

logo
             NVLYRIRFYFPRWYCSGSNRAYRHGISRGAEAPLLDDFVMSYLFAQWRHDFVHGWIKVPVTHETQEECLGMAVLDMMRIAKENDQTPLAIYNSISYKTFLPKCIRAKIQD
TOFACITINIB  ..............................................................................................................    0
PF07714.1    ..............................................................................................................    0
PF07714      ..............................................................................................................    0
PF00017      ..............................................................................................................    0
JAK2-202     ..............................................................................................................   26
JAK2-201     NVLYRIRFYFPRWYCSGSNRAYRHGISRGAEAPLLDDFVMSYLFAQWRHDFVHGWIKVPVTHETQEECLGMAVLDMMRIAKENDQTPLAIYNSISYKTFLPKCIRAKIQD  220
JAK2-204     NVLYRIRFYFPRWYCSGSNRAYRHGISRGAEAPLLDDFVMSYLFAQWRHDFVHGWIKVPVTHETQEECLGMAVLDMMRIAKENDQTPLAIYNSISYKTFLPKCIRAKIQD  220

logo
             YHILTRKRIRYRFRRFIQQFSQCKATARNLKLKYLINLETLQSAFYTEKFEVKEPGSGPSGEEIFATIIIITGNGGIQWSRGKHKESETLTEQDLQLYCDFPNIIDVSIKQ
TOFACITINIB  ..............................................................................................................    0
PF07714.1    ..............................................................................................................    0
PF07714      ..............................................................................................................    0
PF00017      ..............................................................................................................    0
JAK2-202     ..............................................................................................................   26
JAK2-201     YHILTRKRIRYRFRRFIQQFSQCKATARNLKLKYLINLETLQSAFYTEKFEVKEPGSGPSGEEIFATIIIITGNGGIQWSRGKHKESETLTEQDLQLYCDFPNIIDVSIKQ  330
JAK2-204     YHILTRKRIRYRFRRFIQQFSQCKATARNLKLKYLINLETLQSAFYTEKFEVKEPGSGPSGEEIFATIIIITGNGGIQWSRGKHKESETLTEQDLQLYCDFPNIIDVSIKQ  330

logo
             ANQEGSNESRVVTIHKQDGKNLEIELSSLREALSFVSLIDGYYRLTADAHHYLCKEVAPPAVLENIQSNCHGPISMDFAISKLKKAGNQTGLYVLRCSPKDFNKYFLTFA
TOFACITINIB  ..............................................................................................................    0
PF07714.1    ..............................................................................................................    0
PF07714      ..............................................................................................................    0
PF00017      ......................................................................HGPISMDFAISKLKKAGNQTGLYVLRCSPKDFNKYFLTFA   40
JAK2-202     ..............................................................................................................   26
JAK2-201     ANQEGSNESRVVTIHKQDGKNLEIELSSLREALSFVSLIDGYYRLTADAHHYLCKEVAPPAVLENIQSNCHGPISMDFAISKLKKAGNQTGLYVLRCSPKDFNKYFLTFA  440
JAK2-204     ANQEGSNESRVVTIHKQDGKNLEIELSSLREALSFVSLIDGYYRLTADAHHYLCKEVAPPAVLENIQSNCHGPISMDFAISKLKKAGNQTGLYVLRCSPKDFNKYFLTFA  440

logo
             VERENVIEYKHCLITKNENEEYNLSGTKKNFSSLKDLLNCYQMETVRSDNIIFQFTKCCPPKPKDKSNLLVFRTNGVSDVPTSPTLQRPTHMNQMVFHKIRNEDLIFNES
TOFACITINIB  ..............................................................................................................    0
PF07714.1    .........................................................................................................IFNES    5
PF07714      .........................................................................................................IFNES    5
PF00017      VERENVIEYKHCLITKNENEEYNLSGTKKNFSSLKDLLNCY.....................................................................   81
JAK2-202     ..............................................................................................................   26
JAK2-201     VERENVIEYKHCLITKNENEEYNLSGTKKNFSSLKDLLNCYQMETVRSDNIIFQFTKCCPPKPKDKSNLLVFRTNGVSDVPTSPTLQRPTHMNQMVFHKIRNEDLIFNES  550
JAK2-204     VERENVIEYKHCLITKNENEEYNLSGTKKNFSSLKDLLNCYQMETVRSDNIIFQFTKCCPPKPKDKSNLLVFRTNGVSDVPTSPTLQRPTHMNQMVFHKIRNEDLIFNES  550
```

```
logo         LGQGTFTKIFKGVRREVGDYGQLHETEVLLKVLDKAHRNYSESFFEAASMMSKLSHKHLVLNYGVCVCGDENILVQEFVKFGSLDTYLKKNKNCINILWKLEVAKQLAWA
TOFACITINIB  ..............................................................................................................     0
PF07714.1    LGQGTFTKIFKGVRREVGDYGQLHETEVLLKVLDKAHRNYSESFFEAASMMSKLSHKHLVLNYGVCVCGDENILVQEFVKFGSLDTYLKKNKNCINILWKLEVAKQLAWA   115
PF07714      LGQGTFTKIFKGVRREVGDYGQLHETEVLLKVLDKAHRNYSESFFEAASMMSKLSHKHLVLNYGVCVCGDENILVQEFVKFGSLDTYLKKNKNCINILWKLEVAKQLAWA   115
PF00017      ..............................................................................................................    81
JAK2-202     ..............................................................................................................    26
JAK2-201     LGQGTFTKIFKGVRREVGDYGQLHETEVLLKVLDKAHRNYSESFFEAASMMSKLSHKHLVLNYGVCVCGDENILVQEFVKFGSLDTYLKKNKNCINILWKLEVAKQLAWA   660
JAK2-204     LGQGTFTKIFKGVRREVGDYGQLHETEVLLKVLDKAHRNYSESFFEAASMMSKLSHKHLVLNYGVCVCGDENILVQEFVKFGSLDTYLKKNKNCINILWKLEVAKQLAWA   660

logo         MHFLEENTLIHGNVCAKNILLIREEDRKTGNPPFIKLSDPGISITVLPKDILQERIPWVPPECIENPKNLNLATDKWSFGTTLWEICSGGDKPLSALDSQRKLQFYEDRH
TOFACITINIB  ..............................................................................................................     0
PF07714.1    MHFLEENTLIHGNVCAKNILLIREEDRKTGNPPFIKLSDPGISITV................................................................   161
PF07714      MHFLEENTLIHGNVCAKNILLIREEDRKTGNPPFIKLSDPGISITVLPKDILQERIPWVPPECIENPKNLNLATDKWSFGTTLWEICSGGDKPLSALDSQRKLQFYEDRH   225
PF00017      ..............................................................................................................    81
JAK2-202     ..............................................................................................................    26
JAK2-201     MHFLEENTLIHGNVCAKNILLIREEDRKTGNPPFIKLSDPGISITVLPKDILQERIPWVPPECIENPKNLNLATDKWSFGTTLWEICSGGDKPLSALDSQRKLQFYEDRH   770
JAK2-204     MHFLEENTLIHGNVCAKNILLIREEDRKTGNPPFIKLSDPGISITVLPKD............................................................   710

logo         QLPAPKWAELANLINNCMDYEPDFRPSFRAIIRDLNSLFTPDYELLTENDMLPNMRIGALGFSGAFEDRDPTQFEERHLKFLQQLGKGNFGSVEMCRYDPLQDNTGEVVA
TOFACITINIB  ....................................................................................LGKG..GSV................A     8
PF07714.1    ..............................................................................................................   161
PF07714      QLPAPKWAELANLINNCMDYEPDFRPSFRAIIRDL...........................................LKFLQQLGKGNFGSVEMCRYDPLQDNTGEVVA   292
PF00017      ..............................................................................................................    81
JAK2-202     ..............................................................................................................    26
JAK2-201     QLPAPKWAELANLINNCMDYEPDFRPSFRAIIRDLNSLFTPDYELLTENDMLPNMRIGALGFSGAFEDRDPTQFEERHLKFLQQLGKGNFGSVEMCRYDPLQDNTGEVVA   880
JAK2-204     ..............................................................................................................   710

logo         VKKLQHSTEEHLRDFEREIEILKSLQHDNIVKYKGVCYSAGRRNLKLIMEYLPYGSLRDYLQKHKERIDHIKLLQYTSQICKGMEYLGTKRYIHRDLATRNILVENENRV
TOFACITINIB  .K............................V.................MEYL...S...........................................R..L.......    17
PF07714.1    ..............................................................................................................   161
PF07714      VKKLQHSTEEHLRDFEREIEILKSLQHDNIVKYKGVCYSAGRRNLKLIMEYLPYGSLRDYLQKHKERIDHIKLLQYTSQICKGMEYLGTKRYIHRDLATRNILVENENRV   402
PF00017      ..............................................................................................................    81
JAK2-202     ..............................................................................................................    26
JAK2-201     VKKLQHSTEEHLRDFEREIEILKSLQHDNIVKYKGVCYSAGRRNLKLIMEYLPYGSLRDYLQKHKERIDHIKLLQYTSQICKGMEYLGTKRYIHRDLATRNILVENENRV   990
JAK2-204     ..............................................................................................................   710

logo         KIGDFGLTKVLPQDKEYYKVKEPGESPIFWYAPESLTESKFSVASDVWSFGVVLYELFTYIEKSKSPPAEFMRMIGNDKQGQMIVFHLIELLKNNGRLPRPDGCPDEIYM
TOFACITINIB  ..GD..........................................................................................................    19
PF07714.1    ..............................................................................................................   161
PF07714      KIGDFGLTKVLPQDKEYYKVKEPGESPIFWYAPESLTESKFSVASDVWSFGVVLYELFTYIEKSKSPPAEFMRMIGNDKQGQMIVFHLIELLKNNGRLPRPDGCPDEIYM   512
PF00017      ..............................................................................................................    81
JAK2-202     ..............................................................................................................    26
JAK2-201     KIGDFGLTKVLPQDKEYYKVKEPGESPIFWYAPESLTESKFSVASDVWSFGVVLYELFTYIEKSKSPPAEFMRMIGNDKQGQMIVFHLIELLKNNGRLPRPDGCPDEIYM  1100
JAK2-204     ..............................................................................................................   710
```

```
logo
             IMTECWNNNVNQRPSFRDLALRVDQIRDNMAG
TOFACITINIB  ................................    19
PF07714.1    ................................   161
PF07714      IMTECWNNNVNQRPSFRDLA............   532
PF00017      ................................    81
JAK2-202     ................................    26
JAK2-201     IMTECWNNNVNQRPSFRDLALRVDQIRDNMAG  1132
JAK2-204     ................................   710
```

X non conserved

X similar

X $\geq$ 0% conserved

X $\geq$ 50% conserved

```
logo          MAPPSEETPLIPQRSCSLLSTEAGALHVLLPARGPGPPQRLSFSFGDHLAEDLCVQAAKASGILPVYHSLFALATEDLSCWFPPSHIFSVEDASTQVLLYRIRFYFPNWF
CHEMBL21156   ..............................................................................................................    0
PF07714.1     ..............................................................................................................    0
PF07714       ..............................................................................................................    0
JAK3-201      MAPPSEETPLIPQRSCSLLSTEAGALHVLLPARGPGPPQRLSFSFGDHLAEDLCVQAAKASGILPVYHSLFALATEDLSCWFPPSHIFSVEDASTQVLLYRIRFYFPNWF  110
JAK3-204      MAPPSEETPLIPQRSCSLLSTEAGALHVLLPARGPGPPQRLSFSFGDHLAEDLCVQAAKASGILPVYHSLFALATEDLSCWFPPSHIFSVEDASTQVLLYRIRFYFPNWF  110
JAK3-207      MAPPSEETPLIPQRSCSLLSTEAGALHVLLPARGPGPPQRLSFSFGDHLAEDLCVQAAKASGILPVYHSLFALATEDLSCWFPPSHIFSVEDASTQVLLYRIRFYFPNWF  110

logo          GLEKCHRFGLRKDLASAILDLPVLEHLFAQHRSDLVSGRLPVGLSLKEQGECLSLAVLDLARMAREQAQRPGELLKTVSYKACLPPSLRDLIQGLSFVTRRRIRRTVRRA
CHEMBL21156   ..............................................................................................................    0
PF07714.1     ..............................................................................................................    0
PF07714       ..............................................................................................................    0
JAK3-201      GLEKCHRFGLRKDLASAILDLPVLEHLFAQHRSDLVSGRLPVGLSLKEQGECLSLAVLDLARMAREQAQRPGELLKTVSYKACLPPSLRDLIQGLSFVTRRRIRRTVRRA  220
JAK3-204      GLEKCHRFGLRKDLASAILDLPVLEHLFAQHRSDLVSGRLPVGLSLKEQGECLSLAVLDLARMAREQAQRPGELLKTVSYKACLPPSLRDLIQGLSFVTRRRIRRTVRRA  220
JAK3-207      GLEKCHRFGLRKDLASAILDLPVLEHLFAQHRSDLVSGRLPVGLSLKEQGECLSLAVLDLARMAREQAQRPGELLKTVSYKACLPPSLRDLIQGLSFVTRRRIRRTVRRA  220

logo          LRRVAACQADRHSLMAKYIMDLERLDPAGAAETFHVGLPGALGGHDGLGLLRVAGDGGIAWTQGEQEVLQPFCDFPEIVDISIKQAPRVGPAGEHRLVTVTRTDNQILEA
CHEMBL21156   ..............................................................................................................    0
PF07714.1     ..............................................................................................................    0
PF07714       ..............................................................................................................    0
JAK3-201      LRRVAACQADRHSLMAKYIMDLERLDPAGAAETFHVGLPGALGGHDGLGLLRVAGDGGIAWTQGEQEVLQPFCDFPEIVDISIKQAPRVGPAGEHRLVTVTRTDNQILEA  330
JAK3-204      LRRVAACQADRHSLMAKYIMDLERLDPAGAAETFHVGLPGALGGHDGLGLLRVAGDGGIAWTQGEQEVLQPFCDFPEIVDISIKQAPRVGPAGEHRLVTVTRTDNQILEA  330
JAK3-207      LRRVAACQADRHSLMAKYIMDLERLDPAGAAETFHVGLPGALGGHDGLGLLRVAGDGGIAWTQGEQEVLQPFCDFPEIVDISIKQAPRVGPAGEHRLVTVTRTDNQILEA  330

logo          EFPGLPEALSFVALVDGYFRLTTDSQHFFCKEVAPPRLLEEVAEQCHGPITLDFAINKLKTGGSRPGSYVLRRSPQDFDSFLLTVCVQNPLGPDYKGCLIRRSPTGTFLL
CHEMBL21156   ..............................................................................................................    0
PF07714.1     ..............................................................................................................    0
PF07714       ..............................................................................................................    0
JAK3-201      EFPGLPEALSFVALVDGYFRLTTDSQHFFCKEVAPPRLLEEVAEQCHGPITLDFAINKLKTGGSRPGSYVLRRSPQDFDSFLLTVCVQNPLGPDYKGCLIRRSPTGTFLL  440
JAK3-204      EFPGLPEALSFVALVDGYFRLTTDSQHFFCKEVAPPRLLEEVAEQCHGPITLDFAINKLKTGGSRPGSYVLRRSPQDFDSFLLTVCVQNPLGPDYKGCLIRRSPTGTFLL  440
JAK3-207      EFPGLPEALSFVALVDGYFRLTTDSQHFFCKEVAPPRLLEEVAEQCHGPITLDFAINKLKTGGSRPGSYVLRRSPQDFDSFLLTVCVQNPLGPDYKGCLIRRSPTGTFLL  440

logo          VGLSRPHSSLRELLATCWDGGLHVDGVAVTLTSCCIPRPKEKSNLIVVQRGHSPPTSSLVQPQSQYQLSQMTFHKIPADSLEWHENLGHGSFTKIYRGCRHEVVDGEARK
CHEMBL21156   ..............................................................................................................    0
PF07714.1     ................................................................................LEWHENLGHGSFTKIYRGCRHEVVDGEARK   30
PF07714       ................................................................................LEWHENLGHGSFTKIYRGCRHEVVDGEARK   30
JAK3-201      VGLSRPHSSLRELLATCWDGGLHVDGVAVTLTSCCIPRPKEKSNLIVVQRGHSPPTSSLVQPQSQYQLSQMTFHKIPADSLEWHENLGHGSFTKIYRGCRHEVVDGEARK  550
JAK3-204      VGLSRPHSSLRELLATCWDGGLHVDGVAVTLTSCCIPRPKEKSNLIVVQRGHSPPTSSLVQPQSQYQLSQMTFHKIPADSLEWHENLGHGSFTKIYRGCRHEVVDGEARK  550
JAK3-207      VGLSRPHSSLRELLATCWDGGLHVDGVAVTLTSCCIPRPKEKSNLIVVQRGHSPPTSSLVQPQSQYQLSQMTFHKIPADSLEWHENLGHGSFTKIYRGCRHEVVDGEARK  550
```

logo 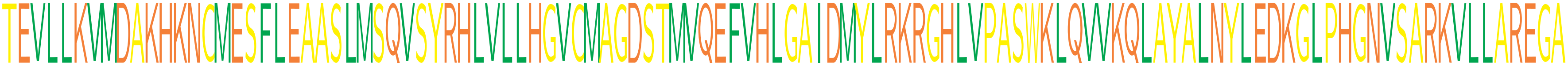

```
CHEMBL21156  ..............................................................................................................     0
PF07714.1    TEVLLKVMDAKHKNCMESFLEAASLMSQVSYRHLVLLHGVCMAGDSTMVQEFVHLGAIDMYLRKRGHLVPASWKLQVVKQLAYALNYLEDKGLPHGNVSARKVLLAREGA   140
PF07714      TEVLLKVMDAKHKNCMESFLEAASLMSQVSYRHLVLLHGVCMAGDSTMVQEFVHLGAIDMYLRKRGHLVPASWKLQVVKQLAYALNYLEDKGLPHGNVSARKVLLAREGA   140
JAK3-201     TEVLLKVMDAKHKNCMESFLEAASLMSQVSYRHLVLLHGVCMAGDSTMVQEFVHLGAIDMYLRKRGHLVPASWKLQVVKQLAYALNYLEDKGLPHGNVSARKVLLAREGA   660
JAK3-204     TEVLLKVMDAKHKNCMESFLEAASLMSQVSYRHLVLLHGVCMAGDSTMVQEFVHLGAIDMYLRKRGHLVPASWKLQVVKQLAYALNYLEDKGLPHGNVSARKVLLAREGA   660
JAK3-207     TEVLLKVMDAKHKNCMESFLEAASLMSQVSYRHLVLLHGVCMAGDSTMVQEFVHLGAIDMYLRKRGHLVPASWKLQVVKQLAYALNYLEDKGLPHGNVSARKVLLAREGA   660
```

logo 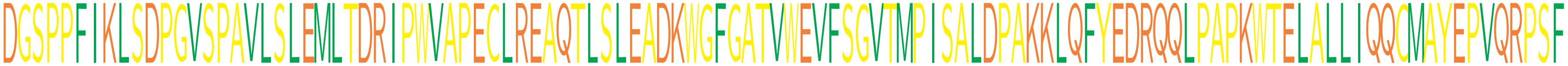

```
CHEMBL21156  ..............................................................................................................     0
PF07714.1    DGSPPFIKLSDPGVSPAVLSLEMLTDRIPWVAPECLREAQTLSLEADKWGFGATVWEVFSGVTMPISALDPAKKLQFYEDRQQLPAPKWTELALLIQQCMAYEPVQRPSF   250
PF07714      DGSPPFIKLSDPGVSPAVLSLEMLTDRIPWVAPECLREAQTLSLEADKWGFGATVWEVFSGVTMPISALDPAKKLQFYEDRQQLPAPKWTELALLIQQCMAYEPVQRPSF   250
JAK3-201     DGSPPFIKLSDPGVSPAVLSLEMLTDRIPWVAPECLREAQTLSLEADKWGFGATVWEVFSGVTMPISALDPAKKLQFYEDRQQLPAPKWTELALLIQQCMAYEPVQRPSF   770
JAK3-204     DGSPPFIKLSDPGVSPAVLSLEMLTDRIPWVAPECLREAQTLSLEADKWGFGATVWEVFSGVTMPISALDPAKKLQFYEDRQQLPAPKWTELALLIQQCMAYEPVQRPSF   770
JAK3-207     DGSPPFIKLSDPGVSPAVLSLEMLTDRIPWVAPECLREAQTLSLEADKWGFGATVWEVFSGVTMPISALDPAKKLQFYEDRQQLPAPKWTELALLIQQCMAYEPVQRPSF   770
```

logo 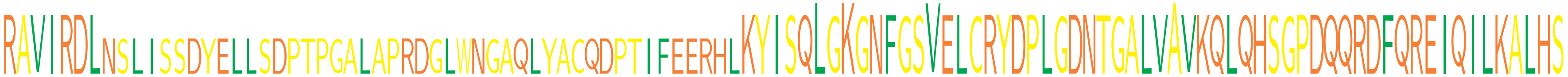

```
CHEMBL21156  .........................................................L.K.....V................A..........................     4
PF07714.1    RAVIRDL.............................................KYISQLGKGNFGSVELCRYDPLGDNTGALVAVKQLHSGPDQQRDFQREIQILKALHS   315
PF07714      RAVIRDL.............................................KYISQLGKGNFGSVELCRYDPLGDNTGALVAVKQLHSGPDQQRDFQREIQILKALHS   315
JAK3-201     RAVIRDLNSLISSDYELLSDPTPGALAPRDGLWNGAQLYACQDPTIFEERHLKYISQLGKGNFGSVELCRYDPLGDNTGALVAVKQLHSGPDQQRDFQREIQILKALHS   880
JAK3-204     RAVIRDLNSLISSDYELLSDPTPGALAPRDGLWNGAQLYACQDPTIFEERHLKYISQLGKGNFGSVELCRYDPLGDNTGALVAVKQLHSGPDQQRDFQREIQILKALHS   880
JAK3-207     RAVIRDLNSLISSDYELLSDPTPGALAPRDGLWNGAQLYACQDPTIFEERHLKYISQLGKGNFGSVELCRYDPLGDNTGALVAVKQLHSGPDQQRDFQREIQILKALHS   880
```

logo 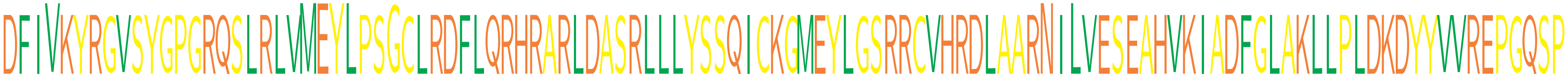

```
CHEMBL21156  ...V.................MEYL..G.............................................N.L..................................    12
PF07714.1    DFIVKYRGVSYGPGRQSLRLVMEYLPSGCLRDFLQRHRARLDASRLLLYSSQICKGMEYLGSRRCVHRDLAARNILVESEAHVKIADFGLAKLLPLDKDYYVVREPGQSP   425
PF07714      DFIVKYRGVSYGPGRQSLRLVMEYLPSGCLRDFLQRHRARLDASRLLLYSSQICKGMEYLGSRRCVHRDLAARNILVESEAHVKIADFGLAKLLPLDKDYYVVREPGQSP   425
JAK3-201     DFIVKYRGVSYGPGRQSLRLVMEYLPSGCLRDFLQRHRARLDASRLLLYSSQICKGMEYLGSRRCVHRDLAARNILVESEAHVKIADFGLAKLLPLDKDYYVVREPGQSP   990
JAK3-204     DFIVKYRGVSYGPGRQSLRLVMEYLPSGCLRDFLQRHRARLDASRLLLYSSQICKGMEYLGSRRCVHRDLAARNILVESEAHVKIADFGLAKLLPLDKDYYVVREPGQSP   990
JAK3-207     DFIVKYRGVSYGPGRQSLRLVMEYLPSGCLRDFLQRHRARLDASRLLLYSSQICKGMEYLGSRRCVHRDLAARNILVESEAHVKIADFGLAKLLPLDKDYYVVREPGQSP   990
```

logo 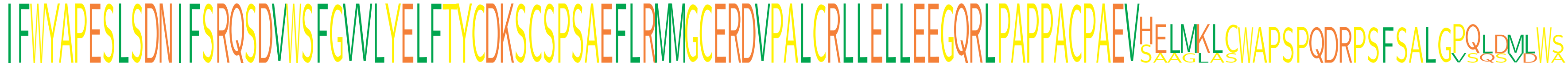

```
CHEMBL21156  ..............................................................................................................    12
PF07714.1    IFWYAPESLSDNIFSRQSDVWSFGVVLYELFTYCDKSCSPSAEFLRMMGCERDVPALCRLLELLEEGQRLPAPPACPAEVS.............................   506
PF07714      IFWYAPESLSDNIFSRQSDVWSFGVVLYELFTYCDKSCSPSAEFLRMMGCERDVPALCRLLELLEEGQRLPAPPACPAEVHELMKLCWAPSPQDRPSFSALGPQ......   529
JAK3-201     IFWYAPESLSDNIFSRQSDVWSFGVVLYELFTYCDKSCSPSAEFLRMMGCERDVPALCRLLELLEEGQRLPAPPACPAEVHELMKLCWAPSPQDRPSFSALGPQLDMLWS  1100
JAK3-204     IFWYAPESLSDNIFSRQSDVWSFGVVLYELFTYCDKSCSPSAEFLRMMGCERDVPALCRLLELLEEGQRLPAPPACPAEVHELMKLCWAPSPQDRPSFSALGPQLDMLWS  1100
JAK3-207     IFWYAPESLSDNIFSRQSDVWSFGVVLYELFTYCDKSCSPSAEFLRMMGCERDVPALCRLLELLEEGQRLPAPPACPAEVSAAGLAS...............VSQSVDWA  1085
```

logo

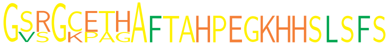

```
CHEMBL21156   ........................   12
PF07714.1     ........................  506
PF07714       ........................  529
JAK3-201      GSRGCETHAFTAHPEGKHHSLSFS 1124
JAK3-204      GSRGCETHAFTAHPEGKHHSLSFS 1124
JAK3-207      GVSGKPAGA............... 1094
```

X non conserved
X similar
X $\geq$ 0% conserved
X $\geq$ 50% conserved

```
logo
             MAPPSEETPLIPQRSCSLLSTEAGALHVLLPARGPGPPQRLSFSFGDHLAEDLCVQAAKASGILPVYHSLFALATEDLSCWFPPSHIFSVEDASTQVLLYRIRFYFPNWF
TOFACITINIB  ..............................................................................................................     0
PF07714.1    ..............................................................................................................     0
PF07714      ..............................................................................................................     0
JAK3-201     MAPPSEETPLIPQRSCSLLSTEAGALHVLLPARGPGPPQRLSFSFGDHLAEDLCVQAAKASGILPVYHSLFALATEDLSCWFPPSHIFSVEDASTQVLLYRIRFYFPNWF   110
JAK3-204     MAPPSEETPLIPQRSCSLLSTEAGALHVLLPARGPGPPQRLSFSFGDHLAEDLCVQAAKASGILPVYHSLFALATEDLSCWFPPSHIFSVEDASTQVLLYRIRFYFPNWF   110
JAK3-207     MAPPSEETPLIPQRSCSLLSTEAGALHVLLPARGPGPPQRLSFSFGDHLAEDLCVQAAKASGILPVYHSLFALATEDLSCWFPPSHIFSVEDASTQVLLYRIRFYFPNWF   110

logo
             GLEKCHRFGLRKDLASAILDLPVLEHLFAQHRSDLVSGRLPVGLSLKEQGECLSLAVLDLARMAREQAQRPGELLKTVSYKACLPPSLRDLIQGLSFVTRRRIRRTVRRA
TOFACITINIB  ..............................................................................................................     0
PF07714.1    ..............................................................................................................     0
PF07714      ..............................................................................................................     0
JAK3-201     GLEKCHRFGLRKDLASAILDLPVLEHLFAQHRSDLVSGRLPVGLSLKEQGECLSLAVLDLARMAREQAQRPGELLKTVSYKACLPPSLRDLIQGLSFVTRRRIRRTVRRA   220
JAK3-204     GLEKCHRFGLRKDLASAILDLPVLEHLFAQHRSDLVSGRLPVGLSLKEQGECLSLAVLDLARMAREQAQRPGELLKTVSYKACLPPSLRDLIQGLSFVTRRRIRRTVRRA   220
JAK3-207     GLEKCHRFGLRKDLASAILDLPVLEHLFAQHRSDLVSGRLPVGLSLKEQGECLSLAVLDLARMAREQAQRPGELLKTVSYKACLPPSLRDLIQGLSFVTRRRIRRTVRRA   220

logo
             LRRVAACQADRHSLMAKYIMDLERLDPAGAAETFHVGLPGALGGHDGLGLLRVAGDGGIAWTQGEQEVLQPFCDFPEIVDISIKQAPRVGPAGEHRLVTVTRTDNQILEA
TOFACITINIB  ..............................................................................................................     0
PF07714.1    ..............................................................................................................     0
PF07714      ..............................................................................................................     0
JAK3-201     LRRVAACQADRHSLMAKYIMDLERLDPAGAAETFHVGLPGALGGHDGLGLLRVAGDGGIAWTQGEQEVLQPFCDFPEIVDISIKQAPRVGPAGEHRLVTVTRTDNQILEA   330
JAK3-204     LRRVAACQADRHSLMAKYIMDLERLDPAGAAETFHVGLPGALGGHDGLGLLRVAGDGGIAWTQGEQEVLQPFCDFPEIVDISIKQAPRVGPAGEHRLVTVTRTDNQILEA   330
JAK3-207     LRRVAACQADRHSLMAKYIMDLERLDPAGAAETFHVGLPGALGGHDGLGLLRVAGDGGIAWTQGEQEVLQPFCDFPEIVDISIKQAPRVGPAGEHRLVTVTRTDNQILEA   330

logo
             EFPGLPEALSFVALVDGYFRLTTDSQHFFCKEVAPPRLLEEVAEQCHGPITLDFAINKLKTGGSRPGSYVLRRSPQDFDSFLLTVCVQNPLGPDYKGCLIRRSPTGTFLL
TOFACITINIB  ..............................................................................................................     0
PF07714.1    ..............................................................................................................     0
PF07714      ..............................................................................................................     0
JAK3-201     EFPGLPEALSFVALVDGYFRLTTDSQHFFCKEVAPPRLLEEVAEQCHGPITLDFAINKLKTGGSRPGSYVLRRSPQDFDSFLLTVCVQNPLGPDYKGCLIRRSPTGTFLL   440
JAK3-204     EFPGLPEALSFVALVDGYFRLTTDSQHFFCKEVAPPRLLEEVAEQCHGPITLDFAINKLKTGGSRPGSYVLRRSPQDFDSFLLTVCVQNPLGPDYKGCLIRRSPTGTFLL   440
JAK3-207     EFPGLPEALSFVALVDGYFRLTTDSQHFFCKEVAPPRLLEEVAEQCHGPITLDFAINKLKTGGSRPGSYVLRRSPQDFDSFLLTVCVQNPLGPDYKGCLIRRSPTGTFLL   440

logo
             VGLSRPHSSLRELLATCWDGGLHVDGVAVTLTSCCIPRPKEKSNLIVVQRGHSPPTSSLVQPQSQYQLSQMTFHKIPADSLEWHENLGHGSFTKIYRGCRHEVVDGEARK
TOFACITINIB  ..............................................................................................................     0
PF07714.1    ................................................................................LEWHENLGHGSFTKIYRGCRHEVVDGEARK    30
PF07714      ................................................................................LEWHENLGHGSFTKIYRGCRHEVVDGEARK    30
JAK3-201     VGLSRPHSSLRELLATCWDGGLHVDGVAVTLTSCCIPRPKEKSNLIVVQRGHSPPTSSLVQPQSQYQLSQMTFHKIPADSLEWHENLGHGSFTKIYRGCRHEVVDGEARK   550
JAK3-204     VGLSRPHSSLRELLATCWDGGLHVDGVAVTLTSCCIPRPKEKSNLIVVQRGHSPPTSSLVQPQSQYQLSQMTFHKIPADSLEWHENLGHGSFTKIYRGCRHEVVDGEARK   550
JAK3-207     VGLSRPHSSLRELLATCWDGGLHVDGVAVTLTSCCIPRPKEKSNLIVVQRGHSPPTSSLVQPQSQYQLSQMTFHKIPADSLEWHENLGHGSFTKIYRGCRHEVVDGEARK   550
```

logo

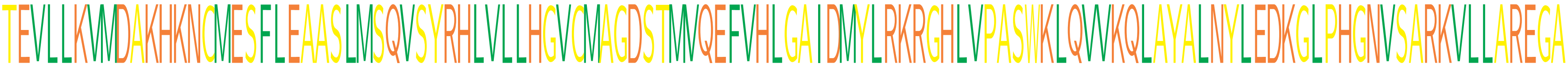

```
TOFACITINIB   ..............................................................................................................     0
PF07714.1     TEVLLKVMDAKHKNCMESFLEAASLMSQVSYRHLVLLHGVCMAGDSTMVQEFVHLGAIDMYLRKRGHLVPASWKLQVVKQLAYALNYLEDKGLPHGNVSARKVLLAREGA   140
PF07714       TEVLLKVMDAKHKNCMESFLEAASLMSQVSYRHLVLLHGVCMAGDSTMVQEFVHLGAIDMYLRKRGHLVPASWKLQVVKQLAYALNYLEDKGLPHGNVSARKVLLAREGA   140
JAK3-201      TEVLLKVMDAKHKNCMESFLEAASLMSQVSYRHLVLLHGVCMAGDSTMVQEFVHLGAIDMYLRKRGHLVPASWKLQVVKQLAYALNYLEDKGLPHGNVSARKVLLAREGA   660
JAK3-204      TEVLLKVMDAKHKNCMESFLEAASLMSQVSYRHLVLLHGVCMAGDSTMVQEFVHLGAIDMYLRKRGHLVPASWKLQVVKQLAYALNYLEDKGLPHGNVSARKVLLAREGA   660
JAK3-207      TEVLLKVMDAKHKNCMESFLEAASLMSQVSYRHLVLLHGVCMAGDSTMVQEFVHLGAIDMYLRKRGHLVPASWKLQVVKQLAYALNYLEDKGLPHGNVSARKVLLAREGA   660
```

logo

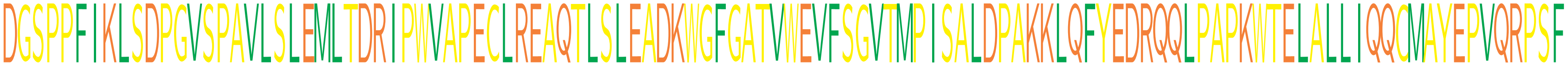

```
TOFACITINIB   ..............................................................................................................     0
PF07714.1     DGSPPFIKLSDPGVSPAVLSLEMLTDRIPWVAPECLREAQTLSLEADKWGFGATVWEVFSGVTMPISALDPAKKLQFYEDRQQLPAPKWTELALLIQQCMAYEPVQRPSF   250
PF07714       DGSPPFIKLSDPGVSPAVLSLEMLTDRIPWVAPECLREAQTLSLEADKWGFGATVWEVFSGVTMPISALDPAKKLQFYEDRQQLPAPKWTELALLIQQCMAYEPVQRPSF   250
JAK3-201      DGSPPFIKLSDPGVSPAVLSLEMLTDRIPWVAPECLREAQTLSLEADKWGFGATVWEVFSGVTMPISALDPAKKLQFYEDRQQLPAPKWTELALLIQQCMAYEPVQRPSF   770
JAK3-204      DGSPPFIKLSDPGVSPAVLSLEMLTDRIPWVAPECLREAQTLSLEADKWGFGATVWEVFSGVTMPISALDPAKKLQFYEDRQQLPAPKWTELALLIQQCMAYEPVQRPSF   770
JAK3-207      DGSPPFIKLSDPGVSPAVLSLEMLTDRIPWVAPECLREAQTLSLEADKWGFGATVWEVFSGVTMPISALDPAKKLQFYEDRQQLPAPKWTELALLIQQCMAYEPVQRPSF   770
```

logo

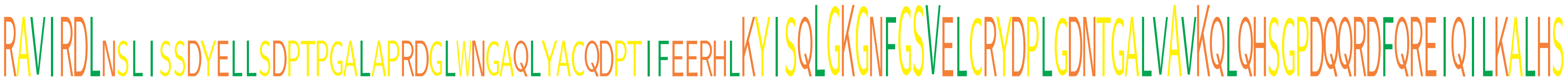

```
TOFACITINIB   .........................................................LGKG..GSV................A.K.........................     9
PF07714.1     RAVIRDL.............................................KYISQLGKGNFGSVELCRYDPLGDNTGALVAVKQLQHSGPDQQRDFQREIQILKALHS   315
PF07714       RAVIRDL.............................................KYISQLGKGNFGSVELCRYDPLGDNTGALVAVKQLQHSGPDQQRDFQREIQILKALHS   315
JAK3-201      RAVIRDLNSLISSDYELLSDPTPGALAPRDGLWNGAQLYACQDPTIFEERHLKYISQLGKGNFGSVELCRYDPLGDNTGALVAVKQLQHSGPDQQRDFQREIQILKALHS   880
JAK3-204      RAVIRDLNSLISSDYELLSDPTPGALAPRDGLWNGAQLYACQDPTIFEERHLKYISQLGKGNFGSVELCRYDPLGDNTGALVAVKQLQHSGPDQQRDFQREIQILKALHS   880
JAK3-207      RAVIRDLNSLISSDYELLSDPTPGALAPRDGLWNGAQLYACQDPTIFEERHLKYISQLGKGNFGSVELCRYDPLGDNTGALVAVKQLQHSGPDQQRDFQREIQILKALHS   880
```

logo

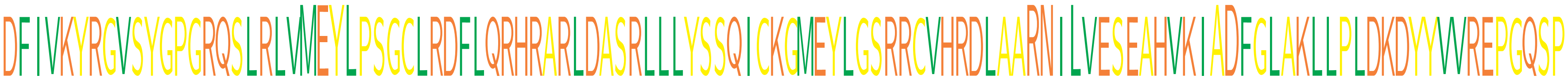

```
TOFACITINIB   .....................MEYL...............................................RN.L.........AD.......................    18
PF07714.1     DFIVKYRGVSYGPGRQSLRLVMEYLPSGCLRDFLQRHRARLDASRLLLYSSQICKGMEYLGSRRCVHRDLAARNILVESEAHVKIADFGLAKLLPLDKDYYVVREPGQSP   425
PF07714       DFIVKYRGVSYGPGRQSLRLVMEYLPSGCLRDFLQRHRARLDASRLLLYSSQICKGMEYLGSRRCVHRDLAARNILVESEAHVKIADFGLAKLLPLDKDYYVVREPGQSP   425
JAK3-201      DFIVKYRGVSYGPGRQSLRLVMEYLPSGCLRDFLQRHRARLDASRLLLYSSQICKGMEYLGSRRCVHRDLAARNILVESEAHVKIADFGLAKLLPLDKDYYVVREPGQSP   990
JAK3-204      DFIVKYRGVSYGPGRQSLRLVMEYLPSGCLRDFLQRHRARLDASRLLLYSSQICKGMEYLGSRRCVHRDLAARNILVESEAHVKIADFGLAKLLPLDKDYYVVREPGQSP   990
JAK3-207      DFIVKYRGVSYGPGRQSLRLVMEYLPSGCLRDFLQRHRARLDASRLLLYSSQICKGMEYLGSRRCVHRDLAARNILVESEAHVKIADFGLAKLLPLDKDYYVVREPGQSP   990
```

logo

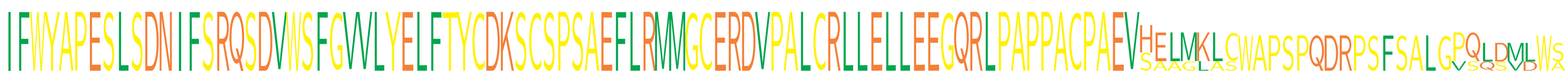

```
TOFACITINIB   ..............................................................................................................    18
PF07714.1     IFWYAPESLSDNIFSRQSDVWSFGVVLYELFTYCDKSCSPSAEFLRMMGCERDVPALCRLLELLEEGQRLPAPPACPAEVS.............................   506
PF07714       IFWYAPESLSDNIFSRQSDVWSFGVVLYELFTYCDKSCSPSAEFLRMMGCERDVPALCRLLELLEEGQRLPAPPACPAEVHELMKLCWAPSPQDRPSFSALGPQ......   529
JAK3-201      IFWYAPESLSDNIFSRQSDVWSFGVVLYELFTYCDKSCSPSAEFLRMMGCERDVPALCRLLELLEEGQRLPAPPACPAEVHELMKLCWAPSPQDRPSFSALGPQLDMLWS  1100
JAK3-204      IFWYAPESLSDNIFSRQSDVWSFGVVLYELFTYCDKSCSPSAEFLRMMGCERDVPALCRLLELLEEGQRLPAPPACPAEVHELMKLCWAPSPQDRPSFSALGPQLDMLWS  1100
JAK3-207      IFWYAPESLSDNIFSRQSDVWSFGVVLYELFTYCDKSCSPSAEFLRMMGCERDVPALCRLLELLEEGQRLPAPPACPAEVSAAGLAS...............VSQSVDWA  1085
```

```
logo
```

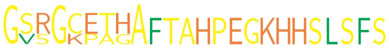

```
TOFACITININB  ........................   18
PF07714.1     ........................  506
PF07714       ........................  529
JAK3-201      GSRGCETHAFTAHPEGKHHSLSFS 1124
JAK3-204      GSRGCETHAFTAHPEGKHHSLSFS 1124
JAK3-207      GVSGKPAGA............... 1094
```

X non conserved
X similar
X $\geq 0\%$ conserved
X $\geq 50\%$ conserved

```
logo
         MQSKVLLAVALWLCVETRAASVGLPSVSLDLPRLSIQKDILTIKANTTLQITCRGQRDLDWLWPNNQSGSEQRVEVTECSDGLFCKTLTIPKVIGNDTGAYKCFYRETDLASVIY
AXITINIB .................................................................................................................... 0
PF07679  .................................................................................................................... 0
PF13927  .................................................................................................................... 0
PF07714  .................................................................................................................... 0
PF00047  .................................................................................................................... 0
KDR-201  MQSKVLLAVALWLCVETRAASVGLPSVSLDLPRLSIQKDILTIKANTTLQITCRGQRDLDWLWPNNQSGSEQRVEVTECSDGLFCKTLTIPKVIGNDTGAYKCFYRETDLASVIY 115

logo
         VYVQDYRSPFIASVSDQHGVVYITENKNKTVVIPCLGSISNLNVSLCARYPEKRFVPDGNRISWDSKKGFTIPSYMISYAGMVFCEAKINDESYQSIMYIVVVVGYRIYDVVLSP
AXITINIB .................................................................................................................... 0
PF07679  .................................................................................................................... 0
PF13927  .................................................................................................................... 0
PF07714  .................................................................................................................... 0
PF00047  ...................................................................................................................P 1
KDR-201  VYVQDYRSPFIASVSDQHGVVYITENKNKTVVIPCLGSISNLNVSLCARYPEKRFVPDGNRISWDSKKGFTIPSYMISYAGMVFCEAKINDESYQSIMYIVVVVGYRIYDVVLSP 230

logo
         SHGIELSVGEKLVLNCTARTELNVGIDFNWEYPSSKHQHKKLVNRDLKTQSGSEMKKFLSTLTIDGVTRSDQGLYTCAASSGLMTKKNSTFVRVHEKPFVAFGSGMESLVEATVG
AXITINIB .................................................................................................................... 0
PF07679  ..............................................................................................................VEATVG 6
PF13927  .................................................................................................................... 0
PF07714  .................................................................................................................... 0
PF00047  SHGIELSVGEKLVLNCTARTELNVGIDFNWEYPSSKHQHKKLVNRDLKTQSGSEMKKFLSTLTIDGVTRSDQGLYTCAAS................................... 81
KDR-201  SHGIELSVGEKLVLNCTARTELNVGIDFNWEYPSSKHQHKKLVNRDLKTQSGSEMKKFLSTLTIDGVTRSDQGLYTCAASSGLMTKKNSTFVRVHEKPFVAFGSGMESLVEATVG 345

logo
         ERVRIPAKYLGYPPPEIKWYKNGIPLESNHTIKAGHVLTIMEVSERDTGNYTVILTNPISKEKQSHVVSLVVYVPPQIGEKSLISPVDSYQYGTTQTLTCTVYAIPPPHHIHWYW
AXITINIB .................................................................................................................... 0
PF07679  ERVRIPAKYLGYPPPEIKWYKNGIPLESNHTIKAGHVLTIMEVSERDTGNYTVILTNPISKEKQS................................................. 71
PF13927  .................................................................................................................... 0
PF07714  .................................................................................................................... 0
PF00047  .................................................................................................................... 81
KDR-201  ERVRIPAKYLGYPPPEIKWYKNGIPLESNHTIKAGHVLTIMEVSERDTGNYTVILTNPISKEKQSHVVSLVVYVPPQIGEKSLISPVDSYQYGTTQTLTCTVYAIPPPHHIHWYW 460

logo
         QLEEECANEPSQAVSVTNPYPCEEWRSVEDFQGGNKIEVNKNQFALIEGKNKTVSTLVIQAANVSALYKCEAVNKVGRGERVISFHVTRGPEITLQPDMQPTEQESVSLWCTADR
AXITINIB .................................................................................................................... 0
PF07679  .................................................................................................................... 71
PF13927  ..........................................................................................PEITLQPDMQPTEQESVSLWCTADR 25
PF07714  .................................................................................................................... 0
PF00047  .................................................................................................................... 81
KDR-201  QLEEECANEPSQAVSVTNPYPCEEWRSVEDFQGGNKIEVNKNQFALIEGKNKTVSTLVIQAANVSALYKCEAVNKVGRGERVISFHVTRGPEITLQPDMQPTEQESVSLWCTADR 575
```

```
logo
          STFENLTWYKLGPQPLPIHVGELPTPVCKNLDTLWKLNATMFSNSTNDILIMELKNASLQDQGDYVCLAQDRKTKKRHCVVRQLTVLERVAPTITGNLENQTTSIGESIEVSCTA
AXITINIB  ...................................................................................................................     0
PF07679   ...........................................................................................PTITGNLENQTTSIGESIEVSCTA    95
PF13927   STFENLTWYKLGPQPLPIHVGELPTPVCKNLDTLWKLNATMFSNSTNDILIMELKNASLQDQGDYVCLAQ.............................................    95
PF07714   ...................................................................................................................     0
PF00047   ...................................................................................................................    81
KDR-201   STFENLTWYKLGPQPLPIHVGELPTPVCKNLDTLWKLNATMFSNSTNDILIMELKNASLQDQGDYVCLAQDRKTKKRHCVVRQLTVLERVAPTITGNLENQTTSIGESIEVSCTA   690

logo
          SGNPPPQIMWFKDNETLVEDSGIVLKDGNRNLTIRRVRKEDEGLYTCQACSVLGCAKVEAFFIIEGAQEKTNLEIIILVGTAVIAMFFWLLLVIILRTVKRANGGELKTGYLSIV
AXITINIB  ...................................................................................................................     0
PF07679   SGNPPPQIMWFKDNETLVEDSGIVLKDGNRNLTIRRVRKEDEGLYTCQACSVLGCAKVEA.......................................................   155
PF13927   ...................................................................................................................    95
PF07714   ...................................................................................................................     0
PF00047   ...................................................................................................................    81
KDR-201   SGNPPPQIMWFKDNETLVEDSGIVLKDGNRNLTIRRVRKEDEGLYTCQACSVLGCAKVEAFFIIEGAQEKTNLEIIILVGTAVIAMFFWLLLVIILRTVKRANGGELKTGYLSIV   805

logo
          MDPDELPLDEHCERLPYDASKWEFPRDRLKLGKPLGRGAFGQVIEADAFGIDKTATCRTVAVKMLKEGATHSEHRALMSELKILIHIGHHLNVVNLLGACTKPGGPLMVIVEFCK
AXITINIB  ..................................L.......V.................A.K................E.............V..............V.VEFCK    12
PF07679   ...................................................................................................................   155
PF13927   ...................................................................................................................    95
PF07714   ............................LKLGKPLGRGAFGQVIEADAFGIDKTATCRTVAVKMLKEGATHSEHRALMSELKILIHIGHHLNVVNLLGACTKPGGPLMVIVEFCK    87
PF00047   ...................................................................................................................    81
KDR-201   MDPDELPLDEHCERLPYDASKWEFPRDRLKLGKPLGRGAFGQVIEADAFGIDKTATCRTVAVKMLKEGATHSEHRALMSELKILIHIGHHLNVVNLLGACTKPGGPLMVIVEFCK   920

logo
          FGNLSTYLRSKRNEFVPYKTKGARFRQGKDYVGAIPVDLKRRLDSITSSQSSASSGFVEEKSLSDVEEEEAPEDLYKDFLTLEHLICYSFQVAKGMEFLASRKCIHRDLAARNIL
AXITINIB  .G................................................................................................................L    14
PF07679   ...................................................................................................................   155
PF13927   ...................................................................................................................    95
PF07714   FGNLSTYLRSKRNEFVPYKTKGARFRQGKDYVGAIPVDLKRRLDSITSSQSSASSGFVEEKSLSDVEEEEAPEDLYKDFLTLEHLICYSFQVAKGMEFLASRKCIHRDLAARNIL   202
PF00047   ...................................................................................................................    81
KDR-201   FGNLSTYLRSKRNEFVPYKTKGARFRQGKDYVGAIPVDLKRRLDSITSSQSSASSGFVEEKSLSDVEEEEAPEDLYKDFLTLEHLICYSFQVAKGMEFLASRKCIHRDLAARNIL  1035

logo
          LSEKNVVKICDFGLARDIYKDPDYVRKGDARLPLKWMAPETIFDRVYTIQSDVWSFGVLLWEIFSLGASPYPGVKIDEEFCRRLKEGTRMRAPDYTTPEMYQTMLDCWHGEPSQR
AXITINIB  .........CDF.......................................................................................................    17
PF07679   ...................................................................................................................   155
PF13927   ...................................................................................................................    95
PF07714   LSEKNVVKICDFGLARDIYKDPDYVRKGDARLPLKWMAPETIFDRVYTIQSDVWSFGVLLWEIFSLGASPYPGVKIDEEFCRRLKEGTRMRAPDYTTPEMYQTMLDCWHGEPSQR   317
PF00047   ...................................................................................................................    81
KDR-201   LSEKNVVKICDFGLARDIYKDPDYVRKGDARLPLKWMAPETIFDRVYTIQSDVWSFGVLLWEIFSLGASPYPGVKIDEEFCRRLKEGTRMRAPDYTTPEMYQTMLDCWHGEPSQR  1150
```

```
logo
          PTFSELVEHLGNLLQANAQQDGKDYIVLPISETLSMEEDSGLSLPTSPVSCMEEEEVCDPKFHYDNTAGISQYLQNSKRKSRPVSVKTFEDIPLEEPEVKVIPDDNQTDSGMVLA
AXITINIB  ...................................................................................................................    17
PF07679   ...................................................................................................................   155
PF13927   ...................................................................................................................    95
PF07714   PTFSELVEHL.........................................................................................................   327
PF00047   ...................................................................................................................    81
KDR-201   PTFSELVEHLGNLLQANAQQDGKDYIVLPISETLSMEEDSGLSLPTSPVSCMEEEEVCDPKFHYDNTAGISQYLQNSKRKSRPVSVKTFEDIPLEEPEVKVIPDDNQTDSGMVLA  1265


logo
          SEELKTLEDRTKLSPSFGGMVPSKSRESVASEGSNQTSGYQSGYHSDDTDTTVYSSEEAELLKLIEIGVQTGSTAQILQPDSGTTLSSPPV
AXITINIB  ...........................................................................................    17
PF07679   ...........................................................................................   155
PF13927   ...........................................................................................    95
PF07714   ...........................................................................................   327
PF00047   ...........................................................................................    81
KDR-201   SEELKTLEDRTKLSPSFGGMVPSKSRESVASEGSNQTSGYQSGYHSDDTDTTVYSSEEAELLKLIEIGVQTGSTAQILQPDSGTTLSSPPV  1356
```

- X non conserved
- X similar
- X $\geq 0\%$ conserved
- X $\geq 50\%$ conserved

```
logo
           MQSKVLLAVALWLCVETRAASVGLPSVSLDLPRLSIQKDILTIKANTTLQITCRGQRDLDWLWPNNQSGSEQRVEVTECSDGLFCKTLTIPKVIGNDTGAYKCFYRETDL
LENVATINIB ..............................................................................................................   0
PF07679    ..............................................................................................................   0
PF13927    ..............................................................................................................   0
PF07714    ..............................................................................................................   0
PF00047    ..............................................................................................................   0
KDR-201    MQSKVLLAVALWLCVETRAASVGLPSVSLDLPRLSIQKDILTIKANTTLQITCRGQRDLDWLWPNNQSGSEQRVEVTECSDGLFCKTLTIPKVIGNDTGAYKCFYRETDL 110

logo
           ASVIYVYVQDYRSPFIASVSDQHGVVYITENKNKTVVIPCLGSISNLNVSLCARYPEKRFVPDGNRISWDSKKGFTIPSYMISYAGMVFCEAKINDESYQSIMYIVVVVG
LENVATINIB ..............................................................................................................   0
PF07679    ..............................................................................................................   0
PF13927    ..............................................................................................................   0
PF07714    ..............................................................................................................   0
PF00047    ..............................................................................................................   0
KDR-201    ASVIYVYVQDYRSPFIASVSDQHGVVYITENKNKTVVIPCLGSISNLNVSLCARYPEKRFVPDGNRISWDSKKGFTIPSYMISYAGMVFCEAKINDESYQSIMYIVVVVG 220

logo
           YRIYDVVLSPSHGIELSVGEKLVLNCTARTELNVGIDFNWEYPSSKHQHKKLVNRDLKTQSGSEMKKFLSTLTIDGVTRSDQGLYTCAASSGLMTKKNSTFVRVHEKPFV
LENVATINIB ..............................................................................................................   0
PF07679    ..............................................................................................................   0
PF13927    ..............................................................................................................   0
PF07714    ..............................................................................................................   0
PF00047    .........PSHGIELSVGEKLVLNCTARTELNVGIDFNWEYPSSKHQHKKLVNRDLKTQSGSEMKKFLSTLTIDGVTRSDQGLYTCAAS....................  81
KDR-201    YRIYDVVLSPSHGIELSVGEKLVLNCTARTELNVGIDFNWEYPSSKHQHKKLVNRDLKTQSGSEMKKFLSTLTIDGVTRSDQGLYTCAASSGLMTKKNSTFVRVHEKPFV 330

logo
           AFGSGMESLVEATVGERVRIPAKYLGYPPPEIKWYKNGIPLESNHTIKAGHVLTIMEVSERDTGNYTVILTNPISKEKQSHVVSLVVYVPPQIGEKSLISPVDSYQYGTT
LENVATINIB ..............................................................................................................   0
PF07679    .........VEATVGERVRIPAKYLGYPPPEIKWYKNGIPLESNHTIKAGHVLTIMEVSERDTGNYTVILTNPISKEKQS..............................  71
PF13927    ..............................................................................................................   0
PF07714    ..............................................................................................................   0
PF00047    ..............................................................................................................  81
KDR-201    AFGSGMESLVEATVGERVRIPAKYLGYPPPEIKWYKNGIPLESNHTIKAGHVLTIMEVSERDTGNYTVILTNPISKEKQSHVVSLVVYVPPQIGEKSLISPVDSYQYGTT 440

logo
           QTLTCTVYAIPPPHHIHWYWQLEEECANEPSQAVSVTNPYPCEEWRSVEDFQGGNKIEVNKNQFALIEGKNKTVSTLVIQAANVSALYKCEAVNKVGRGERVISFHVTRG
LENVATINIB ..............................................................................................................   0
PF07679    ..............................................................................................................  71
PF13927    ..............................................................................................................   0
PF07714    ..............................................................................................................   0
PF00047    ..............................................................................................................  81
KDR-201    QTLTCTVYAIPPPHHIHWYWQLEEECANEPSQAVSVTNPYPCEEWRSVEDFQGGNKIEVNKNQFALIEGKNKTVSTLVIQAANVSALYKCEAVNKVGRGERVISFHVTRG 550
```

```
logo
            PEITLQPDMQPTEQESVSLWCTADRSTFENLTWYKLGPQPLPIHVGELPTPVCKNLDTLWKLNATMFSNSTNDILIMELKNASLQDQGDYVCLAQDRKTKKRHCVVRQLT
LENVATINIB  ..............................................................................................................    0
PF07679     ..............................................................................................................   71
PF13927     PEITLQPDMQPTEQESVSLWCTADRSTFENLTWYKLGPQPLPIHVGELPTPVCKNLDTLWKLNATMFSNSTNDILIMELKNASLQDQGDYVCLAQ...............   95
PF07714     ..............................................................................................................    0
PF00047     ..............................................................................................................   81
KDR-201     PEITLQPDMQPTEQESVSLWCTADRSTFENLTWYKLGPQPLPIHVGELPTPVCKNLDTLWKLNATMFSNSTNDILIMELKNASLQDQGDYVCLAQDRKTKKRHCVVRQLT  660

logo
            VLERVAPTITGNLENQTTSIGESIEVSCTASGNPPPQIMWFKDNETLVEDSGIVLKDGNRNLTIRRVRKEDEGLYTCQACSVLGCAKVEAFFIIEGAQEKTNLEIIILVG
LENVATINIB  ..............................................................................................................    0
PF07679     ......PTITGNLENQTTSIGESIEVSCTASGNPPPQIMWFKDNETLVEDSGIVLKDGNRNLTIRRVRKEDEGLYTCQACSVLGCAKVEA....................  155
PF13927     ..............................................................................................................   95
PF07714     ..............................................................................................................    0
PF00047     ..............................................................................................................   81
KDR-201     VLERVAPTITGNLENQTTSIGESIEVSCTASGNPPPQIMWFKDNETLVEDSGIVLKDGNRNLTIRRVRKEDEGLYTCQACSVLGCAKVEAFFIIEGAQEKTNLEIIILVG  770

logo
            TAVIAMFFWLLLVIILRTVKRANGGELKTGYLSIVMDPDELPLDEHCERLPYDASKWEFPRDRLKLGKPLGRGAFGQVIEADAFGIDKTATCRTVAVKMLKEGATHSEHR
LENVATINIB  .....................................................................LG........................A.K............    4
PF07679     ..............................................................................................................  155
PF13927     ..............................................................................................................   95
PF07714     ...............................................................LKLGKPLGRGAFGQVIEADAFGIDKTATCRTVAVKMLKEGATHSEHR   47
PF00047     ..............................................................................................................   81
KDR-201     TAVIAMFFWLLLVIILRTVKRANGGELKTGYLSIVMDPDELPLDEHCERLPYDASKWEFPRDRLKLGKPLGRGAFGQVIEADAFGIDKTATCRTVAVKMLKEGATHSEHR  880

logo
            ALMSELKILIHIGHHLNVVNLLGACTKPGGPLMVIVEFCKFGNLSTYLRSKRNEFVPYKTKGARFRQGKDYVGAIPVDLKRRLDSITSSQSSASSGFVEEKSLSDVEEEE
LENVATINIB  ....E..IL.........V................VEFCK.G....................................................................   14
PF07679     ..............................................................................................................  155
PF13927     ..............................................................................................................   95
PF07714     ALMSELKILIHIGHHLNVVNLLGACTKPGGPLMVIVEFCKFGNLSTYLRSKRNEFVPYKTKGARFRQGKDYVGAIPVDLKRRLDSITSSQSSASSGFVEEKSLSDVEEEE  157
PF00047     ..............................................................................................................   81
KDR-201     ALMSELKILIHIGHHLNVVNLLGACTKPGGPLMVIVEFCKFGNLSTYLRSKRNEFVPYKTKGARFRQGKDYVGAIPVDLKRRLDSITSSQSSASSGFVEEKSLSDVEEEE  990

logo
            APEDLYKDFLTLEHLICYSFQVAKGMEFLASRKCIHRDLAARNILLSEKNVVKICDFGLARDIYKDPDYVRKGDARLPLKWMAPETIFDRVYTIQSDVWSFGVLLWEIFS
LENVATINIB  ............................................L.........CDF.L...................................................   19
PF07679     ..............................................................................................................  155
PF13927     ..............................................................................................................   95
PF07714     APEDLYKDFLTLEHLICYSFQVAKGMEFLASRKCIHRDLAARNILLSEKNVVKICDFGLARDIYKDPDYVRKGDARLPLKWMAPETIFDRVYTIQSDVWSFGVLLWEIFS  267
PF00047     ..............................................................................................................   81
KDR-201     APEDLYKDFLTLEHLICYSFQVAKGMEFLASRKCIHRDLAARNILLSEKNVVKICDFGLARDIYKDPDYVRKGDARLPLKWMAPETIFDRVYTIQSDVWSFGVLLWEIFS 1100
```

```
logo
            LGASPYPGVKIDEEFCRRLKEGTRMRAPDYTTPEMYQTMLDCWHGEPSQRPTFSELVEHLGNLLQANAQQDGKDYIVLPISETLSMEEDSGLSLPTSPVSCMEEEEVCDP
LENVATINIB  ..............................................................................................................    19
PF07679     ..............................................................................................................   155
PF13927     ..............................................................................................................    95
PF07714     LGASPYPGVKIDEEFCRRLKEGTRMRAPDYTTPEMYQTMLDCWHGEPSQRPTFSELVEHL..................................................   327
PF00047     ..............................................................................................................    81
KDR-201     LGASPYPGVKIDEEFCRRLKEGTRMRAPDYTTPEMYQTMLDCWHGEPSQRPTFSELVEHLGNLLQANAQQDGKDYIVLPISETLSMEEDSGLSLPTSPVSCMEEEEVCDP  1210

logo
            KFHYDNTAGISQYLQNSKRKSRPVSVKTFEDIPLEEPEVKVIPDDNQTDSGMVLASEELKTLEDRTKLSPSFGGMVPSKSRESVASEGSNQTSGYQSGYHSDDTDTTVYS
LENVATINIB  ..............................................................................................................    19
PF07679     ..............................................................................................................   155
PF13927     ..............................................................................................................    95
PF07714     ..............................................................................................................   327
PF00047     ..............................................................................................................    81
KDR-201     KFHYDNTAGISQYLQNSKRKSRPVSVKTFEDIPLEEPEVKVIPDDNQTDSGMVLASEELKTLEDRTKLSPSFGGMVPSKSRESVASEGSNQTSGYQSGYHSDDTDTTVYS  1320

logo
            SEEAELLKLIEIGVQTGSTAQILQPDSGTTLSSPPV
LENVATINIB  ....................................    19
PF07679     ....................................   155
PF13927     ....................................    95
PF07714     ....................................   327
PF00047     ....................................    81
KDR-201     SEEAELLKLIEIGVQTGSTAQILQPDSGTTLSSPPV  1356
```

X non conserved

X similar

X $\geq 0\%$ conserved

X $\geq 50\%$ conserved

```
logo
             MQSKVLLAVALWLCVETRAASVGLPSVSLDLPRLSIQKDILTIKANTTLQITCRGQRDLDWLWPNNQSGSEQRVEVTECSDGLFCKTLTIPKVIGNDTGAYKCFYRETDL
MOTESANIB    ..............................................................................................................     0
PF07679      ..............................................................................................................     0
PF13927      ..............................................................................................................     0
PF07714      ..............................................................................................................     0
PF00047      ..............................................................................................................     0
KDR-201      MQSKVLLAVALWLCVETRAASVGLPSVSLDLPRLSIQKDILTIKANTTLQITCRGQRDLDWLWPNNQSGSEQRVEVTECSDGLFCKTLTIPKVIGNDTGAYKCFYRETDL   110

logo
             ASVIYVYVQDYRSPFIASVSDQHGVVYITENKNKTVVIPCLGSISNLNVSLCARYPEKRFVPDGNRISWDSKKGFTIPSYMISYAGMVFCEAKINDESYQSIMYIVVVVG
MOTESANIB    ..............................................................................................................     0
PF07679      ..............................................................................................................     0
PF13927      ..............................................................................................................     0
PF07714      ..............................................................................................................     0
PF00047      ..............................................................................................................     0
KDR-201      ASVIYVYVQDYRSPFIASVSDQHGVVYITENKNKTVVIPCLGSISNLNVSLCARYPEKRFVPDGNRISWDSKKGFTIPSYMISYAGMVFCEAKINDESYQSIMYIVVVVG   220

logo
             YRIYDVVLSPSHGIELSVGEKLVLNCTARTELNVGIDFNWEYPSSKHQHKKLVNRDLKTQSGSEMKKFLSTLTIDGVTRSDQGLYTCAASSGLMTKKNSTFVRVHEKPFV
MOTESANIB    ..............................................................................................................     0
PF07679      ..............................................................................................................     0
PF13927      ..............................................................................................................     0
PF07714      ..............................................................................................................     0
PF00047      .........PSHGIELSVGEKLVLNCTARTELNVGIDFNWEYPSSKHQHKKLVNRDLKTQSGSEMKKFLSTLTIDGVTRSDQGLYTCAAS....................    81
KDR-201      YRIYDVVLSPSHGIELSVGEKLVLNCTARTELNVGIDFNWEYPSSKHQHKKLVNRDLKTQSGSEMKKFLSTLTIDGVTRSDQGLYTCAASSGLMTKKNSTFVRVHEKPFV   330

logo
             AFGSGMESLVEATVGERVRIPAKYLGYPPPEIKWYKNGIPLESNHTIKAGHVLTIMEVSERDTGNYTVILTNPISKEKQSHVVSLVVYVPPQIGEKSLISPVDSYQYGTT
MOTESANIB    ..............................................................................................................     0
PF07679      .........VEATVGERVRIPAKYLGYPPPEIKWYKNGIPLESNHTIKAGHVLTIMEVSERDTGNYTVILTNPISKEKQS..............................    71
PF13927      ..............................................................................................................     0
PF07714      ..............................................................................................................     0
PF00047      ..............................................................................................................    81
KDR-201      AFGSGMESLVEATVGERVRIPAKYLGYPPPEIKWYKNGIPLESNHTIKAGHVLTIMEVSERDTGNYTVILTNPISKEKQSHVVSLVVYVPPQIGEKSLISPVDSYQYGTT   440

logo
             QTLTCTVYAIPPPHHIHWYWQLEEECANEPSQAVSVTNPYPCEEWRSVEDFQGGNKIEVNKNQFALIEGKNKTVSTLVIQAANVSALYKCEAVNKVGRGERVISFHVTRG
MOTESANIB    ..............................................................................................................     0
PF07679      ..............................................................................................................    71
PF13927      ..............................................................................................................     0
PF07714      ..............................................................................................................     0
PF00047      ..............................................................................................................    81
KDR-201      QTLTCTVYAIPPPHHIHWYWQLEEECANEPSQAVSVTNPYPCEEWRSVEDFQGGNKIEVNKNQFALIEGKNKTVSTLVIQAANVSALYKCEAVNKVGRGERVISFHVTRG   550
```

```
logo
MOTESANIB  ..............................................................................................................    0
PF07679    ..............................................................................................................   71
PF13927    PEITLQPDMQPTEQESVSLWCTADRSTFENLTWYKLGPQPLPIHVGELPTPVCKNLDTLWKLNATMFSNSTNDILIMELKNASLQDQGDYVCLAQ...............   95
PF07714    ..............................................................................................................    0
PF00047    ..............................................................................................................   81
KDR-201    PEITLQPDMQPTEQESVSLWCTADRSTFENLTWYKLGPQPLPIHVGELPTPVCKNLDTLWKLNATMFSNSTNDILIMELKNASLQDQGDYVCLAQDRKTKKRHCVVRQLT  660

logo
MOTESANIB  ..............................................................................................................    0
PF07679    ......PTITGNLENQTTSIGESIEVSCTASGNPPPQIMWFKDNETLVEDSGIVLKDGNRNLTIRRVRKEDEGLYTCQACSVLGCAKVEA....................  155
PF13927    ..............................................................................................................   95
PF07714    ..............................................................................................................    0
PF00047    ..............................................................................................................   81
KDR-201    VLERVAPTITGNLENQTTSIGESIEVSCTASGNPPPQIMWFKDNETLVEDSGIVLKDGNRNLTIRRVRKEDEGLYTCQACSVLGCAKVEAFFIIEGAQEKTNLEIIILVG  770

logo
MOTESANIB  .....................................................................L.......V.................AVK............    5
PF07679    ..............................................................................................................  155
PF13927    ..............................................................................................................   95
PF07714    ...............................................................LKLGKPLGRGAFGQVIEADAFGIDKTATCRTVAVKMLKEGATHSEHR   47
PF00047    ..............................................................................................................   81
KDR-201    TAVIAMFFWLLLVIILRTVKRANGGELKTGYLSIVMDPDELPLDEHCERLPYDASKWEFPRDRLKLGKPLGRGAFGQVIEADAFGIDKTATCRTVAVKMLKEGATHSEHR  880

logo
MOTESANIB  ....E...L..I......V..............V.VEFC.......................................................................   14
PF07679    ..............................................................................................................  155
PF13927    ..............................................................................................................   95
PF07714    ALMSELKILIHIGHHLNVVNLLGACTKPGGPLMVIVEFCKFGNLSTYLRSKRNEFVPYKTKGARFRQGKDYVGAIPVDLKRRLDSITSSQSSASSGFVEEKSLSDVEEEE  157
PF00047    ..............................................................................................................   81
KDR-201    ALMSELKILIHIGHHLNVVNLLGACTKPGGPLMVIVEFCKFGNLSTYLRSKRNEFVPYKTKGARFRQGKDYVGAIPVDLKRRLDSITSSQSSASSGFVEEKSLSDVEEEE  990

logo
MOTESANIB  ............................L......H........L.........CDF.....................................................   20
PF07679    ..............................................................................................................  155
PF13927    ..............................................................................................................   95
PF07714    APEDLYKDFLTLEHLICYSFQVAKGMEFLASRKCIHRDLAARNILLSEKNVVKICDFGLARDIYKDPDYVRKGDARLPLKWMAPETIFDRVYTIQSDVWSFGVLLWEIFS  267
PF00047    ..............................................................................................................   81
KDR-201    APEDLYKDFLTLEHLICYSFQVAKGMEFLASRKCIHRDLAARNILLSEKNVVKICDFGLARDIYKDPDYVRKGDARLPLKWMAPETIFDRVYTIQSDVWSFGVLLWEIFS 1100
```

```
logo
          LGASPYPGVKIDEEFCRRLKEGTRMRAPDYTTPEMYQTMLDCWHGEPSQRPTFSELVEHLGNLLQANAQQDGKDYIVLPISETLSMEEDSGLSLPTSPVSCMEEEEVCDP
MOTESANIB ..............................................................................................................    20
PF07679   ..............................................................................................................   155
PF13927   ..............................................................................................................    95
PF07714   LGASPYPGVKIDEEFCRRLKEGTRMRAPDYTTPEMYQTMLDCWHGEPSQRPTFSELVEHL..................................................   327
PF00047   ..............................................................................................................    81
KDR-201   LGASPYPGVKIDEEFCRRLKEGTRMRAPDYTTPEMYQTMLDCWHGEPSQRPTFSELVEHLGNLLQANAQQDGKDYIVLPISETLSMEEDSGLSLPTSPVSCMEEEEVCDP  1210

logo
          KFHYDNTAGISQYLQNSKRKSRPVSVKTFEDIPLEEPEVKVIPDDNQTDSGMVLASEELKTLEDRTKLSPSFGGMVPSKSRESVASEGSNQTSGYQSGYHSDDTDTTVYS
MOTESANIB ..............................................................................................................    20
PF07679   ..............................................................................................................   155
PF13927   ..............................................................................................................    95
PF07714   ..............................................................................................................   327
PF00047   ..............................................................................................................    81
KDR-201   KFHYDNTAGISQYLQNSKRKSRPVSVKTFEDIPLEEPEVKVIPDDNQTDSGMVLASEELKTLEDRTKLSPSFGGMVPSKSRESVASEGSNQTSGYQSGYHSDDTDTTVYS  1320

logo
          SEEAELLKLIEIGVQTGSTAQILQPDSGTTLSSPPV
MOTESANIB ....................................    20
PF07679   ....................................   155
PF13927   ....................................    95
PF07714   ....................................   327
PF00047   ....................................    81
KDR-201   SEEAELLKLIEIGVQTGSTAQILQPDSGTTLSSPPV  1356
```

X non conserved

X similar

X ≥ 0% conserved

X ≥ 50% conserved

```
logo
            MQSKVLLAVALWLCVETRAASVGLPSVSLDLPRLSIQKDILTIKANTTLQITCRGQRDLDWLWPNNQSGSEQRVEVTECSDGLFCKTLTIPKVIGNDTGAYKCFYRETDL
NINTEDANIB  ..............................................................................................................     0
PF07679     ..............................................................................................................     0
PF13927     ..............................................................................................................     0
PF07714     ..............................................................................................................     0
PF00047     ..............................................................................................................     0
KDR-201     MQSKVLLAVALWLCVETRAASVGLPSVSLDLPRLSIQKDILTIKANTTLQITCRGQRDLDWLWPNNQSGSEQRVEVTECSDGLFCKTLTIPKVIGNDTGAYKCFYRETDL   110

logo
            ASVIYVYVQDYRSPFIASVSDQHGVVYITENKNKTVVIPCLGSISNLNVSLCARYPEKRFVPDGNRISWDSKKGFTIPSYMISYAGMVFCEAKINDESYQSIMYIVVVVG
NINTEDANIB  ..............................................................................................................     0
PF07679     ..............................................................................................................     0
PF13927     ..............................................................................................................     0
PF07714     ..............................................................................................................     0
PF00047     ..............................................................................................................     0
KDR-201     ASVIYVYVQDYRSPFIASVSDQHGVVYITENKNKTVVIPCLGSISNLNVSLCARYPEKRFVPDGNRISWDSKKGFTIPSYMISYAGMVFCEAKINDESYQSIMYIVVVVG   220

logo
            YRIYDVVLSPSHGIELSVGEKLVLNCTARTELNVGIDFNWEYPSSKHQHKKLVNRDLKTQSGSEMKKFLSTLTIDGVTRSDQGLYTCAASSGLMTKKNSTFVRVHEKPFV
NINTEDANIB  ..............................................................................................................     0
PF07679     ..............................................................................................................     0
PF13927     ..............................................................................................................     0
PF07714     ..............................................................................................................     0
PF00047     .........PSHGIELSVGEKLVLNCTARTELNVGIDFNWEYPSSKHQHKKLVNRDLKTQSGSEMKKFLSTLTIDGVTRSDQGLYTCAAS....................    81
KDR-201     YRIYDVVLSPSHGIELSVGEKLVLNCTARTELNVGIDFNWEYPSSKHQHKKLVNRDLKTQSGSEMKKFLSTLTIDGVTRSDQGLYTCAASSGLMTKKNSTFVRVHEKPFV   330

logo
            AFGSGMESLVEATVGERVRIPAKYLGYPPPEIKWYKNGIPLESNHTIKAGHVLTIMEVSERDTGNYTVILTNPISKEKQSHVVSLVVYVPPQIGEKSLISPVDSYQYGTT
NINTEDANIB  ..............................................................................................................     0
PF07679     .........VEATVGERVRIPAKYLGYPPPEIKWYKNGIPLESNHTIKAGHVLTIMEVSERDTGNYTVILTNPISKEKQS..............................    71
PF13927     ..............................................................................................................     0
PF07714     ..............................................................................................................     0
PF00047     ..............................................................................................................    81
KDR-201     AFGSGMESLVEATVGERVRIPAKYLGYPPPEIKWYKNGIPLESNHTIKAGHVLTIMEVSERDTGNYTVILTNPISKEKQSHVVSLVVYVPPQIGEKSLISPVDSYQYGTT   440

logo
            QTLTCTVYAIPPPHHIHWYWQLEEECANEPSQAVSVTNPYPCEEWRSVEDFQGGNKIEVNKNQFALIEGKNKTVSTLVIQAANVSALYKCEAVNKVGRGERVISFHVTRG
NINTEDANIB  ..............................................................................................................     0
PF07679     ..............................................................................................................    71
PF13927     ..............................................................................................................     0
PF07714     ..............................................................................................................     0
PF00047     ..............................................................................................................    81
KDR-201     QTLTCTVYAIPPPHHIHWYWQLEEECANEPSQAVSVTNPYPCEEWRSVEDFQGGNKIEVNKNQFALIEGKNKTVSTLVIQAANVSALYKCEAVNKVGRGERVISFHVTRG   550
```

```
logo        PEITLQPDMQPTEQESVSLWCTADRSTFENLTWYKLGPQPLPIHVGELPTPVCKNLDTLWKLNATMFSNSTNDILIMELKNASLQDQGDYVCLAQDRKTKKRHCVVRQLT
NINTEDANIB  ..............................................................................................................     0
PF07679     ..............................................................................................................    71
PF13927     PEITLQPDMQPTEQESVSLWCTADRSTFENLTWYKLGPQPLPIHVGELPTPVCKNLDTLWKLNATMFSNSTNDILIMELKNASLQDQGDYVCLAQ...............    95
PF07714     ..............................................................................................................     0
PF00047     ..............................................................................................................    81
KDR-201     PEITLQPDMQPTEQESVSLWCTADRSTFENLTWYKLGPQPLPIHVGELPTPVCKNLDTLWKLNATMFSNSTNDILIMELKNASLQDQGDYVCLAQDRKTKKRHCVVRQLT   660

logo        VLERVAPTITGNLENQTTSIGESIEVSCTASGNPPPQIMWFKDNETLVEDSGIVLKDGNRNLTIRRVRKEDEGLYTCQACSVLGCAKVEAFFIIEGAQEKTNLEIIILVG
NINTEDANIB  ..............................................................................................................     0
PF07679     ......PTITGNLENQTTSIGESIEVSCTASGNPPPQIMWFKDNETLVEDSGIVLKDGNRNLTIRRVRKEDEGLYTCQACSVLGCAKVEA....................   155
PF13927     ..............................................................................................................    95
PF07714     ..............................................................................................................     0
PF00047     ..............................................................................................................    81
KDR-201     VLERVAPTITGNLENQTTSIGESIEVSCTASGNPPPQIMWFKDNETLVEDSGIVLKDGNRNLTIRRVRKEDEGLYTCQACSVLGCAKVEAFFIIEGAQEKTNLEIIILVG   770

logo        TAVIAMFFWLLLVIILRTVKRANGGELKTGYLSIVMDPDELPLDEHCERLPYDASKWEFPRDRLKLGKPLGRGAFGQVIEADAFGIDKTATCRTVAVKMLKEGATHSEHR
NINTEDANIB  .....................................................................LG........E...............A.K............     5
PF07679     ..............................................................................................................   155
PF13927     ..............................................................................................................    95
PF07714     ...............................................................LKLGKPLGRGAFGQVIEADAFGIDKTATCRTVAVKMLKEGATHSEHR    47
PF00047     ..............................................................................................................    81
KDR-201     TAVIAMFFWLLLVIILRTVKRANGGELKTGYLSIVMDPDELPLDEHCERLPYDASKWEFPRDRLKLGKPLGRGAFGQVIEADAFGIDKTATCRTVAVKMLKEGATHSEHR   880

logo        ALMSELKILIHIGHHLNVVNLLGACTKPGGPLMVIVEFCKFGNLSTYLRSKRNEFVPYKTKGARFRQGKDYVGAIPVDLKRRLDSITSSQSSASSGFVEEKSLSDVEEEE
NINTEDANIB  ..................V................VEFCK.G....................................................................    12
PF07679     ..............................................................................................................   155
PF13927     ..............................................................................................................    95
PF07714     ALMSELKILIHIGHHLNVVNLLGACTKPGGPLMVIVEFCKFGNLSTYLRSKRNEFVPYKTKGARFRQGKDYVGAIPVDLKRRLDSITSSQSSASSGFVEEKSLSDVEEEE   157
PF00047     ..............................................................................................................    81
KDR-201     ALMSELKILIHIGHHLNVVNLLGACTKPGGPLMVIVEFCKFGNLSTYLRSKRNEFVPYKTKGARFRQGKDYVGAIPVDLKRRLDSITSSQSSASSGFVEEKSLSDVEEEE   990

logo        APEDLYKDFLTLEHLICYSFQVAKGMEFLASRKCIHRDLAARNILLSEKNVVKICDFGLARDIYKDPDYVRKGDARLPLKWMAPETIFDRVYTIQSDVWSFGVLLWEIFS
NINTEDANIB  ............................................L.........C.......................................................    14
PF07679     ..............................................................................................................   155
PF13927     ..............................................................................................................    95
PF07714     APEDLYKDFLTLEHLICYSFQVAKGMEFLASRKCIHRDLAARNILLSEKNVVKICDFGLARDIYKDPDYVRKGDARLPLKWMAPETIFDRVYTIQSDVWSFGVLLWEIFS   267
PF00047     ..............................................................................................................    81
KDR-201     APEDLYKDFLTLEHLICYSFQVAKGMEFLASRKCIHRDLAARNILLSEKNVVKICDFGLARDIYKDPDYVRKGDARLPLKWMAPETIFDRVYTIQSDVWSFGVLLWEIFS  1100
```

```
logo
            LGASPYPGVKIDEEFCRRLKEGTRMRAPDYTTPEMYQTMLDCWHGEPSQRPTFSELVEHLGNLLQANAQQDGKDYIVLPISETLSMEEDSGLSLPTSPVSCMEEEEVCDP
NINTEDANIB  ..............................................................................................................    14
PF07679     ..............................................................................................................   155
PF13927     ..............................................................................................................    95
PF07714     LGASPYPGVKIDEEFCRRLKEGTRMRAPDYTTPEMYQTMLDCWHGEPSQRPTFSELVEHL..................................................   327
PF00047     ..............................................................................................................    81
KDR-201     LGASPYPGVKIDEEFCRRLKEGTRMRAPDYTTPEMYQTMLDCWHGEPSQRPTFSELVEHLGNLLQANAQQDGKDYIVLPISETLSMEEDSGLSLPTSPVSCMEEEEVCDP  1210


logo
            KFHYDNTAGISQYLQNSKRKSRPVSVKTFEDIPLEEPEVKVIPDDNQTDSGMVLASEELKTLEDRTKLSPSFGGMVPSKSRESVASEGSNQTSGYQSGYHSDDTDTTVYS
NINTEDANIB  ..............................................................................................................    14
PF07679     ..............................................................................................................   155
PF13927     ..............................................................................................................    95
PF07714     ..............................................................................................................   327
PF00047     ..............................................................................................................    81
KDR-201     KFHYDNTAGISQYLQNSKRKSRPVSVKTFEDIPLEEPEVKVIPDDNQTDSGMVLASEELKTLEDRTKLSPSFGGMVPSKSRESVASEGSNQTSGYQSGYHSDDTDTTVYS  1320


logo
            SEEAELLKLIEIGVQTGSTAQILQPDSGTTLSSPPV
NINTEDANIB  ....................................    14
PF07679     ....................................   155
PF13927     ....................................    95
PF07714     ....................................   327
PF00047     ....................................    81
KDR-201     SEEAELLKLIEIGVQTGSTAQILQPDSGTTLSSPPV  1356
```

X non conserved
X similar
X ≥ 0% conserved
X ≥ 50% conserved

```
logo
           MQSKVLLAVALWLCVETRAASVGLPSVSLDLPRLSIQKDILTIKANTTLQITCRGQRDLDWLWPNNQSGSEQRVEVTECSDGLFCKTLTIPKVIGNDTGAYKCFYRETDL
SORAFENIB  ..............................................................................................................     0
PF07679    ..............................................................................................................     0
PF13927    ..............................................................................................................     0
PF07714    ..............................................................................................................     0
PF00047    ..............................................................................................................     0
KDR-201    MQSKVLLAVALWLCVETRAASVGLPSVSLDLPRLSIQKDILTIKANTTLQITCRGQRDLDWLWPNNQSGSEQRVEVTECSDGLFCKTLTIPKVIGNDTGAYKCFYRETDL   110

logo
           ASVIYVYVQDYRSPFIASVSDQHGVVYITENKNKTVVIPCLGSISNLNVSLCARYPEKRFVPDGNRISWDSKKGFTIPSYMISYAGMVFCEAKINDESYQSIMYIVVVVG
SORAFENIB  ..............................................................................................................     0
PF07679    ..............................................................................................................     0
PF13927    ..............................................................................................................     0
PF07714    ..............................................................................................................     0
PF00047    ..............................................................................................................     0
KDR-201    ASVIYVYVQDYRSPFIASVSDQHGVVYITENKNKTVVIPCLGSISNLNVSLCARYPEKRFVPDGNRISWDSKKGFTIPSYMISYAGMVFCEAKINDESYQSIMYIVVVVG   220

logo
           YRIYDVVLSPSHGIELSVGEKLVLNCTARTELNVGIDFNWEYPSSKHQHKKLVNRDLKTQSGSEMKKFLSTLTIDGVTRSDQGLYTCAASSGLMTKKNSTFVRVHEKPFV
SORAFENIB  ..............................................................................................................     0
PF07679    ..............................................................................................................     0
PF13927    ..............................................................................................................     0
PF07714    ..............................................................................................................     0
PF00047    .........PSHGIELSVGEKLVLNCTARTELNVGIDFNWEYPSSKHQHKKLVNRDLKTQSGSEMKKFLSTLTIDGVTRSDQGLYTCAAS....................    81
KDR-201    YRIYDVVLSPSHGIELSVGEKLVLNCTARTELNVGIDFNWEYPSSKHQHKKLVNRDLKTQSGSEMKKFLSTLTIDGVTRSDQGLYTCAASSGLMTKKNSTFVRVHEKPFV   330

logo
           AFGSGMESLVEATVGERVRIPAKYLGYPPPEIKWYKNGIPLESNHTIKAGHVLTIMEVSERDTGNYTVILTNPISKEKQSHVVSLVVYVPPQIGEKSLISPVDSYQYGTT
SORAFENIB  ..............................................................................................................     0
PF07679    .........VEATVGERVRIPAKYLGYPPPEIKWYKNGIPLESNHTIKAGHVLTIMEVSERDTGNYTVILTNPISKEKQS..............................    71
PF13927    ..............................................................................................................     0
PF07714    ..............................................................................................................     0
PF00047    ..............................................................................................................    81
KDR-201    AFGSGMESLVEATVGERVRIPAKYLGYPPPEIKWYKNGIPLESNHTIKAGHVLTIMEVSERDTGNYTVILTNPISKEKQSHVVSLVVYVPPQIGEKSLISPVDSYQYGTT   440

logo
           QTLTCTVYAIPPPHHIHWYWQLEEECANEPSQAVSVTNPYPCEEWRSVEDFQGGNKIEVNKNQFALIEGKNKTVSTLVIQAANVSALYKCEAVNKVGRGERVISFHVTRG
SORAFENIB  ..............................................................................................................     0
PF07679    ..............................................................................................................    71
PF13927    ..............................................................................................................     0
PF07714    ..............................................................................................................     0
PF00047    ..............................................................................................................    81
KDR-201    QTLTCTVYAIPPPHHIHWYWQLEEECANEPSQAVSVTNPYPCEEWRSVEDFQGGNKIEVNKNQFALIEGKNKTVSTLVIQAANVSALYKCEAVNKVGRGERVISFHVTRG   550
```

```
logo         PEITLQPDMQPTEQESVSLWCTADRSTFENLTWYKLGPQPLPIHVGELPTPVCKNLDTLWKLNATMFSNSTNDILIMELKNASLQDQGDYVCLAQDRKTKKRHCVVRQLT
SORAFENIB    ..............................................................................................................    0
PF07679      ..............................................................................................................   71
PF13927      PEITLQPDMQPTEQESVSLWCTADRSTFENLTWYKLGPQPLPIHVGELPTPVCKNLDTLWKLNATMFSNSTNDILIMELKNASLQDQGDYVCLAQ...............   95
PF07714      ..............................................................................................................    0
PF00047      ..............................................................................................................   81
KDR-201      PEITLQPDMQPTEQESVSLWCTADRSTFENLTWYKLGPQPLPIHVGELPTPVCKNLDTLWKLNATMFSNSTNDILIMELKNASLQDQGDYVCLAQDRKTKKRHCVVRQLT  660

logo         VLERVAPTITGNLENQTTSIGESIEVSCTASGNPPPQIMWFKDNETLVEDSGIVLKDGNRNLTIRRVRKEDEGLYTCQACSVLGCAKVEAFFIIEGAQEKTNLEIIILVG
SORAFENIB    ..............................................................................................................    0
PF07679      ......PTITGNLENQTTSIGESIEVSCTASGNPPPQIMWFKDNETLVEDSGIVLKDGNRNLTIRRVRKEDEGLYTCQACSVLGCAKVEA....................  155
PF13927      ..............................................................................................................   95
PF07714      ..............................................................................................................    0
PF00047      ..............................................................................................................   81
KDR-201      VLERVAPTITGNLENQTTSIGESIEVSCTASGNPPPQIMWFKDNETLVEDSGIVLKDGNRNLTIRRVRKEDEGLYTCQACSVLGCAKVEAFFIIEGAQEKTNLEIIILVG  770

logo         TAVIAMFFWLLLVIILRTVKRANGGELKTGYLSIVMDPDELPLDEHCERLPYDASKWEFPRDRLKLGKPLGRGAFGQVIEADAFGIDKTATCRTVAVKMLKEGATHSEHR
SORAFENIB    .....................................................................L.......V.................A..............    3
PF07679      ..............................................................................................................  155
PF13927      ..............................................................................................................   95
PF07714      ...............................................................LKLGKPLGRGAFGQVIEADAFGIDKTATCRTVAVKMLKEGATHSEHR   47
PF00047      ..............................................................................................................   81
KDR-201      TAVIAMFFWLLLVIILRTVKRANGGELKTGYLSIVMDPDELPLDEHCERLPYDASKWEFPRDRLKLGKPLGRGAFGQVIEADAFGIDKTATCRTVAVKMLKEGATHSEHR  880

logo         ALMSELKILIHIGHHLNVVNLLGACTKPGGPLMVIVEFCKFGNLSTYLRSKRNEFVPYKTKGARFRQGKDYVGAIPVDLKRRLDSITSSQSSASSGFVEEKSLSDVEEEE
SORAFENIB    ....E...L..I.....VV................VEFCK.G....................................................................   14
PF07679      ..............................................................................................................  155
PF13927      ..............................................................................................................   95
PF07714      ALMSELKILIHIGHHLNVVNLLGACTKPGGPLMVIVEFCKFGNLSTYLRSKRNEFVPYKTKGARFRQGKDYVGAIPVDLKRRLDSITSSQSSASSGFVEEKSLSDVEEEE  157
PF00047      ..............................................................................................................   81
KDR-201      ALMSELKILIHIGHHLNVVNLLGACTKPGGPLMVIVEFCKFGNLSTYLRSKRNEFVPYKTKGARFRQGKDYVGAIPVDLKRRLDSITSSQSSASSGFVEEKSLSDVEEEE  990

logo         APEDLYKDFLTLEHLICYSFQVAKGMEFLASRKCIHRDLAARNILLSEKNVVKICDFGLARDIYKDPDYVRKGDARLPLKWMAPETIFDRVYTIQSDVWSFGVLLWEIFS
SORAFENIB    ...................................H........L........ICDF.....................................................   20
PF07679      ..............................................................................................................  155
PF13927      ..............................................................................................................   95
PF07714      APEDLYKDFLTLEHLICYSFQVAKGMEFLASRKCIHRDLAARNILLSEKNVVKICDFGLARDIYKDPDYVRKGDARLPLKWMAPETIFDRVYTIQSDVWSFGVLLWEIFS  267
PF00047      ..............................................................................................................   81
KDR-201      APEDLYKDFLTLEHLICYSFQVAKGMEFLASRKCIHRDLAARNILLSEKNVVKICDFGLARDIYKDPDYVRKGDARLPLKWMAPETIFDRVYTIQSDVWSFGVLLWEIFS 1100
```

```
logo
            LGASPYPGVKIDEEFCRRLKEGTRMRAPDYTTPEMYQTMLDCWHGEPSQRPTFSELVEHLGNLLQANAQQDGKDYIVLPISETLSMEEDSGLSLPTSPVSCMEEEEVCDP
SORAFENIB   ..............................................................................................................    20
PF07679     ..............................................................................................................   155
PF13927     ..............................................................................................................    95
PF07714     LGASPYPGVKIDEEFCRRLKEGTRMRAPDYTTPEMYQTMLDCWHGEPSQRPTFSELVEHL..................................................   327
PF00047     ..............................................................................................................    81
KDR-201     LGASPYPGVKIDEEFCRRLKEGTRMRAPDYTTPEMYQTMLDCWHGEPSQRPTFSELVEHLGNLLQANAQQDGKDYIVLPISETLSMEEDSGLSLPTSPVSCMEEEEVCDP  1210

logo
            KFHYDNTAGISQYLQNSKRKSRPVSVKTFEDIPLEEPEVKVIPDDNQTDSGMVLASEELKTLEDRTKLSPSFGGMVPSKSRESVASEGSNQTSGYQSGYHSDDTDTTVYS
SORAFENIB   ..............................................................................................................    20
PF07679     ..............................................................................................................   155
PF13927     ..............................................................................................................    95
PF07714     ..............................................................................................................   327
PF00047     ..............................................................................................................    81
KDR-201     KFHYDNTAGISQYLQNSKRKSRPVSVKTFEDIPLEEPEVKVIPDDNQTDSGMVLASEELKTLEDRTKLSPSFGGMVPSKSRESVASEGSNQTSGYQSGYHSDDTDTTVYS  1320

logo
            SEEAELLKLIEIGVQTGSTAQILQPDSGTTLSSPPV
SORAFENIB   ....................................    20
PF07679     ....................................   155
PF13927     ....................................    95
PF07714     ....................................   327
PF00047     ....................................    81
KDR-201     SEEAELLKLIEIGVQTGSTAQILQPDSGTTLSSPPV  1356

            X  non conserved
            X  similar
            X  ≥ 0% conserved
            X  ≥ 50% conserved
```

```
logo
               MQSKVLLAVALWLCVETRAASVGLPSVSLDLPRLSIQKDILTIKANTTLQITCRGQRDLDWLWPNNQSGSEQRVEVTECSDGLFCKTLTIPKVIGNDTGAYKCFYRETDL
SUNITINIB      ..............................................................................................................      0
PF07679        ..............................................................................................................      0
PF13927        ..............................................................................................................      0
PF07714        ..............................................................................................................      0
PF00047        ..............................................................................................................      0
KDR-201        MQSKVLLAVALWLCVETRAASVGLPSVSLDLPRLSIQKDILTIKANTTLQITCRGQRDLDWLWPNNQSGSEQRVEVTECSDGLFCKTLTIPKVIGNDTGAYKCFYRETDL    110

logo
               ASVIYVYVQDYRSPFIASVSDQHGVVYITENKNKTVVIPCLGSISNLNVSLCARYPEKRFVPDGNRISWDSKKGFTIPSYMISYAGMVFCEAKINDESYQSIMYIVVVVG
SUNITINIB      ..............................................................................................................      0
PF07679        ..............................................................................................................      0
PF13927        ..............................................................................................................      0
PF07714        ..............................................................................................................      0
PF00047        ..............................................................................................................      0
KDR-201        ASVIYVYVQDYRSPFIASVSDQHGVVYITENKNKTVVIPCLGSISNLNVSLCARYPEKRFVPDGNRISWDSKKGFTIPSYMISYAGMVFCEAKINDESYQSIMYIVVVVG    220

logo
               YRIYDVVLSPSHGIELSVGEKLVLNCTARTELNVGIDFNWEYPSSKHQHKKLVNRDLKTQSGSEMKKFLSTLTIDGVTRSDQGLYTCAASSGLMTKKNSTFVRVHEKPFV
SUNITINIB      ..............................................................................................................      0
PF07679        ..............................................................................................................      0
PF13927        ..............................................................................................................      0
PF07714        ..............................................................................................................      0
PF00047        .........PSHGIELSVGEKLVLNCTARTELNVGIDFNWEYPSSKHQHKKLVNRDLKTQSGSEMKKFLSTLTIDGVTRSDQGLYTCAAS....................     81
KDR-201        YRIYDVVLSPSHGIELSVGEKLVLNCTARTELNVGIDFNWEYPSSKHQHKKLVNRDLKTQSGSEMKKFLSTLTIDGVTRSDQGLYTCAASSGLMTKKNSTFVRVHEKPFV    330

logo
               AFGSGMESLVEATVGERVRIPAKYLGYPPPEIKWYKNGIPLESNHTIKAGHVLTIMEVSERDTGNYTVILTNPISKEKQSHVVSLVVYVPPQIGEKSLISPVDSYQYGTT
SUNITINIB      ..............................................................................................................      0
PF07679        .........VEATVGERVRIPAKYLGYPPPEIKWYKNGIPLESNHTIKAGHVLTIMEVSERDTGNYTVILTNPISKEKQS..............................     71
PF13927        ..............................................................................................................      0
PF07714        ..............................................................................................................      0
PF00047        ..............................................................................................................     81
KDR-201        AFGSGMESLVEATVGERVRIPAKYLGYPPPEIKWYKNGIPLESNHTIKAGHVLTIMEVSERDTGNYTVILTNPISKEKQSHVVSLVVYVPPQIGEKSLISPVDSYQYGTT    440

logo
               QTLTCTVYAIPPPHHIHWYWQLEEECANEPSQAVSVTNPYPCEEWRSVEDFQGGNKIEVNKNQFALIEGKNKTVSTLVIQAANVSALYKCEAVNKVGRGERVISFHVTRG
SUNITINIB      ..............................................................................................................      0
PF07679        ..............................................................................................................     71
PF13927        ..............................................................................................................      0
PF07714        ..............................................................................................................      0
PF00047        ..............................................................................................................     81
KDR-201        QTLTCTVYAIPPPHHIHWYWQLEEECANEPSQAVSVTNPYPCEEWRSVEDFQGGNKIEVNKNQFALIEGKNKTVSTLVIQAANVSALYKCEAVNKVGRGERVISFHVTRG    550
```

```
logo
           PEITLQPDMQPTEQESVSLWCTADRSTFENLTWYKLGPQPLPIHVGELPTPVCKNLDTLWKLNATMFSNSTNDILIMELKNASLQDQGDYVCLAQDRKTKKRHCVVRQLT
SUNITINIB  ..............................................................................................................     0
PF07679    ..............................................................................................................    71
PF13927    PEITLQPDMQPTEQESVSLWCTADRSTFENLTWYKLGPQPLPIHVGELPTPVCKNLDTLWKLNATMFSNSTNDILIMELKNASLQDQGDYVCLAQ...............    95
PF07714    ..............................................................................................................     0
PF00047    ..............................................................................................................    81
KDR-201    PEITLQPDMQPTEQESVSLWCTADRSTFENLTWYKLGPQPLPIHVGELPTPVCKNLDTLWKLNATMFSNSTNDILIMELKNASLQDQGDYVCLAQDRKTKKRHCVVRQLT   660

logo
           VLERVAPTITGNLENQTTSIGESIEVSCTASGNPPPQIMWFKDNETLVEDSGIVLKDGNRNLTIRRVRKEDEGLYTCQACSVLGCAKVEAFFIIEGAQEKTNLEIIILVG
SUNITINIB  ..............................................................................................................     0
PF07679    ......PTITGNLENQTTSIGESIEVSCTASGNPPPQIMWFKDNETLVEDSGIVLKDGNRNLTIRRVRKEDEGLYTCQACSVLGCAKVEA....................   155
PF13927    ..............................................................................................................    95
PF07714    ..............................................................................................................     0
PF00047    ..............................................................................................................    81
KDR-201    VLERVAPTITGNLENQTTSIGESIEVSCTASGNPPPQIMWFKDNETLVEDSGIVLKDGNRNLTIRRVRKEDEGLYTCQACSVLGCAKVEAFFIIEGAQEKTNLEIIILVG   770

logo
           TAVIAMFFWLLLVIILRTVKRANGGELKTGYLSIVMDPDELPLDEHCERLPYDASKWEFPRDRLKLGKPLGRGAFGQVIEADAFGIDKTATCRTVAVKMLKEGATHSEHR
SUNITINIB  .....................................................................L.........................A.K............     3
PF07679    ..............................................................................................................   155
PF13927    ..............................................................................................................    95
PF07714    ...............................................................LKLGKPLGRGAFGQVIEADAFGIDKTATCRTVAVKMLKEGATHSEHR    47
PF00047    ..............................................................................................................    81
KDR-201    TAVIAMFFWLLLVIILRTVKRANGGELKTGYLSIVMDPDELPLDEHCERLPYDASKWEFPRDRLKLGKPLGRGAFGQVIEADAFGIDKTATCRTVAVKMLKEGATHSEHR   880

logo
           ALMSELKILIHIGHHLNVVNLLGACTKPGGPLMVIVEFCKFGNLSTYLRSKRNEFVPYKTKGARFRQGKDYVGAIPVDLKRRLDSITSSQSSASSGFVEEKSLSDVEEEE
SUNITINIB  ..................V................VEFCK.G....................................................................    10
PF07679    ..............................................................................................................   155
PF13927    ..............................................................................................................    95
PF07714    ALMSELKILIHIGHHLNVVNLLGACTKPGGPLMVIVEFCKFGNLSTYLRSKRNEFVPYKTKGARFRQGKDYVGAIPVDLKRRLDSITSSQSSASSGFVEEKSLSDVEEEE   157
PF00047    ..............................................................................................................    81
KDR-201    ALMSELKILIHIGHHLNVVNLLGACTKPGGPLMVIVEFCKFGNLSTYLRSKRNEFVPYKTKGARFRQGKDYVGAIPVDLKRRLDSITSSQSSASSGFVEEKSLSDVEEEE   990

logo
           APEDLYKDFLTLEHLICYSFQVAKGMEFLASRKCIHRDLAARNILLSEKNVVKICDFGLARDIYKDPDYVRKGDARLPLKWMAPETIFDRVYTIQSDVWSFGVLLWEIFS
SUNITINIB  ............................................L..........DF.....................................................    13
PF07679    ..............................................................................................................   155
PF13927    ..............................................................................................................    95
PF07714    APEDLYKDFLTLEHLICYSFQVAKGMEFLASRKCIHRDLAARNILLSEKNVVKICDFGLARDIYKDPDYVRKGDARLPLKWMAPETIFDRVYTIQSDVWSFGVLLWEIFS   267
PF00047    ..............................................................................................................    81
KDR-201    APEDLYKDFLTLEHLICYSFQVAKGMEFLASRKCIHRDLAARNILLSEKNVVKICDFGLARDIYKDPDYVRKGDARLPLKWMAPETIFDRVYTIQSDVWSFGVLLWEIFS  1100
```

```
logo
             LGASPYPGVKIDEEFCRRLKEGTRMRAPDYTTPEMYQTMLDCWHGEPSQRPTFSELVEHLGNLLQANAQQDGKDYIVLPISETLSMEEDSGLSLPTSPVSCMEEEEVCDP
SUNITINIB    ..............................................................................................................    13
PF07679      ..............................................................................................................   155
PF13927      ..............................................................................................................    95
PF07714      LGASPYPGVKIDEEFCRRLKEGTRMRAPDYTTPEMYQTMLDCWHGEPSQRPTFSELVEHL..................................................   327
PF00047      ..............................................................................................................    81
KDR-201      LGASPYPGVKIDEEFCRRLKEGTRMRAPDYTTPEMYQTMLDCWHGEPSQRPTFSELVEHLGNLLQANAQQDGKDYIVLPISETLSMEEDSGLSLPTSPVSCMEEEEVCDP  1210

logo
             KFHYDNTAGISQYLQNSKRKSRPVSVKTFEDIPLEEPEVKVIPDDNQTDSGMVLASEELKTLEDRTKLSPSFGGMVPSKSRESVASEGSNQTSGYQSGYHSDDTDTTVYS
SUNITINIB    ..............................................................................................................    13
PF07679      ..............................................................................................................   155
PF13927      ..............................................................................................................    95
PF07714      ..............................................................................................................   327
PF00047      ..............................................................................................................    81
KDR-201      KFHYDNTAGISQYLQNSKRKSRPVSVKTFEDIPLEEPEVKVIPDDNQTDSGMVLASEELKTLEDRTKLSPSFGGMVPSKSRESVASEGSNQTSGYQSGYHSDDTDTTVYS  1320

logo
             SEEAELLKLIEIGVQTGSTAQILQPDSGTTLSSPPV
SUNITINIB    ....................................    13
PF07679      ....................................   155
PF13927      ....................................    95
PF07714      ....................................   327
PF00047      ....................................    81
KDR-201      SEEAELLKLIEIGVQTGSTAQILQPDSGTTLSSPPV  1356
```

X non conserved

X similar

X $\geq 0\%$ conserved

X $\geq 50\%$ conserved

```
logo

           MQSKVLLAVALWLCVETRAASVGLPSVSLDLPRLSIQKDILTIKANTTLQITCRGQRDLDWLWPNNQSGSEQRVEVTECSDGLFCKTLTIPKVIGNDTGAYKCFYRETDL
TIVOZANIB  ..............................................................................................................    0
PF07679    ..............................................................................................................    0
PF13927    ..............................................................................................................    0
PF07714    ..............................................................................................................    0
PF00047    ..............................................................................................................    0
KDR-201    MQSKVLLAVALWLCVETRAASVGLPSVSLDLPRLSIQKDILTIKANTTLQITCRGQRDLDWLWPNNQSGSEQRVEVTECSDGLFCKTLTIPKVIGNDTGAYKCFYRETDL  110


logo

           ASVIYVYVQDYRSPFIASVSDQHGVVYITENKNKTVVIPCLGSISNLNVSLCARYPEKRFVPDGNRISWDSKKGFTIPSYMISYAGMVFCEAKINDESYQSIMYIVVVVG
TIVOZANIB  ..............................................................................................................    0
PF07679    ..............................................................................................................    0
PF13927    ..............................................................................................................    0
PF07714    ..............................................................................................................    0
PF00047    ..............................................................................................................    0
KDR-201    ASVIYVYVQDYRSPFIASVSDQHGVVYITENKNKTVVIPCLGSISNLNVSLCARYPEKRFVPDGNRISWDSKKGFTIPSYMISYAGMVFCEAKINDESYQSIMYIVVVVG  220


logo

           YRIYDVVLSPSHGIELSVGEKLVLNCTARTELNVGIDFNWEYPSSKHQHKKLVNRDLKTQSGSEMKKFLSTLTIDGVTRSDQGLYTCAASSGLMTKKNSTFVRVHEKPFV
TIVOZANIB  ..............................................................................................................    0
PF07679    ..............................................................................................................    0
PF13927    ..............................................................................................................    0
PF07714    ..............................................................................................................    0
PF00047    .........PSHGIELSVGEKLVLNCTARTELNVGIDFNWEYPSSKHQHKKLVNRDLKTQSGSEMKKFLSTLTIDGVTRSDQGLYTCAAS....................   81
KDR-201    YRIYDVVLSPSHGIELSVGEKLVLNCTARTELNVGIDFNWEYPSSKHQHKKLVNRDLKTQSGSEMKKFLSTLTIDGVTRSDQGLYTCAASSGLMTKKNSTFVRVHEKPFV  330


logo

           AFGSGMESLVEATVGERVRIPAKYLGYPPPEIKWYKNGIPLESNHTIKAGHVLTIMEVSERDTGNYTVILTNPISKEKQSHVVSLVVYVPPQIGEKSLISPVDSYQYGTT
TIVOZANIB  ..............................................................................................................    0
PF07679    .........VEATVGERVRIPAKYLGYPPPEIKWYKNGIPLESNHTIKAGHVLTIMEVSERDTGNYTVILTNPISKEKQS..............................   71
PF13927    ..............................................................................................................    0
PF07714    ..............................................................................................................    0
PF00047    ..............................................................................................................   81
KDR-201    AFGSGMESLVEATVGERVRIPAKYLGYPPPEIKWYKNGIPLESNHTIKAGHVLTIMEVSERDTGNYTVILTNPISKEKQSHVVSLVVYVPPQIGEKSLISPVDSYQYGTT  440


logo

           QTLTCTVYAIPPPHHIHWYWQLEEECANEPSQAVSVTNPYPCEEWRSVEDFQGGNKIEVNKNQFALIEGKNKTVSTLVIQAANVSALYKCEAVNKVGRGERVISFHVTRG
TIVOZANIB  ..............................................................................................................    0
PF07679    ..............................................................................................................   71
PF13927    ..............................................................................................................    0
PF07714    ..............................................................................................................    0
PF00047    ..............................................................................................................   81
KDR-201    QTLTCTVYAIPPPHHIHWYWQLEEECANEPSQAVSVTNPYPCEEWRSVEDFQGGNKIEVNKNQFALIEGKNKTVSTLVIQAANVSALYKCEAVNKVGRGERVISFHVTRG  550
```

```
logo
             PEITLQPDMQPTEQESVSLWCTADRSTFENLTWYKLGPQPLPIHVGELPTPVCKNLDTLWKLNATMFSNSTNDILIMELKNASLQDQGDYVCLAQDRKTKKRHCVVRQLT
TIVOZANIB    ..............................................................................................................    0
PF07679      ..............................................................................................................   71
PF13927      PEITLQPDMQPTEQESVSLWCTADRSTFENLTWYKLGPQPLPIHVGELPTPVCKNLDTLWKLNATMFSNSTNDILIMELKNASLQDQGDYVCLAQ...............   95
PF07714      ..............................................................................................................    0
PF00047      ..............................................................................................................   81
KDR-201      PEITLQPDMQPTEQESVSLWCTADRSTFENLTWYKLGPQPLPIHVGELPTPVCKNLDTLWKLNATMFSNSTNDILIMELKNASLQDQGDYVCLAQDRKTKKRHCVVRQLT  660

logo
             VLERVAPTITGNLENQTTSIGESIEVSCTASGNPPPQIMWFKDNETLVEDSGIVLKDGNRNLTIRRVRKEDEGLYTCQACSVLGCAKVEAFFIIEGAQEKTNLEIIILVG
TIVOZANIB    ..............................................................................................................    0
PF07679      ......PTITGNLENQTTSIGESIEVSCTASGNPPPQIMWFKDNETLVEDSGIVLKDGNRNLTIRRVRKEDEGLYTCQACSVLGCAKVEA....................  155
PF13927      ..............................................................................................................   95
PF07714      ..............................................................................................................    0
PF00047      ..............................................................................................................   81
KDR-201      VLERVAPTITGNLENQTTSIGESIEVSCTASGNPPPQIMWFKDNETLVEDSGIVLKDGNRNLTIRRVRKEDEGLYTCQACSVLGCAKVEAFFIIEGAQEKTNLEIIILVG  770

logo
             TAVIAMFFWLLLVIILRTVKRANGGELKTGYLSIVMDPDELPLDEHCERLPYDASKWEFPRDRLKLGKPLGRGAFGQVIEADAFGIDKTATCRTVAVKMLKEGATHSEHR
TIVOZANIB    .....................................................................L.......V.................A.K............    4
PF07679      ..............................................................................................................  155
PF13927      ..............................................................................................................   95
PF07714      ...............................................................LKLGKPLGRGAFGQVIEADAFGIDKTATCRTVAVKMLKEGATHSEHR   47
PF00047      ..............................................................................................................   81
KDR-201      TAVIAMFFWLLLVIILRTVKRANGGELKTGYLSIVMDPDELPLDEHCERLPYDASKWEFPRDRLKLGKPLGRGAFGQVIEADAFGIDKTATCRTVAVKMLKEGATHSEHR  880

logo
             ALMSELKILIHIGHHLNVVNLLGACTKPGGPLMVIVEFCKFGNLSTYLRSKRNEFVPYKTKGARFRQGKDYVGAIPVDLKRRLDSITSSQSSASSGFVEEKSLSDVEEEE
TIVOZANIB    ....E...L.........V................VEFCK......................................................................   12
PF07679      ..............................................................................................................  155
PF13927      ..............................................................................................................   95
PF07714      ALMSELKILIHIGHHLNVVNLLGACTKPGGPLMVIVEFCKFGNLSTYLRSKRNEFVPYKTKGARFRQGKDYVGAIPVDLKRRLDSITSSQSSASSGFVEEKSLSDVEEEE  157
PF00047      ..............................................................................................................   81
KDR-201      ALMSELKILIHIGHHLNVVNLLGACTKPGGPLMVIVEFCKFGNLSTYLRSKRNEFVPYKTKGARFRQGKDYVGAIPVDLKRRLDSITSSQSSASSGFVEEKSLSDVEEEE  990

logo
             APEDLYKDFLTLEHLICYSFQVAKGMEFLASRKCIHRDLAARNILLSEKNVVKICDFGLARDIYKDPDYVRKGDARLPLKWMAPETIFDRVYTIQSDVWSFGVLLWEIFS
TIVOZANIB    ............................................L.........CDF.....................................................   16
PF07679      ..............................................................................................................  155
PF13927      ..............................................................................................................   95
PF07714      APEDLYKDFLTLEHLICYSFQVAKGMEFLASRKCIHRDLAARNILLSEKNVVKICDFGLARDIYKDPDYVRKGDARLPLKWMAPETIFDRVYTIQSDVWSFGVLLWEIFS  267
PF00047      ..............................................................................................................   81
KDR-201      APEDLYKDFLTLEHLICYSFQVAKGMEFLASRKCIHRDLAARNILLSEKNVVKICDFGLARDIYKDPDYVRKGDARLPLKWMAPETIFDRVYTIQSDVWSFGVLLWEIFS 1100
```

```
logo
            LGASPYPGVKIDEEFCRRLKEGTRMRAPDYTTPEMYQTMLDCWHGEPSQRPTFSELVEHLGNLLQANAQQDGKDYIVLPISETLSMEEDSGLSLPTSPVSCMEEEEVCDP
TIVOZANIB   ..............................................................................................................    16
PF07679     ..............................................................................................................   155
PF13927     ..............................................................................................................    95
PF07714     LGASPYPGVKIDEEFCRRLKEGTRMRAPDYTTPEMYQTMLDCWHGEPSQRPTFSELVEHL..................................................   327
PF00047     ..............................................................................................................    81
KDR-201     LGASPYPGVKIDEEFCRRLKEGTRMRAPDYTTPEMYQTMLDCWHGEPSQRPTFSELVEHLGNLLQANAQQDGKDYIVLPISETLSMEEDSGLSLPTSPVSCMEEEEVCDP  1210

logo
            KFHYDNTAGISQYLQNSKRKSRPVSVKTFEDIPLEEPEVKVIPDDNQTDSGMVLASEELKTLEDRTKLSPSFGGMVPSKSRESVASEGSNQTSGYQSGYHSDDTDTTVYS
TIVOZANIB   ..............................................................................................................    16
PF07679     ..............................................................................................................   155
PF13927     ..............................................................................................................    95
PF07714     ..............................................................................................................   327
PF00047     ..............................................................................................................    81
KDR-201     KFHYDNTAGISQYLQNSKRKSRPVSVKTFEDIPLEEPEVKVIPDDNQTDSGMVLASEELKTLEDRTKLSPSFGGMVPSKSRESVASEGSNQTSGYQSGYHSDDTDTTVYS  1320

logo
            SEEAELLKLIEIGVQTGSTAQILQPDSGTTLSSPPV
TIVOZANIB   ....................................    16
PF07679     ....................................   155
PF13927     ....................................    95
PF07714     ....................................   327
PF00047     ....................................    81
KDR-201     SEEAELLKLIEIGVQTGSTAQILQPDSGTTLSSPPV  1356
```

X non conserved
X similar
X $\geq$ 0% conserved
X $\geq$ 50% conserved

```
logo      MRGARGAWDFLCVLLLLLRVQTGSSQPSVSPGEPSPPSIHPGKSDLIVRVGDEIRLLCTDPGFVKWTFEILDETNENKQNEWITEKAEATNTGKYTCTNKHGLSNSIYVFVRDPA
IMATINIB  ...................................................................................................................   0
PF07714   ...................................................................................................................   0
PF00047   ...................................................................................................................   0
KIT-201   MRGARGAWDFLCVLLLLLRVQTGSSQPSVSPGEPSPPSIHPGKSDLIVRVGDEIRLLCTDPGFVKWTFEILDETNENKQNEWITEKAEATNTGKYTCTNKHGLSNSIYVFVRDPA 115
KIT-202   MRGARGAWDFLCVLLLLLRVQTGSSQPSVSPGEPSPPSIHPGKSDLIVRVGDEIRLLCTDPGFVKWTFEILDETNENKQNEWITEKAEATNTGKYTCTNKHGLSNSIYVFVRDPA 115

logo      KLFLVDRSLYGKEDNDTLVRCPLTDPEVTNYSLKGCQGKPLPKDLRFIPDPKAGIMIKSVKRAYHRLCLHCSVDQEGKSVLSEKFILKVRPAFKAVPVVSVSKASYLLREGEEFT
IMATINIB  ...................................................................................................................   0
PF07714   ...................................................................................................................   0
PF00047   .....................................................................................................SKASYLLREGEEFT  14
KIT-201   KLFLVDRSLYGKEDNDTLVRCPLTDPEVTNYSLKGCQGKPLPKDLRFIPDPKAGIMIKSVKRAYHRLCLHCSVDQEGKSVLSEKFILKVRPAFKAVPVVSVSKASYLLREGEEFT 230
KIT-202   KLFLVDRSLYGKEDNDTLVRCPLTDPEVTNYSLKGCQGKPLPKDLRFIPDPKAGIMIKSVKRAYHRLCLHCSVDQEGKSVLSEKFILKVRPAFKAVPVVSVSKASYLLREGEEFT 230

logo      VTCTIKDVSSSVYSTWKRENSQTKLQEKYNSWHHGDFNYERQATLTISSARVNDSGVFMCYANNTFGSANVTTTLEVVDKGFINIFPMINTTVFVNDGENVDLIVEYEAFPKPEH
IMATINIB  ...................................................................................................................   0
PF07714   ...................................................................................................................   0
PF00047   VTCTIKDVSSSVYSTWKRENSQTKLQEKYNSWHHGDFNYERQATLTISSARVNDSGVFMCYANNTFGSANVTTT.........................................  88
KIT-201   VTCTIKDVSSSVYSTWKRENSQTKLQEKYNSWHHGDFNYERQATLTISSARVNDSGVFMCYANNTFGSANVTTTLEVVDKGFINIFPMINTTVFVNDGENVDLIVEYEAFPKPEH 345
KIT-202   VTCTIKDVSSSVYSTWKRENSQTKLQEKYNSWHHGDFNYERQATLTISSARVNDSGVFMCYANNTFGSANVTTTLEVVDKGFINIFPMINTTVFVNDGENVDLIVEYEAFPKPEH 345

logo      QQWIYMNRTFTDKWEDYPKSENESNIRYVSELHLTRLKGTEGGTYTFLVSNSDVNAAIAFNVYVNTKPEILTYDRLVNGMLQCVAAGFPEPTIDWYFCPGTEQRCSASVLPVDVQ
IMATINIB  ...................................................................................................................   0
PF07714   ...................................................................................................................   0
PF00047   ...................................................................................................................  88
KIT-201   QQWIYMNRTFTDKWEDYPKSENESNIRYVSELHLTRLKGTEGGTYTFLVSNSDVNAAIAFNVYVNTKPEILTYDRLVNGMLQCVAAGFPEPTIDWYFCPGTEQRCSASVLPVDVQ 460
KIT-202   QQWIYMNRTFTDKWEDYPKSENESNIRYVSELHLTRLKGTEGGTYTFLVSNSDVNAAIAFNVYVNTKPEILTYDRLVNGMLQCVAAGFPEPTIDWYFCPGTEQRCSASVLPVDVQ 460

logo      TLNSSGPPFGKLVVQSSIDSSAFKHNGTVECKAYNDVGKTSAYFNFAFKGNNKEQIHPHTLFTPLLIGFVIVAGMMCIIVMILTYKYLQKPMYEVQWKVVEEINGNNYVYIDPTQ
IMATINIB  ...................................................................................................................   0
PF07714   ...................................................................................................................   0
PF00047   ...................................................................................................................  88
KIT-201   TLNSSGPPFGKLVVQSSIDSSAFKHNGTVECKAYNDVGKTSAYFNFAFKGNNKEQIHPHTLFTPLLIGFVIVAGMMCIIVMILTYKYLQKPMYEVQWKVVEEINGNNYVYIDPTQ 575
KIT-202   TLNSSGPPFGKLVVQSSIDSSAFKHNGTVECKAYNDVGKTSAYFNFAFK....EQIHPHTLFTPLLIGFVIVAGMMCIIVMILTYKYLQKPMYEVQWKVVEEINGNNYVYIDPTQ 571

logo      LPYDHKWEFPRNRLSFGKTLGAGAFGKVVEATAYGLIKSDAAMTVAVKMLKPSAHLTEREALMSELKVLSYLGNHMNIVNLLGACTIGGPTLVITEYCCYGDLLNFLRRKRDSFI
IMATINIB  ...................L.......V.................AVK................E..VL.......................V.T.YC..G..............  13
PF07714   .............LSFGKTLGAGAFGKVVEATAYGLIKSDAAMTVAVKMLKPSAHLTEREALMSELKVLSYLGNHMNIVNLLGACTIGGPTLVITEYCCYGDLLNFLRRKRDSFI 102
PF00047   ...................................................................................................................  88
KIT-201   LPYDHKWEFPRNRLSFGKTLGAGAFGKVVEATAYGLIKSDAAMTVAVKMLKPSAHLTEREALMSELKVLSYLGNHMNIVNLLGACTIGGPTLVITEYCCYGDLLNFLRRKRDSFI 690
KIT-202   LPYDHKWEFPRNRLSFGKTLGAGAFGKVVEATAYGLIKSDAAMTVAVKMLKPSAHLTEREALMSELKVLSYLGNHMNIVNLLGACTIGGPTLVITEYCCYGDLLNFLRRKRDSFI 686
```

```
logo      CSKQEDHAEAALYKNLLHSKESSCSDSTNEYMDMKPGVSYVVPTKADKRRSVRIGSYIERDVTPAIMEDDELALDLEDLLSFSYQVAKGMAFLASKNCIHRDLAARNILLTHGRI
IMATINIB  ..................................................................................................IHR.......L......   17
PF07714   CSKQEDHAEAALYKNLLHSKESSCSDSTNEYMDMKPGVSYVVPTKADKRRSVRIGSYIERDVTPAIMEDDELALDLEDLLSFSYQVAKGMAFLASKNCIHRDLAARNILLTHGRI  217
PF00047   ...................................................................................................................   88
KIT-201   CSKQEDHAEAALYKNLLHSKESSCSDSTNEYMDMKPGVSYVVPTKADKRRSVRIGSYIERDVTPAIMEDDELALDLEDLLSFSYQVAKGMAFLASKNCIHRDLAARNILLTHGRI  805
KIT-202   CSKQEDHAEAALYKNLLHSKESSCSDSTNEYMDMKPGVSYVVPTKADKRRSVRIGSYIERDVTPAIMEDDELALDLEDLLSFSYQVAKGMAFLASKNCIHRDLAARNILLTHGRI  801

logo      TKICDFGLARDIKNDSNYVVKGNARLPVKWMAPESIFNCVYTFESDVWSYGIFLWELFSLGSSPYPGMPVDSKFYKMIKEGFRMLSPEHAPAEMYDIMKTCWDADPLKRPTFKQI
IMATINIB  ...CDF.............................................................................................................   20
PF07714   TKICDFGLARDIKNDSNYVVKGNARLPVKWMAPESIFNCVYTFESDVWSYGIFLWELFSLGSSPYPGMPVDSKFYKMIKEGFRMLSPEHAPAEMYDIMKTCWDADPLKRPTFKQI  332
PF00047   ...................................................................................................................   88
KIT-201   TKICDFGLARDIKNDSNYVVKGNARLPVKWMAPESIFNCVYTFESDVWSYGIFLWELFSLGSSPYPGMPVDSKFYKMIKEGFRMLSPEHAPAEMYDIMKTCWDADPLKRPTFKQI  920
KIT-202   TKICDFGLARDIKNDSNYVVKGNARLPVKWMAPESIFNCVYTFESDVWSYGIFLWELFSLGSSPYPGMPVDSKFYKMIKEGFRMLSPEHAPAEMYDIMKTCWDADPLKRPTFKQI  916

logo      VQLIEKQISESTNHIYSNLANCSPNRQKPVVDHSVRINSVGSTASSSQPLLVHDDV
IMATINIB  ........................................................   20
PF07714   VQL.....................................................  335
PF00047   ........................................................   88
KIT-201   VQLIEKQISESTNHIYSNLANCSPNRQKPVVDHSVRINSVGSTASSSQPLLVHDDV  976
KIT-202   VQLIEKQISESTNHIYSNLANCSPNRQKPVVDHSVRINSVGSTASSSQPLLVHDDV  972
```

X non conserved
X similar
X ≥ 0% conserved
X ≥ 50% conserved

```
logo
             MRGARGAWDFLCVLLLLLRVQTGSSQPSVSPGEPSPPSIHPGKSDLIVRVGDEIRLLCTDPGFVKWTFEILDETNENKQNEWITEKAEATNTGKYTCTNKHGLSNSIYVF
PONATINIB    ..............................................................................................................     0
PF07714      ..............................................................................................................     0
PF00047      ..............................................................................................................     0
KIT-201      MRGARGAWDFLCVLLLLLRVQTGSSQPSVSPGEPSPPSIHPGKSDLIVRVGDEIRLLCTDPGFVKWTFEILDETNENKQNEWITEKAEATNTGKYTCTNKHGLSNSIYVF   110
KIT-202      MRGARGAWDFLCVLLLLLRVQTGSSQPSVSPGEPSPPSIHPGKSDLIVRVGDEIRLLCTDPGFVKWTFEILDETNENKQNEWITEKAEATNTGKYTCTNKHGLSNSIYVF   110

logo
             VRDPAKLFLVDRSLYGKEDNDTLVRCPLTDPEVTNYSLKGCQGKPLPKDLRFIPDPKAGIMIKSVKRAYHRLCLHCSVDQEGKSVLSEKFILKVRPAFKAVPVVSVSKAS
PONATINIB    ..............................................................................................................     0
PF07714      ..............................................................................................................     0
PF00047      ..........................................................................................................SKAS     4
KIT-201      VRDPAKLFLVDRSLYGKEDNDTLVRCPLTDPEVTNYSLKGCQGKPLPKDLRFIPDPKAGIMIKSVKRAYHRLCLHCSVDQEGKSVLSEKFILKVRPAFKAVPVVSVSKAS   220
KIT-202      VRDPAKLFLVDRSLYGKEDNDTLVRCPLTDPEVTNYSLKGCQGKPLPKDLRFIPDPKAGIMIKSVKRAYHRLCLHCSVDQEGKSVLSEKFILKVRPAFKAVPVVSVSKAS   220

logo
             YLLREGEEFTVTCTIKDVSSSVYSTWKRENSQTKLQEKYNSWHHGDFNYERQATLTISSARVNDSGVFMCYANNTFGSANVTTTLEVVDKGFINIFPMINTTVFVNDGEN
PONATINIB    ..............................................................................................................     0
PF07714      ..............................................................................................................     0
PF00047      YLLREGEEFTVTCTIKDVSSSVYSTWKRENSQTKLQEKYNSWHHGDFNYERQATLTISSARVNDSGVFMCYANNTFGSANVTTT..........................    88
KIT-201      YLLREGEEFTVTCTIKDVSSSVYSTWKRENSQTKLQEKYNSWHHGDFNYERQATLTISSARVNDSGVFMCYANNTFGSANVTTTLEVVDKGFINIFPMINTTVFVNDGEN   330
KIT-202      YLLREGEEFTVTCTIKDVSSSVYSTWKRENSQTKLQEKYNSWHHGDFNYERQATLTISSARVNDSGVFMCYANNTFGSANVTTTLEVVDKGFINIFPMINTTVFVNDGEN   330

logo
             VDLIVEYEAFPKPEHQQWIYMNRTFTDKWEDYPKSENESNIRYVSELHLTRLKGTEGGTYTFLVSNSDVNAAIAFNVYVNTKPEILTYDRLVNGMLQCVAAGFPEPTIDW
PONATINIB    ..............................................................................................................     0
PF07714      ..............................................................................................................     0
PF00047      ..............................................................................................................    88
KIT-201      VDLIVEYEAFPKPEHQQWIYMNRTFTDKWEDYPKSENESNIRYVSELHLTRLKGTEGGTYTFLVSNSDVNAAIAFNVYVNTKPEILTYDRLVNGMLQCVAAGFPEPTIDW   440
KIT-202      VDLIVEYEAFPKPEHQQWIYMNRTFTDKWEDYPKSENESNIRYVSELHLTRLKGTEGGTYTFLVSNSDVNAAIAFNVYVNTKPEILTYDRLVNGMLQCVAAGFPEPTIDW   440

logo
             YFCPGTEQRCSASVLPVDVQTLNSSGPPFGKLVVQSSIDSSAFKHNGTVECKAYNDVGKTSAYFNFAFKGNNKEQIHPHTLFTPLLIGFVIVAGMMCIIVMILTYKYLQK
PONATINIB    ..............................................................................................................     0
PF07714      ..............................................................................................................     0
PF00047      ..............................................................................................................    88
KIT-201      YFCPGTEQRCSASVLPVDVQTLNSSGPPFGKLVVQSSIDSSAFKHNGTVECKAYNDVGKTSAYFNFAFKGNNKEQIHPHTLFTPLLIGFVIVAGMMCIIVMILTYKYLQK   550
KIT-202      YFCPGTEQRCSASVLPVDVQTLNSSGPPFGKLVVQSSIDSSAFKHNGTVECKAYNDVGKTSAYFNFAFK....EQIHPHTLFTPLLIGFVIVAGMMCIIVMILTYKYLQK   546

logo
             PMYEVQWKVVEEINGNNYVYIDPTQLPYDHKWEFPRNRLSFGKTLGAGAFGKVVEATAYGLIKSDAAMTVAVKMLKPSAHLTEREALMSELKVLSYLGNHMNIVNLLGAC
PONATINIB    ............................................L.........................AVK................E...L..L.....IV......     9
PF07714      ......................................LSFGKTLGAGAFGKVVEATAYGLIKSDAAMTVAVKMLKPSAHLTEREALMSELKVLSYLGNHMNIVNLLGAC    72
PF00047      ..............................................................................................................    88
KIT-201      PMYEVQWKVVEEINGNNYVYIDPTQLPYDHKWEFPRNRLSFGKTLGAGAFGKVVEATAYGLIKSDAAMTVAVKMLKPSAHLTEREALMSELKVLSYLGNHMNIVNLLGAC   660
KIT-202      PMYEVQWKVVEEINGNNYVYIDPTQLPYDHKWEFPRNRLSFGKTLGAGAFGKVVEATAYGLIKSDAAMTVAVKMLKPSAHLTEREALMSELKVLSYLGNHMNIVNLLGAC   656
```

```
logo        TIGGPTLVITEYCCYGDLLNFLRRKRDSFICSKQEDHAEAALYKNLLHSKESSCSDSTNEYMDMKPGVSYVVPTKADKRRSVRIGSYIERDVTPAIMEDDELALDLEDLL
PONATINIB   .........TEYC.............................................................................................  13
PF07714     TIGGPTLVITEYCCYGDLLNFLRRKRDSFICSKQEDHAEAALYKNLLHSKESSCSDSTNEYMDMKPGVSYVVPTKADKRRSVRIGSYIERDVTPAIMEDDELALDLEDLL 182
PF00047     ..............................................................................................................  88
KIT-201     TIGGPTLVITEYCCYGDLLNFLRRKRDSFICSKQEDHAEAALYKNLLHSKESSCSDSTNEYMDMKPGVSYVVPTKADKRRSVRIGSYIERDVTPAIMEDDELALDLEDLL 770
KIT-202     TIGGPTLVITEYCCYGDLLNFLRRKRDSFICSKQEDHAEAALYKNLLHSKESSCSDSTNEYMDMKPGVSYVVPTKADKRRSVRIGSYIERDVTPAIMEDDELALDLEDLL 766

logo        SFSYQVAKGMAFLASKNCIHRDLAARNILLTHGRITKICDFGLARDIKNDSNYVVKGNARLPVKWMAPESIFNCVYTFESDVWSYGIFLWELFSLGSSPYPGMPVDSKFY
PONATINIB   .................CIHR.......L........ICDF.........................................................................  22
PF07714     SFSYQVAKGMAFLASKNCIHRDLAARNILLTHGRITKICDFGLARDIKNDSNYVVKGNARLPVKWMAPESIFNCVYTFESDVWSYGIFLWELFSLGSSPYPGMPVDSKFY 292
PF00047     ..............................................................................................................  88
KIT-201     SFSYQVAKGMAFLASKNCIHRDLAARNILLTHGRITKICDFGLARDIKNDSNYVVKGNARLPVKWMAPESIFNCVYTFESDVWSYGIFLWELFSLGSSPYPGMPVDSKFY 880
KIT-202     SFSYQVAKGMAFLASKNCIHRDLAARNILLTHGRITKICDFGLARDIKNDSNYVVKGNARLPVKWMAPESIFNCVYTFESDVWSYGIFLWELFSLGSSPYPGMPVDSKFY 876

logo        KMIKEGFRMLSPEHAPAEMYDIMKTCWDADPLKRPTFKQIVQLIEKQISESTNHIYSNLANCSPNRQKPVVDHSVRINSVGSTASSSQPLLVHDDV
PONATINIB   .................................................................................................  22
PF07714     KMIKEGFRMLSPEHAPAEMYDIMKTCWDADPLKRPTFKQIVQL......................................................  335
PF00047     .................................................................................................  88
KIT-201     KMIKEGFRMLSPEHAPAEMYDIMKTCWDADPLKRPTFKQIVQLIEKQISESTNHIYSNLANCSPNRQKPVVDHSVRINSVGSTASSSQPLLVHDDV 976
KIT-202     KMIKEGFRMLSPEHAPAEMYDIMKTCWDADPLKRPTFKQIVQLIEKQISESTNHIYSNLANCSPNRQKPVVDHSVRINSVGSTASSSQPLLVHDDV 972
```

X non conserved
X similar
X $\geq 0\%$ conserved
X $\geq 50\%$ conserved

```
logo        MRGARGAWDFLCVLLLLLRVQTGSSQPSVSPGEPSPPSIHPGKSDLIVRVGDEIRLLCTDPGFVKWTFEILDETNENKQNEWITEKAEATNTGKYTCTNKHGLSNSIYVF
SUNITINIB   ..............................................................................................................    0
PF07714     ..............................................................................................................    0
PF00047     ..............................................................................................................    0
KIT-201     MRGARGAWDFLCVLLLLLRVQTGSSQPSVSPGEPSPPSIHPGKSDLIVRVGDEIRLLCTDPGFVKWTFEILDETNENKQNEWITEKAEATNTGKYTCTNKHGLSNSIYVF  110
KIT-202     MRGARGAWDFLCVLLLLLRVQTGSSQPSVSPGEPSPPSIHPGKSDLIVRVGDEIRLLCTDPGFVKWTFEILDETNENKQNEWITEKAEATNTGKYTCTNKHGLSNSIYVF  110

logo        VRDPAKLFLVDRSLYGKEDNDTLVRCPLTDPEVTNYSLKGCQGKPLPKDLRFIPDPKAGIMIKSVKRAYHRLCLHCSVDQEGKSVLSEKFILKVRPAFKAVPVVSVSKAS
SUNITINIB   ..............................................................................................................    0
PF07714     ..............................................................................................................    0
PF00047     ..........................................................................................................SKAS    4
KIT-201     VRDPAKLFLVDRSLYGKEDNDTLVRCPLTDPEVTNYSLKGCQGKPLPKDLRFIPDPKAGIMIKSVKRAYHRLCLHCSVDQEGKSVLSEKFILKVRPAFKAVPVVSVSKAS  220
KIT-202     VRDPAKLFLVDRSLYGKEDNDTLVRCPLTDPEVTNYSLKGCQGKPLPKDLRFIPDPKAGIMIKSVKRAYHRLCLHCSVDQEGKSVLSEKFILKVRPAFKAVPVVSVSKAS  220

logo        YLLREGEEFTVTCTIKDVSSSVYSTWKRENSQTKLQEKYNSWHHGDFNYERQATLTISSARVNDSGVFMCYANNTFGSANVTTTLEVVDKGFINIFPMINTTVFVNDGEN
SUNITINIB   ..............................................................................................................    0
PF07714     ..............................................................................................................    0
PF00047     YLLREGEEFTVTCTIKDVSSSVYSTWKRENSQTKLQEKYNSWHHGDFNYERQATLTISSARVNDSGVFMCYANNTFGSANVTTT..........................   88
KIT-201     YLLREGEEFTVTCTIKDVSSSVYSTWKRENSQTKLQEKYNSWHHGDFNYERQATLTISSARVNDSGVFMCYANNTFGSANVTTTLEVVDKGFINIFPMINTTVFVNDGEN  330
KIT-202     YLLREGEEFTVTCTIKDVSSSVYSTWKRENSQTKLQEKYNSWHHGDFNYERQATLTISSARVNDSGVFMCYANNTFGSANVTTTLEVVDKGFINIFPMINTTVFVNDGEN  330

logo        VDLIVEYEAFPKPEHQQWIYMNRTFTDKWEDYPKSENESNIRYVSELHLTRLKGTEGGTYTFLVSNSDVNAAIAFNVYVNTKPEILTYDRLVNGMLQCVAAGFPEPTIDW
SUNITINIB   ..............................................................................................................    0
PF07714     ..............................................................................................................    0
PF00047     ..............................................................................................................   88
KIT-201     VDLIVEYEAFPKPEHQQWIYMNRTFTDKWEDYPKSENESNIRYVSELHLTRLKGTEGGTYTFLVSNSDVNAAIAFNVYVNTKPEILTYDRLVNGMLQCVAAGFPEPTIDW  440
KIT-202     VDLIVEYEAFPKPEHQQWIYMNRTFTDKWEDYPKSENESNIRYVSELHLTRLKGTEGGTYTFLVSNSDVNAAIAFNVYVNTKPEILTYDRLVNGMLQCVAAGFPEPTIDW  440

logo        YFCPGTEQRCSASVLPVDVQTLNSSGPPFGKLVVQSSIDSSAFKHNGTVECKAYNDVGKTSAYFNFAFKGNNKEQIHPHTLFTPLLIGFVIVAGMMCIIVMILTYKYLQK
SUNITINIB   ..............................................................................................................    0
PF07714     ..............................................................................................................    0
PF00047     ..............................................................................................................   88
KIT-201     YFCPGTEQRCSASVLPVDVQTLNSSGPPFGKLVVQSSIDSSAFKHNGTVECKAYNDVGKTSAYFNFAFKGNNKEQIHPHTLFTPLLIGFVIVAGMMCIIVMILTYKYLQK  550
KIT-202     YFCPGTEQRCSASVLPVDVQTLNSSGPPFGKLVVQSSIDSSAFKHNGTVECKAYNDVGKTSAYFNFAFK....EQIHPHTLFTPLLIGFVIVAGMMCIIVMILTYKYLQK  546

logo        PMYEVQWKVVEEINGNNYVYIDPTQLPYDHKWEFPRNRLSFGKTLGAGAFGKVVEATAYGLIKSDAAMTVAVKMLKPSAHLTEREALMSELKVLSYLGNHMNIVNLLGAC
SUNITINIB   ............................................L.......V.................A.......................................    3
PF07714     ......................................LSFGKTLGAGAFGKVVEATAYGLIKSDAAMTVAVKMLKPSAHLTEREALMSELKVLSYLGNHMNIVNLLGAC   72
PF00047     ..............................................................................................................   88
KIT-201     PMYEVQWKVVEEINGNNYVYIDPTQLPYDHKWEFPRNRLSFGKTLGAGAFGKVVEATAYGLIKSDAAMTVAVKMLKPSAHLTEREALMSELKVLSYLGNHMNIVNLLGAC  660
KIT-202     PMYEVQWKVVEEINGNNYVYIDPTQLPYDHKWEFPRNRLSFGKTLGAGAFGKVVEATAYGLIKSDAAMTVAVKMLKPSAHLTEREALMSELKVLSYLGNHMNIVNLLGAC  656
```

```
logo
```
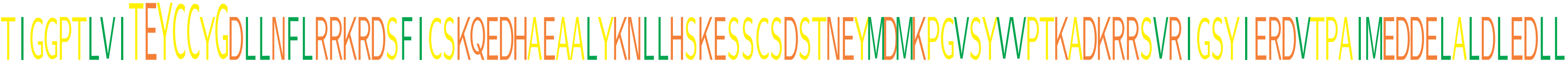

```
SUNITINIB   .........TEYCC.G.............................................................................................   9
PF07714     TIGGPTLVITEYCCYGDLLNFLRRKRDSFICSKQEDHAEAALYKNLLHSKESSCSDSTNEYMDMKPGVSYVVPTKADKRRSVRIGSYIERDVTPAIMEDDELALDLEDLL   182
PF00047     ..............................................................................................................   88
KIT-201     TIGGPTLVITEYCCYGDLLNFLRRKRDSFICSKQEDHAEAALYKNLLHSKESSCSDSTNEYMDMKPGVSYVVPTKADKRRSVRIGSYIERDVTPAIMEDDELALDLEDLL   770
KIT-202     TIGGPTLVITEYCCYGDLLNFLRRKRDSFICSKQEDHAEAALYKNLLHSKESSCSDSTNEYMDMKPGVSYVVPTKADKRRSVRIGSYIERDVTPAIMEDDELALDLEDLL   766
```

```
logo
```
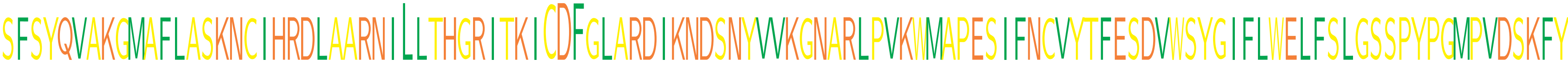

```
SUNITINIB   ............................L.........CDF.....................................................................   13
PF07714     SFSYQVAKGMAFLASKNCIHRDLAARNILLTHGRITKICDFGLARDIKNDSNYVVKGNARLPVKWMAPESIFNCVYTFESDVWSYGIFLWELFSLGSSPYPGMPVDSKFY   292
PF00047     ..............................................................................................................   88
KIT-201     SFSYQVAKGMAFLASKNCIHRDLAARNILLTHGRITKICDFGLARDIKNDSNYVVKGNARLPVKWMAPESIFNCVYTFESDVWSYGIFLWELFSLGSSPYPGMPVDSKFY   880
KIT-202     SFSYQVAKGMAFLASKNCIHRDLAARNILLTHGRITKICDFGLARDIKNDSNYVVKGNARLPVKWMAPESIFNCVYTFESDVWSYGIFLWELFSLGSSPYPGMPVDSKFY   876
```

```
logo
```
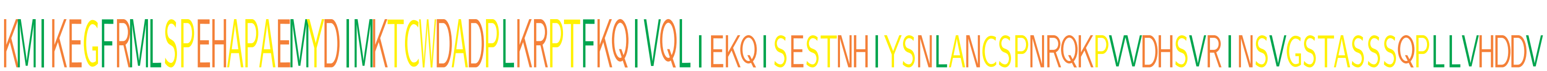

```
SUNITINIB   ................................................................................................   13
PF07714     KMIKEGFRMLSPEHAPAEMYDIMKTCWDADPLKRPTFKQIVQL.....................................................   335
PF00047     ................................................................................................   88
KIT-201     KMIKEGFRMLSPEHAPAEMYDIMKTCWDADPLKRPTFKQIVQLIEKQISESTNHIYSNLANCSPNRQKPVVDHSVRINSVGSTASSSQPLLVHDDV   976
KIT-202     KMIKEGFRMLSPEHAPAEMYDIMKTCWDADPLKRPTFKQIVQLIEKQISESTNHIYSNLANCSPNRQKPVVDHSVRINSVGSTASSSQPLLVHDDV   972
```

- X non conserved
- X similar
- X $\geq$ 0% conserved
- X $\geq$ 50% conserved

```
logo
UBENIMEX   ..............................................................................................................   0
PF01433    ..............................................................................................................   0
PF09127    ..............................................................................................................   0
LTA4H-201  MPEIVDTCSLASPASVCRTKHLHLRCSVDFTRRTLTGTAALTVQSQEDNLRSLVLDTKDLTIEKVVINGQEVKYALGERQSYKGSPMEISLPIALSKNQEIVIEISFETS 110
LTA4H-207  MPEIVDTCSLASPASVCRTKHLHLRCSVDFTRRTLTGTAALTVQSQEDNLRSLVLDTKDLTIEKVVINGQEVKYALGERQSYKGSPMEISLPIALSNGSLLNRLLGRNTH 110
LTA4H-209  .......................MLPQRNLSKRQ.VPTMHIPVKTRRLLAALKVLDTKDLTIEKVVINGQEVKYALGERQSYKGSPMEISLPIALSKNQEIVIEISFETS  86
LTA4H-202  .......................MLPQRNLSKRQ.VPTMHIPVKTRRLLAALKVLDTKDLTIEKVVINGQEVKYALGERQSYKGSPMEISLPIALSKNQEIVIEISFETS  86

logo
UBENIMEX   ........................Q.Q...................................................................................   2
PF01433    ............................................................................................................TL   2
PF09127    ..............................................................................................................   0
LTA4H-201  PKSSALQWLTPEQTSGKEHPYLFSQCQAIHCRAILPCQDTPSVKLTYTAEVSVPKELVALMSAIRDGETPDPEDPSRKIYKFIQKVPIPCYLIALVVGALESRQIGPRTL 220
LTA4H-207  I.....SL.VSARPSTAEQSFLVRTL........LL.......................................................................... 132
LTA4H-209  PKSSALQWLTPEQTSGKEHPYLFSQCQAIHCRAILPCQDTPSVKLTYTAEVSVPKELVALMSAIRDGETPDPEDPSRKIYKFIQKVPIPCYLIALVVGALESRQIGPRTL 196
LTA4H-202  PKSSALQWLTPEQTSGKEHPYLFSQCQAIHCRAILPCQDTPSVKLTYTAEVSVPKELVALMSAIRDGETPDPEDPSRKIYKFIQKVPIPCYLIALVVGALESRQIGPRTL 196

logo
UBENIMEX   ...............................................YGGME.......................HE..H..............F...E...........  12
PF01433    VWSEKEQVEKSAYEFSETESMLKIAEDLGGPYVWGQYDLLVLPPSFPYGGMENPCLTFVTPTLLAGDKSLSNVIAHEISHSWTGNLVTNKTWDHFWLNEGHTVYLERHIC 112
PF09127    ..............................................................................................................   0
LTA4H-201  VWSEKEQVEKSAYEFSETESMLKIAEDLGGPYVWGQYDLLVLPPSFPYGGMENPCLTFVTPTLLAGDKSLSNVIAHEISHSWTGNLVTNKTWDHFWLNEGHTVYLERHIC 330
LTA4H-207  .............................................................................................................. 132
LTA4H-209  VWSEKEQVEKSAYEFSETESMLKIAEDLGGPYVWGQYDLLVLPPSFPYGGMENPCLTFVTPTLLAGDKSLSNVIAHEISHSWTGNLVTNKTWDHFWLNEGHTVYLERHIC 306
LTA4H-202  VWSEKEQVEKSAYEFSETESMLKIAEDLGGPYVWGQYDLLVLPPSFPYGGMENPCLTFVTPTLLAGDKSLSNVIAHEISHSWTGNLVTNKTWDHFWLNEGHTVYLERHIC 306

logo
UBENIMEX   ................................................Y....Y........................................................  14
PF01433    GRLFGEKFRHFNALGGWGELQNSVKTFGETHPFTKLVVDLTDIDPDVAYSSVPYEKGFALLFYLEQLLGGPEIFLGFLKAYVEKFSYKSITTDDWKDFLYSYFKDKVDV. 221
PF09127    ..............................................................................................................   0
LTA4H-201  GRLFGEKFRHFNALGGWGELQNSVKTFGETHPFTKLVVDLTDIDPDVAYSSVPYEKGFALLFYLEQLLGGPEIFLGFLKAYVEKFSYKSITTDDWKDFLYSYFKDKVDVL 440
LTA4H-207  .............................................................................................................. 132
LTA4H-209  GRLFGEKFRHFNALGGWGELQNSVKTFGETHPFTKLVVDLTDIDPDVAYSSVPYEKGFALLFYLEQLLGGPEIFLGFLKAYVEKFSYKSITTDDWKDFLYSYFKDKVDVL 416
LTA4H-202  GRLFGEKFRHFNALGGWGELQNSVKTFGETHPFTKLVVDLTDIDPDVAYSSVPYEKGFALLFYLEQLLGGPEIFLGFLKAYVEKFSYKSITTDDWKDFLYSYFKDKVDVL 416

logo
UBENIMEX   ..............................................................................................................  14
PF01433    .............................................................................................................. 221
PF09127    ......................................................LSSHQLNEFLAQTLQRAPLPLGHIKRMQEVYNFNAINNSEIRFRWLRLCIQSKWED  56
LTA4H-201  NQVDWNAWLYSPGLPPIKPNYDMTLTNACIALSQRWITAKEDDLNSFNATDLKDLSSHQLNEFLAQTLQRAPLPLGHIKRMQEVYNFNAINNSEIRFRWLRLCIQSKWED 550
LTA4H-207  .............................................................................................................. 132
LTA4H-209  NQVDWNAWLYSPGLPPIKPNYDMTLTNACIALSQRWITAKEDDLNSFNATDLKDLSSHQLNEFLAQTLQRAPLPLGHIKRMQEVYNFNAINNSEIRFRWLRLCIQSKWED 526
LTA4H-202  NQVDWNAWLYSPGLPPIKPNYDMTLTNACIALSQRWITAKEDDLNSFNATDLKDLSSHQLNEFLAQTLQRMAAALHSIQVGG.RNSFGAKDGN................. 508
```

```
logo
             AIPLALKMATEQGRMKFTRPLFKDLAAFDKSHDQAVRTYQEHKASMHPVTAMLVGKDLKVD
UBENIMEX     .............................................................    14
PF01433      .............................................................   221
PF09127      AIPLALKMATEQGRMKFTRPLFKDLAAFDKSHDQAVRTYQEHKASMHPVTAMLVGKDL...   114
LTA4H-201    AIPLALKMATEQGRMKFTRPLFKDLAAFDKSHDQAVRTYQEHKASMHPVTAMLVGKDLKVD   611
LTA4H-207    .............................................................   132
LTA4H-209    AIPLALKMATEQGRMKFTRPLFKDLAAFDKSHDQAVRTYQEHKASMHPVTAMLVGKDLKVD   587
LTA4H-202    .............................................................   508
```

X non conserved

X similar

X $\geq$ 0% conserved

X $\geq$ 50% conserved

```
logo
COBIMETINIB   .............................................................................N..................K.............   2
PF00069       ...................................................................FEKISELGAGNGGVVFKVSHKPSGLVMARKLIHLEIKPAIRNQ   43
PF00069.1     ..............................................................................................................   0
MAP2K1-201    MPKKKPTPIQLNPAPDGSAVNGTSSAETNLEALQKKLEELELDEQQRKRLEAFLTQKQKVGELKDDDFEKISELGAGNGGVVFKVSHKPSGLVMARKLIHLEIKPAIRNQ   110
MAP2K1-203    ..............................................................................................................   0

logo
COBIMETINIB   ....L..L........V.............I.M..............................................D....N............DF.VS..L...M.   15
PF00069       IIRELQVLHECNSPYIVGFYGAFYSDGEISICMEHMDGGSLDQVLKKAGRIPEQILGKVSIAVIKGLTYLREKHKIMHRDVKPSNILVNSRGEIKLCDFGVSGQLIDSMA   153
PF00069.1     ................................................................................VKPSNILVNSRGEIKLCDFGVSGQLIDSMA   30
MAP2K1-201    IIRELQVLHECNSPYIVGFYGAFYSDGEISICMEHMDGGSLDQVLKKAGRIPEQILGKVSIAVIKGLTYLREKHKIMHRDVKPSNILVNSRGEIKLCDFGVSGQLIDSMA   220
MAP2K1-203    ..................................................................MVPVHQQFLSKQHNVKPSNILVNSRGEIKLCDFGVSGQLIDSMA   44

logo
COBIMETINIB   ....G.........................................................................................................   16
PF00069       NSFVGTRSYMSPERLQGTHYSVQSDIWSMGLSLVEMAVGRYPIPPPDAKELELMFGCQVEGDAAETPPRPRTPGRPLSSYGMDSRPPMAIFELLDYIVNEPPPKLPSGVF   263
PF00069.1     NSFVGTRSYMSPERLQGTHYSVQSDIWSMGLSLVEMAVGRYPIPPPDAKELELMFGCQVEGDAAETPPRPRTPGRPLSSYGMDSRPPMAIFELLDYIVNEPPPKLPSGVF   140
MAP2K1-201    NSFVGTRSYMSPERLQGTHYSVQSDIWSMGLSLVEMAVGRYPIPPPDAKELELMFGCQVEGDAAETPPRPRTPGRPLSSYGMDSRPPMAIFELLDYIVNEPPPKLPSGVF   330
MAP2K1-203    NSFVGTRSYMSPERLQGTHYSVQSDIWSMGLSLVEMAVGRYPIPPPDAKELELMFGCQVEGDAAETPPRPRTPGRPLSSYGMDSRPPMAIFELLDYIVNEPPPKLPSGVF   154

logo
COBIMETINIB   ...............................................................   16
PF00069       SLEFQDFVNKCLIKNPAERADLKQLMVHAFI................................   294
PF00069.1     SLEFQDFVNKCLIKNPAERADLKQLMVHAFI................................   171
MAP2K1-201    SLEFQDFVNKCLIKNPAERADLKQLMVHAFIKRSDAEEVDFAGWLCSTIGLNQPSTPTHAAGV   393
MAP2K1-203    SLEFQDFVNKCLIKNPAERADLKQLMVHAFIKRSDAEEVDFAGWLCSTIGLNQPSTPTHAAGV   217
```

X non conserved
X similar
X $\geq$ 0% conserved
X $\geq$ 50% conserved

logo

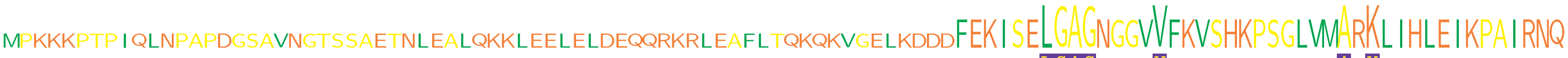

```
K-252A       .........................................................................LGAG....V............A.K.............   7
PF00069      ...................................................................FEKISELGAGNGGVVFKVSHKPSGLVMARKLIHLEIKPAIRNQ  43
PF00069.1    ..............................................................................................................   0
MAP2K1-201   MPKKKPTPIQLNPAPDGSAVNGTSSAETNLEALQKKLEELELDEQQRKRLEAFLTQKQKVGELKDDDFEKISELGAGNGGVVFKVSHKPSGLVMARKLIHLEIKPAIRNQ 110
MAP2K1-203   ..............................................................................................................   0
```

logo

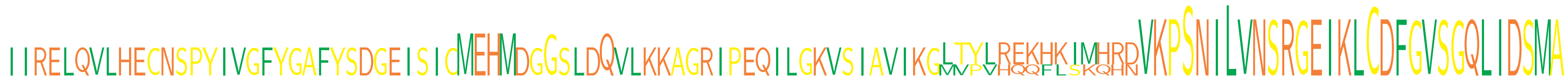

```
K-252A       ................................MEHM..G...Q........................................S..L.........C.............  16
PF00069      IIRELQVLHECNSPYIVGFYGAFYSDGEISICMEHMDGGSLDQVLKKAGRIPEQILGKVSIAVIKGLTYLREKHKIMHRDVKPSNILVNSRGEIKLCDFGVSGQLIDSMA 153
PF00069.1    ................................................................................VKPSNILVNSRGEIKLCDFGVSGQLIDSMA  30
MAP2K1-201   IIRELQVLHECNSPYIVGFYGAFYSDGEISICMEHMDGGSLDQVLKKAGRIPEQILGKVSIAVIKGLTYLREKHKIMHRDVKPSNILVNSRGEIKLCDFGVSGQLIDSMA 220
MAP2K1-203   ..................................................................MVPVHQQFLSKQHNVKPSNILVNSRGEIKLCDFGVSGQLIDSMA  44
```

logo

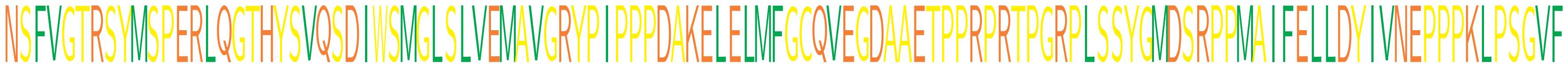

```
K-252A       ..............................................................................................................  16
PF00069      NSFVGTRSYMSPERLQGTHYSVQSDIWSMGLSLVEMAVGRYPIPPPDAKELELMFGCQVEGDAAETPPRPRTPGRPLSSYGMDSRPPMAIFELLDYIVNEPPPKLPSGVF 263
PF00069.1    NSFVGTRSYMSPERLQGTHYSVQSDIWSMGLSLVEMAVGRYPIPPPDAKELELMFGCQVEGDAAETPPRPRTPGRPLSSYGMDSRPPMAIFELLDYIVNEPPPKLPSGVF 140
MAP2K1-201   NSFVGTRSYMSPERLQGTHYSVQSDIWSMGLSLVEMAVGRYPIPPPDAKELELMFGCQVEGDAAETPPRPRTPGRPLSSYGMDSRPPMAIFELLDYIVNEPPPKLPSGVF 330
MAP2K1-203   NSFVGTRSYMSPERLQGTHYSVQSDIWSMGLSLVEMAVGRYPIPPPDAKELELMFGCQVEGDAAETPPRPRTPGRPLSSYGMDSRPPMAIFELLDYIVNEPPPKLPSGVF 154
```

logo

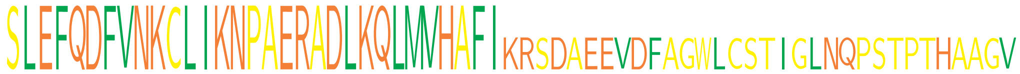

```
K-252A       ...............................................................   16
PF00069      SLEFQDFVNKCLIKNPAERADLKQLMVHAFI................................  294
PF00069.1    SLEFQDFVNKCLIKNPAERADLKQLMVHAFI................................  171
MAP2K1-201   SLEFQDFVNKCLIKNPAERADLKQLMVHAFIKRSDAEEVDFAGWLCSTIGLNQPSTPTHAAGV  393
MAP2K1-203   SLEFQDFVNKCLIKNPAERADLKQLMVHAFIKRSDAEEVDFAGWLCSTIGLNQPSTPTHAAGV  217
```

[X] non conserved
[X] similar
[X] ≥ 0% conserved
[X] ≥ 50% conserved

logo 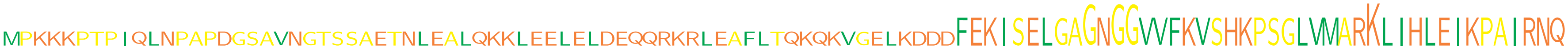

```
PD-0325901   ..........................................................................................G.GG....................K.............     4
PF00069      ................................................................FEKISELGAGNGGVVFKVSHKPSGLVMARKLIHLEIKPAIRNQ    43
PF00069.1    .............................................................................................................     0
MAP2K1-201   MPKKKPTPIQLNPAPDGSAVNGTSSAETNLEALQKKLEELELDEQQRKRLEAFLTQKQKVGELKDDDFEKISELGAGNGGVVFKVSHKPSGLVMARKLIHLEIKPAIRNQ   110
MAP2K1-203   .............................................................................................................     0
```

logo 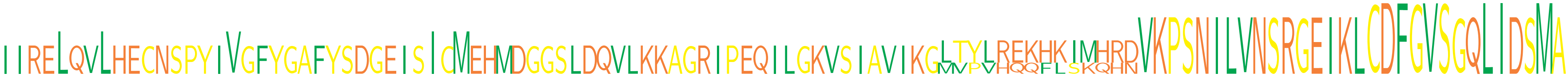

```
PD-0325901   ....L..L........V..............I.M.................................................CDFGVS..LI..M.    18
PF00069      IIRELQVLHECNSPYIVGFYGAFYSDGEISICMEHMDGGSLDQVLKKAGRIPEQILGKVSIAVIKGLTYLREKHKIMHRDVKPSNILVNSRGEIKLCDFGVSGQLIDSMA   153
PF00069.1    ................................................................................VKPSNILVNSRGEIKLCDFGVSGQLIDSMA    30
MAP2K1-201   IIRELQVLHECNSPYIVGFYGAFYSDGEISICMEHMDGGSLDQVLKKAGRIPEQILGKVSIAVIKGLTYLREKHKIMHRDVKPSNILVNSRGEIKLCDFGVSGQLIDSMA   220
MAP2K1-203   ..................................................................MVPVHQQFLSKQHNVKPSNILVNSRGEIKLCDFGVSGQLIDSMA    44
```

logo 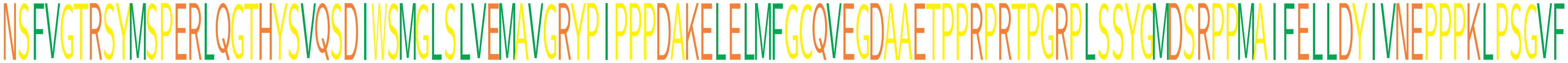

```
PD-0325901   .............................................................................................................    18
PF00069      NSFVGTRSYMSPERLQGTHYSVQSDIWSMGLSLVEMAVGRYPIPPPDAKELELMFGCQVEGDAAETPPRPRTPGRPLSSYGMDSRPPMAIFELLDYIVNEPPPKLPSGVF   263
PF00069.1    NSFVGTRSYMSPERLQGTHYSVQSDIWSMGLSLVEMAVGRYPIPPPDAKELELMFGCQVEGDAAETPPRPRTPGRPLSSYGMDSRPPMAIFELLDYIVNEPPPKLPSGVF   140
MAP2K1-201   NSFVGTRSYMSPERLQGTHYSVQSDIWSMGLSLVEMAVGRYPIPPPDAKELELMFGCQVEGDAAETPPRPRTPGRPLSSYGMDSRPPMAIFELLDYIVNEPPPKLPSGVF   330
MAP2K1-203   NSFVGTRSYMSPERLQGTHYSVQSDIWSMGLSLVEMAVGRYPIPPPDAKELELMFGCQVEGDAAETPPRPRTPGRPLSSYGMDSRPPMAIFELLDYIVNEPPPKLPSGVF   154
```

logo 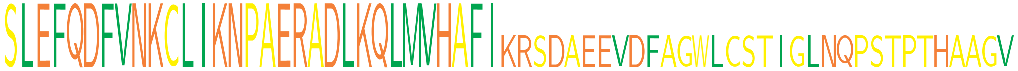

```
PD-0325901   ..............................................................    18
PF00069      SLEFQDFVNKCLIKNPAERADLKQLMVHAFI...............................   294
PF00069.1    SLEFQDFVNKCLIKNPAERADLKQLMVHAFI...............................   171
MAP2K1-201   SLEFQDFVNKCLIKNPAERADLKQLMVHAFIKRSDAEEVDFAGWLCSTIGLNQPSTPTHAAGV   393
MAP2K1-203   SLEFQDFVNKCLIKNPAERADLKQLMVHAFIKRSDAEEVDFAGWLCSTIGLNQPSTPTHAAGV   217
```

- X non conserved
- X similar
- X $\geq$ 0% conserved
- X $\geq$ 50% conserved

```
logo         MPKKKPTPIQLNPAPDGSAVNGTSSAETNLEALQKKLEELELDEQQRKRLEAFLTQKQKVGELKDDDFEKISELGAGNGGVVFKVSHKPSGLVMARKLIHLEIKPAIRNQ
RO-4987655   ..............................................................................GG................K.............   3
PF00069      ...................................................................FEKISELGAGNGGVVFKVSHKPSGLVMARKLIHLEIKPAIRNQ  43
PF00069.1    ..............................................................................................................   0
MAP2K1-201   MPKKKPTPIQLNPAPDGSAVNGTSSAETNLEALQKKLEELELDEQQRKRLEAFLTQKQKVGELKDDDFEKISELGAGNGGVVFKVSHKPSGLVMARKLIHLEIKPAIRNQ 110
MAP2K1-203   ..............................................................................................................   0

logo         IIRELQVLHECNSPYIVGFYGAFYSDGEISICMEHMDGGSLDQVLKKAGRIPEQILGKVSIAVIKGLTYLREKHKIMHRDVKPSNILVNSRGEIKLCDFGVSGQLIDSMA
RO-4987655   ....L..L........V.............I.M............................................HR..................DFGVS..LI....  17
PF00069      IIRELQVLHECNSPYIVGFYGAFYSDGEISICMEHMDGGSLDQVLKKAGRIPEQILGKVSIAVIKGLTYLREKHKIMHRDVKPSNILVNSRGEIKLCDFGVSGQLIDSMA 153
PF00069.1    ................................................................................VKPSNILVNSRGEIKLCDFGVSGQLIDSMA  30
MAP2K1-201   IIRELQVLHECNSPYIVGFYGAFYSDGEISICMEHMDGGSLDQVLKKAGRIPEQILGKVSIAVIKGLTYLREKHKIMHRDVKPSNILVNSRGEIKLCDFGVSGQLIDSMA 220
MAP2K1-203   ..................................................................MVPVHQQFLSKQHNVKPSNILVNSRGEIKLCDFGVSGQLIDSMA  44

logo         NSFVGTRSYMSPERLQGTHYSVQSDIWSMGLSLVEMAVGRYPIPPPDAKELELMFGCQVEGDAAETPPRPRTPGRPLSSYGMDSRPPMAIFELLDYIVNEPPPKLPSGVF
RO-4987655   N.............................................................................................................  18
PF00069      NSFVGTRSYMSPERLQGTHYSVQSDIWSMGLSLVEMAVGRYPIPPPDAKELELMFGCQVEGDAAETPPRPRTPGRPLSSYGMDSRPPMAIFELLDYIVNEPPPKLPSGVF 263
PF00069.1    NSFVGTRSYMSPERLQGTHYSVQSDIWSMGLSLVEMAVGRYPIPPPDAKELELMFGCQVEGDAAETPPRPRTPGRPLSSYGMDSRPPMAIFELLDYIVNEPPPKLPSGVF 140
MAP2K1-201   NSFVGTRSYMSPERLQGTHYSVQSDIWSMGLSLVEMAVGRYPIPPPDAKELELMFGCQVEGDAAETPPRPRTPGRPLSSYGMDSRPPMAIFELLDYIVNEPPPKLPSGVF 330
MAP2K1-203   NSFVGTRSYMSPERLQGTHYSVQSDIWSMGLSLVEMAVGRYPIPPPDAKELELMFGCQVEGDAAETPPRPRTPGRPLSSYGMDSRPPMAIFELLDYIVNEPPPKLPSGVF 154

logo         SLEFQDFVNKCLIKNPAERADLKQLMVHAFIKRSDAEEVDFAGWLCSTIGLNQPSTPTHAAGV
RO-4987655   ...............................................................  18
PF00069      SLEFQDFVNKCLIKNPAERADLKQLMVHAFI................................ 294
PF00069.1    SLEFQDFVNKCLIKNPAERADLKQLMVHAFI................................ 171
MAP2K1-201   SLEFQDFVNKCLIKNPAERADLKQLMVHAFIKRSDAEEVDFAGWLCSTIGLNQPSTPTHAAGV 393
MAP2K1-203   SLEFQDFVNKCLIKNPAERADLKQLMVHAFIKRSDAEEVDFAGWLCSTIGLNQPSTPTHAAGV 217
```

X non conserved
X similar
X ≥ 0% conserved
X ≥ 50% conserved

```
logo        MPKKKPTPIQLNPAPDGSAVNGTSSAETNLEALQKKLEELELDEQQRKRLEAFLTQKQKVGELKDDDFEKISELGAGNGGVVFKVSHKPSGLVMARKLIHLEIKPAIRNQ
TAK-733     .............................................................................N.G................K.............    3
PF00069     ...................................................................FEKISELGAGNGGVVFKVSHKPSGLVMARKLIHLEIKPAIRNQ   43
PF00069.1   ..............................................................................................................    0
MAP2K1-201  MPKKKPTPIQLNPAPDGSAVNGTSSAETNLEALQKKLEELELDEQQRKRLEAFLTQKQKVGELKDDDFEKISELGAGNGGVVFKVSHKPSGLVMARKLIHLEIKPAIRNQ  110
MAP2K1-203  ..............................................................................................................    0

logo        IIRELQVLHECNSPYIVGFYGAFYSDGEISICMEHMDGGSLDQVLKKAGRIPEQILGKVSIAVIKGLTYLREKHKIMHRDVKPSNILVNSRGEIKLCDFGVSGQLIDSMA
                                                                              MVPVHQQFLSKQHN
TAK-733     ....L..L........V.............I.M................................................................DFGVS..LI....   15
PF00069     IIRELQVLHECNSPYIVGFYGAFYSDGEISICMEHMDGGSLDQVLKKAGRIPEQILGKVSIAVIKGLTYLREKHKIMHRDVKPSNILVNSRGEIKLCDFGVSGQLIDSMA  153
PF00069.1   ................................................................................VKPSNILVNSRGEIKLCDFGVSGQLIDSMA   30
MAP2K1-201  IIRELQVLHECNSPYIVGFYGAFYSDGEISICMEHMDGGSLDQVLKKAGRIPEQILGKVSIAVIKGLTYLREKHKIMHRDVKPSNILVNSRGEIKLCDFGVSGQLIDSMA  220
MAP2K1-203  ..................................................................MVPVHQQFLSKQHNVKPSNILVNSRGEIKLCDFGVSGQLIDSMA   44

logo        NSFVGTRSYMSPERLQGTHYSVQSDIWSMGLSLVEMAVGRYPIPPPDAKELELMFGCQVEGDAAETPPRPRTPGRPLSSYGMDSRPPMAIFELLDYIVNEPPPKLPSGVF
TAK-733     ..............................................................................................................   15
PF00069     NSFVGTRSYMSPERLQGTHYSVQSDIWSMGLSLVEMAVGRYPIPPPDAKELELMFGCQVEGDAAETPPRPRTPGRPLSSYGMDSRPPMAIFELLDYIVNEPPPKLPSGVF  263
PF00069.1   NSFVGTRSYMSPERLQGTHYSVQSDIWSMGLSLVEMAVGRYPIPPPDAKELELMFGCQVEGDAAETPPRPRTPGRPLSSYGMDSRPPMAIFELLDYIVNEPPPKLPSGVF  140
MAP2K1-201  NSFVGTRSYMSPERLQGTHYSVQSDIWSMGLSLVEMAVGRYPIPPPDAKELELMFGCQVEGDAAETPPRPRTPGRPLSSYGMDSRPPMAIFELLDYIVNEPPPKLPSGVF  330
MAP2K1-203  NSFVGTRSYMSPERLQGTHYSVQSDIWSMGLSLVEMAVGRYPIPPPDAKELELMFGCQVEGDAAETPPRPRTPGRPLSSYGMDSRPPMAIFELLDYIVNEPPPKLPSGVF  154

logo        SLEFQDFVNKCLIKNPAERADLKQLMVHAFIKRSDAEEVDFAGWLCSTIGLNQPSTPTHAAGV
TAK-733     ...............................................................   15
PF00069     SLEFQDFVNKCLIKNPAERADLKQLMVHAFI................................  294
PF00069.1   SLEFQDFVNKCLIKNPAERADLKQLMVHAFI................................  171
MAP2K1-201  SLEFQDFVNKCLIKNPAERADLKQLMVHAFIKRSDAEEVDFAGWLCSTIGLNQPSTPTHAAGV  393
MAP2K1-203  SLEFQDFVNKCLIKNPAERADLKQLMVHAFIKRSDAEEVDFAGWLCSTIGLNQPSTPTHAAGV  217
```

X non conserved
X similar
X ≥0% conserved
X ≥50% conserved

```
logo
DORAMAPIMOD   .....................................V..............K.............R...E..LL..M.....I...................L.T.LMG   13
PF00069.4     .............................................................................MKHENVIGLLDVFTPARSLEEFNDVYLVTHLMG   33
PF00069.2     .......................YQNLSPVGSGAYGSVCAAFDTKTGLRVAVKKLSRPFQSIIHAKRTYRELRLLKHMKHENVIGLLDVFTPARSLEEFNDVYLVTHLMG   87
PF00069.1     .......................YQNLSPVGSGAYGSVCAAFDTKTGLRVAVKKLSRPFQSIIHAKRTYRELRLLKHMKHENVIGLLDVFTPARSLEEFNDVYLVTHLMG   87
PF00069.3     .............................................................................MKHENVIGLLDVFTPARSLEEFNDVYLVTHLMG   33
PF00069       .......................YQNLSPVGSGAYGSVCAAFDTKTGLRVAVKKLSRPFQSIIHAKRTYRELRLLKHMKHENVIGLLDVFTPARSLEEFNDVYLVTHLMG   87
PF00069.5     .......................YQNLSPVGSGAYGSVCAAFDTKTGLRVAVKKLSRPFQSIIHAKRTYRELRLLKHMKHENVIGLLDVFTPARSLEEFNDVYLVTHLMG   87
MAPK14-204    .............................................................................MKHENVIGLLDVFTPARSLEEFNDVYLVTHLMG   33
MAPK14-202    MSQERPTFYRQELNKTIWEVPERYQNLSPVGSGAYGSVCAAFDTKTGLRVAVKKLSRPFQSIIHAKRTYRELRLLKHMKHENVIGLLDVFTPARSLEEFNDVYLVTHLMG  110
MAPK14-205    .............................................................................MKHENVIGLLDVFTPARSLEEFNDVYLVTHLMG   33
MAPK14-206    .............XKTIWEVPERYQNLSPVGSGAYGSVCAAFDTKTGLRVAVKKLSRPFQSIIHAKRTYRELRLLKHMKHENVIGLLDVFTPARSLEEFNDVA.......   90
MAPK14-201    MSQERPTFYRQELNKTIWEVPERYQNLSPVGSGAYGSVCAAFDTKTGLRVAVKKLSRPFQSIIHAKRTYRELRLLKHMKHENVIGLLDVFTPARSLEEFNDVYLVTHLMG  110
MAPK14-210    MSQERPTFYRQELNKTIWEVPERYQNLSPVGSGAYGSVCAAFDTKTGLRVAVKKLSRPFQSIIHAKRTYRELRLLKHMKHENVIGLLDVFTPARSLEEFNDVYLVTHLMG  110
MAPK14-208    MSQERPTFYRQELNKTIWEVPERYQNLSPVGSGAYGSVCAAFDTKTGLRVAVKKLSRPFQSIIHAKRTYRELRLLKHMKHENVIGLLDVFTPARSLEEFNDVYLVTHLMG  110
MAPK14-203    MSQERPTFYRQELNKTIWEVPERYQNLSPVGSGAYGSVCAAFDTKTGLRVAVKKLSRPFQSIIHAKRTYRELRLLKHMKHENVIGLLDVFTPARSLEEFNDVYLVTHLMG  110

logo
DORAMAPIMOD   .....................................H..................LDF...................................................   17
PF00069.4     ADLNNIVKCQKLTDDHVQFLIYQILRGLKYIHSADIIHRDLKPSNLAVNEDCELKILDFGLARHTDDEMTGYVATRWYRAPEIMLNWMHYNQTVDIWSVGCIMAELLTGR  143
PF00069.2     ADLNNIVKCQKLTDDHVQFLIYQILRGLKYIHSADIIHRDLKPSNLAVNEDCELKILDFGLARHTDDEMTGYVATRWYRAPEIMLNWMHYNQTVDIWSVGCIMAELLTGR  197
PF00069.1     ADLNNIVKCQKLTDDHVQFLIYQILRGLKYIHSADIIHRDLKPSNLAVNEDCELKILDFGLARHTDDEMTGYVATRWYRAPEIMLNWMHYNQTVDIWSVGCIMAELLTGR  197
PF00069.3     ADLNNIVKCQKLTDDHVQFLIYQILRGLKYIHSADIIHRDLKPSNLAVNEDCELKILDFGLARHTDDEMTGYVATRWYRAPEIMLNWMHYNQTVDIWSV...........  132
PF00069       ADLNNIVKCQKLTDDHVQFLIYQILRGLKYIHSADIIHRDLKPSNLAVNEDCELKILDFGLARHTDDEMTGYVATRWYRAPEIMLNWMHYNQTVDIWSVGCIMAELLTGR  197
PF00069.5     ADLNNIVKCQKLTDDHVQFLIYQILRGLKYIHSADIIHRDLKPSNLAVNEDCELK.......................................................  142
MAPK14-204    ADLNNIVKCQKLTDDHVQFLIYQILRGLKYIHSADIIHRDLKPSNLAVNEDCELKILDFGLARHTDDEMTGYVATRWYRAPEIMLNWMHYNQTVDIWSVGCIMAELLTGR  143
MAPK14-202    ADLNNIVKCQKLTDDHVQFLIYQILRGLKYIHSADIIHRDLKPSNLAVNEDCELKILDFGLARHTDDEMTGYVATRWYRAPEIMLNWMHYNQTVDIWSVGCIMAELLTGR  220
MAPK14-205    ADLNNIVKCQKLTDDHVQFLIYQILRGLKYIHSADIIHRDLKPSNLAVNEDCELKILDFGLARHTDDEMTGYVATRWYRAPEIMLNWMHYNQTVDIWSV...........  132
MAPK14-206    ..............................................................................................................   90
MAPK14-201    ADLNNIVKCQKLTDDHVQFLIYQILRGLKYIHSADIIHRDLKPSNLAVNEDCELKILDFGLARHTDDEMTGYVATRWYRAPEIMLNWMHYNQTVDIWSVGCIMAELLTGR  220
MAPK14-210    ADLNNIVKCQKLTDDHVQFLIYQILRGLKYIHSADIIHRDLKPSNLAVNEDCELKFVCSSSAL...............................................  173
MAPK14-208    ADLNNIVKCQKLTDDHVQFLIYQILRGLKYIHSADIIHRDLKPSNLAVNEDCELKFVCSSSAL...............................................  173
MAPK14-203    ADLNNIVKCQKLTDDHVQFLIYQILRGLKYIHSADIIHRDLKPSNLAVNEDCELKILDFGLARHTDDEMTGYVATRWYRAPEIMLNWMHYNQTVDIWSVGCIMAELLTGR  220

logo
DORAMAPIMOD   ..............................................................................................................   17
PF00069.4     TLFPGTDHIDQLKLILRLVGTPGAELLKKISSESARNYIQSLTQMPKMNFANVFIGANPLAVDLLEKMLVLDSDKRITAAQALAHAYF......................  231
PF00069.2     TLFPGTDHIDQLKLILRLVGTPGAELLK..................................................................................  225
PF00069.1     TLFPGTDHINQLQQIMRLTGTPPAYLINRMPSHEARNYIQSLTQMPKMNFANVFIGANPLAVDLLEKMLVLDSDKRITAAQALAHAYF......................  285
PF00069.3     ..............................................................................................................  132
PF00069       TLFPGTDHIDQLKLILRLVGTPGAELLKKISSESARNYIQSLTQMPKMNFANVFIGANPLAVDLLEKMLVLDSDKRITAAQALAHAYF......................  285
PF00069.5     ..............................................................................................................  142
MAPK14-204    TLFPGTDHIDQLKLILRLVGTPGAELLKKISSESARNYIQSLTQMPKMNFANVFIGANPLAVDLLEKMLVLDSDKRITAAQALAHAYFAQYHDPDDEPVADPYDQSFESR  253
MAPK14-202    TLFPGTDHINQLQQIMRLTGTPPAYLINRMPSHEARNYIQSLTQMPKMNFANVFIGANPLAVDLLEKMLVLDSDKRITAAQALAHAYFAQYHDPDDEPVADPYDQSFESR  330
MAPK14-205    ..............................................................................................................  132
MAPK14-206    ..............................................................................................................   90
MAPK14-201    TLFPGTDHIDQLKLILRLVGTPGAELLKKISSESARNYIQSLTQMPKMNFANVFIGANPLAVDLLEKMLVLDSDKRITAAQALAHAYFAQYHDPDDEPVADPYDQSFESR  330
MAPK14-210    ..............................................................................................................  173
MAPK14-208    ..............................................................................................................  173
MAPK14-203    TLFPGTDHIDQLKLILRLVGTPGAELLKKISSESLSTCWRRCLYWTQI...RELQRPKPLHMPTLL.........................................STT  286
```

logo

```
              DLLIDEWKSLTYDEVISFVPPPLDQEEMES
DORAMAPIMOD   ..............................   17
PF00069.4     ..............................  231
PF00069.2     ..............................  225
PF00069.1     ..............................  285
PF00069.3     ..............................  132
PF00069       ..............................  285
PF00069.5     ..............................  142
MAPK14-204    DLLIDEWKSLTYDEVISFVPPPLDQEEMES  283
MAPK14-202    DLLIDEWKSLTYDEVISFVPPPLDQEEMES  360
MAPK14-205    ..............................  132
MAPK14-206    ..............................   90
MAPK14-201    DLLIDEWKSLTYDEVISFVPPPLDQEEMES  360
MAPK14-210    ..............................  173
MAPK14-208    ..............................  173
MAPK14-203    ILMMNQWPILMISPLKAGT.......SL..  307
```

X non conserved
X similar
X $\geq$ 0% conserved
X $\geq$ 50% conserved

```
logo
CHEMBL1231206  .....................................................................LG.G....V............A...................    5
PF00069        ..................................................................SQVLGLGINGKVLQIFNKRTQEKFALKMLQDCPKARREVELHWR   44
MAPKAPK2-202   MLSNSQGQSPPVPFPAPAPPPQPPTPALPHPPAQPPPPPPQQFPQFHVKSGLQIKKNAIIDDYKVTSQVLGLGINGKVLQIFNKRTQEKFALKMLQDCPKARREVELHWR  110
MAPKAPK2-201   MLSNSQGQSPPVPFPAPAPPPQPPTPALPHPPAQPPPPPPQQFPQFHVKSGLQIKKNAIIDDYKVTSQVLGLGINGKVLQIFNKRTQEKFALKMLQDCPKARREVELHWR  110

logo
CHEMBL1231206  ...........................MECLD................................................N.L............TD.............   14
PF00069        ASQCPHIVRIVDVYENLYAGRKCLLIVMECLDGGELFSRIQDRGDQAFTEREASEIMKSIGEAIQYLHSINIAHRDVKPENLLYTSKRPNAILKLTDFGFAKETTSHNSL  154
MAPKAPK2-202   ASQCPHIVRIVDVYENLYAGRKCLLIVMECLDGGELFSRIQDRGDQAFTEREASEIMKSIGEAIQYLHSINIAHRDVKPENLLYTSKRPNAILKLTDFGFAKETTSHNSL  220
MAPKAPK2-201   ASQCPHIVRIVDVYENLYAGRKCLLIVMECLDGGELFSRIQDRGDQAFTEREASEIMKSIGEAIQYLHSINIAHRDVKPENLLYTSKRPNAILKLTDFGFAKETTSHNSL  220

logo
CHEMBL1231206  ..............................................................................................................   14
PF00069        TTPCYTPYYVAPEVLGPEKYDKSCDMWSLGVIMYILLCGYPPFYSNHGLAISPGMKTRIRMGQYEFPNPEWSEVSEEVKMLIRNLLKTEPTQRMTITEFMNHPWI.....  259
MAPKAPK2-202   TTPCYTPYYVAPEVLGPEKYDKSCDMWSLGVIMYILLCGYPPFYSNHGLAISPGMKTRIRMGQYEFPNPEWSEVSEEVKMLIRNLLKTEPTQRMTITEFMNHPWIMQSTK  330
MAPKAPK2-201   TTPCYTPYYVAPEVLGPEKYDKSCDMWSLGVIMYILLCGYPPFYSNHGLAISPGMKTRIRMGQYEFPNPEWSEVSEEVKMLIRNLLKTEPTQRMTITEFMNHPWIMQSTK  330

logo
CHEMBL1231206  ......................................................................   14
PF00069        ......................................................................  259
MAPKAPK2-202   VPQTPLHTSRVLKEDKERWEDVKEEMTSALATMRVDYEQIKIKKIEDASNPLLLKRRKKARALEAAALAH  400
MAPKAPK2-201   VPQTPLHTSRVLKEDKERWEDVKGCLHDKNSDQATWLTRL..............................  370
```

X non conserved

X similar

X $\geq 0\%$ conserved

X $\geq 50\%$ conserved

```
logo
             MKSKSKSLAECFPYDKPLIMKAPAVLAPGILVLLFTLVQRSNGECKEALAKSEMNVNMKYQLPNFTAETPIQNVILHEHHIFLGATNYIYVLNEEDLQKVAEYKTGPVLE
BMS-777607   ..............................................................................................................    0
PF01403      ...........................................................................LHEHHIFLGATNYIYVLNEEDLQKVAEYKTGPVLE   35
PF07714      ..............................................................................................................    0
PF01437      ..............................................................................................................    0
PF01833.3    ..............................................................................................................    0
PF01833      ..............................................................................................................    0
PF01403.1    ...........................................................................LHEHHIFLGATNYIYVLNEEDLQKVAEYKTGPVLE   35
PF01833.2    ..............................................................................................................    0
PF01833.1    ..............................................................................................................    0
MET-205      ..............................................................................................................    0
MET-201      ...................MKAPAVLAPGILVLLFTLVQRSNGECKEALAKSEMNVNMKYQLPNFTAETPIQNVILHEHHIFLGATNYIYVLNEEDLQKVAEYKTGPVLE   91
MET-206      MKSKSKSLAECFPYDKPLIMKAPAVLAPGILVLLFTLVQRSNGECKEALAKSEMNVNMKYQLPNFTAETPIQNVILHEHHIFLGATNYIYVLNEEDLQKVAEYKTGPVLE  110
MET-203      ..............................................................................................................    0
MET-202      ...................MKAPAVLAPGILVLLFTLVQRSNGECKEALAKSEMNVNMKYQLPNFTAETPIQNVILHEHHIFLGATNYIYVLNEEDLQKVAEYKTGPVLE   91
MET-204      ...................MKAPAVLAPGILVLLFTLVQRSNGECKEALAKSEMNVNMKYQLPNFTAETPIQNVILHEHHIFLGATNYIYVLNEEDLQKVAEYKTGPVLE   91

logo
             HPDCFPCQDCSSKANLSGGVWKDNINMALVVDTYYDDQLISCGSVNRGTCQRHVFPHNHTADIQSEVHCIFSPQIEEPSQCPDCVVSALGAKVLSSVKDRFINFFVGNTI
BMS-777607   ..............................................................................................................    0
PF01403      HPDCFPCQDCSSKANLSGGVWKDNINMALVVDTYYDDQLISCGSVNRGTCQRHVFPHNHTADIQSEVHCIFSPQIEEPSQCPDCVVSALGAKVLSSVKDRFINFFVGNTI  145
PF07714      ..............................................................................................................    0
PF01437      ..............................................................................................................    0
PF01833.3    ..............................................................................................................    0
PF01833      ..............................................................................................................    0
PF01403.1    HPDCFPCQDCSSKANLSGGVWKDNINMALVVDTYYDDQLISCGSVNRGTCQRHVFPHNHTADIQSEVHCIFSPQ....................................  109
PF01833.2    ..............................................................................................................    0
PF01833.1    ..............................................................................................................    0
MET-205      ..............................................................................................................    0
MET-201      HPDCFPCQDCSSKANLSGGVWKDNINMALVVDTYYDDQLISCGSVNRGTCQRHVFPHNHTADIQSEVHCIFSPQIEEPSQCPDCVVSALGAKVLSSVKDRFINFFVGNTI  201
MET-206      HPDCFPCQDCSSKANLSGGVWKDNINMALVVDTYYDDQLISCGSVNRGTCQRHVFPHNHTADIQSEVHCIFSPQIEEPSQ..............................  190
MET-203      ..............................................................................................................    0
MET-202      HPDCFPCQDCSSKANLSGGVWKDNINMALVVDTYYDDQLISCGSVNRGTCQRHVFPHNHTADIQSEVHCIFSPQIEEPSQCPDCVVSALGAKVLSSVKDRFINFFVGNTI  201
MET-204      HPDCFPCQDCSSKANLSGGVWKDNINMALVVDTYYDDQLISCGSVNRGTCQRHVFPHNHTADIQSEVHCIFSPQIEEPSQCPDCVVSALGAKVLSSVKDRFINFFVGNTI  201

logo
             NSSYFPDHPLHSISVRRLKETKDGFMFLTDQSYIDVLPEFRDSYPIKYVHAFESNNFIYFLTVQRETLDAQTFHTRIIRFCSINSGLHSYMEMPLECILTEKRKKRSTKK
BMS-777607   ..............................................................................................................    0
PF01403      NSSYFPDHPLHSISVRRLKETKDGFMFLTDQSYIDVLPEFRDSYPIKYVHAFESNNFIYFLTVQRETLDAQTFHTRIIRFCSINSGLHSYMEMPLECILTEKRKKRSTKK  255
PF07714      ..............................................................................................................    0
PF01437      ..............................................................................................................    0
PF01833.3    ..............................................................................................................    0
PF01833      ..............................................................................................................    0
PF01403.1    ..............................................................................................................  109
PF01833.2    ..............................................................................................................    0
PF01833.1    ..............................................................................................................    0
MET-205      ..............................................................................................................    0
MET-201      NSSYFPDHPLHSISVRRLKETKDGFMFLTDQSYIDVLPEFRDSYPIKYVHAFESNNFIYFLTVQRETLDAQTFHTRIIRFCSINSGLHSYMEMPLECILTEKRKKRSTKK  311
MET-206      ..............................................................................................................  190
MET-203      ..............................................................................................................    0
MET-202      NSSYFPDHPLHSISVRRLKETKDGFMFLTDQSYIDVLPEFRDSYPIKYVHAFESNNFIYFLTVQRETLDAQTFHTRIIRFCSINSGLHSYMEMPLECILTEKRKKRSTKK  311
MET-204      NSSYFPDHPLHSISVRRLKETKDGFMFLTDQSYIDVLPEFRDSYPIKYVHAFESNNFIYFLTVQRETLDAQTFHTRIIRFCSINSGLHSYMEMPLECILTEKRKKRSTKK  311
```

```
logo
             EVFNILQAAYVSKPGAQLARQIGASLNDDILFGVFAQSKPDSAEPMDRSAMCAFPIKYVNDFFNKIVNKNNVRCLQHFYGPNHEHCFNRTLLRNSSGCEARRDEYRTEFT
BMS-777607   ..............................................................................................................   0
PF01403      EVFNILQAAYVSKPGAQLARQIGASLNDDILFGVFAQSKPDSAEPMDRSAMCAFPIKYVNDFFNKIVNKNNVRCLQHFYGPNHEHCFNRTLLRNSSGCEARRDEYRTEFT 365
PF07714      ..............................................................................................................   0
PF01437      ..............................................................................................................   0
PF01833.3    ..............................................................................................................   0
PF01833      ..............................................................................................................   0
PF01403.1    .............................................................................................................. 109
PF01833.2    ..............................................................................................................   0
PF01833.1    ..............................................................................................................   0
MET-205      ..............................................................................................................   0
MET-201      EVFNILQAAYVSKPGAQLARQIGASLNDDILFGVFAQSKPDSAEPMDRSAMCAFPIKYVNDFFNKIVNKNNVRCLQHFYGPNHEHCFNRTLLRNSSGCEARRDEYRTEFT 421
MET-206      .............................................................................................................. 190
MET-203      ..............................................................................................................   0
MET-202      EVFNILQAAYVSKPGAQLARQIGASLNDDILFGVFAQSKPDSAEPMDRSAMCAFPIKYVNDFFNKIVNKNNVRCLQHFYGPNHEHCFNRTLLRNSSGCEARRDEYRTEFT 421
MET-204      EVFNILQAAYVSKPGAQLARQIGASLNDDILFGVFAQSKPDSAEPMDRSAMCAFPIKYVNDFFNKIVNKNNVRCLQHFYGPNHEHCFNRTLLRNSSGCEARRDEYRTEFT 421

logo
             TALQRVDLFMGQFSEVLLTSISTFIKGDLTIANLGTSEGRFMQVVVSRSGPSTPHVNFLLDSHPVSPEVIVEHTLNQNGYTLVITGKKITKIPLNGLGCRHFQSCSQCLS
BMS-777607   ..............................................................................................................   0
PF01403      TALQRVDLFMGQFSEVLLTSISTFIKGDLTIANLGTSEGRFMQVVVSRSGPSTPHVNFLLDSHPVSPEVI........................................ 435
PF07714      ..............................................................................................................   0
PF01437      ..................................................................................................CRHFQSCSQCLS  12
PF01833.3    ..............................................................................................................   0
PF01833      ..............................................................................................................   0
PF01403.1    .............................................................................................................. 109
PF01833.2    ..............................................................................................................   0
PF01833.1    ..............................................................................................................   0
MET-205      ..............................................................................................................   0
MET-201      TALQRVDLFMGQFSEVLLTSISTFIKGDLTIANLGTSEGRFMQVVVSRSGPSTPHVNFLLDSHPVSPEVIVEHTLNQNGYTLVITGKKITKIPLNGLGCRHFQSCSQCLS 531
MET-206      .............................................................................................................. 190
MET-203      ..............................................................................................................   0
MET-202      TALQRVDLFMGQFSEVLLTSISTFIKGDLTIANLGTSEGRFMQVVVSRSGPSTPHVNFLLDSHPVSPEVIVEHTLNQNGYTLVITGKKITKIPLNGLGCRHFQSCSQCLS 531
MET-204      TALQRVDLFMGQFSEVLLTSISTFIKGDLTIANLGTSEGRFMQVVVSRSGPSTPHVNFLLDSHPVSPEVIVEHTLNQNGYTLVITGKKITKIPLNGLGCRHFQSCSQCLS 531

logo
             APPFVQCGWCHDKCVRSEECLSGTWTQQICLPAIYKVFPNSAPLEGGTRLTICGWDFGFRRNNKFDLKKTRVLLGNESCTLTLSESTMNTLKCTVGPAMNKHFNMSIIIS
BMS-777607   ..............................................................................................................   0
PF01403      .............................................................................................................. 435
PF07714      ..............................................................................................................   0
PF01437      APPFVQCGWCHDKCVRSEECLSGTWTQQ..................................................................................  40
PF01833.3    ...............................PAIYKVFPNSAPLEGGTRLTICGWDFGFRRNNKFDLKKTRVLLGNESCTLTLSESTMNTLKCTVGPAMNKHFNMSIIIS  79
PF01833      ...............................PAIYKVFPNSAPLEGGTRLTICGWDFGFRRNNKFDLKKTRVLLGNESCTLTLSESTMNTLKCTVGPAMNKHFNMSIIIS  79
PF01403.1    .............................................................................................................. 109
PF01833.2    ..............................................................................................................   0
PF01833.1    ...............................PAIYKVFPNSAPLEGGTRLTICGWDFGFRRNNKFDLKKTRVLLGNESCTLTLSESTMNTLKCTVGPAMNKHFNMSIIIS  79
MET-205      ..............................................................................................................   0
MET-201      APPFVQCGWCHDKCVRSEECLSGTWTQQICLPAIYKVFPNSAPLEGGTRLTICGWDFGFRRNNKFDLKKTRVLLGNESCTLTLSESTMNTLKCTVGPAMNKHFNMSIIIS 641
MET-206      .............................................................................................................. 190
MET-203      ..............................................................................................................   0
MET-202      APPFVQCGWCHDKCVRSEECLSGTWTQQICLPAIYKVFPNSAPLEGGTRLTICGWDFGFRRNNKFDLKKTRVLLGNESCTLTLSESTMNTLKCTVGPAMNKHFNMSIIIS 641
MET-204      APPFVQCGWCHDKCVRSEECLSGTWTQQICLPAIYKVFPNSAPLEGGTRLTICGWDFGFRRNNKFDLKKTRVLLGNESCTLTLSESTMNTLKCTVGPAMNKHFNMSIIIS 641
```

```
logo
             NGHGTTQYSTFSYVDPVITSISPKYGPMAGGTLLTLTGNYLNSGNSRHISIGGKTCTLKSVSNSILECYTPAQTISTEFAVKLKIDLANRETSIFSYREDPIVYEIHPTK
BMS-777607   ..............................................................................................................     0
PF01403      ..............................................................................................................   435
PF07714      ..............................................................................................................     0
PF01437      ..............................................................................................................    40
PF01833.3    NGHGTTQYSTFSY..PVITSISPKYGPMAGGTLLTLTGNYLNSGNSRHISIGGKTCTLKSVSNSILECYTPAQTISTEFAVKLKIDL.......................   164
PF01833      NGHGTTQYSTFSY..PVITSISPKYGPMAGGTLLTLTGNYLNSGNSRHISIGGKTCTLKSVSNSILECYTPAQTISTEFAVKLKIDL.......................   164
PF01403.1    ..............................................................................................................   109
PF01833.2    ....................................................................................................PIVYEIHPTK    10
PF01833.1    NGHGTTQYSTFSY..PVITSISPKYGPMAGGTLLTLTGNYLNSGNSRHISIGGKTCTLKSVSNSILECYTPAQTISTEFAVKLKIDL.............PIVYEIHPTK   174
MET-205      ..............................................................................................................     0
MET-201      NGHGTTQYSTFSYVDPVITSISPKYGPMAGGTLLTLTGNYLNSGNSRHISIGGKTCTLKSVSNSILECYTPAQTISTEFAVKLKIDLANRETSIFSYREDPIVYEIHPTK   751
MET-206      ..............................................................................................................   190
MET-203      ...............................................................................AVKLKIDLANRETSIFSYREDPIVYEIHPTK    31
MET-202      NGHGTTQYSTFSYVDPVITSISPKYGPMAGGTLLTLTGNYLNSGNSRHISIGGKTCTLKSVSNSILECYTPAQTISTEFAVKLKIDLANRETSIFSYREDPIVYEIHPTK   751
MET-204      NGHGTTQYSTFSYVDPVITSISPKYGPMAGGTLLTLTGNYLNSGNSRHISIGGKTCTLKSVSNSILECYTPAQTISTEFAVKLKIDLANRETSIFSYREDPIVYEIHPTK   751

logo
             SFISTWWKEPLNIVSFLFCFASGGSTITGVGKNLNSVSVPRMVINVHEAGRNFTVACQHRSNSEIICCTTPSLQQLNLQLPLKTKAFFMLDGILSKYFDLIYVHNPVFKP
BMS-777607   ..............................................................................................................     0
PF01403      ..............................................................................................................   435
PF07714      ..............................................................................................................     0
PF01437      ..............................................................................................................    40
PF01833.3    ..............................................................................................................   164
PF01833      ................LFCFASGGSTITGVGKNLNSVSVPRMVINVHEAGRNFTVACQHRSNSEIICCTTPSLQQLNLQLPLKT..........................   232
PF01403.1    ..............................................................................................................   109
PF01833.2    SFI..................SGGSTITGVGKNLNSVSVPRMVINVHEAGRNFTVACQHRSNSEIICCTTPSLQQLNLQLPLKT..........................    76
PF01833.1    SFI..................SGGSTITGVGKNLNSVSVPRMVINVHEAGRNFTVACQHRSNSEIICCTTPSLQQLNLQLPLKT..........................   240
MET-205      ..............................................................................................................     0
MET-201      SFISTWWKEPLNIVSFLFCFASGGSTITGVGKNLNSVSVPRMVINVHEAGRNFTVACQHRSNSEIICCTTPSLQQLNLQLPLKTKAFFMLDGILSKYFDLIYVHNPVFKP   861
MET-206      ..............................................................................................................   190
MET-203      SFI..................SGGSTITGVGKNLNSVSVPRMVINVHEAGRNFTVACQHRSNSEIICCTTPSLQQLNLQLPLKTKAFFMLDGILSKYFDLIYVHNPVFKP   123
MET-202      SFI..................SGGSTITGVGKNLNSVSVPRMVINVHEAGRNFTVACQHRSNSEIICCTTPSLQQLNLQLPLKTKAFFMLDGILSKYFDLIYVHNPVFKP   843
MET-204      SFIRHVNIALIQR.................................................................................................   764

logo
             FEKPVMISMGNENVLEIKGNDIDPEAVKGEVLKVGNKSCENIHLHSEAVLCTVPNDLLKLNSELNIEWKQAISSTVLGKVIVQPDQNFTGLIAGVVSISTALLLLLGFFL
BMS-777607   ..............................................................................................................     0
PF01403      ..............................................................................................................   435
PF07714      ..............................................................................................................     0
PF01437      ..............................................................................................................    40
PF01833.3    ..............................................................................................................   164
PF01833      ..............................................................................................................   232
PF01403.1    ..............................................................................................................   109
PF01833.2    ..............................................................................................................    76
PF01833.1    ..............................................................................................................   240
MET-205      .........................AVKGEVLKVGNKSCENIHLHSEAVLCTVPNDLLKLNSELNIEWKQAISSTVLGKVIVQPDQNFTGLIAGVVSISTALLLLLGFFL    85
MET-201      FEKPVMISMGNENVLEIKGNDIDPEAVKGEVLKVGNKSCENIHLHSEAVLCTVPNDLLKLNSELNIEWKQAISSTVLGKVIVQPDQNFTGLIAGVVSISTALLLLLGFFL   971
MET-206      ..............................................................................................................   190
MET-203      FEKPVMISMGNENVLEIKGNDIDPEAVKGEVLKVGNKSCENIHLHSEAVLCTVPNDLLKLNSELNIEVGFLHSSHDVNKEAS.VIMLFSGLK..................   214
MET-202      FEKPVMISMGNENVLEIKGNDIDPEAVKGEVLKVGNKSCENIHLHSEAVLCTVPNDLLKLNSELNIEWKQAISSTVLGKVIVQPDQNFTGLIAGVVSISTALLLLLGFFL   953
MET-204      ..............................................................................................................   764
```

```
logo
            WLKKRKQIKDLGSELVRYDARVHTPHLDRLVSARSVSPTTEMVSNESVDYRATFPEDQFPNSSQNGSCRQVQYPLTDMSPILTSGDSDISSPLLQNTVHIDLSALNPELV
BMS-777607  ..............................................................................................................     0
PF01403     ..............................................................................................................   435
PF07714     ..............................................................................................................     0
PF01437     ..............................................................................................................    40
PF01833.3   ..............................................................................................................   164
PF01833     ..............................................................................................................   232
PF01403.1   ..............................................................................................................   109
PF01833.2   ..............................................................................................................    76
PF01833.1   ..............................................................................................................   240
MET-205     WLKKRKQIK...............................................DQFPNSSQNGSCRQVQYPLTDMSPILTSGDSDISSPLLQNTVHIDLSALNPELV   148
MET-201     WLKKRKQIKDLGSELVRYDARVHTPHLDRLVSARSVSPTTEMVSNESVDYRATFPEDQFPNSSQNGSCRQVQYPLTDMSPILTSGDSDISSPLLQNTVHIDLSALNPELV  1081
MET-206     ..............................................................................................................   190
MET-203     ..............................................................................................................   214
MET-202     WLKKRKQIKDLGSELVRYDARVHTPHLDRLVSARSVSPTTEMVSNESVDYRATFPEDQFPNSSQNGSCRQVQYPLTDMSPILTSGDSDISSPLLQNTVHIDLSALNPELV  1063
MET-204     ..............................................................................................................   764

logo
            QAVQHVVIGPSSLIVHFNEVIGRGHFGCVYHGTLLDNDGKKIHCAVKSLNRITDIGEVSQFLTEGIIMKDFSHPNVLSLLGICLRSEGSPLVVLPYMKHGDLRNFIRNET
BMS-777607  ....................I.......V...............A.K.............F..E...M..F....VL..............V.LPYM.............    15
PF01403     ..............................................................................................................   435
PF07714     ...............HFNEVIGRGHFGCVYHGTLLDNDGKKIHCAVKSLNRITDIGEVSQFLTEGIIMKDFSHPNVLSLLGICLRSEGSPLVVLPYMKHGDLRNFIRNET    95
PF01437     ..............................................................................................................    40
PF01833.3   ..............................................................................................................   164
PF01833     ..............................................................................................................   232
PF01403.1   ..............................................................................................................   109
PF01833.2   ..............................................................................................................    76
PF01833.1   ..............................................................................................................   240
MET-205     QAVQHVVIGP....................................................................................................   158
MET-201     QAVQHVVIGPSSLIVHFNEVIGRGHFGCVYHGTLLDNDGKKIHCAVKSLNRITDIGEVSQFLTEGIIMKDFSHPNVLSLLGICLRSEGSPLVVLPYMKHGDLRNFIRNET  1191
MET-206     ..............................................................................................................   190
MET-203     ..............................................................................................................   214
MET-202     QAVQHVVIGPSSLIVHFNEVIGRGHFGCVYHGTLLDNDGKKIHCAVKSLNRITDIGEVSQFLTEGIIMKDFSHPNVLSLLGICLRSEGSPLVVLPYMKHGDLRNFIRNET  1173
MET-204     ..............................................................................................................   764

logo
            HNPTVKDLIGFGLQVAKGMKYLASKKFVHRDLAARNCMLDEKFTVKVADFGLARDMYDKEYYSVHNKTGAKLPVKWMALESLQTQKFTTKSDVWSFGVLLWELMTRGAPP
BMS-777607  .....................L....F.H........M........VADF............................................................    23
PF01403     ..............................................................................................................   435
PF07714     HNPTVKDLIGFGLQVAKGMKYLASKKFVHRDLAARNCMLDEKFTVKVADFGLARDMYDKEYYSVHNKTGAKLPVKWMALESLQTQKFTTKSDVWSFGVLLWELMTRGAPP   205
PF01437     ..............................................................................................................    40
PF01833.3   ..............................................................................................................   164
PF01833     ..............................................................................................................   232
PF01403.1   ..............................................................................................................   109
PF01833.2   ..............................................................................................................    76
PF01833.1   ..............................................................................................................   240
MET-205     ..............................................................................................................   158
MET-201     HNPTVKDLIGFGLQVAKGMKYLASKKFVHRDLAARNCMLDEKFTVKVADFGLARDMYDKEYYSVHNKTGAKLPVKWMALESLQTQKFTTKSDVWSFGVLLWELMTRGAPP  1301
MET-206     ..............................................................................................................   190
MET-203     ..............................................................................................................   214
MET-202     HNPTVKDLIGFGLQVAKGMKYLASKKFVHRDLAARNCMLDEKFTVKVADFGLARDMYDKEYYSVHNKTGAKLPVKWMALESLQTQKFTTKSDVWSFGVLLWELMTRGAPP  1283
MET-204     ..............................................................................................................   764
```

```
logo
              YPDVNTFDITVYLLQGRRLLQPEYCPDPLYEVMLKCWHPKAEMRPSFSELVSRISAIFSTFIGEHYVHVNATYVNVKCVAPYPSLLSSEDNADDEVDTRPASFWETS
BMS-777607    ...........................................................................................................   23
PF01403       ...........................................................................................................  435
PF07714       YPDVNTFDITVYLLQGRRLLQPEYCPDPLYEVMLKCWHPKAEMRPSFSELVSR......................................................  258
PF01437       ...........................................................................................................   40
PF01833.3     ...........................................................................................................  164
PF01833       ...........................................................................................................  232
PF01403.1     ...........................................................................................................  109
PF01833.2     ...........................................................................................................   76
PF01833.1     ...........................................................................................................  240
MET-205       ...........................................................................................................  158
MET-201       YPDVNTFDITVYLLQGRRLLQPEYCPDPLYEVMLKCWHPKAEMRPSFSELVSRISAIFSTFIGEHYVHVNATYVNVKCVAPYPSLLSSEDNADDEVDTRPASFWETS 1408
MET-206       ...........................................................................................................  190
MET-203       ...........................................................................................................  214
MET-202       YPDVNTFDITVYLLQGRRLLQPEYCPDPLYEVMLKCWHPKAEMRPSFSELVSRISAIFSTFIGEHYVHVNATYVNVKCVAPYPSLLSSEDNADDEVDTRPASFWETS 1390
MET-204       ...........................................................................................................  764
```

X non conserved
X similar
X ≥0% conserved
X ≥50% conserved

```
logo
             MKSKSKSLAECFPYDKPLIMKAPAVLAPGILVLLFTLVQRSNGECKEALAKSEMNVNMKYQLPNFTAETPIQNVILHEHHIFLGATNYIYVLNEEDLQKVAEYKTGPVLE
CRIZOTINIB   ..............................................................................................................     0
PF01403      ...........................................................................LHEHHIFLGATNYIYVLNEEDLQKVAEYKTGPVLE    35
PF07714      ..............................................................................................................     0
PF01437      ..............................................................................................................     0
PF01833.3    ..............................................................................................................     0
PF01833      ..............................................................................................................     0
PF01403.1    ...........................................................................LHEHHIFLGATNYIYVLNEEDLQKVAEYKTGPVLE    35
PF01833.2    ..............................................................................................................     0
PF01833.1    ..............................................................................................................     0
MET-205      ..............................................................................................................     0
MET-201      ...................MKAPAVLAPGILVLLFTLVQRSNGECKEALAKSEMNVNMKYQLPNFTAETPIQNVILHEHHIFLGATNYIYVLNEEDLQKVAEYKTGPVLE    91
MET-206      MKSKSKSLAECFPYDKPLIMKAPAVLAPGILVLLFTLVQRSNGECKEALAKSEMNVNMKYQLPNFTAETPIQNVILHEHHIFLGATNYIYVLNEEDLQKVAEYKTGPVLE   110
MET-203      ..............................................................................................................     0
MET-202      ...................MKAPAVLAPGILVLLFTLVQRSNGECKEALAKSEMNVNMKYQLPNFTAETPIQNVILHEHHIFLGATNYIYVLNEEDLQKVAEYKTGPVLE    91
MET-204      ...................MKAPAVLAPGILVLLFTLVQRSNGECKEALAKSEMNVNMKYQLPNFTAETPIQNVILHEHHIFLGATNYIYVLNEEDLQKVAEYKTGPVLE    91

logo
             HPDCFPCQDCSSKANLSGGVWKDNINMALVVDTYYDDQLISCGSVNRGTCQRHVFPHNHTADIQSEVHCIFSPQIEEPSQCPDCVVSALGAKVLSSVKDRFINFFVGNTI
CRIZOTINIB   ..............................................................................................................     0
PF01403      HPDCFPCQDCSSKANLSGGVWKDNINMALVVDTYYDDQLISCGSVNRGTCQRHVFPHNHTADIQSEVHCIFSPQIEEPSQCPDCVVSALGAKVLSSVKDRFINFFVGNTI   145
PF07714      ..............................................................................................................     0
PF01437      ..............................................................................................................     0
PF01833.3    ..............................................................................................................     0
PF01833      ..............................................................................................................     0
PF01403.1    HPDCFPCQDCSSKANLSGGVWKDNINMALVVDTYYDDQLISCGSVNRGTCQRHVFPHNHTADIQSEVHCIFSPQ....................................   109
PF01833.2    ..............................................................................................................     0
PF01833.1    ..............................................................................................................     0
MET-205      ..............................................................................................................     0
MET-201      HPDCFPCQDCSSKANLSGGVWKDNINMALVVDTYYDDQLISCGSVNRGTCQRHVFPHNHTADIQSEVHCIFSPQIEEPSQCPDCVVSALGAKVLSSVKDRFINFFVGNTI   201
MET-206      HPDCFPCQDCSSKANLSGGVWKDNINMALVVDTYYDDQLISCGSVNRGTCQRHVFPHNHTADIQSEVHCIFSPQIEEPSQ..............................   190
MET-203      ..............................................................................................................     0
MET-202      HPDCFPCQDCSSKANLSGGVWKDNINMALVVDTYYDDQLISCGSVNRGTCQRHVFPHNHTADIQSEVHCIFSPQIEEPSQCPDCVVSALGAKVLSSVKDRFINFFVGNTI   201
MET-204      HPDCFPCQDCSSKANLSGGVWKDNINMALVVDTYYDDQLISCGSVNRGTCQRHVFPHNHTADIQSEVHCIFSPQIEEPSQCPDCVVSALGAKVLSSVKDRFINFFVGNTI   201

logo
             NSSYFPDHPLHSISVRRLKETKDGFMFLTDQSYIDVLPEFRDSYPIKYVHAFESNNFIYFLTVQRETLDAQTFHTRIIRFCSINSGLHSYMEMPLECILTEKRKKRSTKK
CRIZOTINIB   ..............................................................................................................     0
PF01403      NSSYFPDHPLHSISVRRLKETKDGFMFLTDQSYIDVLPEFRDSYPIKYVHAFESNNFIYFLTVQRETLDAQTFHTRIIRFCSINSGLHSYMEMPLECILTEKRKKRSTKK   255
PF07714      ..............................................................................................................     0
PF01437      ..............................................................................................................     0
PF01833.3    ..............................................................................................................     0
PF01833      ..............................................................................................................     0
PF01403.1    ..............................................................................................................   109
PF01833.2    ..............................................................................................................     0
PF01833.1    ..............................................................................................................     0
MET-205      ..............................................................................................................     0
MET-201      NSSYFPDHPLHSISVRRLKETKDGFMFLTDQSYIDVLPEFRDSYPIKYVHAFESNNFIYFLTVQRETLDAQTFHTRIIRFCSINSGLHSYMEMPLECILTEKRKKRSTKK   311
MET-206      ..............................................................................................................   190
MET-203      ..............................................................................................................     0
MET-202      NSSYFPDHPLHSISVRRLKETKDGFMFLTDQSYIDVLPEFRDSYPIKYVHAFESNNFIYFLTVQRETLDAQTFHTRIIRFCSINSGLHSYMEMPLECILTEKRKKRSTKK   311
MET-204      NSSYFPDHPLHSISVRRLKETKDGFMFLTDQSYIDVLPEFRDSYPIKYVHAFESNNFIYFLTVQRETLDAQTFHTRIIRFCSINSGLHSYMEMPLECILTEKRKKRSTKK   311
```

```
logo
            EVFNILQAAYVSKPGAQLARQIGASLNDDILFGVFAQSKPDSAEPMDRSAMCAFPIKYVNDFFNKIVNKNNVRCLQHFYGPNHEHCFNRTLLRNSSGCEARRDEYRTEFT
CRIZOTINIB  ..............................................................................................................   0
PF01403     EVFNILQAAYVSKPGAQLARQIGASLNDDILFGVFAQSKPDSAEPMDRSAMCAFPIKYVNDFFNKIVNKNNVRCLQHFYGPNHEHCFNRTLLRNSSGCEARRDEYRTEFT 365
PF07714     ..............................................................................................................   0
PF01437     ..............................................................................................................   0
PF01833.3   ..............................................................................................................   0
PF01833     ..............................................................................................................   0
PF01403.1   .............................................................................................................. 109
PF01833.2   ..............................................................................................................   0
PF01833.1   ..............................................................................................................   0
MET-205     ..............................................................................................................   0
MET-201     EVFNILQAAYVSKPGAQLARQIGASLNDDILFGVFAQSKPDSAEPMDRSAMCAFPIKYVNDFFNKIVNKNNVRCLQHFYGPNHEHCFNRTLLRNSSGCEARRDEYRTEFT 421
MET-206     .............................................................................................................. 190
MET-203     ..............................................................................................................   0
MET-202     EVFNILQAAYVSKPGAQLARQIGASLNDDILFGVFAQSKPDSAEPMDRSAMCAFPIKYVNDFFNKIVNKNNVRCLQHFYGPNHEHCFNRTLLRNSSGCEARRDEYRTEFT 421
MET-204     EVFNILQAAYVSKPGAQLARQIGASLNDDILFGVFAQSKPDSAEPMDRSAMCAFPIKYVNDFFNKIVNKNNVRCLQHFYGPNHEHCFNRTLLRNSSGCEARRDEYRTEFT 421
```

```
logo
            TALQRVDLFMGQFSEVLLTSISTFIKGDLTIANLGTSEGRFMQVVVSRSGPSTPHVNFLLDSHPVSPEVIVEHTLNQNGYTLVITGKKITKIPLNGLGCRHFQSCSQCLS
CRIZOTINIB  ..............................................................................................................   0
PF01403     TALQRVDLFMGQFSEVLLTSISTFIKGDLTIANLGTSEGRFMQVVVSRSGPSTPHVNFLLDSHPVSPEVI........................................ 435
PF07714     ..............................................................................................................   0
PF01437     ..................................................................................................CRHFQSCSQCLS  12
PF01833.3   ..............................................................................................................   0
PF01833     ..............................................................................................................   0
PF01403.1   .............................................................................................................. 109
PF01833.2   ..............................................................................................................   0
PF01833.1   ..............................................................................................................   0
MET-205     ..............................................................................................................   0
MET-201     TALQRVDLFMGQFSEVLLTSISTFIKGDLTIANLGTSEGRFMQVVVSRSGPSTPHVNFLLDSHPVSPEVIVEHTLNQNGYTLVITGKKITKIPLNGLGCRHFQSCSQCLS 531
MET-206     .............................................................................................................. 190
MET-203     ..............................................................................................................   0
MET-202     TALQRVDLFMGQFSEVLLTSISTFIKGDLTIANLGTSEGRFMQVVVSRSGPSTPHVNFLLDSHPVSPEVIVEHTLNQNGYTLVITGKKITKIPLNGLGCRHFQSCSQCLS 531
MET-204     TALQRVDLFMGQFSEVLLTSISTFIKGDLTIANLGTSEGRFMQVVVSRSGPSTPHVNFLLDSHPVSPEVIVEHTLNQNGYTLVITGKKITKIPLNGLGCRHFQSCSQCLS 531
```

```
logo
            APPFVQCGWCHDKCVRSEECLSGTWTQQICLPAIYKVFPNSAPLEGGTRLTICGWDFGFRRNNKFDLKKTRVLLGNESCTLTLSESTMNTLKCTVGPAMNKHFNMSIIIS
CRIZOTINIB  ..............................................................................................................   0
PF01403     .............................................................................................................. 435
PF07714     ..............................................................................................................   0
PF01437     APPFVQCGWCHDKCVRSEECLSGTWTQQ..................................................................................  40
PF01833.3   ...............................PAIYKVFPNSAPLEGGTRLTICGWDFGFRRNNKFDLKKTRVLLGNESCTLTLSESTMNTLKCTVGPAMNKHFNMSIIIS  79
PF01833     ...............................PAIYKVFPNSAPLEGGTRLTICGWDFGFRRNNKFDLKKTRVLLGNESCTLTLSESTMNTLKCTVGPAMNKHFNMSIIIS  79
PF01403.1   .............................................................................................................. 109
PF01833.2   ..............................................................................................................   0
PF01833.1   ...............................PAIYKVFPNSAPLEGGTRLTICGWDFGFRRNNKFDLKKTRVLLGNESCTLTLSESTMNTLKCTVGPAMNKHFNMSIIIS  79
MET-205     ..............................................................................................................   0
MET-201     APPFVQCGWCHDKCVRSEECLSGTWTQQICLPAIYKVFPNSAPLEGGTRLTICGWDFGFRRNNKFDLKKTRVLLGNESCTLTLSESTMNTLKCTVGPAMNKHFNMSIIIS 641
MET-206     .............................................................................................................. 190
MET-203     ..............................................................................................................   0
MET-202     APPFVQCGWCHDKCVRSEECLSGTWTQQICLPAIYKVFPNSAPLEGGTRLTICGWDFGFRRNNKFDLKKTRVLLGNESCTLTLSESTMNTLKCTVGPAMNKHFNMSIIIS 641
MET-204     APPFVQCGWCHDKCVRSEECLSGTWTQQICLPAIYKVFPNSAPLEGGTRLTICGWDFGFRRNNKFDLKKTRVLLGNESCTLTLSESTMNTLKCTVGPAMNKHFNMSIIIS 641
```

logo

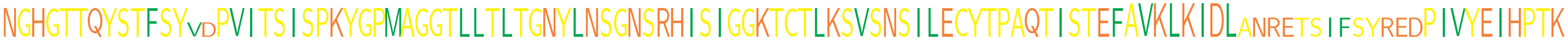

```
CRIZOTINIB   ..............................................................................................................    0
PF01403      ..............................................................................................................  435
PF07714      ..............................................................................................................    0
PF01437      ..............................................................................................................   40
PF01833.3    NGHGTTQYSTFSY..PVITSISPKYGPMAGGTLLTLTGNYLNSGNSRHISIGGKTCTLKSVSNSILECYTPAQTISTEFAVKLKIDL.......................  164
PF01833      NGHGTTQYSTFSY..PVITSISPKYGPMAGGTLLTLTGNYLNSGNSRHISIGGKTCTLKSVSNSILECYTPAQTISTEFAVKLKIDL.......................  164
PF01403.1    ..............................................................................................................  109
PF01833.2    ....................................................................................................PIVYEIHPTK   10
PF01833.1    NGHGTTQYSTFSY..PVITSISPKYGPMAGGTLLTLTGNYLNSGNSRHISIGGKTCTLKSVSNSILECYTPAQTISTEFAVKLKIDL.............PIVYEIHPTK  174
MET-205      ..............................................................................................................    0
MET-201      NGHGTTQYSTFSYVDPVITSISPKYGPMAGGTLLTLTGNYLNSGNSRHISIGGKTCTLKSVSNSILECYTPAQTISTEFAVKLKIDLANRETSIFSYREDPIVYEIHPTK  751
MET-206      ..............................................................................................................  190
MET-203      ...............................................................................AVKLKIDLANRETSIFSYREDPIVYEIHPTK   31
MET-202      NGHGTTQYSTFSYVDPVITSISPKYGPMAGGTLLTLTGNYLNSGNSRHISIGGKTCTLKSVSNSILECYTPAQTISTEFAVKLKIDLANRETSIFSYREDPIVYEIHPTK  751
MET-204      NGHGTTQYSTFSYVDPVITSISPKYGPMAGGTLLTLTGNYLNSGNSRHISIGGKTCTLKSVSNSILECYTPAQTISTEFAVKLKIDLANRETSIFSYREDPIVYEIHPTK  751
```

logo

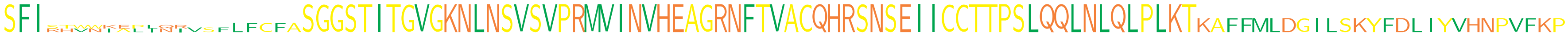

```
CRIZOTINIB   ..............................................................................................................    0
PF01403      ..............................................................................................................  435
PF07714      ..............................................................................................................    0
PF01437      ..............................................................................................................   40
PF01833.3    ..............................................................................................................  164
PF01833      ................LFCFASGGSTITGVGKNLNSVSVPRMVINVHEAGRNFTVACQHRSNSEIICCTTPSLQQLNLQLPLKT..........................  232
PF01403.1    ..............................................................................................................  109
PF01833.2    SFI..................SGGSTITGVGKNLNSVSVPRMVINVHEAGRNFTVACQHRSNSEIICCTTPSLQQLNLQLPLKT..........................   76
PF01833.1    SFI..................SGGSTITGVGKNLNSVSVPRMVINVHEAGRNFTVACQHRSNSEIICCTTPSLQQLNLQLPLKT..........................  240
MET-205      ..............................................................................................................    0
MET-201      SFISTWWKEPLNIVSFLFCFASGGSTITGVGKNLNSVSVPRMVINVHEAGRNFTVACQHRSNSEIICCTTPSLQQLNLQLPLKTKAFFMLDGILSKYFDLIYVHNPVFKP  861
MET-206      ..............................................................................................................  190
MET-203      SFI..................SGGSTITGVGKNLNSVSVPRMVINVHEAGRNFTVACQHRSNSEIICCTTPSLQQLNLQLPLKTKAFFMLDGILSKYFDLIYVHNPVFKP  123
MET-202      SFI..................SGGSTITGVGKNLNSVSVPRMVINVHEAGRNFTVACQHRSNSEIICCTTPSLQQLNLQLPLKTKAFFMLDGILSKYFDLIYVHNPVFKP  843
MET-204      SFIRHVNIALIQR.................................................................................................  764
```

logo

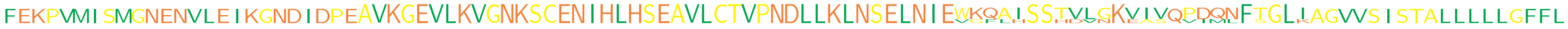

```
CRIZOTINIB   ..............................................................................................................    0
PF01403      ..............................................................................................................  435
PF07714      ..............................................................................................................    0
PF01437      ..............................................................................................................   40
PF01833.3    ..............................................................................................................  164
PF01833      ..............................................................................................................  232
PF01403.1    ..............................................................................................................  109
PF01833.2    ..............................................................................................................   76
PF01833.1    ..............................................................................................................  240
MET-205      .........................AVKGEVLKVGNKSCENIHLHSEAVLCTVPNDLLKLNSELNIEWKQAISSTVLGKVIVQPDQNFTGLIAGVVSISTALLLLLGFFL   85
MET-201      FEKPVMISMGNENVLEIKGNDIDPEAVKGEVLKVGNKSCENIHLHSEAVLCTVPNDLLKLNSELNIEWKQAISSTVLGKVIVQPDQNFTGLIAGVVSISTALLLLLGFFL  971
MET-206      ..............................................................................................................  190
MET-203      FEKPVMISMGNENVLEIKGNDIDPEAVKGEVLKVGNKSCENIHLHSEAVLCTVPNDLLKLNSELNIEVGFLHSSHDVNKEAS.VIMLFSGLK..................  214
MET-202      FEKPVMISMGNENVLEIKGNDIDPEAVKGEVLKVGNKSCENIHLHSEAVLCTVPNDLLKLNSELNIEWKQAISSTVLGKVIVQPDQNFTGLIAGVVSISTALLLLLGFFL  953
MET-204      ..............................................................................................................  764
```

```
logo
             WLKKRKQIKDLGSELVRYDARVHTPHLDRLVSARSVSPTTEMVSNESVDYRATFPEDQFPNSSQNGSCRQVQYPLTDMSPILTSGDSDISSPLLQNTVHIDLSALNPELV
CRIZOTINIB   ..............................................................................................................    0
PF01403      ..............................................................................................................  435
PF07714      ..............................................................................................................    0
PF01437      ..............................................................................................................   40
PF01833.3    ..............................................................................................................  164
PF01833      ..............................................................................................................  232
PF01403.1    ..............................................................................................................  109
PF01833.2    ..............................................................................................................   76
PF01833.1    ..............................................................................................................  240
MET-205      WLKKRKQIK...............................................DQFPNSSQNGSCRQVQYPLTDMSPILTSGDSDISSPLLQNTVHIDLSALNPELV  148
MET-201      WLKKRKQIKDLGSELVRYDARVHTPHLDRLVSARSVSPTTEMVSNESVDYRATFPEDQFPNSSQNGSCRQVQYPLTDMSPILTSGDSDISSPLLQNTVHIDLSALNPELV 1081
MET-206      ..............................................................................................................  190
MET-203      ..............................................................................................................  214
MET-202      WLKKRKQIKDLGSELVRYDARVHTPHLDRLVSARSVSPTTEMVSNESVDYRATFPEDQFPNSSQNGSCRQVQYPLTDMSPILTSGDSDISSPLLQNTVHIDLSALNPELV 1063
MET-204      ..............................................................................................................  764

logo
             QAVQHVVIGPSSLIVHFNEVIGRGHFGCVYHTGLLDNDGKKIHCAVKSLNRITDIGEVSQFLTEGIIMKDFSHPNVLSLLGICLRSEGSPLVVLPYMKHGDLRNFIRNET
CRIZOTINIB   ....................IG......V...............A...............................L................LPYM..G..........   10
PF01403      ..............................................................................................................  435
PF07714      ...............HFNEVIGRGHFGCVYHTGLLDNDGKKIHCAVKSLNRITDIGEVSQFLTEGIIMKDFSHPNVLSLLGICLRSEGSPLVVLPYMKHGDLRNFIRNET   95
PF01437      ..............................................................................................................   40
PF01833.3    ..............................................................................................................  164
PF01833      ..............................................................................................................  232
PF01403.1    ..............................................................................................................  109
PF01833.2    ..............................................................................................................   76
PF01833.1    ..............................................................................................................  240
MET-205      QAVQHVVIGP....................................................................................................  158
MET-201      QAVQHVVIGPSSLIVHFNEVIGRGHFGCVYHTGLLDNDGKKIHCAVKSLNRITDIGEVSQFLTEGIIMKDFSHPNVLSLLGICLRSEGSPLVVLPYMKHGDLRNFIRNET 1191
MET-206      ..............................................................................................................  190
MET-203      ..............................................................................................................  214
MET-202      QAVQHVVIGPSSLIVHFNEVIGRGHFGCVYHTGLLDNDGKKIHCAVKSLNRITDIGEVSQFLTEGIIMKDFSHPNVLSLLGICLRSEGSPLVVLPYMKHGDLRNFIRNET 1173
MET-204      ..............................................................................................................  764

logo
             HNPTVKDLIGFGLQVAKGMKYLASKKFVHRDLAARNCMLDEKFTVKVADFGLARDMYDKEYYSVHNKTGAKLPVKWMALESLQTQKFTTKSDVWSFGVLLWELMTRGAPP
CRIZOTINIB   ..................................R..M.........AD.......Y.....................................................   15
PF01403      ..............................................................................................................  435
PF07714      HNPTVKDLIGFGLQVAKGMKYLASKKFVHRDLAARNCMLDEKFTVKVADFGLARDMYDKEYYSVHNKTGAKLPVKWMALESLQTQKFTTKSDVWSFGVLLWELMTRGAPP  205
PF01437      ..............................................................................................................   40
PF01833.3    ..............................................................................................................  164
PF01833      ..............................................................................................................  232
PF01403.1    ..............................................................................................................  109
PF01833.2    ..............................................................................................................   76
PF01833.1    ..............................................................................................................  240
MET-205      ..............................................................................................................  158
MET-201      HNPTVKDLIGFGLQVAKGMKYLASKKFVHRDLAARNCMLDEKFTVKVADFGLARDMYDKEYYSVHNKTGAKLPVKWMALESLQTQKFTTKSDVWSFGVLLWELMTRGAPP 1301
MET-206      ..............................................................................................................  190
MET-203      ..............................................................................................................  214
MET-202      HNPTVKDLIGFGLQVAKGMKYLASKKFVHRDLAARNCMLDEKFTVKVADFGLARDMYDKEYYSVHNKTGAKLPVKWMALESLQTQKFTTKSDVWSFGVLLWELMTRGAPP 1283
MET-204      ..............................................................................................................  764
```

logo

```
            YPDVNTFDITVYLLQGRRLLQPEYCPDPLYEVMLKCWHPKAEMRPSFSELVSRISAIFSTFIGEHYVHVNATYVNVKCVAPYPSLLSSEDNADDEVDTRPASFWETS
CRIZOTINIB  ...........................................................................................................   15
PF01403     ...........................................................................................................  435
PF07714     YPDVNTFDITVYLLQGRRLLQPEYCPDPLYEVMLKCWHPKAEMRPSFSELVSR......................................................  258
PF01437     ...........................................................................................................   40
PF01833.3   ...........................................................................................................  164
PF01833     ...........................................................................................................  232
PF01403.1   ...........................................................................................................  109
PF01833.2   ...........................................................................................................   76
PF01833.1   ...........................................................................................................  240
MET-205     ...........................................................................................................  158
MET-201     YPDVNTFDITVYLLQGRRLLQPEYCPDPLYEVMLKCWHPKAEMRPSFSELVSRISAIFSTFIGEHYVHVNATYVNVKCVAPYPSLLSSEDNADDEVDTRPASFWETS 1408
MET-206     ...........................................................................................................  190
MET-203     ...........................................................................................................  214
MET-202     YPDVNTFDITVYLLQGRRLLQPEYCPDPLYEVMLKCWHPKAEMRPSFSELVSRISAIFSTFIGEHYVHVNATYVNVKCVAPYPSLLSSEDNADDEVDTRPASFWETS 1390
MET-204     ...........................................................................................................  764
```

X non conserved
X similar
X ≥ 0% conserved
X ≥ 50% conserved

```
logo
             MKSKSKSLAECFPYDKPLIMKAPAVLAPGILVLLFTLVQRSNGECKEALAKSEMNVNMKYQLPNFTAETPIQNVILHEHHIFLGATNYIYVLNEEDLQKVAEYKTGPVLE
FORETINIB    ..............................................................................................................    0
PF01403      ...........................................................................LHEHHIFLGATNYIYVLNEEDLQKVAEYKTGPVLE   35
PF07714      ..............................................................................................................    0
PF01437      ..............................................................................................................    0
PF01833.3    ..............................................................................................................    0
PF01833      ..............................................................................................................    0
PF01403.1    ...........................................................................LHEHHIFLGATNYIYVLNEEDLQKVAEYKTGPVLE   35
PF01833.2    ..............................................................................................................    0
PF01833.1    ..............................................................................................................    0
MET-205      ..............................................................................................................    0
MET-201      ...................MKAPAVLAPGILVLLFTLVQRSNGECKEALAKSEMNVNMKYQLPNFTAETPIQNVILHEHHIFLGATNYIYVLNEEDLQKVAEYKTGPVLE   91
MET-206      MKSKSKSLAECFPYDKPLIMKAPAVLAPGILVLLFTLVQRSNGECKEALAKSEMNVNMKYQLPNFTAETPIQNVILHEHHIFLGATNYIYVLNEEDLQKVAEYKTGPVLE  110
MET-203      ..............................................................................................................    0
MET-202      ...................MKAPAVLAPGILVLLFTLVQRSNGECKEALAKSEMNVNMKYQLPNFTAETPIQNVILHEHHIFLGATNYIYVLNEEDLQKVAEYKTGPVLE   91
MET-204      ...................MKAPAVLAPGILVLLFTLVQRSNGECKEALAKSEMNVNMKYQLPNFTAETPIQNVILHEHHIFLGATNYIYVLNEEDLQKVAEYKTGPVLE   91

logo
             HPDCFPCQDCSSKANLSGGVWKDNINMALVVDTYYDDQLISCGSVNRGTCQRHVFPHNHTADIQSEVHCIFSPQIEEPSQCPDCVVSALGAKVLSSVKDRFINFFVGNTI
FORETINIB    ..............................................................................................................    0
PF01403      HPDCFPCQDCSSKANLSGGVWKDNINMALVVDTYYDDQLISCGSVNRGTCQRHVFPHNHTADIQSEVHCIFSPQIEEPSQCPDCVVSALGAKVLSSVKDRFINFFVGNTI  145
PF07714      ..............................................................................................................    0
PF01437      ..............................................................................................................    0
PF01833.3    ..............................................................................................................    0
PF01833      ..............................................................................................................    0
PF01403.1    HPDCFPCQDCSSKANLSGGVWKDNINMALVVDTYYDDQLISCGSVNRGTCQRHVFPHNHTADIQSEVHCIFSPQ....................................  109
PF01833.2    ..............................................................................................................    0
PF01833.1    ..............................................................................................................    0
MET-205      ..............................................................................................................    0
MET-201      HPDCFPCQDCSSKANLSGGVWKDNINMALVVDTYYDDQLISCGSVNRGTCQRHVFPHNHTADIQSEVHCIFSPQIEEPSQCPDCVVSALGAKVLSSVKDRFINFFVGNTI  201
MET-206      HPDCFPCQDCSSKANLSGGVWKDNINMALVVDTYYDDQLISCGSVNRGTCQRHVFPHNHTADIQSEVHCIFSPQIEEPSQ..............................  190
MET-203      ..............................................................................................................    0
MET-202      HPDCFPCQDCSSKANLSGGVWKDNINMALVVDTYYDDQLISCGSVNRGTCQRHVFPHNHTADIQSEVHCIFSPQIEEPSQCPDCVVSALGAKVLSSVKDRFINFFVGNTI  201
MET-204      HPDCFPCQDCSSKANLSGGVWKDNINMALVVDTYYDDQLISCGSVNRGTCQRHVFPHNHTADIQSEVHCIFSPQIEEPSQCPDCVVSALGAKVLSSVKDRFINFFVGNTI  201

logo
             NSSYFPDHPLHSISVRRLKETKDGFMFLTDQSYIDVLPEFRDSYPIKYVHAFESNNFIYFLTVQRETLDAQTFHTRIIRFCSINSGLHSYMEMPLECILTEKRKKRSTKK
FORETINIB    ..............................................................................................................    0
PF01403      NSSYFPDHPLHSISVRRLKETKDGFMFLTDQSYIDVLPEFRDSYPIKYVHAFESNNFIYFLTVQRETLDAQTFHTRIIRFCSINSGLHSYMEMPLECILTEKRKKRSTKK  255
PF07714      ..............................................................................................................    0
PF01437      ..............................................................................................................    0
PF01833.3    ..............................................................................................................    0
PF01833      ..............................................................................................................    0
PF01403.1    ..............................................................................................................  109
PF01833.2    ..............................................................................................................    0
PF01833.1    ..............................................................................................................    0
MET-205      ..............................................................................................................    0
MET-201      NSSYFPDHPLHSISVRRLKETKDGFMFLTDQSYIDVLPEFRDSYPIKYVHAFESNNFIYFLTVQRETLDAQTFHTRIIRFCSINSGLHSYMEMPLECILTEKRKKRSTKK  311
MET-206      ..............................................................................................................  190
MET-203      ..............................................................................................................    0
MET-202      NSSYFPDHPLHSISVRRLKETKDGFMFLTDQSYIDVLPEFRDSYPIKYVHAFESNNFIYFLTVQRETLDAQTFHTRIIRFCSINSGLHSYMEMPLECILTEKRKKRSTKK  311
MET-204      NSSYFPDHPLHSISVRRLKETKDGFMFLTDQSYIDVLPEFRDSYPIKYVHAFESNNFIYFLTVQRETLDAQTFHTRIIRFCSINSGLHSYMEMPLECILTEKRKKRSTKK  311
```

```
logo
             EVFNILQAAYVSKPGAQLARQIGASLNDDILFGVFAQSKPDSAEPMDRSAMCAFPIKYVNDFFNKIVNKNNVRCLQHFYGPNHEHCFNRTLLRNSSGCEARRDEYRTEFT
FORETINIB    ..............................................................................................................    0
PF01403      EVFNILQAAYVSKPGAQLARQIGASLNDDILFGVFAQSKPDSAEPMDRSAMCAFPIKYVNDFFNKIVNKNNVRCLQHFYGPNHEHCFNRTLLRNSSGCEARRDEYRTEFT  365
PF07714      ..............................................................................................................    0
PF01437      ..............................................................................................................    0
PF01833.3    ..............................................................................................................    0
PF01833      ..............................................................................................................    0
PF01403.1    ..............................................................................................................  109
PF01833.2    ..............................................................................................................    0
PF01833.1    ..............................................................................................................    0
MET-205      ..............................................................................................................    0
MET-201      EVFNILQAAYVSKPGAQLARQIGASLNDDILFGVFAQSKPDSAEPMDRSAMCAFPIKYVNDFFNKIVNKNNVRCLQHFYGPNHEHCFNRTLLRNSSGCEARRDEYRTEFT  421
MET-206      ..............................................................................................................  190
MET-203      ..............................................................................................................    0
MET-202      EVFNILQAAYVSKPGAQLARQIGASLNDDILFGVFAQSKPDSAEPMDRSAMCAFPIKYVNDFFNKIVNKNNVRCLQHFYGPNHEHCFNRTLLRNSSGCEARRDEYRTEFT  421
MET-204      EVFNILQAAYVSKPGAQLARQIGASLNDDILFGVFAQSKPDSAEPMDRSAMCAFPIKYVNDFFNKIVNKNNVRCLQHFYGPNHEHCFNRTLLRNSSGCEARRDEYRTEFT  421

logo
             TALQRVDLFMGQFSEVLLTSISTFIKGDLTIANLGTSEGRFMQVVVSRSGPSTPHVNFLLDSHPVSPEVIVEHTLNQNGYTLVITGKKITKIPLNGLGCRHFQSCSQCLS
FORETINIB    ..............................................................................................................    0
PF01403      TALQRVDLFMGQFSEVLLTSISTFIKGDLTIANLGTSEGRFMQVVVSRSGPSTPHVNFLLDSHPVSPEVI........................................  435
PF07714      ..............................................................................................................    0
PF01437      ..................................................................................................CRHFQSCSQCLS   12
PF01833.3    ..............................................................................................................    0
PF01833      ..............................................................................................................    0
PF01403.1    ..............................................................................................................  109
PF01833.2    ..............................................................................................................    0
PF01833.1    ..............................................................................................................    0
MET-205      ..............................................................................................................    0
MET-201      TALQRVDLFMGQFSEVLLTSISTFIKGDLTIANLGTSEGRFMQVVVSRSGPSTPHVNFLLDSHPVSPEVIVEHTLNQNGYTLVITGKKITKIPLNGLGCRHFQSCSQCLS  531
MET-206      ..............................................................................................................  190
MET-203      ..............................................................................................................    0
MET-202      TALQRVDLFMGQFSEVLLTSISTFIKGDLTIANLGTSEGRFMQVVVSRSGPSTPHVNFLLDSHPVSPEVIVEHTLNQNGYTLVITGKKITKIPLNGLGCRHFQSCSQCLS  531
MET-204      TALQRVDLFMGQFSEVLLTSISTFIKGDLTIANLGTSEGRFMQVVVSRSGPSTPHVNFLLDSHPVSPEVIVEHTLNQNGYTLVITGKKITKIPLNGLGCRHFQSCSQCLS  531

logo
             APPFVQCGWCHDKCVRSEECLSGTWTQQICLPAIYKVFPNSAPLEGGTRLTICGWDFGFRRNNKFDLKKTRVLLGNESCTLTLSESTMNTLKCTVGPAMNKHFNMSIIIS
FORETINIB    ..............................................................................................................    0
PF01403      ..............................................................................................................  435
PF07714      ..............................................................................................................    0
PF01437      APPFVQCGWCHDKCVRSEECLSGTWTQQ..................................................................................   40
PF01833.3    ...............................PAIYKVFPNSAPLEGGTRLTICGWDFGFRRNNKFDLKKTRVLLGNESCTLTLSESTMNTLKCTVGPAMNKHFNMSIIIS   79
PF01833      ...............................PAIYKVFPNSAPLEGGTRLTICGWDFGFRRNNKFDLKKTRVLLGNESCTLTLSESTMNTLKCTVGPAMNKHFNMSIIIS   79
PF01403.1    ..............................................................................................................  109
PF01833.2    ..............................................................................................................    0
PF01833.1    ...............................PAIYKVFPNSAPLEGGTRLTICGWDFGFRRNNKFDLKKTRVLLGNESCTLTLSESTMNTLKCTVGPAMNKHFNMSIIIS   79
MET-205      ..............................................................................................................    0
MET-201      APPFVQCGWCHDKCVRSEECLSGTWTQQICLPAIYKVFPNSAPLEGGTRLTICGWDFGFRRNNKFDLKKTRVLLGNESCTLTLSESTMNTLKCTVGPAMNKHFNMSIIIS  641
MET-206      ..............................................................................................................  190
MET-203      ..............................................................................................................    0
MET-202      APPFVQCGWCHDKCVRSEECLSGTWTQQICLPAIYKVFPNSAPLEGGTRLTICGWDFGFRRNNKFDLKKTRVLLGNESCTLTLSESTMNTLKCTVGPAMNKHFNMSIIIS  641
MET-204      APPFVQCGWCHDKCVRSEECLSGTWTQQICLPAIYKVFPNSAPLEGGTRLTICGWDFGFRRNNKFDLKKTRVLLGNESCTLTLSESTMNTLKCTVGPAMNKHFNMSIIIS  641
```

logo
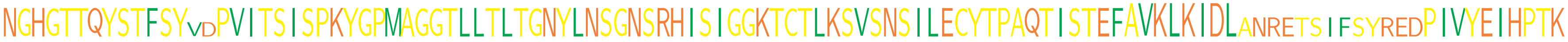

```
FORETINIB   ..............................................................................................................    0
PF01403     ..............................................................................................................  435
PF07714     ..............................................................................................................    0
PF01437     ..............................................................................................................   40
PF01833.3   NGHGTTQYSTFSY..PVITSISPKYGPMAGGTLLTLTGNYLNSGNSRHISIGGKTCTLKSVSNSILECYTPAQTISTEFAVKLKIDL.......................  164
PF01833     NGHGTTQYSTFSY..PVITSISPKYGPMAGGTLLTLTGNYLNSGNSRHISIGGKTCTLKSVSNSILECYTPAQTISTEFAVKLKIDL.......................  164
PF01403.1   ..............................................................................................................  109
PF01833.2   ....................................................................................................PIVYEIHPTK   10
PF01833.1   NGHGTTQYSTFSY..PVITSISPKYGPMAGGTLLTLTGNYLNSGNSRHISIGGKTCTLKSVSNSILECYTPAQTISTEFAVKLKIDL.............PIVYEIHPTK  174
MET-205     ..............................................................................................................    0
MET-201     NGHGTTQYSTFSYVDPVITSISPKYGPMAGGTLLTLTGNYLNSGNSRHISIGGKTCTLKSVSNSILECYTPAQTISTEFAVKLKIDLANRETSIFSYREDPIVYEIHPTK  751
MET-206     ..............................................................................................................  190
MET-203     ...............................................................................AVKLKIDLANRETSIFSYREDPIVYEIHPTK   31
MET-202     NGHGTTQYSTFSYVDPVITSISPKYGPMAGGTLLTLTGNYLNSGNSRHISIGGKTCTLKSVSNSILECYTPAQTISTEFAVKLKIDLANRETSIFSYREDPIVYEIHPTK  751
MET-204     NGHGTTQYSTFSYVDPVITSISPKYGPMAGGTLLTLTGNYLNSGNSRHISIGGKTCTLKSVSNSILECYTPAQTISTEFAVKLKIDLANRETSIFSYREDPIVYEIHPTK  751
```

logo
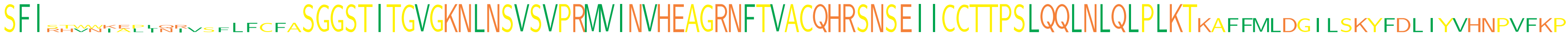

```
FORETINIB   ..............................................................................................................    0
PF01403     ..............................................................................................................  435
PF07714     ..............................................................................................................    0
PF01437     ..............................................................................................................   40
PF01833.3   ..............................................................................................................  164
PF01833     ................LFCFASGGSTITGVGKNLNSVSVPRMVINVHEAGRNFTVACQHRSNSEIICCTTPSLQQLNLQLPLKT..........................  232
PF01403.1   ..............................................................................................................  109
PF01833.2   SFI..................SGGSTITGVGKNLNSVSVPRMVINVHEAGRNFTVACQHRSNSEIICCTTPSLQQLNLQLPLKT..........................   76
PF01833.1   SFI..................SGGSTITGVGKNLNSVSVPRMVINVHEAGRNFTVACQHRSNSEIICCTTPSLQQLNLQLPLKT..........................  240
MET-205     ..............................................................................................................    0
MET-201     SFISTWWKEPLNIVSFLFCFASGGSTITGVGKNLNSVSVPRMVINVHEAGRNFTVACQHRSNSEIICCTTPSLQQLNLQLPLKTKAFFMLDGILSKYFDLIYVHNPVFKP  861
MET-206     ..............................................................................................................  190
MET-203     SFI..................SGGSTITGVGKNLNSVSVPRMVINVHEAGRNFTVACQHRSNSEIICCTTPSLQQLNLQLPLKTKAFFMLDGILSKYFDLIYVHNPVFKP  123
MET-202     SFI..................SGGSTITGVGKNLNSVSVPRMVINVHEAGRNFTVACQHRSNSEIICCTTPSLQQLNLQLPLKTKAFFMLDGILSKYFDLIYVHNPVFKP  843
MET-204     SFIRHVNIALIQR.................................................................................................  764
```

logo
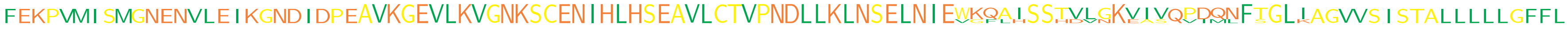

```
FORETINIB   ..............................................................................................................    0
PF01403     ..............................................................................................................  435
PF07714     ..............................................................................................................    0
PF01437     ..............................................................................................................   40
PF01833.3   ..............................................................................................................  164
PF01833     ..............................................................................................................  232
PF01403.1   ..............................................................................................................  109
PF01833.2   ..............................................................................................................   76
PF01833.1   ..............................................................................................................  240
MET-205     .........................AVKGEVLKVGNKSCENIHLHSEAVLCTVPNDLLKLNSELNIEWKQAISSTVLGKVIVQPDQNFTGLIAGVVSISTALLLLLGFFL   85
MET-201     FEKPVMISMGNENVLEIKGNDIDPEAVKGEVLKVGNKSCENIHLHSEAVLCTVPNDLLKLNSELNIEWKQAISSTVLGKVIVQPDQNFTGLIAGVVSISTALLLLLGFFL  971
MET-206     ..............................................................................................................  190
MET-203     FEKPVMISMGNENVLEIKGNDIDPEAVKGEVLKVGNKSCENIHLHSEAVLCTVPNDLLKLNSELNIEVGFLHSSHDVNKEAS.VIMLFSGLK.................  214
MET-202     FEKPVMISMGNENVLEIKGNDIDPEAVKGEVLKVGNKSCENIHLHSEAVLCTVPNDLLKLNSELNIEWKQAISSTVLGKVIVQPDQNFTGLIAGVVSISTALLLLLGFFL  953
MET-204     ..............................................................................................................  764
```

```
logo
             WLKKRKQIKDLGSELVRYDARVHTPHLDRLVSARSVSPTTEMVSNESVDYRATFPEDQFPNSSQNGSCRQVQYPLTDMSPILTSGDSDISSPLLQNTVHIDLSALNPELV
FORETINIB    ..............................................................................................................     0
PF01403      ..............................................................................................................   435
PF07714      ..............................................................................................................     0
PF01437      ..............................................................................................................    40
PF01833.3    ..............................................................................................................   164
PF01833      ..............................................................................................................   232
PF01403.1    ..............................................................................................................   109
PF01833.2    ..............................................................................................................    76
PF01833.1    ..............................................................................................................   240
MET-205      WLKKRKQIK...............................................DQFPNSSQNGSCRQVQYPLTDMSPILTSGDSDISSPLLQNTVHIDLSALNPELV   148
MET-201      WLKKRKQIKDLGSELVRYDARVHTPHLDRLVSARSVSPTTEMVSNESVDYRATFPEDQFPNSSQNGSCRQVQYPLTDMSPILTSGDSDISSPLLQNTVHIDLSALNPELV  1081
MET-206      ..............................................................................................................   190
MET-203      ..............................................................................................................   214
MET-202      WLKKRKQIKDLGSELVRYDARVHTPHLDRLVSARSVSPTTEMVSNESVDYRATFPEDQFPNSSQNGSCRQVQYPLTDMSPILTSGDSDISSPLLQNTVHIDLSALNPELV  1063
MET-204      ..............................................................................................................   764

logo
             QAVQHVVIGPSSLIVHFNEVIGRGHFGCVYHGTLLDNDGKKIHCAVKSLNRITDIGEVSQFLTEGIIMKDFSHPNVLSLLGICLRSEGSPLVVLPYMKHGDLRNFIRNET
FORETINIB    ....................I........V..............A.K................E...M..F....VL................LPYMK.G..........    15
PF01403      ..............................................................................................................   435
PF07714      ...............HFNEVIGRGHFGCVYHGTLLDNDGKKIHCAVKSLNRITDIGEVSQFLTEGIIMKDFSHPNVLSLLGICLRSEGSPLVVLPYMKHGDLRNFIRNET    95
PF01437      ..............................................................................................................    40
PF01833.3    ..............................................................................................................   164
PF01833      ..............................................................................................................   232
PF01403.1    ..............................................................................................................   109
PF01833.2    ..............................................................................................................    76
PF01833.1    ..............................................................................................................   240
MET-205      QAVQHVVIGP....................................................................................................   158
MET-201      QAVQHVVIGPSSLIVHFNEVIGRGHFGCVYHGTLLDNDGKKIHCAVKSLNRITDIGEVSQFLTEGIIMKDFSHPNVLSLLGICLRSEGSPLVVLPYMKHGDLRNFIRNET  1191
MET-206      ..............................................................................................................   190
MET-203      ..............................................................................................................   214
MET-202      QAVQHVVIGPSSLIVHFNEVIGRGHFGCVYHGTLLDNDGKKIHCAVKSLNRITDIGEVSQFLTEGIIMKDFSHPNVLSLLGICLRSEGSPLVVLPYMKHGDLRNFIRNET  1173
MET-204      ..............................................................................................................   764

logo
             HNPTVKDLIGFGLQVAKGMKYLASKKFVHRDLAARNCMLDEKFTVKVADFGLARDMYDKEYYSVHNKTGAKLPVKWMALESLQTQKFTTKSDVWSFGVLLWELMTRGAPP
FORETINIB    .....................L....F.H........M........VADF............................................................    23
PF01403      ..............................................................................................................   435
PF07714      HNPTVKDLIGFGLQVAKGMKYLASKKFVHRDLAARNCMLDEKFTVKVADFGLARDMYDKEYYSVHNKTGAKLPVKWMALESLQTQKFTTKSDVWSFGVLLWELMTRGAPP   205
PF01437      ..............................................................................................................    40
PF01833.3    ..............................................................................................................   164
PF01833      ..............................................................................................................   232
PF01403.1    ..............................................................................................................   109
PF01833.2    ..............................................................................................................    76
PF01833.1    ..............................................................................................................   240
MET-205      ..............................................................................................................   158
MET-201      HNPTVKDLIGFGLQVAKGMKYLASKKFVHRDLAARNCMLDEKFTVKVADFGLARDMYDKEYYSVHNKTGAKLPVKWMALESLQTQKFTTKSDVWSFGVLLWELMTRGAPP  1301
MET-206      ..............................................................................................................   190
MET-203      ..............................................................................................................   214
MET-202      HNPTVKDLIGFGLQVAKGMKYLASKKFVHRDLAARNCMLDEKFTVKVADFGLARDMYDKEYYSVHNKTGAKLPVKWMALESLQTQKFTTKSDVWSFGVLLWELMTRGAPP  1283
MET-204      ..............................................................................................................   764
```

logo

```
            YPDVNTFDITVYLLQGRRLLQPEYCPDPLYEVMLKCWHPKAEMRPSFSELVSRISAIFSTFIGEHYVHVNATYVNVKCVAPYPSLLSSEDNADDEVDTRPASFWETS
FORETINIB   ...........................................................................................................    23
PF01403     ...........................................................................................................   435
PF07714     YPDVNTFDITVYLLQGRRLLQPEYCPDPLYEVMLKCWHPKAEMRPSFSELVSR......................................................   258
PF01437     ...........................................................................................................    40
PF01833.3   ...........................................................................................................   164
PF01833     ...........................................................................................................   232
PF01403.1   ...........................................................................................................   109
PF01833.2   ...........................................................................................................    76
PF01833.1   ...........................................................................................................   240
MET-205     ...........................................................................................................   158
MET-201     YPDVNTFDITVYLLQGRRLLQPEYCPDPLYEVMLKCWHPKAEMRPSFSELVSRISAIFSTFIGEHYVHVNATYVNVKCVAPYPSLLSSEDNADDEVDTRPASFWETS  1408
MET-206     ...........................................................................................................   190
MET-203     ...........................................................................................................   214
MET-202     YPDVNTFDITVYLLQGRRLLQPEYCPDPLYEVMLKCWHPKAEMRPSFSELVSRISAIFSTFIGEHYVHVNATYVNVKCVAPYPSLLSSEDNADDEVDTRPASFWETS  1390
MET-204     ...........................................................................................................   764
```

X non conserved
X similar
X $\geq$ 0% conserved
X $\geq$ 50% conserved

```
logo
           MKSKSKSLAECFPYDKPLIMKAPAVLAPGILVLLFTLVQRSNGECKEALAKSEMNVNMKYQLPNFTAETPIQNVILHEHHIFLGATNYIYVLNEEDLQKVAEYKTGPVLE
K-252A     ..............................................................................................................     0
PF01403    ...........................................................................LHEHHIFLGATNYIYVLNEEDLQKVAEYKTGPVLE    35
PF07714    ..............................................................................................................     0
PF01437    ..............................................................................................................     0
PF01833.3  ..............................................................................................................     0
PF01833    ..............................................................................................................     0
PF01403.1  ...........................................................................LHEHHIFLGATNYIYVLNEEDLQKVAEYKTGPVLE    35
PF01833.2  ..............................................................................................................     0
PF01833.1  ..............................................................................................................     0
MET-205    ..............................................................................................................     0
MET-201    ...................MKAPAVLAPGILVLLFTLVQRSNGECKEALAKSEMNVNMKYQLPNFTAETPIQNVILHEHHIFLGATNYIYVLNEEDLQKVAEYKTGPVLE    91
MET-206    MKSKSKSLAECFPYDKPLIMKAPAVLAPGILVLLFTLVQRSNGECKEALAKSEMNVNMKYQLPNFTAETPIQNVILHEHHIFLGATNYIYVLNEEDLQKVAEYKTGPVLE   110
MET-203    ..............................................................................................................     0
MET-202    ...................MKAPAVLAPGILVLLFTLVQRSNGECKEALAKSEMNVNMKYQLPNFTAETPIQNVILHEHHIFLGATNYIYVLNEEDLQKVAEYKTGPVLE    91
MET-204    ...................MKAPAVLAPGILVLLFTLVQRSNGECKEALAKSEMNVNMKYQLPNFTAETPIQNVILHEHHIFLGATNYIYVLNEEDLQKVAEYKTGPVLE    91

logo
           HPDCFPCQDCSSKANLSGGVWKDNINMALVVDTYYDDQLISCGSVNRGTCQRHVFPHNHTADIQSEVHCIFSPQIEEPSQCPDCVVSALGAKVLSSVKDRFINFFVGNTI
K-252A     ..............................................................................................................     0
PF01403    HPDCFPCQDCSSKANLSGGVWKDNINMALVVDTYYDDQLISCGSVNRGTCQRHVFPHNHTADIQSEVHCIFSPQIEEPSQCPDCVVSALGAKVLSSVKDRFINFFVGNTI   145
PF07714    ..............................................................................................................     0
PF01437    ..............................................................................................................     0
PF01833.3  ..............................................................................................................     0
PF01833    ..............................................................................................................     0
PF01403.1  HPDCFPCQDCSSKANLSGGVWKDNINMALVVDTYYDDQLISCGSVNRGTCQRHVFPHNHTADIQSEVHCIFSPQ....................................   109
PF01833.2  ..............................................................................................................     0
PF01833.1  ..............................................................................................................     0
MET-205    ..............................................................................................................     0
MET-201    HPDCFPCQDCSSKANLSGGVWKDNINMALVVDTYYDDQLISCGSVNRGTCQRHVFPHNHTADIQSEVHCIFSPQIEEPSQCPDCVVSALGAKVLSSVKDRFINFFVGNTI   201
MET-206    HPDCFPCQDCSSKANLSGGVWKDNINMALVVDTYYDDQLISCGSVNRGTCQRHVFPHNHTADIQSEVHCIFSPQIEEPSQ..............................   190
MET-203    ..............................................................................................................     0
MET-202    HPDCFPCQDCSSKANLSGGVWKDNINMALVVDTYYDDQLISCGSVNRGTCQRHVFPHNHTADIQSEVHCIFSPQIEEPSQCPDCVVSALGAKVLSSVKDRFINFFVGNTI   201
MET-204    HPDCFPCQDCSSKANLSGGVWKDNINMALVVDTYYDDQLISCGSVNRGTCQRHVFPHNHTADIQSEVHCIFSPQIEEPSQCPDCVVSALGAKVLSSVKDRFINFFVGNTI   201

logo
           NSSYFPDHPLHSISVRRLKETKDGFMFLTDQSYIDVLPEFRDSYPIKYVHAFESNNFIYFLTVQRETLDAQTFHTRIIRFCSINSGLHSYMEMPLECILTEKRKKRSTKK
K-252A     ..............................................................................................................     0
PF01403    NSSYFPDHPLHSISVRRLKETKDGFMFLTDQSYIDVLPEFRDSYPIKYVHAFESNNFIYFLTVQRETLDAQTFHTRIIRFCSINSGLHSYMEMPLECILTEKRKKRSTKK   255
PF07714    ..............................................................................................................     0
PF01437    ..............................................................................................................     0
PF01833.3  ..............................................................................................................     0
PF01833    ..............................................................................................................     0
PF01403.1  ..............................................................................................................   109
PF01833.2  ..............................................................................................................     0
PF01833.1  ..............................................................................................................     0
MET-205    ..............................................................................................................     0
MET-201    NSSYFPDHPLHSISVRRLKETKDGFMFLTDQSYIDVLPEFRDSYPIKYVHAFESNNFIYFLTVQRETLDAQTFHTRIIRFCSINSGLHSYMEMPLECILTEKRKKRSTKK   311
MET-206    ..............................................................................................................   190
MET-203    ..............................................................................................................     0
MET-202    NSSYFPDHPLHSISVRRLKETKDGFMFLTDQSYIDVLPEFRDSYPIKYVHAFESNNFIYFLTVQRETLDAQTFHTRIIRFCSINSGLHSYMEMPLECILTEKRKKRSTKK   311
MET-204    NSSYFPDHPLHSISVRRLKETKDGFMFLTDQSYIDVLPEFRDSYPIKYVHAFESNNFIYFLTVQRETLDAQTFHTRIIRFCSINSGLHSYMEMPLECILTEKRKKRSTKK   311
```

```
logo
            EVFNILQAAYVSKPGAQLARQIGASLNDDILFGVFAQSKPDSAEPMDRSAMCAFPIKYVNDFFNKIVNKNNVRCLQHFYGPNHEHCFNRTLLRNSSGCEARRDEYRTEFT
K-252A      ..............................................................................................................   0
PF01403     EVFNILQAAYVSKPGAQLARQIGASLNDDILFGVFAQSKPDSAEPMDRSAMCAFPIKYVNDFFNKIVNKNNVRCLQHFYGPNHEHCFNRTLLRNSSGCEARRDEYRTEFT 365
PF07714     ..............................................................................................................   0
PF01437     ..............................................................................................................   0
PF01833.3   ..............................................................................................................   0
PF01833     ..............................................................................................................   0
PF01403.1   .............................................................................................................. 109
PF01833.2   ..............................................................................................................   0
PF01833.1   ..............................................................................................................   0
MET-205     ..............................................................................................................   0
MET-201     EVFNILQAAYVSKPGAQLARQIGASLNDDILFGVFAQSKPDSAEPMDRSAMCAFPIKYVNDFFNKIVNKNNVRCLQHFYGPNHEHCFNRTLLRNSSGCEARRDEYRTEFT 421
MET-206     .............................................................................................................. 190
MET-203     ..............................................................................................................   0
MET-202     EVFNILQAAYVSKPGAQLARQIGASLNDDILFGVFAQSKPDSAEPMDRSAMCAFPIKYVNDFFNKIVNKNNVRCLQHFYGPNHEHCFNRTLLRNSSGCEARRDEYRTEFT 421
MET-204     EVFNILQAAYVSKPGAQLARQIGASLNDDILFGVFAQSKPDSAEPMDRSAMCAFPIKYVNDFFNKIVNKNNVRCLQHFYGPNHEHCFNRTLLRNSSGCEARRDEYRTEFT 421

logo
            TALQRVDLFMGQFSEVLLTSISTFIKGDLTIANLGTSEGRFMQVVVSRSGPSTPHVNFLLDSHPVSPEVIVEHTLNQNGYTLVITGKKITKIPLNGLGCRHFQSCSQCLS
K-252A      ..............................................................................................................   0
PF01403     TALQRVDLFMGQFSEVLLTSISTFIKGDLTIANLGTSEGRFMQVVVSRSGPSTPHVNFLLDSHPVSPEVI........................................ 435
PF07714     ..............................................................................................................   0
PF01437     ..................................................................................................CRHFQSCSQCLS  12
PF01833.3   ..............................................................................................................   0
PF01833     ..............................................................................................................   0
PF01403.1   .............................................................................................................. 109
PF01833.2   ..............................................................................................................   0
PF01833.1   ..............................................................................................................   0
MET-205     ..............................................................................................................   0
MET-201     TALQRVDLFMGQFSEVLLTSISTFIKGDLTIANLGTSEGRFMQVVVSRSGPSTPHVNFLLDSHPVSPEVIVEHTLNQNGYTLVITGKKITKIPLNGLGCRHFQSCSQCLS 531
MET-206     .............................................................................................................. 190
MET-203     ..............................................................................................................   0
MET-202     TALQRVDLFMGQFSEVLLTSISTFIKGDLTIANLGTSEGRFMQVVVSRSGPSTPHVNFLLDSHPVSPEVIVEHTLNQNGYTLVITGKKITKIPLNGLGCRHFQSCSQCLS 531
MET-204     TALQRVDLFMGQFSEVLLTSISTFIKGDLTIANLGTSEGRFMQVVVSRSGPSTPHVNFLLDSHPVSPEVIVEHTLNQNGYTLVITGKKITKIPLNGLGCRHFQSCSQCLS 531

logo
            APPFVQCGWCHDKCVRSEECLSGTWTQQICLPAIYKVFPNSAPLEGGTRLTICGWDFGFRRNNKFDLKKTRVLLGNESCTLTLSESTMNTLKCTVGPAMNKHFNMSIIIS
K-252A      ..............................................................................................................   0
PF01403     .............................................................................................................. 435
PF07714     ..............................................................................................................   0
PF01437     APPFVQCGWCHDKCVRSEECLSGTWTQQ..................................................................................  40
PF01833.3   ...............................PAIYKVFPNSAPLEGGTRLTICGWDFGFRRNNKFDLKKTRVLLGNESCTLTLSESTMNTLKCTVGPAMNKHFNMSIIIS  79
PF01833     ...............................PAIYKVFPNSAPLEGGTRLTICGWDFGFRRNNKFDLKKTRVLLGNESCTLTLSESTMNTLKCTVGPAMNKHFNMSIIIS  79
PF01403.1   .............................................................................................................. 109
PF01833.2   ..............................................................................................................   0
PF01833.1   ...............................PAIYKVFPNSAPLEGGTRLTICGWDFGFRRNNKFDLKKTRVLLGNESCTLTLSESTMNTLKCTVGPAMNKHFNMSIIIS  79
MET-205     ..............................................................................................................   0
MET-201     APPFVQCGWCHDKCVRSEECLSGTWTQQICLPAIYKVFPNSAPLEGGTRLTICGWDFGFRRNNKFDLKKTRVLLGNESCTLTLSESTMNTLKCTVGPAMNKHFNMSIIIS 641
MET-206     .............................................................................................................. 190
MET-203     ..............................................................................................................   0
MET-202     APPFVQCGWCHDKCVRSEECLSGTWTQQICLPAIYKVFPNSAPLEGGTRLTICGWDFGFRRNNKFDLKKTRVLLGNESCTLTLSESTMNTLKCTVGPAMNKHFNMSIIIS 641
MET-204     APPFVQCGWCHDKCVRSEECLSGTWTQQICLPAIYKVFPNSAPLEGGTRLTICGWDFGFRRNNKFDLKKTRVLLGNESCTLTLSESTMNTLKCTVGPAMNKHFNMSIIIS 641
```

```
logo
            NGHGTTQYSTFSYVDPVITSISPKYGPMAGGTLLTLTGNYLNSGNSRHISIGGKTCTLKSVSNSILECYTPAQTISTEFAVKLKIDLANRETSIFSYREDPIVYEIHPTK
K-252A      ..............................................................................................................    0
PF01403     ..............................................................................................................  435
PF07714     ..............................................................................................................    0
PF01437     ..............................................................................................................   40
PF01833.3   NGHGTTQYSTFSY..PVITSISPKYGPMAGGTLLTLTGNYLNSGNSRHISIGGKTCTLKSVSNSILECYTPAQTISTEFAVKLKIDL.......................  164
PF01833     NGHGTTQYSTFSY..PVITSISPKYGPMAGGTLLTLTGNYLNSGNSRHISIGGKTCTLKSVSNSILECYTPAQTISTEFAVKLKIDL.......................  164
PF01403.1   ..............................................................................................................  109
PF01833.2   ....................................................................................................PIVYEIHPTK   10
PF01833.1   NGHGTTQYSTFSY..PVITSISPKYGPMAGGTLLTLTGNYLNSGNSRHISIGGKTCTLKSVSNSILECYTPAQTISTEFAVKLKIDL.............PIVYEIHPTK  174
MET-205     ..............................................................................................................    0
MET-201     NGHGTTQYSTFSYVDPVITSISPKYGPMAGGTLLTLTGNYLNSGNSRHISIGGKTCTLKSVSNSILECYTPAQTISTEFAVKLKIDLANRETSIFSYREDPIVYEIHPTK  751
MET-206     ..............................................................................................................  190
MET-203     ...............................................................................AVKLKIDLANRETSIFSYREDPIVYEIHPTK   31
MET-202     NGHGTTQYSTFSYVDPVITSISPKYGPMAGGTLLTLTGNYLNSGNSRHISIGGKTCTLKSVSNSILECYTPAQTISTEFAVKLKIDLANRETSIFSYREDPIVYEIHPTK  751
MET-204     NGHGTTQYSTFSYVDPVITSISPKYGPMAGGTLLTLTGNYLNSGNSRHISIGGKTCTLKSVSNSILECYTPAQTISTEFAVKLKIDLANRETSIFSYREDPIVYEIHPTK  751

logo
            SFI[illegible]LFCFASGGSTITGVGKNLNSVSVPRMVINVHEAGRNFTVACQHRSNSEIICCTTPSLQQLNLQLPLKTKAFFMLDGILSKYFDLIYVHNPVFKP
K-252A      ..............................................................................................................    0
PF01403     ..............................................................................................................  435
PF07714     ..............................................................................................................    0
PF01437     ..............................................................................................................   40
PF01833.3   ..............................................................................................................  164
PF01833     ................LFCFASGGSTITGVGKNLNSVSVPRMVINVHEAGRNFTVACQHRSNSEIICCTTPSLQQLNLQLPLKT..........................  232
PF01403.1   ..............................................................................................................  109
PF01833.2   SFI..................SGGSTITGVGKNLNSVSVPRMVINVHEAGRNFTVACQHRSNSEIICCTTPSLQQLNLQLPLKT..........................   76
PF01833.1   SFI..................SGGSTITGVGKNLNSVSVPRMVINVHEAGRNFTVACQHRSNSEIICCTTPSLQQLNLQLPLKT..........................  240
MET-205     ..............................................................................................................    0
MET-201     SFISTWWKEPLNIVSFLFCFASGGSTITGVGKNLNSVSVPRMVINVHEAGRNFTVACQHRSNSEIICCTTPSLQQLNLQLPLKTKAFFMLDGILSKYFDLIYVHNPVFKP  861
MET-206     ..............................................................................................................  190
MET-203     SFI..................SGGSTITGVGKNLNSVSVPRMVINVHEAGRNFTVACQHRSNSEIICCTTPSLQQLNLQLPLKTKAFFMLDGILSKYFDLIYVHNPVFKP  123
MET-202     SFI..................SGGSTITGVGKNLNSVSVPRMVINVHEAGRNFTVACQHRSNSEIICCTTPSLQQLNLQLPLKTKAFFMLDGILSKYFDLIYVHNPVFKP  843
MET-204     SFIRHVNIALIQR.................................................................................................  764

logo
            FEKPVMISMGNENVLEIKGNDIDPEAVKGEVLKVGNKSCENIHLHSEAVLCTVPNDLLKLNSELNIEWKQAISSTVLGKVIVQPDQNFTGLIAGVVSISTALLLLLGFFL
K-252A      ..............................................................................................................    0
PF01403     ..............................................................................................................  435
PF07714     ..............................................................................................................    0
PF01437     ..............................................................................................................   40
PF01833.3   ..............................................................................................................  164
PF01833     ..............................................................................................................  232
PF01403.1   ..............................................................................................................  109
PF01833.2   ..............................................................................................................   76
PF01833.1   ..............................................................................................................  240
MET-205     .........................AVKGEVLKVGNKSCENIHLHSEAVLCTVPNDLLKLNSELNIEWKQAISSTVLGKVIVQPDQNFTGLIAGVVSISTALLLLLGFFL   85
MET-201     FEKPVMISMGNENVLEIKGNDIDPEAVKGEVLKVGNKSCENIHLHSEAVLCTVPNDLLKLNSELNIEWKQAISSTVLGKVIVQPDQNFTGLIAGVVSISTALLLLLGFFL  971
MET-206     ..............................................................................................................  190
MET-203     FEKPVMISMGNENVLEIKGNDIDPEAVKGEVLKVGNKSCENIHLHSEAVLCTVPNDLLKLNSELNIEVGFLHSSHDVNKEAS.VIMLFSGLK..................  214
MET-202     FEKPVMISMGNENVLEIKGNDIDPEAVKGEVLKVGNKSCENIHLHSEAVLCTVPNDLLKLNSELNIEWKQAISSTVLGKVIVQPDQNFTGLIAGVVSISTALLLLLGFFL  953
MET-204     ..............................................................................................................  764
```

```
logo
             WLKKRKQIKDLGSELVRYDARVHTPHLDRLVSARSVSPTTEMVSNESVDYRATFPEDQFPNSSQNGSCRQVQYPLTDMSPILTSGDSDISSPLLQNTVHIDLSALNPELV
K-252A       ..............................................................................................................    0
PF01403      ..............................................................................................................  435
PF07714      ..............................................................................................................    0
PF01437      ..............................................................................................................   40
PF01833.3    ..............................................................................................................  164
PF01833      ..............................................................................................................  232
PF01403.1    ..............................................................................................................  109
PF01833.2    ..............................................................................................................   76
PF01833.1    ..............................................................................................................  240
MET-205      WLKKRKQIK...............................................DQFPNSSQNGSCRQVQYPLTDMSPILTSGDSDISSPLLQNTVHIDLSALNPELV  148
MET-201      WLKKRKQIKDLGSELVRYDARVHTPHLDRLVSARSVSPTTEMVSNESVDYRATFPEDQFPNSSQNGSCRQVQYPLTDMSPILTSGDSDISSPLLQNTVHIDLSALNPELV 1081
MET-206      ..............................................................................................................  190
MET-203      ..............................................................................................................  214
MET-202      WLKKRKQIKDLGSELVRYDARVHTPHLDRLVSARSVSPTTEMVSNESVDYRATFPEDQFPNSSQNGSCRQVQYPLTDMSPILTSGDSDISSPLLQNTVHIDLSALNPELV 1063
MET-204      ..............................................................................................................  764

logo
             QAVQHVVIGPSSLIVHFNEVIGRGHFGCVYHGTLLDNDGKKIHCAVKSLNRITDIGEVSQFLTEGIIMKDFSHPNVLSLLGICLRSEGSPLVVLPYMKHGDLRNFIRNET
K-252A       ....................IG...F..V...............A.K..............................................LPYM..G..........   11
PF01403      ..............................................................................................................  435
PF07714      ...............HFNEVIGRGHFGCVYHGTLLDNDGKKIHCAVKSLNRITDIGEVSQFLTEGIIMKDFSHPNVLSLLGICLRSEGSPLVVLPYMKHGDLRNFIRNET   95
PF01437      ..............................................................................................................   40
PF01833.3    ..............................................................................................................  164
PF01833      ..............................................................................................................  232
PF01403.1    ..............................................................................................................  109
PF01833.2    ..............................................................................................................   76
PF01833.1    ..............................................................................................................  240
MET-205      QAVQHVVIGP....................................................................................................  158
MET-201      QAVQHVVIGPSSLIVHFNEVIGRGHFGCVYHGTLLDNDGKKIHCAVKSLNRITDIGEVSQFLTEGIIMKDFSHPNVLSLLGICLRSEGSPLVVLPYMKHGDLRNFIRNET 1191
MET-206      ..............................................................................................................  190
MET-203      ..............................................................................................................  214
MET-202      QAVQHVVIGPSSLIVHFNEVIGRGHFGCVYHGTLLDNDGKKIHCAVKSLNRITDIGEVSQFLTEGIIMKDFSHPNVLSLLGICLRSEGSPLVVLPYMKHGDLRNFIRNET 1173
MET-204      ..............................................................................................................  764

logo
             HNPTVKDLIGFGLQVAKGMKYLASKKFVHRDLAARNCMLDEKFTVKVADFGLARDMYDKEYYSVHNKTGAKLPVKWMALESLQTQKFTTKSDVWSFGVLLWELMTRGAPP
K-252A       .....................................M...............R.MY.....................................................   15
PF01403      ..............................................................................................................  435
PF07714      HNPTVKDLIGFGLQVAKGMKYLASKKFVHRDLAARNCMLDEKFTVKVADFGLARDMYDKEYYSVHNKTGAKLPVKWMALESLQTQKFTTKSDVWSFGVLLWELMTRGAPP  205
PF01437      ..............................................................................................................   40
PF01833.3    ..............................................................................................................  164
PF01833      ..............................................................................................................  232
PF01403.1    ..............................................................................................................  109
PF01833.2    ..............................................................................................................   76
PF01833.1    ..............................................................................................................  240
MET-205      ..............................................................................................................  158
MET-201      HNPTVKDLIGFGLQVAKGMKYLASKKFVHRDLAARNCMLDEKFTVKVADFGLARDMYDKEYYSVHNKTGAKLPVKWMALESLQTQKFTTKSDVWSFGVLLWELMTRGAPP 1301
MET-206      ..............................................................................................................  190
MET-203      ..............................................................................................................  214
MET-202      HNPTVKDLIGFGLQVAKGMKYLASKKFVHRDLAARNCMLDEKFTVKVADFGLARDMYDKEYYSVHNKTGAKLPVKWMALESLQTQKFTTKSDVWSFGVLLWELMTRGAPP 1283
MET-204      ..............................................................................................................  764
```

```
logo
            YPDVNTFDITVYLLQGRRLLQPEYCPDPLYEVMLKCWHPKAEMRPSFSELVSRISAIFSTFIGEHYVHVNATYVNVKCVAPYPSLLSSEDNADDEVDTRPASFWETS
K-252A      ...........................................................................................................    15
PF01403     ...........................................................................................................   435
PF07714     YPDVNTFDITVYLLQGRRLLQPEYCPDPLYEVMLKCWHPKAEMRPSFSELVSR......................................................   258
PF01437     ...........................................................................................................    40
PF01833.3   ...........................................................................................................   164
PF01833     ...........................................................................................................   232
PF01403.1   ...........................................................................................................   109
PF01833.2   ...........................................................................................................    76
PF01833.1   ...........................................................................................................   240
MET-205     ...........................................................................................................   158
MET-201     YPDVNTFDITVYLLQGRRLLQPEYCPDPLYEVMLKCWHPKAEMRPSFSELVSRISAIFSTFIGEHYVHVNATYVNVKCVAPYPSLLSSEDNADDEVDTRPASFWETS  1408
MET-206     ...........................................................................................................   190
MET-203     ...........................................................................................................   214
MET-202     YPDVNTFDITVYLLQGRRLLQPEYCPDPLYEVMLKCWHPKAEMRPSFSELVSRISAIFSTFIGEHYVHVNATYVNVKCVAPYPSLLSSEDNADDEVDTRPASFWETS  1390
MET-204     ...........................................................................................................   764
```

- X non conserved
- X similar
- X $\geq$ 0% conserved
- X $\geq$ 50% conserved

```
logo
             MKSKSKSLAECFPYDKPLIMKAPAVLAPGILVLLFTLVQRSNGECKEALAKSEMNVNMKYQLPNFTAETPIQNVILHEHHIFLGATNYIYVLNEEDLQKVAEYKTGPVLE
MERESTINIB   ..............................................................................................................   0
PF01403      ...........................................................................LHEHHIFLGATNYIYVLNEEDLQKVAEYKTGPVLE  35
PF07714      ..............................................................................................................   0
PF01437      ..............................................................................................................   0
PF01833.3    ..............................................................................................................   0
PF01833      ..............................................................................................................   0
PF01403.1    ...........................................................................LHEHHIFLGATNYIYVLNEEDLQKVAEYKTGPVLE  35
PF01833.2    ..............................................................................................................   0
PF01833.1    ..............................................................................................................   0
MET-205      ..............................................................................................................   0
MET-201      ...................MKAPAVLAPGILVLLFTLVQRSNGECKEALAKSEMNVNMKYQLPNFTAETPIQNVILHEHHIFLGATNYIYVLNEEDLQKVAEYKTGPVLE  91
MET-206      MKSKSKSLAECFPYDKPLIMKAPAVLAPGILVLLFTLVQRSNGECKEALAKSEMNVNMKYQLPNFTAETPIQNVILHEHHIFLGATNYIYVLNEEDLQKVAEYKTGPVLE 110
MET-203      ..............................................................................................................   0
MET-202      ...................MKAPAVLAPGILVLLFTLVQRSNGECKEALAKSEMNVNMKYQLPNFTAETPIQNVILHEHHIFLGATNYIYVLNEEDLQKVAEYKTGPVLE  91
MET-204      ...................MKAPAVLAPGILVLLFTLVQRSNGECKEALAKSEMNVNMKYQLPNFTAETPIQNVILHEHHIFLGATNYIYVLNEEDLQKVAEYKTGPVLE  91

logo
             HPDCFPCQDCSSKANLSGGVWKDNINMALVVDTYYDDQLISCGSVNRGTCQRHVFPHNHTADIQSEVHCIFSPQIEEPSQCPDCVVSALGAKVLSSVKDRFINFFVGNTI
MERESTINIB   ..............................................................................................................   0
PF01403      HPDCFPCQDCSSKANLSGGVWKDNINMALVVDTYYDDQLISCGSVNRGTCQRHVFPHNHTADIQSEVHCIFSPQIEEPSQCPDCVVSALGAKVLSSVKDRFINFFVGNTI 145
PF07714      ..............................................................................................................   0
PF01437      ..............................................................................................................   0
PF01833.3    ..............................................................................................................   0
PF01833      ..............................................................................................................   0
PF01403.1    HPDCFPCQDCSSKANLSGGVWKDNINMALVVDTYYDDQLISCGSVNRGTCQRHVFPHNHTADIQSEVHCIFSPQ.................................... 109
PF01833.2    ..............................................................................................................   0
PF01833.1    ..............................................................................................................   0
MET-205      ..............................................................................................................   0
MET-201      HPDCFPCQDCSSKANLSGGVWKDNINMALVVDTYYDDQLISCGSVNRGTCQRHVFPHNHTADIQSEVHCIFSPQIEEPSQCPDCVVSALGAKVLSSVKDRFINFFVGNTI 201
MET-206      HPDCFPCQDCSSKANLSGGVWKDNINMALVVDTYYDDQLISCGSVNRGTCQRHVFPHNHTADIQSEVHCIFSPQIEEPSQ.............................. 190
MET-203      ..............................................................................................................   0
MET-202      HPDCFPCQDCSSKANLSGGVWKDNINMALVVDTYYDDQLISCGSVNRGTCQRHVFPHNHTADIQSEVHCIFSPQIEEPSQCPDCVVSALGAKVLSSVKDRFINFFVGNTI 201
MET-204      HPDCFPCQDCSSKANLSGGVWKDNINMALVVDTYYDDQLISCGSVNRGTCQRHVFPHNHTADIQSEVHCIFSPQIEEPSQCPDCVVSALGAKVLSSVKDRFINFFVGNTI 201

logo
             NSSYFPDHPLHSISVRRLKETKDGFMFLTDQSYIDVLPEFRDSYPIKYVHAFESNNFIYFLTVQRETLDAQTFHTRIIRFCSINSGLHSYMEMPLECILTEKRKKRSTKK
MERESTINIB   ..............................................................................................................   0
PF01403      NSSYFPDHPLHSISVRRLKETKDGFMFLTDQSYIDVLPEFRDSYPIKYVHAFESNNFIYFLTVQRETLDAQTFHTRIIRFCSINSGLHSYMEMPLECILTEKRKKRSTKK 255
PF07714      ..............................................................................................................   0
PF01437      ..............................................................................................................   0
PF01833.3    ..............................................................................................................   0
PF01833      ..............................................................................................................   0
PF01403.1    .............................................................................................................. 109
PF01833.2    ..............................................................................................................   0
PF01833.1    ..............................................................................................................   0
MET-205      ..............................................................................................................   0
MET-201      NSSYFPDHPLHSISVRRLKETKDGFMFLTDQSYIDVLPEFRDSYPIKYVHAFESNNFIYFLTVQRETLDAQTFHTRIIRFCSINSGLHSYMEMPLECILTEKRKKRSTKK 311
MET-206      .............................................................................................................. 190
MET-203      ..............................................................................................................   0
MET-202      NSSYFPDHPLHSISVRRLKETKDGFMFLTDQSYIDVLPEFRDSYPIKYVHAFESNNFIYFLTVQRETLDAQTFHTRIIRFCSINSGLHSYMEMPLECILTEKRKKRSTKK 311
MET-204      NSSYFPDHPLHSISVRRLKETKDGFMFLTDQSYIDVLPEFRDSYPIKYVHAFESNNFIYFLTVQRETLDAQTFHTRIIRFCSINSGLHSYMEMPLECILTEKRKKRSTKK 311
```

```
logo
             EVFNILQAAYVSKPGAQLARQIGASLNDDILFGVFAQSKPDSAEPMDRSAMCAFPIKYVNDFFNKIVNKNNVRCLQHFYGPNHEHCFNRTLLRNSSGCEARRDEYRTEFT
MERESTINIB   ..............................................................................................................   0
PF01403      EVFNILQAAYVSKPGAQLARQIGASLNDDILFGVFAQSKPDSAEPMDRSAMCAFPIKYVNDFFNKIVNKNNVRCLQHFYGPNHEHCFNRTLLRNSSGCEARRDEYRTEFT 365
PF07714      ..............................................................................................................   0
PF01437      ..............................................................................................................   0
PF01833.3    ..............................................................................................................   0
PF01833      ..............................................................................................................   0
PF01403.1    .............................................................................................................. 109
PF01833.2    ..............................................................................................................   0
PF01833.1    ..............................................................................................................   0
MET-205      ..............................................................................................................   0
MET-201      EVFNILQAAYVSKPGAQLARQIGASLNDDILFGVFAQSKPDSAEPMDRSAMCAFPIKYVNDFFNKIVNKNNVRCLQHFYGPNHEHCFNRTLLRNSSGCEARRDEYRTEFT 421
MET-206      .............................................................................................................. 190
MET-203      ..............................................................................................................   0
MET-202      EVFNILQAAYVSKPGAQLARQIGASLNDDILFGVFAQSKPDSAEPMDRSAMCAFPIKYVNDFFNKIVNKNNVRCLQHFYGPNHEHCFNRTLLRNSSGCEARRDEYRTEFT 421
MET-204      EVFNILQAAYVSKPGAQLARQIGASLNDDILFGVFAQSKPDSAEPMDRSAMCAFPIKYVNDFFNKIVNKNNVRCLQHFYGPNHEHCFNRTLLRNSSGCEARRDEYRTEFT 421

logo
             TALQRVDLFMGQFSEVLLTSISTFIKGDLTIANLGTSEGRFMQVVVSRSGPSTPHVNFLLDSHPVSPEVIVEHTLNQNGYTLVITGKKITKIPLNGLGCRHFQSCSQCLS
MERESTINIB   ..............................................................................................................   0
PF01403      TALQRVDLFMGQFSEVLLTSISTFIKGDLTIANLGTSEGRFMQVVVSRSGPSTPHVNFLLDSHPVSPEVI........................................ 435
PF07714      ..............................................................................................................   0
PF01437      ..................................................................................................CRHFQSCSQCLS  12
PF01833.3    ..............................................................................................................   0
PF01833      ..............................................................................................................   0
PF01403.1    .............................................................................................................. 109
PF01833.2    ..............................................................................................................   0
PF01833.1    ..............................................................................................................   0
MET-205      ..............................................................................................................   0
MET-201      TALQRVDLFMGQFSEVLLTSISTFIKGDLTIANLGTSEGRFMQVVVSRSGPSTPHVNFLLDSHPVSPEVIVEHTLNQNGYTLVITGKKITKIPLNGLGCRHFQSCSQCLS 531
MET-206      .............................................................................................................. 190
MET-203      ..............................................................................................................   0
MET-202      TALQRVDLFMGQFSEVLLTSISTFIKGDLTIANLGTSEGRFMQVVVSRSGPSTPHVNFLLDSHPVSPEVIVEHTLNQNGYTLVITGKKITKIPLNGLGCRHFQSCSQCLS 531
MET-204      TALQRVDLFMGQFSEVLLTSISTFIKGDLTIANLGTSEGRFMQVVVSRSGPSTPHVNFLLDSHPVSPEVIVEHTLNQNGYTLVITGKKITKIPLNGLGCRHFQSCSQCLS 531

logo
             APPFVQCGWCHDKCVRSEECLSGTWTQQICLPAIYKVFPNSAPLEGGTRLTICGWDFGFRRNNKFDLKKTRVLLGNESCTLTLSESTMNTLKCTVGPAMNKHFNMSIIIS
MERESTINIB   ..............................................................................................................   0
PF01403      .............................................................................................................. 435
PF07714      ..............................................................................................................   0
PF01437      APPFVQCGWCHDKCVRSEECLSGTWTQQ..................................................................................  40
PF01833.3    ...............................PAIYKVFPNSAPLEGGTRLTICGWDFGFRRNNKFDLKKTRVLLGNESCTLTLSESTMNTLKCTVGPAMNKHFNMSIIIS  79
PF01833      ...............................PAIYKVFPNSAPLEGGTRLTICGWDFGFRRNNKFDLKKTRVLLGNESCTLTLSESTMNTLKCTVGPAMNKHFNMSIIIS  79
PF01403.1    .............................................................................................................. 109
PF01833.2    ..............................................................................................................   0
PF01833.1    ...............................PAIYKVFPNSAPLEGGTRLTICGWDFGFRRNNKFDLKKTRVLLGNESCTLTLSESTMNTLKCTVGPAMNKHFNMSIIIS  79
MET-205      ..............................................................................................................   0
MET-201      APPFVQCGWCHDKCVRSEECLSGTWTQQICLPAIYKVFPNSAPLEGGTRLTICGWDFGFRRNNKFDLKKTRVLLGNESCTLTLSESTMNTLKCTVGPAMNKHFNMSIIIS 641
MET-206      .............................................................................................................. 190
MET-203      ..............................................................................................................   0
MET-202      APPFVQCGWCHDKCVRSEECLSGTWTQQICLPAIYKVFPNSAPLEGGTRLTICGWDFGFRRNNKFDLKKTRVLLGNESCTLTLSESTMNTLKCTVGPAMNKHFNMSIIIS 641
MET-204      APPFVQCGWCHDKCVRSEECLSGTWTQQICLPAIYKVFPNSAPLEGGTRLTICGWDFGFRRNNKFDLKKTRVLLGNESCTLTLSESTMNTLKCTVGPAMNKHFNMSIIIS 641
```

logo

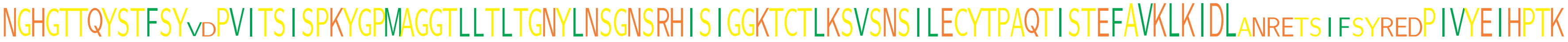

```
MERESTINIB   ..............................................................................................................     0
PF01403      ..............................................................................................................   435
PF07714      ..............................................................................................................     0
PF01437      ..............................................................................................................    40
PF01833.3    NGHGTTQYSTFSY..PVITSISPKYGPMAGGTLLTLTGNYLNSGNSRHISIGGKTCTLKSVSNSILECYTPAQTISTEFAVKLKIDL.......................   164
PF01833      NGHGTTQYSTFSY..PVITSISPKYGPMAGGTLLTLTGNYLNSGNSRHISIGGKTCTLKSVSNSILECYTPAQTISTEFAVKLKIDL.......................   164
PF01403.1    ..............................................................................................................   109
PF01833.2    ....................................................................................................PIVYEIHPTK    10
PF01833.1    NGHGTTQYSTFSY..PVITSISPKYGPMAGGTLLTLTGNYLNSGNSRHISIGGKTCTLKSVSNSILECYTPAQTISTEFAVKLKIDL.............PIVYEIHPTK   174
MET-205      ..............................................................................................................     0
MET-201      NGHGTTQYSTFSYVDPVITSISPKYGPMAGGTLLTLTGNYLNSGNSRHISIGGKTCTLKSVSNSILECYTPAQTISTEFAVKLKIDLANRETSIFSYREDPIVYEIHPTK   751
MET-206      ..............................................................................................................   190
MET-203      ...............................................................................AVKLKIDLANRETSIFSYREDPIVYEIHPTK    31
MET-202      NGHGTTQYSTFSYVDPVITSISPKYGPMAGGTLLTLTGNYLNSGNSRHISIGGKTCTLKSVSNSILECYTPAQTISTEFAVKLKIDLANRETSIFSYREDPIVYEIHPTK   751
MET-204      NGHGTTQYSTFSYVDPVITSISPKYGPMAGGTLLTLTGNYLNSGNSRHISIGGKTCTLKSVSNSILECYTPAQTISTEFAVKLKIDLANRETSIFSYREDPIVYEIHPTK   751
```

logo

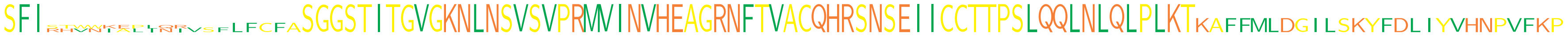

```
MERESTINIB   ..............................................................................................................     0
PF01403      ..............................................................................................................   435
PF07714      ..............................................................................................................     0
PF01437      ..............................................................................................................    40
PF01833.3    ..............................................................................................................   164
PF01833      ................LFCFASGGSTITGVGKNLNSVSVPRMVINVHEAGRNFTVACQHRSNSEIICCTTPSLQQLNLQLPLKT..........................   232
PF01403.1    ..............................................................................................................   109
PF01833.2    SFI..................SGGSTITGVGKNLNSVSVPRMVINVHEAGRNFTVACQHRSNSEIICCTTPSLQQLNLQLPLKT..........................    76
PF01833.1    SFI..................SGGSTITGVGKNLNSVSVPRMVINVHEAGRNFTVACQHRSNSEIICCTTPSLQQLNLQLPLKT..........................   240
MET-205      ..............................................................................................................     0
MET-201      SFISTWWKEPLNIVSFLFCFASGGSTITGVGKNLNSVSVPRMVINVHEAGRNFTVACQHRSNSEIICCTTPSLQQLNLQLPLKTKAFFMLDGILSKYFDLIYVHNPVFKP   861
MET-206      ..............................................................................................................   190
MET-203      SFI..................SGGSTITGVGKNLNSVSVPRMVINVHEAGRNFTVACQHRSNSEIICCTTPSLQQLNLQLPLKTKAFFMLDGILSKYFDLIYVHNPVFKP   123
MET-202      SFI..................SGGSTITGVGKNLNSVSVPRMVINVHEAGRNFTVACQHRSNSEIICCTTPSLQQLNLQLPLKTKAFFMLDGILSKYFDLIYVHNPVFKP   843
MET-204      SFIRHVNIALIQR.................................................................................................   764
```

logo

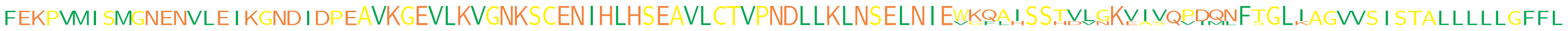

```
MERESTINIB   ..............................................................................................................     0
PF01403      ..............................................................................................................   435
PF07714      ..............................................................................................................     0
PF01437      ..............................................................................................................    40
PF01833.3    ..............................................................................................................   164
PF01833      ..............................................................................................................   232
PF01403.1    ..............................................................................................................   109
PF01833.2    ..............................................................................................................    76
PF01833.1    ..............................................................................................................   240
MET-205      .........................AVKGEVLKVGNKSCENIHLHSEAVLCTVPNDLLKLNSELNIEWKQAISSTVLGKVIVQPDQNFTGLIAGVVSISTALLLLLGFFL    85
MET-201      FEKPVMISMGNENVLEIKGNDIDPEAVKGEVLKVGNKSCENIHLHSEAVLCTVPNDLLKLNSELNIEWKQAISSTVLGKVIVQPDQNFTGLIAGVVSISTALLLLLGFFL   971
MET-206      ..............................................................................................................   190
MET-203      FEKPVMISMGNENVLEIKGNDIDPEAVKGEVLKVGNKSCENIHLHSEAVLCTVPNDLLKLNSELNIEVGFLHSSHDVNKEAS.VIMLFSGLK..................   214
MET-202      FEKPVMISMGNENVLEIKGNDIDPEAVKGEVLKVGNKSCENIHLHSEAVLCTVPNDLLKLNSELNIEWKQAISSTVLGKVIVQPDQNFTGLIAGVVSISTALLLLLGFFL   953
MET-204      ..............................................................................................................   764
```

```
logo
               WLKKRKQIKDLGSELVRYDARVHTPHLDRLVSARSVSPTTEMVSNESVDYRATFPEDQFPNSSQNGSCRQVQYPLTDMSPILTSGDSDISSPLLQNTVHIDLSALNPELV
MERESTINIB    ..............................................................................................................    0
PF01403       ..............................................................................................................  435
PF07714       ..............................................................................................................    0
PF01437       ..............................................................................................................   40
PF01833.3     ..............................................................................................................  164
PF01833       ..............................................................................................................  232
PF01403.1     ..............................................................................................................  109
PF01833.2     ..............................................................................................................   76
PF01833.1     ..............................................................................................................  240
MET-205       WLKKRKQIK...............................................DQFPNSSQNGSCRQVQYPLTDMSPILTSGDSDISSPLLQNTVHIDLSALNPELV  148
MET-201       WLKKRKQIKDLGSELVRYDARVHTPHLDRLVSARSVSPTTEMVSNESVDYRATFPEDQFPNSSQNGSCRQVQYPLTDMSPILTSGDSDISSPLLQNTVHIDLSALNPELV 1081
MET-206       ..............................................................................................................  190
MET-203       ..............................................................................................................  214
MET-202       WLKKRKQIKDLGSELVRYDARVHTPHLDRLVSARSVSPTTEMVSNESVDYRATFPEDQFPNSSQNGSCRQVQYPLTDMSPILTSGDSDISSPLLQNTVHIDLSALNPELV 1063
MET-204       ..............................................................................................................  764

logo
              QAVQHVVIGPSSLIVHFNEVIGRGHFGCVYHGTLLDNDGKKIHCAVKSLNRITDIGEVSQFLTEGIIMKDFSHPNVLSLLGICLRSEGSPLVVLPYMKHGDLRNFIRNET
MERESTINIB    ....................I.......V...............A.K................E..IM..F......................LPYM.............   12
PF01403       ..............................................................................................................  435
PF07714       ...............HFNEVIGRGHFGCVYHGTLLDNDGKKIHCAVKSLNRITDIGEVSQFLTEGIIMKDFSHPNVLSLLGICLRSEGSPLVVLPYMKHGDLRNFIRNET   95
PF01437       ..............................................................................................................   40
PF01833.3     ..............................................................................................................  164
PF01833       ..............................................................................................................  232
PF01403.1     ..............................................................................................................  109
PF01833.2     ..............................................................................................................   76
PF01833.1     ..............................................................................................................  240
MET-205       QAVQHVVIGP....................................................................................................  158
MET-201       QAVQHVVIGPSSLIVHFNEVIGRGHFGCVYHGTLLDNDGKKIHCAVKSLNRITDIGEVSQFLTEGIIMKDFSHPNVLSLLGICLRSEGSPLVVLPYMKHGDLRNFIRNET 1191
MET-206       ..............................................................................................................  190
MET-203       ..............................................................................................................  214
MET-202       QAVQHVVIGPSSLIVHFNEVIGRGHFGCVYHGTLLDNDGKKIHCAVKSLNRITDIGEVSQFLTEGIIMKDFSHPNVLSLLGICLRSEGSPLVVLPYMKHGDLRNFIRNET 1173
MET-204       ..............................................................................................................  764

logo
              HNPTVKDLIGFGLQVAKGMKYLASKKFVHRDLAARNCMLDEKFTVKVADFGLARDMYDKEYYSVHNKTGAKLPVKWMALESLQTQKFTTKSDVWSFGVLLWELMTRGAPP
MERESTINIB    .....................L....F.H........M........VADF............................................................   20
PF01403       ..............................................................................................................  435
PF07714       HNPTVKDLIGFGLQVAKGMKYLASKKFVHRDLAARNCMLDEKFTVKVADFGLARDMYDKEYYSVHNKTGAKLPVKWMALESLQTQKFTTKSDVWSFGVLLWELMTRGAPP  205
PF01437       ..............................................................................................................   40
PF01833.3     ..............................................................................................................  164
PF01833       ..............................................................................................................  232
PF01403.1     ..............................................................................................................  109
PF01833.2     ..............................................................................................................   76
PF01833.1     ..............................................................................................................  240
MET-205       ..............................................................................................................  158
MET-201       HNPTVKDLIGFGLQVAKGMKYLASKKFVHRDLAARNCMLDEKFTVKVADFGLARDMYDKEYYSVHNKTGAKLPVKWMALESLQTQKFTTKSDVWSFGVLLWELMTRGAPP 1301
MET-206       ..............................................................................................................  190
MET-203       ..............................................................................................................  214
MET-202       HNPTVKDLIGFGLQVAKGMKYLASKKFVHRDLAARNCMLDEKFTVKVADFGLARDMYDKEYYSVHNKTGAKLPVKWMALESLQTQKFTTKSDVWSFGVLLWELMTRGAPP 1283
MET-204       ..............................................................................................................  764
```

logo

```
             YPDVNTFDITVYLLQGRRLLQPEYCPDPLYEVMLKCWHPKAEMRPSFSELVSRISAIFSTFIGEHYVHVNATYVNVKCVAPYPSLLSSEDNADDEVDTRPASFWETS
MERESTINIB   ...........................................................................................................    20
PF01403      ...........................................................................................................   435
PF07714      YPDVNTFDITVYLLQGRRLLQPEYCPDPLYEVMLKCWHPKAEMRPSFSELVSR......................................................   258
PF01437      ...........................................................................................................    40
PF01833.3    ...........................................................................................................   164
PF01833      ...........................................................................................................   232
PF01403.1    ...........................................................................................................   109
PF01833.2    ...........................................................................................................    76
PF01833.1    ...........................................................................................................   240
MET-205      ...........................................................................................................   158
MET-201      YPDVNTFDITVYLLQGRRLLQPEYCPDPLYEVMLKCWHPKAEMRPSFSELVSRISAIFSTFIGEHYVHVNATYVNVKCVAPYPSLLSSEDNADDEVDTRPASFWETS  1408
MET-206      ...........................................................................................................   190
MET-203      ...........................................................................................................   214
MET-202      YPDVNTFDITVYLLQGRRLLQPEYCPDPLYEVMLKCWHPKAEMRPSFSELVSRISAIFSTFIGEHYVHVNATYVNVKCVAPYPSLLSSEDNADDEVDTRPASFWETS  1390
MET-204      ...........................................................................................................   764
```

X non conserved
X similar
X $\geq 0\%$ conserved
X $\geq 50\%$ conserved

```
logo
            MKSKSKSLAECFPYDKPLIMKAPAVLAPGILVLLFTLVQRSNGECKEALAKSEMNVNMKYQLPNFTAETPIQNVILHEHHIFLGATNYIYVLNEEDLQKVAEYKTGPVLE
PF-04217903 ..............................................................................................................   0
PF01403     ...........................................................................LHEHHIFLGATNYIYVLNEEDLQKVAEYKTGPVLE  35
PF07714     ..............................................................................................................   0
PF01437     ..............................................................................................................   0
PF01833.3   ..............................................................................................................   0
PF01833     ..............................................................................................................   0
PF01403.1   ...........................................................................LHEHHIFLGATNYIYVLNEEDLQKVAEYKTGPVLE  35
PF01833.2   ..............................................................................................................   0
PF01833.1   ..............................................................................................................   0
MET-205     ..............................................................................................................   0
MET-201     ...................MKAPAVLAPGILVLLFTLVQRSNGECKEALAKSEMNVNMKYQLPNFTAETPIQNVILHEHHIFLGATNYIYVLNEEDLQKVAEYKTGPVLE  91
MET-206     MKSKSKSLAECFPYDKPLIMKAPAVLAPGILVLLFTLVQRSNGECKEALAKSEMNVNMKYQLPNFTAETPIQNVILHEHHIFLGATNYIYVLNEEDLQKVAEYKTGPVLE 110
MET-203     ..............................................................................................................   0
MET-202     ...................MKAPAVLAPGILVLLFTLVQRSNGECKEALAKSEMNVNMKYQLPNFTAETPIQNVILHEHHIFLGATNYIYVLNEEDLQKVAEYKTGPVLE  91
MET-204     ...................MKAPAVLAPGILVLLFTLVQRSNGECKEALAKSEMNVNMKYQLPNFTAETPIQNVILHEHHIFLGATNYIYVLNEEDLQKVAEYKTGPVLE  91

logo
            HPDCFPCQDCSSKANLSGGVWKDNINMALVVDTYYDDQLISCGSVNRGTCQRHVFPHNHTADIQSEVHCIFSPQIEEPSQCPDCVVSALGAKVLSSVKDRFINFFVGNTI
PF-04217903 ..............................................................................................................   0
PF01403     HPDCFPCQDCSSKANLSGGVWKDNINMALVVDTYYDDQLISCGSVNRGTCQRHVFPHNHTADIQSEVHCIFSPQIEEPSQCPDCVVSALGAKVLSSVKDRFINFFVGNTI 145
PF07714     ..............................................................................................................   0
PF01437     ..............................................................................................................   0
PF01833.3   ..............................................................................................................   0
PF01833     ..............................................................................................................   0
PF01403.1   HPDCFPCQDCSSKANLSGGVWKDNINMALVVDTYYDDQLISCGSVNRGTCQRHVFPHNHTADIQSEVHCIFSPQ.................................... 109
PF01833.2   ..............................................................................................................   0
PF01833.1   ..............................................................................................................   0
MET-205     ..............................................................................................................   0
MET-201     HPDCFPCQDCSSKANLSGGVWKDNINMALVVDTYYDDQLISCGSVNRGTCQRHVFPHNHTADIQSEVHCIFSPQIEEPSQCPDCVVSALGAKVLSSVKDRFINFFVGNTI 201
MET-206     HPDCFPCQDCSSKANLSGGVWKDNINMALVVDTYYDDQLISCGSVNRGTCQRHVFPHNHTADIQSEVHCIFSPQIEEPSQ.............................. 190
MET-203     ..............................................................................................................   0
MET-202     HPDCFPCQDCSSKANLSGGVWKDNINMALVVDTYYDDQLISCGSVNRGTCQRHVFPHNHTADIQSEVHCIFSPQIEEPSQCPDCVVSALGAKVLSSVKDRFINFFVGNTI 201
MET-204     HPDCFPCQDCSSKANLSGGVWKDNINMALVVDTYYDDQLISCGSVNRGTCQRHVFPHNHTADIQSEVHCIFSPQIEEPSQCPDCVVSALGAKVLSSVKDRFINFFVGNTI 201

logo
            NSSYFPDHPLHSISVRRLKETKDGFMFLTDQSYIDVLPEFRDSYPIKYVHAFESNNFIYFLTVQRETLDAQTFHTRIIRFCSINSGLHSYMEMPLECILTEKRKKRSTKK
PF-04217903 ..............................................................................................................   0
PF01403     NSSYFPDHPLHSISVRRLKETKDGFMFLTDQSYIDVLPEFRDSYPIKYVHAFESNNFIYFLTVQRETLDAQTFHTRIIRFCSINSGLHSYMEMPLECILTEKRKKRSTKK 255
PF07714     ..............................................................................................................   0
PF01437     ..............................................................................................................   0
PF01833.3   ..............................................................................................................   0
PF01833     ..............................................................................................................   0
PF01403.1   .............................................................................................................. 109
PF01833.2   ..............................................................................................................   0
PF01833.1   ..............................................................................................................   0
MET-205     ..............................................................................................................   0
MET-201     NSSYFPDHPLHSISVRRLKETKDGFMFLTDQSYIDVLPEFRDSYPIKYVHAFESNNFIYFLTVQRETLDAQTFHTRIIRFCSINSGLHSYMEMPLECILTEKRKKRSTKK 311
MET-206     .............................................................................................................. 190
MET-203     ..............................................................................................................   0
MET-202     NSSYFPDHPLHSISVRRLKETKDGFMFLTDQSYIDVLPEFRDSYPIKYVHAFESNNFIYFLTVQRETLDAQTFHTRIIRFCSINSGLHSYMEMPLECILTEKRKKRSTKK 311
MET-204     NSSYFPDHPLHSISVRRLKETKDGFMFLTDQSYIDVLPEFRDSYPIKYVHAFESNNFIYFLTVQRETLDAQTFHTRIIRFCSINSGLHSYMEMPLECILTEKRKKRSTKK 311
```

```
logo
             EVFNILQAAYVSKPGAQLARQIGASLNDDILFGVFAQSKPDSAEPMDRSAMCAFPIKYVNDFFNKIVNKNNVRCLQHFYGPNHEHCFNRTLLRNSSGCEARRDEYRTEFT
PF-04217903  ..............................................................................................................    0
PF01403      EVFNILQAAYVSKPGAQLARQIGASLNDDILFGVFAQSKPDSAEPMDRSAMCAFPIKYVNDFFNKIVNKNNVRCLQHFYGPNHEHCFNRTLLRNSSGCEARRDEYRTEFT  365
PF07714      ..............................................................................................................    0
PF01437      ..............................................................................................................    0
PF01833.3    ..............................................................................................................    0
PF01833      ..............................................................................................................    0
PF01403.1    ..............................................................................................................  109
PF01833.2    ..............................................................................................................    0
PF01833.1    ..............................................................................................................    0
MET-205      ..............................................................................................................    0
MET-201      EVFNILQAAYVSKPGAQLARQIGASLNDDILFGVFAQSKPDSAEPMDRSAMCAFPIKYVNDFFNKIVNKNNVRCLQHFYGPNHEHCFNRTLLRNSSGCEARRDEYRTEFT  421
MET-206      ..............................................................................................................  190
MET-203      ..............................................................................................................    0
MET-202      EVFNILQAAYVSKPGAQLARQIGASLNDDILFGVFAQSKPDSAEPMDRSAMCAFPIKYVNDFFNKIVNKNNVRCLQHFYGPNHEHCFNRTLLRNSSGCEARRDEYRTEFT  421
MET-204      EVFNILQAAYVSKPGAQLARQIGASLNDDILFGVFAQSKPDSAEPMDRSAMCAFPIKYVNDFFNKIVNKNNVRCLQHFYGPNHEHCFNRTLLRNSSGCEARRDEYRTEFT  421

logo
             TALQRVDLFMGQFSEVLLTSISTFIKGDLTIANLGTSEGRFMQVVVSRSGPSTPHVNFLLDSHPVSPEVIVEHTLNQNGYTLVITGKKITKIPLNGLGCRHFQSCSQCLS
PF-04217903  ..............................................................................................................    0
PF01403      TALQRVDLFMGQFSEVLLTSISTFIKGDLTIANLGTSEGRFMQVVVSRSGPSTPHVNFLLDSHPVSPEVI........................................  435
PF07714      ..............................................................................................................    0
PF01437      ..................................................................................................CRHFQSCSQCLS   12
PF01833.3    ..............................................................................................................    0
PF01833      ..............................................................................................................    0
PF01403.1    ..............................................................................................................  109
PF01833.2    ..............................................................................................................    0
PF01833.1    ..............................................................................................................    0
MET-205      ..............................................................................................................    0
MET-201      TALQRVDLFMGQFSEVLLTSISTFIKGDLTIANLGTSEGRFMQVVVSRSGPSTPHVNFLLDSHPVSPEVIVEHTLNQNGYTLVITGKKITKIPLNGLGCRHFQSCSQCLS  531
MET-206      ..............................................................................................................  190
MET-203      ..............................................................................................................    0
MET-202      TALQRVDLFMGQFSEVLLTSISTFIKGDLTIANLGTSEGRFMQVVVSRSGPSTPHVNFLLDSHPVSPEVIVEHTLNQNGYTLVITGKKITKIPLNGLGCRHFQSCSQCLS  531
MET-204      TALQRVDLFMGQFSEVLLTSISTFIKGDLTIANLGTSEGRFMQVVVSRSGPSTPHVNFLLDSHPVSPEVIVEHTLNQNGYTLVITGKKITKIPLNGLGCRHFQSCSQCLS  531

logo
             APPFVQCGWCHDKCVRSEECLSGTWTQQICLPAIYKVFPNSAPLEGGTRLTICGWDFGFRRNNKFDLKKTRVLLGNESCTLTLSESTMNTLKCTVGPAMNKHFNMSIIIS
PF-04217903  ..............................................................................................................    0
PF01403      ..............................................................................................................  435
PF07714      ..............................................................................................................    0
PF01437      APPFVQCGWCHDKCVRSEECLSGTWTQQ..................................................................................   40
PF01833.3    ...............................PAIYKVFPNSAPLEGGTRLTICGWDFGFRRNNKFDLKKTRVLLGNESCTLTLSESTMNTLKCTVGPAMNKHFNMSIIIS   79
PF01833      ...............................PAIYKVFPNSAPLEGGTRLTICGWDFGFRRNNKFDLKKTRVLLGNESCTLTLSESTMNTLKCTVGPAMNKHFNMSIIIS   79
PF01403.1    ..............................................................................................................  109
PF01833.2    ..............................................................................................................    0
PF01833.1    ...............................PAIYKVFPNSAPLEGGTRLTICGWDFGFRRNNKFDLKKTRVLLGNESCTLTLSESTMNTLKCTVGPAMNKHFNMSIIIS   79
MET-205      ..............................................................................................................    0
MET-201      APPFVQCGWCHDKCVRSEECLSGTWTQQICLPAIYKVFPNSAPLEGGTRLTICGWDFGFRRNNKFDLKKTRVLLGNESCTLTLSESTMNTLKCTVGPAMNKHFNMSIIIS  641
MET-206      ..............................................................................................................  190
MET-203      ..............................................................................................................    0
MET-202      APPFVQCGWCHDKCVRSEECLSGTWTQQICLPAIYKVFPNSAPLEGGTRLTICGWDFGFRRNNKFDLKKTRVLLGNESCTLTLSESTMNTLKCTVGPAMNKHFNMSIIIS  641
MET-204      APPFVQCGWCHDKCVRSEECLSGTWTQQICLPAIYKVFPNSAPLEGGTRLTICGWDFGFRRNNKFDLKKTRVLLGNESCTLTLSESTMNTLKCTVGPAMNKHFNMSIIIS  641
```

```
logo
             NGHGTTQYSTFSYVDPVITSISPKYGPMAGGTLLTLTGNYLNSGNSRHISIGGKTCTLKSVSNSILECYTPAQTISTEFAVKLKIDLANRETSIFSYREDPIVYEIHPTK
PF-04217903  ..............................................................................................................    0
PF01403      ..............................................................................................................  435
PF07714      ..............................................................................................................    0
PF01437      ..............................................................................................................   40
PF01833.3    NGHGTTQYSTFSY..PVITSISPKYGPMAGGTLLTLTGNYLNSGNSRHISIGGKTCTLKSVSNSILECYTPAQTISTEFAVKLKIDL.......................  164
PF01833      NGHGTTQYSTFSY..PVITSISPKYGPMAGGTLLTLTGNYLNSGNSRHISIGGKTCTLKSVSNSILECYTPAQTISTEFAVKLKIDL.......................  164
PF01403.1    ..............................................................................................................  109
PF01833.2    ....................................................................................................PIVYEIHPTK   10
PF01833.1    NGHGTTQYSTFSY..PVITSISPKYGPMAGGTLLTLTGNYLNSGNSRHISIGGKTCTLKSVSNSILECYTPAQTISTEFAVKLKIDL.............PIVYEIHPTK  174
MET-205      ..............................................................................................................    0
MET-201      NGHGTTQYSTFSYVDPVITSISPKYGPMAGGTLLTLTGNYLNSGNSRHISIGGKTCTLKSVSNSILECYTPAQTISTEFAVKLKIDLANRETSIFSYREDPIVYEIHPTK  751
MET-206      ..............................................................................................................  190
MET-203      ...............................................................................AVKLKIDLANRETSIFSYREDPIVYEIHPTK   31
MET-202      NGHGTTQYSTFSYVDPVITSISPKYGPMAGGTLLTLTGNYLNSGNSRHISIGGKTCTLKSVSNSILECYTPAQTISTEFAVKLKIDLANRETSIFSYREDPIVYEIHPTK  751
MET-204      NGHGTTQYSTFSYVDPVITSISPKYGPMAGGTLLTLTGNYLNSGNSRHISIGGKTCTLKSVSNSILECYTPAQTISTEFAVKLKIDLANRETSIFSYREDPIVYEIHPTK  751

logo
             SFI[illegible]LFCFASGGSTITGVGKNLNSVSVPRMVINVHEAGRNFTVACQHRSNSEIICCTTPSLQQLNLQLPLKTKAFFMLDGILSKYFDLIYVHNPVFKP
PF-04217903  ..............................................................................................................    0
PF01403      ..............................................................................................................  435
PF07714      ..............................................................................................................    0
PF01437      ..............................................................................................................   40
PF01833.3    ..............................................................................................................  164
PF01833      ................LFCFASGGSTITGVGKNLNSVSVPRMVINVHEAGRNFTVACQHRSNSEIICCTTPSLQQLNLQLPLKT..........................  232
PF01403.1    ..............................................................................................................  109
PF01833.2    SFI..................SGGSTITGVGKNLNSVSVPRMVINVHEAGRNFTVACQHRSNSEIICCTTPSLQQLNLQLPLKT..........................   76
PF01833.1    SFI..................SGGSTITGVGKNLNSVSVPRMVINVHEAGRNFTVACQHRSNSEIICCTTPSLQQLNLQLPLKT..........................  240
MET-205      ..............................................................................................................    0
MET-201      SFISTWWKEPLNIVSFLFCFASGGSTITGVGKNLNSVSVPRMVINVHEAGRNFTVACQHRSNSEIICCTTPSLQQLNLQLPLKTKAFFMLDGILSKYFDLIYVHNPVFKP  861
MET-206      ..............................................................................................................  190
MET-203      SFI..................SGGSTITGVGKNLNSVSVPRMVINVHEAGRNFTVACQHRSNSEIICCTTPSLQQLNLQLPLKTKAFFMLDGILSKYFDLIYVHNPVFKP  123
MET-202      SFI..................SGGSTITGVGKNLNSVSVPRMVINVHEAGRNFTVACQHRSNSEIICCTTPSLQQLNLQLPLKTKAFFMLDGILSKYFDLIYVHNPVFKP  843
MET-204      SFIRHVNIALIQR.................................................................................................  764

logo
             FEKPVMISMGNENVLEIKGNDIDPEAVKGEVLKVGNKSCENIHLHSEAVLCTVPNDLLKLNSELNIEWKQAISSTVLGKVIVQPDQNFTGLIAGVVSISTALLLLLGFFL
PF-04217903  ..............................................................................................................    0
PF01403      ..............................................................................................................  435
PF07714      ..............................................................................................................    0
PF01437      ..............................................................................................................   40
PF01833.3    ..............................................................................................................  164
PF01833      ..............................................................................................................  232
PF01403.1    ..............................................................................................................  109
PF01833.2    ..............................................................................................................   76
PF01833.1    ..............................................................................................................  240
MET-205      .........................AVKGEVLKVGNKSCENIHLHSEAVLCTVPNDLLKLNSELNIEWKQAISSTVLGKVIVQPDQNFTGLIAGVVSISTALLLLLGFFL   85
MET-201      FEKPVMISMGNENVLEIKGNDIDPEAVKGEVLKVGNKSCENIHLHSEAVLCTVPNDLLKLNSELNIEWKQAISSTVLGKVIVQPDQNFTGLIAGVVSISTALLLLLGFFL  971
MET-206      ..............................................................................................................  190
MET-203      FEKPVMISMGNENVLEIKGNDIDPEAVKGEVLKVGNKSCENIHLHSEAVLCTVPNDLLKLNSELNIEVGFLHSSHDVNKEAS.VIMLFSGLK..................  214
MET-202      FEKPVMISMGNENVLEIKGNDIDPEAVKGEVLKVGNKSCENIHLHSEAVLCTVPNDLLKLNSELNIEWKQAISSTVLGKVIVQPDQNFTGLIAGVVSISTALLLLLGFFL  953
MET-204      ..............................................................................................................  764
```

```
logo
              WLKKRKQIKDLGSELVRYDARVHTPHLDRLVSARSVSPTTEMVSNESVDYRATFPEDQFPNSSQNGSCRQVQYPLTDMSPILTSGDSDISSPLLQNTVHIDLSALNPELV
PF-04217903   ..............................................................................................................    0
PF01403       ..............................................................................................................  435
PF07714       ..............................................................................................................    0
PF01437       ..............................................................................................................   40
PF01833.3     ..............................................................................................................  164
PF01833       ..............................................................................................................  232
PF01403.1     ..............................................................................................................  109
PF01833.2     ..............................................................................................................   76
PF01833.1     ..............................................................................................................  240
MET-205       WLKKRKQIK...............................................DQFPNSSQNGSCRQVQYPLTDMSPILTSGDSDISSPLLQNTVHIDLSALNPELV  148
MET-201       WLKKRKQIKDLGSELVRYDARVHTPHLDRLVSARSVSPTTEMVSNESVDYRATFPEDQFPNSSQNGSCRQVQYPLTDMSPILTSGDSDISSPLLQNTVHIDLSALNPELV 1081
MET-206       ..............................................................................................................  190
MET-203       ..............................................................................................................  214
MET-202       WLKKRKQIKDLGSELVRYDARVHTPHLDRLVSARSVSPTTEMVSNESVDYRATFPEDQFPNSSQNGSCRQVQYPLTDMSPILTSGDSDISSPLLQNTVHIDLSALNPELV 1063
MET-204       ..............................................................................................................  764

logo
              QAVQHVVIGPSSLIVHFNEVIGRGHFGCVYHGTLLDNDGKKIHCAVKSLNRITDIGEVSQFLTEGIIMKDFSHPNVLSLLGICLRSEGSPLVVLPYMKHGDLRNFIRNET
PF-04217903   ....................I.......V...............A................................................LPYM...D.........    8
PF01403       ..............................................................................................................  435
PF07714       ...............HFNEVIGRGHFGCVYHGTLLDNDGKKIHCAVKSLNRITDIGEVSQFLTEGIIMKDFSHPNVLSLLGICLRSEGSPLVVLPYMKHGDLRNFIRNET   95
PF01437       ..............................................................................................................   40
PF01833.3     ..............................................................................................................  164
PF01833       ..............................................................................................................  232
PF01403.1     ..............................................................................................................  109
PF01833.2     ..............................................................................................................   76
PF01833.1     ..............................................................................................................  240
MET-205       QAVQHVVIGP....................................................................................................  158
MET-201       QAVQHVVIGPSSLIVHFNEVIGRGHFGCVYHGTLLDNDGKKIHCAVKSLNRITDIGEVSQFLTEGIIMKDFSHPNVLSLLGICLRSEGSPLVVLPYMKHGDLRNFIRNET 1191
MET-206       ..............................................................................................................  190
MET-203       ..............................................................................................................  214
MET-202       QAVQHVVIGPSSLIVHFNEVIGRGHFGCVYHGTLLDNDGKKIHCAVKSLNRITDIGEVSQFLTEGIIMKDFSHPNVLSLLGICLRSEGSPLVVLPYMKHGDLRNFIRNET 1173
MET-204       ..............................................................................................................  764

logo
              HNPTVKDLIGFGLQVAKGMKYLASKKFVHRDLAARNCMLDEKFTVKVADFGLARDMYDKEYYSVHNKTGAKLPVKWMALESLQTQKFTTKSDVWSFGVLLWELMTRGAPP
PF-04217903   ..................................R..M.........AD...A...YD....................................................   15
PF01403       ..............................................................................................................  435
PF07714       HNPTVKDLIGFGLQVAKGMKYLASKKFVHRDLAARNCMLDEKFTVKVADFGLARDMYDKEYYSVHNKTGAKLPVKWMALESLQTQKFTTKSDVWSFGVLLWELMTRGAPP  205
PF01437       ..............................................................................................................   40
PF01833.3     ..............................................................................................................  164
PF01833       ..............................................................................................................  232
PF01403.1     ..............................................................................................................  109
PF01833.2     ..............................................................................................................   76
PF01833.1     ..............................................................................................................  240
MET-205       ..............................................................................................................  158
MET-201       HNPTVKDLIGFGLQVAKGMKYLASKKFVHRDLAARNCMLDEKFTVKVADFGLARDMYDKEYYSVHNKTGAKLPVKWMALESLQTQKFTTKSDVWSFGVLLWELMTRGAPP 1301
MET-206       ..............................................................................................................  190
MET-203       ..............................................................................................................  214
MET-202       HNPTVKDLIGFGLQVAKGMKYLASKKFVHRDLAARNCMLDEKFTVKVADFGLARDMYDKEYYSVHNKTGAKLPVKWMALESLQTQKFTTKSDVWSFGVLLWELMTRGAPP 1283
MET-204       ..............................................................................................................  764
```

logo

```
              YPDVNTFDITVYLLQGRRLLQPEYCPDPLYEVMLKCWHPKAEMRPSFSELVSRISAIFSTFIGEHYVHVNATYVNVKCVAPYPSLLSSEDNADDEVDTRPASFWETS
PF-04217903   ...........................................................................................................   15
PF01403       ...........................................................................................................  435
PF07714       YPDVNTFDITVYLLQGRRLLQPEYCPDPLYEVMLKCWHPKAEMRPSFSELVSR......................................................  258
PF01437       ...........................................................................................................   40
PF01833.3     ...........................................................................................................  164
PF01833       ...........................................................................................................  232
PF01403.1     ...........................................................................................................  109
PF01833.2     ...........................................................................................................   76
PF01833.1     ...........................................................................................................  240
MET-205       ...........................................................................................................  158
MET-201       YPDVNTFDITVYLLQGRRLLQPEYCPDPLYEVMLKCWHPKAEMRPSFSELVSRISAIFSTFIGEHYVHVNATYVNVKCVAPYPSLLSSEDNADDEVDTRPASFWETS 1408
MET-206       ...........................................................................................................  190
MET-203       ...........................................................................................................  214
MET-202       YPDVNTFDITVYLLQGRRLLQPEYCPDPLYEVMLKCWHPKAEMRPSFSELVSRISAIFSTFIGEHYVHVNATYVNVKCVAPYPSLLSSEDNADDEVDTRPASFWETS 1390
MET-204       ...........................................................................................................  764
```

X non conserved

X similar

X ≥ 0% conserved

X ≥ 50% conserved

```
logo
             MKSKSKSLAECFPYDKPLIMKAPAVLAPGILVLLFTLVQRSNGECKEALAKSEMNVNMKYQLPNFTAETPIQNVILHEHHIFLGATNYIYVLNEEDLQKVAEYKTGPVLE
SGX-523      ..............................................................................................................     0
PF01403      ...........................................................................LHEHHIFLGATNYIYVLNEEDLQKVAEYKTGPVLE    35
PF07714      ..............................................................................................................     0
PF01437      ..............................................................................................................     0
PF01833.3    ..............................................................................................................     0
PF01833      ..............................................................................................................     0
PF01403.1    ...........................................................................LHEHHIFLGATNYIYVLNEEDLQKVAEYKTGPVLE    35
PF01833.2    ..............................................................................................................     0
PF01833.1    ..............................................................................................................     0
MET-205      ..............................................................................................................     0
MET-201      ...................MKAPAVLAPGILVLLFTLVQRSNGECKEALAKSEMNVNMKYQLPNFTAETPIQNVILHEHHIFLGATNYIYVLNEEDLQKVAEYKTGPVLE    91
MET-206      MKSKSKSLAECFPYDKPLIMKAPAVLAPGILVLLFTLVQRSNGECKEALAKSEMNVNMKYQLPNFTAETPIQNVILHEHHIFLGATNYIYVLNEEDLQKVAEYKTGPVLE   110
MET-203      ..............................................................................................................     0
MET-202      ...................MKAPAVLAPGILVLLFTLVQRSNGECKEALAKSEMNVNMKYQLPNFTAETPIQNVILHEHHIFLGATNYIYVLNEEDLQKVAEYKTGPVLE    91
MET-204      ...................MKAPAVLAPGILVLLFTLVQRSNGECKEALAKSEMNVNMKYQLPNFTAETPIQNVILHEHHIFLGATNYIYVLNEEDLQKVAEYKTGPVLE    91

logo
             HPDCFPCQDCSSKANLSGGVWKDNINMALVVDTYYDDQLISCGSVNRGTCQRHVFPHNHTADIQSEVHCIFSPQIEEPSQCPDCVVSALGAKVLSSVKDRFINFFVGNTI
SGX-523      ..............................................................................................................     0
PF01403      HPDCFPCQDCSSKANLSGGVWKDNINMALVVDTYYDDQLISCGSVNRGTCQRHVFPHNHTADIQSEVHCIFSPQIEEPSQCPDCVVSALGAKVLSSVKDRFINFFVGNTI   145
PF07714      ..............................................................................................................     0
PF01437      ..............................................................................................................     0
PF01833.3    ..............................................................................................................     0
PF01833      ..............................................................................................................     0
PF01403.1    HPDCFPCQDCSSKANLSGGVWKDNINMALVVDTYYDDQLISCGSVNRGTCQRHVFPHNHTADIQSEVHCIFSPQ....................................   109
PF01833.2    ..............................................................................................................     0
PF01833.1    ..............................................................................................................     0
MET-205      ..............................................................................................................     0
MET-201      HPDCFPCQDCSSKANLSGGVWKDNINMALVVDTYYDDQLISCGSVNRGTCQRHVFPHNHTADIQSEVHCIFSPQIEEPSQCPDCVVSALGAKVLSSVKDRFINFFVGNTI   201
MET-206      HPDCFPCQDCSSKANLSGGVWKDNINMALVVDTYYDDQLISCGSVNRGTCQRHVFPHNHTADIQSEVHCIFSPQIEEPSQ..............................   190
MET-203      ..............................................................................................................     0
MET-202      HPDCFPCQDCSSKANLSGGVWKDNINMALVVDTYYDDQLISCGSVNRGTCQRHVFPHNHTADIQSEVHCIFSPQIEEPSQCPDCVVSALGAKVLSSVKDRFINFFVGNTI   201
MET-204      HPDCFPCQDCSSKANLSGGVWKDNINMALVVDTYYDDQLISCGSVNRGTCQRHVFPHNHTADIQSEVHCIFSPQIEEPSQCPDCVVSALGAKVLSSVKDRFINFFVGNTI   201

logo
             NSSYFPDHPLHSISVRRLKETKDGFMFLTDQSYIDVLPEFRDSYPIKYVHAFESNNFIYFLTVQRETLDAQTFHTRIIRFCSINSGLHSYMEMPLECILTEKRKKRSTKK
SGX-523      ..............................................................................................................     0
PF01403      NSSYFPDHPLHSISVRRLKETKDGFMFLTDQSYIDVLPEFRDSYPIKYVHAFESNNFIYFLTVQRETLDAQTFHTRIIRFCSINSGLHSYMEMPLECILTEKRKKRSTKK   255
PF07714      ..............................................................................................................     0
PF01437      ..............................................................................................................     0
PF01833.3    ..............................................................................................................     0
PF01833      ..............................................................................................................     0
PF01403.1    ..............................................................................................................   109
PF01833.2    ..............................................................................................................     0
PF01833.1    ..............................................................................................................     0
MET-205      ..............................................................................................................     0
MET-201      NSSYFPDHPLHSISVRRLKETKDGFMFLTDQSYIDVLPEFRDSYPIKYVHAFESNNFIYFLTVQRETLDAQTFHTRIIRFCSINSGLHSYMEMPLECILTEKRKKRSTKK   311
MET-206      ..............................................................................................................   190
MET-203      ..............................................................................................................     0
MET-202      NSSYFPDHPLHSISVRRLKETKDGFMFLTDQSYIDVLPEFRDSYPIKYVHAFESNNFIYFLTVQRETLDAQTFHTRIIRFCSINSGLHSYMEMPLECILTEKRKKRSTKK   311
MET-204      NSSYFPDHPLHSISVRRLKETKDGFMFLTDQSYIDVLPEFRDSYPIKYVHAFESNNFIYFLTVQRETLDAQTFHTRIIRFCSINSGLHSYMEMPLECILTEKRKKRSTKK   311
```

```
logo
            EVFNILQAAYVSKPGAQLARQIGASLNDDILFGVFAQSKPDSAEPMDRSAMCAFPIKYVNDFFNKIVNKNNVRCLQHFYGPNHEHCFNRTLLRNSSGCEARRDEYRTEFT
SGX-523     ..............................................................................................................   0
PF01403     EVFNILQAAYVSKPGAQLARQIGASLNDDILFGVFAQSKPDSAEPMDRSAMCAFPIKYVNDFFNKIVNKNNVRCLQHFYGPNHEHCFNRTLLRNSSGCEARRDEYRTEFT 365
PF07714     ..............................................................................................................   0
PF01437     ..............................................................................................................   0
PF01833.3   ..............................................................................................................   0
PF01833     ..............................................................................................................   0
PF01403.1   .............................................................................................................. 109
PF01833.2   ..............................................................................................................   0
PF01833.1   ..............................................................................................................   0
MET-205     ..............................................................................................................   0
MET-201     EVFNILQAAYVSKPGAQLARQIGASLNDDILFGVFAQSKPDSAEPMDRSAMCAFPIKYVNDFFNKIVNKNNVRCLQHFYGPNHEHCFNRTLLRNSSGCEARRDEYRTEFT 421
MET-206     .............................................................................................................. 190
MET-203     ..............................................................................................................   0
MET-202     EVFNILQAAYVSKPGAQLARQIGASLNDDILFGVFAQSKPDSAEPMDRSAMCAFPIKYVNDFFNKIVNKNNVRCLQHFYGPNHEHCFNRTLLRNSSGCEARRDEYRTEFT 421
MET-204     EVFNILQAAYVSKPGAQLARQIGASLNDDILFGVFAQSKPDSAEPMDRSAMCAFPIKYVNDFFNKIVNKNNVRCLQHFYGPNHEHCFNRTLLRNSSGCEARRDEYRTEFT 421

logo
            TALQRVDLFMGQFSEVLLTSISTFIKGDLTIANLGTSEGRFMQVVVSRSGPSTPHVNFLLDSHPVSPEVIVEHTLNQNGYTLVITGKKITKIPLNGLGCRHFQSCSQCLS
SGX-523     ..............................................................................................................   0
PF01403     TALQRVDLFMGQFSEVLLTSISTFIKGDLTIANLGTSEGRFMQVVVSRSGPSTPHVNFLLDSHPVSPEVI........................................ 435
PF07714     ..............................................................................................................   0
PF01437     ..................................................................................................CRHFQSCSQCLS  12
PF01833.3   ..............................................................................................................   0
PF01833     ..............................................................................................................   0
PF01403.1   .............................................................................................................. 109
PF01833.2   ..............................................................................................................   0
PF01833.1   ..............................................................................................................   0
MET-205     ..............................................................................................................   0
MET-201     TALQRVDLFMGQFSEVLLTSISTFIKGDLTIANLGTSEGRFMQVVVSRSGPSTPHVNFLLDSHPVSPEVIVEHTLNQNGYTLVITGKKITKIPLNGLGCRHFQSCSQCLS 531
MET-206     .............................................................................................................. 190
MET-203     ..............................................................................................................   0
MET-202     TALQRVDLFMGQFSEVLLTSISTFIKGDLTIANLGTSEGRFMQVVVSRSGPSTPHVNFLLDSHPVSPEVIVEHTLNQNGYTLVITGKKITKIPLNGLGCRHFQSCSQCLS 531
MET-204     TALQRVDLFMGQFSEVLLTSISTFIKGDLTIANLGTSEGRFMQVVVSRSGPSTPHVNFLLDSHPVSPEVIVEHTLNQNGYTLVITGKKITKIPLNGLGCRHFQSCSQCLS 531

logo
            APPFVQCGWCHDKCVRSEECLSGTWTQQICLPAIYKVFPNSAPLEGGTRLTICGWDFGFRRNNKFDLKKTRVLLGNESCTLTLSESTMNTLKCTVGPAMNKHFNMSIIIS
SGX-523     ..............................................................................................................   0
PF01403     .............................................................................................................. 435
PF07714     ..............................................................................................................   0
PF01437     APPFVQCGWCHDKCVRSEECLSGTWTQQ..................................................................................  40
PF01833.3   ...............................PAIYKVFPNSAPLEGGTRLTICGWDFGFRRNNKFDLKKTRVLLGNESCTLTLSESTMNTLKCTVGPAMNKHFNMSIIIS  79
PF01833     ...............................PAIYKVFPNSAPLEGGTRLTICGWDFGFRRNNKFDLKKTRVLLGNESCTLTLSESTMNTLKCTVGPAMNKHFNMSIIIS  79
PF01403.1   .............................................................................................................. 109
PF01833.2   ..............................................................................................................   0
PF01833.1   ...............................PAIYKVFPNSAPLEGGTRLTICGWDFGFRRNNKFDLKKTRVLLGNESCTLTLSESTMNTLKCTVGPAMNKHFNMSIIIS  79
MET-205     ..............................................................................................................   0
MET-201     APPFVQCGWCHDKCVRSEECLSGTWTQQICLPAIYKVFPNSAPLEGGTRLTICGWDFGFRRNNKFDLKKTRVLLGNESCTLTLSESTMNTLKCTVGPAMNKHFNMSIIIS 641
MET-206     .............................................................................................................. 190
MET-203     ..............................................................................................................   0
MET-202     APPFVQCGWCHDKCVRSEECLSGTWTQQICLPAIYKVFPNSAPLEGGTRLTICGWDFGFRRNNKFDLKKTRVLLGNESCTLTLSESTMNTLKCTVGPAMNKHFNMSIIIS 641
MET-204     APPFVQCGWCHDKCVRSEECLSGTWTQQICLPAIYKVFPNSAPLEGGTRLTICGWDFGFRRNNKFDLKKTRVLLGNESCTLTLSESTMNTLKCTVGPAMNKHFNMSIIIS 641
```

```
logo
```

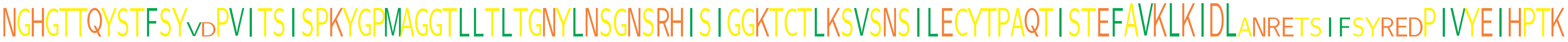

```
SGX-523     ..............................................................................................................    0
PF01403     ..............................................................................................................  435
PF07714     ..............................................................................................................    0
PF01437     ..............................................................................................................   40
PF01833.3   NGHGTTQYSTFSY..PVITSISPKYGPMAGGTLLTLTGNYLNSGNSRHISIGGKTCTLKSVSNSILECYTPAQTISTEFAVKLKIDL.......................  164
PF01833     NGHGTTQYSTFSY..PVITSISPKYGPMAGGTLLTLTGNYLNSGNSRHISIGGKTCTLKSVSNSILECYTPAQTISTEFAVKLKIDL.......................  164
PF01403.1   ..............................................................................................................  109
PF01833.2   ....................................................................................................PIVYEIHPTK   10
PF01833.1   NGHGTTQYSTFSY..PVITSISPKYGPMAGGTLLTLTGNYLNSGNSRHISIGGKTCTLKSVSNSILECYTPAQTISTEFAVKLKIDL.............PIVYEIHPTK  174
MET-205     ..............................................................................................................    0
MET-201     NGHGTTQYSTFSYVDPVITSISPKYGPMAGGTLLTLTGNYLNSGNSRHISIGGKTCTLKSVSNSILECYTPAQTISTEFAVKLKIDLANRETSIFSYREDPIVYEIHPTK  751
MET-206     ..............................................................................................................  190
MET-203     ................................................................................AVKLKIDLANRETSIFSYREDPIVYEIHPTK   31
MET-202     NGHGTTQYSTFSYVDPVITSISPKYGPMAGGTLLTLTGNYLNSGNSRHISIGGKTCTLKSVSNSILECYTPAQTISTEFAVKLKIDLANRETSIFSYREDPIVYEIHPTK  751
MET-204     NGHGTTQYSTFSYVDPVITSISPKYGPMAGGTLLTLTGNYLNSGNSRHISIGGKTCTLKSVSNSILECYTPAQTISTEFAVKLKIDLANRETSIFSYREDPIVYEIHPTK  751
```

```
logo
```

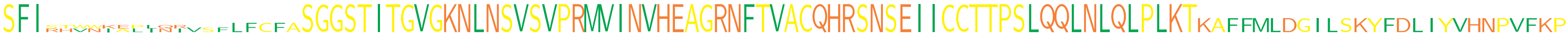

```
SGX-523     ..............................................................................................................    0
PF01403     ..............................................................................................................  435
PF07714     ..............................................................................................................    0
PF01437     ..............................................................................................................   40
PF01833.3   ..............................................................................................................  164
PF01833     ................LFCFASGGSTITGVGKNLNSVSVPRMVINVHEAGRNFTVACQHRSNSEIICCTTPSLQQLNLQLPLKT..........................  232
PF01403.1   ..............................................................................................................  109
PF01833.2   SFI..................SGGSTITGVGKNLNSVSVPRMVINVHEAGRNFTVACQHRSNSEIICCTTPSLQQLNLQLPLKT..........................   76
PF01833.1   SFI..................SGGSTITGVGKNLNSVSVPRMVINVHEAGRNFTVACQHRSNSEIICCTTPSLQQLNLQLPLKT..........................  240
MET-205     ..............................................................................................................    0
MET-201     SFISTWWKEPLNIVSFLFCFASGGSTITGVGKNLNSVSVPRMVINVHEAGRNFTVACQHRSNSEIICCTTPSLQQLNLQLPLKTKAFFMLDGILSKYFDLIYVHNPVFKP  861
MET-206     ..............................................................................................................  190
MET-203     SFI..................SGGSTITGVGKNLNSVSVPRMVINVHEAGRNFTVACQHRSNSEIICCTTPSLQQLNLQLPLKTKAFFMLDGILSKYFDLIYVHNPVFKP  123
MET-202     SFI..................SGGSTITGVGKNLNSVSVPRMVINVHEAGRNFTVACQHRSNSEIICCTTPSLQQLNLQLPLKTKAFFMLDGILSKYFDLIYVHNPVFKP  843
MET-204     SFIRHVNIALIQR.................................................................................................  764
```

```
logo
```

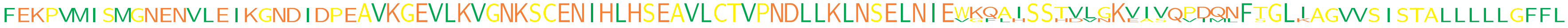

```
SGX-523     ..............................................................................................................    0
PF01403     ..............................................................................................................  435
PF07714     ..............................................................................................................    0
PF01437     ..............................................................................................................   40
PF01833.3   ..............................................................................................................  164
PF01833     ..............................................................................................................  232
PF01403.1   ..............................................................................................................  109
PF01833.2   ..............................................................................................................   76
PF01833.1   ..............................................................................................................  240
MET-205     .........................AVKGEVLKVGNKSCENIHLHSEAVLCTVPNDLLKLNSELNIEWKQAISSTVLGKVIVQPDQNFTGLIAGVVSISTALLLLLGFFL   85
MET-201     FEKPVMISMGNENVLEIKGNDIDPEAVKGEVLKVGNKSCENIHLHSEAVLCTVPNDLLKLNSELNIEWKQAISSTVLGKVIVQPDQNFTGLIAGVVSISTALLLLLGFFL  971
MET-206     ..............................................................................................................  190
MET-203     FEKPVMISMGNENVLEIKGNDIDPEAVKGEVLKVGNKSCENIHLHSEAVLCTVPNDLLKLNSELNIEVGFLHSSHDVNKEAS.VIMLFSGLK.................  214
MET-202     FEKPVMISMGNENVLEIKGNDIDPEAVKGEVLKVGNKSCENIHLHSEAVLCTVPNDLLKLNSELNIEWKQAISSTVLGKVIVQPDQNFTGLIAGVVSISTALLLLLGFFL  953
MET-204     ..............................................................................................................  764
```

```
logo
            WLKKRKQIKDLGSELVRYDARVHTPHLDRLVSARSVSPTTEMVSNESVDYRATFPEDQFPNSSQNGSCRQVQYPLTDMSPILTSGDSDISSPLLQNTVHIDLSALNPELV
SGX-523     ..............................................................................................................     0
PF01403     ..............................................................................................................   435
PF07714     ..............................................................................................................     0
PF01437     ..............................................................................................................    40
PF01833.3   ..............................................................................................................   164
PF01833     ..............................................................................................................   232
PF01403.1   ..............................................................................................................   109
PF01833.2   ..............................................................................................................    76
PF01833.1   ..............................................................................................................   240
MET-205     WLKKRKQIK...............................................DQFPNSSQNGSCRQVQYPLTDMSPILTSGDSDISSPLLQNTVHIDLSALNPELV   148
MET-201     WLKKRKQIKDLGSELVRYDARVHTPHLDRLVSARSVSPTTEMVSNESVDYRATFPEDQFPNSSQNGSCRQVQYPLTDMSPILTSGDSDISSPLLQNTVHIDLSALNPELV  1081
MET-206     ..............................................................................................................   190
MET-203     ..............................................................................................................   214
MET-202     WLKKRKQIKDLGSELVRYDARVHTPHLDRLVSARSVSPTTEMVSNESVDYRATFPEDQFPNSSQNGSCRQVQYPLTDMSPILTSGDSDISSPLLQNTVHIDLSALNPELV  1063
MET-204     ..............................................................................................................   764

logo
            QAVQHVVIGPSSLIVHFNEVIGRGHFGCVYHGTLLDNDGKKIHCAVKSLNRITDIGEVSQFLTEGIIMKDFSHPNVLSLLGICLRSEGSPLVVLPYMKHGDLRNFIRNET
SGX-523     ....................I.......................A................................................LPYM...D.........     7
PF01403     ..............................................................................................................   435
PF07714     ...............HFNEVIGRGHFGCVYHGTLLDNDGKKIHCAVKSLNRITDIGEVSQFLTEGIIMKDFSHPNVLSLLGICLRSEGSPLVVLPYMKHGDLRNFIRNET    95
PF01437     ..............................................................................................................    40
PF01833.3   ..............................................................................................................   164
PF01833     ..............................................................................................................   232
PF01403.1   ..............................................................................................................   109
PF01833.2   ..............................................................................................................    76
PF01833.1   ..............................................................................................................   240
MET-205     QAVQHVVIGP....................................................................................................   158
MET-201     QAVQHVVIGPSSLIVHFNEVIGRGHFGCVYHGTLLDNDGKKIHCAVKSLNRITDIGEVSQFLTEGIIMKDFSHPNVLSLLGICLRSEGSPLVVLPYMKHGDLRNFIRNET  1191
MET-206     ..............................................................................................................   190
MET-203     ..............................................................................................................   214
MET-202     QAVQHVVIGPSSLIVHFNEVIGRGHFGCVYHGTLLDNDGKKIHCAVKSLNRITDIGEVSQFLTEGIIMKDFSHPNVLSLLGICLRSEGSPLVVLPYMKHGDLRNFIRNET  1173
MET-204     ..............................................................................................................   764

logo
            HNPTVKDLIGFGLQVAKGMKYLASKKFVHRDLAARNCMLDEKFTVKVADFGLARDMYDKEYYSVHNKTGAKLPVKWMALESLQTQKFTTKSDVWSFGVLLWELMTRGAPP
SGX-523     ..................................R..M.........AD....A........................................................    12
PF01403     ..............................................................................................................   435
PF07714     HNPTVKDLIGFGLQVAKGMKYLASKKFVHRDLAARNCMLDEKFTVKVADFGLARDMYDKEYYSVHNKTGAKLPVKWMALESLQTQKFTTKSDVWSFGVLLWELMTRGAPP   205
PF01437     ..............................................................................................................    40
PF01833.3   ..............................................................................................................   164
PF01833     ..............................................................................................................   232
PF01403.1   ..............................................................................................................   109
PF01833.2   ..............................................................................................................    76
PF01833.1   ..............................................................................................................   240
MET-205     ..............................................................................................................   158
MET-201     HNPTVKDLIGFGLQVAKGMKYLASKKFVHRDLAARNCMLDEKFTVKVADFGLARDMYDKEYYSVHNKTGAKLPVKWMALESLQTQKFTTKSDVWSFGVLLWELMTRGAPP  1301
MET-206     ..............................................................................................................   190
MET-203     ..............................................................................................................   214
MET-202     HNPTVKDLIGFGLQVAKGMKYLASKKFVHRDLAARNCMLDEKFTVKVADFGLARDMYDKEYYSVHNKTGAKLPVKWMALESLQTQKFTTKSDVWSFGVLLWELMTRGAPP  1283
MET-204     ..............................................................................................................   764
```

```
logo
            YPDVNTFDITVYLLQGRRLLQPEYCPDPLYEVMLKCWHPKAEMRPSFSELVSRISAIFSTFIGEHYVHVNATYVNVKCVAPYPSLLSSEDNADDEVDTRPASFWETS
SGX-523     ...........................................................................................................    12
PF01403     ...........................................................................................................   435
PF07714     YPDVNTFDITVYLLQGRRLLQPEYCPDPLYEVMLKCWHPKAEMRPSFSELVSR......................................................   258
PF01437     ...........................................................................................................    40
PF01833.3   ...........................................................................................................   164
PF01833     ...........................................................................................................   232
PF01403.1   ...........................................................................................................   109
PF01833.2   ...........................................................................................................    76
PF01833.1   ...........................................................................................................   240
MET-205     ...........................................................................................................   158
MET-201     YPDVNTFDITVYLLQGRRLLQPEYCPDPLYEVMLKCWHPKAEMRPSFSELVSRISAIFSTFIGEHYVHVNATYVNVKCVAPYPSLLSSEDNADDEVDTRPASFWETS  1408
MET-206     ...........................................................................................................   190
MET-203     ...........................................................................................................   214
MET-202     YPDVNTFDITVYLLQGRRLLQPEYCPDPLYEVMLKCWHPKAEMRPSFSELVSRISAIFSTFIGEHYVHVNATYVNVKCVAPYPSLLSSEDNADDEVDTRPASFWETS  1390
MET-204     ...........................................................................................................   764
```

X non conserved
X similar
X ≥ 0% conserved
X ≥ 50% conserved

logo 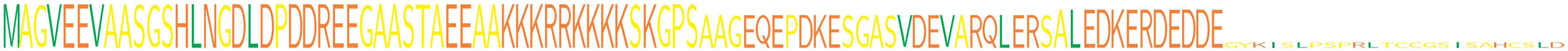

```
FUMAGILLIN   ..............................................................................................................    0
PF00557.2    ..............................................................................................................    0
PF00557      ..............................................................................................................    0
PF00557.1    ..............................................................................................................    0
METAP2-211   MAGVEEVAASGSHLNGDLDPDDREEGAASTAEEAAKKKRRKKKKSKGPSA........................ALEDKERDEDDE........................   62
METAP2-208   ..............................................................................................................    0
METAP2-209   MAGVEEVAASGSHLNGDLDPDDREEGAASTAEEAAKKKRRKKKKSKGPSA............................................................   50
METAP2-205   MAGVEEVAASGSHLNGDLDPDDREEGAASTAEEAAKKKRRKKKKSKGPSAAGEQEPDKESGASVDEVARQLERSALEDKERDEDDE........................   86
METAP2-202   MAGVEEVAASGSHLNGDLDPDDREEGAASTAEEAAKKKRRKKKKSKGPSAAGEQEPDKESGASVDEVARQLERSALEDKERDEDDE........................   86
METAP2-210   MAGVEEVAASGSHLNGDLDPDDREEGAASTAEEAAKKKRRKKKKSKGPS.AGEQEPDKESGASVDEVARQLERSALEDKERDEDDE........................   85
METAP2-207   MAGVEEVAASGSHLNGDLDPDDREEGAASTAEEAAKKKRRKKKKSKGPSAAGEQEPDKESGASVDEVARQLERSALEDKERDEDDEGYKISLPSPRLTCCGSISAHCSLD  110
METAP2-203   MAGVEEVAASGSHLNGDLDPDDREEGAASTAEEAAKKKRRKKKKSKGPSAAGEQEPDKESGASVDEVARQLERSALEDKERDEDDE........................   86
METAP2-201   MAGVEEVAASGSHLNGDLDPDDREEGAASTAEEAAKKKRRKKKKSKGPS.AGEQEPDKESGASVDEVARQLERSALEDKERDEDDE........................   85
```

logo 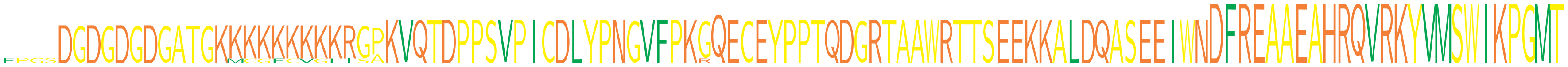

```
FUMAGILLIN   ..............................................................................................................    0
PF00557.2    .....................................................................................DFREAAEAHRQVRKYVMSWIKPGMT   25
PF00557      .....................................................................................DFREAAEAHRQVRKYVMSWIKPGMT   25
PF00557.1    .....................................................................................DFREAAEAHRQVRKYVMSWIKPGMT   25
METAP2-211   ....DGDGDGDGATGKKKKKKKKKRGPKVQTDPPSVPICDLYPNGVFPKGQECEYPPTQDGRTAAWRTTSEEKKALDQASEEIWNDFREAAEAHRQVRKYVMSWIKPGMT  168
METAP2-208   ................MCGFCVGLISAKVQTDPPSVPICDLYPNGVFPKGQECEYPPTQDGRTAAWRTTSEEKKALDQASEEIWNDFREAAEAHRQVRKYVMSWIKPGMT   94
METAP2-209   ....DGDGDGDGATGKKKKKKKKKRGPKVQTDPPSVPICDLYPNGVFPKGQECEYPPTQDGRTAAWRTTSEEKKALDQASEEIWNDFREAAEAHRQVRKYVMSWIKPGMT  156
METAP2-205   ....DGDGDGDGATGKKKKKKKKKRG.......................RQECEYPPTQDGRTAAWRTTSEEKKALDQASEEIWNDFREAAEAHRQVRKYVMSWIKPGMT  169
METAP2-202   ....DGDGDGDGATGKKKKKKKKKRGPKVQTDPPSVPICDLYPNGVFPKGQECEYPPTQDGRTAAWRTTSEEKKALDQASEEIWNDFREAAEAHRQVRKYVMSWIKPGMT  192
METAP2-210   ....DGDGDGDGATGKKKKKKKKKRGPKVQTDPPSVPICDLYPNGVFPKGQECEYPPTQDGRTAAWRTTSEEKKALDQASEEIWNDFREAAEAHRQVRKYVMSWIKPGMT  191
METAP2-207   FPGSDGDGDGDGATGKKKKKKKKKRGPKVQTDPPSVPICDLYPNGVFPKGQ...........................................................  161
METAP2-203   ....DGDGDGDGATGKKKKKKKKKRGPKVQTDPPSVPICDLYPNGVFPKGQECEYPPTQDGRTAAWRTTSEEKKALDQASEEIWNDFREAAEAHRQVRKYVMSWIKPGMT  192
METAP2-201   ..........................AKVQTDPPSVPICDLYPNGVFPKGQECEYPPTQDGRTAAWRTTSEEKKALDQASEEIWNDFREAAEAHRQVRKYVMSWIKPGMT  169
```

logo 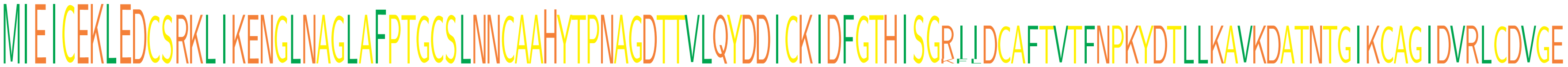

```
FUMAGILLIN   ..........................F...........H.......................................................................    2
PF00557.2    MIEICEKLE.....................................................................................................   34
PF00557      MIEICEKLEDCSRKLIKENGLNAGLAFPTGCSLNNCAAHYTPNAGDTTVLQYDDICKIDFGTHISGRIIDCAFTVTFNPKYDTLLKAVKDATNTGIKCAGIDVRLCDVGE  135
PF00557.1    MIEICEKLEDCSRKLIKENGLNAGLAFPTGCSLNNCAAHYTPNAGDTTVLQYDDICKIDFGTHISG............................................   91
METAP2-211   MIEICEKLED....................................................................................................  178
METAP2-208   MIEICEKLEDCSRKLIKENGLNAGLAFPTGCSLNNCAAHYTPNAGDTTVLQYDDICKIDFGTHISGKFL.........................................  163
METAP2-209   MIEICEKLEDCSRKLIKENGLNAGLAFPTGCSLNNCAAHYTPNAGDTTVLQYDDICKIDFGTHISGRIIDCAFTVTFNPKYDTLLKAVKDATNTGIKCAGIDVRLCDVGE  266
METAP2-205   MIEICEKLEDCSRKLIKENGLNAGLAFPTGCSLNNCAAHYTPNAGDTTVLQYDDICKIDFGTHISGRIIDCAFTVTFNPKYDTLLKAVKDATNTGIKCAGIDVRLCDVGE  279
METAP2-202   MIEICEKLEDCSRKLIKENGLNAGLAFPTGCSLNNCAAHYTPNAGDTTVLQYDDICKIDFGTHISGRIIDCAFTVTFNPKYDTLLKAVKDATNTGIKCAGIDVRLCDVGE  302
METAP2-210   MIEICEKLEDCSRKLIKENGLNAGLAFPTGCSLNNCAAHYTPNAGDTTVLQYDDICKIDFGTHISGRIIDCAFTVTFNPKYDTLLKAVKDATNTGIKCAGIDVRLCDVGE  301
METAP2-207   ..............................................................................................................  161
METAP2-203   MIEICEKLEDCSRKLIKENGLNAGLAFPTGCSLNNCAAHYTPNAGDTTVLQYDDICKIDFGTHISGRIIDCAFTVTFNPKYDTLLKAVKDATNTGIKCAGIDVRLCDVGE  302
METAP2-201   MIEICEKLEDCSRKLIKENGLNAGLAFPTGCSLNNCAAHYTPNAGDTTVLQYDDICKIDFGTHISGRIIDCAFTVTFNPKYDTLLKAVKDATNTGIKCAGIDVRLCDVGE  279
```

logo 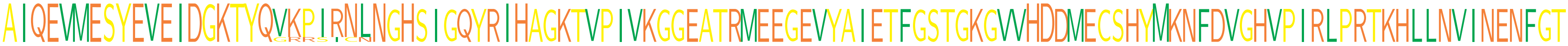

```
FUMAGILLIN    ........................NLN.H......IH...................................HD.......M............................   11
PF00557.2     ..............................................................................................................   34
PF00557       AIQEVMESYEVEIDGKTYQVKPIRNLNGHSIGQYRIHAGKTVPIVKGGEATRMEEGEVYAIETFGSTGKGVVHDDMECSHYMKNFDVGHVPIRLPRTKHLLNVINENFGT  245
PF00557.1     ..............................................................................................................   91
METAP2-211    ..............................................................................................................  178
METAP2-208    ..............................................................................................................  163
METAP2-209    AIQEVMESYEVEIDGKTYQVKPIRNLNGHSIGQYRIHAGKTVPIVKGGEATRMEEGEVYAIETFGSTGKGVVHDDMECSHYMKNFDVGHVPIRLPRTKHLLNVINENFGT  376
METAP2-205    AIQEVMESYEVEIDGKTYQVKPIRNLNGHSIGQYRIHAGKTVPIVKGGEATRMEEGEVYAIETFGSTGKGVVHDDMECSHYMKNFDVGHVPIRLPRTKHLLNVINENFGT  389
METAP2-202    AIQEVMESYEVEIDGKTYQVKPIRNLNGHSIGQYRIHAGKTVPIVKGGEATRMEEGEVYAIETFGSTGKGVVHDDMECSHYMKNFDVGHVPIRLPRTKHLLNVINENFGT  412
METAP2-210    AIQEVMESYEVEIDGKTYQVKPIRNLNGHSIGQYRIHAGKTVPIVKGGEATRMEEGEVYAIETFGSTGKGVVHDDMECSHYMKNFDVGHVPIRLPRTKHLLNVINENFGT  411
METAP2-207    ..............................................................................................................  161
METAP2-203    AIQEVMESYEVEIDGKTYQGRRSICN....................................................................................  328
METAP2-201    AIQEVMESYEVEIDGKTYQVKPIRNLNGHSIGQYRIHAGKTVPIVKGGEATRMEEGEVYAIETFGSTGKGVVHDDMECSHYMKNFDVGHVPIRLPRTKHLLNVINENFGT  389
```

logo 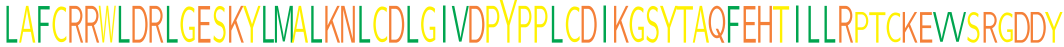

```
FUMAGILLIN    ...............................Y..................................   12
PF00557.2     ..................................................................   34
PF00557       LAFCRRWLDRLGESKYLMALKNLCDLGIVDPYPPLCDIKGSYTAQFEHTILLR.............  298
PF00557.1     ..................................................................   91
METAP2-211    ..................................................................  178
METAP2-208    ..................................................................  163
METAP2-209    LAFCRRWLDRLGESKYLMALKNLCDLGIVDPYPPLCDIKGSYTAQFEHTILLRPTCKEVVSRGDDY  442
METAP2-205    LAFCRRWLDRLGESKYLMALKNLCDLGIVDPYPPLCDIKGSYTAQFEHTILLRPTCKEVVSRGDDY  455
METAP2-202    LAFCRRWLDRLGESKYLMALKNLCDLGIVDPYPPLCDIKGSYTAQFEHTILLRPTCKEVVSRGDDY  478
METAP2-210    LAFCRRWLDRLGESKYLMALKNLCDLGIVDPYPPLCDIKGSYTAQFEHTILLRPTCKEVVSRGDDY  477
METAP2-207    ..................................................................  161
METAP2-203    ..................................................................  328
METAP2-201    LAFCRRWLDRLGESKYLMALKNLCDLGIVDPYPPLCDIKGSYTAQFEHTILLRPTCKEVVSRGDDY  455
```

X non conserved
X similar
X ≥ 0% conserved
X ≥ 50% conserved

```
logo        MKFLLILLLQATASGALPLNSSTSLEKNNVLFGERYLEKFYGLEINKLPVTKMKYSGNLMKEKIQEMQHFLGLKVTGQLDTSTLEMMHAPRCGVPDVHHFREMPGGPVWR
BATIMASTAT  ..............................................................................................................    0
PF00413     ............................................................................................................WR    2
PF00045     ..............................................................................................................    0
PF01471     ..........................KNNVLFGERYLEKFYGLEINKLPVTKMKYSGNLMKEKIQEMQHFLGLKVTGQLDTSTLEMM.......................   61
MMP12-201   MKFLLILLLQATASGALPLNSSTSLEKNNVLFGERYLEKFYGLEINKLPVTKMKYSGNLMKEKIQEMQHFLGLKVTGQLDTSTLEMMHAPRCGVPDVHHFREMPGGPVWR  110

logo        KHYITYRINNYTPDMNREDVDYAIRKAFQVWSNVTPLKFSKINTGMADILVVFARGAHGDFHAFDGKGGILAHAFGPGSGIGGDAHFDEDEFWTTHSGGTNLFLTAVHEI
BATIMASTAT  ....................................................................GILA...................................H..    5
PF00413     KHYITYRINNYTPDMNREDVDYAIRKAFQVWSNVTPLKFSKINTGMADILVVFARGAHGDFHAFDGKGGILAHAFGPGSGIGGDAHFDEDEFWTTHSGGTNLFLTAVHEI  112
PF00045     ..............................................................................................................    0
PF01471     ..............................................................................................................   61
MMP12-201   KHYITYRINNYTPDMNREDVDYAIRKAFQVWSNVTPLKFSKINTGMADILVVFARGAHGDFHAFDGKGGILAHAFGPGSGIGGDAHFDEDEFWTTHSGGTNLFLTAVHEI  220

logo        GHSLGLGHSSDPKAVMFPTYKYVDINTFRLSADDIRGIQSLYGDPKENQRLPNPDNSEPALCDPNLSFDAVTTVGNKIFFFKDRFFWLKVSERPKTSVNLISSLWPTLPS
BATIMASTAT  .H.....H........FPTY..........................................................................................   11
PF00413     GHSLGLGHSSDPKAVMFPTYKYVDINTFRLSADDIRGIQSLYG...................................................................  155
PF00045     ...................................................................FDAVTTVGNKIFFFKDRFFWLKVSERPKTSVNLISSLWPTLPS   43
PF01471     ..............................................................................................................   61
MMP12-201   GHSLGLGHSSDPKAVMFPTYKYVDINTFRLSADDIRGIQSLYGDPKENQRLPNPDNSEPALCDPNLSFDAVTTVGNKIFFFKDRFFWLKVSERPKTSVNLISSLWPTLPS  330

logo        GIEAAYEIEARNQVFLFKDDKYWLISNLRPEPNYPKSIHSFGFPNFVKKIDAAVFNPRFYRTYFFVDNQYWRYDERRQMMDPGYPKLITKNFQGIGPKIDAVFYSKNKYY
BATIMASTAT  ..............................................................................................................   11
PF00413     ..............................................................................................................  155
PF00045     .IEAAYEIEARNQVFLFKDDKYWLISNLRPEPNYPKSIHSFGFP.....IDAAVFNPRFYRTYFFVDNQYWRYDERRQMMDPGYPKLITKNFQGIG..IDAVFYSKNKYY  145
PF01471     ..............................................................................................................   61
MMP12-201   GIEAAYEIEARNQVFLFKDDKYWLISNLRPEPNYPKSIHSFGFPNFVKKIDAAVFNPRFYRTYFFVDNQYWRYDERRQMMDPGYPKLITKNFQGIGPKIDAVFYSKNKYY  440

logo        YFFQGSNQFEYDFLLQRITKTLKSNSWFGC
BATIMASTAT  ..............................   11
PF00413     ..............................  155
PF00045     YFFQGSNQFEYDFLLQRITKTLKSNSWFGC  175
PF01471     ..............................   61
MMP12-201   YFFQGSNQFEYDFLLQRITKTLKSNSWFGC  470
```

X non conserved
X similar
X ≥0% conserved
X ≥50% conserved

```
logo
           MRDGRYCRRAVLKPGPLSRLLYLSTGRRLDFVHHSGVFFLQTLLWILCATVCGTEQYFNVEVWLQKYGYLPPTDPRMSVLRSAETMQSALAAMQQFYGINMTGKVDRNTI
BATIMASTAT ..............................................................................................................   0
PF11857    ..............................................................................................................   0
PF01471    ...........................................................VEVWLQKYGYLPPTDPRMSVLRSAETMQSALAAMQQFYGINMTGKVDRNTI  51
PF00413    ..............................................................................................................   0
PF00045    ..............................................................................................................   0
MMP16-201  .................MILLTFSTGRRLDFVHHSGVFFLQTLLWILCATVCGTEQYFNVEVWLQKYGYLPPTDPRMSVLRSAETMQSALAAMQQFYGINMTGKVDRNTI  93
MMP16-203  MRDGRYCRRAVLKPGPLSRAGYLRTGRRLDFVHHSGVFFLQTLLWILCATVCGTEQYFNVEVWLQKYGYLPPTDPRMSVLRSAETMQSALAAMQQFYGINMTGKVDRNTI 110

logo
           DWMKKPRCGVPDQTRGSSKFHIRRKRYALTGQKWQHKHITYSIKNVTPKVGDPETRKAIRRAFDVWQNVTPLTFEEVPYSELENGKRDVDITIIFASGFHGDSSPFDGEG
BATIMASTAT ..............................................................................................................   0
PF11857    ..............................................................................................................   0
PF01471    DWM...........................................................................................................  54
PF00413    ................................KWQHKHITYSIKNVTPKVGDPETRKAIRRAFDVWQNVTPLTFEEVPYSELENGKRDVDITIIFASGFHGDSSPFDGEG  78
PF00045    ..............................................................................................................   0
MMP16-201  DWMKKPRCGVPDQTRGSSKFHIRRKRYALTGQKWQHKHITYSIKNVTPKVGDPETRKAIRRAFDVWQNVTPLTFEEVPYSELENGKRDVDITIIFASGFHGDSSPFDGEG 203
MMP16-203  DWMKKPRCGVPDQTRGSSKFHIRRKRYALTGQKWQHKHITYSIKNVTPKVGDPETRKA.................................................... 168

logo
           GFLAHAYFPGPGIGGDTHFDSDEPWTLGNPNHDGNDLFLVAVHELGHALGLEHSNDPTAIMAPFYQYMETDNFKLPNDDLQGIQKIYGPPDKIPPPTRPLPTVPPHRSIP
BATIMASTAT GFLA...................................V..HE..H.....H.........PFY.............................................  12
PF11857    ..............................................................................................................   0
PF01471    ..............................................................................................................  54
PF00413    GFLAHAYFPGPGIGGDTHFDSDEPWTLGNPNHDGNDLFLVAVHELGHALGLEHSNDPTAIMAPFYQYMETDNFKLPNDDLQGIQKIYG...................... 166
PF00045    ..............................................................................................................   0
MMP16-201  GFLAHAYFPGPGIGGDTHFDSDEPWTLGNPNHDGNDLFLVAVHELGHALGLEHSNDPTAIMAPFYQYMETDNFKLPNDDLQGIQKIYGPPDKIPPPTRPLPTVPPHRSIP 313
MMP16-203  .............................................................................................................. 168

logo
           PADPRKNDRPKPPRPPTGRPSYPGAKPNICDGNFNTLAILRREMFVFKDQWFWRVRNNRVMDGYPMQITYFWRGLPPSIDAVYENSDGNFVFFKGNKYWVFKDTTLQPGY
BATIMASTAT ..............................................................................................................  12
PF11857    ..............................................................................................................   0
PF01471    ..............................................................................................................  54
PF00413    .............................................................................................................. 166
PF00045    .........................................REMFVFKDQWFWRVRNNRVMDGYPMQITYFWRGLPP.IDAVYENSDGNFVFFKGNKYWVFKDTTLQPGY  68
MMP16-201  PADPRKNDRPKPPRPPTGRPSYPGAKPNICDGNFNTLAILRREMFVFKDQWFWRVRNNRVMDGYPMQITYFWRGLPPSIDAVYENSDGNFVFFKGNKYWVFKDTTLQPGY 423
MMP16-203  .............................................................................................................. 168

logo
           PHDLITLGSGIPPHGIDSAIWWEDVGKTYFFKGDRYWRYSEEMKTMDPGYPKPITVWKGIPESPQGAFVHKENGFTYFYKGKEYWKFNNQILKVEPGYPRSILKDFMGCD
BATIMASTAT ..............................................................................................................  12
PF11857    ..............................................................................................................   0
PF01471    ..............................................................................................................  54
PF00413    .............................................................................................................. 166
PF00045    PHDLITLGSGIP...IDSAIWWEDVGKTYFFKGDRYWRYSEEMKTMDPGYPKPITVWKGIP....GAFVHKENGFTYFYKGKEYWKFNNQILKVEPGYPRSILKDFMGC. 170
MMP16-201  PHDLITLGSGIPPHGIDSAIWWEDVGKTYFFKGDRYWRYSEEMKTMDPGYPKPITVWKGIPESPQGAFVHKENGFTYFYKGKEYWKFNNQILKVEPGYPRSILKDFMGCD 533
MMP16-203  .............................................................................................................. 168
```

logo

```
             GPTDRVKEGHSPPDDVDIVIKLDNTASTVKAIAIVIPCILALCLLVLVYTVFQFKRKGTPRHILYCKRSMQEWV
BATIMASTAT   ..........................................................................   12
PF11857      ....RVKEGHSPPDDVDIVIKLDNTASTVKAIAIVIPCILALCLLVLVYTVFQFKRKGTPRHILYCKRSMQEWV   70
PF01471      ..........................................................................   54
PF00413      ..........................................................................  166
PF00045      ..........................................................................  170
MMP16-201    GPTDRVKEGHSPPDDVDIVIKLDNTASTVKAIAIVIPCILALCLLVLVYTVFQFKRKGTPRHILYCKRSMQEWV  607
MMP16-203    ..........................................................................  168
```

X non conserved
X similar
X $\geq$ 0% conserved
X $\geq$ 50% conserved

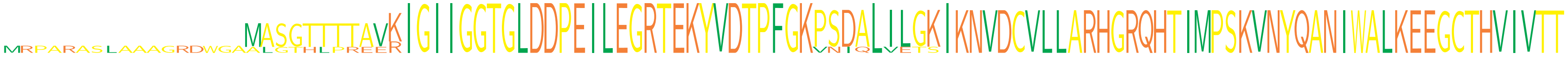

```
logo
FORMYCIN    ..............................................................................................................   0
PF01048     ...........................KIGIIGGTGLDDPEILEGRTEKYVDTPFGKPSDALILGKIKNVDCVLLARHGRQHTIMPSKVNYQANIWALKEEGCTHVIVTT  83
PF01048.1   ...........................RIGIIGGTGLDDPEILEGRTEKYVDTPFGKPSDALILGKIKNVDCVLLARHGRQHTIMPSKVNYQANIWALKEEGCTHVIVTT  83
PF01048.2   ..............................................................................................................   0
MTAP-202    .................MASGTTTTAVKIGIIGGTGLDDPEILEGRTEKYVDTPFGKVNIQLVETSI...........................................  50
MTAP-206    ..............................................................................................................   0
MTAP-210    .................MASGTTTTAVKIGIIGGTGLDDPEILEGRTEKYVDTPFGKPSDALILGKIKNVDCVLLARHGRQHTIMPSKVNYQANIWALKEEGCTHVIVTT  93
MTAP-201    .................MASGTTTTAVKIGIIGGTGLDDPEILEGRTEKYVDTPFGKPSDALILGKIKNVDCVLLARHGRQHTIMPSKVNYQANIWALKEEGCTHVIVTT  93
MTAP-209    .................MASGTTTTAVKIGIIGGTGLDDPEILEGRTEKYVDTPFGKPSDALILGKIKNVDCVLLARHGRQHTIMPSKVNYQANIWALKEEGCTHVIVTT  93
MTAP-204    MRPARASLAAAGRDWGAALGTHLPREERIGIIGGTGLDDPEILEGRTEKYVDTPFGKPSDALILGKIKNVDCVLLARHGRQHTIMPSKVNYQANIWALKEEGCTHVIVTT 110
```

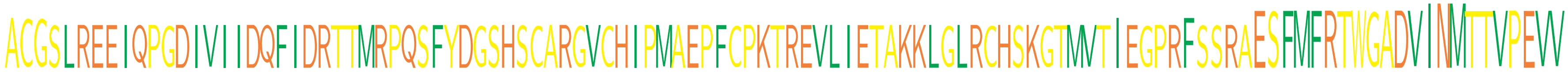

```
logo
FORMYCIN    ACG...........................................................................I....F................INM.......   8
PF01048     ACGSLREEIQPGDIVIIDQFIDRTTMRPQSFYDGSHSCARGVCHIPMAEPFCPKTREVLIETAKKLGLRCHSKGTMVTIEGPRFSSRAESFMFRTWGADVINMTTVPEVV 193
PF01048.1   ACGSLREEIQPGDIVIIDQFIDRTTMRPQSFYDGSHSCARGVCHIPMAEPFCPKTREVLIETAKKLGLRCHSKGTMVTIEGPRFSSRAESFMFRTWGADVINMTTVPEVV 193
PF01048.2   ........................................................................................ESFMFRTWGADVINMTTVPEVV  22
MTAP-202    ..............................................................................................................  50
MTAP-206    ........................................................................................ESFMFRTWGADVINMTTVPEVV  22
MTAP-210    ACGSLREEIQPGDIVIIDQFIDRTTMRPQSFYDGSHSCARGVCHIPMAEPFCPKTREVLIETAKKLGLRCHSKGTMVTIEGPRFSSRAESFMFRTWGADVINMTTVPEVV 203
MTAP-201    ACGSLREEIQPGDIVIIDQFIDRTTMRPQSFYDGSHSCARGVCHIPMAEPFCPKTREVLIETAKKLGLRCHSKGTMVTIEGPRFSSRAESFMFRTWGADVINMTTVPEVV 203
MTAP-209    ACGSLREEIQPGDIVIIDQFIDRTTMRPQSFYDGSHSCARGVCHIPMAEPFCPKTREVLIETAKKLGLRCHSKGTMVTIEGPRFSSRAESFMFRTWGADVINMTTVPEVV 203
MTAP-204    ACGSLREEIQPGDIVIIDQFIDRTTMRPQSFYDGSHSCARGVCHIPMAEPFCPKTREVLIETAKKLGLRCHSKGTMVTIEGPRFSSRAESFMFRTWGADVINMTTVPEVV 220
```

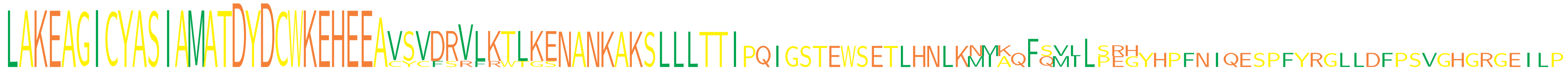

```
logo
FORMYCIN    ................D.D.............V.............................................................................  11
PF01048     LAKEAGICYASIAMATDYDCWKEHEEAVSVDRVLKTLKENANKAKSLLLTTI.......................................................... 245
PF01048.1   LAKEAGICYASIAMATDYDCWKEHEEAVSVDRVLKTLKENANKAKSLLLTTI.......................................................... 245
PF01048.2   LAKEAGICYASIAMATDYDCWKEHEE....................................................................................  48
MTAP-202    ..............................................................................................................  50
MTAP-206    LAKEAGICYASIAMATDYDCWKEHEEA.........................................MIK.FQMILSEGYHPFNIQESPFYRGLLDFPSVGHGRGEILP  90
MTAP-210    LAKEAGICYASIAMATDYDCWKEHEEAVSVDRVLKTLKENANKAKSLLLTTIPQIGSTEWSETLHNLKMIK.FQMILSEGYHPFNIQESPFYRGLLDFPSVGHGRGEILP 312
MTAP-201    LAKEAGICYASIAMATDYDCWKEHEEAVSVDRVLKTLKENANKAKSLLLTTIPQIGSTEWSETLHNLKNMAQFSVLLPRH.............................. 283
MTAP-209    LAKEAGICYASIAMATDYDCWKEHEEACYCFSRFRWTGS....................................................................... 242
MTAP-204    LAKEAGICYASIAMATDYDCWKEHEEAVSVDRVLKTLKENANKAKSLLLTTIPQIGSTEWSETLHNLKNMAQFSVLLPRH.............................. 300
```

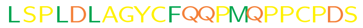

```
logo
FORMYCIN    ......................  11
PF01048     ...................... 245
PF01048.1   ...................... 245
PF01048.2   ......................  48
MTAP-202    ......................  50
MTAP-206    LSPLDLAGYCFQQPMQPPCPDS 112
MTAP-210    LSPLDLAGYCFQQPMQPPCPDS 334
MTAP-201    ...................... 283
MTAP-209    ...................... 242
MTAP-204    ...................... 300
```

X non conserved
X similar
X $\geq 0\%$ conserved
X $\geq 50\%$ conserved

```
logo

SIROLIMUS    ..............................................Y................FD..H.R...F..........GEVI..W...................    11
PF00454.1    ..............................................................................................................     0
PF02259      ..............................................................................................................     0
PF02259.1    ..............................................................................................................     0
PF08771      ..............................................................................................................     0
PF11865      ..............................................................................................................     0
PF00454      ..............................................................................................................     0
PF02260      ..............................................................................................................     0
MTOR-203     ..............................................................................................................     0
MTOR-202     ..............................................................................................................     0
MTOR-201     MLGTGPAAATTAATTSSNVSVLQQFASGLKSRNEETRAKAAKELQHYVTMELREMSQEESTRFYDQLNHHIFELVSSSDANERKGGILAIASLIGVEG....GNATRIGR   106

logo

SIROLIMUS    ...Y.............KI.........F....Y.........FD.....................L......................GKVI..W..............    24
PF00454.1    ..............................................................................................................     0
PF02259      ..............................................................................................................     0
PF02259.1    ..............................................................................................................     0
PF08771      ..............................................................................................................     0
PF11865      ..............................................................................................................     0
PF00454      ..............................................................................................................     0
PF02260      ..............................................................................................................     0
MTOR-203     ..............................................................................................................     0
MTOR-202     ..............................................................................................................     0
MTOR-201     FANYLRNLLPSNDPVVMEMASKAIGRLAMAGDTFTA...EYVEFEVKRALEWLGADRNEGRRHAAVLVLRELAISVPTFFFQQVQPFFDNIFVAVWDPKQAIREGAVAAL   213

logo

SIROLIMUS    ....................Y.......A.I.......F.......................................................................    28
PF00454.1    ..............................................................................................................     0
PF02259      ..............................................................................................................     0
PF02259.1    ..............................................................................................................     0
PF08771      ..............................................................................................................     0
PF11865      ..............................................................................................................     0
PF00454      ..............................................................................................................     0
PF02260      ..............................................................................................................     0
MTOR-203     ..............................................................................................................     0
MTOR-202     ..............................................................................................................     0
MTOR-201     RACLILTTQREPKEMQKPQWYRHTFE.......EAEKGFDETLAKEKGMNRDDRIHGALLILNELVRISSMEGERLREEMEEITQQQLVHDKYCKDLMGFGTKPRHITPF   316
```

```
logo

             TSFQAVQPQQSNALVGLLGYSSHQGLMGFGTSPSPAKSTLVESRCCRDLMEEKFDQVCQWVLKCRNSKNSLIQMTILNLLPRLAAFRPSAFTDTQYLQDTMNHVLSCVKK
SIROLIMUS    ..............................................................................................................   28
PF00454.1    ..............................................................................................................    0
PF02259      ..............................................................................................................    0
PF02259.1    ..............................................................................................................    0
PF08771      ..............................................................................................................    0
PF11865      ..............................................................................................................    0
PF00454      ..............................................................................................................    0
PF02260      ..............................................................................................................    0
MTOR-203     ..............................................................................................................    0
MTOR-202     ..............................................................................................................    0
MTOR-201     TSFQAVQPQQSNALVGLLGYSSHQGLMGFGTSPSPAKSTLVESRCCRDLMEEKFDQVCQWVLKCRNSKNSLIQMTILNLLPRLAAFRPSAFTDTQYLQDTMNHVLSCVKK  426

logo

             EKERTAAFQALGLLSVAVRSEFKVYLPRVLDIIRAALPPKDFAHKRQKAMQVDATVFTCISMLARAMGPGIQQDIKELLEPMLAVGLSPALTAVLYDLSRQIPQLKKDIQ
SIROLIMUS    ..............................................................................................................   28
PF00454.1    ..............................................................................................................    0
PF02259      ..............................................................................................................    0
PF02259.1    ..............................................................................................................    0
PF08771      ..............................................................................................................    0
PF11865      ..............................................................................................................    0
PF00454      ..............................................................................................................    0
PF02260      ..............................................................................................................    0
MTOR-203     ..............................................................................................................    0
MTOR-202     ..............................................................................................................    0
MTOR-201     EKERTAAFQALGLLSVAVRSEFKVYLPRVLDIIRAALPPKDFAHKRQKAMQVDATVFTCISMLARAMGPGIQQDIKELLEPMLAVGLSPALTAVLYDLSRQIPQLKKDIQ  536

logo

             DGLLKMLSLVLMHKPLRHPGMPKGLAHQLASPGLTTLPEASDVGSITLALRTLGSFEFEGHSLTQFVRHCADHFLNSEHKEIRMEAARTCSRLLTPSIHLISGHAHVVSQ
SIROLIMUS    ..............................................................................................................   28
PF00454.1    ..............................................................................................................    0
PF02259      ..............................................................................................................    0
PF02259.1    ..............................................................................................................    0
PF08771      ..............................................................................................................    0
PF11865      ..............................................................................................................    0
PF00454      ..............................................................................................................    0
PF02260      ..............................................................................................................    0
MTOR-203     ..............................................................................................................    0
MTOR-202     ..............................................................................................................    0
MTOR-201     DGLLKMLSLVLMHKPLRHPGMPKGLAHQLASPGLTTLPEASDVGSITLALRTLGSFEFEGHSLTQFVRHCADHFLNSEHKEIRMEAARTCSRLLTPSIHLISGHAHVVSQ  646
```

```
logo
            TAVQVVADVLSKLLVVGITDPDPDIRYCVLASLDERFDAHLAQAENLQALFVALNDQVFEIRELAICTVGRLSSMNPAFVMPFLRKMLIQILTELEHSGIGRIKEQSARM
SIROLIMUS   ..............................................................................................................   28
PF00454.1   ..............................................................................................................    0
PF02259     ..............................................................................................................    0
PF02259.1   ..............................................................................................................    0
PF08771     ..............................................................................................................    0
PF11865     ..............................................................................................................    0
PF00454     ..............................................................................................................    0
PF02260     ..............................................................................................................    0
MTOR-203    ..............................................................................................................    0
MTOR-202    ..............................................................................................................    0
MTOR-201    TAVQVVADVLSKLLVVGITDPDPDIRYCVLASLDERFDAHLAQAENLQALFVALNDQVFEIRELAICTVGRLSSMNPAFVMPFLRKMLIQILTELEHSGIGRIKEQSARM  756

logo
            LGHLVSNAPRLIRPYMEPILKALILKLKDPDPDPNPGVINNVLATIGELAQVSGLEMRKWVDELFIIIMDMLQDSSLLAKRQVALWTLGQLVASTGYVVEPYRKYPTLLE
SIROLIMUS   ..............................................................................................................   28
PF00454.1   ..............................................................................................................    0
PF02259     ..............................................................................................................    0
PF02259.1   ..............................................................................................................    0
PF08771     ..............................................................................................................    0
PF11865     .................................................................................................VVEPYRKYPTLLE   13
PF00454     ..............................................................................................................    0
PF02260     ..............................................................................................................    0
MTOR-203    ..............................................................................................................    0
MTOR-202    ..............................................................................................................    0
MTOR-201    LGHLVSNAPRLIRPYMEPILKALILKLKDPDPDPNPGVINNVLATIGELAQVSGLEMRKWVDELFIIIMDMLQDSSLLAKRQVALWTLGQLVASTGYVVEPYRKYPTLLE  866

logo
            VLLNFLKTEQNQGTRREAIRVLGLLGALDPYKHKVNIGMIDQSRDASAVSLSESKSSQDSSDYSTSEMLVNMGNLPLDEFYPAVSMVALMRIFRDQSLSHHHTMVVQAIT
SIROLIMUS   ..............................................................................................................   28
PF00454.1   ..............................................................................................................    0
PF02259     ..............................................................................................................    0
PF02259.1   ..............................................................................................................    0
PF08771     ..............................................................................................................    0
PF11865     VLLNFLKTEQNQGTRREAIRVLGLLGALDPYKHKVNIGMIDQSRDASAVSLSESKSSQDSSDYSTSEMLVNMGNLPLDEFYPAVSMVALMRIFRDQSLSHHHTMVVQAIT  123
PF00454     ..............................................................................................................    0
PF02260     ..............................................................................................................    0
MTOR-203    ..............................................................................................................    0
MTOR-202    ..............................................................................................................    0
MTOR-201    VLLNFLKTEQNQGTRREAIRVLGLLGALDPYKHKVNIGMIDQSRDASAVSLSESKSSQDSSDYSTSEMLVNMGNLPLDEFYPAVSMVALMRIFRDQSLSHHHTMVVQAIT  976
```

```
logo
           FIFKSLGLKCVQFLPQVMPTFLNVIRVCDGAIREFLFQQLGMLVSFVKSHIRPYMDEIVTLMREFWVMNTSIQSTIILLIEQIVVALGGEFKLYLPQLIPHMLRVFMHDN
SIROLIMUS  ..............................................................................................................    28
PF00454.1  ..............................................................................................................     0
PF02259    ..............................................................................................................     0
PF02259.1  ..............................................................................................................     0
PF08771    ..............................................................................................................     0
PF11865    FIFKSLGLKCVQFLPQVMPTFLNVIRVCDGAIREFLFQQLGMLVSFVK..............................................................   171
PF00454    ..............................................................................................................     0
PF02260    ..............................................................................................................     0
MTOR-203   ..............................................................................................................     0
MTOR-202   ..............................................................................................................     0
MTOR-201   FIFKSLGLKCVQFLPQVMPTFLNVIRVCDGAIREFLFQQLGMLVSFVKSHIRPYMDEIVTLMREFWVMNTSIQSTIILLIEQIVVALGGEFKLYLPQLIPHMLRVFMHDN  1086
```

```
logo
           SPGRIVSIKLLAAIQLFGANLDDYLHLLLPPIVKLFDAPEAPLPSRKAALETVDRLTESLDFTDYASRIIHPIVRTLDQSPELRSTAMDTLSSLVFQLGKKYQIFIPMVN
SIROLIMUS  ..............................................................................................................    28
PF00454.1  ..............................................................................................................     0
PF02259    ..............................................................................................................     0
PF02259.1  ..............................................................................................................     0
PF08771    ..............................................................................................................     0
PF11865    ..............................................................................................................   171
PF00454    ..............................................................................................................     0
PF02260    ..............................................................................................................     0
MTOR-203   ..............................................................................................................     0
MTOR-202   ..............................................................................................................     0
MTOR-201   SPGRIVSIKLLAAIQLFGANLDDYLHLLLPPIVKLFDAPEAPLPSRKAALETVDRLTESLDFTDYASRIIHPIVRTLDQSPELRSTAMDTLSSLVFQLGKKYQIFIPMVN  1196
```

```
logo
           KVLVRHRINHQRYDVLICRIVKGYTLADEEEDPLIYQHRMLRSGQGDALASGPVETGPMKKLHVSTINLQKAWGAARRVSKDDWLEWLRRLSLELLKDSSSPSLRSCWAL
SIROLIMUS  ..............................................................................................................    28
PF00454.1  ..............................................................................................................     0
PF02259    ..............................................................................................................     0
PF02259.1  ..............................................................................................................     0
PF08771    ..............................................................................................................     0
PF11865    ..............................................................................................................   171
PF00454    ..............................................................................................................     0
PF02260    ..............................................................................................................     0
MTOR-203   ..............................................................................................................     0
MTOR-202   ..............................................................................................................     0
MTOR-201   KVLVRHRINHQRYDVLICRIVKGYTLADEEEDPLIYQHRMLRSGQGDALASGPVETGPMKKLHVSTINLQKAWGAARRVSKDDWLEWLRRLSLELLKDSSSPSLRSCWAL  1306
```

```
logo

           AQAYNPMARDLFNAAFVSCWSELNEDQQDELIRSIELALTSQDIAEVTQTLLNLAEFMEHSDKGPLPLRDDNGIVLLGERAAKCRAYAKALHYKELEFQKGPTPAILESL
SIROLIMUS  ..............................................................................................................    28
PF00454.1  ..............................................................................................................     0
PF02259    ..............................................................................................................     0
PF02259.1  ..............................................................................................................     0
PF08771    ..............................................................................................................     0
PF11865    ..............................................................................................................   171
PF00454    ..............................................................................................................     0
PF02260    ..............................................................................................................     0
MTOR-203   ..............................................................................................................     0
MTOR-202   ..............................................................................................................     0
MTOR-201   AQAYNPMARDLFNAAFVSCWSELNEDQQDELIRSIELALTSQDIAEVTQTLLNLAEFMEHSDKGPLPLRDDNGIVLLGERAAKCRAYAKALHYKELEFQKGPTPAILESL  1416

logo

           ISINNKLQQPEAAAGVLEYAMKHFGELEIQATWYEKLHEWEDALVAYDKKMDTNKDDPELMLGRMRCLEALGEWGQLHQQCCEKWTLVNDETQAKMARMAAAAAWGLGQW
SIROLIMUS  ..............................................................................................................    28
PF00454.1  ..............................................................................................................     0
PF02259    ................................................................................................ARMAAAAAWGLGQW    14
PF02259.1  ..............................................................................................................     0
PF08771    ..............................................................................................................     0
PF11865    ..............................................................................................................   171
PF00454    ..............................................................................................................     0
PF02260    ..............................................................................................................     0
MTOR-203   ..............................................................................................................     0
MTOR-202   ..............................................................................................................     0
MTOR-201   ISINNKLQQPEAAAGVLEYAMKHFGELEIQATWYEKLHEWEDALVAYDKKMDTNKDDPELMLGRMRCLEALGEWGQLHQQCCEKWTLVNDETQAKMARMAAAAAWGLGQW  1526

logo

           DSMEEYTCMIPRDTHDGAFYRAVLALHQDLFSLAQQCIDKARDLLDAELTAMAGESYSRAYGAMVSCHMLSELEEVIQYKLVPERREIIRQIWWERLQGCQRIVEDWQKI
SIROLIMUS  ..............................................................................................................    28
PF00454.1  ..............................................................................................................     0
PF02259    DSMEEYTCMIPRDTHDGAFYRAVLALHQDLFSLAQQCIDKARDLLDAELTAMAGESYSRAYGAMVSCHMLSELEEVIQYKLVPERREIIRQIWWERLQGCQRIVEDWQKI   124
PF02259.1  ..............................................................................................................     0
PF08771    ..............................................................................................................     0
PF11865    ..............................................................................................................   171
PF00454    ..............................................................................................................     0
PF02260    ..............................................................................................................     0
MTOR-203   ..............................................................................................................     0
MTOR-202   ..............................................................................................................     0
MTOR-201   DSMEEYTCMIPRDTHDGAFYRAVLALHQDLFSLAQQCIDKARDLLDAELTAMAGESYSRAYGAMVSCHMLSELEEVIQYKLVPERREIIRQIWWERLQGCQRIVEDWQKI  1636
```

```
logo
            LMVRSLVVSPHEDMRTWLKYASLCGKSGRLALAHKTLVLLLGVDPSRQLDHPLPTVHPQVTYAYMKNMWKSARKIDAFQHMQHFVQTMQQQAQHAIATED
SIROLIMUS   ..............................................................................................................   28
PF00454.1   ..............................................................................................................    0
PF02259     LMVRSLVVSPHEDMRTWLKYASLCGKSGRLALAHKTLVLLLGVDPSRQLDHPLPTVHPQVTYAYMKNMWKSARKIDAFQHMQHFVQTMQQQAQHAIATED..........  224
PF02259.1   ..............................................................................................................    0
PF08771     ..............................................................................................................    0
PF11865     ..............................................................................................................  171
PF00454     ..............................................................................................................    0
PF02260     ..............................................................................................................    0
MTOR-203    ..............................................................................................................    0
MTOR-202    ..............................................................................................................    0
MTOR-201    LMVRSLVVSPHEDMRTWLKYASLCGKSGRLALAHKTLVLLLGVDPSRQLDHPLPTVHPQVTYAYMKNMWKSARKIDAFQHMQHFVQTMQQQAQHAIATED.......... 1736

logo
                                                                       QQ HKQE LHK LMAR CFLKL GEWQ LNLQG INES TIPKV LQYYS
SIROLIMUS   ..............................................................................................................   28
PF00454.1   ..............................................................................................................    0
PF02259     ...........................................................QQ.HKQE.LHK.LMAR.CFLKL.GEWQ.LNLQG.INES.TIPKV.LQYYS.  265
PF02259.1   ..............................................................................................................    0
PF08771     ..............................................................................................................    0
PF11865     ..............................................................................................................  171
PF00454     ..............................................................................................................    0
PF02260     ..............................................................................................................    0
MTOR-203    ..............................................................................................................    0
MTOR-202    ..............................................................................................................    0
MTOR-201    ...........................................................QQ.HKQE.LHK.LMAR.CFLKL.GEWQ.LNLQG.INES.TIPKV.LQYYS. 1777

logo
            AATEHD RSWYKA WHAWAV MNFEAVL HYKHQN QARDEKK KLRHAS GANITNA TTAATTA ATATTTAS TEGSNSES EAESTENS PTPSPLQKKVT EDLS
SIROLIMUS   ..............................................................................................................   28
PF00454.1   ..............................................................................................................    0
PF02259     AATEHD.RSWYKA.WHAWAV.MNFEAVL.HYKHQN.QARDEKK.KLRHAS.GANITNA.TTAATTA.ATATTTAS.TEGSNSES.EAESTENS.PTPSPLQKKVT.EDLS  362
PF02259.1   .....................MNFEAVL.HYKHQN.QARDEKK.KLRHAS.GANITNA.TTAATTA.ATATTTAS.TEGSNSES.EAESTENS.PTPSPLQKKVT.EDLS   79
PF08771     ..............................................................................................................    0
PF11865     ..............................................................................................................  171
PF00454     ..............................................................................................................    0
PF02260     ..............................................................................................................    0
MTOR-203    ..............................................................................................................    0
MTOR-202    .....................MNFEAVL.HYKHQN.QARDEKK.KLRHAS.GANITNA.TTAATTA.ATATTTAS.TEGSNSES.EAESTENS.PTPSPLQKKVT.EDLS   79
MTOR-201    AATEHD.RSWYKA.WHAWAV.MNFEAVL.HYKHQN.QARDEKK.KLRHAS.GANITNA.TTAATTA.ATATTTAS.TEGSNSES.EAESTENS.PTPSPLQKKVT.EDLS 1874
```

```
logo
              KTLLMYTV PAVQGFFRSISLS RGNNLQDTLRVLTLWF DYGHWPDVNEALVEG VKAIQIDTWLQVIPQLIARIDTPRPLVGRLIHQLLTDIGRYHPQALIYPLTVAS
SIROLIMUS     ..............................................................................................................    28
PF00454.1     ..............................................................................................................     0
PF02259       KTLLMYTV.PAVQGFFRSISLS.RGNNLQDTLRVLT..........................................................................   396
PF02259.1     KTLLMYTV.PAVQGFFRSISLS.RGNNLQDTLRVLT..........................................................................   113
PF08771       ..............................................................................................................     0
PF11865       ..............................................................................................................   171
PF00454       ..............................................................................................................     0
PF02260       ..............................................................................................................     0
MTOR-203      ..............................................................................................................     0
MTOR-202      KTLLMYTV.PAVQGFFRSISLS.RGNNLQDTLRVLTLWF.DYGHWPDVNEALVEG.VKAIQIDTWLQVIPQLIARIDTPRPLVGRLIHQLLTDIGRYHPQALIYPLTVAS   185
MTOR-201      KTLLMYTV.PAVQGFFRSISLS.RGNNLQDTLRVLTLWF.DYGHWPDVNEALVEG.VKAIQIDTWLQVIPQLIARIDTPRPLVGRLIHQLLTDIGRYHPQALIYPLTVAS  1980

logo
              KSTTTARHNAANKILKNMCEHSNTLVQQAMMVSEELIRVAILWHEMWHEGLEEASRLYFGERNVKGMFEVLEPLHAMMERGPQTLKETSFNQAYGRDLMEAQEWCRKYMK
SIROLIMUS     ..................................................LE..SR..FG..................................................    34
PF00454.1     ..............................................................................................................     0
PF02259       ..............................................................................................................   396
PF02259.1     ..............................................................................................................   113
PF08771       ..................................ELIRVAILWHEMWHEGLEEASRLYFGERNVKGMFEVLEPLHAMMERGPQTLKETSFNQAYGRDLMEAQEWCRKYMK    76
PF11865       ..............................................................................................................   171
PF00454       ..............................................................................................................     0
PF02260       ..............................................................................................................     0
MTOR-203      ..............................................................................................................     0
MTOR-202      KSTTTARHNAANKILKNMCEHSNTLVQQAMMVSEELIRVAILWHEMWHEGLEEASRLYFGERNVKGMFEVLEPLHAMMERGPQTLKETSFNQAYGRDLMEAQEWCRKYMK   295
MTOR-201      KSTTTARHNAANKILKNMCEHSNTLVQQAMMVSEELIRVAILWHEMWHEGLEEASRLYFGERNVKGMFEVLEPLHAMMERGPQTLKETSFNQAYGRDLMEAQEWCRKYMK  2090

logo
              SGNVKDLTQAWDLYYHVFRRISKQLPQLTSLELQYVSPKLLMCRDLELAVPGTYDPNQPIIRIQSIAPSLQVITSKQRPRKLTLMGSNGHEFVFLLKGHEDLRQDERVMQ
SIROLIMUS     .......T..WD..Y..F............................................................................................    39
PF00454.1     ..............................................................................................................     0
PF02259       ..............................................................................................................   396
PF02259.1     ..............................................................................................................   113
PF08771       SGNVKDLTQAWDLYYHVFRRISK.......................................................................................    99
PF11865       ..............................................................................................................   171
PF00454       ............................................................................................VFLLKGHEDLRQDERVMQ    18
PF02260       ..............................................................................................................     0
MTOR-203      ..............................................................................................................     0
MTOR-202      SGNVKDLTQAWDLYYHVFRRISKQLPQLTSLELQYVSPKLLMCRDLELAVPGTYDPNQPIIRIQSIAPSLQVITSKQRPRKLTLMGSNGHEFVFLLKGHEDLRQDERVMQ   405
MTOR-201      SGNVKDLTQAWDLYYHVFRRISKQLPQLTSLELQYVSPKLLMCRDLELAVPGTYDPNQPIIRIQSIAPSLQVITSKQRPRKLTLMGSNGHEFVFLLKGHEDLRQDERVMQ  2200
```

```
logo
           LFGLVNTLLANDPTSLRKNLSIQRYAVIPLSTNSGLIGWVPHCDTLHALIRDYREKKKILLNIEHRIMLRMAPDYDHLTLMQKVEVFEHAVNNTAGDDLAKLLWLKSPSS
SIROLIMUS  ..............................................................................................................   39
PF00454.1  ..............................................................................................................    0
PF02259    ..............................................................................................................  396
PF02259.1  ..............................................................................................................  113
PF08771    ..............................................................................................................   99
PF11865    ..............................................................................................................  171
PF00454    LFGLVNTLLANDPTSLRKNLSIQRYAVIPLSTNSGLIGWVPHCDTLHALIRDYREKKKILLNIEHRIMLRMAPDYDHLTLMQKVEVFEHAVNNTAGDDLAKLLWLKSPSS  128
PF02260    ..............................................................................................................    0
MTOR-203   ..............................................................................................................    0
MTOR-202   LFGLVNTLLANDPTSLRKNLSIQRYAVIPLSTNSGLIGWVPHCDTLHALIRDYREKKKILLNIEHRIMLRMAPDYDHLTLMQKVEVFEHAVNNTAGDDLAKLLWLKSPSS  515
MTOR-201   LFGLVNTLLANDPTSLRKNLSIQRYAVIPLSTNSGLIGWVPHCDTLHALIRDYREKKKILLNIEHRIMLRMAPDYDHLTLMQKVEVFEHAVNNTAGDDLAKLLWLKSPSS 2310

logo
           EVWFDRRTNYTRSLAVMSMVGYILGLGDRHPSNLMLDRLSGKILHIDFGDCFEVAMTREKFPEKIPFRLTRMLTNAMEVTGLDGNYRITCHTVMEVLREHKDSVMAVLEA
SIROLIMUS  ..............................................................................................................   39
PF00454.1  ..................................MLDRLSGKILHIDFGDCFEVAMTREKFPEKIPFRLTRMLTNAMEVTGLDGNYRITCHTVMEVLREHKDSVMAVLEA   76
PF02259    ..............................................................................................................  396
PF02259.1  ..............................................................................................................  113
PF08771    ..............................................................................................................   99
PF11865    ..............................................................................................................  171
PF00454    EVWFDRRTNYTRSLAVMSMVGYILGLGDRHPSNLMLDRLSGKILHIDFGDCFEVAMTREKFPEKIPFRLTRMLTNAMEVTGLDGNYRITCHTVMEVLREHKDSVMAVLEA  238
PF02260    ..............................................................................................................    0
MTOR-203   ..................................MLDRLSGKILHIDFGDCFEVAMTREKFPEKIPFRLTRMLTNAMEVTGLDGNYRITCHTVMEVLREHKDSVMAVLEA   76
MTOR-202   EVWFDRRTNYTRSLAVMSMVGYILGLGDRHPSNLMLDRLSGKILHIDFGDCFEVAMTREKFPEKIPFRLTRMLTNAMEVTGLDGNYRITCHTVMEVLREHKDSVMAVLEA  625
MTOR-201   EVWFDRRTNYTRSLAVMSMVGYILGLGDRHPSNLMLDRLSGKILHIDFGDCFEVAMTREKFPEKIPFRLTRMLTNAMEVTGLDGNYRITCHTVMEVLREHKDSVMAVLEA 2420

logo
           FVYDPLLNWRLMDTNTKGNKRSRTRTDSYSAGQSVEILDGVELGEPAHKKTGTTVPESIHSFIGDGLVKPEALNKKAIQIINRVRDKLTGRDFSHDDTLDVPTQVELLIK
SIROLIMUS  ..............................................................................................................   39
PF00454.1  FVYDPLLNWR....................................................................................................   86
PF02259    ..............................................................................................................  396
PF02259.1  ..............................................................................................................  113
PF08771    ..............................................................................................................   99
PF11865    ..............................................................................................................  171
PF00454    FVYDPLLNWR....................................................................................................  248
PF02260    ..................................................................................................LDVPTQVELLIK   12
MTOR-203   FVYDPLLNWRLMDTNTKGNKRSRTRTDSYSAGQSVEILDGVELGEPAHKKTGTTVPESIHSFIGDGLVKPEALNKKAIQIINRVR.........................  161
MTOR-202   FVYDPLLNWRLMDTNTKGNKRSRTRTDSYSAGQSVEILDGVELGEPAHKKTGTTVPESIHSFIGDGLVKPEALNKKAIQIINRVRDKLTGRDFSHDDTLDVPTQVELLIK  735
MTOR-201   FVYDPLLNWRLMDTNTKGNKRSRTRTDSYSAGQSVEILDGVELGEPAHKKTGTTVPESIHSFIGDGLVKPEALNKKAIQIINRVRDKLTGRDFSHDDTLDVPTQVELLIK 2530
```

```
logo
            QATSHENLCQCYIGWCPFW
SIROLIMUS   ...................     39
PF00454.1   ...................     86
PF02259     ...................    396
PF02259.1   ...................    113
PF08771     ...................     99
PF11865     ...................    171
PF00454     ...................    248
PF02260     QATSHENLCQCYIGWCPFW     31
MTOR-203    ...................    161
MTOR-202    QATSHENLCQCYIGWCPFW    754
MTOR-201    QATSHENLCQCYIGWCPFW   2549
```

X non conserved
X similar
X ≥ 0% conserved
X ≥ 50% conserved

```
logo
```

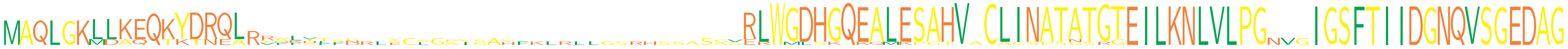

```
PEVONEDISTAT  ...........................................................................G.GG...............................   3
PF00899.3     ......................................................WGDHGQEALESAHV.CLINATATGTEILKNLVLPG...IGSFTIIDGNQVSGEDAG  52
PF00899.2     ....................................................RLWGDHGQEALESAHV.CLINATATGTEILKNLVLPG...IGSFTIIDGNQVSGEDAG  54
PF00899.4     ....................................................RLWGDHGQEALESAHV.CLINATATGTEILKNLVLPG...IGSFTIIDGNQVSGEDAG  54
PF00899.1     ............YDRQL...................................RLWGDHGQEALESAHV.CLINATATGTEILKNLVLPGNVGIGSFTIIDGNQVSGEDAG  62
PF00899       ............YDRQL...................................RLWGDHGQEALESAHV.CLINATATGTEILKNLVLPG...IGSFTIIDGNQVSGEDAG  59
NAE1-209      ..............................................................................................................   0
NAE1-215      ......MDAQQTKTNEA...................................RLWGDHGQEALESAHV.CLINATATGTEILKNLVLPG...IGSFTIIDGNQVSGEDAG  65
NAE1-203      ......MDAQQTKTNEA...................................RLWGDHGQEALESAHV.CLINATATGTEILKNLVLPG...IGSFTIIDGNQVSGEDAG  65
NAE1-211      MAQLGKLLKEQKYDRQLRVQKTF.......................................................................................  23
NAE1-216      ..............................................................................................................   0
NAE1-204      ..............................................................................................................   0
NAE1-205      ..............................................................................................................   0
NAE1-218      MAQLGKLLKEQKYDRQLRYWFVY.N..Y..................................................................................  25
NAE1-214      MAQLGKLLKEQKYDRQLRRSLALSPRLECSGAISAHFKLRLLGSRHSSASASELWGDHGQEALESAHV.CLINATATGTEILKNLVLPG...IGSFTIIDGNQVSGEDAG 106
NAE1-207      ..............................................................................................................   0
NAE1-201      MAQLGKLLKEQKYDRQL...................................RLWGDHGQEALESAHV.CLINATATGTEILKNLVLPG...IGSFTIIDGNQVSGEDAG  71
NAE1-220      MAQLGKLLKEQKYDRQLRRFLHTFD..SCLGPYGAY.............SGVERWMLSKQKQMKPGTVACACSPSNSRG...............................  64
NAE1-221      MAQLGKLLKEQKYDRQL...................................RLWGDHGQEALESAHV.CLINATATGTEILKNLVLPG...IGSFTIIDGNQVSGEDAG  71
NAE1-202      MAQLGKLLKEQKYDRQL...................................RLWGDHGQEALESAHV.CLINATATGTEILKNLVLPGNVGIGSFTIIDGNQVSGEDAG  74
```

```
logo
```

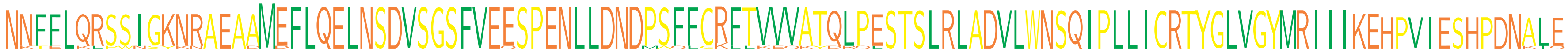

```
PEVONEDISTAT  .................DMD...............Q..............K.......................................................NKIQ  12
PF00899.3     NNFFLQRSSIGKNRAEAAMEFLQELNSDVSGSFVEESPENLLDNDPSFFCRFTVVVA..................................................... 109
PF00899.2     NNFFLQRSSIGKNRAEAAMEFLQELNSDVSGSFVEESPENLLDNDPSFFCRFTVVVATQLPESTSLRLADVLWNSQIPLLICRTYGLVGYMRIIIKEHPVIESHPDNALE 164
PF00899.4     NNFFLQRSSIGKNRAEAAMEFLQELNSDVSGSFVEESPENLLDNDPSFFCRFTVVVATQLPESTSLRLADVLWNSQIPLLICRTYGLVGYMRIII............... 149
PF00899.1     NNFFLQRSSIGKNRAEAAMEFLQELNSDVSGSFVEESPENLLDNDPSFFCRFTVVVATQLPESTSLRLADVLWNSQIPLLICRTYGLVGYMRIIIKEHPVIESHPDNALE 172
PF00899       NNFFLQRSSIGKNRAEAAMEFLQELNSDVSGSFVEESPENLLDNDPSFFCRFTVVVATQLPESTSLRLADVLWNSQIPLLICRTYGLVGYMRIIIKEHPVIESHPDNALE 169
NAE1-209      .............................................MAQLGKLLKEQKYDRQLSTSLRLADVLWNSQIPLLICRTYGLVGYMRIIIKEHPVIESHPDNALE  65
NAE1-215      NNFFLQRSSIGKNRAEAAMEFLQELNSDVSGSFVEESPENLLDNDPSFFCRFTVVVATQLPESTSLRLADVLWNSQIPLLICRTYGLVGYMRIIIKE............. 162
NAE1-203      NNFFLQRSSIGKNRAEAAMEFLQELNSDVSGSFVEESPENLLDNDPSFFCRFTVVVATQLPESTSLRLADVLWNSQIPLLICRTYGLVGYMRIIIKEHPVIESHPDNALE 175
NAE1-211      ..............................................................................................................  23
NAE1-216      ..............................................................................................................   0
NAE1-204      ..................MEFLQELNSDVSGSFVEESPENLLDNDPSFFCRFTVVVATQLPESTSLRLADVLWNSQIPLLICRTYGLVGYMRIIIKEHPVIESHPDNALE  92
NAE1-205      ..................MEFLQELNSDVSGSFVEESPENLLDNDPSFFCRFTVVVATQLPESTSLRLADVLWNSQIPLLICRTYGLVGYMRIIIKEHPVIESHPDNALE  92
NAE1-218      ..............................................................................................................  25
NAE1-214      NNFFLQRSSIGKNRAEAAMEFLQELNSDVSGSFVEESPENLLDNDPSFFCRFTVVVATQLP................................................. 167
NAE1-207      ..............................................................................................................   0
NAE1-201      NNFFLQRSSIGKNRAEAAMEFLQELNSDVSGSFVEESPENLLDNDPSFFCRFTVVVATQLPESTSLRLADVLWNSQIPLLICRTYGLVGYMRIIIKEHPVIESHPDNALE 181
NAE1-220      ..............................................................................................................  64
NAE1-221      NKTELKLPWNSYKN................................................................................................  85
NAE1-202      NNFFLQRSSIGKNRAEAAMEFLQELNSDVSGSFVEESPENLLDNDPSFFCRFTVVVATQLPESTSLRLADVLWNSQIPLLICRTYGLVGYMRIIIKEHPVIESHPDNALE 184
```

```
logo
               DLRLDKPFPELREHFQSYDLDHMEKKDHSHTPWIVIIAKYLAQWYSETNGRIPKTYKEKEDFRDLIRQGILKNENGAPEDEENFEEAIKNVNTALNTTQIPSSIEDIFND
PEVONEDISTAT   ..............................................................................................................   12
PF00899.3      ..............................................................................................................  109
PF00899.2      DLRLDKPFPELREHFQSYDLDHMEKKDHSHTPWIVIIAKYLAQWYSETNGRIPKTYKEKEDFRDLIRQGILKNENGAPEDEENFEEAIKNVNTALNTTQIPSSIEDIFND  274
PF00899.4      ..............................................................................................................  149
PF00899.1      DLRLDKPFPELREHFQSYDLDHMEKKDHSHTPWIVIIAKYLAQWYSETNGRIPKTYKEKEDFRDLIRQGILKNENGAPEDEENFEEAIKNVNTALNTTQIPSSIEDIFND  282
PF00899        DLRLDKPFPELREHFQSYDLDHMEKKDHSHTPWIVIIAKYLAQWYSETNGRIPKTYKEKEDFRDLIRQGILKNENGAPEDEENFEEAIKNVNTALNTTQIPSSIEDIFND  279
NAE1-209       DLRLDKPFPELREHFQSYDLDHMEKKDHSHTPWIVIIAKYLAQW..................................................................  109
NAE1-215       ..............................................................................................................  162
NAE1-203       DLRLDKPFPELREHFQSYDLDHMEKKDHSHTPWIVIIAKYLAQWYSETNGRIPKTYKEKEDFRDLIRQGILKNENGAPEDEENFEEAIKNVNTALNTTQIPSSIEDIFND  285
NAE1-211       ..............................................................................................................   23
NAE1-216       ..............................................................................................................    0
NAE1-204       DLRLDKPFPELREHFQSYDLDHMEKKDHSHTPWIVIIAKYLAQWYSETNGRIPKTYKEKEDFRDLIRQGILKNENGAPEDEENFEEAIKNVNTALNTTQIPSSIEDIFND  202
NAE1-205       DLRLDKPFPELREHFQSYDLDHMEKKDHSHTP..............................................................................  124
NAE1-218       ..............................................................................................................   25
NAE1-214       ..............................................................................................................  167
NAE1-207       ..............................................................................................................    0
NAE1-201       DLRLDKPFPELREHFQSYDLDHMEKKDHSHTPWIVIIAKYLAQWYSETNGRIPKTYKEKEDFRDLIRQGILKNENGAPEDEENFEEAIKNVNTALNTTQIPSSIEDIFND  291
NAE1-220       ..............................................................................................................   64
NAE1-221       ..............................................................................................................   85
NAE1-202       DLRLDKPFPELREHFQSYDLDHMEKKDHSHTPWIVIIAKYLAQWYSETNGRIPKTYKEKEDFRDLIRQGILKNENGAPEDEENFEEAIKNVNTALNTTQIPSSIEDIFND  294
```

```
logo
               DRCINITKQTPSFWILARALKEFVAKEGQGNLPVRGTIPDMIADSGKYIKLQNVYREKAKKDAAAVGNHVAKLLQSIGQAPESISEKELKLLCSNSAFLRVVRCRSLAEE
PEVONEDISTAT   ..............................................................................................................   12
PF00899.3      ..............................................................................................................  109
PF00899.2      DRCINITKQTPSFWILARALKEFVAKEGQGNLPVRGTIPDMIADSGKYIKLQNVYREKAKKDAAAVGNHVAKLLQSIGQAPESISEKELKLLCSNSAFLRVVRCRSLAEE  384
PF00899.4      ..............................................................................................................  149
PF00899.1      DRCINITKQTPSFWILARALKEFVAKEGQGNLPVRGTIPDMIADSGKYIKLQNVYREKAKKDAAAVGNHVAKLLQSIGQAPESISEKELKLLCSNSAFLRVVRCRSLAEE  392
PF00899        DRCINITKQTPSFWILARALKEFVAKEGQGNLPVRGTIPDMIADSGKYIKLQNVYREKAKKDAAAVGNHVAKLLQSIGQAPESISEKELKLLCSNSAFLRVVRCRSLAEE  389
NAE1-209       ..............................................................................................................  109
NAE1-215       ..............................................................................................................  162
NAE1-203       DRCINITKQTPSFWILARALKEFVAKEGQGNLPVRGTIPDMIADSGKYIKLQNVYREKAKKDAAAVGNHVAKLLQSIGQAPESISEKELKLLCSNSAFLRVVRCRSLAEE  395
NAE1-211       ..............................................................................................................   23
NAE1-216       ...............................................................................APESISEKELKLLCSNSAFLRVVRCRSLAEE   31
NAE1-204       DRCINITKQTPSFWILARALKEFVAKEGQGNLPVRGTIPDMIADSGKYIKLQNVYREKAKKDAAAVGNHVAKLLQSIGQAPESISEKELKLLCSNSAFLRVVRCRSLAEE  312
NAE1-205       ..............................................................................................................  124
NAE1-218       ..............................................................................................................   25
NAE1-214       ..............................................................................................................  167
NAE1-207       ..............................................................................................................    0
NAE1-201       DRCINITKQTPSFWILARALKEFVAKEGQGNLPVRGTIPDMIADSGKYIKLQNVYREKAKKDAAAVGNHVAKLLQSIGQAPESISEKELKLLCSNSAFLRVVRCRSLAEE  401
NAE1-220       ..............................................................................................................   64
NAE1-221       ..............................................................................................................   85
NAE1-202       DRCINITKQTPSFWILARALKEFVAKEGQGNLPVRGTIPDMIADSGKYIKLQNVYREKAKKDAAAVGNHVAKLLQSIGQAPESISEKELKLLCSNSAFLRVVRCRSLAEE  404
```

```
logo          YGLDTINKDEIISSMDNPDNEIVLYLMLRAVDRFHKQQGRYPGVSNYQVEEDIGKLKSCLTGFLQEYGLSVMVKDDYVHEFCR          Y  GAAEPH TIAAFLG
PEVONEDISTAT  ...................................................................GLD..IA..W.................................    18
PF00899.3     ..............................................................................................................   109
PF00899.2     YGLDTINKDEIISSMDNPDNEIVLYLMLRAVDRFHKQQGRYPGVSNYQVEEDIGKLKSCLTGFLQEYGLSVMVKDDYVHEFCR..........Y..GAAEPH.TIAAFLG   481
PF00899.4     ..............................................................................................................   149
PF00899.1     YGLDTINKDEIISSMDNPDNEIVLYLMLRAVDRFHKQQGRYPGVSNYQVEEDIGKLKSCLTGFLQEYGLSVMVKDDYVHEFCR..........Y..GAAEPH.TIAAFLG   489
PF00899       YGLDTINKDEIISSMDNPDNEIVLYLMLRAVDRFHKQQGRYPGVSNYQVEEDIGKLKSCLTGFLQEYGLSVMVKDDYVHEFCR..........Y..GAAEPH.TIAAFLG   486
NAE1-209      ..............................................................................................................   109
NAE1-215      ..............................................................................................................   162
NAE1-203      YGLDTINKDEIISSMDNPDNEIVLYLMLRAVDRFHKQQGRYPGVSNYQVEEDIGKLKSCLTGFLQEYGLSVMVKDDYVHEFCR..........Y..GAAEPH.TIAAFLG   492
NAE1-211      ..............................................................................................................    23
NAE1-216      YGLDTINKDEIISSMDNPDNEIVLYLMLRAVDRFHKQQGRYPGVSNYQVEEDIGKLKSCLTGFLQEYGLSVMVKDDYVHELSC..........CSRGHQNNHQTICNF..   129
NAE1-204      YGLDTINKDEIISSMDNPDNEIVLYLMLRAVDRFHKQQGRYPGVSNYQVEEDIGKLKSCLTGFLQEYGLSVMVKDDYVHEFCR..........Y..GAAEPH.TIAAFLG   409
NAE1-205      ..............................................................................................................   124
NAE1-218      ..............................................................................................................    25
NAE1-214      ..............................................................................................................   167
NAE1-207      .................................FHKQQGRYPGVSNYQVEEDIGKLKSCLTGFLQEYGLSVMVKDDYVHELLIKIHVSVVSFSCRYGAAEPH.TIAAFLG    76
NAE1-201      YGLDTINKDEIISSMDNPDNEIVLYLMLRAVDRFHKQQGRYPGVSNYQVEEDIGKLKSCLTGFLQEYGLSVMVKDDYVHEFCR..........Y..GAAEPH.TIAAFLG   498
NAE1-220      ..............................................................................................................    64
NAE1-221      ..............................................................................................................    85
NAE1-202      YGLDTINKDEIISSMDNPDNEIVLYLMLRAVDRFHKQQGRYPGVSNYQVEEDIGKLKSCLTGFLQEYGLSVMVKDDYVHEFCR..........Y..GAAEPH.TIAAFLG   501

logo          GAAAQEVIKIITKQFVIFNNTYIYSGMSQTSATFQL
PEVONEDISTAT  ....................................    18
PF00899.3     ....................................   109
PF00899.2     GAAAQEVIKIITKQFVIFNN................   501
PF00899.4     ....................................   149
PF00899.1     GAAAQEVIKIITKQFVIFNN................   509
PF00899       GAAAQEVIKIITKQFVIFNN................   506
NAE1-209      ....................................   109
NAE1-215      ....................................   162
NAE1-203      GAAAQEVIKIITKQFVIFNNTYIYSGMSQTSATFQL   528
NAE1-211      ....................................    23
NAE1-216      ....................................   129
NAE1-204      GAAAQEVIKIITKQFVIFNNTYIYSGMSQTSATFQL   445
NAE1-205      ....................................   124
NAE1-218      ....................................    25
NAE1-214      ....................................   167
NAE1-207      GAAAQEVIKIITKQFVIFNNTYIYSGMSQTSATFQL   112
NAE1-201      GAAAQEVIKIITKQFVIFNNTYIYSGMSQTSATFQL   534
NAE1-220      ....................................    64
NAE1-221      ....................................    85
NAE1-202      GAAAQEVIKIITKQFVIFNNTYIYSGMSQTSATFQL   537
```

X non conserved

X similar

X $\geq$0% conserved

X $\geq$50% conserved

```
logo
             MSGLGDSSSDPANPDSHKRKGSPCDTLASSTEKRRREQENKYLEELAELLSANISDIDSLSVKPDKCKILKKTVDQIQLMKRMEQEKSTTDDDVQKSDISSSSQGVIEKE
GENISTEIN    ..............................................................................................................    0
PF14598      ..............................................................................................................    0
PF00989      ..............................................................................................................    0
PF08832      ..............................................................................................................    0
PF00010      ............................SSTEKRRREQENKYLEELAELLSANISDIDSLSVKPDKCKILKKTVDQIQL...............................   51
PF08815      ..............................................................................................................    0
PF16665      ..............................................................................................................    0
PF07469      ..............................................................................................................    0
NCOA1-204    MSGLGDSSSDPANPDSHKRKGSPCDTLASSTEKRRREQENKYLEELAELLSANISDIDSLSVKPDKCKILKKTVDQIQLMKRMEQEKSTTDDDVQKSDISSSSQGVIEKE  110
NCOA1-202    MSGLGDSSSDPANPDSHKRKGSPCDTLASSTEKRRREQENKYLEELAELLSANISDIDSLSVKPDKCKILKKTVDQIQLMKRMEQEKSTTDDDVQKSDISSSSQGVIEKE  110
NCOA1-201    MSGLGDSSSDPANPDSHKRKGSPCDTLASSTEKRRREQENKYLEELAELLSANISDIDSLSVKPDKCKILKKTVDQIQLMKRMEQEKSTTDDDVQKSDISSSSQGVIEKE  110
NCOA1-206    ..............................................................................................................    0
NCOA1-205    MSGLGDSSSDPANPDSHKRKGSPCDTLASSTEKRRREQENKYLEELAELLSANISDIDSLSVKPDKCKILKKTVDQIQLMKRMEQEKSTTDDDVQKSDISSSSQGVIEKE  110
NCOA1-203    MSGLGDSSSDPANPDSHKRKGSPCDTLASSTEKRRREQENKYLEELAELLSANISDIDSLSVKPDKCKILKKTVDQIQLMKRMEQEKSTTDDDVQKSDISSSSQGVIEKE  110

logo
             SLGPLLLEALDGFFFVVNCEGRIVFVSENVTSYLGYNQEELMNTSVYSILHVGDHAEFVKNLLPKSLVNGVPWPQEATRRNSHTFNCRMLIHPPDEPGTENQEACQRYEV
GENISTEIN    .L..L..............E.........................................L.................R..............................    5
PF14598      ..............................................................................................................    0
PF00989      ....LLLEALDGFFFVVNCEGRIVFVSENVTSYLGYNQEELMNTSVYSILHVGDHAEFVKNL................................................   58
PF08832      ..............................................................................................................    0
PF00010      ..............................................................................................................   51
PF08815      ..............................................................................................................    0
PF16665      ..............................................................................................................    0
PF07469      ..............................................................................................................    0
NCOA1-204    SLGPLLLEALDGFFFVVNCEGRIVFVSENVTSYLGYNQEELMNTSVYSILHVGDHAEFVKNLLPKSLVNGVPWPQEATRRNSHTFNCRMLIHPPDEPGTENQEACQRYEV  220
NCOA1-202    SLGPLLLEALDGFFFVVNCEGRIVFVSENVTSYLGYNQEELMNTSVYSILHVGDHAEFVKNLLPKSLVNGVPWPQEATRRNSHTFNCRMLIHPPDEPGTENQEACQRYEV  220
NCOA1-201    SLGPLLLEALDGFFFVVNCEGRIVFVSENVTSYLGYNQEELMNTSVYSILHVGDHAEFVKNLLPKSLVNGVPWPQEATRRNSHTFNCRMLIHPPDEPGTENQEACQRYEV  220
NCOA1-206    .........................................MNTSVYSILHVGDHAEFVKNLLPKSLVNGVPWPQEATRRNSHTFNCRMLIHPPDEPGTENQEACQRYEV   69
NCOA1-205    SLGPLLLEALDGFFFVVNCEGRIVFVSENVTSYLGYNQEELMNTSVYSILHVGDHAEFVKNLLPKSLVNGVPWPQEATRRNSHTFNCRMLIHPPDEPGTENQEACQRYEV  220
NCOA1-203    SLGPLLLEALDGFFFVVNCEGRIVFVSENVTSYLGYNQEELMNTSVYSILHVGDHAEFVKNLLPKSLVNGVPWPQEATRRNSHTFNCRMLIHPPDEPGTENQEACQRYEV  220

logo
             MQCFTVSQPKSIQEDGEDFQSCLICIARRLPRPPAITGVESFMTKQDTTGKIISIDTSSLRAAGRTGWEDLVRKCIYAFFQPQGREPSYARQLFQEVMTRGTASSPSYRF
GENISTEIN    ...........I..................................................................................................    6
PF14598      .......................................ESFMTKQDTTGKIISIDTSSLRAAGRTGWEDLVRKCIYAFFQPQGREPSYARQLFQEVMTRGTASSPSYRF   71
PF00989      ..............................................................................................................   58
PF08832      ..............................................................................................................    0
PF00010      ..............................................................................................................   51
PF08815      ..............................................................................................................    0
PF16665      ..............................................................................................................    0
PF07469      ..............................................................................................................    0
NCOA1-204    MQCFTVSQPKSIQEDGEDFQSCLICIARRLPRPPAITGVESFMTKQDTTGKIISIDTSSLRAAGRTGWEDLVRKCIYAFFQPQGREPSYARQLFQEVMTRGTASSPSYRF  330
NCOA1-202    MQCFTVSQPKSIQEDGEDFQSCLICIARRLPRPPAITGVESFMTKQDTTGKIISIDTSSLRAAGRTGWEDLVRKCIYAFFQPQGREPSYARQLFQEVMTRGTASSPSYRF  330
NCOA1-201    MQCFTVSQPKSIQEDGEDFQSCLICIARRLPRPPAITGVESFMTKQDTTGKIISIDTSSLRAAGRTGWEDLVRKCIYAFFQPQGREPSYARQLFQEVMTRGTASSPSYRF  330
NCOA1-206    MQCFTVSQPKSIQEDGEDFQSCLICIARRLPRPPAITGVESFMTKQDTTGKIISIDTSSLRAAGRTGWEDLVRKCIYAFFQPQGREPSYARQLFQEVMTRGTASSPSYRF  179
NCOA1-205    MQCFTVSQPKSIQEDGEDFQSCLICIARRLPRPPAITGVESFMTKQDTTGKIISIDTSSLRAAGRTGWEDLVRKCIYAFFQPQGREPSYARQLFQEVMTRGTASSPSYRF  330
NCOA1-203    MQCFTVSQPKSIQEDGEDFQSCLICIARRLPRPPAITGVESFMTKQDTTGKIISIDTSSLRAAGRTGWEDLVRKCIYAFFQPQGREPSYARQLFQEVMTRGTASSPSYRF  330
```

```
logo         ILNDGTMLSAHTKCKLCYPQSPDMQPFIMGIHIIDREHSGLSPQDDTNSGMSIPRVNPSVNPSISPAHGVARSSTLPPSNSNMVSTRINRQQSSDLHSSSHSNSSNSQGS
GENISTEIN    ...............................HL.............................................................................    8
PF14598      ILNDGTMLSAHTKCKLCYPQSPDMQPFIMGIHIIDREHS.......................................................................  110
PF00989      ..............................................................................................................   58
PF08832      ..............................................................................................................    0
PF00010      ..............................................................................................................   51
PF08815      ..............................................................................................................    0
PF16665      ..............................................................................................................    0
PF07469      ..............................................................................................................    0
NCOA1-204    ILNDGTMLSAHTKCKLCYPQSPDMQPFIMGIHIIDREHSGLSPQDDTNSGMSIPRVNPSVNPSISPAHGVARSSTLPPSNSNMVSTRINRQQSSDLHSSSHSNSSNSQGS  440
NCOA1-202    ILNDGTMLSAHTKCKLCYPQSPDMQPFIMGIHIIDREHSGLSPQDDTNSGMSIPRVNPSVNPSISPAHGVARSSTLPPSNSNMVSTRINRQQSSDLHSSSHSNSSNSQGS  440
NCOA1-201    ILNDGTMLSAHTKCKLCYPQSPDMQPFIMGIHIIDREHSGLSPQDDTNSGMSIPRVNPSVNPSISPAHGVARSSTLPPSNSNMVSTRINRQQSSDLHSSSHSNSSNSQGS  440
NCOA1-206    ILNDGTMLSAHTKCKLCYPQSPDMQPFIMGIHIIDREHSGLSPQDDTNSGMSIPRVNPSVNPSISPAHGVARSSTLPPSNSNMVSTRINRQQSSDLHSSSHSNSSNSQGS  289
NCOA1-205    ILNDGTMLSAHTKCKLCYPQSPDMQPFIMGIHIIDREHSGLSPQDDTNSGMSIPRVNPSVNPSISPAHGVARSSTLPPSNSNMVSTRINRQQSSDLHSSSHSNSSNSQGS  440
NCOA1-203    ILNDGTMLSAHTKCKLCYPQSPDMQPFIMGIHIIDREHSGLSPQDDTNSGMSIPRVNPSVNPSISPAHGVARSSTLPPSNSNMVSTRINRQQSSDLHSSSHSNSSNSQGS  440

logo         FGCSPGSQIVANVALNQGQASSQSSNPSLNLNNSPMEGTGISLAQFMSPRRQVTSGLATRPRMPNNSFPPNISTLSSPVGMTSSACNNNNRSYSNIPVTSLQGMNEGPNN
GENISTEIN    ..............................................................................................................    8
PF14598      ..............................................................................................................  110
PF00989      ..............................................................................................................   58
PF08832      ..............................................................................................................    0
PF00010      ..............................................................................................................   51
PF08815      ..............................................................................................................    0
PF16665      ...........................SLNLNNSPMEGTGISLAQFMSPRRQVTSGLATRPRMPNNSFPPNISTLSSPVGMTSSACNNNNRSYSNIPVTSLQGMNEGPNN   83
PF07469      ..............................................................................................................    0
NCOA1-204    FGCSPGSQIVANVALNQGQASSQSSNPSLNLNNSPMEGTGISLAQFMSPRRQVTSGLATRPRMPNNSFPPNISTLSSPVGMTSSACNNNNRSYSNIPVTSLQGMNEGPNN  550
NCOA1-202    FGCSPGSQIVANVALNQGQASSQSSNPSLNLNNSPMEGTGISLAQFMSPRRQVTSGLATRPRMPNNSFPPNISTLSSPVGMTSSACNNNNRSYSNIPVTSLQGMNEGPNN  550
NCOA1-201    FGCSPGSQIVANVALNQGQASSQSSNPSLNLNNSPMEGTGISLAQFMSPRRQVTSGLATRPRMPNNSFPPNISTLSSPVGMTSSACNNNNRSYSNIPVTSLQGMNEGPNN  550
NCOA1-206    FGCSPGSQIVANVALNQGQASSQSSNPSLNLNNSPMEGTGISLAQFMSPRRQVTSGLATRPRMPNNSFPPNISTLSSPVGMTSSACNNNNRSYSNIPVTSLQGMNEGPNN  399
NCOA1-205    FGCSPGSQIVANVALNQGQASSQSSNPSLNLNNSPMEGTGISLAQFMSPRRQVTSGLATRPRMPNNSFPPNISTLSSPVGMTSSACNNNNRSYSNIPVTSLQGMNEGPNN  550
NCOA1-203    FGCSPGSQIVANVALNQGQASSQSSNPSLNLNNSPMEGTGISLAQFMSPRRQVTSGLATRPRMPNNSFPPNISTLSSPVGMTSSACNNNNRSYSNIPVTSLQGMNEGPNN  550

logo         SVGFSASSPVLRQMSSQNSPSRLNIQPAKAESKDNKEIASILNEMIQSDNSSSDGKPLDSGLLHNNDRLSDGDSKYSQTSHKLVQLLTTTAEQQLRHADIDTSCKDVLSC
GENISTEIN    ..............................................................................................................    8
PF14598      ..............................................................................................................  110
PF00989      ..............................................................................................................   58
PF08832      ...............................................................................SHKLVQLLTTTAEQQLRHADIDTSCKDVLSC   31
PF00010      ..............................................................................................................   51
PF08815      ..............................................................................................................    0
PF16665      SVGFSASSPVLRQMSSQNSPSRLNIQPAKAESKDNKEIAS......................................................................  123
PF07469      ..............................................................................................................    0
NCOA1-204    SVGFSASSPVLRQMSSQNSPSRLNIQPAKAESKDNKEIASILNEMIQSDNSSSDGKPLDSGLLHNNDRLSDGDSKYSQTSHKLVQLLTTTAEQQLRHADIDTSCKDVLSC  660
NCOA1-202    SVGFSASSPVLRQMSSQNSPSRLNIQPAKAESKDNKEIASILNEMIQSDNSSSDGKPLDSGLLHNNDRLSDGDSKYSQTSHKLVQLLTTTAEQQLRHADIDTSCKDVLSC  660
NCOA1-201    SVGFSASSPVLRQMSSQNSPSRLNIQPAKAESKDNKEIASILNEMIQSDNSSSDGKPLDSGLLHNNDRLSDGDSKYSQTSHKLVQLLTTTAEQQLRHADIDTSCKDVLSC  660
NCOA1-206    SVGFSASSPVLRQMSSQNSPSRLNIQPAKAESKDNKEIASILNEMIQSDNSSSDGKPLDSGLLHNNDRLSDGDSKYSQTSHKLVQLLTTTAEQQLRHADIDTSCKDVLSC  509
NCOA1-205    SVGFSASSPVLRQMSSQNSPSRLNIQPAKAESKDNKEIASILNEMIQSDNSSSDGKPLDSGLLHNNDRLSDGDSKYSQTSHKLVQLLTTTAEQQLRHADIDTSCKDVLSC  660
NCOA1-203    SVGFSASSPVLRQMSSQNSPSRLNIQPAKAESKDNKEIASILNEMIQSDNSSSDGKPLDSGLLHNNDRLSDGDSKYSQTSHKLVQLLTTTAEQQLRHADIDTSCKDVLSC  660
```

```
logo        TGTSNSASANSSGGSCPSSHSSLTERHKILHRLLQEGSPSDITTLSVEPDKKDSASTSVSVTGQVQGNSSIKLELDASKKKESKDHQLLRYLLDKDEKDLRSTPNLSLDD
GENISTEIN   ..............................................................................................................     8
PF14598     ..............................................................................................................   110
PF00989     ..............................................................................................................    58
PF08832     TGTSNSASANSSGGSCPSSHSSLTERHKILHRLLQEGSPSDITTLSVE..............................................................    79
PF00010     ..............................................................................................................    51
PF08815     ..............................................................................................................     0
PF16665     ..............................................................................................................   123
PF07469     ..............................................................................................................     0
NCOA1-204   TGTSNSASANSSGGSCPSSHSSLTERHKILHRLLQEGSPSDITTLSVEPDKKDSASTSVSVTGQVQGNSSIKLELDASKKKESKDHQLLRYLLDKDEKDLRSTPNLSLDD   770
NCOA1-202   TGTSNSASANSSGGSCPSSHSSLTERHKILHRLLQEGSPSDITTLSVEPDKKDSASTSVSVTGQVQGNSSIKLELDASKKKESKDHQLLRYLLDKDEKDLRSTPNLSLDD   770
NCOA1-201   TGTSNSASANSSGGSCPSSHSSLTERHKILHRLLQEGSPSDITTLSVEPDKKDSASTSVSVTGQVQGNSSIKLELDASKKKESKDHQLLRYLLDKDEKDLRSTPNLSLDD   770
NCOA1-206   TGTSNSASANSSGGSCPSSHSSLTERHKILHRLLQEGSPSDITTLSVEPDKKDSASTSVSVTGQVQGNSSIKLELDASKKKESKDHQLLRYLLDKDEKDLRSTPNLSLDD   619
NCOA1-205   TGTSNSASANSSGGSCPSSHSSLTERHKILHRLLQEGSPSDITTLSVEPDKKDSASTSVSVTGQVQGNSSIKLELDASKKKESKDHQLLRYLLDKDEKDLRSTPNLSLDD   770
NCOA1-203   TGTSNSASANSSGGSCPSSHSSLTERHKILHRLLQEGSPSDITTLSVEPDKKDSASTSVSVTGQVQGNSSIKLELDASKKKESKDHQLLRYLLDKDEKDLRSTPNLSLDD   770

logo        VKVKVEKKEQMDPCNTNPTPMTKPTPEEIKLEAQSQFTADLDQFDQLLPTLEKAAQLPGLCETDRMDGAVTSVTIKSEILPASLQSATARPTSRLNRLPELELEAIDNQF
GENISTEIN   ..............................................................................................................     8
PF14598     ..............................................................................................................   110
PF00989     ..............................................................................................................    58
PF08832     ..............................................................................................................    79
PF00010     ..............................................................................................................    51
PF08815     ..............................................................................................................     0
PF16665     ..............................................................................................................   123
PF07469     ..............................................................................................................     0
NCOA1-204   VKVKVEKKEQMDPCNTNPTPMTKPTPEEIKLEAQSQFTADLDQFDQLLPTLEKAAQLPGLCETDRMDGAVTSVTIKSEILPASLQSATARPTSRLNRLPELELEAIDNQF   880
NCOA1-202   VKVKVEKKEQMDPCNTNPTPMTKPTPEEIKLEAQSQFTADLDQFDQLLPTLEKAAQLPGLCETDRMDGAVTSVTIKSEILPASLQSATARPTSRLNRLPELELEAIDNQF   880
NCOA1-201   VKVKVEKKEQMDPCNTNPTPMTKPTPEEIKLEAQSQFTADLDQFDQLLPTLEKAAQLPGLCETDRMDGAVTSVTIKSEILPASLQSATARPTSRLNRLPELELEAIDNQF   880
NCOA1-206   VKVKVEKKEQMDPCNTNPTPMTKPTPEEIKLEAQSQFTADLDQFDQLLPTLEKAAQLPGLCETDRMDGAVTSVTIKSEILPASLQSATARPTSRLNRLPELELEAIDNQF   729
NCOA1-205   VKVKVEKKEQMDPCNTNPTPMTKPTPEEIKLEAQSQFTADLDQFDQLLPTLEKAAQLPGLCETDRMDGAVTSVTIKSEILPASLQSATARPTSRLNRLPELELEAIDNQF   880
NCOA1-203   VKVKVEKKEQMDPCNTNPTPMTKPTPEEIKLEAQSQFTADLDQFDQLLPTLEKAAQLPGLCETDRMDGAVTSVTIKSEILPASLQSATARPTSRLNRLPELELEAIDNQF   880

logo        GQPGTGDQIPWTNNTVTAINQSKSEDQCISSQLDELLCPPTTVEGRNDEKALLEQLVSFLSGKDETELAELDRALGIDKLVQGGGLDVLSERFPPQQATPPLIMEERPNL
GENISTEIN   ..............................................................................................................     8
PF14598     ..............................................................................................................   110
PF00989     ..............................................................................................................    58
PF08832     ..............................................................................................................    79
PF00010     ..............................................................................................................    51
PF08815     ...........................................EGRNDEKALLEQLVSFLSGKDETELAELDRALGIDKLVQGGGLDVL.....................    46
PF16665     ..............................................................................................................   123
PF07469     ..............................................................................................................     0
NCOA1-204   GQPGTGDQIPWTNNTVTAINQSKSEDQCISSQLDELLCPPTTVEGRNDEKALLEQLVSFLSGKDETELAELDRALGIDKLVQGGGLDVLSERFPPQQATPPLIMEERPNL   990
NCOA1-202   GQPGTGDQIPWTNNTVTAINQSKSEDQCISSQLDELLCPPTTVEGRNDEKALLEQLVSFLSGKDETELAELDRALGIDKLVQGGGLDVLSERFPPQQATPPLIMEERPNL   990
NCOA1-201   GQPGTGDQIPWTNNTVTAINQSKSEDQCISSQLDELLCPPTTVEGRNDEKALLEQLVSFLSGKDETELAELDRALGIDKLVQGGGLDVLSERFPPQQATPPLIMEERPNL   990
NCOA1-206   GQPGTGDQIPWTNNTVTAINQSKSEDQCISSQLDELLCPPTTVEGRNDEKALLEQLVSFLSGKDETELAELDRALGIDKLVQGGGLDVLSERFPPQQATPPLIMEERPNL   839
NCOA1-205   GQPGTGDQIPWTNNTVTAINQSKSEDQCISSQLDELLCPPTTVEGRNDEKALLEQLVSFLSGKDETELAELDRALGIDKLVQGGGLDVLSERFPPQQATPPLIMEERPNL   990
NCOA1-203   GQPGTGDQIPWTNNTVTAINQSKSEDQCISSQLDELLCPPTTVEGRNDEKALLEQLVSFLSGKDETELAELDRALGIDKLVQGGGLDVLSERFPPQQATPPLIMEERPNL   990
```

```
logo
             YSQPYSSPSPTANLPSPFQGMVRQKPSLGTMPVQVTPPRGAFSPGMGMQPRQTLNRPPAAPNQLRLQLQQRLQGQQQLIHQNRQAILNQFAATAPVGINMRSGMQQQITP
GENISTEIN    ..............................................................................................................    8
PF14598      ..............................................................................................................  110
PF00989      ..............................................................................................................   58
PF08832      ..............................................................................................................   79
PF00010      ..............................................................................................................   51
PF08815      ..............................................................................................................   46
PF16665      ..............................................................................................................  123
PF07469      ..............................................................................................................    0
NCOA1-204    YSQPYSSPSPTANLPSPFQGMVRQKPSLGTMPVQVTPPRGAFSPGMGMQPRQTLNRPPAAPNQLRLQLQQRLQGQQQLIHQNRQAILNQFAATAPVGINMRSGMQQQITP 1100
NCOA1-202    YSQPYSSPSPTANLPSPFQGMVRQKPSLGTMPVQVTPPRGAFSPGMGMQPRQTLNRPPAAPNQLRLQLQQRLQGQQQLIHQNRQAILNQFAATAPVGINMRSGMQQQITP 1100
NCOA1-201    YSQPYSSPSPTANLPSPFQGMVRQKPSLGTMPVQVTPPRGAFSPGMGMQPRQTLNRPPAAPNQLRLQLQQRLQGQQQLIHQNRQAILNQFAATAPVGINMRSGMQQQITP 1100
NCOA1-206    YSQPYSSPSPTANLPSPFQGMVRQKPSLGTMPVQVTPPRGAFSPGMGMQPRQTLNRPPAAPNQLRLQLQQRLQGQQQLIHQNRQAILNQFAATAPVGINMRSGMQQQITP  949
NCOA1-205    YSQPYSSPSPTANLPSPFQGMVRQKPSLGTMPVQVTPPRGAFSPGMGMQPRQTLNRPPAAPNQLRLQLQQRLQGQQQLIHQNRQAILNQFAATAPVGINMRSGMQQQITP 1100
NCOA1-203    YSQPYSSPSPTANLPSPFQGMVRQKPSLGTMPVQVTPPRGAFSPGMGMQPRQTLNRPPAAPNQLRLQLQQRLQGQQQLIHQNRQAILNQFAATAPVGINMRSGMQQQITP 1100

logo
             QPPLNAQMLAQRQRELYSQQHRQRQLIQQQRAMLMRQQSFGNNLPPSSGLPVQMGNPRLPQGAPQQFPYPPNYGTNPGTPPASTSPFSQLAANPEASLANRNSMVSRGMT
GENISTEIN    ..............................................................................................................    8
PF14598      ..............................................................................................................  110
PF00989      ..............................................................................................................   58
PF08832      ..............................................................................................................   79
PF00010      ..............................................................................................................   51
PF08815      ..............................................................................................................   46
PF16665      ..............................................................................................................  123
PF07469      ................................................GLPVQMGNPRLPQGAPQQFPYPPNYGTNPGTPPASTSPFSQLAANPEASLANRNSM......   56
NCOA1-204    QPPLNAQMLAQRQRELYSQQHRQRQLIQQQRAMLMRQQSFGNNLPPSSGLPVQMGNPRLPQGAPQQFPYPPNYGTNPGTPPASTSPFSQLAANPEASLANRNSMVSRGMT 1210
NCOA1-202    QPPLNAQMLAQRQRELYSQQHRQRQLIQQQRAMLMRQQSFGNNLPPSSGLPVQMGNPRLPQGAPQQFPYPPNYGTNPGTPPASTSPFSQLAANPEASLANRNSMVSRGMT 1210
NCOA1-201    QPPLNAQMLAQRQRELYSQQHRQRQLIQQQRAMLMRQQSFGNNLPPSSGLPVQMGNPRLPQGAPQQFPYPPNYGTNPGTPPASTSPFSQLAANPEASLANRNSMVSRGMT 1210
NCOA1-206    QPPLNAQMLAQRQRELYSQQHRQRQLIQQQRAMLMRQQSFGNNLPPSSGLPVQMGNPRLPQGAPQQFPYPPNYGTNPGTPPASTSPFSQLAANPEASLANRNSMVSRGMT 1059
NCOA1-205    QPPLNAQMLAQRQRELYSQQHRQRQLIQQQRAMLMRQQSFGNNLPPSSGLPVQMGNPRLPQGAPQQFPYPPNYGTNPGTPPASTSPFSQLAANPEASLANRNSMVSRGMT 1210
NCOA1-203    QPPLNAQMLAQRQRELYSQQHRQRQLIQQQRAMLMRQQSFGNNLPPSSGLPVQMGNPRLPQGAPQQFPYPPNYGTNPGTPPASTSPFSQLAANPEASLANRNSMVSRGMT 1210

logo
             GNIGGQFGTGINPQMQQNVFQYPGAGMVPQGEANFAPSLSPGSSMVPMPIPPPQSSLLQQTPPASGYQSPDMKAWQQGAIGNNNVFSQAVQNQPTAQPGVYNNMSITVS
GENISTEIN    ..............................................................................................................    8
PF14598      ..............................................................................................................  110
PF00989      ..............................................................................................................   58
PF08832      ..............................................................................................................   79
PF00010      ..............................................................................................................   51
PF08815      ..............................................................................................................   46
PF16665      ..............................................................................................................  123
PF07469      .NIGGQFGTGINPQMQQNVFQYPGAGMVPQGEANFAPSLSPGSSMVPMPIPPPQSSL.....................................................  112
NCOA1-204    GNIGGQFGTGINPQMQQNVFQYPGAGMVPQGEANFAPSLSPGSSMVPMPIPPPQSSLLQQTPPASGYQSPDMKAWQQGAIGNNNVFSQAVQNQPTAQPGVYNNMSITVS 1320
NCOA1-202    GNIGGQFGTGINPQMQQNVFQYPGAGMVPQGEANFAPSLSPGSSMVPMPIPPPQSSLLQQTPPASGYQSPDMKAWQQGAIGNNNVFSQAVQNQPTAQPGVYNNMSITVS 1320
NCOA1-201    GNIGGQFGTGINPQMQQNVFQYPGAGMVPQGEANFAPSLSPGSSMVPMPIPPPQSSLLQQTPPASGYQSPDMKAWQQGAIGNNNVFSQAVQNQPTAQPGVYNNMSITVS 1320
NCOA1-206    GNIGGQFGTGINPQMQQNVFQYPGAGMVPQGEANFAPSLSPGSSMVPMPIPPPQSSLLQQTPPASGYQSPDMKAWQQGAIGNNNVFSQAVQNQPTAQPGVYNNMSITVS 1169
NCOA1-205    GNIGGQFGTGINPQMQQNVFQYPGAGMVPQGEANFAPSLSPGSSMVPMPIPPPQSSLLQQTPPASGYQSPDMKAWQQGAIGNNNVFSQAVQNQPTAQPGVYNNMSITVS 1320
NCOA1-203    GNIGGQFGTGINPQMQQNVFQYPGAGMVPQGEANFAPSLSPGSSMVPMPIPPPQSSLLQQTPPASGYQSPDMKAWQQGAIGNNNVFSQAVQNQPTAQPGVYNNMSITVS 1320
```

logo

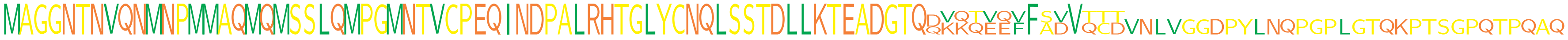

```
GENISTEIN    .............................................................................................................. 8
PF14598      .............................................................................................................. 110
PF00989      .............................................................................................................. 58
PF08832      .............................................................................................................. 79
PF00010      .............................................................................................................. 51
PF08815      .............................................................................................................. 46
PF16665      .............................................................................................................. 123
PF07469      .............................................................................................................. 112
NCOA1-204    MAGGNTNVQNMNPMMAQMQMSSLQMPGMNTVCPEQINDPALRHTGLYCNQLSSTDLLKTEADGTQDKKTEEFFSVVTTD............................... 1399
NCOA1-202    MAGGNTNVQNMNPMMAQMQMSSLQMPGMNTVCPEQINDPALRHTGLYCNQLSSTDLLKTEADGTQQVQQVQVFADVQCTVNLVGGDPYLNQPGPLGTQKPTSGPQTPQAQ 1430
NCOA1-201    MAGGNTNVQNMNPMMAQMQMSSLQMPGMNTVCPEQINDPALRHTGLYCNQLSSTDLLKTEADGTQDKKTEEFFSVVTTD............................... 1399
NCOA1-206    MAGGNTNVQNMNPMMAQMQMSSLQMPGMNTVCPEQINDPALRHTGLYCNQLSSTDLLKTEADGTQDKKTEEFFSVVTTD............................... 1248
NCOA1-205    MAGGNTNVQNMNPMMAQMQMSSLQMPGMNTVCPEQINDPALRHTGLYCNQLSSTDLLKTEADGTQQVQQVQVFADVQCTVNLVGGDPYLNQPGPLGTQKPTSGPQTPQAQ 1430
NCOA1-203    MAGGNTNVQNMNPMMAQMQMSSLQMPGMNTVCPEQINDPALRHTGLYCNQLSSTDLLKTEADGTQ.VQQVQVFADVQCTVNLVGGDPYLNQPGPLGTQKPTSGPQTPQAQ 1429
```

logo

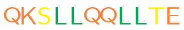

```
GENISTEIN    ...........     8
PF14598      ...........   110
PF00989      ...........    58
PF08832      ...........    79
PF00010      ...........    51
PF08815      ...........    46
PF16665      ...........   123
PF07469      ...........   112
NCOA1-204    ...........  1399
NCOA1-202    QKSLLQQLLTE  1441
NCOA1-201    ...........  1399
NCOA1-206    ...........  1248
NCOA1-205    QKSLLQQLLTE  1441
NCOA1-203    QKSLLQQLLTE  1440
```

- X non conserved
- X similar
- X $\geq$ 0% conserved
- X $\geq$ 50% conserved

```
logo         MAGKKVLIVYAHQEPKSFNGSLKNVAVDELSRQGCTVTVSDLYAMNLEPRATDKDITGTLSNPEVFNYGVETHEAYKQRSLASDITDEQKKVREADLVIFQFPLYWFSVP
RESVERATROL  ......................................................................G.......................................WF...    3
PF02525.3    ...KKVLIVYAHQEPKSFNGSLKNVAVDELSRQGCTVTVSDLYAMNLEPRATDKDITG.................................................................   55
PF02525      ...KKVLIVYAHQEPKSFNGSLKNVAVDELSRQGCTVTVSDLYAMNLEPRATDKDITGTLSNPEVFNYGVETHEAYKQRSLASDITDEQKKVREADLVIFQFPLYWFSVP  107
PF02525.1    ...KKVLIVYAHQEPKSFNGSLKNVAVDELSRQGCTVTVSDLYAMNLEPRATDKDITGTLSNPEVFNYGVETHEAYKQRSLASDITDEQKKVREADLVIFQ.........   98
PF02525.2    ...KKVLIVYAHQEPKSFNGSLKNVAVDELSRQGCTVTVSDLYAMNLEPRATDKDITGTLSNPEVFNYGVETHEAYKQRSLASDITDEQKKVREADLVIFQFPLYWFSVP  107
NQ02-206     MAGKKVLIVYAHQEPKSFNGSLKNVAVDELSRQGCTVTVSDLYAMNLEPRATDKDITGTLSNPEVFNYGVETHEAYKQRSLASDITDEQKKVREADLVIFQFPLYWFSVP  110
NQ02-207     MAGKKVLIVYAHQEPKSFNGSLKNVAVDELSRQGCTVTVSDLYAMNLEPRATDKDITGTLSNPEVFNYGVETHEAYKQRSLASDITDEQKKVREADLVIFQFPLYWFSVP  110
NQ02-202     MAGKKVLIVYAHQEPKSFNGSLKNVAVDELSRQGCTVTVSDLYAMNLEPRATDKDITGTLSNPEVFNYGVETHEAYKQRSLASDITDEQKKVREADLVIFQFPLYWFSVP  110
NQ02-204     MAGKKVLIVYAHQEPKSFNGSLKNVAVDELSRQGCTVTVSDLYAMNLEPRATDKDITGTLSNPEVFNYGVETHEAYKQRSLASDITDEQKKVREADLVIF..........  100
NQ02-201     MAGKKVLIVYAHQEPKSFNGSLKNVAVDELSRQGCTVTVSDLYAMNLEPRATDKDITGTLSNPEVFNYGVETHEAYKQRSLASDITDEQKKVREADLVIFQFPLYWFSVP  110
NQ02-205     MAGKKVLIVYAHQEPKSFNGSLKNVAVDELSRQGCTVTVSDLYAMNLEPRATDKDITGTLSNPEVFNYGVETHEAYKQRSLASDITDEQKKVREADLVIFQFPLYWFSVP  110
NQ02-203     MAGKKVLIVYAHQEPKSFNGSLKNVAVDELSRQGCTVTVSDLYAMNLEPRATDKDITGTLSNPEVFNYGVETHEAYKQRSLASDITDEQKKVREADLVIF..........  100
NQ02-209     ...........................................................................................................    0
NQ02-208     MAGKKVLIVYAHQEPKSFNGSLKNVAVDELSRQGCTVTVSDLYAMNLEPRATDKDITGTLSNP............................................   63

logo         AILKGWMDRVLCQQGFAFDIPGFYDSGLLQGKLALLSVTTGGTAEMYTKTGVNGDSRYFLWPLQHGTLHFCGFKVLAPQISFAPEIASEEERKGMVAAWSQRLQTIWKEEP
RESVERATROL  ..........L.....F.....Y.................G.........N............G...F..........................................   10
PF02525.3    ..............................................................................................................   55
PF02525      AILKGWMDRVLCQQGFAFDIPGFYDSGLLQGKLALLSVTTGGTAEMYTKTGVNGDSRYFLWPLQHGTLHFCGFKVLAPQISFAPEIASEEERKGMVAAWSQRL........  209
PF02525.1    ..............................................................................................................   98
PF02525.2    AILKGWMDRVLCQQGFAFD...........................................................................................  125
NQ02-206     AILKGWMDRVLCQQGFAFDIPGFYDSGLLQ................................................................................  139
NQ02-207     AILKGWMDRVLCQQGFAFDIPGFYDSGLLQ................................................................................  139
NQ02-202     AILKGWMDRVLCQQGFAFDIPGFYDSGLLQGKLALLSVTTGGTAEMYTKTGVNGDSRYFLWPLQHGTLHFCGFKVLAPQISFAPEIASEEERKGMVAAWSQRLQTIWKEEP  220
NQ02-204     ............................QGKLALLSVTTGGTAEMYTKTGVNGDSRYFLWPLQHGTLHFCGFKVLAPQISFAPEIASEEERKGMVAAWSQRLQTIWKEEP  182
NQ02-201     AILKGWMDRVLCQQGFAFDIPGFYDSGLLQGKLALLSVTTGGTAEMYTKTGVNGDSRYFLWPLQHGTLHFCGFKVLAPQISFAPEIASEEERKGMVAAWSQRLQTIWKEEP  220
NQ02-205     AILKGWMDRVLCQQGFAFDIPGFYDSGLLQGKLALLSVTTGGTAEMYTKTGVNGDSRYFLWPLQHGTLHFCGFKVLAPQISFAPEIASEEERKGMVAAWSQRLQTIWKEEP  220
NQ02-203     ............................QGKLALLSVTTGGTAEMYTKTGVNGDSRYFLWPLQHGTLHFCGFKVLAPQISFAPEIASEEERKGMVAAWSQRLQTIWKEEP  182
NQ02-209     ...........MAEFR.....FLKSGA....LPILSLLCLQAL.KFSLGCSRGSSE......................................................   35
NQ02-208     ..............................................................................................................   63

logo         IPCTAHWHFGQ
RESVERATROL  ...........   10
PF02525.3    ...........   55
PF02525      ...........  209
PF02525.1    ...........   98
PF02525.2    ...........  125
NQ02-206     ...........  139
NQ02-207     ...........  139
NQ02-202     IPCTAHWHFGQ  231
NQ02-204     IPCTAHWHFGQ  193
NQ02-201     IPCTAHWHFGQ  231
NQ02-205     IPCTAHWHFGQ  231
NQ02-203     IPCTAHWHFGQ  193
NQ02-209     ...........   35
NQ02-208     ...........   63
```

X non conserved
X similar
X $\geq$ 0% conserved
X $\geq$ 50% conserved

```
logo
```
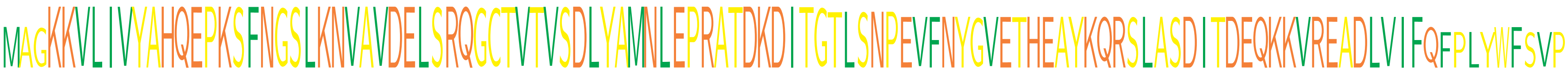

```
TRETAZICAR  .........................................................................................................WF...    2
PF02525.3   ...KKVLIVYAHQEPKSFNGSLKNVAVDELSRQGCTVTVSDLYAMNLEPRATDKDITG....................................................   55
PF02525     ...KKVLIVYAHQEPKSFNGSLKNVAVDELSRQGCTVTVSDLYAMNLEPRATDKDITGTLSNPEVFNYGVETHEAYKQRSLASDITDEQKKVREADLVIFQFPLYWFSVP  107
PF02525.1   ...KKVLIVYAHQEPKSFNGSLKNVAVDELSRQGCTVTVSDLYAMNLEPRATDKDITGTLSNPEVFNYGVETHEAYKQRSLASDITDEQKKVREADLVIFQ.........   98
PF02525.2   ...KKVLIVYAHQEPKSFNGSLKNVAVDELSRQGCTVTVSDLYAMNLEPRATDKDITGTLSNPEVFNYGVETHEAYKQRSLASDITDEQKKVREADLVIFQFPLYWFSVP  107
NQO2-206    MAGKKVLIVYAHQEPKSFNGSLKNVAVDELSRQGCTVTVSDLYAMNLEPRATDKDITGTLSNPEVFNYGVETHEAYKQRSLASDITDEQKKVREADLVIFQFPLYWFSVP  110
NQO2-207    MAGKKVLIVYAHQEPKSFNGSLKNVAVDELSRQGCTVTVSDLYAMNLEPRATDKDITGTLSNPEVFNYGVETHEAYKQRSLASDITDEQKKVREADLVIFQFPLYWFSVP  110
NQO2-202    MAGKKVLIVYAHQEPKSFNGSLKNVAVDELSRQGCTVTVSDLYAMNLEPRATDKDITGTLSNPEVFNYGVETHEAYKQRSLASDITDEQKKVREADLVIFQFPLYWFSVP  110
NQO2-204    MAGKKVLIVYAHQEPKSFNGSLKNVAVDELSRQGCTVTVSDLYAMNLEPRATDKDITGTLSNPEVFNYGVETHEAYKQRSLASDITDEQKKVREADLVIF..........  100
NQO2-201    MAGKKVLIVYAHQEPKSFNGSLKNVAVDELSRQGCTVTVSDLYAMNLEPRATDKDITGTLSNPEVFNYGVETHEAYKQRSLASDITDEQKKVREADLVIFQFPLYWFSVP  110
NQO2-205    MAGKKVLIVYAHQEPKSFNGSLKNVAVDELSRQGCTVTVSDLYAMNLEPRATDKDITGTLSNPEVFNYGVETHEAYKQRSLASDITDEQKKVREADLVIFQFPLYWFSVP  110
NQO2-203    MAGKKVLIVYAHQEPKSFNGSLKNVAVDELSRQGCTVTVSDLYAMNLEPRATDKDITGTLSNPEVFNYGVETHEAYKQRSLASDITDEQKKVREADLVIF..........  100
NQO2-209    ..............................................................................................................    0
NQO2-208    MAGKKVLIVYAHQEPKSFNGSLKNVAVDELSRQGCTVTVSDLYAMNLEPRATDKDITGTLSNP...............................................   63
```

```
logo
```
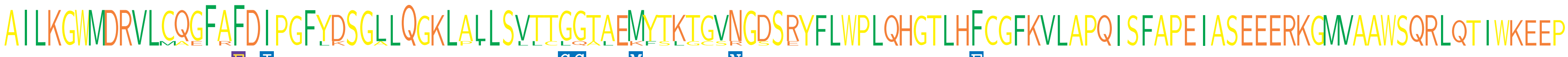

```
TRETAZICAR  ................F.I....................GG...M......N................F.........................................    9
PF02525.3   ..............................................................................................................   55
PF02525     AILKGWMDRVLCQGFAFDIPGFYDSGLLQGKLALLSVTTGGTAEMYTKTGVNGDSRYFLWPLQHGTLHFCGFKVLAPQISFAPEIASEEERKGMVAAWSQRL........  209
PF02525.1   ..............................................................................................................   98
PF02525.2   AILKGWMDRVLCQGFAFD............................................................................................  125
NQO2-206    AILKGWMDRVLCQGFAFDIPGFYDSGLLQ.................................................................................  139
NQO2-207    AILKGWMDRVLCQGFAFDIPGFYDSGLLQ.................................................................................  139
NQO2-202    AILKGWMDRVLCQGFAFDIPGFYDSGLLQGKLALLSVTTGGTAEMYTKTGVNGDSRYFLWPLQHGTLHFCGFKVLAPQISFAPEIASEEERKGMVAAWSQRLQTIWKEEP  220
NQO2-204    ............................QGKLALLSVTTGGTAEMYTKTGVNGDSRYFLWPLQHGTLHFCGFKVLAPQISFAPEIASEEERKGMVAAWSQRLQTIWKEEP  182
NQO2-201    AILKGWMDRVLCQGFAFDIPGFYDSGLLQGKLALLSVTTGGTAEMYTKTGVNGDSRYFLWPLQHGTLHFCGFKVLAPQISFAPEIASEEERKGMVAAWSQRLQTIWKEEP  220
NQO2-205    AILKGWMDRVLCQGFAFDIPGFYDSGLLQGKLALLSVTTGGTAEMYTKTGVNGDSRYFLWPLQHGTLHFCGFKVLAPQISFAPEIASEEERKGMVAAWSQRLQTIWKEEP  220
NQO2-203    ............................QGKLALLSVTTGGTAEMYTKTGVNGDSRYFLWPLQHGTLHFCGFKVLAPQISFAPEIASEEERKGMVAAWSQRLQTIWKEEP  182
NQO2-209    ...........MAEFR.....FLKSGA....LPILSLLCLQAL.KFSLGCSRGSSE.......................................................   35
NQO2-208    ..............................................................................................................   63
```

```
logo
```
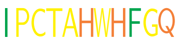

```
TRETAZICAR  ...........    9
PF02525.3   ...........   55
PF02525     ...........  209
PF02525.1   ...........   98
PF02525.2   ...........  125
NQO2-206    ...........  139
NQO2-207    ...........  139
NQO2-202    IPCTAHWHFGQ  231
NQO2-204    IPCTAHWHFGQ  193
NQO2-201    IPCTAHWHFGQ  231
NQO2-205    IPCTAHWHFGQ  231
NQO2-203    IPCTAHWHFGQ  193
NQO2-209    ...........   35
NQO2-208    ...........   63
```

X non conserved
X similar
X $\geq$ 0% conserved
X $\geq$ 50% conserved

```
logo           MDSKESLTPGREENPSSVLAQERGDVMDFYKTLRGGATVKVSASSPSLAVASQSDSKQRRLLVDFPKGSVSNAQQPDLSKAVSLSMGLYMGETETKVMGNDLGFPQQGQI
DEXAMETHASONE  ..............................................................................................................    0
PF02155        .........................VMDFYKTLRGGATVKVSASSPSLAVASQSDSKQRRLLVDFPKGSVSNAQQPDLSKAVSLSMGLYMGETETKVMGNDLGFPQQGQI   85
PF02155.2      .........................VMDFYKTLRGGATVKVSASSPSLAVASQSDSKQRRLLVDFPKGSVSNAQQPDLSKAVSLSMGLYMGETETKVMGNDLGFPQQGQI   85
PF00105.1      ..............................................................................................................    0
PF00105        ..............................................................................................................    0
PF00104.1      ..............................................................................................................    0
PF02155.3      .........................VMDFYKTLRGGATVKVSASSPSLAVASQSDSKQRRLLVDFPKGSVSNAQQPDLSKAVSLSMGLYMGETETKVMGNDLGFPQQGQI   85
PF02155.1      .........................VMDFYKTLRGGATVKVSASSPSLAVASQSDSKQRRLLVDFPKGSVSNAQQPDLSKAVSLSMGLYMGETETKVMGNDLGFPQQGQI   85
PF00104        ..............................................................................................................    0
NR3C1-212      MDSKESLTPGREENPSSVLAQERGDVMDFYKTLRGGATVKVSASSPSLAVASQSDSKQRRLLVDFPKGSVSNAQQPDLSKAVSLSMGLYMGETETKVMGNDLGFPQQGQI  110
NR3C1-207      MDSKESLTPGREENPSSVLAQERGDVMDFYKTLRGGATVKVSASSPSLAVASQSDSKQRRLLVDFPKGSVSNAQQPDLSKAVSLSMGLYMGETETKVMGNDLGFPQQGQI  110
NR3C1-201      MDSKESLTPGREENPSSVLAQERGDVMDFYKTLRGGATVKVSASSPSLAVASQSDSKQRRLLVDFPKGSVSNAQQPDLSKAVSLSMGLYMGETETKVMGNDLGFPQQGQI  110
NR3C1-208      MDSKESLTPGREENPSSVLAQERGDVMDFYKTLRGGATVKVSASSPSLAVASQSDSKQRRLLVDFPKGSVSNAQQPDLSKAVSLSMGLYMGETETKVMGNDLGFPQQGQI  110
NR3C1-205      MDSKESLTPGREENPSSVLAQERGDVMDFYKTLRGGATVKVSASSPSLAVASQSDSKQRRLLVDFPKGSVSNAQQPDLSKAVSLSMGLYMGETETKVMGNDLGFPQQGQI  110
NR3C1-215      MDSKESLTPGREENPSSVLAQERGDVMDFYKTLRGGATVKVSASSPSLAVASQSDSKQRRLLVDFPKGSVSNAQQPDLSKAVSLSMGLYMGETETKVMGNDLGFPQQGQI  110
NR3C1-214      MDSKESLTPGREENPSSVLAQERGDVMDFYKTLRGGATVKVSASSPSLAVASQSDSKQRRLLVDFPKGSVSNAQQPDLSKAVSLSMGLYMGETETKVMGNDLGFPQQGQI  110
NR3C1-206      MDSKESLTPGREENPSSVLAQERGDVMDFYKTLRGGATVKVSASSPSLAVASQSDSKQRRLLVDFPKGSVSNAQQPDLSKAVSLSMGLYMGETETKVMGNDLGFPQQGQI  110
NR3C1-203      MDSKESLTPGREENPSSVLAQERGDVMDFYKTLRGGATVKVSASSPSLAVASQSDSKQRRLLVDFPKGSVSNAQQPDLSKAVSLSMGLYMGETETKVMGNDLGFPQQGQI  110
NR3C1-209      MDSKESLTPGREENPSSVLAQERGDVMDFYKTLRGGATVKVSASSPSLAVASQSDSKQRRLLVDFPKGSVSNAQQPDLSKAVSLSMGLYMGETETKVMGNDLGFPQQGQI  110
NR3C1-216      MDSKESLTPGREENPSSVLAQERGDVMDFYKTLRGGATVKVSASSPSLAVASQSDSKQRRLLVDFPKGSVSNAQQPDLSKAVSLSMGLYMGETETKVMGNDLGFPQQGQI  110
NR3C1-202      MDSKESLTPGREENPSSVLAQERGDVMDFYKTLRGGATVKVSASSPSLAVASQSDSKQRRLLVDFPKGSVSNAQQPDLSKAVSLSMGLYMGETETKVMGNDLGFPQQGQI  110
NR3C1-204      MDSKESLTPGREENPSSVLAQERGDVMDFYKTLRGGATVKVSASSPSLAVASQSDSKQRRLLVDFPKGSVSNAQQPDLSKAVSLSMGLYMGETETKVMGNDLGFPQQGQI  110

logo           SLSSGETDLKLLEESIANLNRSTSVPENPKSSASTAVSAAPTEKEFPKTHSDVSSEQQHLKGQTGTNGGNVKLYTTDQSTFDILQDLEFSSGSPGKETNESPWRSDLLID
DEXAMETHASONE  ..............................................................................................................    0
PF02155        SLSSGETDLKLLEESIANLNRSTSVPENPKSSASTAVSAAPTEKEFPKTHSDVSSEQQHLKGQTGTNGGNVKLYTTDQSTFDILQDLEFSSGSPGKETNESPWRSDLLID  195
PF02155.2      SLSSGETDLKLLEESIANLNRSTSVPENPKSSAS............................................................................  119
PF00105.1      ..............................................................................................................    0
PF00105        ..............................................................................................................    0
PF00104.1      ..............................................................................................................    0
PF02155.3      SLSSGETDLKLLEESIANLNRSTSVPENPKSSA.............................................................................  118
PF02155.1      SLSSGETDLKLLEESIANLNRSTSVPENPKSSASTAVSAAPTEKEFPKTHSDVSSEQQHLKGQTGTNGGNVKLYTTDQSTFDILQDLEFSSGSPGKETNESPWRSDLLID  195
PF00104        ..............................................................................................................    0
NR3C1-212      SLSSGETDLKLLEESIANLNRSTSVPENPKSSASTAVSAAPTEKEFPKTHSDVSSEQQHLKGQTGTNGGNVKLYTTDQSTFDILQDLEFSSGSPGKETNESPWRSDLLID  220
NR3C1-207      SLSSGETDLKLLEESIANLNRSTSVPENPKSSAS............................................................................  144
NR3C1-201      SLSSGETDLKLLEESIANLNRSTSVPENPKSSASTAVSAAPTEKEFPKTHSDVSSEQQHLKGQTGTNGGNVKLYTTDQSTFDILQDLEFSSGSPGKETNESPWRSDLLID  220
NR3C1-208      SLSSGETDLKLLEESIANLNRSTSVPENPKSSAST...........................................................................  145
NR3C1-205      SLSSGETDLKLLEESIANLNRSTSVPENPKSSASTAVSAAPTEKEFPKTHSDVSSEQQHLKGQTGTNGGNVKLYTTDQSTFDILQDLEFSSGSPGKETNESPWRSDLLID  220
NR3C1-215      SLSSGETDLKLLEESIANLNRSTSVPENPKSSAST...........................................................................  145
NR3C1-214      SLSSGETDLKLLEESIANLNRSTSVPENPKSSAST...........................................................................  145
NR3C1-206      SLSSGETDLKLLEESIANLNRSTSVPENPKSSASTAVSAAPTEKEFPKTHSDVSSEQQHLKGQTGTNGGNVKLYTTDQSTFDILQDLEFSSGSPGKETNESPWRSDLLID  220
NR3C1-203      SLSSGETDLKLLEESIANLNRSTSVPENPKSSASTAVSAAPTEKEFPKTHSDVSSEQQHLKGQTGTNGGNVKLYTTDQSTFDILQDLEFSSGSPGKETNESPWRSDLLID  220
NR3C1-209      SLSSGETDLKLLEESIANLNRSTSVPENPKSSASTAVSAAPTEKEFPKTHSDVSSEQQHLKGQTGTNGGNVKLYTTDQSTFDILQDLEFSSGSPGKETNESPWRSDLLID  220
NR3C1-216      SLSSGETDLKLLEESIANLNRSTSVPENPKSSAST...........................................................................  145
NR3C1-202      SLSSGETDLKLLEESIANLNRSTSVPENPKSSASTAVSAAPTEKEFPKTHSDVSSEQQHLKGQTGTNGGNVKLYTTDQSTFDILQDLEFSSGSPGKETNESPWRSDLLID  220
NR3C1-204      SLSSGETDLKLLEESIANLNRSTSVPENPKSSASTAVSAAPTEKEFPKTHSDVSSEQQHLKGQTGTNGGNVKLYTTDQSTFDILQDLEFSSGSPGKETNESPWRSDLLID  220
```

```
logo           ENCLLSPLAGEDDSFLLEGNSNEDCKPLILPDTKPKIKDNGDLVLSSPSNVTLPQVKTEKEDFIELCTPGVIKQEKLGTVYCQASFPGANIIGNKMSAISVHGVSTSGGQ
DEXAMETHASONE  ..............................................................................................................    0
PF02155        ENCLLSPLAGEDDSFLLEGNSNEDCKPLILPDTKPKIKDNGDLVLSSPSNVTLPQVKTEKEDFIELCTPGVIKQEKLGTVYCQASFPGANIIGNKMSAISVHGVSTSGGQ  305
PF02155.2      ..............................................................................................................  119
PF00105.1      ..............................................................................................................    0
PF00105        ..............................................................................................................    0
PF00104.1      ..............................................................................................................    0
PF02155.3      ..............................................................................................................  118
PF02155.1      ENCLLSPLAGEDDSFLLEGNSNEDCKPLILPDTKPKIKDNGDLVLSSPSNVTLPQVKTEKEDFIELCTPGVIKQEKLGTVYCQASFPGANIIA.................  288
PF00104        ..............................................................................................................    0
NR3C1-212      ENCLLSPLAGEDDSFLLEGNSNEDCKPLILPDTKPKIKDNGDLVLSSPSNVTLPQVKTEKEDFIELCTPGVIKQEKLGTVYCQASFPGANIIGNKMSAISVHGVSTSGGQ  330
NR3C1-207      ..............................................................................................................  144
NR3C1-201      ENCLLSPLAGEDDSFLLEGNSNEDCKPLILPDTKPKIKDNGDLVLSSPSNVTLPQVKTEKEDFIELCTPGVIKQEKLGTVYCQASFPGANIIGNKMSAISVHGVSTSGGQ  330
NR3C1-208      ..............................................................................................................  145
NR3C1-205      ENCLLSPLAGEDDSFLLEGNSNEDCKPLILPDTKPKIKDNGDLVLSSPSNVTLPQVKTEKEDFIELCTPGVIKQEKLGTVYCQASFPGANIIGNKMSAISVHGVSTSGGQ  330
NR3C1-215      ..............................................................................................................  145
NR3C1-214      ..............................................................................................................  145
NR3C1-206      ENCLLSPLAGEDDSFLLEGNSNEDCKPLILPDTKPKIKDNGDLVLSSPSNVTLPQVKTEKEDFIELCTPGVIKQEKLGTVYCQASFPGANIIA.................  313
NR3C1-203      ENCLLSPLAGEDDSFLLEGNSNEDCKPLILPDTKPKIKDNGDLVLSSPSNVTLPQVKTEKEDFIELCTPGVIKQEKLGTVYCQASFPGANIIGNKMSAISVHGVSTSGGQ  330
NR3C1-209      ENCLLSPLAGEDDSFLLEGNSNEDCKPLILPDTKPKIKDNGDLVLSSPSNVTLPQVKTEKEDFIELCTPGVIKQEKLGTVYCQASFPGANIIGNKMSAISVHGVSTSGGQ  330
NR3C1-216      ..............................................................................................................  145
NR3C1-202      ENCLLSPLAGEDDSFLLEGNSNEDCKPLILPDTKPKIKDNGDLVLSSPSNVTLPQVKTEKEDFIELCTPGVIKQEKLGTVYCQASFPGANIIGNKMSAISVHGVSTSGGQ  330
NR3C1-204      ENCLLSPLAGEDDSFLLEGNSNEDCKPLILPDTKPKIKDNGDLVLSSPSNVTLPQVKTEKEDFIELCTPGVIKQEKLGTVYCQASFPGANIIGNKMSAISVHGVSTSGGQ  330
```

```
logo           MYHYDMNTASLSQQQDQKPIFNVIPPIPVGSENWNRCQGSGDDNLTSLGTLNFPGRTVFSNGYSSPSMRPDVSSPPSSSSTATTGPPPKLCLVCSDEASGCHYGVLTCGS
DEXAMETHASONE  ..............................................................................................................    0
PF02155        MYHYDMNTASLSQQQDQKPIFNVIPPIPVGSENWNRCQGSGDDNLTSLGTLNFPGRTVFSNGYSSPSMRPD.......................................  376
PF02155.2      ..............................................................................................................  119
PF00105.1      .........................................................................................LCLVCSDEASGCHYGVLTCGS   21
PF00105        .........................................................................................LCLVCSDEASGCHYGVLTCGS   21
PF00104.1      ..............................................................................................................    0
PF02155.3      ..............................................................................................................  118
PF02155.1      .........SLSQQQDQKPIFNVIPPIPVGSENWNRCQGSGDDNLTSLGTLNFPGRTVFSNGYSSPSMRPD.......................................  350
PF00104        ..............................................................................................................    0
NR3C1-212      MYHYDMNTASLSQQQDQKPIFNVIPPIPVGSENWNRCQGSGDDNLTSLGTLNFPGRTVFSNGYSSPSMRPDVSSPPSSSSTATTGPPPKLCLVCSDEASGCHYGVLTCGS  440
NR3C1-207      ..............................................................................................................  144
NR3C1-201      MYHYDMNTASLSQQQDQKPIFNVIPPIPVGSENWNRCQGSGDDNLTSLGTLNFPGRTVFSNGYSSPSMRPDVSSPPSSSSTATTGPPPKLCLVCSDEASGCHYGVLTCGS  440
NR3C1-208      ..............................................................................................................  145
NR3C1-205      MYHYDMNTASLSQQQDQKPIFNVIPPIPVGSENWNRCQGSGDDNLTSLGTLNFPGRTVFSNGYSSPSMRPDVSSPPSSSSTATTGPPPKLCLVCSDEASGCHYGVLTCGS  440
NR3C1-215      ..............................................................................................................  145
NR3C1-214      ..............................................................................................................  145
NR3C1-206      .........SLSQQQDQKPIFNVIPPIPVGSENWNRCQGSGDDNLTSLGTLNFPGRTVFSNGYSSPSMRPDVSSPPSSSSTATTGPPPKLCLVCSDEASGCHYGVLTCGS  414
NR3C1-203      MYHYDMNTASLSQQQDQKPIFNVIPPIPVGSENWNRCQGSGDDNLTSLGTLNFPGRTVFSNGYSSPSMRPDVSSPPSSSSTATTGPPPKLCLVCSDEASGCHYGVLTCGS  440
NR3C1-209      MYHYDMNTASLSQQQDQKPIFNVIPPIPVGSENWNRCQGSGDDNLTSLGTLNFPGRTVFSNGYSSPSMRPDVSSPPSSSSTATTGPPPKLCLVCSDEASGCHYGVLTCGS  440
NR3C1-216      ..............................................................................................................  145
NR3C1-202      MYHYDMNTASLSQQQDQKPIFNVIPPIPVGSENWNRCQGSGDDNLTSLGTLNFPGRTVFSNGYSSPSMRPDVSSPPSSSSTATTGPPPKLCLVCSDEASGCHYGVLTCGS  440
NR3C1-204      MYHYDMNTASLSQQQDQKPIFNVIPPIPVGSENWNRCQGSGDDNLTSLGTLNFPGRTVFSNGYSSPSMRPDVSSPPSSSSTATTGPPPKLCLVCSDEASGCHYGVLTCGS  440
```

```
logo           CKVFFKRAVEGRQHNYLCAGRNDCIIDKIRRKNCPACRYRKCLQAGMNLEARKTKKKIKGIQQATTGVSQETSENPGNKTIVPATLPQLTPTLVSLLEVIEPEVLYAGYD
DEXAMETHASONE  ..............................................................................................................   0
PF02155        ..............................................................................................................   376
PF02155.2      ..............................................................................................................   119
PF00105.1      CKVFFKRAVEG.QHNYLCAGRNDCIIDKIRRKNCPACRYRKCLQAGMN..............................................................   68
PF00105        CKVFFKRAVEGRQHNYLCAGRNDCIIDKIRRKNCPACRYRKCLQAGMN..............................................................   69
PF00104.1      ..............................................................................................................   0
PF02155.3      ..............................................................................................................   118
PF02155.1      ..............................................................................................................   350
PF00104        ..............................................................................................................   0
NR3C1-212      CKVFFKRAVEGRQHNYLCAGRNDCIIDKIRRKNCPACRYRKCLQAGMNLEARKTKKKIKGIQQATTGVSQETSENPGNKTIVPATLPQLTPTLVSLLEVIEPEVLYAGYD   550
NR3C1-207      ..............................................................................................................   144
NR3C1-201      CKVFFKRAVEGRQHNYLCAGRNDCIIDKIRRKNCPACRYRKCLQAGMNLEARKTKKKIKGIQQATTGVSQETSENPGNKTIVPATLPQLTPTLVSLLEVIEPEVLYAGYD   550
NR3C1-208      ..............................................................................................................   145
NR3C1-205      CKVFFKRAVEG.QHNYLCAGRNDCIIDKIRRKNCPACRYRKCLQAGMNLEARKTKKKIKGIQQATTGVSQETSENPGNKTIVPATLPQLTPTLVSLLEVIEPEVLYAGYD   549
NR3C1-215      ..............................................................................................................   145
NR3C1-214      ..............................................................................................................   145
NR3C1-206      CKVFFKRAVEG.QHNYLCAGRNDCIIDKIRRKNCPACRYRKCLQAGMNLEARKTKKKIKGIQQATTGVSQETSENPGNKTIVPATLPQLTPTLVSLLEVIEPEVLYAGYD   523
NR3C1-203      CKVFFKRAVEG.QHNYLCAGRNDCIIDKIRRKNCPACRYRKCLQAGMNLEARKTKKKIKGIQQATTGVSQETSENPGNKTIVPATLPQLTPTLVSLLEVIEPEVLYAGYD   549
NR3C1-209      CKVFFKRAVEG.QHNYLCAGRNDCIIDKIRRKNCPACRYRKCLQAGMNLEARKTKKKIKGIQQATTGVSQETSENPGNKTIVPATLPQLTPTLVSLLEVIEPEVLYAGYD   549
NR3C1-216      ..............................................................................................................   145
NR3C1-202      CKVFFKRAVEG.QHNYLCAGRNDCIIDKIRRKNCPACRYRKCLQAGMNLEARKTKKKIKGIQQATTGVSQETSENPGNKTIVPATLPQLTPTLVSLLEVIEPEVLYAGYD   549
NR3C1-204      CKVFFKRAVEGRQHNYLCAGRNDCIIDKIRRKNCPACRYRKCLQAGMNLEARKTKKKIKGIQQATTGVSQETSENPGNKTIVPATLPQLTPTLVSLLEVIEPEVLYAGYD   550

logo           SSVPDSTWRIMTTLNMLGGRQVIAAVKWAKAIPGFRNLHLDDQMTLLQYSWMFLMAFALGWRSYRQSSANLLCFAPDLIINEQRMTLPCMYDQCKHMLYVSSELHRLQVS
DEXAMETHASONE  ..........M..LN.LG..Q.............................WM..M...L..R...........F..................Q...M.............   14
PF02155        ..............................................................................................................   376
PF02155.2      ..............................................................................................................   119
PF00105.1      ..............................................................................................................   68
PF00105        ..............................................................................................................   69
PF00104.1      ...PDSTWRIMTTLNMLGGRQVIAAVKWAKAIPGFRNLHLDDQMTLLQYSWMFLMAFALGWRSYRQSSANLLCFAPDLIINEQRMTLPCMYDQCKHMLYVSSELHRLQVS   107
PF02155.3      ..............................................................................................................   118
PF02155.1      ..............................................................................................................   350
PF00104        ...PDSTWRIMTTLNMLGGRQVIAAVKWAKAIPGFRNLHLDDQMTLLQYSWMFLMAFALGWRSYRQSSANLLCFAPDLIINEQRMTLPCMYDQCKHMLYVSSELHRLQVS   107
NR3C1-212      SSVPDSTWRIMTTLNMLGGRQVIAAVKWAKAIPGFRNLHLDDQMTLLQYSWMFLMAFALGWRSYRQSSANLLCFAPDLIINEQRMTLPCMYDQCKHMLYVSSELHRLQVS   660
NR3C1-207      ..............................................................................................................   144
NR3C1-201      SSVPDSTWRIMTTLNMLGGRQVIAAVKWAKAIPGFRNLHLDDQMTLLQYSWMFLMAFALGWRSYRQSSANLLCFAPDLIINEQRMTLPCMYDQCKHMLYVSSELHRLQVS   660
NR3C1-208      ..............................................................................................................   145
NR3C1-205      SSVPDSTWRIMTTLNMLGGRQVIAAVKWAKAIPGFRNLHLDDQMTLLQYSWMFLMAFALGWRSYRQSSANLLCFAPDLIINEQRMTLPCMYDQCKHMLYVSSELHRLQVS   659
NR3C1-215      ..............................................................................................................   145
NR3C1-214      ..............................................................................................................   145
NR3C1-206      SSVPDSTWRIMTTLNMLGGRQVIAAVKWAKAIPGFRNLHLDDQMTLLQYSWMFLMAFALGWRSYRQSSANLLCFAPDLIINEQRMTLPCMYDQCKHMLYVSSELHRLQVS   633
NR3C1-203      SSVPDSTWRIMTTLNMLGGRQVIAAVKWAKAIPGFRNLHLDDQMTLLQYSWMFLMAFALGWRSYRQSSANLLCFAPDLIINEQRMTLPCMYDQCKHMLYVSSELHRLQVS   659
NR3C1-209      SSVPDSTWRIMTTLNMLGGRQVIAAVKWAKAIPGFRNLHLDDQMTLLQYSWMFLMAFALGWRSYRQSSANLLCFAPDLIINEQRMTLPCMYDQCKHMLYVSSELHRLQVS   659
NR3C1-216      ..............................................................................................................   145
NR3C1-202      SSVPDSTWRIMTTLNMLGGRQVIAAVKWAKAIPGFRNLHLDDQMTLLQYSWMFLMAFALGWRSYRQSSANLLCFAPDLIINEQRMTLPCMYDQCKHMLYVSSELHRLQVS   659
NR3C1-204      SSVPDSTWRIMTTLNMLGGRQVIAAVKWAKAIPGFRNLHLDDQMTLLQYSWMFLMAFALGWRSYRQSSANLLCFAPDLIINEQRMTLPCMYDQCKHMLYVSSELHRLQVS   660
```

```
logo
                YEEYLCMKTLLLLSSVPKDGLKSQELFDEIRMTYIKELGKAIVKREGNSSQNWQRFYQLTKLLDSMHEVVENLLNYCFQTFLDKTMSIEFPEMLAEIITNQIPKYSNGNI
DEXAMETHASONE   ...........................................................................YC..T.......I.F....................   19
PF02155         ..............................................................................................................  376
PF02155.2       ..............................................................................................................  119
PF00105.1       ..............................................................................................................   68
PF00105         ..............................................................................................................   69
PF00104.1       YEEYLCMKTLLLLSSVPKDGLKSQELFDEIRMTYIKELGKAIVKREGNSSQNWQRFYQLTKLLDSMHENV........................................  177
PF02155.3       ..............................................................................................................  118
PF02155.1       ..............................................................................................................  350
PF00104         YEEYLCMKTLLLLSSVPKDGLKSQELFDEIRMTYIKELGKAIVKREGNSSQNWQRFYQLTKLLDSMHEVVENLLNYC.................................  184
NR3C1-212       YEEYLCMKTLLLLSSVPKDGLKSQELFDEIRMTYIKELGKAIVKREGNSSQNWQRFYQLTKLLDSMHEVVENLLNYCFQTFLDKTMSIEFPEMLAEIITNQIPKYSNGNI  770
NR3C1-207       ..............................................................................................................  144
NR3C1-201       YEEYLCMKTLLLLSSVPKDGLKSQELFDEIRMTYIKELGKAIVKREGNSSQNWQRFYQLTKLLDSMHEVVENLLNYCFQTFLDKTMSIEFPEMLAEIITNQIPKYSNGNI  770
NR3C1-208       ..............................................................................................................  145
NR3C1-205       YEEYLCMKTLLLLSSVPKDGLKSQELFDEIRMTYIKELGKAIVKREGNSSQNWQRFYQLTKLLDSMHENVMWLKPESTSHTLI...........................  742
NR3C1-215       ..............................................................................................................  145
NR3C1-214       ..............................................................................................................  145
NR3C1-206       YEEYLCMKTLLLLSSVPKDGLKSQELFDEIRMTYIKELGKAIVKREGNSSQNWQRFYQLTKLLDSMHEVVENLLNYCFQTFLDKTMSIEFPEMLAEIITNQIPKYSNGNI  743
NR3C1-203       YEEYLCMKTLLLLSSVPKDGLKSQELFDEIRMTYIKELGKAIVKREGNSSQNWQRFYQLTKLLDSMHEVVENLLNYCFQTFLDKTMSIEFPEMLAEIITNQIPKYSNGNI  769
NR3C1-209       YEEYLCMKTLLLLSSVPKDGLKSQELFDEIRMTYIKELGKAIVKREGNSSQNWQRFYQLTKLLDSMHEVVENLLNYCFQTFLDKTMSIEFPEMLAEIITNQIPKYSNGNI  769
NR3C1-216       ..............................................................................................................  145
NR3C1-202       YEEYLCMKTLLLLSSVPKDGLKSQELFDEIRMTYIKELGKAIVKREGNSSQNWQRFYQLTKLLDSMHEVVENLLNYCFQTFLDKTMSIEFPEMLAEIITNQIPKYSNGNI  769
NR3C1-204       YEEYLCMKTLLLLSSVPKDGLKSQELFDEIRMTYIKELGKAIVKREGNSSQNWQRFYQLTKLLDSMHEVVENLLNYCFQTFLDKTMSIEFPEMLAEIITNQIPKYSNGNI  770

logo
                KKLLFHQK
DEXAMETHASONE   ........   19
PF02155         ........  376
PF02155.2       ........  119
PF00105.1       ........   68
PF00105         ........   69
PF00104.1       ........  177
PF02155.3       ........  118
PF02155.1       ........  350
PF00104         ........  184
NR3C1-212       KKLLFHQK  778
NR3C1-207       ........  144
NR3C1-201       KKLLFHQK  778
NR3C1-208       ........  145
NR3C1-205       ........  742
NR3C1-215       ........  145
NR3C1-214       ........  145
NR3C1-206       KKLLFHQK  751
NR3C1-203       KKLLFHQK  777
NR3C1-209       KKLLFHQK  777
NR3C1-216       ........  145
NR3C1-202       KKLLFHQK  777
NR3C1-204       KKLLFHQK  778
```

X non conserved
X similar
X $\geq 0\%$ conserved
X $\geq 50\%$ conserved

```
logo           METKGYHSLPEGLDMERRWGQVSQAVERSSLGPTERTDENNYMEIVNVSCVSGAIPNNSTQGSSKEKQELLPCLQQDNNRPGILTSDIKTELESKELSATVAESMGLYMD
DEXAMETHASONE  ..............................................................................................................    0
PF00105.1      ..............................................................................................................    0
PF00105        ..............................................................................................................    0
PF00104        ..............................................................................................................    0
PF00104.1      ..............................................................................................................    0
NR3C2-203      METKGYHSLPEGLDMERRWGQVSQAVERSSLGPTERTDENNYMEIVNVSCVSGAIPNNSTQGSSKEKQELLPCLQQDNNRPGILTSDIKTELESKELSATVAESMGLYMD  110
NR3C2-208      METKGYHSLPEGLDMERRWGQVSQAVERSSLGPTERTDENNYMEIVNVSCVSGAIPNNSTQGSSKEKQELLPCLQQDNNRPGILTSDIKTELESKELSATVAESMGLYMD  110
NR3C2-209      METKGYHSLPEGLDMERRWGQVSQAVERSSLGPTERTDENNYMEIVNVSCVSGAIPNNSTQGSSKEKQELLPCLQQDNNRPGILTSDIKTELESKELSATVAESMGLYMD  110
NR3C2-202      METKGYHSLPEGLDMERRWGQVSQAVERSSLGPTERTDENNYMEIVNVSCVSGAIPNNSTQGSSKEKQELLPCLQQDNNRPGILTSDIKTELESKELSATVAESMGLYMD  110
NR3C2-207      METKGYHSLPEGLDMERRWGQVSQAVERSSLGPTERTDENNYMEIVNVSCVSGAIPNNSTQGSSKEKQELLPCLQQDNNRPGILTSDIKTELESKELSATVAESMGLYMD  110
NR3C2-201      METKGYHSLPEGLDMERRWGQVSQAVERSSLGPTERTDENNYMEIVNVSCVSGAIPNNSTQGSSKEKQELLPCLQQDNNRPGILTSDIKTELESKELSATVAESMGLYMD  110

logo           SVRDADYSYEQQNQQGSMSPAKIYQNVEQLVKFYKGNGHRPSTLSCVNTPLRSFMSDSGSSVNGGVMRAVVKSPIMCHEKSPSVCSPLNMTSSVCSPAGINSVSSTTASF
DEXAMETHASONE  ..............................................................................................................    0
PF00105.1      ..............................................................................................................    0
PF00105        ..............................................................................................................    0
PF00104        ..............................................................................................................    0
PF00104.1      ..............................................................................................................    0
NR3C2-203      SVRDADYSYEQQNQQGSMSPAKIYQNVEQLVKFYKGNGHRPSTLSCVNTPLRSFMSDSGSSVNGGVMRAVVKSPIMCHEKSPSVCSPLNMTSSVCSPAGINSVSSTTASF  220
NR3C2-208      SVRDADYSYEQQNQQGSMSPAKIYQNVEQLVKFYKGNGHRPSTLSCVNTPLRSFMSDSGSSVNGGVMRAVVKSPIMCHEKSPSVCSPLNMTSSVCSPAGINSVSSTTASF  220
NR3C2-209      SVRDADYSYEQQNQQGSMSPAKIYQNVEQLVKFYKGNGHRPSTLSCVNTPLRSFMSDSGSSVNGGVMRAVVKSPIMCHEKSPSVCSPLNMTSSVCSPAGINSVSSTTASF  220
NR3C2-202      SVRDADYSYEQQNQQGSMSPAKIYQNVEQLVKFYKGNGHRPSTLSCVNTPLRSFMSDSGSSVNGGVMRAVVKSPIMCHEKSPSVCSPLNMTSSVCSPAGINSVSSTTASF  220
NR3C2-207      SVRDADYSYEQQNQQGSMSPAKIYQNVEQLVKFYKGNGHRPSTLSCVNTPLRSFMSDSGSSVNGGVMRAVVKSPIMCHEKSPSVCSPLNMTSSVCSPAGINSVSSTTASF  220
NR3C2-201      SVRDADYSYEQQNQQGSMSPAKIYQNVEQLVKFYKGNGHRPSTLSCVNTPLRSFMSDSGSSVNGGVMRAVVKSPIMCHEKSPSVCSPLNMTSSVCSPAGINSVSSTTASF  220

logo           GSFPVHSPITQGTPLTCSPNVENRGSRSHSPAHASNVGSPLSSPLSSMKSSISSPPSHCSVKSPVSSPNNVTLRSSVSSPANINNSRCSVSSPSNTNNRSTLSSPAASTV
DEXAMETHASONE  ..............................................................................................................    0
PF00105.1      ..............................................................................................................    0
PF00105        ..............................................................................................................    0
PF00104        ..............................................................................................................    0
PF00104.1      ..............................................................................................................    0
NR3C2-203      GSFPVHSPITQGTPLTCSPNVENRGSRSHSPAHASNVGSPLSSPLSSMKSSISSPPSHCSVKSPVSSPNNVTLRSSVSSPANINNSRCSVSSPSNTNNRSTLSSPAASTV  330
NR3C2-208      GSFPVHSPITQGTPLTCSPNVENRGSRSHSPAHASNVGSPLSSPLSSMKSSISSPPSHCSVKSPVSSPNNVTLRSSVSSPANINNSRCSVSSPSNTNNRSTLSSPAASTV  330
NR3C2-209      GSFPVHSPITQGTPLTCSPNVENRGSRSHSPAHASNVGSPLSSPLSSMKSSISSPPSHCSVKSPVSSPNNVTLRSSVSSPANINNSRCSVSSPSNTNNRSTLSSPAASTV  330
NR3C2-202      GSFPVHSPITQGTPLTCSPNVENRGSRSHSPAHASNVGSPLSSPLSSMKSSISSPPSHCSVKSPVSSPNNVTLRSSVSSPANINNSRCSVSSPSNTNNRSTLSSPAASTV  330
NR3C2-207      GSFPVHSPITQGTPLTCSPNVENRGSRSHSPAHASNVGSPLSSPLSSMKSSISSPPSHCSVKSPVSSPNNVTLRSSVSSPANINNSRCSVSSPSNTNNRSTLSSPAASTV  330
NR3C2-201      GSFPVHSPITQGTPLTCSPNVENRGSRSHSPAHASNVGSPLSSPLSSMKSSISSPPSHCSVKSPVSSPNNVTLRSSVSSPANINNSRCSVSSPSNTNNRSTLSSPAASTV  330
```

```
logo           GSICSPVNNAFSYTASGTSAGSSTLRDVVPSPDTQEKGAQEVPFPKTEEVESAISNGVTGQLNIVQYIKPEPDGAFSSSCLGGNSKINSDSSFSVPIKQESTKHSCSGTS
DEXAMETHASONE  ..............................................................................................................   0
PF00105.1      ..............................................................................................................   0
PF00105        ..............................................................................................................   0
PF00104        ..............................................................................................................   0
PF00104.1      ..............................................................................................................   0
NR3C2-203      GSICSPVNNAFSYTASGTSAGSSTLRDVVPSPDTQEKGAQEVPFPKTEEVESAISNGVTGQLNIVQYIKPEPDGAFSSSCLGGNSKINSDSSFSVPIKQESTKHSCSGTS 440
NR3C2-208      GSICSPVNNAFSYTASGTSAGSSTLRDVVPSPDTQEKGAQEVPFPKTEEVESAISNGVTGQLNIVQYIKPEPDGAFSSSCLGGNSKINSDSSFSVPIKQESTKHSCSGTS 440
NR3C2-209      GSICSPVNNAFSYTASGTSAGSSTLRDVVPSPDTQEKGAQEVPFPKTEEVESAISNGVTGQLNIVQYIKPEPDGAFSSSCLGGNSKINSDSSFSVPIKQESTKHSCSGTS 440
NR3C2-202      GSICSPVNNAFSYTASGTSAGSSTLRDVVPSPDTQEKGAQEVPFPKTEEVESAISNGVTGQLNIVQYIKPEPDGAFSSSCLGGNSKINSDSSFSVPIKQESTKHSCSGTS 440
NR3C2-207      GSICSPVNNAFSYTASGTSAGSSTLRDVVPSPDTQEKGAQEVPFPKTEEVESAISNGVTGQLNIVQYIKPEPDGAFSSSCLGGNSKINSDSSFSVPIKQESTKHSCSGTS 440
NR3C2-201      GSICSPVNNAFSYTASGTSAGSSTLRDVVPSPDTQEKGAQEVPFPKTEEVESAISNGVTGQLNIVQYIKPEPDGAFSSSCLGGNSKINSDSSFSVPIKQESTKHSCSGTS 440

logo           FKGNPTVNPFPFMDGSYFSFMDDKDYYSLSGILGPPVPGFDGNCEGSGFPVGIKQEPDDGSYYPEASIPSSAIVGVNSGGQSFHYRIGAQGTISLSRSARDQSFQHLSSF
DEXAMETHASONE  ..............................................................................................................   0
PF00105.1      ..............................................................................................................   0
PF00105        ..............................................................................................................   0
PF00104        ..............................................................................................................   0
PF00104.1      ..............................................................................................................   0
NR3C2-203      FKGNPTVNPFPFMDGSYFSFMDDKDYYSLSGILGPPVPGFDGNCEGSGFPVGIKQEPDDGSYYPEASIPSSAIVGVNSGGQSFHYRIGAQGTISLSRSARDQSFQHLSSF 550
NR3C2-208      FKGNPTVNPFPFMDGSYFSFMDDKDYYSLSGILGPPVPGFDGNCEGSGFPVGIKQEPDDGSYYPEASIPSSAIVGVNSGGQSFHYRIGAQGTISLSRSARDQSFQHLSSF 550
NR3C2-209      FKGNPTVNPFPFMDGSYFSFMDDKDYYSLSGILGPPVPGFDGNCEGSGFPVGIKQEPDDGSYYPEASIPSSAIVGVNSGGQSFHYRIGAQGTISLSRSARDQSFQHLSSF 550
NR3C2-202      FKGNPTVNPFPFMDGSYFSFMDDKDYYSLSGILGPPVPGFDGNCEGSGFPVGIKQEPDDGSYYPEASIPSSAIVGVNSGGQSFHYRIGAQGTISLSRSARDQSFQHLSSF 550
NR3C2-207      FKGNPTVNPFPFMDGSYFSFMDDKDYYSLSGILGPPVPGFDGNCEGSGFPVGIKQEPDDGSYYPEASIPSSAIVGVNSGGQSFHYRIGAQGTISLSRSARDQSFQHLSSF 550
NR3C2-201      FKGNPTVNPFPFMDGSYFSFMDDKDYYSLSGILGPPVPGFDGNCEGSGFPVGIKQEPDDGSYYPEASIPSSAIVGVNSGGQSFHYRIGAQGTISLSRSARDQSFQHLSSF 550

logo           PPVNTLVESWKSHGDLSSRRSDGYPVLEYIPENVSSSTLRSVSTGSSRPSKICLVCGDEASGCHYGVVTCGSCKVFFKRAVEGKCSWQHNYLCAGRNDCIIDKIRRKNCP
DEXAMETHASONE  ..............................................................................................................   0
PF00105.1      ...................................................ICLVCGDEASGCHYGVVTCGSCKVFFKRAVEGKCSWQHNYLCAGRNDCIIDKIRRKNCP  59
PF00105        ...................................................ICLVCGDEASGCHYGVVTCGSCKVFFKRAVEG....QHNYLCAGRNDCIIDKIRRKNCP  55
PF00104        ..............................................................................................................   0
PF00104.1      ..............................................................................................................   0
NR3C2-203      PPVNTLVESWKSHGDLSSRRSDGYPVLEYIPENVSSSTLRSVSTGSSRPSKICLVCGDEASGCHYGVVTCGSCKVFFKRAVEG....QHNYLCAGRNDCIIDKIRRKNCP 656
NR3C2-208      PPVNTLVESWKSHGDLSSRRSDGYPVLEYIPENVSSSTLRSVSTGSSRPSKICLVCGDEASGCHYGVVTCGSCKVFFKRAVEG....QHNYLCAGRNDCIIDKIRRKNCP 656
NR3C2-209      PPVNTLVESWKSHGDLSSRRSDGYPVLEYIPENVSSSTLRSVSTGSSRPSKICLVCGDEASGCHYGVVTCGSCKVFFKRAVEGKCSWQHNYLCAGRNDCIIDKIRRKNCP 660
NR3C2-202      PPVNTLVESWKSHGDLSSRRSDGYPVLEYIPENVSSSTLRSVSTGSSRPSKICLVCGDEASGCHYGVVTCGSCKVFFKRAVEG....QHNYLCAGRNDCIIDKIRRKNCP 656
NR3C2-207      PPVNTLVESWKSHGDLSSRRSDGYPVLEYIPENVSSSTLRSVSTGSSRPSKICLVCGDEASGCHYGVVTCGSCKVFFKRAVEGKCSWQHNYLCAGRNDCIIDKIRRKNCP 660
NR3C2-201      PPVNTLVESWKSHGDLSSRRSDGYPVLEYIPENVSSSTLRSVSTGSSRPSKICLVCGDEASGCHYGVVTCGSCKVFFKRAVEG....QHNYLCAGRNDCIIDKIRRKNCP 656
```

logo

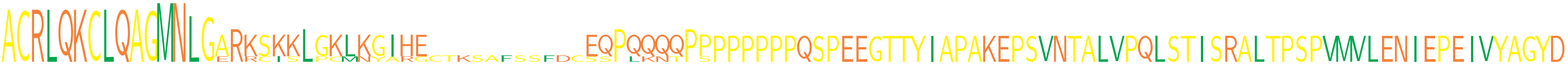

```
DEXAMETHASONE  ..............................................................................................................  0
PF00105.1      ACRLQKCLQAGMN.................................................................................................  72
PF00105        ACRLQKCLQAGMN.................................................................................................  68
PF00104        ..............................................................................................................  0
PF00104.1      ...........MNLG...............................................................................................  4
NR3C2-203      ACRLQKCLQAGMNLGARKSKKLGKLKGIHE...........EQPQQQQPPPPPPPPQSPEEGTTYIAPAKEPSVNTALVPQLSTISRALTPSPVMVLENIEPEIVYAGYD  755
NR3C2-208      ACRLQKCLQAGMNLG...............................................................................................  671
NR3C2-209      ACRLQKCLQAGMNLGARKSKKLGKLKGIHE...........EQPQQQQPPPPPPPPQSPEEGTTYIAPAKEPSVNTALVPQLSTISRALTPSPVMVLENIEPEIVYAGYD  759
NR3C2-202      ACRLQKCLQAGMNLGARKSKKLGKLKGIHE...........EQPQQQQPPPPPPPPQSPEEGTTYIAPAKEPSVNTALVPQLSTISRALTPSPVMVLENIEPEIVYAGYD  755
NR3C2-207      ACRLQKCLQAGMNLGARKSKKLGKLKGIHE...........EQPQQQQPPPPPPPPQSPEEGTTYIAPAKEPSVNTALVPQLSTISRALTPSPVMVLENIEPEIVYAGYD  759
NR3C2-201      ACRLQKCLQAGMNLGERRCISLPCMNYARGCTKSAFSSFDCSSPLKNTPS............................................................  706
```

logo

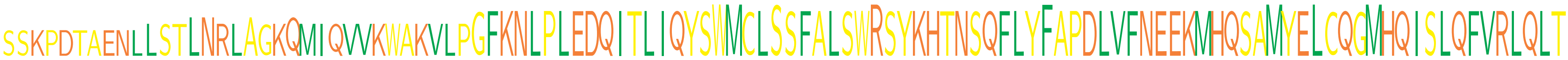

```
DEXAMETHASONE  ..........L..LN..A..Q.............................WM..S......R...........F...............M..L...M.............  13
PF00105.1      ..............................................................................................................  72
PF00105        ..............................................................................................................  68
PF00104        ...........STLNRLAGKQMIQVVKWAKVLPGFKNLPLEDQITLIQYSWMCLSSFALSWRSYKHTNSQFLYFAPDLVFNEEKMHQSAMYELCQGMHQISLQFVRLQLT  99
PF00104.1      .................................GFKNLPLEDQITLIQYSWMCLSSFALSWRSYKHTNSQFLYFAPDLVFNEEKMHQSAMYELCQGMHQISLQFVRLQLT  81
NR3C2-203      SSKPDTAENLLSTLNRLAGKQMIQVVKWAKVLPGFKNLPLEDQITLIQYSWMCLSSFALSWRSYKHTNSQFLYFAPDLVFNEEKMHQSAMYELCQGMHQISLQFVRLQLT  865
NR3C2-208      .................................GFKNLPLEDQITLIQYSWMCLSSFALSWRSYKHTNSQFLYFAPDLVFNEEKMHQSAMYELCQGMHQISLQFVRLQLT  748
NR3C2-209      SSKPDTAENLLSTLNRLAGKQMIQVVKWAKVLPGFKNLPLEDQITLIQYSWMCLSSFALSWRSYKHTNSQFLYFAPDLVFNEEKMHQSAMYELCQGMHQISLQFVRLQLT  869
NR3C2-202      SSKPDTAENLLSTLNRLAGKQMIQVVKWAKVLPGFKNLPLEDQITLIQYSWMCLSSFALSWRSYKHTNSQFLYFAPDLVFNEEKMHQSAMYELCQGMHQISLQFVRLQLT  865
NR3C2-207      SSKPDTAENLLSTLNRLAGKQMIQVVKWAKVLPGFKNLPLEDQITLIQYSWMCLSSFALSWRSYKHTNSQFLYFAPDLVFNEEKMHQSAMYELCQGMHQISLQFVRLQLT  869
NR3C2-201      ..............................................................................................................  706
```

logo

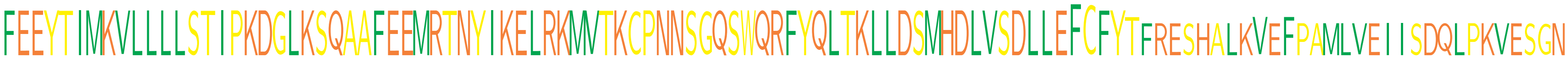

```
DEXAMETHASONE  ...........................................................................FC..T........V.F...................  18
PF00105.1      ..............................................................................................................  72
PF00105        ..............................................................................................................  68
PF00104        FEEYTIMKVLLLLSTIPKDGLKSQAAFEEMRTNYIKELRKMVTKCPNNSGQSWQRFYQLTKLLDSMHDLVSDLLEFCFY...............................  178
PF00104.1      FEEYTIMKVLLLLSTIPKDGLKSQAAFEEMRTNYIKELRKMVTKCPNNSGQSWQRFYQLTKLLDSMHDLVSDLLEFCFY...............................  160
NR3C2-203      FEEYTIMKVLLLLSTIPKDGLKSQAAFEEMRTNYIKELRKMVTKCPNNSGQSWQRFYQLTKLLDSMHDLVSDLLEFCFYTFRESHALKVEFPAMLVEIISDQLPKVESGN  975
NR3C2-208      FEEYTIMKVLLLLSTIPKDGLKSQAAFEEMRTNYIKELRKMVTKCPNNSGQSWQRFYQLTKLLDSMHDLVSDLLEFCFYTFRESHALKVEFPAMLVEIISDQLPKVESGN  858
NR3C2-209      FEEYTIMKVLLLLSTIPKDGLKSQAAFEEMRTNYIKELRKMVTKCPNNSGQSWQRFYQLTKLLDSMHDLVSDLLEFCFYTFRESHALKVEFPAMLVEIISDQLPKVESGN  979
NR3C2-202      FEEYTIMKVLLLLSTIPKDGLKSQAAFEEMRTNYIKELRKMVTKCPNNSGQSWQRFYQLTKLLDSMHDLVSDLLEFCFYTFRESHALKVEFPAMLVEIISDQLPKVESGN  975
NR3C2-207      FEEYTIMKVLLLLSTIPKDGLKSQAAFEEMRTNYIKELRKMVTKCPNNSGQSWQRFYQLTKLLDSMHDLVSDLLEFCFYTFRESHALKVEFPAMLVEIISDQLPKVESGN  979
NR3C2-201      ..............................................................................................................  706
```

logo

AKPLYFHRK

DEXAMETHASONE	....	18
PF00105.1	....	72
PF00105	....	68
PF00104	....	178
PF00104.1	....	160
NR3C2-203	AKPLYFHRK	984
NR3C2-208	AKPLYFHRK	867
NR3C2-209	AKPLYFHRK	988
NR3C2-202	AKPLYFHRK	984
NR3C2-207	AKPLYFHRK	988
NR3C2-201	....	706

X non conserved
X similar
X $\geq 0\%$ conserved
X $\geq 50\%$ conserved

logo

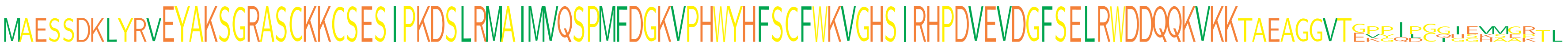

```
NIRAPARIB    ..............................................................................................................    0
PF00645      ...........EYAKSGRASCKKCSESIPKDSLRMAIMVQSPMFDGKVPHWYHFSCFWKVGHSIRHPDVEVDGFSELRWDDQQKVKK.......................   76
PF08063      ..............................................................................................................    0
PF00645.1    ...........EYAKSGRASCKKCSESIPKDSLRMAIMVQSPMFDGKVPHWYHFSCFWKVGHSIRHPDVEVDGFSELRWDDQQKVKK.......................   76
PF05406      ..............................................................................................................    0
PF02877      ..............................................................................................................    0
PF00533      ..............................................................................................................    0
PF00644      ..............................................................................................................    0
PARP1-201    MAESSDKLYRVEYAKSGRASCKKCSESIPKDSLRMAIMVQSPMFDGKVPHWYHFSCFWKVGHSIRHPDVEVDGFSELRWDDQQKVKKTAEAGGVTGVYILGQHGHSAPTL  110
PARP1-210    MAESSDKLYRVEYAKSGRASCKKCSESIPKDSLRMAIMVQSPMFDGKVPHWYHFSCFWKVGHSIRHPDVEVDGFSELRWDDQQKVKKTAEAGGVTEPPIPCGIEVMGR..  108
PARP1-202    MAESSDKLYRVEYAKSGRASCKKCSESIPKDSLRMAIMVQSPMFDGKVPHWYHFSCFWKVGHSIRHPDVEVDGFSELRWDDQQKVKKTAEAGGVTEPPIPCGIEVMGR..  108
PARP1-203    MAESSDKLYRVEYAKSGRASCKKCSESIPKDSLRMAIMVQSPMFDGKVPHWYHFSCFWKVGHSIRHPDVEVDGFSELRWDDQQKVKKTAEAGGVTGKGQDGIGSKAEKTL  110
```

logo

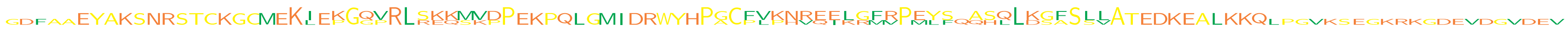

```
NIRAPARIB    ..............................................................................................................    0
PF00645      ..............................................................................................................   76
PF08063      ..............................................................................................................    0
PF00645.1    .....EYAKSNRSTCKGCMEKIEKGQVRLSKKMVDPEKPQLGMIDRWYHPGCFVKNREELGFRPEYS.ASQLKGFSLLATEDKEALKKQ.....................  159
PF05406      ..............................................................................................................    0
PF02877      ..............................................................................................................    0
PF00533      ..............................................................................................................    0
PF00644      ..............................................................................................................    0
PARP1-201    ....................KL.PGGPRLREQSKPP.............PACPLPHVQIKRMVPMLFQQHLLDSASSVA...............................  155
PARP1-210    ..............................................................................................................  108
PARP1-202    ..............................................................................................................  108
PARP1-203    GDFAAEYAKSNRSTCKGCMEKIEKGQVRLSKKMVDPEKPQLGMIDRWYHPGCFVKNREELGFRPEYS.ASQLKGFSLLATEDKEALKKQLPGVKSEGKRKGDEVDGVDEV  219
```

logo

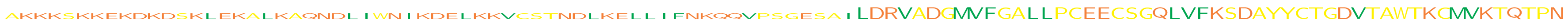

```
NIRAPARIB    ..............................................................................................................    0
PF00645      ..............................................................................................................   76
PF08063      ............................................................LDRVADGMVFGALLPCEECSGQLVFKSDAYYCTGDVTAWTKCMVKTQTPN   50
PF00645.1    ..............................................................................................................  159
PF05406      ..............................................................................................................    0
PF02877      ..............................................................................................................    0
PF00533      ..............................................................................................................    0
PF00644      ..............................................................................................................    0
PARP1-201    ..............................................................................................................  155
PARP1-210    ..............................................................................................................  108
PARP1-202    ..............................................................................................................  108
PARP1-203    AKKKSKKEKDKDSKLEKALKAQNDLIWNIKDELKKVCSTNDLKELLIFNKQQVPSGESAILDRVADGMVFGALLPCEECSGQLVFKSDAYYCTGDVTAWTKCMVKTQTPN  329
```

```
logo
              RKEWVTPKEFREISYLKKLKVKKQDRIFPPETSASVAATPPPSTASAPAAVNSSASADKPLSNMKILTLGKLSRNKDEVKAMIEKLGGKLTGTANKASLCISTKKEVEKM
NIRAPARIB     ..............................................................................................................    0
PF00645       ..............................................................................................................   76
PF08063       RK............................................................................................................   52
PF00645.1     ..............................................................................................................  159
PF05406       ..............................................................................................................    0
PF02877       ..............................................................................................................    0
PF00533       ..........................................................KPLSNMKILTLGKLSRNKDEVKAMIEKLGGKLTGTANKASLCISTKKEVEKM   52
PF00644       ..............................................................................................................    0
PARP1-201     ..............................................................................................................  155
PARP1-210     ..............................................................................................................  108
PARP1-202     ..............................................................................................................  108
PARP1-203     RKEWVTPKEFREISYLKKLKVKKQDRIFPPETSASVAATPPPSTASAPAAVNSSASADKPLSNMKILTLGKLSRNKDEVKAMIEKLGGKLTGTANKASLCISTKKEVEKM  439
```

```
logo
              NKKMEEVKEANIRVVSEDFLQDVSASTKSLQELFLAHILSPWGAEVKAEPVEVVAPRGKSGAALSKKSKGQVKEEGINKSEKRMKLTLKGGAAVDPDSGLEHSAHVLEKG
NIRAPARIB     ..............................................................................................................    0
PF00645       ..............................................................................................................   76
PF08063       ..............................................................................................................   52
PF00645.1     ..............................................................................................................  159
PF05406       ..............................................................................................................    0
PF02877       ..............................................................................................................    0
PF00533       NKKMEEVKEANIRVVSEDFLQD........................................................................................   74
PF00644       ..............................................................................................................    0
PARP1-201     ..............................................................................................................  155
PARP1-210     ..............................................................................................................  108
PARP1-202     ..............................................................................................................  108
PARP1-203     NKKMEEVKEANIRVVSEDFLQDVSASTKSLQELFLAHILSPWGAEVKAEPVEVVAPRGKSGAALSKKSKGQVKEEGINKSEKRMKLTLKGGAAVDPDSGLEHSAHVLEKG  549
```

```
logo
              GKVFSATLGLVDIVKGTNSYYKLQLLEDDKENRYWIFRSWGRVGTVIGSNKLEQMPSKEDAIEHFMKLYEEKTGNAWHSKNFTKYPKKFYPLEIDYGQDEEAVKKLTVNP
NIRAPARIB     ..............................................................................................................    0
PF00645       ..............................................................................................................   76
PF08063       ..............................................................................................................   52
PF00645.1     ..............................................................................................................  159
PF05406       ....SATLGLVDIVKGTNSYYKLQLLEDDKENRYWIFRSWGRVGTVIGSNKLEQMPSKEDAIEHFMKLYEEKTGNAWHSKN.............................   77
PF02877       ..............................................................................................................    0
PF00533       ..............................................................................................................   74
PF00644       ..............................................................................................................    0
PARP1-201     ..............................................................................................................  155
PARP1-210     ..............................................................................................................  108
PARP1-202     ..............................................................................................................  108
PARP1-203     GKVFSATLGLVDIVKGTNSYYKLQLLEDDKENRYWIFRSWGRVGTVIGSNKLEQMPSKEDAIEHFMKLYEEKTGNAWHSKNFTKYPKKFYPLEIDYGQDEEAVKKLTVNP  659
```

```
logo
             GTKSKLPKPVQDLIKMIFDVESMKKAMVEYEIDLQKMPLGKLSKRQIQAAYSILSEVQQAVSQGSSDSQILDLSNRFYTLIPHDFGMKKPPLLNNADSVQAKVEMLDNLL
NIRAPARIB    ...................................................................A...D......................................     2
PF00645      ..............................................................................................................    76
PF08063      ..............................................................................................................    52
PF00645.1    ..............................................................................................................   159
PF05406      ..............................................................................................................    77
PF02877      ...SKLPKPVQDLIKMIFDVESMKKAMVEYEIDLQKMPLGKLSKRQIQAAYSILSEVQQAVSQGSSDSQILDLSNRFYTLIPHDFGMKKPPLLNNADSVQAKVEMLDNLL   107
PF00533      ..............................................................................................................    74
PF00644      ..............................................................................................................     0
PARP1-201    ..............................................................................................................   155
PARP1-210    ..............................................................................................................   108
PARP1-202    ..............................................................................................................   108
PARP1-203    GTKSKLPKPVQDLIKMIFDVESMKKAMVEYEIDLQKMPLGKLSKRQIQAAYSILSEVQQAVSQGSSDSQILDLSNRFYTLIPHDFGMKKPPLLNNADSVQAKVEMLDNLL   769

logo
             DIEVAYSLLRGGSDDSSKDPIDVNYEKLKTDIKVVDRDSEEAEIIRKYVKNTHATTHNAYDLEVIDIFKIEREGECQRYKPFKQLHNRRLLWHGSRTTNFAGILSQGLRI
NIRAPARIB    ............................................................................................HG................     4
PF00645      ..............................................................................................................    76
PF08063      ..............................................................................................................    52
PF00645.1    ..............................................................................................................   159
PF05406      ..............................................................................................................    77
PF02877      DIEVAYSLLRGGSDDSSKDPIDVNY.....................................................................................   132
PF00533      ..............................................................................................................    74
PF00644      ......................................SEEAEIIRKYVKNTHATTHNAYDLEVIDIFKIEREGECQRYKPFKQLHNRRLLWHGSRTTNFAGILSQGLRI    72
PARP1-201    ..............................................................................................................   155
PARP1-210    ..............................................................................................................   108
PARP1-202    ..............................................................................................................   108
PARP1-203    DIEVAYSLLRGGSDDSSKDPIDVNYEKLKTDIKVVDRDSEEAEIIRKYVKNTHATTHNAYDLEVIDIFKIEREGECQRYKPFKQLHNRRLLWHGSRTTNFAGILSQGLRI   879

logo
             APPEAPVTGYMFGKGIYFADMVSKSANYCHTSQGDPIGLILLGEVALGNMYELKHASHISKLPKGKHSVKGLGKTTPDPSANISLDGVDVPLGTGISSGVNDTSLLYNEY
NIRAPARIB    ........GY......YFA....KS..Y................................................................................E.    13
PF00645      ..............................................................................................................    76
PF08063      ..............................................................................................................    52
PF00645.1    ..............................................................................................................   159
PF05406      ..............................................................................................................    77
PF02877      ..............................................................................................................   132
PF00533      ..............................................................................................................    74
PF00644      APPEAPVTGYMFGKGIYFADMVSKSANYCHTSQGDPIGLILLGEVALGNMYELKHASHISKLPKGKHSVKGLGKTTPDPSANISLDGVDVPLGTGISSGVNDTSLLYNEY   182
PARP1-201    ..............................................................................................................   155
PARP1-210    ..............................................................................................................   108
PARP1-202    ..............................................................................................................   108
PARP1-203    APPEAPVTGYMFGKGIYFADMVSKSANYCHTSQGDPIGLILLGEVALGNMYELKHASHISKLPKGKHSVKGLGKTTPDPSANISLDGVDVPLGTGISSGVNDTSLLYNEY   989
```

logo

```
           IVYDIAQVNLKYLLKLKFNFKTSLW
NIRAPARIB  .........................    13
PF00645    .........................    76
PF08063    .........................    52
PF00645.1  .........................   159
PF05406    .........................    77
PF02877    .........................   132
PF00533    .........................    74
PF00644    IVYDIAQVNLKYLLKLKF.......   200
PARP1-201  .........................   155
PARP1-210  .........................   108
PARP1-202  .........................   108
PARP1-203  IVYDIAQVNLKYLLKLKFNFKTSLW  1014
```

X non conserved
X similar
X ≥ 0% conserved
X ≥ 50% conserved

logo

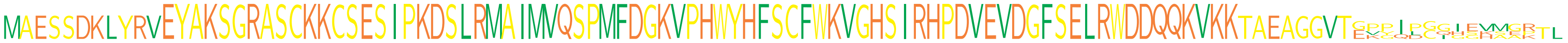

```
OLAPARIB     ..............................................................................................................    0
PF00645      ...........EYAKSGRASCKKCSESIPKDSLRMAIMVQSPMFDGKVPHWYHFSCFWKVGHSIRHPDVEVDGFSELRWDDQQKVKK.......................   76
PF08063      ..............................................................................................................    0
PF00645.1    ...........EYAKSGRASCKKCSESIPKDSLRMAIMVQSPMFDGKVPHWYHFSCFWKVGHSIRHPDVEVDGFSELRWDDQQKVKK.......................   76
PF05406      ..............................................................................................................    0
PF02877      ..............................................................................................................    0
PF00533      ..............................................................................................................    0
PF00644      ..............................................................................................................    0
PARP1-201    MAESSDKLYRVEYAKSGRASCKKCSESIPKDSLRMAIMVQSPMFDGKVPHWYHFSCFWKVGHSIRHPDVEVDGFSELRWDDQQKVKKTAEAGGVTGVYILGQHGHSAPTL  110
PARP1-210    MAESSDKLYRVEYAKSGRASCKKCSESIPKDSLRMAIMVQSPMFDGKVPHWYHFSCFWKVGHSIRHPDVEVDGFSELRWDDQQKVKKTAEAGGVTEPPIPCGIEVMGR..  108
PARP1-202    MAESSDKLYRVEYAKSGRASCKKCSESIPKDSLRMAIMVQSPMFDGKVPHWYHFSCFWKVGHSIRHPDVEVDGFSELRWDDQQKVKKTAEAGGVTEPPIPCGIEVMGR..  108
PARP1-203    MAESSDKLYRVEYAKSGRASCKKCSESIPKDSLRMAIMVQSPMFDGKVPHWYHFSCFWKVGHSIRHPDVEVDGFSELRWDDQQKVKKTAEAGGVTGKGQDGIGSKAEKTL  110
```

logo

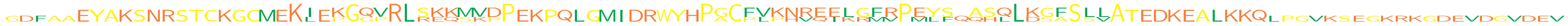

```
OLAPARIB     ..............................................................................................................    0
PF00645      ..............................................................................................................   76
PF08063      ..............................................................................................................    0
PF00645.1    .....EYAKSNRSTCKGCMEKIEKGQVRLSKKMVDPEKPQLGMIDRWYHPGCFVKNREELGFRPEYS.ASQLKGFSLLATEDKEALKKQ.....................  159
PF05406      ..............................................................................................................    0
PF02877      ..............................................................................................................    0
PF00533      ..............................................................................................................    0
PF00644      ..............................................................................................................    0
PARP1-201    ....................KL.PGGPRLREQSKPP.............PACPLPHVQIKRMVPMLFQQHLLDSASSVA...............................  155
PARP1-210    ..............................................................................................................  108
PARP1-202    ..............................................................................................................  108
PARP1-203    GDFAAEYAKSNRSTCKGCMEKIEKGQVRLSKKMVDPEKPQLGMIDRWYHPGCFVKNREELGFRPEYS.ASQLKGFSLLATEDKEALKKQLPGVKSEGKRKGDEVDGVDEV  219
```

logo

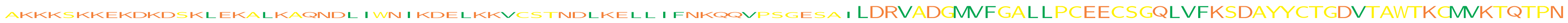

```
OLAPARIB     ..............................................................................................................    0
PF00645      ..............................................................................................................   76
PF08063      ............................................................LDRVADGMVFGALLPCEECSGQLVFKSDAYYCTGDVTAWTKCMVKTQTPN   50
PF00645.1    ..............................................................................................................  159
PF05406      ..............................................................................................................    0
PF02877      ..............................................................................................................    0
PF00533      ..............................................................................................................    0
PF00644      ..............................................................................................................    0
PARP1-201    ..............................................................................................................  155
PARP1-210    ..............................................................................................................  108
PARP1-202    ..............................................................................................................  108
PARP1-203    AKKKSKKEKDKDSKLEKALKAQNDLIWNIKDELKKVCSTNDLKELLIFNKQQVPSGESAILDRVADGMVFGALLPCEECSGQLVFKSDAYYCTGDVTAWTKCMVKTQTPN  329
```

```
logo
              RKEWVTPKEFREISYLKKLKVKKQDRIFPPETSASVAATPPPSTASAPAAVNSSASADKPLSNMKILTLGKLSRNKDEVKAMIEKLGGKLTGTANKASLCISTKKEVEKM
OLAPARIB      ..............................................................................................................   0
PF00645       ..............................................................................................................  76
PF08063       RK............................................................................................................  52
PF00645.1     .............................................................................................................. 159
PF05406       ..............................................................................................................   0
PF02877       ..............................................................................................................   0
PF00533       ..........................................................KPLSNMKILTLGKLSRNKDEVKAMIEKLGGKLTGTANKASLCISTKKEVEKM  52
PF00644       ..............................................................................................................   0
PARP1-201     .............................................................................................................. 155
PARP1-210     .............................................................................................................. 108
PARP1-202     .............................................................................................................. 108
PARP1-203     RKEWVTPKEFREISYLKKLKVKKQDRIFPPETSASVAATPPPSTASAPAAVNSSASADKPLSNMKILTLGKLSRNKDEVKAMIEKLGGKLTGTANKASLCISTKKEVEKM 439


logo
              NKKMEEVKEANIRVVSEDFLQDVSASTKSLQELFLAHILSPWGAEVKAEPVEVVAPRGKSGAALSKKSKGQVKEEGINKSEKRMKLTLKGGAAVDPDSGLEHSAHVLEKG
OLAPARIB      ..............................................................................................................   0
PF00645       ..............................................................................................................  76
PF08063       ..............................................................................................................  52
PF00645.1     .............................................................................................................. 159
PF05406       ..............................................................................................................   0
PF02877       ..............................................................................................................   0
PF00533       NKKMEEVKEANIRVVSEDFLQD........................................................................................  74
PF00644       ..............................................................................................................   0
PARP1-201     .............................................................................................................. 155
PARP1-210     .............................................................................................................. 108
PARP1-202     .............................................................................................................. 108
PARP1-203     NKKMEEVKEANIRVVSEDFLQDVSASTKSLQELFLAHILSPWGAEVKAEPVEVVAPRGKSGAALSKKSKGQVKEEGINKSEKRMKLTLKGGAAVDPDSGLEHSAHVLEKG 549


logo
              GKVFSATLGLVDIVKGTNSYYKLQLLEDDKENRYWIFRSWGRVGTVIGSNKLEQMPSKEDAIEHFMKLYEEKTGNAWHSKNFTKYPKKFYPLEIDYGQDEEAVKKLTVNP
OLAPARIB      ..............................................................................................................   0
PF00645       ..............................................................................................................  76
PF08063       ..............................................................................................................  52
PF00645.1     .............................................................................................................. 159
PF05406       ....SATLGLVDIVKGTNSYYKLQLLEDDKENRYWIFRSWGRVGTVIGSNKLEQMPSKEDAIEHFMKLYEEKTGNAWHSKN.............................  77
PF02877       ..............................................................................................................   0
PF00533       ..............................................................................................................  74
PF00644       ..............................................................................................................   0
PARP1-201     .............................................................................................................. 155
PARP1-210     .............................................................................................................. 108
PARP1-202     .............................................................................................................. 108
PARP1-203     GKVFSATLGLVDIVKGTNSYYKLQLLEDDKENRYWIFRSWGRVGTVIGSNKLEQMPSKEDAIEHFMKLYEEKTGNAWHSKNFTKYPKKFYPLEIDYGQDEEAVKKLTVNP 659
```

```
logo
              GTKSKLPKPVQDLIKMIFDVESMKKAMVEYEIDLQKMPLGKLSKRQIQAAYSILSEVQQAVSQGSSDSQILDLSNRFYTLIPHDFGMKKPPLLNNADSVQAKVEMLDNLL
OLAPARIB      ..............................................................................................................    0
PF00645       ..............................................................................................................   76
PF08063       ..............................................................................................................   52
PF00645.1     ..............................................................................................................  159
PF05406       ..............................................................................................................   77
PF02877       ...SKLPKPVQDLIKMIFDVESMKKAMVEYEIDLQKMPLGKLSKRQIQAAYSILSEVQQAVSQGSSDSQILDLSNRFYTLIPHDFGMKKPPLLNNADSVQAKVEMLDNLL  107
PF00533       ..............................................................................................................   74
PF00644       ..............................................................................................................    0
PARP1-201     ..............................................................................................................  155
PARP1-210     ..............................................................................................................  108
PARP1-202     ..............................................................................................................  108
PARP1-203     GTKSKLPKPVQDLIKMIFDVESMKKAMVEYEIDLQKMPLGKLSKRQIQAAYSILSEVQQAVSQGSSDSQILDLSNRFYTLIPHDFGMKKPPLLNNADSVQAKVEMLDNLL  769
```

```
logo
              DIEVAYSLLRGGSDDSSKDPIDVNYEKLKTDIKVVDRDSEEAEIIRKYVKNTHATTHNAYDLEVIDIFKIEREGECQRYKPFKQLHNRRLLWHGSRTTNFAGILSQGLRI
OLAPARIB      ............................................................................................HG.............LR.    4
PF00645       ..............................................................................................................   76
PF08063       ..............................................................................................................   52
PF00645.1     ..............................................................................................................  159
PF05406       ..............................................................................................................   77
PF02877       DIEVAYSLLRGGSDDSSKDPIDVNY.....................................................................................  132
PF00533       ..............................................................................................................   74
PF00644       ......................................SEEAEIIRKYVKNTHATTHNAYDLEVIDIFKIEREGECQRYKPFKQLHNRRLLWHGSRTTNFAGILSQGLRI   72
PARP1-201     ..............................................................................................................  155
PARP1-210     ..............................................................................................................  108
PARP1-202     ..............................................................................................................  108
PARP1-203     DIEVAYSLLRGGSDDSSKDPIDVNYEKLKTDIKVVDRDSEEAEIIRKYVKNTHATTHNAYDLEVIDIFKIEREGECQRYKPFKQLHNRRLLWHGSRTTNFAGILSQGLRI  879
```

```
logo
              APPEAPVTGYMFGKGIYFADMVSKSANYCHTSQGDPIGLILLGEVALGNMYELKHASHISKLPKGKHSVKGLGKTTPDPSANISLDGVDVPLGTGISSGVNDTSLLYNEY
OLAPARIB      .........Y....GIYF.....KS..Y................................................................................E.   13
PF00645       ..............................................................................................................   76
PF08063       ..............................................................................................................   52
PF00645.1     ..............................................................................................................  159
PF05406       ..............................................................................................................   77
PF02877       ..............................................................................................................  132
PF00533       ..............................................................................................................   74
PF00644       APPEAPVTGYMFGKGIYFADMVSKSANYCHTSQGDPIGLILLGEVALGNMYELKHASHISKLPKGKHSVKGLGKTTPDPSANISLDGVDVPLGTGISSGVNDTSLLYNEY  182
PARP1-201     ..............................................................................................................  155
PARP1-210     ..............................................................................................................  108
PARP1-202     ..............................................................................................................  108
PARP1-203     APPEAPVTGYMFGKGIYFADMVSKSANYCHTSQGDPIGLILLGEVALGNMYELKHASHISKLPKGKHSVKGLGKTTPDPSANISLDGVDVPLGTGISSGVNDTSLLYNEY  989
```

logo

```
           IVYDIAQVNLKYLLKLKFNFKTSLW
OLAPARIB   .........................    13
PF00645    .........................    76
PF08063    .........................    52
PF00645.1  .........................   159
PF05406    .........................    77
PF02877    .........................   132
PF00533    .........................    74
PF00644    IVYDIAQVNLKYLLKLKF.......   200
PARP1-201  .........................   155
PARP1-210  .........................   108
PARP1-202  .........................   108
PARP1-203  IVYDIAQVNLKYLLKLKFNFKTSLW  1014
```

- X non conserved
- X similar
- X $\geq$ 0% conserved
- X $\geq$ 50% conserved

```
logo
RUCAPARIB    ..............................................................................................................    0
PF00645      ...........EYAKSGRASCKKCSESIPKDSLRMAIMVQSPMFDGKVPHWYHFSCFWKVGHSIRHPDVEVDGFSELRWDDQQKVKK.......................   76
PF08063      ..............................................................................................................    0
PF00645.1    ...........EYAKSGRASCKKCSESIPKDSLRMAIMVQSPMFDGKVPHWYHFSCFWKVGHSIRHPDVEVDGFSELRWDDQQKVKK.......................   76
PF05406      ..............................................................................................................    0
PF02877      ..............................................................................................................    0
PF00533      ..............................................................................................................    0
PF00644      ..............................................................................................................    0
PARP1-201    MAESSDKLYRVEYAKSGRASCKKCSESIPKDSLRMAIMVQSPMFDGKVPHWYHFSCFWKVGHSIRHPDVEVDGFSELRWDDQQKVKKTAEAGGVTGVYILGQHGHSAPTL  110
PARP1-210    MAESSDKLYRVEYAKSGRASCKKCSESIPKDSLRMAIMVQSPMFDGKVPHWYHFSCFWKVGHSIRHPDVEVDGFSELRWDDQQKVKKTAEAGGVTEPPIPCGIEVMGR..  108
PARP1-202    MAESSDKLYRVEYAKSGRASCKKCSESIPKDSLRMAIMVQSPMFDGKVPHWYHFSCFWKVGHSIRHPDVEVDGFSELRWDDQQKVKKTAEAGGVTEPPIPCGIEVMGR..  108
PARP1-203    MAESSDKLYRVEYAKSGRASCKKCSESIPKDSLRMAIMVQSPMFDGKVPHWYHFSCFWKVGHSIRHPDVEVDGFSELRWDDQQKVKKTAEAGGVTGKGQDGIGSKAEKTL  110

logo
RUCAPARIB    ..............................................................................................................    0
PF00645      ..............................................................................................................   76
PF08063      ..............................................................................................................    0
PF00645.1    .....EYAKSNRSTCKGCMEKIEKGQVRLSKKMVDPEKPQLGMIDRWYHPGCFVKNREELGFRPEYS.ASQLKGFSLLATEDKEALKKQ.....................  159
PF05406      ..............................................................................................................    0
PF02877      ..............................................................................................................    0
PF00533      ..............................................................................................................    0
PF00644      ..............................................................................................................    0
PARP1-201    ....................KL.PGGPRLREQSKPP.............PACPLPHVQIKRMVPMLFQQHLLDSASSVA...............................  155
PARP1-210    ..............................................................................................................  108
PARP1-202    ..............................................................................................................  108
PARP1-203    GDFAAEYAKSNRSTCKGCMEKIEKGQVRLSKKMVDPEKPQLGMIDRWYHPGCFVKNREELGFRPEYS.ASQLKGFSLLATEDKEALKKQLPGVKSEGKRKGDEVDGVDEV  219

logo
RUCAPARIB    ..............................................................................................................    0
PF00645      ..............................................................................................................   76
PF08063      ............................................................LDRVADGMVFGALLPCEECSGQLVFKSDAYYCTGDVTAWTKCMVKTQTPN   50
PF00645.1    ..............................................................................................................  159
PF05406      ..............................................................................................................    0
PF02877      ..............................................................................................................    0
PF00533      ..............................................................................................................    0
PF00644      ..............................................................................................................    0
PARP1-201    ..............................................................................................................  155
PARP1-210    ..............................................................................................................  108
PARP1-202    ..............................................................................................................  108
PARP1-203    AKKKSKKEKDKDSKLEKALKAQNDLIWNIKDELKKVCSTNDLKELLIFNKQQVPSGESAILDRVADGMVFGALLPCEECSGQLVFKSDAYYCTGDVTAWTKCMVKTQTPN  329
```

```
logo
           RKEWVTPKEFREISYLKKLKVKKQDRIFPPETSASVAATPPPSTASAPAAVNSSASADKPLSNMKILTLGKLSRNKDEVKAMIEKLGGKLTGTANKASLCISTKKEVEKM
RUCAPARIB  ..............................................................................................................   0
PF00645    ..............................................................................................................  76
PF08063    RK............................................................................................................  52
PF00645.1  .............................................................................................................. 159
PF05406    ..............................................................................................................   0
PF02877    ..............................................................................................................   0
PF00533    ..........................................................KPLSNMKILTLGKLSRNKDEVKAMIEKLGGKLTGTANKASLCISTKKEVEKM  52
PF00644    ..............................................................................................................   0
PARP1-201  .............................................................................................................. 155
PARP1-210  .............................................................................................................. 108
PARP1-202  .............................................................................................................. 108
PARP1-203  RKEWVTPKEFREISYLKKLKVKKQDRIFPPETSASVAATPPPSTASAPAAVNSSASADKPLSNMKILTLGKLSRNKDEVKAMIEKLGGKLTGTANKASLCISTKKEVEKM 439
```

```
logo
           NKKMEEVKEANIRVVSEDFLQDVSASTKSLQELFLAHILSPWGAEVKAEPVEVVAPRGKSGAALSKKSKGQVKEEGINKSEKRMKLTLKGGAAVDPDSGLEHSAHVLEKG
RUCAPARIB  ..............................................................................................................   0
PF00645    ..............................................................................................................  76
PF08063    ..............................................................................................................  52
PF00645.1  .............................................................................................................. 159
PF05406    ..............................................................................................................   0
PF02877    ..............................................................................................................   0
PF00533    NKKMEEVKEANIRVVSEDFLQD........................................................................................  74
PF00644    ..............................................................................................................   0
PARP1-201  .............................................................................................................. 155
PARP1-210  .............................................................................................................. 108
PARP1-202  .............................................................................................................. 108
PARP1-203  NKKMEEVKEANIRVVSEDFLQDVSASTKSLQELFLAHILSPWGAEVKAEPVEVVAPRGKSGAALSKKSKGQVKEEGINKSEKRMKLTLKGGAAVDPDSGLEHSAHVLEKG 549
```

```
logo
           GKVFSATLGLVDIVKGTNSYYKLQLLEDDKENRYWIFRSWGRVGTVIGSNKLEQMPSKEDAIEHFMKLYEEKTGNAWHSKNFTKYPKKFYPLEIDYGQDEEAVKKLTVNP
RUCAPARIB  ..............................................................................................................   0
PF00645    ..............................................................................................................  76
PF08063    ..............................................................................................................  52
PF00645.1  .............................................................................................................. 159
PF05406    ....SATLGLVDIVKGTNSYYKLQLLEDDKENRYWIFRSWGRVGTVIGSNKLEQMPSKEDAIEHFMKLYEEKTGNAWHSKN.............................  77
PF02877    ..............................................................................................................   0
PF00533    ..............................................................................................................  74
PF00644    ..............................................................................................................   0
PARP1-201  .............................................................................................................. 155
PARP1-210  .............................................................................................................. 108
PARP1-202  .............................................................................................................. 108
PARP1-203  GKVFSATLGLVDIVKGTNSYYKLQLLEDDKENRYWIFRSWGRVGTVIGSNKLEQMPSKEDAIEHFMKLYEEKTGNAWHSKNFTKYPKKFYPLEIDYGQDEEAVKKLTVNP 659
```

```
logo
            GTKSKLPKPVQDLIKMIFDVESMKKAMVEYEIDLQKMPLGKLSKRQIQAAYSILSEVQQAVSQGSSDSQILDLSNRFYTLIPHDFGMKKPPLLNNADSVQAKVEMLDNLL
RUCAPARIB   ...................................................................................................Q...E......    2
PF00645     ..............................................................................................................   76
PF08063     ..............................................................................................................   52
PF00645.1   ..............................................................................................................  159
PF05406     ..............................................................................................................   77
PF02877     ...SKLPKPVQDLIKMIFDVESMKKAMVEYEIDLQKMPLGKLSKRQIQAAYSILSEVQQAVSQGSSDSQILDLSNRFYTLIPHDFGMKKPPLLNNADSVQAKVEMLDNLL  107
PF00533     ..............................................................................................................   74
PF00644     ..............................................................................................................    0
PARP1-201   ..............................................................................................................  155
PARP1-210   ..............................................................................................................  108
PARP1-202   ..............................................................................................................  108
PARP1-203   GTKSKLPKPVQDLIKMIFDVESMKKAMVEYEIDLQKMPLGKLSKRQIQAAYSILSEVQQAVSQGSSDSQILDLSNRFYTLIPHDFGMKKPPLLNNADSVQAKVEMLDNLL  769

logo
            DIEVAYSLLRGGSDDSSKDPIDVNYEKLKTDIKVVDRDSEEAEIIRKYVKNTHATTHNAYDLEVIDIFKIEREGECQRYKPFKQLHNRRLLWHGSRTTNFAGILSQGLRI
RUCAPARIB   ............................................................................................HG................    4
PF00645     ..............................................................................................................   76
PF08063     ..............................................................................................................   52
PF00645.1   ..............................................................................................................  159
PF05406     ..............................................................................................................   77
PF02877     DIEVAYSLLRGGSDDSSKDPIDVNY.....................................................................................  132
PF00533     ..............................................................................................................   74
PF00644     ......................................SEEAEIIRKYVKNTHATTHNAYDLEVIDIFKIEREGECQRYKPFKQLHNRRLLWHGSRTTNFAGILSQGLRI   72
PARP1-201   ..............................................................................................................  155
PARP1-210   ..............................................................................................................  108
PARP1-202   ..............................................................................................................  108
PARP1-203   DIEVAYSLLRGGSDDSSKDPIDVNYEKLKTDIKVVDRDSEEAEIIRKYVKNTHATTHNAYDLEVIDIFKIEREGECQRYKPFKQLHNRRLLWHGSRTTNFAGILSQGLRI  879

logo
            APPEAPVTGYMFGKGIYFADMVSKSANYCHTSQGDPIGLILLGEVALGNMYELKHASHISKLPKGKHSVKGLGKTTPDPSANISLDGVDVPLGTGISSGVNDTSLLYNEY
RUCAPARIB   .......TGY......YFA....KS..Y................................................................................E.   14
PF00645     ..............................................................................................................   76
PF08063     ..............................................................................................................   52
PF00645.1   ..............................................................................................................  159
PF05406     ..............................................................................................................   77
PF02877     ..............................................................................................................  132
PF00533     ..............................................................................................................   74
PF00644     APPEAPVTGYMFGKGIYFADMVSKSANYCHTSQGDPIGLILLGEVALGNMYELKHASHISKLPKGKHSVKGLGKTTPDPSANISLDGVDVPLGTGISSGVNDTSLLYNEY  182
PARP1-201   ..............................................................................................................  155
PARP1-210   ..............................................................................................................  108
PARP1-202   ..............................................................................................................  108
PARP1-203   APPEAPVTGYMFGKGIYFADMVSKSANYCHTSQGDPIGLILLGEVALGNMYELKHASHISKLPKGKHSVKGLGKTTPDPSANISLDGVDVPLGTGISSGVNDTSLLYNEY  989
```

logo

```
           IVYDIAQVNLKYLLKLKFNFKTSLW
RUCAPARIB  .........................    14
PF00645    .........................    76
PF08063    .........................    52
PF00645.1  .........................   159
PF05406    .........................    77
PF02877    .........................   132
PF00533    .........................    74
PF00644    IVYDIAQVNLKYLLKLKF.......   200
PARP1-201  .........................   155
PARP1-210  .........................   108
PARP1-202  .........................   108
PARP1-203  IVYDIAQVNLKYLLKLKFNFKTSLW  1014
```

X non conserved
X similar
X $\geq$ 0% conserved
X $\geq$ 50% conserved

logo

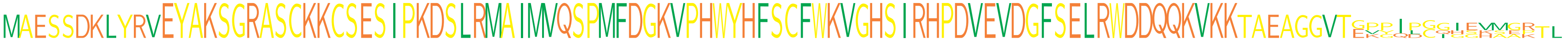

```
TALAZOPARIB   ..............................................................................................................    0
PF00645       ...........EYAKSGRASCKKCSESIPKDSLRMAIMVQSPMFDGKVPHWYHFSCFWKVGHSIRHPDVEVDGFSELRWDDQQKVKK.......................   76
PF08063       ..............................................................................................................    0
PF00645.1     ...........EYAKSGRASCKKCSESIPKDSLRMAIMVQSPMFDGKVPHWYHFSCFWKVGHSIRHPDVEVDGFSELRWDDQQKVKK.......................   76
PF05406       ..............................................................................................................    0
PF02877       ..............................................................................................................    0
PF00533       ..............................................................................................................    0
PF00644       ..............................................................................................................    0
PARP1-201     MAESSDKLYRVEYAKSGRASCKKCSESIPKDSLRMAIMVQSPMFDGKVPHWYHFSCFWKVGHSIRHPDVEVDGFSELRWDDQQKVKKTAEAGGVTGVYILGQHGHSAPTL  110
PARP1-210     MAESSDKLYRVEYAKSGRASCKKCSESIPKDSLRMAIMVQSPMFDGKVPHWYHFSCFWKVGHSIRHPDVEVDGFSELRWDDQQKVKKTAEAGGVTEPPIPCGIEVMGR..  108
PARP1-202     MAESSDKLYRVEYAKSGRASCKKCSESIPKDSLRMAIMVQSPMFDGKVPHWYHFSCFWKVGHSIRHPDVEVDGFSELRWDDQQKVKKTAEAGGVTEPPIPCGIEVMGR..  108
PARP1-203     MAESSDKLYRVEYAKSGRASCKKCSESIPKDSLRMAIMVQSPMFDGKVPHWYHFSCFWKVGHSIRHPDVEVDGFSELRWDDQQKVKKTAEAGGVTGKGQDGIGSKAEKTL  110
```

logo

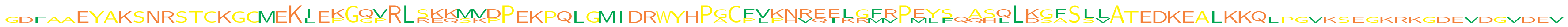

```
TALAZOPARIB   ..............................................................................................................    0
PF00645       ..............................................................................................................   76
PF08063       ..............................................................................................................    0
PF00645.1     .....EYAKSNRSTCKGCMEKIEKGQVRLSKKMVDPEKPQLGMIDRWYHPGCFVKNREELGFRPEYS.ASQLKGFSLLATEDKEALKKQ.....................  159
PF05406       ..............................................................................................................    0
PF02877       ..............................................................................................................    0
PF00533       ..............................................................................................................    0
PF00644       ..............................................................................................................    0
PARP1-201     ....................KL.PGGPRLREQSKPP.............PACPLPHVQIKRMVPMLFQQHLLDSASSVA...............................  155
PARP1-210     ..............................................................................................................  108
PARP1-202     ..............................................................................................................  108
PARP1-203     GDFAAEYAKSNRSTCKGCMEKIEKGQVRLSKKMVDPEKPQLGMIDRWYHPGCFVKNREELGFRPEYS.ASQLKGFSLLATEDKEALKKQLPGVKSEGKRKGDEVDGVDEV  219
```

logo

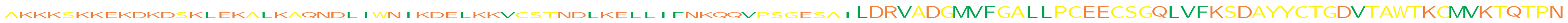

```
TALAZOPARIB   ..............................................................................................................    0
PF00645       ..............................................................................................................   76
PF08063       ............................................................LDRVADGMVFGALLPCEECSGQLVFKSDAYYCTGDVTAWTKCMVKTQTPN   50
PF00645.1     ..............................................................................................................  159
PF05406       ..............................................................................................................    0
PF02877       ..............................................................................................................    0
PF00533       ..............................................................................................................    0
PF00644       ..............................................................................................................    0
PARP1-201     ..............................................................................................................  155
PARP1-210     ..............................................................................................................  108
PARP1-202     ..............................................................................................................  108
PARP1-203     AKKKSKKEKDKDSKLEKALKAQNDLIWNIKDELKKVCSTNDLKELLIFNKQQVPSGESAILDRVADGMVFGALLPCEECSGQLVFKSDAYYCTGDVTAWTKCMVKTQTPN  329
```

```
logo
             RKEWVTPKEFREISYLKKLKVKKQDRIFPPETSASVAATPPPSTASAPAAVNSSASADKPLSNMKILTLGKLSRNKDEVKAMIEKLGGKLTGTANKASLCISTKKEVEKM
TALAZOPARIB  ..............................................................................................................    0
PF00645      ..............................................................................................................   76
PF08063      RK............................................................................................................   52
PF00645.1    ..............................................................................................................  159
PF05406      ..............................................................................................................    0
PF02877      ..............................................................................................................    0
PF00533      ..........................................................KPLSNMKILTLGKLSRNKDEVKAMIEKLGGKLTGTANKASLCISTKKEVEKM   52
PF00644      ..............................................................................................................    0
PARP1-201    ..............................................................................................................  155
PARP1-210    ..............................................................................................................  108
PARP1-202    ..............................................................................................................  108
PARP1-203    RKEWVTPKEFREISYLKKLKVKKQDRIFPPETSASVAATPPPSTASAPAAVNSSASADKPLSNMKILTLGKLSRNKDEVKAMIEKLGGKLTGTANKASLCISTKKEVEKM  439

logo
             NKKMEEVKEANIRVVSEDFLQDVSASTKSLQELFLAHILSPWGAEVKAEPVEVVAPRGKSGAALSKKSKGQVKEEGINKSEKRMKLTLKGGAAVDPDSGLEHSAHVLEKG
TALAZOPARIB  ..............................................................................................................    0
PF00645      ..............................................................................................................   76
PF08063      ..............................................................................................................   52
PF00645.1    ..............................................................................................................  159
PF05406      ..............................................................................................................    0
PF02877      ..............................................................................................................    0
PF00533      NKKMEEVKEANIRVVSEDFLQD........................................................................................   74
PF00644      ..............................................................................................................    0
PARP1-201    ..............................................................................................................  155
PARP1-210    ..............................................................................................................  108
PARP1-202    ..............................................................................................................  108
PARP1-203    NKKMEEVKEANIRVVSEDFLQDVSASTKSLQELFLAHILSPWGAEVKAEPVEVVAPRGKSGAALSKKSKGQVKEEGINKSEKRMKLTLKGGAAVDPDSGLEHSAHVLEKG  549

logo
             GKVFSATLGLVDIVKGTNSYYKLQLLEDDKENRYWIFRSWGRVGTVIGSNKLEQMPSKEDAIEHFMKLYEEKTGNAWHSKNFTKYPKKFYPLEIDYGQDEEAVKKLTVNP
TALAZOPARIB  ..............................................................................................................    0
PF00645      ..............................................................................................................   76
PF08063      ..............................................................................................................   52
PF00645.1    ..............................................................................................................  159
PF05406      ....SATLGLVDIVKGTNSYYKLQLLEDDKENRYWIFRSWGRVGTVIGSNKLEQMPSKEDAIEHFMKLYEEKTGNAWHSKN.............................   77
PF02877      ..............................................................................................................    0
PF00533      ..............................................................................................................   74
PF00644      ..............................................................................................................    0
PARP1-201    ..............................................................................................................  155
PARP1-210    ..............................................................................................................  108
PARP1-202    ..............................................................................................................  108
PARP1-203    GKVFSATLGLVDIVKGTNSYYKLQLLEDDKENRYWIFRSWGRVGTVIGSNKLEQMPSKEDAIEHFMKLYEEKTGNAWHSKNFTKYPKKFYPLEIDYGQDEEAVKKLTVNP  659
```

```
logo
GTKSKLPKPVQDLIKMIFDVESMKKAMVEYEIDLQKMPLGKLSKRQIQAAYSILSEVQQAVSQGSSDSQILDLSNRFYTLIPHDFGMKKPPLLNNADSVQAKVEMLDNLL
TALAZOPARIB   .......................................................................................................E......   1
PF00645       ..............................................................................................................   76
PF08063       ..............................................................................................................   52
PF00645.1     ..............................................................................................................   159
PF05406       ..............................................................................................................   77
PF02877       ...SKLPKPVQDLIKMIFDVESMKKAMVEYEIDLQKMPLGKLSKRQIQAAYSILSEVQQAVSQGSSDSQILDLSNRFYTLIPHDFGMKKPPLLNNADSVQAKVEMLDNLL   107
PF00533       ..............................................................................................................   74
PF00644       ..............................................................................................................   0
PARP1-201     ..............................................................................................................   155
PARP1-210     ..............................................................................................................   108
PARP1-202     ..............................................................................................................   108
PARP1-203     GTKSKLPKPVQDLIKMIFDVESMKKAMVEYEIDLQKMPLGKLSKRQIQAAYSILSEVQQAVSQGSSDSQILDLSNRFYTLIPHDFGMKKPPLLNNADSVQAKVEMLDNLL   769

logo
DIEVAYSLLRGGSDDSSKDPIDVNYEKLKTDIKVVDRDSEEAEIIRKYVKNTHATTHNAYDLEVIDIFKIEREGECQRYKPFKQLHNRRLLWHGSRTTNFAGILSQGLRI
TALAZOPARIB   ............................................................................................HG................   3
PF00645       ..............................................................................................................   76
PF08063       ..............................................................................................................   52
PF00645.1     ..............................................................................................................   159
PF05406       ..............................................................................................................   77
PF02877       DIEVAYSLLRGGSDDSSKDPIDVNY.....................................................................................   132
PF00533       ..............................................................................................................   74
PF00644       ......................................SEEAEIIRKYVKNTHATTHNAYDLEVIDIFKIEREGECQRYKPFKQLHNRRLLWHGSRTTNFAGILSQGLRI   72
PARP1-201     ..............................................................................................................   155
PARP1-210     ..............................................................................................................   108
PARP1-202     ..............................................................................................................   108
PARP1-203     DIEVAYSLLRGGSDDSSKDPIDVNYEKLKTDIKVVDRDSEEAEIIRKYVKNTHATTHNAYDLEVIDIFKIEREGECQRYKPFKQLHNRRLLWHGSRTTNFAGILSQGLRI   879

logo
APPEAPVTGYMFGKGIYFADMVSKSANYCHTSQGDPIGLILLGEVALGNMYELKHASHISKLPKGKHSVKGLGKTTPDPSANISLDGVDVPLGTGISSGVNDTSLLYNEY
TALAZOPARIB   ........GY.....IYFA....KS..Y................................................................................E.   13
PF00645       ..............................................................................................................   76
PF08063       ..............................................................................................................   52
PF00645.1     ..............................................................................................................   159
PF05406       ..............................................................................................................   77
PF02877       ..............................................................................................................   132
PF00533       ..............................................................................................................   74
PF00644       APPEAPVTGYMFGKGIYFADMVSKSANYCHTSQGDPIGLILLGEVALGNMYELKHASHISKLPKGKHSVKGLGKTTPDPSANISLDGVDVPLGTGISSGVNDTSLLYNEY   182
PARP1-201     ..............................................................................................................   155
PARP1-210     ..............................................................................................................   108
PARP1-202     ..............................................................................................................   108
PARP1-203     APPEAPVTGYMFGKGIYFADMVSKSANYCHTSQGDPIGLILLGEVALGNMYELKHASHISKLPKGKHSVKGLGKTTPDPSANISLDGVDVPLGTGISSGVNDTSLLYNEY   989
```

logo

```
               IVYDIAQVNLKYLLKLKFNFKTSLW
TALAZOPARIB    .........................     13
PF00645        .........................     76
PF08063        .........................     52
PF00645.1      .........................    159
PF05406        .........................     77
PF02877        .........................    132
PF00533        .........................     74
PF00644        IVYDIAQVNLKYLLKLKF.......    200
PARP1-201      .........................    155
PARP1-210      .........................    108
PARP1-202      .........................    108
PARP1-203      IVYDIAQVNLKYLLKLKFNFKTSLW   1014
```

X non conserved

X similar

X $\geq$ 0% conserved

X $\geq$ 50% conserved

logo

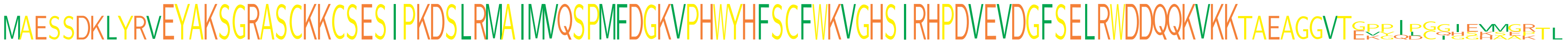

```
VELIPARIB   ..............................................................................................................    0
PF00645     ...........EYAKSGRASCKKCSESIPKDSLRMAIMVQSPMFDGKVPHWYHFSCFWKVGHSIRHPDVEVDGFSELRWDDQQKVKK.......................   76
PF08063     ..............................................................................................................    0
PF00645.1   ...........EYAKSGRASCKKCSESIPKDSLRMAIMVQSPMFDGKVPHWYHFSCFWKVGHSIRHPDVEVDGFSELRWDDQQKVKK.......................   76
PF05406     ..............................................................................................................    0
PF02877     ..............................................................................................................    0
PF00533     ..............................................................................................................    0
PF00644     ..............................................................................................................    0
PARP1-201   MAESSDKLYRVEYAKSGRASCKKCSESIPKDSLRMAIMVQSPMFDGKVPHWYHFSCFWKVGHSIRHPDVEVDGFSELRWDDQQKVKKTAEAGGVTGVYILGQHGHSAPTL  110
PARP1-210   MAESSDKLYRVEYAKSGRASCKKCSESIPKDSLRMAIMVQSPMFDGKVPHWYHFSCFWKVGHSIRHPDVEVDGFSELRWDDQQKVKKTAEAGGVTEPPIPCGIEVMGR..  108
PARP1-202   MAESSDKLYRVEYAKSGRASCKKCSESIPKDSLRMAIMVQSPMFDGKVPHWYHFSCFWKVGHSIRHPDVEVDGFSELRWDDQQKVKKTAEAGGVTEPPIPCGIEVMGR..  108
PARP1-203   MAESSDKLYRVEYAKSGRASCKKCSESIPKDSLRMAIMVQSPMFDGKVPHWYHFSCFWKVGHSIRHPDVEVDGFSELRWDDQQKVKKTAEAGGVTGKGQDGIGSKAEKTL  110
```

logo

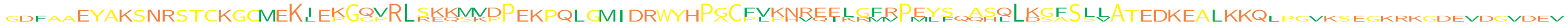

```
VELIPARIB   ..............................................................................................................    0
PF00645     ..............................................................................................................   76
PF08063     ..............................................................................................................    0
PF00645.1   .....EYAKSNRSTCKGCMEKIEKGQVRLSKKMVDPEKPQLGMIDRWYHPGCFVKNREELGFRPEYS.ASQLKGFSLLATEDKEALKKQ.....................  159
PF05406     ..............................................................................................................    0
PF02877     ..............................................................................................................    0
PF00533     ..............................................................................................................    0
PF00644     ..............................................................................................................    0
PARP1-201   ....................KL.PGGPRLREQSKPP.............PACPLPHVQIKRMVPMLFQQHLLDSASSVA...............................  155
PARP1-210   ..............................................................................................................  108
PARP1-202   ..............................................................................................................  108
PARP1-203   GDFAAEYAKSNRSTCKGCMEKIEKGQVRLSKKMVDPEKPQLGMIDRWYHPGCFVKNREELGFRPEYS.ASQLKGFSLLATEDKEALKKQLPGVKSEGKRKGDEVDGVDEV  219
```

logo

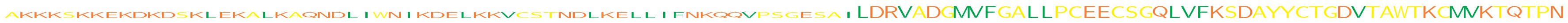

```
VELIPARIB   ..............................................................................................................    0
PF00645     ..............................................................................................................   76
PF08063     ............................................................LDRVADGMVFGALLPCEECSGQLVFKSDAYYCTGDVTAWTKCMVKTQTPN   50
PF00645.1   ..............................................................................................................  159
PF05406     ..............................................................................................................    0
PF02877     ..............................................................................................................    0
PF00533     ..............................................................................................................    0
PF00644     ..............................................................................................................    0
PARP1-201   ..............................................................................................................  155
PARP1-210   ..............................................................................................................  108
PARP1-202   ..............................................................................................................  108
PARP1-203   AKKKSKKEKDKDSKLEKALKAQNDLIWNIKDELKKVCSTNDLKELLIFNKQQVPSGESAILDRVADGMVFGALLPCEECSGQLVFKSDAYYCTGDVTAWTKCMVKTQTPN  329
```

```
logo
             RKEWVTPKEFREISYLKKLKVKKQDRIFPPETSASVAATPPPSTASAPAAVNSSASADKPLSNMKILTLGKLSRNKDEVKAMIEKLGGKLTGTANKASLCISTKKEVEKM
VELIPARIB    ..............................................................................................................    0
PF00645      ..............................................................................................................   76
PF08063      RK............................................................................................................   52
PF00645.1    ..............................................................................................................  159
PF05406      ..............................................................................................................    0
PF02877      ..............................................................................................................    0
PF00533      ..........................................................KPLSNMKILTLGKLSRNKDEVKAMIEKLGGKLTGTANKASLCISTKKEVEKM   52
PF00644      ..............................................................................................................    0
PARP1-201    ..............................................................................................................  155
PARP1-210    ..............................................................................................................  108
PARP1-202    ..............................................................................................................  108
PARP1-203    RKEWVTPKEFREISYLKKLKVKKQDRIFPPETSASVAATPPPSTASAPAAVNSSASADKPLSNMKILTLGKLSRNKDEVKAMIEKLGGKLTGTANKASLCISTKKEVEKM  439

logo
             NKKMEEVKEANIRVVSEDFLQDVSASTKSLQELFLAHILSPWGAEVKAEPVEVVAPRGKSGAALSKKSKGQVKEEGINKSEKRMKLTLKGGAAVDPDSGLEHSAHVLEKG
VELIPARIB    ..............................................................................................................    0
PF00645      ..............................................................................................................   76
PF08063      ..............................................................................................................   52
PF00645.1    ..............................................................................................................  159
PF05406      ..............................................................................................................    0
PF02877      ..............................................................................................................    0
PF00533      NKKMEEVKEANIRVVSEDFLQD........................................................................................   74
PF00644      ..............................................................................................................    0
PARP1-201    ..............................................................................................................  155
PARP1-210    ..............................................................................................................  108
PARP1-202    ..............................................................................................................  108
PARP1-203    NKKMEEVKEANIRVVSEDFLQDVSASTKSLQELFLAHILSPWGAEVKAEPVEVVAPRGKSGAALSKKSKGQVKEEGINKSEKRMKLTLKGGAAVDPDSGLEHSAHVLEKG  549

logo
             GKVFSATLGLVDIVKGTNSYYKLQLLEDDKENRYWIFRSWGRVGTVIGSNKLEQMPSKEDAIEHFMKLYEEKTGNAWHSKNFTKYPKKFYPLEIDYGQDEEAVKKLTVNP
VELIPARIB    ..............................................................................................................    0
PF00645      ..............................................................................................................   76
PF08063      ..............................................................................................................   52
PF00645.1    ..............................................................................................................  159
PF05406      ....SATLGLVDIVKGTNSYYKLQLLEDDKENRYWIFRSWGRVGTVIGSNKLEQMPSKEDAIEHFMKLYEEKTGNAWHSKN.............................   77
PF02877      ..............................................................................................................    0
PF00533      ..............................................................................................................   74
PF00644      ..............................................................................................................    0
PARP1-201    ..............................................................................................................  155
PARP1-210    ..............................................................................................................  108
PARP1-202    ..............................................................................................................  108
PARP1-203    GKVFSATLGLVDIVKGTNSYYKLQLLEDDKENRYWIFRSWGRVGTVIGSNKLEQMPSKEDAIEHFMKLYEEKTGNAWHSKNFTKYPKKFYPLEIDYGQDEEAVKKLTVNP  659
```

```
logo
           GTKSKLPKPVQDLIKMIFDVESMKKAMVEYEIDLQKMPLGKLSKRQIQAAYSILSEVQQAVSQGSSDSQILDLSNRFYTLIPHDFGMKKPPLLNNADSVQAKVEMLDNLL
VELIPARIB  ..............................................................................................................    0
PF00645    ..............................................................................................................   76
PF08063    ..............................................................................................................   52
PF00645.1  ..............................................................................................................  159
PF05406    ..............................................................................................................   77
PF02877    ...SKLPKPVQDLIKMIFDVESMKKAMVEYEIDLQKMPLGKLSKRQIQAAYSILSEVQQAVSQGSSDSQILDLSNRFYTLIPHDFGMKKPPLLNNADSVQAKVEMLDNLL  107
PF00533    ..............................................................................................................   74
PF00644    ..............................................................................................................    0
PARP1-201  ..............................................................................................................  155
PARP1-210  ..............................................................................................................  108
PARP1-202  ..............................................................................................................  108
PARP1-203  GTKSKLPKPVQDLIKMIFDVESMKKAMVEYEIDLQKMPLGKLSKRQIQAAYSILSEVQQAVSQGSSDSQILDLSNRFYTLIPHDFGMKKPPLLNNADSVQAKVEMLDNLL  769

logo
           DIEVAYSLLRGGSDDSSKDPIDVNYEKLKTDIKVVDRDSEEAEIIRKYVKNTHATTHNAYDLEVIDIFKIEREGECQRYKPFKQLHNRRLLWHGSRTTNFAGILSQGLRI
VELIPARIB  ............................................................................................HG................    2
PF00645    ..............................................................................................................   76
PF08063    ..............................................................................................................   52
PF00645.1  ..............................................................................................................  159
PF05406    ..............................................................................................................   77
PF02877    DIEVAYSLLRGGSDDSSKDPIDVNY.....................................................................................  132
PF00533    ..............................................................................................................   74
PF00644    ......................................SEEAEIIRKYVKNTHATTHNAYDLEVIDIFKIEREGECQRYKPFKQLHNRRLLWHGSRTTNFAGILSQGLRI   72
PARP1-201  ..............................................................................................................  155
PARP1-210  ..............................................................................................................  108
PARP1-202  ..............................................................................................................  108
PARP1-203  DIEVAYSLLRGGSDDSSKDPIDVNYEKLKTDIKVVDRDSEEAEIIRKYVKNTHATTHNAYDLEVIDIFKIEREGECQRYKPFKQLHNRRLLWHGSRTTNFAGILSQGLRI  879

logo
           APPEAPVTGYMFGKGIYFADMVSKSANYCHTSQGDPIGLILLGEVALGNMYELKHASHISKLPKGKHSVKGLGKTTPDPSANISLDGVDVPLGTGISSGVNDTSLLYNEY
VELIPARIB  .........Y......YFA....KS..Y................................................................................E.   10
PF00645    ..............................................................................................................   76
PF08063    ..............................................................................................................   52
PF00645.1  ..............................................................................................................  159
PF05406    ..............................................................................................................   77
PF02877    ..............................................................................................................  132
PF00533    ..............................................................................................................   74
PF00644    APPEAPVTGYMFGKGIYFADMVSKSANYCHTSQGDPIGLILLGEVALGNMYELKHASHISKLPKGKHSVKGLGKTTPDPSANISLDGVDVPLGTGISSGVNDTSLLYNEY  182
PARP1-201  ..............................................................................................................  155
PARP1-210  ..............................................................................................................  108
PARP1-202  ..............................................................................................................  108
PARP1-203  APPEAPVTGYMFGKGIYFADMVSKSANYCHTSQGDPIGLILLGEVALGNMYELKHASHISKLPKGKHSVKGLGKTTPDPSANISLDGVDVPLGTGISSGVNDTSLLYNEY  989
```

logo

```
           IVYDIAQVNLKYLLKLKFNFKTSLW
VELIPARIB  .........................    10
PF00645    .........................    76
PF08063    .........................    52
PF00645.1  .........................   159
PF05406    .........................    77
PF02877    .........................   132
PF00533    .........................    74
PF00644    IVYDIAQVNLKYLLKLKF.......   200
PARP1-201  .........................   155
PARP1-210  .........................   108
PARP1-202  .........................   108
PARP1-203  IVYDIAQVNLKYLLKLKFNFKTSLW  1014
```

X non conserved

X similar

X $\geq 0\%$ conserved

X $\geq 50\%$ conserved

```
logo
            MAARRRRSTGGGRARALNESKRVNNGNTAPEDSSPAKKTRRCQRQESKKMPVAGGKANKDRTEDKQDGMPGRSWASKRVSESVKALLLKGKAPVDPECTAKVGKAHVYCE
OLAPARIB    ..............................................................................................................      0
PF02877     ..............................................................................................................      0
PF00644     ..............................................................................................................      0
PF02877.1   ..............................................................................................................      0
PF00644.1   ..............................................................................................................      0
PF05406     ..............................................................................................................      0
PF00644.2   ..............................................................................................................      0
PARP2-202   MAARRRRSTGGGRARALNESKRVNNGNTAPEDSSPAKKTRRCQRQESKKMPVAGGKANKDRTEDKQD.............ESVKALLLKGKAPVDPECTAKVGKAHVYCE     97
PARP2-204   MAARRRRSTGGGRARALNESKRVNNGNTAPEDSSPAKKTRRCQRQESKKMPVAGGKANKDRTEDKQDGMPGRSWASKRVSESVKALLLKGKAPVDPECTAKVGKAHVYCE    110
PARP2-210   ..............................................................................................................      0
PARP2-206   MAARRRRSTGGGRARALNESKRVNNGNTAPEDSSPAKKTRRCQRQESKKMPVAGGKANKDRTEDKQD.............ESVKALLLKGKAPVDPECTAKVGKAHVYCE     97
PARP2-201   MAARRRRSTGGGRARALNESKRVNNGNTAPEDSSPAKKTRRCQRQESKKMPVAGGKANKDRTEDKQDGMPGRSWASKRVSESVKALLLKGKAPVDPECTAKVGKAHVYCE    110

logo
            GNDVYDVMLNQTNLQFNNNKYYLIQLLEDDAQRNFSVWMRWGRVGKMGQHSLVACSGNLNKAKEIFQKKFLDKTKNNWEDREKFEKVPGKYDMLQMDYATNTQDEEETKK
OLAPARIB    ..............................................................................................................      0
PF02877     ..............................................................................................................      0
PF00644     ..............................................................................................................      0
PF02877.1   ..............................................................................................................      0
PF00644.1   ..............................................................................................................      0
PF05406     ....YDVMLNQTNLQFNNNKYYLIQLLEDDAQRNFSVWMRWGRVGKMGQHSLVACSGNLNKAKEIFQKKFLDKTKNNWEDREKF..........................     80
PF00644.2   ..............................................................................................................      0
PARP2-202   GNDVYDVMLNQTNLQFNNNKYYLIQLLEDDAQRNFSVWMRWGRVGKMGQHSLVACSGNLNKAKEIFQKKFLDKTKNNWEDREKFEKVPGKYDMLQMDYATNTQDEEETKK    207
PARP2-204   GNDVYDVMLNQTNLQFNNNKYYLIQLLEDDAQRNFSVWMRWGRVGKMGQHSLVACSGNLNKAKEIFQKKFLDKTKNNWEDREKFEKVPGKYDMLQMDYATNTQDEEETKK    220
PARP2-210   ..............................................................................................................      0
PARP2-206   GNDVYDVMLNQIP.................................................................................................    110
PARP2-201   GNDVYDVMLNQTNLQFNNNKYYLIQLLEDDAQRNFSVWMRWGRVGKMGQHSLVACSGNLNKAKEIFQKKFLDKTKNNWEDREKFEKVPGKYDMLQMDYATNTQDEEETKK    220

logo
            EESLKSPLKPESQLDLRVQELIKLICNVQAMEEMMMEMKYNTKKAPLGKLTVAQIKAGYQSLKKIEDCIRAGQHGRALMEACNEFYTRIPHDFGLRTPPLIRTQKELSEK
OLAPARIB    ............................................................................................................E.      1
PF02877     ...........SQLDLRVQELIKLICNVQAMEEMMMEMKYNTKKAPLGKLTVAQIKAGYQSLKKIEDCIRAGQHGRALMEACNEFYTRIPHDFGLRTPPLIRTQKELSEK     99
PF00644     ..............................................................................................................      0
PF02877.1   ........................................................................................................KELSEK      6
PF00644.1   ..............................................................................................................      0
PF05406     ..............................................................................................................     80
PF00644.2   ..............................................................................................................      0
PARP2-202   EESLKSPLKPESQLDLRVQELIKLICNVQAMEEMMMEMKYNTKKAPLGKLTVAQIKAGYQSLKKIEDCIRAGQHGRALMEACNEFYTRIPHDFGLRTPPLIRTQKELSEK    317
PARP2-204   EESLKSPLKPESQLDLRVQELIKLICNVQAMEEMMMEMKYNTKKAPLGKLTVAQIKAGYQSLKKIEDCIRAGQHGRALMEACNEFYTRIPHDFGLRTPPLIRTQKELSEK    330
PARP2-210   .......................................................................................................XKELSEK      7
PARP2-206   ..............................................................................................................    110
PARP2-201   EESLKSPLKPESQLDLRVQELIKLICNVQAMEEMMMEMKYNTKKAPLGKLTVAQIKAGYQSLKKIEDCIRAGQHGRALMEACNEFYTRIPHDFGLRTPPLIRTQKELSEK    330
```

```
logo         IQLLEALGDIEIAIKLVKTELQSPEHPLDQHYRNLHCALRPLDHESYEFKVISQYLQSTHAPTHSDYTMTLLDLFEVEKDGEKEAFREDLHNRMLLWHGSRMSNWVGILS
OLAPARIB     .................................................................................................HG...........   3
PF02877      IQLLEALGDIEIAIKLVKTELQSPEHPLDQHY..............................................................................   131
PF00644      .............................................SYEFKVISQYLQSTHAPTHSDYTMTLLDLFEVEKDGEKEAFREDLHNRMLLWHGSRMSNWVGILS   65
PF02877.1    IQLLEALGDIEIAIKLVKTELQSPEHPLDQHY..............................................................................   38
PF00644.1    .............................................SYEFKVISQYLQSTHAPTHSDYTMTLLDLFEVEKDGEKEAFREDLHNRMLLWHGSRMSNWVGILS   65
PF05406      ..............................................................................................................   80
PF00644.2    .............................................SYEFKVISQYLQSTHAPTHSDYTMTLLDLFEVEKDGEKEAFREDLHNRMLLWHGSRMSNWVGILS   65
PARP2-202    IQLLEALGDIEIAIKLVKTELQSPEHPLDQHYRNLHCALRPLDHESYEFKVISQYLQSTHAPTHSDYTMTLLDLFEVEKDGEKEAFREDLHNRMLLWHGSRMSNWVGILS   427
PARP2-204    IQLLEALGDIEIAIKLVKTELQSPEHPLDQHYRNLHCALRPLDHESYEFKVISQYLQSTHAPTHSDYTMTLLDLFEVEKDGEKEAFREDLHNRMLLWHGSRMSNWVGILS   440
PARP2-210    IQLLEALGDIEIAIKLVKTELQSPEHPLDQHYRNLHCALRPLDHESYEFKVISQYLQSTHAPTHSDYTMTLLDLFEVEKDGEKEAFREDLHNRMLLWHGSRMSNWVGILS   117
PARP2-206    ..............................................................................................................   110
PARP2-201    IQLLEALGDIEIAIKLVKTELQSPEHPLDQHYRNLHCALRPLDHESYEFKVISQYLQSTHAPTHSDYTMTLLDLFEVEKDGEKEAFREDLHNRMLLWHGSRMSNWVGILS   440

logo         HGLRIAPPEAPITGYMFGKGIYFADMSSKSANYCFASRLKNTGLLLLSEVALGQCNELLEANPKAEGLLQGKHSTKGLGKMAPSSAHFVTLNGSTVPLGPASDTGILNPD
OLAPARIB     ..LR.A........Y....GIYF......S..Y.............................................................................   13
```
[truncated: 619,453 more chars]
